# Phylostratigraphic analysis of gene co-expression network reveals the evolution of functional modules for ovarian cancer

**DOI:** 10.1038/s41598-019-40023-9

**Published:** 2019-02-22

**Authors:** Luoyan Zhang, Yi Tan, Shoujin Fan, Xuejie Zhang, Zhen Zhang

**Affiliations:** 1 Key Lab of Plant Stress Research, College of Life Science, Shandong Normal University, Jinan, 250014 Shandong China; 2 Laboratory for Molecular Immunology, Institute of Basic Medicine, Shandong Academy of Medical Sciences, Jinan, 250062 Shandong China; 3 Qilu Cell Therapy Technology Co., Ltd, Jinan, 250000 Shandong China

## Abstract

Ovarian cancer (OV) is an extremely lethal disease. However, the evolutionary machineries of OV are still largely unknown. Here, we used a method that combines phylostratigraphy information with gene co-expression networks to extensively study the evolutionary compositions of OV. The present co-expression network construction yielded 18,549 nodes and 114,985 edges based on 307 OV expression samples obtained from the Genome Data Analysis Centers database. A total of 20 modules were identified as OV related clusters. The human genome sequences were divided into 19 phylostrata (PS), the majority (67.45%) of OV genes was already present in the eukaryotic ancestor. There were two strong peaks of the emergence of OV genes screened by hypergeometric test: the evolution of the multicellular metazoan organisms (PS5 and PS6, *P value* = 0.002) and the emergence of bony fish (PS11 and PS12, *P value* = 0.009). Hence, the origin of OV is far earlier than its emergence. The integrated analysis of the topology of OV modules and the phylogenetic data revealed an evolutionary pattern of OV in human, namely, OV modules have arisen step by step during the evolution of the respective lineages. New genes have evolved and become locked into a pathway, where more and more biological pathways are fixed into OV modules by recruiting new genes during human evolution.

## Introduction

Ovarian cancer (OV) is an extremely lethal disease, which afflicts approximately 204,000 women worldwide each year^1–3^. Ovarian carcinomas belong to a heterogeneous group of neoplasms sub-classified based on types and degrees of differentiation. Thus, it is challenging for current clinical management of ovarian carcinoma to take all the neoplasms into account^3–5^. Developing representative genetic defects of each major histological type to improve targeted treatment strategies is an effective and promising solution for OV. Knowledge of the morphological features, biological behaviors, and molecular/genetic controlling machineries of ovarian carcinomas in model animals may contribute to studying ovarian cancer biology and enhancing pre-clinical testing of molecularly targeted therapeutics. Such knowledge may ultimately lead to better clinical outcomes for women with ovarian cancer^3,4^.

Cancer is thought to be a probabilistic event determined by a series of mutations occurring in cancer-associated genes. For OV, it seems that several thousand of genes could contribute to ovarian tumors’ development^2,6,7^. However, mechanistically, these genes do not all contribute in the same way to cancer progression. Microarray or RNA sequencing is an effective choice for detecting potential participants of complicated traits on a genome-wide scale in animals, fungi, plants and microorganisms^8–20^. Thousands of analyses about cancers^15,16,21–23^, immune systems^24–31^ and genetic diseases^32–38^ in model animals have been widely conducted using sequencing technologies. The integrations of genes’ spatio-temporal expression patterns and OV related traits have helped to identify the potential genes and controlling machineries of ovarian carcinomas^4,15^.

Gene co-expression networks based on sequencing data facilitate a global view of gene-gene co-expressed relationships^39–44^. Genes involved in related biological processes tend to be co-expressed and clustered as functional modules which can help to discover how the interplay between inter-connected genes accomplish specific biological functions^34,40,45–49^. The ever-increasing number of sequenced genomes and expression datasets have promoted the use of genomic phylostratigraphic analysis as a tool to understand OV evolution. The phylostratigraphic method was designed to uncover the evolutionary origin of gene families by tracing to their earliest common ancestor, and consequently assess the age of the studied families^50–55^.

Cancer developments depend on both genetic factors and environmental pressures^56–58^. Cancer genes could be divided into two categories based on their functional characters and phylostratigraphic information. First is the “caretakers” of older phylostrata, like phylostratum (PS) PS1–PS2, which participate in fundamental functions that support genome stability. The other is “gatekeepers” of younger phylostrata, like PS6, PS11 and PS12, which are involved in cellular signaling and regulating processes^53,54,58,59^. “Gatekeepers” genes’ mutations promote tumour progression directly by changing cell differentiation, growth and death rates^53,59^, whereas mutations in “caretakers” promote tumour via increasing the chances that mutations will hit some genes within the “gatekeepers”. However, both “gatekeepers” and “caretakers” genes can possibly influence the convergence and/or divergence of complex traits^53,57,59^.

Genomic phylostratigraphic analysis could infer genes’ evolutionary origin on the basis of the functions of the genes in extant organisms^50,53–55,60,61^. Genomic phylostratigraphy assumes that protein families are initiated by founder genes throughout evolutionary time^53–55^. New functional proteins’ generations are associated with new functional forms and they evolve very quickly until becoming locked into a pathway by interacting with older genes. These characteristics are essential for speculating the evolution of cancers and other complicated traits^53–55^. The phylostratigraphy method has been used for tracking the formation of metazoa specific complex traits in animals, such as in embryonic developments, cancers and human genetic diseases^50,52,54–62^, and uncovering the genomic history of major adaptations in plants^63^. However, the study of evolutionary origins of human cancer genes based on phylostratigraphy analysis was rarely utilized in ovarian cancers.

To explore the macro-evolutionary patterns of genes and functional modules involved in OV, we conducted the phylostratigraphic analysis of gene co-expression network in human. The phylostratigraphic information was assigned to the genes and functional modules for identifying their evolution patterns. Our analysis focused on uncovering the peaks of the emergence of ovarian cancer genes and the evolutionary manners of new genes, gene-gene interactions and the biological processes being recruited to the OV network. These processes pose great values in understanding the macro-evolutionary mechanisms of OV in human and developing better clinical treatments for women with ovarian cancer.

## Results

### Expression profiling samples and marker genes extraction in H. sapiens

A total of 307 OV samples of 18,549 genome genes were retrieved from the GDAC database and used for co-expression network construction (Supplemental Table S1). A total of 45,239 articles were collected from PubMed by searching the key words. Of them, 17,961 articles offered empirical evidences on the functions of genes in OV. A total of 2313 genes with clear OV functions were identified by text mining and treated as functionally verified OV marker genes (Supplemental Table S2 and Table S4). Of them, 1900 genes were identified as GAD_DISEASE genes by DAVID database, like 2′–5′-oligoadenylate synthetase 3 (OAS3), 5′-nucleotidase ecto (NT5E), growth arrest and DNA damage inducible gamma (GADD45G) and growth arrest specific 1 (GAS1), etc (Supplemental Table S2).

### Network construction and functional module identification

**Figure 1:**
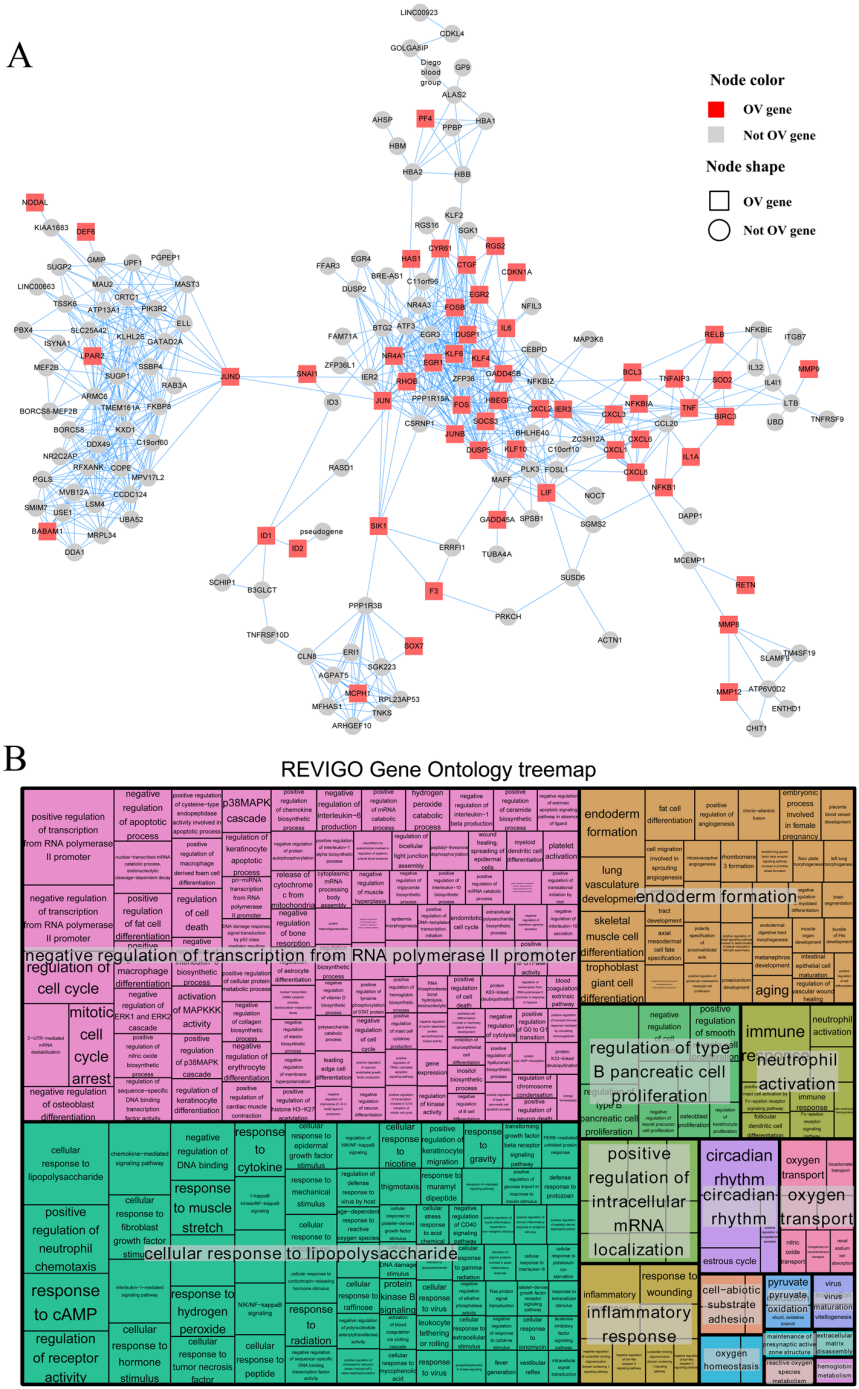
**(A)** Features of HCCA gene module M_11, it was identified as ovarian cancer (OV) associated functional modules by hypergeometric test. Nodes in this cluster, or gene-level network, represent genes, while edges depict the co-expressed relations between any two nodes. Node colors and node shapes depict gene’s functional categories, respectively. Red or grey nodes represent the OV genes. Rectangle or circular nodes depict the OV marker genes or not markers. **(B)** The REVIGO analysis result for the genes in module M_11. Each rectangle is a single cluster representative. The representatives are joined into “superclusters” of loosely related terms, visualized with different colors. Size of the rectangles was adjusted to reflect the p value of the GO term calculated by TopGO. The 386 biological processes (BP) enriched by genes of module M_11 were summarized to seventeen subsets by REVIGO analysis.

The present co-expression network construction yielded 18,549 nodes and 114,985 edges with a HRR coefficient of 30. This global network was further clustered into 208 co-expressed clusters using HCCA method (Supplemental Table S3, Table S5 and Table S6). The gene ontology enrichment results of 208 clusters were recorded in Table S6. A total of 20 modules were identified as OV related clusters by hypergeometric test (Table 1) under the significance cutoff *P value* < 0.05. Of them, genes of module M_11 were significantly enriched in “positive regulation of transcription from RNA polymerase II promoter” (*P value* < 0.01), module M_25 was discovered to have functioned in “anterior/posterior pattern specification” (*P value* < 0.01) and members of module M_26 were uncovered to have participated in “cell division” (*P value* < 0.01) (Table 1). The gene-gene co-expression relations of the 20 OV modules are shown in Supplemental Table S5. A total of 31.6% (56 of 177, *P value* < 0.001) nodes of module M_11 were identified as OV marker genes (Table 1, Fig. 1A). The 386 biological processes (BP) enriched by genes of module M_11 were summarized into seventeen subsets by REVIGO analysis, as “negative regulation of transcription from RNA polymerase II promoter” (including 107 terms), “cellular response to lipopolysaccharide” (including 71 terms) and “endoderm formation” (including 34 terms) (Fig. 1B).

**Table 1:** 20 modules identified as the OV-related modules.

Module	Module gene num.	Module OV gene num.	Pval	Module function
M_11	177	56	1.53E-11	positive regulation of transcription from RNA polymerase II promoter
M_25	81	19	4.43E-03	anterior/posterior pattern specification
M_26	177	44	4.49E-06	cell division
M_29	69	22	1.94E-05	positive regulation of myoblast fusion
M_34	179	48	1.52E-07	extracellular matrix organization
M_47	83	17	2.58E-02	condensed mesenchymal cell proliferation
M_76	82	23	1.20E-04	negative regulation of ERK1 and ERK2 cascade
M_84	55	19	1.98E-05	glutamate biosynthetic process
M_91	72	16	1.45E-02	O-glycan processing
M_100	102	22	6.75E-03	ceramide metabolic process
M_102	82	36	1.48E-12	collagen fibril organization
M_116	116	32	9.05E-06	epidermis development
M_117	107	20	4.08E-02	antigen processing and presentation of exogenous peptide antigen via MHC class I, TAP-dependent
M_118	59	13	2.77E-02	negative regulation of cell migration
M_144	63	17	1.42E-03	bicellular tight junction assembly
M_147	63	23	9.03E-07	negative regulation of endopeptidase activity
M_158	82	19	5.10E-03	negative regulation of focal adhesion assembly
M_159	45	12	7.58E-03	notochord regression
M_184	61	25	2.09E-08	type I interferon signaling pathway
M_194	45	11	1.99E-02	positive regulation of dendritic spine morphogenesis

Notes: Module gene num. = gene number of the module; Module OV gene num. = OV gene number of the module; Pval = *P value* of hypergeometric test for filtering OV-related modules; Module function = the top gene ontology (GO) biological term (BP) significantly enriched by the module members.

### Phylostratigraphy of genome and OV genes

**Figure 2:**
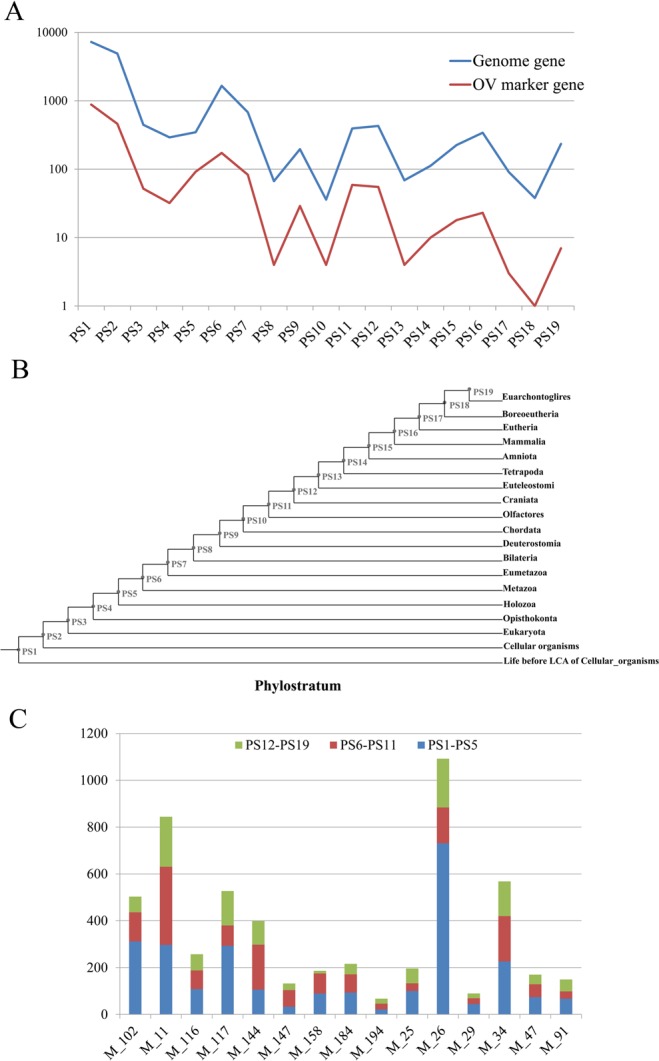
**(A)** Statistical analysis of human whole genome and OV datasets on the phylostratigraphic map. Blue line denote the whole genome dataset (N = 17,812) and red line denote the OV dataset (red line, N = 1994). there are three strong peaks of the emergence of ovarian cancer genes, at the phylostrata of the origin of the first cell (PS1 and PS2), the evolution of the multicellular metazoan organisms (PS6) and the emergence of bony fish (PS11 and PS12). **(B)** The phylostratigraphic map of *H*. *sapiens* and phylogeny used in the search for the evolutionary origin of human genes, 19 genomic phylostrata that correspond to the phylogenetic internodes. **(C)** The distribution of the gene-gene co-expression relations of the 20 OV modules in the three phylostratigraphic time-points (PS1–PS5, PS6–PS11 and PS12–PS19).

Based on the previously described phylostratigraphic procedure, the human genome sequence was divided into 19 phylostrata (Fig. 2A,B). Figure 2A shows the origin of 17,812 human genes plotted onto the 19 phylostrata (PS). Approximately 70% (12,156 of 17,812) of the genes were traced back to the origin of life and the emergence of cellular organisms (PS1 and PS2). The other three peaks of gene emergence were associated with the evolution of multicellularity (PS6) and the emergence of bony fish/tetrapoda (PS11 and PS12). Similarly, the 1994 OV marker genes were assigned to the 19 phylostrata and the distribution pattern of genes in phylostrata was the same as the genome genes. A total of 67.45% (hypergeometric test *P value* = 0.798), 13.29% (*P value* = 0.002) and 5.72% (*P value* = 0.009) functionally verified OV genes’ origins were found in “Life before LCA of Cellular organisms” and “Cellular organisms” (PS1 and PS2), “Holozoa” and “Metazoa” (PS5 and PS6), and “Olfactores” and “Craniata” (PS11 and PS12) respectively (Fig. 2A). The phylostratigraphic information of human genes was mapped to the gene-gene co-expression relations of the 20 OV modules as shown in Fig. 2C. Consequently, all the modules were discovered to contain edges of the three phylostratigraphic time-points (PS1–PS5, PS6–PS11 and PS12–PS19). For module M_147, 53.79% (71 of 132, *P value* < *0*.*001*) and 21.21% (28 of 132, *P value* < *0*.*001*) co-expression edges were found to have emerged in PS6–PS11 and PS12–PS19 (Fig. 2C).

### Evolution of functional modules for ovarian cancer

**Figure 3:**
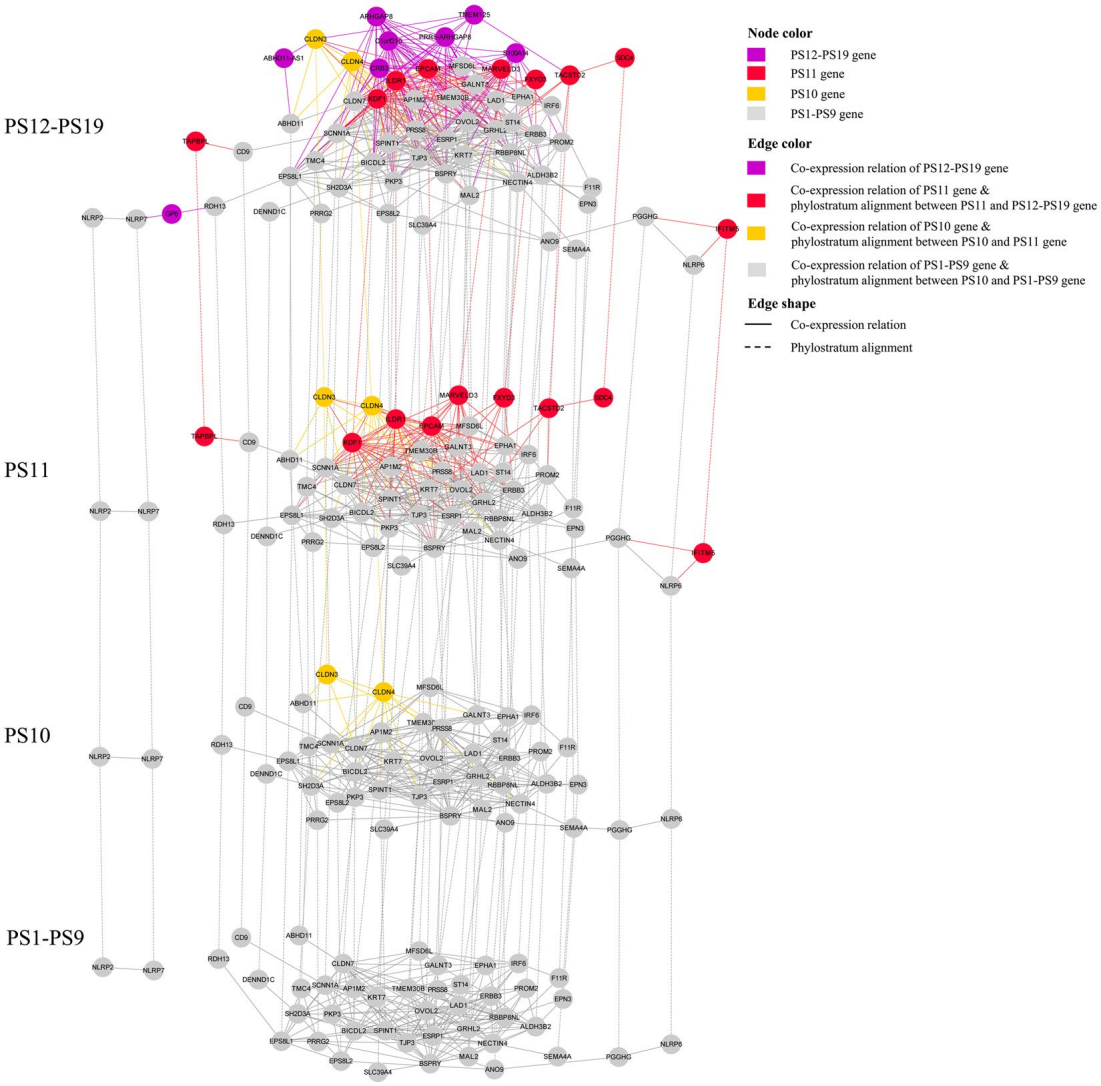
The phylostratigraphic alignment of co-expression relations’ constrictions in four periods: PS1–PS9, PS10, PS11 and PS12–PS19. Nodes in this module in different phylostratigraphic time-points represent genes, while edges depict two kinds of relations: the co-expressed relations between any two nodes inner one period and phylostratum alignment between genes from diverse phylogenetic time-ponits. Node colors depict gene’s origin time (phylostratigraphic time-points). Edge colors depict gene’s co-expression or phylostratigraphic alignment relation emergence time-points. Edge shapes depict genes’ relation type, the solid line indicate co-expression relation and long dash line means phylostratum alignment.

A total of 15 OV related modules were enriched for at least one phylostratum by hypergeometric test under the significance cutoff *P value* < 0.05 (Tables 2 and S7). For module M_11, 24 genes’ emergence were associated with the evolution of multicellularity (PS6: Metazoa, *P value* = 0.026) and 11 genes evolved accompanying the emergence of bony fish (PS11: Olfactores, *P value* = 0.001) (Table 2). The module M_184, which functioned in “type I interferon signaling pathway”, was uncovered to have been enriched in four phylostrata: “Bilateria” (PS8, *P value* = 0.021), “Olfactores” (PS11, *P value* < *0*.*001*), “Tetrapoda” (PS14, *P value* = 0.006), and “Eutheria” (PS17, *P value* = 0.036) (Tables 2 and S7). The significance of OV functional modules enriched in more than one phylostratum indicated the dynamic evolution process of them. For module M_144, three peaks of gene emergence were associated with the evolution of deuterostomia (PS9, *P value* = 0.004), chordata (PS10, *P value* = 0.007), olfactores (PS11, *P value < 0*.*001*), which indicated the three phylostrata as representing key points in time of evolution for this module. Figure 3 shows the phylostratigraphic alignment of co-expression relations’ constrictions of module M_144 in four periods: PS1–PS9, PS10, PS11 and PS12–PS19. The emergence of the claudin family members, CLDN3 and CLDN4 in “Chordata” (PS10), generated 14 co-expression relations to module M_144. These co-expressions may be associated with their OV related function, namely the ovarian tumor cell apoptosis resistance, migration and targeting extracellular loop interactions of claudin-4 may have therapeutic implications for reducing ovarian tumor burden (Fig. 3). A total of eight genes were discovered to have evolved in “Olfactores” (PS11, *P value* < *0*.*001*), including OV functional genes of epithelial cell adhesion molecule EPCAM and tumor associated calcium signal transducer TACSTD2. These genes brought 21.80% (87 of 399, *P value* < *0*.*001*) gene-genes relations to this module, which demonstrated the emergence of new genes and gene-gene cooperation in this phylostratum as crucial steps for functional construction of this module (Fig. 3).

**Table 2:** Phylostratigraphic enrichment result of 20 OV-related modules.

Module	Phylostratum	PS gene num.	Module gene num.	Overlap	Pval
M_11	PS6	1658	177	24	0.026
	PS11	394	177	11	0.001
M_25	PS3	445	81	7	0.003
	PS5	348	81	7	0.001
M_26	PS2	4915	177	59	0.025
	PS3	445	177	9	0.027
M_29	PS5	348	69	4	0.041
M_34	PS10	36	179	2	0.047
M_47	PS6	1658	83	13	0.032
M_91	PS16	341	72	5	0.010
M_102	PS1	7241	82	47	0.001
	PS6	1658	82	13	0.030
M_116	PS10	36	116	2	0.021
M_117	PS11	394	107	7	0.008
M_144	PS9	196	63	4	0.004
	PS10	36	63	2	0.007
	PS11	394	63	8	<0.001
M_147	PS11	394	63	9	<0.001
M_158	PS11	394	82	6	0.008
	PS14	112	82	3	0.013
M_184	PS8	67	61	2	0.021
	PS11	394	61	13	<0.001
	PS14	112	61	3	0.006
	PS17	91	61	2	0.036
M_194	PS7	680	45	5	0.024

Notes: PS gene num. = gene number of the phylostratum; Module gene num. = gene number of the module; Overlap = gene number of the overlap of the phylostratum and the module; Pval = *P value* of hypergeometric test for filtering OV-related modules.

## Discussion

Ovarian cancer is a deadly disease afflicting approximately 204,000 women worldwide each year^2,3,15^. Uncovering and understanding the dynamic evolution of functional modules and genes of OV in human, which can help to develop screening modalities, is an important step for dealing with the disease^2,15^. In this study, we discovered the functional modules from the co-expression networks for OV in human and demonstrated phylostratigraphic patterns of OV genes and modules. The following data showed the dynamic evolutionary process of OV in human: (1) the majority (67.45%) of OV genes was already present in the eukaryotic ancestor and there were two strong peaks of the emergence of OV genes screened by hypergeometric test, as the evolution of the multicellular metazoan organisms (PS5 and PS6, P value = 0.002) and the emergence of bony fish (PS11 and PS12, P value = 0.009); (2) the functional modules evolved at multiple time-points during human evolution.

For OV and other complicated traits, like diseases and immunodeficiency in animals and abiotic stress response in plants, a series of physical and biochemical mechanisms were recruited by organisms to respond to the damages caused by genes’ mutations, such as signal transduction, tissue/organ tolerance, macromolecular compound synthesis, membrane structure conversion and biochemistry homeostasis adjustment^2,15,18,64–83^. The new gene emergence phylogenetic time-points were demonstrated to correlate with two major classes of cancer genes: the “caretakers” of older phylostrata, like PS1–PS2, which participate in general functions that support genome stability; the “gatekeepers” of younger phylostrata, like PS6, PS11 and PS12, which are involved in cellular signaling and growth processes^52–54,84,85^ (Supplemental Table S8). Caretakers have evolved earlier and demonstrated as founder genes of cancers, their genome stability functions are of general importance for a cell and the origination of founder genes might correlate with functional novelty^53,54,59^. In this study, many “caretakers” genes participating in general functions that are essential for organism stability and OV were uncovered to be enriched in the original phylostrata. For example, “cell cycle arrest” (GO:0007050) is associated with growth inhibition of tumor cells by cyclin-dependent kinase inhibitors^86^. In this study, 73 OV marker genes were discovered to have emerged in the origin of the first cell (PS1 and PS2), including CCNE1-amplified high-grade serous ovarian cancer (HGSC) dependent kinase CDK2^87^ and the oncogenic capacity of advanced-stage ovarian cancer enhancing protein PRKAB1^88^. Same as most cancers, ovarian cancer is a mammal-specific disease. The phylostratigraphic enrichment of OV founder genes and their participating processes in the older phylostrata indicated the origin of OV as far earlier than its emergence.

Our analysis shows that there is a subset of OV genes that is directly connected to the emergence of multicellularity and a total of 173 OV related “gatekeepers” genes were discovered to have emerged in the “Metazoa” (PS6) phylostratum and enriched in multiple signaling and regulating processes. For instance, there are 14 members of “calcium-mediated signaling” (GO:0019722), 14 proteins of “positive regulation of protein kinase B signaling” (GO:0051897) and 16 genes for “positive regulation of ERK1 and ERK2 cascade” (GO:0070374). Gatekeepers are related to influencing cooperation among cells (oncogenes) or to prevent the expansion of cheater cells (tumor suppressor genes)^53,54,85^. One could predict that both of these gatekeeper functions would be necessary for stable multicellularity^53,54,89,90^ and should, therefore, have predominantly arisen at the time of the emergence of metazoa. Signal transduction, phosphorylation, dephosphorylation, acetylation, ubiquitination and transcriptional regulations are based on the generation of multicellularity and cooperation among cells, which are essential for evolution of OV and other complicated traits.

The biological traits have been verified to work as complex networks with characteristics of power-law-related distribution^39,40^. Genes involved in related biological processes tend to be co-expressed and cluster together as functional modules^40,49^. In this study, the co-expression network analysis was conducted to provide a global view of gene-gene interrelationships. A total of 20 HCCA topology co-expression modules were identified as OV associated ones (Tables 1 and S8). Functional co-expression modules are predicted as combinations of biological pathways^43,91^. The formation of founder genes has a specific role in the emerging gene networks^52,53,85^. The origin of such new genes seems to occur in a punctuated manner, that is, new genes initially evolve very quickly until they become locked into a pathway by interacting with older genes^53,85^. Our study uncovered the increasing recruitments of gene-gene co-expression edges in different phylostratigraphic time-points (Fig. 2C), which indicated older and younger genes are connected by edges representing gene duplications during evolution. The insertion of new genes into a network generated new gene-gene interaction, increased the complication of topological structure, and promoted combination of general biological processes and the younger signaling/regulating processes.

**Figure 4:**
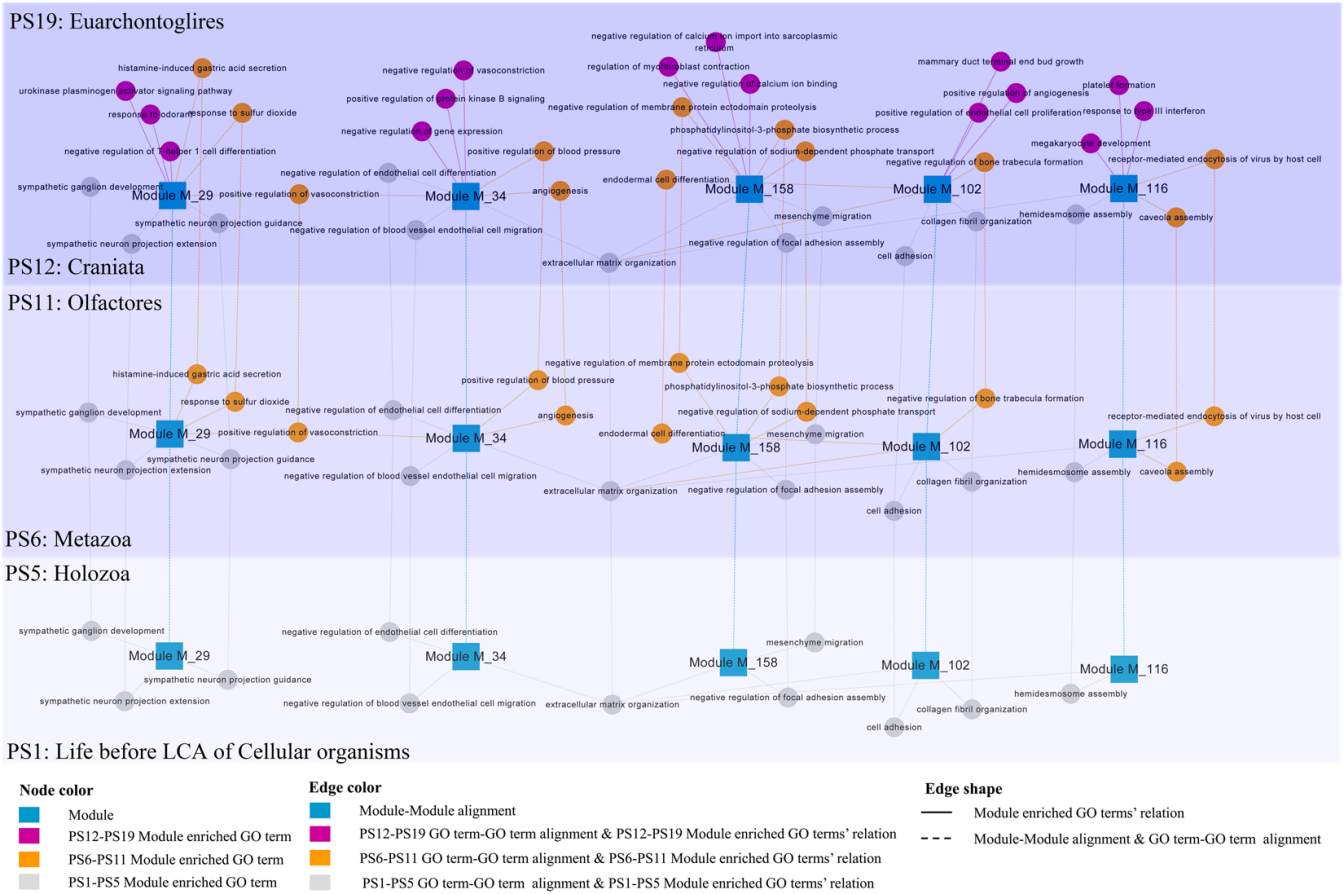
The dynamic evolutionary roles of biological processes in OV network by analyzing the top3 biological pathways enriched by five OV functional modules (M_29, M_34, M_102, M_116 and M_158) in three phylogenetic periods. The phylostratigraphic periods were depicted with different background colors: light blue for PS1–PS5, blue for PS6–PS11 and dark blue for PS12–PS19. Nodes represent genes or modules, while edges depict three kinds of relations: the co-expressed relations between any two nodes inner one period and phylostratum alignment between genes or modules from diverse phylogenetic time-points. Node colors depict gene’s origin time (phylostratigraphic time-points) or module. Edge colors depict gene’s co-expression or phylostratigraphic alignment relation emergence time-points. Edge shapes depict genes’ relation type, the solid lines indicate co-expression relation and long dash line means phylostratum alignment.

We further investigated the dynamic evolutionary roles of biological processes in OV network by analyzing the top 3 biological pathways enriched by five OV functional modules in three phylogenetic periods: PS1–PS5, PS6–PS11 and PS12–PS19 (Fig. 4, Supplemental Table S9). The result shows more and more biological pathways are fixed into OV modules by recruiting new genes into modules during human evolution. In the older phylogenetic period (PS1–PS5), the general biological processes emerged and composed the original functional core of each module, such as “cell adhesion” (GO:0007155) generated in module M_102 and “mesenchyme migration” recruited by module M_158. Surprisingly, several mammal specific or growth biological processes involved by “gatekeepers” genes were discovered to be fixed into OV modules in phylostrata PS1–PS5, like “sympathetic ganglion development” (GO:0061549) into module M_29 and “negative regulation of endothelial cell differentiation” into module M_34 (Fig. 4). The following are some possible reasons. There is an overlap within the classification of “gatekeepers” and “caretakers”, some genes were listed in both of these categories as they act as both caretakers and gatekeepers. Although the majority of “gatekeepers” families emerged in the “Metazoa” (PS6) phylostratum, some of them originated in older phylostrata. Furthermore, the emergence/origin of some biological pathways is earlier than their accomplishment due to the diverse emergence time-points of their members.

The recruitment of signaling and regulating processes into OV network based on integration of “gatekeepers” genes in younger phylostrata are essential for OV trait accomplishment. As shown in Fig. 4, module M_158 recruited ovarian tumor-associated antigen regulating process “negative regulation of sodium-dependent phosphate transport” (GO:2000119) in PS6–PS11, and fixed ovarian cancer myofibroblast differentiation^92^ associated process “regulation of myofibroblast contraction” (GO:1904328) in PS12–PS19. The evolution of single functional module contributes to accomplishing complex networks of ovarian cancer. However, modules should cooperate together to participate in OV. In our study, the increasing emergence of module-module relations via recruiting the same biological processes were discovered during human evolution. As shown in Fig. 4, MAPK (mitogen-activated protein kinases) related process in metastatic serous ovarian carcinoma^93^, “extracellular matrix organization” (GO:0030198) was recruited by four modules in older phylostrata PS1–PS5: M_34, M_102, M_116 and M_158. The ovarian tumor related process^94^ “regulation of receptor activity” (GO:0010469) connected module M_11 and M_194 in phylostrata PS6–PS10, and connected module M_11 and M_84 in phylostrata PS12–PS19. Our findings have some evolutionary implications on macro-evolutionary trends of ovarian cancer, traditionally studied by fossil analysis^65^. The ancient origin of OV genes and their cooperators (founder genes) suggests that they were involved in older biological processes. The emergence of these founder genes and fundamental molecular processes composed the foundation of OV, based on which, new genes, gene-gene interactions and the signaling/regulating biological processes were recruited to the functional network of OV step by step during human evolution.

## Conclusions

We performed a phylostratigraphic analysis of genes and the gene co-expression network of ovarian cancer (OV) in human. The co-expression network construction, which was further divided into 208 co-expressed clusters, yielded 18,549 nodes and 114,985 edges with an HRR coefficient of 30. A total of 20 modules were identified as OV related clusters by hypergeometric test. The human genome sequences were divided into 19 phylostrata. The majority of OV genes was already present in the eukaryotic ancestor. There were two strong peaks of the emergence of ovarian cancer genes screened by hypergeometric test, including the evolution of the multicellular metazoan organisms (PS5 and PS6, *P value* = 0.002) and the emergence of bony fish (PS11 and PS12, *P value* = 0.009). Hence, the origin of OV is far earlier than its emergence. The integrated analysis of the topology of OV modules and the phylogenetic data revealed an evolutionary pattern of OV in human: OV modules have arisen step by step during the evolution of the respective lineages. New genes have evolved and become locked into a pathway and more and more biological pathways are fixed into OV modules by recruiting new genes into modules during human evolution.

## Methods

### Gene expression dataset download and literature search

The ovarian cancer (OV) expression profiling samples of human were obtained from the Genome Data Analysis Centers (GDAC) (http://gdac.broadinstitute.org/runs/stddata__2016_01_28/), which integrates data generated by The Cancer Genome Atlas (TCGA) Research Network. Study Inclusions strictly followed the criteria for dataset selection, including human participant’s ovarian tumor studies, gene expression profiling, complete raw and processed RNA_seq data. Non-human studies, non-ovarian tumor studies, RT-PCR generated profiling studies, review articles, bioinformatics and integrated analysis of expression profiles were excluded. The screened and calculated expression signal matrix file of genome genes for participant’s ovarian tumor samples were downloaded from the GDAC database (gdac.broadinstitute.org_OV.Merge_rnaseqv2__illuminahiseq_rnaseqv2__unc_edu__Level_3__RSEM_genes_normalized__data.Level_3.2016012800.0.0.tar.gz).

PubMed database (https://www.ncbi.nlm.nih.gov/pubmed) was systematically searched for mining functional verified OV marker genes in human. The following key words and their combinations were used: ((((((homo sapiens) OR human)) AND (((((((((((ovarian tumor) OR ovarian cancer) OR ovarian serous cystadenocarcinoma) OR ovarian carcinoma) OR serous ovarian carcinoma) OR serous ovarian cancer) OR ovarian serous carcinoma) OR serous epithelial ovarian cancer) OR epithelial ovarian cancer) OR ovarian carcinoma) OR ovarian malignancy))))) AND ((gene) OR protein)). The latest search was performed on May 31, 2018. Then, the relationships between relevant publications and genes were collected from the gene2pubmed database (ftp://ftp.ncbi.nlm.nih.gov/gene/DATA/gene2pubmed.gz). Publications that were not included in the gene2pubmed database were excluded. The remaining publications were further manually screened to filter articles with empirical evidence of the functional significance of OV genes in *Homo sapiens*. The identified genes were treated as functional verified OV marker genes.

### Co-expression network construction and OV module detection

Pearson-based co-expression network was constructed by using cor() of R based on the combined expression matrix generated by batch effect correction analysis. The Pearson correlation coefficient (PCC) distribution was calculated based on t-distribution significance tests. The Highest-reciprocal rank (HRR) score between genes A and B was calculated according to: HRR(A, B) = max(rank(A, B), rank(B, A)), where rank(A, B) is correlation rank of gene B in gene A’s co-expression list. The criteria of cutoff HRR ≤ 30 was used for HRR based co-expression network^63^. Heuristic Cluster Chiseling Algorithm (HCCA) was used to detect the modules in the HRR co-expression networks using default parameters as follows: step = 3; sizeRange = 40, 200^95^. The co-expression networks were visualized using Cytoscope 2.8.2.

In this study, the statistical methods published by Ruprecht^96^ were referenced for testing the OV related modules, the enrichment of OV genes in twelve phylostrata and phylostrata enrichment within modules. The hypergeometric test was conducted by phyper () of R version 3.4.4. For hypergeometric test of the OV related modules, m is the members’ number of each module, n indicates the OV marker genes’ number, k represents the number of the module members which were classified as OV marker genes, and N is the total genes’ number of the co-expression network. The Benjamini-Hochberg procedure^97^ at a significance level of 0.05 was used for hypothesis testing.

### Phylostratigraphic analysis

The phylostratigraphic method was designed to uncover the evolutionary origin of gene families, giving the opportunity to understand the age of the studied genes and their common ancestor^53,54^. The phylostratigraphic map of *H*. *sapiens* was downloaded from the article published by Domazet-Lošo and Diethard Tautz^53,54^. The Ensembl IDs were converted into Entrnz gene IDs according to the mapping data supplied by the NCBI database. Phylostrata enrichment of modules (including both genes and gene-genes’ co-expressions) was calculated using hypergeometric distribution in R. This method estimates the probability of obtaining k successes (i.e. the number of specific phylostrata [genes/co-expressions] in a module) in m draws (i.e. the number of genes/co-expressions in a module), from a finite population of size N (i.e. number of genes/co-expressions in the genome) that contains exactly n successes (i.e. number of genes/co-expressions the phylostratum of interest is found in the genome). The calculating and correcting methods were the same as the hypergeometric test used for filtering the OV related modules.

### Enrichment analysis

The package TopGO of R 3.3.2^98^ was used in enrichment analysis for gene ontology (GO) of the studied genes, including OV marker genes and the gene members of each module. The GO functional annotations file of *H*. *sapiens* was downloaded from Gene Ontology database (submission date: May, 2018, http://geneontology.org/gene-associations/goa_human_rna.gaf.gz). REVIGO (http:/revigo.irb.hr/) was selected to summarize the long, unintelligible lists of GO terms for the enrichment^99^. The DAVID database (https://david.ncifcrf.gov/) was used for gene information retrieving, gene id conversion and gene function annotation.

The experimental protocols of this study were approved by Shandong Normal University and Shandong Academy of Medical Sciences. All the methods used in this study were performed in accordance with appropriate guidelines, and the relevant publications were cited.

## Supplementary information

Supplementary Tables

## Data Availability

We declare that the all data and materials of this manuscript including the supplementary datasets are available to the journal and all readers.
