# Supplementary material for: Phylostratigraphic analysis of gene co-expression network reveals the evolution of functional modules for ovarian cancer
Source: Sci Rep. 2019 Feb 22;9:2623. doi: 10.1038/s41598-019-40023-9 (PMC6384884; doi:10.1038/s41598-019-40023-9)
Supplement: Supplementary file 1 — Supplementary Tables [file 41598_2019_40023_MOESM1_ESM.pdf]

Title: Phylostratigraphic analysis of gene co-expression network reveals the evolution of functional modules for ovarian cancer
Authers: Luoyan Zhang, Yi Tan, Shoujin Fan, Xuejie Zhang and Zhen Zhang*

Table S1 Information of 307 ovarian cancer (OV) expression profiling samples

**Sample ID**
TCGA-04-1348-01A-01R-1565-13
TCGA-04-1357-01A-01R-1565-13
TCGA-04-1362-01A-01R-1565-13
TCGA-04-1364-01A-01R-1565-13
TCGA-04-1365-01A-01R-1565-13
TCGA-04-1514-01A-01R-1566-13
TCGA-04-1519-01A-01R-1565-13
TCGA-09-0364-01A-02R-1564-13
TCGA-09-0366-01A-01R-1564-13
TCGA-09-0367-01A-01R-1564-13
TCGA-09-0369-01A-01R-1564-13
TCGA-09-1659-01B-01R-1564-13
TCGA-09-1662-01A-01R-1566-13
TCGA-09-1666-01A-01R-1566-13
TCGA-09-1667-01C-01R-1566-13
TCGA-09-1668-01B-01R-1566-13
TCGA-09-1669-01A-01R-1566-13
TCGA-09-1670-01A-01R-1566-13
TCGA-09-1673-01A-01R-1566-13
TCGA-09-1674-01A-01R-1566-13
TCGA-09-2044-01B-01R-1568-13
TCGA-09-2045-01A-01R-1568-13
TCGA-09-2048-01A-01R-1568-13
TCGA-09-2051-01A-01R-1568-13
TCGA-09-2054-01A-01R-1568-13
TCGA-09-2056-01B-01R-1568-13
TCGA-10-0928-01A-02R-1564-13

TCGA-10-0936-01A-01R-1564-13
TCGA-13-0730-01A-01R-1564-13
TCGA-13-0799-01A-01R-1564-13
TCGA-13-0800-01A-01R-1564-13
TCGA-13-0801-01A-01R-1564-13
TCGA-13-0890-01A-01R-1564-13
TCGA-13-0893-01B-01R-1565-13
TCGA-13-0897-01A-01R-1564-13
TCGA-13-0899-01A-01R-1564-13
TCGA-13-0913-01A-01R-1564-13
TCGA-13-0916-01A-01R-1564-13
TCGA-13-0920-01A-01R-1564-13
TCGA-13-0924-01A-01R-1564-13
TCGA-13-1403-01A-01R-1565-13
TCGA-13-1405-01A-01R-1565-13
TCGA-13-1410-01A-01R-1565-13
TCGA-13-1411-01A-01R-1565-13
TCGA-13-1481-01A-01R-1565-13
TCGA-13-1489-02A-01R-1565-13
TCGA-13-1497-01A-01R-1565-13
TCGA-13-1498-01A-01R-1565-13
TCGA-13-1505-01A-01R-1565-13
TCGA-13-1506-01A-01R-1565-13
TCGA-13-1507-01A-01R-1565-13
TCGA-13-1511-01A-01R-1565-13
TCGA-13-1512-01A-01R-1565-13
TCGA-13-2060-01A-01R-1568-13
TCGA-13-A5FT-01A-11R-A406-31
TCGA-20-1682-01A-01R-1564-13
TCGA-20-1683-01A-01R-1566-13
TCGA-20-1684-01A-01R-1566-13
TCGA-20-1685-01A-01R-1566-13

TCGA-20-1687-01A-01R-1566-13
TCGA-23-1023-01R-01R-1564-13
TCGA-23-1026-01B-01R-1569-13
TCGA-23-1027-01A-02R-1564-13
TCGA-23-1029-01B-01R-1567-13
TCGA-23-1109-01A-01R-1564-13
TCGA-23-1111-01A-01R-1567-13
TCGA-23-1114-01B-01R-1566-13
TCGA-23-1120-01A-02R-1565-13
TCGA-23-1122-01A-01R-1565-13
TCGA-23-1123-01A-01R-1565-13
TCGA-23-1809-01A-01R-1566-13
TCGA-23-2077-01A-01R-1568-13
TCGA-23-2078-01A-01R-1568-13
TCGA-23-2081-01A-01R-1568-13
TCGA-23-2084-01A-02R-1568-13
TCGA-24-0975-01A-02R-1565-13
TCGA-24-1103-01A-01R-1565-13
TCGA-24-1413-01A-01R-1565-13
TCGA-24-1416-01A-01R-1565-13
TCGA-24-1417-01A-01R-1565-13
TCGA-24-1418-01A-01R-1565-13
TCGA-24-1419-01A-01R-1565-13
TCGA-24-1423-01A-01R-1565-13
TCGA-24-1424-01A-01R-1565-13
TCGA-24-1425-01A-02R-1566-13
TCGA-24-1427-01A-01R-1565-13
TCGA-24-1428-01A-01R-1564-13
TCGA-24-1430-01A-01R-1566-13
TCGA-24-1434-01A-01R-1566-13
TCGA-24-1435-01A-01R-1566-13
TCGA-24-1436-01A-01R-1566-13

TCGA-24-1463-01A-01R-1566-13
TCGA-24-1467-01A-01R-1566-13
TCGA-24-1469-01A-01R-1566-13
TCGA-24-1474-01A-01R-1566-13
TCGA-24-1544-01A-01R-1566-13
TCGA-24-1545-01A-01R-1566-13
TCGA-24-1546-01A-01R-1566-13
TCGA-24-1548-01A-01R-1566-13
TCGA-24-1549-01A-01R-1566-13
TCGA-24-1550-01A-01R-1566-13
TCGA-24-1551-01A-01R-1566-13
TCGA-24-1552-01A-01R-1566-13
TCGA-24-1553-01A-01R-1566-13
TCGA-24-1555-01A-01R-1566-13
TCGA-24-1556-01A-01R-1566-13
TCGA-24-1557-01A-01R-1566-13
TCGA-24-1558-01A-01R-1566-13
TCGA-24-1560-01A-01R-1566-13
TCGA-24-1562-01A-01R-1566-13
TCGA-24-1563-01A-01R-1566-13
TCGA-24-1564-01A-01R-1566-13
TCGA-24-1567-01A-01R-1566-13
TCGA-24-1603-01A-01R-1566-13
TCGA-24-1604-01A-01R-1566-13
TCGA-24-1616-01A-01R-1566-13
TCGA-24-1842-01A-01R-1567-13
TCGA-24-1843-01A-01R-1567-13
TCGA-24-1844-01A-01R-1567-13
TCGA-24-1846-01A-01R-1567-13
TCGA-24-1847-01A-01R-1566-13
TCGA-24-1849-01A-01R-1567-13
TCGA-24-1850-01A-01R-1567-13

TCGA-24-1924-01A-01R-1567-13
TCGA-24-1928-01A-01R-1567-13
TCGA-24-1930-01A-01R-1567-13
TCGA-24-2019-01A-02R-1568-13
TCGA-24-2020-01A-01R-1567-13
TCGA-24-2023-01A-01R-1567-13
TCGA-24-2024-01A-02R-1568-13
TCGA-24-2026-01A-01R-1567-13
TCGA-24-2027-01A-01R-1567-13
TCGA-24-2033-01A-01R-1568-13
TCGA-24-2035-01A-01R-1568-13
TCGA-24-2036-01A-01R-1568-13
TCGA-24-2038-01A-01R-1568-13
TCGA-24-2254-01A-01R-1568-13
TCGA-24-2261-01A-01R-1568-13
TCGA-24-2262-01A-01R-1568-13
TCGA-24-2267-01A-01R-1568-13
TCGA-24-2271-01A-01R-1568-13
TCGA-24-2281-01A-01R-1568-13
TCGA-24-2288-01A-01R-1568-13
TCGA-24-2289-01A-01R-1568-13
TCGA-24-2290-01A-01R-1568-13
TCGA-24-2293-01A-01R-1568-13
TCGA-24-2297-01A-01R-1568-13
TCGA-24-2298-01A-01R-1569-13
TCGA-25-1312-01A-01R-1565-13
TCGA-25-1313-01A-01R-1565-13
TCGA-25-1314-01A-01R-1565-13
TCGA-25-1315-01A-01R-1565-13
TCGA-25-1316-01A-01R-1565-13
TCGA-25-1317-01A-01R-1565-13
TCGA-25-1318-01A-01R-1565-13

TCGA-25-1319-01A-01R-1565-13
TCGA-25-1320-01A-01R-1565-13
TCGA-25-1321-01A-01R-1565-13
TCGA-25-1322-01A-01R-1565-13
TCGA-25-1323-01A-01R-1565-13
TCGA-25-1324-01A-01R-1565-13
TCGA-25-1326-01A-01R-1565-13
TCGA-25-1328-01A-01R-1565-13
TCGA-25-1329-01A-01R-1565-13
TCGA-25-1623-01A-01R-1566-13
TCGA-25-1625-01A-01R-1566-13
TCGA-25-1626-01A-01R-1566-13
TCGA-25-1627-01A-01R-1566-13
TCGA-25-1628-01A-01R-1566-13
TCGA-25-1630-01A-01R-1566-13
TCGA-25-1631-01A-01R-1569-13
TCGA-25-1632-01A-01R-1566-13
TCGA-25-1633-01A-01R-1566-13
TCGA-25-1634-01A-01R-1566-13
TCGA-25-1635-01A-01R-1566-13
TCGA-25-1870-01A-01R-1567-13
TCGA-25-1871-01A-01R-1567-13
TCGA-25-2042-01A-01R-1568-13
TCGA-25-2391-01A-01R-1569-13
TCGA-25-2392-01A-01R-1569-13
TCGA-25-2393-01A-01R-1569-13
TCGA-25-2396-01A-01R-1569-13
TCGA-25-2397-01A-01R-1569-13
TCGA-25-2398-01A-01R-1569-13
TCGA-25-2399-01A-01R-1569-13
TCGA-25-2400-01A-01R-1569-13
TCGA-25-2401-01A-01R-1569-13

TCGA-25-2404-01A-01R-1569-13
TCGA-25-2409-01A-01R-1569-13
TCGA-29-1688-01A-01R-1566-13
TCGA-29-1690-01A-01R-1566-13
TCGA-29-1691-01A-01R-1566-13
TCGA-29-1693-01A-01R-1567-13
TCGA-29-1694-01A-01R-1567-13
TCGA-29-1695-01A-01R-1567-13
TCGA-29-1696-01A-01R-1567-13
TCGA-29-1697-01A-01R-1567-13
TCGA-29-1698-01A-01R-1567-13
TCGA-29-1699-01A-01R-1567-13
TCGA-29-1701-01A-01R-1567-13
TCGA-29-1702-01A-01R-1567-13
TCGA-29-1703-01A-01R-1567-13
TCGA-29-1705-01A-01R-1567-13
TCGA-29-1710-01A-02R-1567-13
TCGA-29-1711-01A-01R-1567-13
TCGA-29-1761-01A-01R-1567-13
TCGA-29-1762-01A-01R-1567-13
TCGA-29-1763-01A-02R-1567-13
TCGA-29-1770-01A-01R-1567-13
TCGA-29-1770-02A-01R-1567-13
TCGA-29-1776-01A-01R-1567-13
TCGA-29-1778-01A-01R-1567-13
TCGA-29-1781-01A-01R-1567-13
TCGA-29-1783-01A-01R-1567-13
TCGA-29-1784-01A-02R-1567-13
TCGA-29-1785-01A-01R-1567-13
TCGA-29-2414-01A-02R-1569-13
TCGA-29-2414-02A-01R-1569-13
TCGA-29-2425-01A-01R-1569-13

TCGA-29-2427-01A-01R-1569-13
TCGA-29-2428-01A-01R-1569-13
TCGA-29-A5NZ-01A-11R-A406-31
TCGA-30-1718-01A-01R-1567-13
TCGA-30-1855-01A-01R-1567-13
TCGA-30-1857-01A-02R-1569-13
TCGA-30-1860-01A-01R-1568-13
TCGA-30-1861-01A-01R-1568-13
TCGA-30-1862-01A-02R-1568-13
TCGA-30-1891-01A-01R-1568-13
TCGA-31-1944-01A-01R-1568-13
TCGA-31-1946-01A-01R-1568-13
TCGA-31-1951-01A-01R-1568-13
TCGA-31-1953-01A-01R-1568-13
TCGA-31-1956-01A-01R-1568-13
TCGA-31-1959-01A-01R-1568-13
TCGA-36-1568-01A-01R-1566-13
TCGA-36-1569-01A-01R-1566-13
TCGA-36-1570-01A-01R-1566-13
TCGA-36-1571-01A-01R-1566-13
TCGA-36-1574-01A-01R-1566-13
TCGA-36-1575-01A-01R-1566-13
TCGA-36-1576-01A-01R-1566-13
TCGA-36-1577-01A-01R-1566-13
TCGA-36-1578-01A-01R-1566-13
TCGA-36-1580-01A-01R-1566-13
TCGA-36-1581-01A-01R-1566-13
TCGA-3P-A9WA-01A-11R-A406-31
TCGA-57-1582-01A-01R-1566-13
TCGA-57-1583-01A-01R-1566-13
TCGA-57-1584-01A-01R-1566-13
TCGA-57-1585-01A-01R-1566-13

TCGA-57-1993-01A-01R-1568-13
TCGA-57-1994-01A-01R-1568-13
TCGA-59-2348-01A-01R-1569-13
TCGA-59-2350-01A-01R-1569-13
TCGA-59-2351-01A-01R-1569-13
TCGA-59-2352-01A-01R-1569-13
TCGA-59-2354-01A-01R-1569-13
TCGA-59-2355-01A-01R-1569-13
TCGA-59-2363-01A-01R-1569-13
TCGA-59-A5PD-01A-11R-A406-31
TCGA-5X-AA5U-01A-11R-A406-31
TCGA-61-1721-01A-01R-1569-13
TCGA-61-1724-01A-01R-1568-13
TCGA-61-1725-01A-01R-1567-13
TCGA-61-1728-01A-01R-1568-13
TCGA-61-1733-01A-01R-1567-13
TCGA-61-1736-01B-01R-1568-13
TCGA-61-1737-01A-01R-1567-13
TCGA-61-1738-01A-01R-1567-13
TCGA-61-1740-01A-01R-1567-13
TCGA-61-1741-01A-02R-1567-13
TCGA-61-1743-01A-01R-1568-13
TCGA-61-1900-01A-01R-1567-13
TCGA-61-1907-01A-01R-1567-13
TCGA-61-1910-01A-01R-1567-13
TCGA-61-1914-01A-01R-1567-13
TCGA-61-1917-01A-01R-1568-13
TCGA-61-1918-01A-01R-1568-13
TCGA-61-1919-01A-01R-1568-13
TCGA-61-1995-01A-01R-1568-13
TCGA-61-1998-01A-01R-1568-13
TCGA-61-2000-01A-01R-1568-13

TCGA-61-2002-01A-01R-1568-13
TCGA-61-2003-01A-01R-1568-13
TCGA-61-2008-01A-02R-1568-13
TCGA-61-2008-02A-01R-1568-13
TCGA-61-2009-01A-01R-1568-13
TCGA-61-2012-01A-01R-1568-13
TCGA-61-2016-01A-01R-1568-13
TCGA-61-2088-01A-01R-1568-13
TCGA-61-2092-01A-01R-1568-13
TCGA-61-2094-01A-01R-1568-13
TCGA-61-2095-01A-01R-1568-13
TCGA-61-2097-01A-02R-1568-13
TCGA-61-2098-01A-01R-1568-13
TCGA-61-2101-01A-01R-1568-13
TCGA-61-2102-01A-01R-1568-13
TCGA-61-2104-01A-01R-1568-13
TCGA-61-2109-01A-01R-1568-13
TCGA-61-2110-01A-01R-1568-13
TCGA-61-2111-01A-01R-1568-13
TCGA-61-2113-01A-01R-1568-13
TCGA-OY-A56P-01A-12R-A406-31
TCGA-OY-A56Q-01A-11R-A406-31
TCGA-VG-A8LO-01A-11R-A406-31
TCGA-WR-A838-01A-12R-A406-31

Table S2 Information of OV marker genes

**Gene ID**	**Gene Name**	**GAD_DISEASE Gene**	**PMCID**
799	calcitonin receptor(CALCR)	Yes	1331173
961	CD47 molecule(CD47)	Yes	1394148
6765	suppression of tumorigenicity 8 (ovarian)(ST8)	No	1394208
208	AKT serine/threonine kinase 2(AKT2)	Yes	1409633
672	BRCA1, DNA repair associated(BRCA1)	Yes	1676470

2348	folate receptor 1(FOLR1)	Yes	1717147
5741	parathyroid hormone(PTH)	Yes	2215618
2064	erb-b2 receptor tyrosine kinase 2(ERBB2)	Yes	2470152
251	alkaline phosphatase, placental like 2(ALPPL2)	Yes	2921344
8909	endonuclease, poly(U) specific(ENDOU)	Yes	6755403
3487	insulin like growth factor binding protein	Yes	7507078
3857	keratin 9(KRT9)	No	7511021
2798	gonadotropin releasing hormone	Yes	7534732
80755	alanyl-tRNA synthetase domain containing 1(AARSD1)	No	7545954
100885850	PTGES3L-AARSD1 readthrough(PTGES3L-AARSD1)	No	7545954
10493	vesicle amine transport 1(VAT1)	No	7545954
4077	NBR1, autophagy cargo receptor(NBR1)	Yes	7545954
379	ADP ribosylation factor like GTPase	No	7545954
6155	ribosomal protein L27(RPL27)	No	7545954
10197	proteasome activator subunit 3(PSME3)	No	7545954
653361	neutrophil cytosolic factor 1(NCF1)	Yes	7545954
3728	junction plakoglobin(JUP)	Yes	7604000
7982	suppression of tumorigenicity 7(ST7)	Yes	7624150
2321	fms related tyrosine kinase 1(FLT1)	Yes	7707437
3934	lipocalin 2(LCN2)	Yes	7835423
780	discoidin domain receptor tyrosine kinase	Yes	7848919
83953	Fc fragment of IgA and IgM receptor(FCAMR)	Yes	8145026
10495	ecto-NOX disulfide-thiol exchanger 2(ENOX2)	No	8150545
5155	platelet derived growth factor subunit	Yes	8174076
1601	DAB2, clathrin adaptor protein(DAB2)	Yes	8314147
11259	filamin A interacting protein 1 like(FILIP1L)	Yes	8314147
10232	mesothelin(MSLN)	Yes	8552591
2192	fibulin 1(FBLN1)	Yes	8552629
6412	senescence (cellular)-related 6A(SEN6A)	No	8570202
4301	afadin, adherens junction formation	Yes	8589040

1801	diphthamide biosynthesis 1(DPH1)	No	8603384
79073	transmembrane protein 109(TMEM109)	No	8619474
675	BRCA2, DNA repair associated(BRCA2)	Yes	8640235
3726	JunB proto-oncogene, AP-1 transcription factor subunit(JUNB)	Yes	8649827
6259	receptor-like tyrosine kinase(RYK)	Yes	8726462
5653	kallikrein related peptidase 6(KLK6)	Yes	8898378
2492	follicle stimulating hormone receptor(FSHR)	Yes	9100567
1956	epidermal growth factor receptor(EGFR)	Yes	9103388
5328	plasminogen activator, urokinase(PLAU)	Yes	9194591
5054	serpin family E member 1(SERPINE1)	Yes	9194591
2099	estrogen receptor 1(ESR1)	Yes	9195227
3249	hepsin(HPN)	Yes	9230195
8202	nuclear receptor coactivator 3(NCOA3)	Yes	9252329
2067	ERCC excision repair 1, endonuclease non-catalytic subunit(ERCC1)	Yes	9360634
580	BRCA1 associated RING domain 1(BARD1)	Yes	9425226
5728	phosphatase and tensin homolog(PTEN)	Yes	9439675
2100	estrogen receptor 2(ESR2)	Yes	9506768
2778	GNAS complex locus(GNAS)	Yes	9626141
2065	erb-b2 receptor tyrosine kinase 3(ERBB3)	Yes	9681822
572	BCL2 associated agonist of cell death(BAD)	Yes	9824152
581	BCL2 associated X, apoptosis regulator(BAX)	Yes	9824152
598	BCL2 like 1(BCL2L1)	Yes	9824152
9077	DIRAS family GTPase 3(DIRAS3)	Yes	9874798
5290	phosphatidylinositol-4,5-bisphosphate 3-kinase catalytic subunit alpha(PIK3CA)	Yes	9916799
102723547	CSAG family member 2(CSAG2)	No	10095106
389903	CSAG family member 3(CSAG3)	No	10095106
5793	protein tyrosine phosphatase, receptor type G(PTPRG)	Yes	10376795
653483	AFDN antisense RNA 1 (head to head)(AFDN-	No	10382971

100505879	KIF25 antisense RNA 1(KIF25-AS1)	No	10382971
26238	long intergenic non-protein coding RNA 1558(LINC01558)	No	10382971
100128124	uncharacterized LOC100128124(HGC6.3)	Yes	10382971
1499	catenin beta 1(CTNNB1)	Yes	10391090
866	serpin family A member 6(SERPINA6)	Yes	10436419
11202	kallikrein related peptidase 8(KLK8)	Yes	10485494
7161	tumor protein p73(TP73)	Yes	10509157
11045	uroplakin 1A(UPK1A)	Yes	10514386
7348	uroplakin 1B(UPK1B)	No	10514386
7380	uroplakin 3A(UPK3A)	Yes	10514386
7379	uroplakin 2(UPK2)	No	10514386
5747	protein tyrosine kinase 2(PTK2)	Yes	10526262
9170	lysophosphatidic acid receptor 2(LPAR2)	No	10537322
1903	sphingosine-1-phosphate receptor 3(S1PR3)	Yes	10537322
10406	WAP four-disulfide core domain 2(WFDC2)	Yes	10570965
269	anti-Mullerian hormone receptor type	Yes	10589763
5519	protein phosphatase 2 scaffold subunit Abeta(PPP2R1B)	Yes	10597236
6347	C-C motif chemokine ligand 2(CCL2)	Yes	10623817
729230	C-C motif chemokine receptor 2(CCR2)	Yes	10623817
2776	G protein subunit alpha q(GNAQ)	Yes	10633085
2773	G protein subunit alpha i3(GNAI3)	Yes	10633085
4067	LYN proto-oncogene, Src family tyrosine kinase(LYN)	Yes	10633085
10801	septin 9(SEPT9)	Yes	10673329
4829	neuromedin B receptor(NMBR)	Yes	10828496
2796	gonadotropin releasing hormone 1(GNRH1)	Yes	10832105
2252	fibroblast growth factor 7(FGF7)	Yes	11000522
59338	pleckstrin homology domain containing A1(PLEKHA1)	Yes	11001876
60436	TGFB induced factor homeobox 2(TGIF2)	No	11006116

1445	c-src tyrosine kinase(CSK)	Yes	11084024
960	CD44 molecule (Indian blood group)(CD44)	Yes	11084024
6714	SRC proto-oncogene, non-receptor tyrosine kinase(SRC)	Yes	11084024
2530	fucosyltransferase 8(FUT8)	Yes	11093814
4524	methylenetetrahydrofolate reductase(MTHFR)	Yes	11094304
27102	eukaryotic translation initiation factor 2 alpha kinase 1(EIF2AK1)	Yes	11101152
1312	catechol-O-methyltransferase(COMT)	Yes	11142424
2944	glutathione S-transferase mu 1(GSTM1)	Yes	11159742
2950	glutathione S-transferase pi 1(GSTP1)	Yes	11159743
2952	glutathione S-transferase theta 1(GSTT1)	Yes	11159743
54940	OCIA domain containing 1(OCIAD1)	Yes	11162530
81037	CLPTM1 like(CLPTM1L)	Yes	11162647
25818	kallikrein related peptidase 5(KLK5)	Yes	11237385
2052	epoxide hydrolase 1(EPHX1)	Yes	11255266
1545	cytochrome P450 family 1 subfamily B member 1(CYP1B1)	Yes	11303589
8635	ribonuclease T2(RNASET2)	Yes	11314033
5241	progesterone receptor(PGR)	Yes	11323389
56648	eukaryotic translation initiation factor	Yes	11325856
51447	inositol hexakisphosphate kinase 2(IP6K2)	No	11337497
3456	interferon beta 1(IFNB1)	Yes	11337497
10855	heparanase(HPSE)	Yes	11351242
29103	DnaJ heat shock protein family (Hsp40) member C15(DNAJC15)	Yes	11358853
94025	mucin 16, cell surface associated(MUC16)	Yes	11369781
574048	Ovarian cancer, epithelial, susceptibility	No	11440995
722	complement component 4 binding protein alpha(C4BPA)	Yes	11441101
58506	SR-related CTD associated factor 1(SCAF1)	Yes	11461075
650	bone morphogenetic protein 2(BMP2)	Yes	11497252

2885	growth factor receptor bound protein 2(GRB2)	Yes	11606575
7410	vav guanine nucleotide exchange factor	Yes	11606575
284366	kallikrein related peptidase 9(KLK9)	Yes	11691797
4914	neurotrophic receptor tyrosine kinase 1(NTRK1)	Yes	11705863
8312	axin 1(AXIN1)	Yes	11719457
1029	cyclin dependent kinase inhibitor 2A(CDKN2A)	Yes	11733969
1026	cyclin dependent kinase inhibitor 1A(CDKN1A)	Yes	11733969
11200	checkpoint kinase 2(CHEK2)	Yes	11746983
56647	BRCA2 and CDKN1A interacting	Yes	11748848
7422	vascular endothelial growth factor A(VEGFA)	Yes	11748975
4313	matrix metallopeptidase 2(MMP2)	Yes	11748988
7157	tumor protein p53(TP53)	Yes	11751427
6117	replication protein A1(RPA1)	Yes	11751427
6716	steroid 5 alpha-reductase 2(SRD5A2)	Yes	11751447
11012	kallikrein related peptidase 11(KLK11)	Yes	11782391
8647	ATP binding cassette subfamily B member 11(ABCB11)	Yes	11783115
5359	phospholipid scramblase 1(PLSCR1)	No	11809687
2113	ETS proto-oncogene 1, transcription	Yes	11836635
841	caspase 8(CASP8)	Yes	11865194
7046	transforming growth factor beta receptor	Yes	11867510
3685	integrin subunit alpha V(ITGAV)	Yes	11872628
2797	gonadotropin releasing hormone 2(GNRH2)	Yes	11889221
5743	prostaglandin-endoperoxide synthase 2(PTGS2)	Yes	11891188
4085	MAD2 mitotic arrest deficient-like 1 (yeast)(MAD2L1)	Yes	11912137
5655	kallikrein related peptidase 10(KLK10)	Yes	11920956
6696	secreted phosphoprotein 1(SPP1)	Yes	11926891
7490	Wilms tumor 1(WT1)	Yes	11939727
367	androgen receptor(AR)	Yes	11956643
3576	C-X-C motif chemokine ligand 8(CXCL8)	Yes	11964077
836	caspase 3(CASP3)	Yes	11972398

331	X-linked inhibitor of apoptosis(XIAP)	Yes	11972398
11145	phospholipase A2 group XVI(PLA2G16)	No	11973642
6362	C-C motif chemokine ligand 18(CCL18)	Yes	11978786
1806	dihydropyrimidine dehydrogenase(DPYD)	Yes	11992400
1890	thymidine phosphorylase(TYMP)	Yes	11992400
7083	thymidine kinase 1(TK1)	Yes	11992400
7298	thymidylate synthetase(TYMS)	Yes	11992400
1030	cyclin dependent kinase inhibitor 2B(CDKN2B)	Yes	11992549
4292	mutL homolog 1(MLH1)	Yes	12014680
4437	mutS homolog 3(MSH3)	Yes	12014680
1586	cytochrome P450 family 17 subfamily A member 1(CYP17A1)	Yes	12036914
4288	marker of proliferation Ki-67(MKI67)	Yes	12051866
1027	cyclin dependent kinase inhibitor 1B(CDKN1B)	Yes	12051866
1917	eukaryotic translation elongation factor 1 alpha 2(EEF1A2)	No	12053177
3727	JunD proto-oncogene, AP-1 transcription factor subunit(JUND)	Yes	12054733
6774	signal transducer and activator of transcription 3(STAT3)	Yes	12061840
9180	oncostatin M receptor(OSMR)	Yes	12061840
5008	oncostatin M(OSM)	Yes	12061840
814	calcium/calmodulin dependent protein kinase IV(CAMK4)	Yes	12065094
1843	dual specificity phosphatase 1(DUSP1)	Yes	12080474
5894	Raf-1 proto-oncogene, serine/threonine	Yes	12087097
574	B melanoma antigen(BAGE)	No	12115308
332	baculoviral IAP repeat containing 5(BIRC5)	Yes	12119561
5225	progastricsin(PGC)	Yes	12128264
7010	TEK receptor tyrosine kinase(TEK)	Yes	12138242
285	angiopoietin 2(ANGPT2)	Yes	12138242
284	angiopoietin 1(ANGPT1)	Yes	12138242

1576	cytochrome P450 family 3 subfamily A member 4(CYP3A4)	Yes	12142725
4846	nitric oxide synthase 3(NOS3)	Yes	12144818
7421	vitamin D (1,25- dihydroxyvitamin D3) receptor(VDR)	Yes	12174912
10512	semaphorin 3C(SEMA3C)	Yes	12174914
5243	ATP binding cassette subfamily B member 1(ABCB1)	Yes	12174914
4830	NME/NM23 nucleoside diphosphate kinase	Yes	12174914
3643	insulin receptor(INSR)	Yes	12193537
1032	cyclin dependent kinase inhibitor 2D(CDKN2D)	Yes	12203782
1031	cyclin dependent kinase inhibitor 2C(CDKN2C)	Yes	12203782
540	ATPase copper transporting beta(ATP7B)	Yes	12216079
5268	serpin family B member 5(SERPINB5)	No	12231537
10631	periostin(POSTN)	Yes	12235007
3479	insulin like growth factor 1(IGF1)	Yes	12237896
100133941	CD24 molecule(CD24)	Yes	12368195
2592	galactose-1-phosphate	Yes	12370157
374	amphiregulin(AREG)	Yes	12370739
54346	unc-93 homolog A (C. elegans)(UNC93A)	No	12381271
4314	matrix metallopeptidase 3(MMP3)	Yes	12385578
1508	cathepsin B(CTSB)	Yes	12437120
3552	interleukin 1 alpha(IL1A)	Yes	12445604
3553	interleukin 1 beta(IL1B)	Yes	12445604
3557	interleukin 1 receptor antagonist(IL1RN)	Yes	12445604
57828	cation channel sperm associated auxiliary subunit gamma(CATSPERG)	No	12477932
7015	telomerase reverse transcriptase(TERT)	Yes	12496479
7012	telomerase RNA component(TERC)	Yes	12496479
7251	tumor susceptibility 101(TSG101)	Yes	12505256
3688	integrin subunit beta 1(ITGB1)	Yes	12517798
2896	granulin precursor(GRN)	Yes	12538450

84432	prokineticin 1(PROK1)	Yes	12538479
2429	fragile site, aphidicolin type, common, fra(9)(q32)(FRA9E)	No	12555072
83990	BRCA1 interacting protein C-terminal helicase 1(BRIP1)	Yes	12565990
6623	synuclein gamma(SNCG)	Yes	12566312
10653	serine peptidase inhibitor, Kunitz type	No	12569552
958	CD40 molecule(CD40)	Yes	12576427
8743	tumor necrosis factor superfamily member 10(TNFSF10)	Yes	12576447
3397	inhibitor of DNA binding 1, HLH protein(ID1)	Yes	12576450
2277	vascular endothelial growth factor D(VEGFD)	Yes	12610509
5947	retinol binding protein 1(RBP1)	Yes	12631600
356	Fas ligand(FASLG)	Yes	12637505
3611	integrin linked kinase(ILK)	Yes	12642872
3845	KRAS proto-oncogene, GTPase(KRAS)	Yes	12644542
673	B-Raf proto-oncogene, serine/threonine kinase(BRAF)	Yes	12644542
4609	v-myc avian myelocytomatosis viral oncogene homolog(MYC)	Yes	12646176
1543	cytochrome P450 family 1 subfamily A member 1(CYP1A1)	Yes	12646505
23566	lysophosphatidic acid receptor 3(LPAR3)	Yes	12668280
2118	ETS variant 4(ETV4)	No	12684413
6790	aurora kinase A(AURKA)	Yes	12684414
2175	Fanconi anemia complementation group	Yes	12692539
2176	Fanconi anemia complementation group	Yes	12692539
2189	Fanconi anemia complementation group	Yes	12692539
4610	v-myc avian myelocytomatosis viral oncogene lung carcinoma derived homolog(MYCL)	Yes	12707044
5650	kallikrein related peptidase 7(KLK7)	Yes	12738725
6502	S-phase kinase associated protein 2(SKP2)	Yes	12738731

7040	transforming growth factor beta 1(TGFB1)	Yes	12743121
56616	diablo IAP-binding mitochondrial	Yes	12749848
842	caspase 9(CASP9)	Yes	12749848
595	cyclin D1(CCND1)	Yes	12759391
1803	dipeptidyl peptidase 4(DPP4)	Yes	12767062
7070	Thy-1 cell surface antigen(THY1)	Yes	12781446
1001	cadherin 3(CDH3)	Yes	12800191
5046	proprotein convertase subtilisin/kexin type	Yes	12805404
389	ras homolog family member C(RHOC)	Yes	12808121
387	ras homolog family member A(RHOA)	Yes	12808121
3569	interleukin 6(IL6)	Yes	12810629
472	ATM serine/threonine kinase(ATM)	Yes	12810666
50863	neurotrimin(NTM)	Yes	12819783
4978	opioid binding protein/cell adhesion molecule like(OPCML)	Yes	12819783
7386	ubiquinol-cytochrome c reductase, Rieske iron-sulfur polypeptide 1(UQCRFS1)	Yes	12821338
5245	prohibitin(PHB)	Yes	12821355
652	bone morphogenetic protein 4(BMP4)	Yes	12865307
59348	zinc finger protein 350(ZNF350)	Yes	12872252
2405	fragile site, aphidicolin type, common, fra(6)(q26)(FRA6E)	No	12874785
1950	epidermal growth factor(EGF)	Yes	12879019
5893	RAD52 homolog, DNA repair protein(RAD52)	Yes	12883740
5888	RAD51 recombinase(RAD51)	Yes	12883740
5265	serpin family A member 1(SERPINA1)	Yes	12890555
2697	gap junction protein alpha 1(GJA1)	Yes	12907686
1906	endothelin 1(EDN1)	Yes	12907686
4233	MET proto-oncogene, receptor tyrosine	Yes	12915129
4486	macrophage stimulating 1 receptor(MST1R)	Yes	12915129
5515	protein phosphatase 2 catalytic subunit alpha(PPP2CA)	Yes	12915404

5934	RB transcriptional corepressor like 2(RBL2)	Yes	12915404
55554	kallikrein related peptidase 15(KLK15)	Yes	12915603
1544	cytochrome P450 family 1 subfamily A member 2(CYP1A2)	Yes	12925300
5058	p21 (RAC1) activated kinase 1(PAK1)	Yes	12937139
1387	CREB binding protein(CREBBP)	Yes	12947046
25800	solute carrier family 39 member 6(SLC39A6)	No	12960427
2531	3-ketodihydrosphingosine reductase(KDSR)	Yes	12960700
133	adrenomedullin(ADM)	Yes	12969589
4312	matrix metallopeptidase 1(MMP1)	Yes	12969782
7077	TIMP metallopeptidase inhibitor 2(TIMP2)	Yes	12970394
4322	matrix metallopeptidase 13(MMP13)	Yes	12974393
91768	Cdk5 and Abl enzyme substrate 1(CABLES1)	Yes	13679449
4318	matrix metallopeptidase 9(MMP9)	Yes	14500349
8611	phospholipid phosphatase 1(PLPP1)	Yes	14506139
1647	growth arrest and DNA damage inducible alpha(GADD45A)	Yes	14506229
999	cadherin 1(CDH1)	Yes	14507651
6615	snail family transcriptional repressor 1(SNAI1)	Yes	14507651
5329	plasminogen activator, urokinase	Yes	14520707
30848	cancer/testis antigen 2(CTAG2)	Yes	14522938
1485	cancer/testis antigen 1B(CTAG1B)	No	14522938
5327	plasminogen activator, tissue type(PLAT)	Yes	14529669
10072	dipeptidyl peptidase 3(DPP3)	No	14529681
7049	transforming growth factor beta receptor	Yes	14557487
2488	follicle stimulating hormone beta subunit(FSHB)	Yes	14557487
3972	luteinizing hormone beta polypeptide(LHB)	Yes	14557487
7980	tissue factor pathway inhibitor 2(TFPI2)	Yes	14558946
642934	human ovarian cancer-specific transcript	No	14562052
328	apurinic/apyrimidinic endodeoxyribonuclease 1(APEX1)	Yes	14581338

5295	phosphoinositide-3-kinase regulatory subunit 1(PIK3R1)	Yes	14597629
259	alpha-1-microglobulin/bikunin precursor(AMBP)	Yes	14597629
7515	X-ray repair cross complementing 1(XRCC1)	Yes	14614013
2071	ERCC excision repair 3, TFIIH core complex helicase subunit(ERCC3)	Yes	14614013
7018	transferrin(TF)	Yes	14614458
5071	parkin RBR E3 ubiquitin protein ligase(PARK2)	Yes	14614460
10566	A-kinase anchoring protein 3(AKAP3)	No	14618620
4436	mutS homolog 2(MSH2)	Yes	14631366
6928	HNF1 homeobox B(HNF1B)	Yes	14633622
56937	prostate transmembrane protein, androgen induced 1(PMEPA1)	Yes	14639658
10951	chromobox 1(CBX1)	No	14651845
10771	zinc finger MYND-type containing	Yes	14651845
56946	EMSY, BRCA2 interacting transcriptional repressor(EMSY)	Yes	14651845
7850	interleukin 1 receptor type 2(IL1R2)	Yes	14674121
4683	nibrin(NBN)	Yes	14684699
10111	RAD50 double strand break repair	Yes	14684699
4361	MRE11 homolog, double strand break repair nuclease(MRE11)	Yes	14684699
53340	sperm autoantigenic protein 17(SPA17)	Yes	14712480
5654	HtrA serine peptidase 1(HTRA1)	Yes	14716297
1612	death associated protein kinase 1(DAPK1)	Yes	14767518
10642	insulin like growth factor 2 mRNA binding protein 1(IGF2BP1)	Yes	14767552
7424	vascular endothelial growth factor C(VEGFC)	Yes	14966375
4790	nuclear factor kappa B subunit 1(NFKB1)	Yes	14966375
64073	chromosome 19 open reading frame	No	14981917
81470	olfactory receptor family 2 subfamily G member 2(OR2G2)	No	14983052

3596	interleukin 13(IL13)	Yes	14984938
3600	interleukin 15(IL15)	Yes	14984938
898	cyclin E1(CCNE1)	Yes	15007381
3486	insulin like growth factor binding protein	Yes	15014034
2475	mechanistic target of rapamycin(MTOR)	Yes	15028555
6198	ribosomal protein S6 kinase B1(RPS6KB1)	Yes	15028555
3815	KIT proto-oncogene receptor tyrosine	Yes	15033665
3105	major histocompatibility complex, class I,	Yes	15034213
29108	PYD and CARD domain containing(PYCARD)	Yes	15041718
2810	stratifin(SFN)	Yes	15102672
30816	endogenous retrovirus group W member	Yes	15109395
4646	myosin VI(MYO6)	Yes	15146066
596	BCL2, apoptosis regulator(BCL2)	Yes	15156398
2274	four and a half LIM domains 2(FHL2)	Yes	15161045
9166	estrogen receptor binding site associated, antigen, 9(EBAG9)	Yes	15164121
2152	coagulation factor III, tissue factor(F3)	Yes	15182581
2155	coagulation factor VII(F7)	Yes	15182581
1728	NAD(P)H quinone dehydrogenase 1(NQO1)	Yes	15196853
6648	superoxide dismutase 2, mitochondrial(SOD2)	Yes	15196853
4353	myeloperoxidase(MPO)	Yes	15196853
1440	colony stimulating factor 3(CSF3)	Yes	15214947
1365	claudin 3(CLDN3)	No	15240533
4072	epithelial cell adhesion molecule(EPCAM)	Yes	15240533
5595	mitogen-activated protein kinase 3(MAPK3)	Yes	15243131
5594	mitogen-activated protein kinase 1(MAPK1)	Yes	15243131
93659	chorionic gonadotropin beta subunit 5(CGB5)	Yes	15253136
54658	UDP glucuronosyltransferase family 1 member A1(UGT1A1)	Yes	15254716
57602	ubiquitin specific peptidase 36(USP36)	Yes	15254734
8837	CASP8 and FADD like apoptosis	Yes	15258564
10723	solute carrier family 12 member 7(SLC12A7)	Yes	15262997

9990	solute carrier family 12 member 6(SLC12A6)	Yes	15262997
6560	solute carrier family 12 member 4(SLC12A4)	Yes	15262997
4363	ATP binding cassette subfamily C member 1(ABCC1)	Yes	15269137
1495	catenin alpha 1(CTNNA1)	Yes	15297182
1969	EPH receptor A2(EPHA2)	Yes	15297418
6383	syndecan 2(SDC2)	Yes	15297422
6382	syndecan 1(SDC1)	Yes	15297422
2817	glypican 1(GPC1)	Yes	15297422
355	Fas cell surface death receptor(FAS)	Yes	15297424
2247	fibroblast growth factor 2(FGF2)	Yes	15302993
23024	PDZ domain containing ring finger 3(PDZRN3)	Yes	15305371
84700	myosin XVIIIB(MYO18B)	Yes	15305387
54361	Wnt family member 4(WNT4)	Yes	15312687
837	caspase 4(CASP4)	Yes	15326478
834	caspase 1(CASP1)	Yes	15326478
3606	interleukin 18(IL18)	Yes	15326478
6513	solute carrier family 2 member 1(SLC2A1)	Yes	15331928
5732	prostaglandin E receptor 2(PTGER2)	Yes	15347673
5734	prostaglandin E receptor 4(PTGER4)	Yes	15347673
3717	Janus kinase 2(JAK2)	Yes	15358195
64699	transmembrane protease, serine 3(TMPRSS3)	Yes	15361711
8651	suppressor of cytokine signaling 1(SOCS1)	Yes	15361843
8835	suppressor of cytokine signaling 2(SOCS2)	Yes	15361843
9021	suppressor of cytokine signaling 3(SOCS3)	Yes	15361843
4254	KIT ligand(KITLG)	Yes	15378028
3159	high mobility group AT-hook 1(HMGA1)	Yes	15378028
2335	fibronectin 1(FN1)	Yes	15384859
2621	growth arrest specific 6(GAS6)	Yes	15452374
3122	major histocompatibility complex, class II, DR alpha(HLA-DRA)	Yes	15467430
3855	keratin 7(KRT7)	No	15489654

4582	mucin 1, cell surface associated(MUC1)	Yes	15489654
64326	ring finger and WD repeat domain 2(RFWD2)	Yes	15492238
57111	RAB25, member RAS oncogene family(RAB25)	Yes	15502842
1019	cyclin dependent kinase 4(CDK4)	Yes	15505422
4316	matrix metallopeptidase 7(MMP7)	Yes	15523695
130399	activin A receptor type 1C(ACVR1C)	Yes	15531507
7076	TIMP metallopeptidase inhibitor 1(TIMP1)	Yes	15557756
5325	PLAG1 like zinc finger 1(PLAGL1)	Yes	15581945
3586	interleukin 10(IL10)	Yes	15581980
5468	peroxisome proliferator activated receptor gamma(PPARG)	Yes	15583697
2693	growth hormone secretagogue receptor(GHSR)	Yes	15585554
65059	Ras association (RalGDS/AF-6) and pleckstrin homology domains 1(RAPH1)	Yes	15586368
6768	suppression of tumorigenicity 14(ST14)	Yes	15611789
8751	ADAM metallopeptidase domain 15(ADAM15)	Yes	15618016
998	cell division cycle 42(CDC42)	Yes	15655247
2002	ELK1, ETS transcription factor(ELK1)	Yes	15655247
8826	IQ motif containing GTPase activating protein 1(IQGAP1)	No	15655247
348	apolipoprotein E(APOE)	Yes	15665311
10381	tubulin beta 3 class III(TUBB3)	No	15671559
3621	inhibitor of growth family member 1(ING1)	Yes	15677627
3485	insulin like growth factor binding protein	Yes	15686601
5618	prolactin receptor(PRLR)	Yes	15700312
10752	cell adhesion molecule L1 like(CHL1)	Yes	15704102
2139	EYA transcriptional coactivator and phosphatase 2(EYA2)	Yes	15705892
573	BCL2 associated athanogene 1(BAG1)	Yes	15707960
7517	X-ray repair cross complementing 3(XRCC3)	Yes	15734952
7516	X-ray repair cross complementing 2(XRCC2)	Yes	15734952
9839	zinc finger E-box binding homeobox 2(ZEB2)	Yes	15742334

1588	cytochrome P450 family 19 subfamily A member 1(CYP19A1)	Yes	15748828
5159	platelet derived growth factor receptor beta(PDGFRB)	Yes	15791568
5156	platelet derived growth factor receptor alpha(PDGFRA)	Yes	15791568
358	aquaporin 1 (Colton blood group)(AQP1)	Yes	15809704
100113421	fibroblast growth factor 7 pseudogene	No	15809711
728433	fibroblast growth factor 7 pseudogene	No	15809711
654466	fibroblast growth factor 7 pseudogene	No	15809711
387628	fibroblast growth factor 7 pseudogene	No	15809711
728195	fibroblast growth factor 7 pseudogene	No	15809711
653453	fibroblast growth factor 7 pseudogene	No	15809711
5528	protein phosphatase 2 regulatory subunit B'delta(PPP2R5D)	No	15809743
148479	PHD finger protein 13(PHF13)	No	15825179
1839	heparin binding EGF like growth factor(HBEGF)	Yes	15827558
1909	endothelin receptor type A(EDNRA)	Yes	15838262
10555	1-acylglycerol-3-phosphate O-acyltransferase 2(AGPAT2)	No	15841084
7507	XPA, DNA damage recognition and repair factor(XPA)	Yes	15844177
5920	retinoic acid receptor responder 3(RARRES3)	Yes	15856468
8797	TNF receptor superfamily member 10a(TNFRSF10A)	Yes	15863153
56849	transcription elongation factor A like	Yes	15870691
7124	tumor necrosis factor(TNF)	Yes	15870936
3458	interferon gamma(IFNG)	Yes	15870936
79679	V-set domain containing T cell activation inhibitor 1(VTCN1)	Yes	15878339
796	calcitonin related polypeptide alpha(CALCA)	Yes	15880427

1558	cytochrome P450 family 2 subfamily C member 8(CYP2C8)	Yes	15901749
5566	protein kinase cAMP-activated catalytic subunit alpha(PRKACA)	Yes	15905176
6303	spermidine/spermine N1-acetyltransferase	Yes	15905201
7448	vitronectin(VTN)	Yes	15914035
6387	C-X-C motif chemokine ligand 12(CXCL12)	Yes	15921680
3117	major histocompatibility complex, class II, DQ alpha 1(HLA-DQA1)	Yes	15935893
3119	major histocompatibility complex, class II, DQ beta 1(HLA-DQB1)	Yes	15935893
3123	major histocompatibility complex, class II, DR beta 1(HLA-DRB1)	Yes	15935893
3265	HRas proto-oncogene, GTPase(HRAS)	Yes	15940260
5742	prostaglandin-endoperoxide synthase 1(PTGS1)	Yes	15963707
51741	WW domain containing	Yes	15982416
7163	tumor protein D52(TPD52)	Yes	15986428
545	ATR serine/threonine kinase(ATR)	Yes	15987455
80781	collagen type XVIII alpha 1 chain(COL18A1)	Yes	16008891
6470	serine hydroxymethyltransferase 1(SHMT1)	Yes	16049973
7402	utrophin(UTRN)	Yes	16049973
6926	T-box 3(TBX3)	Yes	16049973
91304	transmembrane protein 259(TMEM259)	No	16084606
81027	tubulin beta 1 class VI(TUBB1)	Yes	16095531
213	albumin(ALB)	Yes	16099937
10418	spondin 1(SPON1)	Yes	16103746
5599	mitogen-activated protein kinase 8(MAPK8)	Yes	16105650
2627	GATA binding protein 6(GATA6)	Yes	16110260
2626	GATA binding protein 4(GATA4)	Yes	16110260
5584	protein kinase C iota(PRKCI)	No	16116079
51352	WT1 antisense RNA(WT1-AS)	Yes	16134181
4851	notch 1(NOTCH1)	Yes	16136053

268	anti-Mullerian hormone(AMH)	Yes	16159935
8682	phosphoprotein enriched in astrocytes	Yes	16170361
51773	remodeling and spacing factor 1(RSF1)	No	16172393
11156	protein tyrosine phosphatase type IVA, member 3(PTP4A3)	No	16203771
10061	ATP binding cassette subfamily F member	Yes	16203778
4968	8-oxoguanine DNA glycosylase(OGG1)	Yes	16211227
9473	thymocyte selection associated family member 2(THEMIS2)	Yes	16219472
8091	high mobility group AT-hook 2(HMGA2)	Yes	16222997
4193	MDM2 proto-oncogene(MDM2)	Yes	16239061
857	caveolin 1(CAV1)	Yes	16251425
7991	tumor suppressor candidate 3(TUSC3)	Yes	16270321
5108	pericentriolar material 1(PCM1)	Yes	16270321
23362	pleckstrin and Sec7 domain containing 3(PSD3)	Yes	16270321
5157	platelet derived growth factor receptor	Yes	16270321
53834	fibroblast growth factor receptor-like	Yes	16273302
3814	KiSS-1 metastasis-suppressor(KISS1)	Yes	16283480
6817	sulfotransferase family 1A member 1(SULT1A1)	Yes	16284375
11186	Ras association domain family member	Yes	16315018
50943	forkhead box P3(FOXP3)	Yes	16322292
3690	integrin subunit beta 3(ITGB3)	Yes	16322334
9429	ATP binding cassette subfamily G member 2 (Junior blood group)(ABCG2)	Yes	16343180
7066	thrombopoietin(THPO)	Yes	16359773
9338	transcription elongation factor A like	Yes	16364249
54507	ADAMTS like 4(ADAMTSL4)	No	16364318
6093	Rho associated coiled-coil containing protein kinase 1(ROCK1)	Yes	16371346
3897	L1 cell adhesion molecule(L1CAM)	Yes	16377081
8829	neuropilin 1(NRP1)	Yes	16377081
8991	selenium binding protein 1(SELENBP1)	Yes	16380993

79161	transmembrane protein 243(TMEM243)	No	16382445
947	CD34 molecule(CD34)	Yes	16415795
794	calbindin 2(CALB2)	Yes	16415795
2188	Fanconi anemia complementation group	Yes	16418574
6036	ribonuclease A family member 2(RNASE2)	Yes	16428483
185	angiotensin II receptor type 1(AGTR1)	Yes	16434990
8646	chordin(CHRD)	Yes	16449796
3082	hepatocyte growth factor(HGF)	Yes	16469801
10927	spindlin 1(SPIN1)	Yes	16472086
3481	insulin like growth factor 2(IGF2)	Yes	16489075
51455	REV1, DNA directed polymerase(REV1)	Yes	16495473
3091	hypoxia inducible factor 1 alpha subunit(HIF1A)	Yes	16515634
7153	topoisomerase (DNA) II alpha(TOP2A)	Yes	16533424
1432	mitogen-activated protein kinase 14(MAPK14)	Yes	16538384
4323	matrix metallopeptidase 14(MMP14)	Yes	16551853
991	cell division cycle 20(CDC20)	Yes	16572426
5901	RAN, member RAS oncogene family(RAN)	Yes	16572426
8549	leucine rich repeat containing G protein-coupled receptor 5(LGR5)	Yes	16575208
2664	GDP dissociation inhibitor 1(GDI1)	Yes	16596196
396	Rho GDP dissociation inhibitor	No	16596196
5521	protein phosphatase 2 regulatory subunit Bbeta(PPP2R2B)	Yes	16596250
283120	H19, imprinted maternally expressed transcript (non-protein coding)(H19)	Yes	16603642
2624	GATA binding protein 2(GATA2)	Yes	16607277
2623	GATA binding protein 1(GATA1)	Yes	16607277
90780	pygopus family PHD finger 2(PYGO2)	No	16609037
390998	ribosomal protein L10 pseudogene	Yes	16627977
6134	ribosomal protein L10(RPL10)	Yes	16627977
6416	mitogen-activated protein kinase kinase	Yes	16627982
7430	ezrin(EZR)	Yes	16633060

682	basigin (Ok blood group)(BSG)	Yes	16633062
7345	ubiquitin C-terminal hydrolase L1(UCHL1)	Yes	16642472
2706	gap junction protein beta 2(GJB2)	Yes	16647893
2068	ERCC excision repair 2, TFIIH core complex helicase subunit(ERCC2)	Yes	16677755
4843	nitric oxide synthase 2(NOS2)	Yes	16681724
1364	claudin 4(CLDN4)	No	16714763
6667	Sp1 transcription factor(SP1)	Yes	16714763
92799	SH3KBP1 binding protein 1(SHKBP1)	No	16733801
30011	SH3 domain containing kinase binding protein 1(SH3KBP1)	No	16733801
2041	EPH receptor A1(EPHA1)	Yes	16737551
2044	EPH receptor A5(EPHA5)	Yes	16737551
9940	deleted in lung and esophageal cancer 1(DLEC1)	Yes	16756719
119391	glutathione S-transferase omega 2(GSTO2)	Yes	16761626
10011	steroid receptor RNA activator 1(SRA1)	Yes	16773180
5378	PMS1 homolog 1, mismatch repair system component(PMS1)	Yes	16774946
2956	mutS homolog 6(MSH6)	Yes	16774946
5395	PMS1 homolog 2, mismatch repair system component(PMS2)	Yes	16774946
27030	mutL homolog 3(MLH3)	Yes	16774946
329	baculoviral IAP repeat containing 2(BIRC2)	Yes	16775178
5176	serpin family F member 1(SERPINF1)	Yes	16777976
83658	dynein light chain roadblock-type 1(DYNLRB1)	No	16778097
1316	Kruppel like factor 6(KLF6)	Yes	16778100
2932	glycogen synthase kinase 3 beta(GSK3B)	Yes	16788573
8900	cyclin A1(CCNA1)	Yes	16799873
4162	melanoma cell adhesion molecule(MCAM)	No	16804906
3624	inhibin beta A subunit(INHBA)	Yes	16820918
1244	ATP binding cassette subfamily C member 2(ABCC2)	Yes	16821592

4035	LDL receptor related protein 1(LRP1)	Yes	16821592
6418	SET nuclear proto-oncogene(SET)	Yes	16823850
4052	latent transforming growth factor beta binding protein 1(LTBP1)	Yes	16825507
7052	transglutaminase 2(TGM2)	Yes	16826431
60468	BTB domain and CNC homolog 2(BACH2)	Yes	16832351
43	acetylcholinesterase (Cartwright blood	Yes	16832351
3791	kinase insert domain receptor(KDR)	Yes	16835828
207	AKT serine/threonine kinase 1(AKT1)	Yes	16839745
50624	CUB and zona pellucida like domains 1(CUZD1)	No	16862170
157310	phosphatidylethanolamine binding protein	Yes	16865237
6196	ribosomal protein S6 kinase A2(RPS6KA2)	Yes	16878154
4585	mucin 4, cell surface associated(MUC4)	Yes	16880776
8605	phospholipase A2 group IVC(PLA2G4C)	Yes	16882668
3598	interleukin 13 receptor subunit alpha	Yes	16902988
11073	topoisomerase (DNA) II binding protein	Yes	16930991
4915	neurotrophic receptor tyrosine kinase 2(NTRK2)	Yes	16964397
1630	DCC netrin 1 receptor(DCC)	Yes	16971669
1978	eukaryotic translation initiation factor 4E binding protein 1(EIF4EBP1)	Yes	16983702
6275	S100 calcium binding protein A4(S100A4)	Yes	16984379
3291	hydroxysteroid 11-beta dehydrogenase	Yes	17028049
5925	RB transcriptional corepressor 1(RB1)	Yes	17047088
2919	C-X-C motif chemokine ligand 1(CXCL1)	Yes	17060621
7849	paired box 8(PAX8)	Yes	17064757
861	runt related transcription factor 1(RUNX1)	Yes	17072347
3693	integrin subunit beta 5(ITGB5)	Yes	17074516
440	asparagine synthetase (glutamine-	No	17088436
10000	AKT serine/threonine kinase 3(AKT3)	Yes	17178867
6662	SRY-box 9(SOX9)	Yes	17197889
25803	SAM pointed domain containing ETS transcription factor(SPDEF)	Yes	17197890

174	alpha fetoprotein(AFP)	Yes	17197899
144455	E2F transcription factor 7(E2F7)	Yes	17200349
3927	LIM and SH3 protein 1(LASP1)	Yes	17211471
1525	coxsackie virus and adenovirus	Yes	17278108
23213	sulfatase 1(SULF1)	Yes	17310998
55294	F-box and WD repeat domain containing	Yes	17320938
4835	NAD(P)H quinone dehydrogenase 2(NQO2)	Yes	17332305
5578	protein kinase C alpha(PRKCA)	Yes	17341442
1869	E2F transcription factor 1(E2F1)	Yes	17341621
1870	E2F transcription factor 2(E2F2)	Yes	17341621
2050	EPH receptor B4(EPHB4)	Yes	17353927
445	argininosuccinate synthase 1(ASS1)	Yes	17354225
8678	beclin 1(BECN1)	Yes	17355787
29126	CD274 molecule(CD274)	Yes	17360651
10891	PPARG coactivator 1 alpha(PPARGC1A)	Yes	17372612
5518	protein phosphatase 2 scaffold subunit Aalpha(PPP2R1A)	Yes	17374643
112939	nucleus accumbens associated 1(NACC1)	No	17391728
7136	troponin I2, fast skeletal type(TNNI2)	Yes	17393089
894	cyclin D2(CCND2)	Yes	17409409
896	cyclin D3(CCND3)	Yes	17409409
1017	cyclin dependent kinase 2(CDK2)	Yes	17409409
1021	cyclin dependent kinase 6(CDK6)	Yes	17409409
55892	myoneurin(MYNN)	Yes	17409414
3320	heat shock protein 90 alpha family class A member 1(HSP90AA1)	Yes	17409432
10419	protein arginine methyltransferase 5(PRMT5)	No	17409432
142	poly(ADP-ribose) polymerase 1(PARP1)	Yes	17413981
3336	heat shock protein family E (Hsp10) member 1(HSPE1)	Yes	17419711
3574	interleukin 7(IL7)	Yes	17438097
7852	C-X-C motif chemokine receptor 4(CXCR4)	Yes	17459055

4904	Y-box binding protein 1(YBX1)	No	17459055
5727	patched 1(PTCH1)	Yes	17487419
117581	twist family bHLH transcription factor	Yes	17487558
7291	twist family bHLH transcription factor	Yes	17487558
4680	carcinoembryonic antigen related cell adhesion molecule 6(CEACAM6)	Yes	17503391
2678	gamma-glutamyltransferase 1(GGT1)	Yes	17503391
2101	estrogen related receptor alpha(ESRRA)	Yes	17509876
5076	paired box 2(PAX2)	Yes	17529925
5730	prostaglandin D2 synthase(PTGDS)	Yes	17532558
2246	fibroblast growth factor 1(FGF1)	Yes	17538174
6286	S100 calcium binding protein P(S100P)	No	17539915
3858	keratin 10(KRT10)	No	17542994
3875	keratin 18(KRT18)	Yes	17542994
3640	insulin like 3(INSL3)	Yes	17549672
9354	ubiquitination factor E4A(UBE4A)	No	17553343
9097	ubiquitin specific peptidase 14(USP14)	No	17553343
9099	ubiquitin specific peptidase 2(USP2)	Yes	17553343
7428	von Hippel-Lindau tumor suppressor(VHL)	Yes	17555795
2034	endothelial PAS domain protein 1(EPAS1)	Yes	17555795
85413	solute carrier family 22 member 16(SLC22A16)	Yes	17581421
2719	glypican 3(GPC3)	No	17581422
7216	trophinin(TRO)	No	17597582
6520	solute carrier family 3 member 2(SLC3A2)	Yes	17611393
6517	solute carrier family 2 member 4(SLC2A4)	Yes	17611657
6515	solute carrier family 2 member 3(SLC2A3)	Yes	17611657
84126	ATR interacting protein(ATRIP)	Yes	17616665
3205	homeobox A9(HOXA9)	Yes	17623056
3215	homeobox B5(HOXB5)	Yes	17623056
1381	cellular retinoic acid binding protein 1(CRABP1)	Yes	17623056
92304	secretoglobin family 3A member 1(SCGB3A1)	Yes	17623056
967	CD63 molecule(CD63)	No	17624082

3678	integrin subunit alpha 5(ITGA5)	Yes	17624082
5045	furin, paired basic amino acid cleaving enzyme(FURIN)	Yes	17641413
6647	superoxide dismutase 1, soluble(SOD1)	Yes	17646272
6649	superoxide dismutase 3, extracellular(SOD3)	Yes	17646272
5047	progestagen associated endometrial	Yes	17649816
290	alanyl aminopeptidase, membrane(ANPEP)	Yes	17655775
3107	major histocompatibility complex, class I,	Yes	17661908
3106	major histocompatibility complex, class I,	Yes	17661908
1394	corticotropin releasing hormone receptor	Yes	17667919
1392	corticotropin releasing hormone(CRH)	Yes	17667919
3816	kallikrein 1(KLK1)	Yes	17671125
1490	connective tissue growth factor(CTGF)	Yes	17671176
55743	checkpoint with forkhead and ring finger domains(CHFR)	Yes	17673375
5581	protein kinase C epsilon(PRKCE)	Yes	17678893
26135	SERPINE1 mRNA binding protein 1(SERBP1)	Yes	17698176
63928	calcineurin like EF-hand protein 2(CHP2)	Yes	17708351
29775	caspase recruitment domain family member 10(CARD10)	Yes	17724468
2925	gastrin releasing peptide receptor(GRPR)	Yes	17726264
7508	XPC complex subunit, DNA damage recognition and repair factor(XPC)	Yes	17825393
2073	ERCC excision repair 5, endonuclease(ERCC5)	Yes	17825393
3135	major histocompatibility complex, class I,	Yes	17846022
5294	phosphatidylinositol-4,5-bisphosphate 3-kinase catalytic subunit gamma(PIK3CG)	Yes	17868340
8842	prominin 1(PROM1)	Yes	17868344
55898	unc-45 myosin chaperone A(UNC45A)	No	17872978
81	actinin alpha 4(ACTN4)	Yes	17873890
8626	tumor protein p63(TP63)	Yes	17880529
1028	cyclin dependent kinase inhibitor 1C(CDKN1C)	Yes	17885492

2056	erythropoietin(EPO)	Yes	17893874
2057	erythropoietin receptor(EPOR)	Yes	17893874
8915	B-cell CLL/lymphoma 10(BCL10)	Yes	17893875
5820	Pvt1 oncogene (non-protein coding)(PVT1)	Yes	17908964
7799	PR/SET domain 2(PRDM2)	Yes	17922684
4137	microtubule associated protein tau(MAPT)	Yes	17925548
1577	cytochrome P450 family 3 subfamily A member 5(CYP3A5)	Yes	17925548
5720	proteasome activator subunit 1(PSME1)	No	17939699
4615	myeloid differentiation primary response	Yes	17940625
10481	homeobox B13(HOXB13)	Yes	17942676
11315	Parkinsonism associated deglycase(PARK7)	Yes	17949781
3204	homeobox A7(HOXA7)	Yes	17959889
820	cathelicidin antimicrobial peptide(CAMP)	No	17960624
406883	microRNA let-7a-3(MIRLET7A3)	Yes	17974952
5591	protein kinase, DNA-activated, catalytic polypeptide(PRKDC)	Yes	17991316
84634	KISS1 receptor(KISS1R)	Yes	18005407
4311	membrane metalloendopeptidase(MME)	Yes	18042078
3925	stathmin 1(STMN1)	Yes	18054374
7039	transforming growth factor alpha(TGFA)	Yes	18054376
406991	microRNA 21(MIR21)	Yes	18059191
5605	mitogen-activated protein kinase kinase	Yes	18060073
5604	mitogen-activated protein kinase kinase	Yes	18060073
4345	CD200 molecule(CD200)	Yes	18060403
7045	transforming growth factor beta induced(TGFBI)	Yes	18068629
27346	transmembrane protein 97(TMEM97)	No	18070364
2770	G protein subunit alpha i1(GNAI1)	Yes	18083345
3292	hydroxysteroid 17-beta dehydrogenase	Yes	18083510
5154	platelet derived growth factor subunit	Yes	18084257
3295	hydroxysteroid 17-beta dehydrogenase	Yes	18086758
1786	DNA methyltransferase 1(DNMT1)	Yes	18095639

1789	DNA methyltransferase 3 beta(DNMT3B)	Yes	18095639
140690	CCCTC-binding factor like(CTCFL)	Yes	18095639
3364	HUS1 checkpoint clamp component(HUS1)	Yes	18156970
5883	RAD9 checkpoint clamp component A(RAD9A)	Yes	18156970
6868	ADAM metallopeptidase domain 17(ADAM17)	Yes	18171982
214	activated leukocyte cell adhesion	Yes	18171982
406996	microRNA 214(MIR214)	No	18199536
3164	nuclear receptor subfamily 4 group A member 1(NR4A1)	Yes	18202854
26271	F-box protein 5(FBXO5)	Yes	18204430
4320	matrix metallopeptidase 11(MMP11)	Yes	18208802
1948	ephrin B2(EFNB2)	Yes	18231102
5244	ATP binding cassette subfamily B member 4(ABCB4)	Yes	18231753
94031	HtrA serine peptidase 3(HTRA3)	Yes	18241672
27429	HtrA serine peptidase 2(HTRA2)	Yes	18241672
1401	C-reactive protein(CRP)	Yes	18245530
152831	klotho beta(KLB)	Yes	18259951
2589	polypeptide N-acetylgalactosaminyltransferase 1(GALNT1)	Yes	18268124
3565	interleukin 4(IL4)	Yes	18276105
79728	partner and localizer of BRCA2(PALB2)	Yes	18281473
5178	paternally expressed 3(PEG3)	Yes	18286529
1366	claudin 7(CLDN7)	No	18298564
7514	exportin 1(XPO1)	Yes	18306389
5970	RELA proto-oncogene, NF-kB subunit(RELA)	Yes	18314621
9846	GRB2 associated binding protein 2(GAB2)	Yes	18314909
10857	progesterone receptor membrane component 1(PGRMC1)	Yes	18319313
3692	eukaryotic translation initiation factor 6(EIF6)	Yes	18327211
5168	ectonucleotide pyrophosphatase/phosphodiesterase 2(ENPP2)	Yes	18337445

51366	ubiquitin protein ligase E3 component n-recognin 5(UBR5)	Yes	18349819
11279	Kruppel like factor 8(KLF8)	Yes	18353772
3294	hydroxysteroid 17-beta dehydrogenase	Yes	18372405
3329	heat shock protein family D (Hsp60) member 1(HSPD1)	Yes	18372523
1788	DNA methyltransferase 3 alpha(DNMT3A)	Yes	18381459
7571	zinc finger protein 23(ZNF23)	Yes	18384939
5315	pyruvate kinase, muscle(PKM)	Yes	18394773
8277	transketolase like 1(TKTL1)	Yes	18394773
28996	homeodomain interacting protein kinase	Yes	18395248
3551	inhibitor of kappa light polypeptide gene enhancer in B-cells, kinase beta(IKBKB)	Yes	18408758
406977	microRNA 199a-2(MIR199A2)	No	18408758
406976	microRNA 199a-1(MIR199A1)	No	18408758
3973	luteinizing hormone/choriogonadotropin receptor(LHCGR)	Yes	18411916
1241	leukotriene B4 receptor(LTB4R)	Yes	18421027
56413	leukotriene B4 receptor 2(LTB4R2)	Yes	18421027
240	arachidonate 5-lipoxygenase(ALOX5)	Yes	18421027
4684	neural cell adhesion molecule 1(NCAM1)	Yes	18425046
7764	zinc finger protein 217(ZNF217)	No	18425333
6590	secretory leukocyte peptidase inhibitor(SLPI)	No	18425362
3698	inter-alpha-trypsin inhibitor heavy chain	Yes	18425383
648	BMI1 proto-oncogene, polycomb ring	No	18438345
79923	Nanog homeobox(NANOG)	Yes	18441325
407008	microRNA 223(MIR223)	Yes	18442408
2931	glycogen synthase kinase 3 alpha(GSK3A)	Yes	18450971
7295	thioredoxin(TXN)	Yes	18457020
22914	killer cell lectin like receptor K1(KLRK1)	Yes	18490733
4282	macrophage migration inhibitory factor (glycosylation-inhibiting factor)(MIF)	Yes	18490733

25794	fascin actin-bundling protein 2, retinal(FSCN2)	Yes	18498068
7042	transforming growth factor beta 2(TGFB2)	Yes	18505915
7043	transforming growth factor beta 3(TGFB3)	Yes	18505915
4088	SMAD family member 3(SMAD3)	Yes	18505915
22981	ninein like(NINL)	Yes	18538832
3976	leukemia inhibitory factor(LIF)	Yes	18540977
1482	NK2 homeobox 5(NKX2-5)	Yes	18547965
1385	cAMP responsive element binding protein	Yes	18554190
83737	itchy E3 ubiquitin protein ligase(ITCH)	Yes	18559494
27043	proline, glutamate and leucine rich protein	Yes	18559538
538	ATPase copper transporting alpha(ATP7A)	Yes	18565219
5037	phosphatidylethanolamine binding protein	Yes	18567796
23452	angiopoietin like 2(ANGPTL2)	No	18593905
983	cyclin dependent kinase 1(CDK1)	Yes	18615582
11047	adhesion regulating molecule 1(ADRM1)	No	18615678
7534	tyrosine 3-monooxygenase/tryptophan 5-monooxygenase activation protein	Yes	18618111
3952	leptin(LEP)	Yes	18624749
3438	interferon, type 1, cluster(IFN1@)	No	18632618
182	jagged 1(JAG1)	Yes	18632624
4854	notch 3(NOTCH3)	Yes	18632624
5724	platelet activating factor receptor(PTAFR)	Yes	18632638
5829	paxillin(PXN)	Yes	18632638
1848	dual specificity phosphatase 6(DUSP6)	Yes	18632752
406938	microRNA 146a(MIR146A)	Yes	18660546
2523	fucosyltransferase 1 (H blood group)(FUT1)	Yes	18686606
9128	pre-mRNA processing factor 4(PRPF4)	No	18687998
1958	early growth response 1(EGR1)	Yes	18704310
1435	colony stimulating factor 1(CSF1)	Yes	18708368
2597	glyceraldehyde-3-phosphate	Yes	18708368
5444	paraoxonase 1(PON1)	Yes	18708400
822	capping actin protein, gelsolin like(CAPG)	Yes	18709641

23532	preferentially expressed antigen in melanoma(PRAME)	No	18709641
1191	clusterin(CLU)	Yes	18709641
3065	histone deacetylase 1(HDAC1)	Yes	18714364
8728	ADAM metallopeptidase domain 19(ADAM19)	Yes	18714391
9112	metastasis associated 1(MTA1)	Yes	18719363
10755	GIPC PDZ domain containing family member 1(GIPC1)	Yes	18721484
668	forkhead box L2(FOXL2)	Yes	18721930
3283	hydroxy-delta-5-steroid dehydrogenase, 3 beta- and steroid delta-isomerase 1(HSD3B1)	Yes	18723074
412	steroid sulfatase (microsomal), isozyme S(STS)	Yes	18723074
8856	nuclear receptor subfamily 1 group I member 2(NR1I2)	Yes	18765524
10	N-acetyltransferase 2(NAT2)	Yes	18768514
6624	fascin actin-bundling protein 1(FSCN1)	Yes	18776588
2017	cortactin(CTTN)	Yes	18776588
7057	thrombospondin 1(THBS1)	Yes	18788631
4277	MHC class I polypeptide-related sequence	Yes	18791713
100507436	MHC class I polypeptide-related sequence	Yes	18791713
80328	UL16 binding protein 2(ULBP2)	Yes	18791713
404663	long intergenic non-protein coding RNA 1194(LINC01194)	No	18791715
9111	N-myc and STAT interactor(NMI)	Yes	18794094
3491	cysteine rich angiogenic inducer 61(CYR61)	Yes	18800188
54514	DEAD-box helicase 4(DDX4)	Yes	18805576
6678	secreted protein acidic and cysteine	Yes	18813349
64127	nucleotide binding oligomerization domain containing 2(NOD2)	Yes	18819462
2191	fibroblast activation protein alpha(FAP)	Yes	18823010
5915	retinoic acid receptor beta(RARB)	Yes	18825962
3605	interleukin 17A(IL17A)	Yes	18832156

3172	hepatocyte nuclear factor 4 alpha(HNF4A)	Yes	18844932
2353	Fos proto-oncogene, AP-1 transcription factor subunit(FOS)	Yes	18854825
864	runt related transcription factor 3(RUNX3)	Yes	18937968
1548	cytochrome P450 family 2 subfamily A member 6(CYP2A6)	Yes	18941913
55353	lysosomal protein transmembrane 4	Yes	18949404
8997	kalirin, RhoGEF kinase(KALRN)	Yes	18953434
26038	chromodomain helicase DNA binding protein 5(CHD5)	Yes	18953434
7058	thrombospondin 2(THBS2)	Yes	18955754
4214	mitogen-activated protein kinase kinase kinase 1(MAP3K1)	Yes	18973230
2263	fibroblast growth factor receptor 2(FGFR2)	Yes	18973230
4046	lymphocyte-specific protein 1(LSP1)	Yes	18973230
5087	PBX homeobox 1(PBX1)	Yes	18974129
27063	ankyrin repeat domain 1(ANKRD1)	Yes	18980987
3579	C-X-C motif chemokine receptor 2(CXCR2)	Yes	19010874
10103	tetraspanin 1(TSPAN1)	Yes	19017553
2878	glutathione peroxidase 3(GPX3)	Yes	19020706
1977	eukaryotic translation initiation factor 4E(EIF4E)	Yes	19020722
5562	protein kinase AMP-activated catalytic subunit alpha 1(PRKAA1)	Yes	19020741
27250	programmed cell death 4 (neoplastic transformation inhibitor)(PDCD4)	Yes	19031945
3109	major histocompatibility complex, class II, DM beta(HLA-DMB)	Yes	19047092
3918	laminin subunit gamma 2(LAMC2)	Yes	19047908
1045	caudal type homeobox 2(CDX2)	Yes	19047909
4763	neurofibromin 1(NF1)	Yes	19048115
571	BTB domain and CNC homolog 1(BACH1)	Yes	19064572
29947	DNA methyltransferase 3 like(DNMT3L)	Yes	19064572

8204	nuclear receptor interacting protein 1(NRIP1)	Yes	19064572
10038	poly(ADP-ribose) polymerase 2(PARP2)	Yes	19064572
2147	coagulation factor II, thrombin(F2)	Yes	19064572
2153	coagulation factor V(F5)	Yes	19064572
2162	coagulation factor XIII A chain(F13A1)	Yes	19064572
2177	Fanconi anemia complementation group D2(FANCD2)	Yes	19064572
2178	Fanconi anemia complementation group	Yes	19064572
3630	insulin(INS)	Yes	19064572
2517	fucosidase, alpha-L- 1, tissue(FUCA1)	Yes	19064572
5731	prostaglandin E receptor 1(PTGER1)	Yes	19064572
5733	prostaglandin E receptor 3(PTGER3)	Yes	19064572
4616	growth arrest and DNA damage inducible beta(GADD45B)	Yes	19064572
5740	prostaglandin I2 synthase(PTGIS)	Yes	19064572
5729	prostaglandin D2 receptor(PTGDR)	Yes	19064572
10912	growth arrest and DNA damage inducible gamma(GADD45G)	Yes	19064572
7518	X-ray repair cross complementing 4(XRCC4)	Yes	19064572
7520	X-ray repair cross complementing 5(XRCC5)	Yes	19064572
6462	sex hormone binding globulin(SHBG)	Yes	19064572
1719	dihydrofolate reductase(DHFR)	Yes	19064572
3383	intercellular adhesion molecule 1(ICAM1)	Yes	19064572
1787	tRNA aspartic acid methyltransferase	Yes	19064572
6715	steroid 5 alpha-reductase 1(SRD5A1)	Yes	19064572
4118	mal, T-cell differentiation protein(MAL)	Yes	19064572
4153	mannose binding lectin 2(MBL2)	Yes	19064572
9611	nuclear receptor corepressor 1(NCOR1)	Yes	19064572
8648	nuclear receptor coactivator 1(NCOA1)	Yes	19064572
10800	cysteinyl leukotriene receptor 1(CYSLTR1)	Yes	19064572
92	activin A receptor type 2A(ACVR2A)	Yes	19064572
90	activin A receptor type 1(ACVR1)	Yes	19064572

51720	ubiquitin interaction motif containing 1(UIMC1)	Yes	19064572
3480	insulin like growth factor 1 receptor(IGF1R)	Yes	19064572
3482	insulin like growth factor 2 receptor(IGF2R)	Yes	19064572
3484	insulin like growth factor binding protein	Yes	19064572
324	APC, WNT signaling pathway regulator(APC)	Yes	19064572
9540	tumor protein p53 inducible protein 3(TP53I3)	Yes	19064572
116151	family with sequence similarity 210 member B(FAM210B)	Yes	19064572
9156	exonuclease 1(EXO1)	Yes	19064572
1643	damage specific DNA binding protein 2(DDB2)	Yes	19064572
80142	prostaglandin E synthase 2(PTGES2)	Yes	19064572
3070	helicase, lymphoid-specific(HELLS)	Yes	19064572
3066	histone deacetylase 2(HDAC2)	Yes	19064572
1594	cytochrome P450 family 27 subfamily B member 1(CYP27B1)	Yes	19064572
2690	growth hormone receptor(GHR)	Yes	19064572
3980	DNA ligase 3(LIG3)	Yes	19064572
3981	DNA ligase 4(LIG4)	Yes	19064572
3978	DNA ligase 1(LIG1)	Yes	19064572
1111	checkpoint kinase 1(CHEK1)	Yes	19064572
4438	mutS homolog 4(MSH4)	Yes	19064572
59344	arachidonate lipoxygenase 3(ALOXE3)	Yes	19064572
2072	ERCC excision repair 4, endonuclease catalytic subunit(ERCC4)	Yes	19064572
2074	ERCC excision repair 6, chromatin remodeling factor(ERCC6)	Yes	19064572
1571	cytochrome P450 family 2 subfamily E member 1(CYP2E1)	Yes	19064572
1559	cytochrome P450 family 2 subfamily C member 9(CYP2C9)	Yes	19064572
1551	cytochrome P450 family 3 subfamily A member 7(CYP3A7)	Yes	19064572

2033	E1A binding protein p300(EP300)	Yes	19064572
3554	interleukin 1 receptor type 1(IL1R1)	Yes	19064572
6916	thromboxane A synthase 1(TBXAS1)	Yes	19064572
6915	thromboxane A2 receptor(TBXA2R)	Yes	19064572
3625	inhibin beta B subunit(INHBB)	Yes	19064572
3626	inhibin beta C subunit(INHBC)	Yes	19064572
3623	inhibin alpha subunit(INHA)	Yes	19064572
2547	X-ray repair cross complementing 6(XRCC6)	Yes	19064572
242	arachidonate 12-lipoxygenase, 12R	Yes	19064572
247	arachidonate 15-lipoxygenase, type	Yes	19064572
54578	UDP glucuronosyltransferase family 1 member A6(UGT1A6)	Yes	19064572
5467	peroxisome proliferator activated receptor delta(PPARD)	Yes	19064572
9961	major vault protein(MVP)	No	19080004
3570	interleukin 6 receptor(IL6R)	Yes	19088723
29102	drosha ribonuclease III(DROSHA)	Yes	19092150
23405	dicer 1, ribonuclease III(DICER1)	Yes	19092150
5469	mediator complex subunit 1(MED1)	Yes	19127118
10643	insulin like growth factor 2 mRNA binding protein 3(IGF2BP3)	Yes	19136932
2525	fucosyltransferase 3 (Lewis blood group)(FUT3)	Yes	19137814
10987	COP9 signalosome subunit 5(COPS5)	No	19139918
1317	solute carrier family 31 member 1(SLC31A1)	No	19147760
6692	serine peptidase inhibitor, Kunitz type	Yes	19148468
7471	Wnt family member 1(WNT1)	Yes	19148501
7474	Wnt family member 5A(WNT5A)	Yes	19148501
8321	frizzled class receptor 1(FZD1)	Yes	19148501
7222	transient receptor potential cation channel subfamily C member 3(TRPC3)	Yes	19151765
9212	aurora kinase B(AURKB)	Yes	19157502

5465	peroxisome proliferator activated receptor alpha(PPARA)	Yes	19157507
2309	forkhead box O3(FOXO3)	Yes	19160093
64506	cytoplasmic polyadenylation element binding protein 1(CPEB1)	Yes	19161537
56667	mucin 13, cell surface associated(MUC13)	No	19176398
409	arrestin beta 2(ARRB2)	Yes	19202075
408	arrestin beta 1(ARRB1)	Yes	19202075
7412	vascular cell adhesion molecule 1(VCAM1)	Yes	19208843
5693	proteasome subunit beta 5(PSMB5)	No	19243813
4321	matrix metallopeptidase 12(MMP12)	Yes	19258954
84883	apoptosis inducing factor, mitochondria associated 2(AIFM2)	Yes	19270026
838	caspase 5(CASP5)	Yes	19270026
8607	RuvB like AAA ATPase 1(RUVBL1)	Yes	19270026
64400	AKT interacting protein(AKTIP)	Yes	19270026
10734	stromal antigen 3(STAG3)	Yes	19270026
5932	RB binding protein 8, endonuclease(RBBP8)	Yes	19270026
8313	axin 2(AXIN2)	Yes	19270026
28984	regulator of cell cycle(RGCC)	Yes	19270026
1994	ELAV like RNA binding protein 1(ELAVL1)	Yes	19281587
57167	spalt like transcription factor 4(SALL4)	Yes	19295406
11054	opioid growth factor receptor(OGFR)	No	19297547
5443	proopiomelanocortin(POMC)	Yes	19297547
5744	parathyroid hormone like hormone(PTHLH)	Yes	19304784
643714	cancer susceptibility candidate 16 (non-protein coding)(CASC16)	Yes	19304784
6150	mitochondrial ribosomal protein L23(MRPL23)	Yes	19304784
90480	GADD45G interacting protein 1(GADD45GIP1)	No	19305429
627	brain derived neurotrophic factor(BDNF)	Yes	19307055
8317	cell division cycle 7(CDC7)	Yes	19318489

216	aldehyde dehydrogenase 1 family member A1(ALDH1A1)	Yes	19329942
4591	tripartite motif containing 37(TRIM37)	No	19329943
1827	regulator of calcineurin 1(RCAN1)	Yes	19331211
5232	phosphoglycerate kinase 2(PGK2)	Yes	19333399
140801	ribosomal protein L10 like(RPL10L)	Yes	19333399
173	afamin(AFM)	Yes	19336561
337	apolipoprotein A4(APOA4)	Yes	19336561
6356	C-C motif chemokine ligand 11(CCL11)	Yes	19351767
3732	CD82 molecule(CD82)	Yes	19371633
2938	glutathione S-transferase alpha 1(GSTA1)	Yes	19376514
1555	cytochrome P450 family 2 subfamily B member 6(CYP2B6)	Yes	19376514
6498	SKI like proto-oncogene(SKIL)	Yes	19383336
9732	dedicator of cytokinesis 4(DOCK4)	Yes	19383911
64478	CUB and Sushi multiple domains 1(CSMD1)	Yes	19383911
100313779	microRNA 2117(MIR2117)	No	19390579
100313841	microRNA 548q(MIR548Q)	No	19390579
100313840	microRNA 2115(MIR2115)	No	19390579
100313839	microRNA 2114(MIR2114)	No	19390579
100313886	microRNA 2116(MIR2116)	No	19390579
100313923	microRNA 449c(MIR449C)	No	19390579
8742	tumor necrosis factor superfamily member 12(TNFSF12)	Yes	19398263
10893	matrix metallopeptidase 24(MMP24)	Yes	19403421
28992	MACRO domain containing 1(MACROD1)	No	19403568
1902	lysophosphatidic acid receptor 1(LPAR1)	Yes	19420982
7158	tumor protein p53 binding protein 1(TP53BP1)	Yes	19422301
5585	protein kinase N1(PKN1)	Yes	19427017
3373	hyaluronoglucosaminidase 1(HYAL1)	Yes	19435493
8692	hyaluronoglucosaminidase 2(HYAL2)	No	19435493
3037	hyaluronan synthase 2(HAS2)	Yes	19435493

3036	hyaluronan synthase 1(HAS1)	Yes	19435493
3038	hyaluronan synthase 3(HAS3)	Yes	19435493
51478	hydroxysteroid 17-beta dehydrogenase	Yes	19460435
8644	aldo-keto reductase family 1 member	Yes	19460435
51144	hydroxysteroid 17-beta dehydrogenase 12(HSD17B12)	Yes	19460435
2194	fatty acid synthase(FASN)	Yes	19467222
6657	SRY-box 2(SOX2)	Yes	19483629
10630	podoplanin(PDPN)	Yes	19483629
5460	POU class 5 homeobox 1(POU5F1)	Yes	19483629
311	annexin A11(ANXA11)	Yes	19484149
3162	heme oxygenase 1(HMOX1)	Yes	19484149
9641	inhibitor of kappa light polypeptide gene enhancer in B-cells, kinase epsilon(IKBKE)	Yes	19497997
4793	NFKB inhibitor beta(NFKBIB)	Yes	19500386
4792	NFKB inhibitor alpha(NFKBIA)	Yes	19500386
554210	microRNA 429(MIR429)	No	19501389
406984	microRNA 200b(MIR200B)	No	19501389
406983	microRNA 200a(MIR200A)	Yes	19501389
152330	contactin 4(CNTN4)	Yes	19509545
27255	contactin 6(CNTN6)	Yes	19509545
115761	ADP ribosylation factor like GTPase 11(ARL11)	Yes	19509554
3725	Jun proto-oncogene, AP-1 transcription factor subunit(JUN)	Yes	19513521
55862	ethylmalonyl-CoA decarboxylase 1(ECHDC1)	Yes	19517271
81847	ring finger protein 146(RNF146)	Yes	19517271
1361	carboxypeptidase B2(CPB2)	Yes	19526059
23512	SUZ12 polycomb repressive complex 2 subunit(SUZ12)	No	19542872
221895	JAZF zinc finger 1(JAZF1)	Yes	19542872
941	CD80 molecule(CD80)	Yes	19550123
942	CD86 molecule(CD86)	Yes	19550123

1116	chitinase 3 like 1(CHI3L1)	Yes	19550125
7913	DEK proto-oncogene(DEK)	Yes	19563407
307	annexin A4(ANXA4)	Yes	19598262
701	BUB1 mitotic checkpoint serine/threonine kinase B(BUB1B)	Yes	19603021
5652	protease, serine 8(PRSS8)	Yes	19606239
891	cyclin B1(CCNB1)	Yes	19608149
3832	kinesin family member 11(KIF11)	Yes	19619321
3308	heat shock protein family A (Hsp70) member 4(HSPA4)	Yes	19620938
966	CD59 molecule(CD59)	Yes	19622297
4170	BCL2 family apoptosis regulator(MCL1)	Yes	19634140
4246	secretoglobin family 2A member 1(SCGB2A1)	No	19635143
9134	cyclin E2(CCNE2)	Yes	19638577
25932	chloride intracellular channel 4(CLIC4)	Yes	19639201
6241	ribonucleotide reductase regulatory subunit M2(RRM2)	Yes	19639316
931	membrane spanning 4-domains A1(MS4A1)	Yes	19641607
7072	TIA1 cytotoxic granule associated RNA binding protein(TIA1)	No	19641607
54796	basonuclin 2(BNC2)	Yes	19648919
54875	centlein(CNTLN)	Yes	19648919
362	aquaporin 5(AQP5)	Yes	19664333
23552	cyclin dependent kinase 20(CDK20)	Yes	19672860
407046	microRNA 9-1(MIR9-1)	Yes	19702828
10761	placenta specific 1(PLAC1)	No	19705800
151871	developmental pluripotency associated	Yes	19705800
26085	kallikrein related peptidase 13(KLK13)	Yes	19707197
2923	protein disulfide isomerase family A member 3(PDIA3)	Yes	19714814
3201	homeobox A4(HOXA4)	Yes	19723874
2957	general transcription factor IIA subunit	Yes	19724865

23498	3-hydroxyanthranilate 3,4-dioxygenase(HAAO)	Yes	19724865
439	arsA arsenite transporter, ATP-binding, homolog 1 (bacterial)(ASNA1)	Yes	19724867
6934	transcription factor 7 like 2(TCF7L2)	Yes	19732438
840	caspase 7(CASP7)	Yes	19738422
835	caspase 2(CASP2)	Yes	19738422
5347	polo like kinase 1(PLK1)	Yes	19738611
994	cell division cycle 25B(CDC25B)	Yes	19738611
993	cell division cycle 25A(CDC25A)	Yes	19738611
1876	E2F transcription factor 6(E2F6)	Yes	19738611
1875	E2F transcription factor 5(E2F5)	Yes	19738611
1874	E2F transcription factor 4(E2F4)	Yes	19738611
1871	E2F transcription factor 3(E2F3)	Yes	19738611
7027	transcription factor Dp-1(TFDP1)	Yes	19738611
7029	transcription factor Dp-2(TFDP2)	Yes	19738611
890	cyclin A2(CCNA2)	Yes	19738611
25	ABL proto-oncogene 1, non-receptor tyrosine kinase(ABL1)	Yes	19738611
900	cyclin G1(CCNG1)	Yes	19738611
901	cyclin G2(CCNG2)	Yes	19738611
8454	cullin 1(CUL1)	Yes	19738611
9133	cyclin B2(CCNB2)	Yes	19738611
5933	RB transcriptional corepressor like 1(RBL1)	Yes	19738611
1022	cyclin dependent kinase 7(CDK7)	Yes	19738611
7132	TNF receptor superfamily member	Yes	19741298
4780	nuclear factor, erythroid 2 like 2(NFE2L2)	Yes	19751820
8869	ST3 beta-galactoside alpha-2,3-sialyltransferase 5(ST3GAL5)	Yes	19759399
2947	glutathione S-transferase mu 3(GSTM3)	Yes	19786980
4249	mannosyl (alpha-1,6-)-glycoprotein beta-1,6-N-acetyl-glucosaminyltransferase(MGAT5)	Yes	19787216
22943	dickkopf WNT signaling pathway inhibitor	Yes	19787627

60500	breast cancer 3(BRCA3)	No	19798552
1831	TSC22 domain family member 3(TSC22D3)	Yes	19814803
4090	SMAD family member 5(SMAD5)	Yes	19819941
4086	SMAD family member 1(SMAD1)	Yes	19819941
4087	SMAD family member 2(SMAD2)	Yes	19819941
7133	TNF receptor superfamily member	Yes	19825522
7099	toll like receptor 4(TLR4)	Yes	19826413
567	beta-2-microglobulin(B2M)	Yes	19829086
2197	FAU, ubiquitin like and ribosomal protein S30 fusion(FAU)	No	19830698
4221	menin 1(MEN1)	Yes	19837273
308	annexin A5(ANXA5)	Yes	19846818
4586	mucin 5AC, oligomeric mucus/gel-forming(MUC5AC)	Yes	19856421
8824	carboxylesterase 2(CES2)	Yes	19856659
5338	phospholipase D2(PLD2)	Yes	19864325
5463	POU class 6 homeobox 1(POU6F1)	No	19874398
10620	AT-rich interaction domain 3B(ARID3B)	Yes	19881956
406910	microRNA 125a(MIR125A)	Yes	19881956
22795	nidogen 2(NID2)	Yes	19883638
11178	leucine zipper tumor suppressor 1(LZTS1)	Yes	19885841
6277	S100 calcium binding protein A6(S100A6)	Yes	19888321
5052	peroxiredoxin 1(PRDX1)	Yes	19902980
406948	microRNA 15a(MIR15A)	No	19903841
1514	cathepsin L(CTSL)	No	19915865
1471	cystatin C(CST3)	Yes	19915865
7048	transforming growth factor beta receptor	Yes	19916025
4089	SMAD family member 4(SMAD4)	Yes	19916025
9459	Rac/Cdc42 guanine nucleotide exchange factor 6(ARHGEF6)	Yes	19918261
6271	S100 calcium binding protein A1(S100A1)	Yes	19926575

3316	heat shock protein family B (small) member 2(HSPB2)	Yes	19930842
11168	PC4 and SFRS1 interacting protein 1(PSIP1)	No	19934313
147372	collagen and calcium binding EGF domains	Yes	19935792
466	activating transcription factor 1(ATF1)	Yes	19950226
3838	karyopherin subunit alpha 2(KPNA2)	Yes	19950226
407018	microRNA 27a(MIR27A)	Yes	19950226
407048	microRNA 92a-1(MIR92A1)	Yes	19950226
3489	insulin like growth factor binding protein	Yes	19950226
3488	insulin like growth factor binding protein	Yes	19950226
5111	proliferating cell nuclear antigen(PCNA)	Yes	19950226
494335	microRNA 423(MIR423)	Yes	19950226
406932	microRNA 140(MIR140)	Yes	19950226
8314	BRCA1 associated protein 1(BAP1)	Yes	19950226
406941	microRNA 149(MIR149)	Yes	19950226
8850	lysine acetyltransferase 2B(KAT2B)	Yes	19950226
1509	cathepsin D(CTSD)	Yes	19950226
5440	RNA polymerase II subunit K(POLR2K)	Yes	19950226
3315	heat shock protein family B (small) member 1(HSPB1)	Yes	19955928
4029	LINE1 retrotransposable element 1(L1RE1)	No	19956846
6425	secreted frizzled related protein 5(SFRP5)	Yes	19957335
8076	microfibrillar associated protein 5(MFAP5)	No	19962670
2658	growth differentiation factor 2(GDF2)	Yes	19996292
7068	thyroid hormone receptor beta(THRB)	Yes	20004952
3206	homeobox A10(HOXA10)	Yes	20009888
919	CD247 molecule(CD247)	Yes	20032419
7122	claudin 5(CLDN5)	Yes	20044634
2516	nuclear receptor subfamily 5 group A member 1(NR5A1)	Yes	20045459
8841	histone deacetylase 3(HDAC3)	Yes	20049841
135250	retinoic acid early transcript 1E(RAET1E)	Yes	20054857

23345	spectrin repeat containing nuclear envelope protein 1(SYNE1)	Yes	20056644
4070	tumor-associated calcium signal transducer 2(TACSTD2)	Yes	20060709
114907	F-box protein 32(FBXO32)	Yes	20065949
727897	mucin 5B, oligomeric mucus/gel-	Yes	20066894
133584	EGF like, fibronectin type III and laminin G domains(EGFLAM)	Yes	20078962
406892	microRNA 100(MIR100)	Yes	20081105
149233	interleukin 23 receptor(IL23R)	Yes	20082850
3002	granzyme B(GZMB)	Yes	20087581
3280	hes family bHLH transcription factor 1(HES1)	Yes	20091184
388585	hes family bHLH transcription factor 5(HES5)	No	20091184
79727	lin-28 homolog A(LIN28A)	No	20101213
306	annexin A3(ANXA3)	Yes	20103635
1500	catenin delta 1(CTNND1)	No	20118984
6405	semaphorin 3F(SEMA3F)	Yes	20124444
7869	semaphorin 3B(SEMA3B)	Yes	20124444
5266	peptidase inhibitor 3(PI3)	Yes	20126474
5016	oviductal glycoprotein 1(OVGP1)	Yes	20130498
7033	trefoil factor 3(TFF3)	Yes	20132413
7031	trefoil factor 1(TFF1)	Yes	20132413
771	carbonic anhydrase 12(CA12)	Yes	20132413
3169	forkhead box A1(FOXA1)	Yes	20132413
29923	hypoxia inducible lipid droplet	No	20134266
2022	endoglin(ENG)	Yes	20134268
157638	family with sequence similarity 84 member B(FAM84B)	Yes	20158306
5462	POU class 5 homeobox 1B(POU5F1B)	Yes	20158306
4678	nuclear autoantigenic sperm protein(NASP)	No	20164540
406881	microRNA let-7a-1(MIRLET7A1)	Yes	20167074
442915	microRNA 370(MIR370)	Yes	20167074

407017	microRNA 26b(MIR26B)	Yes	20167074
407007	microRNA 222(MIR222)	Yes	20167074
407006	microRNA 221(MIR221)	Yes	20167074
407037	microRNA 320a(MIR320A)	Yes	20167074
407021	microRNA 29a(MIR29A)	Yes	20167074
407025	microRNA 29b-2(MIR29B2)	Yes	20167074
407050	microRNA 93(MIR93)	Yes	20167074
407051	microRNA 9-3(MIR9-3)	Yes	20167074
407047	microRNA 9-2(MIR9-2)	Yes	20167074
494330	microRNA 381(MIR381)	Yes	20167074
406931	microRNA 139(MIR139)	Yes	20167074
406935	microRNA 143(MIR143)	Yes	20167074
406921	microRNA 132(MIR132)	Yes	20167074
406900	microRNA 106b(MIR106B)	Yes	20167074
406998	microRNA 216a(MIR216A)	Yes	20167074
406975	microRNA 198(MIR198)	Yes	20167074
406974	microRNA 197(MIR197)	Yes	20167074
406982	microRNA 20a(MIR20A)	Yes	20167074
406988	microRNA 205(MIR205)	Yes	20167074
406979	microRNA 19a(MIR19A)	Yes	20167074
406946	microRNA 154(MIR154)	Yes	20167074
406964	microRNA 188(MIR188)	Yes	20167074
406966	microRNA 191(MIR191)	Yes	20167074
406961	microRNA 185(MIR185)	Yes	20167074
2524	fucosyltransferase 2(FUT2)	Yes	20172014
407035	microRNA 31(MIR31)	No	20179198
26136	testin LIM domain protein(TES)	Yes	20180808
25805	BMP and activin membrane bound	Yes	20189233
2300	forkhead box L1(FOXL1)	Yes	20198651
1462	versican(VCAN)	Yes	20204274
3084	neuregulin 1(NRG1)	Yes	20227043
2066	erb-b2 receptor tyrosine kinase 4(ERBB4)	Yes	20227043

1398	CRK proto-oncogene, adaptor protein(CRK)	Yes	20237902
1793	dedicator of cytokinesis 1(DOCK1)	Yes	20237902
5879	ras-related C3 botulinum toxin substrate 1 (rho family, small GTP binding protein Rac1)(RAC1)	Yes	20237902
3248	hydroxyprostaglandin dehydrogenase 15-(NAD)(HPGD)	Yes	20304053
1591	cytochrome P450 family 24 subfamily A member 1(CYP24A1)	Yes	20304053
54984	PIN2/TERF1 interacting, telomerase inhibitor 1(PINX1)	Yes	20367640
51752	endoplasmic reticulum aminopeptidase	Yes	20367925
5592	protein kinase, cGMP-dependent, type I(PRKG1)	Yes	20371672
6331	sodium voltage-gated channel alpha subunit 5(SCN5A)	Yes	20372843
23093	tubulin tyrosine ligase like 5(TTLL5)	Yes	20374646
397	Rho GDP dissociation inhibitor beta(ARHGDIB)	Yes	20375790
8140	solute carrier family 7 member 5(SLC7A5)	Yes	20375792
9825	spermatogenesis associated 2(SPATA2)	Yes	20385810
3902	lymphocyte activating 3(LAG3)	Yes	20385810
5889	RAD51 paralog C(RAD51C)	Yes	20400964
100628315	DNM3 opposite strand/antisense	No	20400975
8877	sphingosine kinase 1(SPHK1)	No	20404323
26060	adaptor protein, phosphotyrosine interacting with PH domain and leucine zipper 1(APPL1)	Yes	20412119
5868	RAB5A, member RAS oncogene	Yes	20412119
112464	protein kinase C delta binding	Yes	20423276
4100	MAGE family member A1(MAGEA1)	Yes	20423514
4102	MAGE family member A3(MAGEA3)	No	20423514
2543	G antigen 1(GAGE1)	No	20423514
6781	stanniocalcin 1(STC1)	Yes	20484106
5590	protein kinase C zeta(PRKCZ)	Yes	20501645
10963	stress induced phosphoprotein 1(STIP1)	Yes	20501939

27324	TOX high mobility group box family member 3(TOX3)	Yes	20502973
10551	anterior gradient 2, protein disulphide isomerase family member(AGR2)	Yes	20525245
4192	midkine (neurite growth-promoting factor	Yes	20525245
27122	dickkopf WNT signaling pathway inhibitor	Yes	20532910
2764	glia maturation factor beta(GMFB)	Yes	20547056
5971	RELB proto-oncogene, NF-kB subunit(RELB)	Yes	20564628
10588	5,10-methenyltetrahydrofolate synthetase (5-formyltetrahydrofolate cyclo-ligase)(MTHFS)	Yes	20570913
4522	methylenetetrahydrofolate dehydrogenase, cyclohydrolase and formyltetrahydrofolate synthetase 1(MTHFD1)	Yes	20570913
9518	growth differentiation factor 15(GDF15)	Yes	20576287
6777	signal transducer and activator of transcription 5B(STAT5B)	Yes	20585448
659	bone morphogenetic protein receptor type	Yes	20587070
657	bone morphogenetic protein receptor type 1A(BMPR1A)	Yes	20587070
658	bone morphogenetic protein receptor type 1B(BMPR1B)	Yes	20587070
51561	interleukin 23 subunit alpha(IL23A)	Yes	20603238
10135	nicotinamide	Yes	20606733
865	core-binding factor beta subunit(CBFB)	Yes	20607802
1081	glycoprotein hormones, alpha polypeptide(CGA)	Yes	20613903
8614	stanniocalcin 2(STC2)	Yes	20619259
9514	galactose-3-O-sulfotransferase 1(GAL3ST1)	No	20624317
1825	desmocollin 3(DSC3)	Yes	20628624
5352	procollagen-lysine,2-oxoglutarate 5-dioxygenase 2(PLOD2)	Yes	20628624
5351	procollagen-lysine,2-oxoglutarate 5-dioxygenase 1(PLOD1)	Yes	20628624

1847	dual specificity phosphatase 5(DUSP5)	Yes	20628624
970	CD70 molecule(CD70)	Yes	20628624
8519	interferon induced transmembrane protein	Yes	20628624
3212	homeobox B2(HOXB2)	Yes	20628624
8974	prolyl 4-hydroxylase subunit alpha 2(P4HA2)	Yes	20628624
3655	integrin subunit alpha 6(ITGA6)	Yes	20628624
2921	C-X-C motif chemokine ligand 3(CXCL3)	Yes	20628624
2537	interferon alpha inducible protein 6(IFI6)	Yes	20628624
3665	interferon regulatory factor 7(IRF7)	Yes	20628624
7060	thrombospondin 4(THBS4)	Yes	20628624
7071	Kruppel like factor 10(KLF10)	Yes	20628624
7078	TIMP metallopeptidase inhibitor 3(TIMP3)	Yes	20628624
4232	mesoderm specific transcript(MEST)	Yes	20628624
4239	microfibrillar associated protein 4(MFAP4)	Yes	20628624
5770	protein tyrosine phosphatase, non-receptor type 1(PTPN1)	Yes	20628624
10581	interferon induced transmembrane protein	Yes	20628624
4283	C-X-C motif chemokine ligand 9(CXCL9)	Yes	20628624
8575	protein activator of interferon induced protein kinase EIF2AK2(PRKRA)	Yes	20628624
51379	cytokine receptor like factor 3(CRLF3)	Yes	20628624
6772	signal transducer and activator of transcription 1(STAT1)	Yes	20628624
6357	C-C motif chemokine ligand 13(CCL13)	Yes	20628624
6354	C-C motif chemokine ligand 7(CCL7)	Yes	20628624
7480	Wnt family member 10B(WNT10B)	Yes	20628624
6372	C-X-C motif chemokine ligand 6(CXCL6)	Yes	20628624
3398	inhibitor of DNA binding 2, HLH protein(ID2)	Yes	20628624
7472	Wnt family member 2(WNT2)	Yes	20628624
2879	glutathione peroxidase 4(GPX4)	Yes	20628624
9636	ISG15 ubiquitin-like modifier(ISG15)	Yes	20628624

4175	minichromosome maintenance complex component 6(MCM6)	Yes	20628624
4171	minichromosome maintenance complex component 2(MCM2)	Yes	20628624
9232	pituitary tumor-transforming 1(PTTG1)	Yes	20628624
6737	tripartite motif containing 21(TRIM21)	Yes	20628624
2323	fms related tyrosine kinase 3 ligand(FLT3LG)	Yes	20628624
5610	eukaryotic translation initiation factor 2 alpha kinase 2(EIF2AK2)	Yes	20628624
5612	THAP domain containing 12(THAP12)	Yes	20628624
10468	follistatin(FST)	Yes	20628624
4599	MX dynamin like GTPase 1(MX1)	Yes	20628624
7803	protein tyrosine phosphatase type IVA, member 1(PTP4A1)	Yes	20628624
101	ADAM metallopeptidase domain 8(ADAM8)	Yes	20628624
7127	TNF alpha induced protein 2(TNFAIP2)	Yes	20628624
7130	TNF alpha induced protein 6(TNFAIP6)	Yes	20628624
51330	TNF receptor superfamily member 12A(TNFRSF12A)	Yes	20628624
7128	TNF alpha induced protein 3(TNFAIP3)	Yes	20628624
8744	tumor necrosis factor superfamily member 9(TNFSF9)	Yes	20628624
718	complement C3(C3)	Yes	20628624
710	serpin family G member 1(SERPING1)	Yes	20628624
8710	serpin family B member 7(SERPINB7)	Yes	20628624
8473	O-linked N-acetylglucosamine (GlcNAc) transferase(OGT)	Yes	20628624
3005	H1 histone family member 0(H1F0)	Yes	20628624
4921	discoidin domain receptor tyrosine kinase	Yes	20628624
9547	C-X-C motif chemokine ligand 14(CXCL14)	Yes	20628624
4938	2'-5'-oligoadenylate synthetase 1(OAS1)	Yes	20628624
4940	2'-5'-oligoadenylate synthetase 3(OAS3)	Yes	20628624

3430	interferon induced protein 35(IFI35)	Yes	20628624
6278	S100 calcium binding protein A7(S100A7)	Yes	20628624
6272	sortilin 1(SORT1)	Yes	20628624
1634	decorin(DCN)	Yes	20628624
3428	interferon gamma inducible protein 16(IFI16)	Yes	20628624
2705	gap junction protein beta 1(GJB1)	Yes	20628624
3434	interferon induced protein with tetratricopeptide repeats 1(IFIT1)	Yes	20628624
24145	pannexin 1(PANX1)	Yes	20628624
9536	prostaglandin E synthase(PTGES)	Yes	20628624
4907	5'-nucleotidase ecto(NT5E)	Yes	20628624
3915	laminin subunit gamma 1(LAMC1)	Yes	20628624
10380	3'(2'), 5'-bisphosphate nucleotidase 1(BPNT1)	Yes	20628624
7305	TYRO protein tyrosine kinase binding protein(TYROBP)	Yes	20628624
6652	sorbitol dehydrogenase(SORD)	Yes	20628624
5997	regulator of G-protein signaling 2(RGS2)	Yes	20628624
8840	WNT1 inducible signaling pathway protein	Yes	20628624
10663	C-X-C motif chemokine receptor 6(CXCR6)	Yes	20628624
684	bone marrow stromal cell antigen 2(BST2)	Yes	20628624
7297	tyrosine kinase 2(TYK2)	Yes	20628624
23710	GABA type A receptor associated protein like 1(GABARAPL1)	Yes	20628624
1513	cathepsin K(CTSK)	Yes	20628624
699	BUB1 mitotic checkpoint serine/threonine kinase(BUB1)	Yes	20628624
649	bone morphogenetic protein 1(BMP1)	Yes	20628624
2619	growth arrest specific 1(GAS1)	Yes	20628624
6940	zinc finger protein 354A(ZNF354A)	Yes	20628624
3572	interleukin 6 signal transducer(IL6ST)	Yes	20628624
1537	cytochrome c1(CYC1)	Yes	20628624

1979	eukaryotic translation initiation factor 4E binding protein 2(EIF4EBP2)	Yes	20628624
4791	nuclear factor kappa B subunit 2(NFKB2)	Yes	20628624
4351	mannose phosphate isomerase(MPI)	Yes	20628624
10252	sprouty RTK signaling antagonist 1(SPRY1)	Yes	20628624
56547	matrix metallopeptidase 26(MMP26)	Yes	20628624
26872	STEAP family member 1(STEAP1)	Yes	20628624
5420	podocalyxin like(PODXL)	Yes	20628624
5055	serpin family B member 2(SERPINB2)	Yes	20628624
1959	early growth response 2(EGR2)	Yes	20628624
91543	radical S-adenosyl methionine domain containing 2(RSAD2)	Yes	20628624
683	bone marrow stromal cell antigen 1(BST1)	Yes	20639476
23136	erythrocyte membrane protein band 4.1 like 3(EPB41L3)	Yes	20651987
9122	solute carrier family 16 member 4(SLC16A4)	No	20658178
28985	MCTS1, re-initiation and release factor(MCTS1)	No	20658178
602	B-cell CLL/lymphoma 3(BCL3)	Yes	20658525
406911	microRNA 125b-1(MIR125B1)	Yes	20658525
406912	microRNA 125b-2(MIR125B2)	Yes	20658525
3077	hemochromatosis(HFE)	Yes	20669231
2013	epithelial membrane protein 2(EMP2)	Yes	20670949
2146	enhancer of zeste 2 polycomb repressive complex 2 subunit(EZH2)	Yes	20686362
11190	centrosomal protein 250(CEP250)	Yes	20708153
23235	salt inducible kinase 2(SIK2)	Yes	20708153
6288	serum amyloid A1(SAA1)	Yes	20713982
5935	RNA binding motif (RNP1, RRM) protein	No	20727170
442914	microRNA 369(MIR369)	Yes	20810544
406952	microRNA 17(MIR17)	Yes	20810544
260436	follicular dendritic cell secreted protein(FDCSP)	Yes	20811673
8289	AT-rich interaction domain 1A(ARID1A)	No	20826764

2354	FosB proto-oncogene, AP-1 transcription factor subunit(FOSB)	Yes	20826776
23569	peptidyl arginine deiminase 4(PADI4)	Yes	20827398
7476	Wnt family member 7A(WNT7A)	Yes	20845993
114569	mal, T-cell differentiation protein 2 (gene/pseudogene)(MAL2)	Yes	20846453
102800310	HAGLR opposite strand (non-protein coding)(HAGLROS)	No	20852632
3231	homeobox D1(HOXD1)	Yes	20852632
3232	homeobox D3(HOXD3)	Yes	20852632
401022	HOXD antisense growth-associated long non-coding RNA(HAGLR)	No	20852632
8631	src kinase associated phosphoprotein 1(SKAP1)	Yes	20852632
25976	TCDD inducible poly(ADP-ribose) polymerase(TIPARP)	Yes	20852632
29086	BRISC and BRCA1 A complex member 1(BABAM1)	Yes	20852633
126549	ankyrin repeat and LEM domain containing 1(ANKLE1)	Yes	20852633
6428	serine and arginine rich splicing factor 3(SRSF3)	Yes	20856201
9149	dual specificity tyrosine phosphorylation regulated kinase 1B(DYRK1B)	No	20857490
10298	p21 (RAC1) activated kinase 4(PAK4)	Yes	20926745
10413	Yes associated protein 1(YAP1)	Yes	20947521
81607	nectin cell adhesion molecule 4(NECTIN4)	No	20959669
84548	transmembrane protein 185A(TMEM185A)	No	20969748
10335	murine retrovirus integration site 1	Yes	20969748
2119	ETS variant 5(ETV5)	Yes	20970160
843	caspase 10(CASP10)	Yes	20978178
6001	regulator of G-protein signaling 10(RGS10)	Yes	21044322
26575	regulator of G-protein signaling 17(RGS17)	Yes	21044322
1749	distal-less homeobox 5(DLX5)	Yes	21045156

8660	insulin receptor substrate 2(IRS2)	Yes	21045156
406985	microRNA 200c(MIR200C)	Yes	21051560
5364	plexin B1(PLXNB1)	Yes	21059203
4176	minichromosome maintenance complex component 7(MCM7)	Yes	21076460
1800	dipeptidase 1 (renal)(DPEP1)	Yes	21076463
83888	fibroblast growth factor binding protein	No	21079801
5425	DNA polymerase delta 2, accessory	Yes	21079801
4194	MDM4, p53 regulator(MDM4)	Yes	21084273
57535	KIAA1324(KIAA1324)	Yes	21102415
5104	serpin family A member 5(SERPINA5)	Yes	21102419
388228	SH3 domain binding kinase 1(SBK1)	Yes	21104019
11118	butyrophilin subfamily 3 member A2(BTN3A2)	Yes	21113407
11119	butyrophilin subfamily 3 member A1(BTN3A1)	Yes	21113407
494337	microRNA 425(MIR425)	No	21118967
56034	platelet derived growth factor C(PDGFC)	Yes	21118967
10006	abl interactor 1(ABI1)	Yes	21118970
2059	epidermal growth factor receptor pathway substrate 8(EPS8)	Yes	21118970
10516	fibulin 5(FBLN5)	Yes	21122382
3756	potassium voltage-gated channel subfamily H member 1(KCNH1)	Yes	21138547
9141	programmed cell death 5(PDCD5)	Yes	21165576
9588	peroxiredoxin 6(PRDX6)	Yes	21166495
2852	G protein-coupled estrogen receptor 1(GPER1)	Yes	21170498
51341	zinc finger and BTB domain containing	No	21176152
407004	microRNA 22(MIR22)	No	21176563
406959	microRNA 183(MIR183)	No	21176563
7341	small ubiquitin-like modifier 1(SUMO1)	Yes	21187340
6352	C-C motif chemokine ligand 5(CCL5)	Yes	21196302
54106	toll like receptor 9(TLR9)	Yes	21223799
442913	microRNA 376c(MIR376C)	No	21224400

407040	microRNA 34a(MIR34A)	No	21225432
1072	cofilin 1(CFL1)	Yes	21237490
7706	tripartite motif containing 25(TRIM25)	Yes	21266548
5564	protein kinase AMP-activated non-catalytic subunit beta 1(PRKAB1)	Yes	21271224
28987	NIN1/PSMD8 binding protein 1	No	21287298
1434	chromosome segregation 1 like(CSE1L)	Yes	21290345
92140	metadherin(MTDH)	Yes	21293286
10664	CCCTC-binding factor(CTCF)	Yes	21296871
54937	spermatogenesis and oogenesis specific basic helix-loop-helix 2(SOHLH2)	Yes	21296871
2268	FGR proto-oncogene, Src family tyrosine kinase(FGR)	Yes	21300758
9531	BCL2 associated athanogene 3(BAG3)	Yes	21316839
1399	CRK like proto-oncogene, adaptor	Yes	21319228
693123	microRNA 449b(MIR449B)	No	21321636
407042	microRNA 34c(MIR34C)	No	21321636
3958	galectin 3(LGALS3)	Yes	21327452
613	BCR, RhoGEF and GTPase activating	Yes	21330459
26084	Rho guanine nucleotide exchange factor 26(ARHGEF26)	No	21332407
26017	family with sequence similarity 32 member A(FAM32A)	Yes	21339736
25855	breast cancer metastasis suppressor 1(BRMS1)	No	21355308
6304	SATB homeobox 1(SATB1)	Yes	21355308
9071	claudin 10(CLDN10)	Yes	21370593
8878	sequestosome 1(SQSTM1)	Yes	21371883
8725	URI1, prefoldin like chaperone(URI1)	Yes	21397856
5501	protein phosphatase 1 catalytic subunit gamma(PPP1CC)	Yes	21397856
23387	SIK family kinase 3(SIK3)	Yes	21399663
60681	FK506 binding protein 10(FKBP10)	No	21399973

10769	polo like kinase 2(PLK2)	Yes	21402713
10644	insulin like growth factor 2 mRNA binding protein 2(IGF2BP2)	Yes	21422097
8434	reversion inducing cysteine rich protein with kazal motifs(RECK)	Yes	21432940
23705	cell adhesion molecule 1(CADM1)	Yes	21436696
8912	calcium voltage-gated channel subunit alpha1 H(CACNA1H)	Yes	21438841
8913	calcium voltage-gated channel subunit alpha1 G(CACNA1G)	Yes	21438841
4000	lamin A/C(LMNA)	Yes	21439080
7978	mitochondrial transcription termination factor 1(MTERF1)	Yes	21447778
817	calcium/calmodulin dependent protein kinase II delta(CAMK2D)	Yes	21447778
4899	nuclear respiratory factor 1(NRF1)	Yes	21447778
155465	anterior gradient 3, protein disulphide isomerase family member(AGR3)	No	21451362
10397	N-myc downstream regulated 1(NDRG1)	Yes	21463610
6091	roundabout guidance receptor 1(ROBO1)	Yes	21465248
9353	slit guidance ligand 2(SLIT2)	Yes	21465248
54538	roundabout guidance receptor 4(ROBO4)	Yes	21465248
6586	slit guidance ligand 3(SLIT3)	Yes	21465248
127	alcohol dehydrogenase 4 (class II), pi polypeptide(ADH4)	Yes	21480392
5782	protein tyrosine phosphatase, non-receptor type 12(PTPN12)	Yes	21483099
5598	mitogen-activated protein kinase 7(MAPK7)	Yes	21483099
4488	msh homeobox 2(MSX2)	Yes	21499300
1942	ephrin A1(EFNA1)	No	21500549
823	calpain 1(CAPN1)	No	21516125
58191	C-X-C motif chemokine ligand 16(CXCL16)	Yes	21527066

101234261	long stress-induced non-coding transcript 5(LSINCT5)	No	21532345
55269	paraspeckle component 1(PSPC1)	No	21532345
283131	nuclear paraspeckle assembly transcript 1 (non-protein coding)(NEAT1)	Yes	21532345
4838	nodal growth differentiation factor(NODAL)	Yes	21532621
4613	v-myc avian myelocytomatosis viral oncogene neuroblastoma derived homolog(MYCN)	Yes	21533284
389421	lin-28 homolog B(LIN28B)	Yes	21533284
862	RUNX1 translocation partner 1(RUNX1T1)	Yes	21540640
7150	topoisomerase (DNA) I(TOP1)	Yes	21542140
7155	topoisomerase (DNA) II beta(TOP2B)	Yes	21542140
23129	plexin D1(PLXND1)	No	21559368
9723	semaphorin 3E(SEMA3E)	Yes	21559368
9759	histone deacetylase 4(HDAC4)	Yes	21571862
2984	guanylate cyclase 2C(GUCY2C)	No	21577013
7050	TGFB induced factor homeobox 1(TGIF1)	Yes	21577013
10140	transducer of ERBB2, 1(TOB1)	Yes	21577013
5798	protein tyrosine phosphatase, receptor type N(PTPRN)	Yes	21577013
9687	growth regulation by estrogen in breast cancer 1(GREB1)	Yes	21577013
145942	transmembrane and coiled-coil domains 5A(TMCO5A)	Yes	21577013
64866	CUB domain containing protein 1(CDCP1)	No	21617380
8038	ADAM metallopeptidase domain 12(ADAM12)	Yes	21617380
9928	kinesin family member 14(KIF14)	No	21618518
406886	microRNA let-7d(MIRLET7D)	No	21618519
406890	microRNA let-7g(MIRLET7G)	No	21618519
6370	C-C motif chemokine ligand 25(CCL25)	Yes	21637913
10803	C-C motif chemokine receptor 9(CCR9)	Yes	21637913
203068	tubulin beta class I(TUBB)	Yes	21653684

10018	BCL2 like 11(BCL2L11)	Yes	21655183
2254	fibroblast growth factor 9(FGF9)	Yes	21666490
9817	kelch like ECH associated protein 1(KEAP1)	Yes	21676886
768	carbonic anhydrase 9(CA9)	Yes	21691815
10718	neuregulin 3(NRG3)	Yes	21705454
10568	solute carrier family 34 member 2(SLC34A2)	No	21716206
10599	solute carrier organic anion transporter family member 1B1(SLCO1B1)	Yes	21719246
28234	solute carrier organic anion transporter family member 1B3(SLCO1B3)	Yes	21719246
406963	microRNA 187(MIR187)	No	21725366
2260	fibroblast growth factor receptor 1(FGFR1)	Yes	21739604
8111	G protein-coupled receptor 68(GPR68)	No	21740742
3146	high mobility group box 1(HMGB1)	Yes	21743489
6376	C-X3-C motif chemokine ligand 1(CX3CL1)	Yes	21750716
164091	progestin and adipoQ receptor family member 7(PAQR7)	No	21761364
54852	progestin and adipoQ receptor family member 5(PAQR5)	Yes	21761364
85315	progestin and adipoQ receptor family member 8(PAQR8)	No	21761364
2069	epiregulin(EREG)	Yes	21769422
354	kallikrein related peptidase 3(KLK3)	Yes	21787114
5892	RAD51 paralog D(RAD51D)	Yes	21822267
64078	solute carrier family 28 member 3(SLC28A3)	Yes	21822668
222962	solute carrier family 29 member 4(SLC29A4)	Yes	21822668
2030	solute carrier family 29 member 1 (Augustine blood group)(SLC29A1)	Yes	21822668
3177	solute carrier family 29 member 2(SLC29A2)	Yes	21822668
578	BCL2 antagonist/killer 1(BAK1)	Yes	21823019
116173	CKLF like MARVEL transmembrane domain containing 5(CMTM5)	No	21841490

11031	RAB31, member RAS oncogene family(RAB31)	Yes	21848504
26298	ETS homologous factor(EHF)	Yes	21855111
51435	scavenger receptor class A member 3(SCARA3)	Yes	21855113
1796	docking protein 1(DOK1)	Yes	21856257
2305	forkhead box M1(FOXM1)	Yes	21858223
27113	BCL2 binding component 3(BBC3)	Yes	21863213
4065	lymphocyte antigen 75(LY75)	No	21887537
26354	G protein nucleolar 3(GNL3)	Yes	21892090
57650	KIAA1524(KIAA1524)	No	21897396
346389	MACC1, MET transcriptional	Yes	21923915
10848	protein phosphatase 1 regulatory subunit 13 like(PPP1R13L)	Yes	21926165
8493	protein phosphatase, Mg2+/Mn2+ dependent 1D(PPM1D)	Yes	21927021
11009	interleukin 24(IL24)	Yes	21931671
4057	lactotransferrin(LTF)	Yes	21937479
6664	SRY-box 11(SOX11)	Yes	21943380
25858	testis expressed 40(TEX40)	No	21949640
9564	BCAR1, Cas family scaffolding protein(BCAR1)	Yes	21957230
406940	microRNA 148a(MIR148A)	No	21971665
406943	microRNA 152(MIR152)	No	21971665
6887	TAL bHLH transcription factor 2(TAL2)	Yes	21971700
7329	ubiquitin conjugating enzyme E2 I(UBE2I)	Yes	21971700
3609	interleukin enhancer binding factor 3(ILF3)	Yes	21971700
4091	SMAD family member 6(SMAD6)	Yes	21984931
406920	microRNA 130b(MIR130B)	No	22005523
137492	VPS37A, ESCRT-I subunit(VPS37A)	Yes	22016507
5950	retinol binding protein 4(RBP4)	Yes	22020625
79084	WD repeat domain 77(WDR77)	Yes	22022581
407032	microRNA 30c-2(MIR30C2)	Yes	22024689
607	B-cell CLL/lymphoma 9(BCL9)	Yes	22024689
51701	nemo like kinase(NLK)	Yes	22027747

2264	fibroblast growth factor receptor 4(FGFR4)	Yes	22034009
7498	xanthine dehydrogenase(XDH)	Yes	22044687
1524	C-X3-C motif chemokine receptor 1(CX3CR1)	Yes	22064656
7113	transmembrane protease, serine 2(TMPRSS2)	Yes	22076164
2078	ERG, ETS transcription factor(ERG)	Yes	22076164
407014	microRNA 25(MIR25)	No	22076535
28514	delta like canonical Notch ligand 1(DLL1)	Yes	22080880
8870	immediate early response 3(IER3)	Yes	22085302
928	CD9 molecule(CD9)	Yes	22095071
7019	transcription factor A, mitochondrial(TFAM)	Yes	22098591
406933	microRNA 141(MIR141)	No	22101765
79094	ChaC glutathione specific gamma-glutamylcyclotransferase 1(CHAC1)	No	22108517
23113	cullin 9(CUL9)	Yes	22117079
29979	ubiquilin 1(UBQLN1)	Yes	22134777
4673	nucleosome assembly protein 1 like 1(NAP1L1)	No	22134777
6523	solute carrier family 5 member 1(SLC5A1)	Yes	22159627
2308	forkhead box O1(FOXO1)	Yes	22159921
7280	tubulin beta 2A class IIa(TUBB2A)	Yes	22180309
7090	transducin like enhancer of split 3(TLE3)	Yes	22194527
9966	tumor necrosis factor superfamily member 15(TNFSF15)	Yes	22210436
3718	Janus kinase 3(JAK3)	Yes	22221142
51147	inhibitor of growth family member 4(ING4)	No	22228137
6591	snail family transcriptional repressor 2(SNAI2)	Yes	22232246
1277	collagen type I alpha 1 chain(COL1A1)	Yes	22249249
9823	armadillo repeat containing, X-linked	Yes	22249249
9724	UTP14, small subunit processome component homolog C (S. cerevisiae)(UTP14C)	No	22285623
89778	serpin family B member 11 (gene/pseudogene)(SERPINB11)	Yes	22289513
10763	nestin(NES)	Yes	22296500

406958	microRNA 182(MIR182)	Yes	22322863
824	calpain 2(CAPN2)	Yes	22335024
54756	interleukin 17 receptor D(IL17RD)	Yes	22335910
114757	cytoglobin(CYGB)	No	22344671
79651	rhomboid 5 homolog 2(RHBDF2)	Yes	22344671
442892	microRNA 148b(MIR148B)	No	22344713
22891	zinc finger protein 365(ZNF365)	Yes	22351618
6597	SWI/SNF related, matrix associated, actin dependent regulator of chromatin, subfamily a, member 4(SMARCA4)	Yes	22363625
6424	secreted frizzled related protein 4(SFRP4)	Yes	22363760
1602	dachshund family transcription factor 1(DACH1)	Yes	22367319
10016	programmed cell death 6(PDCD6)	No	22369209
3956	galectin 1(LGALS1)	Yes	22386573
4803	nerve growth factor(NGF)	Yes	22411587
8115	T-cell leukemia/lymphoma 1A(TCL1A)	Yes	22448662
2729	glutamate-cysteine ligase catalytic	Yes	22452920
406937	microRNA 145(MIR145)	Yes	22452920
406922	microRNA 133a-1(MIR133A1)	Yes	22452920
10635	RAD51 associated protein 1(RAD51AP1)	Yes	22452920
7074	T-cell lymphoma invasion and metastasis	Yes	22469921
80310	platelet derived growth factor D(PDGFD)	Yes	22471482
3309	heat shock protein family A (Hsp70) member 5(HSPA5)	Yes	22495374
1915	eukaryotic translation elongation factor 1 alpha 1(EEF1A1)	No	22531302
79812	multimerin 2(MMRN2)	No	22532453
7965	aminoacyl tRNA synthetase complex interacting multifunctional protein 2(AIMP2)	Yes	22532625
4015	lysyl oxidase(LOX)	Yes	22533780
5196	platelet factor 4(PF4)	Yes	22555803
238	anaplastic lymphoma receptor tyrosine	Yes	22570254

1636	angiotensin I converting enzyme(ACE)	Yes	22571796
7431	vimentin(VIM)	Yes	22584948
5366	phorbol-12-myristate-13-acetate-induced protein 1(PMAIP1)	Yes	22590594
10544	protein C receptor(PROCR)	Yes	22614534
406919	microRNA 130a(MIR130A)	No	22614869
2920	C-X-C motif chemokine ligand 2(CXCL2)	Yes	22615136
5308	paired like homeodomain 2(PITX2)	Yes	22615897
9168	thymosin beta 10(TMSB10)	No	22623951
7482	Wnt family member 2B(WNT2B)	Yes	22635028
2891	glutamate ionotropic receptor AMPA type subunit 2(GRIA2)	Yes	22644307
26523	argonaute 1, RISC catalytic component(AGO1)	Yes	22647351
27161	argonaute 2, RISC catalytic component(AGO2)	Yes	22647351
4296	mitogen-activated protein kinase kinase kinase 11(MAP3K11)	No	22652451
5978	RE1 silencing transcription factor(REST)	Yes	22684772
1454	casein kinase 1 epsilon(CSNK1E)	Yes	22707389
292	solute carrier family 25 member 5(SLC25A5)	No	22707389
57019	cytokine induced apoptosis inhibitor 1(CIAPIN1)	No	22713669
4926	nuclear mitotic apparatus protein 1(NUMA1)	Yes	22719996
8936	WAS protein family member 1(WASF1)	No	22721732
132299	OCIA domain containing 2(OCIAD2)	No	22726067
3667	insulin receptor substrate 1(IRS1)	Yes	22729394
7546	Zic family member 2(ZIC2)	Yes	22733541
54878	dipeptidyl peptidase 8(DPP8)	No	22736146
91039	dipeptidyl peptidase 9(DPP9)	Yes	22736146
5550	prolyl endopeptidase(PREP)	Yes	22740343
54739	XIAP associated factor 1(XAF1)	No	22759793
10962	myeloid/lymphoid or mixed-lineage leukemia; translocated to, 11(MLLT11)	No	22761939
79444	baculoviral IAP repeat containing 7(BIRC7)	No	22766624

708	complement C1q binding protein(C1QBP)	Yes	22771308
25953	paroxysmal nonkinesigenic dyskinesia(PNKD)	Yes	22780969
2833	C-X-C motif chemokine receptor 3(CXCR3)	Yes	22798340
5423	DNA polymerase beta(POLB)	Yes	22799326
1015	cadherin 17(CDH17)	Yes	22810971
79805	vasohibin 2(VASH2)	No	22826464
407045	microRNA 7-3(MIR7-3)	No	22853714
407043	microRNA 7-1(MIR7-1)	No	22853714
407044	microRNA 7-2(MIR7-2)	No	22853714
406916	microRNA 128-2(MIR128-2)	Yes	22853714
406915	microRNA 128-1(MIR128-1)	Yes	22853714
10320	IKAROS family zinc finger 1(IKZF1)	Yes	22859015
574504	microRNA 502(MIR502)	No	22867998
387893	lysine methyltransferase 5A(KMT5A)	Yes	22867998
7415	valosin containing protein(VCP)	Yes	22870330
7007	tectorin alpha(TECTA)	Yes	22877241
25950	RWD domain containing 3(RWDD3)	Yes	22877241
51422	protein kinase AMP-activated non-catalytic subunit gamma 2(PRKAG2)	Yes	22897928
5565	protein kinase AMP-activated non-catalytic subunit beta 2(PRKAB2)	Yes	22897928
5563	protein kinase AMP-activated catalytic subunit alpha 2(PRKAA2)	Yes	22897928
5571	protein kinase AMP-activated non-catalytic subunit gamma 1(PRKAG1)	Yes	22897928
4016	lysyl oxidase like 1(LOXL1)	Yes	22906264
3984	LIM domain kinase 1(LIMK1)	Yes	22906279
693124	microRNA 532(MIR532)	No	22925189
145226	retinol dehydrogenase 12 (all-trans/9-cis/11-cis)(RDH12)	Yes	22926683
9734	histone deacetylase 9(HDAC9)	Yes	22945647
1488	C-terminal binding protein 2(CTBP2)	Yes	22945647

2302	forkhead box J1(FOXJ1)	Yes	22945654
2829	X-C motif chemokine receptor 1(XCR1)	Yes	22964431
8739	harakiri, BCL2 interacting protein(HRK)	Yes	22964433
27123	dickkopf WNT signaling pathway inhibitor	Yes	22964660
10131	TNF receptor associated protein 1(TRAP1)	Yes	22978347
10321	cysteine rich secretory protein 3(CRISP3)	No	22993349
4477	microseminoprotein beta(MSMB)	Yes	22993349
22809	activating transcription factor 5(ATF5)	Yes	23018213
9557	chromodomain helicase DNA binding protein 1 like(CHD1L)	Yes	23020525
23414	zinc finger protein, FOG family member	Yes	23029311
151126	zinc finger protein 385B(ZNF385B)	Yes	23029477
6197	ribosomal protein S6 kinase A3(RPS6KA3)	Yes	23041051
7178	tumor protein, translationally-controlled 1(TPT1)	Yes	23042265
58	actin, alpha 1, skeletal muscle(ACTA1)	Yes	23042265
330	baculoviral IAP repeat containing 3(BIRC3)	Yes	23052480
7114	thymosin beta 4, X-linked(TMSB4X)	Yes	23055022
5764	pleiotrophin(PTN)	Yes	23056589
10403	NDC80, kinetochore complex	Yes	23056589
83540	NUF2, NDC80 kinetochore complex component(NUF2)	Yes	23056589
860	runt related transcription factor 2(RUNX2)	Yes	23093845
55742	parvin alpha(PARVA)	Yes	23099104
54751	filamin binding LIM protein 1(FBLIM1)	No	23099104
29780	parvin beta(PARVB)	Yes	23099104
6573	solute carrier family 19 member 1(SLC19A1)	Yes	23144806
3880	keratin 19(KRT19)	Yes	23171677
3916	lysosomal associated membrane protein	Yes	23172893
6464	SHC adaptor protein 1(SHC1)	Yes	23178716
6317	serpin family B member 3(SERPINB3)	Yes	23185467
55135	WD repeat containing antisense to	Yes	23192612
94234	forkhead box Q1(FOXQ1)	Yes	23203039

84464	SLX4 structure-specific endonuclease	No	23211700
1410	crystallin alpha B(CRYAB)	Yes	23225306
4324	matrix metallopeptidase 15(MMP15)	Yes	23228395
65986	zinc finger and BTB domain containing	Yes	23254909
51651	peptidyl-tRNA hydrolase 2(PTRH2)	Yes	23259782
6776	signal transducer and activator of transcription 5A(STAT5A)	Yes	23262295
407053	microRNA 96(MIR96)	No	23262295
6297	spalt like transcription factor 2(SALL2)	Yes	23273547
8092	ALX homeobox 1(ALX1)	Yes	23288509
968	CD68 molecule(CD68)	No	23289476
9332	CD163 molecule(CD163)	Yes	23289476
51029	desumoylating isopeptidase 2(DESI2)	No	23322088
51364	zinc finger MYND-type containing	No	23329649
5987	tripartite motif containing 27(TRIM27)	Yes	23342271
6920	transcription elongation factor A3(TCEA3)	Yes	23357533
7979	split hand/foot malformation (ectrodactyly) type 1(SHFM1)	Yes	23371468
10507	semaphorin 4D(SEMA4D)	Yes	23376478
1003	cadherin 5(CDH5)	Yes	23376478
56892	chromosome 8 open reading frame 4(C8orf4)	Yes	23377761
3654	interleukin 1 receptor associated kinase	Yes	23383610
6659	SRY-box 4(SOX4)	Yes	23389731
9370	adiponectin, C1Q and collagen domain containing(ADIPOQ)	Yes	23402904
8379	MAD1 mitotic arrest deficient like 1(MAD1L1)	Yes	23407047
574511	microRNA 506(MIR506)	No	23410973
389136	vestigial like family member 3(VGLL3)	Yes	23415753
3673	integrin subunit alpha 2(ITGA2)	Yes	23418787
3872	keratin 17(KRT17)	No	23430585
3218	homeobox B8(HOXB8)	No	23438671

4722	NADH:ubiquinone oxidoreductase core subunit S3(NDUFS3)	Yes	23446378
54207	potassium two pore domain channel subfamily K member 10(KCNK10)	Yes	23479219
3776	potassium two pore domain channel subfamily K member 2(KCNK2)	Yes	23479219
10935	peroxiredoxin 3(PRDX3)	Yes	23503975
2122	MDS1 and EVI1 complex locus(MECOM)	Yes	23517670
9076	claudin 1(CLDN1)	Yes	23523916
406947	microRNA 155(MIR155)	Yes	23523916
9947	MAGE family member C1(MAGEC1)	Yes	23529156
347051	solute carrier family 10 member 5(SLC10A5)	No	23535730
6871	transcriptional adaptor 2A(TADA2A)	Yes	23535730
5375	peripheral myelin protein 2(PMP2)	Yes	23535730
2167	fatty acid binding protein 4(FABP4)	Yes	23535730
2171	fatty acid binding protein 5(FABP5)	Yes	23535730
92421	charged multivesicular body protein	No	23535730
284099	chromosome 17 open reading frame	Yes	23535730
10529	nebulette(NEBL)	Yes	23535730
79752	zinc finger AN1-type containing 1(ZFAND1)	No	23535730
11056	DExD-box helicase 52(DDX52)	Yes	23535730
11072	dual specificity phosphatase 14(DUSP14)	Yes	23535730
31	acetyl-CoA carboxylase alpha(ACACA)	Yes	23535730
646486	fatty acid binding protein 12(FABP12)	No	23535730
64089	sorting nexin 16(SNX16)	Yes	23535730
440435	G protein-coupled receptor 179(GPR179)	No	23535730
387638	chromosome 10 open reading frame	Yes	23535730
387640	SKI/DACH domain containing 1(SKIDA1)	No	23535730
729873	TBC1 domain family member 3(TBC1D3)	No	23535730
8028	myeloid/lymphoid or mixed-lineage leukemia; translocated to, 10(MLLT10)	Yes	23535730
30837	suppressor of cytokine signaling 7(SOCS7)	No	23535730

84218	TBC1 domain family member 3F(TBC1D3F)	No	23535730
11276	synergin gamma(SYNRG)	No	23535730
3612	inositol monophosphatase 1(IMPA1)	No	23535730
64215	DnaJ heat shock protein family (Hsp40) member C1(DNAJC1)	Yes	23535730
84311	mitochondrial ribosomal protein L45(MRPL45)	Yes	23535730
57636	Rho GTPase activating protein 23(ARHGAP23)	No	23535730
1616	death domain associated protein(DAXX)	Yes	23539629
50861	stathmin 3(STMN3)	No	23543364
4905	N-ethylmaleimide sensitive factor, vesicle fusing ATPase(NSF)	Yes	23544013
9842	pleckstrin homology and RUN domain containing M1(PLEKHM1)	Yes	23544013
30010	neurexophilin 1(NXPH1)	Yes	23544013
8945	beta-transducin repeat containing E3 ubiquitin protein ligase(BTRC)	Yes	23546975
406942	microRNA 150(MIR150)	No	23554878
2735	GLI family zinc finger 1(GLI1)	Yes	23555905
51305	potassium two pore domain channel subfamily K member 9(KCNK9)	Yes	23564779
1318	solute carrier family 31 member 2(SLC31A2)	Yes	23564780
3963	galectin 7(LGALS7)	No	23564797
7468	Wolf-Hirschhorn syndrome candidate	Yes	23566000
3757	potassium voltage-gated channel subfamily H member 2(KCNH2)	Yes	23571109
177	advanced glycosylation end-product specific receptor(AGER)	Yes	23571222
23200	ATPase phospholipid transporting 11B (putative)(ATP11B)	No	23585472
8674	vesicle associated membrane protein 4(VAMP4)	Yes	23585472
10228	syntaxin 6(STX6)	Yes	23585472
406968	microRNA 193a(MIR193A)	No	23588298

9798	IST1, ESCRT-III associated factor(IST1)	Yes	23609236
3694	integrin subunit beta 6(ITGB6)	Yes	23615713
10367	mitochondrial calcium uptake 1(MICU1)	Yes	23615904
64210	MMS19 homolog, cytosolic iron-sulfur assembly component(MMS19)	Yes	23632208
56729	resistin(RETN)	Yes	23652833
10950	BTG anti-proliferation factor 3(BTG3)	Yes	23657964
3313	heat shock protein family A (Hsp70) member 9(HSPA9)	Yes	23665506
3236	homeobox D10(HOXD10)	Yes	23670532
406903	microRNA 10b(MIR10B)	Yes	23670532
7026	nuclear receptor subfamily 2 group F member 2(NR2F2)	Yes	23690307
494324	microRNA 375(MIR375)	No	23696927
619553	microRNA 484(MIR484)	No	23697367
693227	microRNA 642a(MIR642A)	No	23697367
7423	vascular endothelial growth factor B(VEGFB)	Yes	23697367
406999	microRNA 217(MIR217)	No	23697367
9948	WD repeat domain 1(WDR1)	Yes	23707566
558	AXL receptor tyrosine kinase(AXL)	Yes	23707658
8693	polypeptide N-acetylgalactosaminyltransferase 4(GALNT4)	Yes	23708057
79623	polypeptide N-acetylgalactosaminyltransferase 14(GALNT14)	Yes	23708057
599	BCL2 like 2(BCL2L2)	Yes	23708561
442904	microRNA 335(MIR335)	No	23708561
1058	centromere protein A(CENPA)	No	23712606
7094	talin 1(TLN1)	No	23722670
7011	telomerase associated protein 1(TEP1)	Yes	23739867
143	poly(ADP-ribose) polymerase family member 4(PARP4)	Yes	23739867
11197	WNT inhibitory factor 1(WIF1)	Yes	23746174

6434	transformer 2 beta homolog	Yes	23748175
6426	serine and arginine rich splicing factor 1(SRSF1)	Yes	23748175
5819	nectin cell adhesion molecule 2(NECTIN2)	Yes	23758976
25945	nectin cell adhesion molecule 3(NECTIN3)	Yes	23758976
4925	nucleobindin 2(NUCB2)	Yes	23764403
7545	Zic family member 1(ZIC1)	Yes	23774800
114335	chorionic gonadotropin beta subunit 1(CGB1)	No	23774837
114336	chorionic gonadotropin beta subunit 2(CGB2)	No	23774837
7169	tropomyosin 2 (beta)(TPM2)	Yes	23792823
7168	tropomyosin 1 (alpha)(TPM1)	Yes	23792823
7171	tropomyosin 4(TPM4)	Yes	23792823
7170	tropomyosin 3(TPM3)	Yes	23792823
1192	chloride intracellular channel 1(CLIC1)	Yes	23792823
10553	HIV-1 Tat interactive protein 2(HTATIP2)	No	23800048
6191	ribosomal protein S4, X-linked(RPS4X)	No	23800275
2324	fms related tyrosine kinase 4(FLT4)	Yes	23803010
693176	microRNA 591(MIR591)	No	23807165
406899	microRNA 106a(MIR106A)	No	23807165
5753	protein tyrosine kinase 6(PTK6)	Yes	23814047
6720	sterol regulatory element binding transcription factor 1(SREBF1)	Yes	23818099
10647	secretoglobin family 1D member 2(SCGB1D2)	Yes	23819652
83700	junctional adhesion molecule 3(JAM3)	Yes	23825230
7409	vav guanine nucleotide exchange factor	Yes	23856093
688	Kruppel like factor 5(KLF5)	Yes	23869764
10068	interleukin 18 binding protein(IL18BP)	Yes	23873689
57144	p21 (RAC1) activated kinase 5(PAK5)	Yes	23877225
84299	migration and invasion enhancer 1(MIEN1)	Yes	23880825
2828	G protein-coupled receptor 4(GPR4)	No	23888957
335	apolipoprotein A1(APOA1)	Yes	23902290
7056	thrombomodulin(THBD)	Yes	23918310
1301	collagen type XI alpha 1 chain(COL11A1)	Yes	23934190

10795	zinc finger protein 268(ZNF268)	Yes	23946776
5965	RecQ like helicase(RECQL)	Yes	23951333
83998	regenerating family member 4(REG4)	Yes	23958547
10424	progesterone receptor membrane component 2(PGRMC2)	Yes	23970345
10395	DLC1 Rho GTPase activating protein(DLC1)	Yes	23988121
1984	eukaryotic translation initiation factor	No	23991020
2036	erythrocyte membrane protein band 4.1 like 1(EPB41L1)	Yes	23994105
51514	denticleless E3 ubiquitin protein ligase	Yes	23995842
54894	ring finger protein 43(RNF43)	Yes	24001777
84868	hepatitis A virus cellular receptor 2(HAVCR2)	Yes	24007284
8817	fibroblast growth factor 18(FGF18)	Yes	24018557
10257	ATP binding cassette subfamily C member 4(ABCC4)	Yes	24024181
2054	syntaxin 2(STX2)	No	24039787
2768	G protein subunit alpha 12(GNA12)	Yes	24055910
4010	LIM homeobox transcription factor 1	Yes	24056967
694	BTG anti-proliferation factor 1(BTG1)	Yes	24084718
7425	VGF nerve growth factor inducible(VGF)	Yes	24086249
253260	RPTOR independent companion of MTOR complex 2(RICTOR)	Yes	24086535
8763	CD164 molecule(CD164)	Yes	24094005
7035	tissue factor pathway inhibitor(TFPI)	Yes	24094893
29893	PSMC3 interacting protein(PSMC3IP)	Yes	24097974
406928	microRNA 137(MIR137)	No	24144591
9582	apolipoprotein B mRNA editing enzyme catalytic subunit 3B(APOBEC3B)	Yes	24154874
6794	serine/threonine kinase 11(STK11)	Yes	24170201
8795	TNF receptor superfamily member	Yes	24190693
3682	integrin subunit alpha E(ITGAE)	Yes	24190978
9476	napsin A aspartic peptidase(NAPSA)	No	24191930

29117	bromodomain containing 7(BRD7)	Yes	24198243
388	ras homolog family member B(RHOB)	Yes	24223801
2202	EGF containing fibulin like extracellular matrix protein 1(EFEMP1)	Yes	24236050
875	cystathionine-beta-synthase(CBS)	Yes	24236104
6201	ribosomal protein S7(RPS7)	No	24244431
250	alkaline phosphatase, placental(ALPP)	Yes	24247025
9131	apoptosis inducing factor, mitochondria associated 1(AIFM1)	Yes	24247248
1642	damage specific DNA binding protein 1(DDB1)	No	24249678
8823	fibroblast growth factor 16(FGF16)	No	24253043
8341	histone cluster 1 H2B family member n(HIST1H2BN)	Yes	24256813
115201	autophagy related 4A cysteine	Yes	24256813
6935	zinc finger E-box binding homeobox 1(ZEB1)	Yes	24257549
148756	ovarian adenocarcinoma amplified long non-coding RNA(OVAAL)	No	24265805
1119	choline kinase alpha(CHKA)	Yes	24281000
3959	galectin 3 binding protein(LGALS3BP)	Yes	24302979
4172	minichromosome maintenance complex component 3(MCM3)	Yes	24324072
4583	mucin 2, oligomeric mucus/gel-forming(MUC2)	Yes	24324582
4751	NIMA related kinase 2(NEK2)	Yes	24337664
246778	interleukin 27(IL27)	Yes	24352695
41	acid sensing ion channel subunit 1(ASIC1)	Yes	24375028
23492	chromobox 7(CBX7)	No	24375438
23481	pescadillo ribosomal biogenesis factor 1(PES1)	No	24376209
134466	zinc finger protein 300 pseudogene	No	24393131
91975	zinc finger protein 300(ZNF300)	Yes	24393131
4092	SMAD family member 7(SMAD7)	Yes	24394555
406954	microRNA 181a-2(MIR181A2)	No	24394555
7316	ubiquitin C(UBC)	Yes	24403071

7321	ubiquitin conjugating enzyme E2 D1(UBE2D1)	Yes	24403071
7323	ubiquitin conjugating enzyme E2 D3(UBE2D3)	Yes	24403071
55611	OTU deubiquitinase, ubiquitin aldehyde binding 1(OTUB1)	No	24403071
50807	ArfGAP with SH3 domain, ankyrin repeat and PH domain 1(ASAP1)	Yes	24427349
2047	EPH receptor B1(EPHB1)	Yes	24427352
5062	p21 (RAC1) activated kinase 2(PAK2)	Yes	24434518
4739	neural precursor cell expressed, developmentally down-regulated 9(NEDD9)	Yes	24439227
1476	cystatin B(CSTB)	Yes	24452274
57448	baculoviral IAP repeat containing 6(BIRC6)	Yes	24453032
2261	fibroblast growth factor receptor 3(FGFR3)	Yes	24456664
407055	microRNA 99a(MIR99A)	No	24456664
64083	golgi phosphoprotein 3(GOLPH3)	Yes	24458516
407015	microRNA 26a-1(MIR26A1)	Yes	24466274
5426	DNA polymerase epsilon, catalytic	Yes	24472300
10399	receptor for activated C kinase 1(RACK1)	Yes	24481459
2591	polypeptide N-acetylgalactosaminyltransferase 3(GALNT3)	Yes	24504219
4869	nucleophosmin(NPM1)	Yes	24515769
102	ADAM metallopeptidase domain 10(ADAM10)	Yes	24518602
2185	protein tyrosine kinase 2 beta(PTK2B)	Yes	24523919
9020	mitogen-activated protein kinase kinase kinase 14(MAP3K14)	Yes	24533079
3691	integrin subunit beta 4(ITGB4)	Yes	24533083
5284	polymeric immunoglobulin receptor(PIGR)	Yes	24568264
1033	cyclin dependent kinase inhibitor 3(CDKN3)	Yes	24573179
100302242	microRNA 1236(MIR1236)	No	24573236
3240	haptoglobin(HP)	Yes	24576319
83706	fermitin family member 3(FERMT3)	Yes	24583125
81624	diaphanous related formin 3(DIAPH3)	Yes	24587343

10459	MAD2 mitotic arrest deficient-like 2 (yeast)(MAD2L2)	Yes	24597627
4250	secretoglobin family 2A member 2(SCGB2A2)	Yes	24603286
406989	microRNA 206(MIR206)	Yes	24604205
9770	Ras association domain family member	Yes	24606436
7481	Wnt family member 11(WNT11)	Yes	24606488
406925	microRNA 135a-1(MIR135A1)	No	24607788
407011	microRNA 23b(MIR23B)	No	24613919
642	bleomycin hydrolase(BLMH)	Yes	24615029
22974	TPX2, microtubule nucleation factor(TPX2)	Yes	24625450
23039	exportin 7(XPO7)	Yes	24625450
2908	nuclear receptor subfamily 3 group C member 1(NR3C1)	Yes	24629067
6236	RRAD, Ras related glycolysis inhibitor and calcium channel regulator(RRAD)	Yes	24648519
406978	microRNA 199b(MIR199B)	No	24659709
407024	microRNA 29b-1(MIR29B1)	Yes	24662327
100124700	HOX transcript antisense RNA(HOTAIR)	No	24662839
3001	granzyme A(GZMA)	Yes	24673566
115825	WD repeat and FYVE domain containing	No	24675677
407034	microRNA 30e(MIR30E)	Yes	24676806
407029	microRNA 30a(MIR30A)	Yes	24676806
406893	microRNA 101-1(MIR101-1)	No	24677166
494327	microRNA 378a(MIR378A)	No	24680769
574442	microRNA 489(MIR489)	No	24686007
2303	forkhead box C2(FOXC2)	Yes	24700112
10019	SH2B adaptor protein 3(SH2B3)	Yes	24704825
1785	dynamin 2(DNM2)	Yes	24706848
6528	solute carrier family 5 member 5(SLC5A5)	No	24708099
406992	microRNA 210(MIR210)	No	24715221
5321	phospholipase A2 group IVA(PLA2G4A)	Yes	24721622
3371	tenascin C(TNC)	Yes	24722824

26524	large tumor suppressor kinase 2(LATS2)	Yes	24735543
406955	microRNA 181b-1(MIR181B1)	Yes	24735543
3803	killer cell immunoglobulin like receptor, two Ig domains and long cytoplasmic tail 2(KIR2DL2)	Yes	24755350
125	alcohol dehydrogenase 1B (class I), beta polypeptide(ADH1B)	Yes	24756370
6423	secreted frizzled related protein 2(SFRP2)	Yes	24761891
6422	secreted frizzled related protein 1(SFRP1)	Yes	24761891
29028	ATPase family, AAA domain containing	No	24761900
5602	mitogen-activated protein kinase 10(MAPK10)	Yes	24767251
79065	autophagy related 9A(ATG9A)	Yes	24767251
25816	TNF alpha induced protein 8(TNFAIP8)	Yes	24767861
407054	microRNA 98(MIR98)	No	24771265
10542	late endosomal/lysosomal adaptor, MAPK and MTOR activator 5(LAMTOR5)	Yes	24788380
10082	glypican 6(GPC6)	Yes	24804215
81578	collagen type XXI alpha 1 chain(COL21A1)	Yes	24804215
4256	matrix Gla protein(MGP)	Yes	24804215
1303	collagen type XII alpha 1 chain(COL12A1)	Yes	24804215
1278	collagen type I alpha 2 chain(COL1A2)	Yes	24804215
3912	laminin subunit beta 1(LAMB1)	Yes	24804215
4060	lumican(LUM)	Yes	24804215
3908	laminin subunit alpha 2(LAMA2)	Yes	24804215
100313781	microRNA 718(MIR718)	No	24815691
57007	atypical chemokine receptor 3(ACKR3)	Yes	24819308
9844	engulfment and cell motility 1(ELMO1)	Yes	24819662
55	acid phosphatase, prostate(ACPP)	Yes	24829029
79648	microcephalin 1(MCPH1)	Yes	24830737
259266	abnormal spindle microtubule assembly(ASPM)	Yes	24830737
79893	gametogenetin binding protein 2(GGNBP2)	No	24842157
7253	thyroid stimulating hormone receptor(TSHR)	Yes	24844218
145258	goosecoid homeobox(GSC)	Yes	24858567

574456	microRNA 497(MIR497)	No	24858688
55384	maternally expressed 3 (non-protein	Yes	24859196
6605	SWI/SNF related, matrix associated, actin dependent regulator of chromatin, subfamily e, member 1(SMARCE1)	Yes	24880093
2237	flap structure-specific endonuclease 1(FEN1)	Yes	24880630
5027	purinergic receptor P2X 7(P2RX7)	Yes	24913779
2313	Fli-1 proto-oncogene, ETS transcription	Yes	24923303
54822	transient receptor potential cation channel subfamily M member 7(TRPM7)	Yes	24935580
57402	S100 calcium binding protein A14(S100A14)	Yes	24939856
54567	delta like canonical Notch ligand 4(DLL4)	No	24949865
8809	interleukin 18 receptor 1(IL18R1)	Yes	24963217
79858	NIMA related kinase 11(NEK11)	Yes	24969318
3659	interferon regulatory factor 1(IRF1)	Yes	24995581
84876	ORAI calcium release-activated calcium modulator 1(ORAI1)	No	25015419
6786	stromal interaction molecule 1(STIM1)	Yes	25015419
407009	microRNA 224(MIR224)	No	25017423
5580	protein kinase C delta(PRKCD)	Yes	25017423
3009	histone cluster 1 H1 family member	Yes	25023763
7704	zinc finger and BTB domain containing	Yes	25023763
3833	kinesin family member C1(KIFC1)	Yes	25028599
5983	replication factor C subunit 3(RFC3)	Yes	25030735
8324	frizzled class receptor 7(FZD7)	Yes	25032869
4317	matrix metallopeptidase 8(MMP8)	Yes	25034366
2332	fragile X mental retardation 1(FMR1)	Yes	25036526
4919	receptor tyrosine kinase like orphan receptor 1(ROR1)	Yes	25056203
7465	WEE1 G2 checkpoint kinase(WEE1)	Yes	25093290
3429	interferon alpha inducible protein 27(IFI27)	Yes	25103640
8452	cullin 3(CUL3)	Yes	25114896

9978	ring-box 1(RBX1)	No	25114896
10919	euchromatic histone lysine methyltransferase 2(EHMT2)	Yes	25115793
51377	ubiquitin C-terminal hydrolase L5(UCHL5)	Yes	25123264
6838	surfeit 6(SURF6)	No	25134534
159	adenylosuccinate synthase(ADSS)	Yes	25134534
1306	collagen type XV alpha 1 chain(COL15A1)	Yes	25134534
28	ABO blood group (transferase A, alpha 1-3-N-acetylgalactosaminyltransferase; transferase B, alpha 1-3-galactosyltransferase)(ABO)	Yes	25134534
54101	receptor interacting serine/threonine kinase	Yes	25134534
91074	ankyrin repeat domain 30A(ANKRD30A)	Yes	25134534
57522	SLIT-ROBO Rho GTPase activating protein 1(SRGAP1)	No	25134534
84557	microtubule associated protein 1 light chain 3 alpha(MAP1LC3A)	No	25136588
9314	Kruppel like factor 4(KLF4)	Yes	25137052
6897	threonyl-tRNA synthetase(TARS)	No	25163878
1236	C-C motif chemokine receptor 7(CCR7)	Yes	25168373
1284	collagen type IV alpha 2 chain(COL4A2)	Yes	25169943
406	aryl hydrocarbon receptor nuclear translocator like(ARNTL)	Yes	25175925
554213	microRNA 449a(MIR449A)	No	25179844
641	Bloom syndrome RecQ like helicase(BLM)	Yes	25182961
406929	microRNA 138-1(MIR138-1)	No	25190487
406930	microRNA 138-2(MIR138-2)	No	25190487
406906	microRNA 122(MIR122)	No	25195148
406994	microRNA 212(MIR212)	No	25201063
9462	RAS protein activator like 2(RASAL2)	Yes	25216515
687	Kruppel like factor 9(KLF9)	No	25216959
1763	DNA replication helicase/nuclease 2(DNA2)	Yes	25238049
79971	wntless Wnt ligand secretion mediator(WLS)	Yes	25258105

406894	microRNA 101-2(MIR101-2)	No	25260883
6293	VPS52, GARP complex subunit(VPS52)	Yes	25269472
53635	prostate tumor overexpressed 1(PTOV1)	No	25270739
7182	nuclear receptor subfamily 2 group C member 2(NR2C2)	Yes	25277189
7134	troponin C1, slow skeletal and cardiac	Yes	25277212
406884	microRNA let-7b(MIRLET7B)	Yes	25292198
26301	globoside alpha-1,3-N-acetylgalactosaminyltransferase 1(GBGT1)	Yes	25294702
574443	microRNA 490(MIR490)	No	25297343
83595	SRY-box 7(SOX7)	No	25297608
574444	microRNA 491(MIR491)	No	25299770
4893	neuroblastoma RAS viral oncogene	Yes	25316818
23411	sirtuin 1(SIRT1)	Yes	25323003
2249	fibroblast growth factor 4(FGF4)	Yes	25329002
54541	DNA damage inducible transcript 4(DDIT4)	Yes	25337238
2243	fibrinogen alpha chain(FGA)	Yes	25347092
399979	sorting nexin 19(SNX19)	Yes	25356737
9362	copine 6(CPNE6)	Yes	25356737
116028	RecQ mediated genome instability 2(RMI2)	Yes	25356737
124817	cyclin N-terminal domain containing 1(CNTD1)	No	25356737
84933	chromosome 8 open reading frame 76(C8orf76)	Yes	25356737
1397	cysteine rich protein 2(CRIP2)	No	25356737
10882	complement C1q like 1(C1QL1)	Yes	25356737
11060	WW domain containing E3 ubiquitin protein ligase 2(WWP2)	Yes	25356737
79446	WD repeat domain 25(WDR25)	Yes	25356737
124975	gamma-glutamyltransferase 6(GGT6)	No	25356737
7139	troponin T2, cardiac type(TNNT2)	Yes	25356737
744	metallophosphoesterase domain containing 2(MPPED2)	Yes	25356737
27173	solute carrier family 39 member 1(SLC39A1)	Yes	25356737

9532	BCL2 associated athanogene 2(BAG2)	No	25356737
9139	CBFA2/RUNX1 translocation partner	No	25356737
9517	serine palmitoyltransferase long chain base subunit 2(SPTLC2)	Yes	25356737
5979	ret proto-oncogene(RET)	Yes	25356737
91408	basic transcription factor 3 like 4(BTF3L4)	No	25356737
55113	XK related 8(XKR8)	No	25356737
10174	sorbin and SH3 domain containing 3(SORBS3)	Yes	25356737
3516	recombination signal binding protein for immunoglobulin kappa J region(RBPJ)	Yes	25356737
66000	transmembrane protein 108(TMEM108)	Yes	25356737
5478	peptidylprolyl isomerase A(PPIA)	Yes	25356737
977	CD151 molecule (Raph blood group)(CD151)	No	25356755
3675	integrin subunit alpha 3(ITGA3)	Yes	25356755
302	annexin A2(ANXA2)	Yes	25362534
23326	ubiquitin specific peptidase 22(USP22)	Yes	25369910
126393	heat shock protein family B (small) member 6(HSPB6)	Yes	25423708
6469	sonic hedgehog(SHH)	Yes	25432698
10059	dynamin 1 like(DNM1L)	Yes	25446129
442918	microRNA 373(MIR373)	Yes	25460499
57403	RAB22A, member RAS oncogene	Yes	25460499
2289	FK506 binding protein 5(FKBP5)	Yes	25460502
9025	ring finger protein 8(RNF8)	Yes	25483088
84148	lysine acetyltransferase 8(KAT8)	Yes	25483274
6390	succinate dehydrogenase complex iron sulfur subunit B(SDHB)	Yes	25491408
27086	forkhead box P1(FOXP1)	Yes	25500588
4543	melatonin receptor 1A(MTNR1A)	Yes	25514412
10253	sprouty RTK signaling antagonist 2(SPRY2)	Yes	25533808
664614	microRNA 545(MIR545)	No	25537514
10155	tripartite motif containing 28(TRIM28)	No	25548895

5754	protein tyrosine kinase 7 (inactive)(PTK7)	Yes	25550828
23600	alpha-methylacyl-CoA racemase(AMACR)	Yes	25551297
7525	YES proto-oncogene 1, Src family tyrosine kinase(YES1)	Yes	25561420
407028	microRNA 302a(MIR302A)	No	25562167
5908	RAP1B, member of RAS oncogene	Yes	25569036
100126333	microRNA 708(MIR708)	No	25569036
873	carbonyl reductase 1(CBR1)	Yes	25572536
4255	O-6-methylguanine-DNA	Yes	25579142
9656	mediator of DNA damage checkpoint 1(MDC1)	Yes	25592380
9154	solute carrier family 28 member 1(SLC28A1)	Yes	25600708
84817	thioredoxin domain containing 17(TXNDC17)	No	25607466
81631	microtubule associated protein 1 light chain 3 beta(MAP1LC3B)	No	25607473
2889	Rap guanine nucleotide exchange factor 1(RAPGEF1)	Yes	25617801
54386	TERF2 interacting protein(TERF2IP)	Yes	25617801
25949	SYF2 pre-mRNA splicing factor(SYF2)	No	25623116
81848	sprouty RTK signaling antagonist 4(SPRY4)	Yes	25630587
9271	piwi like RNA-mediated gene silencing	Yes	25630839
6363	C-C motif chemokine ligand 19(CCL19)	Yes	25636509
1855	dishevelled segment polarity protein 1(DVL1)	Yes	25643607
6367	C-C motif chemokine ligand 22(CCL22)	Yes	25647263
10487	adenylate cyclase associated protein 1(CAP1)	Yes	25652936
3903	leukocyte associated immunoglobulin like receptor 1(LAIR1)	No	25660999
10765	lysine demethylase 5B(KDM5B)	Yes	25663457
6690	serine peptidase inhibitor, Kazal type 1(SPINK1)	Yes	25667483
574034	microRNA 433(MIR433)	Yes	25684390
3133	major histocompatibility complex, class I,	Yes	25711417
8445	dual specificity tyrosine phosphorylation regulated kinase 2(DYRK2)	Yes	25712377

5069	pappalysin 1(PAPPA)	Yes	25720282
26353	heat shock protein family B (small) member 8(HSPB8)	Yes	25731856
5646	protease, serine 3(PRSS3)	Yes	25735255
1080	cystic fibrosis transmembrane conductance regulator(CFTR)	Yes	25738998
29128	ubiquitin like with PHD and ring finger domains 1(UHRF1)	Yes	25743796
9590	A-kinase anchoring protein 12(AKAP12)	Yes	25748058
2200	fibrillin 1(FBN1)	Yes	25749384
3939	lactate dehydrogenase A(LDHA)	Yes	25762632
442906	microRNA 338(MIR338)	No	25776272
377630	ubiquitin specific peptidase 17-like family member 2(USP17L2)	No	25776484
574455	microRNA 193b(MIR193B)	No	25798837
116844	leucine rich alpha-2-glycoprotein 1(LRG1)	No	25799488
51548	sirtuin 6(SIRT6)	No	25807436
10371	semaphorin 3A(SEMA3A)	Yes	25812535
7301	TYRO3 protein tyrosine kinase(TYRO3)	Yes	25815442
54487	DGCR8, microprocessor complex	Yes	25823356
57805	cell cycle and apoptosis regulator 2(CCAR2)	No	25823848
9365	klotho(KL)	Yes	25827069
4204	methyl-CpG binding protein 2(MECP2)	Yes	25827305
54790	tet methylcytosine dioxygenase 2(TET2)	Yes	25827305
56941	5-hydroxymethylcytosine (hmC) binding, ES cell-specific(HMCES)	No	25827305
5429	DNA polymerase eta(POLH)	Yes	25831546
9113	large tumor suppressor kinase 1(LATS1)	Yes	25841306
100048912	CDKN2B antisense RNA 1(CDKN2B-AS1)	No	25845387
7123	C-type lectin domain family 3 member	Yes	25846370
56998	catenin beta interacting protein 1(CTNNBIP1)	No	25849888
23095	kinesin family member 1B(KIF1B)	Yes	25854172

79778	MICAL like 2(MICALL2)	No	25864591
406962	microRNA 186(MIR186)	No	25867064
9475	Rho associated coiled-coil containing protein kinase 2(ROCK2)	Yes	25872785
55076	transmembrane protein 45A(TMEM45A)	No	25872785
30008	EGF containing fibulin like extracellular matrix protein 2(EFEMP2)	Yes	25885889
3490	insulin like growth factor binding protein	Yes	25886299
100302174	microRNA 1307(MIR1307)	No	25887170
154664	ATP binding cassette subfamily A member 13(ABCA13)	Yes	25889687
406993	microRNA 211(MIR211)	No	25889927
23476	bromodomain containing 4(BRD4)	No	25892415
693157	microRNA 572(MIR572)	No	25893382
406918	microRNA 129-2(MIR129-2)	No	25895125
406917	microRNA 129-1(MIR129-1)	No	25895125
25937	WW domain containing transcription regulator 1(WWTR1)	Yes	25895125
415116	Pim-3 proto-oncogene, serine/threonine	No	25921139
1748	distal-less homeobox 4(DLX4)	Yes	25924901
8462	Kruppel like factor 11(KLF11)	Yes	25931269
60529	ALX homeobox 4(ALX4)	Yes	25944620
7020	transcription factor AP-2 alpha(TFAP2A)	Yes	25953442
4969	osteoglycin(OGN)	Yes	25953442
64321	SRY-box 17(SOX17)	Yes	25953442
80854	SET domain containing lysine methyltransferase 7(SETD7)	Yes	25954928
406944	microRNA 153-1(MIR153-1)	No	25954928
23028	lysine demethylase 1A(KDM1A)	Yes	25956476
10297	APC2, WNT signaling pathway regulator(APC2)	Yes	25960217
100126351	microRNA 939(MIR939)	No	25960217
9290	G protein-coupled receptor 55(GPR55)	Yes	25989290

1901	sphingosine-1-phosphate receptor 1(S1PR1)	Yes	26004261
10971	tyrosine 3-monooxygenase/tryptophan 5-monooxygenase activation protein	Yes	26033570
91942	NADH:ubiquinone oxidoreductase complex assembly factor 2(NDUFAF2)	Yes	26033570
695	Bruton tyrosine kinase(BTK)	Yes	26036311
2746	glutamate dehydrogenase 1(GLUD1)	Yes	26045471
6566	solute carrier family 16 member 1(SLC16A1)	Yes	26045776
3953	leptin receptor(LEPR)	Yes	26053184
574460	microRNA 498(MIR498)	No	26054675
3235	homeobox D9(HOXD9)	Yes	26075790
5624	protein C, inactivator of coagulation factors Va and VIIIa(PROC)	Yes	26082331
50515	carbohydrate sulfotransferase 11(CHST11)	Yes	26084610
406973	microRNA 196a-2(MIR196A2)	Yes	26097603
406972	microRNA 196a-1(MIR196A1)	Yes	26097603
541466	cancer/testis antigen family 45 member	No	26098711
554212	microRNA 448(MIR448)	No	26103953
56957	OTU deubiquitinase 7B(OTUD7B)	No	26112491
23327	neural precursor cell expressed, developmentally down-regulated 4-like, E3 ubiquitin protein ligase(NEDD4L)	Yes	26130719
7189	TNF receptor associated factor 6(TRAF6)	Yes	26151128
100302213	microRNA 1181(MIR1181)	No	26151663
9891	NUAK family kinase 1(NUAK1)	Yes	26151663
55272	IMP3, U3 small nucleolar	No	26158423
55450	calcium/calmodulin dependent protein kinase II inhibitor 1(CAMK2N1)	No	26175272
8821	inositol polyphosphate-4-phosphatase type II B(INPP4B)	Yes	26189250
84519	acrosin binding protein(ACRBP)	No	26189257
3014	H2A histone family member X(H2AFX)	Yes	26191270

8661	eukaryotic translation initiation factor 3 subunit A(EIF3A)	Yes	26213845
25852	armadillo repeat containing 8(ARMC8)	No	26232863
5074	pro-apoptotic WT1 regulator(PAWR)	Yes	26246468
846	calcium sensing receptor(CASR)	Yes	26252839
3577	C-X-C motif chemokine receptor 1(CXCR1)	Yes	26267317
27124	inositol polyphosphate-5-phosphatase J(INPP5J)	No	26282217
724031	microRNA 661(MIR661)	No	26282217
1201	CLN3, battenin(CLN3)	Yes	26299671
7942	transcription factor EB(TFEB)	Yes	26307679
8089	YEATS domain containing 4(YEATS4)	Yes	26307679
1280	collagen type II alpha 1 chain(COL2A1)	Yes	26311224
386757	solute carrier family 6 member 10, pseudogene(SLC6A10P)	No	26311224
959	CD40 ligand(CD40LG)	Yes	26313915
8737	receptor interacting serine/threonine kinase	Yes	26313915
9788	MTSS1, I-BAR domain containing(MTSS1)	Yes	26316204
6009	Ras homolog enriched in brain(RHEB)	Yes	26319900
9965	fibroblast growth factor 19(FGF19)	Yes	26323668
5725	polypyrimidine tract binding protein 1(PTBP1)	Yes	26336992
100302175	microRNA 1207(MIR1207)	No	26337084
693215	microRNA 630(MIR630)	No	26345808
113510	helicase, POLQ-like(HELQ)	Yes	26351136
64805	purinergic receptor P2Y12(P2RY12)	Yes	26353776
6595	SWI/SNF related, matrix associated, actin dependent regulator of chromatin, subfamily a, member 2(SMARCA2)	Yes	26356327
406924	microRNA 134(MIR134)	No	26363097
5716	proteasome 26S subunit, non-ATPase	No	26364616
586	branched chain amino acid transaminase	Yes	26372729
3778	potassium calcium-activated channel subfamily M alpha 1(KCNMA1)	Yes	26386726

442890	microRNA 133b(MIR133B)	Yes	26396496
5228	placental growth factor(PGF)	Yes	26402225
100126335	microRNA 543(MIR543)	No	26402225
1281	collagen type III alpha 1 chain(COL3A1)	Yes	26406420
1282	collagen type IV alpha 1 chain(COL4A1)	Yes	26406420
79577	cell division cycle 73(CDC73)	Yes	26409451
221883	HOXA11 antisense RNA(HOXA11-AS)	No	26430965
90865	interleukin 33(IL33)	Yes	26433471
2316	filamin A(FLNA)	Yes	26446354
115908	collagen triple helix repeat containing	Yes	26452130
22846	vasohibin 1(VASH1)	No	26460696
404672	general transcription factor IIH subunit	Yes	26463438
4758	neuraminidase 1(NEU1)	Yes	26463994
374383	natural killer cell cytotoxicity receptor 3 ligand 1(NCR3LG1)	No	26464699
2051	EPH receptor B6(EPHB6)	No	26468391
30968	stomatin like 2(STOML2)	Yes	26487491
4772	nuclear factor of activated T-cells 1(NFATC1)	Yes	26501422
60674	growth arrest specific 5 (non-protein	No	26503132
3156	3-hydroxy-3-methylglutaryl-CoA	Yes	26503475
4920	receptor tyrosine kinase like orphan receptor 2(ROR2)	Yes	26515598
2730	glutamate-cysteine ligase modifier	Yes	26520442
6923	transcription elongation factor B subunit	No	26531153
5133	programmed cell death 1(PDCD1)	Yes	26541760
29843	SUMO1/sentrin specific peptidase 1(SENP1)	No	26548925
10788	IQ motif containing GTPase activating protein 2(IQGAP2)	Yes	26549344
59351	prostate and breast cancer overexpressed	Yes	26549570
442907	microRNA 339(MIR339)	No	26553360
604	B-cell CLL/lymphoma 6(BCL6)	Yes	26553360

643911	colorectal neoplasia differentially expressed (non-protein coding)(CRNDE)	No	26556866
4440	musashi RNA binding protein 1(MSI1)	No	26563371
100313892	microRNA 761(MIR761)	No	26563371
10666	CD226 molecule(CD226)	Yes	26563374
3689	integrin subunit beta 2(ITGB2)	Yes	26563750
494331	microRNA 382(MIR382)	No	26575700
64236	PDZ and LIM domain 2(PDLIM2)	No	26593252
406960	microRNA 184(MIR184)	No	26601424
488	ATPase sarcoplasmic/endoplasmic reticulum Ca2+ transporting 2(ATP2A2)	Yes	26607901
3068	hepatoma-derived growth factor(HDGF)	No	26612514
55165	centrosomal protein 55(CEP55)	Yes	26615423
200958	mucin 20, cell surface associated(MUC20)	Yes	26616226
285231	F-box and WD repeat domain containing 12(FBXW12)	No	26617728
2886	growth factor receptor bound protein 7(GRB7)	Yes	26617853
6195	ribosomal protein S6 kinase A1(RPS6KA1)	Yes	26625210
693198	microRNA 613(MIR613)	No	26631045
6695	SPARC/osteonectin, cwcv and kazal like domains proteoglycan 1(SPOCK1)	Yes	26638890
245711	speedy/RINGO cell cycle regulator family member A(SPDYA)	No	26644004
7220	transient receptor potential cation channel subfamily C member 1(TRPC1)	Yes	26647723
114904	C1q and tumor necrosis factor related protein 6(C1QTNF6)	Yes	26648301
634	carcinoembryonic antigen related cell adhesion molecule 1(CEACAM1)	Yes	26653024
406936	microRNA 144(MIR144)	No	26662316
406997	microRNA 215(MIR215)	No	26676658
5966	REL proto-oncogene, NF-kB subunit(REL)	Yes	26683819

4692	necdin, MAGE family member(NDN)	Yes	26689988
9452	integral membrane protein 2A(ITM2A)	Yes	26691219
574452	microRNA 494(MIR494)	No	26695144
1662	DEAD-box helicase 10(DDX10)	Yes	26713367
1374	carnitine palmitoyltransferase 1A(CPT1A)	Yes	26716645
23367	La ribonucleoprotein domain family member 1(LARP1)	No	26717985
23657	solute carrier family 7 member 11(SLC7A11)	Yes	26729415
27330	ribosomal protein S6 kinase A6(RPS6KA6)	Yes	26732474
693129	microRNA 548c(MIR548C)	No	26762267
55626	autophagy and beclin 1 regulator 1(AMBRA1)	No	26763392
7528	YY1 transcription factor(YY1)	Yes	26768613
5218	cyclin dependent kinase 14(CDK14)	Yes	26772918
343	aquaporin 8(AQP8)	Yes	26779650
363	aquaporin 6(AQP6)	No	26779650
57124	CD248 molecule(CD248)	Yes	26782745
406995	microRNA 181a-1(MIR181A1)	No	26782955
100379571	BACE1 antisense RNA(BACE1-AS)	No	26783004
6548	solute carrier family 9 member A1(SLC9A1)	Yes	26821117
10270	A-kinase anchoring protein 8(AKAP8)	No	26823747
6389	succinate dehydrogenase complex flavoprotein subunit A(SDHA)	Yes	26826064
219771	cyclin Y(CCNY)	Yes	26831658
100126316	microRNA 873(MIR873)	No	26850595
652995	urothelial cancer associated 1 (non-protein coding)(UCA1)	No	26851957
10562	olfactomedin 4(OLFM4)	Yes	26871282
619554	microRNA 486-1(MIR486-1)	No	26871282
79977	grainyhead like transcription factor 2(GRHL2)	Yes	26887977
1048	carcinoembryonic antigen related cell adhesion molecule 5(CEACAM5)	No	26892612
28978	transmembrane protein 14A(TMEM14A)	Yes	26896463

3856	keratin 8(KRT8)	Yes	26911253
9510	ADAM metallopeptidase with thrombospondin type 1 motif 1(ADAMTS1)	Yes	26916548
9507	ADAM metallopeptidase with thrombospondin type 1 motif 4(ADAMTS4)	Yes	26916548
990	cell division cycle 6(CDC6)	Yes	26920249
6281	S100 calcium binding protein A10(S100A10)	Yes	26925708
6663	SRY-box 10(SOX10)	Yes	26951260
56262	leucine rich repeat containing 8 family member A(LRRC8A)	Yes	26984736
4215	mitogen-activated protein kinase kinase kinase 3(MAP3K3)	Yes	26997451
79658	Rho GTPase activating protein 10(ARHGAP10)	Yes	27010858
317	apoptotic peptidase activating factor 1(APAF1)	Yes	27021436
81671	vacuole membrane protein 1(VMP1)	No	27023910
51474	LIM domain and actin binding 1(LIMA1)	Yes	27035883
79915	ATPase family, AAA domain containing	Yes	27045477
150094	salt inducible kinase 1(SIK1)	Yes	27081781
8864	period circadian clock 2(PER2)	Yes	27082164
8239	ubiquitin specific peptidase 9, X-linked(USP9X)	Yes	27120977
23328	SAM and SH3 domain containing 1(SASH1)	Yes	27123075
407033	microRNA 30d(MIR30D)	No	27141829
200424	tet methylcytosine dioxygenase 3(TET3)	Yes	27141829
64798	DEP domain containing MTOR-interacting protein(DEPTOR)	Yes	27211906
57521	regulatory associated protein of MTOR complex 1(RPTOR)	Yes	27211906
23410	sirtuin 3(SIRT3)	Yes	27216459
54843	synaptotagmin like 2(SYTL2)	Yes	27220283
6658	SRY-box 3(SOX3)	Yes	27251670
80198	MUS81 structure-specific endonuclease subunit(MUS81)	Yes	27255997

9358	integrin subunit beta like 1(ITGBL1)	Yes	27261588
29789	Obg like ATPase 1(OLA1)	Yes	27271530
10411	Rap guanine nucleotide exchange factor 3(RAPGEF3)	Yes	27277757
84525	HOP homeobox(HOPX)	No	27287041
92162	transmembrane protein 88(TMEM88)	No	27374141
3053	serpin family D member 1(SERPIND1)	Yes	27430660
6656	SRY-box 1(SOX1)	Yes	27452040
50619	DEF6, guanine nucleotide exchange	Yes	27488395
3627	C-X-C motif chemokine ligand 10(CXCL10)	Yes	27490802
84981	MIR22 host gene(MIR22HG)	No	27535740
407010	microRNA 23a(MIR23A)	No	27537390
54879	suppression of tumorigenicity 7 like(ST7L)	No	27537390
1147	conserved helix-loop-helix ubiquitous	Yes	27537390
252995	fibronectin type III domain containing	No	27545213
494332	microRNA 383(MIR383)	No	27567588
9529	BCL2 associated athanogene 5(BAG5)	Yes	27571744
406914	microRNA 127(MIR127)	No	27571744
2633	guanylate binding protein 1(GBP1)	Yes	27590579
124540	musashi RNA binding protein 2(MSI2)	Yes	27600258
55016	membrane associated ring-CH-type finger 1(MARCH1)	Yes	27633480
257397	TGF-beta activated kinase 1/MAP3K7 binding protein 3(TAB3)	Yes	27651027
406986	microRNA 203a(MIR203A)	No	27655286
100144748	killin, p53-regulated DNA replication	No	27663866
29065	ASAP1 intronic transcript 1(ASAP1-IT1)	No	27667152
23591	family with sequence similarity 215 member A (non-protein coding)(FAM215A)	No	27667152
79940	long intergenic non-protein coding RNA 472(LINC00472)	No	27667152
55000	taurine up-regulated 1 (non-protein	Yes	27693324

9730	DDB1 and CUL4 associated factor 1(DCAF1)	Yes	27721409
64112	modulator of apoptosis 1(MOAP1)	No	27721409
100126348	microRNA 760(MIR760)	No	27726922
693136	microRNA 551b(MIR551B)	No	27743201
11074	tripartite motif containing 31(TRIM31)	Yes	27743201
10928	ralA binding protein 1(RALBP1)	Yes	27748916
6495	SIX homeobox 1(SIX1)	No	27780136
56254	ring finger protein 20(RNF20)	No	27798111
9810	ring finger protein 40(RNF40)	No	27798111
9368	SLC9A3 regulator 1(SLC9A3R1)	Yes	27823775
8773	synaptosome associated protein 23(SNAP23)	Yes	27855700
6487	ST3 beta-galactoside alpha-2,3-sialyltransferase 3(ST3GAL3)	Yes	27871859
637	BH3 interacting domain death agonist(BID)	Yes	27878291
80023	neurensin 2(NRSN2)	Yes	27908706
101929335	ADAMTS9 antisense RNA 1(ADAMTS9-AS1)	No	27941916
8451	cullin 4A(CUL4A)	Yes	27983981
9051	proline-serine-threonine phosphatase interacting protein 1(PSTPIP1)	Yes	28039608
5873	RAB27A, member RAS oncogene	Yes	28043921
84289	inhibitor of growth family member 5(ING5)	Yes	28086946
377	ADP ribosylation factor 3(ARF3)	Yes	28098897
8862	apelin(APLN)	Yes	28111160
2049	EPH receptor B3(EPHB3)	Yes	28120491
728855	long intergenic non-protein coding RNA 623(LINC00623)	No	28128423
100642175	SPRY4 intronic transcript 1(SPRY4-IT1)	No	28129625
80312	tet methylcytosine dioxygenase 1(TET1)	Yes	28150354
2001	E74 like ETS transcription factor 5(ELF5)	Yes	28184931
9833	maternal embryonic leucine zipper	Yes	28214016
171024	synaptopodin 2(SYNPO2)	Yes	28231729
22933	sirtuin 2(SIRT2)	No	28259910

79674	ventricular zone expressed PH domain containing 1(VEPH1)	Yes	28301874
27074	lysosomal associated membrane protein	Yes	28349821
26168	SUMO1/sentrin/SMT3 specific peptidase	Yes	28351334
406887	microRNA let-7e(MIRLET7E)	No	28376831
2217	Fc fragment of IgG receptor and	Yes	28450240

Table S3 The 208 modules and their gene members

**Module ID**	**Module member gene**	**Gene description**
M_0	51686	ornithine decarboxylase antizyme 3(OAZ3)
M_0	5525	protein phosphatase 2 regulatory subunit
M_0	11311	vacuolar protein sorting 45 homolog(VPS45)
M_0	117145	thioesterase superfamily member 4(THEM4)
M_0	23557	SNAP associated protein(SNAPIN)
M_0	3068	hepatoma-derived growth factor(HDGF)
M_0	9191	death effector domain containing(DEDD)
M_0	51093	ribosomal RNA adenine dimethylase domain containing 1(RRNAD1)
M_0	51094	adiponectin receptor 1(ADIPOR1)
M_0	8674	vesicle associated membrane protein 4(VAMP4)
M_0	6944	vacuolar protein sorting 72 homolog(VPS72)
M_0	55154	misato 1, mitochondrial distribution and morphology regulator(MSTO1)
M_0	65123	integrator complex subunit 3(INTS3)
M_0	57459	GATA zinc finger domain containing 2B(GATAD2B)
M_0	11022	tudor and KH domain containing(TDRKH)
M_0	10262	splicing factor 3b subunit 4(SF3B4)
M_0	51107	aph-1 homolog A, gamma-secretase subunit(APH1A)
M_0	55870	ASH1 like histone lysine methyltransferase(ASH1L)
M_0	6391	succinate dehydrogenase complex subunit C(SDHC)
M_0	4179	CD46 molecule(CD46)
M_0	7371	uridine-cytidine kinase 2(UCK2)

M_0	54856	gon-4 like(GON4L)
M_0	9181	Rho/Rac guanine nucleotide exchange factor
M_0	80308	flavin adenine dinucleotide synthetase 1(FLAD1)
M_0	6746	signal sequence receptor subunit 2(SSR2)
M_0	65005	mitochondrial ribosomal protein L9(MRPL9)
M_0	2029	endosulfine alpha(ENSA)
M_0	405	aryl hydrocarbon receptor nuclear
M_0	5692	proteasome subunit beta 4(PSMB4)
M_0	57592	zinc finger protein 687(ZNF687)
M_0	6016	Ras like without CAAX 1(RIT1)
M_0	54953	chromosome 1 open reading frame 27(C1orf27)
M_0	4259	microsomal glutathione S-transferase 3(MGST3)
M_0	10092	actin related protein 2/3 complex subunit 5(ARPC5)
M_0	55204	golgi phosphoprotein 3 like(GOLPH3L)
M_0	6045	ring finger protein 2(RNF2)
M_0	5824	peroxisomal biogenesis factor 19(PEX19)
M_0	92344	golgin, RAB6 interacting(GORAB)
M_0	92342	methyltransferase like 18(METTL18)
M_0	261726	TOR signaling pathway regulator(TIPRL)
M_0	57147	SCY1 like pseudokinase 3(SCYL3)
M_0	51706	cytochrome b5 reductase 1(CYB5R1)
M_0	125150	zinc finger SWIM-type containing 7(ZSWIM7)
M_0	5202	prefoldin subunit 2(PFDN2)
M_0	5087	PBX homeobox 1(PBX1)
M_0	7818	death associated protein 3(DAP3)
M_0	8804	cellular repressor of E1A stimulated genes 1(CREG1)
M_0	29956	ceramide synthase 2(CERS2)
M_0	90780	pygopus family PHD finger 2(PYGO2)
M_0	7203	chaperonin containing TCP1 subunit 3(CCT3)
M_0	360023	zinc finger and BTB domain containing 41(ZBTB41)
M_0	58497	prune exopolyphosphatase(PRUNE1)
M_0	607	B-cell CLL/lymphoma 9(BCL9)

M_0	27173	solute carrier family 39 member 1(SLC39A1)
M_0	54964	chromosome 1 open reading frame 56(C1orf56)
M_0	100131187	thiosulfate sulfurtransferase like domain containing
M_0	6051	arginyl aminopeptidase(RNPEP)
M_0	9869	SET domain bifurcated 1(SETDB1)
M_0	6738	TROVE domain family member 2(TROVE2)
M_0	54823	SWT1, RNA endoribonuclease homolog(SWT1)
M_0	81627	tRNA methyltransferase 1 like(TRMT1L)
M_0	93183	phosphatidylinositol glycan anchor biosynthesis class M(PIGM)
M_0	9588	peroxiredoxin 6(PRDX6)
M_0	7391	upstream transcription factor 1(USF1)
M_0	140609	NIMA related kinase 7(NEK7)
M_0	63931	mitochondrial ribosomal protein S14(MRPS14)
M_0	51506	ubiquitin-fold modifier conjugating enzyme 1(UFC1)
M_0	81609	sorting nexin family member 27(SNX27)
M_0	9129	pre-mRNA processing factor 3(PRPF3)
M_0	79590	mitochondrial ribosomal protein L24(MRPL24)
M_0	10067	secretory carrier membrane protein 3(SCAMP3)
M_0	4758	neuraminidase 1(NEU1)
M_0	5636	phosphoribosyl pyrophosphate synthetase associated protein 2(PRPSAP2)
M_0	79577	cell division cycle 73(CDC73)
M_0	23381	SMG5, nonsense mediated mRNA decay
M_0	55974	solute carrier family 50 member 1(SLC50A1)
M_0	112770	glycosylated lysosomal membrane protein(GLMP)
M_0	8548	basic leucine zipper nuclear factor 1(BLZF1)
M_0	11266	dual specificity phosphatase 12(DUSP12)
M_0	23248	regulation of nuclear pre-mRNA domain containing
M_0	7170	tropomyosin 3(TPM3)
M_0	128240	NAD(P)HX epimerase(NAXE)
M_0	10899	jumping translocation breakpoint(JTB)

M_0	10456	HCLS1 associated protein X-1(HAX1)
M_0	148327	cAMP responsive element binding protein 3 like
M_0	266971	PIP5K1A and PSMD4-like, pseudogene(PIPSL)
M_0	57645	pogo transposable element with KRAB
M_0	10623	RNA polymerase III subunit C(POLR3C)
M_0	1945	ephrin A4(EFNA4)
M_0	1944	ephrin A3(EFNA3)
M_0	3608	interleukin enhancer binding factor 2(ILF2)
M_0	5279	phosphatidylinositol glycan anchor biosynthesis class
M_0	10712	family with sequence similarity 189 member
M_0	54460	mitochondrial ribosomal protein S21(MRPS21)
M_0	23623	RUN and SH3 domain containing 1(RUSC1)
M_0	64222	torsin family 3 member A(TOR3A)
M_0	51027	bolA family member 1(BOLA1)
M_0	79005	sodium channel modifier 1(SCNM1)
M_0	127829	ADP ribosylation factor like GTPase 8A(ARL8A)
M_0	388695	LysM domain containing 1(LYSMD1)
M_0	10440	translocase of inner mitochondrial membrane 17 homolog A (yeast)(TIMM17A)
M_0	1196	CDC like kinase 2(CLK2)
M_0	1197	CDC like kinase 2, pseudogene 1(CLK2P1)
M_0	51478	hydroxysteroid 17-beta dehydrogenase 7(HSD17B7)
M_0	25912	chromosome 1 open reading frame 43(C1orf43)
M_0	9898	ubiquitin associated protein 2 like(UBAP2L)
M_0	9673	solute carrier family 25 member 44(SLC25A44)
M_0	8703	beta-1,4-galactosyltransferase 3(B4GALT3)
M_0	54499	transmembrane and coiled-coil domains 1(TMCO1)
M_0	5710	proteasome 26S subunit, non-ATPase 4(PSMD4)
M_0	27005	ubiquitin specific peptidase 21(USP21)
M_0	27246	ring finger protein 115(RNF115)
M_0	126823	kelch domain containing 9(KLHDC9)
M_0	8533	COP9 signalosome subunit 3(COPS3)

M_0	5546	papillary renal cell carcinoma (translocation-associated)(PRCC)
M_0	84320	acyl-CoA binding domain containing 6(ACBD6)
M_0	80222	threonyl-tRNA synthetase 2, mitochondrial
M_0	28956	late endosomal/lysosomal adaptor, MAPK and MTOR activator 2(LAMTOR2)
M_0	56893	ubiquilin 4(UBQLN4)
M_0	11000	solute carrier family 27 member 3(SLC27A3)
M_0	51377	ubiquitin C-terminal hydrolase L5(UCHL5)
M_0	11243	polyamine modulated factor 1(PMF1)
M_0	5877	RAB interacting factor(RABIF)
M_0	653519	G protein-coupled receptor 89A(GPR89A)
M_0	2224	farnesyl diphosphate synthase(FDPS)
M_0	117143	transcriptional adaptor 1(TADA1)
M_0	1819	developmentally regulated GTP binding protein
M_0	200185	keratinocyte associated protein 2(KRTCAP2)
M_0	27101	calcyclin binding protein(CACYBP)
M_0	200186	CREB regulated transcription coactivator 2(CRTC2)
M_0	27252	kelch like family member 20(KLHL20)
M_0	8394	phosphatidylinositol-4-phosphate 5-kinase type 1 alpha(PIP5K1A)
M_0	91647	ATP synthase mitochondrial F1 complex assembly factor 2(ATPAF2)
M_0	83479	DEAD-box helicase 59(DDX59)
M_0	10654	phosphomevalonate kinase(PMVK)
M_0	26092	torsin 1A interacting protein 1(TOR1AIP1)
M_0	26097	chromatin target of PRMT1(CHTOP)
M_0	23126	pogo transposable element with ZNF domain(POGZ)
M_0	56882	CDC42 small effector 1(CDC42SE1)
M_0	5298	phosphatidylinositol 4-kinase beta(PI4KB)
M_0	54344	dolichyl-phosphate mannosyltransferase subunit
M_0	4817	nitrilase 1(NIT1)

M_0	25874	mitochondrial pyruvate carrier 2(MPC2)
M_0	116461	tRNA splicing endonuclease subunit 15(TSEN15)
M_0	55249	YY1 associated protein 1(YY1AP1)
M_0	64326	ring finger and WD repeat domain 2(RFWD2)
M_0	55585	ubiquitin conjugating enzyme E2 Q1(UBE2Q1)
M_0	8799	peroxisomal biogenesis factor 11 beta(PEX11B)
M_0	5565	protein kinase AMP-activated non-catalytic subunit beta 2(PRKAB2)
M_0	5498	protoporphyrinogen oxidase(PPOX)
M_0	9939	RNA binding motif protein 8A(RBM8A)
M_0	57657	hyperpolarization activated cyclic nucleotide gated potassium channel 3(HCN3)
M_0	81611	acidic nuclear phosphoprotein 32 family member
M_0	56957	OTU deubiquitinase 7B(OTUD7B)
M_0	10625	influenza virus NS1A binding protein(IVNS1ABP)
M_0	84265	RNA polymerase III subunit G like(POLR3GL)
M_0	4170	BCL2 family apoptosis regulator(MCL1)
M_0	51043	zinc finger and BTB domain containing 7B(ZBTB7B)
M_0	632	bone gamma-carboxyglutamate protein(BGLAP)
M_0	9722	nitric oxide synthase 1 adaptor protein(NOS1AP)
M_0	728932	#N/A
M_0	83759	RNA binding motif protein 4B(RBM4B)
M_0	4720	NADH:ubiquinone oxidoreductase core subunit
M_0	55157	aspartyl-tRNA synthetase 2, mitochondrial(DARS2)
M_0	9557	chromodomain helicase DNA binding protein 1
M_0	8834	transmembrane protein 11(TMEM11)
M_0	128077	limb and CNS expressed 1 like(LIX1L)
M_0	7286	tuftelin 1(TUFT1)
M_0	50717	DDB1 and CUL4 associated factor 8(DCAF8)
M_0	51603	methyltransferase like 13(METTL13)
M_0	9909	DENN domain containing 4B(DENND4B)

M_0	114814	gonadotropin releasing hormone receptor 2 (pseudogene)(GNRHR2)
M_0	126626	GA binding protein transcription factor beta subunit 2(GABPB2)
M_0	11159	RAB, member of RAS oncogene family-like
M_0	5993	regulatory factor X5(RFX5)
M_0	284615	ankyrin repeat domain 34A(ANKRD34A)
M_0	9659	phosphodiesterase 4D interacting protein(PDE4DIP)
M_0	29937	neudesin neurotrophic factor(NENF)
M_0	400793	chromosome 1 open reading frame 226(C1orf226)
M_0	9482	syntaxin 8(STX8)
M_0	81875	interferon stimulated exonuclease gene 20 like
M_0	93185	immunoglobulin superfamily member 8(IGSF8)
M_0	57549	immunoglobulin superfamily member 9(IGSF9)
M_0	582	Bardet-Biedl syndrome 1(BBS1)
M_0	284680	chromosome 1 open reading frame 111(C1orf111)
M_0	25979	dehydrogenase/reductase 7B(DHRS7B)
M_0	282566	long intergenic non-protein coding RNA
M_1	51691	LSM8 homolog, U6 small nuclear RNA
M_1	440699	leucine rich repeat containing 52(LRRC52)
M_1	23325	KIAA1033(KIAA1033)
M_1	79955	PDZ domain containing 7(PDZD7)
M_1	146754	dynein axonemal heavy chain 2(DNAH2)
M_1	933	CD22 molecule(CD22)
M_1	4659	protein phosphatase 1 regulatory subunit
M_1	100188954	DNMBP antisense RNA 1(DNMBP-AS1)
M_1	54346	unc-93 homolog A (C. elegans)(UNC93A)
M_1	100128675	HPN antisense RNA 1(HPN-AS1)
M_1	90488	transmembrane protein 263(TMEM263)
M_1	121441	neural precursor cell expressed, developmentally down-regulated 1(NEDD1)
M_1	6258	retinoid X receptor gamma(RXRG)

M_1	28977	mitochondrial ribosomal protein L42(MRPL42)
M_1	91298	chromosome 12 open reading frame 29(C12orf29)
M_1	26232	F-box protein 2(FBXO2)
M_1	2880	glutathione peroxidase 5(GPX5)
M_1	170067	SPT20 homolog, SAGA complex component-like 2(SUPT20HL2)
M_1	400794	uncharacterized LOC400794(LOC400794)
M_1	50804	myelin expression factor 2(MYEF2)
M_1	317	apoptotic peptidase activating factor 1(APAF1)
M_1	55591	vezatin, adherens junctions transmembrane
M_1	5224	phosphoglycerate mutase 2(PGAM2)
M_1	11279	Kruppel like factor 8(KLF8)
M_1	79962	DnaJ heat shock protein family (Hsp40) member C22(DNAJC22)
M_1	10761	placenta specific 1(PLAC1)
M_1	85416	Zic family member 5(ZIC5)
M_1	25859	prostate androgen-regulated transcript 1 (non-protein coding)(PART1)
M_1	440925	long intergenic non-protein coding RNA
M_1	5128	cyclin dependent kinase 17(CDK17)
M_1	338761	complement C1q like 4(C1QL4)
M_1	10083	USH1 protein network component harmonin(USH1C)
M_1	284276	long intergenic non-protein coding RNA
M_1	57469	paraneoplastic Ma antigen family like 2(PNMAL2)
M_1	55681	SCY1 like pseudokinase 2(SCYL2)
M_1	93611	F-box protein 44(FBXO44)
M_1	11174	ADAM metallopeptidase with thrombospondin type 1 motif 6(ADAMTS6)
M_1	400	ADP ribosylation factor like GTPase 1(ARL1)
M_1	83887	tubulin tyrosine ligase like 2(TTLL2)
M_1	400043	uncharacterized LOC400043(LOC400043)
M_1	283652	solute carrier family 24 member 5(SLC24A5)

M_1	23416	potassium voltage-gated channel subfamily H member 3(KCNH3)
M_1	728464	methyltransferase like 24(METTL24)
M_1	259215	lymphocyte antigen 6 complex, locus G6F(LY6G6F)
M_1	4099	myelin associated glycoprotein(MAG)
M_1	93099	dermokine(DMKN)
M_1	144983	heterogeneous nuclear ribonucleoprotein A1-like 2(HNRNPA1L2)
M_1	3178	heterogeneous nuclear ribonucleoprotein
M_1	84931	long intergenic non-protein coding RNA
M_1	1420	crystallin gamma C(CRYGC)
M_1	414332	lipocalin 10(LCN10)
M_1	157807	clavesin 1(CLVS1)
M_1	58530	lymphocyte antigen 6 complex, locus G6D(LY6G6D)
M_1	23268	dynamin binding protein(DNMBP)
M_1	23074	UHRF1 binding protein 1 like(UHRF1BP1L)
M_1	118432	ribosomal protein L29 pseudogene 2(RPL29P2)
M_1	552889	ataxin 7 like 3B(ATXN7L3B)
M_1	83987	coiled-coil domain containing 8(CCDC8)
M_1	440101	uncharacterized LOC440101(FLJ12825)
M_1	100131390	Sp9 transcription factor(SP9)
M_1	1080	cystic fibrosis transmembrane conductance
M_1	64431	ARP6 actin-related protein 6 homolog(ACTR6)
M_1	5250	solute carrier family 25 member 3(SLC25A3)
M_1	654429	leucine rich repeats and transmembrane domains
M_1	1419	crystallin gamma B(CRYGB)
M_1	50632	calcyon neuron specific vesicular protein(CALY)
M_1	160418	transmembrane and tetratricopeptide repeat containing 3(TMTC3)
M_1	7181	nuclear receptor subfamily 2 group C member
M_1	83992	cortactin binding protein 2(CTTNBP2)
M_1	118471	proline rich acidic protein 1(PRAP1)

M_1	54550	N-terminal EF-hand calcium binding protein
M_1	644943	Ras association domain family member 10(RASSF10)
M_1	81621	Kazal type serine peptidase inhibitor domain
M_1	255189	phospholipase A2 group IVF(PLA2G4F)
M_1	29080	coiled-coil domain containing 59(CCDC59)
M_1	11163	nudix hydrolase 4(NUDT4)
M_1	5173	prodynorphin(PDYN)
M_1	1188	chloride voltage-gated channel Kb(CLCNKB)
M_1	114795	transmembrane protein 132B(TMEM132B)
M_1	4673	nucleosome assembly protein 1 like 1(NAP1L1)
M_1	10970	cytoskeleton associated protein 4(CKAP4)
M_1	388533	keratinocyte differentiation associated
M_1	146167	solute carrier family 38 member 8(SLC38A8)
M_1	8431	nuclear receptor subfamily 0 group B member
M_1	340527	NHS like 2(NHSL2)
M_1	340526	retrotransposon gag domain containing 4(RGAG4)
M_1	80031	semaphorin 6D(SEMA6D)
M_1	8549	leucine rich repeat containing G protein-coupled receptor 5(LGR5)
M_1	9658	zinc finger protein 516(ZNF516)
M_1	3222	homeobox C5(HOXC5)
M_1	3221	homeobox C4(HOXC4)
M_1	5169	ectonucleotide pyrophosphatase/phosphodiesterase
M_1	7546	Zic family member 2(ZIC2)
M_1	10988	methionyl aminopeptidase 2(METAP2)
M_1	55532	solute carrier family 30 member 10(SLC30A10)
M_1	23014	F-box protein 21(FBXO21)
M_1	3963	galectin 7(LGALS7)
M_1	23475	quinolinate phosphoribosyltransferase(QPRT)
M_1	116437	long intergenic non-protein coding RNA
M_1	23105	follistatin like 4(FSTL4)
M_1	653499	galectin 7B(LGALS7B)

M_1	79136	lymphocyte antigen 6 complex, locus G6E(LY6G6E)
M_1	136853	scavenger receptor cysteine rich family member with 4 domains(SSC4D)
M_1	6540	solute carrier family 6 member 13(SLC6A13)
M_1	340596	lipoma HMGIC fusion partner-like 1(LHFPL1)
M_1	100240734	uncharacterized LOC100240734(LOC100240734)
M_1	100240735	uncharacterized LOC100240735(LOC100240735)
M_1	2703	gap junction protein alpha 8(GJA8)
M_1	8411	early endosome antigen 1(EEA1)
M_1	55228	paraneoplastic Ma antigen family like 1(PNMAL1)
M_1	4048	leukotriene A4 hydrolase(LTA4H)
M_1	29915	host cell factor C2(HCFC2)
M_1	50939	interphotoreceptor matrix proteoglycan 2(IMPG2)
M_1	9122	solute carrier family 16 member 4(SLC16A4)
M_1	51702	peptidyl arginine deiminase 3(PADI3)
M_1	613212	cortexin 3(CTXN3)
M_1	643236	transmembrane protein 72(TMEM72)
M_1	257202	glutathione peroxidase 6(GPX6)
M_1	389058	Sp5 transcription factor(SP5)
M_1	653275	cripto, FRL-1, cryptic family 1B(CFC1B)
M_1	2864	free fatty acid receptor 1(FFAR1)
M_1	6557	solute carrier family 12 member 1(SLC12A1)
M_1	2814	glycoprotein V platelet(GP5)
M_1	11103	KRR1, small subunit processome component
M_1	143686	sestrin 3(SESN3)
M_1	170572	5-hydroxytryptamine receptor 3C(HTR3C)
M_1	7334	ubiquitin conjugating enzyme E2 N(UBE2N)
M_1	114882	oxysterol binding protein like 8(OSBPL8)
M_1	7135	troponin I1, slow skeletal type(TNNI1)
M_1	5144	phosphodiesterase 4D(PDE4D)
M_1	79738	Bardet-Biedl syndrome 10(BBS10)
M_1	3249	hepsin(HPN)

M_1	129868	tripartite motif containing 43(TRIM43)
M_1	150527	uncharacterized LOC150527(TISP43)
M_1	54072	long intergenic non-protein coding RNA
M_1	54094	#N/A
M_1	83733	solute carrier family 25 member 18(SLC25A18)
M_1	9965	fibroblast growth factor 19(FGF19)
M_1	8048	cysteine and glycine rich protein 3(CSRP3)
M_1	2248	fibroblast growth factor 3(FGF3)
M_1	120863	DEP domain containing 4(DEPDC4)
M_1	7296	thioredoxin reductase 1(TXNRD1)
M_1	144717	family with sequence similarity 109 member
M_1	7044	left-right determination factor 2(LEFTY2)
M_1	83639	testis expressed 101(TEX101)
M_1	118461	chromosome 10 open reading frame 71(C10orf71)
M_1	145957	neuregulin 4(NRG4)
M_1	22987	synaptic vesicle glycoprotein 2C(SV2C)
M_1	2701	gap junction protein alpha 4(GJA4)
M_1	5015	orthodenticle homeobox 2(OTX2)
M_1	100170841	chromosome 17 open reading frame 96(C17orf96)
M_1	10242	potassium calcium-activated channel subfamily M regulatory beta subunit 2(KCNMB2)
M_1	2895	glutamate ionotropic receptor delta type subunit
M_1	4892	nebulin related anchoring protein(NRAP)
M_1	113451	antizyme inhibitor 2(AZIN2)
M_1	3850	keratin 3(KRT3)
M_1	84190	methyltransferase like 25(METTL25)
M_1	3547	immunoglobulin superfamily member 1(IGSF1)
M_1	344657	leucine rich repeats and IQ motif containing
M_1	27316	RNA binding motif protein, X-linked(RBMX)
M_1	64221	roundabout guidance receptor 3(ROBO3)
M_1	5074	pro-apoptotic WT1 regulator(PAWR)
M_1	414157	chromosome 10 open reading frame 62(C10orf62)

M_1	112817	4-hydroxy-2-oxoglutarate aldolase 1(HOGA1)
M_1	50940	phosphodiesterase 11A(PDE11A)
M_1	2249	fibroblast growth factor 4(FGF4)
M_1	129025	zinc finger protein 280A(ZNF280A)
M_1	129080	EMI domain containing 1(EMID1)
M_1	1583	cytochrome P450 family 11 subfamily A member 1(CYP11A1)
M_1	123591	transmembrane protein 266(TMEM266)
M_1	353189	solute carrier organic anion transporter family member 4C1(SLCO4C1)
M_1	284233	cytochrome P450 family 4 subfamily F member 35, pseudogene(CYP4F35P)
M_1	121457	IKBKB interacting protein(IKBIP)
M_1	121551	BTB domain containing 11(BTBD11)
M_1	2070	EYA transcriptional coactivator and phosphatase
M_1	6953	t-complex 10(TCP10)
M_1	126432	Ras and Rab interactor like(RINL)
M_1	401285	t-complex 10-like 2(TCP10L2)
M_1	6636	small nuclear ribonucleoprotein polypeptide
M_1	8738	CASP2 and RIPK1 domain containing adaptor with death domain(CRADD)
M_1	147111	NOTUM, palmitoleoyl-protein
M_1	348487	family with sequence similarity 131 member
M_1	80712	ESX homeobox 1(ESX1)
M_1	54831	bestrophin 2(BEST2)
M_1	51019	coiled-coil domain containing 53(CCDC53)
M_1	767	carbonic anhydrase 8(CA8)
M_1	79782	leucine rich repeat containing 31(LRRC31)
M_1	5630	peripherin(PRPH)
M_1	100190940	uncharacterized LOC100190940(LOC100190940)
M_1	79023	nucleoporin 37(NUP37)
M_1	84848	MIR503 host gene(MIR503HG)

M_1	105	adenosine deaminase, RNA specific B2
M_1	23434	long intergenic non-protein coding RNA
M_1	285419	long intergenic non-protein coding RNA
M_1	57497	leucine rich repeat and fibronectin type III domain containing 2(LRFN2)
M_1	3827	kininogen 1(KNG1)
M_1	10117	enamelin(ENAM)
M_1	2172	fatty acid binding protein 6(FABP6)
M_1	391267	ankyrin repeat domain 20 family member A11, pseudogene(ANKRD20A11P)
M_1	10788	IQ motif containing GTPase activating protein
M_1	94274	protein phosphatase 1 regulatory inhibitor subunit 14A(PPP1R14A)
M_1	220296	hepatic and glial cell adhesion molecule(HEPACAM)
M_1	80835	taste 1 receptor member 1(TAS1R1)
M_1	5897	recombination activating 2(RAG2)
M_1	158763	Rho GTPase activating protein 36(ARHGAP36)
M_1	283600	solute carrier family 25 member 47(SLC25A47)
M_1	1463	neurocan(NCAN)
M_1	126306	junctional sarcoplasmic reticulum protein 1(JSRP1)
M_1	6863	tachykinin precursor 1(TAC1)
M_2	51692	cleavage and polyadenylation specific factor
M_2	79954	nucleolar protein 10(NOL10)
M_2	55904	lysine methyltransferase 2E(KMT2E)
M_2	25851	tectonin beta-propeller repeat containing 1(TECPR1)
M_2	57665	retinol dehydrogenase 14 (all-trans/9-cis/11-
M_2	114049	Williams-Beuren syndrome chromosome region 22(WBSCR22)
M_2	154743	base methyltransferase of 25S rRNA 2
M_2	100128553	CTAGE family member 4(CTAGE4)
M_2	54927	coiled-coil-helix-coiled-coil-helix domain containing 3(CHCHD3)

M_2	63917	polypeptide N-acetylgalactosaminyltransferase
M_2	9031	bromodomain adjacent to zinc finger domain
M_2	9014	TATA-box binding protein associated factor, RNA polymerase I subunit B(TAF1B)
M_2	100132707	PAXIP1 antisense RNA 2(PAXIP1-AS2)
M_2	151507	male-specific lethal 3 homolog (Drosophila) pseudogene 1(MSL3P1)
M_2	10922	Fas activated serine/threonine kinase(FASTK)
M_2	55112	WD repeat domain 60(WDR60)
M_2	7260	tumor suppressing subtransferable candidate
M_2	2969	general transcription factor IIi(GTF2I)
M_2	27000	DnaJ heat shock protein family (Hsp40) member C2(DNAJC2)
M_2	4191	malate dehydrogenase 2(MDH2)
M_2	445328	Rho guanine nucleotide exchange factor
M_2	4289	muskelin 1(MKLN1)
M_2	54517	pseudouridylate synthase 7 (putative)(PUS7)
M_2	51667	negative regulator of ubiquitin like proteins 1(NUB1)
M_2	4189	DnaJ heat shock protein family (Hsp40) member B9(DNAJB9)
M_2	8295	transformation/transcription domain associated protein(TRRAP)
M_2	22973	laminin subunit beta 2 pseudogene 1(LAMB2P1)
M_2	168451	THAP domain containing 5(THAP5)
M_2	22976	PAX interacting protein 1(PAXIP1)
M_2	54892	non-SMC condensin II complex subunit
M_2	55607	protein phosphatase 1 regulatory subunit
M_2	54106	toll like receptor 9(TLR9)
M_2	285971	zinc finger protein 775(ZNF775)
M_2	7784	zona pellucida glycoprotein 3(ZP3)
M_2	23165	nucleoporin 205(NUP205)
M_2	9747	TRPM8 channel associated factor 1(TCAF1)

M_2	54480	chondroitin polymerizing factor 2(CHPF2)
M_2	83590	transmembrane and ubiquitin like domain containing 1(TMUB1)
M_2	441294	CTAGE family member 15(CTAGE15)
M_2	441295	olfactory receptor family 2 subfamily A member 9 pseudogene(OR2A9P)
M_2	57414	rhomboid domain containing 2(RHBDD2)
M_2	81554	RCC1 like(RCC1L)
M_2	830	capping actin protein of muscle Z-line alpha subunit 2(CAPZA2)
M_2	140545	ring finger protein 32(RNF32)
M_2	835	caspase 2(CASP2)
M_2	23608	makorin ring finger protein 1(MKRN1)
M_2	51657	serine/threonine/tyrosine interacting like 1(STYXL1)
M_2	257415	family with sequence similarity 133 member
M_2	5447	cytochrome p450 oxidoreductase(POR)
M_2	28996	homeodomain interacting protein kinase 2(HIPK2)
M_2	401409	RAB19, member RAS oncogene family(RAB19)
M_2	401427	olfactory receptor family 2 subfamily A member
M_2	401397	long intergenic non-protein coding RNA
M_2	100101267	POM121 transmembrane nucleoporin C(POM121C)
M_2	401431	ATP6V0E2 antisense RNA 1(ATP6V0E2-AS1)
M_2	51422	protein kinase AMP-activated non-catalytic subunit gamma 2(PRKAG2)
M_2	9690	ubiquitin protein ligase E3C(UBE3C)
M_2	57488	extended synaptotagmin 2(ESYT2)
M_2	6868	ADAM metallopeptidase domain 17(ADAM17)
M_2	8805	tripartite motif containing 24(TRIM24)
M_2	11194	ATP binding cassette subfamily B member 8(ABCB8)
M_2	22853	lemur tyrosine kinase 2(LMTK2)
M_2	26608	transducin beta like 2(TBL2)
M_2	643854	CTAGE family member 9(CTAGE9)

M_2	85453	TSPY like 5(TSPYL5)
M_2	254048	ubinuclein 2(UBN2)
M_2	10061	ATP binding cassette subfamily F member 2(ABCF2)
M_2	54627	microtubule associated protein 10(MAP10)
M_2	6733	SRSF protein kinase 2(SRPK2)
M_2	647087	chromosome 7 open reading frame 73(C7orf73)
M_2	55256	acireductone dioxygenase 1(ADI1)
M_2	55750	acylglycerol kinase(AGK)
M_2	29062	WD repeat domain 91(WDR91)
M_2	51631	LUC7 like 2, pre-mRNA splicing factor(LUC7L2)
M_2	3315	heat shock protein family B (small) member
M_2	113763	ZBED6 C-terminal like(ZBED6CL)
M_2	64434	nucleolar protein with MIF4G domain 1(NOM1)
M_2	29803	replication initiator 1(REPIN1)
M_2	8454	cullin 1(CUL1)
M_2	60412	exocyst complex component 4(EXOC4)
M_2	57189	KIAA1147(KIAA1147)
M_2	643641	zinc finger protein 862(ZNF862)
M_2	55798	methyltransferase like 2B(METTL2B)
M_2	50640	patatin like phospholipase domain containing
M_2	7458	eukaryotic translation initiation factor 4H(EIF4H)
M_2	1876	E2F transcription factor 6(E2F6)
M_2	7328	ubiquitin conjugating enzyme E2 H(UBE2H)
M_2	58508	lysine methyltransferase 2C(KMT2C)
M_2	6522	solute carrier family 4 member 2(SLC4A2)
M_2	23382	adenosylhomocysteinase like 2(AHCYL2)
M_2	340307	CTAGE family member 6(CTAGE6)
M_2	55973	B-cell receptor associated protein 29(BCAP29)
M_2	100134229	JHDM1D antisense RNA 1 (head to head)(JHDM1D-
M_2	4953	ornithine decarboxylase 1(ODC1)
M_2	65999	leucine rich repeat containing 61(LRRC61)
M_2	9601	protein disulfide isomerase family A member

M_2	84277	DnaJ heat shock protein family (Hsp40) member C30(DNAJC30)
M_2	402573	chromosome 7 open reading frame 61(C7orf61)
M_2	729375	family with sequence similarity 86, member A pseudogene(FAM86HP)
M_2	4850	CCR4-NOT transcription complex subunit 4(CNOT4)
M_2	57180	ARP3 actin related protein 3 homolog B(ACTR3B)
M_2	2146	enhancer of zeste 2 polycomb repressive complex 2 subunit(EZH2)
M_2	653857	ARP3 actin-related protein 3 homolog C(ACTR3C)
M_2	64418	transmembrane protein 168(TMEM168)
M_2	9883	POM121 transmembrane nucleoporin(POM121)
M_2	3092	huntingtin interacting protein 1(HIP1)
M_2	10971	tyrosine 3-monooxygenase/tryptophan 5-monooxygenase activation protein theta(YWHAQ)
M_2	2970	general transcription factor IIi pseudogene
M_2	79872	Cbl proto-oncogene like 1(CBLL1)
M_2	54556	inhibitor of growth family member 3(ING3)
M_2	52	acid phosphatase 1, soluble(ACP1)
M_2	11179	zinc finger protein 277(ZNF277)
M_2	79541	olfactory receptor family 2 subfamily A member
M_2	8427	zinc finger protein 282(ZNF282)
M_2	10130	protein disulfide isomerase family A member
M_2	79571	GRIP and coiled-coil domain containing 1(GCC1)
M_2	90956	aarF domain containing kinase 2(ADCK2)
M_2	129790	chromosome 7 open reading frame 13(C7orf13)
M_2	100134713	NDUFB2 antisense RNA 1(NDUFB2-AS1)
M_2	92092	zinc finger CCCH-type containing, antiviral 1 like(ZC3HAV1L)
M_2	80853	lysine demethylase 7A(KDM7A)
M_2	10466	component of oligomeric golgi complex 5(COG5)
M_2	23008	kelch domain containing 10(KLHDC10)

M_2	25913	protection of telomeres 1(POT1)
M_2	728743	zinc finger protein pseudogene(LOC728743)
M_2	1020	cyclin dependent kinase 5(CDK5)
M_2	6009	Ras homolog enriched in brain(RHEB)
M_2	84255	solute carrier family 37 member 3(SLC37A3)
M_2	767558	leucine zipper protein 6(LUZP6)
M_2	55131	RNA binding motif protein 28(RBM28)
M_2	10049	DnaJ heat shock protein family (Hsp40) member B6(DNAJB6)
M_2	23534	transportin 3(TNPO3)
M_2	27153	zinc finger protein 777(ZNF777)
M_2	168850	zinc finger protein 800(ZNF800)
M_2	7586	zinc finger with KRAB and SCAN domains
M_2	10142	A-kinase anchoring protein 9(AKAP9)
M_2	84626	KRAB-A domain containing 1(KRBA1)
M_2	9741	lysosomal protein transmembrane 4
M_2	78996	chromosome 7 open reading frame 49(C7orf49)
M_2	9275	BCL tumor suppressor 7B(BCL7B)
M_2	378884	NHL repeat containing E3 ubiquitin protein ligase
M_2	728640	family with sequence similarity 133, member A pseudogene(FAM133CP)
M_2	57541	zinc finger protein 398(ZNF398)
M_2	1653	DEAD-box helicase 1(DDX1)
M_2	116988	ArfGAP with GTPase domain, ankyrin repeat and PH domain 3(AGAP3)
M_2	7516	X-ray repair cross complementing 2(XRCC2)
M_2	55695	NOP2/Sun RNA methyltransferase family member 5(NSUN5)
M_2	83862	transmembrane protein 120A(TMEM120A)
M_2	246243	ribonuclease H1(RNASEH1)
M_2	26959	HMG-box transcription factor 1(HBP1)
M_2	136051	zinc finger protein 786(ZNF786)

M_2	8976	Wiskott-Aldrich syndrome like(WASL)
M_2	7988	zinc finger protein 212(ZNF212)
M_2	7982	suppression of tumorigenicity 7(ST7)
M_2	7984	Rho guanine nucleotide exchange factor
M_2	4698	NADH:ubiquinone oxidoreductase subunit
M_2	26053	autism susceptibility candidate 2(AUTS2)
M_2	64327	limb development membrane protein 1(LMBR1)
M_2	155435	RNA binding motif protein 33(RBM33)
M_2	673	B-Raf proto-oncogene, serine/threonine kinase(BRAF)
M_2	155066	ATPase H+ transporting V0 subunit e2(ATP6V0E2)
M_2	155061	zinc finger protein 746(ZNF746)
M_2	155054	zinc finger protein 425(ZNF425)
M_2	9270	integrin subunit beta 1 binding protein 1(ITGB1BP1)
M_2	10447	family with sequence similarity 3 member C(FAM3C)
M_2	100130742	leucine rich repeat containing 69(LRRC69)
M_2	5982	replication factor C subunit 2(RFC2)
M_2	811	calreticulin(CALR)
M_2	813	calumenin(CALU)
M_2	285148	isoamyl acetate-hydrolyzing esterase 1
M_2	113878	deltex E3 ubiquitin ligase 2(DTX2)
M_2	376940	zinc finger CCCH-type containing 6(ZC3H6)
M_2	10926	DBF4 zinc finger(DBF4)
M_2	339804	chromosome 2 open reading frame 74(C2orf74)
M_2	643414	lipase family member K(LIPK)
M_2	3984	LIM domain kinase 1(LIMK1)
M_2	5445	paraoxonase 2(PON2)
M_2	727896	cysteine and histidine rich domain containing 1 pseudogene(LOC727896)
M_2	80063	activating transcription factor 7 interacting protein
M_2	4176	minichromosome maintenance complex component
M_2	1039	cerebellar degeneration related protein 2(CDR2)
M_2	129787	transmembrane protein 18(TMEM18)

M_2	81844	tripartite motif containing 56(TRIM56)
M_2	26973	cysteine and histidine rich domain containing
M_2	346528	olfactory receptor family 2 subfamily A member
M_2	10089	potassium two pore domain channel subfamily K member 7(KCNK7)
M_2	374308	patched domain containing 3(PTCHD3)
M_3	51693	trafficking protein particle complex 2
M_3	113540	CKLF like MARVEL transmembrane domain containing 1(CMTM1)
M_3	29123	ankyrin repeat domain 11(ANKRD11)
M_3	55159	ring finger and WD repeat domain 3(RFWD3)
M_3	57610	RAN binding protein 10(RANBP10)
M_3	84342	component of oligomeric golgi complex 8(COG8)
M_3	6687	SPG7, paraplegin matrix AAA peptidase
M_3	54920	dihydrouridine synthase 2(DUS2)
M_3	27183	vacuolar protein sorting 4 homolog A(VPS4A)
M_3	54386	TERF2 interacting protein(TERF2IP)
M_3	2653	glycine cleavage system protein H(GCSH)
M_3	3735	lysyl-tRNA synthetase(KARS)
M_3	55783	cap methyltransferase 2(CMTR2)
M_3	1506	chymotrypsin like(CTRL)
M_3	80262	chromosome 16 open reading frame 70(C16orf70)
M_3	10328	ER membrane protein complex subunit 8(EMC8)
M_3	23644	enhancer of mRNA decapping 4(EDC4)
M_3	23450	splicing factor 3b subunit 3(SF3B3)
M_3	54849	differentially expressed in FDCP 8 homolog(DEF8)
M_3	16	alanyl-tRNA synthetase(AARS)
M_3	57707	TBC/LysM-associated domain containing 1(TLDC1)
M_3	23199	Gse1 coiled-coil protein(GSE1)
M_3	5681	protein serine kinase H1(PSKH1)
M_3	23563	carbohydrate sulfotransferase 5(CHST5)

M_3	172	AFG3 like matrix AAA peptidase subunit 1, pseudogene(AFG3L1P)
M_3	5119	charged multivesicular body protein 1A(CHMP1A)
M_3	10428	craniofacial development protein 1(CFDP1)
M_3	51388	NIP7, nucleolar pre-rRNA processing protein(NIP7)
M_3	123775	chromosome 16 open reading frame 46(C16orf46)
M_3	9057	solute carrier family 7 member 6(SLC7A6)
M_3	5432	RNA polymerase II subunit C(POLR2C)
M_3	7084	thymidine kinase 2, mitochondrial(TK2)
M_3	2734	golgi glycoprotein 1(GLG1)
M_3	64779	methenyltetrahydrofolate synthetase domain containing(MTHFSD)
M_3	3281	heat shock factor binding protein 1(HSBP1)
M_3	1874	E2F transcription factor 4(E2F4)
M_3	23300	ATM interactor(ATMIN)
M_3	54758	kelch domain containing 4(KLHDC4)
M_3	342371	ataxin 1 like(ATXN1L)
M_3	54957	thioredoxin like 4B(TXNL4B)
M_3	51647	family with sequence similarity 96 member
M_3	115992	ring finger protein 166(RNF166)
M_3	23035	PH domain and leucine rich repeat protein phosphatase 2(PHLPP2)
M_3	164	adaptor related protein complex 1 gamma 1
M_3	83693	hydroxysteroid dehydrogenase like 1(HSDL1)
M_3	23417	malonyl-CoA decarboxylase(MLYCD)
M_3	22980	transcription factor 25(TCF25)
M_3	55697	Vac14, PIKFYVE complex component(VAC14)
M_3	4597	mevalonate diphosphate decarboxylase(MVD)
M_3	10725	nuclear factor of activated T-cells 5(NFAT5)
M_3	65057	adrenocortical dysplasia homolog(ACD)
M_3	56942	C-X9-C motif containing 2(CMC2)
M_3	9114	ATPase H+ transporting V0 subunit d1(ATP6V0D1)

M_3	64146	peptide deformylase (mitochondrial)(PDF)
M_3	80152	centromere protein T(CENPT)
M_3	3038	hyaluronan synthase 3(HAS3)
M_3	79786	kelch like family member 36(KLHL36)
M_3	11060	WW domain containing E3 ubiquitin protein ligase
M_3	124491	transmembrane protein 170A(TMEM170A)
M_3	23174	zinc finger CCHC-type containing 14(ZCCHC14)
M_3	197320	zinc finger protein 778(ZNF778)
M_3	197258	fucokinase(FUK)
M_3	2622	growth arrest specific 8(GAS8)
M_3	84916	UTP4, small subunit processome component(UTP4)
M_3	7014	telomeric repeat binding factor 2(TERF2)
M_3	55794	DEAD-box helicase 28(DDX28)
M_3	84080	enkurin domain containing 1(ENKD1)
M_3	8558	cyclin dependent kinase 10(CDK10)
M_3	8883	NEDD8 activating enzyme E1 subunit 1(NAE1)
M_3	1783	dynein cytoplasmic 1 light intermediate chain
M_3	124044	spermatogenesis associated 2 like(SPATA2L)
M_3	124045	spermatogenesis associated 33(SPATA33)
M_3	118460	exosome component 6(EXOSC6)
M_3	9013	TATA-box binding protein associated factor, RNA polymerase I subunit C(TAF1C)
M_3	124245	zinc finger CCCH-type containing 18(ZC3H18)
M_3	197322	acyl-CoA synthetase family member 3(ACSF3)
M_3	161882	zinc finger protein, FOG family member 1(ZFPM1)
M_3	54971	BTG3 associated nuclear protein(BANP)
M_3	79583	transmembrane protein 231(TMEM231)
M_3	80011	family with sequence similarity 192 member
M_3	9798	IST1, ESCRT-III associated factor(IST1)
M_3	4775	nuclear factor of activated T-cells 3(NFATC3)
M_3	11269	DEAD-box helicase 19B(DDX19B)
M_3	353	adenine phosphoribosyltransferase(APRT)

M_3	57017	coenzyme Q9(COQ9)
M_3	2588	galactosamine (N-acetyl)-6-sulfatase(GALNS)
M_3	9605	VPS9 domain containing 1(VPS9D1)
M_3	22879	MON1 homolog B, secretory trafficking
M_3	25839	component of oligomeric golgi complex 4(COG4)
M_3	8720	membrane bound transcription factor peptidase, site 1(MBTPS1)
M_3	1723	dihydroorotate dehydrogenase (quinone)(DHODH)
M_3	55066	pyruvate dehydrogenase phosphatase regulatory subunit(PDPR)
M_3	5713	proteasome 26S subunit, non-ATPase 7(PSMD7)
M_3	348180	cytosolic thiouridylase subunit 2(CTU2)
M_3	11345	GABA type A receptor associated protein like 2(GABARAPL2)
M_3	81631	microtubule associated protein 1 light chain 3 beta(MAP1LC3B)
M_3	51741	WW domain containing oxidoreductase(WWOX)
M_3	84138	solute carrier family 7 member 6 opposite
M_3	79007	dysbindin domain containing 1(DBNDD1)
M_3	79791	F-box protein 31(FBXO31)
M_3	57546	pyruvate dehyrogenase phosphatase catalytic subunit
M_3	9100	ubiquitin specific peptidase 10(USP10)
M_3	29100	transmembrane protein 208(TMEM208)
M_3	10300	katanin regulatory subunit B1(KATNB1)
M_3	29105	cilia and flagella associated protein 20(CFAP20)
M_3	146198	ZFP90 zinc finger protein(ZFP90)
M_3	23536	adenosine deaminase, tRNA specific 1(ADAT1)
M_3	51192	chemokine like factor(CKLF)
M_3	10200	M-phase phosphoprotein 6(MPHOSPH6)
M_3	92806	CENPB DNA-binding domain containing
M_3	1327	cytochrome c oxidase subunit 4I1(COX4I1)
M_3	9785	DEAH-box helicase 38(DHX38)

M_3	92822	zinc finger protein 276(ZNF276)
M_3	283970	pyridoxal dependent decarboxylase domain containing 2, pseudogene(PDXDC2P)
M_3	10204	nuclear transport factor 2(NUTF2)
M_3	10664	CCCTC-binding factor(CTCF)
M_3	79726	WD repeat domain 59(WDR59)
M_3	388284	chromosome 16 open reading frame 86(C16orf86)
M_3	55565	zinc finger protein 821(ZNF821)
M_3	81577	glucose-fructose oxidoreductase domain containing
M_3	54496	protein arginine methyltransferase 7(PRMT7)
M_3	643246	microtubule associated protein 1 light chain 3 beta 2(MAP1LC3B2)
M_3	865	core-binding factor beta subunit(CBFB)
M_3	84937	zinc and ring finger 1(ZNRF1)
M_3	7567	zinc finger protein 19(ZNF19)
M_3	7571	zinc finger protein 23(ZNF23)
M_3	57215	THAP domain containing 11(THAP11)
M_3	162239	ZFP1 zinc finger protein(ZFP1)
M_3	2806	glutamic-oxaloacetic transaminase 2(GOT2)
M_3	54921	chromosome transmission fidelity factor 8(CHTF8)
M_3	57019	cytokine induced apoptosis inhibitor 1(CIAPIN1)
M_3	28987	NIN1/PSMD8 binding protein 1 homolog(NOB1)
M_3	100130015	urate (hydroxyiso-) hydrolase, pseudogene(URAHP)
M_3	283922	pyruvate dehydrogenase phosphatase regulatory subunit pseudogene(LOC283922)
M_3	100129637	#N/A
M_3	26231	leucine rich repeat containing 29(LRRC29)
M_3	55625	zinc finger DHHC-type containing 7(ZDHHC7)
M_3	80777	cytochrome b5 type B(CYB5B)
M_3	80790	c-Maf inducing protein(CMIP)
M_3	55308	DEAD-box helicase 19A(DDX19A)
M_3	283927	nudix hydrolase 7(NUDT7)

M_3	8139	gigaxonin(GAN)
M_3	79613	transport and golgi organization 6
M_3	23659	phospholipase A2 group XV(PLA2G15)
M_3	64785	GINS complex subunit 3(GINS3)
M_3	750	GAS8 antisense RNA 1(GAS8-AS1)
M_3	50855	par-6 family cell polarity regulator alpha(PARD6A)
M_4	51727	cytidine/uridine monophosphate kinase 1(CMPK1)
M_4	4343	Mov10 RISC complex RNA helicase(MOV10)
M_4	5236	phosphoglucomutase 1(PGM1)
M_4	84455	EF-hand calcium binding domain 7(EFCAB7)
M_4	83941	TM2 domain containing 1(TM2D1)
M_4	54741	leptin receptor overlapping transcript(LEPROT)
M_4	23507	leucine rich repeat containing 8 family member
M_4	10207	PATJ, crumbs cell polarity complex
M_4	54665	round spermatid basic protein 1(RSBN1)
M_4	10542	late endosomal/lysosomal adaptor, MAPK and MTOR activator 5(LAMTOR5)
M_4	10745	putative homeodomain transcription factor 1(PHTF1)
M_4	23032	ubiquitin specific peptidase 33(USP33)
M_4	51100	SH3 domain containing GRB2 like endophilin
M_4	127253	tRNA-yW synthesizing protein 3 homolog(TYW3)
M_4	55791	ligand dependent nuclear receptor interacting factor
M_4	34	acyl-CoA dehydrogenase, C-4 to C-12 straight chain(ACADM)
M_4	9653	heparan sulfate 2-O-sulfotransferase 1(HS2ST1)
M_4	4893	neuroblastoma RAS viral oncogene homolog(NRAS)
M_4	55144	leucine rich repeat containing 8 family member
M_4	4898	nardilysin convertase(NRDC)
M_4	80263	tripartite motif containing 45(TRIM45)
M_4	57708	MIER1 transcriptional regulator(MIER1)
M_4	55119	pre-mRNA processing factor 38B(PRPF38B)
M_4	23648	single stranded DNA binding protein 3(SSBP3)

M_4	11218	DEAD-box helicase 20(DDX20)
M_4	389	ras homolog family member C(RHOC)
M_4	494115	RNA binding motif protein, X-linked like
M_4	54680	zinc finger HIT-type containing 6(ZNHIT6)
M_4	79961	DENN domain containing 2D(DENND2D)
M_4	5586	protein kinase N2(PKN2)
M_4	91408	basic transcription factor 3 like 4(BTF3L4)
M_4	1629	dihydrolipoamide branched chain transacylase
M_4	84144	synapse defective Rho GTPase homolog 2(SYDE2)
M_4	51668	heat shock protein family B (small) member
M_4	10768	adenosylhomocysteinase like 1(AHCYL1)
M_4	284695	zinc finger protein 326(ZNF326)
M_4	2135	exostosin like glycosyltransferase 2(EXTL2)
M_4	178	amylo-alpha-1, 6-glucosidase, 4-alpha-glucanotransferase(AGL)
M_4	8202	nuclear receptor coactivator 3(NCOA3)
M_4	127428	transcription elongation factor A N-terminal and central domain containing 2(TCEANC2)
M_4	284697	BTB domain containing 8(BTBD8)
M_4	6884	TATA-box binding protein associated factor
M_4	2730	glutamate-cysteine ligase modifier subunit(GCLM)
M_4	1491	cystathionine gamma-lyase(CTH)
M_4	55225	ribonucleoprotein, PTB binding 2(RAVER2)
M_4	9372	zinc finger FYVE-type containing 9(ZFYVE9)
M_4	80135	ribosome production factor 1 homolog(RPF1)
M_4	7268	tetratricopeptide repeat domain 4(TTC4)
M_4	25974	methylmalonic aciduria (cobalamin deficiency) cblC type, with homocystinuria(MMACHC)
M_4	25973	prolyl-tRNA synthetase 2, mitochondrial
M_4	64783	RNA binding motif protein 15(RBM15)
M_4	8880	far upstream element binding protein 1(FUBP1)
M_4	80143	suppressor of IKBKE 1(SIKE1)

M_4	64645	major facilitator superfamily domain containing 14A(MFSD14A)
M_4	829	capping actin protein of muscle Z-line alpha subunit 1(CAPZA1)
M_4	85440	dedicator of cytokinesis 7(DOCK7)
M_4	606495	cytochrome b5 reductase like(CYB5RL)
M_4	5825	ATP binding cassette subfamily D member 3(ABCD3)
M_4	65260	cytochrome c oxidase assembly factor 7
M_4	9528	transmembrane protein 59(TMEM59)
M_4	23421	integrin subunit beta 3 binding protein(ITGB3BP)
M_4	54834	ganglioside induced differentiation associated protein 2(GDAP2)
M_4	55917	CTTNBP2 N-terminal like(CTTNBP2NL)
M_4	260425	membrane associated guanylate kinase, WW and PDZ domain containing 3(MAGI3)
M_4	79183	alpha tocopherol transfer protein like(TTPAL)
M_4	6780	staufen double-stranded RNA binding protein
M_4	4774	nuclear factor I A(NFIA)
M_4	23179	ral guanine nucleotide dissociation stimulator like
M_4	79989	tetratricopeptide repeat domain 26(TTC26)
M_4	10564	ADP ribosylation factor guanine nucleotide exchange factor 2(ARFGEF2)
M_4	5686	proteasome subunit alpha 5(PSMA5)
M_4	9860	leucine rich repeats and immunoglobulin like domains 2(LRIG2)
M_4	57489	outer dense fiber of sperm tails 2 like(ODF2L)
M_4	2773	G protein subunit alpha i3(GNAI3)
M_4	7813	ecotropic viral integration site 5(EVI5)
M_4	7812	cold shock domain containing E1(CSDE1)
M_4	117178	SSX family member 2 interacting protein(SSX2IP)
M_4	1429	crystallin zeta(CRYZ)
M_4	1434	chromosome segregation 1 like(CSE1L)

M_4	26009	zinc finger ZZ-type containing 3(ZZZ3)
M_4	515	ATP synthase, H+ transporting, mitochondrial Fo complex subunit B1(ATP5F1)
M_4	965	CD58 molecule(CD58)
M_4	374986	mitoguardin 1(MIGA1)
M_4	10286	breast carcinoma amplified sequence 2(BCAS2)
M_4	79699	zyg-11 family member B, cell cycle
M_4	22823	metal response element binding transcription factor
M_4	54432	Yip1 domain family member 1(YIPF1)
M_4	25950	RWD domain containing 3(RWDD3)
M_4	23169	solute carrier family 35 member D1(SLC35D1)
M_4	51060	thioredoxin domain containing 12(TXNDC12)
M_4	113802	HEN1 methyltransferase homolog 1(HENMT1)
M_4	6342	sterol carrier protein 2(SCP2)
M_4	2060	epidermal growth factor receptor pathway substrate 15(EPS15)
M_4	8458	transcription termination factor 2(TTF2)
M_4	55631	leucine rich repeat containing 40(LRRC40)
M_4	55661	DEAD-box helicase 27(DDX27)
M_4	23285	KIAA1107(KIAA1107)
M_4	7398	ubiquitin specific peptidase 1(USP1)
M_4	51249	transmembrane protein 69(TMEM69)
M_4	26135	SERPINE1 mRNA binding protein 1(SERBP1)
M_4	387338	NOP2/Sun RNA methyltransferase family member 4(NSUN4)
M_4	6301	seryl-tRNA synthetase(SARS)
M_4	204851	homeodomain interacting protein kinase 1(HIPK1)
M_4	3652	intracisternal A particle-promoted polypeptide(IPP)
M_4	8556	cell division cycle 14A(CDC14A)
M_4	22807	IKAROS family zinc finger 2(IKZF2)
M_4	81839	VANGL planar cell polarity protein 1(VANGL1)

M_4	4116	mago homolog, exon junction complex core component(MAGOH)
M_4	23155	chloride channel CLIC like 1(CLCC1)
M_4	55970	G protein subunit gamma 12(GNG12)
M_4	23443	solute carrier family 35 member A3(SLC35A3)
M_4	11146	glomulin, FKBP associated protein(GLMN)
M_4	10905	mannosidase alpha class 1A member 2(MAN1A2)
M_4	10390	choline/ethanolamine phosphotransferase 1(CEPT1)
M_4	1376	carnitine palmitoyltransferase 2(CPT2)
M_4	8915	B-cell CLL/lymphoma 10(BCL10)
M_4	9406	zinc finger RANBP2-type containing 2(ZRANB2)
M_4	9403	selenoprotein F(SELENOF)
M_4	51611	diphthamide biosynthesis 5(DPH5)
M_4	29957	solute carrier family 25 member 24(SLC25A24)
M_4	271	adenosine monophosphate deaminase 2(AMPD2)
M_4	148423	chromosome 1 open reading frame 52(C1orf52)
M_4	30836	deoxynucleotidyltransferase terminal interacting protein 2(DNTTIP2)
M_4	79871	RNA polymerase II associated protein 2(RPAP2)
M_4	10026	phosphatidylinositol glycan anchor biosynthesis class
M_4	199857	ALG14, UDP-N-acetylglucosaminyltransferase subunit(ALG14)
M_4	343099	coiled-coil domain containing 18(CCDC18)
M_4	6272	sortilin 1(SORT1)
M_4	2787	G protein subunit gamma 5(GNG5)
M_4	54987	chromosome 1 open reading frame 123(C1orf123)
M_4	10717	adaptor related protein complex 4 beta 1
M_4	84146	zinc finger protein 644(ZNF644)
M_4	9554	SEC22 homolog B, vesicle trafficking protein (gene/pseudogene)(SEC22B)
M_4	6814	syntaxin binding protein 3(STXBP3)
M_4	55329	meiosis specific nuclear structural 1(MNS1)

M_4	8634	RNA 3'-terminal phosphate cyclase(RTCA)
M_4	54874	formin binding protein 1 like(FNBP1L)
M_4	9295	serine and arginine rich splicing factor 11(SRSF11)
M_4	29929	ALG6, alpha-1,3-glucosyltransferase(ALG6)
M_4	3716	Janus kinase 1(JAK1)
M_4	64858	DNA cross-link repair 1B(DCLRE1B)
M_4	22911	WD repeat domain 47(WDR47)
M_4	84950	pre-mRNA processing factor 38A(PRPF38A)
M_4	60313	GC-rich promoter binding protein 1 like 1(GPBP1L1)
M_4	492303	gem nuclear organelle associated protein 8 pseudogene 4(GEMIN8P4)
M_4	115209	OMA1 zinc metallopeptidase(OMA1)
M_4	51592	tripartite motif containing 33(TRIM33)
M_4	163786	SAS-6 centriolar assembly protein(SASS6)
M_4	56267	kynurenine aminotransferase 3(KYAT3)
M_4	85369	striatin interacting protein 1(STRIP1)
M_4	50999	transmembrane p24 trafficking protein 5(TMED5)
M_4	23358	ubiquitin specific peptidase 24(USP24)
M_4	11124	Fas associated factor 1(FAF1)
M_4	26994	ring finger protein 11(RNF11)
M_4	58155	polypyrimidine tract binding protein 2(PTBP2)
M_4	10352	tryptophanyl tRNA synthetase 2,
M_4	1486	chitobiase(CTBS)
M_4	5906	RAP1A, member of RAS oncogene family(RAP1A)
M_4	64173	#N/A
M_4	56900	transmembrane protein 167B(TMEM167B)
M_4	10885	WD repeat domain 3(WDR3)
M_4	115353	leucine rich repeat containing 42(LRRC42)
M_4	51253	mitochondrial ribosomal protein L37(MRPL37)
M_4	5876	Rab geranylgeranyltransferase beta
M_4	127002	ataxin 7 like 2(ATXN7L2)
M_4	148867	solute carrier family 30 member 7(SLC30A7)

M_4	84938	autophagy related 4C cysteine peptidase(ATG4C)
M_4	54482	tRNA methyltransferase 13 homolog(TRMT13)
M_4	54558	spermatogenesis associated 6(SPATA6)
M_4	112970	KTI12 chromatin associated homolog(KTI12)
M_4	84852	ATP1A1 antisense RNA 1(ATP1A1-AS1)
M_4	1810	down-regulator of transcription 1(DR1)
M_4	128338	DNA damage regulated autophagy modulator
M_4	55706	NDC1 transmembrane nucleoporin(NDC1)
M_4	64756	ATP synthase mitochondrial F1 complex assembly factor 1(ATPAF1)
M_4	1952	cadherin EGF LAG seven-pass G-type receptor
M_4	63916	engulfment and cell motility 2(ELMO2)
M_4	23318	zinc finger CCHC-type containing 11(ZCCHC11)
M_4	54879	suppression of tumorigenicity 7 like(ST7L)
M_4	114883	oxysterol binding protein like 9(OSBPL9)
M_4	8790	fucose-1-phosphate guanylyltransferase(FPGT)
M_4	81573	ankyrin repeat domain 13C(ANKRD13C)
M_4	6789	serine/threonine kinase 4(STK4)
M_4	84100	ADP ribosylation factor like GTPase 6(ARL6)
M_4	374618	testis expressed 9(TEX9)
M_4	2104	estrogen related receptor gamma(ESRRG)
M_4	51361	hook microtubule tethering protein 1(HOOK1)
M_4	2959	general transcription factor IIB(GTF2B)
M_4	257	ALX homeobox 3(ALX3)
M_4	8543	LIM domain only 4(LMO4)
M_4	730101	uncharacterized LOC730101(LOC730101)
M_4	6566	solute carrier family 16 member 1(SLC16A1)
M_4	253152	epoxide hydrolase 4(EPHX4)
M_4	200014	coiled-coil and C2 domain containing 1B(CC2D1B)
M_4	164153	ubiquitin like 4B(UBL4B)
M_4	149469	#N/A
M_4	9813	EF-hand calcium binding domain 14(EFCAB14)

M_4	126661	coiled-coil domain containing 163(CCDC163)
M_4	4678	nuclear autoantigenic sperm protein(NASP)
M_4	440603	BCL2 like 15(BCL2L15)
M_4	114803	Myb like, SWIRM and MPN domains 1(MYSM1)
M_4	26027	acyl-CoA thioesterase 11(ACOT11)
M_4	64799	IQ motif containing H(IQCH)
M_4	23139	microtubule associated serine/threonine kinase
M_4	3074	hexosaminidase subunit beta(HEXB)
M_4	1266	calponin 3(CNN3)
M_4	148932	MOB kinase activator 3C(MOB3C)
M_4	11080	DnaJ heat shock protein family (Hsp40) member B4(DNAJB4)
M_4	8569	MAP kinase interacting serine/threonine kinase
M_4	246182	aldo-keto reductase family 7 member A2 pseudogene 1(AKR7A2P1)
M_5	53635	prostate tumor overexpressed 1(PTOV1)
M_5	83596	BCL2 like 12(BCL2L12)
M_5	84335	AKT1 substrate 1(AKT1S1)
M_5	7376	nuclear receptor subfamily 1 group H member
M_5	79760	gem nuclear organelle associated protein 7(GEMIN7)
M_5	59348	zinc finger protein 350(ZNF350)
M_5	9040	ubiquitin conjugating enzyme E2 M(UBE2M)
M_5	10856	RuvB like AAA ATPase 2(RUVBL2)
M_5	8775	NSF attachment protein alpha(NAPA)
M_5	730051	zinc finger protein 814(ZNF814)
M_5	399669	zinc finger protein 321, pseudogene(ZNF321P)
M_5	56848	sphingosine kinase 2(SPHK2)
M_5	91661	zinc finger protein 765(ZNF765)
M_5	140612	ZFP28 zinc finger protein(ZFP28)
M_5	79090	trafficking protein particle complex 6A(TRAPPC6A)
M_5	55786	zinc finger protein 415(ZNF415)
M_5	581	BCL2 associated X, apoptosis regulator(BAX)

M_5	80110	zinc finger protein 614(ZNF614)
M_5	3276	protein arginine methyltransferase 1(PRMT1)
M_5	342945	zinc finger and SCAN domain containing
M_5	587	branched chain amino acid transaminase 2(BCAT2)
M_5	2512	ferritin light chain(FTL)
M_5	3661	interferon regulatory factor 3(IRF3)
M_5	162966	zinc finger protein 600(ZNF600)
M_5	162967	zinc finger protein 320(ZNF320)
M_5	162968	zinc finger protein 497(ZNF497)
M_5	162972	zinc finger protein 550(ZNF550)
M_5	11284	polynucleotide kinase 3'-phosphatase(PNKP)
M_5	1628	D-box binding PAR bZIP transcription factor(DBP)
M_5	7694	zinc finger protein 135(ZNF135)
M_5	284309	zinc finger protein 776(ZNF776)
M_5	284307	zinc finger protein interacting with K protein 1(ZIK1)
M_5	65982	zinc finger and SCAN domain containing
M_5	79673	zinc finger protein 329(ZNF329)
M_5	125893	zinc finger protein 816(ZNF816)
M_5	51545	zinc finger protein 581(ZNF581)
M_5	284306	zinc finger protein 547(ZNF547)
M_5	79818	zinc finger protein 552(ZNF552)
M_5	89887	zinc finger protein 628(ZNF628)
M_5	7728	zinc finger protein 175(ZNF175)
M_5	126119	Josephin domain containing 2(JOSD2)
M_5	3770	potassium voltage-gated channel subfamily J member 14(KCNJ14)
M_5	125919	zinc finger protein 543(ZNF543)
M_5	11133	kaptin, actin binding protein(KPTN)
M_5	593	branched chain keto acid dehydrogenase E1, alpha polypeptide(BCKDHA)
M_5	374928	zinc finger protein 773(ZNF773)
M_5	22870	protein phosphatase 6 regulatory subunit 1(PPP6R1)

M_5	79763	isochorismatase domain containing 2(ISOC2)
M_5	84671	zinc finger protein 347(ZNF347)
M_5	284312	zinc finger and SCAN domain containing 1(ZSCAN1)
M_5	388567	zinc finger protein 749(ZNF749)
M_5	54958	transmembrane protein 160(TMEM160)
M_5	388558	zinc finger protein 808(ZNF808)
M_5	388561	zinc finger protein 761(ZNF761)
M_5	6223	ribosomal protein S19(RPS19)
M_5	51070	nitric oxide synthase interacting protein(NOSIP)
M_5	100131691	MZF1 antisense RNA 1(MZF1-AS1)
M_5	160	adaptor related protein complex 2 alpha 1
M_5	6158	ribosomal protein L28(RPL28)
M_5	606551	ubiquitin conjugating enzyme E2 M pseudogene
M_5	84798	chromosome 19 open reading frame 48(C19orf48)
M_5	10155	tripartite motif containing 28(TRIM28)
M_5	84922	FLT3 interacting zinc finger 1(FIZ1)
M_5	10567	Rab acceptor 1(RABAC1)
M_5	126208	zinc finger protein 787(ZNF787)
M_5	84787	lysine methyltransferase 5C(KMT5C)
M_5	57479	proline rich 12(PRR12)
M_5	90485	zinc finger protein 835(ZNF835)
M_5	51231	vaccinia related kinase 3(VRK3)
M_5	26121	pre-mRNA processing factor 31(PRPF31)
M_5	147687	zinc finger protein 417(ZNF417)
M_5	64130	lin-7 homolog B, crumbs cell polarity complex component(LIN7B)
M_5	10945	KDEL endoplasmic reticulum protein retention receptor 1(KDELR1)
M_5	90321	zinc finger protein 766(ZNF766)
M_5	90317	zinc finger protein 616(ZNF616)
M_5	10055	SUMO1 activating enzyme subunit 1(SAE1)
M_5	6205	ribosomal protein S11(RPS11)

M_5	147694	zinc finger protein 548(ZNF548)
M_5	6203	ribosomal protein S9(RPS9)
M_5	51157	zinc finger protein 580(ZNF580)
M_5	126017	zinc finger protein 813(ZNF813)
M_5	80095	zinc finger protein 606(ZNF606)
M_5	29998	glioma tumor suppressor candidate region gene
M_5	29997	glioma tumor suppressor candidate region gene
M_5	6415	selenoprotein W(SELENOW)
M_5	84436	zinc finger protein 528(ZNF528)
M_5	1175	adaptor related protein complex 2 sigma 1
M_5	10597	trafficking protein particle complex 2B(TRAPPC2B)
M_5	29844	TCF3 fusion partner(TFPT)
M_5	55659	zinc finger protein 416(ZNF416)
M_5	2997	glycogen synthase 1(GYS1)
M_5	7696	zinc finger protein 137, pseudogene(ZNF137P)
M_5	81857	mediator complex subunit 25(MED25)
M_5	81856	zinc finger protein 611(ZNF611)
M_5	84914	zinc finger protein 587(ZNF587)
M_5	7691	zinc finger protein 132(ZNF132)
M_5	201514	zinc finger protein 584(ZNF584)
M_5	7693	zinc finger protein 134(ZNF134)
M_5	58491	zinc finger protein 71(ZNF71)
M_5	63934	zinc finger protein 667(ZNF667)
M_5	29903	coiled-coil domain containing 106(CCDC106)
M_5	55762	zinc finger protein 701(ZNF701)
M_5	6193	ribosomal protein S5(RPS5)
M_5	54814	glutaminyl-peptide cyclotransferase like(QPCTL)
M_5	147686	zinc finger protein 418(ZNF418)
M_5	79149	zinc finger and SCAN domain containing
M_5	90233	zinc finger protein 551(ZNF551)
M_5	55663	zinc finger protein 446(ZNF446)
M_5	808	calmodulin 3(CALM3)

M_5	9266	cytohesin 2(CYTH2)
M_5	58506	SR-related CTD associated factor 1(SCAF1)
M_5	147660	zinc finger protein 578(ZNF578)
M_5	91664	zinc finger protein 845(ZNF845)
M_5	255043	transmembrane protein 86B(TMEM86B)
M_5	388566	zinc finger protein 470(ZNF470)
M_5	65996	CENPB DNA-binding domains containing 1 pseudogene 1(CENPBD1P1)
M_5	6633	small nuclear ribonucleoprotein D2
M_5	116412	zinc finger protein 837(ZNF837)
M_5	4849	CCR4-NOT transcription complex subunit 3(CNOT3)
M_5	79165	leukocyte receptor cluster member 1(LENG1)
M_5	147657	zinc finger protein 480(ZNF480)
M_5	80199	fuzzy planar cell polarity protein(FUZ)
M_5	10794	zinc finger protein 460(ZNF460)
M_5	29888	striatin 4(STRN4)
M_5	56915	exosome component 5(EXOSC5)
M_5	55011	PIH1 domain containing 1(PIH1D1)
M_5	57343	zinc finger protein 304(ZNF304)
M_5	339344	Myb related transcription factor, partner of
M_5	342926	zinc finger protein 677(ZNF677)
M_5	57573	zinc finger protein 471(ZNF471)
M_5	54776	protein phosphatase 1 regulatory subunit
M_5	9704	DExH-box helicase 34(DHX34)
M_5	170958	zinc finger protein 525(ZNF525)
M_5	5536	protein phosphatase 5 catalytic subunit(PPP5C)
M_5	348327	zinc finger protein 530(ZNF530)
M_5	9422	zinc finger protein 264(ZNF264)
M_5	57106	N-acetyltransferase 14 (putative)(NAT14)
M_5	83743	glutamate rich WD repeat containing 1(GRWD1)
M_5	10782	zinc finger protein 274(ZNF274)
M_5	3978	DNA ligase 1(LIG1)

M_5	2067	ERCC excision repair 1, endonuclease non-catalytic subunit(ERCC1)
M_5	4924	nucleobindin 1(NUCB1)
M_5	256051	zinc finger protein 549(ZNF549)
M_5	25799	zinc finger protein 324(ZNF324)
M_5	162963	zinc finger protein 610(ZNF610)
M_5	9668	zinc finger protein 432(ZNF432)
M_5	255783	InaF motif containing 1(INAFM1)
M_5	84878	zinc finger and BTB domain containing 45(ZBTB45)
M_5	5424	DNA polymerase delta 1, catalytic subunit(POLD1)
M_5	162962	zinc finger protein 836(ZNF836)
M_5	7593	myeloid zinc finger 1(MZF1)
M_5	90353	cytosolic thiouridylase subunit 1(CTU1)
M_5	388569	zinc finger protein 324B(ZNF324B)
M_5	90338	zinc finger protein 160(ZNF160)
M_5	27243	charged multivesicular body protein 2A(CHMP2A)
M_5	90333	zinc finger protein 468(ZNF468)
M_5	11338	U2 small nuclear RNA auxiliary factor 2(U2AF2)
M_5	84765	zinc finger protein 577(ZNF577)
M_5	4696	NADH:ubiquinone oxidoreductase subunit
M_5	7565	zinc finger protein 17(ZNF17)
M_5	79735	TBC1 domain family member 17(TBC1D17)
M_5	10998	solute carrier family 27 member 5(SLC27A5)
M_5	7554	zinc finger protein 8(ZNF8)
M_5	400720	zinc finger protein 772(ZNF772)
M_5	10520	zinc finger protein 211(ZNF211)
M_5	147949	zinc finger protein 583(ZNF583)
M_5	147948	zinc finger protein 582(ZNF582)
M_5	147947	zinc finger protein 542, pseudogene(ZNF542P)
M_5	163033	zinc finger protein 579(ZNF579)
M_5	29924	epsin 1(EPN1)
M_5	390980	zinc finger protein 805(ZNF805)

M_5	2109	electron transfer flavoprotein beta subunit(ETFB)
M_5	25865	protein kinase D2(PRKD2)
M_5	79898	zinc finger protein 613(ZNF613)
M_5	79042	tRNA splicing endonuclease subunit 34(TSEN34)
M_5	23521	ribosomal protein L13a(RPL13A)
M_5	10172	zinc finger protein 256(ZNF256)
M_5	79143	membrane bound O-acyltransferase domain containing 7(MBOAT7)
M_5	79147	fukutin related protein(FKRP)
M_5	284371	zinc finger protein 841(ZNF841)
M_5	7576	zinc finger protein 28(ZNF28)
M_5	27300	zinc finger protein 544(ZNF544)
M_5	284370	zinc finger protein 615(ZNF615)
M_5	26093	coiled-coil domain containing 9(CCDC9)
M_5	57191	vomeronasal 1 receptor 1(VN1R1)
M_5	5518	protein phosphatase 2 scaffold subunit
M_5	2909	Rho GTPase activating protein 35(ARHGAP35)
M_5	65251	zinc finger protein 649(ZNF649)
M_5	6141	ribosomal protein L18(RPL18)
M_5	79744	zinc finger protein 419(ZNF419)
M_5	147804	tropomyosin 3 pseudogene 9(TPM3P9)
M_5	147807	zinc finger protein 524(ZNF524)
M_5	147808	zinc finger protein 784(ZNF784)
M_5	400713	zinc finger protein 880(ZNF880)
M_5	23636	nucleoporin 62(NUP62)
M_5	25888	zinc finger protein 473(ZNF473)
M_5	23640	HSPA (Hsp70) binding protein 1(HSPBP1)
M_5	55311	zinc finger protein 444(ZNF444)
M_5	55260	transmembrane protein 143(TMEM143)
M_5	6911	T-box 6(TBX6)
M_5	7620	zinc finger protein 69(ZNF69)
M_5	80741	lymphocyte antigen 6 complex, locus G5C(LY6G5C)

M_5	147658	zinc finger protein 534(ZNF534)
M_5	55769	zinc finger protein 83(ZNF83)
M_5	79788	zinc finger protein 665(ZNF665)
M_5	22809	activating transcription factor 5(ATF5)
M_6	54476	ring finger protein 216(RNF216)
M_6	23086	exophilin 5(EXPH5)
M_6	165140	oxoeicosanoid receptor 1(OXER1)
M_6	283149	B-cell CLL/lymphoma 9-like(BCL9L)
M_6	472	ATM serine/threonine kinase(ATM)
M_6	4297	lysine methyltransferase 2A(KMT2A)
M_6	255082	cancer susceptibility candidate 2 (non-protein
M_6	357	shroom family member 2(SHROOM2)
M_6	10413	Yes associated protein 1(YAP1)
M_6	9183	zw10 kinetochore protein(ZW10)
M_6	51608	golgi to ER traffic protein 4(GET4)
M_6	441194	PMS2 C-terminal like pseudogene(PMS2CL)
M_6	55841	WWC family member 3(WWC3)
M_6	114908	transmembrane protein 123(TMEM123)
M_6	114796	PSMG3 antisense RNA 1 (head to head)(PSMG3-
M_6	26147	PHD finger protein 19(PHF19)
M_6	57646	ubiquitin specific peptidase 28(USP28)
M_6	9354	ubiquitination factor E4A(UBE4A)
M_6	80195	transmembrane protein 254(TMEM254)
M_6	221955	diacylglycerol lipase beta(DAGLB)
M_6	23498	3-hydroxyanthranilate 3,4-dioxygenase(HAAO)
M_6	23288	IQ motif containing E(IQCE)
M_6	22897	centrosomal protein 164(CEP164)
M_6	8065	cullin 5(CUL5)
M_6	10693	chaperonin containing TCP1 subunit 6B(CCT6B)
M_6	9702	centrosomal protein 57(CEP57)
M_6	60496	aminoadipate-semialdehyde dehydrogenase-phosphopantetheinyl transferase(AASDHPPT)

M_6	80028	F-box and leucine rich repeat protein 18(FBXL18)
M_6	23621	beta-secretase 1(BACE1)
M_6	114818	kelch like family member 29(KLHL29)
M_6	143884	CWF19-like 2, cell cycle control (S.
M_6	1662	DEAD-box helicase 10(DDX10)
M_6	23235	salt inducible kinase 2(SIK2)
M_6	140699	maestro heat like repeat family member 8(MROH8)
M_6	79780	coiled-coil domain containing 82(CCDC82)
M_6	79778	MICAL like 2(MICALL2)
M_6	64403	cadherin 24(CDH24)
M_6	55216	chromosome 11 open reading frame 57(C11orf57)
M_6	26173	integrator complex subunit 1(INTS1)
M_6	51092	SID1 transmembrane family member 2(SIDT2)
M_6	29960	mitochondrial rRNA methyltransferase 2(MRM2)
M_6	375190	family with sequence similarity 228 member
M_6	91801	alkB homolog 8, tRNA methyltransferase(ALKBH8)
M_6	221458	kinesin family member 6(KIF6)
M_6	57786	RB associated KRAB zinc finger(RBAK)
M_6	8379	MAD1 mitotic arrest deficient like 1(MAD1L1)
M_6	9907	adaptor related protein complex 5 zeta 1
M_6	4935	G protein-coupled receptor 143(GPR143)
M_6	24145	pannexin 1(PANX1)
M_6	5962	radixin(RDX)
M_6	4863	nuclear protein, coactivator of histone
M_6	219347	TMEM254 antisense RNA 1(TMEM254-AS1)
M_6	84132	ubiquitin specific peptidase 42(USP42)
M_6	7559	zinc finger protein 12(ZNF12)
M_6	9159	proprotein convertase subtilisin/kexin type 7(PCSK7)
M_6	143888	KDEL motif containing 2(KDELC2)
M_6	23353	Sad1 and UNC84 domain containing 1(SUN1)
M_6	221927	BRCA1 associated ATM activator 1(BRAT1)
M_6	8898	myotubularin related protein 2(MTMR2)

M_6	9440	mediator complex subunit 17(MED17)
M_6	221937	forkhead box K1(FOXK1)
M_6	329	baculoviral IAP repeat containing 2(BIRC2)
M_6	867	Cbl proto-oncogene(CBL)
M_6	150771	inositol 1,4,5-trisphosphate receptor interacting protein-like 1(ITPRIPL1)
M_6	85459	centrosomal protein 295(CEP295)
M_6	85458	DIX domain containing 1(DIXDC1)
M_6	5395	PMS1 homolog 2, mismatch repair system
M_6	29941	protein kinase N3(PKN3)
M_6	8662	eukaryotic translation initiation factor 3 subunit
M_6	91893	ferredoxin-fold anticodon binding domain containing 1(FDXACB1)
M_6	23365	Rho guanine nucleotide exchange factor
M_6	143689	piwi like RNA-mediated gene silencing 4(PIWIL4)
M_6	79796	ALG9, alpha-1,2-mannosyltransferase(ALG9)
M_6	54919	dynein axonemal assembly factor 5(DNAAF5)
M_6	143684	family with sequence similarity 76 member
M_6	4361	MRE11 homolog, double strand break repair nuclease(MRE11)
M_6	5519	protein phosphatase 2 scaffold subunit
M_6	260341	transcription factor A, mitochondrial pseudogene
M_6	84437	Myb/SANT DNA binding domain containing 4 with coiled-coils(MSANTD4)
M_6	11155	LIM domain binding 3(LDB3)
M_6	201456	F-box protein 15(FBXO15)
M_6	6907	transducin beta like 1X-linked(TBL1X)
M_6	90639	COX19, cytochrome c oxidase assembly
M_6	2526	fucosyltransferase 4(FUT4)
M_6	7975	MAF bZIP transcription factor K(MAFK)
M_6	727	complement C5(C5)
M_6	56980	PR/SET domain 10(PRDM10)

M_6	432	asialoglycoprotein receptor 1(ASGR1)
M_6	25946	zinc finger protein 385A(ZNF385A)
M_6	649946	ribosomal protein L23a pseudogene 64(RPL23AP64)
M_6	23157	septin 6(SEPT6)
M_6	9678	PHD finger protein 14(PHF14)
M_6	79659	dynein cytoplasmic 2 heavy chain 1(DYNC2H1)
M_6	869	cerebellin 1 precursor(CBLN1)
M_6	55501	carbohydrate sulfotransferase 12(CHST12)
M_6	25884	chordin like 2(CHRDL2)
M_6	84629	trinucleotide repeat containing 18(TNRC18)
M_6	113263	glucocorticoid induced 1(GLCCI1)
M_6	5575	protein kinase cAMP-dependent type I regulatory subunit beta(PRKAR1B)
M_6	29886	sorting nexin 8(SNX8)
M_6	60	actin beta(ACTB)
M_6	6450	SH3 domain binding glutamate rich protein(SH3BGR)
M_6	221476	peptidase inhibitor 16(PI16)
M_6	6752	somatostatin receptor 2(SSTR2)
M_6	115827	RAB3C, member RAS oncogene family(RAB3C)
M_7	54539	NADH:ubiquinone oxidoreductase subunit
M_7	2664	GDP dissociation inhibitor 1(GDI1)
M_7	25847	anaphase promoting complex subunit 13(ANAPC13)
M_7	56180	motile sperm domain containing 1(MOSPD1)
M_7	26071	family with sequence similarity 127 member
M_7	158801	NFKB activating protein pseudogene 1(NKAPP1)
M_7	9130	family with sequence similarity 50 member
M_7	9131	apoptosis inducing factor, mitochondria associated
M_7	399668	small integral membrane protein 10 like
M_7	8776	myotubularin related protein 1(MTMR1)
M_7	6845	vesicle associated membrane protein 7(VAMP7)
M_7	7739	zinc finger protein 185 (LIM domain)(ZNF185)
M_7	3423	iduronate 2-sulfatase(IDS)

M_7	3421	isocitrate dehydrogenase 3 (NAD(+)) gamma(IDH3G)
M_7	8517	inhibitor of kappa light polypeptide gene enhancer in B-cells, kinase gamma(IKBKG)
M_7	3920	lysosomal associated membrane protein 2(LAMP2)
M_7	65109	UPF3 regulator of nonsense transcripts homolog B (yeast)(UPF3B)
M_7	3149	high mobility group box 3(HMGB3)
M_7	23641	leucine zipper down-regulated in cancer 1(LDOC1)
M_7	50814	NAD(P) dependent steroid dehydrogenase-
M_7	5230	phosphoglycerate kinase 1(PGK1)
M_7	64219	praja ring finger ubiquitin ligase 1(PJA1)
M_7	8933	family with sequence similarity 127 member
M_7	27018	brain expressed X-linked 3(BEX3)
M_7	51132	ring finger protein, LIM domain interacting(RLIM)
M_7	55609	zinc finger protein 280C(ZNF280C)
M_7	90121	TSR2, ribosome maturation factor(TSR2)
M_7	10838	zinc finger protein 275(ZNF275)
M_7	203547	VMA21 vacuolar H+-ATPase homolog (S. cerevisiae)(VMA21)
M_7	203523	zinc finger protein 449(ZNF449)
M_7	203522	integrator complex subunit 6 like(INTS6L)
M_7	7789	zinc finger, X-linked, duplicated A(ZXDA)
M_7	2717	galactosidase alpha(GLA)
M_7	3597	interleukin 13 receptor subunit alpha 1(IL13RA1)
M_7	3028	hydroxysteroid 17-beta dehydrogenase
M_7	537	ATPase H+ transporting accessory protein
M_7	84295	PHD finger protein 6(PHF6)
M_7	139081	MAGE family member C3(MAGEC3)
M_7	79921	transcription elongation factor A like 4(TCEAL4)
M_7	51566	armadillo repeat containing, X-linked 3(ARMCX3)
M_7	84061	magnesium transporter 1(MAGT1)
M_7	55916	nuclear transport factor 2 like export factor 2(NXT2)

M_7	83692	CD99 molecule like 2(CD99L2)
M_7	286410	ATPase phospholipid transporting 11C(ATP11C)
M_7	79184	BRCA1/BRCA2-containing complex subunit
M_7	7737	ring finger protein 113A(RNF113A)
M_7	53344	cysteine rich hydrophobic domain 1(CHIC1)
M_7	54470	armadillo repeat containing, X-linked 6(ARMCX6)
M_7	84187	transmembrane protein 164(TMEM164)
M_7	10009	zinc finger and BTB domain containing 33(ZBTB33)
M_7	4534	myotubularin 1(MTM1)
M_7	7411	VHL binding protein 1(VBP1)
M_7	159091	family with sequence similarity 122C(FAM122C)
M_7	159090	family with sequence similarity 122B(FAM122B)
M_7	29978	ubiquilin 2(UBQLN2)
M_7	90736	family with sequence similarity 104 member
M_7	10813	UTP14A small subunit processome
M_7	2010	emerin(EMD)
M_7	2000	E74 like ETS transcription factor 4(ELF4)
M_7	644596	small integral membrane protein 10 like
M_7	550643	long intergenic non-protein coding RNA
M_7	55922	NFKB repressing factor(NKRF)
M_7	9016	solute carrier family 25 member 14(SLC25A14)
M_7	57826	RAP2C, member of RAS oncogene family(RAP2C)
M_7	51634	RNA binding motif protein, X-linked 2(RBMX2)
M_7	81887	LAS1 like, ribosome biogenesis factor(LAS1L)
M_7	2332	fragile X mental retardation 1(FMR1)
M_7	6901	tafazzin(TAZ)
M_7	10735	stromal antigen 2(STAG2)
M_7	8450	cullin 4B(CUL4B)
M_7	51248	PDZ domain containing 11(PDZD11)
M_7	1774	deoxyribonuclease 1 like 1(DNASE1L1)
M_7	63932	chromosome X open reading frame 56(CXorf56)
M_7	57187	THO complex 2(THOC2)

M_7	84707	brain expressed X-linked 2(BEX2)
M_7	3654	interleukin 1 receptor associated kinase 1(IRAK1)
M_7	5631	phosphoribosyl pyrophosphate synthetase 1(PRPS1)
M_7	280655	immunoglobulin (CD79A) binding protein 1 pseudogene 1(IGBP1P1)
M_7	7319	ubiquitin conjugating enzyme E2 A(UBE2A)
M_7	6191	ribosomal protein S4, X-linked(RPS4X)
M_7	6748	signal sequence receptor subunit 4(SSR4)
M_7	79576	NFKB activating protein(NKAP)
M_7	4515	mature T-cell proliferation 1(MTCP1)
M_7	90843	transcription elongation factor A like 8(TCEAL8)
M_7	29071	C1GALT1 specific chaperone 1(C1GALT1C1)
M_7	139596	uracil phosphoribosyltransferase homolog(UPRT)
M_7	4952	OCRL, inositol polyphosphate-5-phosphatase(OCRL)
M_7	9721	G protein regulated inducer of neurite outgrowth
M_7	65991	FUN14 domain containing 2(FUNDC2)
M_7	4841	non-POU domain containing, octamer-
M_7	93380	membrane magnesium transporter 1(MMGT1)
M_7	8409	ubiquitously expressed prefoldin like
M_7	55285	RNA binding motif protein 41(RBM41)
M_7	2157	coagulation factor VIII(F8)
M_7	79868	ALG13, UDP-N-acetylglucosaminyltransferase subunit(ALG13)
M_7	1736	dyskerin pseudouridine synthase 1(DKC1)
M_7	9643	mortality factor 4 like 2(MORF4L2)
M_7	7105	tetraspanin 6(TSPAN6)
M_7	1741	discs large MAGUK scaffold protein 3(DLG3)
M_7	10857	progesterone receptor membrane component
M_7	3188	heterogeneous nuclear ribonucleoprotein H2
M_7	5540	neuropeptide Y receptor Y4(NPY4R)
M_7	51186	transcription elongation factor A like 9(TCEAL9)
M_7	10134	B-cell receptor-associated protein 31(BCAP31)

M_7	27336	HIV-1 Tat specific factor 1(HTATSF1)
M_7	6173	ribosomal protein L36a(RPL36A)
M_7	22	ATP binding cassette subfamily B member 7(ABCB7)
M_7	28985	MCTS1, re-initiation and release factor(MCTS1)
M_7	92002	family with sequence similarity 58 member
M_7	7626	zinc finger protein 75D(ZNF75D)
M_7	85012	transcription elongation factor A like 3(TCEAL3)
M_7	554203	JPX transcript, XIST activator (non-protein
M_7	29934	sorting nexin 12(SNX12)
M_7	3476	immunoglobulin (CD79A) binding protein 1(IGBP1)
M_7	215	ATP binding cassette subfamily D member 1(ABCD1)
M_7	5716	proteasome 26S subunit, non-ATPase 10(PSMD10)
M_7	4204	methyl-CpG binding protein 2(MECP2)
M_7	84548	transmembrane protein 185A(TMEM185A)
M_7	115201	autophagy related 4A cysteine peptidase(ATG4A)
M_7	56271	brain expressed X-linked 4(BEX4)
M_7	728024	chromosome X open reading frame 56 pseudogene(LOC728024)
M_7	10479	solute carrier family 9 member A6(SLC9A6)
M_7	331	X-linked inhibitor of apoptosis(XIAP)
M_7	79979	tRNA methyltransferase 2 homolog B(TRMT2B)
M_7	100130086	heat shock transcription factor family, X-linked
M_7	286451	Yip1 domain family member 6(YIPF6)
M_7	91966	chromosome X open reading frame 40A(CXorf40A)
M_7	1478	cleavage stimulation factor subunit 2(CSTF2)
M_7	8269	transmembrane protein 187(TMEM187)
M_7	8270	L antigen family member 3(LAGE3)
M_7	55869	histone deacetylase 8(HDAC8)
M_7	441518	family with sequence similarity 127 member
M_7	1678	translocase of inner mitochondrial membrane 8 homolog A (yeast)(TIMM8A)
M_7	2539	glucose-6-phosphate dehydrogenase(G6PD)

M_7	54521	WD repeat domain 44(WDR44)
M_7	55559	HAUS augmin like complex subunit 7(HAUS7)
M_7	55558	plexin A3(PLXNA3)
M_7	728758	peptidylprolyl cis/trans isomerase, NIMA-interacting 4 pseudogene 1(PIN4P1)
M_7	292	solute carrier family 25 member 5(SLC25A5)
M_7	1069	centrin 2(CETN2)
M_7	158586	zinc finger, X-linked, duplicated B(ZXDB)
M_7	100272147	C-X9-C motif containing 4(CMC4)
M_7	27301	apurinic/apyrimidinic endodeoxyribonuclease
M_7	60343	family with sequence similarity 3 member A(FAM3A)
M_7	5303	peptidylprolyl cis/trans isomerase, NIMA-interacting
M_7	8266	ubiquitin like 4A(UBL4A)
M_7	8263	coagulation factor VIII-associated 1(F8A1)
M_7	79649	MAP7 domain containing 3(MAP7D3)
M_7	9338	transcription elongation factor A like 1(TCEAL1)
M_7	8260	N(alpha)-acetyltransferase 10, NatA catalytic subunit(NAA10)
M_7	51260	polysaccharide biosynthesis domain containing
M_7	3251	hypoxanthine phosphoribosyltransferase 1(HPRT1)
M_7	10495	ecto-NOX disulfide-thiol exchanger 2(ENOX2)
M_7	541578	chromosome X open reading frame 40B(CXorf40B)
M_7	51114	zinc finger DHHC-type containing 9(ZDHHC9)
M_7	8273	solute carrier family 10 member 3(SLC10A3)
M_7	100126270	FMR1 antisense RNA 1(FMR1-AS1)
M_7	730971	uncharacterized LOC730971(FLJ36777)
M_7	158931	transcription elongation factor A like 6(TCEAL6)
M_7	139231	family with sequence similarity 199, X-
M_7	9383	TSIX transcript, XIST antisense RNA(TSIX)
M_7	169981	spindlin family member 3(SPIN3)
M_7	538	ATPase copper transporting alpha(ATP7A)
M_7	139322	apolipoprotein O like(APOOL)

M_7	29121	C-type lectin domain family 2 member D(CLEC2D)
M_7	4983	oligophrenin 1(OPHN1)
M_7	51616	TATA-box binding protein associated factor
M_7	340562	spermidine/spermine N1-acetyl transferase-like
M_7	139886	spindlin family member 4(SPIN4)
M_7	9823	armadillo repeat containing, X-linked 2(ARMCX2)
M_7	474343	spindlin family member 2B(SPIN2B)
M_7	1121	CHM, Rab escort protein 1(CHM)
M_7	84541	kelch repeat and BTB domain containing 8(KBTBD8)
M_7	54830	nucleoporin 62 C-terminal like(NUP62CL)
M_7	6345	sarcalumenin(SRL)
M_7	221481	armadillo repeat containing 12(ARMC12)
M_7	7503	X inactive specific transcript (non-protein
M_7	166614	doublecortin like kinase 2(DCLK2)
M_7	285847	uncharacterized LOC285847(LOC285847)
M_8	54540	family with sequence similarity 193 member
M_8	91368	CDKN2A interacting protein N-terminal
M_8	84851	tripartite motif containing 52(TRIM52)
M_8	114825	PWWP domain containing 2A(PWWP2A)
M_8	6949	treacle ribosome biogenesis factor 1(TCOF1)
M_8	63920	zinc finger BED-type containing 8(ZBED8)
M_8	6940	zinc finger protein 354A(ZNF354A)
M_8	10111	RAD50 double strand break repair protein(RAD50)
M_8	202181	SUMO interacting motifs containing 1 pseudogene(LOC202181)
M_8	492311	IgA inducing protein(IGIP)
M_8	283458	NME/NM23 nucleoside diphosphate kinase 2 pseudogene 1(NME2P1)
M_8	64374	SIL1 nucleotide exchange factor(SIL1)
M_8	81545	F-box protein 38(FBXO38)
M_8	80762	Nedd4 family interacting protein 1(NDFIP1)
M_8	23291	F-box and WD repeat domain containing

M_8	375484	SUMO interacting motifs containing 1(SIMC1)
M_8	23567	zinc finger protein 346(ZNF346)
M_8	11285	beta-1,4-galactosyltransferase 7(B4GALT7)
M_8	5813	purine rich element binding protein A(PURA)
M_8	23197	Fas associated factor family member 2(FAF2)
M_8	2870	G protein-coupled receptor kinase 6(GRK6)
M_8	57472	CCR4-NOT transcription complex subunit 6(CNOT6)
M_8	55374	transmembrane and coiled-coil domains 6(TMCO6)
M_8	54853	WD repeat domain 55(WDR55)
M_8	7320	ubiquitin conjugating enzyme E2 B(UBE2B)
M_8	51128	secretion associated Ras related GTPase 1B(SAR1B)
M_8	51780	lysine demethylase 3B(KDM3B)
M_8	94081	sideroflexin 1(SFXN1)
M_8	10827	family with sequence similarity 114 member
M_8	6879	TATA-box binding protein associated factor 7(TAF7)
M_8	10011	steroid receptor RNA activator 1(SRA1)
M_8	81789	tigger transposable element derived 6(TIGD6)
M_8	1495	catenin alpha 1(CTNNA1)
M_8	345462	zinc finger protein 879(ZNF879)
M_8	9443	mediator complex subunit 7(MED7)
M_8	64777	required for meiotic nuclear division 5 homolog
M_8	79770	thioredoxin domain containing 15(TXNDC15)
M_8	373863	DND microRNA-mediated repression inhibitor
M_8	56990	CDC42 small effector 2(CDC42SE2)
M_8	134553	chromosome 5 open reading frame 24(C5orf24)
M_8	192286	HIG1 hypoxia inducible domain family member
M_8	9782	matrin 3(MATR3)
M_8	134510	ubiquitin like domain containing CTD phosphatase
M_8	134492	NudC domain containing 2(NUDCD2)
M_8	51649	mitochondrial ribosomal protein S23(MRPS23)
M_8	5601	mitogen-activated protein kinase 9(MAPK9)
M_8	54439	RNA binding motif protein 27(RBM27)

M_8	79685	SAP30 like(SAP30L)
M_8	153830	ring finger protein 145(RNF145)
M_8	54908	spindle apparatus coiled-coil protein 1(SPDL1)
M_8	10307	amyloid beta precursor protein binding family B member 3(APBB3)
M_8	154064	retinoic acid early transcript 1L(RAET1L)
M_8	85007	5-phosphohydroxy-L-lysine phospho-lyase(PHYKPL)
M_8	10569	SLU7 homolog, splicing factor(SLU7)
M_8	55696	RNA binding motif protein 22(RBM22)
M_8	4090	SMAD family member 5(SMAD5)
M_8	5201	prefoldin subunit 1(PFDN1)
M_8	10007	glucosamine-6-phosphate deaminase 1(GNPDA1)
M_8	51428	DEAD-box helicase 41(DDX41)
M_8	26262	tetraspanin 17(TSPAN17)
M_8	643836	ZFP62 zinc finger protein(ZFP62)
M_8	3308	heat shock protein family A (Hsp70) member
M_8	10949	heterogeneous nuclear ribonucleoprotein
M_8	516	ATP synthase, H+ transporting, mitochondrial Fo complex subunit C1 (subunit 9)(ATP5G1)
M_8	134266	GrpE like 2, mitochondrial(GRPEL2)
M_8	134353	LSM11, U7 small nuclear RNA associated(LSM11)
M_8	819	calcium modulating ligand(CAMLG)
M_8	27166	PRELI domain containing 1(PRELID1)
M_8	27430	methionine adenosyltransferase 2B(MAT2B)
M_8	51306	family with sequence similarity 13 member
M_8	23367	La ribonucleoprotein domain family member
M_8	51307	family with sequence similarity 53 member
M_8	9260	PDZ and LIM domain 7(PDLIM7)
M_8	9879	DEAD-box helicase 46(DDX46)
M_8	91272	biorientation of chromosomes in cell division
M_8	3313	heat shock protein family A (Hsp70) member
M_8	3035	histidyl-tRNA synthetase(HARS)

M_8	5245	prohibitin(PHB)
M_8	9685	clathrin interactor 1(CLINT1)
M_8	113829	solute carrier family 35 member A4(SLC35A4)
M_8	51247	poly(A) binding protein interacting protein 2(PAIP2)
M_8	81555	Yip1 domain family member 5(YIPF5)
M_8	57222	endoplasmic reticulum-golgi intermediate compartment 1(ERGIC1)
M_8	117608	zinc finger protein 354B(ZNF354B)
M_8	3550	IK cytokine, down-regulator of HLA II(IK)
M_8	51520	leucyl-tRNA synthetase(LARS)
M_8	56951	chromosome 5 open reading frame 15(C5orf15)
M_8	51164	dynactin subunit 4(DCTN4)
M_8	475	antioxidant 1 copper chaperone(ATOX1)
M_8	644873	long intergenic non-protein coding RNA
M_8	7322	ubiquitin conjugating enzyme E2 D2(UBE2D2)
M_8	54819	zinc finger CCHC-type containing 10(ZCCHC10)
M_8	153527	zinc finger matrin-type 2(ZMAT2)
M_8	285598	ADP ribosylation factor like GTPase 10(ARL10)
M_8	54974	tRNA-histidine guanylyltransferase 1 like(THG1L)
M_8	9794	mastermind like transcriptional coactivator
M_8	153222	CREB3 regulatory factor(CREBRF)
M_8	11267	SNF8, ESCRT-II complex subunit(SNF8)
M_8	9604	ring finger protein 14(RNF14)
M_8	92181	ubiquitin domain containing 2(UBTD2)
M_8	55651	NHP2 ribonucleoprotein(NHP2)
M_8	4238	microfibrillar associated protein 3(MFAP3)
M_8	10902	bromodomain containing 8(BRD8)
M_8	26149	zinc finger protein 658(ZNF658)
M_8	57179	KIAA1191(KIAA1191)
M_8	22993	HMG-box containing 3(HMGXB3)
M_8	1452	casein kinase 1 alpha 1(CSNK1A1)
M_8	3094	histidine triad nucleotide binding protein 1(HINT1)

M_8	51651	peptidyl-tRNA hydrolase 2(PTRH2)
M_8	900	cyclin G1(CCNG1)
M_8	29093	mitochondrial ribosomal protein L22(MRPL22)
M_8	30832	zinc finger protein 354C(ZNF354C)
M_8	202052	DnaJ heat shock protein family (Hsp40) member C18(DNAJC18)
M_8	202051	spermatogenesis associated 24(SPATA24)
M_8	9812	KIAA0141(KIAA0141)
M_8	7416	voltage dependent anion channel 1(VDAC1)
M_8	136	adenosine A2b receptor(ADORA2B)
M_8	90933	tripartite motif containing 41(TRIM41)
M_8	4831	NME/NM23 nucleoside diphosphate kinase 2(NME2)
M_8	4830	NME/NM23 nucleoside diphosphate kinase 1(NME1)
M_8	55819	ring finger protein 130(RNF130)
M_8	3182	heterogeneous nuclear ribonucleoprotein
M_8	9555	H2A histone family member Y(H2AFY)
M_8	4245	mannosyl (alpha-1,3-)-glycoprotein beta-1,2-N-acetylglucosaminyltransferase(MGAT1)
M_8	25929	gem nuclear organelle associated protein 5(GEMIN5)
M_8	51149	MRN complex interacting protein(MRNIP)
M_8	23061	TBC1 domain family member 9B(TBC1D9B)
M_8	5917	arginyl-tRNA synthetase(RARS)
M_8	57396	CDC like kinase 4(CLK4)
M_8	51265	cyclin dependent kinase like 3(CDKL3)
M_8	22838	ring finger protein 44(RNF44)
M_8	51264	mitochondrial ribosomal protein L27(MRPL27)
M_8	1729	diaphanous related formin 1(DIAPH1)
M_8	653501	#N/A
M_8	388955	PRELI domain-containing protein 1, mitochondrial pseudogene(LOC388955)
M_8	53917	RAB24, member RAS oncogene family(RAB24)

M_8	27089	ubiquinol-cytochrome c reductase complex III subunit VII(UQCRQ)
M_8	54882	ankyrin repeat and KH domain containing
M_8	8841	histone deacetylase 3(HDAC3)
M_8	10146	G3BP stress granule assembly factor 1(G3BP1)
M_8	54732	transmembrane p24 trafficking protein 9(TMED9)
M_8	8697	cell division cycle 23(CDC23)
M_8	285676	zinc finger protein 454(ZNF454)
M_8	84321	THO complex 3(THOC3)
M_8	6827	SPT4 homolog, DSIF elongation factor
M_8	4869	nucleophosmin(NPM1)
M_8	4695	NADH:ubiquinone oxidoreductase subunit
M_8	51720	ubiquitin interaction motif containing 1(UIMC1)
M_8	1212	clathrin light chain B(CLTB)
M_8	728554	THO complex 3 pseudogene(LOC728554)
M_8	662	BCL2 interacting protein 1(BNIP1)
M_8	64901	RAN binding protein 17(RANBP17)
M_8	51491	NOP16 nucleolar protein(NOP16)
M_8	29108	PYD and CARD domain containing(PYCARD)
M_8	1627	drebrin 1(DBN1)
M_8	10237	solute carrier family 35 member B1(SLC35B1)
M_8	821	calnexin(CANX)
M_8	5515	protein phosphatase 2 catalytic subunit
M_8	23438	histidyl-tRNA synthetase 2, mitochondrial(HARS2)
M_8	90624	LYR motif containing 7(LYRM7)
M_8	83463	MAX dimerization protein 3(MXD3)
M_8	80230	RUN and FYVE domain containing 1(RUFY1)
M_8	79646	pantothenate kinase 3(PANK3)
M_8	8992	ATPase H+ transporting V0 subunit e1(ATP6V0E1)
M_8	2916	glutamate metabotropic receptor 6(GRM6)
M_8	84105	pterin-4 alpha-carbinolamine dehydratase 2(PCBD2)
M_8	2107	eukaryotic translation termination factor 1(ETF1)

M_8	6500	S-phase kinase associated protein 1(SKP1)
M_8	10915	transcription elongation regulator 1(TCERG1)
M_8	7265	tetratricopeptide repeat domain 1(TTC1)
M_8	10960	lectin, mannose binding 2(LMAN2)
M_8	51121	ribosomal protein L26 like 1(RPL26L1)
M_8	64324	nuclear receptor binding SET domain protein
M_8	9337	CCR4-NOT transcription complex subunit 8(CNOT8)
M_8	200407	cellular repressor of E1A stimulated genes 2(CREG2)
M_8	3187	heterogeneous nuclear ribonucleoprotein H1
M_8	283933	zinc finger protein 843(ZNF843)
M_8	51765	serine/threonine protein kinase 26(STK26)
M_8	10636	regulator of G-protein signaling 14(RGS14)
M_8	10318	TNFAIP3 interacting protein 1(TNIP1)
M_8	729678	long intergenic non-protein coding RNA
M_8	11282	mannosyl (alpha-1,3-)-glycoprotein beta-1,4-N-acetylglucosaminyltransferase, isozyme B(MGAT4B)
M_8	84217	zinc finger MYND-type containing 12(ZMYND12)
M_8	79616	cyclin J like(CCNJL)
M_8	80108	ZFP2 zinc finger protein(ZFP2)
M_8	23245	astrotactin 2(ASTN2)
M_8	654466	fibroblast growth factor 7 pseudogene 3(FGF7P3)
M_8	389741	glioblastoma down-regulated RNA(GLIDR)
M_8	80315	cytoplasmic polyadenylation element binding protein
M_8	400798	chromosome 1 open reading frame 220(C1orf220)
M_8	442421	PTGER4P2-CDK2AP2P2 readthrough, transcribed pseudogene(PTGER4P2-CDK2AP2P2)
M_8	404734	ANKHD1-EIF4EBP3 readthrough(ANKHD1-
M_8	347732	cation channel sperm associated 3(CATSPER3)
M_8	6584	solute carrier family 22 member 5(SLC22A5)
M_8	6583	solute carrier family 22 member 4(SLC22A4)
M_8	116842	liver enriched antimicrobial peptide 2(LEAP2)
M_9	54726	OTU deubiquitinase 4(OTUD4)

M_9	26269	F-box protein 8(FBXO8)
M_9	113510	helicase, POLQ-like(HELQ)
M_9	200316	apolipoprotein B mRNA editing enzyme catalytic subunit 3F(APOBEC3F)
M_9	8649	late endosomal/lysosomal adaptor, MAPK and MTOR activator 3(LAMTOR3)
M_9	83452	RAB33B, member RAS oncogene family(RAB33B)
M_9	27236	ADP ribosylation factor interacting protein
M_9	4717	NADH:ubiquinone oxidoreductase subunit
M_9	8467	SWI/SNF related, matrix associated, actin dependent regulator of chromatin, subfamily a, member
M_9	493856	CDGSH iron sulfur domain 2(CISD2)
M_9	55435	adaptor related protein complex 1 associated regulatory protein(AP1AR)
M_9	3428	interferon gamma inducible protein 16(IFI16)
M_9	92610	TRAF interacting protein with forkhead associated domain(TIFA)
M_9	3148	high mobility group box 2(HMGB2)
M_9	55602	CDKN2A interacting protein(CDKN2AIP)
M_9	58478	enolase-phosphatase 1(ENOPH1)
M_9	1977	eukaryotic translation initiation factor 4E(EIF4E)
M_9	55294	F-box and WD repeat domain containing 7(FBXW7)
M_9	79960	jade family PHD finger 1(JADE1)
M_9	2549	GRB2 associated binding protein 1(GAB1)
M_9	3660	interferon regulatory factor 2(IRF2)
M_9	57117	integrator complex subunit 12(INTS12)
M_9	175	aspartylglucosaminidase(AGA)
M_9	10427	SEC24 homolog B, COPII coat complex
M_9	51809	polypeptide N-acetylgalactosaminyltransferase
M_9	27350	apolipoprotein B mRNA editing enzyme catalytic subunit 3C(APOBEC3C)
M_9	84162	KIAA1109(KIAA1109)

M_9	10561	interferon induced protein 44(IFI44)
M_9	6672	SP100 nuclear antigen(SP100)
M_9	65008	mitochondrial ribosomal protein L1(MRPL1)
M_9	56672	A-kinase interacting protein 1(AKIP1)
M_9	56674	TMEM9 domain family member B(TMEM9B)
M_9	84068	solute carrier family 10 member 7(SLC10A7)
M_9	3033	hydroxyacyl-CoA dehydrogenase(HADH)
M_9	83891	sorting nexin 25(SNX25)
M_9	2483	FSHD region gene 1(FRG1)
M_9	8819	Sin3A associated protein 30(SAP30)
M_9	1519	cathepsin O(CTSO)
M_9	987	LPS responsive beige-like anchor protein(LRBA)
M_9	836	caspase 3(CASP3)
M_9	7098	toll like receptor 3(TLR3)
M_9	4086	SMAD family member 1(SMAD1)
M_9	92345	nuclear assembly factor 1 ribonucleoprotein(NAF1)
M_9	79682	centromere protein U(CENPU)
M_9	166378	spermatogenesis associated 5(SPATA5)
M_9	83666	poly(ADP-ribose) polymerase family member
M_9	27152	inturned planar cell polarity protein(INTU)
M_9	166785	methylmalonic aciduria (cobalamin deficiency) cblA type(MMAA)
M_9	128	alcohol dehydrogenase 5 (class III), chi
M_9	51138	COP9 signalosome subunit 4(COPS4)
M_9	9693	Rap guanine nucleotide exchange factor 2(RAPGEF2)
M_9	60489	apolipoprotein B mRNA editing enzyme catalytic subunit 3G(APOBEC3G)
M_9	57587	cilia and flagella associated protein 97(CFAP97)
M_9	9111	N-myc and STAT interactor(NMI)
M_9	64135	interferon induced with helicase C domain 1(IFIH1)
M_9	80155	N(alpha)-acetyltransferase 15, NatA auxiliary subunit(NAA15)

M_9	1998	E74 like ETS transcription factor 2(ELF2)
M_9	54625	poly(ADP-ribose) polymerase family member
M_9	27068	pyrophosphatase (inorganic) 2(PPA2)
M_9	4750	NIMA related kinase 1(NEK1)
M_9	6059	ATP binding cassette subfamily E member 1(ABCE1)
M_9	55751	transmembrane protein 184C(TMEM184C)
M_9	23173	methionyl aminopeptidase 1(METAP1)
M_9	54969	histone PARylation factor 1(HPF1)
M_9	9255	aminoacyl tRNA synthetase complex interacting multifunctional protein 1(AIMP1)
M_9	11157	LSM6 homolog, U6 small nuclear RNA and mRNA degradation associated(LSM6)
M_9	1182	chloride voltage-gated channel 3(CLCN3)
M_9	3005	H1 histone family member 0(H1F0)
M_9	11162	nudix hydrolase 6(NUDT6)
M_9	255520	ELMO domain containing 2(ELMOD2)
M_9	80167	abhydrolase domain containing 18(ABHD18)
M_9	6737	tripartite motif containing 21(TRIM21)
M_9	5356	pleiotropic regulator 1(PLRG1)
M_9	79884	microtubule associated protein 9(MAP9)
M_9	5188	glutamyl-tRNA amidotransferase subunit B(GATB)
M_9	5359	phospholipid scramblase 1(PLSCR1)
M_9	54809	sterile alpha motif domain containing 9(SAMD9)
M_9	5393	exosome component 9(EXOSC9)
M_9	7323	ubiquitin conjugating enzyme E2 D3(UBE2D3)
M_9	58505	oligosaccharyltransferase complex non-catalytic subunit(OSTC)
M_9	11275	kelch like family member 2(KLHL2)
M_9	90826	protein arginine methyltransferase 9(PRMT9)
M_9	54433	GAR1 ribonucleoprotein(GAR1)
M_9	23158	TBC1 domain family member 9(TBC1D9)
M_9	834	caspase 1(CASP1)

M_9	9246	ubiquitin conjugating enzyme E2 L6(UBE2L6)
M_9	3015	H2A histone family member Z(H2AFZ)
M_9	10384	butyrophilin subfamily 3 member A3(BTN3A3)
M_9	60592	short coiled-coil protein(SCOC)
M_9	84142	family with sequence similarity 175 member
M_9	10393	anaphase promoting complex subunit 10(ANAPC10)
M_9	64108	receptor transporter protein 4(RTP4)
M_9	10964	interferon induced protein 44 like(IFI44L)
M_9	256309	coiled-coil domain containing 110(CCDC110)
M_9	57182	ankyrin repeat domain 50(ANKRD50)
M_9	6307	methylsterol monooxygenase 1(MSMO1)
M_9	53840	tripartite motif containing 34(TRIM34)
M_9	55132	La ribonucleoprotein domain family member
M_9	219285	sterile alpha motif domain containing 9
M_9	3611	integrin linked kinase(ILK)
M_9	55212	Bardet-Biedl syndrome 7(BBS7)
M_9	60559	signal peptidase complex subunit 3(SPCS3)
M_9	201973	primase and DNA directed polymerase(PRIMPOL)
M_9	201965	RWD domain containing 4(RWDD4)
M_9	201931	transmembrane protein 192(TMEM192)
M_9	151636	deltex E3 ubiquitin ligase 3L(DTX3L)
M_9	51023	mitochondrial ribosomal protein S18C(MRPS18C)
M_9	55325	UFM1 specific peptidase 2(UFSP2)
M_9	56916	SWI/SNF-related, matrix-associated actin-dependent regulator of chromatin, subfamily a, containing DEAD/H box 1(SMARCAD1)
M_9	79725	THAP domain containing 9(THAP9)
M_9	80854	SET domain containing lysine methyltransferase
M_9	23240	KIAA0922(KIAA0922)
M_9	93587	tRNA methyltransferase 10A(TRMT10A)
M_9	5481	peptidylprolyl isomerase D(PPID)
M_9	55534	mastermind like transcriptional coactivator

M_9	10424	progesterone receptor membrane component
M_9	57721	methyltransferase like 14(METTL14)
M_9	3965	galectin 9(LGALS9)
M_9	53343	nudix hydrolase 9(NUDT9)
M_9	839	caspase 6(CASP6)
M_9	51574	La ribonucleoprotein domain family member
M_9	256471	major facilitator superfamily domain containing
M_9	3622	inhibitor of growth family member 2(ING2)
M_9	84869	carbonyl reductase 4(CBR4)
M_9	201798	tigger transposable element derived 4(TIGD4)
M_9	201725	chromosome 4 open reading frame 46(C4orf46)
M_9	114769	caspase recruitment domain family member
M_9	79982	DnaJ heat shock protein family (Hsp40) member B14(DNAJB14)
M_9	11118	butyrophilin subfamily 3 member A2(BTN3A2)
M_9	11119	butyrophilin subfamily 3 member A1(BTN3A1)
M_9	84992	phosphatidylinositol glycan anchor biosynthesis class
M_9	80216	alpha kinase 1(ALPK1)
M_9	6609	sphingomyelin phosphodiesterase 1(SMPD1)
M_9	64761	poly(ADP-ribose) polymerase family member
M_9	5910	Rap1 GTPase-GDP dissociation stimulator
M_9	55319	translation machinery associated 16
M_9	132320	sodium channel and clathrin linker 1(SCLT1)
M_9	132321	chromosome 4 open reading frame 33(C4orf33)
M_9	140564	apolipoprotein B mRNA editing enzyme catalytic subunit 3D(APOBEC3D)
M_9	291	solute carrier family 25 member 4(SLC25A4)
M_9	152485	zinc finger protein 827(ZNF827)
M_9	152503	SH3 domain containing 19(SH3D19)
M_9	27309	zinc finger protein 330(ZNF330)
M_9	1635	dCMP deaminase(DCTD)
M_9	60684	trafficking protein particle complex 11(TRAPPC11)

M_9	84640	ubiquitin specific peptidase 38(USP38)
M_9	54739	XIAP associated factor 1(XAF1)
M_9	3430	interferon induced protein 35(IFI35)
M_9	3431	SP110 nuclear body protein(SP110)
M_9	3433	interferon induced protein with tetratricopeptide repeats 2(IFIT2)
M_9	3434	interferon induced protein with tetratricopeptide repeats 1(IFIT1)
M_9	3437	interferon induced protein with tetratricopeptide repeats 3(IFIT3)
M_9	2110	electron transfer flavoprotein dehydrogenase(ETFDH)
M_9	80817	centrosomal protein 44(CEP44)
M_9	256880	uncharacterized LOC256880(LOC256880)
M_9	24138	interferon induced protein with tetratricopeptide repeats 5(IFIT5)
M_9	51170	hydroxysteroid 17-beta dehydrogenase
M_9	837	caspase 4(CASP4)
M_9	3606	interleukin 18(IL18)
M_9	57205	ATPase phospholipid transporting 10D
M_9	51296	solute carrier family 15 member 3(SLC15A3)
M_9	345275	hydroxysteroid 17-beta dehydrogenase
M_9	27235	coenzyme Q2, polyprenyltransferase(COQ2)
M_9	202459	oligosaccharyltransferase complex subunit pseudogene 1(OSTCP1)
M_9	353322	ankyrin repeat domain 37(ANKRD37)
M_9	79132	DExH-box helicase 58(DHX58)
M_9	6786	stromal interaction molecule 1(STIM1)
M_9	10855	heparanase(HPSE)
M_10	54813	kelch like family member 28(KLHL28)
M_10	4860	purine nucleoside phosphorylase(PNP)
M_10	26020	LDL receptor related protein 10(LRP10)
M_10	80344	DDB1 and CUL4 associated factor 11(DCAF11)

M_10	29082	charged multivesicular body protein 4A(CHMP4A)
M_10	10548	transmembrane 9 superfamily member 1(TM9SF1)
M_10	4776	nuclear factor of activated T-cells 4(NFATC4)
M_10	54930	HAUS augmin like complex subunit 4(HAUS4)
M_10	84837	ARHGAP5 antisense RNA 1 (head to head)(ARHGAP5-AS1)
M_10	51222	zinc finger protein 219(ZNF219)
M_10	79711	importin 4(IPO4)
M_10	55837	E2F associated phosphoprotein(EAPP)
M_10	55644	O-sialoglycoprotein endopeptidase(OSGEP)
M_10	23256	sec1 family domain containing 1(SCFD1)
M_10	4901	neural retina leucine zipper(NRL)
M_10	55449	DHRS4 antisense RNA 1(DHRS4-AS1)
M_10	64745	methyltransferase like 17(METTL17)
M_10	394	Rho GTPase activating protein 5(ARHGAP5)
M_10	25831	HECT domain E3 ubiquitin protein ligase
M_10	25983	neuroguidin(NGDN)
M_10	112487	D-tyrosyl-tRNA deacylase 2 (putative)(DTD2)
M_10	10038	poly(ADP-ribose) polymerase 2(PARP2)
M_10	5720	proteasome activator subunit 1(PSME1)
M_10	57680	chromodomain helicase DNA binding protein
M_10	5018	OXA1L, mitochondrial inner membrane
M_10	55147	RNA binding motif protein 23(RBM23)
M_10	51292	guanosine monophosphate reductase 2(GMPR2)
M_10	10419	protein arginine methyltransferase 5(PRMT5)
M_10	122664	tubulin polymerization promoting protein family member 2(TPPP2)
M_10	79178	thiamine triphosphatase(THTPA)
M_10	8487	gem nuclear organelle associated protein 2(GEMIN2)
M_10	5687	proteasome subunit alpha 6(PSMA6)
M_10	599	BCL2 like 2(BCL2L2)
M_10	5693	proteasome subunit beta 5(PSMB5)

M_10	4738	neural precursor cell expressed, developmentally down-regulated 8(NEDD8)
M_10	57594	homeobox and leucine zipper encoding(HOMEZ)
M_10	643866	cerebellin 3 precursor(CBLN3)
M_10	23116	family with sequence similarity 179 member
M_10	51016	ER membrane protein complex subunit 9(EMC9)
M_10	55632	G2/M-phase specific E3 ubiquitin protein
M_10	161424	NOP9 nucleolar protein(NOP9)
M_10	6729	signal recognition particle 54(SRP54)
M_10	63874	abhydrolase domain containing 4(ABHD4)
M_10	25938	HEAT repeat containing 5A(HEATR5A)
M_10	22985	apoptotic chromatin condensation inducer 1(ACIN1)
M_10	161253	RRAD and GEM like GTPase 2(REM2)
M_10	328	apurinic/apyrimidinic endodeoxyribonuclease
M_10	51562	MAP3K12 binding inhibitory protein 1(MBIP)
M_10	80224	nucleotide binding protein like(NUBPL)
M_10	11198	SPT16 homolog, facilitates chromatin remodeling subunit(SUPT16H)
M_10	84312	breast cancer metastasis-suppressor 1-like(BRMS1L)
M_10	9692	KIAA0391(KIAA0391)
M_10	122553	trafficking protein particle complex 6B(TRAPPC6B)
M_10	55017	chromosome 14 open reading frame 119(C14orf119)
M_10	29966	striatin 3(STRN3)
M_10	5875	Rab geranylgeranyltransferase alpha
M_10	90809	transmembrane protein 55B(TMEM55B)
M_10	56948	short chain dehydrogenase/reductase family 39U member 1(SDR39U1)
M_10	23351	KH and NYN domain containing(KHNYN)
M_10	5721	proteasome activator subunit 2(PSME2)
M_10	643382	transmembrane protein 253(TMEM253)
M_10	1603	defender against cell death 1(DAD1)
M_10	145553	magnesium dependent phosphatase 1(MDP1)

M_10	84932	RAB2B, member RAS oncogene family(RAB2B)
M_10	171546	serine palmitoyltransferase small subunit A(SPTSSA)
M_10	115817	dehydrogenase/reductase 1(DHRS1)
M_10	253959	Ral GTPase activating protein catalytic alpha subunit 1(RALGAPA1)
M_10	9878	TOX high mobility group box family member
M_10	60686	chromosome 14 open reading frame 93(C14orf93)
M_10	55701	Rho guanine nucleotide exchange factor
M_10	57820	cyclin B1 interacting protein 1(CCNB1IP1)
M_10	7011	telomerase associated protein 1(TEP1)
M_10	122704	mitochondrial ribosomal protein L52(MRPL52)
M_10	8106	poly(A) binding protein nuclear 1(PABPN1)
M_10	91875	tetratricopeptide repeat domain 5(TTC5)
M_10	5106	phosphoenolpyruvate carboxykinase 2,
M_10	11154	adaptor related protein complex 4 sigma 1
M_10	26277	TERF1 interacting nuclear factor 2(TINF2)
M_10	56339	methyltransferase like 3(METTL3)
M_10	2287	FK506 binding protein 3(FKBP3)
M_10	254170	F-box protein 33(FBXO33)
M_10	57523	NYN domain and retroviral integrase
M_10	3183	heterogeneous nuclear ribonucleoprotein C (C1/C2)(HNRNPC)
M_10	58533	sorting nexin 6(SNX6)
M_10	8906	adaptor related protein complex 1 gamma 2
M_10	57447	NDRG family member 2(NDRG2)
M_10	55072	ring finger protein 31(RNF31)
M_10	10903	myotubularin related protein 11(MTMR11)
M_10	80054	CEBPA antisense RNA 1 (head to head)(CEBPA-
M_10	1050	CCAAT/enhancer binding protein alpha(CEBPA)
M_10	56963	repulsive guidance molecule family member
M_10	7026	nuclear receptor subfamily 2 group F member
M_10	27141	cell death-inducing DFFA-like effector b(CIDEB)

M_10	90673	protein phosphatase 1 regulatory subunit
M_10	283629	testis specific serine kinase 4(TSSK4)
M_10	114130	#N/A
M_11	54815	GATA zinc finger domain containing 2A(GATAD2A)
M_11	150094	salt inducible kinase 1(SIK1)
M_11	11316	coatomer protein complex subunit epsilon(COPE)
M_11	1843	dual specificity phosphatase 1(DUSP1)
M_11	1844	dual specificity phosphatase 2(DUSP2)
M_11	387763	chromosome 11 open reading frame 96(C11orf96)
M_11	7538	ZFP36 ring finger protein(ZFP36)
M_11	1052	CCAAT/enhancer binding protein delta(CEBPD)
M_11	1847	dual specificity phosphatase 5(DUSP5)
M_11	83595	SRY-box 7(SOX7)
M_11	54858	pyroglutamyl-peptidase I(PGPEP1)
M_11	8553	basic helix-loop-helix family member e40(BHLHE40)
M_11	83983	testis specific serine kinase 6(TSSK6)
M_11	23383	MAU2 sister chromatid cohesion factor(MAU2)
M_11	54929	transmembrane protein 161A(TMEM161A)
M_11	100271849	myocyte enhancer factor 2B(MEF2B)
M_11	4616	growth arrest and DNA damage inducible
M_11	23031	microtubule associated serine/threonine kinase
M_11	79086	small integral membrane protein 7(SMIM7)
M_11	51291	GEM interacting protein(GMIP)
M_11	9592	immediate early response 2(IER2)
M_11	729991	BLOC-1 related complex subunit 8(BORCS8)
M_11	8625	regulatory factor X associated ankyrin containing protein(RFXANK)
M_11	8013	nuclear receptor subfamily 4 group A member
M_11	8870	immediate early response 3(IER3)
M_11	23645	protein phosphatase 1 regulatory subunit
M_11	55295	kelch like family member 26(KLHL26)
M_11	388	ras homolog family member B(RHOB)

M_11	1316	Kruppel like factor 6(KLF6)
M_11	7071	Kruppel like factor 10(KLF10)
M_11	5583	protein kinase C eta(PRKCH)
M_11	79648	microcephalin 1(MCPH1)
M_11	29086	BRISC and BRCA1 A complex member 1(BABAM1)
M_11	8061	FOS like 1, AP-1 transcription factor subunit(FOSL1)
M_11	54555	DEAD-box helicase 49(DDX49)
M_11	115098	coiled-coil domain containing 124(CCDC124)
M_11	2152	coagulation factor III, tissue factor(F3)
M_11	7311	ubiquitin A-52 residue ribosomal protein fusion product 1(UBA52)
M_11	467	activating transcription factor 3(ATF3)
M_11	284439	solute carrier family 25 member 42(SLC25A42)
M_11	6446	serum/glucocorticoid regulated kinase 1(SGK1)
M_11	54206	ERBB receptor feedback inhibitor 1(ERRFI1)
M_11	23764	MAF bZIP transcription factor F(MAFF)
M_11	9021	suppressor of cytokine signaling 3(SOCS3)
M_11	157285	homolog of rat pragma of Rnd2(SGK223)
M_11	90459	exoribonuclease 1(ERI1)
M_11	23770	FK506 binding protein 8(FKBP8)
M_11	4792	NFKB inhibitor alpha(NFKBIA)
M_11	55326	1-acylglycerol-3-phosphate O-acyltransferase
M_11	3399	inhibitor of DNA binding 3, HLH protein(ID3)
M_11	1490	connective tissue growth factor(CTGF)
M_11	3397	inhibitor of DNA binding 1, HLH protein(ID1)
M_11	8178	elongation factor for RNA polymerase II(ELL)
M_11	64981	mitochondrial ribosomal protein L34(MRPL34)
M_11	87	actinin alpha 1(ACTN1)
M_11	3569	interleukin 6(IL6)
M_11	2921	C-X-C motif chemokine ligand 3(CXCL3)
M_11	3727	JunD proto-oncogene, AP-1 transcription factor subunit(JUND)

M_11	5864	RAB3A, member RAS oncogene family(RAB3A)
M_11	644128	ribosomal protein L23a pseudogene 53(RPL23AP53)
M_11	3976	leukemia inhibitory factor(LIF)
M_11	3725	Jun proto-oncogene, AP-1 transcription factor
M_11	2353	Fos proto-oncogene, AP-1 transcription factor
M_11	64651	cysteine and serine rich nuclear protein 1(CSRNP1)
M_11	79016	DET1 and DDB1 associated 1(DDA1)
M_11	25804	LSM4 homolog, U6 small nuclear RNA and mRNA degradation associated(LSM4)
M_11	3576	C-X-C motif chemokine ligand 8(CXCL8)
M_11	5997	regulator of G-protein signaling 2(RGS2)
M_11	4207	BORCS8-MEF2B readthrough(BORCS8-MEF2B)
M_11	6372	C-X-C motif chemokine ligand 6(CXCL6)
M_11	51655	ras related dexamethasone induced 1(RASD1)
M_11	9170	lysophosphatidic acid receptor 2(LPAR2)
M_11	57794	SURP and G-patch domain containing 1(SUGP1)
M_11	5473	pro-platelet basic protein(PPBP)
M_11	9639	Rho guanine nucleotide exchange factor
M_11	79660	protein phosphatase 1 regulatory subunit
M_11	7832	BTG anti-proliferation factor 2(BTG2)
M_11	10365	Kruppel like factor 2(KLF2)
M_11	11067	chromosome 10 open reading frame 10(C10orf10)
M_11	55049	chromosome 19 open reading frame 60(C19orf60)
M_11	84769	MPV17 mitochondrial inner membrane protein like 2(MPV17L2)
M_11	80149	zinc finger CCCH-type containing 12A(ZC3H12A)
M_11	1839	heparin binding EGF like growth factor(HBEGF)
M_11	100302650	BRE antisense RNA 1(BRE-AS1)
M_11	2055	ceroid-lipofuscinosis, neuronal 8(CLN8)
M_11	149647	family with sequence similarity 71 member
M_11	3036	hyaluronan synthase 1(HAS1)

M_11	2354	FosB proto-oncogene, AP-1 transcription factor subunit(FOSB)
M_11	3040	hemoglobin subunit alpha 2(HBA2)
M_11	3039	hemoglobin subunit alpha 1(HBA1)
M_11	3164	nuclear receptor subfamily 4 group A member
M_11	212	5'-aminolevulinate synthase 2(ALAS2)
M_11	1263	polo like kinase 3(PLK3)
M_11	9766	sushi domain containing 6(SUSD6)
M_11	79036	KxDL motif containing 1(KXD1)
M_11	25796	6-phosphogluconolactonase(PGLS)
M_11	6364	C-C motif chemokine ligand 20(CCL20)
M_11	6004	regulator of G-protein signaling 16(RGS16)
M_11	57130	ATPase 13A1(ATP13A1)
M_11	2865	free fatty acid receptor 3(FFAR3)
M_11	8658	tankyrase(TNKS)
M_11	9314	Kruppel like factor 4(KLF4)
M_11	170463	single stranded DNA binding protein 4(SSBP4)
M_11	9258	malignant fibrous histiocytoma amplified sequence 1(MFHAS1)
M_11	23373	CREB regulated transcription coactivator 1(CRTC1)
M_11	5196	platelet factor 4(PF4)
M_11	5976	UPF1, RNA helicase and ATPase(UPF1)
M_11	3726	JunB proto-oncogene, AP-1 transcription factor subunit(JUNB)
M_11	5296	phosphoinositide-3-kinase regulatory subunit
M_11	126382	nuclear receptor 2C2 associated protein(NR2C2AP)
M_11	2920	C-X-C motif chemokine ligand 2(CXCL2)
M_11	2919	C-X-C motif chemokine ligand 1(CXCL1)
M_11	55850	unconventional SNARE in the ER 1(USE1)
M_11	93343	multivesicular body subunit 12A(MVB12A)
M_11	1326	mitogen-activated protein kinase kinase kinase
M_11	1959	early growth response 2(EGR2)

M_11	1960	early growth response 3(EGR3)
M_11	1958	early growth response 1(EGR1)
M_11	3043	hemoglobin subunit beta(HBB)
M_11	64332	NFKB inhibitor zeta(NFKBIZ)
M_11	677	ZFP36 ring finger protein like 1(ZFP36L1)
M_11	80176	splA/ryanodine receptor domain and SOCS box containing 1(SPSB1)
M_11	3491	cysteine rich angiogenic inducer 61(CYR61)
M_11	93436	armadillo repeat containing 6(ARMC6)
M_11	1961	early growth response 4(EGR4)
M_11	1647	growth arrest and DNA damage inducible
M_11	51477	inositol-3-phosphate synthase 1(ISYNA1)
M_11	330	baculoviral IAP repeat containing 3(BIRC3)
M_11	6521	solute carrier family 4 member 1 (Diego blood group)(SLC4A1)
M_11	10147	SURP and G-patch domain containing 2(SUGP2)
M_11	7277	tubulin alpha 4a(TUBA4A)
M_11	29970	schwannomin interacting protein 1(SCHIP1)
M_11	3042	hemoglobin subunit mu(HBM)
M_11	6615	snail family transcriptional repressor 1(SNAI1)
M_11	1026	cyclin dependent kinase inhibitor 1A(CDKN1A)
M_11	3552	interleukin 1 alpha(IL1A)
M_11	7124	tumor necrosis factor(TNF)
M_11	51327	alpha hemoglobin stabilizing protein(AHSP)
M_11	4794	NFKB inhibitor epsilon(NFKBIE)
M_11	4783	nuclear factor, interleukin 3 regulated(NFIL3)
M_11	4790	nuclear factor kappa B subunit 1(NFKB1)
M_11	284440	long intergenic non-protein coding RNA
M_11	3398	inhibitor of DNA binding 2, HLH protein(ID2)
M_11	259307	interleukin 4 induced 1(IL4I1)
M_11	145173	beta 3-glucosyltransferase(B3GLCT)
M_11	8793	TNF receptor superfamily member 10d(TNFRSF10D)

M_11	602	B-cell CLL/lymphoma 3(BCL3)
M_11	5971	RELB proto-oncogene, NF-kB subunit(RELB)
M_11	50619	DEF6, guanine nucleotide exchange factor(DEF6)
M_11	25819	nocturnin(NOCT)
M_11	80714	PBX homeobox 4(PBX4)
M_11	80726	KIAA1683(KIAA1683)
M_11	4838	nodal growth differentiation factor(NODAL)
M_11	199675	mast cell expressed membrane protein 1(MCEMP1)
M_11	9235	interleukin 32(IL32)
M_11	56729	resistin(RETN)
M_11	283796	golgin A8 family member I, pseudogene(GOLGA8IP)
M_11	6648	superoxide dismutase 2, mitochondrial(SOD2)
M_11	166929	sphingomyelin synthase 2(SGMS2)
M_11	3695	integrin subunit beta 7(ITGB7)
M_11	2815	glycoprotein IX platelet(GP9)
M_11	7128	TNF alpha induced protein 3(TNFAIP3)
M_11	84099	inhibitor of DNA binding 2B, HLH protein (pseudogene)(ID2B)
M_11	27071	dual adaptor of phosphotyrosine and 3-phosphoinositides 1(DAPP1)
M_11	4317	matrix metallopeptidase 8(MMP8)
M_11	245972	ATPase H+ transporting V0 subunit d2(ATP6V0D2)
M_11	150350	ENTH domain containing 1(ENTHD1)
M_11	4050	lymphotoxin beta(LTB)
M_11	1118	chitinase 1(CHIT1)
M_11	344387	cyclin dependent kinase like 4(CDKL4)
M_11	91948	long intergenic non-protein coding RNA
M_11	116211	transmembrane 4 L six family member 19(TM4SF19)
M_11	4318	matrix metallopeptidase 9(MMP9)
M_11	4321	matrix metallopeptidase 12(MMP12)
M_11	10537	ubiquitin D(UBD)
M_11	3604	TNF receptor superfamily member 9(TNFRSF9)

M_11	89886	SLAM family member 9(SLAMF9)
M_12	54816	zinc finger protein 280D(ZNF280D)
M_12	171423	protein disulfide isomerase family A member 3 pseudogene 1(PDIA3P1)
M_12	3712	isovaleryl-CoA dehydrogenase(IVD)
M_12	79029	spermatogenesis associated 5 like 1(SPATA5L1)
M_12	117155	cation channel sperm associated 2(CATSPER2)
M_12	10776	cAMP regulated phosphoprotein 19(ARPP19)
M_12	161823	adenosine deaminase like(ADAL)
M_12	161779	piggyBac transposable element derived 4(PGBD4)
M_12	8669	eukaryotic translation initiation factor 3 subunit
M_12	100128569	chromosome 19 open reading frame 71(C19orf71)
M_12	51621	Kruppel like factor 13(KLF13)
M_12	54629	family with sequence similarity 63 member
M_12	56204	family with sequence similarity 214 member
M_12	7337	ubiquitin protein ligase E3A(UBE3A)
M_12	81614	non imprinted in Prader-Willi/Angelman syndrome
M_12	8773	synaptosome associated protein 23(SNAP23)
M_12	23312	Dmx like 2(DMXL2)
M_12	8924	HECT and RLD domain containing E3 ubiquitin protein ligase 2(HERC2)
M_12	4236	microfibrillar associated protein 1(MFAP1)
M_12	10169	small EDRK-rich factor 2(SERF2)
M_12	2585	galactokinase 2(GALK2)
M_12	114791	tubulin gamma complex associated protein
M_12	9836	leucine carboxyl methyltransferase 2(LCMT2)
M_12	80021	transmembrane protein 62(TMEM62)
M_12	56924	p21 (RAC1) activated kinase 6(PAK6)
M_12	23339	VPS39, HOPS complex subunit(VPS39)
M_12	51103	NADH:ubiquinone oxidoreductase complex assembly factor 1(NDUFAF1)
M_12	283742	family with sequence similarity 98 member

M_12	55192	DnaJ heat shock protein family (Hsp40) member C17(DNAJC17)
M_12	1854	deoxyuridine triphosphatase(DUT)
M_12	113201	cancer susceptibility candidate 4(CASC4)
M_12	146050	zinc finger and SCAN domain containing
M_12	51187	ribosomal L24 domain containing 1(RSL24D1)
M_12	146059	codanin 1(CDAN1)
M_12	29766	tropomodulin 3(TMOD3)
M_12	123169	LEO1 homolog, Paf1/RNA polymerase II complex component(LEO1)
M_12	8681	JMJD7-PLA2G4B readthrough(JMJD7-PLA2G4B)
M_12	90416	chromosome 15 open reading frame 57(C15orf57)
M_12	729082	OIP5 antisense RNA 1(OIP5-AS1)
M_12	54989	zinc finger protein 770(ZNF770)
M_12	9236	cell cycle progression 1(CCPG1)
M_12	2595	glucosidase alpha, neutral C(GANC)
M_12	55142	HAUS augmin like complex subunit 2(HAUS2)
M_12	255252	leucine rich repeat containing 57(LRRC57)
M_12	7082	tight junction protein 1(TJP1)
M_12	2553	GA binding protein transcription factor beta subunit 1(GABPB1)
M_12	7158	tumor protein p53 binding protein 1(TP53BP1)
M_12	26258	biogenesis of lysosomal organelles complex 1 subunit 6(BLOC1S6)
M_12	56851	ER membrane protein complex subunit 7(EMC7)
M_12	64864	regulatory factor X7(RFX7)
M_12	56986	DTW domain containing 1(DTWD1)
M_12	55177	regulator of microtubule dynamics 3(RMDN3)
M_12	6938	transcription factor 12(TCF12)
M_12	25963	transmembrane protein 87A(TMEM87A)
M_12	55056	uncharacterized protein FLJ10038(FLJ10038)
M_12	9101	ubiquitin specific peptidase 8(USP8)

M_12	79768	katanin regulatory subunit B1 like 1(KATNBL1)
M_12	80208	spastic paraplegia 11 (autosomal recessive)(SPG11)
M_12	102	ADAM metallopeptidase domain 10(ADAM10)
M_12	22909	FANCD2/FANCI-associated nuclease 1(FAN1)
M_12	100129387	GABPB1 antisense RNA 1(GABPB1-AS1)
M_12	5597	mitogen-activated protein kinase 6(MAPK6)
M_12	113189	carbohydrate sulfotransferase 14(CHST14)
M_12	27229	tubulin gamma complex associated protein
M_12	27079	RNA pseudouridylate synthase domain containing
M_12	9677	diphosphoinositol pentakisphosphate kinase
M_12	57099	apoptosis and caspase activation inhibitor(AVEN)
M_12	197131	ubiquitin protein ligase E3 component n-recognin
M_12	4643	myosin IE(MYO1E)
M_12	23582	cyclin D1 binding protein 1(CCNDBP1)
M_12	84529	chromosome 15 open reading frame 41(C15orf41)
M_12	89978	diphthamine biosynthesis 6(DPH6)
M_12	23741	EP300 interacting inhibitor of differentiation 1(EID1)
M_12	6652	sorbitol dehydrogenase(SORD)
M_12	196951	family with sequence similarity 227 member
M_12	6727	signal recognition particle 14(SRP14)
M_12	440275	eukaryotic translation initiation factor 2 alpha kinase 4(EIF2AK4)
M_12	440278	cation channel sperm associated 2 pseudogene 1(CATSPER2P1)
M_12	84888	signal peptide peptidase like 2A(SPPL2A)
M_12	23191	cytoplasmic FMR1 interacting protein 1(CYFIP1)
M_12	22893	bromo adjacent homology domain containing
M_12	1159	creatine kinase, mitochondrial 1B(CKMT1B)
M_12	9318	COP9 signalosome subunit 2(COPS2)
M_12	23005	mitogen-activated protein kinase binding protein 1(MAPKBP1)
M_12	146057	tau tubulin kinase 2(TTBK2)

M_12	51234	ER membrane protein complex subunit 4(EMC4)
M_12	26015	RNA polymerase II associated protein 1(RPAP1)
M_12	51496	CTD small phosphatase like 2(CTDSPL2)
M_12	64397	zinc finger protein 106(ZNF106)
M_12	54893	myotubularin related protein 10(MTMR10)
M_12	54617	INO80 complex subunit(INO80)
M_12	644809	chromosome 15 open reading frame 56(C15orf56)
M_12	23269	MGA, MAX dimerization protein(MGA)
M_12	23431	adaptor related protein complex 4 epsilon 1
M_12	84936	zinc finger FYVE-type containing 19(ZFYVE19)
M_12	643338	chromosome 15 open reading frame 62(C15orf62)
M_12	9716	aquarius intron-binding spliceosomal factor(AQR)
M_12	79664	interactor of little elongation complex ELL subunit
M_12	11261	calcineurin like EF-hand protein 1(CHP1)
M_12	663	BCL2 interacting protein 2(BNIP2)
M_12	2923	protein disulfide isomerase family A member
M_12	25764	huntingtin interacting protein K(HYPK)
M_12	54822	transient receptor potential cation channel subfamily M member 7(TRPM7)
M_12	23168	RTF1 homolog, Paf1/RNA polymerase II complex component(RTF1)
M_12	9728	SECIS binding protein 2 like(SECISBP2L)
M_12	548596	creatine kinase, mitochondrial 1A(CKMT1A)
M_12	80237	elongation factor for RNA polymerase II 3(ELL3)
M_12	54778	ring finger protein 111(RNF111)
M_12	9990	solute carrier family 12 member 6(SLC12A6)
M_12	57617	VPS18, CORVET/HOPS core subunit(VPS18)
M_12	729176	katanin regulatory subunit B1 like 1 pseudogene 6(KATNBL1P6)
M_12	254531	lysophosphatidylcholine acyltransferase 4(LPCAT4)
M_12	2644	GTP cyclohydrolase I feedback regulator(GCHFR)
M_12	55505	NOP10 ribonucleoprotein(NOP10)

M_12	30844	EH domain containing 4(EHD4)
M_12	9488	phosphatidylinositol glycan anchor biosynthesis class
M_12	81488	RNA polymerase II subunit M(POLR2M)
M_12	56160	NSE3 homolog, SMC5-SMC6 complex
M_12	4644	myosin VA(MYO5A)
M_12	653483	AFDN antisense RNA 1 (head to head)(AFDN-AS1)
M_12	152641	WWC2 antisense RNA 2(WWC2-AS2)
M_12	54067	C21orf62 antisense RNA 1(C21orf62-AS1)
M_12	1160	creatine kinase, mitochondrial 2(CKMT2)
M_12	619189	serine incorporator 4(SERINC4)
M_12	80014	WW and C2 domain containing 2(WWC2)
M_12	4213	Meis homeobox 3 pseudogene 1(MEIS3P1)
M_12	29767	tropomodulin 2(TMOD2)
M_12	7782	solute carrier family 30 member 4(SLC30A4)
M_12	4301	afadin, adherens junction formation factor(AFDN)
M_12	221016	#N/A
M_13	54976	chromosome 20 open reading frame 27(C20orf27)
M_13	22803	5'-3' exoribonuclease 2(XRN2)
M_13	63926	ankyrin repeat and EF-hand domain containing
M_13	22888	U-box domain containing 5(UBOX5)
M_13	51340	crooked neck pre-mRNA splicing factor 1(CRNKL1)
M_13	55968	NSFL1 cofactor(NSFL1C)
M_13	3420	isocitrate dehydrogenase 3 (NAD(+)) beta(IDH3B)
M_13	92667	mitochondrial genome maintenance exonuclease
M_13	56255	thioredoxin related transmembrane protein 4(TMX4)
M_13	119587	carboxypeptidase X, M14 family member 2(CPXM2)
M_13	65992	DDRGK domain containing 1(DDRGK1)
M_13	25943	chromosome 20 open reading frame 194(C20orf194)
M_13	55317	adaptor related protein complex 5 sigma 1
M_13	6628	small nuclear ribonucleoprotein polypeptides B and B1(SNRPB)
M_13	140680	chromosome 20 open reading frame 96(C20orf96)

M_13	57325	lysine acetyltransferase 14(KAT14)
M_13	9454	homer scaffolding protein 3(HOMER3)
M_13	57506	mitochondrial antiviral signaling protein(MAVS)
M_13	1457	casein kinase 2 alpha 1(CSNK2A1)
M_13	113278	solute carrier family 52 member 3(SLC52A3)
M_13	10621	RNA polymerase III subunit F(POLR3F)
M_13	54675	cardiolipin synthase 1(CRLS1)
M_13	140809	sulfiredoxin 1(SRXN1)
M_13	55184	double zinc ribbon and ankyrin repeat domains
M_13	2849	G protein-coupled receptor 26(GPR26)
M_13	51605	tRNA methyltransferase 6(TRMT6)
M_13	6666	SRY-box 12(SOX12)
M_13	128637	TBC1 domain family member 20(TBC1D20)
M_13	80025	pantothenate kinase 2(PANK2)
M_13	80023	neurensin 2(NRSN2)
M_13	9145	synaptogyrin 1(SYNGR1)
M_13	9491	proteasome inhibitor subunit 1(PSMF1)
M_13	4248	mannosyl (beta-1,4-)-glycoprotein beta-1,4-N-acetylglucosaminyltransferase(MGAT3)
M_13	23236	phospholipase C beta 1(PLCB1)
M_13	55614	kinesin family member 16B(KIF16B)
M_13	149840	chromosome 20 open reading frame 196(C20orf196)
M_13	51466	Enah/Vasp-like(EVL)
M_13	64773	PC-esterase domain containing 1A(PCED1A)
M_13	64601	VPS16, CORVET/HOPS core subunit(VPS16)
M_13	8760	CDP-diacylglycerol synthase 2(CDS2)
M_13	8195	McKusick-Kaufman syndrome(MKKS)
M_13	51575	ESF1 nucleolar pre-rRNA processing protein
M_13	22903	BTB domain containing 3(BTBD3)
M_13	10616	RANBP2-type and C3HC4-type zinc finger containing 1(RBCK1)
M_13	54498	spermine oxidase(SMOX)

M_13	10528	NOP56 ribonucleoprotein(NOP56)
M_13	10483	Sec23 homolog B, coat complex II
M_13	85364	zinc finger CCHC-type containing 3(ZCCHC3)
M_13	79175	zinc finger protein 343(ZNF343)
M_13	55617	taspase 1(TASP1)
M_13	60493	FAST kinase domains 5(FASTKD5)
M_13	83541	family with sequence similarity 110 member
M_13	11237	ring finger protein 24(RNF24)
M_13	2009	echinoderm microtubule associated protein like
M_13	3704	inosine triphosphatase(ITPA)
M_13	10858	cytochrome P450 family 46 subfamily A member 1(CYP46A1)
M_13	5786	protein tyrosine phosphatase, receptor type A(PTPRA)
M_13	283106	casein kinase 2 alpha 3(CSNK2A3)
M_13	1059	centromere protein B(CENPB)
M_13	55321	transmembrane protein 74B(TMEM74B)
M_13	140901	serine/threonine kinase 35(STK35)
M_13	400831	chromosome 20 open reading frame 202(C20orf202)
M_13	54751	filamin binding LIM protein 1(FBLIM1)
M_13	29058	transmembrane protein 230(TMEM230)
M_13	84515	minichromosome maintenance 8 homologous recombination repair factor(MCM8)
M_13	10741	RB binding protein 9, serine hydrolase(RBBP9)
M_13	8455	attractin(ATRN)
M_13	41	acid sensing ion channel subunit 1(ASIC1)
M_13	2280	FK506 binding protein 1A(FKBP1A)
M_13	7692	zinc finger protein 133(ZNF133)
M_13	64949	mitochondrial ribosomal protein S26(MRPS26)
M_13	128710	SLX4 interacting protein(SLX4IP)
M_13	54453	Ras and Rab interactor 2(RIN2)
M_13	9962	solute carrier family 23 member 2(SLC23A2)
M_13	64412	GDNF inducible zinc finger protein 1(GZF1)

M_13	9469	carbohydrate sulfotransferase 3(CHST3)
M_13	4163	mutated in colorectal cancers(MCC)
M_13	5111	proliferating cell nuclear antigen(PCNA)
M_13	100270804	uncharacterized LOC100270804(LOC100270804)
M_13	994	cell division cycle 25B(CDC25B)
M_13	55857	kizuna centrosomal protein(KIZ)
M_14	1521	cathepsin W(CTSW)
M_14	22914	killer cell lectin like receptor K1(KLRK1)
M_14	7535	zeta chain of T cell receptor associated protein kinase 70(ZAP70)
M_14	10225	CD96 molecule(CD96)
M_14	939	CD27 molecule(CD27)
M_14	1493	cytotoxic T-lymphocyte associated protein 4(CTLA4)
M_14	6352	C-C motif chemokine ligand 5(CCL5)
M_14	925	CD8a molecule(CD8A)
M_14	53347	ubiquitin associated and SH3 domain containing A(UBASH3A)
M_14	201633	T-cell immunoreceptor with Ig and ITIM
M_14	27240	signaling threshold regulating transmembrane adaptor
M_14	5551	perforin 1(PRF1)
M_14	356	Fas ligand(FASLG)
M_14	8698	sphingosine-1-phosphate receptor 4(S1PR4)
M_14	4818	natural killer cell granule protein 7(NKG7)
M_14	114836	SLAM family member 6(SLAMF6)
M_14	10663	C-X-C motif chemokine receptor 6(CXCR6)
M_14	84174	Src like adaptor 2(SLA2)
M_14	27334	purinergic receptor P2Y10(P2RY10)
M_14	921	CD5 molecule(CD5)
M_14	919	CD247 molecule(CD247)
M_14	916	CD3e molecule(CD3E)
M_14	914	CD2 molecule(CD2)
M_14	3560	interleukin 2 receptor subunit beta(IL2RB)

M_14	2833	C-X-C motif chemokine receptor 3(CXCR3)
M_14	149628	pyrin and HIN domain family member 1(PYHIN1)
M_14	50615	interleukin 21 receptor(IL21R)
M_14	3702	IL2 inducible T-cell kinase(ITK)
M_14	3662	interferon regulatory factor 4(IRF4)
M_14	4283	C-X-C motif chemokine ligand 9(CXCL9)
M_14	3561	interleukin 2 receptor subunit gamma(IL2RG)
M_14	4063	lymphocyte antigen 9(LY9)
M_14	4068	SH2 domain containing 1A(SH2D1A)
M_14	915	CD3d molecule(CD3D)
M_14	30009	T-box 21(TBX21)
M_14	5790	protein tyrosine phosphatase, receptor type C associated protein(PTPRCAP)
M_14	154075	sterile alpha motif domain containing 3(SAMD3)
M_14	57823	SLAM family member 7(SLAMF7)
M_14	55423	signal regulatory protein gamma(SIRPG)
M_14	3003	granzyme K(GZMK)
M_14	29851	inducible T-cell costimulator(ICOS)
M_14	3001	granzyme A(GZMA)
M_14	3002	granzyme B(GZMB)
M_14	2999	granzyme H(GZMH)
M_14	5880	ras-related C3 botulinum toxin substrate 2 (rho family, small GTP binding protein Rac2)(RAC2)
M_14	6504	signaling lymphocytic activation molecule family member 1(SLAMF1)
M_14	8530	cystatin F(CST7)
M_14	729230	C-C motif chemokine receptor 2(CCR2)
M_14	9744	ArfGAP with coiled-coil, ankyrin repeat and PH domains 1(ACAP1)
M_14	257101	zinc finger protein 683(ZNF683)
M_14	387751	GTPase, very large interferon inducible pseudogene 1(GVINP1)

M_14	3594	interleukin 12 receptor subunit beta 1(IL12RB1)
M_14	387357	thymocyte selection associated(THEMIS)
M_14	29909	G protein-coupled receptor 171(GPR171)
M_14	924	CD7 molecule(CD7)
M_14	399	ras homolog family member H(RHOH)
M_14	50852	T cell receptor associated transmembrane adaptor
M_14	8807	interleukin 18 receptor accessory protein(IL18RAP)
M_14	80380	programmed cell death 1 ligand 2(PDCD1LG2)
M_14	917	CD3g molecule(CD3G)
M_14	50943	forkhead box P3(FOXP3)
M_14	1236	C-C motif chemokine receptor 7(CCR7)
M_14	10563	C-X-C motif chemokine ligand 13(CXCL13)
M_14	26279	phospholipase A2 group IID(PLA2G2D)
M_14	9402	GRB2-related adaptor protein 2(GRAP2)
M_14	283897	chromosome 16 open reading frame 54(C16orf54)
M_14	3004	granzyme M(GZMM)
M_14	8320	eomesodermin(EOMES)
M_14	10673	tumor necrosis factor superfamily member
M_14	286530	purinergic receptor P2Y8(P2RY8)
M_14	2672	growth factor independent 1 transcriptional
M_14	27299	ADAM like decysin 1(ADAMDEC1)
M_14	26253	C-type lectin domain family 4 member E(CLEC4E)
M_14	3932	LCK proto-oncogene, Src family tyrosine
M_14	5133	programmed cell death 1(PDCD1)
M_14	926	CD8b molecule(CD8B)
M_15	55761	tetratricopeptide repeat domain 17(TTC17)
M_15	54765	tripartite motif containing 44(TRIM44)
M_15	23279	nucleoporin 160(NUP160)
M_15	23378	ribosomal RNA processing 8, methyltransferase, homolog (yeast)(RRP8)
M_15	84364	ADP ribosylation factor GTPase activating protein 2(ARFGAP2)

M_15	120227	cytochrome P450 family 2 subfamily R member
M_15	81846	SET binding factor 2(SBF2)
M_15	54503	zinc finger DHHC-type containing 13(ZDHHC13)
M_15	143458	low density lipoprotein receptor class A domain containing 3(LDLRAD3)
M_15	7465	WEE1 G2 checkpoint kinase(WEE1)
M_15	50862	ring finger protein 141(RNF141)
M_15	6240	ribonucleotide reductase catalytic subunit M1(RRM1)
M_15	7748	zinc finger protein 195(ZNF195)
M_15	966	CD59 molecule(CD59)
M_15	57053	cholinergic receptor nicotinic alpha 10
M_15	9646	CTR9 homolog, Paf1/RNA polymerase II complex component(CTR9)
M_15	9409	peroxisomal biogenesis factor 16(PEX16)
M_15	55343	solute carrier family 35 member C1(SLC35C1)
M_15	5702	proteasome 26S subunit, ATPase 3(PSMC3)
M_15	7251	tumor susceptibility 101(TSG101)
M_15	55293	UEV and lactate/malate dehyrogenase
M_15	23647	ADP ribosylation factor interacting protein
M_15	440026	transmembrane protein 41B(TMEM41B)
M_15	26297	secretion regulating guanine nucleotide exchange factor(SERGEF)
M_15	2965	general transcription factor IIH subunit 1(GTF2H1)
M_15	392	Rho GTPase activating protein 1(ARHGAP1)
M_15	5682	proteasome subunit alpha 1(PSMA1)
M_15	196074	methyltransferase like 15(METTL15)
M_15	8050	pyruvate dehydrogenase complex component
M_15	196294	inner mitochondrial membrane peptidase subunit
M_15	29763	protein kinase C and casein kinase substrate in neurons 3(PACSIN3)
M_15	1408	cryptochrome circadian clock 2(CRY2)
M_15	2132	exostosin glycosyltransferase 2(EXT2)

M_15	120526	DnaJ heat shock protein family (Hsp40) member C24(DNAJC24)
M_15	120534	ADP ribosylation factor like GTPase 14 effector protein(ARL14EP)
M_15	22800	related RAS viral (r-ras) oncogene homolog
M_15	4722	NADH:ubiquinone oxidoreductase core subunit
M_15	5954	reticulocalbin 1(RCN1)
M_15	84280	BTB domain containing 10(BTBD10)
M_15	119710	chromosome 11 open reading frame 74(C11orf74)
M_15	79096	chromosome 11 open reading frame 49(C11orf49)
M_15	55327	lin-7 homolog C, crumbs cell polarity complex component(LIN7C)
M_15	79832	glutamine and serine rich 1(QSER1)
M_15	84067	family with sequence similarity 160 member
M_15	10527	importin 7(IPO7)
M_15	6764	suppression of tumorigenicity 5(ST5)
M_15	11234	HPS5, biogenesis of lysosomal organelles complex 2 subunit 2(HPS5)
M_15	1315	coatomer protein complex subunit beta 1(COPB1)
M_15	26515	translocase of inner mitochondrial membrane
M_15	1982	eukaryotic translation initiation factor 4 gamma
M_15	10612	tripartite motif containing 3(TRIM3)
M_15	29099	COMM domain containing 9(COMMD9)
M_15	113174	serum amyloid A like 1(SAAL1)
M_15	833	cysteinyl-tRNA synthetase(CARS)
M_15	55128	tripartite motif containing 68(TRIM68)
M_15	4076	cell cycle associated protein 1(CAPRIN1)
M_15	144108	SPT2 chromatin protein domain containing
M_15	847	catalase(CAT)
M_15	4928	nucleoporin 98(NUP98)
M_15	91057	coiled-coil domain containing 34(CCDC34)
M_15	51144	hydroxysteroid 17-beta dehydrogenase

M_15	51317	PHD finger protein 21A(PHF21A)
M_15	4925	nucleobindin 2(NUCB2)
M_15	51074	APAF1 interacting protein(APIP)
M_15	258010	small VCP interacting protein(SVIP)
M_15	8539	apoptosis inhibitor 5(API5)
M_15	10658	CUGBP, Elav-like family member 1(CELF1)
M_15	283254	harbinger transposase derived 1(HARBI1)
M_15	283267	long intergenic non-protein coding RNA
M_15	7003	TEA domain transcription factor 1(TEAD1)
M_15	84188	fatty acyl-CoA reductase 1(FAR1)
M_15	1479	cleavage stimulation factor subunit 3(CSTF3)
M_15	53	acid phosphatase 2, lysosomal(ACP2)
M_15	55226	N-acetyltransferase 10(NAT10)
M_15	26610	elongator acetyltransferase complex subunit 4(ELP4)
M_15	55031	ubiquitin specific peptidase 47(USP47)
M_15	23788	mitochondrial carrier 2(MTCH2)
M_15	7189	TNF receptor associated factor 6(TRAF6)
M_15	10944	chromosome 11 open reading frame 58(C11orf58)
M_15	221120	alkB homolog 3, alpha-ketoglutaratedependent dioxygenase(ALKBH3)
M_15	114971	protein tyrosine phosphatase, mitochondrial
M_15	23075	SWAP switching B-cell complex 70kDa
M_15	9776	autophagy related 13(ATG13)
M_15	7702	zinc finger protein 143(ZNF143)
M_15	23258	DENN domain containing 5A(DENND5A)
M_15	55709	kelch repeat and BTB domain containing 4(KBTBD4)
M_15	8567	MAP kinase activating death domain(MADD)
M_15	79797	zinc finger protein 408(ZNF408)
M_15	10196	protein arginine methyltransferase 3(PRMT3)
M_15	5286	phosphatidylinositol-4-phosphate 3-kinase catalytic subunit type 2 alpha(PIK3C2A)
M_15	8525	diacylglycerol kinase zeta(DGKZ)

M_15	55626	autophagy and beclin 1 regulator 1(AMBRA1)
M_15	26273	F-box protein 3(FBXO3)
M_15	4676	nucleosome assembly protein 1 like 4(NAP1L4)
M_15	2188	Fanconi anemia complementation group F(FANCF)
M_15	55346	t-complex 11 like 1(TCP11L1)
M_15	58486	zinc finger BED-type containing 5(ZBED5)
M_15	493860	coiled-coil domain containing 73(CCDC73)
M_15	91252	solute carrier family 39 member 13(SLC39A13)
M_15	650368	tumor suppressing subtransferable candidate 2 pseudogene(TSSC2)
M_15	84457	phytanoyl-CoA 2-hydroxylase interacting protein like(PHYHIPL)
M_15	406	aryl hydrocarbon receptor nuclear translocator
M_15	7761	zinc finger protein 214(ZNF214)
M_15	6041	ribonuclease L(RNASEL)
M_15	79582	sperm associated antigen 16(SPAG16)
M_15	3939	lactate dehydrogenase A(LDHA)
M_15	1643	damage specific DNA binding protein 2(DDB2)
M_15	338323	NLR family pyrin domain containing 14(NLRP14)
M_15	54922	Ras interacting protein 1(RASIP1)
M_15	10480	eukaryotic translation initiation factor 3 subunit
M_15	55742	parvin alpha(PARVA)
M_15	3140	major histocompatibility complex, class I-
M_15	84740	AFAP1 antisense RNA 1(AFAP1-AS1)
M_15	27288	RNA binding motif protein, X-linked like
M_15	84674	caspase recruitment domain family member
M_16	55766	H2A histone family member J(H2AFJ)
M_16	8356	histone cluster 1 H3 family member j(HIST1H3J)
M_16	8357	histone cluster 1 H3 family member h(HIST1H3H)
M_16	8354	histone cluster 1 H3 family member i(HIST1H3I)
M_16	8355	histone cluster 1 H3 family member g(HIST1H3G)
M_16	8352	histone cluster 1 H3 family member c(HIST1H3C)

M_16	8353	histone cluster 1 H3 family member e(HIST1H3E)
M_16	8350	histone cluster 1 H3 family member a(HIST1H3A)
M_16	85236	histone cluster 1 H2B family member k(HIST1H2BK)
M_16	8348	histone cluster 1 H2B family member o(HIST1H2BO)
M_16	8349	histone cluster 2 H2B family member e(HIST2H2BE)
M_16	121504	histone cluster 4 H4(HIST4H4)
M_16	3007	histone cluster 1 H1 family member d(HIST1H1D)
M_16	3010	histone cluster 1 H1 family member t(HIST1H1T)
M_16	3009	histone cluster 1 H1 family member b(HIST1H1B)
M_16	3013	histone cluster 1 H2A family member d(HIST1H2AD)
M_16	3012	histone cluster 1 H2A family member e(HIST1H2AE)
M_16	3018	histone cluster 1 H2B family member b(HIST1H2BB)
M_16	3017	histone cluster 1 H2B family member d(HIST1H2BD)
M_16	128312	histone cluster 3 H2B family member b(HIST3H2BB)
M_16	8367	histone cluster 1 H4 family member e(HIST1H4E)
M_16	8366	histone cluster 1 H4 family member b(HIST1H4B)
M_16	8294	histone cluster 1 H4 family member i(HIST1H4I)
M_16	85235	histone cluster 1 H2A family member h(HIST1H2AH)
M_16	8358	histone cluster 1 H3 family member b(HIST1H3B)
M_16	8361	histone cluster 1 H4 family member f(HIST1H4F)
M_16	8360	histone cluster 1 H4 family member d(HIST1H4D)
M_16	8363	histone cluster 1 H4 family member j(HIST1H4J)
M_16	8362	histone cluster 1 H4 family member k(HIST1H4K)
M_16	8365	histone cluster 1 H4 family member h(HIST1H4H)
M_16	8364	histone cluster 1 H4 family member c(HIST1H4C)
M_16	126961	histone cluster 2 H3 family member c(HIST2H3C)
M_16	3024	histone cluster 1 H1 family member a(HIST1H1A)
M_16	8368	histone cluster 1 H4 family member l(HIST1H4L)
M_16	92815	histone cluster 3 H2A(HIST3H2A)
M_16	8370	histone cluster 2 H4 family member a(HIST2H4A)
M_16	8346	histone cluster 1 H2B family member i(HIST1H2BI)
M_16	8347	histone cluster 1 H2B family member c(HIST1H2BC)

M_16	8342	histone cluster 1 H2B family member
M_16	8343	histone cluster 1 H2B family member f(HIST1H2BF)
M_16	8344	histone cluster 1 H2B family member e(HIST1H2BE)
M_16	8345	histone cluster 1 H2B family member h(HIST1H2BH)
M_16	8338	histone cluster 2 H2A family member c(HIST2H2AC)
M_16	8339	histone cluster 1 H2B family member g(HIST1H2BG)
M_16	8340	histone cluster 1 H2B family member l(HIST1H2BL)
M_16	8341	histone cluster 1 H2B family member n(HIST1H2BN)
M_16	81697	olfactory receptor family 2 subfamily B member
M_16	8351	histone cluster 1 H3 family member d(HIST1H3D)
M_16	26212	olfactory receptor family 2 subfamily B member
M_16	8968	histone cluster 1 H3 family member f(HIST1H3F)
M_16	8969	histone cluster 1 H2A family member g(HIST1H2AG)
M_16	8970	histone cluster 1 H2B family member j(HIST1H2BJ)
M_16	653604	histone cluster 2 H3 family member d(HIST2H3D)
M_16	3008	histone cluster 1 H1 family member e(HIST1H1E)
M_16	440689	histone cluster 2 H2B family member f(HIST2H2BF)
M_16	317772	histone cluster 2 H2A family member b(HIST2H2AB)
M_16	8337	histone cluster 2 H2A family member
M_16	8336	histone cluster 1 H2A family member
M_16	8335	histone cluster 1 H2A family member b(HIST1H2AB)
M_16	8334	histone cluster 1 H2A family member c(HIST1H2AC)
M_16	8332	histone cluster 1 H2A family member l(HIST1H2AL)
M_16	8331	histone cluster 1 H2A family member j(HIST1H2AJ)
M_16	8330	histone cluster 1 H2A family member k(HIST1H2AK)
M_16	7012	telomerase RNA component(TERC)
M_16	3006	histone cluster 1 H1 family member c(HIST1H1C)
M_16	8359	histone cluster 1 H4 family member a(HIST1H4A)
M_17	55892	myoneurin(MYNN)
M_17	152002	xyloside xylosyltransferase 1(XXYLT1)
M_17	23200	ATPase phospholipid transporting 11B
M_17	85476	G elongation factor mitochondrial 1(GFM1)

M_17	220729	succinate dehydrogenase complex flavoprotein subunit A pseudogene(LOC220729)
M_17	6747	signal sequence receptor subunit 3(SSR3)
M_17	84343	HPS3, biogenesis of lysosomal organelles complex 2 subunit 1(HPS3)
M_17	9718	endothelin converting enzyme 2(ECE2)
M_17	51726	DnaJ heat shock protein family (Hsp40) member B11(DNAJB11)
M_17	9711	RUN and cysteine rich domain containing beclin 1 interacting protein(RUBCN)
M_17	5130	phosphate cytidylyltransferase 1, choline,
M_17	80012	polyhomeotic homolog 3(PHC3)
M_17	9197	solute carrier family 33 member 1(SLC33A1)
M_17	6434	transformer 2 beta homolog (Drosophila)(TRA2B)
M_17	59343	SUMO1/sentrin/SMT3 specific peptidase 2(SENP2)
M_17	55341	large 60S subunit nuclear export GTPase 1(LSG1)
M_17	5984	replication factor C subunit 4(RFC4)
M_17	84223	IQ motif containing G(IQCG)
M_17	57560	intraflagellar transport 80(IFT80)
M_17	1181	chloride voltage-gated channel 2(CLCN2)
M_17	4711	NADH:ubiquinone oxidoreductase subunit
M_17	6801	striatin(STRN)
M_17	84517	actin related protein T3(ACTRT3)
M_17	5504	protein phosphatase 1 regulatory inhibitor subunit
M_17	5062	p21 (RAC1) activated kinase 2(PAK2)
M_17	1974	eukaryotic translation initiation factor 4A2(EIF4A2)
M_17	58477	SRP receptor beta subunit(SRPRB)
M_17	56922	methylcrotonoyl-CoA carboxylase 1(MCCC1)
M_17	5584	protein kinase C iota(PRKCI)
M_17	1857	dishevelled segment polarity protein 3(DVL3)
M_17	1739	discs large MAGUK scaffold protein 1(DLG1)
M_17	165918	ring finger protein 168(RNF168)

M_17	55186	solute carrier family 25 member 36(SLC25A36)
M_17	7095	SEC62 homolog, preprotein translocation
M_17	9616	ring finger protein 7(RNF7)
M_17	90407	transmembrane protein 41A(TMEM41A)
M_17	23350	U2 snRNP associated SURP domain
M_17	256356	glycerol kinase 5 (putative)(GK5)
M_17	5437	RNA polymerase II subunit H(POLR2H)
M_17	79572	ATPase 13A3(ATP13A3)
M_17	54464	5'-3' exoribonuclease 1(XRN1)
M_17	27230	stress associated endoplasmic reticulum protein
M_17	55324	ATP binding cassette subfamily F member 3(ABCF3)
M_17	84248	forty-two-three domain containing 1(FYTTD1)
M_17	4026	LIM domain containing preferred translocation partner in lipoma(LPP)
M_17	55689	YEATS domain containing 2(YEATS2)
M_17	116832	ribosomal protein L39 like(RPL39L)
M_17	79718	transducin beta like 1 X-linked receptor 1(TBL1XR1)
M_17	54165	defective in cullin neddylation 1 domain containing 1(DCUN1D1)
M_17	64778	fibronectin type III domain containing 3B(FNDC3B)
M_17	64110	MAGE family member F1(MAGEF1)
M_17	22916	nuclear cap binding protein subunit 2(NCBP2)
M_17	11235	programmed cell death 10(PDCD10)
M_17	1981	eukaryotic translation initiation factor 4 gamma
M_17	727956	succinate dehydrogenase complex flavoprotein subunit A pseudogene 2(SDHAP2)
M_17	8087	FMR1 autosomal homolog 1(FXR1)
M_17	152137	coiled-coil domain containing 50(CCDC50)
M_17	79929	MAP6 domain containing 1(MAP6D1)
M_17	86	actin like 6A(ACTL6A)
M_17	5708	proteasome 26S subunit, non-ATPase 2(PSMD2)

M_17	27094	potassium calcium-activated channel subfamily M regulatory beta subunit 3(KCNMB3)
M_17	11342	ring finger protein 13(RNF13)
M_17	131118	DnaJ heat shock protein family (Hsp40) member C19(DNAJC19)
M_17	51193	zinc finger protein 639(ZNF639)
M_17	54800	kelch like family member 24(KLHL24)
M_17	5291	phosphatidylinositol-4,5-bisphosphate 3-kinase catalytic subunit beta(PIK3CB)
M_17	5290	phosphatidylinositol-4,5-bisphosphate 3-kinase catalytic subunit alpha(PIK3CA)
M_17	51319	arginine and serine rich coiled-coil 1(RSRC1)
M_17	89782	leishmanolysin like peptidase(LMLN)
M_17	348793	WD repeat domain 53(WDR53)
M_17	23527	ArfGAP with coiled-coil, ankyrin repeat and PH domains 2(ACAP2)
M_17	23355	VPS8, CORVET complex subunit(VPS8)
M_17	4976	OPA1, mitochondrial dynamin like GTPase(OPA1)
M_17	93109	transmembrane protein 44(TMEM44)
M_17	6498	SKI like proto-oncogene(SKIL)
M_17	51714	selenoprotein T(SELENOT)
M_17	83939	eukaryotic translation initiation factor 2A(EIF2A)
M_17	622	3-hydroxybutyrate dehydrogenase, type 1(BDH1)
M_17	2992	glycogenin 1(GYG1)
M_17	255812	succinate dehydrogenase complex flavoprotein subunit A pseudogene 1(SDHAP1)
M_17	8893	eukaryotic translation initiation factor 2B subunit epsilon(EIF2B5)
M_17	253461	zinc finger and BTB domain containing 38(ZBTB38)
M_17	205564	SUMO1/sentrin specific peptidase 5(SENP5)
M_17	152217	NCBP2 antisense RNA 2 (head to head)(NCBP2-AS2)
M_17	55171	TBCC domain containing 1(TBCCD1)

M_17	131408	family with sequence similarity 131 member
M_17	26043	UBX domain protein 7(UBXN7)
M_17	10057	ATP binding cassette subfamily C member 5(ABCC5)
M_17	84984	centrosomal protein 19(CEP19)
M_17	84859	leucine rich repeats and calponin homology domain containing 3(LRCH3)
M_17	257313	urotensin 2B(UTS2B)
M_17	3840	karyopherin subunit alpha 4(KPNA4)
M_17	54965	phosphatidylinositol glycan anchor biosynthesis class
M_17	10188	tyrosine kinase non receptor 2(TNK2)
M_17	5966	REL proto-oncogene, NF-kB subunit(REL)
M_17	51068	NMD3 ribosome export adaptor(NMD3)
M_17	153743	protein phosphatase 1 regulatory inhibitor subunit 2 pseudogene 3(PPP1R2P3)
M_17	8706	beta-1,3-N-acetylgalactosaminyltransferase 1 (globoside blood group)(B3GALNT1)
M_17	7037	transferrin receptor(TFRC)
M_17	10195	ALG3, alpha-1,3- mannosyltransferase(ALG3)
M_17	4154	muscleblind like splicing regulator 1(MBNL1)
M_17	57018	cyclin L1(CCNL1)
M_17	170506	DEAH-box helicase 36(DHX36)
M_17	545	ATR serine/threonine kinase(ATR)
M_17	1173	adaptor related protein complex 2 mu 1
M_17	200933	F-box protein 45(FBXO45)
M_17	57129	mitochondrial ribosomal protein L47(MRPL47)
M_17	5922	RAS p21 protein activator 2(RASA2)
M_17	8833	guanine monophosphate synthase(GMPS)
M_17	55486	presenilin associated rhomboid like(PARL)
M_17	51122	COMM domain containing 2(COMMD2)
M_17	6596	helicase like transcription factor(HLTF)
M_17	55669	mitofusin 1(MFN1)
M_17	10051	structural maintenance of chromosomes 4(SMC4)

M_17	25976	TCDD inducible poly(ADP-ribose)
M_17	25937	WW domain containing transcription regulator
M_17	401093	MBNL1 antisense RNA 1(MBNL1-AS1)
M_17	2048	EPH receptor B2(EPHB2)
M_17	151613	tetratricopeptide repeat domain 14(TTC14)
M_17	4291	myeloid leukemia factor 1(MLF1)
M_17	85463	zinc finger CCCH-type containing 12C(ZC3H12C)
M_17	286827	tripartite motif containing 59(TRIM59)
M_17	645121	cyclin I family member 2(CCNI2)
M_18	55893	zinc finger protein 395(ZNF395)
M_18	23087	tripartite motif containing 35(TRIM35)
M_18	7419	voltage dependent anion channel 3(VDAC3)
M_18	59339	pleckstrin homology domain containing
M_18	54928	inositol monophosphatase domain containing
M_18	84376	hook microtubule tethering protein 3(HOOK3)
M_18	114926	small integral membrane protein 19(SMIM19)
M_18	84513	phospholipid phosphatase 5(PLPP5)
M_18	55290	BRF2, RNA polymerase III transcription initiation factor 50 kDa subunit(BRF2)
M_18	1978	eukaryotic translation initiation factor 4E binding protein 1(EIF4EBP1)
M_18	80185	TELO2 interacting protein 2(TTI2)
M_18	2961	general transcription factor IIE subunit 2(GTF2E2)
M_18	83877	TM2 domain containing 2(TM2D2)
M_18	23484	leptin receptor overlapping transcript-like
M_18	10671	dynactin subunit 6(DCTN6)
M_18	51669	store-operated calcium entry associated regulatory factor(SARAF)
M_18	51606	ATPase H+ transporting V1 subunit H(ATP6V1H)
M_18	2137	exostosin like glycosyltransferase 3(EXTL3)
M_18	55140	elongator acetyltransferase complex subunit 3(ELP3)
M_18	51125	golgin A7(GOLGA7)

M_18	55145	THAP domain containing 1(THAP1)
M_18	2936	glutathione-disulfide reductase(GSR)
M_18	81790	ring finger protein 170(RNF170)
M_18	6575	solute carrier family 20 member 2(SLC20A2)
M_18	137886	UBX domain protein 2B(UBXN2B)
M_18	137964	glycerol-3-phosphate acyltransferase 4(GPAT4)
M_18	137994	leucine zipper and EF-hand containing transmembrane protein 2(LETM2)
M_18	138050	heparan-alpha-glucosaminide N-
M_18	54904	Wolf-Hirschhorn syndrome candidate 1-like
M_18	84296	GINS complex subunit 4(GINS4)
M_18	9530	BCL2 associated athanogene 4(BAG4)
M_18	84549	MAK16 homolog(MAK16)
M_18	203102	ADAM metallopeptidase domain 32(ADAM32)
M_18	7486	Werner syndrome RecQ like helicase(WRN)
M_18	5423	DNA polymerase beta(POLB)
M_18	6917	transcription elongation factor A1(TCEA1)
M_18	9821	RB1 inducible coiled-coil 1(RB1CC1)
M_18	157574	F-box protein 16(FBXO16)
M_18	96764	trimethylguanosine synthase 1(TGS1)
M_18	80223	RAB11 family interacting protein 1(RAB11FIP1)
M_18	79618	homeobox containing 1(HMBOX1)
M_18	84750	fucosyltransferase 10(FUT10)
M_18	8439	neutral sphingomyelinase activation associated factor(NSMAF)
M_18	8754	ADAM metallopeptidase domain 9(ADAM9)
M_18	2053	epoxide hydrolase 2(EPHX2)
M_18	137695	transmembrane protein 68(TMEM68)
M_18	10947	adaptor related protein complex 3 mu 2
M_18	646405	transmembrane phosphoinositide 3-phosphatase and tensin homolog 2 pseudogene 1(TPTE2P1)
M_18	23259	DDHD domain containing 2(DDHD2)

M_18	55756	integrator complex subunit 9(INTS9)
M_18	27257	LSM1 homolog, mRNA degradation
M_18	339524	long intergenic non-protein coding RNA
M_18	5516	protein phosphatase 2 catalytic subunit beta(PPP2CB)
M_18	55246	coiled-coil domain containing 25(CCDC25)
M_18	79845	ring finger protein 122(RNF122)
M_18	11160	ER lipid raft associated 2(ERLIN2)
M_18	7976	frizzled class receptor 3(FZD3)
M_18	7993	UBX domain protein 8(UBXN8)
M_18	7994	lysine acetyltransferase 6A(KAT6A)
M_18	2339	farnesyltransferase, CAAX box, alpha(FNTA)
M_18	374491	transmembrane phosphoinositide 3-phosphatase and tensin homolog 2 pseudogene 6(TPTE2P6)
M_18	3551	inhibitor of kappa light polypeptide gene enhancer in B-cells, kinase beta(IKBKB)
M_18	9070	ASH2 like histone lysine methyltransferase complex subunit(ASH2L)
M_18	11212	proline synthetase cotranscribed homolog
M_18	5264	phytanoyl-CoA 2-hydroxylase(PHYH)
M_18	389649	chromosome 8 open reading frame 86(C8orf86)
M_18	2260	fibroblast growth factor receptor 1(FGFR1)
M_18	133308	solute carrier family 9 member B2(SLC9B2)
M_18	23491	carboxylesterase 3(CES3)
M_18	78986	dual specificity phosphatase 26 (putative)(DUSP26)
M_18	150677	otospiralin(OTOS)
M_18	8601	regulator of G-protein signaling 20(RGS20)
M_18	200558	aprataxin and PNKP like factor(APLF)
M_18	554251	F-box protein 48(FBXO48)
M_18	10434	lysophospholipase I(LYPLA1)
M_18	4846	nitric oxide synthase 3(NOS3)
M_18	23514	scaffolding protein involved in DNA repair(SPIDR)
M_18	29088	mitochondrial ribosomal protein L15(MRPL15)

M_18	132946	ADP ribosylation factor like GTPase 9(ARL9)
M_18	1409	crystallin alpha A(CRYAA)
M_18	56521	DnaJ heat shock protein family (Hsp40) member C12(DNAJC12)
M_19	55897	mesoderm posterior bHLH transcription factor
M_19	27124	inositol polyphosphate-5-phosphatase J(INPP5J)
M_19	7533	tyrosine 3-monooxygenase/tryptophan 5-monooxygenase activation protein eta(YWHAH)
M_19	200312	ring finger protein 215(RNF215)
M_19	253143	proline rich 14 like(PRR14L)
M_19	338809	chromosome 12 open reading frame 74(C12orf74)
M_19	154754	protease, serine 3 pseudogene 2(PRSS3P2)
M_19	24144	tuftelin interacting protein 11(TFIP11)
M_19	56478	eukaryotic translation initiation factor 4E nuclear import factor 1(EIF4ENIF1)
M_19	290	alanyl aminopeptidase, membrane(ANPEP)
M_19	120071	LARGE xylosyl- and glucuronyltransferase
M_19	249	alkaline phosphatase, liver/bone/kidney(ALPL)
M_19	4771	neurofibromin 2(NF2)
M_19	652968	GATS protein like 3(GATSL3)
M_19	83729	inhibin beta E subunit(INHBE)
M_19	79370	BCL2 like 14(BCL2L14)
M_19	80339	patatin like phospholipase domain containing
M_19	23598	POZ/BTB and AT hook containing zinc finger
M_19	89781	HPS4, biogenesis of lysosomal organelles complex 3 subunit 2(HPS4)
M_19	23481	pescadillo ribosomal biogenesis factor 1(PES1)
M_19	26291	fibroblast growth factor 21(FGF21)
M_19	83874	TBC1 domain family member 10A(TBC1D10A)
M_19	25807	rhomboid domain containing 3(RHBDD3)
M_19	4192	midkine (neurite growth-promoting factor 2)(MDK)
M_19	3985	LIM domain kinase 2(LIMK2)

M_19	91445	ring finger protein 185(RNF185)
M_19	6525	smoothelin(SMTN)
M_19	25793	F-box protein 7(FBXO7)
M_19	80020	FAD dependent oxidoreductase domain containing 2(FOXRED2)
M_19	51537	mitochondrial fission process 1(MTFP1)
M_19	6819	sulfotransferase family 1C member 2(SULT1C2)
M_19	79094	ChaC glutathione specific gamma-glutamylcyclotransferase 1(CHAC1)
M_19	9182	Ras association domain family member 9(RASSF9)
M_19	84164	activating signal cointegrator 1 complex subunit
M_19	8563	THO complex 5(THOC5)
M_19	402055	SRR1 domain containing(SRRD)
M_19	8508	nipsnap homolog 1 (C. elegans)(NIPSNAP1)
M_19	55613	myotubularin related protein 8(MTMR8)
M_19	3475	interferon related developmental regulator 1(IFRD1)
M_19	145873	mesoderm posterior bHLH transcription factor
M_19	2122	MDS1 and EVI1 complex locus(MECOM)
M_19	10042	HMG-box containing 4(HMGXB4)
M_19	142689	ankyrin repeat and SOCS box containing 12(ASB12)
M_19	4733	developmentally regulated GTP binding protein
M_19	140462	ankyrin repeat and SOCS box containing 9(ASB9)
M_19	23762	oxysterol binding protein 2(OSBP2)
M_19	4922	neurotensin(NTS)
M_19	55000	taurine up-regulated 1 (non-protein coding)(TUG1)
M_19	23761	phosphatidylserine decarboxylase(PISD)
M_19	23760	phosphatidylinositol transfer protein beta(PITPNB)
M_19	150291	MORC2 antisense RNA 1(MORC2-AS1)
M_19	9814	SFI1 centrin binding protein(SFI1)
M_19	9479	mitogen-activated protein kinase 8 interacting protein 1(MAPK8IP1)

M_19	29796	ubiquinol-cytochrome c reductase, complex III subunit X(UQCR10)
M_19	150275	coiled-coil domain containing 117(CCDC117)
M_19	440	asparagine synthetase (glutamine-hydrolyzing)(ASNS)
M_19	8897	myotubularin related protein 3(MTMR3)
M_19	6296	acyl-CoA synthetase medium-chain family member 3(ACSM3)
M_19	150290	dual specificity phosphatase 18(DUSP18)
M_19	54894	ring finger protein 43(RNF43)
M_19	51493	RNA 2',3'-cyclic phosphate and 5'-OH ligase(RTCB)
M_19	5646	protease, serine 3(PRSS3)
M_19	5644	protease, serine 1(PRSS1)
M_19	50487	phospholipase A2 group III(PLA2G3)
M_19	550631	coiled-coil domain containing 157(CCDC157)
M_19	440107	pleckstrin homology and RhoGEF domain containing G7(PLEKHG7)
M_19	10661	Kruppel like factor 1(KLF1)
M_19	55954	zinc finger matrin-type 5(ZMAT5)
M_19	23541	SEC14 like lipid binding 2(SEC14L2)
M_19	10291	splicing factor 3a subunit 1(SF3A1)
M_19	339665	solute carrier family 35 member E4(SLC35E4)
M_19	22880	MORC family CW-type zinc finger 2(MORC2)
M_19	2130	EWS RNA binding protein 1(EWSR1)
M_19	9681	DEP domain containing 5(DEPDC5)
M_19	11200	checkpoint kinase 2(CHEK2)
M_19	1649	DNA damage inducible transcript 3(DDIT3)
M_19	54682	MANSC domain containing 1(MANSC1)
M_19	56981	PR/SET domain 11(PRDM11)
M_19	4174	minichromosome maintenance complex component
M_19	57026	pyridoxal phosphatase(PDXP)
M_19	3177	solute carrier family 29 member 2(SLC29A2)
M_19	140883	zinc finger protein 280B(ZNF280B)

M_19	1152	creatine kinase B(CKB)
M_19	9633	testis expressed metallothionein like
M_19	84842	4-hydroxyphenylpyruvate dioxygenase like(HPDL)
M_19	744	metallophosphoesterase domain containing
M_19	113791	phosphoinositide-3-kinase interacting protein
M_19	7494	X-box binding protein 1(XBP1)
M_20	56101	protocadherin gamma subfamily B, 5(PCDHGB5)
M_20	56132	protocadherin beta 3(PCDHB3)
M_20	29930	protocadherin beta 1(PCDHB1)
M_20	84054	protocadherin beta 19 pseudogene(PCDHB19P)
M_20	56124	protocadherin beta 12(PCDHB12)
M_20	54661	protocadherin beta 17 pseudogene(PCDHB17P)
M_20	54660	protocadherin beta 18 pseudogene(PCDHB18P)
M_20	56129	protocadherin beta 7(PCDHB7)
M_20	56128	protocadherin beta 8(PCDHB8)
M_20	56104	protocadherin gamma subfamily B, 1(PCDHGB1)
M_20	56103	protocadherin gamma subfamily B, 2(PCDHGB2)
M_20	56123	protocadherin beta 13(PCDHB13)
M_20	56099	protocadherin gamma subfamily B, 7(PCDHGB7)
M_20	56121	protocadherin beta 15(PCDHB15)
M_20	56120	protocadherin gamma subfamily B, 8 pseudogene(PCDHGB8P)
M_20	56127	protocadherin beta 9(PCDHB9)
M_20	56126	protocadherin beta 10(PCDHB10)
M_20	56125	protocadherin beta 11(PCDHB11)
M_20	56097	protocadherin gamma subfamily C, 5(PCDHGC5)
M_20	56141	protocadherin alpha 7(PCDHA7)
M_20	56140	protocadherin alpha 8(PCDHA8)
M_20	56143	protocadherin alpha 5(PCDHA5)
M_20	56142	protocadherin alpha 6(PCDHA6)
M_20	56145	protocadherin alpha 3(PCDHA3)
M_20	56144	protocadherin alpha 4(PCDHA4)

M_20	56147	protocadherin alpha 1(PCDHA1)
M_20	56146	protocadherin alpha 2(PCDHA2)
M_20	8641	protocadherin gamma subfamily B, 4(PCDHGB4)
M_20	56098	protocadherin gamma subfamily C, 4(PCDHGC4)
M_20	56134	protocadherin alpha subfamily C, 2(PCDHAC2)
M_20	56135	protocadherin alpha subfamily C, 1(PCDHAC1)
M_20	56136	protocadherin alpha 13(PCDHA13)
M_20	56137	protocadherin alpha 12(PCDHA12)
M_20	56130	protocadherin beta 6(PCDHB6)
M_20	56131	protocadherin beta 4(PCDHB4)
M_20	9708	protocadherin gamma subfamily A, 8(PCDHGA8)
M_20	56133	protocadherin beta 2(PCDHB2)
M_20	56138	protocadherin alpha 11(PCDHA11)
M_20	56139	protocadherin alpha 10(PCDHA10)
M_20	5098	protocadherin gamma subfamily C, 3(PCDHGC3)
M_20	9752	protocadherin alpha 9(PCDHA9)
M_20	56113	protocadherin gamma subfamily A, 2(PCDHGA2)
M_20	56114	protocadherin gamma subfamily A, 1(PCDHGA1)
M_20	56111	protocadherin gamma subfamily A, 4(PCDHGA4)
M_20	56112	protocadherin gamma subfamily A, 3(PCDHGA3)
M_20	56109	protocadherin gamma subfamily A, 6(PCDHGA6)
M_20	56110	protocadherin gamma subfamily A, 5(PCDHGA5)
M_20	56108	protocadherin gamma subfamily A, 7(PCDHGA7)
M_20	56105	protocadherin gamma subfamily A, 11(PCDHGA11)
M_20	56106	protocadherin gamma subfamily A, 10(PCDHGA10)
M_20	56100	protocadherin gamma subfamily B, 6(PCDHGB6)
M_20	57717	protocadherin beta 16(PCDHB16)
M_20	56122	protocadherin beta 14(PCDHB14)
M_20	26167	protocadherin beta 5(PCDHB5)
M_20	56102	protocadherin gamma subfamily B, 3(PCDHGB3)
M_20	56107	protocadherin gamma subfamily A, 9(PCDHGA9)

M_21	1589	cytochrome P450 family 21 subfamily A member 2(CYP21A2)
M_21	440695	ETS variant 3 like(ETV3L)
M_21	100129424	ribosomal protein L19 pseudogene 12(RPL19P12)
M_21	5383	PMS1 homolog 2, mismatch repair system component pseudogene 5(PMS2P5)
M_21	10224	zinc finger protein 443(ZNF443)
M_21	653162	ribosomal protein SA pseudogene 9(RPSAP9)
M_21	222901	ribosomal protein L23 pseudogene 8(RPL23P8)
M_21	100272216	#N/A
M_21	343477	heat shock protein 90 beta family member 3, pseudogene(HSP90B3P)
M_21	83955	nascent polypeptide associated complex alpha subunit pseudogene 1(NACAP1)
M_21	8843	hydroxycarboxylic acid receptor 3(HCAR3)
M_21	5379	PMS1 homolog 2, mismatch repair system component pseudogene 1(PMS2P1)
M_21	649330	heterogeneous nuclear ribonucleoprotein C-like 3(HNRNPCL3)
M_21	161142	family with sequence similarity 71 member
M_21	305	annexin A2 pseudogene 3(ANXA2P3)
M_21	647033	proliferation-associated 2G4 pseudogene 4(PA2G4P4)
M_21	338442	hydroxycarboxylic acid receptor 2(HCAR2)
M_21	100130331	POTE ankyrin domain family, member F pseudogene(LOC100130331)
M_21	441251	speedy/RINGO cell cycle regulator family member E7, pseudogene(SPDYE7P)
M_21	6129	ribosomal protein L7(RPL7)
M_21	10901	dehydrogenase/reductase 4(DHRS4)
M_21	1564	cytochrome P450 family 2 subfamily D member 7 (gene/pseudogene)(CYP2D7)
M_21	2171	fatty acid binding protein 5(FABP5)

M_21	440359	Y-box binding protein 3 pseudogene 1(YBX3P1)
M_21	720	complement C4A (Rodgers blood group)(C4A)
M_21	10965	acyl-CoA thioesterase 2(ACOT2)
M_21	2629	glucosylceramidase beta(GBA)
M_21	3304	heat shock protein family A (Hsp70) member
M_21	3303	heat shock protein family A (Hsp70) member
M_21	445582	POTE ankyrin domain family member E(POTEE)
M_21	641455	POTE ankyrin domain family member M(POTEM)
M_21	100132832	PMS1 homolog 2, mismatch repair system component pseudogene 9(PMS2P9)
M_21	303	annexin A2 pseudogene 1(ANXA2P1)
M_21	644511	ribosomal protein L13a pseudogene 6(RPL13AP6)
M_21	342538	nascent polypeptide associated complex alpha subunit 2(NACA2)
M_21	441818	WW domain binding protein 11 pseudogene
M_21	347376	H3 histone, family 3A, pseudogene 5(H3F3AP5)
M_21	90576	zinc finger protein 799(ZNF799)
M_21	728378	POTE ankyrin domain family member F(POTEF)
M_21	641371	acyl-CoA thioesterase 1(ACOT1)
M_21	158381	ATPase phospholipid transporting 8B5, pseudogene(ATP8B5P)
M_21	8531	Y-box binding protein 3(YBX3)
M_21	728635	dehydrogenase/reductase 4 like 1(DHRS4L1)
M_21	2630	glucosylceramidase beta pseudogene 1(GBAP1)
M_21	392490	TPT1-like protein(FLJ44635)
M_21	441032	eukaryotic translation elongation factor 1 alpha 1 pseudogene 9(EEF1A1P9)
M_21	150786	RAB6C-like(WTH3DI)
M_21	26255	pituitary tumor-transforming 3, pseudogene(PTTG3P)
M_21	317749	dehydrogenase/reductase 4 like 2(DHRS4L2)
M_21	337875	histone cluster 2 H2B family member a (pseudogene)(HIST2H2BA)

M_21	4580	metaxin 1(MTX1)
M_21	442454	ubiquinol-cytochrome c reductase binding protein pseudogene 1(UQCRBP1)
M_21	643387	TAR DNA binding protein pseudogene(LOC643387)
M_21	441272	speedy/RINGO cell cycle regulator family member E3(SPDYE3)
M_21	339047	#N/A
M_21	9284	nuclear pore complex interacting protein family member A1(NPIPA1)
M_21	6139	ribosomal protein L17(RPL17)
M_21	55073	leucine rich repeat containing 37 member A4, pseudogene(LRRC37A4P)
M_21	1915	eukaryotic translation elongation factor 1 alpha
M_21	387841	ribosomal protein L13a pseudogene 20(RPL13AP20)
M_21	286140	ring finger protein 5 pseudogene 1(RNF5P1)
M_21	55138	family with sequence similarity 90 member
M_21	441317	putative protein FAM90A7(FAM90A7P)
M_21	604	B-cell CLL/lymphoma 6(BCL6)
M_21	55889	golgin A6 family member B(GOLGA6B)
M_21	342096	golgin A6 family member A(GOLGA6A)
M_21	653641	golgin A6 family member C(GOLGA6C)
M_22	1607	diacylglycerol kinase beta(DGKB)
M_22	158038	leucine rich repeat and Ig domain containing
M_22	285220	EPH receptor A6(EPHA6)
M_22	204962	solute carrier family 44 member 5(SLC44A5)
M_22	392636	alkylglycerol monooxygenase(AGMO)
M_22	280657	SSX family member 6, pseudogene(SSX6)
M_22	90827	zinc finger protein 479(ZNF479)
M_22	158983	H2B histone family member W, testis
M_22	10281	Down syndrome critical region 4(DSCR4)
M_22	2792	G protein subunit gamma transducin 1(GNGT1)
M_22	10178	teneurin transmembrane protein 1(TENM1)

M_22	728269	MAGE family member A9B(MAGEA9B)
M_22	85285	keratin associated protein 4-1(KRTAP4-1)
M_22	7681	makorin ring finger protein 3(MKRN3)
M_22	5314	polycystic kidney and hepatic disease 1 (autosomal recessive)(PKHD1)
M_22	441234	zinc finger protein 716(ZNF716)
M_22	344875	collagen type VI alpha 4 pseudogene 1(COL6A4P1)
M_22	53942	contactin 5(CNTN5)
M_22	57512	G protein-coupled receptor 158(GPR158)
M_22	374470	chromosome 12 open reading frame 42(C12orf42)
M_22	1129	cholinergic receptor muscarinic 2(CHRM2)
M_22	1131	cholinergic receptor muscarinic 3(CHRM3)
M_22	27004	T-cell leukemia/lymphoma 6 (non-protein
M_22	11281	POU class 6 homeobox 2(POU6F2)
M_22	256764	WD repeat domain 72(WDR72)
M_22	3358	5-hydroxytryptamine receptor 2C(HTR2C)
M_22	154215	Na+/K+ transporting ATPase interacting 2(NKAIN2)
M_22	347365	inter-alpha-trypsin inhibitor heavy chain family member 6(ITIH6)
M_22	2262	glypican 5(GPC5)
M_22	441521	cancer/testis antigen family 45 member A5(CT45A5)
M_22	81623	defensin beta 126(DEFB126)
M_22	133482	solute carrier organic anion transporter family member 6A1(SLCO6A1)
M_22	728911	cancer/testis antigen family 45 member A2(CT45A2)
M_22	168417	zinc finger protein 679(ZNF679)
M_22	5321	phospholipase A2 group IVA(PLA2G4A)
M_22	1361	carboxypeptidase B2(CPB2)
M_22	2556	gamma-aminobutyric acid type A receptor alpha3 subunit(GABRA3)
M_22	6759	SSX family member 4(SSX4)
M_22	4110	MAGE family member A11(MAGEA11)

M_22	55530	SV2 related protein(SVOP)
M_22	56651	long intergenic non-protein coding RNA
M_22	10941	UDP glucuronosyltransferase family 2 member A1 complex locus(UGT2A1)
M_22	4103	MAGE family member A4(MAGEA4)
M_22	6579	solute carrier organic anion transporter family member 1A2(SLCO1A2)
M_22	4109	MAGE family member A10(MAGEA10)
M_22	84677	Down syndrome critical region 8(DSCR8)
M_22	441520	#N/A
M_22	190	nuclear receptor subfamily 0 group B member
M_22	29953	thyrotropin releasing hormone degrading
M_22	9623	T-cell leukemia/lymphoma 1B(TCL1B)
M_22	153910	uncharacterized LOC153910(LOC153910)
M_22	283392	TRHDE antisense RNA 1(TRHDE-AS1)
M_22	286436	H2B histone family member M(H2BFM)
M_22	51557	lengsin, lens protein with glutamine synthetase
M_22	54714	cyclic nucleotide gated channel beta 3(CNGB3)
M_22	132430	poly(A) binding protein cytoplasmic 4
M_22	2566	gamma-aminobutyric acid type A receptor gamma2 subunit(GABRG2)
M_22	130399	activin A receptor type 1C(ACVR1C)
M_22	2569	gamma-aminobutyric acid type A receptor rho1 subunit(GABRR1)
M_22	26609	variable charge, X-linked(VCX)
M_22	147945	NLR family pyrin domain containing 4(NLRP4)
M_22	51481	variable charge, X-linked 3A(VCX3A)
M_22	56979	PR/SET domain 9(PRDM9)
M_22	152404	immunoglobulin superfamily member 11(IGSF11)
M_22	641516	keratoconus gene 6(KC6)
M_22	80070	ADAM metallopeptidase with thrombospondin type 1 motif 20(ADAMTS20)

M_22	647121	embigin pseudogene 1(EMBP1)
M_22	8708	beta-1,3-galactosyltransferase 1(B3GALT1)
M_22	5726	taste 2 receptor member 38(TAS2R38)
M_22	643955	zinc finger protein 733, pseudogene(ZNF733P)
M_22	57575	protocadherin 10(PCDH10)
M_22	66037	boule homolog, RNA binding protein(BOLL)
M_22	56884	follistatin like 5(FSTL5)
M_22	5101	protocadherin 9(PCDH9)
M_22	10345	triadin(TRDN)
M_22	57554	leucine rich repeat containing 7(LRRC7)
M_22	4703	nebulin(NEB)
M_22	7399	usherin(USH2A)
M_22	11005	serine peptidase inhibitor, Kazal type 5(SPINK5)
M_22	26050	SLIT and NTRK like family member 5(SLITRK5)
M_22	159963	solute carrier family 5 member 12(SLC5A12)
M_22	541466	cancer/testis antigen family 45 member A1(CT45A1)
M_22	56164	serine/threonine kinase 31(STK31)
M_22	132954	phosducin like 2(PDCL2)
M_22	9312	potassium voltage-gated channel subfamily B member 2(KCNB2)
M_22	1630	DCC netrin 1 receptor(DCC)
M_22	83604	transmembrane protein 47(TMEM47)
M_22	152024	long intergenic non-protein coding RNA
M_22	222223	KIAA1324 like(KIAA1324L)
M_22	284124	uncharacterized FLJ36000(FLJ36000)
M_22	30010	neurexophilin 1(NXPH1)
M_22	5967	regenerating family member 1 alpha(REG1A)
M_22	3792	Kell blood group, metallo-endopeptidase(KEL)
M_22	730291	zinc finger protein 735(ZNF735)
M_22	55511	sarcoma antigen 1(SAGE1)
M_22	25945	nectin cell adhesion molecule 3(NECTIN3)
M_22	9153	solute carrier family 28 member 2(SLC28A2)

M_22	220082	spermatid associated(SPERT)
M_22	2329	flavin containing monooxygenase 4(FMO4)
M_22	4100	MAGE family member A1(MAGEA1)
M_22	5896	recombination activating 1(RAG1)
M_22	11148	HERV-H LTR-associating 2(HHLA2)
M_22	56667	mucin 13, cell surface associated(MUC13)
M_22	2913	glutamate metabotropic receptor 3(GRM3)
M_22	85417	cyclin B3(CCNB3)
M_22	135927	chromosome 7 open reading frame 34(C7orf34)
M_22	441519	cancer/testis antigen family 45 member A3(CT45A3)
M_22	117156	secretoglobin family 3A member 2(SCGB3A2)
M_22	390442	olfactory receptor family 11 subfamily H member
M_22	6857	synaptotagmin 1(SYT1)
M_22	341208	hephaestin like 1(HEPHL1)
M_22	404744	NPSR1 antisense RNA 1(NPSR1-AS1)
M_22	204801	NLR family pyrin domain containing 11(NLRP11)
M_22	1006	cadherin 8(CDH8)
M_22	122748	olfactory receptor family 11 subfamily H member
M_22	5544	proline rich protein BstNI subfamily 3(PRB3)
M_22	84059	adhesion G protein-coupled receptor V1(ADGRV1)
M_22	84700	myosin XVIIIB(MYO18B)
M_22	121278	tryptophan hydroxylase 2(TPH2)
M_22	23784	POTE ankyrin domain family member H(POTEH)
M_22	348825	tetrapeptide repeat homeobox like(TPRXL)
M_22	6326	sodium voltage-gated channel alpha subunit
M_22	138046	RALY RNA binding protein-like(RALYL)
M_22	152405	chromosome 3 open reading frame 30(C3orf30)
M_22	53833	interleukin 20 receptor subunit beta(IL20RB)
M_22	221938	monocyte to macrophage differentiation associated
M_22	4286	melanogenesis associated transcription factor(MITF)
M_22	146723	chromosome 17 open reading frame 77(C17orf77)

M_22	2974	guanylate cyclase 1 soluble subunit beta 2 (pseudogene)(GUCY1B2)
M_22	80319	CXXC finger protein 4(CXXC4)
M_22	114805	polypeptide N-acetylgalactosaminyltransferase
M_22	152015	rhophilin associated tail protein 1B(ROPN1B)
M_22	80059	leucine rich repeat transmembrane neuronal
M_22	425054	variable charge, X-linked 3B(VCX3B)
M_22	283025	long intergenic non-protein coding RNA
M_22	4104	MAGE family member A5(MAGEA5)
M_22	1948	ephrin B2(EFNB2)
M_22	65217	protocadherin related 15(PCDH15)
M_22	440905	fatty acyl-CoA reductase 2 pseudogene 1(FAR2P1)
M_22	404785	POTE ankyrin domain family member G(POTEG)
M_22	92	activin A receptor type 2A(ACVR2A)
M_22	56302	transient receptor potential cation channel subfamily V member 5(TRPV5)
M_22	158833	acyl-CoA wax alcohol acyltransferase 1(AWAT1)
M_22	129684	contactin associated protein like 5(CNTNAP5)
M_22	200810	ALG1, chitobiosyldiphosphodolichol beta-mannosyltransferase like(ALG1L)
M_22	115111	solute carrier family 26 member 7(SLC26A7)
M_22	255631	collagen type XXIV alpha 1 chain(COL24A1)
M_22	135924	olfactory receptor family 9 subfamily A member
M_22	339010	#N/A
M_22	388468	POTE ankyrin domain family member C(POTEC)
M_22	23284	adhesion G protein-coupled receptor L3(ADGRL3)
M_22	27033	zinc finger and BTB domain containing 32(ZBTB32)
M_22	23266	adhesion G protein-coupled receptor L2(ADGRL2)
M_22	346517	olfactory receptor family 6 subfamily V member
M_22	1016	cadherin 18(CDH18)
M_22	440153	olfactory receptor family 11 subfamily H member 12(OR11H12)

M_22	10877	complement factor H related 4(CFHR4)
M_22	10878	complement factor H related 3(CFHR3)
M_22	22871	neuroligin 1(NLGN1)
M_22	646243	coxsackie virus and adenovirus receptor pseudogene 2(CXADRP2)
M_22	8456	forkhead box N1(FOXN1)
M_22	5746	parathyroid hormone 2 receptor(PTH2R)
M_22	377047	protease, serine 45(PRSS45)
M_22	317754	POTE ankyrin domain family member D(POTED)
M_22	6328	sodium voltage-gated channel alpha subunit
M_22	100132708	cytochrome P450 family 4 subfamily F member 30, pseudogene(CYP4F30P)
M_22	54763	rhophilin associated tail protein 1(ROPN1)
M_22	223117	semaphorin 3D(SEMA3D)
M_22	768206	progressive rod-cone degeneration(PRCD)
M_22	115749	chromosome 12 open reading frame 56(C12orf56)
M_22	153201	solute carrier family 36 member 2(SLC36A2)
M_22	440224	coxsackie virus and adenovirus receptor pseudogene 3(CXADRP3)
M_22	29951	PDZ domain containing ring finger 4(PDZRN4)
M_22	644936	actin, beta pseudogene(LOC644936)
M_22	11245	G protein-coupled receptor 176(GPR176)
M_22	84654	spermatogenic leucine zipper 1(SPZ1)
M_23	1639	dynactin subunit 1(DCTN1)
M_23	84186	zinc finger CCHC-type containing 7(ZCCHC7)
M_23	25853	DDB1 and CUL4 associated factor 12(DCAF12)
M_23	51271	ubiquitin associated protein 1(UBAP1)
M_23	9925	zinc finger and BTB domain containing 5(ZBTB5)
M_23	10280	sigma non-opioid intracellular receptor 1(SIGMAR1)
M_23	23177	centrosomal protein 68(CEP68)
M_23	9827	RGP1 homolog, RAB6A GEF complex partner
M_23	54980	chromosome 2 open reading frame 42(C2orf42)

M_23	9380	glyoxylate and hydroxypyruvate reductase(GRHPR)
M_23	392307	family with sequence similarity 221 member
M_23	65083	nucleolar protein 6(NOL6)
M_23	10322	SMYD family member 5(SMYD5)
M_23	26267	F-box protein 10(FBXO10)
M_23	203259	family with sequence similarity 219 member
M_23	203260	coiled-coil domain containing 107(CCDC107)
M_23	1211	clathrin light chain A(CLTA)
M_23	30968	stomatin like 2(STOML2)
M_23	2592	galactose-1-phosphate uridylyltransferase(GALT)
M_23	64425	RNA polymerase I subunit E(POLR1E)
M_23	55833	ubiquitin associated protein 2(UBAP2)
M_23	55234	DNA replication regulator and spliceosomal
M_23	57704	glucosylceramidase beta 2(GBA2)
M_23	400961	poly(A) binding protein interacting protein
M_23	3301	DnaJ heat shock protein family (Hsp40) member A1(DNAJA1)
M_23	79269	DDB1 and CUL4 associated factor 10(DCAF10)
M_23	4799	nuclear transcription factor, X-box binding 1(NFX1)
M_23	138716	ribonuclease P/MRP subunit p25 like(RPP25L)
M_23	51010	exosome component 3(EXOSC3)
M_23	692094	microseminoprotein, prostate associated(MSMP)
M_23	7094	talin 1(TLN1)
M_23	54926	ubiquitin conjugating enzyme E2 R2(UBE2R2)
M_23	152006	ring finger protein 38(RNF38)
M_23	158234	tRNA methyltransferase 10B(TRMT10B)
M_23	11258	dynactin subunit 3(DCTN3)
M_23	92014	solute carrier family 25 member 51(SLC25A51)
M_23	7415	valosin containing protein(VCP)
M_23	10210	TOP1 binding arginine/serine rich protein(TOPORS)
M_23	126147	netrin 5(NTN5)
M_23	54840	aprataxin(APTX)

M_23	7016	testis-specific kinase 1(TESK1)
M_23	92973	long intergenic non-protein coding RNA
M_23	51754	transmembrane protein 8B(TMEM8B)
M_23	84720	phosphatidylinositol glycan anchor biosynthesis class
M_23	64427	tetratricopeptide repeat domain 31(TTC31)
M_23	9853	RUN and SH3 domain containing 2(RUSC2)
M_23	392309	olfactory receptor family 13 subfamily J member
M_23	84460	zinc finger matrin-type 1(ZMAT1)
M_23	10497	unc-13 homolog B(UNC13B)
M_23	2189	Fanconi anemia complementation group G(FANCG)
M_23	80256	family with sequence similarity 214 member
M_23	10488	cAMP responsive element binding protein 3(CREB3)
M_23	653677	secretory blood group 1, pseudogene(SEC1P)
M_23	573	BCL2 associated athanogene 1(BAG1)
M_23	51510	charged multivesicular body protein 5(CHMP5)
M_23	219	aldehyde dehydrogenase 1 family member
M_23	84865	coiled-coil domain containing 142(CCDC142)
M_23	58476	tumor protein p53 inducible nuclear protein
M_23	347240	kinesin family member 24(KIF24)
M_23	84681	histidine triad nucleotide binding protein 2(HINT2)
M_23	401505	translocase of outer mitochondrial membrane
M_23	318	nudix hydrolase 2(NUDT2)
M_23	441454	prothymosin, alpha pseudogene(LOC441454)
M_23	6242	rhotekin(RTKN)
M_23	84904	Rho guanine nucleotide exchange factor
M_23	48	aconitase 1(ACO1)
M_23	144455	E2F transcription factor 7(E2F7)
M_23	6461	SH2 domain containing adaptor protein B(SHB)
M_23	6512	solute carrier family 1 member 7(SLC1A7)
M_23	158401	chromosome 9 open reading frame 84(C9orf84)
M_23	4712	NADH:ubiquinone oxidoreductase subunit
M_23	7841	mannosyl-oligosaccharide glucosidase(MOGS)

M_23	402176	ribosomal protein L21 pseudogene 44(RPL21P44)
M_23	26045	leucine rich repeat transmembrane neuronal
M_24	60673	autophagy related 101(ATG101)
M_24	117177	RAB3A interacting protein(RAB3IP)
M_24	79022	transmembrane protein 106C(TMEM106C)
M_24	6302	tetraspanin 31(TSPAN31)
M_24	84975	major facilitator superfamily domain containing
M_24	2799	glucosamine (N-acetyl)-6-sulfatase(GNS)
M_24	4193	MDM2 proto-oncogene(MDM2)
M_24	10540	dynactin subunit 2(DCTN2)
M_24	22818	coatomer protein complex subunit zeta 1(COPZ1)
M_24	79837	phosphatidylinositol-5-phosphate 4-kinase type 2 gamma(PIP4K2C)
M_24	57122	nucleoporin 107(NUP107)
M_24	55832	cullin associated and neddylation dissociated
M_24	144233	BCDIN3 domain containing RNA methyltransferase(BCDIN3D)
M_24	83448	pseudouridylate synthase 7 like(PUS7L)
M_24	55266	transmembrane protein 19(TMEM19)
M_24	10329	transmembrane protein 5(TMEM5)
M_24	10330	canopy FGF signaling regulator 2(CNPY2)
M_24	11052	cleavage and polyadenylation specific factor
M_24	967	CD63 molecule(CD63)
M_24	7024	transcription factor CP2(TFCP2)
M_24	6895	TARBP2, RISC loading complex RNA binding subunit(TARBP2)
M_24	5756	twinfilin actin binding protein 1(TWF1)
M_24	5571	protein kinase AMP-activated non-catalytic subunit gamma 1(PRKAG1)
M_24	1019	cyclin dependent kinase 4(CDK4)
M_24	144438	#N/A
M_24	3856	keratin 8(KRT8)

M_24	85437	zinc finger CCHC-type and RNA binding motif containing 1(ZCRB1)
M_24	91419	ATP23 metallopeptidase and ATP synthase assembly factor homolog(ATP23)
M_24	140461	ankyrin repeat and SOCS box containing 8(ASB8)
M_24	113251	La ribonucleoprotein domain family member
M_24	84324	SAP domain containing ribonucleoprotein(SARNP)
M_24	9802	DAZ associated protein 2(DAZAP2)
M_24	23344	extended synaptotagmin 1(ESYT1)
M_24	283464	glucoside xylosyltransferase 1(GXYLT1)
M_24	54458	proline rich 13(PRR13)
M_24	51535	periphilin 1(PPHLN1)
M_24	4891	solute carrier family 11 member 2(SLC11A2)
M_24	10576	chaperonin containing TCP1 subunit 2(CCT2)
M_24	9416	DEAD-box helicase 23(DDX23)
M_24	9169	SR-related CTD associated factor 11(SCAF11)
M_24	6821	sulfite oxidase(SUOX)
M_24	83591	THAP domain containing 2(THAP2)
M_24	4637	myosin light chain 6(MYL6)
M_24	23011	RAB21, member RAS oncogene family(RAB21)
M_24	144577	chromosome 12 open reading frame 66(C12orf66)
M_24	5869	RAB5B, member RAS oncogene family(RAB5B)
M_24	29095	ORMDL sphingolipid biosynthesis regulator
M_24	84298	LLP homolog, long-term synaptic facilitation(LLPH)
M_24	8086	aladin WD repeat nucleoporin(AAAS)
M_24	65244	spermatogenesis associated serine rich 2(SPATS2)
M_24	8089	YEATS domain containing 4(YEATS4)
M_24	84872	zinc finger CCCH-type containing 10(ZC3H10)
M_24	79035	nucleic acid binding protein 2(NABP2)
M_24	3875	keratin 18(KRT18)
M_24	23583	single-strand-selective monofunctional uracil-DNA glycosylase 1(SMUG1)

M_24	10138	YY1 associated factor 2(YAF2)
M_24	377	ADP ribosylation factor 3(ARF3)
M_24	91012	ceramide synthase 5(CERS5)
M_24	51135	interleukin 1 receptor associated kinase 4(IRAK4)
M_24	283377	SPRY domain containing 4(SPRYD4)
M_24	9958	ubiquitin specific peptidase 15(USP15)
M_24	4141	methionyl-tRNA synthetase(MARS)
M_24	5094	poly(rC) binding protein 2(PCBP2)
M_24	92797	DNA helicase B(HELB)
M_24	79657	RNA polymerase II associated protein 3(RPAP3)
M_24	8445	dual specificity tyrosine phosphorylation regulated kinase 2(DYRK2)
M_24	54934	KAT8 regulatory NSL complex subunit 2(KANSL2)
M_24	5036	proliferation-associated 2G4(PA2G4)
M_24	10728	prostaglandin E synthase 3(PTGES3)
M_24	64786	TBC1 domain family member 15(TBC1D15)
M_24	81566	cysteine and serine rich nuclear protein 2(CSRNP2)
M_24	1431	citrate synthase(CS)
M_24	466	activating transcription factor 1(ATF1)
M_24	10818	fibroblast growth factor receptor substrate 2(FRS2)
M_24	283349	Ras association domain family member 3(RASSF3)
M_24	84987	COX14, cytochrome c oxidase assembly
M_24	140465	myosin light chain 6B(MYL6B)
M_24	29110	TANK binding kinase 1(TBK1)
M_24	4848	CCR4-NOT transcription complex subunit 2(CNOT2)
M_24	23592	LEM domain containing 3(LEMD3)
M_24	11260	exportin for tRNA(XPOT)
M_24	4234	methyltransferase like 1(METTL1)
M_24	51643	transmembrane BAX inhibitor motif containing
M_24	60314	chromosome 12 open reading frame 10(C12orf10)
M_24	10102	Ts translation elongation factor,
M_24	7009	transmembrane BAX inhibitor motif containing

M_24	29843	SUMO1/sentrin specific peptidase 1(SENP1)
M_24	196528	AT-rich interaction domain 2(ARID2)
M_24	5908	RAP1B, member of RAS oncogene family(RAP1B)
M_24	84216	transmembrane protein 117(TMEM117)
M_24	10445	microspherule protein 1(MCRS1)
M_24	84305	PYM homolog 1, exon junction complex associated factor(PYM1)
M_24	56890	Mdm1 nuclear protein(MDM1)
M_24	506	ATP synthase, H+ transporting, mitochondrial F1 complex, beta polypeptide(ATP5B)
M_24	10193	ring finger protein 41(RNF41)
M_24	55508	solute carrier family 35 member E3(SLC35E3)
M_24	196441	zinc finger C3H1-type containing(ZFC3H1)
M_24	27165	glutaminase 2(GLS2)
M_24	10376	tubulin alpha 1b(TUBA1B)
M_24	6472	serine hydroxymethyltransferase 2(SHMT2)
M_24	784	calcium voltage-gated channel auxiliary subunit beta 3(CACNB3)
M_24	57460	protein phosphatase, Mg2+/Mn2+ dependent
M_24	26227	phosphoglycerate dehydrogenase(PHGDH)
M_24	51054	pleckstrin homology domain containing A8 pseudogene 1(PLEKHA8P1)
M_24	196527	anoctamin 6(ANO6)
M_24	11226	polypeptide N-acetylgalactosaminyltransferase
M_24	92979	membrane associated ring-CH-type finger
M_24	93058	coenzyme Q10A(COQ10A)
M_24	5947	retinol binding protein 1(RBP1)
M_24	54407	solute carrier family 38 member 2(SLC38A2)
M_24	81539	solute carrier family 38 member 1(SLC38A1)
M_24	5213	phosphofructokinase, muscle(PFKM)
M_24	4665	NGFI-A binding protein 2(NAB2)
M_24	84790	tubulin alpha 1c(TUBA1C)

M_25	1750	distal-less homeobox 6(DLX6)
M_25	1749	distal-less homeobox 5(DLX5)
M_25	54541	DNA damage inducible transcript 4(DDIT4)
M_25	27022	forkhead box D3(FOXD3)
M_25	3651	pancreatic and duodenal homeobox 1(PDX1)
M_25	1045	caudal type homeobox 2(CDX2)
M_25	6913	T-box 15(TBX15)
M_25	3198	homeobox A1(HOXA1)
M_25	154761	family with sequence similarity 115, member C pseudogene(LOC154761)
M_25	3200	homeobox A3(HOXA3)
M_25	3201	homeobox A4(HOXA4)
M_25	3202	homeobox A5(HOXA5)
M_25	3203	homeobox A6(HOXA6)
M_25	3204	homeobox A7(HOXA7)
M_25	3205	homeobox A9(HOXA9)
M_25	30012	T-cell leukemia homeobox 3(TLX3)
M_25	3206	homeobox A10(HOXA10)
M_25	3207	homeobox A11(HOXA11)
M_25	3209	homeobox A13(HOXA13)
M_25	57057	T-box 20(TBX20)
M_25	2297	forkhead box D1(FOXD1)
M_25	51299	neuritin 1(NRN1)
M_25	2774	G protein subunit alpha L(GNAL)
M_25	3229	homeobox C13(HOXC13)
M_25	63950	DMRT like family A2(DMRTA2)
M_25	360205	prostate cancer susceptibility candidate 2(PRAC2)
M_25	1761	doublesex and mab-3 related transcription factor
M_25	2306	forkhead box D2(FOXD2)
M_25	171586	abhydrolase domain containing 3(ABHD3)
M_25	3199	homeobox A2(HOXA2)
M_25	84793	FOXD2 antisense RNA 1 (head to head)(FOXD2-

M_25	58524	doublesex and mab-3 related transcription factor
M_25	3223	homeobox C6(HOXC6)
M_25	133	adrenomedullin(ADM)
M_25	10716	T-box, brain 1(TBR1)
M_25	51129	angiopoietin like 4(ANGPTL4)
M_25	388662	solute carrier family 6 member 17(SLC6A17)
M_25	100124700	HOX transcript antisense RNA(HOTAIR)
M_25	8935	src kinase associated phosphoprotein 2(SKAP2)
M_25	3226	homeobox C10(HOXC10)
M_25	3225	homeobox C9(HOXC9)
M_25	3224	homeobox C8(HOXC8)
M_25	10481	homeobox B13(HOXB13)
M_25	4488	msh homeobox 2(MSX2)
M_25	3227	homeobox C11(HOXC11)
M_25	285966	TRPM8 channel associated factor 2(TCAF2)
M_25	285987	DLX6 antisense RNA 1(DLX6-AS1)
M_25	80328	UL16 binding protein 2(ULBP2)
M_25	221883	HOXA11 antisense RNA(HOXA11-AS)
M_25	114787	G protein regulated inducer of neurite outgrowth
M_25	6338	sodium channel epithelial 1 beta subunit(SCNN1B)
M_25	6340	sodium channel epithelial 1 gamma subunit(SCNN1G)
M_25	9568	gamma-aminobutyric acid type B receptor subunit 2(GABBR2)
M_25	147495	APC down-regulated 1(APCDD1)
M_25	5457	POU class 4 homeobox 1(POU4F1)
M_25	343472	BarH like homeobox 2(BARHL2)
M_25	144195	solute carrier family 2 member 14(SLC2A14)
M_25	64711	heparan sulfate-glucosamine 3-sulfotransferase
M_25	59336	PR/SET domain 13(PRDM13)
M_25	10655	doublesex and mab-3 related transcription factor
M_25	84959	ubiquitin associated and SH3 domain containing B(UBASH3B)

M_25	1780	dynein cytoplasmic 1 intermediate chain 1(DYNC1I1)
M_25	112399	egl-9 family hypoxia inducible factor 3(EGLN3)
M_25	687	Kruppel like factor 9(KLF9)
M_25	260434	pyrin domain containing 1(PYDC1)
M_25	6781	stanniocalcin 1(STC1)
M_25	479	ATPase H+/K+ transporting non-gastric alpha2 subunit(ATP12A)
M_25	29091	syntaxin binding protein 6(STXBP6)
M_25	2300	forkhead box L1(FOXL1)
M_25	221833	Sp8 transcription factor(SP8)
M_25	51278	immediate early response 5(IER5)
M_25	83850	extended synaptotagmin 3(ESYT3)
M_25	5165	pyruvate dehydrogenase kinase 3(PDK3)
M_25	254528	meiosis specific with OB domains(MEIOB)
M_25	5731	prostaglandin E receptor 1(PTGER1)
M_25	55867	solute carrier family 22 member 11(SLC22A11)
M_25	92370	2-phosphoxylose phosphatase 1(PXYLP1)
M_25	2303	forkhead box C2(FOXC2)
M_25	6515	solute carrier family 2 member 3(SLC2A3)
M_25	5292	Pim-1 proto-oncogene, serine/threonine kinase(PIM1)
M_25	3772	potassium voltage-gated channel subfamily J member 15(KCNJ15)
M_26	79019	centromere protein M(CENPM)
M_26	29127	Rac GTPase activating protein 1(RACGAP1)
M_26	23397	non-SMC condensin I complex subunit H(NCAPH)
M_26	8318	cell division cycle 45(CDC45)
M_26	9928	kinesin family member 14(KIF14)
M_26	701	BUB1 mitotic checkpoint serine/threonine kinase
M_26	284403	WD repeat domain 62(WDR62)
M_26	9133	cyclin B2(CCNB2)
M_26	24137	kinesin family member 4A(KIF4A)
M_26	699	BUB1 mitotic checkpoint serine/threonine

M_26	128977	chromosome 22 open reading frame 39(C22orf39)
M_26	10635	RAD51 associated protein 1(RAD51AP1)
M_26	64976	mitochondrial ribosomal protein L40(MRPL40)
M_26	55789	DEP domain containing 1B(DEPDC1B)
M_26	63967	claspin(CLSPN)
M_26	5902	RAN binding protein 1(RANBP1)
M_26	55143	cell division cycle associated 8(CDCA8)
M_26	80764	THAP domain containing 7(THAP7)
M_26	1869	E2F transcription factor 1(E2F1)
M_26	4282	macrophage migration inhibitory factor (glycosylation-inhibiting factor)(MIF)
M_26	4288	marker of proliferation Ki-67(MKI67)
M_26	995	cell division cycle 25C(CDC25C)
M_26	993	cell division cycle 25A(CDC25A)
M_26	991	cell division cycle 20(CDC20)
M_26	990	cell division cycle 6(CDC6)
M_26	55388	minichromosome maintenance 10 replication initiation factor(MCM10)
M_26	9232	pituitary tumor-transforming 1(PTTG1)
M_26	22974	TPX2, microtubule nucleation factor(TPX2)
M_26	332	baculoviral IAP repeat containing 5(BIRC5)
M_26	259266	abnormal spindle microtubule assembly(ASPM)
M_26	8475	#N/A
M_26	7083	thymidine kinase 1(TK1)
M_26	6491	SCL/TAL1 interrupting locus(STIL)
M_26	4605	MYB proto-oncogene like 2(MYBL2)
M_26	729533	family with sequence similarity 72 member
M_26	55771	proline rich 11(PRR11)
M_26	151648	shugoshin 1(SGO1)
M_26	2491	centromere protein I(CENPI)
M_26	55215	Fanconi anemia complementation group I(FANCI)
M_26	494514	TYMS opposite strand(TYMSOS)

M_26	56992	kinesin family member 15(KIF15)
M_26	1062	centromere protein E(CENPE)
M_26	9787	DLG associated protein 5(DLGAP5)
M_26	983	cyclin dependent kinase 1(CDK1)
M_26	146909	kinesin family member 18B(KIF18B)
M_26	4085	MAD2 mitotic arrest deficient-like 1
M_26	6241	ribonucleotide reductase regulatory subunit
M_26	9212	aurora kinase B(AURKB)
M_26	54443	anillin actin binding protein(ANLN)
M_26	84057	meiotic nuclear divisions 1(MND1)
M_26	150223	YdjC homolog (bacterial)(YDJC)
M_26	5347	polo like kinase 1(PLK1)
M_26	79058	ASPSCR1, UBX domain containing tether for SLC2A4(ASPSCR1)
M_26	10721	DNA polymerase theta(POLQ)
M_26	6576	solute carrier family 25 member 1(SLC25A1)
M_26	8438	RAD54-like (S. cerevisiae)(RAD54L)
M_26	83540	NUF2, NDC80 kinetochore complex
M_26	10112	kinesin family member 20A(KIF20A)
M_26	9768	KIAA0101(KIAA0101)
M_26	1763	DNA replication helicase/nuclease 2(DNA2)
M_26	3838	karyopherin subunit alpha 2(KPNA2)
M_26	3832	kinesin family member 11(KIF11)
M_26	3833	kinesin family member C1(KIFC1)
M_26	27440	cat eye syndrome chromosome region, candidate
M_26	1894	epithelial cell transforming 2(ECT2)
M_26	128239	IQ motif containing GTPase activating protein
M_26	55165	centrosomal protein 55(CEP55)
M_26	4751	NIMA related kinase 2(NEK2)
M_26	2873	G protein pathway suppressor 1(GPS1)
M_26	10189	Aly/REF export factor(ALYREF)

M_26	54821	ERCC excision repair 6 like, spindle assembly checkpoint helicase(ERCC6L)
M_26	27037	tRNA methyltransferase 2 homolog A(TRMT2A)
M_26	4998	origin recognition complex subunit 1(ORC1)
M_26	55635	DEP domain containing 1(DEPDC1)
M_26	10733	polo like kinase 4(PLK4)
M_26	57082	kinetochore scaffold 1(KNL1)
M_26	51512	G2 and S-phase expressed 1(GTSE1)
M_26	29899	G-protein signaling modulator 2(GPSM2)
M_26	51514	denticleless E3 ubiquitin protein ligase
M_26	55355	Holliday junction recognition protein(HJURP)
M_26	3161	hyaluronan mediated motility receptor(HMMR)
M_26	284184	NADH:ubiquinone oxidoreductase complex assembly factor 8(NDUFAF8)
M_26	91687	centromere protein L(CENPL)
M_26	641	Bloom syndrome RecQ like helicase(BLM)
M_26	54478	family with sequence similarity 64 member
M_26	10403	NDC80, kinetochore complex component(NDC80)
M_26	221150	spindle and kinetochore associated complex subunit
M_26	8220	DiGeorge syndrome critical region gene 14(DGCR14)
M_26	11065	ubiquitin conjugating enzyme E2 C(UBE2C)
M_26	10460	transforming acidic coiled-coil containing protein
M_26	201254	centromere protein X(CENPX)
M_26	51529	anaphase promoting complex subunit 11(ANAPC11)
M_26	10587	thioredoxin reductase 2(TXNRD2)
M_26	57650	KIAA1524(KIAA1524)
M_26	653820	family with sequence similarity 72 member
M_26	80119	PIF1 5'-to-3' DNA helicase(PIF1)
M_26	9088	protein kinase, membrane associated tyrosine/threonine 1(PKMYT1)
M_26	83461	cell division cycle associated 3(CDCA3)
M_26	7298	thymidylate synthetase(TYMS)

M_26	64105	centromere protein K(CENPK)
M_26	1033	cyclin dependent kinase inhibitor 3(CDKN3)
M_26	890	cyclin A2(CCNA2)
M_26	891	cyclin B1(CCNB1)
M_26	899	cyclin F(CCNF)
M_26	10024	trophinin associated protein(TROAP)
M_26	9700	extra spindle pole bodies like 1, separase(ESPL1)
M_26	728833	family with sequence similarity 72 member
M_26	285643	kinesin family member 4B(KIF4B)
M_26	348262	MAPK regulated corepressor interacting protein
M_26	2305	forkhead box M1(FOXM1)
M_26	7153	topoisomerase (DNA) II alpha(TOP2A)
M_26	9493	kinesin family member 23(KIF23)
M_26	55732	chromosome 1 open reading frame 112(C1orf112)
M_26	6182	mitochondrial ribosomal protein L12(MRPL12)
M_26	57405	SPC25, NDC80 kinetochore complex
M_26	113115	mitochondrial fission regulator 2(MTFR2)
M_26	113130	cell division cycle associated 5(CDCA5)
M_26	10615	sperm associated antigen 5(SPAG5)
M_26	29089	ubiquitin conjugating enzyme E2 T(UBE2T)
M_26	83903	germ cell associated 2, haspin(GSG2)
M_26	339229	oxidoreductase like domain containing 1(OXLD1)
M_26	339231	ADP ribosylation factor like GTPase 16(ARL16)
M_26	7353	ubiquitin fusion degradation 1 like (yeast)(UFD1L)
M_26	81930	kinesin family member 18A(KIF18A)
M_26	11339	Opa interacting protein 5(OIP5)
M_26	90381	TOPBP1 interacting checkpoint and replication regulator(TICRR)
M_26	79733	E2F transcription factor 8(E2F8)
M_26	9156	exonuclease 1(EXO1)
M_26	78995	chromosome 17 open reading frame 53(C17orf53)
M_26	9055	protein regulator of cytokinesis 1(PRC1)

M_26	23753	stromal cell derived factor 2 like 1(SDF2L1)
M_26	1063	centromere protein F(CENPF)
M_26	1652	D-dopachrome tautomerase(DDT)
M_26	220134	spindle and kinetochore associated complex subunit
M_26	1058	centromere protein A(CENPA)
M_26	146956	essential meiotic structure-specific endonuclease
M_26	9833	maternal embryonic leucine zipper kinase(MELK)
M_26	51203	nucleolar and spindle associated protein 1(NUSAP1)
M_26	85359	DiGeorge syndrome critical region gene 6-
M_26	7332	ubiquitin conjugating enzyme E2 L3(UBE2L3)
M_26	6790	aurora kinase A(AURKA)
M_26	11004	kinesin family member 2C(KIF2C)
M_26	7272	TTK protein kinase(TTK)
M_26	150468	cytoskeleton associated protein 2 like(CKAP2L)
M_26	55247	nei like DNA glycosylase 3(NEIL3)
M_26	64151	non-SMC condensin I complex subunit G(NCAPG)
M_26	9342	synaptosome associated protein 29(SNAP29)
M_26	5888	RAD51 recombinase(RAD51)
M_26	81610	family with sequence similarity 83 member
M_26	89839	Rho GTPase activating protein 11B(ARHGAP11B)
M_26	11113	citron rho-interacting serine/threonine kinase(CIT)
M_26	57332	chromobox 8(CBX8)
M_26	219790	rhotekin 2(RTKN2)
M_26	125061	arylformamidase(AFMID)
M_26	22995	centrosomal protein 152(CEP152)
M_26	8914	timeless circadian clock(TIMELESS)
M_26	9837	GINS complex subunit 1(GINS1)
M_26	85001	uncharacterized protein MGC16275(MGC16275)
M_26	400916	coiled-coil-helix-coiled-coil-helix domain containing 10(CHCHD10)
M_26	79172	centromere protein O(CENPO)
M_26	83990	BRCA1 interacting protein C-terminal helicase

M_26	9585	kinesin family member 20B(KIF20B)
M_26	1164	CDC28 protein kinase regulatory subunit 2(CKS2)
M_26	1163	CDC28 protein kinase regulatory subunit 1B(CKS1B)
M_26	8535	chromobox 4(CBX4)
M_26	54584	G protein subunit beta 1 like(GNB1L)
M_26	55010	PARP1 binding protein(PARPBP)
M_26	55872	PDZ binding kinase(PBK)
M_26	100130717	CECR5 antisense RNA 1(CECR5-AS1)
M_26	90417	kinetochore localized astrin/SPAG5 binding
M_26	23306	nuclear envelope integral membrane protein
M_26	79980	DSN1 homolog, MIS12 kinetochore complex component(DSN1)
M_26	3619	inner centromere protein(INCENP)
M_26	151246	shugoshin 2(SGO2)
M_26	9824	Rho GTPase activating protein 11A(ARHGAP11A)
M_26	157570	establishment of sister chromatid cohesion N-acetyltransferase 2(ESCO2)
M_26	157313	cell division cycle associated 2(CDCA2)
M_27	79020	chromosome 7 open reading frame 25(C7orf25)
M_27	79017	gamma-glutamylcyclotransferase(GGCT)
M_27	348174	C-type lectin domain family 18 member A(CLEC18A)
M_27	6671	Sp4 transcription factor(SP4)
M_27	54664	transmembrane protein 106B(TMEM106B)
M_27	83605	CCM2 scaffolding protein(CCM2)
M_27	196	aryl hydrocarbon receptor(AHR)
M_27	5382	PMS1 homolog 2, mismatch repair system component pseudogene 4(PMS2P4)
M_27	64951	mitochondrial ribosomal protein S24(MRPS24)
M_27	493754	RAB guanine nucleotide exchange factor 1 pseudogene(GS1-124K5.11)
M_27	8512	mannose binding lectin 1, pseudogene(MBL1P)
M_27	28969	basic leucine zipper and W2 domains 2(BZW2)

M_27	154807	vitamin K epoxide reductase complex subunit 1 like 1(VKORC1L1)
M_27	5898	RAS like proto-oncogene A(RALA)
M_27	255031	long intergenic non-protein coding RNA
M_27	154881	potassium channel tetramerization domain containing 7(KCTD7)
M_27	3268	ArfGAP with FG repeats 2(AGFG2)
M_27	5683	proteasome subunit alpha 2(PSMA2)
M_27	51678	membrane palmitoylated protein 6(MPP6)
M_27	25870	sulfatase modifying factor 2(SUMF2)
M_27	5814	purine rich element binding protein B(PURB)
M_27	989	septin 7(SEPT7)
M_27	83637	zinc finger MIZ-type containing 2(ZMIZ2)
M_27	54205	cytochrome c, somatic(CYCS)
M_27	9238	transforming growth factor beta regulator 4(TBRG4)
M_27	84820	RNA polymerase II subunit J4,
M_27	6878	TATA-box binding protein associated factor 6(TAF6)
M_27	10652	YKT6 v-SNARE homolog (S. cerevisiae)(YKT6)
M_27	401331	RAS p21 protein activator 4C,
M_27	6119	replication protein A3(RPA3)
M_27	64983	mitochondrial ribosomal protein L32(MRPL32)
M_27	285958	small nucleolar RNA host gene 15(SNHG15)
M_27	221830	TWIST neighbor(TWISTNB)
M_27	8887	Tax1 binding protein 1(TAX1BP1)
M_27	9179	adaptor related protein complex 4 mu 1
M_27	497190	C-type lectin domain family 18 member B(CLEC18B)
M_27	168374	zinc finger protein 92(ZNF92)
M_27	677847	small nucleolar RNA, H/ACA box 81(SNORA81)
M_27	435	argininosuccinate lyase(ASL)
M_27	27072	VPS41, HOPS complex subunit(VPS41)
M_27	223082	zinc and ring finger 2(ZNRF2)
M_27	10156	RAS p21 protein activator 4(RASA4)

M_27	79706	PRKR interacting protein 1 (IL11
M_27	51427	zinc finger protein 107(ZNF107)
M_27	84182	family with sequence similarity 188 member
M_27	3364	HUS1 checkpoint clamp component(HUS1)
M_27	6100	retinitis pigmentosa 9 (autosomal dominant)(RP9)
M_27	26261	F-box protein 24(FBXO24)
M_27	90693	coiled-coil domain containing 126(CCDC126)
M_27	221895	JAZF zinc finger 1(JAZF1)
M_27	7205	thyroid hormone receptor interactor 6(TRIP6)
M_27	54606	DEAD-box helicase 56(DDX56)
M_27	55665	upregulator of cell proliferation(URGCP)
M_27	9771	Rap guanine nucleotide exchange factor 5(RAPGEF5)
M_27	440093	H3 histone family member 3C(H3F3C)
M_27	644591	peptidylprolyl isomerase A like 4G(PPIAL4G)
M_27	548644	RNA polymerase II subunit J3(POLR2J3)
M_27	644	biliverdin reductase A(BLVRA)
M_27	55253	tRNA-yW synthesizing protein 1 homolog(TYW1)
M_27	25948	kelch repeat and BTB domain containing 2(KBTBD2)
M_27	340277	family with sequence similarity 221 member
M_27	340252	zinc finger protein 680(ZNF680)
M_27	7697	zinc finger protein 138(ZNF138)
M_27	29896	transformer 2 alpha homolog(TRA2A)
M_27	646762	uncharacterized LOC646762(LOC646762)
M_27	23080	AVL9 cell migration associated(AVL9)
M_27	23658	LSM5 homolog, U6 small nuclear RNA and mRNA degradation associated(LSM5)
M_27	23660	zinc finger with KRAB and SCAN domains
M_27	26031	oxysterol binding protein like 3(OSBPL3)
M_27	26024	pentatricopeptide repeat domain 1(PTCD1)
M_27	54543	translocase of outer mitochondrial membrane
M_27	11097	nucleoporin like 2(NUPL2)
M_27	55975	kelch like family member 7(KLHL7)

M_27	23386	NudC domain containing 3(NUDCD3)
M_27	4967	oxoglutarate dehydrogenase(OGDH)
M_27	51619	ubiquitin conjugating enzyme E2 D4
M_27	55262	chromosome 7 open reading frame 43(C7orf43)
M_27	23161	sorting nexin 13(SNX13)
M_27	10392	nucleotide binding oligomerization domain containing
M_27	100130932	small nuclear ribonucleoprotein polypeptide G pseudogene 15(SNRPGP15)
M_27	441212	retinitis pigmentosa 9 pseudogene(RP9P)
M_27	441208	zinc and ring finger 2 pseudogene 1(ZNRF2P1)
M_27	441250	tRNA-yW synthesizing protein 1 homolog
M_27	10898	cleavage and polyadenylation specific factor
M_27	8621	cyclin dependent kinase 13(CDK13)
M_27	10793	zinc finger protein 273(ZNF273)
M_27	256364	echinoderm microtubule associated protein like
M_27	11328	FK506 binding protein 9(FKBP9)
M_27	55069	transmembrane protein 248(TMEM248)
M_27	9805	secernin 1(SCRN1)
M_27	677814	small nucleolar RNA, H/ACA box 31(SNORA31)
M_27	27342	RAB guanine nucleotide exchange factor
M_27	692205	small nucleolar RNA, C/D box 89(SNORD89)
M_27	64224	HERPUD family member 2(HERPUD2)
M_27	81628	TSC22 domain family member 4(TSC22D4)
M_27	84725	pleckstrin homology domain containing
M_27	5478	peptidylprolyl isomerase A(PPIA)
M_27	55536	cell division cycle associated 7 like(CDCA7L)
M_27	7551	zinc finger protein 3(ZNF3)
M_27	2617	glycyl-tRNA synthetase(GARS)
M_27	4034	leucine rich repeats and calponin homology domain containing 4(LRCH4)
M_27	222068	transmembrane p24 trafficking protein 4(TMED4)
M_27	222161	uncharacterized protein

M_27	222229	leucine rich repeats and WD repeat domain containing 1(LRWD1)
M_27	5425	DNA polymerase delta 2, accessory subunit(POLD2)
M_27	27241	Bardet-Biedl syndrome 9(BBS9)
M_27	83930	STARD3 N-terminal like(STARD3NL)
M_27	7589	zinc finger and SCAN domain containing
M_27	136895	chromosome 7 open reading frame 31(C7orf31)
M_27	90342	fer-1 like family member 5(FER1L5)
M_27	136647	M-phase specific PLK1 interacting protein(MPLKIP)
M_27	57002	Yae1 domain containing 1(YAE1D1)
M_27	11335	chromobox 3(CBX3)
M_27	56996	solute carrier family 12 member 9(SLC12A9)
M_27	84124	zinc finger protein 394(ZNF394)
M_27	4697	NDUFA4, mitochondrial complex
M_27	55744	cytochrome c oxidase assembly factor 1
M_27	2990	glucuronidase beta(GUSB)
M_27	51373	mitochondrial ribosomal protein S17(MRPS17)
M_27	115416	mitochondrial assembly of ribosomal large subunit 1(MALSU1)
M_27	653598	peptidylprolyl isomerase A like 4C(PPIAL4C)
M_27	723972	acidic nuclear phosphoprotein 32 family member A pseudogene 1(ANP32AP1)
M_27	283971	C-type lectin domain family 18 member C(CLEC18C)
M_27	643253	chaperonin containing TCP1 subunit 6 pseudogene
M_27	1815	dopamine receptor D4(DRD4)
M_27	11333	PDGFA associated protein 1(PDAP1)
M_27	24146	claudin 15(CLDN15)
M_27	51119	SBDS ribosome assembly guanine nucleotide exchange factor(SBDS)
M_27	56257	methylphosphate capping enzyme(MEPCE)
M_27	26824	RNA, U11 small nuclear(RNU11)
M_27	27297	CGRP receptor component(CRCP)

M_27	11112	3-hydroxyisobutyrate dehydrogenase(HIBADH)
M_27	64940	stromal antigen 3-like 4 (pseudogene)(STAG3L4)
M_27	28988	drebrin like(DBNL)
M_27	57037	ankyrin repeat and MYND domain containing
M_27	54784	alkB homolog 4, lysine demethylase(ALKBH4)
M_27	27434	DNA polymerase mu(POLM)
M_27	6094	retinal outer segment membrane protein 1(ROM1)
M_27	155370	Shwachman-Bodian-Diamond syndrome pseudogene 1(SBDSP1)
M_27	246721	RNA polymerase II subunit J2(POLR2J2)
M_27	57154	SMAD specific E3 ubiquitin protein ligase
M_27	349075	zinc finger protein 713(ZNF713)
M_27	349114	long intergenic non-protein coding RNA
M_27	9603	nuclear factor, erythroid 2 like 3(NFE2L3)
M_27	55915	LanC like 2(LANCL2)
M_27	7049	transforming growth factor beta receptor 3(TGFBR3)
M_27	3181	heterogeneous nuclear ribonucleoprotein A2/B1(HNRNPA2B1)
M_27	55971	BAI1 associated protein 2 like 1(BAIAP2L1)
M_27	9734	histone deacetylase 9(HDAC9)
M_27	51251	5'-nucleotidase, cytosolic IIIA(NT5C3A)
M_27	131544	crystallin beta-gamma domain containing 3(CRYBG3)
M_27	389538	MGC72080 pseudogene(MGC72080)
M_27	3614	inosine monophosphate dehydrogenase 1(IMPDH1)
M_27	677805	small nucleolar RNA, H/ACA box 18(SNORA18)
M_27	8985	procollagen-lysine,2-oxoglutarate 5-dioxygenase
M_27	6804	syntaxin 1A(STX1A)
M_27	27044	staphylococcal nuclease and tudor domain containing
M_27	360132	FK506 binding protein 9 pseudogene 1(FKBP9P1)
M_27	51142	coiled-coil-helix-coiled-coil-helix domain containing 2(CHCHD2)
M_27	51593	serrate, RNA effector molecule(SRRT)

M_27	1523	cut like homeobox 1(CUX1)
M_27	23480	Sec61 translocon gamma subunit(SEC61G)
M_27	574042	small nucleolar RNA, H/ACA box 10(SNORA10)
M_27	9856	KIAA0319(KIAA0319)
M_27	254102	EH domain binding protein 1 like 1(EHBP1L1)
M_27	154664	ATP binding cassette subfamily A member
M_28	79064	transmembrane protein 223(TMEM223)
M_28	10072	dipeptidyl peptidase 3(DPP3)
M_28	11007	coiled-coil domain containing 85B(CCDC85B)
M_28	4296	mitogen-activated protein kinase kinase kinase
M_28	9973	copper chaperone for superoxide dismutase(CCS)
M_28	6048	ring finger protein 5(RNF5)
M_28	54663	WD repeat domain 74(WDR74)
M_28	10263	cyclin dependent kinase 2 associated protein
M_28	79081	LBH domain containing 1(LBHD1)
M_28	5499	protein phosphatase 1 catalytic subunit
M_28	10589	DR1 associated protein 1(DRAP1)
M_28	10897	Yip1 interacting factor homolog A, membrane trafficking protein(YIF1A)
M_28	219927	mitochondrial ribosomal protein L21(MRPL21)
M_28	11041	beta-1,4-glucuronyltransferase 1(B4GAT1)
M_28	4714	NADH:ubiquinone oxidoreductase subunit
M_28	374395	transmembrane protein 179B(TMEM179B)
M_28	1460	casein kinase 2 beta(CSNK2B)
M_28	57175	coronin 1B(CORO1B)
M_28	9092	squamous cell carcinoma antigen recognized by T-cells 1(SART1)
M_28	80194	transmembrane protein 134(TMEM134)
M_28	80198	MUS81 structure-specific endonuclease
M_28	738	VPS51, GARP complex subunit(VPS51)
M_28	79004	CUE domain containing 2(CUEDC2)
M_28	30834	zinc ribbon domain containing 1(ZNRD1)

M_28	25855	breast cancer metastasis suppressor 1(BRMS1)
M_28	5936	RNA binding motif protein 4(RBM4)
M_28	4728	NADH:ubiquinone oxidoreductase core subunit
M_28	83638	chromosome 11 open reading frame 68(C11orf68)
M_28	23625	family with sequence similarity 89 member
M_28	4723	NADH:ubiquinone oxidoreductase core subunit
M_28	51596	cutA divalent cation tolerance homolog(CUTA)
M_28	5436	RNA polymerase II subunit G(POLR2G)
M_28	84285	eukaryotic translation initiation factor 1A domain containing(EIF1AD)
M_28	55611	OTU deubiquitinase, ubiquitin aldehyde binding
M_28	7918	G-patch domain and ankyrin repeats 1(GPANK1)
M_28	84153	ribonuclease H2 subunit C(RNASEH2C)
M_28	9049	aryl hydrocarbon receptor interacting protein(AIP)
M_28	83786	FERM domain containing 8(FRMD8)
M_28	79176	F-box and leucine rich repeat protein 15(FBXL15)
M_28	10471	prefoldin subunit 6(PFDN6)
M_28	65003	mitochondrial ribosomal protein L11(MRPL11)
M_28	8815	barrier to autointegration factor 1(BANF1)
M_28	9446	glutathione S-transferase omega 1(GSTO1)
M_28	402	ADP ribosylation factor like GTPase 2(ARL2)
M_28	403	ADP ribosylation factor like GTPase 3(ARL3)
M_28	84545	mitochondrial ribosomal protein L43(MRPL43)
M_28	119392	SWI5 dependent homologous recombination repair protein 1(SFR1)
M_28	51013	exosome component 1(EXOSC1)
M_28	25824	peroxiredoxin 5(PRDX5)
M_28	282991	biogenesis of lysosomal organelles complex 1 subunit 2(BLOC1S2)
M_28	119391	glutathione S-transferase omega 2(GSTO2)
M_28	84300	ubiquinol-cytochrome c reductase complex assembly factor 2(UQCC2)

M_28	55004	late endosomal/lysosomal adaptor, MAPK and MTOR activator 1(LAMTOR1)
M_28	10534	Sjogren syndrome/scleroderma autoantigen
M_28	572	BCL2 associated agonist of cell death(BAD)
M_28	57804	DNA polymerase delta 4, accessory subunit(POLD4)
M_28	10992	splicing factor 3b subunit 2(SF3B2)
M_28	10524	lysine acetyltransferase 5(KAT5)
M_28	7936	negative elongation factor complex member
M_28	1351	cytochrome c oxidase subunit 8A(COX8A)
M_28	10360	nucleophosmin/nucleoplasmin 3(NPM3)
M_28	78999	leucine rich repeat and fibronectin type III domain containing 4(LRFN4)
M_28	26472	protein phosphatase 1 regulatory inhibitor subunit 14B(PPP1R14B)
M_28	63943	FK506 binding protein like(FKBPL)
M_28	740	mitochondrial ribosomal protein L49(MRPL49)
M_28	254359	zinc finger DHHC-type containing 24(ZDHHC24)
M_28	57410	SCY1 like pseudokinase 1(SCYL1)
M_28	7542	zinc finger protein like 1(ZFPL1)
M_28	790955	ubiquinol-cytochrome c reductase complex assembly factor 3(UQCC3)
M_28	25911	deleted in primary ciliary dyskinesia homolog
M_28	79897	ribonuclease P/MRP subunit p21(RPP21)
M_28	6199	ribosomal protein S6 kinase B2(RPS6KB2)
M_28	83707	tRNA phosphotransferase 1(TRPT1)
M_28	10556	ribonuclease P/MRP subunit p30(RPP30)
M_28	55280	CWF19-like 1, cell cycle control (S.
M_28	7580	zinc finger protein 32(ZNF32)
M_28	2197	FAU, ubiquitin like and ribosomal protein S30
M_28	9158	FGF1 intracellular binding protein(FIBP)
M_28	57819	LSM2 homolog, U6 small nuclear RNA and mRNA degradation associated(LSM2)

M_28	5977	double PHD fingers 2(DPF2)
M_28	81876	RAB1B, member RAS oncogene family(RAB1B)
M_28	1072	cofilin 1(CFL1)
M_28	5970	RELA proto-oncogene, NF-kB subunit(RELA)
M_28	84304	nudix hydrolase 22(NUDT22)
M_28	9986	Ras converting CAAX endopeptidase 1(RCE1)
M_28	2286	FK506 binding protein 2(FKBP2)
M_28	254552	nudix hydrolase 8(NUDT8)
M_28	6631	small nuclear ribonucleoprotein polypeptide
M_28	29901	SAC3 domain containing 1(SAC3D1)
M_28	2950	glutathione S-transferase pi 1(GSTP1)
M_28	51504	tRNA methyltransferase 11-2 homolog (S. cerevisiae)(TRMT112)
M_28	29907	sorting nexin 15(SNX15)
M_28	6494	signal-induced proliferation-associated 1(SIPA1)
M_28	2101	estrogen related receptor alpha(ESRRA)
M_28	28992	MACRO domain containing 1(MACROD1)
M_28	51035	UBX domain protein 1(UBXN1)
M_28	79703	chromosome 11 open reading frame 80(C11orf80)
M_28	7423	vascular endothelial growth factor B(VEGFB)
M_28	3338	DnaJ heat shock protein family (Hsp40) member C4(DNAJC4)
M_28	92482	BBSome interacting protein 1(BBIP1)
M_28	200916	ribosomal protein L22 like 1(RPL22L1)
M_28	64837	kinesin light chain 2(KLC2)
M_28	118881	catechol-O-methyltransferase domain containing
M_28	6520	solute carrier family 3 member 2(SLC3A2)
M_29	79883	podocan like 1(PODNL1)
M_29	23670	transmembrane protein 2(TMEM2)
M_29	5526	protein phosphatase 2 regulatory subunit
M_29	90102	pleckstrin homology like domain family B member 2(PHLDB2)

M_29	22808	muscle RAS oncogene homolog(MRAS)
M_29	22885	actin binding LIM protein family member 3(ABLIM3)
M_29	4907	5'-nucleotidase ecto(NT5E)
M_29	2706	gap junction protein beta 2(GJB2)
M_29	4837	nicotinamide N-methyltransferase(NNMT)
M_29	9764	KIAA0513(KIAA0513)
M_29	4478	moesin(MSN)
M_29	2180	acyl-CoA synthetase long-chain family member
M_29	8829	neuropilin 1(NRP1)
M_29	718	complement C3(C3)
M_29	55062	WD repeat domain, phosphoinositide interacting
M_29	8828	neuropilin 2(NRP2)
M_29	84981	MIR22 host gene(MIR22HG)
M_29	860	runt related transcription factor 2(RUNX2)
M_29	861	runt related transcription factor 1(RUNX1)
M_29	6288	serum amyloid A1(SAA1)
M_29	6289	serum amyloid A2(SAA2)
M_29	9023	cholesterol 25-hydroxylase(CH25H)
M_29	10397	N-myc downstream regulated 1(NDRG1)
M_29	139818	dedicator of cytokinesis 11(DOCK11)
M_29	7421	vitamin D (1,25- dihydroxyvitamin D3)
M_29	1827	regulator of calcineurin 1(RCAN1)
M_29	55824	phosphoprotein membrane anchor with glycosphingolipid microdomains 1(PAG1)
M_29	5732	prostaglandin E receptor 2(PTGER2)
M_29	5328	plasminogen activator, urokinase(PLAU)
M_29	9180	oncostatin M receptor(OSMR)
M_29	8507	ectodermal-neural cortex 1(ENC1)
M_29	414236	chromosome 10 open reading frame 55(C10orf55)
M_29	389336	chromosome 5 open reading frame 46(C5orf46)
M_29	56169	gasdermin C(GSDMC)
M_29	84171	lysyl oxidase like 4(LOXL4)

M_29	51474	LIM domain and actin binding 1(LIMA1)
M_29	3566	interleukin 4 receptor(IL4R)
M_29	147920	IGF like family member 2(IGFL2)
M_29	57194	ATPase phospholipid transporting 10A
M_29	8740	tumor necrosis factor superfamily member
M_29	5271	serpin family B member 8(SERPINB8)
M_29	355	Fas cell surface death receptor(FAS)
M_29	5873	RAB27A, member RAS oncogene family(RAB27A)
M_29	6291	serum amyloid A4, constitutive(SAA4)
M_29	29940	dermatan sulfate epimerase(DSE)
M_29	388650	family with sequence similarity 69 member
M_29	7942	transcription factor EB(TFEB)
M_29	5329	plasminogen activator, urokinase receptor(PLAUR)
M_29	54757	FAM20A, golgi associated secretory pathway pseudokinase(FAM20A)
M_29	57559	STAM binding protein like 1(STAMBPL1)
M_29	30846	EH domain containing 2(EHD2)
M_29	23213	sulfatase 1(SULF1)
M_29	9953	heparan sulfate-glucosamine 3-sulfotransferase 3B1(HS3ST3B1)
M_29	9955	heparan sulfate-glucosamine 3-sulfotransferase 3A1(HS3ST3A1)
M_29	3730	anosmin 1(ANOS1)
M_29	9641	inhibitor of kappa light polypeptide gene enhancer in B-cells, kinase epsilon(IKBKE)
M_29	84815	uncharacterized protein MGC12916(MGC12916)
M_29	3274	histamine receptor H2(HRH2)
M_29	7227	transcriptional repressor GATA binding 1(TRPS1)
M_29	716	complement C1s(C1S)
M_29	715	complement C1r(C1R)
M_29	90853	SPOC domain containing 1(SPOCD1)
M_29	55350	vanin 3(VNN3)

M_29	4734	neural precursor cell expressed, developmentally down-regulated 4, E3 ubiquitin protein
M_29	8714	ATP binding cassette subfamily C member 3(ABCC3)
M_29	22898	DENN domain containing 3(DENND3)
M_29	3383	intercellular adhesion molecule 1(ICAM1)
M_29	57188	ADAMTS like 3(ADAMTSL3)
M_29	57616	teashirt zinc finger homeobox 3(TSHZ3)
M_30	79885	histone deacetylase 11(HDAC11)
M_30	10217	CTD small phosphatase like(CTDSPL)
M_30	84522	jagunal homolog 1(JAGN1)
M_30	285267	zinc finger protein 619(ZNF619)
M_30	9779	TBC1 domain family member 5(TBC1D5)
M_30	4292	mutL homolog 1(MLH1)
M_30	7324	ubiquitin conjugating enzyme E2 E1(UBE2E1)
M_30	55768	N-glycanase 1(NGLY1)
M_30	51095	tRNA nucleotidyl transferase 1(TRNT1)
M_30	54859	elongator acetyltransferase complex subunit 6(ELP6)
M_30	54977	solute carrier family 25 member 38(SLC25A38)
M_30	23609	makorin ring finger protein 2(MKRN2)
M_30	9797	TatD DNase domain containing 2(TATDN2)
M_30	4968	8-oxoguanine DNA glycosylase(OGG1)
M_30	51099	abhydrolase domain containing 5(ABHD5)
M_30	686	biotinidase(BTD)
M_30	6396	SEC13 homolog, nuclear pore and COPII coat complex component(SEC13)
M_30	30	acetyl-CoA acyltransferase 1(ACAA1)
M_30	51185	cereblon(CRBN)
M_30	64419	myotubularin related protein 14(MTMR14)
M_30	55209	SET domain containing 5(SETD5)
M_30	5894	Raf-1 proto-oncogene, serine/threonine kinase(RAF1)
M_30	85403	ELL associated factor 1(EAF1)

M_30	112616	CKLF like MARVEL transmembrane domain containing 7(CMTM7)
M_30	10474	transcriptional adaptor 3(TADA3)
M_30	84892	protein O-linked mannose N-acetylglucosaminyltransferase 2 (beta 1,4-
M_30	26061	2-hydroxyacyl-CoA lyase 1(HACL1)
M_30	151903	coiled-coil domain containing 12(CCDC12)
M_30	7428	von Hippel-Lindau tumor suppressor(VHL)
M_30	132001	TAM41 mitochondrial translocator assembly and maintenance homolog(TAMM41)
M_30	131965	methyltransferase like 6(METTL6)
M_30	55129	anoctamin 10(ANO10)
M_30	84818	interleukin 17 receptor C(IL17RC)
M_30	1499	catenin beta 1(CTNNB1)
M_30	80746	tRNA splicing endonuclease subunit 2(TSEN2)
M_30	10015	programmed cell death 6 interacting
M_30	7862	bromodomain and PHD finger containing 1(BRPF1)
M_30	2720	galactosidase beta 1(GLB1)
M_30	23243	ankyrin repeat domain 28(ANKRD28)
M_30	10533	autophagy related 7(ATG7)
M_30	92106	oxidoreductase NAD binding domain containing
M_30	5868	RAB5A, member RAS oncogene family(RAB5A)
M_30	25904	CCR4-NOT transcription complex subunit
M_30	64689	golgi reassembly stacking protein 1(GORASP1)
M_30	7584	zinc finger protein 35(ZNF35)
M_30	55207	ADP ribosylation factor like GTPase 8B(ARL8B)
M_30	23473	calpain 7(CAPN7)
M_30	10093	actin related protein 2/3 complex subunit 4(ARPC4)
M_30	28512	NFKB inhibitor interacting Ras like 1(NKIRAS1)
M_30	131474	coiled-coil-helix-coiled-coil-helix domain containing 4(CHCHD4)

M_30	55845	BRICK1, SCAR/WAVE actin nucleating complex subunit(BRK1)
M_30	285381	diphthamide biosynthesis 3(DPH3)
M_30	201595	STT3B, catalytic subunit of the oligosaccharyltransferase complex(STT3B)
M_30	152189	CKLF like MARVEL transmembrane domain containing 8(CMTM8)
M_30	51188	SS18 like 2(SS18L2)
M_30	79188	transmembrane protein 43(TMEM43)
M_30	152302	cell death-inducing DFFA-like effector c pseudogene(CIDECP)
M_30	51143	dynein cytoplasmic 1 light intermediate chain
M_30	152100	C-X9-C motif containing 1(CMC1)
M_30	10201	NME/NM23 nucleoside diphosphate kinase 6(NME6)
M_30	54995	3-oxoacyl-ACP synthase, mitochondrial(OXSM)
M_30	25917	THUMP domain containing 3(THUMPD3)
M_30	9117	SEC22 homolog C, vesicle trafficking
M_30	51372	translation machinery associated 7 homolog(TMA7)
M_30	401052	uncharacterized LOC401052(LOC401052)
M_30	51244	coiled-coil domain containing 174(CCDC174)
M_30	64145	rabenosyn, RAB effector(RBSN)
M_30	7508	XPC complex subunit, DNA damage recognition and repair factor(XPC)
M_30	285362	sulfatase modifying factor 1(SUMF1)
M_30	285367	RNA pseudouridylate synthase domain containing
M_30	10289	eukaryotic translation initiation factor 1B(EIF1B)
M_30	10402	ST3 beta-galactoside alpha-2,3-sialyltransferase
M_30	27258	LSM3 homolog, U6 small nuclear RNA and mRNA degradation associated(LSM3)
M_30	7342	upstream binding protein 1 (LBP-1a)(UBP1)
M_30	6419	SET domain and mariner transposase fusion

M_30	54918	CKLF like MARVEL transmembrane domain containing 6(CMTM6)
M_30	23016	exosome component 7(EXOSC7)
M_30	55831	ER membrane protein complex subunit 3(EMC3)
M_30	78987	cysteine rich with EGF like domains 1(CRELD1)
M_30	64432	mitochondrial ribosomal protein S25(MRPS25)
M_30	56852	RAD18, E3 ubiquitin protein ligase(RAD18)
M_30	9686	vestigial like family member 4(VGLL4)
M_30	9209	LRR binding FLII interacting protein 2(LRRFIP2)
M_30	7182	nuclear receptor subfamily 2 group C member
M_30	25994	HIG1 hypoxia inducible domain family member
M_30	64343	5-azacytidine induced 2(AZI2)
M_30	10491	cartilage associated protein(CRTAP)
M_30	100128640	ACVR2B antisense RNA 1(ACVR2B-AS1)
M_30	54861	SNF related kinase(SNRK)
M_30	22908	SAC1 suppressor of actin mutations 1-like
M_30	9941	exo/endonuclease G(EXOG)
M_30	23171	glycerol-3-phosphate dehydrogenase 1-like(GPD1L)
M_30	152098	zinc finger CW-type and PWWP domain containing 2(ZCWPW2)
M_30	9497	solute carrier family 4 member 7(SLC4A7)
M_30	285343	T-cell activation inhibitor, mitochondrial(TCAIM)
M_30	729085	family with sequence similarity 198 member
M_30	51304	zinc finger DHHC-type containing 3(ZDHHC3)
M_30	9975	nuclear receptor subfamily 1 group D member
M_30	497258	BDNF antisense RNA(BDNF-AS)
M_30	159686	cilia and flagella associated protein 58(CFAP58)
M_30	643853	transmembrane protein with metallophosphoesterase domain(TMPPE)
M_30	8372	hyaluronoglucosaminidase 3(HYAL3)
M_30	116931	mediator complex subunit 12 like(MED12L)
M_30	5333	phospholipase C delta 1(PLCD1)

M_30	9852	EPM2A interacting protein 1(EPM2AIP1)
M_30	25827	F-box and leucine rich repeat protein 2(FBXL2)
M_31	79895	ATPase phospholipid transporting 8B4
M_31	11314	CD300a molecule(CD300A)
M_31	11309	solute carrier organic anion transporter family member 2B1(SLCO2B1)
M_31	3903	leukocyte associated immunoglobulin like receptor
M_31	929	CD14 molecule(CD14)
M_31	313	acyloxyacyl hydrolase(AOAH)
M_31	27036	sialic acid binding Ig like lectin 7(SIGLEC7)
M_31	6039	ribonuclease A family member k6(RNASE6)
M_31	51225	ABI family member 3(ABI3)
M_31	6688	Spi-1 proto-oncogene(SPI1)
M_31	284759	signal regulatory protein beta 2(SIRPB2)
M_31	199	allograft inflammatory factor 1(AIF1)
M_31	695	Bruton tyrosine kinase(BTK)
M_31	7462	linker for activation of T-cells family member
M_31	963	CD53 molecule(CD53)
M_31	10859	leukocyte immunoglobulin like receptor B1(LILRB1)
M_31	962	CD48 molecule(CD48)
M_31	968	CD68 molecule(CD68)
M_31	56833	SLAM family member 8(SLAMF8)
M_31	3689	integrin subunit beta 2(ITGB2)
M_31	3687	integrin subunit alpha X(ITGAX)
M_31	3684	integrin subunit alpha M(ITGAM)
M_31	3683	integrin subunit alpha L(ITGAL)
M_31	27128	cytohesin 4(CYTH4)
M_31	155038	GTPase, IMAP family member 8(GIMAP8)
M_31	9459	Rac/Cdc42 guanine nucleotide exchange factor
M_31	1230	C-C motif chemokine receptor 1(CCR1)
M_31	58475	membrane spanning 4-domains A7(MS4A7)
M_31	719	complement C3a receptor 1(C3AR1)

M_31	714	complement C1q C chain(C1QC)
M_31	57705	WDFY family member 4(WDFY4)
M_31	712	complement C1q A chain(C1QA)
M_31	713	complement C1q B chain(C1QB)
M_31	1536	cytochrome b-245 beta chain(CYBB)
M_31	945	CD33 molecule(CD33)
M_31	54518	amyloid beta precursor protein binding family B member 1 interacting protein(APBB1IP)
M_31	942	CD86 molecule(CD86)
M_31	285195	solute carrier family 9 member A9(SLC9A9)
M_31	54209	triggering receptor expressed on myeloid cells
M_31	8676	syntaxin 11(STX11)
M_31	241	arachidonate 5-lipoxygenase activating
M_31	168537	GTPase, IMAP family member 7(GIMAP7)
M_31	51284	toll like receptor 7(TLR7)
M_31	4332	myeloid cell nuclear differentiation antigen(MNDA)
M_31	64231	membrane spanning 4-domains A6A(MS4A6A)
M_31	22797	transcription factor EC(TFEC)
M_31	140	adenosine A3 receptor(ADORA3)
M_31	139716	GRB2 associated binding protein 3(GAB3)
M_31	2207	Fc fragment of IgE receptor Ig(FCER1G)
M_31	2209	Fc fragment of IgG receptor Ia(FCGR1A)
M_31	8477	G protein-coupled receptor 65(GPR65)
M_31	2212	Fc fragment of IgG receptor IIa(FCGR2A)
M_31	2210	Fc fragment of IgG receptor Ib(FCGR1B)
M_31	2214	Fc fragment of IgG receptor IIIa(FCGR3A)
M_31	1439	colony stimulating factor 2 receptor beta common subunit(CSF2RB)
M_31	9332	CD163 molecule(CD163)
M_31	2313	Fli-1 proto-oncogene, ETS transcription factor(FLI1)
M_31	79626	TNF alpha induced protein 8 like 2(TNFAIP8L2)
M_31	219972	macrophage expressed 1(MPEG1)

M_31	64407	regulator of G-protein signaling 18(RGS18)
M_31	9450	lymphocyte antigen 86(LY86)
M_31	64581	C-type lectin domain family 7 member A(CLEC7A)
M_31	30817	adhesion G protein-coupled receptor E2(ADGRE2)
M_31	286336	family with sequence similarity 78 member
M_31	3071	NCK associated protein 1 like(NCKAP1L)
M_31	2359	formyl peptide receptor 3(FPR3)
M_31	55911	apolipoprotein B receptor(APOBR)
M_31	51338	membrane spanning 4-domains A4A(MS4A4A)
M_31	84868	hepatitis A virus cellular receptor 2(HAVCR2)
M_31	7099	toll like receptor 4(TLR4)
M_31	10871	CD300c molecule(CD300C)
M_31	54440	SAM and SH3 domain containing 3(SASH3)
M_31	6916	thromboxane A synthase 1(TBXAS1)
M_31	2124	ecotropic viral integration site 2B(EVI2B)
M_31	2123	ecotropic viral integration site 2A(EVI2A)
M_31	5294	phosphatidylinositol-4,5-bisphosphate 3-kinase catalytic subunit gamma(PIK3CG)
M_31	5552	serglycin(SRGN)
M_31	474344	GTPase, IMAP family member 6(GIMAP6)
M_31	23533	phosphoinositide-3-kinase regulatory subunit
M_31	51411	bridging integrator 2(BIN2)
M_31	55619	dedicator of cytokinesis 10(DOCK10)
M_31	6503	Src-like-adaptor(SLA)
M_31	1234	C-C motif chemokine receptor 5
M_31	81704	dedicator of cytokinesis 8(DOCK8)
M_31	7805	lysosomal protein transmembrane 5(LAPTM5)
M_31	4689	neutrophil cytosolic factor 4(NCF4)
M_31	4481	macrophage scavenger receptor 1(MSR1)
M_31	1794	dedicator of cytokinesis 2(DOCK2)
M_31	4046	lymphocyte-specific protein 1(LSP1)
M_31	1436	colony stimulating factor 1 receptor(CSF1R)

M_31	2533	FYN binding protein(FYB)
M_31	10875	fibrinogen like 2(FGL2)
M_31	26191	protein tyrosine phosphatase, non-receptor type
M_31	5788	protein tyrosine phosphatase, receptor type C(PTPRC)
M_31	5341	pleckstrin(PLEK)
M_31	2857	G protein-coupled receptor 34(GPR34)
M_31	7305	TYRO protein tyrosine kinase binding
M_31	55340	GTPase, IMAP family member 5(GIMAP5)
M_31	653361	neutrophil cytosolic factor 1(NCF1)
M_31	4064	CD180 molecule(CD180)
M_31	27180	sialic acid binding Ig like lectin 9(SIGLEC9)
M_31	92241	RCSD domain containing 1(RCSD1)
M_31	124460	sorting nexin 20(SNX20)
M_31	10288	leukocyte immunoglobulin like receptor B2(LILRB2)
M_31	11326	V-set and immunoglobulin domain containing
M_31	55803	ArfGAP with dual PH domains 2(ADAP2)
M_31	7454	Wiskott-Aldrich syndrome(WAS)
M_31	51311	toll like receptor 8(TLR8)
M_31	83706	fermitin family member 3(FERMT3)
M_31	80231	chromosome X open reading frame 21(CXorf21)
M_31	3055	HCK proto-oncogene, Src family tyrosine
M_31	64926	RAS protein activator like 3(RASAL3)
M_31	126364	leucine rich repeat containing 25(LRRC25)
M_31	4542	myosin IF(MYO1F)
M_31	11006	leukocyte immunoglobulin like receptor B4(LILRB4)
M_31	951	CD37 molecule(CD37)
M_31	55303	GTPase, IMAP family member 4(GIMAP4)
M_31	117289	T-cell activation RhoGTPase activating
M_31	8832	CD84 molecule(CD84)
M_31	170575	GTPase, IMAP family member 1(GIMAP1)
M_31	3937	lymphocyte cytosolic protein 2(LCP2)
M_31	3587	interleukin 10 receptor subunit alpha(IL10RA)

M_31	64333	Rho GTPase activating protein 9(ARHGAP9)
M_31	89790	sialic acid binding Ig like lectin 10(SIGLEC10)
M_31	10320	IKAROS family zinc finger 1(IKZF1)
M_31	64092	SAM domain, SH3 domain and nuclear localization signals 1(SAMSN1)
M_31	120425	junction adhesion molecule like(JAML)
M_31	4069	lysozyme(LYZ)
M_31	10870	hematopoietic cell signal transducer(HCST)
M_31	51393	transient receptor potential cation channel subfamily V member 2(TRPV2)
M_31	53831	G protein-coupled receptor 84(GPR84)
M_31	654817	neutrophil cytosolic factor 1C pseudogene(NCF1C)
M_31	7456	WAS/WASL interacting protein family member
M_31	7940	leukocyte specific transcript 1(LST1)
M_31	2213	Fc fragment of IgG receptor IIb(FCGR2B)
M_31	128346	chromosome 1 open reading frame 162(C1orf162)
M_31	80342	TRAF3 interacting protein 3(TRAF3IP3)
M_31	9404	leupaxin(LPXN)
M_31	2357	formyl peptide receptor 1(FPR1)
M_31	3676	integrin subunit alpha 4(ITGA4)
M_31	114548	NLR family pyrin domain containing 3(NLRP3)
M_31	9535	glia maturation factor gamma(GMFG)
M_31	11027	leukocyte immunoglobulin like receptor A2(LILRA2)
M_31	55843	Rho GTPase activating protein 15(ARHGAP15)
M_31	9034	C-C motif chemokine receptor like 2(CCRL2)
M_31	397	Rho GDP dissociation inhibitor beta(ARHGDIB)
M_31	9595	cytohesin 1 interacting protein(CYTIP)
M_31	10261	immunoglobulin superfamily member 6(IGSF6)
M_31	23166	stabilin 1(STAB1)
M_31	50856	C-type lectin domain family 4 member A(CLEC4A)
M_31	202309	GRB2 binding adaptor protein,
M_31	79690	galactose-3-O-sulfotransferase 4(GAL3ST4)

M_31	9103	Fc fragment of IgG receptor IIc
M_31	146722	CD300 molecule like family member f(CD300LF)
M_31	6614	sialic acid binding Ig like lectin 1(SIGLEC1)
M_31	53829	purinergic receptor P2Y13(P2RY13)
M_31	81793	toll like receptor 10(TLR10)
M_32	79948	phospholipid phosphatase related 3(PLPPR3)
M_32	91304	transmembrane protein 259(TMEM259)
M_32	91300	R3H domain containing 4(R3HDM4)
M_32	79875	thrombospondin type 1 domain containing 4(THSD4)
M_32	29985	solute carrier family 39 member 3(SLC39A3)
M_32	1613	death associated protein kinase 3(DAPK3)
M_32	54531	MIER family member 2(MIER2)
M_32	6455	SH3 domain containing GRB2 like 1, endophilin
M_32	23396	phosphatidylinositol-4-phosphate 5-kinase type 1 gamma(PIP5K1C)
M_32	80017	chromosome 14 open reading frame 159(C14orf159)
M_32	54760	proprotein convertase subtilisin/kexin type 4(PCSK4)
M_32	2788	G protein subunit gamma 7(GNG7)
M_32	51343	fizzy/cell division cycle 20 related 1(FZR1)
M_32	127281	family with sequence similarity 213 member
M_32	5442	RNA polymerase mitochondrial(POLRMT)
M_32	60680	CUGBP, Elav-like family member 5(CELF5)
M_32	730112	family with sequence similarity 166 member
M_32	399664	mex-3 RNA binding family member D(MEX3D)
M_32	26528	DAZ associated protein 1(DAZAP1)
M_32	81926	abhydrolase domain containing 17A(ABHD17A)
M_32	6449	small glutamine rich tetratricopeptide repeat containing alpha(SGTA)
M_32	5990	regulatory factor X2(RFX2)
M_32	6794	serine/threonine kinase 11(STK11)
M_32	2593	guanidinoacetate N-methyltransferase(GAMT)
M_32	55111	pleckstrin homology domain containing J1(PLEKHJ1)

M_32	148254	zinc finger protein 555(ZNF555)
M_32	148223	chromosome 19 open reading frame 25(C19orf25)
M_32	148252	DIRAS family GTPase 1(DIRAS1)
M_32	58509	cactin, spliceosome C complex subunit(CACTIN)
M_32	407835	mitogen-activated protein kinase kinase 2 pseudogene(LOC407835)
M_32	1455	casein kinase 1 gamma 2(CSNK1G2)
M_32	83475	deoxyhypusine hydroxylase/monooxygenase(DOHH)
M_32	997	cell division cycle 34(CDC34)
M_32	7089	transducin like enhancer of split 2(TLE2)
M_32	10025	mediator complex subunit 16(MED16)
M_32	5725	polypyrimidine tract binding protein 1(PTBP1)
M_32	6929	transcription factor 3(TCF3)
M_32	4782	nuclear factor I C(NFIC)
M_32	51548	sirtuin 6(SIRT6)
M_32	84823	lamin B2(LMNB2)
M_32	5434	RNA polymerase II subunit E(POLR2E)
M_32	84717	hepatoma-derived growth factor-related protein
M_32	8938	BAI1 associated protein 3(BAIAP3)
M_32	91978	tubulin polyglutamylase complex subunit 1(TPGS1)
M_32	8943	adaptor related protein complex 3 delta 1
M_32	9546	amyloid beta precursor protein binding family A member 3(APBA3)
M_32	55527	fem-1 homolog A(FEM1A)
M_32	56928	signal peptide peptidase like 2B(SPPL2B)
M_32	55702	coiled-coil domain containing 94(CCDC94)
M_32	56926	nicalin(NCLN)
M_32	148046	CIRBP antisense RNA 1(CIRBP-AS1)
M_32	116444	glutamate ionotropic receptor NMDA type subunit 3B(GRIN3B)
M_32	55643	BTB domain containing 2(BTBD2)
M_32	113178	secretory carrier membrane protein 4(SCAMP4)

M_32	115196	zinc finger protein 554(ZNF554)
M_32	113179	adenosine deaminase, tRNA specific 3(ADAT3)
M_32	51588	protein inhibitor of activated STAT 4(PIAS4)
M_32	8175	splicing factor 3a subunit 2(SF3A2)
M_32	2530	fucosyltransferase 8(FUT8)
M_32	79258	membrane metalloendopeptidase like 1(MMEL1)
M_32	91039	dipeptidyl peptidase 9(DPP9)
M_32	90007	midnolin(MIDN)
M_32	79816	transducin like enhancer of split 6(TLE6)
M_32	166	amino-terminal enhancer of split(AES)
M_32	92840	receptor accessory protein 6(REEP6)
M_32	219293	ATPase family, AAA domain containing
M_32	83855	Kruppel like factor 16(KLF16)
M_32	10002	nuclear receptor subfamily 2 group E member
M_32	84444	DOT1 like histone lysine methyltransferase(DOT1L)
M_32	126295	zinc finger protein 57(ZNF57)
M_32	1153	cold inducible RNA binding protein(CIRBP)
M_32	645431	FUT8 antisense RNA 1(FUT8-AS1)
M_32	10362	high mobility group 20B(HMG20B)
M_32	51341	zinc finger and BTB domain containing 7A(ZBTB7A)
M_32	57418	WD repeat domain 18(WDR18)
M_32	1943	ephrin A2(EFNA2)
M_32	58492	zinc finger protein 77(ZNF77)
M_32	84939	melanoma associated antigen (mutated) 1(MUM1)
M_32	53615	methyl-CpG binding domain protein 3(MBD3)
M_32	7064	thimet oligopeptidase 1(THOP1)
M_32	11076	tubulin polymerization promoting protein(TPPP)
M_32	2872	MAP kinase interacting serine/threonine kinase
M_32	79844	zinc finger DHHC-type containing 11(ZDHHC11)
M_32	682	basigin (Ok blood group)(BSG)
M_32	5605	mitogen-activated protein kinase kinase 2(MAP2K2)
M_32	2767	G protein subunit alpha 11(GNA11)

M_32	55658	ring finger protein 126(RNF126)
M_32	23624	Cbl proto-oncogene C(CBLC)
M_32	126308	MOB kinase activator 3A(MOB3A)
M_32	126321	major facilitator superfamily domain containing
M_32	80700	UBX domain protein 6(UBXN6)
M_32	145482	prostaglandin reductase 2(PTGR2)
M_32	57526	protocadherin 19(PCDH19)
M_32	57455	RNA exonuclease 1 homolog(REXO1)
M_32	566	azurocidin 1(AZU1)
M_32	55620	signal transducing adaptor family member 2(STAP2)
M_32	10226	perilipin 3(PLIN3)
M_32	29128	ubiquitin like with PHD and ring finger domains
M_32	84954	MPN domain containing(MPND)
M_32	23566	lysophosphatidic acid receptor 3(LPAR3)
M_32	10036	chromatin assembly factor 1 subunit A(CHAF1A)
M_32	4602	MYB proto-oncogene, transcription factor(MYB)
M_32	10432	RNA binding motif protein 14(RBM14)
M_32	56005	myeloid derived growth factor(MYDGF)
M_32	255231	mucolipin 2(MCOLN2)
M_32	339456	transmembrane protein 52(TMEM52)
M_32	148022	toll like receptor adaptor molecule 1(TICAM1)
M_32	54039	poly(rC) binding protein 3(PCBP3)
M_32	7536	splicing factor 1(SF1)
M_32	22904	strawberry notch homolog 2(SBNO2)
M_33	1841	deoxythymidylate kinase(DTYMK)
M_33	55759	WD repeat domain 12(WDR12)
M_33	23549	aspartyl aminopeptidase(DNPEP)
M_33	57140	arginyl aminopeptidase like 1(RNPEPL1)
M_33	79568	matrix AAA peptidase interacting protein 1(MAIP1)
M_33	9818	nucleoporin 58(NUP58)
M_33	471	5-aminoimidazole-4-carboxamide ribonucleotide formyltransferase/IMP cyclohydrolase(ATIC)

M_33	8864	period circadian clock 2(PER2)
M_33	79411	galactosidase beta 1 like(GLB1L)
M_33	5757	prothymosin, alpha(PTMA)
M_33	79840	non-homologous end joining factor 1(NHEJ1)
M_33	4691	nucleolin(NCL)
M_33	3069	high density lipoprotein binding protein(HDLBP)
M_33	56947	mitochondrial fission factor(MFF)
M_33	5510	protein phosphatase 1 regulatory subunit 7(PPP1R7)
M_33	9759	histone deacetylase 4(HDAC4)
M_33	79083	melanophilin(MLPH)
M_33	4709	NADH:ubiquinone oxidoreductase subunit
M_33	54097	family with sequence similarity 3 member B(FAM3B)
M_33	151477	long intergenic non-protein coding RNA
M_33	26146	TRAF3 interacting protein 1(TRAF3IP1)
M_33	58190	CTD small phosphatase 1(CTDSP1)
M_33	580	BRCA1 associated RING domain 1(BARD1)
M_33	285193	dual specificity phosphatase 28(DUSP28)
M_33	10920	COP9 signalosome subunit 8(COPS8)
M_33	14	angio associated migratory cell protein(AAMP)
M_33	64708	COP9 signalosome subunit 7B(COPS7B)
M_33	3267	ArfGAP with FG repeats 1(AGFG1)
M_33	7520	X-ray repair cross complementing 5(XRCC5)
M_33	222484	ligand of numb-protein X 2(LNX2)
M_33	375307	ciliogenesis associated TTC17 interacting
M_33	2181	acyl-CoA synthetase long-chain family member
M_33	27013	cyclin Pas1/PHO80 domain containing 1(CNPPD1)
M_33	27148	serine/threonine kinase 36(STK36)
M_33	23192	autophagy related 4B cysteine peptidase(ATG4B)
M_33	51665	ankyrin repeat and SOCS box containing 1(ASB1)
M_33	143	poly(ADP-ribose) polymerase family member
M_33	84236	rhomboid domain containing 1(RHBDD1)
M_33	4719	NADH:ubiquinone oxidoreductase core subunit

M_33	6541	solute carrier family 7 member 1(SLC7A1)
M_33	130617	zinc finger AN1-type containing 2B(ZFAND2B)
M_33	55139	ankyrin repeat and zinc finger domain containing
M_33	9654	tubulin tyrosine ligase like 4(TTLL4)
M_33	51540	selenocysteine lyase(SCLY)
M_33	51602	NOP58 ribonucleoprotein(NOP58)
M_33	79137	family with sequence similarity 134 member
M_33	55686	melanoregulin(MREG)
M_33	93010	UDP-GlcNAc:betaGal beta-1,3-N-acetylglucosaminyltransferase 7(B3GNT7)
M_33	9855	FERM, ARH/RhoGEF and pleckstrin domain protein 2(FARP2)
M_33	8576	serine/threonine kinase 16(STK16)
M_33	23242	cordon-bleu WH2 repeat protein(COBL)
M_33	6120	ribulose-5-phosphate-3-epimerase(RPE)
M_33	474338	SUMO1 pseudogene 3(SUMO1P3)
M_33	64114	transmembrane BAX inhibitor motif containing
M_33	11132	calpain 10(CAPN10)
M_33	55054	autophagy related 16 like 1(ATG16L1)
M_33	57590	WD repeat and FYVE domain containing 1(WDFY1)
M_33	22868	FAST kinase domains 2(FASTKD2)
M_33	80210	armadillo repeat containing 9(ARMC9)
M_33	129563	DIS3 like 3'-5' exoribonuclease 2(DIS3L2)
M_33	84289	inhibitor of growth family member 5(ING5)
M_33	729020	ribulose-5-phosphate-3-epimerase like 1(RPEL1)
M_33	27242	TNF receptor superfamily member 21(TNFRSF21)
M_33	253832	zinc finger DHHC-type containing 20(ZDHHC20)
M_33	140739	ubiquitin conjugating enzyme E2 F (putative)(UBE2F)
M_33	64320	ring finger protein 25(RNF25)
M_33	4735	septin 2(SEPT2)
M_33	54737	M-phase phosphoprotein 8(MPHOSPH8)
M_33	2859	G protein-coupled receptor 35(GPR35)

M_33	130888	F-box protein 36(FBXO36)
M_33	130916	mitochondrial transcription termination factor
M_33	51078	THAP domain containing 4(THAP4)
M_33	84812	phospholipase C delta 4(PLCD4)
M_33	23178	PAS domain containing serine/threonine
M_33	219333	ubiquitin specific peptidase 12(USP12)
M_33	9320	thyroid hormone receptor interactor 12(TRIP12)
M_33	284996	ring finger protein 149(RNF149)
M_33	51281	ankyrin repeat and MYND domain containing
M_33	60491	NGG1 interacting factor 3 like 1(NIF3L1)
M_33	65080	mitochondrial ribosomal protein L44(MRPL44)
M_33	79065	autophagy related 9A(ATG9A)
M_33	29081	methyltransferase like 5(METTL5)
M_33	29926	GDP-mannose pyrophosphorylase A(GMPPA)
M_33	3329	heat shock protein family D (Hsp60) member
M_33	55230	ubiquitin specific peptidase 40(USP40)
M_33	3300	DnaJ heat shock protein family (Hsp40) member B2(DNAJB2)
M_33	55359	serine/threonine/tyrosine kinase 1(STYK1)
M_33	151194	methyltransferase like 21A(METTL21A)
M_33	617	BCS1 homolog, ubiquinol-cytochrome c reductase complex chaperone(BCS1L)
M_33	26058	GRB10 interacting GYF protein 2(GIGYF2)
M_33	10056	phenylalanyl-tRNA synthetase beta subunit(FARSB)
M_33	51719	calcium binding protein 39(CAB39)
M_33	3336	heat shock protein family E (Hsp10) member
M_33	50485	SWI/SNF related, matrix associated, actin dependent regulator of chromatin, subfamily a like
M_33	150678	COP9 signalosome subunit 9(COPS9)
M_33	116987	ArfGAP with GTPase domain, ankyrin repeat and PH domain 1(AGAP1)
M_33	130367	sphingosine-1-phosphate phosphatase 2(SGPP2)

M_33	339122	RAB43, member RAS oncogene family(RAB43)
M_33	6049	ring finger protein 6(RNF6)
M_33	57695	ubiquitin specific peptidase 37(USP37)
M_33	7341	small ubiquitin-like modifier 1(SUMO1)
M_33	25953	paroxysmal nonkinesigenic dyskinesia(PNKD)
M_33	728294	D-2-hydroxyglutarate dehydrogenase(D2HGDH)
M_33	4999	origin recognition complex subunit 2(ORC2)
M_33	80895	ILK associated serine/threonine phosphatase(ILKAP)
M_33	7701	zinc finger protein 142(ZNF142)
M_33	5707	proteasome 26S subunit, non-ATPase 1(PSMD1)
M_33	9205	zinc finger MYM-type containing 5(ZMYM5)
M_33	4705	NADH:ubiquinone oxidoreductase subunit
M_33	283932	FBXL19 antisense RNA 1 (head to head)(FBXL19-
M_33	8452	cullin 3(CUL3)
M_33	9208	LRR binding FLII interacting protein 1(LRRFIP1)
M_33	5147	phosphodiesterase 6D(PDE6D)
M_33	84910	transmembrane protein 87B(TMEM87B)
M_33	9470	eukaryotic translation initiation factor 4E family member 2(EIF4E2)
M_33	9125	CCR4-NOT transcription complex subunit 9(CNOT9)
M_33	50636	anoctamin 7(ANO7)
M_33	10109	actin related protein 2/3 complex subunit 2(ARPC2)
M_33	64328	exportin 4(XPO4)
M_33	64284	RAB17, member RAS oncogene family(RAB17)
M_33	114790	serine/threonine kinase 11 interacting
M_33	10494	serine/threonine kinase 25(STK25)
M_33	10010	TRAF family member associated NFKB
M_33	54865	G-patch domain containing 4(GPATCH4)
M_33	221178	spermatogenesis associated 13(SPATA13)
M_33	283948	NHL repeat containing 4(NHLRC4)
M_33	375775	patatin like phospholipase domain containing
M_33	90	activin A receptor type 1(ACVR1)

M_33	2139	EYA transcriptional coactivator and phosphatase
M_33	8682	phosphoprotein enriched in astrocytes 15(PEA15)
M_33	374977	maestro heat like repeat family member 7(MROH7)
M_33	7852	C-X-C motif chemokine receptor 4(CXCR4)
M_33	28316	cadherin 20(CDH20)
M_34	1842	extracellular matrix protein 2(ECM2)
M_34	4005	LIM domain only 2(LMO2)
M_34	54538	roundabout guidance receptor 4(ROBO4)
M_34	3910	laminin subunit alpha 4(LAMA4)
M_34	187	apelin receptor(APLNR)
M_34	22936	elongation factor for RNA polymerase II 2(ELL2)
M_34	57125	plexin domain containing 1(PLXDC1)
M_34	57124	CD248 molecule(CD248)
M_34	6403	selectin P(SELP)
M_34	59345	G protein subunit beta 4(GNB4)
M_34	10750	GRB2-related adaptor protein(GRAP)
M_34	2828	G protein-coupled receptor 4(GPR4)
M_34	4121	mannosidase alpha class 1A member 1(MAN1A1)
M_34	7123	C-type lectin domain family 3 member B(CLEC3B)
M_34	9754	StAR related lipid transfer domain containing
M_34	4223	mesenchyme homeobox 2(MEOX2)
M_34	2321	fms related tyrosine kinase 1(FLT1)
M_34	51294	protocadherin 12(PCDH12)
M_34	10924	sphingomyelin phosphodiesterase acid like
M_34	7122	claudin 5(CLDN5)
M_34	81537	sphingosine-1-phosphate phosphatase 1(SGPP1)
M_34	7075	tyrosine kinase with immunoglobulin like and EGF like domains 1(TIE1)
M_34	57514	Rho GTPase activating protein 31(ARHGAP31)
M_34	3142	H2.0 like homeobox(HLX)
M_34	10501	semaphorin 6B(SEMA6B)

M_34	9507	ADAM metallopeptidase with thrombospondin type 1 motif 4(ADAMTS4)
M_34	3090	HIC ZBTB transcriptional repressor 1(HIC1)
M_34	8728	ADAM metallopeptidase domain 19(ADAM19)
M_34	2022	endoglin(ENG)
M_34	50509	collagen type V alpha 3 chain(COL5A3)
M_34	22899	Rho guanine nucleotide exchange factor
M_34	948	CD36 molecule(CD36)
M_34	54567	delta like canonical Notch ligand 4(DLL4)
M_34	6935	zinc finger E-box binding homeobox 1(ZEB1)
M_34	221395	adhesion G protein-coupled receptor F5(ADGRF5)
M_34	170954	protein phosphatase 1 regulatory subunit
M_34	286753	tumor suppressor candidate 5(TUSC5)
M_34	10268	receptor activity modifying protein 3(RAMP3)
M_34	90952	endothelial cell adhesion molecule(ESAM)
M_34	94	activin A receptor like type 1(ACVRL1)
M_34	8639	amine oxidase, copper containing 3(AOC3)
M_34	7035	tissue factor pathway inhibitor(TFPI)
M_34	6358	C-C motif chemokine ligand 14(CCL14)
M_34	1306	collagen type XV alpha 1 chain(COL15A1)
M_34	25960	adhesion G protein-coupled receptor A2(ADGRA2)
M_34	358	aquaporin 1 (Colton blood group)(AQP1)
M_34	8611	phospholipid phosphatase 1(PLPP1)
M_34	254228	family with sequence similarity 26 member
M_34	64123	adhesion G protein-coupled receptor L4(ADGRL4)
M_34	2167	fatty acid binding protein 4(FABP4)
M_34	11178	leucine zipper tumor suppressor 1(LZTS1)
M_34	64641	early B-cell factor 2(EBF2)
M_34	57381	ras homolog family member J(RHOJ)
M_34	116441	transmembrane 4 L six family member 18(TM4SF18)
M_34	4855	notch 4(NOTCH4)
M_34	3672	integrin subunit alpha 1(ITGA1)

M_34	22918	CD93 molecule(CD93)
M_34	6366	C-C motif chemokine ligand 21(CCL21)
M_34	10044	SH2 domain containing 3C(SH2D3C)
M_34	51313	family with sequence similarity 198 member
M_34	161198	C-type lectin domain family 14 member A(CLEC14A)
M_34	8490	regulator of G-protein signaling 5(RGS5)
M_34	90332	exocyst complex component 3 like 2(EXOC3L2)
M_34	114757	cytoglobin(CYGB)
M_34	6915	thromboxane A2 receptor(TBXA2R)
M_34	10203	calcitonin receptor like receptor(CALCRL)
M_34	5468	peroxisome proliferator activated receptor
M_34	5159	platelet derived growth factor receptor beta(PDGFRB)
M_34	1149	cell death-inducing DFFA-like effector a(CIDEA)
M_34	11117	elastin microfibril interfacer 1(EMILIN1)
M_34	1003	cadherin 5(CDH5)
M_34	283298	olfactomedin like 1(OLFML1)
M_34	167465	zinc finger protein 366(ZNF366)
M_34	79652	transmembrane protein 204(TMEM204)
M_34	125	alcohol dehydrogenase 1B (class I), beta
M_34	255877	B-cell CLL/lymphoma 6B(BCL6B)
M_34	11185	indolethylamine N-methyltransferase(INMT)
M_34	25927	cannabinoid receptor interacting protein 1(CNRIP1)
M_34	8578	scavenger receptor class F member 1(SCARF1)
M_34	22795	nidogen 2(NID2)
M_34	5787	protein tyrosine phosphatase, receptor type B(PTPRB)
M_34	51705	endomucin(EMCN)
M_34	118429	anthrax toxin receptor 2(ANTXR2)
M_34	5346	perilipin 1(PLIN1)
M_34	5175	platelet and endothelial cell adhesion molecule
M_34	132720	chromosome 4 open reading frame 32(C4orf32)
M_34	375033	platelet endothelial aggregation receptor 1(PEAR1)

M_34	641700	endothelial cell surface expressed chemotaxis and apoptosis regulator(ECSCR)
M_34	1901	sphingosine-1-phosphate receptor 1(S1PR1)
M_34	2869	G protein-coupled receptor kinase 5(GRK5)
M_34	7450	von Willebrand factor(VWF)
M_34	8404	SPARC like 1(SPARCL1)
M_34	10186	lipoma HMGIC fusion partner(LHFP)
M_34	392617	extracellular leucine rich repeat and fibronectin type III domain containing 1(ELFN1)
M_34	51267	C-type lectin domain family 1 member A(CLEC1A)
M_34	9370	adiponectin, C1Q and collagen domain
M_34	3791	kinase insert domain receptor(KDR)
M_34	126326	GIPC PDZ domain containing family member
M_34	79742	chromosome X open reading frame 36(CXorf36)
M_34	8291	dysferlin(DYSF)
M_34	9079	LIM domain binding 2(LDB2)
M_34	80177	myc target 1(MYCT1)
M_34	4811	nidogen 1(NID1)
M_34	2532	atypical chemokine receptor 1 (Duffy blood
M_34	83552	membrane frizzled-related protein(MFRP)
M_34	5414	septin 4(SEPT4)
M_34	10019	SH2B adaptor protein 3(SH2B3)
M_34	3952	leptin(LEP)
M_34	6886	TAL bHLH transcription factor 1, erythroid differentiation factor(TAL1)
M_34	10154	plexin C1(PLXNC1)
M_34	4017	lysyl oxidase like 2(LOXL2)
M_34	1601	DAB2, clathrin adaptor protein(DAB2)
M_34	9936	CD302 molecule(CD302)
M_34	285	angiopoietin 2(ANGPT2)
M_34	2078	ERG, ETS transcription factor(ERG)
M_34	7045	transforming growth factor beta induced(TGFBI)

M_34	2202	EGF containing fibulin like extracellular matrix protein 1(EFEMP1)
M_34	1359	carboxypeptidase A3(CPA3)
M_34	27115	phosphodiesterase 7B(PDE7B)
M_34	5138	phosphodiesterase 2A(PDE2A)
M_34	29969	MyoD family inhibitor domain containing(MDFIC)
M_34	256949	KN motif and ankyrin repeat domains 3(KANK3)
M_34	9839	zinc finger E-box binding homeobox 2(ZEB2)
M_34	2040	stomatin(STOM)
M_34	11010	GLI pathogenesis related 1(GLIPR1)
M_34	81035	collectin subfamily member 12(COLEC12)
M_34	1511	cathepsin G(CTSG)
M_34	1464	chondroitin sulfate proteoglycan 4(CSPG4)
M_34	2162	coagulation factor XIII A chain(F13A1)
M_34	4094	MAF bZIP transcription factor(MAF)
M_34	8516	integrin subunit alpha 8(ITGA8)
M_34	23643	lymphocyte antigen 96(LY96)
M_34	552	arginine vasopressin receptor 1A(AVPR1A)
M_34	7010	TEK receptor tyrosine kinase(TEK)
M_34	2206	membrane spanning 4-domains A2(MS4A2)
M_34	6489	ST8 alpha-N-acetyl-neuraminide alpha-2,8-sialyltransferase 1(ST8SIA1)
M_34	84329	hydrogen voltage gated channel 1(HVCN1)
M_34	3929	lipopolysaccharide binding protein(LBP)
M_34	1282	collagen type IV alpha 1 chain(COL4A1)
M_34	1284	collagen type IV alpha 2 chain(COL4A2)
M_34	2113	ETS proto-oncogene 1, transcription factor(ETS1)
M_34	283537	solute carrier family 46 member 3(SLC46A3)
M_34	7056	thrombomodulin(THBD)
M_34	10184	lipoma HMGIC fusion partner-like 2(LHFPL2)
M_34	23452	angiopoietin like 2(ANGPTL2)
M_34	2350	folate receptor beta(FOLR2)

M_34	8862	apelin(APLN)
M_34	3678	integrin subunit alpha 5(ITGA5)
M_34	51751	HIG1 hypoxia inducible domain family member
M_34	63895	piezo type mechanosensitive ion channel component 2(PIEZO2)
M_34	126669	Src homology 2 domain containing E(SHE)
M_34	126668	tudor domain containing 10(TDRD10)
M_34	54331	G protein subunit gamma 2(GNG2)
M_34	122786	FERM domain containing 6(FRMD6)
M_34	6401	selectin E(SELE)
M_34	10990	leukocyte immunoglobulin like receptor B5(LILRB5)
M_34	7177	tryptase alpha/beta 1(TPSAB1)
M_34	221303	family with sequence similarity 162 member
M_34	115123	membrane associated ring-CH-type finger
M_34	4360	mannose receptor, C type 1(MRC1)
M_34	25900	intermediate filament family orphan 1(IFFO1)
M_34	947	CD34 molecule(CD34)
M_34	11031	RAB31, member RAS oncogene family(RAB31)
M_34	9060	3'-phosphoadenosine 5'-phosphosulfate synthase
M_34	11082	endothelial cell specific molecule 1(ESM1)
M_34	4208	myocyte enhancer factor 2C(MEF2C)
M_34	6357	C-C motif chemokine ligand 13(CCL13)
M_34	6362	C-C motif chemokine ligand 18(CCL18)
M_34	176	aggrecan(ACAN)
M_34	23430	tryptase delta 1(TPSD1)
M_34	57574	membrane associated ring-CH-type finger
M_34	143503	olfactory receptor family 51 subfamily E member
M_34	9194	solute carrier family 16 member 7(SLC16A7)
M_34	3603	interleukin 16(IL16)
M_34	30835	CD209 molecule(CD209)
M_34	9627	synuclein alpha interacting protein(SNCAIP)
M_34	64499	tryptase beta 2 (gene/pseudogene)(TPSB2)

M_34	940	CD28 molecule(CD28)
M_34	81285	olfactory receptor family 51 subfamily E member
M_35	84696	abhydrolase domain containing 1(ABHD1)
M_35	5820	Pvt1 oncogene (non-protein coding)(PVT1)
M_35	284353	NTPase, KAP family P-loop domain containing
M_35	25825	beta-site APP-cleaving enzyme 2(BACE2)
M_35	284486	thioesterase superfamily member 5(THEM5)
M_35	285596	family with sequence similarity 153 member
M_35	170691	ADAM metallopeptidase with thrombospondin type 1 motif 17(ADAMTS17)
M_35	57146	transmembrane protein 159(TMEM159)
M_35	120376	colorectal cancer associated 2(COLCA2)
M_35	26238	long intergenic non-protein coding RNA
M_35	1556	cytochrome P450 family 2 subfamily B member 7, pseudogene(CYP2B7P)
M_35	399948	colorectal cancer associated 1(COLCA1)
M_35	9413	family with sequence similarity 189 member
M_35	1572	cytochrome P450 family 2 subfamily F member
M_35	388182	spermatogenesis associated 41 (non-protein coding)(SPATA41)
M_35	148808	major facilitator superfamily domain containing
M_35	257019	FERM domain containing 3(FRMD3)
M_35	100191040	C2 calcium dependent domain containing
M_35	116933	CLRN1 antisense RNA 1(CLRN1-AS1)
M_35	7478	Wnt family member 8A(WNT8A)
M_35	653316	family with sequence similarity 153, member C, pseudogene(FAM153C)
M_35	284835	long intergenic non-protein coding RNA
M_35	202134	family with sequence similarity 153 member
M_35	10669	cell growth regulator with EF-hand domain
M_35	25787	DiGeorge syndrome critical region gene 9 (non-protein coding)(DGCR9)

M_35	349565	nicotinamide nucleotide adenylyltransferase
M_35	100128782	long intergenic non-protein coding RNA
M_35	22881	ankyrin repeat domain 6(ANKRD6)
M_35	284578	uncharacterized LOC284578(LOC284578)
M_35	7401	clarin 1(CLRN1)
M_35	4609	v-myc avian myelocytomatosis viral oncogene
M_35	3795	ketohexokinase(KHK)
M_35	729234	fumarylacetoacetate hydrolase domain containing 2C, pseudogene(FAHD2CP)
M_35	100132111	uncharacterized LOC100132111(LOC100132111)
M_35	1553	cytochrome P450 family 2 subfamily A member 13(CYP2A13)
M_35	1548	cytochrome P450 family 2 subfamily A member
M_35	1549	cytochrome P450 family 2 subfamily A member
M_35	347088	adhesion G protein-coupled receptor D2(ADGRD2)
M_35	26222	DiGeorge syndrome critical region gene 10 (non-protein coding)(DGCR10)
M_35	3749	potassium voltage-gated channel subfamily C member 4(KCNC4)
M_35	151313	fumarylacetoacetate hydrolase domain containing 2B(FAHD2B)
M_35	254122	sorting nexin 32(SNX32)
M_35	204219	ceramide synthase 3(CERS3)
M_35	563	alpha-2-glycoprotein 1, zinc-binding(AZGP1)
M_35	83998	regenerating family member 4(REG4)
M_35	4013	von Willebrand factor A domain containing
M_35	91133	l(3)mbt-like 4 (Drosophila)(L3MBTL4)
M_35	7380	uroplakin 3A(UPK3A)
M_35	6920	transcription elongation factor A3(TCEA3)
M_35	140691	tripartite motif containing 69(TRIM69)
M_35	7955	RNF217 antisense RNA 1 (head to head)(RNF217-
M_35	7200	thyrotropin releasing hormone(TRH)

M_35	84983	FAM222A antisense RNA 1(FAM222A-AS1)
M_35	390072	olfactory receptor family 52 subfamily N member 4 (gene/pseudogene)(OR52N4)
M_35	11255	histamine receptor H3(HRH3)
M_35	57415	chromosome 3 open reading frame 14(C3orf14)
M_35	154214	ring finger protein 217(RNF217)
M_35	387748	olfactory receptor family 56 subfamily B member
M_35	197135	PAT1 homolog 2(PATL2)
M_35	11166	SRY-box 21(SOX21)
M_35	2253	fibroblast growth factor 8(FGF8)
M_35	8820	HESX homeobox 1(HESX1)
M_36	84895	mitoguardin 2(MIGA2)
M_36	57109	REX4 homolog, 3'-5' exonuclease(REXO4)
M_36	138162	chromosome 9 open reading frame 116(C9orf116)
M_36	5524	protein phosphatase 2 phosphatase activator(PTPA)
M_36	138428	peptidyl-tRNA hydrolase 1 homolog(PTRH1)
M_36	23511	nucleoporin 188(NUP188)
M_36	116225	zinc finger MYND-type containing 19(ZMYND19)
M_36	23203	peptidase, mitochondrial processing alpha
M_36	29988	solute carrier family 2 member 8(SLC2A8)
M_36	11092	sperm acrosome associated 9(SPACA9)
M_36	11094	calcium channel flower domain containing
M_36	29085	phosphohistidine phosphatase 1(PHPT1)
M_36	266655	long intergenic non-protein coding RNA
M_36	54662	TBC1 domain family member 13(TBC1D13)
M_36	158067	adenylate kinase 8(AK8)
M_36	399665	family with sequence similarity 102 member
M_36	2889	Rap guanine nucleotide exchange factor 1(RAPGEF1)
M_36	92715	diphthamide biosynthesis 7(DPH7)
M_36	64975	mitochondrial ribosomal protein L41(MRPL41)
M_36	10383	tubulin beta 4B class IVb(TUBB4B)
M_36	8021	nucleoporin 214(NUP214)

M_36	10585	protein O-mannosyltransferase 1(POMT1)
M_36	8019	bromodomain containing 3(BRD3)
M_36	6130	ribosomal protein L7a(RPL7A)
M_36	286257	chromosome 9 open reading frame 142(C9orf142)
M_36	7248	tuberous sclerosis 1(TSC1)
M_36	29882	anaphase promoting complex subunit 2(ANAPC2)
M_36	8721	endothelial differentiation related factor 1(EDF1)
M_36	6836	surfeit 4(SURF4)
M_36	5900	ral guanine nucleotide dissociation
M_36	4702	NADH:ubiquinone oxidoreductase subunit
M_36	84885	zinc finger DHHC-type containing 12(ZDHHC12)
M_36	1861	torsin family 1 member A(TOR1A)
M_36	26995	TruB pseudouridine synthase family member
M_36	57171	dolichyldiphosphatase 1(DOLPP1)
M_36	25792	CDKN1A interacting zinc finger protein 1(CIZ1)
M_36	143241	DPY30 domain containing 1(DYDC1)
M_36	94107	transmembrane protein 203(TMEM203)
M_36	84332	DPY30 domain containing 2(DYDC2)
M_36	85026	ARRDC1 antisense RNA 1(ARRDC1-AS1)
M_36	54461	F-box and WD repeat domain containing 5(FBXW5)
M_36	11091	WD repeat domain 5(WDR5)
M_36	9919	SEC16 homolog A, endoplasmic reticulum export factor(SEC16A)
M_36	10422	UBA domain containing 1(UBAC1)
M_36	85014	transmembrane protein 141(TMEM141)
M_36	2733	GLE1, RNA export mediator(GLE1)
M_36	84726	proline rich coiled-coil 2B(PRRC2B)
M_36	55684	RAB, member RAS oncogene family-like 6(RABL6)
M_36	8939	far upstream element binding protein 3(FUBP3)
M_36	79095	chromosome 9 open reading frame 16(C9orf16)
M_36	25	ABL proto-oncogene 1, non-receptor tyrosine
M_36	92399	mitochondrial ribosome recycling factor(MRRF)

M_36	8636	SS nuclear autoantigen 1(SSNA1)
M_36	7633	zinc finger protein 79(ZNF79)
M_36	56262	leucine rich repeat containing 8 family member
M_36	80142	prostaglandin E synthase 2(PTGES2)
M_36	9442	mediator complex subunit 27(MED27)
M_36	11224	ribosomal protein L35(RPL35)
M_36	64794	DEAD-box helicase 31(DDX31)
M_36	83549	uridine-cytidine kinase 1(UCK1)
M_36	5695	proteasome subunit beta 7(PSMB7)
M_36	57720	G protein-coupled receptor 107(GPR107)
M_36	64855	family with sequence similarity 129 member
M_36	2021	endonuclease G(ENDOG)
M_36	8818	dolichyl-phosphate mannosyltransferase subunit 2, regulatory(DPM2)
M_36	6621	small nuclear RNA activating complex polypeptide 4(SNAPC4)
M_36	157922	calmodulin regulated spectrin associated protein 1(CAMSAP1)
M_36	1025	cyclin dependent kinase 9(CDK9)
M_36	10244	Rab9 effector protein with kelch motifs(RABEPK)
M_36	27090	ST6 N-acetylgalactosaminide alpha-2,6-sialyltransferase 4(ST6GALNAC4)
M_36	30815	ST6 N-acetylgalactosaminide alpha-2,6-sialyltransferase 6(ST6GALNAC6)
M_36	22845	dolichol kinase(DOLK)
M_36	79813	euchromatic histone lysine methyltransferase
M_36	728489	DNL-type zinc finger(DNLZ)
M_36	140459	ankyrin repeat and SOCS box containing 6(ASB6)
M_36	27158	NADPH dependent diflavin oxidoreductase
M_36	28989	N-terminal Xaa-Pro-Lys N-methyltransferase
M_36	6834	SURF1, cytochrome c oxidase assembly
M_36	10999	solute carrier family 27 member 4(SLC27A4)

M_36	6835	surfeit 2(SURF2)
M_36	6838	surfeit 6(SURF6)
M_36	6837	mediator complex subunit 22(MED22)
M_36	89891	WD repeat domain 34(WDR34)
M_36	81605	ubiquitin related modifier 1(URM1)
M_36	25920	negative elongation factor complex member
M_36	2356	folylpolyglutamate synthase(FPGS)
M_36	9329	general transcription factor IIIC subunit 4(GTF3C4)
M_36	4957	outer dense fiber of sperm tails 2(ODF2)
M_36	51490	SPOUT domain containing methyltransferase
M_36	7186	TNF receptor associated factor 2(TRAF2)
M_36	23064	senataxin(SETX)
M_36	203	adenylate kinase 1(AK1)
M_36	92714	arrestin domain containing 1(ARRDC1)
M_36	51759	chromosome 9 open reading frame 78(C9orf78)
M_36	27433	torsin family 2 member A(TOR2A)
M_36	10555	1-acylglycerol-3-phosphate O-acyltransferase
M_36	23404	exosome component 2(EXOSC2)
M_36	6418	SET nuclear proto-oncogene(SET)
M_36	11253	mannosidase alpha class 1B member 1(MAN1B1)
M_36	56904	SH3 domain containing GRB2 like endophilin
M_36	51117	coenzyme Q4(COQ4)
M_36	441089	mediator complex subunit 27 pseudogene(CRSP8P)
M_36	10868	ubiquitin specific peptidase 20(USP20)
M_36	56623	inositol polyphosphate-5-phosphatase E(INPP5E)
M_36	10444	zyg-11 related cell cycle regulator(ZER1)
M_36	81873	actin related protein 2/3 complex subunit 5
M_36	7270	transcription termination factor 1(TTF1)
M_36	9328	general transcription factor IIIC subunit 5(GTF3C5)
M_36	90120	chromosome 9 open reading frame 69(C9orf69)
M_36	51116	mitochondrial ribosomal protein S2(MRPS2)

M_36	90678	leucine rich repeat and sterile alpha motif containing 1(LRSAM1)
M_36	9858	protein phosphatase 1 regulatory subunit
M_36	10807	serologically defined colon cancer antigen
M_36	114789	solute carrier family 25 member 25(SLC25A25)
M_36	27348	torsin family 1 member B(TOR1B)
M_36	286262	taperin(TPRN)
M_36	29952	dipeptidyl peptidase 7(DPP7)
M_36	158055	chromosome 9 open reading frame 163(C9orf163)
M_37	84964	alkB homolog 6(ALKBH6)
M_37	6183	mitochondrial ribosomal protein S12(MRPS12)
M_37	1891	enoyl-CoA hydratase 1(ECH1)
M_37	778	calcium voltage-gated channel subunit alpha1
M_37	5714	proteasome 26S subunit, non-ATPase 8(PSMD8)
M_37	163126	EP300 interacting inhibitor of differentiation 2(EID2)
M_37	10298	p21 (RAC1) activated kinase 4(PAK4)
M_37	22933	sirtuin 2(SIRT2)
M_37	90522	Yip1 interacting factor homolog B, membrane trafficking protein(YIF1B)
M_37	84807	NFKB inhibitor delta(NFKBID)
M_37	10430	transmembrane protein 147(TMEM147)
M_37	23354	HAUS augmin like complex subunit 5(HAUS5)
M_37	6217	ribosomal protein S16(RPS16)
M_37	6829	SPT5 homolog, DSIF elongation factor
M_37	1155	tubulin folding cofactor B(TBCB)
M_37	5704	proteasome 26S subunit, ATPase 4(PSMC4)
M_37	4438	mutS homolog 4(MSH4)
M_37	92609	translocase of inner mitochondrial membrane
M_37	126433	F-box protein 27(FBXO27)
M_37	199720	gametogenetin(GGN)
M_37	147968	calpain 12(CAPN12)
M_37	147965	family with sequence similarity 98 member

M_37	27335	eukaryotic translation initiation factor 3 subunit
M_37	2091	fibrillarin(FBL)
M_37	11184	mitogen-activated protein kinase kinase kinase kinase 1(MAP4K1)
M_37	826	calpain small subunit 1(CAPNS1)
M_37	54623	PAF1 homolog, Paf1/RNA polymerase II complex component(PAF1)
M_37	54938	seryl-tRNA synthetase 2, mitochondrial(SARS2)
M_37	199746	U2 small nuclear RNA auxiliary factor 1 like
M_37	23094	signal induced proliferation associated 1 like
M_37	9149	dual specificity tyrosine phosphorylation regulated kinase 1B(DYRK1B)
M_37	55957	lin-37 DREAM MuvB core complex
M_37	9757	lysine methyltransferase 2B(KMT2B)
M_37	6261	ryanodine receptor 1(RYR1)
M_37	55095	sterile alpha motif domain containing 4B(SAMD4B)
M_37	115290	F-box protein 17(FBXO17)
M_37	4793	NFKB inhibitor beta(NFKBIB)
M_37	10653	serine peptidase inhibitor, Kunitz type 2(SPINT2)
M_37	399473	sprouty related EVH1 domain containing 3(SPRED3)
M_37	79171	RNA binding motif protein 42(RBM42)
M_37	112755	syntaxin 1B(STX1B)
M_37	81	actinin alpha 4(ACTN4)
M_37	55588	mediator complex subunit 29(MED29)
M_37	57828	cation channel sperm associated auxiliary subunit gamma(CATSPERG)
M_37	115703	Rho GTPase activating protein 33(ARHGAP33)
M_37	644096	succinate dehydrogenase complex assembly factor 1(SDHAF1)
M_37	55851	presenilin enhancer gamma-secretase
M_37	3191	heterogeneous nuclear ribonucleoprotein L(HNRNPL)
M_37	148137	proline and serine rich 3(PROSER3)

M_37	64857	pleckstrin homology and RhoGEF domain containing G2(PLEKHG2)
M_37	3960	galectin 4(LGALS4)
M_37	57622	leucine rich repeat and fibronectin type III domain containing 1(LRFN1)
M_37	3766	potassium voltage-gated channel subfamily J member 10(KCNJ10)
M_37	126272	EP300 interacting inhibitor of differentiation
M_37	6490	premelanosome protein(PMEL)
M_37	2116	ETS variant 2(ETV2)
M_37	7643	zinc finger protein 90(ZNF90)
M_38	85478	coiled-coil domain containing 65(CCDC65)
M_38	200403	von Willebrand factor A domain containing
M_38	84698	calcyphosine 2(CAPS2)
M_38	4117	male germ cell associated kinase(MAK)
M_38	168090	chromosome 6 open reading frame 118(C6orf118)
M_38	134121	chromosome 5 open reading frame 49(C5orf49)
M_38	120379	PIH1 domain containing 2(PIH1D2)
M_38	83450	dynein regulatory complex subunit 3(DRC3)
M_38	57562	centrosomal protein 126(CEP126)
M_38	92565	fibronectin type III and ankyrin repeat domains
M_38	254956	MORN repeat containing 5(MORN5)
M_38	56964	WD repeat domain 93(WDR93)
M_38	345895	radial spoke head 4 homolog A(RSPH4A)
M_38	374467	#N/A
M_38	129852	chromosome 2 open reading frame 73(C2orf73)
M_38	154865	IQ motif and ubiquitin domain containing(IQUB)
M_38	286207	cilia and flagella associated protein 157(CFAP157)
M_38	728621	coiled-coil domain containing 30(CCDC30)
M_38	27019	dynein axonemal intermediate chain 1(DNAI1)
M_38	339416	ankyrin repeat domain 45(ANKRD45)
M_38	130752	malate dehydrogenase 1B(MDH1B)

M_38	286187	protein phosphatase 1 regulatory subunit
M_38	123872	dynein axonemal assembly factor 1(DNAAF1)
M_38	85016	chromosome 11 open reading frame 70(C11orf70)
M_38	154313	cilia and flagella associated protein 206(CFAP206)
M_38	128153	spermatogenesis associated 17(SPATA17)
M_38	89876	MYCBP associated and testis expressed 1(MAATS1)
M_38	219670	enkurin, TRPC channel interacting protein(ENKUR)
M_38	219681	armadillo repeat containing 3(ARMC3)
M_38	136288	chromosome 7 open reading frame 57(C7orf57)
M_38	401551	WD repeat domain 38(WDR38)
M_38	149465	cilia and flagella associated protein 57(CFAP57)
M_38	55130	armadillo repeat containing 4(ARMC4)
M_38	92340	proline rich 29(PRR29)
M_38	126820	WD repeat domain 63(WDR63)
M_38	285755	peptidylprolyl isomerase like 6(PPIL6)
M_38	26074	cilia and flagella associated protein 61(CFAP61)
M_38	53340	sperm autoantigenic protein 17(SPA17)
M_38	79858	NIMA related kinase 11(NEK11)
M_38	83659	tektin 1(TEKT1)
M_38	348807	cilia and flagella associated protein 100(CFAP100)
M_38	152110	NIMA related kinase 10(NEK10)
M_38	4867	nephrocystin 1(NPHP1)
M_38	84125	leucine rich repeats and IQ motif containing
M_38	127733	UBX domain protein 10(UBXN10)
M_38	80217	cilia and flagella associated protein 43(CFAP43)
M_38	2302	forkhead box J1(FOXJ1)
M_38	83853	rhophilin associated tail protein 1 like(ROPN1L)
M_38	89765	radial spoke head 1 homolog(RSPH1)
M_38	51364	zinc finger MYND-type containing 10(ZMYND10)
M_38	55036	coiled-coil domain containing 40(CCDC40)
M_38	83538	tetratricopeptide repeat domain 25(TTC25)
M_38	129881	coiled-coil domain containing 173(CCDC173)

M_38	54768	HYDIN, axonemal central pair apparatus
M_38	26206	sperm associated antigen 8(SPAG8)
M_38	440585	family with sequence similarity 183 member
M_38	90506	leucine rich repeat containing 46(LRRC46)
M_38	220136	cilia and flagella associated protein 53(CFAP53)
M_38	387885	cilia and flagella associated protein 73(CFAP73)
M_38	164395	tubulin tyrosine ligase like 9(TTLL9)
M_38	257177	cilia and flagella associated protein 126(CFAP126)
M_38	1768	dynein axonemal heavy chain 6(DNAH6)
M_38	92749	dynein regulatory complex subunit 1(DRC1)
M_38	257236	coiled-coil domain containing 96(CCDC96)
M_38	127003	chromosome 1 open reading frame 194(C1orf194)
M_38	158787	RIB43A domain with coiled-coils 1(RIBC1)
M_38	27285	tektin 2(TEKT2)
M_38	153643	family with sequence similarity 81 member
M_38	55259	cancer susceptibility candidate 1(CASC1)
M_38	93190	chromosome 1 open reading frame 158(C1orf158)
M_38	25790	cilia and flagella associated protein 45(CFAP45)
M_38	128344	primary cilia formation(PIFO)
M_38	114327	EF-hand domain containing 1(EFHC1)
M_38	79740	zinc finger B-box domain containing(ZBBX)
M_38	56171	dynein axonemal heavy chain 7(DNAH7)
M_38	9940	deleted in lung and esophageal cancer 1(DLEC1)
M_38	25876	sperm flagellar 1(SPEF1)
M_38	146177	von Willebrand factor A domain containing
M_38	139212	PIH1 domain containing 3(PIH1D3)
M_38	389799	cilia and flagella associated protein 77(CFAP77)
M_38	390205	leucine rich repeat containing 10B(LRRC10B)
M_38	118491	cilia and flagella associated protein 70(CFAP70)
M_38	400891	leucine rich repeat containing 74B(LRRC74B)
M_38	1770	dynein axonemal heavy chain 9(DNAH9)
M_38	153657	tetratricopeptide repeat domain 23 like(TTC23L)

M_38	161835	fibrous sheath interacting protein 1(FSIP1)
M_38	129880	Bardet-Biedl syndrome 5(BBS5)
M_38	10324	kelch like family member 41(KLHL41)
M_38	79925	sperm flagellar 2(SPEF2)
M_38	79645	EF-hand calcium binding domain 1(EFCAB1)
M_38	6103	retinitis pigmentosa GTPase regulator(RPGR)
M_38	83894	tetratricopeptide repeat domain 29(TTC29)
M_38	133690	calcyphosine like(CAPSL)
M_39	113655	major facilitator superfamily domain containing
M_39	23513	scribbled planar cell polarity protein(SCRIB)
M_39	84875	poly(ADP-ribose) polymerase family member
M_39	414919	chromosome 8 open reading frame 82(C8orf82)
M_39	90987	zinc finger protein 251(ZNF251)
M_39	116447	topoisomerase (DNA) I, mitochondrial(TOP1MT)
M_39	1936	eukaryotic translation elongation factor 1
M_39	727957	maestro heat like repeat family member 1(MROH1)
M_39	340371	nuclear receptor binding protein 2(NRBP2)
M_39	65263	pyrroline-5-carboxylate reductase-like(PYCRL)
M_39	79581	solute carrier family 52 member 2(SLC52A2)
M_39	286077	family with sequence similarity 83 member
M_39	54512	exosome component 4(EXOSC4)
M_39	28991	COMM domain containing 5(COMMD5)
M_39	8694	diacylglycerol O-acyltransferase 1(DGAT1)
M_39	2738	GLI family zinc finger 4(GLI4)
M_39	286101	zinc finger protein 252, pseudogene(ZNF252P)
M_39	5339	plectin(PLEC)
M_39	26233	F-box and leucine rich repeat protein 6(FBXL6)
M_39	7564	zinc finger protein 16(ZNF16)
M_39	2907	glutamate ionotropic receptor NMDA type subunit associated protein 1(GRINA)
M_39	7553	zinc finger protein 7(ZNF7)
M_39	6132	ribosomal protein L8(RPL8)

M_39	286075	zinc finger protein 707(ZNF707)
M_39	79792	gasdermin D(GSDMD)
M_39	51236	HGH1 homolog(HGH1)
M_39	50626	cysteine and histidine rich 1(CYHR1)
M_39	58500	zinc finger protein 250(ZNF250)
M_39	3297	heat shock transcription factor 1(HSF1)
M_39	29894	cleavage and polyadenylation specific factor
M_39	79943	zinc finger protein 696(ZNF696)
M_39	84988	protein phosphatase 1 regulatory subunit
M_39	23144	zinc finger CCCH-type containing 3(ZC3H3)
M_39	23246	block of proliferation 1(BOP1)
M_39	51160	VPS28, ESCRT-I subunit(VPS28)
M_39	51337	thioesterase superfamily member 6(THEM6)
M_39	22827	poly(U) binding splicing factor 60(PUF60)
M_39	84232	MAF1 homolog, negative regulator of RNA polymerase III(MAF1)
M_39	1537	cytochrome c1(CYC1)
M_39	286128	ZFP41 zinc finger protein(ZFP41)
M_39	83696	trafficking protein particle complex 9(TRAPPC9)
M_39	90990	kinesin family member C2(KIFC2)
M_39	9831	zinc finger protein 623(ZNF623)
M_39	203062	t-SNARE domain containing 1(TSNARE1)
M_39	340385	zinc finger protein 517(ZNF517)
M_39	81858	SHANK associated RH domain interactor(SHARPIN)
M_39	114822	rhophilin Rho GTPase binding protein 1(RHPN1)
M_39	80728	Rho GTPase activating protein 39(ARHGAP39)
M_39	9684	leucine rich repeat containing 14(LRRC14)
M_39	8733	glycosylphosphatidylinositol anchor attachment
M_39	7264	tissue specific transplantation antigen P35B(TSTA3)
M_39	203054	aarF domain containing kinase 5(ADCK5)
M_39	80778	zinc finger protein 34(ZNF34)
M_39	78998	RHPN1 antisense RNA 1 (head to head)(RHPN1-

M_39	225689	mitogen-activated protein kinase 15(MAPK15)
M_39	57210	solute carrier family 45 member 4(SLC45A4)
M_39	8629	Jrk helix-turn-helix protein(JRK)
M_39	286102	transmembrane p24 trafficking protein 10 pseudogene 1(TMED10P1)
M_39	26873	5-oxoprolinase (ATP-hydrolysing)(OPLAH)
M_39	5747	protein tyrosine kinase 2(PTK2)
M_39	65265	chromosome 8 open reading frame 33(C8orf33)
M_39	7318	ubiquitin like modifier activating enzyme 7(UBA7)
M_39	83481	epiplakin 1(EPPK1)
M_39	84948	tigger transposable element derived 5(TIGD5)
M_39	286076	breast cancer estrogen-induced apoptosis 2(BREA2)
M_39	23639	leucine rich repeat containing 6(LRRC6)
M_39	340390	WD repeat domain 97(WDR97)
M_39	255352	#N/A
M_39	55337	chromosome 19 open reading frame 66(C19orf66)
M_39	84941	hematopoietic SH2 domain containing(HSH2D)
M_40	116150	NUS1 dehydrodolichyl diphosphate synthase
M_40	51696	hdc homolog, cell cycle regulator(HECA)
M_40	5238	phosphoglucomutase 3(PGM3)
M_40	57120	golgi associated PDZ and coiled-coil motif
M_40	5134	programmed cell death 2(PDCD2)
M_40	23036	zinc finger protein 292(ZNF292)
M_40	83443	splicing factor 3b subunit 5(SF3B5)
M_40	202	absent in melanoma 1(AIM1)
M_40	26235	F-box and leucine rich repeat protein 4(FBXL4)
M_40	9841	zinc finger and BTB domain containing 24(ZBTB24)
M_40	9324	high mobility group nucleosomal binding domain
M_40	55788	LMBR1 domain containing 1(LMBRD1)
M_40	57515	serine incorporator 1(SERINC1)
M_40	7259	TSPY like 1(TSPYL1)
M_40	2519	fucosidase, alpha-L- 2, plasma(FUCA2)

M_40	55122	akirin 2(AKIRIN2)
M_40	113402	SFT2 domain containing 1(SFT2D1)
M_40	10767	HBS1 like translational GTPase(HBS1L)
M_40	28962	osteopetrosis associated transmembrane protein
M_40	387119	centrosomal protein 85 like(CEP85L)
M_40	23195	midasin AAA ATPase 1(MDN1)
M_40	54516	mitochondrial translational release factor 1
M_40	57673	BEN domain containing 3(BEND3)
M_40	387263	chromosome 6 open reading frame 120(C6orf120)
M_40	55389	#N/A
M_40	10973	activating signal cointegrator 1 complex subunit
M_40	85021	RALBP1 associated Eps domain containing 1(REPS1)
M_40	51805	coenzyme Q3, methyltransferase(COQ3)
M_40	84816	reticulon 4 interacting protein 1(RTN4IP1)
M_40	85015	ubiquitin specific peptidase 45(USP45)
M_40	3066	histone deacetylase 2(HDAC2)
M_40	51390	androgen induced 1(AIG1)
M_40	55780	ER membrane associated RNA
M_40	51389	RWD domain containing 1(RWDD1)
M_40	729515	transmembrane protein 242(TMEM242)
M_40	23376	UFM1 specific ligase 1(UFL1)
M_40	8504	peroxisomal biogenesis factor 3(PEX3)
M_40	84071	armadillo repeat containing 2(ARMC2)
M_40	79624	acidic residue methyltransferase 1(ARMT1)
M_40	5689	proteasome subunit beta 1(PSMB1)
M_40	594	branched chain keto acid dehydrogenase E1 subunit beta(BCKDHB)
M_40	55023	pleckstrin homology domain interacting protein(PHIP)
M_40	404672	general transcription factor IIH subunit 5(GTF2H5)
M_40	55278	glutaminyl-tRNA synthase (glutamine-hydrolyzing)-like 1(QRSL1)
M_40	134637	adenosine deaminase, tRNA specific 2(ADAT2)

M_40	25821	mitochondrial tRNA translation optimization
M_40	100133941	CD24 molecule(CD24)
M_40	92521	sperm antigen with calponin homology and coiled-coil domains 1(SPECC1)
M_40	23033	dopey family member 1(DOPEY1)
M_40	90025	ubiquitin protein ligase E3D(UBE3D)
M_40	57492	AT-rich interaction domain 1B(ARID1B)
M_40	7803	protein tyrosine phosphatase type IVA, member
M_40	60487	tRNA methyltransferase 11 homolog(TRMT11)
M_40	9113	large tumor suppressor kinase 1(LATS1)
M_40	9439	mediator complex subunit 23(MED23)
M_40	57579	family with sequence similarity 135 member
M_40	254394	minichromosome maintenance 9 homologous recombination repair factor(MCM9)
M_40	57583	transmembrane protein 181(TMEM181)
M_40	10559	solute carrier family 35 member A1(SLC35A1)
M_40	9774	BCL2 associated transcription factor 1(BCLAF1)
M_40	58527	ABRA C-terminal like(ABRACL)
M_40	22832	centrosomal protein 162(CEP162)
M_40	22828	SR-related CTD associated factor 8(SCAF8)
M_40	25957	PNN interacting serine and arginine rich
M_40	348995	nucleoporin 43(NUP43)
M_40	79694	mannosidase endo-alpha(MANEA)
M_40	55754	transmembrane protein 30A(TMEM30A)
M_40	7402	utrophin(UTRN)
M_40	9994	caspase 8 associated protein 2(CASP8AP2)
M_40	5191	peroxisomal biogenesis factor 7(PEX7)
M_40	60682	small ArfGAP 1(SMAP1)
M_40	5110	protein-L-isoaspartate (D-aspartate) O-methyltransferase(PCMT1)
M_40	2309	forkhead box O3(FOXO3)

M_40	5980	REV3 like, DNA directed polymerase zeta catalytic subunit(REV3L)
M_40	6908	TATA-box binding protein(TBP)
M_40	51250	chromosome 6 open reading frame 203(C6orf203)
M_40	57226	LYR motif containing 2(LYRM2)
M_40	23593	heme binding protein 2(HEBP2)
M_40	3298	heat shock transcription factor 2(HSF2)
M_40	6993	dynein light chain Tctex-type 1(DYNLT1)
M_40	9519	TATA-box binding protein like 1(TBPL1)
M_40	25998	inhibitor of Bruton tyrosine kinase(IBTK)
M_40	51167	cytochrome b5 reductase 4(CYB5R4)
M_40	3841	karyopherin subunit alpha 5(KPNA5)
M_40	51660	mitochondrial pyruvate carrier 1(MPC1)
M_40	23270	TSPY like 4(TSPYL4)
M_40	221294	5'-nucleotidase domain containing 1(NT5DC1)
M_40	221302	zinc finger with UFM1 specific peptidase
M_40	55274	PHD finger protein 10(PHF10)
M_40	221264	adenylate kinase 9(AK9)
M_40	81847	ring finger protein 146(RNF146)
M_40	29074	mitochondrial ribosomal protein L18(MRPL18)
M_40	221322	TBC1 domain family member 32(TBC1D32)
M_40	29078	NADH:ubiquinone oxidoreductase complex assembly factor 4(NDUFAF4)
M_40	8417	syntaxin 7(STX7)
M_40	7164	tumor protein D52-like 1(TPD52L1)
M_40	9589	Wilms tumor 1 associated protein(WTAP)
M_40	57038	arginyl-tRNA synthetase 2, mitochondrial(RARS2)
M_40	3097	human immunodeficiency virus type I enhancer binding protein 2(HIVEP2)
M_40	8724	sorting nexin 3(SNX3)
M_40	262	adenosylmethionine decarboxylase 1(AMD1)
M_40	23469	PHD finger protein 3(PHF3)

M_40	25842	anti-silencing function 1A histone chaperone(ASF1A)
M_40	3482	insulin like growth factor 2 receptor(IGF2R)
M_40	112611	RWD domain containing 2A(RWDD2A)
M_40	892	cyclin C(CCNC)
M_40	112495	general transcription factor IIIC subunit 6(GTF3C6)
M_40	54806	Abelson helper integration site 1(AHI1)
M_40	23097	cyclin dependent kinase 19(CDK19)
M_40	135114	histidine triad nucleotide binding protein 3(HINT3)
M_40	51175	tubulin epsilon 1(TUBE1)
M_40	134728	interleukin 1 receptor associated kinase 1 binding protein 1(IRAK1BP1)
M_40	2310	forkhead box O3B pseudogene(FOXO3B)
M_40	84498	family with sequence similarity 120B(FAM120B)
M_40	57107	prenyl (decaprenyl) diphosphate synthase, subunit
M_40	11231	SEC63 homolog, protein translocation
M_40	51465	ubiquitin conjugating enzyme E2 J1(UBE2J1)
M_40	8732	RNA guanylyltransferase and 5'-
M_40	9896	FIG4 phosphoinositide 5-phosphatase(FIG4)
M_40	8763	CD164 molecule(CD164)
M_40	51020	HD domain containing 2(HDDC2)
M_40	51534	vesicle trafficking 1(VTA1)
M_40	84154	ribosome production factor 2 homolog(RPF2)
M_40	84947	serine active site containing 1(SERAC1)
M_40	84946	LTV1 ribosome biogenesis factor(LTV1)
M_40	27244	sestrin 1(SESN1)
M_40	55005	required for meiotic nuclear division 1
M_40	285753	centrosomal protein 57 like 1(CEP57L1)
M_40	5550	prolyl endopeptidase(PREP)
M_40	56995	tubby like protein 4(TULP4)
M_40	7957	epilepsy, progressive myoclonus type 2A, Lafora disease (laforin)(EPM2A)
M_40	57231	sorting nexin 14(SNX14)

M_40	55862	ethylmalonyl-CoA decarboxylase 1(ECHDC1)
M_40	6610	sphingomyelin phosphodiesterase 2(SMPD2)
M_40	57150	small integral membrane protein 8(SMIM8)
M_40	91749	major facilitator superfamily domain containing
M_40	85313	peptidylprolyl isomerase like 4(PPIL4)
M_40	57480	pleckstrin homology and RhoGEF domain containing G1(PLEKHG1)
M_40	23595	origin recognition complex subunit 3(ORC3)
M_40	253714	MMS22 like, DNA repair protein(MMS22L)
M_40	116254	glycoprotein integral membrane 1(GINM1)
M_40	4216	mitogen-activated protein kinase kinase kinase
M_40	257218	SNF2 histone linker PHD RING helicase(SHPRH)
M_40	83861	radial spoke 3 homolog(RSPH3)
M_40	11116	FGFR1 oncogene partner(FGFR1OP)
M_40	135293	peptidase M20 domain containing 2(PM20D2)
M_40	11104	katanin catalytic subunit A1(KATNA1)
M_40	6950	t-complex 1(TCP1)
M_40	246269	lactation elevated 1(LACE1)
M_40	6885	mitogen-activated protein kinase kinase kinase
M_40	23118	TGF-beta activated kinase 1/MAP3K7 binding protein 2(TAB2)
M_40	10957	proline rich nuclear receptor coactivator 1(PNRC1)
M_40	84085	F-box protein 30(FBXO30)
M_40	57621	zinc finger and BTB domain containing 2(ZBTB2)
M_40	10492	synaptotagmin binding cytoplasmic RNA interacting protein(SYNCRIP)
M_40	9474	autophagy related 5(ATG5)
M_40	51362	cell division cycle 40(CDC40)
M_40	57531	HECT domain and ankyrin repeat containing E3 ubiquitin protein ligase 1(HACE1)
M_40	26054	SUMO1/sentrin specific peptidase 6(SENP6)
M_40	3592	interleukin 12A(IL12A)

M_40	135154	succinate dehydrogenase complex assembly factor 4(SDHAF4)
M_40	135152	beta-1,3-glucuronyltransferase 2(B3GAT2)
M_40	729603	calcineurin like EF-hand protein 1
M_40	10981	RAB32, member RAS oncogene family(RAB32)
M_40	25902	methylenetetrahydrofolate dehydrogenase (NADP+ dependent) 1-like(MTHFD1L)
M_40	63914	long intergenic non-protein coding RNA
M_40	3459	interferon gamma receptor 1(IFNGR1)
M_40	39	acetyl-CoA acetyltransferase 2(ACAT2)
M_40	56648	eukaryotic translation initiation factor 5A2(EIF5A2)
M_40	8871	synaptojanin 2(SYNJ2)
M_40	25901	coiled-coil domain containing 28A(CCDC28A)
M_40	91614	DEP domain containing 7(DEPDC7)
M_40	51106	transcription factor B1, mitochondrial(TFB1M)
M_40	346007	eyes shut homolog (Drosophila)(EYS)
M_40	206412	chromosome 6 open reading frame 163(C6orf163)
M_41	118472	zinc finger protein 511(ZNF511)
M_41	55760	DEAH-box helicase 32 (putative)(DHX32)
M_41	10539	glutaredoxin 3(GLRX3)
M_41	54764	zinc finger RANBP2-type containing 1(ZRANB1)
M_41	55717	WD repeat domain 11(WDR11)
M_41	9184	BUB3, mitotic checkpoint protein(BUB3)
M_41	22876	inositol polyphosphate-5-phosphatase F(INPP5F)
M_41	198437	LKAAEAR motif containing 1(LKAAEAR1)
M_41	2832	neuropeptides B/W receptor 2(NPBWR2)
M_41	51214	IGF2 antisense RNA(IGF2-AS)
M_41	143384	CDK2 associated cullin domain 1(CACUL1)
M_41	80007	chromosome 10 open reading frame 88(C10orf88)
M_41	92170	mitochondrial ribosome associated GTPase 1(MTG1)
M_41	119559	sideroflexin 4(SFXN4)
M_41	36	acyl-CoA dehydrogenase, short/branched

M_41	55844	protein phosphatase 2 regulatory subunit
M_41	57700	family with sequence similarity 160 member
M_41	7073	TIA1 cytotoxic granule associated RNA binding protein like 1(TIAL1)
M_41	3481	insulin like growth factor 2(IGF2)
M_41	8036	SHOC2, leucine rich repeat scaffold protein(SHOC2)
M_41	100192386	uncharacterized LOC100192386(FLJ16779)
M_41	55088	coiled-coil domain containing 186(CCDC186)
M_41	57678	glycerol-3-phosphate acyltransferase,
M_41	9024	BR serine/threonine kinase 2(BRSK2)
M_41	54780	NSE4 homolog A, SMC5-SMC6 complex component(NSMCE4A)
M_41	2263	fibroblast growth factor receptor 2(FGFR2)
M_41	142940	TruB pseudouridine synthase family member
M_41	4661	myelin transcription factor 1(MYT1)
M_41	143187	vesicle transport through interaction with t-SNAREs 1A(VTI1A)
M_41	10579	transforming acidic coiled-coil containing protein
M_41	10844	tubulin gamma complex associated protein
M_41	399818	methyltransferase like 10(METTL10)
M_41	56647	BRCA2 and CDKN1A interacting protein(BCCIP)
M_41	10935	peroxiredoxin 3(PRDX3)
M_41	374354	NHL repeat containing 2(NHLRC2)
M_41	404636	family with sequence similarity 45 member
M_41	4255	O-6-methylguanine-DNA methyltransferase(MGMT)
M_41	282974	serine/threonine kinase 32C(STK32C)
M_41	1892	enoyl-CoA hydratase, short chain 1(ECHS1)
M_41	118987	PDZ domain containing 8(PDZD8)
M_41	128414	Na+/K+ transporting ATPase interacting 4(NKAIN4)
M_41	63877	family with sequence similarity 204 member
M_41	11101	arginyltransferase 1(ATE1)
M_41	4987	opioid related nociceptin receptor 1(OPRL1)

M_41	120	adducin 3(ADD3)
M_41	203190	leucine rich repeat LGI family member 3(LGI3)
M_41	1793	dedicator of cytokinesis 1(DOCK1)
M_41	64376	IKAROS family zinc finger 5(IKZF5)
M_41	118672	phosphoseryl-tRNA kinase(PSTK)
M_41	80313	leucine rich repeat containing 27(LRRC27)
M_41	7511	X-prolyl aminopeptidase 1(XPNPEP1)
M_41	723961	INS-IGF2 readthrough(INS-IGF2)
M_41	822	capping actin protein, gelsolin like(CAPG)
M_41	3632	inositol polyphosphate-5-phosphatase A(INPP5A)
M_41	79892	minichromosome maintenance complex binding protein(MCMBP)
M_41	10285	survival motor neuron domain containing
M_41	8661	eukaryotic translation initiation factor 3 subunit
M_41	10287	regulator of G-protein signaling 19(RGS19)
M_41	170394	PWWP domain containing 2B(PWWP2B)
M_41	23172	family with sequence similarity 175 member
M_41	340719	nanos C2HC-type zinc finger 1(NANOS1)
M_41	59338	pleckstrin homology domain containing
M_41	26098	erythroid differentiation regulatory factor 1(EDRF1)
M_41	64429	zinc finger DHHC-type containing 6(ZDHHC6)
M_41	4583	mucin 2, oligomeric mucus/gel-forming(MUC2)
M_41	55855	family with sequence similarity 45, member A pseudogene(FAM45BP)
M_41	9937	DNA cross-link repair 1A(DCLRE1A)
M_41	7390	uroporphyrinogen III synthase(UROS)
M_41	9679	family with sequence similarity 53 member
M_41	196743	polyamine oxidase(PAOX)
M_41	9126	structural maintenance of chromosomes 3(SMC3)
M_41	11196	SEC23 interacting protein(SEC23IP)
M_41	57698	shootin 1(SHTN1)
M_41	1488	C-terminal binding protein 2(CTBP2)

M_41	57580	phosphatidylinositol-3,4,5-trisphosphate dependent Rac exchange factor 1(PREX1)
M_41	503542	shadow of prion protein homolog (zebrafish)(SPRN)
M_41	80758	proline rich 7, synaptic(PRR7)
M_41	282969	fucose mutarotase(FUOM)
M_41	10461	MER proto-oncogene, tyrosine kinase(MERTK)
M_41	79683	zinc finger DHHC-type containing 14(ZDHHC14)
M_41	91010	formin like 3(FMNL3)
M_41	4773	nuclear factor of activated T-cells 2(NFATC2)
M_41	27439	cat eye syndrome chromosome region, candidate
M_41	8857	Fc fragment of IgG binding protein(FCGBP)
M_41	1524	C-X3-C motif chemokine receptor 1(CX3CR1)
M_41	27306	hematopoietic prostaglandin D synthase(HPGDS)
M_41	122618	phospholipase D family member 4(PLD4)
M_41	57121	lysophosphatidic acid receptor 5(LPAR5)
M_41	64805	purinergic receptor P2Y12(P2RY12)
M_42	124152	IQ motif containing K(IQCK)
M_42	5531	protein phosphatase 4 catalytic subunit(PPP4C)
M_42	84901	nuclear factor of activated T-cells 2 interacting protein(NFATC2IP)
M_42	23274	C-type lectin domain family 16 member A(CLEC16A)
M_42	54820	nudE neurodevelopment protein 1(NDE1)
M_42	5930	RB binding protein 6, ubiquitin ligase(RBBP6)
M_42	55718	RNA polymerase III subunit E(POLR3E)
M_42	7874	ubiquitin specific peptidase 7(USP7)
M_42	197407	zinc finger protein 48(ZNF48)
M_42	83719	yippee like 3(YPEL3)
M_42	11273	ataxin 2 like(ATXN2L)
M_42	730094	chromosome 16 open reading frame 52(C16orf52)
M_42	84516	dynactin subunit 5(DCTN5)
M_42	6810	syntaxin 4(STX4)

M_42	80270	hydroxy-delta-5-steroid dehydrogenase, 3 beta- and steroid delta-isomerase 7(HSD3B7)
M_42	92595	zinc finger protein 764(ZNF764)
M_42	2935	G1 to S phase transition 1(GSPT1)
M_42	123811	FGFR1OP N-terminal like(FOPNL)
M_42	55114	Rho GTPase activating protein 17(ARHGAP17)
M_42	29035	chromosome 16 open reading frame 72(C16orf72)
M_42	51108	methyltransferase like 9(METTL9)
M_42	28955	Dexi homolog(DEXI)
M_42	54700	RRN3 homolog, RNA polymerase I transcription factor(RRN3)
M_42	79874	rabaptin, RAB GTPase binding effector protein
M_42	65988	zinc finger protein 747(ZNF747)
M_42	56061	ubiquitin family domain containing 1(UBFD1)
M_42	9665	KIAA0430(KIAA0430)
M_42	51283	bifunctional apoptosis regulator(BFAR)
M_42	10308	zinc finger protein 267(ZNF267)
M_42	8303	stannin(SNN)
M_42	10423	CDP-diacylglycerol--inositol 3-phosphatidyltransferase(CDIPT)
M_42	10421	CD2 cytoplasmic tail binding protein 2(CD2BP2)
M_42	226	aldolase, fructose-bisphosphate A(ALDOA)
M_42	23042	pyridoxal dependent decarboxylase domain containing 1(PDXDC1)
M_42	51333	zinc finger protein 771(ZNF771)
M_42	124411	zinc finger protein 720(ZNF720)
M_42	8479	HIRA interacting protein 3(HIRIP3)
M_42	6818	sulfotransferase family 1A member 3(SULT1A3)
M_42	92017	sorting nexin 29(SNX29)
M_42	10847	Snf2 related CREBBP activator protein(SRCAP)
M_42	57478	ubiquitin specific peptidase 31(USP31)

M_42	100131998	RRN3 homolog, RNA polymerase I transcription factor pseudogene 3(RRN3P3)
M_42	100132341	clustered mitochondria homolog pseudogene
M_42	1201	CLN3, battenin(CLN3)
M_42	5073	poly(A)-specific ribonuclease(PARN)
M_42	79759	zinc finger protein 668(ZNF668)
M_42	25970	SH2B adaptor protein 1(SH2B1)
M_42	2975	general transcription factor IIIC subunit 1(GTF3C1)
M_42	57020	chromosome 16 open reading frame 62(C16orf62)
M_42	4363	ATP binding cassette subfamily C member 1(ABCC1)
M_42	2072	ERCC excision repair 4, endonuclease catalytic subunit(ERCC4)
M_42	5595	mitogen-activated protein kinase 3(MAPK3)
M_42	51573	glycerophosphodiester phosphodiesterase 1(GDE1)
M_42	23214	exportin 6(XPO6)
M_42	23062	golgi associated, gamma adaptin ear containing, ARF binding protein 2(GGA2)
M_42	283899	INO80 complex subunit E(INO80E)
M_42	10295	branched chain ketoacid dehydrogenase
M_42	112479	ERI1 exoribonuclease family member 2(ERI2)
M_42	23361	zinc finger protein 629(ZNF629)
M_42	79724	zinc finger protein 768(ZNF768)
M_42	79447	PAXIP1 associated glutamate rich protein 1(PAGR1)
M_42	27327	trinucleotide repeat containing 6A(TNRC6A)
M_42	84148	lysine acetyltransferase 8(KAT8)
M_42	83985	sphingolipid transporter 1 (putative)(SPNS1)
M_42	64319	fibrosin(FBRS)
M_42	93129	ORAI calcium release-activated calcium modulator
M_42	9738	centriolar coiled-coil protein 110(CCP110)
M_42	9739	SET domain containing 1A(SETD1A)
M_42	84127	#N/A
M_42	91949	component of oligomeric golgi complex 7(COG7)

M_42	55623	THUMP domain containing 1(THUMPD1)
M_42	79728	partner and localizer of BRCA2(PALB2)
M_42	78994	proline rich 14(PRR14)
M_42	440354	SMG1P2, nonsense mediated mRNA decay associated PI3K related kinase pseudogene 2(SMG1P2)
M_42	115509	zinc finger protein 689(ZNF689)
M_42	3835	kinesin family member 22(KIF22)
M_42	54620	F-box and leucine rich repeat protein 19(FBXL19)
M_42	400506	lysine rich nucleolar protein 1(KNOP1)
M_42	400508	CRYM antisense RNA 1(CRYM-AS1)
M_42	7284	Tu translation elongation factor,
M_42	79008	SLX1 homolog B, structure-specific endonuclease subunit(SLX1B)
M_42	10229	coenzyme Q7, hydroxylase(COQ7)
M_42	253980	potassium channel tetramerization domain containing 13(KCTD13)
M_42	552900	bolA family member 2(BOLA2)
M_42	146540	zinc finger protein 785(ZNF785)
M_42	64755	chromosome 16 open reading frame 58(C16orf58)
M_42	23247	KIAA0556(KIAA0556)
M_42	79798	armadillo repeat containing 5(ARMC5)
M_42	29066	zinc finger CCCH-type containing 7A(ZC3H7A)
M_42	124454	glutamyl-tRNA synthetase 2, mitochondrial(EARS2)
M_42	51061	thioredoxin domain containing 11(TXNDC11)
M_42	4150	MYC associated zinc finger protein(MAZ)
M_42	9726	zinc finger protein 646(ZNF646)
M_42	57496	MKL1/myocardin like 2(MKL2)
M_42	112869	SAGA complex associated factor 29(SGF29)
M_42	22928	selenophosphate synthetase 2(SEPHS2)
M_42	29904	eukaryotic elongation factor 2 kinase(EEF2K)
M_42	9810	ring finger protein 40(RNF40)
M_42	9344	TAO kinase 2(TAOK2)

M_42	5261	phosphorylase kinase catalytic subunit gamma
M_42	26000	TBC1 domain family member 10B(TBC1D10B)
M_42	79831	lysine demethylase 8(KDM8)
M_42	9274	BCL tumor suppressor 7C(BCL7C)
M_42	9961	major vault protein(MVP)
M_42	7385	ubiquinol-cytochrome c reductase core protein
M_42	23049	SMG1, nonsense mediated mRNA decay associated PI3K related kinase(SMG1)
M_42	23594	origin recognition complex subunit 6(ORC6)
M_42	283820	NODAL modulator 2(NOMO2)
M_42	408050	NODAL modulator 3(NOMO3)
M_42	4706	NADH:ubiquinone oxidoreductase subunit
M_42	79077	dCTP pyrophosphatase 1(DCTPP1)
M_42	388242	SAGA complex associated factor 29 pseudogene(LOC388242)
M_42	51451	leucine carboxyl methyltransferase 1(LCMT1)
M_42	79801	SHC binding and spindle associated 1(SHCBP1)
M_42	79001	vitamin K epoxide reductase complex subunit
M_42	1489	cardiotrophin 1(CTF1)
M_42	728689	eukaryotic translation initiation factor 3 subunit C-like(EIF3CL)
M_42	2521	FUS RNA binding protein(FUS)
M_42	197370	NSE1 homolog, SMC5-SMC6 complex
M_42	146542	zinc finger protein 688(ZNF688)
M_42	146547	protease, serine 36(PRSS36)
M_42	124446	transmembrane protein 219(TMEM219)
M_42	342357	zinc finger with KRAB and SCAN domains
M_42	8663	eukaryotic translation initiation factor 3 subunit
M_42	23420	NODAL modulator 1(NOMO1)
M_43	124936	cytochrome b5 domain containing 2(CYB5D2)
M_43	11315	Parkinsonism associated deglycase(PARK7)
M_43	79947	dehydrodolichyl diphosphate synthase

M_43	11313	lysophospholipase II(LYPLA2)
M_43	56063	transmembrane protein 234(TMEM234)
M_43	23207	pleckstrin homology and RUN domain containing M2(PLEKHM2)
M_43	113444	small integral membrane protein 12(SMIM12)
M_43	5226	phosphogluconate dehydrogenase(PGD)
M_43	9927	mitofusin 2(MFN2)
M_43	3065	histone deacetylase 1(HDAC1)
M_43	8668	eukaryotic translation initiation factor 3 subunit
M_43	22934	ribose 5-phosphate isomerase A(RPIA)
M_43	5928	RB binding protein 4, chromatin remodeling
M_43	55721	IQ motif containing C(IQCC)
M_43	127262	tumor protein p63 regulated 1 like(TPRG1L)
M_43	56181	mitochondrial fission regulator 1 like(MTFR1L)
M_43	6248	regulator of solute carriers 1(RSC1A1)
M_43	23038	WD and tetratricopeptide repeats 1(WDTC1)
M_43	23028	lysine demethylase 1A(KDM1A)
M_43	64960	mitochondrial ribosomal protein S15(MRPS15)
M_43	118442	G protein-coupled receptor 62(GPR62)
M_43	55116	transmembrane protein 39B(TMEM39B)
M_43	3151	high mobility group nucleosomal binding domain
M_43	50809	heterochromatin protein 1 binding protein 3(HP1BP3)
M_43	55113	XK related 8(XKR8)
M_43	51102	mitochondrial trans-2-enoyl-CoA reductase(MECR)
M_43	2517	fucosidase, alpha-L- 1, tissue(FUCA1)
M_43	3155	3-hydroxymethyl-3-methylglutaryl-CoA
M_43	490	ATPase plasma membrane Ca2+ transporting
M_43	388611	CD164 molecule like 2(CD164L2)
M_43	1855	dishevelled segment polarity protein 1(DVL1)
M_43	998	cell division cycle 42(CDC42)
M_43	5590	protein kinase C zeta(PRKCZ)
M_43	8073	protein tyrosine phosphatase type IVA, member

M_43	54707	GPN-loop GTPase 2(GPN2)
M_43	10163	WAS protein family member 2(WASF2)
M_43	84328	leucine zipper and CTNNBIP1 domain
M_43	2140	EYA transcriptional coactivator and phosphatase
M_43	338758	long intergenic non-protein coding RNA
M_43	51538	zinc finger CCHC-type containing 17(ZCCHC17)
M_43	63906	G-patch domain containing 3(GPATCH3)
M_43	57470	leucine rich repeat containing 47(LRRC47)
M_43	65018	PTEN induced putative kinase 1(PINK1)
M_43	6883	TATA-box binding protein associated factor
M_43	401375	GTF2I repeat domain containing 2 pseudogene 1(GTF2IRD2P1)
M_43	6118	replication protein A2(RPA2)
M_43	84163	GTF2I repeat domain containing 2(GTF2IRD2)
M_43	4524	methylenetetrahydrofolate reductase(MTHFR)
M_43	84069	pleckstrin homology domain containing
M_43	55052	mitochondrial ribosomal protein L20(MRPL20)
M_43	5690	proteasome subunit beta 2(PSMB2)
M_43	257044	chromosome 1 open reading frame 101(C1orf101)
M_43	2475	mechanistic target of rapamycin(MTOR)
M_43	55219	transmembrane protein 57(TMEM57)
M_43	84065	transmembrane protein 222(TMEM222)
M_43	79777	acyl-CoA binding domain containing 4(ACBD4)
M_43	90529	sperm tail PG-rich repeat containing 1(STPG1)
M_43	832	capping actin protein of muscle Z-line beta
M_43	3669	interferon stimulated exonuclease gene 20(ISG20)
M_43	23585	transmembrane protein 50A(TMEM50A)
M_43	553115	penta-EF-hand domain containing 1(PEF1)
M_43	55920	regulator of chromosome condensation 2(RCC2)
M_43	65979	phosphatase and actin regulator 4(PHACTR4)
M_43	100129354	neurotrophin receptor associated death domain, pseudogene(NRADDP)

M_43	54455	F-box protein 42(FBXO42)
M_43	84808	PPARGC1 and ESRR induced regulator, muscle
M_43	1104	regulator of chromosome condensation 1(RCC1)
M_43	7709	zinc finger and BTB domain containing 17(ZBTB17)
M_43	6429	serine and arginine rich splicing factor 4(SRSF4)
M_43	729614	uncharacterized LOC729614(FLJ37453)
M_43	204	adenylate kinase 2(AK2)
M_43	10726	nuclear distribution C, dynein complex
M_43	9863	membrane associated guanylate kinase, WW and PDZ domain containing 2(MAGI2)
M_43	64793	centrosomal protein 85(CEP85)
M_43	26119	low density lipoprotein receptor adaptor protein
M_43	23065	ER membrane protein complex subunit 1(EMC1)
M_43	51154	MRT4 homolog, ribosome maturation factor(MRTO4)
M_43	51150	stromal cell derived factor 4(SDF4)
M_43	118424	ubiquitin conjugating enzyme E2 J2(UBE2J2)
M_43	23261	calmodulin binding transcription activator
M_43	79363	REM2 and RAB like small GTPase 1(RSG1)
M_43	6195	ribosomal protein S6 kinase A1(RPS6KA1)
M_43	8565	tyrosyl-tRNA synthetase(YARS)
M_43	22826	DnaJ heat shock protein family (Hsp40) member C8(DNAJC8)
M_43	79594	mitochondrial E3 ubiquitin protein ligase 1(MUL1)
M_43	25949	SYF2 pre-mRNA splicing factor(SYF2)
M_43	8574	aldo-keto reductase family 7 member A2(AKR7A2)
M_43	23463	isoprenylcysteine carboxyl methyltransferase(ICMT)
M_43	5195	peroxisomal biogenesis factor 14(PEX14)
M_43	5192	peroxisomal biogenesis factor 10(PEX10)
M_43	8659	aldehyde dehydrogenase 4 family member
M_43	57085	angiotensin II receptor associated protein(AGTRAP)
M_43	2582	UDP-galactose-4-epimerase(GALE)
M_43	55629	proline rich nuclear receptor coactivator 2(PNRC2)

M_43	2023	enolase 1(ENO1)
M_43	11332	acyl-CoA thioesterase 7(ACOT7)
M_43	57185	NIPA like domain containing 3(NIPAL3)
M_43	64802	nicotinamide nucleotide adenylyltransferase
M_43	339487	zinc finger and BTB domain containing 8 opposite strand(ZBTB8OS)
M_43	56888	potassium channel modulatory factor 1(KCMF1)
M_43	84967	LSM10, U7 small nuclear RNA associated(LSM10)
M_43	90231	KIAA2013(KIAA2013)
M_43	339448	chromosome 1 open reading frame 174(C1orf174)
M_43	5394	exosome component 10(EXOSC10)
M_43	54952	tRNA selenocysteine 1 associated protein
M_43	49856	WD repeat containing, antisense to TP73(WRAP73)
M_43	23673	syntaxin 12(STX12)
M_43	54973	cleavage and polyadenylation specific factor 3-
M_43	255104	transmembrane and coiled-coil domains 4(TMCO4)
M_43	51042	zinc finger protein 593(ZNF593)
M_43	10691	glucocorticoid modulatory element binding protein
M_43	29101	SSU72 homolog, RNA polymerase II CTD phosphatase(SSU72)
M_43	2035	erythrocyte membrane protein band 4.1(EPB41)
M_43	9410	small nuclear ribonucleoprotein U5 subunit
M_43	84514	GH3 domain containing(GHDC)
M_43	55650	phosphatidylinositol glycan anchor biosynthesis class
M_43	10459	MAD2 mitotic arrest deficient-like 2
M_43	60672	migration and invasion inhibitory protein(MIIP)
M_43	148413	uncharacterized LOC148413(LOC148413)
M_43	55063	zinc finger CW-type and PWWP domain containing 1(ZCWPW1)
M_43	26155	NOC2 like nucleolar associated transcriptional repressor(NOC2L)
M_43	54797	mediator complex subunit 18(MED18)

M_43	29092	long intergenic non-protein coding RNA
M_43	84958	synaptotagmin like 1(SYTL1)
M_43	55092	transmembrane protein 51(TMEM51)
M_43	199870	family with sequence similarity 76 member
M_43	84243	zinc finger DHHC-type containing 18(ZDHHC18)
M_43	11079	retention in endoplasmic reticulum sorting receptor
M_43	2782	G protein subunit beta 1(GNB1)
M_43	54991	chromosome 1 open reading frame 159(C1orf159)
M_43	83442	SH3 domain binding glutamate rich protein like
M_43	23341	DnaJ heat shock protein family (Hsp40) member C16(DNAJC16)
M_43	100130581	long intergenic non-protein coding RNA
M_43	55735	DnaJ heat shock protein family (Hsp40) member C11(DNAJC11)
M_43	79180	EF-hand domain family member D2(EFHD2)
M_43	84275	solute carrier family 25 member 33(SLC25A33)
M_43	80772	ceramide-1-phosphate transfer protein(CPTP)
M_43	10772	serine and arginine rich splicing factor 10(SRSF10)
M_43	64856	von Willebrand factor A domain containing 1(VWA1)
M_43	653639	lysophospholipase II pseudogene 1(LYPLA2P1)
M_43	199953	transmembrane protein 201(TMEM201)
M_43	199990	Fanconi anemia core complex associated protein
M_43	842	caspase 9(CASP9)
M_43	27095	trafficking protein particle complex 3(TRAPPC3)
M_43	90326	THAP domain containing 3(THAP3)
M_43	91544	UBX domain protein 11(UBXN11)
M_43	27245	AT-hook DNA binding motif containing 1(AHDC1)
M_43	57095	PITH domain containing 1(PITHD1)
M_43	126789	pseudouridylate synthase-like 1(PUSL1)
M_43	6924	transcription elongation factor B subunit 3(TCEB3)
M_43	10256	connector enhancer of kinase suppressor of Ras
M_43	6723	spermidine synthase(SRM)

M_43	56998	catenin beta interacting protein 1(CTNNBIP1)
M_43	54998	aurora kinase A interacting protein 1(AURKAIP1)
M_43	124790	hexamethylene bisacetamide inducible 2(HEXIM2)
M_43	1676	DNA fragmentation factor subunit alpha(DFFA)
M_43	64766	S100P binding protein(S100PBP)
M_43	1677	DNA fragmentation factor subunit beta(DFFB)
M_43	55229	pantothenate kinase 4(PANK4)
M_43	6390	succinate dehydrogenase complex iron sulfur subunit B(SDHB)
M_43	29914	UbiA prenyltransferase domain containing
M_43	54896	PQ loop repeat containing 2(PQLC2)
M_43	126792	beta-1,3-galactosyltransferase 6(B3GALT6)
M_43	378708	centromere protein S(CENPS)
M_43	1650	dolichyl-diphosphooligosaccharide--protein glycosyltransferase non-catalytic subunit(DDOST)
M_43	440574	mitochondrial inner membrane organizing system
M_43	9064	mitogen-activated protein kinase kinase kinase
M_43	149420	PDLIM1 interacting kinase 1 like(PDIK1L)
M_43	388969	chromosome 2 open reading frame 68(C2orf68)
M_43	26099	SUZ RNA binding domain containing 1(SZRD1)
M_43	55707	NECAP endocytosis associated 2(NECAP2)
M_43	93974	ATPase inhibitory factor 1(ATPIF1)
M_43	23400	ATPase 13A2(ATP13A2)
M_43	26270	F-box protein 6(FBXO6)
M_43	5511	protein phosphatase 1 regulatory subunit 8(PPP1R8)
M_43	5051	platelet activating factor acetylhydrolase 2(PAFAH2)
M_43	6548	solute carrier family 9 member A1(SLC9A1)
M_43	84196	ubiquitin specific peptidase 48(USP48)
M_43	3104	zinc finger and BTB domain containing 48(ZBTB48)
M_43	93653	ST7 antisense RNA 1(ST7-AS1)
M_43	84301	DNA damage inducible 1 homolog 2(DDI2)
M_43	51441	YTH N6-methyladenosine RNA binding protein

M_43	65220	NAD kinase(NADK)
M_43	10713	ubiquitin specific peptidase 39(USP39)
M_43	55577	N-acetylglucosamine kinase(NAGK)
M_43	9341	vesicle associated membrane protein 3(VAMP3)
M_43	164091	progestin and adipoQ receptor family member
M_43	127700	organic solute carrier partner 1(OSCP1)
M_43	643837	long intergenic non-protein coding RNA
M_43	127687	chromosome 1 open reading frame 122(C1orf122)
M_43	352954	GATS, stromal antigen 3 opposite strand(GATS)
M_43	388759	chromosome 1 open reading frame 229(C1orf229)
M_43	160335	transmembrane and tetratricopeptide repeat containing 2(TMTC2)
M_43	284677	chromosome 1 open reading frame 204(C1orf204)
M_43	283129	membrane anchored junction protein(MAJIN)
M_43	57190	selenoprotein N(SELENON)
M_43	83667	sestrin 2(SESN2)
M_43	149069	doublecortin domain containing 2B(DCDC2B)
M_43	54936	ADP-ribosylhydrolase like 2(ADPRHL2)
M_43	254173	tubulin tyrosine ligase like 10(TTLL10)
M_43	5351	procollagen-lysine,2-oxoglutarate 5-dioxygenase
M_43	79000	aurora kinase A and ninein interacting
M_43	115399	leucine rich repeat containing 56(LRRC56)
M_43	1870	E2F transcription factor 2(E2F2)
M_43	9903	kelch like family member 21(KLHL21)
M_43	2192	fibulin 1(FBLN1)
M_43	55937	apolipoprotein M(APOM)
M_43	391123	V-set and immunoglobulin domain containing
M_43	283050	ZMIZ1 antisense RNA 1(ZMIZ1-AS1)
M_43	26228	signal transducing adaptor family member 1(STAP1)
M_43	170589	glycoprotein hormone alpha 2(GPHA2)
M_43	202020	TAPT1 antisense RNA 1 (head to head)(TAPT1-AS1)
M_43	3305	heat shock protein family A (Hsp70) member 1

M_43	374946	dorsal inhibitory axon guidance protein(DRAXIN)
M_43	3925	stathmin 1(STMN1)
M_43	98	acylphosphatase 2(ACYP2)
M_43	388722	chromosome 1 open reading frame 53(C1orf53)
M_43	100302401	RASAL2 antisense RNA 1(RASAL2-AS1)
M_43	3693	integrin subunit beta 5(ITGB5)
M_44	146845	cilia and flagella associated protein 52(CFAP52)
M_44	100287718	ankyrin repeat domain 66(ANKRD66)
M_44	389118	cadherin related family member 4(CDHR4)
M_44	403314	apolipoprotein B mRNA editing enzyme catalytic polypeptide like 4(APOBEC4)
M_44	124602	kinesin family member 19(KIF19)
M_44	728194	radial spoke head 10 homolog B2(RSPH10B2)
M_44	389177	transmembrane protein 212(TMEM212)
M_44	201625	dynein axonemal heavy chain 12(DNAH12)
M_44	284485	regulatory subunit of type II PKA R-subunit (RIIa) domain containing 1(RIIAD1)
M_44	161003	stomatin like 3(STOML3)
M_44	128602	chromosome 20 open reading frame 85(C20orf85)
M_44	283518	potassium channel regulator(KCNRG)
M_44	220004	protein phosphatase 1 regulatory subunit
M_44	202500	t-complex-associated-testis-expressed 1(TCTE1)
M_44	165186	family with sequence similarity 179 member
M_44	100130771	EF-hand calcium binding domain 10(EFCAB10)
M_44	399949	chromosome 11 open reading frame 88(C11orf88)
M_44	144809	family with sequence similarity 216 member
M_44	80122	mitogen-activated protein kinase kinase kinase
M_44	221424	leucine rich repeat containing 73(LRRC73)
M_44	133923	zinc finger protein 474(ZNF474)
M_44	166979	cell division cycle 20B(CDC20B)
M_44	283726	stabilizer of axonemal microtubules 2(SAXO2)
M_44	222256	cadherin related family member 3(CDHR3)

M_44	51673	tubulin polymerization promoting protein family member 3(TPPP3)
M_44	149499	leucine rich repeat containing 71(LRRC71)
M_44	84688	chromosome 9 open reading frame 24(C9orf24)
M_44	132203	sentan, cilia apical structure protein(SNTN)
M_44	339145	family with sequence similarity 92 member
M_44	221409	spermatogenesis associated serine rich 1(SPATS1)
M_44	10309	cyclin O(CCNO)
M_44	80086	tubulin alpha 4b(TUBA4B)
M_44	150147	UMODL1 antisense RNA 1(UMODL1-AS1)
M_44	161502	cilia and flagella associated protein 161(CFAP161)
M_44	160777	coiled-coil domain containing 60(CCDC60)
M_44	127795	chromosome 1 open reading frame 87(C1orf87)
M_44	60494	coiled-coil domain containing 81(CCDC81)
M_44	79747	androglobin(ADGB)
M_44	2940	glutathione S-transferase alpha 3(GSTA3)
M_44	64446	dynein axonemal intermediate chain 2(DNAI2)
M_44	388701	chromosome 1 open reading frame 189(C1orf189)
M_44	121643	forkhead box N4(FOXN4)
M_44	80133	maestro heat like repeat family member 9(MROH9)
M_44	150483	tektin 4(TEKT4)
M_44	257062	cation channel sperm associated auxiliary subunit delta(CATSPERD)
M_44	148930	kinocilin(KNCN)
M_44	474354	leucine rich repeat containing 18(LRRC18)
M_44	255119	chromosome 4 open reading frame 22(C4orf22)
M_44	3568	interleukin 5 receptor subunit alpha(IL5RA)
M_44	151790	WD repeat domain 49(WDR49)
M_44	221421	radial spoke head 9 homolog(RSPH9)
M_44	5276	serpin family I member 2(SERPINI2)
M_44	123264	solute carrier family 51 beta subunit(SLC51B)
M_44	401124	death domain containing 1(DTHD1)

M_44	138255	chromosome 9 open reading frame 135(C9orf135)
M_44	132671	spermatogenesis associated 18(SPATA18)
M_44	127254	glutamate rich 3(ERICH3)
M_44	389161	ankyrin repeat and ubiquitin domain containing
M_44	158798	A-kinase anchoring protein 14(AKAP14)
M_44	375298	ceramide kinase like(CERKL)
M_44	348378	family with sequence similarity 159 member
M_45	170302	aristaless related homeobox(ARX)
M_45	401498	transmembrane protein 215(TMEM215)
M_45	256536	transcription elongation regulator 1 like(TCERG1L)
M_45	8671	solute carrier family 4 member 4(SLC4A4)
M_45	846	calcium sensing receptor(CASR)
M_45	7103	tetraspanin 8(TSPAN8)
M_45	6770	steroidogenic acute regulatory protein(STAR)
M_45	133121	ectonucleotide pyrophosphatase/phosphodiesterase
M_45	6943	transcription factor 21(TCF21)
M_45	347731	leucine rich repeat transmembrane neuronal
M_45	27324	TOX high mobility group box family member
M_45	5178	paternally expressed 3(PEG3)
M_45	80731	thrombospondin type 1 domain containing
M_45	8618	calcium dependent secretion activator(CADPS)
M_45	9971	nuclear receptor subfamily 1 group H member
M_45	6705	small proline rich protein 2F(SPRR2F)
M_45	2100	estrogen receptor 2(ESR2)
M_45	269	anti-Mullerian hormone receptor type 2(AMHR2)
M_45	2626	GATA binding protein 4(GATA4)
M_45	8745	ADAM metallopeptidase domain 23(ADAM23)
M_45	84867	protein tyrosine phosphatase, non-receptor type
M_45	1908	endothelin 3(EDN3)
M_45	55821	allantoicase(ALLC)
M_45	26059	ELKS/RAB6-interacting/CAST family member
M_45	387890	transmembrane protein 233(TMEM233)

M_45	26033	attractin like 1(ATRNL1)
M_45	7474	Wnt family member 5A(WNT5A)
M_45	5046	proprotein convertase subtilisin/kexin type 6(PCSK6)
M_45	2516	nuclear receptor subfamily 5 group A member
M_45	79825	EF-hand and coiled-coil domain containing 1(EFCC1)
M_45	347442	DDB1 and CUL4 associated factor 8 like
M_45	84432	prokineticin 1(PROK1)
M_45	6000	regulator of G-protein signaling 7(RGS7)
M_45	84709	mitochondria localized glutamic acid rich
M_45	8788	delta like non-canonical Notch ligand 1(DLK1)
M_45	78989	collectin subfamily member 11(COLEC11)
M_45	401089	FOXL2 neighbor(FOXL2NB)
M_45	129293	TraB domain containing 2A(TRABD2A)
M_45	93649	myocardin(MYOCD)
M_45	668	forkhead box L2(FOXL2)
M_45	345557	phosphatidylinositol specific phospholipase C X domain containing 3(PLCXD3)
M_45	730	complement C7(C7)
M_45	711	ERC2 intronic transcript 1(ERC2-IT1)
M_45	23742	nuclear pore associated protein 1(NPAP1)
M_45	3131	HLF, PAR bZIP transcription factor(HLF)
M_45	791114	Prader-Willi region non-protein coding RNA
M_45	3354	5-hydroxytryptamine receptor 1E(HTR1E)
M_45	9630	G protein subunit alpha 14(GNA14)
M_45	6997	teratocarcinoma-derived growth factor 1(TDGF1)
M_45	6998	teratocarcinoma-derived growth factor 1 pseudogene 3(TDGF1P3)
M_45	10202	dehydrogenase/reductase 2(DHRS2)
M_45	2742	glycine receptor alpha 2(GLRA2)
M_45	114132	sialic acid binding Ig like lectin 11(SIGLEC11)
M_45	389332	uncharacterized LOC389332(LOC389332)
M_45	2628	glycine amidinotransferase(GATM)

M_45	400709	sialic acid binding Ig like lectin 16 (gene/pseudogene)(SIGLEC16)
M_45	57683	zinc finger DBF-type containing 2(ZDBF2)
M_45	23281	microtubule associated tumor suppressor candidate
M_45	5090	PBX homeobox 3(PBX3)
M_45	5199	complement factor properdin(CFP)
M_45	389125	musculoskeletal, embryonic nuclear protein
M_45	2646	glucokinase regulator(GCKR)
M_45	30819	potassium voltage-gated channel interacting protein 2(KCNIP2)
M_45	8620	neuropeptide FF-amide peptide precursor(NPFF)
M_46	192134	UDP-GlcNAc:betaGal beta-1,3-N-acetylglucosaminyltransferase 6(B3GNT6)
M_46	474	atonal bHLH transcription factor 1(ATOH1)
M_46	390874	one cut homeobox 3(ONECUT3)
M_46	388364	transmembrane and immunoglobulin domain containing 1(TMIGD1)
M_46	644844	proline, histidine and glycine rich 1(PHGR1)
M_46	54860	membrane spanning 4-domains A12(MS4A12)
M_46	135644	tripartite motif containing 40(TRIM40)
M_46	81850	keratin associated protein 1-3(KRTAP1-3)
M_46	139604	MAGE family member B16(MAGEB16)
M_46	140453	mucin 17, cell surface associated(MUC17)
M_46	257629	ankyrin repeat and sterile alpha motif domain containing 4B(ANKS4B)
M_46	85293	keratin associated protein 3-3(KRTAP3-3)
M_46	54474	keratin 20(KRT20)
M_46	162605	keratin 28(KRT28)
M_46	83901	keratin associated protein 9-8(KRTAP9-8)
M_46	26239	late cornified envelope 2B(LCE2B)
M_46	1671	defensin alpha 6(DEFA6)
M_46	5068	regenerating family member 3 alpha(REG3A)

M_46	5968	regenerating family member 1 beta(REG1B)
M_46	83899	keratin associated protein 9-2(KRTAP9-2)
M_46	1559	cytochrome P450 family 2 subfamily C member
M_46	29974	APOBEC1 complementation factor(A1CF)
M_46	1015	cadherin 17(CDH17)
M_46	389434	iodotyrosine deiodinase(IYD)
M_46	646625	ureidoimidazoline (2-oxo-4-hydroxy-4-carboxy-5-) decarboxylase(URAD)
M_46	389761	SPATA31 subfamily D member 4(SPATA31D4)
M_46	613211	defensin beta 134(DEFB134)
M_46	613210	defensin beta 136(DEFB136)
M_46	448835	late cornified envelope 6A(LCE6A)
M_46	54064	long intergenic non-protein coding RNA
M_46	26062	hyaluronoglucosaminidase pseudogene 1(HYALP1)
M_46	130120	regenerating family member 3 gamma(REG3G)
M_46	79799	UDP glucuronosyltransferase family 2 member
M_46	353139	late cornified envelope 2A(LCE2A)
M_46	353131	late cornified envelope 1A(LCE1A)
M_46	27283	tubulointerstitial nephritis antigen(TINAG)
M_46	353133	late cornified envelope 1C(LCE1C)
M_46	84560	metallothionein 4(MT4)
M_46	26529	olfactory receptor family 12 subfamily D member 2 (gene/pseudogene)(OR12D2)
M_46	229	aldolase, fructose-bisphosphate B(ALDOB)
M_46	54827	neurexophilin and PC-esterase domain family member 4(NXPE4)
M_46	6476	sucrase-isomaltase(SI)
M_46	100169752	TEX36 antisense RNA 1(TEX36-AS1)
M_46	729767	carcinoembryonic antigen related cell adhesion molecule 18(CEACAM18)
M_46	1670	defensin alpha 5(DEFA5)
M_46	3441	interferon alpha 4(IFNA4)

M_46	7152	topoisomerase (DNA) I pseudogene 2(TOP1P2)
M_46	120400	neurexophilin and PC-esterase domain family member 1(NXPE1)
M_46	337959	keratin associated protein 13-2(KRTAP13-2)
M_46	387755	inscuteable homolog (Drosophila)(INSC)
M_46	1179	chloride channel accessory 1(CLCA1)
M_46	729121	RGPD4 antisense RNA 1 (head to head)(RGPD4-
M_46	2980	guanylate cyclase activator 2A(GUCA2A)
M_46	57084	solute carrier family 17 member 6(SLC17A6)
M_46	347127	SPATA31 subfamily D member 5, pseudogene(SPATA31D5P)
M_46	2168	fatty acid binding protein 1(FABP1)
M_46	2981	guanylate cyclase activator 2B(GUCA2B)
M_46	151325	myeloid associated differentiation marker like (pseudogene)(MYADML)
M_46	1557	cytochrome P450 family 2 subfamily C member 19(CYP2C19)
M_46	762	carbonic anhydrase 4(CA4)
M_46	5269	serpin family B member 6(SERPINB6)
M_46	2641	glucagon(GCG)
M_46	26240	family with sequence similarity 50 member
M_46	124274	G protein-coupled receptor 139(GPR139)
M_46	1577	cytochrome P450 family 3 subfamily A member
M_46	4640	myosin IA(MYO1A)
M_46	339479	BMP/retinoic acid inducible neural specific
M_46	654463	fer-1 like family member 6(FER1L6)
M_46	4835	NAD(P)H quinone dehydrogenase 2(NQO2)
M_46	146456	transmembrane p24 trafficking protein 6(TMED6)
M_46	3605	interleukin 17A(IL17A)
M_46	404203	serine peptidase inhibitor, Kazal type 6(SPINK6)
M_46	646627	LY6/PLAUR domain containing 8(LYPD8)
M_46	145858	chromosome 15 open reading frame 32(C15orf32)

M_46	400866	long intergenic non-protein coding RNA
M_46	27290	serine peptidase inhibitor, Kazal type 4(SPINK4)
M_46	221074	solute carrier family 39 member 12(SLC39A12)
M_46	55211	developmental pluripotency associated 4(DPPA4)
M_46	253012	HEPACAM family member 2(HEPACAM2)
M_46	7365	UDP glucuronosyltransferase family 2 member B10(UGT2B10)
M_46	1551	cytochrome P450 family 3 subfamily A member
M_46	27121	dickkopf WNT signaling pathway inhibitor 4(DKK4)
M_46	5149	phosphodiesterase 6H(PDE6H)
M_46	390010	NK1 homeobox 2(NKX1-2)
M_46	1562	cytochrome P450 family 2 subfamily C member 18(CYP2C18)
M_46	254958	REX1, RNA exonuclease 1 homolog-like 1, pseudogene(REXO1L1P)
M_46	7404	ubiquitously transcribed tetratricopeptide repeat containing, Y-linked(UTY)
M_46	151871	developmental pluripotency associated 2(DPPA2)
M_47	221091	LRRN4 C-terminal like(LRRN4CL)
M_47	3912	laminin subunit beta 1(LAMB1)
M_47	6925	transcription factor 4(TCF4)
M_47	54551	MAGE family member L2(MAGEL2)
M_47	344558	SH3 domain containing ring finger 3(SH3RF3)
M_47	285382	chromosome 3 open reading frame 70(C3orf70)
M_47	9638	fasciculation and elongation protein zeta 1(FEZ1)
M_47	285590	SH3 and PX domains 2B(SH3PXD2B)
M_47	94031	HtrA serine peptidase 3(HTRA3)
M_47	728264	cardiac mesoderm enhancer-associated non-coding RNA(CARMN)
M_47	7837	peroxidasin(PXDN)
M_47	441094	NR2F1 antisense RNA 1(NR2F1-AS1)
M_47	11167	follistatin like 1(FSTL1)

M_47	8642	dachsous cadherin-related 1(DCHS1)
M_47	7025	nuclear receptor subfamily 2 group F member
M_47	9865	TLR4 interactor with leucine rich repeats(TRIL)
M_47	8646	chordin(CHRD)
M_47	79633	FAT atypical cadherin 4(FAT4)
M_47	155051	crystallin gamma N(CRYGN)
M_47	57722	immunoglobulin superfamily DCC subclass member 4(IGDCC4)
M_47	57537	sortilin related VPS10 domain containing receptor
M_47	83700	junctional adhesion molecule 3(JAM3)
M_47	25817	family with sequence similarity 19 member A5, C-C motif chemokine like(FAM19A5)
M_47	1124	chimerin 2(CHN2)
M_47	83872	hemicentin 1(HMCN1)
M_47	10052	gap junction protein gamma 1(GJC1)
M_47	85407	naked cuticle homolog 1(NKD1)
M_47	441631	tetraspanin 11(TSPAN11)
M_47	55083	kinesin family member 26B(KIF26B)
M_47	5654	HtrA serine peptidase 1(HTRA1)
M_47	220164	docking protein 6(DOK6)
M_47	51661	FK506 binding protein 7(FKBP7)
M_47	91179	scavenger receptor class F member 2(SCARF2)
M_47	349136	WD repeat domain 86(WDR86)
M_47	55384	maternally expressed 3 (non-protein coding)(MEG3)
M_47	89795	neuron navigator 3(NAV3)
M_47	55959	sulfatase 2(SULF2)
M_47	23768	fibronectin leucine rich transmembrane protein
M_47	27303	RNA binding motif single stranded interacting protein 3(RBMS3)
M_47	775	calcium voltage-gated channel subunit alpha1
M_47	22836	Rho related BTB domain containing 3(RHOBTB3)
M_47	10082	glypican 6(GPC6)

M_47	203447	Nik related kinase(NRK)
M_47	1272	contactin 1(CNTN1)
M_47	196740	V-set and transmembrane domain containing
M_47	8532	carboxypeptidase Z(CPZ)
M_47	8482	semaphorin 7A (John Milton Hagen blood
M_47	8406	sushi repeat containing protein, X-linked(SRPX)
M_47	114905	C1q and tumor necrosis factor related protein
M_47	4239	microfibrillar associated protein 4(MFAP4)
M_47	64094	SPARC related modular calcium binding 2(SMOC2)
M_47	222865	transmembrane protein 130(TMEM130)
M_47	56937	prostate transmembrane protein, androgen induced 1(PMEPA1)
M_47	117581	twist family bHLH transcription factor 2(TWIST2)
M_47	55022	phosphotyrosine interaction domain containing
M_47	81794	ADAM metallopeptidase with thrombospondin type 1 motif 10(ADAMTS10)
M_47	11173	ADAM metallopeptidase with thrombospondin type 1 motif 7(ADAMTS7)
M_47	27147	DENN domain containing 2A(DENND2A)
M_47	8913	calcium voltage-gated channel subunit alpha1
M_47	2006	elastin(ELN)
M_47	56849	transcription elongation factor A like 7(TCEAL7)
M_47	388015	retrotransposon-like 1(RTL1)
M_47	9891	NUAK family kinase 1(NUAK1)
M_47	862	RUNX1 translocation partner 1(RUNX1T1)
M_47	144347	refilin A(RFLNA)
M_47	150	adrenoceptor alpha 2A(ADRA2A)
M_47	64388	gremlin 2, DAN family BMP antagonist(GREM2)
M_47	114898	C1q and tumor necrosis factor related protein
M_47	338773	transmembrane protein 119(TMEM119)
M_47	124842	transmembrane protein 132E(TMEM132E)

M_47	6695	SPARC/osteonectin, cwcv and kazal like domains proteoglycan 1(SPOCK1)
M_47	9464	heart and neural crest derivatives expressed
M_47	10234	leucine rich repeat containing 17(LRRC17)
M_47	55691	FERM domain containing 4A(FRMD4A)
M_47	1038	cerebellar degeneration related protein 1(CDR1)
M_47	54504	carboxypeptidase, vitellogenic like(CPVL)
M_47	79104	maternally expressed 8 (non-protein coding)(MEG8)
M_47	56944	olfactomedin like 3(OLFML3)
M_47	400591	chromosome 17 open reading frame 102(C17orf102)
M_47	558	AXL receptor tyrosine kinase(AXL)
M_47	7043	transforming growth factor beta 3(TGFB3)
M_47	79804	HAND2 antisense RNA 1 (head to head)(HAND2-
M_47	5452	POU class 2 homeobox 2(POU2F2)
M_48	440078	family with sequence similarity 66 member
M_48	387849	RAB15 effector protein(REP15)
M_48	55110	mago homolog B, exon junction complex core component(MAGOHB)
M_48	23085	ELKS/RAB6-interacting/CAST family member
M_48	1911	polyhomeotic homolog 1(PHC1)
M_48	54477	pleckstrin homology domain containing
M_48	5927	lysine demethylase 5A(KDM5A)
M_48	50865	heme binding protein 1(HEBP1)
M_48	3845	KRAS proto-oncogene, GTPase(KRAS)
M_48	51768	transmembrane 7 superfamily member 3(TM7SF3)
M_48	57613	family with sequence similarity 234 member
M_48	9918	non-SMC condensin I complex subunit D2(NCAPD2)
M_48	341346	single-pass membrane protein with coiled-coil domains 2(SMCO2)
M_48	7167	triosephosphate isomerase 1(TPI1)
M_48	2842	G protein-coupled receptor 19(GPR19)
M_48	121536	AE binding protein 2(AEBP2)

M_48	121512	FYVE, RhoGEF and PH domain containing 4(FGD4)
M_48	11171	serine/threonine kinase receptor associated
M_48	25977	NECAP endocytosis associated 1(NECAP1)
M_48	50813	COP9 signalosome subunit 7A(COPS7A)
M_48	5893	RAD52 homolog, DNA repair protein(RAD52)
M_48	144363	electron transfer flavoprotein regulatory factor
M_48	3800	kinesin family member 5C(KIF5C)
M_48	2288	FK506 binding protein 4(FKBP4)
M_48	5777	protein tyrosine phosphatase, non-receptor type
M_48	374443	C-type lectin domain family 2 member D pseudogene(LOC374443)
M_48	113246	chromosome 12 open reading frame 57(C12orf57)
M_48	10046	mastermind like domain containing 1(MAMLD1)
M_48	10162	lysophosphatidylcholine acyltransferase 3(LPCAT3)
M_48	51290	ERGIC and golgi 2(ERGIC2)
M_48	171017	zinc finger protein 384(ZNF384)
M_48	503693	loss of heterozygosity, 12, chromosomal region 2 (non-protein coding)(LOH12CR2)
M_48	55297	coiled-coil domain containing 91(CCDC91)
M_48	51026	golgi transport 1B(GOLT1B)
M_48	259295	taste 2 receptor member 20(TAS2R20)
M_48	678655	CD27 antisense RNA 1(CD27-AS1)
M_48	51088	kelch like family member 5(KLHL5)
M_48	8496	PPFIA binding protein 1(PPFIBP1)
M_48	84727	splA/ryanodine receptor domain and SOCS box containing 2(SPSB2)
M_48	84318	coiled-coil domain containing 77(CCDC77)
M_48	10526	importin 8(IPO8)
M_48	55726	asunder, spermatogenesis regulator(ASUN)
M_48	55729	activating transcription factor 7 interacting
M_48	1822	atrophin 1(ATN1)
M_48	9847	C2 calcium dependent domain containing 5(C2CD5)

M_48	4055	lymphotoxin beta receptor(LTBR)
M_48	57542	kelch like family member 42(KLHL42)
M_48	7289	tubby like protein 3(TULP3)
M_48	378465	#N/A
M_48	144608	chromosome 12 open reading frame 60(C12orf60)
M_48	144699	F-box and leucine rich repeat protein 14(FBXL14)
M_48	636	BICD cargo adaptor 1(BICD1)
M_48	8079	myeloid leukemia factor 2(MLF2)
M_48	8078	ubiquitin specific peptidase 5(USP5)
M_48	5763	parathymosin(PTMS)
M_48	51258	mitochondrial ribosomal protein L51(MRPL51)
M_48	4074	mannose-6-phosphate receptor, cation
M_48	79912	pyridine nucleotide-disulphide oxidoreductase domain 1(PYROXD1)
M_48	55196	KIAA1551(KIAA1551)
M_48	4040	LDL receptor related protein 6(LRP6)
M_48	1389	cAMP responsive element binding protein like
M_48	55907	cytidine monophosphate N-acetylneuraminic acid synthetase(CMAS)
M_48	57102	chromosome 12 open reading frame 4(C12orf4)
M_48	80824	dual specificity phosphatase 16(DUSP16)
M_48	5554	proline rich protein HaeIII subfamily 1(PRH1)
M_48	9412	mediator complex subunit 21(MED21)
M_48	57103	TP53 induced glycolysis regulatory
M_48	10436	EMG1, N1-specific pseudouridine
M_48	84920	ALG10, alpha-1,2-glucosyltransferase(ALG10)
M_48	5965	RecQ like helicase(RECQL)
M_48	7004	TEA domain transcription factor 4(TEAD4)
M_48	51729	WW domain binding protein 11(WBP11)
M_48	60488	mitochondrial ribosomal protein S35(MRPS35)
M_48	56938	aryl hydrocarbon receptor nuclear translocator like 2(ARNTL2)

M_48	8798	dual specificity tyrosine phosphorylation regulated kinase 4(DYRK4)
M_48	3709	inositol 1,4,5-trisphosphate receptor type 2(ITPR2)
M_48	23012	serine/threonine kinase 38 like(STK38L)
M_48	11272	proline rich 4 (lacrimal)(PRR4)
M_48	283345	ribosomal protein L13 pseudogene 5(RPL13P5)
M_48	118426	BLOC-1 related complex subunit 5(BORCS5)
M_48	51147	inhibitor of growth family member 4(ING4)
M_48	10059	dynamin 1 like(DNM1L)
M_48	51071	deoxyribose-phosphate aldolase(DERA)
M_48	5830	peroxisomal biogenesis factor 5(PEX5)
M_48	58516	family with sequence similarity 60 member
M_48	51202	DEAD-box helicase 47(DDX47)
M_48	83714	nuclear receptor interacting protein 2(NRIP2)
M_48	51067	tyrosyl-tRNA synthetase 2(YARS2)
M_48	11331	prohibitin 2(PHB2)
M_48	83695	RAD9-HUS1-RAD1 interacting nuclear orphan
M_48	79602	adiponectin receptor 2(ADIPOR2)
M_48	196513	decapping mRNA 1B(DCP1B)
M_48	4839	NOP2 nucleolar protein(NOP2)
M_48	144245	ALG10B, alpha-1,2-glucosyltransferase(ALG10B)
M_48	65125	WNK lysine deficient protein kinase 1(WNK1)
M_48	2597	glyceraldehyde-3-phosphate dehydrogenase(GAPDH)
M_48	4704	NADH:ubiquinone oxidoreductase subunit
M_48	7132	TNF receptor superfamily member 1A(TNFRSF1A)
M_48	55846	integrin alpha FG-GAP repeat containing 2(ITFG2)
M_48	57494	ribosomal modification protein rimK like family member B(RIMKLB)
M_48	1687	DFNA5, deafness associated tumor
M_48	55500	ethanolamine kinase 1(ETNK1)
M_48	26127	FGFR1 oncogene partner 2(FGFR1OP2)
M_48	3945	lactate dehydrogenase B(LDHB)

M_48	55810	forkhead box J2(FOXJ2)
M_48	259294	taste 2 receptor member 19(TAS2R19)
M_48	2120	ETS variant 6(ETV6)
M_48	285533	ring finger protein 175(RNF175)
M_48	400027	long intergenic non-protein coding RNA
M_48	196394	antagonist of mitotic exit network 1 homolog(AMN1)
M_48	440087	single-pass membrane protein with coiled-coil domains 3(SMCO3)
M_48	51279	complement C1r subcomponent like(C1RL)
M_48	84519	acrosin binding protein(ACRBP)
M_48	254013	electron transfer flavoprotein beta subunit lysine methyltransferase(ETFBKMT)
M_48	6843	vesicle associated membrane protein 1(VAMP1)
M_48	259290	taste 2 receptor member 31(TAS2R31)
M_49	440730	tripartite motif containing 67(TRIM67)
M_49	1638	dopachrome tautomerase(DCT)
M_49	1395	corticotropin releasing hormone receptor 2(CRHR2)
M_49	389114	zinc finger protein 662(ZNF662)
M_49	344595	DPPA2 upstream binding RNA(DUBR)
M_49	27330	ribosomal protein S6 kinase A6(RPS6KA6)
M_49	1525	coxsackie virus and adenovirus receptor(CXADR)
M_49	389610	XK related 5(XKR5)
M_49	100130691	uncharacterized LOC100130691(LOC100130691)
M_49	2121	EvC ciliary complex subunit 1(EVC)
M_49	100133172	family with sequence similarity 66 member
M_49	8540	alkylglycerone phosphate synthase(AGPS)
M_49	85002	family with sequence similarity 86 member
M_49	349196	long intergenic non-protein coding RNA
M_49	286046	XK related 6(XKR6)
M_49	93655	ST7 overlapping transcript 3(ST7-OT3)
M_49	26246	olfactory receptor family 2 subfamily L member

M_49	26247	olfactory receptor family 2 subfamily L member 1 pseudogene(OR2L1P)
M_49	29761	ubiquitin specific peptidase 25(USP25)
M_49	100302640	long intergenic non-protein coding RNA
M_49	26188	olfactory receptor family 1 subfamily C member
M_49	10950	BTG anti-proliferation factor 3(BTG3)
M_49	25893	tripartite motif containing 58(TRIM58)
M_49	391195	olfactory receptor family 2 subfamily T member
M_49	391191	olfactory receptor family 2 subfamily AK member 2(OR2AK2)
M_49	391192	olfactory receptor family 2 subfamily L member
M_49	140578	chondrolectin(CHODL)
M_49	5651	transmembrane protease, serine 15(TMPRSS15)
M_49	343172	olfactory receptor family 2 subfamily T member
M_49	343169	olfactory receptor family 6 subfamily F member
M_49	343171	olfactory receptor family 2 subfamily W member
M_49	132884	EvC ciliary complex subunit 2(EVC2)
M_49	139324	highly divergent homeobox(HDX)
M_49	158297	stabilizer of axonemal microtubules 1(SAXO1)
M_49	83648	family with sequence similarity 167 member
M_49	653333	family with sequence similarity 86 member
M_49	157310	phosphatidylethanolamine binding protein 4(PEBP4)
M_49	10600	ubiquitin specific peptidase 16(USP16)
M_49	2551	GA binding protein transcription factor alpha
M_49	338069	ST7 overlapping transcript 4(ST7-OT4)
M_49	100132103	family with sequence similarity 66 member
M_49	6782	heat shock protein family A (Hsp70) member
M_49	54149	chromosome 21 open reading frame 91(C21orf91)
M_49	54033	RNA binding motif protein 11(RBM11)
M_49	441933	olfactory receptor family 13 subfamily G member
M_49	26046	listerin E3 ubiquitin protein ligase 1(LTN1)
M_49	571	BTB domain and CNC homolog 1(BACH1)

M_49	284521	olfactory receptor family 2 subfamily L member
M_49	5158	phosphodiesterase 6B(PDE6B)
M_49	100134259	long intergenic non-protein coding RNA
M_49	29104	N-6 adenine-specific DNA methyltransferase 1 (putative)(N6AMT1)
M_49	391190	olfactory receptor family 2 subfamily L member 8 (gene/pseudogene)(OR2L8)
M_49	9715	family with sequence similarity 131 member
M_49	146310	ring finger protein 151(RNF151)
M_49	254778	chromosome 8 open reading frame 46(C8orf46)
M_49	10566	A-kinase anchoring protein 3(AKAP3)
M_49	474382	H2A histone family member B1(H2AFB1)
M_49	283461	chromosome 12 open reading frame 40(C12orf40)
M_49	7791	zyxin(ZYX)
M_49	286042	family with sequence similarity 86, member A pseudogene(FAM86B3P)
M_49	57528	potassium channel tetramerization domain containing 16(KCTD16)
M_49	10069	RWD domain containing 2B(RWDD2B)
M_49	399694	SHC adaptor protein 4(SHC4)
M_49	122665	ribonuclease A family member 8(RNASE8)
M_50	677840	small nucleolar RNA, H/ACA box 71D(SNORA71D)
M_50	677841	small nucleolar RNA, H/ACA box 74B(SNORA74B)
M_50	677842	small nucleolar RNA, H/ACA box 50C(SNORA50C)
M_50	677844	small nucleolar RNA, H/ACA box 78(SNORA78)
M_50	677846	small nucleolar RNA, H/ACA box 80A(SNORA80A)
M_50	619565	small nucleolar RNA, H/ACA box 52(SNORA52)
M_50	677819	small nucleolar RNA, H/ACA box 37(SNORA37)
M_50	677825	small nucleolar RNA, H/ACA box 44(SNORA44)
M_50	114599	small nucleolar RNA, C/D box 15B(SNORD15B)
M_50	677833	small nucleolar RNA, H/ACA box 54(SNORA54)
M_50	677775	small Cajal body-specific RNA 5(SCARNA5)

M_50	6043	small nucleolar RNA, H/ACA box 63(SNORA63)
M_50	677772	small Cajal body-specific RNA 6(SCARNA6)
M_50	677801	small nucleolar RNA, H/ACA box 14A(SNORA14A)
M_50	677802	small nucleolar RNA, H/ACA box 14B(SNORA14B)
M_50	677777	small Cajal body-specific RNA 12(SCARNA12)
M_50	677776	small Cajal body-specific RNA 8(SCARNA8)
M_50	692225	small nucleolar RNA, C/D box 94(SNORD94)
M_50	677799	small nucleolar RNA, H/ACA box 11(SNORA11)
M_50	677828	small nucleolar RNA, H/ACA box 47(SNORA47)
M_50	100124534	small nucleolar RNA, H/ACA box 84(SNORA84)
M_50	619562	small nucleolar RNA, H/ACA box 3A(SNORA3A)
M_50	652966	small nucleolar RNA, C/D box 10(SNORD10)
M_50	9304	small nucleolar RNA, C/D box 22(SNORD22)
M_50	654319	small nucleolar RNA, H/ACA box 5A(SNORA5A)
M_50	652965	small nucleolar RNA, H/ACA box 48(SNORA48)
M_50	677800	small nucleolar RNA, H/ACA box 12(SNORA12)
M_50	677834	small nucleolar RNA, H/ACA box 55(SNORA55)
M_50	692148	small Cajal body-specific RNA 10(SCARNA10)
M_50	6023	RNA component of mitochondrial RNA processing endoribonuclease(RMRP)
M_50	677830	small nucleolar RNA, H/ACA box 50A(SNORA50A)
M_50	677829	small nucleolar RNA, H/ACA box 49(SNORA49)
M_50	574040	small nucleolar RNA, H/ACA box 6(SNORA6)
M_50	677839	small nucleolar RNA, H/ACA box 71C(SNORA71C)
M_50	677781	small Cajal body-specific RNA 16(SCARNA16)
M_50	26784	small nucleolar RNA, H/ACA box 64(SNORA64)
M_50	6079	small nucleolar RNA, C/D box 15A(SNORD15A)
M_50	26781	small nucleolar RNA, H/ACA box 67(SNORA67)
M_50	677808	small nucleolar RNA, H/ACA box 23(SNORA23)
M_50	677810	small nucleolar RNA, H/ACA box 26(SNORA26)
M_50	677679	small Cajal body-specific RNA 3(SCARNA3)
M_50	677681	small Cajal body-specific RNA 20(SCARNA20)

M_50	677763	small Cajal body-specific RNA 21(SCARNA21)
M_50	677807	small nucleolar RNA, H/ACA box 22(SNORA22)
M_50	85495	ribonuclease P RNA component H1(RPPH1)
M_50	692073	small nucleolar RNA, H/ACA box 16A(SNORA16A)
M_50	26777	small nucleolar RNA, H/ACA box 71A(SNORA71A)
M_50	100124536	small nucleolar RNA, H/ACA box 38B(SNORA38B)
M_50	677774	small Cajal body-specific RNA 1(SCARNA1)
M_50	677815	small nucleolar RNA, H/ACA box 2C(SNORA2C)
M_50	692158	small nucleolar RNA, H/ACA box 57(SNORA57)
M_50	692086	small nucleolar RNA, C/D box 17(SNORD17)
M_50	677806	small nucleolar RNA, H/ACA box 20(SNORA20)
M_50	677813	small nucleolar RNA, H/ACA box 30(SNORA30)
M_50	677766	small Cajal body-specific RNA 2(SCARNA2)
M_50	26780	small nucleolar RNA, H/ACA box 68(SNORA68)
M_50	692223	small nucleolar RNA, C/D box 97(SNORD97)
M_50	677809	small nucleolar RNA, H/ACA box 24(SNORA24)
M_50	677767	small Cajal body-specific RNA 7(SCARNA7)
M_50	677797	small nucleolar RNA, H/ACA box 7B(SNORA7B)
M_50	677798	small nucleolar RNA, H/ACA box 9(SNORA9)
M_50	619505	small nucleolar RNA, H/ACA box 21(SNORA21)
M_50	619383	small Cajal body-specific RNA 9(SCARNA9)
M_50	677823	small nucleolar RNA, H/ACA box 80E(SNORA80E)
M_50	100158262	small Cajal body-specific RNA 9-like(SCARNA9L)
M_50	677769	small Cajal body-specific RNA 17(SCARNA17)
M_50	26776	small nucleolar RNA, H/ACA box 71B(SNORA71B)
M_51	100288380	ULK4 pseudogene 2(ULK4P2)
M_51	442523	DPY19L2 pseudogene 4(DPY19L2P4)
M_51	26032	sushi domain containing 5(SUSD5)
M_51	151126	zinc finger protein 385B(ZNF385B)
M_51	338811	family with sequence similarity 19 member A2, C-C motif chemokine like(FAM19A2)
M_51	116966	WD repeat domain 17(WDR17)

M_51	170685	nudix hydrolase 10(NUDT10)
M_51	2823	glycoprotein M6A(GPM6A)
M_51	5140	phosphodiesterase 3B(PDE3B)
M_51	33	acyl-CoA dehydrogenase, long chain(ACADL)
M_51	114880	oxysterol binding protein like 6(OSBPL6)
M_51	2565	gamma-aminobutyric acid type A receptor gamma1 subunit(GABRG1)
M_51	89832	CHRNA7 (exons 5-10) and FAM7A (exons A-E) fusion(CHRFAM7A)
M_51	79632	family with sequence similarity 184 member
M_51	55117	solute carrier family 6 member 15(SLC6A15)
M_51	9515	syntaxin binding protein 5 like(STXBP5L)
M_51	11149	blood vessel epicardial substance(BVES)
M_51	64208	popeye domain containing 3(POPDC3)
M_51	9353	slit guidance ligand 2(SLIT2)
M_51	90293	kelch like family member 13(KLHL13)
M_51	5567	protein kinase cAMP-activated catalytic subunit beta(PRKACB)
M_51	55190	nudix hydrolase 11(NUDT11)
M_51	4139	microtubule affinity regulating kinase 1(MARK1)
M_51	140597	transcription elongation factor A like 2(TCEAL2)
M_51	132851	spermatogenesis associated 4(SPATA4)
M_51	5803	protein tyrosine phosphatase, receptor type
M_51	90161	heparan sulfate 6-O-sulfotransferase 2(HS6ST2)
M_51	89837	ULK4 pseudogene 3(ULK4P3)
M_51	143872	Rho GTPase activating protein 42(ARHGAP42)
M_51	2786	G protein subunit gamma 4(GNG4)
M_51	55086	chromosome X open reading frame 57(CXorf57)
M_51	83935	transmembrane protein 133(TMEM133)
M_51	154810	angiomotin like 1(AMOTL1)
M_51	284293	histocompatibility minor serpin domain
M_51	6422	secreted frizzled related protein 1(SFRP1)

M_51	10982	microtubule associated protein RP/EB family member 2(MAPRE2)
M_51	8092	ALX homeobox 1(ALX1)
M_51	89874	solute carrier family 25 member 21(SLC25A21)
M_51	4547	microsomal triglyceride transfer protein(MTTP)
M_51	8501	solute carrier family 43 member 1(SLC43A1)
M_51	1286	collagen type IV alpha 4 chain(COL4A4)
M_51	79739	tubulin tyrosine ligase like 7(TTLL7)
M_51	7093	tolloid like 2(TLL2)
M_51	26577	procollagen C-endopeptidase enhancer 2(PCOLCE2)
M_51	8835	suppressor of cytokine signaling 2(SOCS2)
M_51	145282	mirror-image polydactyly 1(MIPOL1)
M_51	1114	chromogranin B(CHGB)
M_51	1139	cholinergic receptor nicotinic alpha 7
M_51	9892	synaptosome associated protein 91(SNAP91)
M_51	1285	collagen type IV alpha 3 chain(COL4A3)
M_51	27445	piccolo presynaptic cytomatrix protein(PCLO)
M_51	2558	gamma-aminobutyric acid type A receptor alpha5 subunit(GABRA5)
M_51	221806	von Willebrand factor D and EGF domains(VWDE)
M_51	80350	lipoprotein(a) like 2, pseudogene(LPAL2)
M_51	6691	serine peptidase inhibitor, Kazal type 2(SPINK2)
M_51	5352	procollagen-lysine,2-oxoglutarate 5-dioxygenase
M_51	1380	complement C3d receptor 2(CR2)
M_51	4915	neurotrophic receptor tyrosine kinase 2(NTRK2)
M_51	84668	family with sequence similarity 126 member
M_51	653140	family with sequence similarity 228 member
M_51	2044	EPH receptor A5(EPHA5)
M_51	83657	dynein light chain roadblock-type 2(DYNLRB2)
M_51	2697	gap junction protein alpha 1(GJA1)
M_51	7762	zinc finger protein 215(ZNF215)
M_51	284565	neuroblastoma breakpoint family member

M_51	728936	#N/A
M_51	23333	dpy-19 like 1(DPY19L1)
M_51	54511	3-hydroxymethyl-3-methylglutaryl-CoA lyase like 1(HMGCLL1)
M_51	7480	Wnt family member 10B(WNT10B)
M_51	79589	ring finger protein 128, E3 ubiquitin protein
M_51	10003	N-acetylated alpha-linked acidic dipeptidase
M_51	3973	luteinizing hormone/choriogonadotropin
M_51	727936	glucoside xylosyltransferase 2(GXYLT2)
M_51	220202	atonal bHLH transcription factor 7(ATOH7)
M_51	84107	Zic family member 4(ZIC4)
M_51	81796	solute carrier organic anion transporter family member 5A1(SLCO5A1)
M_51	221981	thrombospondin type 1 domain containing
M_51	79802	HHIP like 2(HHIPL2)
M_51	283417	dpy-19 like 2(DPY19L2)
M_51	202374	serine/threonine kinase 32A(STK32A)
M_51	5915	retinoic acid receptor beta(RARB)
M_51	8382	NME/NM23 family member 5(NME5)
M_51	285016	family with sequence similarity 150 member
M_51	25932	chloride intracellular channel 4(CLIC4)
M_51	55283	mucolipin 3(MCOLN3)
M_51	7471	Wnt family member 1(WNT1)
M_51	148534	transmembrane protein 56(TMEM56)
M_51	11069	Rap guanine nucleotide exchange factor 4(RAPGEF4)
M_51	7545	Zic family member 1(ZIC1)
M_51	55999	nuclear RNA export factor 4 pseudogene(NXF4)
M_51	441509	glycine receptor alpha 4(GLRA4)
M_51	160428	aldehyde dehydrogenase 1 family member
M_51	10665	chromosome 6 open reading frame 10(C6orf10)
M_51	56244	butyrophilin like 2(BTNL2)
M_52	3996	LLGL1, scribble cell polarity complex

M_52	79018	GID complex subunit 4 homolog(GID4)
M_52	23326	ubiquitin specific peptidase 22(USP22)
M_52	1801	diphthamide biosynthesis 1(DPH1)
M_52	51701	nemo like kinase(NLK)
M_52	57659	zinc finger and BTB domain containing 4(ZBTB4)
M_52	124935	solute carrier family 43 member 2(SLC43A2)
M_52	55720	TSR1, ribosome maturation factor(TSR1)
M_52	10743	retinoic acid induced 1(RAI1)
M_52	708	complement C1q binding protein(C1QBP)
M_52	10749	kinesin family member 1C(KIF1C)
M_52	23399	CTD nuclear envelope phosphatase 1(CTDNEP1)
M_52	23135	lysine demethylase 6B(KDM6B)
M_52	37	acyl-CoA dehydrogenase, very long chain(ACADVL)
M_52	23140	zinc finger ZZ-type and EF-hand domain containing
M_52	10514	MYB binding protein 1a(MYBBP1A)
M_52	1973	eukaryotic translation initiation factor 4A1(EIF4A1)
M_52	4621	myosin heavy chain 3(MYH3)
M_52	11216	A-kinase anchoring protein 10(AKAP10)
M_52	1856	dishevelled segment polarity protein 2(DVL2)
M_52	23293	SMG6, nonsense mediated mRNA decay
M_52	1398	CRK proto-oncogene, adaptor protein(CRK)
M_52	5216	profilin 1(PFN1)
M_52	143244	eukaryotic translation initiation factor 5A-like
M_52	55421	nuclear cap binding subunit 3(NCBP3)
M_52	9611	nuclear receptor corepressor 1(NCOR1)
M_52	84817	thioredoxin domain containing 17(TXNDC17)
M_52	1497	cystinosin, lysosomal cystine transporter(CTNS)
M_52	6117	replication protein A1(RPA1)
M_52	9851	KIAA0753(KIAA0753)
M_52	81565	nudE neurodevelopment protein 1 like 1(NDEL1)
M_52	1140	cholinergic receptor nicotinic beta 1
M_52	5694	proteasome subunit beta 6(PSMB6)

M_52	55275	VPS53, GARP complex subunit(VPS53)
M_52	5598	mitogen-activated protein kinase 7(MAPK7)
M_52	147040	potassium channel tetramerization domain containing 11(KCTD11)
M_52	4927	nucleoporin 88(NUP88)
M_52	112483	spermidine/spermine N1-acetyltransferase family member 2(SAT2)
M_52	9905	small G protein signaling modulator 2(SGSM2)
M_52	23587	elongator acetyltransferase complex subunit 5(ELP5)
M_52	284114	transmembrane protein 102(TMEM102)
M_52	1107	chromodomain helicase DNA binding protein
M_52	9135	rabaptin, RAB GTPase binding effector protein
M_52	5338	phospholipase D2(PLD2)
M_52	114826	SET and MYND domain containing 4(SMYD4)
M_52	3927	LIM and SH3 protein 1(LASP1)
M_52	51763	inositol polyphosphate-5-phosphatase K(INPP5K)
M_52	50628	gem nuclear organelle associated protein 4(GEMIN4)
M_52	3682	integrin subunit alpha E(ITGAE)
M_52	400566	chromosome 17 open reading frame 97(C17orf97)
M_52	23729	sedoheptulokinase(SHPK)
M_52	5345	serpin family F member 2(SERPINF2)
M_52	50488	misshapen like kinase 1(MINK1)
M_52	409	arrestin beta 2(ARRB2)
M_52	27043	proline, glutamate and leucine rich protein 1(PELP1)
M_52	4302	MLLT6, PHD finger domain containing(MLLT6)
M_52	1984	eukaryotic translation initiation factor 5A(EIF5A)
M_52	79850	family with sequence similarity 57 member
M_52	124944	chromosome 17 open reading frame 49(C17orf49)
M_52	6844	vesicle associated membrane protein 2(VAMP2)
M_52	8711	tyrosine kinase non receptor 1(TNK1)
M_52	57555	neuroligin 2(NLGN2)
M_52	84268	RPA interacting protein(RPAIN)

M_52	79142	PHD finger protein 23(PHF23)
M_52	79066	methyltransferase like 16(METTL16)
M_52	201163	folliculin(FLCN)
M_52	26001	ring finger protein 167(RNF167)
M_52	80169	CST telomere replication complex component
M_52	2874	G protein pathway suppressor 2(GPS2)
M_52	58485	trafficking protein particle complex 1(TRAPPC1)
M_52	146691	target of myb1 like 2 membrane trafficking
M_52	7326	ubiquitin conjugating enzyme E2 G1(UBE2G1)
M_52	85464	slingshot protein phosphatase 2(SSH2)
M_52	55135	WD repeat containing antisense to TP53(WRAP53)
M_52	23277	clustered mitochondria homolog(CLUH)
M_52	100131454	diazepam binding inhibitor-like 5,
M_52	2314	FLII, actin remodeling protein(FLII)
M_52	10594	pre-mRNA processing factor 8(PRPF8)
M_52	57048	phospholipid scramblase 3(PLSCR3)
M_52	2256	fibroblast growth factor 11(FGF11)
M_52	57649	PHD finger protein 12(PHF12)
M_52	7775	zinc finger protein 232(ZNF232)
M_52	57336	zinc finger protein 287(ZNF287)
M_52	83460	ER membrane protein complex subunit 6(EMC6)
M_52	9513	FMR1 autosomal homolog 2(FXR2)
M_52	256302	N-acetyltransferase domain containing 1(NATD1)
M_52	124641	ovarian tumor suppressor candidate 2(OVCA2)
M_52	124637	cytochrome b5 domain containing 1(CYB5D1)
M_52	124751	KRAB-A domain containing 2(KRBA2)
M_52	30837	suppressor of cytokine signaling 7(SOCS7)
M_52	284040	CMT1A duplicated region transcript 4(CDRT4)
M_52	284029	long intergenic non-protein coding RNA
M_52	284023	uncharacterized LOC284023(LOC284023)
M_52	140775	Smith-Magenis syndrome chromosome region, candidate 8(SMCR8)

M_52	9703	KIAA0100(KIAA0100)
M_52	51031	glyoxalase domain containing 4(GLOD4)
M_52	54785	BLOC-1 related complex subunit 6(BORCS6)
M_52	9706	unc-51 like autophagy activating kinase 2(ULK2)
M_52	4335	MAX network transcriptional repressor(MNT)
M_52	79003	MIS12, kinetochore complex component(MIS12)
M_52	9552	sperm associated antigen 7(SPAG7)
M_52	7156	topoisomerase (DNA) III alpha(TOP3A)
M_52	84461	neuralized E3 ubiquitin protein ligase 4(NEURL4)
M_52	56919	DEAH-box helicase 33(DHX33)
M_52	116840	centrobin, centriole duplication and spindle assembly protein(CNTROB)
M_52	55731	family with sequence similarity 222 member
M_52	57547	zinc finger protein 624(ZNF624)
M_52	55178	mitochondrial rRNA methyltransferase 3(MRM3)
M_52	9098	ubiquitin specific peptidase 6(USP6)
M_52	51479	ankyrin repeat and FYVE domain containing
M_52	26168	SUMO1/sentrin/SMT3 specific peptidase 3(SENP3)
M_52	29928	translocase of inner mitochondrial membrane
M_52	29098	RAN guanine nucleotide release factor(RANGRF)
M_52	5430	RNA polymerase II subunit A(POLR2A)
M_52	782	calcium voltage-gated channel auxiliary subunit beta 1(CACNB1)
M_52	7442	transient receptor potential cation channel subfamily V member 1(TRPV1)
M_52	4641	myosin IC(MYO1C)
M_52	54883	CWC25 spliceosome associated protein
M_52	5198	phosphoribosylformylglycinamidine synthase(PFAS)
M_52	11337	GABA type A receptor-associated
M_52	84622	zinc finger protein 594(ZNF594)
M_52	6830	SPT6 homolog, histone chaperone(SUPT6H)
M_52	9526	mannose-P-dolichol utilization defect 1(MPDU1)

M_52	84314	transmembrane protein 107(TMEM107)
M_52	388324	inhibitor of CDK, cyclin A1 interacting protein
M_52	57532	NUFIP2, FMR1 interacting protein 2(NUFIP2)
M_52	54890	alkB homolog 5, RNA demethylase(ALKBH5)
M_52	8741	tumor necrosis factor superfamily member
M_52	388327	chromosome 17 open reading frame 100(C17orf100)
M_52	51003	mediator complex subunit 31(MED31)
M_52	51009	derlin 2(DERL2)
M_52	124997	WD repeat domain 81(WDR81)
M_52	124961	ZFP3 zinc finger protein(ZFP3)
M_52	100128288	uncharacterized LOC100128288(LOC100128288)
M_52	8402	solute carrier family 25 member 11(SLC25A11)
M_52	22905	epsin 2(EPN2)
M_52	5048	platelet activating factor acetylhydrolase 1b regulatory subunit 1(PAFAH1B1)
M_52	8396	phosphatidylinositol-5-phosphate 4-kinase type 2 beta(PIP4K2B)
M_52	94005	phosphatidylinositol glycan anchor biosynthesis class
M_52	23125	calmodulin binding transcription activator
M_52	6462	sex hormone binding globulin(SHBG)
M_52	5606	mitogen-activated protein kinase kinase 3(MAP2K3)
M_52	5306	phosphatidylinositol transfer protein alpha(PITPNA)
M_52	7126	TNF alpha induced protein 1(TNFAIP1)
M_52	30851	Tax1 binding protein 3(TAX1BP3)
M_52	7531	tyrosine 3-monooxygenase/tryptophan 5-monooxygenase activation protein epsilon(YWHAE)
M_52	84225	zinc finger MYND-type containing 15(ZMYND15)
M_52	10493	vesicle amine transport 1(VAT1)
M_52	84033	obscurin, cytoskeletal calmodulin and titin-interacting RhoGEF(OBSCN)
M_52	5187	period circadian clock 1(PER1)
M_52	162394	schlafen family member 5(SLFN5)

M_52	374768	spermatid maturation 1(SPEM1)
M_52	58191	C-X-C motif chemokine ligand 16(CXCL16)
M_52	644815	family with sequence similarity 83 member
M_52	7703	polycomb group ring finger 2(PCGF2)
M_52	5463	POU class 6 homeobox 1(POU6F1)
M_52	2027	enolase 3(ENO3)
M_52	440400	ribonuclease K(RNASEK)
M_52	487	ATPase sarcoplasmic/endoplasmic reticulum Ca2+ transporting 1(ATP2A1)
M_52	201243	chromosome 17 open reading frame 74(C17orf74)
M_52	81575	apolipoprotein L domain containing 1(APOLD1)
M_52	400569	mediator complex subunit 11(MED11)
M_52	125206	solute carrier family 5 member 10(SLC5A10)
M_53	4087	SMAD family member 2(SMAD2)
M_53	23556	phosphatidylinositol glycan anchor biosynthesis class
M_53	4200	malic enzyme 2(ME2)
M_53	3998	lectin, mannose binding 1(LMAN1)
M_53	7776	zinc finger protein 236(ZNF236)
M_53	400657	long intergenic non-protein coding RNA
M_53	23335	WD repeat domain 7(WDR7)
M_53	79863	ribosome binding factor A (putative)(RBFA)
M_53	5289	phosphatidylinositol 3-kinase catalytic subunit type
M_53	115106	HAUS augmin like complex subunit 1(HAUS1)
M_53	4645	myosin VB(MYO5B)
M_53	57724	ectopic P-granules autophagy protein 5
M_53	51320	mex-3 RNA binding family member C(MEX3C)
M_53	57614	KIAA1468(KIAA1468)
M_53	162681	chromosome 18 open reading frame 54(C18orf54)
M_53	5205	ATPase phospholipid transporting 8B1(ATP8B1)
M_53	9811	cap binding complex dependent translation initiation factor(CTIF)

M_53	23327	neural precursor cell expressed, developmentally down-regulated 4-like, E3 ubiquitin protein
M_53	374868	ATPase phospholipid transporting 9B
M_53	54495	thioredoxin related transmembrane protein 3(TMX3)
M_53	8932	methyl-CpG binding domain protein 2(MBD2)
M_53	90701	SEC11 homolog C, signal peptidase complex
M_53	80148	PQ loop repeat containing 1(PQLC1)
M_53	9306	suppressor of cytokine signaling 6(SOCS6)
M_53	55520	elaC ribonuclease Z 1(ELAC1)
M_53	30827	CXXC finger protein 1(CXXC1)
M_53	22850	ADNP homeobox 2(ADNP2)
M_53	9352	thioredoxin like 1(TXNL1)
M_53	9063	protein inhibitor of activated STAT 2(PIAS2)
M_53	55205	zinc finger protein 532(ZNF532)
M_53	29090	translocase of inner mitochondrial membrane
M_53	1528	cytochrome b5 type A(CYB5A)
M_53	147339	chromosome 18 open reading frame 25(C18orf25)
M_53	54808	dymeclin(DYM)
M_53	9150	CTD phosphatase subunit 1(CTDP1)
M_53	440498	heat shock factor binding protein 1 like 1(HSBP1L1)
M_53	10194	teashirt zinc finger homeobox 1(TSHZ1)
M_53	2531	3-ketodihydrosphingosine reductase(KDSR)
M_53	51124	immediate early response 3 interacting protein
M_53	4155	myelin basic protein(MBP)
M_53	55748	CNDP dipeptidase 2 (metallopeptidase M20
M_53	4152	methyl-CpG binding domain protein 1(MBD1)
M_53	54877	zinc finger CCHC-type containing 2(ZCCHC2)
M_53	4089	SMAD family member 4(SMAD4)
M_53	10892	MALT1 paracaspase(MALT1)
M_53	100130522	PARD6G antisense RNA 1(PARD6G-AS1)
M_53	284273	zinc binding alcohol dehydrogenase domain containing 2(ZADH2)

M_53	10449	acetyl-CoA acyltransferase 2(ACAA2)
M_53	84064	haloacid dehalogenase like hydrolase domain containing 2(HDHD2)
M_53	84735	carnosine dipeptidase 1(CNDP1)
M_53	23239	PH domain and leucine rich repeat protein phosphatase 1(PHLPP1)
M_53	55628	zinc finger protein 407(ZNF407)
M_53	9525	vacuolar protein sorting 4 homolog B(VPS4B)
M_53	497661	chromosome 18 open reading frame 32(C18orf32)
M_53	498	ATP synthase, H+ transporting, mitochondrial F1 complex, alpha subunit 1, cardiac muscle(ATP5A1)
M_53	4677	asparaginyl-tRNA synthetase(NARS)
M_53	10907	thioredoxin like 4A(TXNL4A)
M_53	11201	DNA polymerase iota(POLI)
M_53	25914	rotatin(RTTN)
M_53	2235	ferrochelatase(FECH)
M_53	26040	SET binding protein 1(SETBP1)
M_53	5366	phorbol-12-myristate-13-acetate-induced protein
M_53	347735	serine incorporator 2(SERINC2)
M_53	388125	C2 calcium dependent domain containing
M_53	10135	nicotinamide phosphoribosyltransferase(NAMPT)
M_53	135112	nuclear receptor coactivator 7(NCOA7)
M_53	145741	C2 calcium dependent domain containing
M_53	168455	coiled-coil domain containing 71-like(CCDC71L)
M_53	199920	chromosome 1 open reading frame 168(C1orf168)
M_54	5528	protein phosphatase 2 regulatory subunit
M_54	93643	tight junction associated protein 1(TJAP1)
M_54	89845	ATP binding cassette subfamily C member
M_54	23506	GLTSCR1 like(GLTSCR1L)
M_54	728239	MAGE family member D4(MAGED4)
M_54	2794	G protein nucleolar 1 (putative)(GNL1)
M_54	6046	bromodomain containing 2(BRD2)

M_54	705	bystin like(BYSL)
M_54	23787	mitochondrial carrier 1(MTCH1)
M_54	2030	solute carrier family 29 member 1 (Augustine blood group)(SLC29A1)
M_54	388931	major facilitator superfamily domain containing
M_54	285855	ribosomal protein L7 like 1(RPL7L1)
M_54	5754	protein tyrosine kinase 7 (inactive)(PTK7)
M_54	10919	euchromatic histone lysine methyltransferase
M_54	57510	exportin 5(XPO5)
M_54	54676	GTP binding protein 2(GTPBP2)
M_54	23294	ankyrin repeat and sterile alpha motif domain containing 1A(ANKS1A)
M_54	25862	ubiquitin specific peptidase 49(USP49)
M_54	1616	death domain associated protein(DAXX)
M_54	988	cell division cycle 5 like(CDC5L)
M_54	9025	ring finger protein 8(RNF8)
M_54	9656	mediator of DNA damage checkpoint 1(MDC1)
M_54	6257	retinoid X receptor beta(RXRB)
M_54	7916	proline rich coiled-coil 2A(PRRC2A)
M_54	7917	BCL2 associated athanogene 6(BAG6)
M_54	23	ATP binding cassette subfamily F member 1(ABCF1)
M_54	27154	bromodomain and PHD finger containing 3(BRPF3)
M_54	7922	solute carrier family 39 member 7(SLC39A7)
M_54	55776	SAYSVFN motif domain containing 1(SAYSD1)
M_54	5863	ral guanine nucleotide dissociation stimulator like
M_54	5429	DNA polymerase eta(POLH)
M_54	23304	ubiquitin protein ligase E3 component n-recognin
M_54	129685	TATA-box binding protein associated factor 8(TAF8)
M_54	23113	cullin 9(CUL9)
M_54	221477	chromosome 6 open reading frame 89(C6orf89)
M_54	9820	cullin 7(CUL7)
M_54	5467	peroxisome proliferator activated receptor

M_54	221496	LEM domain containing 2(LEMD2)
M_54	6722	serum response factor(SRF)
M_54	54887	UHRF1 binding protein 1(UHRF1BP1)
M_54	9477	mediator complex subunit 20(MED20)
M_54	64771	chromosome 6 open reading frame 106(C6orf106)
M_54	5089	PBX homeobox 2(PBX2)
M_54	7005	TEA domain transcription factor 3(TEAD3)
M_54	9374	palmitoyl-protein thioesterase 2(PPT2)
M_54	6499	Ski2 like RNA helicase(SKIV2L)
M_54	7407	valyl-tRNA synthetase(VARS)
M_54	56658	tripartite motif containing 39(TRIM39)
M_54	89953	kinesin light chain 4(KLC4)
M_54	9278	zinc finger and BTB domain containing 22(ZBTB22)
M_54	9277	WD repeat domain 46(WDR46)
M_54	2178	Fanconi anemia complementation group E(FANCE)
M_54	4800	nuclear transcription factor Y subunit alpha(NFYA)
M_54	1432	mitogen-activated protein kinase 14(MAPK14)
M_54	55362	transmembrane protein 63B(TMEM63B)
M_54	6293	VPS52, GARP complex subunit(VPS52)
M_54	23070	cap methyltransferase 1(CMTR1)
M_54	6732	SRSF protein kinase 1(SRPK1)
M_54	10554	1-acylglycerol-3-phosphate O-acyltransferase
M_54	57827	chromosome 6 open reading frame 47(C6orf47)
M_54	24149	zinc finger protein 318(ZNF318)
M_54	60685	zinc finger AN1-type containing 3(ZFAND3)
M_54	221504	zinc finger and BTB domain containing 9(ZBTB9)
M_54	11329	serine/threonine kinase 38(STK38)
M_54	222658	potassium channel tetramerization domain containing 20(KCTD20)
M_54	57505	alanyl-tRNA synthetase 2, mitochondrial(AARS2)
M_54	55633	TBC1 domain family member 22B(TBC1D22B)
M_54	114781	BTB domain containing 9(BTBD9)

M_54	3326	heat shock protein 90 alpha family class B member 1(HSP90AB1)
M_54	8449	DEAH-box helicase 16(DHX16)
M_54	3159	high mobility group AT-hook 1(HMGA1)
M_54	54718	butyrophilin subfamily 2 member A3,
M_54	2968	general transcription factor IIH subunit 4(GTF2H4)
M_54	9444	QKI, KH domain containing RNA binding(QKI)
M_54	441151	transmembrane protein 151B(TMEM151B)
M_54	203068	tubulin beta class I(TUBB)
M_54	1388	activating transcription factor 6 beta(ATF6B)
M_54	54535	coiled-coil alpha-helical rod protein 1(CCHCR1)
M_54	7287	tubby like protein 1(TULP1)
M_54	8859	serine/threonine kinase 19(STK19)
M_54	57176	valyl-tRNA synthetase 2, mitochondrial(VARS2)
M_54	79969	alpha tubulin acetyltransferase 1(ATAT1)
M_54	729633	MRS2 pseudogene 2(MRS2P2)
M_55	5529	protein phosphatase 2 regulatory subunit
M_55	7528	YY1 transcription factor(YY1)
M_55	27030	mutL homolog 3(MLH3)
M_55	1743	dihydrolipoamide S-succinyltransferase(DLST)
M_55	122945	NADP dependent oxidoreductase domain containing 1(NOXRED1)
M_55	4293	mitogen-activated protein kinase kinase kinase
M_55	122809	suppressor of cytokine signaling 4(SOCS4)
M_55	55102	autophagy related 2B(ATG2B)
M_55	22938	SNW domain containing 1(SNW1)
M_55	79882	zinc finger CCCH-type containing 14(ZC3H14)
M_55	11099	protein tyrosine phosphatase, non-receptor type
M_55	6400	SEL1L ERAD E3 ligase adaptor subunit(SEL1L)
M_55	112840	WD repeat domain 89(WDR89)
M_55	22890	zinc finger and BTB domain containing 1(ZBTB1)
M_55	55671	protein phosphatase 4 regulatory subunit

M_55	55334	solute carrier family 39 member 9(SLC39A9)
M_55	55333	synaptojanin 2 binding protein(SYNJ2BP)
M_55	55668	G-patch domain containing 2 like(GPATCH2L)
M_55	10640	exocyst complex component 5(EXOC5)
M_55	161394	sterile alpha motif domain containing 15(SAMD15)
M_55	1965	eukaryotic translation initiation factor 2 subunit
M_55	51109	retinol dehydrogenase 11 (all-trans/9-cis/11-
M_55	22990	pecanex homolog 1 (Drosophila)(PCNX1)
M_55	51528	JNK1/MAPK8-associated membrane protein(JKAMP)
M_55	64207	interferon regulatory factor 2 binding protein
M_55	56252	YLP motif containing 1(YLPM1)
M_55	26150	RIB43A domain with coiled-coils 2(RIBC2)
M_55	384	arginase 2(ARG2)
M_55	3091	hypoxia inducible factor 1 alpha subunit(HIF1A)
M_55	10972	transmembrane p24 trafficking protein 10(TMED10)
M_55	122830	N(alpha)-acetyltransferase 30, NatC catalytic subunit(NAA30)
M_55	57143	aarF domain containing kinase 1(ADCK1)
M_55	4287	ataxin 3(ATXN3)
M_55	123016	tetratricopeptide repeat domain 8(TTC8)
M_55	23093	tubulin tyrosine ligase like 5(TTLL5)
M_55	23503	zinc finger FYVE-type containing 26(ZFYVE26)
M_55	9240	paraneoplastic Ma antigen 1(PNMA1)
M_55	10490	vesicle transport through interaction with t-SNAREs 1B(VTI1B)
M_55	4329	aldehyde dehydrogenase 6 family member
M_55	4331	MNAT1, CDK activating kinase assembly
M_55	57475	pleckstrin homology, MyTH4 and FERM domain containing H1(PLEKHH1)
M_55	9252	ribosomal protein S6 kinase A5(RPS6KA5)
M_55	55727	BTB domain containing 7(BTBD7)
M_55	5494	protein phosphatase, Mg2+/Mn2+ dependent

M_55	55775	tyrosyl-DNA phosphodiesterase 1(TDP1)
M_55	64112	modulator of apoptosis 1(MOAP1)
M_55	8816	DDB1 and CUL4 associated factor 5(DCAF5)
M_55	4522	methylenetetrahydrofolate dehydrogenase, cyclohydrolase and formyltetrahydrofolate synthetase
M_55	55051	NRDE-2, necessary for RNA interference, domain containing(NRDE2)
M_55	80127	basal body orientation factor 1(BBOF1)
M_55	57862	zinc finger protein 410(ZNF410)
M_55	9895	tectonin beta-propeller repeat containing 2(TECPR2)
M_55	55218	exonuclease 3'-5' domain containing 2(EXD2)
M_55	22863	autophagy related 14(ATG14)
M_55	9321	thyroid hormone receptor interactor 11(TRIP11)
M_55	5663	presenilin 1(PSEN1)
M_55	54916	transmembrane protein 260(TMEM260)
M_55	53981	cleavage and polyadenylation specific factor
M_55	9786	KIAA0586(KIAA0586)
M_55	5826	ATP binding cassette subfamily D member 4(ABCD4)
M_55	29954	protein O-mannosyltransferase 2(POMT2)
M_55	8846	alkB homolog 1, histone H2A dioxygenase(ALKBH1)
M_55	55812	spermatogenesis associated 7(SPATA7)
M_55	53349	zinc finger FYVE-type containing 1(ZFYVE1)
M_55	6617	small nuclear RNA activating complex polypeptide 1(SNAPC1)
M_55	9950	golgin A5(GOLGA5)
M_55	93487	mitogen-activated protein kinase 1 interacting protein 1 like(MAPK1IP1L)
M_55	63894	VPS33B interacting protein, apical-basolateral polarity regulator, spe-39 homolog(VIPAS39)
M_55	51077	FCF1 rRNA-processing protein(FCF1)
M_55	23186	REST corepressor 1(RCOR1)
M_55	23357	angel homolog 1(ANGEL1)

M_55	10001	mediator complex subunit 6(MED6)
M_55	8650	NUMB, endocytic adaptor protein(NUMB)
M_55	55745	adaptor related protein complex 5 mu 1
M_55	26037	signal induced proliferation associated 1 like
M_55	91754	NIMA related kinase 9(NEK9)
M_55	8892	eukaryotic translation initiation factor 2B subunit
M_55	91748	ELM2 and Myb/SANT domain containing
M_55	5890	RAD51 paralog B(RAD51B)
M_55	2342	farnesyltransferase, CAAX box, beta(FNTB)
M_55	51382	ATPase H+ transporting V1 subunit D(ATP6V1D)
M_55	79789	calmin(CLMN)
M_55	83544	dynein axonemal light chain 1(DNAL1)
M_55	440193	coiled-coil domain containing 88C(CCDC88C)
M_55	122481	adenylate kinase 7(AK7)
M_55	51004	coenzyme Q6, monooxygenase(COQ6)
M_55	8812	cyclin K(CCNK)
M_55	85457	CLOCK interacting pacemaker(CIPC)
M_55	283578	transmembrane p24 trafficking protein family member 8(TMED8)
M_55	145508	centrosomal protein 128(CEP128)
M_55	23405	dicer 1, ribonuclease III(DICER1)
M_55	91750	lin-52 DREAM MuvB core complex
M_55	9870	apoptosis resistant E3 ubiquitin protein ligase
M_55	79697	chromosome 14 open reading frame 169(C14orf169)
M_55	79696	zinc finger C2HC-type containing 1C(ZC2HC1C)
M_55	26094	DDB1 and CUL4 associated factor 4(DCAF4)
M_55	341019	doublecortin domain containing 1(DCDC1)
M_55	64430	pecanex homolog 4 (Drosophila)(PCNX4)
M_55	10598	activator of Hsp90 ATPase activity 1(AHSA1)
M_55	9578	CDC42 binding protein kinase beta(CDC42BPB)
M_55	57570	tRNA methyltransferase 5(TRMT5)
M_55	2581	galactosylceramidase(GALC)

M_55	11161	chromosome 14 open reading frame 1(C14orf1)
M_55	57062	DEAD-box helicase 24(DDX24)
M_55	10914	poly(A) polymerase alpha(PAPOLA)
M_55	2957	general transcription factor IIA subunit 1(GTF2A1)
M_55	64398	membrane palmitoylated protein 5(MPP5)
M_55	2954	glutathione S-transferase zeta 1(GSTZ1)
M_55	1778	dynein cytoplasmic 1 heavy chain 1(DYNC1H1)
M_55	9517	serine palmitoyltransferase long chain base subunit 2(SPTLC2)
M_55	145483	family with sequence similarity 161 member
M_55	957	ectonucleoside triphosphate diphosphohydrolase
M_55	115811	IQ motif containing D(IQCD)
M_55	161436	echinoderm microtubule associated protein like
M_55	255101	cilia and flagella associated protein 65(CFAP65)
M_55	283385	MORN repeat containing 3(MORN3)
M_55	78990	OTU deubiquitinase, ubiquitin aldehyde binding
M_55	151651	EF-hand domain family member B(EFHB)
M_55	112752	intraflagellar transport 43(IFT43)
M_55	10505	ssemaphorin 4F(SEMA4F)
M_55	6430	serine and arginine rich splicing factor 5(SRSF5)
M_56	818	calcium/calmodulin dependent protein kinase II gamma(CAMK2G)
M_56	310	annexin A7(ANXA7)
M_56	221035	receptor accessory protein 3(REEP3)
M_56	4173	minichromosome maintenance complex component
M_56	195828	zinc finger protein 367(ZNF367)
M_56	23234	DnaJ heat shock protein family (Hsp40) member C9(DNAJC9)
M_56	29982	nuclear receptor binding factor 2(NRBF2)
M_56	27069	growth hormone inducible transmembrane
M_56	5532	protein phosphatase 3 catalytic subunit beta(PPP3CB)
M_56	11319	ecdysoneless cell cycle regulator(ECD)

M_56	317662	family with sequence similarity 149 member
M_56	118487	coiled-coil-helix-coiled-coil-helix domain containing 1(CHCHD1)
M_56	219743	trypsin domain containing 1(TYSND1)
M_56	219738	chromosome 10 open reading frame 35(C10orf35)
M_56	26091	HECT and RLD domain containing E3 ubiquitin protein ligase 4(HERC4)
M_56	9134	cyclin E2(CCNE2)
M_56	5464	pyrophosphatase (inorganic) 1(PPA1)
M_56	3070	helicase, lymphoid-specific(HELLS)
M_56	6941	transcription factor 19(TCF19)
M_56	119504	anaphase promoting complex subunit 16(ANAPC16)
M_56	6832	Suv3 like RNA helicase(SUPV3L1)
M_56	5092	pterin-4 alpha-carbinolamine dehydratase 1(PCBD1)
M_56	10592	structural maintenance of chromosomes 2(SMC2)
M_56	2237	flap structure-specific endonuclease 1(FEN1)
M_56	374393	family with sequence similarity 111 member
M_56	8879	sphingosine-1-phosphate lyase 1(SGPL1)
M_56	8034	solute carrier family 25 member 16(SLC25A16)
M_56	79968	WD repeat domain 76(WDR76)
M_56	51008	activating signal cointegrator 1 complex subunit
M_56	5591	protein kinase, DNA-activated, catalytic
M_56	4175	minichromosome maintenance complex component
M_56	26985	adaptor related protein complex 3 mu 1
M_56	90550	mitochondrial calcium uniporter(MCU)
M_56	54788	DnaJ heat shock protein family (Hsp40) member B12(DNAJB12)
M_56	51021	mitochondrial ribosomal protein S16(MRPS16)
M_56	7321	ubiquitin conjugating enzyme E2 D1(UBE2D1)
M_56	7417	voltage dependent anion channel 2(VDAC2)
M_56	10105	peptidylprolyl isomerase F(PPIF)
M_56	142891	sterile alpha motif domain containing 8(SAMD8)

M_56	23411	sirtuin 1(SIRT1)
M_56	10367	mitochondrial calcium uptake 1(MICU1)
M_56	8509	N-deacetylase and N-sulfotransferase 2(NDST2)
M_56	79009	DExD-box helicase 50(DDX50)
M_56	9559	VPS26, retromer complex component A(VPS26A)
M_56	55680	RUN and FYVE domain containing 2(RUFY2)
M_56	9188	DExD-box helicase 21(DDX21)
M_56	9632	SEC24 homolog C, COPII coat complex
M_56	7019	transcription factor A, mitochondrial(TFAM)
M_56	56681	secretion associated Ras related GTPase 1A(SAR1A)
M_56	55847	CDGSH iron sulfur domain 1(CISD1)
M_56	55222	leucine rich repeat containing 20(LRRC20)
M_56	26128	KIF1 binding protein(KIF1BP)
M_56	11130	ZW10 interacting kinetochore protein(ZWINT)
M_56	1979	eukaryotic translation initiation factor 4E binding protein 2(EIF4EBP2)
M_56	132	adenosine kinase(ADK)
M_56	23053	zinc finger SWIM-type containing 8(ZSWIM8)
M_56	8031	nuclear receptor coactivator 4(NCOA4)
M_56	64081	phenazine biosynthesis like protein domain
M_56	83879	cell division cycle associated 7(CDCA7)
M_56	81620	chromatin licensing and DNA replication factor
M_56	170384	fucosyltransferase 11(FUT11)
M_56	4171	minichromosome maintenance complex component
M_56	11270	nurim (nuclear envelope membrane protein)(NRM)
M_56	84989	JMJD1C antisense RNA 1(JMJD1C-AS1)
M_56	5557	primase (DNA) subunit 1(PRIM1)
M_56	159195	ubiquitin specific peptidase 54(USP54)
M_56	23649	DNA polymerase alpha 2, accessory subunit(POLA2)
M_56	25961	nudix hydrolase 13(NUDT13)
M_56	4001	lamin B1(LMNB1)
M_56	2175	Fanconi anemia complementation group A(FANCA)

M_56	51659	GINS complex subunit 2(GINS2)
M_56	253430	inositol polyphosphate multikinase(IPMK)
M_56	55839	centromere protein N(CENPN)
M_56	84890	2-aminoethanethiol dioxygenase(ADO)
M_56	1017	cyclin dependent kinase 2(CDK2)
M_57	23324	mannosidase alpha class 2B member 2(MAN2B2)
M_57	1609	diacylglycerol kinase theta(DGKQ)
M_57	6002	regulator of G-protein signaling 12(RGS12)
M_57	10227	major facilitator superfamily domain containing
M_57	285550	family with sequence similarity 200 member
M_57	317648	NOP14 antisense RNA 1(NOP14-AS1)
M_57	3064	huntingtin(HTT)
M_57	7884	stem-loop binding protein(SLBP)
M_57	6047	ring finger protein 4(RNF4)
M_57	7468	Wolf-Hirschhorn syndrome candidate 1(WHSC1)
M_57	114932	Morf4 family associated protein 1 like 1(MRFAP1L1)
M_57	3425	iduronidase, alpha-L-(IDUA)
M_57	57181	solute carrier family 39 member 10(SLC39A10)
M_57	79155	TNFAIP3 interacting protein 2(TNIP2)
M_57	80273	GrpE like 1, mitochondrial(GRPEL1)
M_57	55646	Ly1 antibody reactive(LYAR)
M_57	10861	solute carrier family 26 member 1(SLC26A1)
M_57	6452	SH3 domain binding protein 2(SH3BP2)
M_57	654254	zinc finger protein 732(ZNF732)
M_57	57654	UV stimulated scaffold protein A(UVSSA)
M_57	3954	leucine zipper and EF-hand containing transmembrane protein 1(LETM1)
M_57	53834	fibroblast growth factor receptor-like 1(FGFRL1)
M_57	79963	ATP binding cassette subfamily A member 11, pseudogene(ABCA11P)
M_57	202018	transmembrane anterior posterior transformation
M_57	152877	family with sequence similarity 53 member

M_57	8310	acyl-CoA oxidase 3, pristanoyl(ACOX3)
M_57	170960	zinc finger protein 721(ZNF721)
M_57	84286	transmembrane protein 175(TMEM175)
M_57	259282	biorientation of chromosomes in cell division 1 like
M_57	93624	transcriptional adaptor 2B(TADA2B)
M_57	93622	Morf4 family associated protein 1 like 1 pseudogene(LOC93622)
M_57	92070	CTBP1 antisense RNA 2 (head to head)(CTBP1-AS2)
M_57	55330	biogenesis of lysosomal organelles complex 1 subunit 4(BLOC1S4)
M_57	1487	C-terminal binding protein 1(CTBP1)
M_57	80306	mediator complex subunit 28(MED28)
M_57	85013	transmembrane protein 128(TMEM128)
M_57	93621	Morf4 family associated protein 1(MRFAP1)
M_57	133015	PARK2 coregulated like(PACRGL)
M_57	57545	coiled-coil and C2 domain containing 2A(CC2D2A)
M_57	9364	RAB28, member RAS oncogene family(RAB28)
M_57	9778	KIAA0232(KIAA0232)
M_57	152687	zinc finger protein 595(ZNF595)
M_57	10336	polycomb group ring finger 3(PCGF3)
M_57	285464	cysteine rich PAK1 inhibitor(CRIPAK)
M_57	642280	zinc finger protein 876, pseudogene(ZNF876P)
M_57	389203	small integral membrane protein 20(SMIM20)
M_57	10296	macrophage erythroblast attacher(MAEA)
M_57	166793	zinc finger and BTB domain containing 49(ZBTB49)
M_57	9948	WD repeat domain 1(WDR1)
M_57	92305	transmembrane protein 129(TMEM129)
M_57	7469	negative elongation factor complex member
M_57	79441	HAUS augmin like complex subunit 3(HAUS3)
M_57	166647	adhesion G protein-coupled receptor A3(ADGRA3)
M_57	348926	family with sequence similarity 86, member A pseudogene(FAM86EP)

M_57	4043	LDL receptor related protein associated protein
M_57	8603	family with sequence similarity 193 member
M_57	8602	NOP14 nucleolar protein(NOP14)
M_57	57533	TBC1 domain family member 14(TBC1D14)
M_57	521	ATP synthase, H+ transporting, mitochondrial Fo complex subunit E(ATP5I)
M_57	339983	N-acetyltransferase 8 like(NAT8L)
M_57	5860	quinoid dihydropteridine reductase(QDPR)
M_57	54876	DDB1 and CUL4 associated factor 16(DCAF16)
M_57	54872	phosphatidylinositol glycan anchor biosynthesis class
M_57	4636	myosin light chain 5(MYL5)
M_57	26234	F-box and leucine rich repeat protein 5(FBXL5)
M_57	118	adducin 1(ADD1)
M_57	7700	zinc finger protein 141(ZNF141)
M_57	2868	G protein-coupled receptor kinase 4(GRK4)
M_57	10608	MAX dimerization protein 4(MXD4)
M_57	255403	zinc finger protein 718(ZNF718)
M_57	2580	cyclin G associated kinase(GAK)
M_57	80333	potassium voltage-gated channel interacting protein 4(KCNIP4)
M_57	353497	DNA polymerase nu(POLN)
M_57	55713	zinc finger protein 334(ZNF334)
M_57	64577	aldehyde dehydrogenase 8 family member
M_57	55888	zinc finger with KRAB and SCAN domains
M_57	100240726	#N/A
M_57	132864	cytoplasmic polyadenylation element binding protein
M_57	2660	myostatin(MSTN)
M_57	57732	zinc finger FYVE-type containing 28(ZFYVE28)
M_57	345193	leucine rich repeat, Ig-like and transmembrane domains 3(LRIT3)
M_57	10692	retinal pigment epithelium-derived rhodopsin
M_57	390598	SKI family transcriptional corepressor 1(SKOR1)

M_58	23654	plexin B2(PLXNB2)
M_58	253190	serine hydrolase-like 2(SERHL2)
M_58	79879	coiled-coil domain containing 134(CCDC134)
M_58	91289	lipase maturation factor 2(LMF2)
M_58	415116	Pim-3 proto-oncogene, serine/threonine kinase(PIM3)
M_58	83642	selenoprotein O(SELENOO)
M_58	468	activating transcription factor 4(ATF4)
M_58	27351	desumoylating isopeptidase 1(DESI1)
M_58	27341	ribosomal RNA processing 7 homolog A(RRP7A)
M_58	84844	PHD finger protein 5A(PHF5A)
M_58	54471	mitochondrial elongation factor 1(MIEF1)
M_58	6942	transcription factor 20(TCF20)
M_58	55267	proline rich 34(PRR34)
M_58	2033	E1A binding protein p300(EP300)
M_58	6721	sterol regulatory element binding transcription factor 2(SREBF2)
M_58	79087	ALG12, alpha-1,6-mannosyltransferase(ALG12)
M_58	10126	dynein axonemal light chain 4(DNAL4)
M_58	84271	DNA polymerase delta interacting protein
M_58	4627	myosin heavy chain 9(MYH9)
M_58	79640	chromosome 22 open reading frame 46(C22orf46)
M_58	414918	DENN domain containing 6B(DENND6B)
M_58	55007	family with sequence similarity 118 member
M_58	25814	ataxin 10(ATXN10)
M_58	4809	SNU13 homolog, small nuclear ribonucleoprotein (U4/U6.U5)(SNU13)
M_58	26286	ADP ribosylation factor GTPase activating protein 3(ARFGAP3)
M_58	25813	SAMM50 sorting and assembly machinery component(SAMM50)
M_58	25809	tubulin tyrosine ligase like 1(TTLL1)
M_58	23151	GRAM domain containing 4(GRAMD4)

M_58	10766	transducer of ERBB2, 2(TOB2)
M_58	23492	chromobox 7(CBX7)
M_58	10762	nucleoporin 50(NUP50)
M_58	91689	single-pass membrane protein with aspartate rich tail 1(SMDT1)
M_58	6319	stearoyl-CoA desaturase(SCD)
M_58	5372	phosphomannomutase 1(PMM1)
M_58	150383	cysteine rich DPF motif domain containing 1(CDPF1)
M_58	9701	protein phosphatase 6 regulatory subunit 2(PPP6R2)
M_58	8863	period circadian clock 3(PER3)
M_58	5435	RNA polymerase II subunit F(POLR2F)
M_58	83933	histone deacetylase 10(HDAC10)
M_58	23774	bromodomain containing 1(BRD1)
M_58	84247	leucine zipper down-regulated in cancer 1
M_58	3949	low density lipoprotein receptor(LDLR)
M_58	80305	TraB domain containing(TRABD)
M_58	6767	suppression of tumorigenicity 13 (colon carcinoma) (Hsp70 interacting protein)(ST13)
M_58	79174	cysteine rich with EGF like domains 2(CRELD2)
M_58	55020	tetratricopeptide repeat domain 38(TTC38)
M_58	64781	ceramide kinase(CERK)
M_58	11135	CDC42 effector protein 1(CDC42EP1)
M_58	57591	megakaryoblastic leukemia (translocation) 1(MKL1)
M_58	9889	zinc finger BED-type containing 4(ZBED4)
M_58	27352	small G protein signaling modulator 3(SGSM3)
M_58	25771	TBC1 domain family member 22A(TBC1D22A)
M_58	27349	malonyl-CoA-acyl carrier protein
M_58	1727	cytochrome b5 reductase 3(CYB5R3)
M_58	1717	7-dehydrocholesterol reductase(DHCR7)
M_58	25828	thioredoxin 2(TXN2)
M_58	55687	tRNA 5-methylaminomethyl-2-thiouridylate methyltransferase(TRMU)

M_58	85378	tubulin gamma complex associated protein
M_58	25777	Sad1 and UNC84 domain containing 2(SUN2)
M_58	25776	chibby family member 1, beta catenin
M_58	6305	SET binding factor 1(SBF1)
M_58	10478	solute carrier family 25 member 17(SLC25A17)
M_58	158	adenylosuccinate lyase(ADSL)
M_58	5465	peroxisome proliferator activated receptor
M_58	6720	sterol regulatory element binding transcription factor 1(SREBF1)
M_58	23112	trinucleotide repeat containing 6B(TNRC6B)
M_58	56993	translocase of outer mitochondrial membrane
M_58	29781	non-SMC condensin II complex subunit
M_58	7008	TEF, PAR bZIP transcription factor(TEF)
M_58	5905	Ran GTPase activating protein 1(RANGAP1)
M_58	145165	suppression of tumorigenicity 13 (colon carcinoma) (Hsp70 interacting protein) pseudogene 4(ST13P4)
M_58	100144603	CHKB antisense RNA 1 (head to head)(CHKB-AS1)
M_58	2547	X-ray repair cross complementing 6(XRCC6)
M_58	23264	zinc finger CCCH-type containing 7B(ZC3H7B)
M_58	171568	RNA polymerase III subunit H(POLR3H)
M_58	3638	insulin induced gene 1(INSIG1)
M_58	410	arylsulfatase A(ARSA)
M_58	1595	cytochrome P450 family 51 subfamily A member 1(CYP51A1)
M_58	85358	SH3 and multiple ankyrin repeat domains
M_58	91582	ribosomal protein S19 binding protein 1(RPS19BP1)
M_58	284942	ribosomal protein L23a pseudogene 82(RPL23AP82)
M_58	23170	tubulin tyrosine ligase like 12(TTLL12)
M_58	94009	serine hydrolase-like (pseudogene)(SERHL)
M_58	11252	protein kinase C and casein kinase substrate in neurons 2(PACSIN2)
M_58	11158	RAB, member of RAS oncogene family-like

M_58	10454	TGF-beta activated kinase 1 (MAP3K7) binding protein 1(TAB1)
M_58	9997	SCO2, cytochrome c oxidase assembly protein(SCO2)
M_58	50	aconitase 2(ACO2)
M_58	83746	L3MBTL2 polycomb repressive complex 1 subunit(L3MBTL2)
M_58	1120	choline kinase beta(CHKB)
M_58	63929	X-prolyl aminopeptidase 3(XPNPEP3)
M_58	283130	solute carrier family 25 member 45(SLC25A45)
M_58	51386	eukaryotic translation initiation factor 3 subunit
M_58	389792	immediate early response 5 like(IER5L)
M_58	5155	platelet derived growth factor subunit B(PDGFB)
M_58	23464	glycine C-acetyltransferase(GCAT)
M_58	83547	Rab interacting lysosomal protein(RILP)
M_58	55615	proline rich 5(PRR5)
M_58	23542	mitogen-activated protein kinase 8 interacting protein 2(MAPK8IP2)
M_58	129138	ankyrin repeat domain 54(ANKRD54)
M_58	79159	nucleolar protein 12(NOL12)
M_58	375759	chromosome 9 open reading frame 50(C9orf50)
M_58	7263	thiosulfate sulfurtransferase(TST)
M_58	150368	family with sequence similarity 109 member
M_58	29780	parvin beta(PARVB)
M_58	8664	eukaryotic translation initiation factor 3 subunit
M_58	23313	KIAA0930(KIAA0930)
M_58	11020	intraflagellar transport 27(IFT27)
M_58	6122	ribosomal protein L3(RPL3)
M_58	4357	mercaptopyruvate sulfurtransferase(MPST)
M_58	4668	alpha-N-acetylgalactosaminidase(NAGA)
M_58	79890	Ras and Rab interactor 3(RIN3)
M_58	5791	protein tyrosine phosphatase, receptor type E(PTPRE)

M_59	26019	UPF2 regulator of nonsense transcripts homolog (yeast)(UPF2)
M_59	9790	BMS1, ribosome biogenesis factor(BMS1)
M_59	8559	pre-mRNA processing factor 18(PRPF18)
M_59	9712	USP6 N-terminal like(USP6NL)
M_59	55149	mitochondrial poly(A) polymerase(MTPAP)
M_59	80013	family with sequence similarity 188 member
M_59	22884	WD repeat domain 37(WDR37)
M_59	22944	Kin17 DNA and RNA binding protein(KIN)
M_59	56243	KIAA1217(KIAA1217)
M_59	3422	isopentenyl-diphosphate delta isomerase 1(IDI1)
M_59	64421	DNA cross-link repair 1C(DCLRE1C)
M_59	3688	integrin subunit beta 1(ITGB1)
M_59	8028	myeloid/lymphoid or mixed-lineage leukemia; translocated to, 10(MLLT10)
M_59	8027	signal transducing adaptor molecule(STAM)
M_59	55526	dehydrogenase E1 and transketolase domain containing 1(DHTKD1)
M_59	2074	ERCC excision repair 6, chromatin remodeling
M_59	23560	GTP binding protein 4(GTPBP4)
M_59	222389	BEN domain containing 7(BEND7)
M_59	54522	ankyrin repeat domain 16(ANKRD16)
M_59	3799	kinesin family member 5B(KIF5B)
M_59	5214	phosphofructokinase, platelet(PFKP)
M_59	84893	F-box protein, helicase, 18(FBXO18)
M_59	84870	R-spondin 3(RSPO3)
M_59	220929	zinc finger protein 438(ZNF438)
M_59	54796	basonuclin 2(BNC2)
M_59	1390	cAMP responsive element modulator(CREM)
M_59	220972	membrane associated ring-CH-type finger
M_59	219771	cyclin Y(CCNY)
M_59	93550	zinc finger AN1-type containing 4(ZFAND4)

M_59	10531	pitrilysin metallopeptidase 1(PITRM1)
M_59	8505	poly(ADP-ribose) glycohydrolase(PARG)
M_59	55176	Sec61 translocon alpha 2 subunit(SEC61A2)
M_59	9317	phosphotriesterase related(PTER)
M_59	54906	family with sequence similarity 208 member
M_59	80314	enhancer of polycomb homolog 1(EPC1)
M_59	10771	zinc finger MYND-type containing 11(ZMYND11)
M_59	56288	par-3 family cell polarity regulator(PARD3)
M_59	221061	family with sequence similarity 171 member
M_59	5599	mitogen-activated protein kinase 8(MAPK8)
M_59	256691	MAM domain containing 2(MAMDC2)
M_59	22931	RAB18, member RAS oncogene family(RAB18)
M_59	253725	family with sequence similarity 21 member
M_59	221079	ADP ribosylation factor like GTPase 5B(ARL5B)
M_59	221078	NOP2/Sun RNA methyltransferase family member 6(NSUN6)
M_59	100169750	psoriasis associated non-protein coding RNA induced by stress(PRINS)
M_59	91452	acyl-CoA binding domain containing 5(ACBD5)
M_59	51322	WW domain containing adaptor with coiled-
M_59	23185	La ribonucleoprotein domain family member
M_59	94134	Rho GTPase activating protein 12(ARHGAP12)
M_59	23414	zinc finger protein, FOG family member 2(ZFPM2)
M_59	10730	YME1 like 1 ATPase(YME1L1)
M_59	10006	abl interactor 1(ABI1)
M_59	1787	tRNA aspartic acid methyltransferase 1(TRDMT1)
M_59	91734	isopentenyl-diphosphate delta isomerase 2(IDI2)
M_59	22852	ankyrin repeat domain 26(ANKRD26)
M_59	57584	Rho GTPase activating protein 21(ARHGAP21)
M_59	118427	olfactomedin 3(OLFM3)
M_59	79896	threonine synthase like 1(THNSL1)
M_59	84991	RNA binding motif protein 17(RBM17)

M_59	6546	solute carrier family 8 member A1(SLC8A1)
M_59	157	G protein-coupled receptor kinase 3(GRK3)
M_59	83860	TATA-box binding protein associated factor 3(TAF3)
M_59	8707	beta-1,3-galactosyltransferase 2(B3GALT2)
M_59	387680	family with sequence similarity 21 member
M_59	387647	patched domain containing 3 pseudogene
M_59	9590	A-kinase anchoring protein 12(AKAP12)
M_59	57569	Rho GTPase activating protein 20(ARHGAP20)
M_59	9723	semaphorin 3E(SEMA3E)
M_59	8453	cullin 2(CUL2)
M_59	55853	IDI2 antisense RNA 1(IDI2-AS1)
M_59	10557	ribonuclease P/MRP subunit p38(RPP38)
M_59	22929	selenophosphate synthetase 1(SEPHS1)
M_59	9397	N-myristoyltransferase 2(NMT2)
M_59	57209	zinc finger protein 248(ZNF248)
M_59	387103	centromere protein W(CENPW)
M_59	50507	NADPH oxidase 4(NOX4)
M_59	5139	phosphodiesterase 3A(PDE3A)
M_59	648	BMI1 proto-oncogene, polycomb ring finger(BMI1)
M_59	11164	nudix hydrolase 5(NUDT5)
M_59	509	ATP synthase, H+ transporting, mitochondrial F1 complex, gamma polypeptide 1(ATP5C1)
M_59	10371	semaphorin 3A(SEMA3A)
M_59	219749	zinc finger protein 25(ZNF25)
M_59	22921	methionine sulfoxide reductase B2(MSRB2)
M_59	9760	thymocyte selection associated high mobility group
M_59	23590	prenyl (decaprenyl) diphosphate synthase, subunit
M_59	79746	enoyl-CoA hydratase domain containing 3(ECHDC3)
M_59	30011	SH3 domain containing kinase binding protein
M_59	10133	optineurin(OPTN)
M_59	51270	transcription factor Dp family member 3(TFDP3)
M_59	6657	SRY-box 2(SOX2)

M_59	79754	ankyrin repeat and SOCS box containing 13(ASB13)
M_59	23283	cleavage stimulation factor subunit 2 tau
M_59	51182	heat shock protein family A (Hsp70) member
M_59	4036	LDL receptor related protein 2(LRP2)
M_59	205	adenylate kinase 4(AK4)
M_59	267004	piggyBac transposable element derived 3(PGBD3)
M_59	347689	SOX2 overlapping transcript(SOX2-OT)
M_59	2824	glycoprotein M6B(GPM6B)
M_59	6251	Ras suppressor protein 1(RSU1)
M_59	7581	zinc finger protein 33A(ZNF33A)
M_59	389941	complement C1q like 3(C1QL3)
M_59	4160	melanocortin 4 receptor(MC4R)
M_59	414149	acyl-CoA binding domain containing 7(ACBD7)
M_59	84930	microtubule associated serine/threonine kinase
M_59	79723	suppressor of variegation 3-9 homolog 2(SUV39H2)
M_59	27143	phosphatase domain containing, paladin 1(PALD1)
M_59	8872	cell division cycle 123(CDC123)
M_59	83844	ubiquitin specific peptidase 26(USP26)
M_59	55747	#N/A
M_59	130589	galactose mutarotase(GALM)
M_59	2665	GDP dissociation inhibitor 2(GDI2)
M_59	23412	COMM domain containing 3(COMMD3)
M_59	9832	janus kinase and microtubule interacting protein
M_59	5588	protein kinase C theta(PRKCQ)
M_59	3769	potassium voltage-gated channel subfamily J member 13(KCNJ13)
M_60	27034	acyl-CoA dehydrogenase family member 8(ACAD8)
M_60	84897	transforming growth factor beta regulator 1(TBRG1)
M_60	23229	Cdc42 guanine nucleotide exchange factor
M_60	83480	pseudouridylate synthase 3(PUS3)
M_60	7753	zinc finger protein 202(ZNF202)
M_60	29068	zinc finger and BTB domain containing 44(ZBTB44)

M_60	23584	V-set and immunoglobulin domain containing
M_60	114609	TIR domain containing adaptor protein(TIRAP)
M_60	334	amyloid beta precursor like protein 2(APLP2)
M_60	57481	KIAA1210(KIAA1210)
M_60	283404	long intergenic non-protein coding RNA
M_60	399979	sorting nexin 19(SNX19)
M_60	3756	potassium voltage-gated channel subfamily H member 1(KCNH1)
M_60	5959	retinol dehydrogenase 5(RDH5)
M_60	4900	neurogranin(NRGN)
M_60	9743	Rho GTPase activating protein 32(ARHGAP32)
M_60	127670	transmembrane epididymal protein 1(TEDDM1)
M_60	196410	methyltransferase like 7B(METTL7B)
M_60	57476	GRAM domain containing 1B(GRAMD1B)
M_60	4798	nuclear factor related to kappaB binding
M_60	22997	immunoglobulin superfamily member 9B(IGSF9B)
M_60	3703	STT3A, catalytic subunit of the oligosaccharyltransferase complex(STT3A)
M_60	112936	VPS26, retromer complex component B(VPS26B)
M_60	84881	RNA pseudouridylate synthase domain containing
M_60	100132994	chromosome X open reading frame 49B(CXorf49B)
M_60	28960	decapping enzyme, scavenger(DCPS)
M_60	3887	keratin 81(KRT81)
M_60	574036	SERTAD4 antisense RNA 1(SERTAD4-AS1)
M_60	3892	keratin 86(KRT86)
M_60	362	aquaporin 5(AQP5)
M_60	219854	transmembrane protein 218(TMEM218)
M_60	359	aquaporin 2(AQP2)
M_60	55800	sodium voltage-gated channel beta subunit 3(SCN3B)
M_60	363	aquaporin 6(AQP6)
M_60	56256	SERTA domain containing 4(SERTAD4)
M_60	54414	sialic acid acetylesterase(SIAE)

M_60	1465	cysteine and glycine rich protein 1(CSRP1)
M_60	283174	MIR4697 host gene(MIR4697HG)
M_60	92249	long intergenic non-protein coding RNA
M_60	6484	ST3 beta-galactoside alpha-2,3-sialyltransferase
M_60	9538	EI24, autophagy associated transmembrane
M_60	55733	hedgehog acyltransferase(HHAT)
M_60	376693	ribosomal protein S10 pseudogene 7(RPS10P7)
M_60	6734	SRP receptor alpha subunit(SRPRA)
M_60	254187	testis specific 10 interacting protein(TSGA10IP)
M_60	81706	protein phosphatase 1 regulatory inhibitor subunit 14C(PPP1R14C)
M_60	79861	tubulin alpha like 3(TUBAL3)
M_60	146434	zinc finger protein 597(ZNF597)
M_60	139285	APC membrane recruitment protein 1(AMER1)
M_60	58499	zinc finger protein 462(ZNF462)
M_60	1636	angiotensin I converting enzyme(ACE)
M_60	220323	out at first homolog(OAF)
M_60	4303	forkhead box O4(FOXO4)
M_61	27292	DIM1 dimethyladenosine transferase 1
M_61	55109	angiogenic factor with G-patch and FHA domains
M_61	3842	transportin 1(TNPO1)
M_61	1022	cyclin dependent kinase 7(CDK7)
M_61	643155	small integral membrane protein 15(SMIM15)
M_61	83594	nudix hydrolase 12(NUDT12)
M_61	84340	G elongation factor mitochondrial 2(GFM2)
M_61	10412	NSA2, ribosome biogenesis homolog(NSA2)
M_61	317671	Rieske Fe-S domain containing(RFESD)
M_61	285600	KIAA0825(KIAA0825)
M_61	153364	metallo-beta-lactamase domain containing
M_61	153339	transmembrane protein 167A(TMEM167A)
M_61	23398	peptidylprolyl isomerase domain and WD repeat containing 1(PPWD1)

M_61	9652	tetratricopeptide repeat domain 37(TTC37)
M_61	57561	arrestin domain containing 3(ARRDC3)
M_61	202243	coiled-coil domain containing 125(CCDC125)
M_61	55781	RIO kinase 2(RIOK2)
M_61	83734	autophagy related 10(ATG10)
M_61	9765	zinc finger FYVE-type containing 16(ZFYVE16)
M_61	2150	F2R like trypsin receptor 1(F2RL1)
M_61	9456	homer scaffolding protein 1(HOMER1)
M_61	3156	3-hydroxy-3-methylglutaryl-CoA reductase(HMGCR)
M_61	10622	RNA polymerase III subunit G(POLR3G)
M_61	375449	microtubule associated serine/threonine kinase family member 4(MAST4)
M_61	54557	small glutamine rich tetratricopeptide repeat containing beta(SGTB)
M_61	90355	chromosome 5 open reading frame 30(C5orf30)
M_61	6607	survival of motor neuron 2, centromeric(SMN2)
M_61	8293	small EDRK-rich factor 1A(SERF1A)
M_61	84240	zinc finger CCHC-type containing 9(ZCCHC9)
M_61	84327	zinc finger BED-type containing 3(ZBED3)
M_61	133746	junction mediating and regulatory protein, p53
M_61	4950	#N/A
M_61	285672	SREK1 interacting protein 1(SREK1IP1)
M_61	8546	adaptor related protein complex 3 beta 1
M_61	4437	mutS homolog 3(MSH3)
M_61	6880	TATA-box binding protein associated factor 9(TAF9)
M_61	64924	solute carrier family 30 member 5(SLC30A5)
M_61	84135	UTP15, small subunit processome component(UTP15)
M_61	83989	family with sequence similarity 172 member
M_61	133383	SET domain containing 9(SETD9)
M_61	728723	ZBED3 antisense RNA 1(ZBED3-AS1)
M_61	6902	tubulin folding cofactor A(TBCA)
M_61	345778	metaxin 3(MTX3)

M_61	83890	spermatogenesis associated 9(SPATA9)
M_61	116068	LysM domain containing 3(LYSMD3)
M_61	54505	DExH-box helicase 29(DHX29)
M_61	10087	collagen type IV alpha 3 binding protein(COL4A3BP)
M_61	23107	mitochondrial ribosomal protein S27(MRPS27)
M_61	1719	dihydrofolate reductase(DHFR)
M_61	5921	RAS p21 protein activator 1(RASA1)
M_61	373	tripartite motif containing 23(TRIM23)
M_61	51194	importin 11(IPO11)
M_61	57763	ankyrin repeat family A member 2(ANKRA2)
M_61	55914	erbb2 interacting protein(ERBIN)
M_61	79810	pentatricopeptide repeat domain 2(PTCD2)
M_61	64283	Rho guanine nucleotide exchange factor
M_61	84250	SMC5-SMC6 complex localization factor 1(SLF1)
M_61	55814	B double prime 1, subunit of RNA polymerase III transcription initiation factor IIIB(BDP1)
M_61	91942	NADH:ubiquinone oxidoreductase complex assembly factor 2(NDUFAF2)
M_61	1105	chromodomain helicase DNA binding protein
M_61	51426	DNA polymerase kappa(POLK)
M_61	4214	mitogen-activated protein kinase kinase kinase
M_61	57486	neurolysin(NLN)
M_61	80006	trafficking protein particle complex 13(TRAPPC13)
M_61	166968	MIER family member 3(MIER3)
M_61	65056	GC-rich promoter binding protein 1(GPBP1)
M_61	115548	FCH domain only 2(FCHO2)
M_61	153396	transmembrane protein 161B(TMEM161B)
M_61	79993	ELOVL fatty acid elongase 7(ELOVL7)
M_61	167153	poly(A) RNA polymerase D4, non-canonical(PAPD4)
M_61	140890	splicing regulatory glutamic acid and lysine rich protein 1(SREK1)
M_61	134359	POC5 centriolar protein(POC5)

M_61	7518	X-ray repair cross complementing 4(XRCC4)
M_61	1070	centrin 3(CETN3)
M_61	23262	diphosphoinositol pentakisphosphate kinase
M_61	23517	Ski2 like RNA helicase 2(SKIV2L2)
M_61	92259	mitochondrial ribosomal protein S36(MRPS36)
M_61	55255	WD repeat domain 41(WDR41)
M_61	689	basic transcription factor 3(BTF3)
M_61	153642	arylsulfatase family member K(ARSK)
M_61	728340	GTF2H2 family member C(GTF2H2C)
M_61	153562	MARVEL domain containing 2(MARVELD2)
M_61	54826	gypsy retrotransposon integrase 1(GIN1)
M_61	1161	ERCC excision repair 8, CSA ubiquitin ligase complex subunit(ERCC8)
M_61	3796	kinesin family member 2A(KIF2A)
M_61	153129	solute carrier family 38 member 9(SLC38A9)
M_61	64946	centromere protein H(CENPH)
M_61	902	cyclin H(CCNH)
M_61	10283	CWC27 spliceosome associated protein
M_61	23635	single stranded DNA binding protein 2(SSBP2)
M_61	1350	cytochrome c oxidase subunit 7C(COX7C)
M_61	64087	methylcrotonoyl-CoA carboxylase 2(MCCC2)
M_61	5884	RAD17 checkpoint clamp loader component(RAD17)
M_61	9522	secretory carrier membrane protein 1(SCAMP1)
M_61	57688	zinc finger SWIM-type containing 6(ZSWIM6)
M_61	8905	adaptor related protein complex 1 sigma 2
M_61	167555	family with sequence similarity 151 member
M_61	3087	hematopoietically expressed homeobox(HHEX)
M_61	200132	Tctex1 domain containing 1(TCTEX1D1)
M_61	4724	NADH:ubiquinone oxidoreductase subunit
M_61	653653	adaptor related protein complex 1 sigma 2 subunit pseudogene(LOC653653)
M_61	345757	family with sequence similarity 174 member

M_61	2966	general transcription factor IIH subunit 2(GTF2H2)
M_61	6606	survival of motor neuron 1, telomeric(SMN1)
M_61	647859	occludin pseudogene(LOC647859)
M_62	163059	zinc finger protein 433(ZNF433)
M_62	163051	zinc finger protein 709(ZNF709)
M_62	163050	zinc finger protein 564(ZNF564)
M_62	163049	zinc finger protein 791(ZNF791)
M_62	342979	paralemmin 3(PALM3)
M_62	126070	zinc finger protein 440(ZNF440)
M_62	374882	transmembrane protein 205(TMEM205)
M_62	84705	GTP binding protein 3 (mitochondrial)(GTPBP3)
M_62	8666	eukaryotic translation initiation factor 3 subunit
M_62	84337	elongation factor 1 homolog(ELOF1)
M_62	54862	coiled-coil and C2 domain containing 1A(CC2D1A)
M_62	55723	anti-silencing function 1B histone chaperone(ASF1B)
M_62	90480	GADD45G interacting protein 1(GADD45GIP1)
M_62	284391	zinc finger protein 844(ZNF844)
M_62	112812	ferredoxin 1 like(FDX1L)
M_62	54811	zinc finger protein 562(ZNF562)
M_62	126068	zinc finger protein 441(ZNF441)
M_62	58513	epidermal growth factor receptor pathway substrate 15 like 1(EPS15L1)
M_62	51295	ECSIT signalling integrator(ECSIT)
M_62	28974	chromosome 19 open reading frame 53(C19orf53)
M_62	84971	autophagy related 4D cysteine peptidase(ATG4D)
M_62	11018	transmembrane p24 trafficking protein 1(TMED1)
M_62	4713	NADH:ubiquinone oxidoreductase subunit
M_62	1725	deoxyhypusine synthase(DHPS)
M_62	773	calcium voltage-gated channel subunit alpha1
M_62	10498	coactivator associated arginine methyltransferase
M_62	1032	cyclin dependent kinase inhibitor 2D(CDKN2D)
M_62	5585	protein kinase N1(PKN1)

M_62	10755	GIPC PDZ domain containing family member
M_62	5589	protein kinase C substrate 80K-H(PRKCSH)
M_62	125950	ribonucleoprotein, PTB binding 1(RAVER1)
M_62	81576	coiled-coil domain containing 130(CCDC130)
M_62	113230	MISP family member 3(MISP3)
M_62	3609	interleukin enhancer binding factor 3(ILF3)
M_62	59286	ubiquitin like 5(UBL5)
M_62	10270	A-kinase anchoring protein 8(AKAP8)
M_62	8677	syntaxin 10(STX10)
M_62	56342	peter pan homolog (Drosophila)(PPAN)
M_62	10212	DExD-box helicase 39A(DDX39A)
M_62	54850	F-box and leucine rich repeat protein 12(FBXL12)
M_62	100125288	zinc finger, GATA-like protein 1(ZGLP1)
M_62	51398	WD repeat domain 83 opposite strand(WDR83OS)
M_62	7730	zinc finger protein 177(ZNF177)
M_62	79002	chromosome 19 open reading frame 43(C19orf43)
M_62	84167	chromosome 19 open reading frame 44(C19orf44)
M_62	57474	zinc finger protein 490(ZNF490)
M_62	126074	SWIM-type zinc finger 7 associated protein
M_62	126075	coiled-coil domain containing 159(CCDC159)
M_62	79173	chromosome 19 open reading frame 57(C19orf57)
M_62	9545	RAB3D, member RAS oncogene family(RAB3D)
M_62	3385	intercellular adhesion molecule 3(ICAM3)
M_62	9294	sphingosine-1-phosphate receptor 2(S1PR2)
M_62	84245	methylthioribose-1-phosphate isomerase 1(MRI1)
M_62	25959	KN motif and ankyrin repeat domains 2(KANK2)
M_62	10781	zinc finger protein 266(ZNF266)
M_62	148156	zinc finger protein 558(ZNF558)
M_62	55552	zinc finger protein 823(ZNF823)
M_62	256126	synaptonemal complex central element protein
M_62	11140	cell division cycle 37(CDC37)
M_62	23476	bromodomain containing 4(BRD4)

M_62	84292	WD repeat domain 83(WDR83)
M_62	400120	serine rich and transmembrane domain containing
M_62	7297	tyrosine kinase 2(TYK2)
M_62	23309	SIN3 transcription regulator family member B(SIN3B)
M_62	115950	zinc finger protein 653(ZNF653)
M_62	57153	solute carrier family 44 member 2(SLC44A2)
M_62	90378	sterile alpha motif domain containing 1(SAMD1)
M_62	51073	mitochondrial ribosomal protein L4(MRPL4)
M_62	9817	kelch like ECH associated protein 1(KEAP1)
M_62	10535	ribonuclease H2 subunit A(RNASEH2A)
M_62	26993	A-kinase anchoring protein 8 like(AKAP8L)
M_62	90379	DDB1 and CUL4 associated factor 15(DCAF15)
M_62	93134	zinc finger protein 561(ZNF561)
M_62	81890	queuine tRNA-ribosyltransferase catalytic subunit
M_62	7001	peroxiredoxin 2(PRDX2)
M_62	10994	ilvB acetolactate synthase like(ILVBL)
M_62	55621	tRNA methyltransferase 1(TRMT1)
M_62	284390	zinc finger protein 763(ZNF763)
M_62	162993	zinc finger protein 846(ZNF846)
M_62	10523	calcium homeostasis endoplasmic reticulum
M_62	78992	Yip1 domain family member 2(YIPF2)
M_62	5032	purinergic receptor P2Y11(P2RY11)
M_62	199692	zinc finger protein 627(ZNF627)
M_62	439	arsA arsenite transporter, ATP-binding, homolog 1 (bacterial)(ASNA1)
M_62	9441	mediator complex subunit 26(MED26)
M_62	1785	dynamin 2(DNM2)
M_62	1786	DNA methyltransferase 1(DNMT1)
M_62	8900	cyclin A1(CCNA1)
M_62	374879	zinc finger protein 699(ZNF699)
M_62	7675	zinc finger protein 121(ZNF121)
M_62	51710	zinc finger protein 44(ZNF44)

M_62	112939	nucleus accumbens associated 1(NACC1)
M_62	84527	zinc finger protein 559(ZNF559)
M_62	3337	DnaJ heat shock protein family (Hsp40) member B1(DNAJB1)
M_62	5566	protein kinase cAMP-activated catalytic subunit alpha(PRKACA)
M_62	79088	zinc finger protein 426(ZNF426)
M_62	58525	widely interspaced zinc finger motifs(WIZ)
M_62	79939	solute carrier family 35 member E1(SLC35E1)
M_62	90580	translocase of inner mitochondrial membrane
M_62	84449	zinc finger protein 333(ZNF333)
M_62	90594	zinc finger protein 439(ZNF439)
M_62	90592	zinc finger protein 700(ZNF700)
M_62	90589	zinc finger protein 625(ZNF625)
M_62	6597	SWI/SNF related, matrix associated, actin dependent regulator of chromatin, subfamily a, member
M_62	30000	transportin 2(TNPO2)
M_62	7568	zinc finger protein 20(ZNF20)
M_62	57572	dedicator of cytokinesis 6(DOCK6)
M_62	5300	peptidylprolyl cis/trans isomerase, NIMA-interacting
M_62	64748	phospholipid phosphatase related 2(PLPPR2)
M_62	7695	zinc finger protein 136(ZNF136)
M_62	84261	F-box and WD repeat domain containing 9(FBXW9)
M_62	4650	myosin IXB(MYO9B)
M_62	65095	KRI1 homolog(KRI1)
M_62	147837	zinc finger protein 563(ZNF563)
M_62	8907	adaptor related protein complex 1 mu 1
M_62	5886	RAD23 homolog A, nucleotide excision repair protein(RAD23A)
M_62	29911	hook microtubule tethering protein 2(HOOK2)
M_62	57693	zinc finger protein 317(ZNF317)
M_62	9524	trans-2,3-enoyl-CoA reductase(TECR)

M_62	5989	regulatory factor X1(RFX1)
M_62	2639	glutaryl-CoA dehydrogenase(GCDH)
M_62	2193	phenylalanyl-tRNA synthetase alpha subunit(FARSA)
M_62	126069	zinc finger protein 491(ZNF491)
M_62	23111	spastic paraplegia 20 (Troyer syndrome)(SPG20)
M_62	9466	interleukin 27 receptor subunit alpha(IL27RA)
M_62	728591	coiled-coil domain containing 169(CCDC169)
M_62	388507	zinc finger family member 788(ZNF788)
M_62	729747	zinc finger protein 878(ZNF878)
M_62	147841	SPC24, NDC80 kinetochore complex
M_62	4093	SMAD family member 9(SMAD9)
M_62	57719	anoctamin 8(ANO8)
M_62	54937	spermatogenesis and oogenesis specific basic helix-loop-helix 2(SOHLH2)
M_62	79973	zinc finger protein 442(ZNF442)
M_62	1777	deoxyribonuclease 2, lysosomal(DNASE2)
M_62	65249	zinc finger SWIM-type containing 4(ZSWIM4)
M_62	376497	solute carrier family 27 member 1(SLC27A1)
M_62	401898	zinc finger protein 833, pseudogene(ZNF833P)
M_62	117579	relaxin 3(RLN3)
M_62	389257	leucine rich repeat containing 14B(LRRC14B)
M_62	4125	mannosidase alpha class 2B member 1(MAN2B1)
M_63	253260	RPTOR independent companion of MTOR complex 2(RICTOR)
M_63	1611	death associated protein(DAP)
M_63	29102	drosha ribonuclease III(DROSHA)
M_63	54545	myotubularin related protein 12(MTMR12)
M_63	55100	WD repeat domain 70(WDR70)
M_63	25845	uncharacterized LOC25845(PP7080)
M_63	134218	DnaJ heat shock protein family (Hsp40) member C21(DNAJC21)
M_63	23600	alpha-methylacyl-CoA racemase(AMACR)

M_63	23379	interactor of little elongation complex ELL subunit
M_63	10299	membrane associated ring-CH-type finger
M_63	728411	glucuronidase, beta pseudogene 1(GUSBP1)
M_63	55722	centrosomal protein 72(CEP72)
M_63	11336	exocyst complex component 3(EXOC3)
M_63	133957	coiled-coil domain containing 127(CCDC127)
M_63	54888	NOP2/Sun RNA methyltransferase family member 2(NSUN2)
M_63	134145	family with sequence similarity 173 member
M_63	7690	zinc finger protein 131(ZNF131)
M_63	6389	succinate dehydrogenase complex flavoprotein subunit A(SDHA)
M_63	64979	mitochondrial ribosomal protein L36(MRPL36)
M_63	10723	solute carrier family 12 member 7(SLC12A7)
M_63	6897	threonyl-tRNA synthetase(TARS)
M_63	11044	poly(A) RNA polymerase D7, non-canonical(PAPD7)
M_63	10884	mitochondrial ribosomal protein S30(MRPS30)
M_63	25836	NIPBL, cohesin loading factor(NIPBL)
M_63	10923	SUB1 homolog, transcriptional regulator(SUB1)
M_63	728613	programmed cell death 6 pseudogene(LOC728613)
M_63	7204	trio Rho guanine nucleotide exchange factor(TRIO)
M_63	55322	chromosome 5 open reading frame 22(C5orf22)
M_63	116349	EXOC3 antisense RNA 1(EXOC3-AS1)
M_63	90268	OTU deubiquitinase with linear linkage
M_63	5810	RAD1 checkpoint DNA exonuclease(RAD1)
M_63	728609	succinate dehydrogenase complex flavoprotein subunit A pseudogene 3(SDHAP3)
M_63	51663	zinc finger RNA binding protein(ZFR)
M_63	5562	protein kinase AMP-activated catalytic subunit alpha 1(PRKAA1)
M_63	285636	chromosome 5 open reading frame 51(C5orf51)
M_63	92255	LMBR1 domain containing 2(LMBRD2)

M_63	22948	chaperonin containing TCP1 subunit 5(CCT5)
M_63	79888	lysophosphatidylcholine acyltransferase 1(LPCAT1)
M_63	65980	bromodomain containing 9(BRD9)
M_63	6715	steroid 5 alpha-reductase 1(SRD5A1)
M_63	90441	zinc finger protein 622(ZNF622)
M_63	84246	mediator complex subunit 10(MED10)
M_63	81037	CLPTM1 like(CLPTM1L)
M_63	10016	programmed cell death 6(PDCD6)
M_63	10605	poly(A) binding protein interacting protein 1(PAIP1)
M_63	79072	FAST kinase domains 3(FASTKD3)
M_63	65250	chromosome 5 open reading frame 42(C5orf42)
M_63	4552	5-methyltetrahydrofolate-homocysteine methyltransferase reductase(MTRR)
M_63	4726	NADH:ubiquinone oxidoreductase subunit
M_63	55299	BRX1, biogenesis of ribosomes(BRIX1)
M_63	64083	golgi phosphoprotein 3(GOLPH3)
M_63	9319	thyroid hormone receptor interactor 13(TRIP13)
M_63	439931	THAP7 antisense RNA 1(THAP7-AS1)
M_63	375444	chromosome 5 open reading frame 34(C5orf34)
M_63	56172	ANKH inorganic pyrophosphate transport
M_63	10293	TRAF interacting protein(TRAIP)
M_63	25886	POC1 centriolar protein A(POC1A)
M_63	57116	zinc finger protein 695(ZNF695)
M_63	23530	nicotinamide nucleotide transhydrogenase(NNT)
M_63	3157	3-hydroxy-3-methylglutaryl-CoA synthase
M_63	2177	Fanconi anemia complementation group
M_63	84722	proline and serine rich coiled-coil 1(PSRC1)
M_64	55331	alkaline ceramidase 3(ACER3)
M_64	84649	diacylglycerol O-acyltransferase 2(DGAT2)
M_64	387787	lipoyl(octanoyl) transferase 2 (putative)(LIPT2)
M_64	55199	family with sequence similarity 86 member
M_64	29991	odorant binding protein 2A(OBP2A)

M_64	29989	odorant binding protein 2B(OBP2B)
M_64	9789	signal peptidase complex subunit 2(SPCS2)
M_64	4926	nuclear mitotic apparatus protein 1(NUMA1)
M_64	100131726	FAM83A antisense RNA 1(FAM83A-AS1)
M_64	92105	integrator complex subunit 4(INTS4)
M_64	7351	uncoupling protein 2(UCP2)
M_64	51773	remodeling and spacing factor 1(RSF1)
M_64	80227	proteasomal ATPase associated factor 1(PAAF1)
M_64	8500	PTPRF interacting protein alpha 1(PPFIA1)
M_64	4647	myosin VIIA(MYO7A)
M_64	4975	olfactory marker protein(OMP)
M_64	79053	ALG8, alpha-1,3-glucosyltransferase(ALG8)
M_64	57558	ubiquitin specific peptidase 35(USP35)
M_64	4718	NADH:ubiquinone oxidoreductase subunit
M_64	9846	GRB2 associated binding protein 2(GAB2)
M_64	84084	RAB6C, member RAS oncogene family(RAB6C)
M_64	28971	adipogenesis associated Mth938 domain
M_64	7405	UV radiation resistance associated(UVRAG)
M_64	79731	asparaginyl-tRNA synthetase 2, mitochondrial (putative)(NARS2)
M_64	5058	p21 (RAC1) activated kinase 1(PAK1)
M_64	220032	glycerophosphodiester phosphodiesterase domain containing 4(GDPD4)
M_64	2017	cortactin(CTTN)
M_64	56946	EMSY, BRCA2 interacting transcriptional
M_64	81570	ClpB homolog, mitochondrial AAA ATPase chaperonin(CLPB)
M_64	7069	thyroid hormone responsive(THRSP)
M_64	254295	phytanoyl-CoA dioxygenase domain containing
M_64	653566	signal peptidase complex subunit 2 homolog (S. cerevisiae) pseudogene 4(SPCS2P4)
M_64	10825	neuraminidase 3(NEU3)

M_64	26005	C2 calcium dependent domain containing 3(C2CD3)
M_64	84985	family with sequence similarity 83 member
M_64	645332	family with sequence similarity 86, member A pseudogene(FAM86C2P)
M_64	3636	inositol polyphosphate phosphatase like 1(INPPL1)
M_64	282679	aquaporin 11(AQP11)
M_64	644619	integrator complex subunit 4 pseudogene 2(INTS4P2)
M_64	116985	ArfGAP with RhoGAP domain, ankyrin repeat and PH domain 1(ARAP1)
M_64	3623	inhibin alpha subunit(INHA)
M_64	51287	cytochrome c oxidase assembly factor 4
M_64	65987	potassium channel tetramerization domain containing 14(KCTD14)
M_64	25987	tsukushi, small leucine rich proteoglycan(TSKU)
M_64	9873	FCH and double SH3 domains 2(FCHSD2)
M_64	10714	DNA polymerase delta 3, accessory subunit(POLD3)
M_64	51642	mitochondrial ribosomal protein L48(MRPL48)
M_64	143570	X-ray radiation resistance associated 1(XRRA1)
M_64	9828	Rho guanine nucleotide exchange factor
M_64	1207	chloride nucleotide-sensitive channel 1A(CLNS1A)
M_64	283219	potassium channel tetramerization domain containing 21(KCTD21)
M_64	23201	family with sequence similarity 168 member
M_64	5612	THAP domain containing 12(THAP12)
M_64	6508	solute carrier family 4 member 3(SLC4A3)
M_64	51400	protein phosphatase methylesterase 1(PPME1)
M_64	8772	Fas associated via death domain(FADD)
M_64	25906	anaphase promoting complex subunit 15(ANAPC15)
M_64	55298	ring finger protein 121(RNF121)
M_64	726	calpain 5(CAPN5)
M_64	254225	ring finger protein 169(RNF169)
M_64	23363	obscurin like 1(OBSL1)

M_64	5870	RAB6A, member RAS oncogene family(RAB6A)
M_64	221416	chromosome 6 open reading frame 223(C6orf223)
M_64	220074	leucine rich transmembrane and O-methyltransferase domain containing(LRTOMT)
M_64	7352	uncoupling protein 3(UCP3)
M_64	200942	kelch domain containing 8B(KLHDC8B)
M_64	90835	coiled-coil domain containing 189(CCDC189)
M_64	55291	protein phosphatase 6 regulatory subunit 3(PPP6R3)
M_64	3846	keratin associated protein 5-9(KRTAP5-9)
M_64	3508	immunoglobulin mu binding protein 2(IGHMBP2)
M_64	51111	lysine methyltransferase 5B(KMT5B)
M_64	643376	BTB domain containing 18(BTBD18)
M_64	55191	NAD synthetase 1(NADSYN1)
M_64	3933	lipocalin 1(LCN1)
M_64	10008	potassium voltage-gated channel subfamily E regulatory subunit 3(KCNE3)
M_64	1119	choline kinase alpha(CHKA)
M_64	4826	neuronatin(NNAT)
M_64	219931	two pore segment channel 2(TPCN2)
M_64	53841	cadherin related family member 5(CDHR5)
M_64	220064	oral cancer overexpressed 1(ORAOV1)
M_64	2282	FK506 binding protein 1A pseudogene 1(FKBP1AP1)
M_64	57830	keratin associated protein 5-8(KRTAP5-8)
M_64	387273	keratin associated protein 5-10(KRTAP5-10)
M_64	140771	Smith-Magenis syndrome chromosome region, candidate 5 (non-protein coding)(SMCR5)
M_64	440050	keratin associated protein 5-7(KRTAP5-7)
M_64	148696	uncharacterized LOC148696(LOC148696)
M_65	6294	scaffold attachment factor B(SAFB)
M_65	93474	zinc finger protein 670(ZNF670)
M_65	55758	REST corepressor 3(RCOR3)
M_65	267002	piggyBac transposable element derived 2(PGBD2)

M_65	84976	dispatched RND transporter family member 1(DISP1)
M_65	55105	G-patch domain containing 2(GPATCH2)
M_65	116228	COX20, cytochrome c oxidase assembly
M_65	200205	IBA57 homolog, iron-sulfur cluster assembly(IBA57)
M_65	26750	ribosomal protein S6 kinase C1(RPS6KC1)
M_65	51018	ribosomal RNA processing 15 homolog(RRP15)
M_65	142	poly(ADP-ribose) polymerase 1(PARP1)
M_65	7678	zinc finger protein 124(ZNF124)
M_65	23029	RNA binding motif protein 34(RBM34)
M_65	84838	zinc finger protein 496(ZNF496)
M_65	79169	chromosome 1 open reading frame 35(C1orf35)
M_65	9015	TATA-box binding protein associated factor, RNA polymerase I subunit A(TAF1A)
M_65	79573	tetratricopeptide repeat domain 13(TTC13)
M_65	10638	S-phase response (cyclin related)(SPHAR)
M_65	25909	AT-hook containing transcription factor 1(AHCTF1)
M_65	65094	jumonji domain containing 4(JMJD4)
M_65	2590	polypeptide N-acetylgalactosaminyltransferase
M_65	6894	TAR (HIV-1) RNA binding protein 1(TARBP1)
M_65	64216	transcription factor B2, mitochondrial(TFB2M)
M_65	7257	translin associated factor X(TSNAX)
M_65	84284	nucleoside-triphosphatase, cancer-related(NTPCR)
M_65	9453	geranylgeranyl diphosphate synthase 1(GGPS1)
M_65	4670	heterogeneous nuclear ribonucleoprotein
M_65	5362	plexin A2(PLXNA2)
M_65	84886	chromosome 1 open reading frame 198(C1orf198)
M_65	148362	BRO1 domain and CAAX motif containing(BROX)
M_65	126731	centriole, cilia and spindle associated protein(CCSAP)
M_65	9804	translocase of outer mitochondrial membrane
M_65	2271	fumarate hydratase(FH)
M_65	127602	dynein axonemal heavy chain 14(DNAH14)
M_65	22796	component of oligomeric golgi complex 2(COG2)

M_65	286826	lin-9 DREAM MuvB core complex component(LIN9)
M_65	3192	heterogeneous nuclear ribonucleoprotein
M_65	51127	tripartite motif containing 17(TRIM17)
M_65	51029	desumoylating isopeptidase 2(DESI2)
M_65	83932	SprT-like N-terminal domain(SPRTN)
M_65	8476	CDC42 binding protein kinase alpha(CDC42BPA)
M_65	51742	AT-rich interaction domain 4B(ARID4B)
M_65	128061	chromosome 1 open reading frame 131(C1orf131)
M_65	7159	tumor protein p53 binding protein 2(TP53BP2)
M_65	116841	synaptosome associated protein 47(SNAP47)
M_65	80851	SH3 binding domain protein 5 like(SH3BP5L)
M_65	23456	ATP binding cassette subfamily B member
M_65	5867	RAB4A, member RAS oncogene family(RAB4A)
M_65	4931	nuclear VCP-like(NVL)
M_65	55540	interleukin 17 receptor B(IL17RB)
M_65	3020	H3 histone family member 3A(H3F3A)
M_65	81559	tripartite motif containing 11(TRIM11)
M_65	5664	presenilin 2(PSEN2)
M_65	54583	egl-9 family hypoxia inducible factor 1(EGLN1)
M_65	149603	ring finger protein 187(RNF187)
M_65	55127	HEAT repeat containing 1(HEATR1)
M_65	163882	consortin, connexin sorting protein(CNST)
M_65	163859	SDE2 telomere maintenance homolog(SDE2)
M_65	375	ADP ribosylation factor 1(ARF1)
M_65	159	adenylosuccinate synthase(ADSS)
M_65	9667	scaffold attachment factor B2(SAFB2)
M_65	51133	potassium channel tetramerization domain containing 3(KCTD3)
M_65	6726	signal recognition particle 9(SRP9)
M_65	9816	URB2 ribosome biogenesis 2 homolog (S.
M_65	79862	zinc finger protein 669(ZNF669)
M_65	64801	ARV1 homolog, fatty acid homeostasis

M_65	55746	nucleoporin 133(NUP133)
M_65	56997	coenzyme Q8A(COQ8A)
M_65	55740	enabled homolog (Drosophila)(ENAH)
M_65	8443	glyceronephosphate O-acyltransferase(GNPAT)
M_65	84451	mixed lineage kinase 4(MLK4)
M_65	55699	isoleucyl-tRNA synthetase 2, mitochondrial(IARS2)
M_65	339500	zinc finger protein 678(ZNF678)
M_65	2058	glutamyl-prolyl-tRNA synthetase(EPRS)
M_65	149371	exocyst complex component 8(EXOC8)
M_65	25782	RAB3 GTPase activating non-catalytic protein subunit 2(RAB3GAP2)
M_65	2052	epoxide hydrolase 1(EPHX1)
M_65	359948	interferon regulatory factor 2 binding protein
M_65	90806	angel homolog 2(ANGEL2)
M_65	148789	beta-1,3-N-acetylgalactosaminyltransferase
M_65	29920	pyrroline-5-carboxylate reductase family member
M_65	3930	lamin B receptor(LBR)
M_65	23219	F-box protein 28(FBXO28)
M_65	375056	MIA family member 3, ER export factor(MIA3)
M_65	80003	pecanex homolog 2 (Drosophila)(PCNX2)
M_65	79894	zinc finger protein 672(ZNF672)
M_65	27042	digestive organ expansion factor homolog
M_65	27097	TATA-box binding protein associated factor 5
M_65	64746	acyl-CoA binding domain containing 3(ACBD3)
M_65	28982	feline leukemia virus subgroup C cellular receptor
M_65	80232	WD repeat domain 26(WDR26)
M_65	6905	tubulin folding cofactor E(TBCE)
M_65	4548	5-methyltetrahydrofolate-homocysteine methyltransferase(MTR)
M_65	10806	serologically defined colon cancer antigen
M_65	55349	choline dehydrogenase(CHDH)
M_65	23030	lysine demethylase 4B(KDM4B)

M_65	1122	CHM like, Rab escort protein 2(CHML)
M_65	339210	chromosome 17 open reading frame 67(C17orf67)
M_65	5802	protein tyrosine phosphatase, receptor type S(PTPRS)
M_65	56950	SET and MYND domain containing 2(SMYD2)
M_65	51097	saccharopine dehydrogenase (putative)(SCCPDH)
M_65	64754	SET and MYND domain containing 3(SMYD3)
M_65	152992	tRNA methyltransferase 44 homolog (S. cerevisiae)(TRMT44)
M_65	3707	inositol-trisphosphate 3-kinase B(ITPKB)
M_65	9926	lysophosphatidylglycerol acyltransferase 1(LPGAT1)
M_65	6791	aurora kinase A pseudogene 1(AURKAPS1)
M_65	9725	transmembrane protein 63A(TMEM63A)
M_65	163486	DENN domain containing 1B(DENND1B)
M_65	100130093	#N/A
M_65	84288	EF-hand calcium binding domain 2(EFCAB2)
M_65	8526	diacylglycerol kinase epsilon(DGKE)
M_65	6101	retinitis pigmentosa 1 (autosomal dominant)(RP1)
M_66	84294	UTP23, small subunit processome component(UTP23)
M_66	10221	tribbles pseudokinase 1(TRIB1)
M_66	7534	tyrosine 3-monooxygenase/tryptophan 5-monooxygenase activation protein zeta(YWHAZ)
M_66	23471	translocation associated membrane protein 1(TRAM1)
M_66	3646	eukaryotic translation initiation factor 3 subunit
M_66	9401	RecQ like helicase 4(RECQL4)
M_66	83940	TatD DNase domain containing 1(TATDN1)
M_66	8667	eukaryotic translation initiation factor 3 subunit
M_66	9791	phosphatidylserine synthase 1(PTDSS1)
M_66	79752	zinc finger AN1-type containing 1(ZFAND1)
M_66	251	alkaline phosphatase, placental like 2(ALPPL2)
M_66	250	alkaline phosphatase, placental(ALPP)
M_66	8323	frizzled class receptor 6(FZD6)
M_66	7163	tumor protein D52(TPD52)

M_66	92140	metadherin(MTDH)
M_66	51633	OTU domain containing 6B(OTUD6B)
M_66	4715	NADH:ubiquinone oxidoreductase subunit
M_66	9650	mitochondrial fission regulator 1(MTFR1)
M_66	81034	solute carrier family 25 member 32(SLC25A32)
M_66	51110	lactamase beta 2(LACTB2)
M_66	50807	ArfGAP with SH3 domain, ankyrin repeat and PH domain 1(ASAP1)
M_66	51101	zinc finger C2HC-type containing 1A(ZC2HC1A)
M_66	10499	nuclear receptor coactivator 2(NCOA2)
M_66	51105	PHD finger protein 20-like 1(PHF20L1)
M_66	25897	ring finger protein 19A, RBR E3 ubiquitin protein ligase(RNF19A)
M_66	84955	NudC domain containing 1(NUDCD1)
M_66	28957	mitochondrial ribosomal protein S28(MRPS28)
M_66	55093	WDYHV motif containing 1(WDYHV1)
M_66	8895	copine 3(CPNE3)
M_66	54704	pyruvate dehyrogenase phosphatase catalytic subunit
M_66	3612	inositol monophosphatase 1(IMPA1)
M_66	79666	pleckstrin homology and FYVE domain containing 2(PLEKHF2)
M_66	65986	zinc finger and BTB domain containing 10(ZBTB10)
M_66	25788	RAD54 homolog B (S. cerevisiae)(RAD54B)
M_66	90390	mediator complex subunit 30(MED30)
M_66	5440	RNA polymerase II subunit K(POLR2K)
M_66	51123	zinc finger protein 706(ZNF706)
M_66	6713	squalene epoxidase(SQLE)
M_66	157378	transmembrane protein 65(TMEM65)
M_66	27085	MDM2 binding protein(MTBP)
M_66	54108	chromatin accessibility complex 1(CHRAC1)
M_66	51115	regulator of microtubule dynamics 1(RMDN1)
M_66	1666	2,4-dienoyl-CoA reductase 1, mitochondrial(DECR1)

M_66	7013	telomeric repeat binding factor 1(TERF1)
M_66	93594	TBC1 domain family member 31(TBC1D31)
M_66	92421	charged multivesicular body protein 4C(CHMP4C)
M_66	80124	valosin containing protein interacting protein
M_66	25962	KIAA1429(KIAA1429)
M_66	55284	ubiquitin conjugating enzyme E2 W
M_66	9166	estrogen receptor binding site associated, antigen,
M_66	5862	RAB2A, member RAS oncogene family(RAB2A)
M_66	528	ATPase H+ transporting V1 subunit C1(ATP6V1C1)
M_66	55529	transmembrane protein 55A(TMEM55A)
M_66	11236	ring finger protein 139(RNF139)
M_66	8767	receptor interacting serine/threonine kinase 2(RIPK2)
M_66	5828	peroxisomal biogenesis factor 2(PEX2)
M_66	51582	antizyme inhibitor 1(AZIN1)
M_66	1875	E2F transcription factor 5(E2F5)
M_66	253943	YTH N6-methyladenosine RNA binding protein
M_66	51571	family with sequence similarity 49 member
M_66	10247	reactive intermediate imine deaminase A
M_66	85444	leucine rich repeat and coiled-coil centrosomal protein 1(LRRCC1)
M_66	157638	family with sequence similarity 84 member
M_66	27161	argonaute 2, RISC catalytic component(AGO2)
M_66	29028	ATPase family, AAA domain containing 2(ATAD2)
M_66	10987	COP9 signalosome subunit 5(COPS5)
M_66	6921	transcription elongation factor B subunit 1(TCEB1)
M_66	28998	mitochondrial ribosomal protein L13(MRPL13)
M_66	157657	chromosome 8 open reading frame 37(C8orf37)
M_66	26986	poly(A) binding protein cytoplasmic 1(PABPC1)
M_66	79815	NIPA like domain containing 2(NIPAL2)
M_66	157680	vacuolar protein sorting 13 homolog B(VPS13B)
M_66	79139	derlin 1(DERL1)
M_66	5042	poly(A) binding protein cytoplasmic 3(PABPC3)

M_66	10565	ADP ribosylation factor guanine nucleotide exchange factor 1(ARFGEF1)
M_66	401474	sterile alpha motif domain containing 12(SAMD12)
M_66	51366	ubiquitin protein ligase E3 component n-recognin
M_66	6873	TATA-box binding protein associated factor 2(TAF2)
M_66	157769	family with sequence similarity 91 member
M_66	9694	ER membrane protein complex subunit 2(EMC2)
M_66	137209	zinc finger protein 572(ZNF572)
M_66	11244	zinc fingers and homeoboxes 1(ZHX1)
M_66	4796	tonsoku-like, DNA repair protein(TONSL)
M_66	55156	armadillo repeat containing 1(ARMC1)
M_66	137682	NADH:ubiquinone oxidoreductase complex assembly factor 6(NDUFAF6)
M_66	56943	ENY2, transcription and export complex 2
M_66	55039	tRNA methyltransferase 12 homolog(TRMT12)
M_66	51001	mitochondrial transcription termination factor
M_66	23212	ribosome biogenesis regulator homolog(RRS1)
M_66	157777	minichromosome maintenance domain containing 2(MCMDC2)
M_66	23678	serum/glucocorticoid regulated kinase family member 3(SGK3)
M_66	27067	staufen double-stranded RNA binding protein
M_66	50484	ribonucleotide reductase regulatory TP53 inducible subunit M2B(RRM2B)
M_66	84933	chromosome 8 open reading frame 76(C8orf76)
M_66	619279	zinc finger protein 704(ZNF704)
M_66	54968	transmembrane protein 70(TMEM70)
M_66	286144	triple QxxK/R motif containing(TRIQK)
M_66	11059	WW domain containing E3 ubiquitin protein ligase
M_66	56260	chromosome 8 open reading frame 44(C8orf44)
M_66	401466	chromosome 8 open reading frame 59(C8orf59)
M_66	286148	dpy-19 like 4 (C. elegans)(DPY19L4)

M_66	79848	centrosome and spindle pole associated protein
M_66	23167	EFR3 homolog A(EFR3A)
M_66	55656	integrator complex subunit 8(INTS8)
M_66	2131	exostosin glycosyltransferase 1(EXT1)
M_66	1345	cytochrome c oxidase subunit 6C(COX6C)
M_66	6788	serine/threonine kinase 3(STK3)
M_66	79075	DNA replication and sister chromatid cohesion
M_66	64089	sorting nexin 16(SNX16)
M_66	734	oxidative stress induced growth inhibitor family member 2(OSGIN2)
M_66	286053	NSE2/MMS21 homolog, SMC5-SMC6 complex SUMO ligase(NSMCE2)
M_66	55353	lysosomal protein transmembrane 4 beta(LAPTM4B)
M_66	4683	nibrin(NBN)
M_66	157567	ankyrin repeat domain 46(ANKRD46)
M_66	25879	DDB1 and CUL4 associated factor 13(DCAF13)
M_66	9897	KIAA0196(KIAA0196)
M_66	55074	oxidation resistance 1(OXR1)
M_66	5885	RAD21 cohesin complex component(RAD21)
M_66	2635	guanylate binding protein 3(GBP3)
M_66	7381	ubiquinol-cytochrome c reductase binding
M_66	29967	LDL receptor related protein 12(LRP12)
M_66	11144	DNA meiotic recombinase 1(DMC1)
M_66	6156	ribosomal protein L30(RPL30)
M_66	3242	4-hydroxyphenylpyruvate dioxygenase(HPD)
M_66	6674	sperm associated antigen 1(SPAG1)
M_66	80157	cell wall biogenesis 43 C-terminal homolog(CWH43)
M_66	4603	MYB proto-oncogene like 1(MYBL1)
M_66	7336	ubiquitin conjugating enzyme E2 V2(UBE2V2)
M_66	389677	RNA binding motif protein 12B(RBM12B)
M_66	389668	XK related 9(XKR9)
M_66	55472	RBM12B antisense RNA 1(RBM12B-AS1)

M_66	441455	makorin ring finger protein 1
M_67	7327	ubiquitin conjugating enzyme E2 G2(UBE2G2)
M_67	7485	tryptophan rich basic protein(WRB)
M_67	5822	PWP2 periodic tryptophan protein homolog
M_67	23275	protein O-fucosyltransferase 2(POFUT2)
M_67	104	adenosine deaminase, RNA specific B1(ADARB1)
M_67	23181	disco interacting protein 2 homolog A(DIP2A)
M_67	85395	family with sequence similarity 207 member
M_67	57466	SR-related CTD associated factor 4(SCAF4)
M_67	100130872	uncharacterized LOC100130872(LOC100130872)
M_67	114044	MCM3AP antisense RNA 1(MCM3AP-AS1)
M_67	84221	spermatogenesis and centriole associated 1-
M_67	7109	trafficking protein particle complex 10(TRAPPC10)
M_67	9980	dopey family member 2(DOPEY2)
M_67	6612	small ubiquitin-like modifier 3(SUMO3)
M_67	3150	high mobility group nucleosome binding domain
M_67	10785	WD repeat domain 4(WDR4)
M_67	3141	holocarboxylase synthetase(HLCS)
M_67	4047	lanosterol synthase (2,3-oxidosqualene-lanosterol cyclase)(LSS)
M_67	81543	leucine rich repeat containing 3(LRRC3)
M_67	3275	protein arginine methyltransferase 2(PRMT2)
M_67	2114	ETS proto-oncogene 2, transcription factor(ETS2)
M_67	8209	chromosome 21 open reading frame 33(C21orf33)
M_67	1859	dual specificity tyrosine phosphorylation regulated kinase 1A(DYRK1A)
M_67	23308	inducible T-cell costimulator ligand(ICOSLG)
M_67	642852	uncharacterized LOC642852(LOC642852)
M_67	54014	bromodomain and WD repeat domain containing
M_67	5211	phosphofructokinase, liver type(PFKL)
M_67	84536	long intergenic non-protein coding RNA
M_67	285498	ring finger protein 212(RNF212)

M_67	7307	U2 small nuclear RNA auxiliary factor 1(U2AF1)
M_67	54059	ybeY metallopeptidase (putative)(YBEY)
M_67	5116	pericentrin(PCNT)
M_67	54020	solute carrier family 37 member 1(SLC37A1)
M_67	25966	C2 calcium dependent domain containing 2(C2CD2)
M_67	8568	ribosomal RNA processing 1(RRP1)
M_67	284837	apoptosis associated transcript in bladder
M_67	728039	signal sequence receptor subunit 4 pseudogene
M_67	23076	ribosomal RNA processing 1B(RRP1B)
M_67	4731	NADH:ubiquinone oxidoreductase subunit
M_67	56894	1-acylglycerol-3-phosphate O-acyltransferase
M_67	9875	URB1 ribosome biogenesis 1 homolog (S.
M_67	63977	PR/SET domain 15(PRDM15)
M_67	8566	pyridoxal (pyridoxine, vitamin B6) kinase(PDXK)
M_67	7267	tetratricopeptide repeat domain 3(TTC3)
M_67	49854	zinc finger and BTB domain containing 21(ZBTB21)
M_67	755	chromosome 21 open reading frame 2(C21orf2)
M_67	754	pituitary tumor-transforming 1 interacting
M_67	6573	solute carrier family 19 member 1(SLC19A1)
M_67	8888	minichromosome maintenance complex component 3 associated protein(MCM3AP)
M_67	10841	formimidoyltransferase cyclodeaminase(FTCD)
M_67	54093	SET domain containing 4(SETD4)
M_67	257203	Down syndrome critical region 9 (non-protein coding)(DSCR9)
M_67	29947	DNA methyltransferase 3 like(DNMT3L)
M_67	10984	KCNQ1 opposite strand/antisense transcript 1 (non-protein coding)(KCNQ1OT1)
M_67	150082	LCA5L, lebercilin like(LCA5L)
M_67	54069	MIS18 kinetochore protein A(MIS18A)
M_67	56683	chromosome 21 open reading frame 59(C21orf59)
M_67	5316	PBX/knotted 1 homeobox 1(PKNOX1)

M_67	2618	phosphoribosylglycinamide formyltransferase, phosphoribosylglycinamide synthetase, phosphoribosylaminoimidazole synthetase(GART)
M_68	442721	leiomodin 2(LMOD2)
M_68	282775	olfactory receptor family 5 subfamily J member
M_68	54579	UDP glucuronosyltransferase family 1 member
M_68	126520	polo like kinase 5(PLK5)
M_68	100033444	small nucleolar RNA, C/D box 115-7(SNORD115-7)
M_68	389151	proline rich 23B(PRR23B)
M_68	653423	sperm associated antigen 11A(SPAG11A)
M_68	10407	sperm associated antigen 11B(SPAG11B)
M_68	245909	defensin beta 106A(DEFB106A)
M_68	6677	sperm adhesion molecule 1(SPAM1)
M_68	8383	olfactory receptor family 1 subfamily A member
M_68	245908	defensin beta 105A(DEFB105A)
M_68	4225	meprin A subunit beta(MEP1B)
M_68	390327	olfactory receptor family 6 subfamily C member
M_68	729627	proline rich 23A(PRR23A)
M_68	7827	NPHS2, podocin(NPHS2)
M_68	121130	olfactory receptor family 10 subfamily P member
M_68	81470	olfactory receptor family 2 subfamily G member
M_68	503497	membrane spanning 4-domains A13(MS4A13)
M_68	245910	defensin beta 107A(DEFB107A)
M_68	414325	defensin beta 103A(DEFB103A)
M_68	64100	epididymal sperm binding protein 1(ELSPBP1)
M_68	407738	family with sequence similarity 19 member A1, C-C motif chemokine like(FAM19A1)
M_68	146481	FSHD region gene 2 family member D, pseudogene(FRG2DP)
M_68	27179	interleukin 36, alpha(IL36A)
M_68	390883	olfactory receptor family 7 subfamily G member
M_68	389152	proline rich 23C(PRR23C)

M_68	386682	keratin associated protein 10-3(KRTAP10-3)
M_68	140596	defensin beta 104A(DEFB104A)
M_68	386679	keratin associated protein 10-2(KRTAP10-2)
M_68	441932	olfactory receptor family 2 subfamily W member 5 (gene/pseudogene)(OR2W5)
M_68	100131137	binder of sperm protein homolog 1(BSPH1)
M_68	128817	cystatin like 1(CSTL1)
M_68	136263	serine rich single-pass membrane protein 1(SSMEM1)
M_68	5026	purinergic receptor P2X 5(P2RX5)
M_68	245929	defensin beta 115(DEFB115)
M_68	125958	olfactory receptor family 7 subfamily D member
M_68	119749	olfactory receptor family 4 subfamily C member
M_68	125962	olfactory receptor family 7 subfamily G member
M_68	127059	olfactory receptor family 2 subfamily M member
M_68	497634	long intergenic non-protein coding RNA
M_68	386675	keratin associated protein 10-7(KRTAP10-7)
M_68	9287	trace amine associated receptor 2(TAAR2)
M_68	148066	zinc and ring finger 4(ZNRF4)
M_68	3883	keratin 33A(KRT33A)
M_68	386672	keratin associated protein 10-4(KRTAP10-4)
M_68	9313	matrix metallopeptidase 20(MMP20)
M_68	114770	peptidoglycan recognition protein 2(PGLYRP2)
M_68	163479	fibronectin type III domain containing 7(FNDC7)
M_68	390882	olfactory receptor family 7 subfamily G member
M_68	286359	uncharacterized LOC286359(LOC286359)
M_68	81472	olfactory receptor family 2 subfamily C member
M_68	63948	DMRT like family B with proline rich C-terminal
M_68	92346	chromosome 1 open reading frame 105(C1orf105)
M_68	148824	GCSAML antisense RNA 1(GCSAML-AS1)
M_68	84075	fibrous sheath CABYR binding protein(FSCB)
M_68	1007	cadherin 9(CDH9)
M_68	389124	IQ motif containing F5(IQCF5)

M_68	9949	Alport syndrome, mental retardation, midface hypoplasia and elliptocytosis chromosomal region
M_68	64101	leucine rich repeat containing 4(LRRC4)
M_68	690	basic transcription factor 3 pseudogene 11(BTF3P11)
M_68	153328	solute carrier family 25 member 48(SLC25A48)
M_68	170825	GS homeobox 2(GSX2)
M_68	84254	calcium/calmodulin dependent protein kinase kinase 1(CAMKK1)
M_68	163742	solute carrier family 25 member 3 pseudogene
M_68	126767	arylacetamide deacetylase like 3(AADACL3)
M_68	132724	transmembrane protease, serine 11B(TMPRSS11B)
M_68	1413	crystallin beta A4(CRYBA4)
M_68	125963	olfactory receptor family 1 subfamily M member
M_68	4316	matrix metallopeptidase 7(MMP7)
M_68	139105	BEN domain containing 2(BEND2)
M_68	26154	ATP binding cassette subfamily A member
M_68	338398	taste 2 receptor member 60(TAS2R60)
M_68	148823	germinal center associated signaling and motility like(GCSAML)
M_68	55301	oleoyl-ACP hydrolase(OLAH)
M_69	51699	VPS29, retromer complex component(VPS29)
M_69	57666	fibrosin like 1(FBRSL1)
M_69	6249	CAP-Gly domain containing linker protein 1(CLIP1)
M_69	51228	glycolipid transfer protein(GLTP)
M_69	23389	mediator complex subunit 13 like(MED13L)
M_69	7374	uracil DNA glycosylase(UNG)
M_69	27340	UTP20, small subunit processome component(UTP20)
M_69	35	acyl-CoA dehydrogenase, C-2 to C-3 short
M_69	9761	malectin(MLEC)
M_69	32	acetyl-CoA carboxylase beta(ACACB)
M_69	11021	RAB35, member RAS oncogene family(RAB35)
M_69	65117	arginine and serine rich coiled-coil 2(RSRC2)

M_69	7112	thymopoietin(TMPO)
M_69	23141	ankyrin repeat and LEM domain containing
M_69	488	ATPase sarcoplasmic/endoplasmic reticulum Ca2+ transporting 2(ATP2A2)
M_69	80018	N(alpha)-acetyltransferase 25, NatB auxiliary subunit(NAA25)
M_69	5901	RAN, member RAS oncogene family(RAN)
M_69	2967	general transcription factor IIH subunit 3(GTF2H3)
M_69	55596	zinc finger CCHC-type containing 8(ZCCHC8)
M_69	949	scavenger receptor class B member 1(SCARB1)
M_69	55884	WD repeat and SOCS box containing 2(WSB2)
M_69	55188	RIC8 guanine nucleotide exchange factor B(RIC8B)
M_69	1407	cryptochrome circadian clock 1(CRY1)
M_69	9026	huntingtin interacting protein 1 related(HIP1R)
M_69	65985	acetoacetyl-CoA synthetase(AACS)
M_69	5025	purinergic receptor P2X 4(P2RX4)
M_69	8683	serine and arginine rich splicing factor 9(SRSF9)
M_69	9921	ring finger protein 10(RNF10)
M_69	140707	BRI3 binding protein(BRI3BP)
M_69	26259	F-box and WD repeat domain containing 8(FBXW8)
M_69	121642	alkB homolog 2, alpha-ketoglutarate dependent dioxygenase(ALKBH2)
M_69	10645	calcium/calmodulin dependent protein kinase kinase 2(CAMKK2)
M_69	121665	signal peptide peptidase like 3(SPPL3)
M_69	11137	PWP1 homolog, endonuclein(PWP1)
M_69	83892	potassium channel tetramerization domain containing 10(KCTD10)
M_69	8315	BRCA1 associated protein(BRAP)
M_69	10094	actin related protein 2/3 complex subunit 3(ARPC3)
M_69	5829	paxillin(PXN)
M_69	400073	chromosome 12 open reading frame 76(C12orf76)

M_69	84678	lysine demethylase 2B(KDM2B)
M_69	55198	adaptor protein, phosphotyrosine interacting with PH domain and leucine zipper 2(APPL2)
M_69	57634	E1A binding protein p400(EP400)
M_69	79676	2-oxoglutarate and iron dependent oxygenase domain containing 2(OGFOD2)
M_69	80298	mitochondrial transcription termination factor
M_69	9904	RNA binding motif protein 19(RBM19)
M_69	51559	5'-nucleotidase domain containing 3(NT5DC3)
M_69	79050	nucleolar complex associated 4 homolog(NOC4L)
M_69	80724	acyl-CoA dehydrogenase family member
M_69	79039	DEAD-box helicase 54(DDX54)
M_69	65082	VPS33A, CORVET/HOPS core subunit(VPS33A)
M_69	4598	mevalonate kinase(MVK)
M_69	11066	small nuclear ribonucleoprotein U11/U12 subunit 35(SNRNP35)
M_69	605	BCL tumor suppressor 7A(BCL7A)
M_69	23067	SET domain containing 1B(SETD1B)
M_69	192111	PGAM family member 5, mitochondrial serine/threonine protein phosphatase(PGAM5)
M_69	387893	lysine methyltransferase 5A(KMT5A)
M_69	5564	protein kinase AMP-activated non-catalytic subunit beta 1(PRKAB1)
M_69	160760	PTC7 protein phosphatase homolog(PPTC7)
M_69	54434	slingshot protein phosphatase 1(SSH1)
M_69	22877	MLX interacting protein(MLXIP)
M_69	8099	cyclin dependent kinase 2 associated protein
M_69	84260	trichoplein keratin filament binding(TCHP)
M_69	7699	zinc finger protein 140(ZNF140)
M_69	56616	diablo IAP-binding mitochondrial protein(DIABLO)
M_69	255394	t-complex 11 like 2(TCP11L2)
M_69	10959	transmembrane p24 trafficking protein 2(TMED2)

M_69	10961	endoplasmic reticulum protein 29(ERP29)
M_69	84900	ring finger protein, transmembrane 2(RNFT2)
M_69	7574	zinc finger protein 26(ZNF26)
M_69	353116	Rab interacting lysosomal protein like 1(RILPL1)
M_69	8562	density regulated re-initiation and release
M_69	10906	TRAF-type zinc finger domain containing
M_69	5985	replication factor C subunit 5(RFC5)
M_69	8408	unc-51 like autophagy activating kinase 1(ULK1)
M_69	5501	protein phosphatase 1 catalytic subunit
M_69	84274	coenzyme Q5, methyltransferase(COQ5)
M_69	1967	eukaryotic translation initiation factor 2B subunit alpha(EIF2B1)
M_69	57647	DEAH-box helicase 37(DHX37)
M_69	10795	zinc finger protein 268(ZNF268)
M_69	64426	SDS3 homolog, SIN3A corepressor complex component(SUDS3)
M_69	80196	ring finger protein 34(RNF34)
M_69	79867	tectonic family member 2(TCTN2)
M_69	6311	ataxin 2(ATXN2)
M_69	6433	splicing factor SWAP homolog(SFSWAP)
M_69	51184	GPN-loop GTPase 3(GPN3)
M_69	8550	mitogen-activated protein kinase-activated protein kinase 5(MAPKAPK5)
M_69	51329	ADP ribosylation factor like GTPase 6 interacting protein 4(ARL6IP4)
M_69	144348	zinc finger protein 664(ZNF664)
M_69	283450	HECT domain E3 ubiquitin protein ligase
M_69	10985	GCN1, eIF2 alpha kinase activator homolog(GCN1)
M_69	7637	zinc finger protein 84(ZNF84)
M_69	11153	FIC domain containing(FICD)
M_69	79720	VPS37B, ESCRT-I subunit(VPS37B)
M_69	283431	growth arrest specific 2 like 3(GAS2L3)

M_69	144404	transmembrane protein 120B(TMEM120B)
M_69	80324	pseudouridylate synthase 1(PUS1)
M_69	55206	strawberry notch homolog 1(SBNO1)
M_69	80212	coiled-coil domain containing 92(CCDC92)
M_69	23479	iron-sulfur cluster assembly enzyme(ISCU)
M_69	84876	ORAI calcium release-activated calcium modulator
M_69	5426	DNA polymerase epsilon, catalytic subunit(POLE)
M_69	5715	proteasome 26S subunit, non-ATPase 9(PSMD9)
M_69	91574	chromosome 12 open reading frame 65(C12orf65)
M_69	326625	methylmalonic aciduria (cobalamin deficiency) cblB type(MMAB)
M_69	10198	M-phase phosphoprotein 9(MPHOSPH9)
M_69	9815	GIT ArfGAP 2(GIT2)
M_69	5037	phosphatidylethanolamine binding protein 1(PEBP1)
M_69	9733	squamous cell carcinoma antigen recognized by T-cells 3(SART3)
M_69	9735	kinetochore associated 1(KNTC1)
M_69	84747	unc-119 lipid binding chaperone B(UNC119B)
M_69	7556	zinc finger protein 10(ZNF10)
M_69	55743	checkpoint with forkhead and ring finger
M_69	728568	chromosome 12 open reading frame 73(C12orf73)
M_69	100289635	zinc finger protein 605(ZNF605)
M_69	51499	TP53 regulated inhibitor of apoptosis 1(TRIAP1)
M_69	283459	glutamyl-tRNA amidotransferase subunit C(GATC)
M_69	64897	chromosome 12 open reading frame 43(C12orf43)
M_69	5781	protein tyrosine phosphatase, non-receptor type
M_69	84934	RBPJ interacting and tubulin associated 1(RITA1)
M_69	100128191	TMPO antisense RNA 1(TMPO-AS1)
M_69	55703	RNA polymerase III subunit B(POLR3B)
M_69	79794	chromosome 12 open reading frame 49(C12orf49)
M_69	317781	DEAD-box helicase 51(DDX51)
M_69	84749	ubiquitin specific peptidase 30(USP30)

M_69	11108	PR/SET domain 4(PRDM4)
M_69	23545	ATPase H+ transporting V0 subunit a2(ATP6V0A2)
M_69	2802	golgin A3(GOLGA3)
M_69	28981	intraflagellar transport 81(IFT81)
M_69	9612	nuclear receptor corepressor 2(NCOR2)
M_69	51367	POP5 homolog, ribonuclease P/MRP subunit(POP5)
M_69	89910	ubiquitin protein ligase E3B(UBE3B)
M_69	51275	MAPKAPK5 antisense RNA 1(MAPKAPK5-AS1)
M_69	57696	DEAD-box helicase 55(DDX55)
M_69	51433	anaphase promoting complex subunit 5(ANAPC5)
M_69	51434	anaphase promoting complex subunit 7(ANAPC7)
M_69	121053	chromosome 12 open reading frame 45(C12orf45)
M_69	26271	F-box protein 5(FBXO5)
M_69	5933	RB transcriptional corepressor like 1(RBL1)
M_69	253724	tetratricopeptide repeat domain 41,
M_69	6996	thymine DNA glycosylase(TDG)
M_69	5827	peroxisomal membrane protein 2(PXMP2)
M_69	4801	nuclear transcription factor Y subunit beta(NFYB)
M_69	29902	family with sequence similarity 216 member
M_69	387882	chromosome 12 open reading frame 75(C12orf75)
M_69	7184	heat shock protein 90 beta family member
M_69	79369	UDP-GlcNAc:betaGal beta-1,3-N-acetylglucosaminyltransferase 4(B3GNT4)
M_69	1466	cysteine and glycine rich protein 2(CSRP2)
M_70	54812	aftiphilin(AFTPH)
M_70	150962	pseudouridylate synthase 10(PUS10)
M_70	54978	solute carrier family 35 member F6(SLC35F6)
M_70	55133	S1 RNA binding domain 1(SRBD1)
M_70	9792	SERTA domain containing 2(SERTAD2)
M_70	10678	UDP-GlcNAc:betaGal beta-1,3-N-acetylglucosaminyltransferase 2(B3GNT2)
M_70	23160	WD repeat domain 43(WDR43)

M_70	54867	transmembrane protein 214(TMEM214)
M_70	4528	mitochondrial translational initiation factor 2(MTIF2)
M_70	55471	NADH:ubiquinone oxidoreductase complex assembly factor 7(NDUFAF7)
M_70	5500	protein phosphatase 1 catalytic subunit beta(PPP1CB)
M_70	11017	small nuclear ribonucleoprotein U4/U6.U5 subunit 27(SNRNP27)
M_70	7444	vaccinia related kinase 2(VRK2)
M_70	51449	prenylcysteine oxidase 1(PCYOX1)
M_70	10797	methylenetetrahydrofolate dehydrogenase (NADP+ dependent) 2, methenyltetrahydrofolate cyclohydrolase(MTHFD2)
M_70	55120	Fanconi anemia complementation group L(FANCL)
M_70	23198	proteasome activator subunit 4(PSME4)
M_70	1733	deiodinase, iodothyronine type I(DIO1)
M_70	7360	UDP-glucose pyrophosphorylase 2(UGP2)
M_70	2673	glutamine--fructose-6-phosphate transaminase
M_70	29094	galectin like(LGALSL)
M_70	10097	ARP2 actin related protein 2 homolog(ACTR2)
M_70	9801	mitochondrial ribosomal protein L19(MRPL19)
M_70	87178	polyribonucleotide nucleotidyltransferase 1(PNPT1)
M_70	90411	multiple coagulation factor deficiency 2(MCFD2)
M_70	51542	VPS54, GARP complex subunit(VPS54)
M_70	9655	suppressor of cytokine signaling 5(SOCS5)
M_70	5534	protein phosphatase 3 regulatory subunit B,
M_70	6936	GC-rich sequence DNA-binding factor 2(GCFC2)
M_70	284441	actin-related protein 2 pseudogene(LOC284441)
M_70	84140	family with sequence similarity 161 member
M_70	57504	metastasis associated 1 family member 3(MTA3)
M_70	11037	stonin 1(STON1)
M_70	93517	short chain dehydrogenase/reductase family 42E, member 1(SDR42E1)

M_70	5495	protein phosphatase, Mg2+/Mn2+ dependent
M_70	57835	solute carrier family 4 member 5(SLC4A5)
M_70	9177	5-hydroxytryptamine receptor 3B(HTR3B)
M_70	9451	eukaryotic translation initiation factor 2 alpha kinase 3(EIF2AK3)
M_70	84542	KIAA1841(KIAA1841)
M_70	9637	fasciculation and elongation protein zeta 2(FEZ2)
M_70	51652	charged multivesicular body protein 3(CHMP3)
M_70	4084	MAX dimerization protein 1(MXD1)
M_70	10153	CCAAT/enhancer binding protein zeta(CEBPZ)
M_70	8491	mitogen-activated protein kinase kinase kinase kinase 3(MAP4K3)
M_70	63892	THADA, armadillo repeat containing(THADA)
M_70	10199	M-phase phosphoprotein 10(MPHOSPH10)
M_70	25940	family with sequence similarity 98 member
M_70	92906	heterogeneous nuclear ribonucleoprotein L
M_70	27248	endoplasmic reticulum lectin 1(ERLEC1)
M_70	10438	C1D nuclear receptor corepressor(C1D)
M_70	5093	poly(rC) binding protein 1(PCBP1)
M_70	55622	tetratricopeptide repeat domain 27(TTC27)
M_70	253635	G-patch domain containing 11(GPATCH11)
M_70	64395	germ cell-less, spermatogenesis associated 1(GMCL1)
M_70	55233	MOB kinase activator 1A(MOB1A)
M_70	64895	poly(A) polymerase gamma(PAPOLG)
M_70	7514	exportin 1(XPO1)
M_70	23683	protein kinase D3(PRKD3)
M_70	5861	RAB1A, member RAS oncogene family(RAB1A)
M_70	27436	echinoderm microtubule associated protein like
M_70	3344	forkhead box N2(FOXN2)
M_70	4436	mutS homolog 2(MSH2)
M_70	51646	yippee like 5(YPEL5)
M_70	51130	ankyrin repeat and SOCS box containing 3(ASB3)

M_70	57142	reticulon 4(RTN4)
M_70	80204	F-box protein 11(FBXO11)
M_70	5194	peroxisomal biogenesis factor 13(PEX13)
M_70	9581	prolyl endopeptidase-like(PREPL)
M_70	129285	protein phosphatase 1 regulatory subunit
M_70	57223	protein phosphatase 4 regulatory subunit
M_70	2956	mutS homolog 6(MSH6)
M_70	7844	ring finger protein 103(RNF103)
M_70	9263	serine/threonine kinase 17a(STK17A)
M_70	10128	leucine rich pentatricopeptide repeat
M_70	54884	retinol saturase(RETSAT)
M_70	80745	THUMP domain containing 2(THUMPD2)
M_70	5295	phosphoinositide-3-kinase regulatory subunit
M_70	113419	testis expressed 261(TEX261)
M_70	116143	WD repeat domain 92(WDR92)
M_70	64225	atlastin GTPase 2(ATL2)
M_70	2677	gamma-glutamyl carboxylase(GGCX)
M_70	30845	EH domain containing 3(EHD3)
M_70	440854	calpain 14(CAPN14)
M_70	94241	tumor protein p53 inducible nuclear protein
M_70	388403	yippee like 2(YPEL2)
M_71	23077	MYC binding protein 2, E3 ubiquitin protein
M_71	84899	transmembrane and tetratricopeptide repeat containing 4(TMTC4)
M_71	3843	importin 5(IPO5)
M_71	54602	Nedd4 family interacting protein 2(NDFIP2)
M_71	84945	abhydrolase domain containing 13(ABHD13)
M_71	3621	inhibitor of growth family member 1(ING1)
M_71	22894	DIS3 homolog, exosome endoribonuclease and 3'-5' exoribonuclease(DIS3)
M_71	10150	muscleblind like splicing regulator 2(MBNL2)
M_71	5911	RAP2A, member of RAS oncogene family(RAP2A)

M_71	79587	cysteinyl-tRNA synthetase 2, mitochondrial (putative)(CARS2)
M_71	140432	ring finger protein 113B(RNF113B)
M_71	55002	transmembrane and coiled-coil domains 3(TMCO3)
M_71	283489	chromosome alignment maintaining phosphoprotein 1(CHAMP1)
M_71	54841	basic, immunoglobulin-like variable motif
M_71	9375	transmembrane 9 superfamily member 2(TM9SF2)
M_71	93081	testis expressed 30(TEX30)
M_71	10464	progesterone immunomodulatory binding factor
M_71	7027	transcription factor Dp-1(TFDP1)
M_71	55739	NAD(P)HX dehydratase(NAXD)
M_71	5095	propionyl-CoA carboxylase alpha subunit(PCCA)
M_71	65110	UPF3 regulator of nonsense transcripts homolog A (yeast)(UPF3A)
M_71	23483	TDP-glucose 4,6-dehydratase(TGDS)
M_71	3916	lysosomal associated membrane protein 1(LAMP1)
M_71	8874	Rho guanine nucleotide exchange factor
M_71	55208	defective in cullin neddylation 1 domain containing 2(DCUN1D2)
M_71	360200	transmembrane protease, serine 9(TMPRSS9)
M_71	11278	Kruppel like factor 12(KLF12)
M_71	3981	DNA ligase 4(LIG4)
M_71	440145	mitotic spindle organizing protein 1(MZT1)
M_71	8428	serine/threonine kinase 24(STK24)
M_71	79596	ring finger protein 219(RNF219)
M_71	55757	UDP-glucose glycoprotein glucosyltransferase
M_71	10160	FERM, ARH/RhoGEF and pleckstrin domain protein 1(FARP1)
M_71	10426	tubulin gamma complex associated protein
M_71	55608	ankyrin repeat domain 10(ANKRD10)

M_71	5611	DnaJ heat shock protein family (Hsp40) member C3(DNAJC3)
M_71	337867	UBA domain containing 2(UBAC2)
M_71	8451	cullin 4A(CUL4A)
M_71	55795	PCI domain containing 2(PCID2)
M_71	64062	RNA binding motif protein 26(RBM26)
M_71	55082	arginine and glutamate rich 1(ARGLU1)
M_71	26224	F-box and leucine rich repeat protein 3(FBXL3)
M_71	160897	G protein-coupled receptor 180(GPR180)
M_71	7174	tripeptidyl peptidase 2(TPP2)
M_71	8881	cell division cycle 16(CDC16)
M_71	2073	ERCC excision repair 5, endonuclease(ERCC5)
M_71	87769	gamma-glutamylamine cyclotransferase(GGACT)
M_71	113622	ADP-ribosylhydrolase like 1(ADPRHL1)
M_71	79774	growth hormone regulated TBC protein 1(GRTP1)
M_72	22907	DExH-box helicase 30(DHX30)
M_72	3913	laminin subunit beta 2(LAMB2)
M_72	158056	MAM domain containing 4(MAMDC4)
M_72	79443	FYVE and coiled-coil domain containing 1(FYCO1)
M_72	285268	zinc finger protein 621(ZNF621)
M_72	8314	BRCA1 associated protein 1(BAP1)
M_72	23276	kelch like family member 18(KLHL18)
M_72	1605	dystroglycan 1(DAG1)
M_72	386724	adhesion molecule with Ig like domain 3(AMIGO3)
M_72	55802	decapping mRNA 1A(DCP1A)
M_72	22937	SREBF chaperone(SCAP)
M_72	25930	protein tyrosine phosphatase, non-receptor type
M_72	10180	RNA binding motif protein 6(RBM6)
M_72	63891	ring finger protein 123(RNF123)
M_72	7375	ubiquitin specific peptidase 4(USP4)
M_72	9223	membrane associated guanylate kinase, WW and PDZ domain containing 1(MAGI1)

M_72	29072	SET domain containing 2(SETD2)
M_72	10168	zinc finger protein 197(ZNF197)
M_72	353274	zinc finger protein 445(ZNF445)
M_72	51409	HemK methyltransferase family member 1(HEMK1)
M_72	23132	RAD54-like 2 (S. cerevisiae)(RAD54L2)
M_72	4485	macrophage stimulating 1(MST1)
M_72	84276	nicolin 1(NICN1)
M_72	2057	erythropoietin receptor(EPOR)
M_72	29890	RNA binding motif protein 15B(RBM15B)
M_72	80335	WD repeat domain 82(WDR82)
M_72	51447	inositol hexakisphosphate kinase 2(IP6K2)
M_72	11188	nischarin(NISCH)
M_72	23122	cytoplasmic linker associated protein 2(CLASP2)
M_72	5364	plexin B1(PLXNB1)
M_72	55193	polybromo 1(PBRM1)
M_72	23218	neurobeachin like 2(NBEAL2)
M_72	285331	coiled-coil domain containing 66(CCDC66)
M_72	10869	ubiquitin specific peptidase 19(USP19)
M_72	22906	trafficking kinesin protein 1(TRAK1)
M_72	6599	SWI/SNF related, matrix associated, actin dependent regulator of chromatin subfamily c member
M_72	10181	RNA binding motif protein 5(RBM5)
M_72	84126	ATR interacting protein(ATRIP)
M_72	93973	ARP8 actin-related protein 8 homolog(ACTR8)
M_72	9807	inositol hexakisphosphate kinase 1(IP6K1)
M_72	9943	oxidative stress responsive 1(OXSR1)
M_72	10425	ariadne RBR E3 ubiquitin protein ligase 2(ARIH2)
M_72	4134	microtubule associated protein 4(MAP4)
M_72	9730	DDB1 and CUL4 associated factor 1(DCAF1)
M_72	7155	topoisomerase (DNA) II beta(TOP2B)
M_72	91869	RFT1 homolog(RFT1)
M_72	93	activin A receptor type 2B(ACVR2B)

M_72	51385	zinc finger protein 589(ZNF589)
M_72	92999	zinc finger and BTB domain containing 47(ZBTB47)
M_72	23395	leucyl-tRNA synthetase 2, mitochondrial(LARS2)
M_72	57139	ral guanine nucleotide dissociation stimulator like
M_72	55830	glycosyltransferase 8 domain containing 1(GLT8D1)
M_72	57599	WD repeat domain 48(WDR48)
M_72	8994	LIM domains containing 1(LIMD1)
M_72	51517	NCK interacting protein with SH3 domain(NCKIPSD)
M_72	26354	G protein nucleolar 3(GNL3)
M_72	375341	chromosome 3 open reading frame 62(C3orf62)
M_72	6787	NIMA related kinase 4(NEK4)
M_72	54870	glutamine rich 1(QRICH1)
M_72	389813	chromosome 9 open reading frame 172(C9orf172)
M_72	11180	WD repeat domain 6(WDR6)
M_72	54986	unc-51 like kinase 4(ULK4)
M_72	1294	collagen type VII alpha 1 chain(COL7A1)
M_72	275	aminomethyltransferase(AMT)
M_72	90226	urocortin 2(UCN2)
M_72	199221	DAZ interacting zinc finger protein 1 like(DZIP1L)
M_72	84960	coiled-coil domain containing 183(CCDC183)
M_72	284723	solute carrier family 25 member 34(SLC25A34)
M_72	5576	protein kinase cAMP-dependent type II regulatory subunit alpha(PRKAR2A)
M_72	51208	claudin 18(CLDN18)
M_72	54585	leucine zipper transcription factor like 1(LZTFL1)
M_72	64147	kinesin family member 9(KIF9)
M_72	285349	zinc finger protein 660(ZNF660)
M_72	2054	syntaxin 2(STX2)
M_72	4130	microtubule associated protein 1A(MAP1A)
M_72	57456	KIAA1143(KIAA1143)
M_72	113691	tubulin alpha 3f pseudogene(TUBA3FP)
M_72	152206	coiled-coil domain containing 13(CCDC13)

M_72	388428	AATK antisense RNA 1(AATK-AS1)
M_72	8927	bassoon presynaptic cytomatrix protein(BSN)
M_72	64943	5'-nucleotidase domain containing 2(NT5DC2)
M_72	149134	long intergenic non-protein coding RNA
M_72	444882	IGF like family member 4(IGFL4)
M_72	9625	apoptosis associated tyrosine kinase(AATK)
M_73	727924	uncharacterized LOC727924(LOC727924)
M_73	283694	olfactory receptor family 4 subfamily N member
M_73	343068	PRAME family member 5(PRAMEF5)
M_73	642569	tripartite motif containing 53A,
M_73	729384	tripartite motif containing 49D2(TRIM49D2)
M_73	57093	tripartite motif containing 49(TRIM49)
M_73	317701	vomeronasal 1 receptor 2(VN1R2)
M_73	389073	chromosome 2 open reading frame 80(C2orf80)
M_73	392376	olfactory receptor family 13 subfamily C member
M_73	65122	PRAME family member 2(PRAMEF2)
M_73	400735	PRAME family member 4(PRAMEF4)
M_73	139135	PAS domain containing 1(PASD1)
M_73	390429	olfactory receptor family 4 subfamily N member
M_73	125997	methyl-CpG binding domain protein 3 like
M_73	440560	PRAME family member 11(PRAMEF11)
M_73	65121	PRAME family member 1(PRAMEF1)
M_73	84767	tripartite motif-containing 51(TRIM51)
M_73	390081	olfactory receptor family 52 subfamily E member
M_73	606	neurobeachin pseudogene 1(NBEAP1)
M_73	390079	olfactory receptor family 52 subfamily E member
M_73	390078	olfactory receptor family 52 subfamily E member
M_73	390075	olfactory receptor family 52 subfamily N member
M_73	3350	5-hydroxytryptamine receptor 1A(HTR1A)
M_73	440561	PRAME family member 6(PRAMEF6)
M_73	342900	leucine twenty homeobox(LEUTX)
M_73	391002	PRAME family member 8(PRAMEF8)

M_73	390999	PRAME family member 12(PRAMEF12)
M_73	390539	olfactory receptor family 4 subfamily N member 3 pseudogene(OR4N3P)
M_73	343071	PRAME family member 10(PRAMEF10)
M_73	343070	PRAME family member 9(PRAMEF9)
M_73	79473	olfactory receptor family 52 subfamily N member
M_73	92086	gamma-glutamyltransferase light chain 1(GGTLC1)
M_73	284428	methyl-CpG binding domain protein 3 like
M_73	122183	proline rich 20A(PRR20A)
M_73	284355	tetrapeptide repeat homeobox 1(TPRX1)
M_73	400736	PRAME family member 13(PRAMEF13)
M_73	388677	notch 2 N-terminal like(NOTCH2NL)
M_73	79097	tripartite motif containing 48(TRIM48)
M_73	729528	PRAME family member 14(PRAMEF14)
M_73	8749	ADAM metallopeptidase domain 18(ADAM18)
M_73	390538	olfactory receptor family 4 subfamily M member
M_73	440225	neurofibromin 1 pseudogene 2(NF1P2)
M_73	645425	PRAME family member 20(PRAMEF20)
M_73	1418	crystallin gamma A(CRYGA)
M_73	286362	olfactory receptor family 13 subfamily C member
M_73	338751	olfactory receptor family 52 subfamily L member
M_73	646214	p21 protein (Cdc42/Rac)-activated kinase 2 pseudogene(LOC646214)
M_73	390084	olfactory receptor family 56 subfamily A member
M_73	27231	nicotinamide riboside kinase 2(NMRK2)
M_73	646603	chromosome 4 open reading frame 51(C4orf51)
M_73	134391	G protein-coupled receptor 151(GPR151)
M_73	5673	pregnancy specific beta-1-glycoprotein 5(PSG5)
M_73	390066	olfactory receptor family 52 subfamily D member
M_73	390191	olfactory receptor family 5 subfamily B member
M_73	390190	olfactory receptor family 5 subfamily B member
M_73	2169	fatty acid binding protein 2(FABP2)

M_73	112744	interleukin 17F(IL17F)
M_73	221823	phosphoribosyl pyrophosphate synthetase 1-like
M_73	387638	chromosome 10 open reading frame 113(C10orf113)
M_73	84659	ribonuclease A family member 7(RNASE7)
M_73	196335	olfactory receptor family 56 subfamily B member
M_73	390077	olfactory receptor family 52 subfamily N member
M_73	85300	ATCAY, caytaxin(ATCAY)
M_73	729522	acetoacetyl-CoA synthetase pseudogene 1(AACSP1)
M_73	165721	DnaJ heat shock protein family (Hsp40) member B8(DNAJB8)
M_73	339512	coiled-coil domain containing 190(CCDC190)
M_73	84684	INSM transcriptional repressor 2(INSM2)
M_73	54457	TATA-box binding protein associated factor 7
M_73	440163	ribonuclease A family member 13
M_73	374897	suprabasin(SBSN)
M_73	284451	outer dense fiber of sperm tails 3 like 2(ODF3L2)
M_73	220429	CTAGE family member 10, pseudogene(CTAGE10P)
M_74	55763	exocyst complex component 1(EXOC1)
M_74	11019	lipoic acid synthetase(LIAS)
M_74	343066	arylacetamide deacetylase like 4(AADACL4)
M_74	84708	ligand of numb-protein X 1(LNX1)
M_74	51091	Sep (O-phosphoserine) tRNA:Sec (selenocysteine) tRNA synthase(SEPSECS)
M_74	57606	SLAIN motif family member 2(SLAIN2)
M_74	55153	SDA1 domain containing 1(SDAD1)
M_74	1633	deoxycytidine kinase(DCK)
M_74	5981	replication factor C subunit 1(RFC1)
M_74	10463	solute carrier family 30 member 9(SLC30A9)
M_74	6133	ribosomal protein L9(RPL9)
M_74	57050	UTP3, small subunit processome component homolog (S. cerevisiae)(UTP3)
M_74	92597	MOB kinase activator 1B(MOB1B)

M_74	84273	nitric oxide associated 1(NOA1)
M_74	55589	BMP2 inducible kinase(BMP2K)
M_74	6443	sarcoglycan beta(SGCB)
M_74	3093	ubiquitin conjugating enzyme E2 K(UBE2K)
M_74	25898	ring finger and CHY zinc finger domain containing
M_74	22902	RUN and FYVE domain containing 3(RUFY3)
M_74	441024	methylenetetrahydrofolate dehydrogenase (NADP+ dependent) 2-like(MTHFD2L)
M_74	23142	defective in cullin neddylation 1 domain containing 4(DCUN1D4)
M_74	735301	small nucleolar RNA host gene 9(SNHG9)
M_74	285521	COX18, cytochrome c oxidase assembly
M_74	285527	FRY like transcription coactivator(FRYL)
M_74	54940	OCIA domain containing 1(OCIAD1)
M_74	84239	ATPase 13A4(ATP13A4)
M_74	201895	small integral membrane protein 14(SMIM14)
M_74	9662	centrosomal protein 135(CEP135)
M_74	5431	RNA polymerase II subunit B(POLR2B)
M_74	10983	cyclin I(CCNI)
M_74	23244	PDS5 cohesin associated factor A(PDS5A)
M_74	1665	DEAH-box helicase 15(DHX15)
M_74	3184	heterogeneous nuclear ribonucleoprotein
M_74	26511	cysteine rich hydrophobic domain 2(CHIC2)
M_74	81608	factor interacting with PAPOLA and CPSF1(FIP1L1)
M_74	8615	USO1 vesicle transport factor(USO1)
M_74	55276	phosphoglucomutase 2(PGM2)
M_74	7358	UDP-glucose 6-dehydrogenase(UGDH)
M_74	54951	COMM domain containing 8(COMMD8)
M_74	152579	sec1 family domain containing 2(SCFD2)
M_74	7294	TXK tyrosine kinase(TXK)
M_74	152519	NIPA like domain containing 1(NIPAL1)
M_74	152518	nuclear transcription factor, X-box binding like

M_74	1259	cyclic nucleotide gated channel alpha 1(CNGA1)
M_74	152815	THAP domain containing 6(THAP6)
M_74	132299	OCIA domain containing 2(OCIAD2)
M_74	9908	G3BP stress granule assembly factor 2(G3BP2)
M_74	5471	phosphoribosyl pyrophosphate
M_74	950	scavenger receptor class B member 2(SCARB2)
M_74	55858	transmembrane protein 165(TMEM165)
M_74	7006	tec protein tyrosine kinase(TEC)
M_74	9575	clock circadian regulator(CLOCK)
M_74	51274	Kruppel like factor 3(KLF3)
M_74	339977	leucine rich repeat containing 66(LRRC66)
M_74	91746	YTH domain containing 1(YTHDC1)
M_74	55236	ubiquitin like modifier activating enzyme 6(UBA6)
M_74	4807	nescient helix-loop-helix 1(NHLH1)
M_74	26057	ankyrin repeat domain 17(ANKRD17)
M_74	132949	aminoadipate-semialdehyde dehydrogenase(AASDH)
M_74	1060	centromere protein C(CENPC)
M_74	53371	nucleoporin 54(NUP54)
M_74	550112	UBA6 antisense RNA 1 (head to head)(UBA6-AS1)
M_74	55161	transmembrane protein 33(TMEM33)
M_74	29945	anaphase promoting complex subunit 4(ANAPC4)
M_74	2926	G-rich RNA sequence binding factor 1(GRSF1)
M_74	132789	glucosamine-6-phosphate deaminase 2(GNPDA2)
M_74	6731	signal recognition particle 72(SRP72)
M_74	246175	CCR4-NOT transcription complex subunit 6
M_74	29063	zinc finger CCHC-type containing 4(ZCCHC4)
M_74	60558	GUF1 homolog, GTPase(GUF1)
M_74	5978	RE1 silencing transcription factor(REST)
M_74	79644	steroid 5 alpha-reductase 3(SRD5A3)
M_74	64854	ubiquitin specific peptidase 46(USP46)

M_74	10606	phosphoribosylaminoimidazole carboxylase; phosphoribosylaminoimidazolesuccinocarboxamide synthase(PAICS)
M_74	9987	heterogeneous nuclear ribonucleoprotein D
M_74	901	cyclin G2(CCNG2)
M_74	55296	TBC1 domain family member 19(TBC1D19)
M_74	8987	starch binding domain 1(STBD1)
M_74	3516	recombination signal binding protein for immunoglobulin kappa J region(RBPJ)
M_74	57291	differentiation antagonizing non-protein coding
M_74	730668	dynein heavy chain -like pseudogene(LOC730668)
M_74	10396	ATPase phospholipid transporting 8A1(ATP8A1)
M_74	23216	TBC1 domain family member 1(TBC1D1)
M_74	91050	coiled-coil domain containing 149(CCDC149)
M_74	254251	ligand dependent nuclear receptor corepressor
M_74	57620	stromal interaction molecule 2(STIM2)
M_74	407	arrestin 3(ARR3)
M_74	1950	epidermal growth factor(EGF)
M_74	100271722	long intergenic non-protein coding RNA
M_74	768211	RELT like 1(RELL1)
M_74	55300	phosphatidylinositol 4-kinase type 2 beta(PI4K2B)
M_74	54502	RNA binding motif protein 47(RBM47)
M_74	116984	ArfGAP with RhoGAP domain, ankyrin repeat and PH domain 2(ARAP2)
M_74	23231	SEL1L family member 3(SEL1L3)
M_75	138199	carnosine N-methyltransferase 1(CARNMT1)
M_75	388335	transmembrane protein 220(TMEM220)
M_75	27130	inversin(INVS)
M_75	51552	RAB14, member RAS oncogene family(RAB14)
M_75	375748	ERCC excision repair 6 like 2(ERCC6L2)
M_75	375743	protein prenyltransferase alpha subunit repeat containing 1(PTAR1)

M_75	10541	acidic nuclear phosphoprotein 32 family member
M_75	23392	KIAA0368(KIAA0368)
M_75	159013	chromosome X open reading frame 38(CXorf38)
M_75	401548	sorting nexin family member 30(SNX30)
M_75	55342	spermatid perinuclear RNA binding protein(STRBP)
M_75	79762	chromosome 1 open reading frame 115(C1orf115)
M_75	23446	solute carrier family 44 member 1(SLC44A1)
M_75	23230	vacuolar protein sorting 13 homolog A(VPS13A)
M_75	23137	structural maintenance of chromosomes 5(SMC5)
M_75	7743	zinc finger protein 189(ZNF189)
M_75	8518	inhibitor of kappa light polypeptide gene enhancer in B-cells, kinase complex-associated protein(IKBKAP)
M_75	84641	major facilitator superfamily domain containing 14B(MFSD14B)
M_75	114987	WD repeat domain 31(WDR31)
M_75	10927	spindlin 1(SPIN1)
M_75	55014	syntaxin 17(STX17)
M_75	286205	suppressor of cancer cell invasion(SCAI)
M_75	1317	solute carrier family 31 member 1(SLC31A1)
M_75	445577	chromosome 9 open reading frame 129(C9orf129)
M_75	54942	family with sequence similarity 206 member
M_75	23287	ATP/GTP binding protein 1(AGTPBP1)
M_75	23299	BICD cargo adaptor 2(BICD2)
M_75	57684	zinc finger and BTB domain containing 26(ZBTB26)
M_75	7357	UDP-glucose ceramide glucosyltransferase(UGCG)
M_75	54566	erythrocyte membrane protein band 4.1 like
M_75	23196	family with sequence similarity 120A(FAM120A)
M_75	81932	haloacid dehalogenase like hydrolase domain containing 3(HDHD3)
M_75	158293	family with sequence similarity 120A opposite strand(FAM120AOS)
M_75	79670	zinc finger CCHC-type containing 6(ZCCHC6)

M_75	54187	N-acetylneuraminate synthase(NANS)
M_75	22954	tripartite motif containing 32(TRIM32)
M_75	2790	G protein subunit gamma 10(GNG10)
M_75	54542	ring finger and CCCH-type domains 2(RC3H2)
M_75	60560	N(alpha)-acetyltransferase 35, NatC auxiliary subunit(NAA35)
M_75	5537	protein phosphatase 6 catalytic subunit(PPP6C)
M_75	7543	zinc finger protein, X-linked(ZFX)
M_75	54107	DNA polymerase epsilon 3, accessory
M_75	9550	ATPase H+ transporting V1 subunit G1(ATP6V1G1)
M_75	5082	phosducin like(PDCL)
M_75	92400	RNA binding motif protein 18(RBM18)
M_75	9414	tight junction protein 2(TJP2)
M_75	22869	zinc finger protein 510(ZNF510)
M_75	257169	chromosome 9 open reading frame 43(C9orf43)
M_75	10773	zinc finger and BTB domain containing 6(ZBTB6)
M_75	7507	XPA, DNA damage recognition and repair
M_75	5774	protein tyrosine phosphatase, non-receptor type
M_75	23307	FK506 binding protein 15(FKBP15)
M_75	1515	cathepsin V(CTSV)
M_75	5711	proteasome 26S subunit, non-ATPase 5(PSMD5)
M_75	158427	thiosulfate sulfurtransferase like domain containing
M_75	158431	zinc finger protein 782(ZNF782)
M_75	85365	ALG2, alpha-1,3/1,6-mannosyltransferase(ALG2)
M_75	79109	mitogen-activated protein kinase associated protein 1(MAPKAP1)
M_75	54996	mitochondrial amidoxime reducing component
M_75	203286	ankyrin repeat and sterile alpha motif domain containing 6(ANKS6)
M_75	84131	centrosomal protein 78(CEP78)
M_75	7403	lysine demethylase 6A(KDM6A)
M_75	64768	inositol-pentakisphosphate 2-kinase(IPPK)

M_75	2902	glutamate ionotropic receptor NMDA type subunit
M_75	2776	G protein subunit alpha q(GNAQ)
M_75	55035	nucleolar protein 8(NOL8)
M_75	2218	fukutin(FKTN)
M_75	29979	ubiquilin 1(UBQLN1)
M_75	58493	INTS3 and NABP interacting protein(INIP)
M_75	9282	mediator complex subunit 14(MED14)
M_75	210	aminolevulinate dehydratase(ALAD)
M_75	23071	endoplasmic reticulum protein 44(ERP44)
M_75	8727	catenin alpha like 1(CTNNAL1)
M_75	548645	DnaJ heat shock protein family (Hsp40) member C25(DNAJC25)
M_75	402377	#N/A
M_75	23731	transmembrane protein 245(TMEM245)
M_75	7763	zinc finger AN1-type containing 5(ZFAND5)
M_75	10558	serine palmitoyltransferase long chain base subunit 1(SPTLC1)
M_75	79695	polypeptide N-acetylgalactosaminyltransferase
M_75	1654	DEAD-box helicase 3, X-linked(DDX3X)
M_75	56254	ring finger protein 20(RNF20)
M_75	8239	ubiquitin specific peptidase 9, X-linked(USP9X)
M_75	9991	polypyrimidine tract binding protein 3(PTBP3)
M_75	84263	hydroxysteroid dehydrogenase like 2(HSDL2)
M_75	5253	PHD finger protein 2(PHF2)
M_75	389816	leucine rich repeat containing 26(LRRC26)
M_75	7464	coronin 2A(CORO2A)
M_75	5887	RAD23 homolog B, nucleotide excision repair protein(RAD23B)
M_75	4686	nuclear cap binding protein subunit 1(NCBP1)
M_75	55582	kinesin family member 27(KIF27)
M_75	9128	pre-mRNA processing factor 4(PRPF4)
M_75	223	aldehyde dehydrogenase 9 family member

M_75	80709	AT-hook transcription factor(AKNA)
M_75	117531	transmembrane channel like 1(TMC1)
M_75	253039	PSMD5 antisense RNA 1 (head to head)(PSMD5-
M_75	22861	NLR family pyrin domain containing 1(NLRP1)
M_75	158405	KIAA1958(KIAA1958)
M_75	375757	SWI5 homologous recombination repair
M_75	9830	tripartite motif containing 14(TRIM14)
M_75	7504	X-linked Kx blood group(XK)
M_75	119369	nudix hydrolase 9 pseudogene 1(NUDT9P1)
M_76	5793	protein tyrosine phosphatase, receptor type G(PTPRG)
M_76	1846	dual specificity phosphatase 4(DUSP4)
M_76	1848	dual specificity phosphatase 6(DUSP6)
M_76	4088	SMAD family member 3(SMAD3)
M_76	283673	Ewing sarcoma associated transcript 1(EWSAT1)
M_76	28951	tribbles pseudokinase 2(TRIB2)
M_76	4091	SMAD family member 6(SMAD6)
M_76	26018	leucine rich repeats and immunoglobulin like domains 1(LRIG1)
M_76	81848	sprouty RTK signaling antagonist 4(SPRY4)
M_76	2119	ETS variant 5(ETV5)
M_76	2118	ETS variant 4(ETV4)
M_76	9781	ring finger protein 144A(RNF144A)
M_76	130574	LY6/PLAUR domain containing 6(LYPD6)
M_76	130576	LY6/PLAUR domain containing 6B(LYPD6B)
M_76	6563	solute carrier family 14 member 1 (Kidd blood group)(SLC14A1)
M_76	10066	secretory carrier membrane protein 2(SCAMP2)
M_76	4319	matrix metallopeptidase 10(MMP10)
M_76	6397	SEC14 like lipid binding 1(SEC14L1)
M_76	4862	neuronal PAS domain protein 2(NPAS2)
M_76	4092	SMAD family member 7(SMAD7)
M_76	2702	gap junction protein alpha 5(GJA5)

M_76	4123	mannosidase alpha class 2C member 1(MAN2C1)
M_76	4312	matrix metallopeptidase 1(MMP1)
M_76	80153	enhancer of mRNA decapping 3(EDC3)
M_76	8853	ArfGAP with SH3 domain, ankyrin repeat and PH domain 2(ASAP2)
M_76	29895	myosin light chain, phosphorylatable, fast skeletal muscle(MYLPF)
M_76	586	branched chain amino acid transaminase 1(BCAT1)
M_76	200734	sprouty related EVH1 domain containing 2(SPRED2)
M_76	257364	sorting nexin 33(SNX33)
M_76	5371	promyelocytic leukemia(PML)
M_76	5780	protein tyrosine phosphatase, non-receptor type
M_76	10252	sprouty RTK signaling antagonist 1(SPRY1)
M_76	10253	sprouty RTK signaling antagonist 2(SPRY2)
M_76	7090	transducin like enhancer of split 3(TLE3)
M_76	26035	glucuronic acid epimerase(GLCE)
M_76	23531	monocyte to macrophage differentiation
M_76	54852	progestin and adipoQ receptor family member
M_76	284266	sialic acid binding Ig like lectin 15(SIGLEC15)
M_76	56286	defender against cell death 1 pseudogene 1(DAD1P1)
M_76	56905	chromosome 15 open reading frame 39(C15orf39)
M_76	57477	shroom family member 4(SHROOM4)
M_76	56892	chromosome 8 open reading frame 4(C8orf4)
M_76	1198	CDC like kinase 3(CLK3)
M_76	1445	c-src tyrosine kinase(CSK)
M_76	161742	sprouty related EVH1 domain containing 1(SPRED1)
M_76	10801	septin 9(SEPT9)
M_76	25989	unc-51 like kinase 3(ULK3)
M_76	55273	transmembrane protein 100(TMEM100)
M_76	57184	family with sequence similarity 219 member
M_76	2615	leucine rich repeat containing 32(LRRC32)
M_76	58488	phosphatidylcholine transfer protein(PCTP)

M_76	26207	phosphatidylinositol transfer protein, cytoplasmic
M_76	152573	shisa family member 3(SHISA3)
M_76	84504	NK6 homeobox 2(NKX6-2)
M_76	80381	CD276 molecule(CD276)
M_76	64411	ArfGAP with RhoGAP domain, ankyrin repeat and PH domain 3(ARAP3)
M_76	10620	AT-rich interaction domain 3B(ARID3B)
M_76	56034	platelet derived growth factor C(PDGFC)
M_76	2743	glycine receptor beta(GLRB)
M_76	9844	engulfment and cell motility 1(ELMO1)
M_76	10913	ectodysplasin A receptor(EDAR)
M_76	2843	G protein-coupled receptor 20(GPR20)
M_76	4351	mannose phosphate isomerase(MPI)
M_76	5324	PLAG1 zinc finger(PLAG1)
M_76	83440	ADP dependent glucokinase(ADPGK)
M_76	80183	RUN and cysteine rich domain containing beclin 1 interacting protein like(RUBCNL)
M_76	83756	taste 1 receptor member 3(TAS1R3)
M_76	9985	REC8 meiotic recombination protein(REC8)
M_76	56965	poly(ADP-ribose) polymerase family member
M_76	56977	storkhead box 2(STOX2)
M_76	192668	cystin 1(CYS1)
M_76	284593	family with sequence similarity 41 member
M_76	79145	coiled-coil-helix-coiled-coil-helix domain containing 7(CHCHD7)
M_76	80072	HEXA antisense RNA 1(HEXA-AS1)
M_76	353345	G protein-coupled receptor 141(GPR141)
M_76	169693	transmembrane protein 252(TMEM252)
M_76	731779	long intergenic non-protein coding RNA
M_76	4435	Cbp/p300 interacting transactivator with Glu/Asp rich carboxy-terminal domain 1(CITED1)
M_76	3073	hexosaminidase subunit alpha(HEXA)

M_76	51314	NME/NM23 family member 8(NME8)
M_76	5315	pyruvate kinase, muscle(PKM)
M_76	4314	matrix metallopeptidase 3(MMP3)
M_77	5771	protein tyrosine phosphatase, non-receptor type
M_77	6875	TATA-box binding protein associated factor
M_77	84307	zinc finger protein 397(ZNF397)
M_77	55125	centrosomal protein 192(CEP192)
M_77	284252	potassium channel tetramerization domain containing 1(KCTD1)
M_77	55197	regulation of nuclear pre-mRNA domain containing 1A(RPRD1A)
M_77	5932	RB binding protein 8, endonuclease(RBBP8)
M_77	9218	VAMP associated protein A(VAPA)
M_77	81929	SEH1 like nucleoporin(SEH1L)
M_77	4864	NPC intracellular cholesterol transporter 1(NPC1)
M_77	114799	establishment of sister chromatid cohesion N-acetyltransferase 1(ESCO1)
M_77	125228	family with sequence similarity 210 member
M_77	23253	ankyrin repeat domain 12(ANKRD12)
M_77	8780	RIO kinase 3(RIOK3)
M_77	64762	GRB2 associated regulator of MAPK1 subtype
M_77	6093	Rho associated coiled-coil containing protein kinase 1(ROCK1)
M_77	125488	tetratricopeptide repeat domain 39C(TTC39C)
M_77	83539	carbohydrate sulfotransferase 9(CHST9)
M_77	728606	prostate cancer associated transcript 18 (non-protein coding)(PCAT18)
M_77	29919	chromosome 18 open reading frame 8(C18orf8)
M_77	51444	ring finger protein 138(RNF138)
M_77	55364	impact RWD domain protein(IMPACT)
M_77	3613	inositol monophosphatase 2(IMPA2)
M_77	100101467	zinc finger and SCAN domain containing

M_77	79959	centrosomal protein 76(CEP76)
M_77	8774	NSF attachment protein gamma(NAPG)
M_77	147429	AQP4 antisense RNA 1(AQP4-AS1)
M_77	361	aquaporin 4(AQP4)
M_77	55250	elongator acetyltransferase complex subunit 2(ELP2)
M_77	7572	zinc finger protein 24(ZNF24)
M_77	22878	trafficking protein particle complex 8(TRAPPC8)
M_77	85019	transmembrane protein 241(TMEM241)
M_77	65258	metallophosphoesterase 1(MPPE1)
M_77	10778	zinc finger protein 271, pseudogene(ZNF271P)
M_77	26256	calcium binding tyrosine phosphorylation
M_77	1829	desmoglein 2(DSG2)
M_77	6760	SS18, nBAF chromatin remodeling complex
M_77	252884	zinc finger protein 396(ZNF396)
M_77	56984	proteasome assembly chaperone 2(PSMG2)
M_77	57132	charged multivesicular body protein 1B(CHMP1B)
M_77	10939	AFG3 like matrix AAA peptidase subunit 2(AFG3L2)
M_77	8731	RNA guanine-7 methyltransferase(RNMT)
M_77	379013	ring finger protein 138 pseudogene 1(RNF138P1)
M_77	25800	solute carrier family 39 member 6(SLC39A6)
M_77	10928	ralA binding protein 1(RALBP1)
M_77	114876	oxysterol binding protein like 1A(OSBPL1A)
M_77	9989	protein phosphatase 4 regulatory subunit 1(PPP4R1)
M_77	25941	tubulin polyglutamylase complex subunit 2(TPGS2)
M_77	55556	enolase superfamily member 1(ENOSF1)
M_77	201475	RAB12, member RAS oncogene family(RAB12)
M_77	2891	glutamate ionotropic receptor AMPA type subunit
M_77	81563	chromosome 1 open reading frame 21(C1orf21)
M_77	23127	collagen beta(1-O)galactosyltransferase
M_77	9097	ubiquitin specific peptidase 14(USP14)
M_77	4729	NADH:ubiquinone oxidoreductase core subunit
M_77	57536	KIAA1328(KIAA1328)

M_77	57534	mindbomb E3 ubiquitin protein ligase 1(MIB1)
M_77	7525	YES proto-oncogene 1, Src family tyrosine
M_77	5577	protein kinase cAMP-dependent type II regulatory subunit beta(PRKAR2B)
M_77	238	anaplastic lymphoma receptor tyrosine kinase(ALK)
M_77	9984	THO complex 1(THOC1)
M_77	125476	INO80 complex subunit C(INO80C)
M_77	54941	ring finger protein 125(RNF125)
M_77	2589	polypeptide N-acetylgalactosaminyltransferase
M_77	6632	small nuclear ribonucleoprotein D1
M_77	170692	ADAM metallopeptidase with thrombospondin type 1 motif 18(ADAMTS18)
M_77	162655	zinc finger protein 519(ZNF519)
M_77	9663	lipin 2(LPIN2)
M_77	56853	CUGBP, Elav-like family member 4(CELF4)
M_77	23347	structural maintenance of chromosomes flexible hinge domain containing 1(SMCHD1)
M_77	64863	methyltransferase like 4(METTL4)
M_77	57045	twisted gastrulation BMP signaling modulator
M_77	83608	chromosome 18 open reading frame 21(C18orf21)
M_77	8736	myomesin 1(MYOM1)
M_77	127841	long intergenic non-protein coding RNA
M_78	653247	proline rich protein BstNI subfamily 2(PRB2)
M_78	6005	Rh-associated glycoprotein(RHAG)
M_78	440040	glutamate metabotropic receptor 5
M_78	53940	ferritin heavy chain like 17(FTHL17)
M_78	89870	tripartite motif containing 15(TRIM15)
M_78	642265	#N/A
M_78	9947	MAGE family member C1(MAGEC1)
M_78	135398	chromosome 6 open reading frame 141(C6orf141)
M_78	23746	aryl hydrocarbon receptor interacting protein like
M_78	727905	SPATA31 subfamily A member 5(SPATA31A5)

M_78	139420	protein phosphatase 4 regulatory subunit 3C, pseudogene(PPP4R3CP)
M_78	158809	MAGE family member B6(MAGEB6)
M_78	286499	family with sequence similarity 133 member
M_78	286514	MAGE family member B18(MAGEB18)
M_78	2560	gamma-aminobutyric acid type A receptor beta1 subunit(GABRB1)
M_78	389860	PAGE family member 2B(PAGE2B)
M_78	167	cysteine rich secretory protein 1(CRISP1)
M_78	3375	islet amyloid polypeptide(IAPP)
M_78	90737	PAGE family member 5(PAGE5)
M_78	1791	DNA nucleotidylexotransferase(DNTT)
M_78	441452	SPATA31 subfamily C member 1(SPATA31C1)
M_78	139425	DDB1 and CUL4 associated factor 8 like
M_78	139422	MAGE family member B10(MAGEB10)
M_78	346171	ZFP57 zinc finger protein(ZFP57)
M_78	90316	TGFB induced factor homeobox 2 like, X-
M_78	3859	keratin 12(KRT12)
M_78	84779	N(alpha)-acetyltransferase 11, NatA catalytic subunit(NAA11)
M_78	727830	SPATA31 subfamily A member 3(SPATA31A3)
M_78	54514	DEAD-box helicase 4(DDX4)
M_78	643311	cancer/testis antigen family 47, member B1(CT47B1)
M_78	342035	gliomedin(GLDN)
M_78	389730	SPATA31 subfamily A member 6(SPATA31A6)
M_78	6370	C-C motif chemokine ligand 25(CCL25)
M_78	728090	cancer/testis antigen family 47, member A2(CT47A2)
M_78	219487	olfactory receptor family 5 subfamily M member
M_78	728042	cancer/testis antigen family 47, member A9(CT47A9)
M_78	5545	proline rich protein BstNI subfamily 4(PRB4)
M_78	221391	opsin 5(OPN5)
M_78	5542	proline rich protein BstNI subfamily 1(PRB1)

M_78	168400	DEAD-box helicase 53(DDX53)
M_78	100128811	GPR158 antisense RNA 1(GPR158-AS1)
M_78	10599	solute carrier organic anion transporter family member 1B1(SLCO1B1)
M_78	132204	synaptoporin(SYNPR)
M_78	10107	tripartite motif containing 10(TRIM10)
M_78	203074	protease, serine 55(PRSS55)
M_78	219445	olfactory receptor family 7 subfamily E member 5 pseudogene(OR7E5P)
M_78	203569	PAGE family member 2(PAGE2)
M_78	27133	potassium voltage-gated channel subfamily H member 5(KCNH5)
M_78	51438	MAGE family member C2(MAGEC2)
M_78	4112	MAGE family member B1(MAGEB1)
M_78	4113	MAGE family member B2(MAGEB2)
M_78	137970	unc-5 netrin receptor D(UNC5D)
M_78	138009	DDB1 and CUL4 associated factor 4 like
M_78	203413	cancer/testis antigen 83(CT83)
M_78	4105	MAGE family member A6(MAGEA6)
M_78	4102	MAGE family member A3(MAGEA3)
M_78	7180	cysteine rich secretory protein 2(CRISP2)
M_78	4111	MAGE family member A12(MAGEA12)
M_78	4340	myelin oligodendrocyte glycoprotein(MOG)
M_78	89882	tumor protein D52 like 3(TPD52L3)
M_78	161357	MAM domain containing glycosylphosphatidylinositol anchor 2(MDGA2)
M_78	153218	serine peptidase inhibitor, Kazal type 13
M_78	84302	transmembrane protein 246(TMEM246)
M_78	158724	family with sequence similarity 47 member
M_78	5232	phosphoglycerate kinase 2(PGK2)
M_78	139628	forkhead box R2(FOXR2)

M_78	338821	solute carrier organic anion transporter family member 1B7 (putative)(SLCO1B7)
M_78	28234	solute carrier organic anion transporter family member 1B3(SLCO1B3)
M_78	4037	LDL receptor related protein 3(LRP3)
M_78	1588	cytochrome P450 family 19 subfamily A member 1(CYP19A1)
M_78	10886	neuropeptide FF receptor 2(NPFFR2)
M_78	26280	interleukin 1 receptor accessory protein like
M_78	401135	synaptotagmin 14 pseudogene 1(SYT14P1)
M_78	254773	lysozyme g2(LYG2)
M_78	158511	chondrosarcoma associated gene 1(CSAG1)
M_78	728461	#N/A
M_78	79047	potassium channel tetramerization domain containing 15(KCTD15)
M_78	1268	cannabinoid receptor 1(CNR1)
M_78	124	alcohol dehydrogenase 1A (class I), alpha polypeptide(ADH1A)
M_78	53342	interleukin 17D(IL17D)
M_78	2160	coagulation factor XI(F11)
M_78	11249	neurexophilin 2(NXPH2)
M_78	389903	CSAG family member 3(CSAG3)
M_78	9629	chloride channel accessory 3, pseudogene(CLCA3P)
M_78	389208	transmembrane protease, serine 11F(TMPRSS11F)
M_78	220047	coiled-coil domain containing 83(CCDC83)
M_78	1587	ADAM metallopeptidase domain 3A (pseudogene)(ADAM3A)
M_79	84647	phospholipase A2 group XIIB(PLA2G12B)
M_79	642623	upstream binding transcription factor, RNA polymerase I-like 1(UBTFL1)
M_79	50514	deleted in esophageal cancer 1(DEC1)
M_79	54600	UDP glucuronosyltransferase family 1 member

M_79	414926	long intergenic non-protein coding RNA
M_79	89869	phospholipase C zeta 1(PLCZ1)
M_79	127077	olfactory receptor family 2 subfamily T member 11 (gene/pseudogene)(OR2T11)
M_79	51350	keratin 76(KRT76)
M_79	80834	taste 1 receptor member 2(TAS1R2)
M_79	341277	ovochymase 2 (gene/pseudogene)(OVCH2)
M_79	389874	zinc finger CCHC-type containing 13(ZCCHC13)
M_79	390261	olfactory receptor family 6 subfamily M member
M_79	26496	olfactory receptor family 10 subfamily A member
M_79	127069	olfactory receptor family 2 subfamily T member
M_79	391211	olfactory receptor family 2 subfamily G member
M_79	54577	UDP glucuronosyltransferase family 1 member
M_79	338661	transmembrane protein 225(TMEM225)
M_79	157855	potassium calcium-activated channel subfamily U member 1(KCNU1)
M_79	641517	defensin beta 109 pseudogene 1B(DEFB109P1B)
M_79	343173	olfactory receptor family 2 subfamily T member
M_79	5407	pancreatic lipase related protein 1(PNLIPRP1)
M_79	54575	UDP glucuronosyltransferase family 1 member A10(UGT1A10)
M_79	54576	UDP glucuronosyltransferase family 1 member
M_79	353135	late cornified envelope 1E(LCE1E)
M_79	127064	olfactory receptor family 2 subfamily T member
M_79	127068	olfactory receptor family 2 subfamily T member
M_79	219873	olfactory receptor family 10 subfamily S member
M_79	219874	olfactory receptor family 6 subfamily T member
M_79	219875	olfactory receptor family 4 subfamily D member
M_79	677817	small nucleolar RNA, H/ACA box 36A(SNORA36A)
M_79	401993	olfactory receptor family 2 subfamily T member
M_79	403277	olfactory receptor family 5 subfamily K member
M_79	401992	olfactory receptor family 2 subfamily T member

M_79	338322	NLR family pyrin domain containing 10(NLRP10)
M_79	26533	olfactory receptor family 10 subfamily G member
M_79	120066	olfactory receptor family 5 subfamily P member
M_79	6473	short stature homeobox(SHOX)
M_79	338662	olfactory receptor family 8 subfamily D member
M_79	50833	taste 2 receptor member 16(TAS2R16)
M_79	26343	olfactory receptor family 5 subfamily E member 1 pseudogene(OR5E1P)
M_79	2488	follicle stimulating hormone beta subunit(FSHB)
M_79	390093	olfactory receptor family 10 subfamily A member 6 (gene/pseudogene)(OR10A6)
M_79	344901	osteocrin(OSTN)
M_79	137868	sarcoglycan zeta(SGCZ)
M_79	131920	transmembrane protein 207(TMEM207)
M_79	91768	Cdk5 and Abl enzyme substrate 1(CABLES1)
M_79	9942	xylulokinase(XYLB)
M_80	10048	RAN binding protein 9(RANBP9)
M_80	51567	tyrosyl-DNA phosphodiesterase 2(TDP2)
M_80	6745	signal sequence receptor subunit 1(SSR1)
M_80	51000	solute carrier family 35 member B3(SLC35B3)
M_80	57128	LYR motif containing 4(LYRM4)
M_80	81853	transmembrane protein 14B(TMEM14B)
M_80	63915	biogenesis of lysosomal organelles complex 1 subunit 5(BLOC1S5)
M_80	51406	nucleolar protein 7(NOL7)
M_80	56897	Werner helicase interacting protein 1(WRNIP1)
M_80	55003	PAK1 interacting protein 1(PAK1IP1)
M_80	83732	RIO kinase 1(RIOK1)
M_80	84062	dystrobrevin binding protein 1(DTNBP1)
M_80	9972	nucleoporin 153(NUP153)
M_80	8899	pre-mRNA processing factor 4B(PRPF4B)
M_80	1832	desmoplakin(DSP)

M_80	9425	chromodomain Y-like(CDYL)
M_80	389362	proteasome assembly chaperone 4(PSMG4)
M_80	404220	chromosome 6 open reading frame 201(C6orf201)
M_80	1871	E2F transcription factor 3(E2F3)
M_80	81688	chromosome 6 open reading frame 62(C6orf62)
M_80	23408	sirtuin 5(SIRT5)
M_80	55856	acyl-CoA thioesterase 13(ACOT13)
M_80	221710	small integral membrane protein 13(SMIM13)
M_80	221656	lysine demethylase 1B(KDM1B)
M_80	10455	enoyl-CoA delta isomerase 2(ECI2)
M_80	10667	phenylalanyl-tRNA synthetase 2,
M_80	7913	DEK proto-oncogene(DEK)
M_80	55770	exocyst complex component 2(EXOC2)
M_80	10473	high mobility group nucleosomal binding domain
M_80	2762	GDP-mannose 4,6-dehydratase(GMDS)
M_80	9521	eukaryotic translation elongation factor 1 epsilon
M_80	670	biphenyl hydrolase like(BPHL)
M_80	8737	receptor interacting serine/threonine kinase 1(RIPK1)
M_80	63933	mitochondrial calcium uniporter regulator 1(MCUR1)
M_80	51439	family with sequence similarity 8 member
M_80	10799	ribonuclease P/MRP subunit p40(RPP40)
M_80	154007	small nuclear ribonucleoprotein U11/U12 subunit 48(SNRNP48)
M_80	51256	TBC1 domain family member 7(TBC1D7)
M_80	51522	transmembrane protein 14C(TMEM14C)
M_80	57380	MRS2, magnesium transporter(MRS2)
M_80	54901	CDK5 regulatory subunit associated protein 1 like 1(CDKAL1)
M_80	10475	tripartite motif containing 38(TRIM38)
M_80	401232	long intergenic non-protein coding RNA
M_80	7726	tripartite motif containing 26(TRIM26)
M_80	7172	thiopurine S-methyltransferase(TPMT)

M_80	55604	capping protein regulator and myosin 1 linker
M_80	51053	geminin, DNA replication inhibitor(GMNN)
M_80	56940	dual specificity phosphatase 22(DUSP22)
M_80	3077	hemochromatosis(HFE)
M_80	154141	membrane bound O-acyltransferase domain containing 1(MBOAT1)
M_80	221749	PX domain containing 1(PXDC1)
M_81	643834	pepsinogen 3, group I (pepsinogen A)(PGA3)
M_81	9361	lon peptidase 1, mitochondrial(LONP1)
M_81	85462	FH2 domain containing 1(FHDC1)
M_81	5627	protein S (alpha)(PROS1)
M_81	57662	calmodulin regulated spectrin associated protein family member 3(CAMSAP3)
M_81	3643	insulin receptor(INSR)
M_81	353219	kidney associated antigen 1(KAAG1)
M_81	8498	RAN binding protein 3(RANBP3)
M_81	79230	zinc finger protein 557(ZNF557)
M_81	23529	cardiotrophin-like cytokine factor 1(CLCF1)
M_81	200894	ADP ribosylation factor like GTPase 13B(ARL13B)
M_81	79085	solute carrier family 25 member 23(SLC25A23)
M_81	200895	dihydrofolate reductase 2(DHFR2)
M_81	9411	Rho GTPase activating protein 29(ARHGAP29)
M_81	126003	trafficking protein particle complex 5(TRAPPC5)
M_81	51293	CD320 molecule(CD320)
M_81	643847	pepsinogen 4, group I (pepsinogen A)(PGA4)
M_81	374875	hydroxysteroid 11-beta dehydrogenase 1
M_81	56927	G protein-coupled receptor 108(GPR108)
M_81	2962	general transcription factor IIF subunit 1(GTF2F1)
M_81	80763	spexin hormone(SPX)
M_81	80131	leucine rich repeat containing 8 family member
M_81	6813	syntaxin binding protein 2(STXBP2)
M_81	140467	zinc finger protein 358(ZNF358)

M_81	126006	Purkinje cell protein 2(PCP2)
M_81	344787	zinc finger protein 860(ZNF860)
M_81	5222	pepsinogen 5, group I (pepsinogen A)(PGA5)
M_81	23596	opsin 3(OPN3)
M_81	23370	Rho/Rac guanine nucleotide exchange factor
M_81	9230	RAB11B, member RAS oncogene family(RAB11B)
M_81	4298	MLLT1, super elongation complex subunit(MLLT1)
M_81	8570	KH-type splicing regulatory protein(KHSRP)
M_81	24	ATP binding cassette subfamily A member 4(ABCA4)
M_81	63899	NOP2/Sun RNA methyltransferase family member 3(NSUN3)
M_81	404217	cortexin 1(CTXN1)
M_81	221584	zinc finger and SCAN domain containing 12 pseudogene 1(ZSCAN12P1)
M_81	729830	family with sequence similarity 160 member
M_81	5609	mitogen-activated protein kinase kinase 7(MAP2K7)
M_81	9322	thyroid hormone receptor interactor 10(TRIP10)
M_81	7718	zinc finger protein 165(ZNF165)
M_81	114884	oxysterol binding protein like 10(OSBPL10)
M_81	10469	translocase of inner mitochondrial membrane
M_81	56931	dihydrouridine synthase 3 like(DUS3L)
M_81	92960	peroxisomal biogenesis factor 11 gamma(PEX11G)
M_81	4701	NADH:ubiquinone oxidoreductase subunit
M_81	51473	doublecortin domain containing 2(DCDC2)
M_81	57192	mucolipin 1(MCOLN1)
M_81	10908	patatin like phospholipase domain containing
M_81	1544	cytochrome P450 family 1 subfamily A member
M_81	6618	small nuclear RNA activating complex polypeptide 2(SNAPC2)
M_81	1994	ELAV like RNA binding protein 1(ELAVL1)
M_81	51257	membrane associated ring-CH-type finger

M_81	400673	vimentin-type intermediate filament associated coiled-coil protein(VMAC)
M_81	56949	XPA binding protein 2(XAB2)
M_81	115704	ecotropic viral integration site 5 like(EVI5L)
M_81	84330	zinc finger protein 414(ZNF414)
M_81	29968	phosphoserine aminotransferase 1(PSAT1)
M_81	163154	proline rich 22(PRR22)
M_81	199800	adrenomedullin 5 (putative)(ADM5)
M_81	128025	WD repeat domain 64(WDR64)
M_81	1907	endothelin 2(EDN2)
M_81	8412	breast cancer anti-estrogen resistance 3(BCAR3)
M_81	2998	glycogen synthase 2(GYS2)
M_81	25975	EGF like domain multiple 6(EGFL6)
M_81	3598	interleukin 13 receptor subunit alpha 2(IL13RA2)
M_82	1746	distal-less homeobox 2(DLX2)
M_82	1745	distal-less homeobox 1(DLX1)
M_82	192669	argonaute 3, RISC catalytic component(AGO3)
M_82	1503	CTP synthase 1(CTPS1)
M_82	79753	Smad nuclear interacting protein 1(SNIP1)
M_82	441531	phosphoglycerate mutase family member 4(PGAM4)
M_82	8497	PTPRF interacting protein alpha 4(PPFIA4)
M_82	6676	sperm associated antigen 4(SPAG4)
M_82	648791	protein phosphatase 1 regulatory subunit
M_82	10487	adenylate cyclase associated protein 1(CAP1)
M_82	54955	chromosome 1 open reading frame 109(C1orf109)
M_82	1103	choline O-acetyltransferase(CHAT)
M_82	282966	chromosome 10 open reading frame 53(C10orf53)
M_82	11247	neurexophilin 4(NXPH4)
M_82	64769	MYST/Esa1 associated factor 6(MEAF6)
M_82	5210	6-phosphofructo-2-kinase/fructose-2,6-biphosphatase 4(PFKFB4)

M_82	56901	NDUFA4, mitochondrial complex associated like 2(NDUFA4L2)
M_82	4802	nuclear transcription factor Y subunit gamma(NFYC)
M_82	64789	exonuclease 5(EXO5)
M_82	29889	G protein nucleolar 2(GNL2)
M_82	26292	MYC binding protein(MYCBP)
M_82	10946	splicing factor 3a subunit 3(SF3A3)
M_82	5223	phosphoglycerate mutase 1(PGAM1)
M_82	54802	tRNA isopentenyltransferase 1(TRIT1)
M_82	6018	rearranged L-myc fusion(RLF)
M_82	664	BCL2 interacting protein 3(BNIP3)
M_82	10269	zinc metallopeptidase STE24(ZMPSTE24)
M_82	5538	palmitoyl-protein thioesterase 1(PPT1)
M_82	127396	zinc finger protein 684(ZNF684)
M_82	3099	hexokinase 2(HK2)
M_82	83931	serine/threonine kinase 40(STK40)
M_82	6513	solute carrier family 2 member 1(SLC2A1)
M_82	79647	akirin 1(AKIRIN1)
M_82	768	carbonic anhydrase 9(CA9)
M_82	55818	lysine demethylase 3A(KDM3A)
M_82	10450	peptidylprolyl isomerase E(PPIE)
M_82	8140	solute carrier family 7 member 5(SLC7A5)
M_82	5163	pyruvate dehydrogenase kinase 1(PDK1)
M_82	148523	circadian associated repressor of
M_82	64121	Ras related GTP binding C(RRAGC)
M_82	2026	enolase 2(ENO2)
M_82	6572	solute carrier family 18 member A3(SLC18A3)
M_82	51118	UTP11, small subunit processome component homolog (S. cerevisiae)(UTP11)
M_82	8614	stanniocalcin 2(STC2)
M_82	8761	poly(A) binding protein cytoplasmic 4(PABPC4)
M_82	339559	ZFP69 zinc finger protein(ZFP69)

M_82	80307	fer-1 like family member 4, pseudogene(FER1L4)
M_82	57761	tribbles pseudokinase 3(TRIB3)
M_82	5836	phosphorylase, glycogen, liver(PYGL)
M_82	4821	NK2 homeobox 2(NKX2-2)
M_82	9518	growth differentiation factor 15(GDF15)
M_82	7422	vascular endothelial growth factor A(VEGFA)
M_82	4725	NADH:ubiquinone oxidoreductase subunit
M_82	64744	small ArfGAP2(SMAP2)
M_82	4610	v-myc avian myelocytomatosis viral oncogene lung carcinoma derived homolog(MYCL)
M_82	5033	prolyl 4-hydroxylase subunit alpha 1(P4HA1)
M_82	145447	abhydrolase domain containing 12B(ABHD12B)
M_82	27294	dihydrodiol dehydrogenase(DHDH)
M_82	387911	C1q and tumor necrosis factor related protein 9B(C1QTNF9B)
M_82	79693	yrdC N6-threonylcarbamoyltransferase domain containing(YRDC)
M_82	55700	MAP7 domain containing 1(MAP7D1)
M_82	79924	adrenomedullin 2(ADM2)
M_82	10739	ret finger protein like 2(RFPL2)
M_82	8974	prolyl 4-hydroxylase subunit alpha 2(P4HA2)
M_82	338872	C1q and tumor necrosis factor related protein
M_82	55586	myo-inositol oxygenase(MIOX)
M_82	55521	tripartite motif containing 36(TRIM36)
M_82	542767	C1QTNF9B antisense RNA 1(C1QTNF9B-AS1)
M_82	80115	BAI1 associated protein 2 like 2(BAIAP2L2)
M_82	9672	syndecan 3(SDC3)
M_82	140803	transient receptor potential cation channel subfamily M member 6(TRPM6)
M_83	388818	keratin associated protein 26-1(KRTAP26-1)
M_83	219986	olfactory receptor family 4 subfamily D member

M_83	390756	olfactory receptor family 3 subfamily A member 4 pseudogene(OR3A4P)
M_83	6192	ribosomal protein S4, Y-linked 1(RPS4Y1)
M_83	728395	testis specific protein, Y-linked 4(TSPY4)
M_83	8387	olfactory receptor family 1 subfamily E member
M_83	8388	olfactory receptor family 1 subfamily E member
M_83	339779	proline rich 30(PRR30)
M_83	8653	DEAD-box helicase 3, Y-linked(DDX3Y)
M_83	340094	long intergenic non-protein coding RNA
M_83	8287	ubiquitin specific peptidase 9, Y-linked(USP9Y)
M_83	64595	testis-specific transcript, Y-linked 15 (non-protein coding)(TTTY15)
M_83	10798	olfactory receptor family 5 subfamily I member
M_83	26476	olfactory receptor family 10 subfamily J member
M_83	9086	eukaryotic translation initiation factor 1A, Y-
M_83	9087	thymosin beta 4, Y-linked(TMSB4Y)
M_83	80316	protein phosphatase 1 regulatory inhibitor subunit 2 pseudogene 9(PPP1R2P9)
M_83	246126	taxilin gamma pseudogene, Y-linked(TXLNGY)
M_83	386683	keratin associated protein 12-3(KRTAP12-3)
M_83	84663	#N/A
M_83	7299	tyrosinase(TYR)
M_83	27437	heat shock transcription factor, Y-linked 1 pseudogene 1(HSFY1P1)
M_83	401665	olfactory receptor family 51 subfamily T member
M_83	283598	chromosome 14 open reading frame 177(C14orf177)
M_83	8392	olfactory receptor family 3 subfamily A member
M_83	8390	olfactory receptor family 1 subfamily G member
M_83	336	apolipoprotein A2(APOA2)
M_83	4994	olfactory receptor family 3 subfamily A member
M_83	255220	thioredoxin domain containing 8(TXNDC8)
M_83	4995	olfactory receptor family 3 subfamily A member

M_83	286554	BCL6 corepressor pseudogene 1(BCORP1)
M_83	390436	olfactory receptor family 4 subfamily K member
M_83	128372	olfactory receptor family 6 subfamily N member
M_83	121364	olfactory receptor family 10 subfamily A member
M_83	390443	ribonuclease A family member 9 (inactive)(RNASE9)
M_83	128366	olfactory receptor family 6 subfamily P member
M_83	390038	olfactory receptor family 51 subfamily D member
M_83	219965	olfactory receptor family 5 subfamily B member
M_83	219438	olfactory receptor family 5 subfamily D member
M_83	2765	glycosylphosphatidylinositol anchored molecule
M_83	6906	serpin family A member 7(SERPINA7)
M_83	26333	olfactory receptor family 7 subfamily A member
M_83	254786	olfactory receptor family 6 subfamily C member
M_83	84690	spermatogenesis associated 22(SPATA22)
M_83	26658	olfactory receptor family 7 subfamily C member
M_83	256148	olfactory receptor family 4 subfamily S member
M_83	732	complement C8 beta chain(C8B)
M_83	731	complement C8 alpha chain(C8A)
M_83	54070	#N/A
M_83	245802	membrane spanning 4-domains A6E(MS4A6E)
M_83	727677	cancer susceptibility candidate 8 (non-protein
M_83	220081	glutamate rich 6B(ERICH6B)
M_83	387266	keratin associated protein 5-3(KRTAP5-3)
M_83	6290	serum amyloid A3 pseudogene(SAA3P)
M_83	100124537	small nucleolar RNA, H/ACA box 70B(SNORA70B)
M_83	7101	nuclear receptor subfamily 2 group E member
M_83	341152	olfactory receptor family 2 subfamily AT member
M_83	442229	solute carrier family 25 member 51 pseudogene 1(SLC25A51P1)
M_83	3047	hemoglobin subunit gamma 1(HBG1)
M_83	402135	olfactory receptor family 5 subfamily K member
M_83	10876	epididymal protein 3A(EDDM3A)

M_83	221044	upper zone of growth plate and cartilage matrix associated(UCMA)
M_83	51475	calcium binding protein 2(CABP2)
M_83	3048	hemoglobin subunit gamma 2(HBG2)
M_83	64184	epididymal protein 3B(EDDM3B)
M_83	153918	zinc finger C2HC-type containing 1B(ZC2HC1B)
M_83	389090	olfactory receptor family 6 subfamily B member
M_83	339345	nanos C2HC-type zinc finger 2(NANOS2)
M_83	23519	acidic nuclear phosphoprotein 32 family member
M_83	391627	ubiquitin specific peptidase 17-like family member 9, pseudogene(USP17L9P)
M_83	391622	ubiquitin specific peptidase 17-like family member 6, pseudogene(USP17L6P)
M_83	285627	uncharacterized LOC285627(LOC285627)
M_84	221002	RasGEF domain family member 1A(RASGEF1A)
M_84	54537	family with sequence similarity 35 member
M_84	285313	immunoglobulin superfamily member 10(IGSF10)
M_84	119385	ArfGAP with GTPase domain, ankyrin repeat and PH domain 11(AGAP11)
M_84	344752	arylacetamide deacetylase like 2(AADACL2)
M_84	3373	hyaluronoglucosaminidase 1(HYAL1)
M_84	79812	multimerin 2(MMRN2)
M_84	201651	arylacetamide deacetylase pseudogene 1(AADACP1)
M_84	132228	leucine rich single-pass membrane protein
M_84	26085	kallikrein related peptidase 13(KLK13)
M_84	203328	sushi domain containing 3(SUSD3)
M_84	1510	cathepsin E(CTSE)
M_84	3234	homeobox D8(HOXD8)
M_84	3235	homeobox D9(HOXD9)
M_84	3232	homeobox D3(HOXD3)
M_84	3233	homeobox D4(HOXD4)
M_84	3231	homeobox D1(HOXD1)

M_84	10974	adipogenesis regulatory factor(ADIRF)
M_84	13	arylacetamide deacetylase(AADAC)
M_84	414241	family with sequence similarity 35, member A pseudogene(FAM35BP)
M_84	393	Rho GTPase activating protein 4(ARHGAP4)
M_84	5650	kallikrein related peptidase 7(KLK7)
M_84	5653	kallikrein related peptidase 6(KLK6)
M_84	115019	solute carrier family 26 member 9(SLC26A9)
M_84	43849	kallikrein related peptidase 12(KLK12)
M_84	439965	family with sequence similarity 35, member A pseudogene(FAM35DP)
M_84	219527	leucine rich repeat containing 55(LRRC55)
M_84	8424	gamma-butyrobetaine hydroxylase 1(BBOX1)
M_84	554	arginine vasopressin receptor 2(AVPR2)
M_84	284366	kallikrein related peptidase 9(KLK9)
M_84	55816	docking protein 5(DOK5)
M_84	6623	synuclein gamma(SNCG)
M_84	2747	glutamate dehydrogenase 2(GLUD2)
M_84	2746	glutamate dehydrogenase 1(GLUD1)
M_84	11012	kallikrein related peptidase 11(KLK11)
M_84	80704	solute carrier family 19 member 3(SLC19A3)
M_84	11202	kallikrein related peptidase 8(KLK8)
M_84	3292	hydroxysteroid 17-beta dehydrogenase 1(HSD17B1)
M_84	5655	kallikrein related peptidase 10(KLK10)
M_84	43847	kallikrein related peptidase 14(KLK14)
M_84	5370	pro-melanin concentrating hormone like 2 (pseudogene)(PMCHL2)
M_84	5369	pro-melanin concentrating hormone like 1 (pseudogene)(PMCHL1)
M_84	285489	docking protein 7(DOK7)
M_84	6445	sarcoglycan gamma(SGCG)
M_84	6098	ROS proto-oncogene 1, receptor tyrosine

M_84	5794	protein tyrosine phosphatase, receptor type H(PTPRH)
M_84	445	argininosuccinate synthase 1(ASS1)
M_84	90634	NEDD4 binding protein 2 like 1(N4BP2L1)
M_84	10257	ATP binding cassette subfamily C member 4(ABCC4)
M_84	3897	L1 cell adhesion molecule(L1CAM)
M_84	27023	forkhead box B1(FOXB1)
M_84	1010	cadherin 12(CDH12)
M_84	374383	natural killer cell cytotoxicity receptor 3 ligand
M_84	285761	discoidin, CUB and LCCL domain containing
M_84	22977	aldo-keto reductase family 7 member A3(AKR7A3)
M_85	54581	SCAN domain containing 2 pseudogene(SCAND2P)
M_85	55898	unc-45 myosin chaperone A(UNC45A)
M_85	4205	myocyte enhancer factor 2A(MEF2A)
M_85	348110	actin-related protein 2/3 complex inhibitor(ARPIN)
M_85	5428	DNA polymerase gamma, catalytic subunit(POLG)
M_85	84942	WD repeat domain 73(WDR73)
M_85	10239	adaptor related protein complex 3 sigma 2
M_85	220594	ubiquitin specific peptidase 32 pseudogene
M_85	80039	family with sequence similarity 106 member
M_85	123720	WAS protein homolog associated with actin, golgi membranes and microtubules(WHAMM)
M_85	100129396	family with sequence similarity 106 member C, pseudogene(FAM106CP)
M_85	53339	BTB domain containing 1(BTBD1)
M_85	94158	CMT1A duplicated region transcript 15 pseudogene 1(CDRT15P1)
M_85	162632	ubiquitin specific peptidase 32 pseudogene
M_85	348254	coiled-coil domain containing 144C, pseudogene(CCDC144CP)
M_85	123283	threonyl-tRNA synthetase like 2(TARSL2)
M_85	100101490	malignant T-cell amplified sequence 2,
M_85	390637	GDP-D-glucose phosphorylase 1(GDPGP1)

M_85	5151	phosphodiesterase 8A(PDE8A)
M_85	4122	mannosidase alpha class 2A member 2(MAN2A2)
M_85	79631	elongation factor like GTPase 1(EFL1)
M_85	55070	de-etiolated homolog 1 (Arabidopsis)(DET1)
M_85	80154	golgin A2 pseudogene 10(GOLGA2P10)
M_85	8800	peroxisomal biogenesis factor 11 alpha(PEX11A)
M_85	64784	CREB regulated transcription coactivator 3(CRTC3)
M_85	10509	semaphorin 4B(SEMA4B)
M_85	145748	LysM domain containing 4(LYSMD4)
M_85	284047	coiled-coil domain containing 144B
M_85	51335	neugrin, neurite outgrowth associated(NGRN)
M_85	647042	golgin A6 family-like 10(GOLGA6L10)
M_85	140460	ankyrin repeat and SOCS box containing 7(ASB7)
M_85	64927	tetratricopeptide repeat domain 23(TTC23)
M_85	9720	coiled-coil domain containing 144A(CCDC144A)
M_85	26276	VPS33B, late endosome and lysosome
M_85	9640	zinc finger protein 592(ZNF592)
M_85	440295	golgin A6 family-like 9(GOLGA6L9)
M_85	374650	golgin A6 family-like 5, pseudogene(GOLGA6L5P)
M_85	374655	zinc finger protein 710(ZNF710)
M_85	388165	ubiquitin conjugating enzyme E2 Q2 pseudogene 1(UBE2Q2P1)
M_85	2242	FES proto-oncogene, tyrosine kinase(FES)
M_85	55180	lines homolog 1(LINS1)
M_85	5045	furin, paired basic amino acid cleaving
M_85	8826	IQ motif containing GTPase activating protein
M_85	23184	mesoderm development candidate 2(MESDC2)
M_85	503640	arginine-fifty homeobox pseudogene 2(ARGFXP2)
M_85	64410	kelch like family member 25(KLHL25)
M_85	28997	PRO0611 protein(PRO0611)
M_85	59274	mesoderm development candidate 1(MESDC1)
M_85	388152	golgin A2 pseudogene 7(GOLGA2P7)

M_85	283687	ST20 antisense RNA 1(ST20-AS1)
M_85	91433	RCC1 domain containing 1(RCCD1)
M_85	4217	mitogen-activated protein kinase kinase kinase
M_85	342132	zinc finger protein 774(ZNF774)
M_85	64782	apoptosis enhancing nuclease(AEN)
M_85	400410	suppressor of tumorigenicity 20(ST20)
M_85	58489	abhydrolase domain containing 17C(ABHD17C)
M_85	28232	solute carrier organic anion transporter family member 3A1(SLCO3A1)
M_85	23766	GABA type A receptor associated protein like 3 pseudogene(GABARAPL3)
M_86	57446	NDRG family member 3(NDRG3)
M_86	90196	SYS1, golgi trafficking protein(SYS1)
M_86	7529	tyrosine 3-monooxygenase/tryptophan 5-monooxygenase activation protein beta(YWHAB)
M_86	90203	sorting nexin family member 21(SNX21)
M_86	90204	zinc finger SWIM-type containing 1(ZSWIM1)
M_86	55905	ring finger protein 114(RNF114)
M_86	149951	COMM domain containing 7(COMMD7)
M_86	26512	integrator complex subunit 6(INTS6)
M_86	23078	von Willebrand factor A domain containing 8(VWA8)
M_86	26747	NUFIP1, FMR1 interacting protein 1(NUFIP1)
M_86	112858	TP53 regulating kinase(TP53RK)
M_86	51098	intraflagellar transport 52(IFT52)
M_86	5925	RB transcriptional corepressor 1(RB1)
M_86	81617	calcium binding protein 39 like(CAB39L)
M_86	51614	ERGIC and golgi 3(ERGIC3)
M_86	29079	mediator complex subunit 4(MED4)
M_86	22862	fibronectin type III domain containing 3A(FNDC3A)
M_86	51028	vacuolar protein sorting 36 homolog(VPS36)
M_86	388796	small nucleolar RNA host gene 17(SNHG17)
M_86	84078	kelch repeat and BTB domain containing 7(KBTBD7)

M_86	55425	GPALPP motifs containing 1(GPALPP1)
M_86	128869	phosphatidylinositol glycan anchor biosynthesis class
M_86	81550	tudor domain containing 3(TDRD3)
M_86	25980	AAR2 splicing factor homolog(AAR2)
M_86	55213	RCC1 and BTB domain containing protein
M_86	57511	component of oligomeric golgi complex 6(COG6)
M_86	2963	general transcription factor IIF subunit 2(GTF2F2)
M_86	57727	nuclear receptor coactivator 5(NCOA5)
M_86	23143	leucine rich repeats and calponin homology domain containing 1(LRCH1)
M_86	140831	zinc finger SWIM-type containing 3(ZSWIM3)
M_86	116092	deoxynucleotidyltransferase terminal interacting protein 1(DNTTIP1)
M_86	10240	mitochondrial ribosomal protein S31(MRPS31)
M_86	9617	mitochondrial translation release factor 1(MTRF1)
M_86	374500	thrombospondin type 1 domain containing 1 pseudogene 1(THSD1P1)
M_86	10208	ubiquitin specific peptidase like 1(USPL1)
M_86	63905	mannosidase beta like(MANBAL)
M_86	89890	kelch repeat and BTB domain containing 6(KBTBD6)
M_86	8480	ribonucleic acid export 1(RAE1)
M_86	3692	eukaryotic translation initiation factor 6(EIF6)
M_86	10206	tripartite motif containing 13(TRIM13)
M_86	5994	regulatory factor X associated protein(RFXAP)
M_86	8904	copine 1(CPNE1)
M_86	9054	NFS1, cysteine desulfurase(NFS1)
M_86	100	adenosine deaminase(ADA)
M_86	83548	component of oligomeric golgi complex 3(COG3)
M_86	51526	oxidative stress responsive serine rich 1(OSER1)
M_86	8813	dolichyl-phosphate mannosyltransferase subunit 1, catalytic(DPM1)
M_86	11215	A-kinase anchoring protein 11(AKAP11)

M_86	80209	proline and serine rich 1(PROSER1)
M_86	6431	serine and arginine rich splicing factor 6(SRSF6)
M_86	51569	ubiquitin fold modifier 1(UFM1)
M_86	79913	ARP5 actin-related protein 5 homolog(ACTR5)
M_86	9675	TELO2 interacting protein 1(TTI1)
M_86	51654	CDK5 regulatory subunit associated protein
M_86	10443	NEDD4 binding protein 2 like 2(N4BP2L2)
M_86	128439	small nucleolar RNA host gene 11(SNHG11)
M_86	128486	fat storage inducing transmembrane protein 2(FITM2)
M_86	160857	coiled-coil domain containing 122(CCDC122)
M_86	8848	TSC22 domain family member 1(TSC22D1)
M_86	58490	regulation of nuclear pre-mRNA domain containing 1B(RPRD1B)
M_86	10005	acyl-CoA thioesterase 8(ACOT8)
M_86	1102	RCC1 and BTB domain containing protein
M_86	9445	integral membrane protein 2B(ITM2B)
M_86	55741	ER degradation enhancing alpha-mannosidase like protein 2(EDEM2)
M_86	83852	SET domain bifurcated 2(SETDB2)
M_86	79612	N(alpha)-acetyltransferase 16, NatA auxiliary subunit(NAA16)
M_86	81572	p53 and DNA damage regulated 1(PDRG1)
M_86	8803	succinate-CoA ligase ADP-forming beta
M_86	11193	WW domain binding protein 4(WBP4)
M_86	253512	solute carrier family 25 member 30(SLC25A30)
M_86	283507	SGT1 homolog, MIS12 kinetochore complex assembly cochaperone pseudogene 3(SUGT1P3)
M_86	1997	E74 like ETS transcription factor 1(ELF1)
M_86	115825	WD repeat and FYVE domain containing 2(WDFY2)
M_86	51006	solute carrier family 35 member C2(SLC35C2)
M_86	55245	ubiquinol-cytochrome c reductase complex assembly factor 1(UQCC1)

M_86	3839	karyopherin subunit alpha 3(KPNA3)
M_86	387923	stress associated endoplasmic reticulum protein family member 2(SERP2)
M_86	387921	NHL repeat containing 3(NHLRC3)
M_86	4752	NIMA related kinase 3(NEK3)
M_86	11142	protein kinase (cAMP-dependent, catalytic) inhibitor gamma(PKIG)
M_86	23091	zinc finger CCCH-type containing 13(ZC3H13)
M_86	56259	catenin beta like 1(CTNNBL1)
M_86	1477	cleavage stimulation factor subunit 1(CSTF1)
M_86	7335	ubiquitin conjugating enzyme E2 V1(UBE2V1)
M_86	27304	molybdenum cofactor synthesis 3(MOCS3)
M_86	9724	UTP14, small subunit processome component homolog C (S. cerevisiae)(UTP14C)
M_86	63935	PDX1 C-terminal inhibiting factor 1(PCIF1)
M_86	387521	transmembrane protein 189(TMEM189)
M_86	81602	cytidine and dCMP deaminase domain containing 1(CDADC1)
M_86	51507	replication termination factor 2 domain containing
M_86	60625	DEAH-box helicase 35(DHX35)
M_86	10953	translocase of outer mitochondrial membrane
M_86	10955	serine incorporator 3(SERINC3)
M_86	55578	SPT20 homolog, SAGA complex
M_86	57463	adhesion molecule with Ig like domain 1(AMIGO1)
M_86	57148	Ral GTPase activating protein non-catalytic beta subunit(RALGAPB)
M_86	83737	itchy E3 ubiquitin protein ligase(ITCH)
M_86	51230	PHD finger protein 20(PHF20)
M_86	140825	neuralized E3 ubiquitin protein ligase 2(NEURL2)
M_86	22919	microtubule associated protein RP/EB family member 1(MAPRE1)
M_86	647979	non-coding RNA activated by DNA damage(NORAD)

M_86	7150	topoisomerase (DNA) I(TOP1)
M_86	2937	glutathione synthetase(GSS)
M_86	26133	transient receptor potential cation channel subfamily C member 4 associated protein(TRPC4AP)
M_86	9047	SH2 domain containing 2A(SH2D2A)
M_86	7151	topoisomerase (DNA) I pseudogene 1(TOP1P1)
M_86	60436	TGFB induced factor homeobox 2(TGIF2)
M_86	9334	beta-1,4-galactosyltransferase 5(B4GALT5)
M_86	6185	ribophorin II(RPN2)
M_86	5770	protein tyrosine phosphatase, non-receptor type
M_86	51604	phosphatidylinositol glycan anchor biosynthesis class
M_86	22839	DLG associated protein 4(DLGAP4)
M_86	128497	spermatogenesis associated 25(SPATA25)
M_86	23315	solute carrier family 9 member A8(SLC9A8)
M_86	191	adenosylhomocysteinase(AHCY)
M_86	55861	dysbindin domain containing 2(DBNDD2)
M_86	55170	protein arginine methyltransferase 6(PRMT6)
M_87	131601	transmembrane protein adipocyte associated
M_87	27032	ATPase secretory pathway Ca2+ transporting
M_87	79876	ubiquitin like modifier activating enzyme 5(UBA5)
M_87	285282	RAB, member of RAS oncogene family like
M_87	4172	minichromosome maintenance complex component
M_87	25852	armadillo repeat containing 8(ARMC8)
M_87	10063	COX17, cytochrome c oxidase copper
M_87	51163	debranching RNA lariats 1(DBR1)
M_87	22820	coatomer protein complex subunit gamma 1(COPG1)
M_87	7879	RAB7A, member RAS oncogene family(RAB7A)
M_87	7555	CCHC-type zinc finger nucleic acid binding
M_87	23023	transmembrane and coiled-coil domain family
M_87	54931	tRNA methyltransferase 10C, mitochondrial RNase P subunit(TRMT10C)
M_87	2932	glycogen synthase kinase 3 beta(GSK3B)

M_87	55840	ELL associated factor 2(EAF2)
M_87	54554	WD repeat domain 5B(WDR5B)
M_87	7029	transcription factor Dp-2(TFDP2)
M_87	4710	NADH:ubiquinone oxidoreductase subunit
M_87	51300	translocase of inner mitochondrial membrane domain containing 1(TIMMDC1)
M_87	8930	methyl-CpG binding domain 4, DNA
M_87	64422	autophagy related 3(ATG3)
M_87	6184	ribophorin I(RPN1)
M_87	11222	mitochondrial ribosomal protein L3(MRPL3)
M_87	54946	solute carrier family 41 member 3(SLC41A3)
M_87	25871	nucleolus and neural progenitor protein(NEPRO)
M_87	29083	GTP binding protein 8 (putative)(GTPBP8)
M_87	7372	uridine monophosphate synthetase(UMPS)
M_87	9657	IQ motif containing B1(IQCB1)
M_87	6259	receptor-like tyrosine kinase(RYK)
M_87	8723	sorting nexin 4(SNX4)
M_87	55773	TBC1 domain family member 23(TBC1D23)
M_87	1371	coproporphyrinogen oxidase(CPOX)
M_87	8607	RuvB like AAA ATPase 1(RUVBL1)
M_87	80321	centrosomal protein 70(CEP70)
M_87	56983	protein O-glucosyltransferase 1(POGLUT1)
M_87	80218	N(alpha)-acetyltransferase 50, NatE catalytic subunit(NAA50)
M_87	29927	Sec61 translocon alpha 1 subunit(SEC61A1)
M_87	116064	leucine rich repeat containing 58(LRRC58)
M_87	9532	BCL2 associated athanogene 2(BAG2)
M_87	131076	coiled-coil domain containing 58(CCDC58)
M_87	8702	beta-1,4-galactosyltransferase 4(B4GALT4)
M_87	26984	SEC22 homolog A, vesicle trafficking
M_87	79663	HSPB1 associated protein 1(HSPBAP1)
M_87	9868	translocase of outer mitochondrial membrane

M_87	84925	disrupted in renal carcinoma 2(DIRC2)
M_87	7707	zinc finger protein 148(ZNF148)
M_87	375513	glucuronidase, beta pseudogene 4(GUSBP4)
M_87	11073	topoisomerase (DNA) II binding protein 1(TOPBP1)
M_87	4690	NCK adaptor protein 1(NCK1)
M_87	5096	propionyl-CoA carboxylase beta subunit(PCCB)
M_87	55032	solute carrier family 35 member A5(SLC35A5)
M_87	56941	5-hydroxymethylcytosine (hmC) binding, ES cell-specific(HMCES)
M_87	9276	coatomer protein complex subunit beta 2(COPB2)
M_87	56945	mitochondrial ribosomal protein S22(MRPS22)
M_87	523	ATPase H+ transporting V1 subunit A(ATP6V1A)
M_87	57461	ISY1 splicing factor homolog(ISY1)
M_87	3836	karyopherin subunit alpha 1(KPNA1)
M_87	51715	RAB23, member RAS oncogene family(RAB23)
M_87	84319	cms1 ribosomal small subunit homolog
M_87	56954	nitrilase family member 2(NIT2)
M_87	84864	MYC induced nuclear antigen(MINA)
M_87	5558	primase (DNA) subunit 2(PRIM2)
M_87	55167	male-specific lethal 2 homolog (Drosophila)(MSL2)
M_87	60678	eukaryotic elongation factor, selenocysteine-tRNA specific(EEFSEC)
M_87	5523	protein phosphatase 2 regulatory subunit
M_87	55166	centromere protein Q(CENPQ)
M_87	55254	transmembrane protein 39A(TMEM39A)
M_87	79364	ZXD family zinc finger C(ZXDC)
M_87	131870	nudix hydrolase 16(NUDT16)
M_87	26036	zinc finger protein 451(ZNF451)
M_87	23317	DnaJ heat shock protein family (Hsp40) member C13(DNAJC13)
M_87	28976	acyl-CoA dehydrogenase family member 9(ACAD9)
M_87	28978	transmembrane protein 14A(TMEM14A)

M_87	57092	PEST proteolytic signal containing nuclear
M_87	114885	oxysterol binding protein like 11(OSBPL11)
M_87	80254	centrosomal protein 63(CEP63)
M_87	10342	TRK-fused gene(TFG)
M_87	28990	asteroid homolog 1 (Drosophila)(ASTE1)
M_87	56650	claudin domain containing 1(CLDND1)
M_87	30849	phosphoinositide-3-kinase regulatory subunit
M_87	55573	CDV3 homolog(CDV3)
M_87	55347	abhydrolase domain containing 10(ABHD10)
M_87	26355	family with sequence similarity 162 member
M_87	2960	general transcription factor IIE subunit 1(GTF2E1)
M_87	57691	KIAA1586(KIAA1586)
M_87	152185	spindle and centriole associated protein 1(SPICE1)
M_87	201562	3-hydroxyacyl-CoA dehydratase 2(HACD2)
M_87	84303	coiled-coil-helix-coiled-coil-helix domain containing 6(CHCHD6)
M_87	60481	ELOVL fatty acid elongase 5(ELOVL5)
M_87	55179	Fas apoptotic inhibitory molecule(FAIM)
M_87	339942	H1FX antisense RNA 1(H1FX-AS1)
M_87	6478	siah E3 ubiquitin protein ligase 2(SIAH2)
M_87	1302	collagen type XI alpha 2 chain(COL11A2)
M_87	79691	queuine tRNA-ribosyltransferase accessory subunit 2(QTRT2)
M_87	22848	AP2 associated kinase 1(AAK1)
M_87	483	ATPase Na+/K+ transporting subunit beta 3(ATP1B3)
M_87	8971	H1 histone family member X(H1FX)
M_87	79669	chromosome 3 open reading frame 52(C3orf52)
M_87	254887	zinc finger DHHC-type containing 23(ZDHHC23)
M_87	8521	glial cells missing homolog 1(GCM1)
M_88	23210	arginine demethylase and lysine hydroxylase(JMJD6)
M_88	284207	meteorin like, glial cell differentiation

M_88	23510	potassium channel tetramerization domain containing 2(KCTD2)
M_88	5635	phosphoribosyl pyrophosphate synthetase associated protein 1(PRPSAP1)
M_88	23558	WW domain binding protein 2(WBP2)
M_88	6730	signal recognition particle 68(SRP68)
M_88	91107	tripartite motif containing 47(TRIM47)
M_88	23580	CDC42 effector protein 4(CDC42EP4)
M_88	57003	coiled-coil domain containing 47(CCDC47)
M_88	9382	component of oligomeric golgi complex 1(COG1)
M_88	23163	golgi associated, gamma adaptin ear containing, ARF binding protein 3(GGA3)
M_88	11322	transmembrane channel like 6(TMC6)
M_88	201266	solute carrier family 39 member 11(SLC39A11)
M_88	283989	tRNA splicing endonuclease subunit 54(TSEN54)
M_88	283987	HID1 domain containing(HID1)
M_88	84923	family with sequence similarity 104 member
M_88	79637	armadillo repeat containing 7(ARMC7)
M_88	283991	UBA like domain containing 2(UBALD2)
M_88	9400	RecQ like helicase 5(RECQL5)
M_88	10458	BAI1 associated protein 2(BAIAP2)
M_88	2885	growth factor receptor bound protein 2(GRB2)
M_88	3959	galectin 3 binding protein(LGALS3BP)
M_88	26151	N-acetyltransferase 9 (putative)(NAT9)
M_88	57409	MIF4G domain containing(MIF4GD)
M_88	29115	SAP30 binding protein(SAP30BP)
M_88	1534	cytochrome b561(CYB561)
M_88	23265	exocyst complex component 7(EXOC7)
M_88	30833	5', 3'-nucleotidase, cytosolic(NT5C)
M_88	60386	solute carrier family 25 member 19(SLC25A19)
M_88	6603	SWI/SNF related, matrix associated, actin dependent regulator of chromatin, subfamily d, member

M_88	9144	synaptogyrin 2(SYNGR2)
M_88	79902	nucleoporin 85(NUP85)
M_88	100134934	TEN1, CST complex subunit(TEN1)
M_88	51	acyl-CoA oxidase 1(ACOX1)
M_88	2584	galactokinase 1(GALK1)
M_88	79651	rhomboid 5 homolog 2(RHBDF2)
M_88	201292	tripartite motif containing 65(TRIM65)
M_88	30850	cerebellar degeneration related protein 2 like(CDR2L)
M_88	9121	solute carrier family 16 member 5(SLC16A5)
M_88	3021	H3 histone family member 3B(H3F3B)
M_88	11221	dual specificity phosphatase 10(DUSP10)
M_88	8165	A-kinase anchoring protein 1(AKAP1)
M_88	201294	unc-13 homolog D(UNC13D)
M_88	85302	Fas binding factor 1(FBF1)
M_88	353174	zinc activated ion channel(ZACN)
M_88	63910	solute carrier family 17 member 9(SLC17A9)
M_88	2696	gastric inhibitory polypeptide receptor(GIPR)
M_89	165055	coiled-coil domain containing 138(CCDC138)
M_89	23332	cytoplasmic linker associated protein 1(CLASP1)
M_89	57669	erythrocyte membrane protein band 4.1 like
M_89	29986	solute carrier family 39 member 2(SLC39A2)
M_89	1615	aspartyl-tRNA synthetase(DARS)
M_89	23505	transmembrane protein 131(TMEM131)
M_89	84365	nucleolar protein interacting with the FHA domain of MKI67(NIFK)
M_89	83607	AMMECR1 like(AMMECR1L)
M_89	55339	WD repeat domain 33(WDR33)
M_89	64965	mitochondrial ribosomal protein S9(MRPS9)
M_89	84220	RANBP2-like and GRIP domain containing
M_89	84083	zinc finger RANBP2-type containing 3(ZRANB3)
M_89	79074	chromosome 2 open reading frame 49(C2orf49)
M_89	8626	tumor protein p63(TP63)

M_89	9648	GRIP and coiled-coil domain containing 2(GCC2)
M_89	151525	WD repeat, sterile alpha motif and U-box domain containing 1(WDSUB1)
M_89	55289	acyl-CoA oxidase-like(ACOXL)
M_89	5899	RAS like proto-oncogene B(RALB)
M_89	64844	membrane associated ring-CH-type finger
M_89	151011	septin 10(SEPT10)
M_89	54520	coiled-coil domain containing 93(CCDC93)
M_89	23190	UBX domain protein 4(UBXN4)
M_89	10254	signal transducing adaptor molecule 2(STAM2)
M_89	55183	replication timing regulatory factor 1(RIF1)
M_89	200576	phosphoinositide kinase, FYVE-type zinc finger containing(PIKFYVE)
M_89	5937	RNA binding motif single stranded interacting protein 1(RBMS1)
M_89	57406	abhydrolase domain containing 6(ABHD6)
M_89	84826	SFT2 domain containing 3(SFT2D3)
M_89	130827	transmembrane protein 182(TMEM182)
M_89	5433	RNA polymerase II subunit D(POLR2D)
M_89	285190	RANBP2-like and GRIP domain containing
M_89	55677	IWS1, SUPT6H interacting protein(IWS1)
M_89	84524	zinc finger CCCH-type containing 8(ZC3H8)
M_89	729540	RANBP2-like and GRIP domain containing
M_89	55777	methyl-CpG binding domain protein 5(MBD5)
M_89	64682	anaphase promoting complex subunit 1(ANAPC1)
M_89	2071	ERCC excision repair 3, TFIIH core complex helicase subunit(ERCC3)
M_89	7247	translin(TSN)
M_89	65124	sosondowah ankyrin repeat domain family member C(SOWAHC)
M_89	8886	DEAD-box helicase 18(DDX18)
M_89	25801	grancalcin(GCA)

M_89	10096	ARP3 actin related protein 3 homolog(ACTR3)
M_89	5775	protein tyrosine phosphatase, non-receptor type
M_89	124590	USH1 protein network component sans(USH1G)
M_89	92736	otopetrin 2(OTOP2)
M_89	653489	RANBP2-like and GRIP domain containing
M_89	285386	tumor protein p63 regulated 1(TPRG1)
M_89	79134	transmembrane protein 185B(TMEM185B)
M_89	65267	WNK lysine deficient protein kinase 3(WNK3)
M_89	339745	speckle type BTB/POZ protein like(SPOPL)
M_89	84804	major facilitator superfamily domain containing
M_89	51141	insulin induced gene 2(INSIG2)
M_89	283985	fatty acid desaturase 6(FADS6)
M_89	8440	NCK adaptor protein 2(NCK2)
M_89	84172	RNA polymerase I subunit B(POLR1B)
M_89	80146	UDP-glucuronate decarboxylase 1(UXS1)
M_89	55571	CCR4-NOT transcription complex subunit
M_89	54891	INO80 complex subunit D(INO80D)
M_89	400966	RANBP2-like and GRIP domain containing
M_89	26122	enhancer of polycomb homolog 2(EPC2)
M_89	151188	ADP ribosylation factor like GTPase 6 interacting protein 6(ARL6IP6)
M_89	2232	ferredoxin reductase(FDXR)
M_89	3631	inositol polyphosphate-4-phosphatase type I
M_89	84771	DEAD/H-box helicase 11 like 2(DDX11L2)
M_89	118433	ribosomal protein L23a pseudogene 7(RPL23AP7)
M_89	389072	pleckstrin homology domain containing
M_89	5563	protein kinase AMP-activated catalytic subunit alpha 2(PRKAA2)
M_89	23518	R3H domain containing 1(R3HDM1)
M_89	79595	Sin3A associated protein 130(SAP130)
M_89	130074	family with sequence similarity 168 member
M_89	160518	DENN domain containing 5B(DENND5B)

M_89	29994	bromodomain adjacent to zinc finger domain
M_89	22930	RAB3 GTPase activating protein catalytic subunit 1(RAB3GAP1)
M_89	5000	origin recognition complex subunit 4(ORC4)
M_89	56886	UDP-glucose glycoprotein glucosyltransferase
M_89	55660	pre-mRNA processing factor 40 homolog
M_89	80255	solute carrier family 35 member F5(SLC35F5)
M_89	2001	E74 like ETS transcription factor 5(ELF5)
M_89	9635	chloride channel accessory 2(CLCA2)
M_89	55627	sphingomyelin phosphodiesterase 4(SMPD4)
M_89	150776	sphingomyelin phosphodiesterase 4, neutral membrane (neutral sphingomyelinase-3)
M_89	5903	RAN binding protein 2(RANBP2)
M_89	905	cyclin T2(CCNT2)
M_89	26225	ADP ribosylation factor like GTPase 5A(ARL5A)
M_89	10018	BCL2 like 11(BCL2L11)
M_89	80775	transmembrane protein 177(TMEM177)
M_89	375346	transmembrane protein 110(TMEM110)
M_89	678	ZFP36 ring finger protein like 2(ZFP36L2)
M_89	9099	ubiquitin specific peptidase 2(USP2)
M_89	114793	formin like 2(FMNL2)
M_89	150472	COBW domain containing 2(CBWD2)
M_89	445571	COBW domain containing 3(CBWD3)
M_90	146556	chromosome 16 open reading frame 89(C16orf89)
M_90	5154	platelet derived growth factor subunit A(PDGFA)
M_90	117153	#N/A
M_90	54546	ring finger protein 186(RNF186)
M_90	1917	eukaryotic translation elongation factor 1 alpha
M_90	389634	long intergenic non-protein coding RNA
M_90	6519	solute carrier family 3 member 1(SLC3A1)
M_90	353091	retinoic acid early transcript 1G(RAET1G)
M_90	5267	serpin family A member 4(SERPINA4)

M_90	154661	RUN domain containing 3B(RUNDC3B)
M_90	27190	interleukin 17B(IL17B)
M_90	163778	small proline rich protein 4(SPRR4)
M_90	9245	glucosaminyl (N-acetyl) transferase 3, mucin
M_90	3785	potassium voltage-gated channel subfamily Q member 2(KCNQ2)
M_90	6699	small proline rich protein 1B(SPRR1B)
M_90	5618	prolactin receptor(PRLR)
M_90	81033	potassium voltage-gated channel subfamily H member 6(KCNH6)
M_90	58189	WAP four-disulfide core domain 1(WFDC1)
M_90	115677	nitric oxide synthase trafficking(NOSTRIN)
M_90	2243	fibrinogen alpha chain(FGA)
M_90	140828	long intergenic non-protein coding RNA
M_90	3814	KiSS-1 metastasis-suppressor(KISS1)
M_90	6927	HNF1 homeobox A(HNF1A)
M_90	51816	cat eye syndrome chromosome region, candidate
M_90	284656	EPH receptor A10(EPHA10)
M_90	155465	anterior gradient 3, protein disulphide isomerase family member(AGR3)
M_90	283460	HNF1A antisense RNA 1(HNF1A-AS1)
M_90	9914	ATPase secretory pathway Ca2+ transporting
M_90	127845	golgi transport 1A(GOLT1A)
M_90	7032	trefoil factor 2(TFF2)
M_90	7031	trefoil factor 1(TFF1)
M_90	11033	ArfGAP with dual PH domains 1(ADAP1)
M_90	7033	trefoil factor 3(TFF3)
M_90	1555	cytochrome P450 family 2 subfamily B member
M_90	2918	glutamate metabotropic receptor 8(GRM8)
M_90	27112	family with sequence similarity 155 member
M_90	151742	protein phosphatase, Mg2+/Mn2+ dependent
M_90	3081	homogentisate 1,2-dioxygenase(HGD)

M_90	28231	solute carrier organic anion transporter family member 4A1(SLCO4A1)
M_90	286	ankyrin 1(ANK1)
M_90	7429	villin 1(VIL1)
M_90	222183	serine/arginine repetitive matrix 3(SRRM3)
M_90	27075	tetraspanin 13(TSPAN13)
M_90	3881	keratin 31(KRT31)
M_90	140730	regulating synaptic membrane exocytosis 4(RIMS4)
M_90	256472	transmembrane protein 151A(TMEM151A)
M_90	10551	anterior gradient 2, protein disulphide isomerase family member(AGR2)
M_90	4923	neurotensin receptor 1(NTSR1)
M_90	27159	chitinase, acidic(CHIA)
M_90	100127888	SLCO4A1 antisense RNA 1(SLCO4A1-AS1)
M_90	84918	LDL receptor related protein 11(LRP11)
M_90	100130238	uncharacterized LOC100130238(LOC100130238)
M_90	79605	piggyBac transposable element derived 5(PGBD5)
M_90	10570	dihydropyrimidinase like 4(DPYSL4)
M_90	3172	hepatocyte nuclear factor 4 alpha(HNF4A)
M_90	3170	forkhead box A2(FOXA2)
M_90	6700	small proline rich protein 2A(SPRR2A)
M_90	6703	small proline rich protein 2D(SPRR2D)
M_90	6707	small proline rich protein 3(SPRR3)
M_90	9429	ATP binding cassette subfamily G member 2 (Junior blood group)(ABCG2)
M_90	50614	polypeptide N-acetylgalactosaminyltransferase
M_90	53630	beta-carotene oxygenase 1(BCO1)
M_90	1896	ectodysplasin A(EDA)
M_90	5166	pyruvate dehydrogenase kinase 4(PDK4)
M_90	1113	chromogranin A(CHGA)
M_90	149175	mannosidase endo-alpha like(MANEAL)
M_90	5243	ATP binding cassette subfamily B member 1(ABCB1)

M_90	6469	sonic hedgehog(SHH)
M_90	51365	phospholipase A1 member A(PLA1A)
M_90	388021	transmembrane protein 179(TMEM179)
M_90	6698	small proline rich protein 1A(SPRR1A)
M_90	2244	fibrinogen beta chain(FGB)
M_90	725	complement component 4 binding protein
M_90	254427	proline and serine rich 2(PROSER2)
M_90	4014	loricrin(LOR)
M_90	6690	serine peptidase inhibitor, Kazal type 1(SPINK1)
M_90	5408	pancreatic lipase related protein 2 (gene/pseudogene)(PNLIPRP2)
M_90	6317	serpin family B member 3(SERPINB3)
M_90	6318	serpin family B member 4(SERPINB4)
M_90	4161	melanocortin 5 receptor(MC5R)
M_90	65266	WNK lysine deficient protein kinase 4(WNK4)
M_90	4477	microseminoprotein beta(MSMB)
M_90	4986	opioid receptor kappa 1(OPRK1)
M_90	55356	solute carrier family 22 member 15(SLC22A15)
M_90	3885	keratin 34(KRT34)
M_90	3280	hes family bHLH transcription factor 1(HES1)
M_90	6571	solute carrier family 18 member A2(SLC18A2)
M_90	173	afamin(AFM)
M_90	174	alpha fetoprotein(AFP)
M_90	167127	UDP glycosyltransferase family 3 member
M_90	4300	MLLT3, super elongation complex subunit(MLLT3)
M_90	60437	cadherin 26(CDH26)
M_90	27347	serine/threonine kinase 39(STK39)
M_90	56155	testis expressed 14, intercellular bridge forming
M_90	284756	chromosome 20 open reading frame 197(C20orf197)
M_90	9333	transglutaminase 5(TGM5)
M_90	440253	WAS protein homolog associated with actin, golgi membranes and microtubules pseudogene

M_90	26	amine oxidase, copper containing 1(AOC1)
M_90	2266	fibrinogen gamma chain(FGG)
M_90	26084	Rho guanine nucleotide exchange factor
M_90	196374	keratin 78(KRT78)
M_90	131034	copine 4(CPNE4)
M_90	10742	retinoic acid induced 2(RAI2)
M_90	374454	keratin 77(KRT77)
M_90	339005	WAS protein homolog associated with actin, golgi membranes and microtubules pseudogene
M_90	8687	keratin 38(KRT38)
M_90	133688	UDP glycosyltransferase family 3 member
M_90	126868	mab-21 like 3(MAB21L3)
M_90	163259	DENN domain containing 2C(DENND2C)
M_90	56907	spire type actin nucleation factor 1(SPIRE1)
M_90	84660	coiled-coil domain containing 62(CCDC62)
M_90	3882	keratin 32(KRT32)
M_90	245806	vestigial like family member 2(VGLL2)
M_90	2258	fibroblast growth factor 13(FGF13)
M_90	158696	long intergenic non-protein coding RNA
M_90	84203	thioredoxin domain containing 2(TXNDC2)
M_90	26659	olfactory receptor family 7 subfamily A member
M_91	23657	solute carrier family 7 member 11(SLC7A11)
M_91	282809	POC1 centriolar protein B(POC1B)
M_91	199974	cytochrome P450 family 4 subfamily Z member
M_91	29993	protein kinase C and casein kinase substrate in neurons 1(PACSIN1)
M_91	163720	cytochrome P450 family 4 subfamily Z member 2, pseudogene(CYP4Z2P)
M_91	124925	seizure related 6 homolog(SEZ6)
M_91	7499	Xg blood group(XG)
M_91	2850	G protein-coupled receptor 27(GPR27)
M_91	8534	carbohydrate sulfotransferase 1(CHST1)

M_91	9220	TGFB1-induced anti-apoptotic factor 1(TIAF1)
M_91	284415	V-set and transmembrane domain containing
M_91	284541	cytochrome P450 family 4 subfamily A member 22(CYP4A22)
M_91	5016	oviductal glycoprotein 1(OVGP1)
M_91	4147	matrilin 2(MATN2)
M_91	8693	polypeptide N-acetylgalactosaminyltransferase
M_91	96626	LIM zinc finger domain containing 3(LIMS3)
M_91	260293	cytochrome P450 family 4 subfamily X member
M_91	9699	regulating synaptic membrane exocytosis 2(RIMS2)
M_91	51301	glucosaminyl (N-acetyl) transferase 4, core 2(GCNT4)
M_91	27199	oxoglutarate receptor 1(OXGR1)
M_91	399687	myosin XVIIIA(MYO18A)
M_91	79084	WD repeat domain 77(WDR77)
M_91	60675	prokineticin 2(PROK2)
M_91	3680	integrin subunit alpha 9(ITGA9)
M_91	55638	syntabulin(SYBU)
M_91	8975	ubiquitin specific peptidase 13 (isopeptidase T-
M_91	374387	uncharacterized
M_91	57535	KIAA1324(KIAA1324)
M_91	80150	asparaginase like 1(ASRGL1)
M_91	866	serpin family A member 6(SERPINA6)
M_91	152273	FYVE, RhoGEF and PH domain containing 5(FGD5)
M_91	221184	copine 2(CPNE2)
M_91	3987	LIM zinc finger domain containing 1(LIMS1)
M_91	79899	proline rich 5 like(PRR5L)
M_91	10164	carbohydrate sulfotransferase 4(CHST4)
M_91	22824	heat shock protein family A (Hsp70) member 4
M_91	84281	chromosome 2 open reading frame 88(C2orf88)
M_91	100271835	LIMS3-LOC440895 readthrough(LIMS3-
M_91	317649	eukaryotic translation initiation factor 4E family member 3(EIF4E3)

M_91	4250	secretoglobin family 2A member 2(SCGB2A2)
M_91	23604	death associated protein kinase 2(DAPK2)
M_91	10648	secretoglobin family 1D member 1(SCGB1D1)
M_91	10647	secretoglobin family 1D member 2(SCGB1D2)
M_91	51268	pipecolic acid and sarcosine oxidase(PIPOX)
M_91	93035	polycystic kidney and hepatic disease 1 (autosomal recessive)-like 1(PKHD1L1)
M_91	9848	microfibrillar associated protein 3 like(MFAP3L)
M_91	4246	secretoglobin family 2A member 1(SCGB2A1)
M_91	64798	DEP domain containing MTOR-interacting
M_91	9071	claudin 10(CLDN10)
M_91	81501	dendrocyte expressed seven transmembrane protein(DCSTAMP)
M_91	25803	SAM pointed domain containing ETS transcription factor(SPDEF)
M_91	196883	adenylate cyclase 4(ADCY4)
M_91	9368	SLC9A3 regulator 1(SLC9A3R1)
M_91	51776	sterile alpha motif and leucine zipper containing kinase AZK(ZAK)
M_91	9154	solute carrier family 28 member 1(SLC28A1)
M_91	283849	exocyst complex component 3 like 1(EXOC3L1)
M_91	10753	calpain 9(CAPN9)
M_91	5105	phosphoenolpyruvate carboxykinase 1(PCK1)
M_91	8814	cyclin dependent kinase like 1(CDKL1)
M_91	92291	calpain 13(CAPN13)
M_91	30061	solute carrier family 40 member 1(SLC40A1)
M_91	23066	cullin associated and neddylation dissociated 2 (putative)(CAND2)
M_91	647309	geminin coiled-coil domain containing(GMNC)
M_91	53832	interleukin 20 receptor subunit alpha(IL20RA)
M_91	6533	solute carrier family 6 member 6(SLC6A6)

M_91	163732	Cbp/p300 interacting transactivator with Glu/Asp rich carboxy-terminal domain 4(CITED4)
M_91	23305	acyl-CoA synthetase long-chain family member
M_91	3655	integrin subunit alpha 6(ITGA6)
M_91	51555	peroxisomal biogenesis factor 5 like(PEX5L)
M_91	6376	C-X3-C motif chemokine ligand 1(CX3CL1)
M_91	55605	kinesin family member 21A(KIF21A)
M_91	3708	inositol 1,4,5-trisphosphate receptor type 1(ITPR1)
M_92	641298	SMG1P1, nonsense mediated mRNA decay associated PI3K related kinase pseudogene 1(SMG1P1)
M_92	442590	speedy/RINGO cell cycle regulator family member E5(SPDYE5)
M_92	100170939	#N/A
M_92	389517	#N/A
M_92	89849	autophagy related 16 like 2(ATG16L2)
M_92	100272228	long intergenic non-protein coding RNA
M_92	283663	long intergenic non-protein coding RNA
M_92	29990	paired immunoglobin-like type 2 receptor
M_92	653188	glucuronidase, beta pseudogene 3(GUSBP3)
M_92	2796	gonadotropin releasing hormone 1(GNRH1)
M_92	1235	C-C motif chemokine receptor 6(CCR6)
M_92	728963	ribosomal protein S15a pseudogene 10(RPS15AP10)
M_92	4974	oligodendrocyte myelin glycoprotein(OMG)
M_92	79037	poliovirus receptor related immunoglobulin domain containing(PVRIG)
M_92	112476	proline rich transmembrane protein 2(PRRT2)
M_92	1018	cyclin dependent kinase 3(CDK3)
M_92	284618	RUSC1 antisense RNA 1(RUSC1-AS1)
M_92	285955	speedy/RINGO cell cycle regulator family member E1(SPDYE1)
M_92	1375	carnitine palmitoyltransferase 1B(CPT1B)
M_92	283234	coiled-coil domain containing 88B(CCDC88B)

M_92	402483	long intergenic non-protein coding RNA
M_92	9256	TSPO associated protein 1(TSPOAP1)
M_92	440456	pleckstrin homology and RUN domain containing M1 pseudogene 1(PLEKHM1P1)
M_92	114823	leukocyte receptor cluster member 8(LENG8)
M_92	729597	speedy/RINGO cell cycle regulator family member E6(SPDYE6)
M_92	8473	O-linked N-acetylglucosamine (GlcNAc)
M_92	440352	sorting nexin 29 pseudogene 2(SNX29P2)
M_92	51380	cysteine sulfinic acid decarboxylase(CSAD)
M_92	8718	TNF receptor superfamily member 25(TNFRSF25)
M_92	80336	poly(A) binding protein cytoplasmic 1
M_92	378938	metastasis associated lung adenocarcinoma transcript 1 (non-protein coding)(MALAT1)
M_92	8831	synaptic Ras GTPase activating protein 1(SYNGAP1)
M_92	58496	lymphocyte antigen 6 complex, locus G5B(LY6G5B)
M_92	27099	SND1 intronic transcript 1(SND1-IT1)
M_92	3617	interphotoreceptor matrix proteoglycan 1(IMPG1)
M_92	646471	uncharacterized LOC646471(LOC646471)
M_92	146880	Rho GTPase activating protein 27
M_92	100190986	uncharacterized LOC100190986(LOC100190986)
M_92	22900	caspase recruitment domain family member
M_92	388591	ring finger protein 207(RNF207)
M_92	283131	nuclear paraspeckle assembly transcript 1 (non-protein coding)(NEAT1)
M_92	79970	zinc finger family member 767,
M_92	27040	linker for activation of T-cells(LAT)
M_92	285512	FAM13A antisense RNA 1(FAM13A-AS1)
M_92	26140	tubulin tyrosine ligase like 3(TTLL3)
M_92	2570	gamma-aminobutyric acid type A receptor rho2 subunit(GABRR2)
M_92	266747	ral guanine nucleotide dissociation stimulator like

M_92	25956	SEC31 homolog B, COPII coat complex
M_92	26013	l(3)mbt-like 1 (Drosophila)(L3MBTL1)
M_92	100131434	long intergenic non-protein coding RNA
M_92	80022	myosin XVB(MYO15B)
M_92	80035	ANP32A intronic transcript 1(ANP32A-IT1)
M_92	130872	AHA1, activator of heat shock 90kDa protein ATPase homolog 2 (yeast)(AHSA2)
M_92	29057	family with sequence similarity 156 member
M_92	9481	solute carrier family 25 member 27(SLC25A27)
M_92	4671	NLR family apoptosis inhibitory protein(NAIP)
M_92	23117	nuclear pore complex interacting protein family member B3(NPIPB3)
M_92	100132247	nuclear pore complex interacting protein family member B5(NPIPB5)
M_92	57449	pleckstrin homology and RhoGEF domain containing G5(PLEKHG5)
M_92	386593	CHKB-CPT1B readthrough (NMD candidate)(CHKB-CPT1B)
M_92	64599	GRB10 interacting GYF protein 1(GIGYF1)
M_92	100271836	SMG1P3, nonsense mediated mRNA decay associated PI3K related kinase pseudogene 3(SMG1P3)
M_92	177	advanced glycosylation end-product specific
M_92	55422	zinc finger protein 331(ZNF331)
M_92	64061	TSPY like 2(TSPYL2)
M_92	641649	transmembrane protein 91(TMEM91)
M_92	8552	inactivation escape 1 (non-protein coding)(INE1)
M_92	653635	WAS protein family homolog 7
M_92	440270	golgin A8 family member B(GOLGA8B)
M_92	9196	potassium voltage-gated channel subfamily A regulatory beta subunit 3(KCNAB3)
M_92	441273	speedy/RINGO cell cycle regulator family member E2(SPDYE2)

M_92	138724	chromosome 9 open reading frame 131(C9orf131)
M_92	400322	hect domain and RLD 2 pseudogene 2(HERC2P2)
M_92	79667	KLF3 antisense RNA 1(KLF3-AS1)
M_92	1241	leukotriene B4 receptor(LTB4R)
M_92	10110	SGK2, serine/threonine kinase 2(SGK2)
M_92	23015	golgin A8 family member A(GOLGA8A)
M_92	11126	CD160 molecule(CD160)
M_92	441457	NUT family member 2G(NUTM2G)
M_92	29881	NPC1 like intracellular cholesterol transporter
M_92	619190	farnesyl diphosphate synthase pseudogene 2(FDPSP2)
M_92	401105	uncharacterized LOC401105(FLJ42393)
M_92	54754	NUT family member 2F(NUTM2F)
M_92	375260	WAS protein family homolog 2
M_92	29053	uncharacterized LOC29053(PRO0628)
M_92	142686	ankyrin repeat and SOCS box containing 14(ASB14)
M_92	100113386	UCKL1 antisense RNA 1(UCKL1-AS1)
M_92	191585	placenta specific 4(PLAC4)
M_92	374666	WAS protein family homolog 3
M_92	56413	leukotriene B4 receptor 2(LTB4R2)
M_92	100288778	WAS protein family homolog 1
M_92	100302692	FTX transcript, XIST regulator (non-protein
M_92	441263	DTX2P1-UPK3BP1-PMS2P11 readthrough, transcribed pseudogene(DTX2P1-UPK3BP1-
M_92	375690	WAS protein family homolog 5
M_92	388951	TSPY like 6(TSPYL6)
M_92	286006	leucine rich single-pass membrane protein
M_93	54586	equatorin(EQTN)
M_93	51015	isochorismatase domain containing 1(ISOC1)
M_93	84675	tripartite motif containing 55(TRIM55)
M_93	195814	short chain dehydrogenase/reductase family 16C, member 5(SDR16C5)
M_93	6274	S100 calcium binding protein A3(S100A3)

M_93	6275	S100 calcium binding protein A4(S100A4)
M_93	6273	S100 calcium binding protein A2(S100A2)
M_93	5003	solute carrier family 22 member 18
M_93	10690	fucosyltransferase 9(FUT9)
M_93	2861	G protein-coupled receptor 37(GPR37)
M_93	222584	family with sequence similarity 83 member
M_93	123606	non imprinted in Prader-Willi/Angelman syndrome
M_93	91522	collagen type XXIII alpha 1 chain(COL23A1)
M_93	10656	KH RNA binding domain containing, signal transduction associated 3(KHDRBS3)
M_93	9982	fibroblast growth factor binding protein 1(FGFBP1)
M_93	3778	potassium calcium-activated channel subfamily M alpha 1(KCNMA1)
M_93	5874	RAB27B, member RAS oncogene family(RAB27B)
M_93	151647	family with sequence similarity 19 member A4, C-C motif chemokine like(FAM19A4)
M_93	6276	S100 calcium binding protein A5(S100A5)
M_93	54798	dachsous cadherin-related 2(DCHS2)
M_93	165679	serine palmitoyltransferase small subunit B(SPTSSB)
M_93	112597	cytoskeleton regulator RNA(CYTOR)
M_93	29948	oxidative stress induced growth inhibitor 1(OSGIN1)
M_93	28965	solute carrier family 27 member 6(SLC27A6)
M_93	53904	myosin IIIA(MYO3A)
M_93	222537	heparan sulfate-glucosamine 3-sulfotransferase
M_93	5002	solute carrier family 22 member 18(SLC22A18)
M_93	7262	pleckstrin homology like domain family A member 2(PHLDA2)
M_93	79817	MOB kinase activator 3B(MOB3B)
M_93	266722	heparan sulfate 6-O-sulfotransferase 3(HS6ST3)
M_93	152	adrenoceptor alpha 2C(ADRA2C)
M_93	8325	frizzled class receptor 8(FZD8)
M_93	56704	junctophilin 1(JPH1)

M_93	26257	NK2 homeobox 8(NKX2-8)
M_93	5081	paired box 7(PAX7)
M_93	4856	nephroblastoma overexpressed(NOV)
M_93	9200	3-hydroxyacyl-CoA dehydratase 1(HACD1)
M_93	4543	melatonin receptor 1A(MTNR1A)
M_93	8771	TNF receptor superfamily member 6b(TNFRSF6B)
M_93	541471	MIR4435-2 host gene(MIR4435-2HG)
M_93	80323	coiled-coil domain containing 68(CCDC68)
M_93	389658	family with sequence similarity 150 member
M_93	3956	galectin 1(LGALS1)
M_93	8744	tumor necrosis factor superfamily member 9(TNFSF9)
M_93	64409	Williams-Beuren syndrome chromosome region 17(WBSCR17)
M_93	2014	epithelial membrane protein 3(EMP3)
M_93	3174	hepatocyte nuclear factor 4 gamma(HNF4G)
M_93	134701	ripply transcriptional repressor 2(RIPPLY2)
M_93	345630	fibrillarin-like 1(FBLL1)
M_93	10752	cell adhesion molecule L1 like(CHL1)
M_93	412	steroid sulfatase (microsomal), isozyme S(STS)
M_93	1946	ephrin A5(EFNA5)
M_93	57221	ARFGEF family member 3(ARFGEF3)
M_93	134265	actin filament associated protein 1 like 1(AFAP1L1)
M_93	285613	RELT like 2(RELL2)
M_93	970	CD70 molecule(CD70)
M_93	5122	proprotein convertase subtilisin/kexin type 1(PCSK1)
M_93	59351	prostate and breast cancer overexpressed 1(PBOV1)
M_94	84656	glyoxylate reductase 1 homolog(GLYR1)
M_94	217	aldehyde dehydrogenase 2 family
M_94	342346	chromosome 16 open reading frame 96(C16orf96)
M_94	124056	NADPH oxidase organizer 1(NOXO1)
M_94	3718	Janus kinase 3(JAK3)

M_94	51172	N-acetylglucosamine-1-phosphodiester alpha-N-acetylglucosaminidase(NAGPA)
M_94	3386	intercellular adhesion molecule 4 (Landsteiner-Wiener blood group)(ICAM4)
M_94	6543	solute carrier family 8 member A2(SLC8A2)
M_94	5360	phospholipid transfer protein(PLTP)
M_94	161829	exonuclease 3'-5' domain containing 1(EXD1)
M_94	89780	Wnt family member 3A(WNT3A)
M_94	352909	dynein axonemal assembly factor 3(DNAAF3)
M_94	25837	RAB26, member RAS oncogene family(RAB26)
M_94	7138	troponin T1, slow skeletal type(TNNT1)
M_94	7137	troponin I3, cardiac type(TNNI3)
M_94	79641	rogdi homolog(ROGDI)
M_94	2906	glutamate ionotropic receptor NMDA type subunit 2D(GRIN2D)
M_94	7023	transcription factor AP-4(TFAP4)
M_94	57468	solute carrier family 12 member 5(SLC12A5)
M_94	254439	chromosome 11 open reading frame 86(C11orf86)
M_94	56052	ALG1, chitobiosyldiphosphodolichol beta-mannosyltransferase(ALG1)
M_94	51764	G protein subunit gamma 13(GNG13)
M_94	29965	cell death inducing p53 target 1(CDIP1)
M_94	26470	seizure related 6 homolog like 2(SEZ6L2)
M_94	57407	NmrA like redox sensor 1(NMRAL1)
M_94	9093	DnaJ heat shock protein family (Hsp40) member A3(DNAJA3)
M_94	83546	retbindin(RTBDN)
M_94	23149	FCH domain only 1(FCHO1)
M_94	29855	ubinuclein 1(UBN1)
M_94	147699	protein phosphatase, Mg2+/Mn2+ dependent 1N (putative)(PPM1N)
M_94	146433	interleukin 34(IL34)

M_94	23295	mahogunin ring finger 1(MGRN1)
M_94	342667	SH3 and cysteine rich domain 2(STAC2)
M_94	5373	phosphomannomutase 2(PMM2)
M_94	3640	insulin like 3(INSL3)
M_94	5091	pyruvate carboxylase(PC)
M_94	164633	calcium binding protein 7(CABP7)
M_94	253982	aspartate beta-hydroxylase domain containing
M_94	10131	TNF receptor associated protein 1(TRAP1)
M_94	51286	cell cycle exit and neuronal differentiation 1(CEND1)
M_94	196483	eukaryotic elongation factor 2 lysine methyltransferase(EEF2KMT)
M_94	23025	unc-13 homolog A(UNC13A)
M_94	124402	UBA like domain containing 1(UBALD1)
M_94	124401	ankyrin repeat and sterile alpha motif domain containing 3(ANKS3)
M_94	170393	chromosome 10 open reading frame 91(C10orf91)
M_94	9143	synaptogyrin 3(SYNGR3)
M_94	6253	reticulon 2(RTN2)
M_94	6861	synaptotagmin 5(SYT5)
M_94	6820	sulfotransferase family 2B member 1(SULT2B1)
M_94	126133	aldehyde dehydrogenase 16 family member
M_94	79091	methyltransferase like 22(METTL22)
M_94	114783	lemur tyrosine kinase 3(LMTK3)
M_94	7087	intercellular adhesion molecule 5(ICAM5)
M_94	26048	zinc finger protein 500(ZNF500)
M_94	3163	heme oxygenase 2(HMOX2)
M_94	25880	transmembrane protein 186(TMEM186)
M_94	134	adenosine A1 receptor(ADORA1)
M_94	79852	epoxide hydrolase 3(EPHX3)
M_94	60385	testis specific serine kinase substrate(TSKS)
M_94	79025	fibronectin type III domain containing 11(FNDC11)
M_94	4909	neurotrophin 4(NTF4)

M_94	57030	solute carrier family 17 member 7(SLC17A7)
M_94	1474	cystatin E/M(CST6)
M_94	930	CD19 molecule(CD19)
M_94	148170	CDC42 effector protein 5(CDC42EP5)
M_94	94027	chorionic gonadotropin beta subunit 7(CGB7)
M_94	375061	family with sequence similarity 89 member
M_94	148979	GLIS family zinc finger 1(GLIS1)
M_94	162461	transmembrane protein 92(TMEM92)
M_94	51676	ankyrin repeat and SOCS box containing 2(ASB2)
M_94	4004	LIM domain only 1(LMO1)
M_94	79585	coronin 7(CORO7)
M_94	5768	quiescin sulfhydryl oxidase 1(QSOX1)
M_94	6528	solute carrier family 5 member 5(SLC5A5)
M_94	3306	heat shock protein family A (Hsp70) member
M_94	343263	myosin binding protein H like(MYBPHL)
M_94	11036	general transcription factor IIA subunit 1
M_94	23415	potassium voltage-gated channel subfamily H member 4(KCNH4)
M_94	286749	STON1-GTF2A1L readthrough(STON1-GTF2A1L)
M_94	201305	sphingolipid transporter 3 (putative)(SPNS3)
M_94	164118	tetratricopeptide repeat domain 24(TTC24)
M_94	200058	uncharacterized protein FLJ23867(FLJ23867)
M_94	100130958	synaptonemal complex central element protein 1 like(SYCE1L)
M_94	389197	chromosome 4 open reading frame 50(C4orf50)
M_94	51733	beta-ureidopropionase 1(UPB1)
M_95	1748	distal-less homeobox 4(DLX4)
M_95	1747	distal-less homeobox 3(DLX3)
M_95	23551	RASD family member 2(RASD2)
M_95	3816	kallikrein 1(KLK1)
M_95	3817	kallikrein related peptidase 2(KLK2)
M_95	165257	complement C1q like 2(C1QL2)

M_95	90249	unc-5 netrin receptor A(UNC5A)
M_95	343637	R-spondin 4(RSPO4)
M_95	653190	ATP binding cassette subfamily C member 6 pseudogene 1(ABCC6P1)
M_95	1056	carboxyl ester lipase(CEL)
M_95	201232	solute carrier family 16 member 13(SLC16A13)
M_95	6750	somatostatin(SST)
M_95	6517	solute carrier family 2 member 4(SLC2A4)
M_95	7466	wolframin ER transmembrane glycoprotein(WFS1)
M_95	6939	transcription factor 15 (basic helix-loop-
M_95	6457	SH3 domain containing GRB2 like 3, endophilin
M_95	730013	ATP binding cassette subfamily C member 6 pseudogene 2(ABCC6P2)
M_95	140628	GATA binding protein 5(GATA5)
M_95	55450	calcium/calmodulin dependent protein kinase II inhibitor 1(CAMK2N1)
M_95	57338	junctophilin 3(JPH3)
M_95	645840	thioredoxin reductase 3 neighbor(TXNRD3NB)
M_95	255027	MPV17 mitochondrial inner membrane protein
M_95	5799	protein tyrosine phosphatase, receptor type
M_95	1852	dual specificity phosphatase 9(DUSP9)
M_95	166012	carbohydrate sulfotransferase 13(CHST13)
M_95	387267	keratin associated protein 5-4(KRTAP5-4)
M_95	115004	Mab-21 domain containing 1(MB21D1)
M_95	23504	RIMS binding protein 2(RIMBP2)
M_95	51087	Y-box binding protein 2(YBX2)
M_95	54221	syntrophin gamma 2(SNTG2)
M_95	128674	prokineticin receptor 2(PROKR2)
M_95	79887	phospholipase B domain containing 1(PLBD1)
M_95	368	ATP binding cassette subfamily C member 6(ABCC6)
M_95	63976	PR/SET domain 16(PRDM16)
M_95	9001	huntingtin associated protein 1(HAP1)

M_95	23237	activity regulated cytoskeleton associated
M_95	5522	protein phosphatase 2 regulatory subunit
M_95	1143	cholinergic receptor nicotinic beta 4
M_95	1136	cholinergic receptor nicotinic alpha 3
M_95	1138	cholinergic receptor nicotinic alpha 5
M_95	83723	family with sequence similarity 57 member
M_95	282973	Janus kinase and microtubule interacting protein
M_95	978	cytidine deaminase(CDA)
M_95	163933	family with sequence similarity 43 member
M_95	27087	beta-1,3-glucuronyltransferase 1(B3GAT1)
M_95	9622	kallikrein related peptidase 4(KLK4)
M_95	162515	solute carrier family 16 member 11(SLC16A11)
M_95	84258	synaptotagmin 3(SYT3)
M_95	575	adhesion G protein-coupled receptor B1(ADGRB1)
M_95	10439	olfactomedin 1(OLFM1)
M_95	3757	potassium voltage-gated channel subfamily H member 2(KCNH2)
M_95	387496	RAS like family 11 member A(RASL11A)
M_95	6545	solute carrier family 7 member 4(SLC7A4)
M_95	2301	forkhead box E3(FOXE3)
M_95	89927	chromosome 16 open reading frame 45(C16orf45)
M_95	200931	solute carrier family 51 alpha subunit(SLC51A)
M_95	125704	family with sequence similarity 69 member
M_95	10810	WAS protein family member 3(WASF3)
M_95	354	kallikrein related peptidase 3(KLK3)
M_95	55554	kallikrein related peptidase 15(KLK15)
M_95	1428	crystallin mu(CRYM)
M_95	131540	zinc finger DHHC-type containing 19(ZDHHC19)
M_95	84448	actin binding LIM protein family member 2(ABLIM2)
M_95	390928	acid phosphatase 7, tartrate resistant (putative)(ACP7)
M_95	492	ATPase plasma membrane Ca2+ transporting
M_95	440556	long intergenic non-protein coding RNA

M_95	220107	deleted in lymphocytic leukemia, 7(DLEU7)
M_95	150209	apoptosis inducing factor, mitochondria associated
M_95	54436	SH3 domain and tetratricopeptide repeats 1(SH3TC1)
M_95	284367	sialic acid binding Ig like lectin 17,
M_95	8120	adaptor related protein complex 3 beta 2
M_95	6854	synapsin II(SYN2)
M_95	100128239	uncharacterized LOC100128239(LOC100128239)
M_95	8448	double C2 domain alpha(DOC2A)
M_95	9127	purinergic receptor P2X 6(P2RX6)
M_95	57228	small cell adhesion glycoprotein(SMAGP)
M_95	63928	calcineurin like EF-hand protein 2(CHP2)
M_95	51083	galanin and GMAP prepropeptide(GAL)
M_95	256714	MAP7 domain containing 2(MAP7D2)
M_95	326	autoimmune regulator(AIRE)
M_95	50810	hepatoma-derived growth factor, related protein
M_95	654790	Purkinje cell protein 4 like 1(PCP4L1)
M_95	169044	collagen type XXII alpha 1 chain(COL22A1)
M_95	57464	striatin interacting protein 2(STRIP2)
M_95	57016	aldo-keto reductase family 1 member B10(AKR1B10)
M_95	84915	family with sequence similarity 222 member
M_95	157869	somatomedin B and thrombospondin type 1 domain containing(SBSPON)
M_95	55034	molybdenum cofactor sulfurase(MOCOS)
M_95	284076	tubulin tyrosine ligase like 6(TTLL6)
M_95	10021	hyperpolarization activated cyclic nucleotide gated potassium channel 4(HCN4)
M_95	1501	catenin delta 2(CTNND2)
M_95	441282	aldo-keto reductase family 1 member B15(AKR1B15)
M_95	9957	heparan sulfate-glucosamine 3-sulfotransferase
M_95	5443	proopiomelanocortin(POMC)
M_95	144423	glycosyltransferase 1 domain containing 1(GLT1D1)

M_95	3754	potassium voltage-gated channel modifier subfamily F member 1(KCNF1)
M_95	375607	N-acetyltransferase 16 (putative)(NAT16)
M_95	140625	actin related protein T2(ACTRT2)
M_95	59341	transient receptor potential cation channel subfamily V member 4(TRPV4)
M_95	341359	synaptotagmin 10(SYT10)
M_95	340348	tetraspanin 33(TSPAN33)
M_95	606293	kallikrein pseudogene 1(KLKP1)
M_95	126567	C2 calcium dependent domain containing
M_95	22999	regulating synaptic membrane exocytosis 1(RIMS1)
M_95	374569	asparaginase(ASPG)
M_95	1757	sarcosine dehydrogenase(SARDH)
M_95	140767	neurensin 1(NRSN1)
M_95	58157	neuroglobin(NGB)
M_95	116969	ADP-ribosyltransferase 5(ART5)
M_95	55224	ethanolamine kinase 2(ETNK2)
M_95	84433	caspase recruitment domain family member
M_95	25759	SHC adaptor protein 2(SHC2)
M_95	1621	dopamine beta-hydroxylase(DBH)
M_96	79877	dephospho-CoA kinase domain containing(DCAKD)
M_96	100133991	MAP3K14 antisense RNA 1(MAP3K14-AS1)
M_96	9862	mediator complex subunit 24(MED24)
M_96	51351	zinc finger protein 117(ZNF117)
M_96	90110	#N/A
M_96	90507	secernin 2(SCRN2)
M_96	51224	transcription elongation factor B subunit
M_96	4905	N-ethylmaleimide sensitive factor, vesicle fusing ATPase(NSF)
M_96	80279	CDK5 regulatory subunit associated protein
M_96	84197	protein-O-mannose kinase(POMK)
M_96	31	acetyl-CoA carboxylase alpha(ACACA)

M_96	23131	G-patch domain containing 8(GPATCH8)
M_96	6605	SWI/SNF related, matrix associated, actin dependent regulator of chromatin, subfamily e, member
M_96	8405	speckle type BTB/POZ protein(SPOP)
M_96	92591	ankyrin repeat and SOCS box containing 16(ASB16)
M_96	79089	transmembrane and ubiquitin like domain containing 2(TMUB2)
M_96	9043	sperm associated antigen 9(SPAG9)
M_96	56970	ataxin 7 like 3(ATXN7L3)
M_96	284058	KAT8 regulatory NSL complex subunit 1(KANSL1)
M_96	11143	lysine acetyltransferase 7(KAT7)
M_96	644172	mitogen-activated protein kinase 8 interacting protein 1 pseudogene(LOC644172)
M_96	9884	leucine rich repeat containing 37A(LRRC37A)
M_96	80347	Coenzyme A synthase(COASY)
M_96	80755	alanyl-tRNA synthetase domain containing
M_96	54799	mbt domain containing 1(MBTD1)
M_96	996	cell division cycle 27(CDC27)
M_96	124808	coiled-coil domain containing 43(CCDC43)
M_96	84961	F-box and leucine rich repeat protein 20(FBXL20)
M_96	8678	beclin 1(BECN1)
M_96	51326	ADP ribosylation factor like GTPase 17A(ARL17A)
M_96	2145	enhancer of zeste 1 polycomb repressive complex 2 subunit(EZH1)
M_96	11276	synergin gamma(SYNRG)
M_96	6668	Sp2 transcription factor(SP2)
M_96	22794	cancer susceptibility candidate 3(CASC3)
M_96	401884	#N/A
M_96	10014	histone deacetylase 5(HDAC5)
M_96	51755	cyclin dependent kinase 12(CDK12)
M_96	474170	leucine rich repeat containing 37 member
M_96	4836	N-myristoyltransferase 1(NMT1)

M_96	57551	TAO kinase 1(TAOK1)
M_96	55175	kelch like family member 11(KLHL11)
M_96	81558	family with sequence similarity 117 member
M_96	535	ATPase H+ transporting V0 subunit a1(ATP6V0A1)
M_96	4779	nuclear factor, erythroid 2 like 1(NFE2L1)
M_96	147179	WAS/WASL interacting protein family member
M_96	28511	NFKB inhibitor interacting Ras like 2(NKIRAS2)
M_96	147081	CRHR1 intronic transcript 1(CRHR1-IT1)
M_96	1659	DEAH-box helicase 8(DHX8)
M_96	4077	NBR1, autophagy cargo receptor(NBR1)
M_96	51747	LUC7 like 3 pre-mRNA splicing factor(LUC7L3)
M_96	163	adaptor related protein complex 2 beta 1
M_96	65264	ubiquitin conjugating enzyme E2 Z(UBE2Z)
M_96	6777	signal transducer and activator of transcription
M_96	5164	pyruvate dehydrogenase kinase 2(PDK2)
M_96	6774	signal transducer and activator of transcription
M_96	10197	proteasome activator subunit 3(PSME3)
M_96	5469	mediator complex subunit 1(MED1)
M_96	8148	TATA-box binding protein associated factor
M_96	114881	oxysterol binding protein like 7(OSBPL7)
M_96	9570	golgi SNAP receptor complex member 2(GOSR2)
M_96	6871	transcriptional adaptor 2A(TADA2A)
M_96	55316	radical S-adenosyl methionine domain containing
M_96	10951	chromobox 1(CBX1)
M_96	374819	leucine rich repeat containing 37 member
M_96	64132	xylosyltransferase 2(XYLT2)
M_96	339287	male specific lethal 1 homolog(MSL1)
M_96	641522	#N/A
M_96	3837	karyopherin subunit beta 1(KPNB1)
M_96	1267	2',3'-cyclic nucleotide 3' phosphodiesterase(CNP)
M_96	79893	gametogenetin binding protein 2(GGNBP2)
M_96	124989	EF-hand calcium binding domain 13(EFCAB13)

M_96	146923	RUN domain containing 1(RUNDC1)
M_96	22834	zinc finger protein 652(ZNF652)
M_96	100272146	nuclear factor, erythroid 2 like 3 pseudogene
M_96	11056	DExD-box helicase 52(DDX52)
M_96	7343	upstream binding transcription factor, RNA polymerase I(UBTF)
M_96	83473	katanin catalytic subunit A1 like 2(KATNAL2)
M_96	162427	family with sequence similarity 134 member
M_96	339201	ASB16 antisense RNA 1(ASB16-AS1)
M_96	729873	TBC1 domain family member 3(TBC1D3)
M_96	729877	TBC1 domain family member 3H(TBC1D3H)
M_96	414059	TBC1 domain family member 3B(TBC1D3B)
M_96	2648	lysine acetyltransferase 2A(KAT2A)
M_96	47	ATP citrate lyase(ACLY)
M_96	7266	DnaJ heat shock protein family (Hsp40) member C7(DNAJC7)
M_96	9343	elongation factor Tu GTP binding domain containing 2(EFTUD2)
M_96	9520	aminopeptidase puromycin sensitive(NPEPPS)
M_96	3728	junction plakoglobin(JUP)
M_96	84081	nuclear speckle splicing regulatory protein 1(NSRP1)
M_96	4642	myosin ID(MYO1D)
M_96	56953	5',3'-nucleotidase, mitochondrial(NT5M)
M_96	100169851	prostate and testis expressed 3(PATE3)
M_96	9842	pleckstrin homology and RUN domain containing M1(PLEKHM1)
M_96	10677	advillin(AVIL)
M_96	64847	spermatogenesis associated 20(SPATA20)
M_96	399968	prostate and testis expressed 4(PATE4)
M_96	346653	family with sequence similarity 71 member
M_96	6776	signal transducer and activator of transcription
M_96	9020	mitogen-activated protein kinase kinase kinase

M_96	80221	acyl-CoA synthetase family member 2(ACSF2)
M_96	10614	hexamethylene bisacetamide inducible 1(HEXIM1)
M_96	26272	F-box protein 4(FBXO4)
M_96	26574	apoptosis antagonizing transcription factor(AATF)
M_96	201175	#N/A
M_96	201176	Rho GTPase activating protein 27(ARHGAP27)
M_96	414060	TBC1 domain family member 3C(TBC1D3C)
M_96	440452	TBC1 domain family member 3 pseudogene
M_96	360155	cytochrome c, somatic pseudogene 52(CYCSP52)
M_96	84440	RAB11 family interacting protein 4(RAB11FIP4)
M_96	162699	transcription elongation factor B subunit
M_96	401250	mitochondrial coiled-coil domain 1(MCCD1)
M_96	729956	shisa family member 7(SHISA7)
M_96	127495	leucine rich repeat containing 39(LRRC39)
M_96	9027	N-acetyltransferase 8 (putative)(NAT8)
M_96	200420	ALMS1, centrosome and basal body associated protein pseudogene 1(ALMS1P1)
M_97	79944	L-2-hydroxyglutarate dehydrogenase(L2HGDH)
M_97	285237	chromosome 3 open reading frame 38(C3orf38)
M_97	122961	iron-sulfur cluster assembly 2(ISCA2)
M_97	23272	family with sequence similarity 208 member
M_97	122769	leucine rich repeat protein 1(LRR1)
M_97	283554	G protein-coupled receptor 137C(GPR137C)
M_97	5926	AT-rich interaction domain 4A(ARID4A)
M_97	10550	ADP ribosylation factor like GTPase 6 interacting protein 5(ARL6IP5)
M_97	283551	long intergenic non-protein coding RNA
M_97	10175	cornichon family AMPA receptor auxiliary protein
M_97	9039	ubiquitin like modifier activating enzyme 3(UBA3)
M_97	11169	WD repeat and HMG-box DNA binding protein
M_97	7110	TATA element modulatory factor 1(TMF1)
M_97	55030	F-box protein 34(FBXO34)

M_97	81542	thioredoxin related transmembrane protein 1(TMX1)
M_97	25978	charged multivesicular body protein 2B(CHMP2B)
M_97	771	carbonic anhydrase 12(CA12)
M_97	55015	pre-mRNA processing factor 39(PRPF39)
M_97	64693	cutaneous T-cell lymphoma-associated antigen
M_97	2079	enhancer of rudimentary homolog (Drosophila)(ERH)
M_97	64841	glucosamine-phosphate N-acetyltransferase
M_97	26060	adaptor protein, phosphotyrosine interacting with PH domain and leucine zipper 1(APPL1)
M_97	55012	protein phosphatase 2 regulatory subunit B"gamma(PPP2R3C)
M_97	5684	proteasome subunit alpha 3(PSMA3)
M_97	151987	protein phosphatase 4 regulatory subunit 2(PPP4R2)
M_97	23429	RING1 and YY1 binding protein(RYBP)
M_97	8545	CGG triplet repeat binding protein 1(CGGBP1)
M_97	51241	COX16, cytochrome c oxidase assembly
M_97	5283	phosphatidylinositol glycan anchor biosynthesis class
M_97	9147	nuclear export mediator factor(NEMF)
M_97	6815	serine/threonine/tyrosine interacting protein(STYX)
M_97	7597	zinc finger and BTB domain containing 25(ZBTB25)
M_97	5162	pyruvate dehydrogenase (lipoamide) beta(PDHB)
M_97	11183	mitogen-activated protein kinase kinase kinase kinase 5(MAP4K5)
M_97	57544	thioredoxin domain containing 16(TXNDC16)
M_97	55279	zinc finger protein 654(ZNF654)
M_97	11177	bromodomain adjacent to zinc finger domain
M_97	7871	sarcolemma associated protein(SLMAP)
M_97	378	ADP ribosylation factor 4(ARF4)
M_97	4253	melanoma inhibitory activity 2(MIA2)
M_97	5427	DNA polymerase epsilon 2, accessory
M_97	115286	solute carrier family 25 member 26(SLC25A26)
M_97	382	ADP ribosylation factor 6(ARF6)

M_97	201626	phosphodiesterase 12(PDE12)
M_97	201627	DENN domain containing 6A(DENND6A)
M_97	4149	MYC associated factor X(MAX)
M_97	23034	sterile alpha motif domain containing 4A(SAMD4A)
M_97	6655	SOS Ras/Rho guanine nucleotide exchange factor
M_97	23588	kelch domain containing 2(KLHDC2)
M_97	100129075	KTN1 antisense RNA 1(KTN1-AS1)
M_97	122525	chromosome 14 open reading frame 28(C14orf28)
M_97	200845	potassium channel tetramerization domain containing 6(KCTD6)
M_97	55860	actin-related protein 10 homolog(ACTR10)
M_97	79609	valosin containing protein lysine
M_97	9861	proteasome 26S subunit, non-ATPase 6(PSMD6)
M_97	5209	6-phosphofructo-2-kinase/fructose-2,6-biphosphatase 3(PFKFB3)
M_97	60485	salvador family WW domain containing protein
M_97	55320	MIS18 binding protein 1(MIS18BP1)
M_97	80145	THO complex 7(THOC7)
M_97	8801	succinate-CoA ligase GDP-forming beta
M_97	51199	ninein(NIN)
M_97	25923	atlastin GTPase 3(ATL3)
M_97	2632	1,4-alpha-glucan branching enzyme 1(GBE1)
M_97	55172	dynein axonemal assembly factor 2(DNAAF2)
M_97	283635	family with sequence similarity 177 member
M_97	55164	SHQ1, H/ACA ribonucleoprotein assembly
M_97	58517	RNA binding motif protein 25(RBM25)
M_97	27109	ATP synthase, H+ transporting, mitochondrial Fo complex subunit s (factor B)(ATP5S)
M_97	3895	kinectin 1(KTN1)
M_97	26520	translocase of inner mitochondrial membrane
M_97	11102	ribonuclease P/MRP subunit p14(RPP14)
M_97	5411	pinin, desmosome associated protein(PNN)

M_97	30001	endoplasmic reticulum oxidoreductase 1
M_97	91612	churchill domain containing 1(CHURC1)
M_97	10668	cell growth regulator with ring finger domain
M_97	1073	cofilin 2(CFL2)
M_97	5706	proteasome 26S subunit, ATPase 6(PSMC6)
M_97	51637	chromosome 14 open reading frame 166(C14orf166)
M_97	2764	glia maturation factor beta(GMFB)
M_97	145389	solute carrier family 38 member 6(SLC38A6)
M_97	57697	Fanconi anemia complementation group M(FANCM)
M_97	4247	mannosyl (alpha-1,6-)-glycoprotein beta-1,2-N-acetylglucosaminyltransferase(MGAT2)
M_97	50650	Rho guanine nucleotide exchange factor
M_97	8751	ADAM metallopeptidase domain 15(ADAM15)
M_97	152078	PQ loop repeat containing 2 like(PQLC2L)
M_97	6314	ataxin 7(ATXN7)
M_97	7804	LDL receptor related protein 8(LRP8)
M_97	51460	Scm-like with four mbt domains 1(SFMBT1)
M_97	100131827	zinc finger protein 717(ZNF717)
M_97	51062	atlastin GTPase 1(ATL1)
M_97	8317	cell division cycle 7(CDC7)
M_97	54899	PX domain containing serine/threonine kinase
M_97	2317	filamin B(FLNB)
M_97	112849	trans-L-3-hydroxyproline dehydratase(L3HYPDH)
M_97	2649	nuclear receptor subfamily 6 group A member
M_98	84651	serine peptidase inhibitor, Kazal type 7
M_98	431707	LIM homeobox 8(LHX8)
M_98	170062	family with sequence similarity 47 member
M_98	3906	lactalbumin alpha(LALBA)
M_98	56344	calcium binding protein 5(CABP5)
M_98	50508	NADPH oxidase 3(NOX3)
M_98	117159	dermcidin(DCD)
M_98	4622	myosin heavy chain 4(MYH4)

M_98	143502	olfactory receptor family 52 subfamily I member
M_98	143496	olfactory receptor family 52 subfamily B member 4 (gene/pseudogene)(OR52B4)
M_98	283491	olfactory receptor family 7 subfamily E member 156 pseudogene(OR7E156P)
M_98	442194	olfactory receptor family 10 subfamily C member 1 (gene/pseudogene)(OR10C1)
M_98	340074	uncharacterized LOC340074(LOC340074)
M_98	1373	carbamoyl-phosphate synthase 1(CPS1)
M_98	100132403	family with sequence similarity 157 member
M_98	2658	growth differentiation factor 2(GDF2)
M_98	337967	keratin associated protein 6-2(KRTAP6-2)
M_98	442038	sulfotransferase family 1C member 3(SULT1C3)
M_98	389400	GDNF family receptor alpha like(GFRAL)
M_98	9356	solute carrier family 22 member 6(SLC22A6)
M_98	375337	testis and ovary specific PAZ domain containing
M_98	84664	chondroitin sulfate proteoglycan 4 pseudogene 2, Y-linked(CSPG4P2Y)
M_98	64002	PCGEM1, prostate-specific transcript (non-protein coding)(PCGEM1)
M_98	138803	olfactory receptor family 13 subfamily C member
M_98	440888	ACTR3B pseudogene 2(ACTR3BP2)
M_98	200726	hCG1657980(LOC200726)
M_98	154150	hepatoma derived growth factor-like 1(HDGFL1)
M_98	144125	olfactory receptor family 2 subfamily AG member 1 (gene/pseudogene)(OR2AG1)
M_98	7544	zinc finger protein, Y-linked(ZFY)
M_98	119695	olfactory receptor family 52 subfamily R member 1 (gene/pseudogene)(OR52R1)
M_98	390037	olfactory receptor family 52 subfamily I member
M_98	3228	homeobox C12(HOXC12)
M_98	128360	olfactory receptor family 10 subfamily T member

M_98	89883	olfactory receptor family 6 subfamily W member 1 pseudogene(OR6W1P)
M_98	57829	zona pellucida glycoprotein 4(ZP4)
M_98	644076	glycosylation dependent cell adhesion molecule 1 (pseudogene)(GLYCAM1)
M_98	26689	olfactory receptor family 4 subfamily D member
M_98	388946	transmembrane protein 247(TMEM247)
M_98	138805	olfactory receptor family 13 subfamily F member
M_98	138804	olfactory receptor family 13 subfamily C member
M_98	29034	CPS1 intronic transcript 1(CPS1-IT1)
M_98	401082	long intergenic non-protein coding RNA
M_98	90070	lacritin(LACRT)
M_98	386684	keratin associated protein 12-4(KRTAP12-4)
M_98	392360	cathepsin L family member 3, pseudogene(CTSL3P)
M_98	3447	interferon alpha 13(IFNA13)
M_98	100130017	ferritin light chain pseudogene 10(FTLP10)
M_98	3445	interferon alpha 8(IFNA8)
M_98	6702	small proline rich protein 2C (pseudogene)(SPRR2C)
M_98	390181	olfactory receptor family 5 subfamily AK member 2(OR5AK2)
M_98	503835	double homeobox A(DUXA)
M_98	81392	olfactory receptor family 2 subfamily AE member
M_98	79324	olfactory receptor family 51 subfamily G member 1 (gene/pseudogene)(OR51G1)
M_98	3890	keratin 84(KRT84)
M_98	401667	olfactory receptor family 51 subfamily A member
M_98	79310	olfactory receptor family 5 subfamily H member
M_98	386681	keratin associated protein 10-8(KRTAP10-8)
M_98	164380	cystatin 13, pseudogene(CST13P)
M_98	386678	keratin associated protein 10-11(KRTAP10-11)
M_98	283953	transmembrane protein 114(TMEM114)
M_98	677784	family with sequence similarity 138 member

M_98	221613	histone cluster 1 H2A family member a(HIST1H2AA)
M_98	387601	solute carrier family 22 member 25(SLC22A25)
M_98	222894	Fer3 like bHLH transcription factor(FERD3L)
M_98	122740	olfactory receptor family 4 subfamily K member
M_98	219453	olfactory receptor family 8 subfamily K member
M_98	3439	interferon alpha 1(IFNA1)
M_98	3440	interferon alpha 2(IFNA2)
M_98	3442	interferon alpha 5(IFNA5)
M_98	337976	keratin associated protein 20-2(KRTAP20-2)
M_98	122742	olfactory receptor family 4 subfamily L member
M_98	337978	keratin associated protein 21-2(KRTAP21-2)
M_98	119692	olfactory receptor family 51 subfamily S member
M_98	319101	keratin 73(KRT73)
M_98	390110	1-aminocyclopropane-1-carboxylate synthase homolog (inactive) like(ACCSL)
M_98	64663	SPANX family member C(SPANXC)
M_98	219960	olfactory receptor family 10 subfamily Q member
M_98	149685	adipogenin(ADIG)
M_98	677836	small nucleolar RNA, H/ACA box 58(SNORA58)
M_98	6263	ryanodine receptor 3(RYR3)
M_98	728712	SPANX family member A2(SPANXA2)
M_98	10368	calcium voltage-gated channel auxiliary subunit gamma 3(CACNG3)
M_98	677794	small nucleolar RNA, H/ACA box 2B(SNORA2B)
M_98	6406	semenogelin I(SEMG1)
M_98	6407	semenogelin II(SEMG2)
M_98	7252	thyroid stimulating hormone beta(TSHB)
M_98	135935	NOBOX oogenesis homeobox(NOBOX)
M_98	285501	long intergenic non-protein coding RNA
M_98	138802	olfactory receptor family 13 subfamily C member
M_98	94033	ferritin mitochondrial(FTMT)
M_98	4759	neuraminidase 2(NEU2)

M_98	84618	5'-nucleotidase, cytosolic IA(NT5C1A)
M_98	128826	MIR1-1 host gene(MIR1-1HG)
M_98	654502	IQ motif containing J(IQCJ)
M_98	284194	galectin 9B(LGALS9B)
M_98	390323	olfactory receptor family 6 subfamily C member
M_98	128371	olfactory receptor family 6 subfamily K member
M_98	4626	myosin heavy chain 8(MYH8)
M_98	387978	long intergenic non-protein coding RNA
M_98	171489	#N/A
M_98	619499	small nucleolar RNA, H/ACA box 27(SNORA27)
M_98	2290	forkhead box G1(FOXG1)
M_98	765	carbonic anhydrase 6(CA6)
M_98	147323	StAR related lipid transfer domain containing
M_98	88	actinin alpha 2(ACTN2)
M_98	1411	crystallin beta A1(CRYBA1)
M_98	119687	olfactory receptor family 51 subfamily A member
M_98	677765	small Cajal body-specific RNA 18(SCARNA18)
M_98	63904	dual specificity phosphatase 21(DUSP21)
M_98	11023	ventral anterior homeobox 1(VAX1)
M_98	6718	aldo-keto reductase family 1 member D1(AKR1D1)
M_98	643160	chymosin pseudogene(CYMP)
M_98	494119	SPANX family member N2(SPANXN2)
M_98	654346	galectin 9C(LGALS9C)
M_98	4619	myosin heavy chain 1(MYH1)
M_98	4620	myosin heavy chain 2(MYH2)
M_98	780776	trans-golgi network vesicle protein 23 homolog
M_98	29958	dimethylglycine dehydrogenase(DMGDH)
M_98	23743	betaine--homocysteine S-methyltransferase
M_98	680	bombesin receptor subtype 3(BRS3)
M_98	434	agouti signaling protein(ASIP)
M_98	118856	matrix metallopeptidase 21(MMP21)
M_98	635	betaine--homocysteine S-methyltransferase(BHMT)

M_99	91316	glucuronidase, beta pseudogene 11(GUSBP11)
M_99	984	cyclin dependent kinase 11B(CDK11B)
M_99	142678	mindbomb E3 ubiquitin protein ligase 2(MIB2)
M_99	473	arginine-glutamic acid dipeptide repeats(RERE)
M_99	119016	ArfGAP with GTPase domain, ankyrin repeat and PH domain 4(AGAP4)
M_99	90834	#N/A
M_99	728262	family with sequence similarity 157 member
M_99	9696	ciliary rootlet coiled-coil, rootletin(CROCC)
M_99	8672	eukaryotic translation initiation factor 4 gamma
M_99	23360	formin binding protein 4(FNBP4)
M_99	729092	ArfGAP with GTPase domain, ankyrin repeat and PH domain 5(AGAP5)
M_99	9906	solute carrier family 35 member E2(SLC35E2)
M_99	9731	centrosomal protein 104(CEP104)
M_99	100133331	uncharacterized LOC100133331(LOC100133331)
M_99	10277	ubiquitination factor E4B(UBE4B)
M_99	10747	mannan binding lectin serine peptidase 2(MASP2)
M_99	1185	chloride voltage-gated channel 6(CLCN6)
M_99	55672	neuroblastoma breakpoint family member 1(NBPF1)
M_99	728642	cyclin dependent kinase 11A(CDK11A)
M_99	79707	nucleolar protein 9(NOL9)
M_99	6497	SKI proto-oncogene(SKI)
M_99	100133161	long intergenic non-protein coding RNA
M_99	8289	AT-rich interaction domain 1A(ARID1A)
M_99	57212	TP73 antisense RNA 1(TP73-AS1)
M_99	25832	neuroblastoma breakpoint family member
M_99	23352	ubiquitin protein ligase E3 component n-recognin
M_99	25981	dynein axonemal heavy chain 1(DNAH1)
M_99	200030	neuroblastoma breakpoint family member
M_99	55210	ATPase family, AAA domain containing

M_99	401010	NOC2 like nucleolar associated transcriptional repressor pseudogene 2(NOC2LP2)
M_99	84809	ciliary rootlet coiled-coil, rootletin pseudogene
M_99	100132406	neuroblastoma breakpoint family member
M_99	55187	vacuolar protein sorting 13 homolog D(VPS13D)
M_99	23095	kinesin family member 1B(KIF1B)
M_99	10250	serine and arginine repetitive matrix 1(SRRM1)
M_99	83858	ATPase family, AAA domain containing
M_99	9923	zinc finger and BTB domain containing 40(ZBTB40)
M_99	100128842	#N/A
M_99	261734	nephrocystin 4(NPHP4)
M_99	284702	#N/A
M_99	81669	cyclin L2(CCNL2)
M_99	7799	PR/SET domain 2(PRDM2)
M_99	414189	ArfGAP with GTPase domain, ankyrin repeat and PH domain 6(AGAP6)
M_99	114819	ciliary rootlet coiled-coil, rootletin pseudogene
M_99	399844	long intergenic non-protein coding RNA
M_99	55657	zinc finger protein 692(ZNF692)
M_99	116828	N4BPL2 intronic transcript 2(N4BP2L2-IT2)
M_99	100132287	uncharacterized LOC100132287(LOC100132287)
M_99	4820	natural killer cell triggering receptor(NKTR)
M_99	148479	PHD finger protein 13(PHF13)
M_99	285908	long intergenic non-protein coding RNA
M_99	728661	solute carrier family 35 member E2B(SLC35E2B)
M_99	23013	spen family transcriptional repressor(SPEN)
M_99	400818	neuroblastoma breakpoint family member 9(NBPF9)
M_99	22883	calsyntenin 1(CLSTN1)
M_99	8499	PTPRF interacting protein alpha 2(PPFIA2)
M_99	9563	hexose-6-phosphate dehydrogenase/glucose 1-dehydrogenase(H6PD)
M_99	767846	profilin 1 pseudogene 2(PFN1P2)

M_99	1565	cytochrome P450 family 2 subfamily D member
M_99	117852	tripartite motif containing 78, pseudogene(TRIM78P)
M_99	440435	G protein-coupled receptor 179(GPR179)
M_99	84224	neuroblastoma breakpoint family member 3(NBPF3)
M_99	100131496	uncharacterized LOC100131496(LOC100131496)
M_99	503645	divergent-paired related homeobox pseudogene
M_100	91694	LON peptidase N-terminal domain and ring finger 1(LONRF1)
M_100	1048	carcinoembryonic antigen related cell adhesion molecule 5(CEACAM5)
M_100	5533	protein phosphatase 3 catalytic subunit
M_100	79574	EPS8 like 3(EPS8L3)
M_100	79838	transmembrane channel like 5(TMC5)
M_100	6947	transcobalamin 1(TCN1)
M_100	51760	synaptotagmin 17(SYT17)
M_100	55790	chondroitin sulfate N-acetylgalactosaminyltransferase 1(CSGALNACT1)
M_100	197257	lactate dehydrogenase D(LDHD)
M_100	10321	cysteine rich secretory protein 3(CRISP3)
M_100	3783	potassium calcium-activated channel subfamily N member 4(KCNN4)
M_100	29883	CCR4-NOT transcription complex subunit 7(CNOT7)
M_100	9	N-acetyltransferase 1(NAT1)
M_100	8722	cathepsin F(CTSF)
M_100	80346	receptor accessory protein 4(REEP4)
M_100	57509	microtubule associated tumor suppressor 1(MTUS1)
M_100	203069	R3H domain and coiled-coil containing 1(R3HCC1)
M_100	27132	copine 7(CPNE7)
M_100	896	cyclin D3(CCND3)
M_100	339398	leucine rich repeat and Ig domain containing
M_100	79873	nudix hydrolase 18(NUDT18)
M_100	252969	nei like DNA glycosylase 2(NEIL2)

M_100	219970	glycine-N-acyltransferase like 2(GLYATL2)
M_100	661	RNA polymerase III subunit D(POLR3D)
M_100	285629	uncharacterized LOC285629(LOC285629)
M_100	54984	PIN2/TERF1 interacting, telomerase inhibitor
M_100	54793	potassium channel tetramerization domain containing 9(KCTD9)
M_100	63898	SH2 domain containing 4A(SH2D4A)
M_100	23039	exportin 7(XPO7)
M_100	5284	polymeric immunoglobulin receptor(PIGR)
M_100	5190	peroxisomal biogenesis factor 6(PEX6)
M_100	84152	protein phosphatase 1 regulatory inhibitor subunit 1B(PPP1R1B)
M_100	80325	ankyrin repeat and BTB domain containing 1(ABTB1)
M_100	2289	FK506 binding protein 5(FKBP5)
M_100	55174	integrator complex subunit 10(INTS10)
M_100	284340	C-X-C motif chemokine ligand 17(CXCL17)
M_100	163747	lymphocyte expansion molecule(LEXM)
M_100	427	N-acylsphingosine amidohydrolase 1(ASAH1)
M_100	1087	carcinoembryonic antigen related cell adhesion molecule 7(CEACAM7)
M_100	55909	bridging integrator 3(BIN3)
M_100	392465	glyoxalase domain containing 5(GLOD5)
M_100	55808	ST6 N-acetylgalactosaminide alpha-2,6-sialyltransferase 1(ST6GALNAC1)
M_100	55001	tetratricopeptide repeat domain 22(TTC22)
M_100	137075	claudin 23(CLDN23)
M_100	2051	EPH receptor B6(EPHB6)
M_100	157697	glutamate rich 1(ERICH1)
M_100	526	ATPase H+ transporting V1 subunit B2(ATP6V1B2)
M_100	131177	family with sequence similarity 3 member D(FAM3D)
M_100	7704	zinc finger and BTB domain containing 16(ZBTB16)
M_100	2705	gap junction protein beta 1(GJB1)

M_100	4680	carcinoembryonic antigen related cell adhesion molecule 6(CEACAM6)
M_100	80736	solute carrier family 44 member 4(SLC44A4)
M_100	26260	F-box protein 25(FBXO25)
M_100	4482	methionine sulfoxide reductase A(MSRA)
M_100	6097	RAR related orphan receptor C(RORC)
M_100	137492	VPS37A, ESCRT-I subunit(VPS37A)
M_100	9108	myotubularin related protein 7(MTMR7)
M_100	26469	protein tyrosine phosphatase, non-receptor type
M_100	2222	farnesyl-diphosphate farnesyltransferase 1(FDFT1)
M_100	55890	G protein-coupled receptor class C group 5 member C(GPRC5C)
M_100	2185	protein tyrosine kinase 2 beta(PTK2B)
M_100	23516	solute carrier family 39 member 14(SLC39A14)
M_100	158584	fatty acid amide hydrolase 2(FAAH2)
M_100	8795	TNF receptor superfamily member 10b(TNFRSF10B)
M_100	51312	solute carrier family 25 member 37(SLC25A37)
M_100	5520	protein phosphatase 2 regulatory subunit
M_100	9583	ectonucleoside triphosphate diphosphohydrolase
M_100	722	complement component 4 binding protein
M_100	66036	myotubularin related protein 9(MTMR9)
M_100	23120	ATPase phospholipid transporting 10B
M_100	5108	pericentriolar material 1(PCM1)
M_100	91782	charged multivesicular body protein 7(CHMP7)
M_100	8797	TNF receptor superfamily member 10a(TNFRSF10A)
M_100	55240	STEAP3 metalloreductase(STEAP3)
M_100	55503	transient receptor potential cation channel subfamily V member 6(TRPV6)
M_100	2525	fucosyltransferase 3 (Lewis blood group)(FUT3)
M_100	2528	fucosyltransferase 6(FUT6)
M_100	2527	fucosyltransferase 5(FUT5)
M_100	80352	ring finger protein 39(RNF39)

M_100	634	carcinoembryonic antigen related cell adhesion molecule 1(CEACAM1)
M_100	9572	nuclear receptor subfamily 1 group D member
M_100	284632	uncharacterized LOC284632(LOC284632)
M_100	1508	cathepsin B(CTSB)
M_100	11074	tripartite motif containing 31(TRIM31)
M_100	27189	interleukin 17C(IL17C)
M_100	10595	endoplasmic reticulum to nucleus signaling 2(ERN2)
M_100	54435	HLA complex group 4 (non-protein coding)(HCG4)
M_100	3995	fatty acid desaturase 3(FADS3)
M_100	6480	ST6 beta-galactoside alpha-2,6-sialyltransferase
M_100	1362	carboxypeptidase D(CPD)
M_100	4276	#N/A
M_100	79983	premature ovarian failure, 1B(POF1B)
M_100	2908	nuclear receptor subfamily 3 group C member
M_100	5005	orosomucoid 2(ORM2)
M_100	1041	corneodesmosin(CDSN)
M_100	3417	isocitrate dehydrogenase (NADP(+)) 1,
M_100	221468	transmembrane protein 217(TMEM217)
M_100	11030	RNA binding protein with multiple splicing(RBPMS)
M_100	64288	zinc finger and SCAN domain containing
M_100	285830	HLA-F antisense RNA 1(HLA-F-AS1)
M_100	5004	orosomucoid 1(ORM1)
M_100	26576	SRSF protein kinase 3(SRPK3)
M_101	218	aldehyde dehydrogenase 3 family member
M_101	146802	solute carrier family 47 member 2(SLC47A2)
M_101	389084	chromosome 2 open reading frame 82(C2orf82)
M_101	388939	chromosome 2 open reading frame 71(C2orf71)
M_101	124857	WAP, follistatin/kazal, immunoglobulin, kunitz and netrin domain containing 2(WFIKKN2)
M_101	440508	C-type lectin domain family 4 member G pseudogene 1(CLEC4GP1)

M_101	124930	ankyrin repeat domain 13B(ANKRD13B)
M_101	79745	CAP-Gly domain containing linker protein family member 4(CLIP4)
M_101	153478	pleckstrin homology and RhoGEF domain containing G4B(PLEKHG4B)
M_101	10332	C-type lectin domain family 4 member M(CLEC4M)
M_101	57795	BMP/retinoic acid inducible neural specific
M_101	4613	v-myc avian myelocytomatosis viral oncogene neuroblastoma derived homolog(MYCN)
M_101	90525	Src homology 2 domain containing F(SHF)
M_101	51066	ssu-2 homolog (C. elegans)(SSUH2)
M_101	100133234	SET binding factor 1 pseudogene 1(SBF1P1)
M_101	9379	neurexin 2(NRXN2)
M_101	65997	RAS like family 11 member B(RASL11B)
M_101	84501	spire type actin nucleation factor 2(SPIRE2)
M_101	51176	lymphoid enhancer binding factor 1(LEF1)
M_101	80274	signal peptide, CUB domain and EGF like domain containing 1(SCUBE1)
M_101	100073347	MER1 repeat containing imprinted transcript 1 (non-protein coding)(MIMT1)
M_101	8643	patched 2(PTCH2)
M_101	7301	TYRO3 protein tyrosine kinase(TYRO3)
M_101	460	astrotactin 1(ASTN1)
M_101	57172	calcium/calmodulin dependent protein kinase
M_101	10408	MYCN opposite strand(MYCNOS)
M_101	80760	inter-alpha-trypsin inhibitor heavy chain family member 5(ITIH5)
M_101	644150	WAS/WASL interacting protein family member
M_101	118738	zinc finger protein 488(ZNF488)
M_101	7482	Wnt family member 2B(WNT2B)
M_101	28964	GIT ArfGAP 1(GIT1)
M_101	859	caveolin 3(CAV3)

M_101	84502	junctophilin 4(JPH4)
M_101	2534	FYN proto-oncogene, Src family tyrosine
M_101	7475	Wnt family member 6(WNT6)
M_101	25791	neuronal guanine nucleotide exchange factor(NGEF)
M_101	5949	retinol binding protein 3(RBP3)
M_101	5021	oxytocin receptor(OXTR)
M_101	29995	LIM and cysteine rich domains 1(LMCD1)
M_101	23619	zinc finger imprinted 2(ZIM2)
M_101	84467	fibrillin 3(FBN3)
M_101	1280	collagen type II alpha 1 chain(COL2A1)
M_101	56245	chromosome 21 open reading frame 62(C21orf62)
M_101	80863	proline rich transmembrane protein 1(PRRT1)
M_101	84439	HHIP like 1(HHIPL1)
M_101	114786	XK related 4(XKR4)
M_101	80139	zinc finger protein 703(ZNF703)
M_101	57529	retrotransposon gag domain containing 1(RGAG1)
M_101	80326	Wnt family member 10A(WNT10A)
M_101	7060	thrombospondin 4(THBS4)
M_101	29931	long intergenic non-protein coding RNA
M_101	26056	RAB11 family interacting protein 5(RAB11FIP5)
M_101	55220	kelch domain containing 8A(KLHDC8A)
M_101	151393	regulator of microtubule dynamics 2(RMDN2)
M_101	53632	protein kinase AMP-activated non-catalytic subunit gamma 3(PRKAG3)
M_101	283514	siah E3 ubiquitin protein ligase family member
M_101	9498	solute carrier family 4 member 8(SLC4A8)
M_101	4326	matrix metallopeptidase 17(MMP17)
M_101	23705	cell adhesion molecule 1(CADM1)
M_101	29785	cytochrome P450 family 2 subfamily S member
M_101	55924	family with sequence similarity 212 member
M_101	220382	family with sequence similarity 181 member
M_101	64137	ATP binding cassette subfamily G member 4(ABCG4)

M_101	9751	syntaphilin(SNPH)
M_101	18	4-aminobutyrate aminotransferase(ABAT)
M_101	10124	ADP ribosylation factor like GTPase 4A(ARL4A)
M_101	57596	brain enriched guanylate kinase associated(BEGAIN)
M_101	80129	coiled-coil domain containing 170(CCDC170)
M_102	131578	leucine rich repeat containing 15(LRRC15)
M_102	1634	decorin(DCN)
M_102	1513	cathepsin K(CTSK)
M_102	115908	collagen triple helix repeat containing 1(CTHRC1)
M_102	2191	fibroblast activation protein alpha(FAP)
M_102	3624	inhibin beta A subunit(INHBA)
M_102	165	AE binding protein 1(AEBP1)
M_102	6678	secreted protein acidic and cysteine rich(SPARC)
M_102	9242	musculin(MSC)
M_102	4320	matrix metallopeptidase 11(MMP11)
M_102	23704	potassium voltage-gated channel subfamily E regulatory subunit 4(KCNE4)
M_102	10631	periostin(POSTN)
M_102	5176	serpin family F member 1(SERPINF1)
M_102	25903	olfactomedin like 2B(OLFML2B)
M_102	140862	isthmin 1(ISM1)
M_102	83716	cysteine rich secretory protein LCCL domain containing 2(CRISPLD2)
M_102	54829	asporin(ASPN)
M_102	1295	collagen type VIII alpha 1 chain(COL8A1)
M_102	4015	lysyl oxidase(LOX)
M_102	26585	gremlin 1, DAN family BMP antagonist(GREM1)
M_102	1833	epiphycan(EPYC)
M_102	9358	integrin subunit beta like 1(ITGBL1)
M_102	9509	ADAM metallopeptidase with thrombospondin type 1 motif 2(ADAMTS2)
M_102	6356	C-C motif chemokine ligand 11(CCL11)

M_102	8038	ADAM metallopeptidase domain 12(ADAM12)
M_102	1909	endothelin receptor type A(EDNRA)
M_102	85409	naked cuticle homolog 2(NKD2)
M_102	5396	paired related homeobox 1(PRRX1)
M_102	4313	matrix metallopeptidase 2(MMP2)
M_102	2674	GDNF family receptor alpha 1(GFRA1)
M_102	2200	fibrillin 1(FBN1)
M_102	1893	extracellular matrix protein 1(ECM1)
M_102	1300	collagen type X alpha 1 chain(COL10A1)
M_102	22801	integrin subunit alpha 11(ITGA11)
M_102	6423	secreted frizzled related protein 2(SFRP2)
M_102	50863	neurotrimin(NTM)
M_102	1289	collagen type V alpha 1 chain(COL5A1)
M_102	1277	collagen type I alpha 1 chain(COL1A1)
M_102	203430	zinc finger CCHC-type containing 5(ZCCHC5)
M_102	9547	C-X-C motif chemokine ligand 14(CXCL14)
M_102	1462	versican(VCAN)
M_102	1281	collagen type III alpha 1 chain(COL3A1)
M_102	7130	TNF alpha induced protein 6(TNFAIP6)
M_102	2335	fibronectin 1(FN1)
M_102	81792	ADAM metallopeptidase with thrombospondin type 1 motif 12(ADAMTS12)
M_102	115701	alpha kinase 2(ALPK2)
M_102	4060	lumican(LUM)
M_102	1290	collagen type V alpha 2 chain(COL5A2)
M_102	11227	polypeptide N-acetylgalactosaminyltransferase
M_102	7058	thrombospondin 2(THBS2)
M_102	6591	snail family transcriptional repressor 2(SNAI2)
M_102	1301	collagen type XI alpha 1 chain(COL11A1)
M_102	4016	lysyl oxidase like 1(LOXL1)
M_102	1292	collagen type VI alpha 2 chain(COL6A2)
M_102	1291	collagen type VI alpha 1 chain(COL6A1)

M_102	1293	collagen type VI alpha 3 chain(COL6A3)
M_102	6999	tryptophan 2,3-dioxygenase(TDO2)
M_102	4958	osteomodulin(OMD)
M_102	25907	transmembrane protein 158
M_102	4322	matrix metallopeptidase 13(MMP13)
M_102	27319	basic helix-loop-helix family member e22(BHLHE22)
M_102	84627	zinc finger protein 469(ZNF469)
M_102	81832	neuropilin and tolloid like 1(NETO1)
M_102	579	NK3 homeobox 2(NKX3-2)
M_102	1278	collagen type I alpha 2 chain(COL1A2)
M_102	114904	C1q and tumor necrosis factor related protein
M_102	2982	guanylate cyclase 1 soluble subunit alpha(GUCY1A3)
M_102	134111	ubiquitin conjugating enzyme E2 Q family like
M_102	51554	atypical chemokine receptor 4(ACKR4)
M_102	1734	deiodinase, iodothyronine type II(DIO2)
M_102	8877	sphingosine kinase 1(SPHK1)
M_102	84466	multiple EGF like domains 10(MEGF10)
M_102	131873	collagen type VI alpha 6 chain(COL6A6)
M_102	10699	corin, serine peptidase(CORIN)
M_102	5739	prostaglandin I2 (prostacyclin) receptor (IP)(PTGIR)
M_102	7412	vascular cell adhesion molecule 1(VCAM1)
M_102	5592	protein kinase, cGMP-dependent, type I(PRKG1)
M_102	8111	G protein-coupled receptor 68(GPR68)
M_102	113157	ribosomal protein lateral stalk subunit P0 pseudogene 2(RPLP0P2)
M_102	285548	long intergenic non-protein coding RNA
M_102	256076	collagen type VI alpha 5 chain(COL6A5)
M_102	10417	spondin 2(SPON2)
M_103	138151	NACC family member 2(NACC2)
M_103	2671	growth factor, augmenter of liver regeneration(GFER)
M_103	84331	MAPK regulated corepressor interacting protein
M_103	4350	N-methylpurine DNA glycosylase(MPG)

M_103	283871	phosphoglycolate phosphatase(PGP)
M_103	9567	GTP binding protein 1(GTPBP1)
M_103	4716	NADH:ubiquinone oxidoreductase subunit
M_103	84572	N-acetylglucosamine-1-phosphate transferase gamma subunit(GNPTG)
M_103	26088	golgi associated, gamma adaptin ear containing, ARF binding protein 1(GGA1)
M_103	90861	hematological and neurological expressed 1
M_103	9929	Josephin domain containing 1(JOSD1)
M_103	1632	enoyl-CoA delta isomerase 1(ECI1)
M_103	63922	chromosome transmission fidelity factor 18(CHTF18)
M_103	83986	family with sequence similarity 234 member
M_103	23162	mitogen-activated protein kinase 8 interacting protein 3(MAPK8IP3)
M_103	57465	TBC1 domain family member 24(TBC1D24)
M_103	1186	chloride voltage-gated channel 7(CLCN7)
M_103	65993	mitochondrial ribosomal protein S34(MRPS34)
M_103	5310	polycystin 1, transient receptor potential channel interacting(PKD1)
M_103	84219	WD repeat domain 24(WDR24)
M_103	8786	regulator of G-protein signaling 11(RGS11)
M_103	2657	growth differentiation factor 1(GDF1)
M_103	114984	FLYWCH family member 2(FLYWCH2)
M_103	7249	tuberous sclerosis 2(TSC2)
M_103	10921	RNA binding protein with serine rich domain
M_103	1454	casein kinase 1 epsilon(CSNK1E)
M_103	64718	unkempt family like zinc finger(UNKL)
M_103	26063	2,4-dienoyl-CoA reductase 2, peroxisomal(DECR2)
M_103	84326	methyltransferase like 26(METTL26)
M_103	11078	TRIO and F-actin binding protein(TRIOBP)
M_103	10127	zinc finger protein 263(ZNF263)
M_103	79903	N(alpha)-acetyltransferase 60, NatF catalytic

M_103	84231	TNF receptor associated factor 7(TRAF7)
M_103	8312	axin 1(AXIN1)
M_103	7727	zinc finger protein 174(ZNF174)
M_103	51005	amidohydrolase domain containing 2(AMDHD2)
M_103	64223	MTOR associated protein, LST8 homolog(MLST8)
M_103	6256	retinoid X receptor alpha(RXRA)
M_103	65990	family with sequence similarity 173 member
M_103	1820	AT-rich interaction domain 3A(ARID3A)
M_103	10573	mitochondrial ribosomal protein L28(MRPL28)
M_103	84464	SLX4 structure-specific endonuclease subunit(SLX4)
M_103	255057	CACN beta subunit associated regulatory
M_103	7038	thyroglobulin(TG)
M_103	6300	mitogen-activated protein kinase 12(MAPK12)
M_103	9463	protein interacting with PRKCA 1(PICK1)
M_103	527	ATPase H+ transporting V0 subunit c(ATP6V0C)
M_103	9894	telomere maintenance 2(TELO2)
M_103	398	Rho GDP dissociation inhibitor gamma(ARHGDIG)
M_103	3029	hydroxyacylglutathione hydrolase(HAGH)
M_103	2827	G protein-coupled receptor 3(GPR3)
M_103	23059	clusterin associated protein 1(CLUAP1)
M_103	1877	E4F transcription factor 1(E4F1)
M_103	25829	transmembrane protein 184B(TMEM184B)
M_103	5600	mitogen-activated protein kinase 11(MAPK11)
M_103	90850	zinc finger protein 598(ZNF598)
M_103	1387	CREB binding protein(CREBBP)
M_103	752014	cementum protein 1(CEMP1)
M_103	115939	TSR3, acp transferase ribosome maturation
M_103	51162	EGF like domain multiple 7(EGFL7)
M_103	84309	nudix hydrolase 16 like 1(NUDT16L1)
M_103	85377	MICAL like 1(MICALL1)
M_103	5170	3-phosphoinositide dependent protein kinase
M_103	81889	fumarylacetoacetate hydrolase domain containing

M_103	57799	RAB40C, member RAS oncogene family(RAB40C)
M_103	54442	potassium channel tetramerization domain containing 5(KCTD5)
M_103	84256	FLYWCH-type zinc finger 1(FLYWCH1)
M_103	79228	THO complex 6(THOC6)
M_103	6650	calpain 15(CAPN15)
M_103	90864	splA/ryanodine receptor domain and SOCS box containing 3(SPSB3)
M_103	55692	LUC7 like(LUC7L)
M_103	8131	NPR3 like, GATOR1 complex subunit(NPRL3)
M_103	51728	RNA polymerase III subunit K(POLR3K)
M_103	64285	rhomboid 5 homolog 1(RHBDF1)
M_103	51734	methionine sulfoxide reductase B1(MSRB1)
M_103	9742	intraflagellar transport 140(IFT140)
M_103	7410	vav guanine nucleotide exchange factor 2(VAV2)
M_103	89941	ras homolog family member T2(RHOT2)
M_103	10715	ceramide synthase 1(CERS1)
M_103	652276	potassium channel tetramerization domain containing 5 pseudogene(LOC652276)
M_103	10273	STIP1 homology and U-box containing protein
M_103	57585	cramped chromatin regulator homolog 1(CRAMP1)
M_103	610	hyperpolarization activated cyclic nucleotide gated potassium channel 2(HCN2)
M_103	23524	serine/arginine repetitive matrix 2(SRRM2)
M_103	7755	zinc finger protein 205(ZNF205)
M_103	113000	RNA pseudouridylate synthase domain containing
M_103	58986	transmembrane protein 8A(TMEM8A)
M_103	54925	zinc finger and SCAN domain containing
M_103	4913	nth like DNA glycosylase 1(NTHL1)
M_103	9091	phosphatidylinositol glycan anchor biosynthesis class
M_103	197342	essential meiotic structure-specific endonuclease subunit 2(EME2)

M_103	197335	WD repeat domain 90(WDR90)
M_103	10101	nucleotide binding protein 2(NUBP2)
M_103	7329	ubiquitin conjugating enzyme E2 I(UBE2I)
M_103	64428	nuclear prelamin A recognition factor like(NARFL)
M_103	9727	RAB11 family interacting protein 3(RAB11FIP3)
M_103	283951	chromosome 16 open reading frame 91(C16orf91)
M_103	54985	host cell factor C1 regulator 1(HCFC1R1)
M_103	10607	transducin beta like 3(TBL3)
M_103	4851	notch 1(NOTCH1)
M_103	1773	deoxyribonuclease 1(DNASE1)
M_103	645644	uncharacterized LOC645644(FLJ42627)
M_103	80178	chromosome 16 open reading frame 59(C16orf59)
M_103	57524	CASK interacting protein 1(CASKIN1)
M_103	7760	zinc finger protein 213(ZNF213)
M_103	339123	jumonji domain containing 8(JMJD8)
M_103	79622	small nuclear ribonucleoprotein U11/U12 subunit 25(SNRNP25)
M_103	100128385	family with sequence similarity 225 member B (non-protein coding)(FAM225B)
M_103	6923	transcription elongation factor B subunit 2(TCEB2)
M_103	7627	zinc finger protein 75a(ZNF75A)
M_103	7752	zinc finger protein 200(ZNF200)
M_103	23616	SH3 domain binding protein 1(SH3BP1)
M_103	84957	RELT tumor necrosis factor receptor(RELT)
M_103	91151	tigger transposable element derived 7(TIGD7)
M_103	4833	NME/NM23 nucleoside diphosphate kinase 4(NME4)
M_103	4832	NME/NM23 nucleoside diphosphate kinase 3(NME3)
M_103	286333	family with sequence similarity 225 member A (non-protein coding)(FAM225A)
M_103	222962	solute carrier family 29 member 4(SLC29A4)
M_103	51025	presequence translocase associated motor 16 homolog(PAM16)

M_103	11182	solute carrier family 2 member 6(SLC2A6)
M_103	85301	collagen type XXVII alpha 1 chain(COL27A1)
M_104	146562	chromosome 16 open reading frame 71(C16orf71)
M_104	642987	transmembrane protein 232(TMEM232)
M_104	79918	SET domain containing 6(SETD6)
M_104	84706	glutamic--pyruvic transaminase 2(GPT2)
M_104	10294	DnaJ heat shock protein family (Hsp40) member A2(DNAJA2)
M_104	10233	leucine rich repeat containing 23(LRRC23)
M_104	64282	poly(A) RNA polymerase D5, non-canonical(PAPD5)
M_104	339829	coiled-coil domain containing 39(CCDC39)
M_104	199223	tetratricopeptide repeat domain 21A(TTC21A)
M_104	89970	ring finger and SPRY domain containing 1(RSPRY1)
M_104	57567	zinc finger protein 319(ZNF319)
M_104	11051	nudix hydrolase 21(NUDT21)
M_104	10521	DEAD-box helicase 17(DDX17)
M_104	84229	dynein regulatory complex subunit 7(DRC7)
M_104	583	Bardet-Biedl syndrome 2(BBS2)
M_104	1459	casein kinase 2 alpha 2(CSNK2A2)
M_104	388272	chromosome 16 open reading frame 87(C16orf87)
M_104	27156	radial spoke head 14 homolog(RSPH14)
M_104	255919	CTD nuclear envelope phosphatase 1 regulatory subunit 1(CNEP1R1)
M_104	5934	RB transcriptional corepressor like 2(RBL2)
M_104	55239	2-oxoglutarate and iron dependent oxygenase domain containing 1(OGFOD1)
M_104	81533	integrin alpha FG-GAP repeat containing 1(ITFG1)
M_104	347736	NME/NM23 family member 9(NME9)
M_104	267	autocrine motility factor receptor(AMFR)
M_104	23568	ADP ribosylation factor like GTPase 2 binding protein(ARL2BP)
M_104	6477	siah E3 ubiquitin protein ligase 1(SIAH1)

M_104	9688	nucleoporin 93(NUP93)
M_104	29117	bromodomain containing 7(BRD7)
M_104	1540	CYLD lysine 63 deubiquitinase(CYLD)
M_104	388963	chromosome 2 open reading frame 81(C2orf81)
M_104	55815	translin associated factor X interacting protein
M_104	84645	chromosome 22 open reading frame 23(C22orf23)
M_104	23322	RPGRIP1 like(RPGRIP1L)
M_104	85320	ATP binding cassette subfamily C member
M_104	388389	coiled-coil domain containing 103(CCDC103)
M_104	29070	coiled-coil domain containing 113(CCDC113)
M_104	9609	RAB36, member RAS oncogene family(RAB36)
M_104	23019	CCR4-NOT transcription complex subunit 1(CNOT1)
M_104	55737	VPS35, retromer complex component(VPS35)
M_104	5257	phosphorylase kinase regulatory subunit beta(PHKB)
M_104	55779	cilia and flagella associated protein 44(CFAP44)
M_104	219621	chromosome 10 open reading frame 107(C10orf107)
M_104	93233	coiled-coil domain containing 114(CCDC114)
M_104	79650	U6 snRNA biogenesis phosphodiesterase 1(USB1)
M_104	80205	chromodomain helicase DNA binding protein
M_104	84058	WD repeat domain 54(WDR54)
M_104	79068	FTO, alpha-ketoglutarate dependent
M_104	57577	coiled-coil domain containing 191(CCDC191)
M_104	9683	NEDD4 binding protein 1(N4BP1)
M_104	55238	solute carrier family 38 member 7(SLC38A7)
M_104	130813	chromosome 2 open reading frame 50(C2orf50)
M_104	64400	AKT interacting protein(AKTIP)
M_104	400927	TPTE and PTEN homologous inositol lipid phosphatase pseudogene(LOC400927)
M_104	55027	HEAT repeat containing 3(HEATR3)
M_104	646851	family with sequence similarity 227 member
M_104	83752	lon peptidase 2, peroxisomal(LONP2)
M_104	57576	kinesin family member 17(KIF17)

M_104	64800	EF-hand calcium binding domain 6(EFCAB6)
M_104	284900	TTC28 antisense RNA 1(TTC28-AS1)
M_104	8398	phospholipase A2 group VI(PLA2G6)
M_104	4324	matrix metallopeptidase 15(MMP15)
M_104	29799	yippee like 1(YPEL1)
M_104	144448	tetraspanin 19(TSPAN19)
M_104	9666	DAZ interacting zinc finger protein 3(DZIP3)
M_104	9709	homocysteine inducible ER protein with ubiquitin like domain 1(HERPUD1)
M_104	113	adenylate cyclase 7(ADCY7)
M_104	221442	adenylate cyclase 10, soluble pseudogene
M_105	146894	CD300 molecule like family member g(CD300LG)
M_105	7431	vimentin(VIM)
M_105	147463	ankyrin repeat domain 29(ANKRD29)
M_105	283	angiogenin(ANG)
M_105	5629	prospero homeobox 1(PROX1)
M_105	6038	ribonuclease A family member 4(RNASE4)
M_105	54361	Wnt family member 4(WNT4)
M_105	22915	multimerin 1(MMRN1)
M_105	5010	claudin 11(CLDN11)
M_105	90865	interleukin 33(IL33)
M_105	4969	osteoglycin(OGN)
M_105	130497	odd-skipped related transciption factor 1(OSR1)
M_105	167681	protease, serine 35(PRSS35)
M_105	9737	G protein-coupled receptor associated sorting protein 1(GPRASP1)
M_105	338328	glycosylphosphatidylinositol anchored high density lipoprotein binding protein 1(GPIHBP1)
M_105	7148	tenascin XB(TNXB)
M_105	8436	serum deprivation response(SDPR)
M_105	56062	kelch like family member 4(KLHL4)
M_105	10350	ATP binding cassette subfamily A member 9(ABCA9)

M_105	10351	ATP binding cassette subfamily A member 8(ABCA8)
M_105	1808	dihydropyrimidinase like 2(DPYSL2)
M_105	9457	four and a half LIM domains 5(FHL5)
M_105	171024	synaptopodin 2(SYNPO2)
M_105	84525	HOP homeobox(HOPX)
M_105	3699	inter-alpha-trypsin inhibitor heavy chain 3(ITIH3)
M_105	5212	vitrin(VIT)
M_105	665	BCL2 interacting protein 3 like(BNIP3L)
M_105	286133	scavenger receptor class A member 5(SCARA5)
M_105	10894	lymphatic vessel endothelial hyaluronan receptor
M_105	4660	protein phosphatase 1 regulatory subunit
M_105	23460	ATP binding cassette subfamily A member 6(ABCA6)
M_105	4629	myosin heavy chain 11(MYH11)
M_105	155	adrenoceptor beta 3(ADRB3)
M_105	316	aldehyde oxidase 1(AOX1)
M_105	5322	phospholipase A2 group V(PLA2G5)
M_105	5320	phospholipase A2 group IIA(PLA2G2A)
M_105	5168	ectonucleotide pyrophosphatase/phosphodiesterase
M_105	121601	anoctamin 4(ANO4)
M_105	27254	cold shock domain containing C2(CSDC2)
M_105	10687	paraneoplastic Ma antigen 2(PNMA2)
M_105	6332	sodium voltage-gated channel alpha subunit
M_105	2949	glutathione S-transferase mu 5(GSTM5)
M_105	2492	follicle stimulating hormone receptor(FSHR)
M_105	145820	long intergenic non-protein coding RNA
M_105	3290	hydroxysteroid 11-beta dehydrogenase 1(HSD11B1)
M_105	3284	hydroxy-delta-5-steroid dehydrogenase, 3 beta- and steroid delta-isomerase 2(HSD3B2)
M_105	116362	retinol binding protein 7(RBP7)
M_105	79785	RERG like(RERGL)
M_105	56956	LIM homeobox 9(LHX9)

M_105	783	calcium voltage-gated channel auxiliary subunit beta 2(CACNB2)
M_105	8912	calcium voltage-gated channel subunit alpha1
M_105	7448	vitronectin(VTN)
M_105	660	BMX non-receptor tyrosine kinase(BMX)
M_105	7710	zinc finger protein 154(ZNF154)
M_105	79891	zinc finger protein 671(ZNF671)
M_105	10216	proteoglycan 4(PRG4)
M_105	10349	ATP binding cassette subfamily A member
M_105	255324	epithelial mitogen(EPGN)
M_105	147372	collagen and calcium binding EGF domains
M_105	283316	CD163 molecule like 1(CD163L1)
M_105	25823	tryptase gamma 1(TPSG1)
M_105	83849	synaptotagmin 15(SYT15)
M_105	51200	carboxypeptidase A4(CPA4)
M_105	3283	hydroxy-delta-5-steroid dehydrogenase, 3 beta- and steroid delta-isomerase 1(HSD3B1)
M_106	163087	zinc finger protein 383(ZNF383)
M_106	1054	CCAAT/enhancer binding protein gamma(CEBPG)
M_106	55900	zinc finger protein 302(ZNF302)
M_106	390916	nudix hydrolase 19(NUDT19)
M_106	374899	zinc finger protein 829(ZNF829)
M_106	25850	zinc finger protein 345(ZNF345)
M_106	163081	zinc finger protein 567(ZNF567)
M_106	163131	zinc finger protein 780B(ZNF780B)
M_106	374900	zinc finger protein 568(ZNF568)
M_106	51599	lipolysis stimulated lipoprotein receptor(LSR)
M_106	199704	zinc finger protein 585A(ZNF585A)
M_106	284406	ZFP82 zinc finger protein(ZFP82)
M_106	84503	zinc finger protein 527(ZNF527)
M_106	5184	peptidase D(PEPD)
M_106	84079	ankyrin repeat domain 27(ANKRD27)

M_106	148268	zinc finger protein 570(ZNF570)
M_106	1325	cortistatin(CORT)
M_106	148266	zinc finger protein 569(ZNF569)
M_106	84902	centrosomal protein 89(CEP89)
M_106	57655	GRAM domain containing 1A(GRAMD1A)
M_106	339318	zinc finger protein 181(ZNF181)
M_106	339324	zinc finger protein 260(ZNF260)
M_106	26065	LSM14A, mRNA processing body assembly
M_106	199745	THAP domain containing 8(THAP8)
M_106	91442	Fanconi anemia core complex associated protein
M_106	55094	G-patch domain containing 1(GPATCH1)
M_106	147929	zinc finger protein 565(ZNF565)
M_106	57677	ZFP14 zinc finger protein(ZFP14)
M_106	5438	RNA polymerase II subunit I(POLR2I)
M_106	9141	programmed cell death 5(PDCD5)
M_106	57711	zinc finger protein 529(ZNF529)
M_106	11136	solute carrier family 7 member 9(SLC7A9)
M_106	148103	zinc finger protein 599(ZNF599)
M_106	284323	zinc finger protein 780A(ZNF780A)
M_106	388536	zinc finger protein 790(ZNF790)
M_106	284459	HKR1, GLI-Kruppel zinc finger family
M_106	90075	zinc finger protein 30(ZNF30)
M_106	22847	zinc finger protein 507(ZNF507)
M_106	2821	glucose-6-phosphate isomerase(GPI)
M_106	126248	WD repeat domain 88(WDR88)
M_106	126231	zinc finger protein 573(ZNF573)
M_106	84924	zinc finger protein 566(ZNF566)
M_106	51276	zinc finger protein 571(ZNF571)
M_106	147991	dpy-19 like 3 (C. elegans)(DPY19L3)
M_106	85415	rhophilin Rho GTPase binding protein 2(RHPN2)
M_106	79414	leucine rich repeat and fibronectin type III domain containing 3(LRFN3)

M_106	339327	zinc finger protein 546(ZNF546)
M_106	9710	KIAA0355(KIAA0355)
M_106	10054	ubiquitin like modifier activating enzyme 2(UBA2)
M_106	390927	zinc finger protein 793(ZNF793)
M_106	84775	zinc finger protein 607(ZNF607)
M_106	7705	zinc finger protein 146(ZNF146)
M_106	163255	zinc finger protein 540(ZNF540)
M_106	388531	regulator of G-protein signaling 9 binding
M_106	92283	zinc finger protein 461(ZNF461)
M_106	92285	zinc finger protein 585B(ZNF585B)
M_106	22835	ZFP30 zinc finger protein(ZFP30)
M_106	6865	tachykinin receptor 2(TACR2)
M_106	126375	zinc finger protein 792(ZNF792)
M_106	84911	zinc finger protein 382(ZNF382)
M_106	1340	cytochrome c oxidase subunit 6B1(COX6B1)
M_106	7392	upstream transcription factor 2, c-fos
M_106	84306	programmed cell death 2 like(PDCD2L)
M_106	147923	zinc finger protein 420(ZNF420)
M_106	2862	motilin receptor(MLNR)
M_106	8725	URI1, prefoldin like chaperone(URI1)
M_106	5871	mitogen-activated protein kinase kinase kinase kinase 2(MAP4K2)
M_106	53827	FXYD domain containing ion transport regulator
M_106	148189	long intergenic non-protein coding RNA
M_106	10775	POP4 homolog, ribonuclease P/MRP subunit(POP4)
M_106	7386	ubiquinol-cytochrome c reductase, Rieske iron-sulfur polypeptide 1(UQCRFS1)
M_106	6324	sodium voltage-gated channel beta subunit 1(SCN1B)
M_106	898	cyclin E1(CCNE1)
M_106	79156	pleckstrin homology and FYVE domain containing 1(PLEKHF1)
M_106	83636	chromosome 19 open reading frame 12(C19orf12)

M_107	283159	olfactory receptor family 8 subfamily D member
M_107	84539	melanin concentrating hormone receptor 2(MCHR2)
M_107	28513	cadherin 19(CDH19)
M_107	283160	olfactory receptor family 8 subfamily D member 2 (gene/pseudogene)(OR8D2)
M_107	429	achaete-scute family bHLH transcription factor
M_107	100133469	#N/A
M_107	574	B melanoma antigen(BAGE)
M_107	170626	X antigen family member 3(XAGE3)
M_107	170627	X antigen family member 5(XAGE5)
M_107	729408	G antigen 2D(GAGE2D)
M_107	2543	G antigen 1(GAGE1)
M_107	100132399	G antigen 12D(GAGE12D)
M_107	390275	olfactory receptor family 8 subfamily A member
M_107	390271	olfactory receptor family 8 subfamily B member
M_107	100133308	Ras suppressor protein 1 pseudogene 2(RSU1P2)
M_107	26493	olfactory receptor family 8 subfamily B member
M_107	390083	olfactory receptor family 56 subfamily A member
M_107	116337	pannexin 3(PANX3)
M_107	25834	MGAT4 family member C(MGAT4C)
M_107	9503	#N/A
M_107	346288	septin 14(SEPT14)
M_107	280659	SSX family member 8(SSX8)
M_107	280658	SSX family member 7(SSX7)
M_107	100132831	A20-binding inhibitor of NF-kappaB activation 2 pseudogene(LOC100132831)
M_107	171483	family with sequence similarity 9 member B(FAM9B)
M_107	219858	olfactory receptor family 8 subfamily B member
M_107	219865	olfactory receptor family 8 subfamily G member
M_107	259234	Down syndrome critical region 10 (non-protein coding)(DSCR10)
M_107	120793	olfactory receptor family 56 subfamily A member

M_107	170712	cytochrome c oxidase subunit 7B2(COX7B2)
M_107	5053	phenylalanine hydroxylase(PAH)
M_107	8712	PAGE family member 1(PAGE1)
M_107	1081	glycoprotein hormones, alpha polypeptide(CGA)
M_107	283162	olfactory receptor family 8 subfamily B member 4 (gene/pseudogene)(OR8B4)
M_107	6757	SSX family member 2(SSX2)
M_107	6758	SSX family member 5(SSX5)
M_107	2576	G antigen 4(GAGE4)
M_107	6756	SSX family member 1(SSX1)
M_107	2574	G antigen 2C(GAGE2C)
M_107	26595	olfactory receptor family 8 subfamily B member
M_107	59082	caspase recruitment domain family member
M_107	150185	long intergenic non-protein coding RNA
M_107	120796	olfactory receptor family 56 subfamily A member
M_107	400830	defensin beta 132(DEFB132)
M_107	731789	long intergenic non-protein coding RNA 202-
M_107	387644	long intergenic non-protein coding RNA 202-
M_107	85319	B melanoma antigen family member 2(BAGE2)
M_107	7253	thyroid stimulating hormone receptor(TSHR)
M_107	3175	one cut homeobox 1(ONECUT1)
M_107	64478	CUB and Sushi multiple domains 1(CSMD1)
M_107	146512	uncharacterized protein FLJ30679(FLJ30679)
M_107	54967	cancer/testis antigen 55(CT55)
M_107	100101629	G antigen 8(GAGE8)
M_107	7179	transmembrane phosphatase with tensin
M_107	4018	lipoprotein(a)(LPA)
M_108	388387	long intergenic non-protein coding RNA
M_108	727940	Rhox homeobox family member 2B(RHOXF2B)
M_108	29125	chromosome 11 open reading frame 21(C11orf21)
M_108	91653	BOC cell adhesion associated, oncogene
M_108	283150	forkhead box R1(FOXR1)

M_108	314	amine oxidase, copper containing 2(AOC2)
M_108	11093	ADAM metallopeptidase with thrombospondin type 1 motif 13(ADAMTS13)
M_108	84249	pleckstrin and Sec7 domain containing 2(PSD2)
M_108	353149	TBC1 domain family member 26(TBC1D26)
M_108	85363	tripartite motif containing 5(TRIM5)
M_108	84891	zinc finger and SCAN domain containing
M_108	8468	FK506 binding protein 6(FKBP6)
M_108	121214	short chain dehydrogenase/reductase family 9C, member 7(SDR9C7)
M_108	60677	CUGBP, Elav-like family member 6(CELF6)
M_108	4761	neuronal differentiation 2(NEUROD2)
M_108	8029	cubilin(CUBN)
M_108	441204	uncharacterized LOC441204(LOC441204)
M_108	150568	long intergenic non-protein coding RNA
M_108	729288	zinc finger protein 286B(ZNF286B)
M_108	116512	MAS related GPR family member D(MRGPRD)
M_108	22989	myosin heavy chain 15(MYH15)
M_108	151516	aspartic peptidase, retroviral-like 1(ASPRV1)
M_108	148398	sterile alpha motif domain containing 11(SAMD11)
M_108	143630	ubiquilin-like(UBQLNL)
M_108	57335	zinc finger protein 286A(ZNF286A)
M_108	50831	taste 2 receptor member 3(TAS2R3)
M_108	728882	family with sequence similarity 182 member
M_108	55599	RNA binding region (RNP1, RRM) containing
M_108	825	calpain 3(CAPN3)
M_108	100190939	TPT1 antisense RNA 1(TPT1-AS1)
M_108	117854	tripartite motif containing 6(TRIM6)
M_108	23145	SCO-spondin(SSPO)
M_108	5455	POU class 3 homeobox 3(POU3F3)
M_108	8292	collagen like tail subunit of asymmetric acetylcholinesterase(COLQ)

M_108	284805	chromosome 20 open reading frame 203(C20orf203)
M_108	9808	KIAA0087(KIAA0087)
M_108	161497	stereocilin(STRC)
M_108	10077	tetraspanin 32(TSPAN32)
M_108	54429	taste 2 receptor member 5(TAS2R5)
M_108	340980	olfactory receptor family 52 subfamily B member
M_108	7732	ring finger protein 112(RNF112)
M_108	100130417	uncharacterized LOC100130417(LOC100130417)
M_108	221400	tudor domain containing 6(TDRD6)
M_108	8608	retinol dehydrogenase 16 (all-trans)(RDH16)
M_108	2979	guanylate cyclase activator 1B(GUCA1B)
M_108	50832	taste 2 receptor member 4(TAS2R4)
M_108	541473	FK506 binding protein 6, 36kDa
M_108	2620	growth arrest specific 2(GAS2)
M_108	280	amylase, alpha 2B (pancreatic)(AMY2B)
M_108	284800	family with sequence similarity 182 member
M_108	129530	lysozyme g1(LYG1)
M_108	133060	otopetrin 1(OTOP1)
M_108	9651	phospholipase C eta 2(PLCH2)
M_108	1775	deoxyribonuclease 1 like 2(DNASE1L2)
M_108	648740	actin gamma 1 pseudogene 4(ACTG1P4)
M_108	84795	pyridine nucleotide-disulphide oxidoreductase domain 2(PYROXD2)
M_108	785	calcium voltage-gated channel auxiliary subunit beta 4(CACNB4)
M_108	136242	protease, serine 37(PRSS37)
M_108	390595	ubiquitin associated protein 1 like(UBAP1L)
M_108	6010	rhodopsin(RHO)
M_108	56269	immunity related GTPase cinema(IRGC)
M_108	11280	sodium voltage-gated channel alpha subunit
M_108	1244	ATP binding cassette subfamily C member 2(ABCC2)
M_108	728939	#N/A

M_108	671	bactericidal/permeability-increasing protein(BPI)
M_108	27233	sulfotransferase family 1C member 4(SULT1C4)
M_108	401265	kelch like family member 31(KLHL31)
M_108	619207	scavenger receptor protein family member(SCART1)
M_108	268	anti-Mullerian hormone(AMH)
M_108	255239	ankyrin repeat and kinase domain containing
M_108	10297	APC2, WNT signaling pathway regulator(APC2)
M_108	169436	serine/threonine kinase like domain containing
M_108	285429	DDB1 and CUL4 associated factor 4 like
M_108	4336	myelin-associated oligodendrocyte basic
M_108	83875	beta-carotene oxygenase 2(BCO2)
M_108	100129726	long intergenic non-protein coding RNA
M_108	441381	leucine rich repeat containing 24(LRRC24)
M_108	140873	chromosome 20 open reading frame 173(C20orf173)
M_108	55512	sphingomyelin phosphodiesterase 3(SMPD3)
M_108	246181	aldo-keto reductase family 7 like
M_108	1571	cytochrome P450 family 2 subfamily E member
M_108	646300	collagen type VI alpha 4 pseudogene 2(COL6A4P2)
M_109	400223	#N/A
M_109	140711	TBC/LysM-associated domain containing 2(TLDC2)
M_109	100272217	uncharacterized LOC100272217(LOC100272217)
M_109	387032	zinc finger with KRAB and SCAN domains
M_109	100270746	uncharacterized LOC100270746(LOC100270746)
M_109	64241	ATP binding cassette subfamily G member 8(ABCG8)
M_109	152195	nudix hydrolase 16 pseudogene 1(NUDT16P1)
M_109	2822	glycosylphosphatidylinositol specific phospholipase D1(GPLD1)
M_109	28999	Kruppel like factor 15(KLF15)
M_109	166348	kelch repeat and BTB domain containing
M_109	170680	psoriasis susceptibility 1 candidate 2(PSORS1C2)
M_109	10486	CAP, adenylate cyclase-associated protein, 2
M_109	89766	uromodulin like 1(UMODL1)

M_109	100133205	long intergenic non-protein coding RNA
M_109	100130889	psoriasis susceptibility 1 candidate 3 (non-protein coding)(PSORS1C3)
M_109	100216545	KMT2E antisense RNA 1 (head to head)(KMT2E-
M_109	5987	tripartite motif containing 27(TRIM27)
M_109	7741	zinc finger and SCAN domain containing
M_109	7745	zinc finger with KRAB and SCAN domains
M_109	1951	cadherin EGF LAG seven-pass G-type receptor
M_109	7754	zinc finger protein 204, pseudogene(ZNF204P)
M_109	202781	PAXIP1 antisense RNA 1 (head to head)(PAXIP1-
M_109	80345	zinc finger and SCAN domain containing
M_109	170679	psoriasis susceptibility 1 candidate 1(PSORS1C1)
M_109	80317	zinc finger with KRAB and SCAN domains
M_109	346157	zinc finger protein 391(ZNF391)
M_109	344405	prolyl-tRNA synthetase associated domain containing 1, pseudogene(PRORSD1P)
M_109	29777	activator of basal transcription 1(ABT1)
M_109	2686	gamma-glutamyltransferase 7(GGT7)
M_109	6013	relaxin 1(RLN1)
M_109	6019	relaxin 2(RLN2)
M_109	150726	F-box protein 41(FBXO41)
M_109	147727	ILF3 antisense RNA 1 (head to head)(ILF3-AS1)
M_109	5462	POU class 5 homeobox 1B(POU5F1B)
M_109	5460	POU class 5 homeobox 1(POU5F1)
M_109	23428	solute carrier family 7 member 8(SLC7A8)
M_109	8856	nuclear receptor subfamily 1 group I member
M_109	64240	ATP binding cassette subfamily G member 5(ABCG5)
M_109	64757	mitochondrial amidoxime reducing component
M_109	79692	zinc finger protein 322(ZNF322)
M_109	100128822	long intergenic non-protein coding RNA
M_109	9753	zinc finger and SCAN domain containing
M_109	3798	kinesin family member 5A(KIF5A)

M_109	84913	atonal bHLH transcription factor 8(ATOH8)
M_109	7915	aldehyde dehydrogenase 5 family member
M_109	7738	zinc finger protein 184(ZNF184)
M_109	645676	ASH1L antisense RNA 1(ASH1L-AS1)
M_109	7746	zinc finger and SCAN domain containing 9(ZSCAN9)
M_109	3291	hydroxysteroid 11-beta dehydrogenase 2(HSD11B2)
M_109	644019	COBW domain containing 6(CBWD6)
M_109	79854	long intergenic non-protein coding RNA
M_109	84547	piggyBac transposable element derived 1(PGBD1)
M_109	80742	proline rich 3(PRR3)
M_109	286103	ZNF252P antisense RNA 1(ZNF252P-AS1)
M_109	152756	family with sequence similarity 218 member
M_109	11120	butyrophilin subfamily 2 member A1(BTN2A1)
M_109	79849	PDZ domain containing 3(PDZD3)
M_109	253639	zinc finger protein 620(ZNF620)
M_109	100130557	NFYC antisense RNA 1(NFYC-AS1)
M_109	10385	butyrophilin subfamily 2 member A2(BTN2A2)
M_109	400752	long intergenic non-protein coding RNA
M_109	100303728	SLC25A5 antisense RNA 1(SLC25A5-AS1)
M_109	100289341	MAN1B1 antisense RNA 1 (head to head)(MAN1B1-
M_109	2661	growth differentiation factor 9(GDF9)
M_109	92270	ATPase H+ transporting accessory protein 1 like(ATP6AP1L)
M_109	150384	GTSE1 antisense RNA 1 (head to head)(GTSE1-AS1)
M_110	641339	zinc finger protein 674(ZNF674)
M_110	54954	family with sequence similarity 120C(FAM120C)
M_110	219736	storkhead box 1(STOX1)
M_110	54552	G protein nucleolar 3 like(GNL3L)
M_110	256643	chromosome X open reading frame 23(CXorf23)
M_110	84679	solute carrier family 9 member A7(SLC9A7)
M_110	4007	prickle planar cell polarity protein 3(PRICKLE3)
M_110	79917	MAGI family member, X-linked(MAGIX)

M_110	7592	zinc finger protein 41(ZNF41)
M_110	5422	DNA polymerase alpha 1, catalytic subunit(POLA1)
M_110	10682	emopamil binding protein (sterol isomerase)(EBP)
M_110	100133957	UXT antisense RNA 1(UXT-AS1)
M_110	5127	cyclin dependent kinase 16(CDK16)
M_110	257397	TGF-beta activated kinase 1/MAP3K7 binding protein 3(TAB3)
M_110	24140	FtsJ RNA methyltransferase homolog 1 (E.
M_110	90060	coiled-coil domain containing 120(CCDC120)
M_110	1184	chloride voltage-gated channel 5(CLCN5)
M_110	6102	retinitis pigmentosa 2 (X-linked recessive)(RP2)
M_110	7355	solute carrier family 35 member A2(SLC35A2)
M_110	23133	PHD finger protein 8(PHF8)
M_110	389856	ubiquitin specific peptidase 27, X-linked(USP27X)
M_110	6839	suppressor of variegation 3-9 homolog 1(SUV39H1)
M_110	56850	GRIP1 associated protein 1(GRIPAP1)
M_110	2002	ELK1, ETS transcription factor(ELK1)
M_110	55593	OTU deubiquitinase 5(OTUD5)
M_110	80311	kelch like family member 15(KLHL15)
M_110	10075	HECT, UBA and WWE domain containing 1, E3 ubiquitin protein ligase(HUWE1)
M_110	64743	WD repeat domain 13(WDR13)
M_110	7317	ubiquitin like modifier activating enzyme 1(UBA1)
M_110	9767	jade family PHD finger 3(JADE3)
M_110	23096	IQ motif and Sec7 domain 2(IQSEC2)
M_110	10389	sex comb on midleg-like 2 (Drosophila)(SCML2)
M_110	401588	ZNF674 antisense RNA 1 (head to head)(ZNF674-
M_110	347344	zinc finger protein 81(ZNF81)
M_110	7569	zinc finger protein 182(ZNF182)
M_110	10159	ATPase H+ transporting accessory protein
M_110	27238	G-patch domain and KOW motifs(GPKOW)
M_110	6990	dynein light chain Tctex-type 3(DYNLT3)

M_110	369	A-Raf proto-oncogene, serine/threonine
M_110	4943	TBC1 domain family member 25(TBC1D25)
M_110	10013	histone deacetylase 6(HDAC6)
M_110	55634	KRAB box domain containing 4(KRBOX4)
M_110	158747	motile sperm domain containing 2(MOSPD2)
M_110	8237	ubiquitin specific peptidase 11(USP11)
M_110	7030	transcription factor binding to IGHM enhancer
M_110	8243	structural maintenance of chromosomes 1A(SMC1A)
M_110	8242	lysine demethylase 5C(KDM5C)
M_110	11152	WD repeat domain 45(WDR45)
M_110	57232	zinc finger protein 630(ZNF630)
M_110	158572	USP27X antisense RNA 1 (head to head)(USP27X-
M_110	28952	coiled-coil domain containing 22(CCDC22)
M_110	51360	membrane bound transcription factor peptidase, site 2(MBTPS2)
M_110	8241	RNA binding motif protein 10(RBM10)
M_110	89801	protein phosphatase 1 regulatory subunit
M_110	2643	GTP cyclohydrolase 1(GCH1)
M_110	399671	HEAT repeat containing 4(HEATR4)
M_110	6197	ribosomal protein S6 kinase A3(RPS6KA3)
M_110	8573	calcium/calmodulin dependent serine protein
M_110	3750	potassium voltage-gated channel subfamily D member 1(KCND1)
M_110	347404	LanC like 3(LANCL3)
M_110	5355	proteolipid protein 2(PLP2)
M_110	4810	NHS actin remodeling regulator(NHS)
M_110	10245	translocase of inner mitochondrial membrane 17 homolog B (yeast)(TIMM17B)
M_110	1756	dystrophin(DMD)
M_110	64840	porcupine homolog (Drosophila)(PORCN)
M_110	10084	polyglutamine binding protein 1(PQBP1)
M_110	6792	cyclin dependent kinase like 5(CDKL5)

M_110	54825	cadherin related family member 2(CDHR2)
M_110	11230	PRA1 domain family member 2(PRAF2)
M_110	79290	olfactory receptor family 13 subfamily A member
M_110	3266	ES cell expressed Ras(ERAS)
M_110	541468	leucine rich adaptor protein 1(LURAP1)
M_110	5935	RNA binding motif (RNP1, RRM) protein 3(RBM3)
M_110	5638	proline rich and Gla domain 1(PRRG1)
M_110	4281	midline 1(MID1)
M_111	643224	tubulin beta pseudogene 5(TUBBP5)
M_111	375318	aquaporin 12A(AQP12A)
M_111	79905	transmembrane channel like 7(TMC7)
M_111	386757	solute carrier family 6 member 10,
M_111	29122	protease, serine 50(PRSS50)
M_111	50506	dual oxidase 2(DUOX2)
M_111	653437	aquaporin 12B(AQP12B)
M_111	56604	tubulin beta 7 pseudogene(TUBB7P)
M_111	90527	dual oxidase maturation factor 1(DUOXA1)
M_111	4143	methionine adenosyltransferase 1A(MAT1A)
M_111	1145	cholinergic receptor nicotinic epsilon
M_111	114043	TSPEAR antisense RNA 2(TSPEAR-AS2)
M_111	8447	double C2 domain beta(DOC2B)
M_111	54084	thrombospondin type laminin G domain and EAR repeats(TSPEAR)
M_111	100130311	chromosome 17 open reading frame 107(C17orf107)
M_111	83715	espin(ESPN)
M_111	729355	TP53 target 3B(TP53TG3B)
M_111	9002	F2R like thrombin/trypsin receptor 3(F2RL3)
M_111	3171	forkhead box A3(FOXA3)
M_111	774	calcium voltage-gated channel subunit alpha1
M_111	440362	#N/A
M_111	405753	dual oxidase maturation factor 2(DUOXA2)
M_111	147199	secretoglobin family 1C member 1(SCGB1C1)

M_111	93659	chorionic gonadotropin beta subunit 5(CGB5)
M_111	54626	hes family bHLH transcription factor 2(HES2)
M_111	53905	dual oxidase 1(DUOX1)
M_111	29850	transient receptor potential cation channel subfamily M member 5(TRPM5)
M_111	4648	myosin VIIB(MYO7B)
M_111	51305	potassium two pore domain channel subfamily K member 9(KCNK9)
M_111	10316	neuromedin U receptor 1(NMUR1)
M_111	94115	chorionic gonadotropin beta subunit 8(CGB8)
M_111	2264	fibroblast growth factor receptor 4(FGFR4)
M_111	283487	long intergenic non-protein coding RNA
M_111	2840	G protein-coupled receptor 17(GPR17)
M_111	114336	chorionic gonadotropin beta subunit 2(CGB2)
M_111	114335	chorionic gonadotropin beta subunit 1(CGB1)
M_111	1082	chorionic gonadotropin beta subunit 3(CGB3)
M_111	9394	heparan sulfate 6-O-sulfotransferase 1(HS6ST1)
M_111	140690	CCCTC-binding factor like(CTCFL)
M_111	55679	LIM zinc finger domain containing 2(LIMS2)
M_111	9501	rabphilin 3A-like (without C2 domains)(RPH3AL)
M_111	3034	histidine ammonia-lyase(HAL)
M_111	284802	FSHD region gene 1 family member B,
M_111	10840	aldehyde dehydrogenase 1 family member
M_111	57582	potassium sodium-activated channel subfamily T member 1(KCNT1)
M_111	284904	SEC14 like lipid binding 4(SEC14L4)
M_111	3767	potassium voltage-gated channel subfamily J member 11(KCNJ11)
M_111	59344	arachidonate lipoxygenase 3(ALOXE3)
M_111	83878	USH1 protein network component harmonin binding protein 1(USHBP1)

M_111	340665	cytochrome P450 family 26 subfamily C member 1(CYP26C1)
M_111	79935	cyclin N-terminal domain containing 2(CNTD2)
M_111	9540	tumor protein p53 inducible protein 3(TP53I3)
M_111	286122	chromosome 8 open reading frame 31(C8orf31)
M_111	51285	RAS like family 12(RASL12)
M_111	148109	family with sequence similarity 187 member
M_111	2161	coagulation factor XII(F12)
M_111	3972	luteinizing hormone beta polypeptide(LHB)
M_111	113091	parathyroid hormone 2(PTH2)
M_111	242	arachidonate 12-lipoxygenase, 12R type(ALOX12B)
M_111	339965	coiled-coil domain containing 158(CCDC158)
M_111	6932	transcription factor 7 (T-cell specific, HMG-
M_111	21	ATP binding cassette subfamily A member 3(ABCA3)
M_111	648691	uncharacterized LOC648691(LL22NC03-63E9.3)
M_111	56920	semaphorin 3G(SEMA3G)
M_111	389840	mitogen-activated protein kinase kinase kinase
M_111	2028	glutamyl aminopeptidase(ENPEP)
M_111	3270	histidine rich calcium binding protein(HRC)
M_111	375189	profilin family member 4(PFN4)
M_111	7036	transferrin receptor 2(TFR2)
M_111	92162	transmembrane protein 88(TMEM88)
M_111	81569	actin like 8(ACTL8)
M_111	27136	MORC family CW-type zinc finger 1(MORC1)
M_111	23532	preferentially expressed antigen in
M_111	6665	SRY-box 15(SOX15)
M_111	64180	dipeptidase 3(DPEP3)
M_111	125972	calreticulin 3(CALR3)
M_111	641372	acyl-CoA thioesterase 6(ACOT6)
M_111	133584	EGF like, fibronectin type III and laminin G domains(EGFLAM)
M_111	347688	tubulin beta 8 class VIII(TUBB8)

M_111	344967	acyl-CoA thioesterase 7 pseudogene(LOC344967)
M_111	723809	LHFPL3 antisense RNA 2(LHFPL3-AS2)
M_111	650655	ATP binding cassette subfamily A member 17, pseudogene(ABCA17P)
M_111	375612	lipoma HMGIC fusion partner-like 3(LHFPL3)
M_111	494188	F-box protein 47(FBXO47)
M_111	197021	lactase like(LCTL)
M_111	1592	cytochrome P450 family 26 subfamily A member 1(CYP26A1)
M_111	51050	peptidase inhibitor 15(PI15)
M_111	401262	cysteine rich protein 3(CRIP3)
M_111	3645	insulin receptor related receptor(INSRR)
M_111	154386	long intergenic non-protein coding RNA
M_111	22953	purinergic receptor P2X 2(P2RX2)
M_111	6329	sodium voltage-gated channel alpha subunit
M_111	646999	akirin 1 pseudogene(LOC646999)
M_111	9496	T-box 4(TBX4)
M_111	254158	chromosome X open reading frame 58(CXorf58)
M_111	4914	neurotrophic receptor tyrosine kinase 1(NTRK1)
M_112	100132929	protein FAM25(FAM25BP)
M_112	442245	glutathione S-transferase mu 2 pseudogene
M_112	1446	casein alpha s1(CSN1S1)
M_112	1046	caudal type homeobox 4(CDX4)
M_112	5225	progastricsin(PGC)
M_112	728224	keratin associated protein 4-8(KRTAP4-8)
M_112	81851	keratin associated protein 1-1(KRTAP1-1)
M_112	319103	small nucleolar RNA, C/D box 8(SNORD8)
M_112	140258	keratin associated protein 13-1(KRTAP13-1)
M_112	157724	solute carrier family 7 member 13(SLC7A13)
M_112	126541	olfactory receptor family 10 subfamily H member
M_112	392390	olfactory receptor family 1 subfamily L member
M_112	3443	interferon alpha 6(IFNA6)

M_112	401138	amelotin(AMTN)
M_112	4589	mucin 7, secreted(MUC7)
M_112	135941	olfactory receptor family 2 subfamily A member
M_112	504191	olfactory receptor family 9 subfamily G member
M_112	1447	casein beta(CSN2)
M_112	10879	submaxillary gland androgen regulated protein
M_112	3347	histatin 3(HTN3)
M_112	441864	T cell-interacting, activating receptor on myeloid cells 1(TARM1)
M_112	283365	olfactory receptor family 6 subfamily C member
M_112	1527	testis expressed 28(TEX28)
M_112	641451	small nucleolar RNA, H/ACA box 19(SNORA19)
M_112	341418	olfactory receptor family 6 subfamily C member
M_112	131375	lysozyme like 4(LYZL4)
M_112	439934	long intergenic non-protein coding RNA
M_112	644444	transmembrane protein 30C,
M_112	2948	glutathione S-transferase mu 4(GSTM4)
M_112	3346	histatin 1(HTN1)
M_112	54212	syntrophin gamma 1(SNTG1)
M_112	26952	submaxillary gland androgen regulated protein
M_112	85481	protein serine kinase H2(PSKH2)
M_112	203076	chromosome 8 open reading frame 74(C8orf74)
M_112	2946	glutathione S-transferase mu 2(GSTM2)
M_112	283189	olfactory receptor family 9 subfamily G member
M_112	390199	olfactory receptor family 4 subfamily D member
M_112	219968	olfactory receptor family 5 subfamily B member
M_112	2944	glutathione S-transferase mu 1(GSTM1)
M_112	219981	olfactory receptor family 5 subfamily A member
M_112	403257	olfactory receptor family 4 subfamily C member
M_112	338674	olfactory receptor family 5 subfamily F member
M_112	399706	long intergenic non-protein coding RNA
M_112	390326	olfactory receptor family 6 subfamily C member

M_112	126402	coiled-coil domain containing 105(CCDC105)
M_112	386653	interleukin 31(IL31)
M_112	6336	sodium voltage-gated channel alpha subunit
M_112	26779	small nucleolar RNA, H/ACA box 69(SNORA69)
M_112	494118	SPANX family member N1(SPANXN1)
M_112	654322	small nucleolar RNA, H/ACA box 13(SNORA13)
M_112	155184	solute carrier family 2 member 7(SLC2A7)
M_112	2947	glutathione S-transferase mu 3(GSTM3)
M_112	677811	small nucleolar RNA, H/ACA box 28(SNORA28)
M_112	2830	G protein-coupled receptor 6(GPR6)
M_112	10637	left-right determination factor 1(LEFTY1)
M_112	390858	O-acyltransferase like, pseudogene(OACYLP)
M_112	58503	opiorphin prepropeptide(OPRPN)
M_112	55363	hemogen(HEMGN)
M_112	10803	C-C motif chemokine receptor 9(CCR9)
M_112	83902	keratin associated protein 17-1(KRTAP17-1)
M_112	150165	XK related 3(XKR3)
M_112	4295	motilin(MLN)
M_112	6779	statherin(STATH)
M_112	121256	transmembrane protein 132D(TMEM132D)
M_112	2538	glucose-6-phosphatase catalytic subunit(G6PC)
M_112	646113	long intergenic non-protein coding RNA
M_112	728655	hepatocellular carcinoma up-regulated long non-coding RNA(HULC)
M_112	677822	small nucleolar RNA, H/ACA box 40(SNORA40)
M_112	677820	small nucleolar RNA, H/ACA box 38(SNORA38)
M_112	677838	small nucleolar RNA, H/ACA box 61(SNORA61)
M_112	64066	matrix metallopeptidase 27(MMP27)
M_112	158062	lipocalin 6(LCN6)
M_112	344022	notochord homeobox(NOTO)
M_112	140679	solute carrier family 32 member 1(SLC32A1)
M_112	57824	histocompatibility minor HB-1(HMHB1)

M_112	283579	chromosome 14 open reading frame 178(C14orf178)
M_112	9576	sperm associated antigen 6(SPAG6)
M_113	5358	plastin 3(PLS3)
M_113	57633	leucine rich repeat neuronal 1(LRRN1)
M_113	57628	dipeptidyl peptidase like 10(DPP10)
M_113	55714	teneurin transmembrane protein 3(TENM3)
M_113	80816	additional sex combs like 3, transcriptional
M_113	83690	cysteine rich secretory protein LCCL domain containing 1(CRISPLD1)
M_113	222553	solute carrier family 35 member F1(SLC35F1)
M_113	84620	ST6 beta-galactoside alpha-2,6-sialyltransferase
M_113	10000	AKT serine/threonine kinase 3(AKT3)
M_113	2561	gamma-aminobutyric acid type A receptor beta2 subunit(GABRB2)
M_113	100192378	ZFHX4 antisense RNA 1(ZFHX4-AS1)
M_113	285696	uncharacterized LOC285696(LOC285696)
M_113	4045	limbic system-associated membrane protein(LSAMP)
M_113	90768	uncharacterized LOC90768(LOC90768)
M_113	3037	hyaluronan synthase 2(HAS2)
M_113	1837	dystrobrevin alpha(DTNA)
M_113	400940	uncharacterized LOC400940(LOC400940)
M_113	1993	ELAV like RNA binding protein 2(ELAVL2)
M_113	5587	protein kinase D1(PRKD1)
M_113	152330	contactin 4(CNTN4)
M_113	5569	protein kinase (cAMP-dependent, catalytic) inhibitor alpha(PKIA)
M_113	594842	HAS2 antisense RNA 1(HAS2-AS1)
M_113	257194	neuronal growth regulator 1(NEGR1)
M_113	5789	protein tyrosine phosphatase, receptor type D(PTPRD)
M_113	8854	aldehyde dehydrogenase 1 family member
M_113	27255	contactin 6(CNTN6)
M_113	6664	SRY-box 11(SOX11)

M_113	8710	serpin family B member 7(SERPINB7)
M_113	10409	brain abundant membrane attached signal protein
M_113	441061	membrane associated ring-CH-type finger
M_113	2690	growth hormone receptor(GHR)
M_113	80310	platelet derived growth factor D(PDGFD)
M_113	60468	BTB domain and CNC homolog 2(BACH2)
M_113	150622	long intergenic non-protein coding RNA
M_113	5167	ectonucleotide pyrophosphatase/phosphodiesterase
M_113	590	butyrylcholinesterase(BCHE)
M_113	57689	leucine rich repeat containing 4C(LRRC4C)
M_113	4685	neural cell adhesion molecule 2(NCAM2)
M_113	255743	nephronectin(NPNT)
M_113	22865	SLIT and NTRK like family member 3(SLITRK3)
M_113	151354	family with sequence similarity 84 member
M_113	79776	zinc finger homeobox 4(ZFHX4)
M_113	114788	CUB and Sushi multiple domains 3(CSMD3)
M_113	11107	PR/SET domain 5(PRDM5)
M_113	1600	DAB1, reelin adaptor protein(DAB1)
M_113	3355	5-hydroxytryptamine receptor 1F(HTR1F)
M_113	56776	formin 2(FMN2)
M_113	4325	matrix metallopeptidase 16(MMP16)
M_113	132625	ZFP42 zinc finger protein(ZFP42)
M_113	60401	ectodysplasin A2 receptor(EDA2R)
M_113	221935	sidekick cell adhesion molecule 1(SDK1)
M_113	1902	lysophosphatidic acid receptor 1(LPAR1)
M_113	5125	proprotein convertase subtilisin/kexin type 5(PCSK5)
M_113	92126	dermatan sulfate epimerase-like(DSEL)
M_113	83851	synaptotagmin 16(SYT16)
M_113	8654	phosphodiesterase 5A(PDE5A)
M_113	7461	CAP-Gly domain containing linker protein 2(CLIP2)
M_113	2173	fatty acid binding protein 7(FABP7)
M_113	4745	neural EGFL like 1(NELL1)

M_113	8715	nucleolar protein 4(NOL4)
M_113	9037	semaphorin 5A(SEMA5A)
M_113	442213	patched domain containing 4(PTCHD4)
M_113	64393	zinc finger matrin-type 3(ZMAT3)
M_114	656	bone morphogenetic protein 8b(BMP8B)
M_114	23499	microtubule-actin crosslinking factor 1(MACF1)
M_114	23225	nucleoporin 210(NUP210)
M_114	5792	protein tyrosine phosphatase, receptor type F(PTPRF)
M_114	9670	importin 13(IPO13)
M_114	192670	argonaute 4, RISC catalytic component(AGO4)
M_114	1802	DPH2 homolog(DPH2)
M_114	4520	metal regulatory transcription factor 1(MTF1)
M_114	6536	solute carrier family 6 member 9(SLC6A9)
M_114	8704	beta-1,4-galactosyltransferase 2(B4GALT2)
M_114	22887	forkhead box J3(FOXJ3)
M_114	26523	argonaute 1, RISC catalytic component(AGO1)
M_114	10465	peptidylprolyl isomerase H(PPIH)
M_114	79654	HECT domain E3 ubiquitin protein ligase
M_114	55624	protein O-linked mannose N-acetylglucosaminyltransferase 1 (beta 1,2-
M_114	127703	chromosome 1 open reading frame 216(C1orf216)
M_114	4595	mutY DNA glycosylase(MUTYH)
M_114	79639	transmembrane protein 53(TMEM53)
M_114	10327	aldo-keto reductase family 1 member A1(AKR1A1)
M_114	79729	SH3 domain containing 21(SH3D21)
M_114	4904	Y-box binding protein 1(YBX1)
M_114	10969	EBNA1 binding protein 2(EBNA1BP2)
M_114	79033	ERI1 exoribonuclease family member 3(ERI3)
M_114	440567	ubiquinol-cytochrome c reductase hinge protein like(UQCRHL)
M_114	112950	mediator complex subunit 8(MED8)
M_114	3633	inositol polyphosphate-5-phosphatase B(INPP5B)

M_114	54807	zinc finger protein 586(ZNF586)
M_114	643314	KIAA0754(KIAA0754)
M_114	79932	KIAA0319 like(KIAA0319L)
M_114	23154	neurochondrin(NCDN)
M_114	55182	ring finger protein 220(RNF220)
M_114	55929	DNA methyltransferase 1 associated protein
M_114	5052	peroxiredoxin 1(PRDX1)
M_114	22955	sex comb on midleg homolog 1 (Drosophila)(SCMH1)
M_114	51058	zinc finger protein 691(ZNF691)
M_114	10420	testis-specific kinase 2(TESK2)
M_114	79078	chromosome 1 open reading frame 50(C1orf50)
M_114	9682	lysine demethylase 4A(KDM4A)
M_114	7389	uroporphyrinogen decarboxylase(UROD)
M_114	7388	ubiquinol-cytochrome c reductase hinge
M_114	114034	target of EGR1, member 1 (nuclear)(TOE1)
M_114	9967	thyroid hormone receptor associated protein
M_114	81888	hydroxypyruvate isomerase (putative)(HYI)
M_114	8891	eukaryotic translation initiation factor 2B subunit gamma(EIF2B3)
M_114	6487	ST3 beta-galactoside alpha-2,3-sialyltransferase
M_114	353500	bone morphogenetic protein 8a(BMP8A)
M_114	10489	leucine rich repeat containing 41(LRRC41)
M_114	64834	ELOVL fatty acid elongase 1(ELOVL1)
M_114	533	ATPase H+ transporting V0 subunit b(ATP6V0B)
M_114	114625	erythroblast membrane associated protein (Scianna blood group)(ERMAP)
M_114	149473	coiled-coil domain containing 24(CCDC24)
M_114	374969	small vasohibin binding protein(SVBP)
M_114	79717	phosphopantothenoylcysteine synthetase(PPCS)
M_114	23334	seizure threshold 2 homolog (mouse)(SZT2)
M_114	339488	transcription factor AP-2 epsilon(TFAP2E)
M_115	7644	zinc finger protein 91(ZNF91)

M_115	7539	ZFP37 zinc finger protein(ZFP37)
M_115	440515	zinc finger protein 506(ZNF506)
M_115	1586	cytochrome P450 family 17 subfamily A member 1(CYP17A1)
M_115	171392	zinc finger protein 675(ZNF675)
M_115	9534	zinc finger protein 254(ZNF254)
M_115	80032	zinc finger protein 556(ZNF556)
M_115	94039	zinc finger protein 101(ZNF101)
M_115	353088	zinc finger protein 429(ZNF429)
M_115	56242	zinc finger protein 253(ZNF253)
M_115	7561	zinc finger protein 14(ZNF14)
M_115	7639	zinc finger protein 85(ZNF85)
M_115	7562	zinc finger protein 708(ZNF708)
M_115	7652	zinc finger protein 99(ZNF99)
M_115	80264	zinc finger protein 430(ZNF430)
M_115	7594	zinc finger protein 43(ZNF43)
M_115	148203	zinc finger protein 738(ZNF738)
M_115	148198	zinc finger protein 98(ZNF98)
M_115	148213	zinc finger protein 681(ZNF681)
M_115	148206	zinc finger protein 714(ZNF714)
M_115	199777	zinc finger protein 626(ZNF626)
M_115	91975	zinc finger protein 300(ZNF300)
M_115	91392	zinc finger protein 502(ZNF502)
M_115	115560	zinc finger protein 501(ZNF501)
M_115	442319	zinc finger protein 727(ZNF727)
M_115	134466	zinc finger protein 300 pseudogene 1(ZNF300P1)
M_115	163227	zinc finger protein 100(ZNF100)
M_115	7757	zinc finger protein 208(ZNF208)
M_115	441666	zinc finger protein 91 pseudogene(LOC441666)
M_115	57615	zinc finger protein 492(ZNF492)
M_115	163223	zinc finger protein 676(ZNF676)
M_115	81931	zinc finger protein 93(ZNF93)

M_115	221357	glutathione S-transferase alpha 5(GSTA5)
M_115	169834	zinc finger protein 883(ZNF883)
M_115	23620	neurotensin receptor 2(NTSR2)
M_115	170959	zinc finger protein 431(ZNF431)
M_115	284443	zinc finger protein 493(ZNF493)
M_115	2939	glutathione S-transferase alpha 2(GSTA2)
M_115	2938	glutathione S-transferase alpha 1(GSTA1)
M_115	100129842	zinc finger protein 737(ZNF737)
M_115	91120	zinc finger protein 682(ZNF682)
M_115	113835	zinc finger protein 257(ZNF257)
M_115	90649	zinc finger protein 486(ZNF486)
M_115	147741	zinc finger protein 560(ZNF560)
M_115	100101266	hepatitis A virus cellular receptor 1 pseudogene 1(HAVCR1P1)
M_116	875	cystathionine-beta-synthase(CBS)
M_116	843	caspase 10(CASP10)
M_116	653145	annexin A8(ANXA8)
M_116	9806	SPARC/osteonectin, cwcv and kazal like domains proteoglycan 2(SPOCK2)
M_116	1803	dipeptidyl peptidase 4(DPP4)
M_116	57118	calcium/calmodulin dependent protein kinase
M_116	10686	claudin 16(CLDN16)
M_116	4240	milk fat globule-EGF factor 8 protein(MFGE8)
M_116	244	#N/A
M_116	266977	adhesion G protein-coupled receptor F1(ADGRF1)
M_116	4233	MET proto-oncogene, receptor tyrosine kinase(MET)
M_116	1969	EPH receptor A2(EPHA2)
M_116	23650	tripartite motif containing 29(TRIM29)
M_116	286204	crumbs 2, cell polarity complex component(CRB2)
M_116	390	Rho family GTPase 3(RND3)
M_116	346389	MACC1, MET transcriptional regulator(MACC1)
M_116	7051	transglutaminase 1(TGM1)

M_116	53836	G protein-coupled receptor 87(GPR87)
M_116	3853	keratin 6A(KRT6A)
M_116	3854	keratin 6B(KRT6B)
M_116	85411	#N/A
M_116	10626	tripartite motif containing 16(TRIM16)
M_116	10769	polo like kinase 2(PLK2)
M_116	3909	laminin subunit alpha 3(LAMA3)
M_116	286887	keratin 6C(KRT6C)
M_116	824	calpain 2(CAPN2)
M_116	129807	neuraminidase 4(NEU4)
M_116	123879	defective in cullin neddylation 1 domain containing 3(DCUN1D3)
M_116	80740	lymphocyte antigen 6 complex, locus G6C(LY6G6C)
M_116	80739	chromosome 6 open reading frame 25(C6orf25)
M_116	10468	follistatin(FST)
M_116	3696	integrin subunit beta 8(ITGB8)
M_116	5493	periplakin(PPL)
M_116	728643	heterogeneous nuclear ribonucleoprotein A1 pseudogene 33(HNRNPA1P33)
M_116	143903	layilin(LAYN)
M_116	8809	interleukin 18 receptor 1(IL18R1)
M_116	345651	actin, beta like 2(ACTBL2)
M_116	144501	keratin 80(KRT80)
M_116	145864	hyaluronan and proteoglycan link protein 3(HAPLN3)
M_116	3675	integrin subunit alpha 3(ITGA3)
M_116	113146	AHNAK nucleoprotein 2(AHNAK2)
M_116	841	caspase 8(CASP8)
M_116	163702	interferon lambda receptor 1(IFNLR1)
M_116	857	caveolin 1(CAV1)
M_116	57636	Rho GTPase activating protein 23(ARHGAP23)
M_116	9213	xenotropic and polytropic retrovirus receptor 1(XPR1)
M_116	54438	glucose-fructose oxidoreductase domain containing

M_116	57149	LYR motif containing 1(LYRM1)
M_116	219833	chromosome 11 open reading frame 45(C11orf45)
M_116	51421	angiomotin like 2(AMOTL2)
M_116	3762	potassium voltage-gated channel subfamily J member 5(KCNJ5)
M_116	3554	interleukin 1 receptor type 1(IL1R1)
M_116	3556	interleukin 1 receptor accessory protein(IL1RAP)
M_116	3918	laminin subunit gamma 2(LAMC2)
M_116	3914	laminin subunit beta 3(LAMB3)
M_116	8808	interleukin 1 receptor like 2(IL1RL2)
M_116	2012	epithelial membrane protein 1(EMP1)
M_116	646	basonuclin 1(BNC1)
M_116	283070	#N/A
M_116	302	annexin A2(ANXA2)
M_116	304	annexin A2 pseudogene 2(ANXA2P2)
M_116	4065	lymphocyte antigen 75(LY75)
M_116	858	caveolin 2(CAV2)
M_116	1906	endothelin 1(EDN1)
M_116	79413	zinc finger BED-type containing 2(ZBED2)
M_116	79148	matrix metallopeptidase 28(MMP28)
M_116	147	adrenoceptor alpha 1B(ADRA1B)
M_116	3316	heat shock protein family B (small) member
M_116	26136	testin LIM domain protein(TES)
M_116	388115	chromosome 15 open reading frame 52(C15orf52)
M_116	1410	crystallin alpha B(CRYAB)
M_116	58985	interleukin 22 receptor subunit alpha 1(IL22RA1)
M_116	8837	CASP8 and FADD like apoptosis regulator(CFLAR)
M_116	7052	transglutaminase 2(TGM2)
M_116	64090	galactose-3-O-sulfotransferase 2(GAL3ST2)
M_116	9076	claudin 1(CLDN1)
M_116	113026	phospholipase C delta 3(PLCD3)
M_116	366	aquaporin 9(AQP9)

M_116	7348	uroplakin 1B(UPK1B)
M_116	389337	Rho guanine nucleotide exchange factor
M_116	222611	adhesion G protein-coupled receptor F2(ADGRF2)
M_116	26524	large tumor suppressor kinase 2(LATS2)
M_116	151009	long intergenic non-protein coding RNA
M_116	56241	sushi domain containing 2(SUSD2)
M_116	221393	adhesion G protein-coupled receptor F4(ADGRF4)
M_116	84687	protein phosphatase 1 regulatory subunit
M_116	10076	protein tyrosine phosphatase, receptor type U(PTPRU)
M_116	1730	diaphanous related formin 2(DIAPH2)
M_116	301	annexin A1(ANXA1)
M_116	2820	glycerol-3-phosphate dehydrogenase 2(GPD2)
M_116	8796	sciellin(SCEL)
M_116	79689	STEAP4 metalloreductase(STEAP4)
M_116	140685	zinc finger and BTB domain containing 46(ZBTB46)
M_116	5918	retinoic acid receptor responder 1(RARRES1)
M_116	53405	chloride intracellular channel 5(CLIC5)
M_116	53947	alpha 1,4-galactosyltransferase(A4GALT)
M_116	29935	replication protein A4(RPA4)
M_116	2034	endothelial PAS domain protein 1(EPAS1)
M_116	169611	olfactomedin like 2A(OLFML2A)
M_116	29775	caspase recruitment domain family member
M_116	4929	nuclear receptor subfamily 4 group A member
M_116	7980	tissue factor pathway inhibitor 2(TFPI2)
M_116	3694	integrin subunit beta 6(ITGB6)
M_116	51315	lysine rich coiled-coil 1(KRCC1)
M_116	127294	myomesin 3(MYOM3)
M_116	10095	actin related protein 2/3 complex subunit
M_116	7851	mal, T-cell differentiation protein like(MALL)
M_116	7498	xanthine dehydrogenase(XDH)
M_116	374286	CMT1A duplicated region transcript 1(CDRT1)
M_116	8528	D-aspartate oxidase(DDO)

M_116	100126791	eosinophil granule ontogeny transcript (non-protein coding)(EGOT)
M_116	57520	HECT, C2 and WW domain containing E3 ubiquitin protein ligase 2(HECW2)
M_116	11181	trehalase(TREH)
M_116	145474	uncharacterized LOC145474(LOC145474)
M_116	26548	integrin subunit beta 1 binding protein 2(ITGB1BP2)
M_116	56925	latexin(LXN)
M_117	23081	lysine demethylase 4C(KDM4C)
M_117	79956	endoplasmic reticulum metallopeptidase 1(ERMP1)
M_117	3902	lymphocyte activating 3(LAG3)
M_117	79886	caspase activity and apoptosis inhibitor 1(CAAP1)
M_117	9933	pumilio RNA binding family member 3(PUM3)
M_117	4507	methylthioadenosine phosphorylase(MTAP)
M_117	11262	SP140 nuclear body protein(SP140)
M_117	197259	mixed lineage kinase domain like(MLKL)
M_117	10171	RNA terminal phosphate cyclase like 1(RCL1)
M_117	81030	Z-DNA binding protein 1(ZBP1)
M_117	400759	guanylate binding protein 1 pseudogene 1(GBP1P1)
M_117	57462	KIAA1161(KIAA1161)
M_117	55667	DENN domain containing 4C(DENND4C)
M_117	6891	transporter 2, ATP binding cassette subfamily B member(TAP2)
M_117	6890	transporter 1, ATP binding cassette subfamily B member(TAP1)
M_117	55664	cell division cycle 37 like 1(CDC37L1)
M_117	115361	guanylate binding protein 4(GBP4)
M_117	5698	proteasome subunit beta 9(PSMB9)
M_117	3580	C-X-C motif chemokine receptor 2 pseudogene
M_117	5696	proteasome subunit beta 8(PSMB8)
M_117	29126	CD274 molecule(CD274)
M_117	50808	adenylate kinase 3(AK3)

M_117	55064	spermatogenesis associated 6 like(SPATA6L)
M_117	55871	COBW domain containing 1(CBWD1)
M_117	54801	HAUS augmin like complex subunit 6(HAUS6)
M_117	3659	interferon regulatory factor 1(IRF1)
M_117	2633	guanylate binding protein 1(GBP1)
M_117	116071	basic leucine zipper ATF-like transcription factor
M_117	10670	Ras related GTP binding A(RRAGA)
M_117	340485	alkaline ceramidase 2(ACER2)
M_117	340481	zinc finger DHHC-type containing 21(ZDHHC21)
M_117	158219	tetratricopeptide repeat domain 39B(TTC39B)
M_117	4781	nuclear factor I B(NFIB)
M_117	90871	transmembrane protein 261(TMEM261)
M_117	158358	KIAA2026(KIAA2026)
M_117	84166	NLR family CARD domain containing 5(NLRC5)
M_117	6619	small nuclear RNA activating complex polypeptide 3(SNAPC3)
M_117	5991	regulatory factor X3(RFX3)
M_117	3136	major histocompatibility complex, class I, H (pseudogene)(HLA-H)
M_117	3134	major histocompatibility complex, class I, F(HLA-F)
M_117	3133	major histocompatibility complex, class I, E(HLA-E)
M_117	54914	focadhesin(FOCAD)
M_117	3717	Janus kinase 2(JAK2)
M_117	3620	indoleamine 2,3-dioxygenase 1(IDO1)
M_117	6373	C-X-C motif chemokine ligand 11(CXCL11)
M_117	80830	apolipoprotein L6(APOL6)
M_117	80833	apolipoprotein L3(APOL3)
M_117	23189	KN motif and ankyrin repeat domains 1(KANK1)
M_117	352961	HLA complex group 26 (non-protein coding)(HCG26)
M_117	9373	phospholipase A2 activating protein(PLAA)
M_117	203238	coiled-coil domain containing 171(CCDC171)
M_117	401494	3-hydroxyacyl-CoA dehydratase 4(HACD4)

M_117	403313	phospholipid phosphatase 6(PLPP6)
M_117	203228	chromosome 9 open reading frame 72(C9orf72)
M_117	57589	RIC1 homolog, RAB6A GEF complex partner
M_117	115362	guanylate binding protein 5(GBP5)
M_117	2634	guanylate binding protein 2(GBP2)
M_117	115426	ubiquitin like with PHD and ring finger domains
M_117	6355	C-C motif chemokine ligand 8(CCL8)
M_117	3627	C-X-C motif chemokine ligand 10(CXCL10)
M_117	54875	centlein(CNTLN)
M_117	169522	potassium voltage-gated channel modifier subfamily V member 2(KCNV2)
M_117	55958	kelch like family member 9(KLHL9)
M_117	10866	HLA complex P5 (non-protein coding)(HCP5)
M_117	11168	PC4 and SFRS1 interacting protein 1(PSIP1)
M_117	94240	epithelial stromal interaction 1(EPSTI1)
M_117	26953	RAN binding protein 6(RANBP6)
M_117	3105	major histocompatibility complex, class I, A(HLA-A)
M_117	3106	major histocompatibility complex, class I, B(HLA-B)
M_117	10346	tripartite motif containing 22(TRIM22)
M_117	64922	leucine rich repeat containing 19(LRRC19)
M_117	55848	plasminogen receptor with a C-terminal
M_117	51513	ETS variant 7(ETV7)
M_117	80173	intraflagellar transport 74(IFT74)
M_117	952	CD38 molecule(CD38)
M_117	51198	CDKN2A antisense RNA 1 (head to head)(CDKN2A-
M_117	6892	TAP binding protein(TAPBP)
M_117	629	complement factor B(CFB)
M_117	1029	cyclin dependent kinase inhibitor 2A(CDKN2A)
M_117	1030	cyclin dependent kinase inhibitor 2B(CDKN2B)
M_117	2683	beta-1,4-galactosyltransferase 1(B4GALT1)
M_117	57552	neutral cholesterol ester hydrolase 1(NCEH1)
M_117	100048912	CDKN2B antisense RNA 1(CDKN2B-AS1)

M_117	84102	solute carrier family 41 member 2(SLC41A2)
M_117	2298	forkhead box D4(FOXD4)
M_117	717	complement C2(C2)
M_117	1031	cyclin dependent kinase inhibitor 2C(CDKN2C)
M_117	653404	forkhead box D4-like 6(FOXD4L6)
M_117	6347	C-C motif chemokine ligand 2(CCL2)
M_117	6348	C-C motif chemokine ligand 3(CCL3)
M_117	3553	interleukin 1 beta(IL1B)
M_117	8743	tumor necrosis factor superfamily member
M_117	6351	C-C motif chemokine ligand 4(CCL4)
M_117	597	BCL2 related protein A1(BCL2A1)
M_117	200350	forkhead box D4-like 1(FOXD4L1)
M_117	5008	oncostatin M(OSM)
M_117	100036519	#N/A
M_117	6696	secreted phosphoprotein 1(SPP1)
M_117	728	complement C5a receptor 1(C5AR1)
M_117	54210	triggering receptor expressed on myeloid cells
M_117	6556	solute carrier family 11 member 1(SLC11A1)
M_117	5996	regulator of G-protein signaling 1(RGS1)
M_117	23601	C-type lectin domain family 5 member A(CLEC5A)
M_117	353514	leukocyte immunoglobulin like receptor A5(LILRA5)
M_117	1880	G protein-coupled receptor 183(GPR183)
M_117	8685	macrophage receptor with collagenous
M_117	2358	formyl peptide receptor 2(FPR2)
M_118	23328	SAM and SH3 domain containing 1(SASH1)
M_118	390212	G protein-coupled receptor 152(GPR152)
M_118	11128	RNA polymerase III subunit A(POLR3A)
M_118	261729	STEAP2 metalloreductase(STEAP2)
M_118	91683	synaptotagmin 12(SYT12)
M_118	4948	OCA2 melanosomal transmembrane protein(OCA2)
M_118	100131551	long intergenic non-protein coding RNA
M_118	128553	teashirt zinc finger homeobox 2(TSHZ2)

M_118	727897	mucin 5B, oligomeric mucus/gel-forming(MUC5B)
M_118	7414	vinculin(VCL)
M_118	6840	supervillin(SVIL)
M_118	3098	hexokinase 1(HK1)
M_118	3240	haptoglobin(HP)
M_118	11217	A-kinase anchoring protein 2(AKAP2)
M_118	445815	PALM2-AKAP2 readthrough(PALM2-AKAP2)
M_118	1809	dihydropyrimidinase like 3(DPYSL3)
M_118	92747	BPI fold containing family B member 1(BPIFB1)
M_118	85450	inositol 1,4,5-trisphosphate receptor interacting protein(ITPRIP)
M_118	115	adenylate cyclase 9(ADCY9)
M_118	9231	discs large MAGUK scaffold protein 5(DLG5)
M_118	6595	SWI/SNF related, matrix associated, actin dependent regulator of chromatin, subfamily a, member
M_118	5047	progestagen associated endometrial protein(PAEP)
M_118	26872	STEAP family member 1(STEAP1)
M_118	57010	calcium binding protein 4(CABP4)
M_118	90627	StAR related lipid transfer domain containing
M_118	320	amyloid beta precursor protein binding family A member 1(APBA1)
M_118	22874	pleckstrin homology domain containing
M_118	84159	AT-rich interaction domain 5B(ARID5B)
M_118	10580	sorbin and SH3 domain containing 1(SORBS1)
M_118	9749	phosphatase and actin regulator 2(PHACTR2)
M_118	9963	solute carrier family 23 member 1(SLC23A1)
M_118	5129	cyclin dependent kinase 18(CDK18)
M_118	6505	solute carrier family 1 member 1(SLC1A1)
M_118	3250	haptoglobin-related protein(HPR)
M_118	389333	proline rich basic protein 1(PROB1)
M_118	145781	GRINL1A complex locus 1(GCOM1)
M_118	8609	Kruppel like factor 7(KLF7)

M_118	753	low density lipoprotein receptor class A domain containing 4(LDLRAD4)
M_118	3075	complement factor H(CFH)
M_118	83593	Ras association domain family member 5(RASSF5)
M_118	6196	ribosomal protein S6 kinase A2(RPS6KA2)
M_118	4638	myosin light chain kinase(MYLK)
M_118	11145	phospholipase A2 group XVI(PLA2G16)
M_118	342618	schlafen family member 14(SLFN14)
M_118	1755	deleted in malignant brain tumors 1(DMBT1)
M_118	81492	radial spoke head 6 homolog A(RSPH6A)
M_118	5325	PLAG1 like zinc finger 1(PLAGL1)
M_118	1956	epidermal growth factor receptor(EGFR)
M_118	1612	death associated protein kinase 1(DAPK1)
M_118	5795	protein tyrosine phosphatase, receptor type J(PTPRJ)
M_118	284612	synaptophysin like 2(SYPL2)
M_118	195977	anthrax toxin receptor-like(ANTXRL)
M_118	10643	insulin like growth factor 2 mRNA binding protein
M_118	11254	solute carrier family 6 member 14(SLC6A14)
M_118	5336	phospholipase C gamma 2(PLCG2)
M_118	27086	forkhead box P1(FOXP1)
M_118	259236	transmembrane inner ear(TMIE)
M_118	51161	chromosome 3 open reading frame 18(C3orf18)
M_118	57061	hydatidiform mole associated and imprinted (non-protein coding)(HYMAI)
M_119	27291	R3H domain and coiled-coil containing 1
M_119	23223	ribosomal RNA processing 12 homolog(RRP12)
M_119	51684	SUFU negative regulator of hedgehog
M_119	9859	centrosomal protein 170(CEP170)
M_119	23082	peroxisome proliferator-activated receptor gamma, coactivator-related 1(PPRC1)
M_119	54536	exocyst complex component 6(EXOC6)
M_119	23063	WAPL cohesin release factor(WAPL)

M_119	9849	zinc finger protein 518A(ZNF518A)
M_119	339263	chromosome 17 open reading frame 51(C17orf51)
M_119	10121	ARP1 actin-related protein 1 homolog A, centractin alpha(ACTR1A)
M_119	55719	SMC5-SMC6 complex localization factor 2(SLF2)
M_119	9819	TSC22 domain family member 2(TSC22D2)
M_119	54838	WW domain binding protein 1-like(WBP1L)
M_119	9221	nucleolar and coiled-body phosphoprotein 1(NOLC1)
M_119	22978	5'-nucleotidase, cytosolic II(NT5C2)
M_119	22984	programmed cell death 11(PDCD11)
M_119	64318	NOC3 like DNA replication regulator(NOC3L)
M_119	1147	conserved helix-loop-helix ubiquitous kinase(CHUK)
M_119	6877	TATA-box binding protein associated factor 5(TAF5)
M_119	84445	leucine zipper tumor suppressor 2(LZTS2)
M_119	79847	major facilitator superfamily domain containing 13A(MFSD13A)
M_119	10613	ER lipid raft associated 1(ERLIN1)
M_119	1355	COX15, cytochrome c oxidase assembly
M_119	54708	membrane associated ring-CH-type finger
M_119	26148	chromosome 10 open reading frame 12(C10orf12)
M_119	64210	MMS19 homolog, cytosolic iron-sulfur assembly component(MMS19)
M_119	55568	polypeptide N-acetylgalactosaminyltransferase
M_119	79803	HPS6, biogenesis of lysosomal organelles complex 2 subunit 3(HPS6)
M_119	8945	beta-transducin repeat containing E3 ubiquitin protein ligase(BTRC)
M_119	55361	phosphatidylinositol 4-kinase type 2 alpha(PI4K2A)
M_119	26123	tectonic family member 3(TCTN3)
M_119	54619	cyclin J(CCNJ)
M_119	84986	Rho GTPase activating protein 19(ARHGAP19)
M_119	206358	solute carrier family 36 member 1(SLC36A1)

M_119	118813	zinc finger FYVE-type containing 27(ZFYVE27)
M_119	5832	aldehyde dehydrogenase 18 family member
M_119	80351	tankyrase 2(TNKS2)
M_119	84287	zinc finger DHHC-type containing 16(ZDHHC16)
M_119	55704	coiled-coil domain containing 88A(CCDC88A)
M_119	8861	LIM domain binding 1(LDB1)
M_119	79591	chromosome 10 open reading frame 76(C10orf76)
M_119	159371	solute carrier family 35 member G1(SLC35G1)
M_119	27333	golgi integral membrane protein 4(GOLIM4)
M_119	54462	coiled-coil serine rich protein 2(CCSER2)
M_119	56889	transmembrane 9 superfamily member 3(TM9SF3)
M_119	84108	polycomb group ring finger 6(PCGF6)
M_119	3416	insulin degrading enzyme(IDE)
M_119	84912	solute carrier family 35 member B4(SLC35B4)
M_119	57089	ectonucleoside triphosphate diphosphohydrolase
M_119	9748	STE20 like kinase(SLK)
M_119	84458	ligand dependent nuclear receptor corepressor(LCOR)
M_119	57600	folliculin interacting protein 2(FNIP2)
M_119	81603	tripartite motif containing 8(TRIM8)
M_119	8729	golgi brefeldin A resistant guanine nucleotide exchange factor 1(GBF1)
M_119	1112	forkhead box N3(FOXN3)
M_119	55662	hypoxia inducible factor 1 alpha subunit
M_119	953	ectonucleoside triphosphate diphosphohydrolase
M_119	54805	cyclin and CBS domain divalent metal cation transport mediator 2(CNNM2)
M_119	4791	nuclear factor kappa B subunit 2(NFKB2)
M_119	84333	polycomb group ring finger 5(PCGF5)
M_119	79946	chromosome 10 open reading frame 95(C10orf95)
M_120	347862	Parkinson disease 7 domain containing 1(PDDC1)
M_120	6194	ribosomal protein S6(RPS6)
M_120	6187	ribosomal protein S2(RPS2)

M_120	6189	ribosomal protein S3A(RPS3A)
M_120	1933	eukaryotic translation elongation factor 1 beta
M_120	441951	ZNFX1 antisense RNA 1(ZFAS1)
M_120	8665	eukaryotic translation initiation factor 3 subunit
M_120	79751	solute carrier family 25 member 22(SLC25A22)
M_120	6165	ribosomal protein L35a(RPL35A)
M_120	5441	RNA polymerase II subunit L(POLR2L)
M_120	54472	toll interacting protein(TOLLIP)
M_120	6138	ribosomal protein L15(RPL15)
M_120	6137	ribosomal protein L13(RPL13)
M_120	6134	ribosomal protein L10(RPL10)
M_120	6136	ribosomal protein L12(RPL12)
M_120	6888	transaldolase 1(TALDO1)
M_120	6128	ribosomal protein L6(RPL6)
M_120	256329	lamin tail domain containing 2(LMNTD2)
M_120	3265	HRas proto-oncogene, GTPase(HRAS)
M_120	4666	nascent polypeptide-associated complex alpha
M_120	6171	ribosomal protein L41(RPL41)
M_120	8045	Ras association domain family member 7(RASSF7)
M_120	25873	ribosomal protein L36(RPL36)
M_120	9045	ribosomal protein L14(RPL14)
M_120	5719	proteasome 26S subunit, non-ATPase 13(PSMD13)
M_120	59307	single Ig and TIR domain containing(SIGIRR)
M_120	6188	ribosomal protein S3(RPS3)
M_120	50854	chromosome 6 open reading frame 48(C6orf48)
M_120	10078	tumor suppressing subtransferable candidate
M_120	1937	eukaryotic translation elongation factor 1
M_120	6150	mitochondrial ribosomal protein L23(MRPL23)
M_120	6124	ribosomal protein L4(RPL4)
M_120	6881	TATA-box binding protein associated factor
M_120	6175	ribosomal protein lateral stalk subunit P0(RPLP0)
M_120	6176	ribosomal protein lateral stalk subunit P1(RPLP1)

M_120	6167	ribosomal protein L37(RPL37)
M_120	6168	ribosomal protein L37a(RPL37A)
M_120	6169	ribosomal protein L38(RPL38)
M_120	6170	ribosomal protein L39(RPL39)
M_120	8192	caseinolytic mitochondrial matrix peptidase proteolytic subunit(CLPP)
M_120	283232	transmembrane protein 80(TMEM80)
M_120	6224	ribosomal protein S20(RPS20)
M_120	60674	growth arrest specific 5 (non-protein coding)(GAS5)
M_120	10522	DEAF1, transcription factor(DEAF1)
M_120	6154	ribosomal protein L26(RPL26)
M_120	977	CD151 molecule (Raph blood group)(CD151)
M_120	3615	inosine monophosphate dehydrogenase 2(IMPDH2)
M_120	975	CD81 molecule(CD81)
M_120	63875	mitochondrial ribosomal protein L17(MRPL17)
M_120	4736	ribosomal protein L10a(RPL10A)
M_120	161	adaptor related protein complex 2 alpha 2
M_120	6208	ribosomal protein S14(RPS14)
M_120	6209	ribosomal protein S15(RPS15)
M_120	6207	ribosomal protein S13(RPS13)
M_120	6218	ribosomal protein S17(RPS17)
M_120	6222	ribosomal protein S18(RPS18)
M_120	6210	ribosomal protein S15a(RPS15A)
M_120	5204	prefoldin subunit 5(PFDN5)
M_120	6161	ribosomal protein L32(RPL32)
M_120	6160	ribosomal protein L31(RPL31)
M_120	6159	ribosomal protein L29(RPL29)
M_120	100093630	small nucleolar RNA host gene 8(SNHG8)
M_120	6204	ribosomal protein S10(RPS10)
M_120	125144	LRRC75A antisense RNA 1(LRRC75A-AS1)
M_120	51272	Bet1 golgi vesicular membrane trafficking protein
M_120	6166	ribosomal protein L36a like(RPL36AL)

M_120	57104	patatin like phospholipase domain containing
M_120	3921	ribosomal protein SA(RPSA)
M_120	27315	post-GPI attachment to proteins 2(PGAP2)
M_120	517	ATP synthase, H+ transporting, mitochondrial Fo complex subunit C2 (subunit 9)(ATP5G2)
M_120	125988	chromosome 19 open reading frame 70(C19orf70)
M_120	6232	ribosomal protein S27(RPS27)
M_120	6164	ribosomal protein L34(RPL34)
M_120	6233	ribosomal protein S27a(RPS27A)
M_120	6228	ribosomal protein S23(RPS23)
M_120	6227	ribosomal protein S21(RPS21)
M_120	6230	ribosomal protein S25(RPS25)
M_120	6229	ribosomal protein S24(RPS24)
M_120	6202	ribosomal protein S8(RPS8)
M_120	6201	ribosomal protein S7(RPS7)
M_120	6235	ribosomal protein S29(RPS29)
M_120	388524	ribosomal protein SA pseudogene 58(RPSAP58)
M_120	10399	receptor for activated C kinase 1(RACK1)
M_120	6050	ribonuclease/angiogenin inhibitor 1(RNH1)
M_120	114915	EPB41L4A antisense RNA 1(EPB41L4A-AS1)
M_120	23410	sirtuin 3(SIRT3)
M_120	6157	ribosomal protein L27a(RPL27A)
M_120	6142	ribosomal protein L18a(RPL18A)
M_120	6143	ribosomal protein L19(RPL19)
M_120	6147	ribosomal protein L23a(RPL23A)
M_120	66005	chitinase domain containing 1(CHID1)
M_120	6152	ribosomal protein L24(RPL24)
M_120	6155	ribosomal protein L27(RPL27)
M_120	84266	alkB homolog 7(ALKBH7)
M_120	126328	NADH:ubiquinone oxidoreductase subunit
M_120	60626	RIC8 guanine nucleotide exchange factor A(RIC8A)
M_120	81532	MOB kinase activator 2(MOB2)

M_120	5859	glutaminyl-tRNA synthetase(QARS)
M_120	9349	ribosomal protein L23(RPL23)
M_120	6125	ribosomal protein L5(RPL5)
M_120	81490	phosphatidylserine synthase 2(PTDSS2)
M_120	6181	ribosomal protein lateral stalk subunit P2(RPLP2)
M_120	56985	ADP-ribose/CDP-alcohol diphosphatase, manganese dependent(ADPRM)
M_120	6231	ribosomal protein S26(RPS26)
M_120	283120	H19, imprinted maternally expressed transcript (non-protein coding)(H19)
M_120	6135	ribosomal protein L11(RPL11)
M_120	1028	cyclin dependent kinase inhibitor 1C(CDKN1C)
M_120	55367	p53-induced death domain protein 1(PIDD1)
M_120	1509	cathepsin D(CTSD)
M_120	57661	PHD and ring finger domains 1(PHRF1)
M_120	641638	small nucleolar RNA host gene 6(SNHG6)
M_120	3784	potassium voltage-gated channel subfamily Q member 1(KCNQ1)
M_120	6206	ribosomal protein S12(RPS12)
M_120	6146	ribosomal protein L22(RPL22)
M_120	441502	ribosomal protein S26 pseudogene 11(RPS26P11)
M_121	116151	family with sequence similarity 210 member
M_121	55902	acyl-CoA synthetase short-chain family member
M_121	149986	LSM family member 14B(LSM14B)
M_121	3911	laminin subunit alpha 5(LAMA5)
M_121	100287227	TIPARP antisense RNA 1(TIPARP-AS1)
M_121	23394	activity dependent neuroprotector homeobox(ADNP)
M_121	54345	SRY-box 18(SOX18)
M_121	11264	peroxisomal membrane protein 4(PXMP4)
M_121	8675	syntaxin 16(STX16)
M_121	55969	chromosome 20 open reading frame 24(C20orf24)
M_121	284739	long intergenic non-protein coding RNA

M_121	56731	SLC2A4 regulator(SLC2A4RG)
M_121	11054	opioid growth factor receptor(OGFR)
M_121	7165	tumor protein D52 like 2(TPD52L2)
M_121	9978	ring-box 1(RBX1)
M_121	128866	charged multivesicular body protein 4B(CHMP4B)
M_121	1	alpha-1-B glycoprotein(A1BG)
M_121	9885	oxysterol binding protein like 2(OSBPL2)
M_121	389170	leucine, glutamate and lysine rich 1(LEKR1)
M_121	140823	reactive oxygen species modulator 1(ROMO1)
M_121	51282	SCAN domain containing 1(SCAND1)
M_121	54994	GID complex subunit 8 homolog(GID8)
M_121	57473	zinc finger protein 512B(ZNF512B)
M_121	51750	regulator of telomere elongation helicase 1(RTEL1)
M_121	140700	sterile alpha motif domain containing 10(SAMD10)
M_121	140701	abhydrolase domain containing 16B(ABHD16B)
M_121	55734	ZFP64 zinc finger protein(ZFP64)
M_121	5688	proteasome subunit alpha 7(PSMA7)
M_121	80331	DnaJ heat shock protein family (Hsp40) member C5(DNAJC5)
M_121	57403	RAB22A, member RAS oncogene family(RAB22A)
M_121	26164	mitochondrial ribosome associated GTPase 2(MTG2)
M_121	22913	RALY heterogeneous nuclear
M_121	51012	PRELI domain containing 3B(PRELID3B)
M_121	79716	aminopeptidase-like 1(NPEPL1)
M_121	10139	ADP ribosylation factor related protein 1(ARFRP1)
M_121	11047	adhesion regulating molecule 1(ADRM1)
M_121	6919	transcription elongation factor A2(TCEA2)
M_121	81928	Cdk5 and Abl enzyme substrate 2(CABLES2)
M_121	9217	VAMP associated protein B and C(VAPB)
M_121	83658	dynein light chain roadblock-type 1(DYNLRB1)
M_121	84619	zinc finger CCCH-type and G-patch domain containing(ZGPAT)

M_121	2778	GNAS complex locus(GNAS)
M_121	10732	transcription factor like 5(TCFL5)
M_121	5203	prefoldin subunit 4(PFDN4)
M_121	55738	ADP ribosylation factor GTPase activating protein 1(ARFGAP1)
M_121	6874	TATA-box binding protein associated factor 4(TAF4)
M_121	503538	A1BG antisense RNA 1(A1BG-AS1)
M_121	8894	eukaryotic translation initiation factor 2 subunit
M_121	55544	RNA binding motif protein 38(RBM38)
M_121	51497	negative elongation factor complex member
M_121	26205	glucocorticoid modulatory element binding protein
M_121	514	ATP synthase, H+ transporting, mitochondrial F1 complex, epsilon subunit(ATP5E)
M_121	5509	protein phosphatase 1 regulatory subunit
M_121	100132911	diphthamide biosynthesis 3 pseudogene 1(DPH3P1)
M_121	26039	SS18L1, nBAF chromatin remodeling complex subunit(SS18L1)
M_121	24148	pre-mRNA processing factor 6(PRPF6)
M_121	79144	pancreatic progenitor cell differentiation and proliferation factor(PPDPF)
M_121	55257	MRG domain binding protein(MRGBP)
M_121	54963	uridine-cytidine kinase 1 like 1(UCKL1)
M_121	9825	spermatogenesis associated 2(SPATA2)
M_121	54915	YTH N6-methyladenosine RNA binding protein
M_121	6640	syntrophin alpha 1(SNTA1)
M_121	55251	protein-L-isoaspartate (D-aspartate) O-methyltransferase domain containing 2(PCMTD2)
M_121	4700	NADH:ubiquinone oxidoreductase subunit
M_121	63939	family with sequence similarity 217 member
M_121	84269	coiled-coil-helix-coiled-coil-helix domain containing 5(CHCHD5)
M_121	11083	death inducer-obliterator 1(DIDO1)

M_121	432369	ATP synthase, H+ transporting, mitochondrial F1 complex, epsilon subunit pseudogene 2(ATP5EP2)
M_121	57731	spectrin beta, non-erythrocytic 4(SPTBN4)
M_121	63941	N-terminal EF-hand calcium binding protein
M_121	169166	sorting nexin 31(SNX31)
M_121	80097	mitotic spindle organizing protein 2B(MZT2B)
M_121	55653	breast carcinoma amplified sequence 4(BCAS4)
M_121	92856	IMP4 homolog, U3 small nucleolar
M_121	64493	long intergenic non-protein coding RNA
M_121	10004	N-acetylated alpha-linked acidic dipeptidase like 1(NAALADL1)
M_121	100128076	protein tyrosine phosphatase
M_121	653784	mitotic spindle organizing protein 2A(MZT2A)
M_121	1368	carboxypeptidase M(CPM)
M_121	55	acid phosphatase, prostate(ACPP)
M_121	84905	zinc finger protein 341(ZNF341)
M_121	84317	coiled-coil domain containing 115(CCDC115)
M_121	100134368	uncharacterized LOC100134368(LOC100134368)
M_121	1573	cytochrome P450 family 2 subfamily J member
M_122	54508	EPB41L4A antisense RNA 2 (head to head)(EPB41L4A-AS2)
M_122	831	calpastatin(CAST)
M_122	27125	AF4/FMR2 family member 4(AFF4)
M_122	84832	ankyrin repeat domain 36B pseudogene
M_122	324	APC, WNT signaling pathway regulator(APC)
M_122	5229	protein geranylgeranyltransferase type I subunit beta(PGGT1B)
M_122	4124	mannosidase alpha class 2A member 1(MAN2A1)
M_122	9140	autophagy related 12(ATG12)
M_122	51014	transmembrane p24 trafficking protein 7(TMED7)
M_122	153241	centrosomal protein 120(CEP120)
M_122	10114	homeodomain interacting protein kinase 3(HIPK3)

M_122	6728	signal recognition particle 19(SRP19)
M_122	9867	praja ring finger ubiquitin ligase 2(PJA2)
M_122	51735	Rap guanine nucleotide exchange factor 6(RAPGEF6)
M_122	91137	solute carrier family 25 member 46(SLC25A46)
M_122	28966	sorting nexin 24(SNX24)
M_122	153443	serum response factor binding protein 1(SRFBP1)
M_122	64848	YTH domain containing 2(YTHDC2)
M_122	1456	casein kinase 1 gamma 3(CSNK1G3)
M_122	64839	F-box and leucine rich repeat protein 17(FBXL17)
M_122	51397	COMM domain containing 10(COMMD10)
M_122	167227	decapping mRNA 2(DCP2)
M_122	4012	leucyl and cystinyl aminopeptidase(LNPEP)
M_122	134429	StAR related lipid transfer domain containing
M_122	134430	WD repeat domain 36(WDR36)
M_122	11127	kinesin family member 3A(KIF3A)
M_122	153733	coiled-coil domain containing 112(CCDC112)
M_122	96459	folliculin interacting protein 1(FNIP1)
M_122	6558	solute carrier family 12 member 2(SLC12A2)
M_122	51808	phosphorylated adaptor for RNA export(PHAX)
M_122	285605	DTW domain containing 2(DTWD2)
M_122	133619	proline rich coiled-coil 1(PRRC1)
M_122	1176	adaptor related protein complex 3 sigma 1
M_122	64097	erythrocyte membrane protein band 4.1 like
M_122	6643	sorting nexin 2(SNX2)
M_122	7905	receptor accessory protein 5(REEP5)
M_122	51752	endoplasmic reticulum aminopeptidase 1(ERAP1)
M_122	5480	peptidylprolyl isomerase C(PPIC)
M_122	127933	U2AF homology motif kinase 1(UHMK1)
M_122	1657	Dmx like 1(DMXL1)
M_122	3572	interleukin 6 signal transducer(IL6ST)
M_122	501	aldehyde dehydrogenase 7 family member
M_122	10802	SEC24 homolog A, COPII coat complex

M_122	3295	hydroxysteroid 17-beta dehydrogenase 4(HSD17B4)
M_122	23176	septin 8(SEPT8)
M_122	2241	FER tyrosine kinase(FER)
M_122	56929	fem-1 homolog C(FEM1C)
M_122	375035	SFT2 domain containing 2(SFT2D2)
M_122	9095	T-box 19(TBX19)
M_122	26249	kelch like family member 3(KLHL3)
M_122	167410	limb and CNS expressed 1(LIX1)
M_122	65983	GRAM domain containing 3(GRAMD3)
M_122	1836	solute carrier family 26 member 2(SLC26A2)
M_122	4888	neuropeptide Y receptor Y6 (pseudogene)(NPY6R)
M_122	9499	myotilin(MYOT)
M_123	100170229	serine/arginine repetitive matrix 5(SRRM5)
M_123	342909	zinc finger protein 284(ZNF284)
M_123	340286	acyloxyacyl hydrolase (neutrophil)(FAM183BP)
M_123	9138	Rho guanine nucleotide exchange factor
M_123	650662	#N/A
M_123	2931	glycogen synthase kinase 3 alpha(GSK3A)
M_123	10849	CD3e molecule associated protein(CD3EAP)
M_123	401172	FLJ33360 protein(FLJ33360)
M_123	7673	zinc finger protein 222(ZNF222)
M_123	1762	dystrophia myotonica, WD repeat containing(DMWD)
M_123	56971	carcinoembryonic antigen related cell adhesion molecule 19(CEACAM19)
M_123	441425	ankyrin repeat domain 20 family member
M_123	353355	zinc finger protein 233(ZNF233)
M_123	9310	zinc finger protein 235(ZNF235)
M_123	1130	lysosomal trafficking regulator(LYST)
M_123	5817	poliovirus receptor(PVR)
M_123	162979	zinc finger protein 296(ZNF296)
M_123	7770	zinc finger protein 227(ZNF227)
M_123	100144604	long intergenic non-protein coding RNA

M_123	7768	zinc finger protein 225(ZNF225)
M_123	56006	SMG9, nonsense mediated mRNA decay
M_123	79820	cation channel sperm associated auxiliary subunit beta(CATSPERB)
M_123	7733	zinc finger protein 180(ZNF180)
M_123	400451	family with sequence similarity 174 member
M_123	6625	small nuclear ribonucleoprotein U1 subunit
M_123	8189	symplekin(SYMPK)
M_123	10472	zinc finger and BTB domain containing 18(ZBTB18)
M_123	2068	ERCC excision repair 2, TFIIH core complex helicase subunit(ERCC2)
M_123	3293	hydroxysteroid 17-beta dehydrogenase 3(HSD17B3)
M_123	80207	optic atrophy 3 (autosomal recessive, with chorea and spastic paraplegia)(OPA3)
M_123	10780	zinc finger protein 234(ZNF234)
M_123	414301	DNA damage inducible 1 homolog 1(DDI1)
M_123	200010	solute carrier family 5 member 9(SLC5A9)
M_123	388552	biogenesis of lysosomal organelles complex 1 subunit 3(BLOC1S3)
M_123	5819	nectin cell adhesion molecule 2(NECTIN2)
M_123	7772	zinc finger protein 229(ZNF229)
M_123	7773	zinc finger protein 230(ZNF230)
M_123	284349	zinc finger protein 283(ZNF283)
M_123	7771	zinc finger protein 112(ZNF112)
M_123	388550	carcinoembryonic antigen related cell adhesion molecule 22, pseudogene(CEACAM22P)
M_123	7769	zinc finger protein 226(ZNF226)
M_123	153770	PLAC8 like 1(PLAC8L1)
M_123	57787	microtubule affinity regulating kinase 4(MARK4)
M_123	11129	CLK4 associating serine/arginine rich
M_123	26974	zinc finger protein 285(ZNF285)
M_123	7596	zinc finger protein 45(ZNF45)

M_123	126298	immunity related GTPase Q(IRGQ)
M_123	64763	zinc finger protein 574(ZNF574)
M_123	7711	zinc finger protein 155(ZNF155)
M_123	126299	zinc finger protein 428(ZNF428)
M_123	1209	CLPTM1, transmembrane protein(CLPTM1)
M_123	440307	tubulin tyrosine ligase like 13, pseudogene(TTLL13P)
M_123	116115	zinc finger protein 526(ZNF526)
M_123	26579	myeloma overexpressed(MYEOV)
M_123	7515	X-ray repair cross complementing 1(XRCC1)
M_123	653583	pleckstrin homology like domain family B member 3(PHLDB3)
M_123	100132948	family with sequence similarity 27 member
M_123	342908	zinc finger protein 404(ZNF404)
M_123	1760	dystrophia myotonica protein kinase(DMPK)
M_123	100133036	family with sequence similarity 95 member
M_123	728747	ankyrin repeat domain 20 family member
M_123	93953	acidic repeat containing(ACRC)
M_123	23403	F-box protein 46(FBXO46)
M_123	23461	ATP binding cassette subfamily A member 5(ABCA5)
M_123	153684	uncharacterized LOC153684(LOC153684)
M_123	124404	septin 12(SEPT12)
M_123	7766	zinc finger protein 223(ZNF223)
M_123	729171	ankyrin repeat domain 20 family member A8, pseudogene(ANKRD20A8P)
M_123	10452	translocase of outer mitochondrial membrane
M_123	10737	RFPL3 antisense(RFPL3S)
M_123	389289	annexin A2 receptor(ANXA2R)
M_123	147912	SIX homeobox 5(SIX5)
M_123	548321	#N/A
M_123	9389	solute carrier family 22 member 14(SLC22A14)
M_123	7767	zinc finger protein 224(ZNF224)
M_123	130749	carboxypeptidase O(CPO)

M_123	26145	interferon regulatory factor 2 binding protein
M_123	7638	zinc finger protein 221(ZNF221)
M_123	51086	TNNI3 interacting kinase(TNNI3K)
M_123	401027	chromosome 2 open reading frame 66(C2orf66)
M_123	646658	synapse differentiation inducing 1 like(SYNDIG1L)
M_123	9390	solute carrier family 22 member 13(SLC22A13)
M_123	5172	solute carrier family 26 member 4(SLC26A4)
M_123	213	albumin(ALB)
M_123	3781	potassium calcium-activated channel subfamily N member 2(KCNN2)
M_123	374786	EF-hand calcium binding domain 5(EFCAB5)
M_123	286002	SLC26A4 antisense RNA 1(SLC26A4-AS1)
M_123	59340	histamine receptor H4(HRH4)
M_124	374955	spermatogenesis associated 21(SPATA21)
M_124	442191	olfactory receptor family 14 subfamily J member
M_124	84648	late cornified envelope 3D(LCE3D)
M_124	140732	Sad1 and UNC84 domain containing 5(SUN5)
M_124	120065	olfactory receptor family 5 subfamily P member
M_124	54544	cysteine rich C-terminal 1(CRCT1)
M_124	219431	olfactory receptor family 4 subfamily S member
M_124	100129792	coiled-coil domain containing 152(CCDC152)
M_124	340357	uncharacterized LOC340357(LOC340357)
M_124	1539	cylicin 2(CYLC2)
M_124	353145	late cornified envelope 3E(LCE3E)
M_124	245935	defensin beta 122 (pseudogene)(DEFB122)
M_124	353144	late cornified envelope 3C(LCE3C)
M_124	83896	keratin associated protein 3-1(KRTAP3-1)
M_124	389898	ubiquitin conjugating enzyme E2 N like (gene/pseudogene)(UBE2NL)
M_124	9038	trace amine associated receptor 5(TAAR5)
M_124	219428	olfactory receptor family 4 subfamily C member 16 (gene/pseudogene)(OR4C16)

M_124	159119	heat shock transcription factor, Y-linked 2(HSFY2)
M_124	6706	small proline rich protein 2G(SPRR2G)
M_124	494197	SPANX family member N5(SPANXN5)
M_124	3888	keratin 82(KRT82)
M_124	151242	protein phosphatase 1 regulatory inhibitor subunit 1C(PPP1R1C)
M_124	653545	double homeobox 4 like 5(DUX4L5)
M_124	390162	olfactory receptor family 5 subfamily M member
M_124	79339	olfactory receptor family 51 subfamily B member
M_124	440563	heterogeneous nuclear ribonucleoprotein C-like 2(HNRNPCL2)
M_124	282763	olfactory receptor family 51 subfamily B member
M_124	79345	olfactory receptor family 51 subfamily B member 2 (gene/pseudogene)(OR51B2)
M_124	448834	keratinocyte proline rich protein(KPRP)
M_124	343069	heterogeneous nuclear ribonucleoprotein C-like 1(HNRNPCL1)
M_124	7441	pre-B lymphocyte 1(VPREB1)
M_124	5275	serpin family B member 13(SERPINB13)
M_124	4991	olfactory receptor family 1 subfamily D member
M_124	6414	selenoprotein P(SELENOP)
M_124	390059	olfactory receptor family 51 subfamily M member
M_124	401136	TMPRSS11B N-terminal like,
M_124	126637	trichohyalin like 1(TCHHL1)
M_124	126638	repetin(RPTN)
M_124	203611	chromodomain Y-linked 2B(CDY2B)
M_124	390231	tripartite motif containing 77(TRIM77)
M_124	26339	olfactory receptor family 5 subfamily K member
M_124	692157	small nucleolar RNA, H/ACA box 16B(SNORA16B)
M_124	390142	olfactory receptor family 5 subfamily D member 13 (gene/pseudogene)(OR5D13)
M_124	391114	olfactory receptor family 6 subfamily K member

M_124	643161	family with sequence similarity 25 member
M_124	3046	hemoglobin subunit epsilon 1(HBE1)
M_124	350383	G protein-coupled receptor 142(GPR142)
M_124	153571	chromosome 5 open reading frame 38(C5orf38)
M_124	646498	chromosome 3 open reading frame 84(C3orf84)
M_124	219287	APC membrane recruitment protein 2(AMER2)
M_124	677780	small Cajal body-specific RNA 11(SCARNA11)
M_124	677793	small nucleolar RNA, H/ACA box 2A(SNORA2A)
M_124	677827	small nucleolar RNA, H/ACA box 46(SNORA46)
M_124	9758	FERM and PDZ domain containing 4(FRMPD4)
M_124	441308	olfactory receptor family 4 subfamily F member
M_124	9248	G protein-coupled receptor 50(GPR50)
M_124	886	cholecystokinin A receptor(CCKAR)
M_124	153572	iroquois homeobox 2(IRX2)
M_124	353141	late cornified envelope 2D(LCE2D)
M_124	337880	keratin associated protein 11-1(KRTAP11-1)
M_124	2267	fibrinogen like 1(FGL1)
M_124	339240	keratin 17 pseudogene 5(KRT17P5)
M_124	150160	chaperonin containing TCP1 subunit 8 like
M_125	54949	succinate dehydrogenase complex assembly factor 2(SDHAF2)
M_125	280636	selenoprotein H(SELENOH)
M_125	6749	structure specific recognition protein 1(SSRP1)
M_125	1642	damage specific DNA binding protein 1(DDB1)
M_125	10482	nuclear RNA export factor 1(NXF1)
M_125	5007	oxysterol binding protein(OSBP)
M_125	51075	thioredoxin related transmembrane protein 2(TMX2)
M_125	9219	metastasis associated 1 family member 2(MTA2)
M_125	80829	ZFP91 zinc finger protein(ZFP91)
M_125	5553	proteoglycan 2, pro eosinophil major basic
M_125	29015	solute carrier family 43 member 3(SLC43A3)

M_125	219988	PAT1 homolog 1, processing body mRNA decay factor(PATL1)
M_125	349667	reticulon 4 receptor-like 2(RTN4RL2)
M_125	220002	cytochrome b561 family member A3(CYB561A3)
M_125	84447	synoviolin 1(SYVN1)
M_125	79080	coiled-coil domain containing 86(CCDC86)
M_125	56834	G protein-coupled receptor 137(GPR137)
M_125	283237	tetratricopeptide repeat domain 9C(TTC9C)
M_125	6809	syntaxin 3(STX3)
M_125	399909	pecanex homolog 3 (Drosophila)(PCNX3)
M_125	1500	catenin delta 1(CTNND1)
M_125	25921	zinc finger DHHC-type containing 5(ZDHHC5)
M_125	746	transmembrane protein 258(TMEM258)
M_125	22992	lysine demethylase 2A(KDM2A)
M_125	51524	transmembrane protein 138(TMEM138)
M_125	3710	inositol 1,4,5-trisphosphate receptor type 3(ITPR3)
M_125	2011	microtubule affinity regulating kinase 2(MARK2)
M_125	26580	BSCL2, seipin lipid droplet biogenesis
M_125	26007	triokinase and FMN cyclase(TKFC)
M_125	4221	menin 1(MEN1)
M_125	221092	heterogeneous nuclear ribonucleoprotein U like 2(HNRNPUL2)
M_125	1270	ciliary neurotrophic factor(CNTF)
M_125	79869	cleavage and polyadenylation specific factor
M_125	26519	translocase of inner mitochondrial membrane
M_125	10629	TATA-box binding protein associated factor 6
M_125	85456	tankyrase 1 binding protein 1(TNKS1BP1)
M_125	23193	glucosidase II alpha subunit(GANAB)
M_125	54948	mitochondrial ribosomal protein L16(MRPL16)
M_125	10313	reticulon 3(RTN3)
M_125	79829	N(alpha)-acetyltransferase 40, NatD catalytic subunit(NAA40)

M_125	27339	pre-mRNA processing factor 19(PRPF19)
M_125	79842	zinc finger and BTB domain containing 3(ZBTB3)
M_125	63901	family with sequence similarity 111 member
M_125	160851	diacylglycerol kinase eta(DGKH)
M_125	55690	phosphofurin acidic cluster sorting protein 1(PACS1)
M_125	10978	cleavage and polyadenylation factor I subunit
M_125	219541	mediator complex subunit 19(MED19)
M_125	79073	transmembrane protein 109(TMEM109)
M_125	23130	autophagy related 2A(ATG2A)
M_125	51259	transmembrane protein 216(TMEM216)
M_125	55048	VPS37C, ESCRT-I subunit(VPS37C)
M_125	6811	syntaxin 5(STX5)
M_125	64852	terminal uridylyl transferase 1, U6 snRNA-
M_125	26229	beta-1,3-glucuronyltransferase 3(B3GAT3)
M_125	80789	integrator complex subunit 5(INTS5)
M_125	10938	EH domain containing 1(EHD1)
M_125	8986	ribosomal protein S6 kinase A4(RPS6KA4)
M_125	91056	adaptor related protein complex 5 beta 1
M_125	53838	chromosome 11 open reading frame 24(C11orf24)
M_125	823	calpain 1(CAPN1)
M_125	5331	phospholipase C beta 3(PLCB3)
M_125	2785	G protein subunit gamma 3(GNG3)
M_125	10584	collectin subfamily member 10(COLEC10)
M_125	345222	Myb/SANT DNA binding domain containing
M_126	79157	major facilitator superfamily domain containing
M_126	54868	transmembrane protein 104(TMEM104)
M_126	146705	TEPSIN, adaptor related protein complex 4 accessory protein(TEPSIN)
M_126	146712	UDP-GlcNAc:betaGal beta-1,3-N-acetylglucosaminyltransferase-like 1(B3GNTL1)
M_126	57602	ubiquitin specific peptidase 36(USP36)

M_126	79701	2-oxoglutarate and iron dependent oxygenase domain containing 3(OGFOD3)
M_126	5986	RFNG O-fucosylpeptide 3-beta-N-acetylglucosaminyltransferase(RFNG)
M_126	64978	mitochondrial ribosomal protein L38(MRPL38)
M_126	6448	N-sulfoglucosamine sulfohydrolase(SGSH)
M_126	201255	leucine rich repeat containing 45(LRRC45)
M_126	84074	glutamine rich 2(QRICH2)
M_126	55666	NPL4 homolog, ubiquitin recognition
M_126	125058	TBC1 domain family member 16(TBC1D16)
M_126	22994	centrosomal protein 131(CEP131)
M_126	1453	casein kinase 1 delta(CSNK1D)
M_126	57513	CASK interacting protein 2(CASKIN2)
M_126	3607	forkhead box K2(FOXK2)
M_126	284004	hexosaminidase D(HEXDC)
M_126	284001	coiled-coil domain containing 57(CCDC57)
M_126	51181	dicarbonyl and L-xylulose reductase(DCXR)
M_126	51081	mitochondrial ribosomal protein S7(MRPS7)
M_126	79672	fructosamine 3 kinase related protein(FN3KRP)
M_126	51547	sirtuin 7(SIRT7)
M_126	677850	small nucleolar RNA, C/D box 1C(SNORD1C)
M_126	9146	hepatocyte growth factor-regulated tyrosine kinase substrate(HGS)
M_126	10476	ATP synthase, H+ transporting, mitochondrial Fo complex subunit D(ATP5H)
M_126	79092	caspase recruitment domain family member
M_126	3396	mitochondrial ribosomal protein L58(MRPL58)
M_126	8632	dynein axonemal heavy chain 17(DNAH17)
M_126	64772	endo-beta-N-acetylglucosaminidase(ENGASE)
M_126	64122	fructosamine 3 kinase(FN3K)
M_126	396	Rho GDP dissociation inhibitor alpha(ARHGDIA)
M_126	440465	BAIAP2 antisense RNA 1 (head to head)(BAIAP2-

M_126	124512	methyltransferase like 23(METTL23)
M_126	124565	solute carrier family 38 member 10(SLC38A10)
M_126	124583	calcium activated nucleotidase 1(CANT1)
M_126	56270	WD repeat domain 45B(WDR45B)
M_126	339230	coiled-coil domain containing 137(CCDC137)
M_126	63893	ubiquitin conjugating enzyme E2 O(UBE2O)
M_126	79415	chromosome 17 open reading frame 62(C17orf62)
M_126	64118	dihydrouridine synthase 1 like(DUS1L)
M_126	4097	MAF bZIP transcription factor G(MAFG)
M_126	71	actin gamma 1(ACTG1)
M_126	9489	phosphatidylglycerophosphate synthase 1(PGS1)
M_126	5034	prolyl 4-hydroxylase subunit beta(P4HB)
M_126	6613	small ubiquitin-like modifier 2(SUMO2)
M_126	5833	phosphate cytidylyltransferase 2,
M_126	9267	cytohesin 1(CYTH1)
M_126	26502	nuclear prelamin A recognition factor(NARF)
M_126	2548	glucosidase alpha, acid(GAA)
M_126	9775	eukaryotic translation initiation factor 4A3(EIF4A3)
M_126	9772	transmembrane protein 94(TMEM94)
M_126	284131	endonuclease V(ENDOV)
M_126	284129	solute carrier family 26 member 11(SLC26A11)
M_126	5831	pyrroline-5-carboxylate reductase 1(PYCR1)
M_126	85451	unkempt family zinc finger(UNK)
M_126	51204	translational activator of cytochrome c oxidase
M_126	79643	charged multivesicular body protein 6(CHMP6)
M_126	80233	Fanconi anemia core complex associated protein 100(FAAP100)
M_126	1468	solute carrier family 25 member 10(SLC25A10)
M_126	6904	tubulin folding cofactor D(TBCD)
M_126	57521	regulatory associated protein of MTOR complex
M_126	654434	small nucleolar RNA host gene 20(SNHG20)

M_126	5881	ras-related C3 botulinum toxin substrate 3 (rho family, small GTP binding protein Rac3)(RAC3)
M_126	10966	RAB40B, member RAS oncogene family(RAB40B)
M_126	2194	fatty acid synthase(FASN)
M_126	57690	trinucleotide repeat containing 6C(TNRC6C)
M_126	57801	hes family bHLH transcription factor 4(HES4)
M_126	182	jagged 1(JAG1)
M_126	92659	MAFG antisense RNA 1 (head to head)(MAFG-AS1)
M_127	129642	membrane bound O-acyltransferase domain containing 2(MBOAT2)
M_127	10890	RAB10, member RAS oncogene family(RAB10)
M_127	6006	Rh blood group CcEe antigens(RHCE)
M_127	9784	sorting nexin 17(SNX17)
M_127	29959	nuclear receptor binding protein 1(NRBP1)
M_127	165324	UBX domain protein 2A(UBXN2A)
M_127	85465	selenoprotein I(SELENOI)
M_127	79833	gem nuclear organelle associated protein 6(GEMIN6)
M_127	51072	mediator of cell motility 1(MEMO1)
M_127	6683	spastin(SPAST)
M_127	130557	zinc finger protein 513(ZNF513)
M_127	10113	prolactin regulatory element binding(PREB)
M_127	84450	zinc finger protein 512(ZNF512)
M_127	11321	GPN-loop GTPase 1(GPN1)
M_127	9577	brain and reproductive organ-expressed (TNFRSF1A modulator)(BRE)
M_127	55006	tRNA methyltransferase 61B(TRMT61B)
M_127	79635	coiled-coil domain containing 121(CCDC121)
M_127	26751	SH3 and SYLF domain containing 1(SH3YL1)
M_127	253558	lysocardiolipin acyltransferase 1(LCLAT1)
M_127	11147	HERV-H LTR-associating 3(HHLA3)
M_127	51374	all-trans retinoic acid induced differentiation
M_127	644186	synaptonemal complex central element protein

M_127	1838	dystrobrevin beta(DTNB)
M_127	84661	dpy-30, histone methyltransferase complex regulatory subunit(DPY30)
M_127	347744	chromosome 6 open reading frame 52(C6orf52)
M_127	84791	long intergenic non-protein coding RNA
M_127	90271	oligodendrocyte maturation-associated long intergenic non-coding RNA(OLMALINC)
M_127	2976	general transcription factor IIIC subunit 2(GTF3C2)
M_127	64080	ribokinase(RBKS)
M_127	22950	solute carrier family 4 member 1 adaptor
M_127	60509	ATP/GTP binding protein like 5(AGBL5)
M_127	51639	splicing factor 3b subunit 6(SF3B6)
M_127	4358	MPV17, mitochondrial inner membrane
M_127	9913	SPT7-like STAGA complex gamma subunit(SUPT7L)
M_127	5496	protein phosphatase, Mg2+/Mn2+ dependent
M_127	55676	solute carrier family 30 member 6(SLC30A6)
M_127	8890	eukaryotic translation initiation factor 2B subunit delta(EIF2B4)
M_127	84272	Yip1 domain family member 4(YIPF4)
M_127	8884	solute carrier family 5 member 6(SLC5A6)
M_127	64342	HCLS1 binding protein 3(HS1BP3)
M_127	51112	trafficking protein particle complex 12(TRAPPC12)
M_127	3030	hydroxyacyl-CoA dehydrogenase/3-ketoacyl-CoA thiolase/enoyl-CoA hydratase (trifunctional protein), alpha subunit(HADHA)
M_127	3032	hydroxyacyl-CoA dehydrogenase/3-ketoacyl-CoA thiolase/enoyl-CoA hydratase (trifunctional protein), beta subunit(HADHB)
M_127	7349	urocortin(UCN)
M_127	245711	speedy/RINGO cell cycle regulator family member A(SPDYA)
M_127	2498	ferritin heavy chain 1 pseudogene 3(FTH1P3)

M_127	642273	family with sequence similarity 110 member
M_127	55277	FGGY carbohydrate kinase domain
M_127	84226	chromosome 2 open reading frame 16(C2orf16)
M_127	92292	glycine-N-acyltransferase like 1(GLYATL1)
M_128	389123	IQ motif containing F2(IQCF2)
M_128	58510	proline dehydrogenase 2(PRODH2)
M_128	3642	INSM transcriptional repressor 1(INSM1)
M_128	6754	somatostatin receptor 4(SSTR4)
M_128	23436	chymotrypsin like elastase family member
M_128	26762	hepatitis A virus cellular receptor 1(HAVCR1)
M_128	81615	transmembrane protein 163(TMEM163)
M_128	84366	prostate cancer susceptibility candidate 1(PRAC1)
M_128	4224	meprin A subunit alpha(MEP1A)
M_128	402381	spermatogenesis and oogenesis specific basic helix-loop-helix 1(SOHLH1)
M_128	8022	LIM homeobox 3(LHX3)
M_128	2877	glutathione peroxidase 2(GPX2)
M_128	390265	olfactory receptor family 10 subfamily G member
M_128	11330	chymotrypsin C(CTRC)
M_128	486	FXYD domain containing ion transport regulator
M_128	408029	chromosome 2 open reading frame 27B(C2orf27B)
M_128	55600	intelectin 1(ITLN1)
M_128	26285	claudin 17(CLDN17)
M_128	7369	uromodulin(UMOD)
M_128	6315	ATXN8 opposite strand (non-protein
M_128	116123	flavin containing monooxygenase 9
M_128	390433	olfactory receptor family 4 subfamily K member
M_128	55998	nuclear RNA export factor 5(NXF5)
M_128	152065	chromosome 3 open reading frame 22(C3orf22)
M_128	83656	FAM167A antisense RNA 1(FAM167A-AS1)
M_128	7080	NK2 homeobox 1(NKX2-1)
M_128	1444	chorionic somatomammotropin hormone like

M_128	63978	PR/SET domain 14(PRDM14)
M_128	5077	paired box 3(PAX3)
M_128	3698	inter-alpha-trypsin inhibitor heavy chain 2(ITIH2)
M_128	6822	sulfotransferase family 2A member 1(SULT2A1)
M_128	55539	KCNQ1 downstream neighbor (non-protein coding)(KCNQ1DN)
M_128	337972	keratin associated protein 19-5(KRTAP19-5)
M_128	26648	olfactory receptor family 7 subfamily E member
M_128	100151643	keratin associated protein 20-4(KRTAP20-4)
M_128	142683	intelectin 2(ITLN2)
M_128	84873	adhesion G protein-coupled receptor G7(ADGRG7)
M_128	440051	keratin associated protein 5-11(KRTAP5-11)
M_128	728409	long intergenic non-protein coding RNA
M_128	10136	chymotrypsin like elastase family member
M_128	9626	guanylate cyclase activator 1C(GUCA1C)
M_128	4985	opioid receptor delta 1(OPRD1)
M_128	93107	potassium voltage-gated channel modifier subfamily G member 4(KCNG4)
M_128	6701	small proline rich protein 2B(SPRR2B)
M_128	401036	ankyrin repeat and SOCS box containing 18(ASB18)
M_128	81327	olfactory receptor family 4 subfamily A member
M_128	150763	glycerol-3-phosphate acyltransferase 2,
M_128	5456	POU class 3 homeobox 4(POU3F4)
M_128	7276	transthyretin(TTR)
M_128	166863	RNA binding motif protein 46(RBM46)
M_128	643226	glutaredoxin and cysteine rich domain containing
M_128	342898	syncollin(SYCN)
M_128	5406	pancreatic lipase(PNLIP)
M_128	253970	surfactant associated 3(SFTA3)
M_128	391518	VENT homeobox pseudogene 7(VENTXP7)
M_128	337	apolipoprotein A4(APOA4)
M_128	2813	glycoprotein 2(GP2)

M_128	222826	family with sequence similarity 217 member
M_128	345	apolipoprotein C3(APOC3)
M_128	729240	proline rich 20C(PRR20C)
M_128	57626	kelch like family member 1(KLHL1)
M_128	2691	growth hormone releasing hormone(GHRH)
M_128	10590	secretagogin, EF-hand calcium binding
M_128	346606	monoacylglycerol O-acyltransferase 3(MOGAT3)
M_128	255626	histone cluster 1 H2B family member a(HIST1H2BA)
M_128	151278	coiled-coil domain containing 140(CCDC140)
M_128	2638	GC, vitamin D binding protein(GC)
M_128	389812	lipocalin 15(LCN15)
M_128	50674	neurogenin 3(NEUROG3)
M_128	57127	Rh family B glycoprotein (gene/pseudogene)(RHBG)
M_128	116369	solute carrier family 26 member 8(SLC26A8)
M_128	94160	ATP binding cassette subfamily C member
M_128	2893	glutamate ionotropic receptor AMPA type subunit
M_128	10	N-acetyltransferase 2(NAT2)
M_128	284067	chromosome 17 open reading frame 105(C17orf105)
M_128	50945	T-box 22(TBX22)
M_128	206338	laeverin(LVRN)
M_128	1142	cholinergic receptor nicotinic beta 3
M_128	729238	surfactant protein A2(SFTPA2)
M_128	85445	contactin associated protein like 4(CNTNAP4)
M_128	131669	urocanate hydratase 1(UROC1)
M_128	793	calbindin 1(CALB1)
M_128	160728	solute carrier family 5 member 8(SLC5A8)
M_128	122876	glycoprotein hormone beta 5(GPHB5)
M_128	339302	complexin 4(CPLX4)
M_128	140458	ankyrin repeat and SOCS box containing 5(ASB5)
M_128	11085	ADAM metallopeptidase domain 30(ADAM30)
M_128	8170	solute carrier family 14 member 2(SLC14A2)
M_128	53336	CPX chromosome region, candidate 1(CPXCR1)

M_128	3078	complement factor H related 1(CFHR1)
M_128	480	ATPase Na+/K+ transporting subunit alpha
M_129	84879	major facilitator superfamily domain containing
M_129	343035	retinal degeneration 3(RD3)
M_129	1260	cyclic nucleotide gated channel alpha 2(CNGA2)
M_129	5948	retinol binding protein 2(RBP2)
M_129	284573	long intergenic non-protein coding RNA
M_129	127343	diencephalon/mesencephalon homeobox 1(DMBX1)
M_129	170689	ADAM metallopeptidase with thrombospondin type 1 motif 15(ADAMTS15)
M_129	4778	nuclear factor, erythroid 2(NFE2)
M_129	51046	ST8 alpha-N-acetyl-neuraminide alpha-2,8-sialyltransferase 3(ST8SIA3)
M_129	1473	cystatin D(CST5)
M_129	4487	msh homeobox 1(MSX1)
M_129	2652	opsin 1 (cone pigments), medium-wave-
M_129	3212	homeobox B2(HOXB2)
M_129	3213	homeobox B3(HOXB3)
M_129	3214	homeobox B4(HOXB4)
M_129	3215	homeobox B5(HOXB5)
M_129	3216	homeobox B6(HOXB6)
M_129	3217	homeobox B7(HOXB7)
M_129	6495	SIX homeobox 1(SIX1)
M_129	6493	single-minded family bHLH transcription factor
M_129	1014	cadherin 16(CDH16)
M_129	64096	GDNF family receptor alpha 4(GFRA4)
M_129	53407	syntaxin 18(STX18)
M_129	55885	LIM domain only 3(LMO3)
M_129	4308	transient receptor potential cation channel subfamily M member 1(TRPM1)
M_129	6236	RRAD, Ras related glycolysis inhibitor and calcium channel regulator(RRAD)

M_129	9480	one cut homeobox 2(ONECUT2)
M_129	60529	ALX homeobox 4(ALX4)
M_129	3211	homeobox B1(HOXB1)
M_129	168544	zinc finger protein 467(ZNF467)
M_129	51804	SIX homeobox 4(SIX4)
M_129	5956	opsin 1 (cone pigments), long-wave-
M_129	3219	homeobox B9(HOXB9)
M_129	3218	homeobox B8(HOXB8)
M_129	1470	cystatin SA(CST2)
M_129	9580	SRY-box 13(SOX13)
M_129	2043	EPH receptor A4(EPHA4)
M_129	1472	cystatin S(CST4)
M_129	8631	src kinase associated phosphoprotein 1(SKAP1)
M_129	145376	protein phosphatase 1 regulatory subunit
M_129	8613	phospholipid phosphatase 3(PLPP3)
M_129	29906	ST8 alpha-N-acetyl-neuraminide alpha-2,8-sialyltransferase 5(ST8SIA5)
M_129	1469	cystatin SN(CST1)
M_129	3362	5-hydroxytryptamine receptor 6(HTR6)
M_129	388646	guanylate binding protein 7(GBP7)
M_129	54716	solute carrier family 6 member 20(SLC6A20)
M_129	146395	GSG1 like(GSG1L)
M_129	55584	cholinergic receptor nicotinic alpha 9
M_129	6799	sulfotransferase family 1A member 2(SULT1A2)
M_129	317761	chromosome 14 open reading frame 39(C14orf39)
M_129	9435	carbohydrate sulfotransferase 2(CHST2)
M_129	9745	zinc finger protein 536(ZNF536)
M_129	81552	vesicular, overexpressed in cancer, prosurvival protein 1(VOPP1)
M_129	6817	sulfotransferase family 1A member 1(SULT1A1)
M_129	401399	proline rich transmembrane protein 4(PRRT4)
M_129	4990	SIX homeobox 6(SIX6)

M_129	2047	EPH receptor B1(EPHB1)
M_129	89	actinin alpha 3 (gene/pseudogene)(ACTN3)
M_130	4199	malic enzyme 1(ME1)
M_130	138241	chromosome 9 open reading frame 85(C9orf85)
M_130	401541	centromere protein P(CENPP)
M_130	195827	AhpC/TSA antioxidant enzyme domain containing
M_130	23204	ADP ribosylation factor like GTPase 6 interacting protein 1(ARL6IP1)
M_130	140738	transmembrane protein 37(TMEM37)
M_130	116028	RecQ mediated genome instability 2(RMI2)
M_130	23603	coronin 1C(CORO1C)
M_130	25934	nipsnap homolog 3A(NIPSNAP3A)
M_130	54881	testis expressed 10(TEX10)
M_130	80010	RecQ mediated genome instability 1(RMI1)
M_130	3376	isoleucyl-tRNA synthetase(IARS)
M_130	54981	nicotinamide riboside kinase 1(NMRK1)
M_130	2203	fructose-bisphosphatase 1(FBP1)
M_130	79048	SECIS binding protein 2(SECISBP2)
M_130	8208	chromatin assembly factor 1 subunit B(CHAF1B)
M_130	51280	golgi membrane protein 1(GOLM1)
M_130	7295	thioredoxin(TXN)
M_130	11046	solute carrier family 35 member D2(SLC35D2)
M_130	84270	caspase recruitment domain family member
M_130	81689	iron-sulfur cluster assembly 1(ISCA1)
M_130	81691	exonuclease NEF-sp(LOC81691)
M_130	2176	Fanconi anemia complementation group C(FANCC)
M_130	55071	chromosome 9 open reading frame 40(C9orf40)
M_130	10952	Sec61 translocon beta subunit(SEC61B)
M_130	55312	riboflavin kinase(RFK)
M_130	64420	sushi domain containing 1(SUSD1)
M_130	7046	transforming growth factor beta receptor 1(TGFBR1)
M_130	51104	abhydrolase domain containing 17B(ABHD17B)

M_130	26578	osteoclast stimulating factor 1(OSTF1)
M_130	29980	downstream neighbor of SON(DONSON)
M_130	116224	family with sequence similarity 122A(FAM122A)
M_130	414328	IDNK, gluconokinase(IDNK)
M_130	138639	protein tyrosine phosphatase domain containing
M_130	84693	methylmalonyl-CoA epimerase(MCEE)
M_130	22949	prostaglandin reductase 1(PTGR1)
M_130	22927	hyaluronan binding protein 4(HABP4)
M_130	549	AU RNA binding methylglutaconyl-CoA
M_130	3190	heterogeneous nuclear ribonucleoprotein
M_130	54534	mitochondrial ribosomal protein L50(MRPL50)
M_130	91283	Myb/SANT DNA binding domain containing
M_130	84267	chromosome 9 open reading frame 64(C9orf64)
M_130	80318	G kinase anchoring protein 1(GKAP1)
M_130	51531	tRNA methyltransferase O(TRMO)
M_130	5998	regulator of G-protein signaling 3(RGS3)
M_130	83744	zinc finger protein 484(ZNF484)
M_130	23424	tudor domain containing 7(TDRD7)
M_130	4814	ninjurin 1(NINJ1)
M_130	389293	iron-sulfur cluster assembly 1 pseudogene
M_130	2395	frataxin(FXN)
M_130	216	aldehyde dehydrogenase 1 family member
M_130	63826	serine racemase(SRR)
M_130	7378	uridine phosphorylase 1(UPP1)
M_130	23500	dishevelled associated activator of morphogenesis
M_130	8395	phosphatidylinositol-4-phosphate 5-kinase type 1 beta(PIP5K1B)
M_130	5239	phosphoglucomutase 5(PGM5)
M_130	9123	solute carrier family 16 member 3(SLC16A3)
M_130	8555	cell division cycle 14B(CDC14B)
M_130	1514	cathepsin L(CTSL)
M_130	572558	PGM5 antisense RNA 1(PGM5-AS1)

M_130	22822	pleckstrin homology like domain family A member 1(PHLDA1)
M_130	4337	molybdenum cofactor synthesis 1(MOCS1)
M_130	168448	cell division cycle 14C, pseuodgene(CDC14C)
M_131	91860	calmodulin like 4(CALML4)
M_131	23060	zinc finger protein 609(ZNF609)
M_131	54956	poly(ADP-ribose) polymerase family member
M_131	53944	casein kinase 1 gamma 1(CSNK1G1)
M_131	8554	protein inhibitor of activated STAT 1(PIAS1)
M_131	9187	solute carrier family 24 member 1(SLC24A1)
M_131	51324	spastic paraplegia 21 (autosomal recessive, Mast syndrome)(SPG21)
M_131	10302	small nuclear RNA activating complex polypeptide 5(SNAPC5)
M_131	54832	vacuolar protein sorting 13 homolog C(VPS13C)
M_131	79856	sorting nexin 22(SNX22)
M_131	10116	fem-1 homolog B(FEM1B)
M_131	83660	talin 2(TLN2)
M_131	10363	high mobility group 20A(HMG20A)
M_131	8925	HECT and RLD domain containing E3 ubiquitin protein ligase family member 1(HERC1)
M_131	6642	sorting nexin 1(SNX1)
M_131	9325	thyroid hormone receptor interactor 4(TRIP4)
M_131	25820	ariadne RBR E3 ubiquitin protein ligase 1(ARIH1)
M_131	51495	3-hydroxyacyl-CoA dehydratase 3(HACD3)
M_131	123263	mitochondrial methionyl-tRNA
M_131	3658	iron responsive element binding protein 2(IREB2)
M_131	115752	DIS3 like exosome 3'-5' exoribonuclease(DIS3L)
M_131	49855	S-phase cyclin A associated protein in the
M_131	4649	myosin IXA(MYO9A)
M_131	54982	ceroid-lipofuscinosis, neuronal 6, late infantile,
M_131	54962	TIMELESS interacting protein(TIPIN)

M_131	4947	ornithine decarboxylase antizyme 2(OAZ2)
M_131	51065	ribosomal protein S27 like(RPS27L)
M_131	25942	SIN3 transcription regulator family member
M_131	10081	programmed cell death 7(PDCD7)
M_131	54878	dipeptidyl peptidase 8(DPP8)
M_131	5604	mitogen-activated protein kinase kinase 1(MAP2K1)
M_131	10845	caseinolytic mitochondrial matrix peptidase chaperone subunit(CLPX)
M_131	5607	mitogen-activated protein kinase kinase 5(MAP2K5)
M_131	84191	family with sequence similarity 96 member
M_131	5479	peptidylprolyl isomerase B(PPIB)
M_131	348094	ankyrin repeat and death domain containing
M_131	8125	acidic nuclear phosphoprotein 32 family member
M_131	2958	general transcription factor IIA subunit 2(GTF2A2)
M_131	79719	alpha- and gamma-adaptin binding protein(AAGAB)
M_131	10260	DENN domain containing 4A(DENND4A)
M_131	9960	ubiquitin specific peptidase 3(USP3)
M_131	8766	RAB11A, member RAS oncogene family(RAB11A)
M_131	81556	von Willebrand factor A domain containing 9(VWA9)
M_131	145853	chromosome 15 open reading frame 61(C15orf61)
M_131	5955	reticulocalbin 2(RCN2)
M_131	10099	tetraspanin 3(TSPAN3)
M_131	4756	neogenin 1(NEO1)
M_131	55055	zwilch kinetochore protein(ZWILCH)
M_131	55075	uveal autoantigen with coiled-coil domains and ankyrin repeats(UACA)
M_131	585	Bardet-Biedl syndrome 4(BBS4)
M_131	123228	SUMO/sentrin peptidase family member, NEDD8 specific(SENP8)
M_132	1606	diacylglycerol kinase alpha(DGKA)
M_132	10411	Rap guanine nucleotide exchange factor 3(RAPGEF3)
M_132	85441	helicase with zinc finger 2(HELZ2)

M_132	440044	solute carrier family 22 member 20(SLC22A20)
M_132	57658	calcium binding and coiled-coil domain
M_132	163590	torsin 1A interacting protein 2(TOR1AIP2)
M_132	57609	disco interacting protein 2 homolog B(DIP2B)
M_132	9924	PAN2 poly(A) specific ribonuclease subunit(PAN2)
M_132	23390	zinc finger DHHC-type containing 17(ZDHHC17)
M_132	103	adenosine deaminase, RNA specific(ADAR)
M_132	6773	signal transducer and activator of transcription
M_132	51134	centrosomal protein 83(CEP83)
M_132	51564	histone deacetylase 7(HDAC7)
M_132	138474	TATA-box binding protein associated factor 1
M_132	84926	SPRY domain containing 3(SPRYD3)
M_132	55652	solute carrier family 48 member 1(SLC48A1)
M_132	6872	TATA-box binding protein associated factor 1(TAF1)
M_132	121006	family with sequence similarity 186 member
M_132	11016	activating transcription factor 7(ATF7)
M_132	2804	golgin B1(GOLGB1)
M_132	9462	RAS protein activator like 2(RASAL2)
M_132	64375	IKAROS family zinc finger 4(IKZF4)
M_132	8909	endonuclease, poly(U) specific(ENDOU)
M_132	9881	tetratricopeptide repeat and ankyrin repeat containing 1(TRANK1)
M_132	80184	centrosomal protein 290(CEP290)
M_132	23215	proline rich coiled-coil 2C(PRRC2C)
M_132	85406	DnaJ heat shock protein family (Hsp40) member C14(DNAJC14)
M_132	10228	syntaxin 6(STX6)
M_132	283337	zinc finger protein 740(ZNF740)
M_132	283373	ankyrin repeat domain 52(ANKRD52)
M_132	6601	SWI/SNF related, matrix associated, actin dependent regulator of chromatin subfamily c member
M_132	546	ATRX, chromatin remodeler(ATRX)

M_132	5939	RNA binding motif single stranded interacting protein 2(RBMS2)
M_132	6602	SWI/SNF related, matrix associated, actin dependent regulator of chromatin, subfamily d, member
M_132	6667	Sp1 transcription factor(SP1)
M_132	57169	zinc finger NFX1-type containing 1(ZNFX1)
M_132	23041	MON2 homolog, regulator of endosome-to-Golgi trafficking(MON2)
M_132	10106	CTD small phosphatase 2(CTDSP2)
M_132	9826	Rho guanine nucleotide exchange factor
M_132	10896	oculomedin(OCLM)
M_132	9917	FAM20B, glycosaminoglycan
M_132	23468	chromobox 5(CBX5)
M_132	149041	ring finger and CCCH-type domains 1(RC3H1)
M_132	9857	centrosomal protein 350(CEP350)
M_132	27	ABL proto-oncogene 2, non-receptor tyrosine
M_132	57728	WD repeat domain 19(WDR19)
M_132	91	activin A receptor type 1B(ACVR1B)
M_132	114785	methyl-CpG binding domain protein 6(MBD6)
M_132	100129550	uncharacterized LOC100129550(LOC100129550)
M_132	121274	zinc finger protein 641(ZNF641)
M_132	57522	SLIT-ROBO Rho GTPase activating protein
M_132	904	cyclin T1(CCNT1)
M_132	9880	zinc finger and BTB domain containing 39(ZBTB39)
M_132	205717	upstream transcription factor family member 3(USF3)
M_132	80267	ER degradation enhancing alpha-mannosidase like protein 3(EDEM3)
M_132	11176	bromodomain adjacent to zinc finger domain
M_132	10956	OS9, endoplasmic reticulum lectin(OS9)
M_132	5916	retinoic acid receptor gamma(RARG)
M_132	22864	R3H domain containing 2(R3HDM2)
M_132	57674	ring finger protein 213(RNF213)

M_132	56829	zinc finger CCCH-type containing, antiviral
M_132	256987	serine incorporator 5(SERINC5)
M_133	150084	immunoglobulin superfamily member 5(IGSF5)
M_133	5241	progesterone receptor(PGR)
M_133	113457	tubulin alpha 3d(TUBA3D)
M_133	4166	carbohydrate sulfotransferase 6(CHST6)
M_133	5420	podocalyxin like(PODXL)
M_133	22941	SH3 and multiple ankyrin repeat domains
M_133	64321	SRY-box 17(SOX17)
M_133	7436	very low density lipoprotein receptor(VLDLR)
M_133	6542	solute carrier family 7 member 2(SLC7A2)
M_133	4988	opioid receptor mu 1(OPRM1)
M_133	164832	LON peptidase N-terminal domain and ring finger 2(LONRF2)
M_133	285733	#N/A
M_133	154043	CNKSR family member 3(CNKSR3)
M_133	90050	family with sequence similarity 181 member
M_133	114041	B3GALT5 antisense RNA 1(B3GALT5-AS1)
M_133	8204	nuclear receptor interacting protein 1(NRIP1)
M_133	91461	protein kinase domain containing,
M_133	64067	neuronal PAS domain protein 3(NPAS3)
M_133	2037	erythrocyte membrane protein band 4.1 like
M_133	401491	VLDLR antisense RNA 1(VLDLR-AS1)
M_133	4345	CD200 molecule(CD200)
M_133	401190	regulator of G-protein signaling 7 binding
M_133	374407	DnaJ heat shock protein family (Hsp40) member B13(DNAJB13)
M_133	9465	A-kinase anchoring protein 7(AKAP7)
M_133	283284	immunoglobulin superfamily member 22(IGSF22)
M_133	55107	anoctamin 1(ANO1)
M_133	27145	filamin A interacting protein 1(FILIP1)
M_133	57685	cache domain containing 1(CACHD1)

M_133	196383	Rab interacting lysosomal protein like 2(RILPL2)
M_133	283592	FAM181A antisense RNA 1(FAM181A-AS1)
M_133	112714	tubulin alpha 3e(TUBA3E)
M_133	26034	interaction protein for cytohesin exchange factors
M_133	57639	coiled-coil domain containing 146(CCDC146)
M_133	2781	G protein subunit alpha z(GNAZ)
M_133	6660	SRY-box 5(SOX5)
M_133	151888	B and T lymphocyte associated(BTLA)
M_133	367	androgen receptor(AR)
M_133	169792	GLIS family zinc finger 3(GLIS3)
M_133	5625	proline dehydrogenase 1(PRODH)
M_133	221662	RNA binding motif protein 24(RBM24)
M_133	117	ADCYAP receptor type I(ADCYAP1R1)
M_133	158471	prune homolog 2(PRUNE2)
M_133	3899	AF4/FMR2 family member 3(AFF3)
M_133	5923	Ras protein specific guanine nucleotide releasing factor 1(RASGRF1)
M_133	9687	growth regulation by estrogen in breast cancer
M_133	84465	multiple EGF like domains 11(MEGF11)
M_133	129049	small G protein signaling modulator 1(SGSM1)
M_133	10317	beta-1,3-galactosyltransferase 5(B3GALT5)
M_133	11197	WNT inhibitory factor 1(WIF1)
M_133	26220	DiGeorge syndrome critical region gene 5 (non-protein coding)(DGCR5)
M_133	50652	prostate cancer associated 3 (non-protein
M_133	112609	melanocortin 2 receptor accessory protein 2(MRAP2)
M_133	51196	phospholipase C epsilon 1(PLCE1)
M_133	388633	low density lipoprotein receptor class A domain containing 1(LDLRAD1)
M_133	9472	A-kinase anchoring protein 6(AKAP6)
M_133	55567	dynein axonemal heavy chain 3(DNAH3)
M_133	8842	prominin 1(PROM1)

M_133	58528	Ras related GTP binding D(RRAGD)
M_133	80005	dedicator of cytokinesis 5(DOCK5)
M_133	2650	glucosaminyl (N-acetyl) transferase 1, core 2(GCNT1)
M_133	90199	WAP four-disulfide core domain 8(WFDC8)
M_133	9120	solute carrier family 16 member 6(SLC16A6)
M_133	10681	G protein subunit beta 5(GNB5)
M_133	151473	solute carrier family 16 member 14(SLC16A14)
M_133	148145	long intergenic non-protein coding RNA
M_133	85452	cilia and flagella associated protein 74(CFAP74)
M_133	84623	kin of IRRE like 3 (Drosophila)(KIRREL3)
M_133	80201	hexokinase domain containing 1(HKDC1)
M_133	54102	chloride intracellular channel 6(CLIC6)
M_133	288	ankyrin 3(ANK3)
M_133	116135	leucine rich repeat containing 3B(LRRC3B)
M_133	145270	proline rich membrane anchor 1(PRIMA1)
M_133	222255	ataxin 7 like 1(ATXN7L1)
M_133	23576	dimethylarginine dimethylaminohydrolase 1(DDAH1)
M_133	25895	methyltransferase like 21B(METTL21B)
M_133	140870	WAP four-disulfide core domain 6(WFDC6)
M_133	22901	arylsulfatase G(ARSG)
M_133	57119	epididymal peptidase inhibitor(EPPIN)
M_133	10017	BCL2 like 10(BCL2L10)
M_133	10319	laminin subunit gamma 3(LAMC3)
M_133	284100	tyrosine 3-monooxygenase/tryptophan 5-monooxygenase activation protein epsilon pseudogene
M_133	63970	tumor protein p53 regulated apoptosis inducing protein 1(TP53AIP1)
M_134	170082	transcription elongation factor A N-terminal and central domain containing(TCEANC)
M_134	23114	neurofascin(NFASC)
M_134	284348	LY6/PLAUR domain containing 5(LYPD5)
M_134	283856	uncharacterized LOC283856(LOC283856)

M_134	4267	CD99 molecule(CD99)
M_134	273	amphiphysin(AMPH)
M_134	5931	RB binding protein 7, chromatin remodeling
M_134	2775	G protein subunit alpha o1(GNAO1)
M_134	91607	schlafen family member 11(SLFN11)
M_134	340591	carbonic anhydrase 5B pseudogene 1(CA5BP1)
M_134	6399	trafficking protein particle complex 2(TRAPPC2)
M_134	79710	MORC family CW-type zinc finger 4(MORC4)
M_134	10071	mucin 12, cell surface associated(MUC12)
M_134	283981	long intergenic non-protein coding RNA
M_134	8623	acetylserotonin O-methyltransferase-like(ASMTL)
M_134	8226	pseudouridine 5'-phosphatase(PUDP)
M_134	8227	A-kinase anchoring protein 17A(AKAP17A)
M_134	55787	taxilin gamma(TXLNG)
M_134	11043	midline 2(MID2)
M_134	1964	eukaryotic translation initiation factor 1A, X-
M_134	1968	eukaryotic translation initiation factor 2 subunit gamma(EIF2S3)
M_134	6567	solute carrier family 16 member 2(SLC16A2)
M_134	11238	carbonic anhydrase 5B(CA5B)
M_134	645369	transmembrane protein 200C(TMEM200C)
M_134	1831	TSC22 domain family member 3(TSC22D3)
M_134	10943	male-specific lethal 3 homolog (Drosophila)(MSL3)
M_134	80000	growth regulation by estrogen in breast cancer 1 like(GREB1L)
M_134	293	solute carrier family 25 member 6(SLC25A6)
M_134	28227	protein phosphatase 2 regulatory subunit
M_134	145567	tetratricopeptide repeat domain 7B(TTC7B)
M_134	207063	dehydrogenase/reductase X-linked(DHRSX)
M_134	414	arylsulfatase D(ARSD)
M_134	5277	phosphatidylinositol glycan anchor biosynthesis class
M_134	54960	gem nuclear organelle associated protein 8(GEMIN8)

M_134	56474	CTP synthase 2(CTPS2)
M_134	94056	synapse associated protein 1(SYAP1)
M_134	6646	sterol O-acyltransferase 1(SOAT1)
M_134	9189	zinc finger BED-type containing 1(ZBED1)
M_134	8481	OFD1, centriole and centriolar satellite protein(OFD1)
M_134	9495	A-kinase anchoring protein 5(AKAP5)
M_134	8233	zinc finger CCCH-type, RNA binding motif and serine/arginine rich 2(ZRSR2)
M_134	8228	patatin like phospholipase domain containing
M_134	881	calicin(CCIN)
M_134	57393	transmembrane protein 27(TMEM27)
M_134	55344	phosphatidylinositol specific phospholipase C X domain containing 1(PLCXD1)
M_134	80161	ASMTL antisense RNA 1(ASMTL-AS1)
M_134	8908	glycogenin 2(GYG2)
M_134	152007	GLI pathogenesis related 2(GLIPR2)
M_134	1123	chimerin 1(CHN1)
M_134	146857	schlafen family member 13(SLFN13)
M_134	23597	acyl-CoA thioesterase 9(ACOT9)
M_134	5613	protein kinase, X-linked(PRKX)
M_134	157983	chromosome 9 open reading frame 66(C9orf66)
M_134	10100	tetraspanin 2(TSPAN2)
M_134	149297	family with sequence similarity 78 member
M_134	2187	Fanconi anemia complementation group B(FANCB)
M_134	5256	phosphorylase kinase regulatory subunit alpha
M_134	80179	myosin XIX(MYO19)
M_134	4842	nitric oxide synthase 1(NOS1)
M_134	284098	phosphatidylinositol glycan anchor biosynthesis class W(PIGW)
M_134	25822	DnaJ heat shock protein family (Hsp40) member B5(DNAJB5)
M_134	319085	ITPK1 antisense RNA 1(ITPK1-AS1)

M_134	4803	nerve growth factor(NGF)
M_134	220042	DNA damage induced apoptosis suppressor(DDIAS)
M_134	1134	cholinergic receptor nicotinic alpha 1
M_135	5378	PMS1 homolog 1, mismatch repair system
M_135	151050	KAT8 regulatory NSL complex subunit 1
M_135	85461	tetratricopeptide repeat, ankyrin repeat and coiled-coil containing 1(TANC1)
M_135	205327	chromosome 2 open reading frame 69(C2orf69)
M_135	25843	MOB family member 4, phocein(MOB4)
M_135	79675	FAST kinase domains 1(FASTKD1)
M_135	4780	nuclear factor, erythroid 2 like 2(NFE2L2)
M_135	130535	potassium channel tetramerization domain containing 18(KCTD18)
M_135	23451	splicing factor 3b subunit 1(SF3B1)
M_135	130507	ubiquitin protein ligase E3 component n-recognin 3 (putative)(UBR3)
M_135	8520	histone acetyltransferase 1(HAT1)
M_135	55437	STE20-related kinase adaptor beta(STRADB)
M_135	79634	secernin 3(SCRN3)
M_135	10787	NCK associated protein 1(NCKAP1)
M_135	57703	CWC22 spliceosome associated protein
M_135	493911	phosphatase, orphan 2(PHOSPHO2)
M_135	9360	peptidylprolyl isomerase G(PPIG)
M_135	54529	asparagine synthetase domain containing 1(ASNSD1)
M_135	57679	ALS2, alsin Rho guanine nucleotide exchange
M_135	659	bone morphogenetic protein receptor type 2(BMPR2)
M_135	10314	LanC like 1(LANCL1)
M_135	220988	heterogeneous nuclear ribonucleoprotein
M_135	79828	methyltransferase like 8(METTL8)
M_135	94101	ORMDL sphingolipid biosynthesis regulator
M_135	66008	trafficking kinesin protein 2(TRAK2)
M_135	6670	Sp3 transcription factor(SP3)

M_135	285172	family with sequence similarity 126 member
M_135	133686	NAD kinase 2, mitochondrial(NADK2)
M_135	10213	proteasome 26S subunit, non-ATPase 14(PSMD14)
M_135	92104	tetratricopeptide repeat domain 30A(TTC30A)
M_135	92935	methionyl-tRNA synthetase 2,
M_135	9541	corepressor interacting with RBPJ, 1(CIR1)
M_135	10651	metaxin 2(MTX2)
M_135	8502	plakophilin 4(PKP4)
M_135	80856	lunapark, ER junction formation factor(LNPK)
M_135	8604	solute carrier family 25 member 12(SLC25A12)
M_135	57404	cytochrome P450 family 20 subfamily A member 1(CYP20A1)
M_135	129450	tRNA-yW synthesizing protein 5(TYW5)
M_135	142679	dual specificity phosphatase 19(DUSP19)
M_135	9669	eukaryotic translation initiation factor 5B(EIF5B)
M_135	253782	ceramide synthase 6(CERS6)
M_135	1385	cAMP responsive element binding protein 1(CREB1)
M_135	1386	activating transcription factor 2(ATF2)
M_135	80055	post-GPI attachment to proteins 1(PGAP1)
M_135	65977	pleckstrin homology domain containing
M_135	10152	abl interactor 2(ABI2)
M_135	10151	heterogeneous nuclear ribonucleoprotein A3 pseudogene 1(HNRNPA3P1)
M_135	130940	coiled-coil domain containing 148(CCDC148)
M_135	10477	ubiquitin conjugating enzyme E2 E3(UBE2E3)
M_135	79809	tetratricopeptide repeat domain 21B(TTC21B)
M_135	9631	nucleoporin 155(NUP155)
M_135	80219	coenzyme Q10B(COQ10B)
M_135	84128	WD repeat domain 75(WDR75)
M_135	29789	Obg like ATPase 1(OLA1)
M_135	6502	S-phase kinase associated protein 2(SKP2)
M_135	9689	basic leucine zipper and W2 domains 1(BZW1)

M_135	65062	transmembrane protein 237(TMEM237)
M_135	65065	neurobeachin like 1(NBEAL1)
M_135	9330	general transcription factor IIIC subunit 3(GTF3C3)
M_135	151230	kelch like family member 23(KLHL23)
M_135	8575	protein activator of interferon induced protein kinase EIF2AK2(PRKRA)
M_135	26003	golgi reassembly stacking protein 2(GORASP2)
M_135	254042	methionyl aminopeptidase type 1D,
M_135	150709	ankyrin and armadillo repeat containing(ANKAR)
M_135	150737	tetratricopeptide repeat domain 30B(TTC30B)
M_135	80067	DDB1 and CUL4 associated factor 17(DCAF17)
M_135	9874	tousled like kinase 1(TLK1)
M_135	6741	Sjogren syndrome antigen B(SSB)
M_135	6744	sperm specific antigen 2(SSFA2)
M_135	54431	DnaJ heat shock protein family (Hsp40) member C10(DNAJC10)
M_135	129401	nucleoporin 35(NUP35)
M_135	55854	zinc finger CCCH-type containing 15(ZC3H15)
M_135	26275	3-hydroxyisobutyryl-CoA hydrolase(HIBCH)
M_135	64172	O-sialoglycoprotein endopeptidase like 1(OSGEPL1)
M_135	1781	dynein cytoplasmic 1 intermediate chain 2(DYNC1I2)
M_135	54842	major facilitator superfamily domain containing
M_135	27098	clusterin like 1(CLUL1)
M_135	27314	RAB30, member RAS oncogene family(RAB30)
M_135	100131211	nuclear envelope integral membrane protein
M_135	129831	RNA binding motif protein 45(RBM45)
M_135	81567	thioredoxin domain containing 5(TXNDC5)
M_135	3685	integrin subunit alpha V(ITGAV)
M_135	4664	NGFI-A binding protein 1(NAB1)
M_136	1798	dolichyl-phosphate N-acetylglucosaminephosphotransferase 1(DPAGT1)
M_136	27338	ubiquitin conjugating enzyme E2 S(UBE2S)

M_136	10179	RNA binding motif protein 7(RBM7)
M_136	23387	SIK family kinase 3(SIK3)
M_136	10632	ATP synthase, H+ transporting, mitochondrial Fo complex subunit G(ATP5L)
M_136	3014	H2A histone family member X(H2AFX)
M_136	219902	transmembrane protein 136(TMEM136)
M_136	219899	tubulin folding cofactor E like(TBCEL)
M_136	65084	transmembrane protein 135(TMEM135)
M_136	38	acetyl-CoA acetyltransferase 1(ACAT1)
M_136	28970	chromosome 11 open reading frame 54(C11orf54)
M_136	8726	embryonic ectoderm development(EED)
M_136	3145	hydroxymethylbilane synthase(HMBS)
M_136	654320	small nucleolar RNA, H/ACA box 8(SNORA8)
M_136	10929	serine and arginine rich splicing factor 8(SRSF8)
M_136	6309	sterol-C5-desaturase(SC5D)
M_136	196264	myelin protein zero like 3(MPZL3)
M_136	6392	succinate dehydrogenase complex subunit D(SDHD)
M_136	5805	6-pyruvoyltetrahydropterin synthase(PTS)
M_136	84233	transmembrane protein 126A(TMEM126A)
M_136	26521	translocase of inner mitochondrial membrane 8 homolog B(TIMM8B)
M_136	54851	ankyrin repeat domain 49(ANKRD49)
M_136	338657	coiled-coil domain containing 84(CCDC84)
M_136	55823	VPS11, CORVET/HOPS core subunit(VPS11)
M_136	51399	trafficking protein particle complex 4(TRAPPC4)
M_136	79102	ring finger protein 26(RNF26)
M_136	29087	thymocyte nuclear protein 1(THYN1)
M_136	64776	chromosome 11 open reading frame 1(C11orf1)
M_136	257160	ring finger protein 214(RNF214)
M_136	8882	ZPR1 zinc finger(ZPR1)
M_136	372	archain 1(ARCN1)
M_136	1656	DEAD-box helicase 6(DDX6)

M_136	143941	tetratricopeptide repeat domain 36(TTC36)
M_136	79101	TATA-box binding protein associated factor, RNA polymerase I subunit D(TAF1D)
M_136	54733	solute carrier family 35 member F2(SLC35F2)
M_136	84811	BUD13 homolog(BUD13)
M_136	10525	hypoxia up-regulated 1(HYOU1)
M_136	55863	transmembrane protein 126B(TMEM126B)
M_136	55693	lysine demethylase 4D(KDM4D)
M_136	7007	tectorin alpha(TECTA)
M_136	79607	family with sequence similarity 118 member
M_136	60492	coiled-coil domain containing 90B(CCDC90B)
M_136	89944	galactosidase beta 1 like 2(GLB1L2)
M_136	2542	solute carrier family 37 member 4(SLC37A4)
M_136	51503	CWC15 spliceosome associated protein
M_136	51501	Hikeshi, heat shock protein nuclear import
M_136	143879	kelch repeat and BTB domain containing 3(KBTBD3)
M_136	2230	ferredoxin 1(FDX1)
M_136	1737	dihydrolipoamide S-acetyltransferase(DLAT)
M_136	91894	chromosome 11 open reading frame 52(C11orf52)
M_136	84866	transmembrane protein 25(TMEM25)
M_136	219844	HYLS1, centriolar and ciliogenesis
M_136	5049	platelet activating factor acetylhydrolase 1b catalytic subunit 2(PAFAH1B2)
M_136	80071	coiled-coil domain containing 15(CCDC15)
M_136	55572	FAD dependent oxidoreductase domain containing 1(FOXRED1)
M_136	23310	non-SMC condensin II complex subunit
M_136	54970	tetratricopeptide repeat domain 12(TTC12)
M_136	79671	NLR family member X1(NLRX1)
M_136	3309	heat shock protein family A (Hsp70) member
M_136	3312	heat shock protein family A (Hsp70) member
M_136	56912	intraflagellar transport 46(IFT46)

M_136	84259	defective in cullin neddylation 1 domain containing 5(DCUN1D5)
M_136	160140	chromosome 11 open reading frame 65(C11orf65)
M_136	25988	histone H4 transcription factor(HINFP)
M_136	1111	checkpoint kinase 1(CHEK1)
M_136	25996	RNA exonuclease 2(REXO2)
M_136	10205	myelin protein zero like 2(MPZL2)
M_136	399967	prostate and testis expressed 2(PATE2)
M_136	54494	chromosome 11 open reading frame 71(C11orf71)
M_136	5547	prolylcarboxypeptidase(PRCP)
M_136	1075	cathepsin C(CTSC)
M_136	56	acrosomal vesicle protein 1(ACRV1)
M_136	9854	C2CD2 like(C2CD2L)
M_136	220388	coiled-coil domain containing 89(CCDC89)
M_137	5527	protein phosphatase 2 regulatory subunit
M_137	283643	chromosome 14 open reading frame 80(C14orf80)
M_137	283638	centrosomal protein 170B(CEP170B)
M_137	9529	BCL2 associated athanogene 5(BAG5)
M_137	55148	ubiquitin protein ligase E3 component n-recognin 7 (putative)(UBR7)
M_137	4140	microtubule affinity regulating kinase 3(MARK3)
M_137	196872	long intergenic non-protein coding RNA
M_137	51218	glutaredoxin 5(GLRX5)
M_137	122416	ankyrin repeat domain 9(ANKRD9)
M_137	122509	interferon alpha inducible protein 27 like 1(IFI27L1)
M_137	90135	BTB domain containing 6(BTBD6)
M_137	122616	chromosome 14 open reading frame 79(C14orf79)
M_137	10572	SIVA1 apoptosis inducing factor(SIVA1)
M_137	9112	metastasis associated 1(MTA1)
M_137	5700	proteasome 26S subunit, ATPase 1(PSMC1)
M_137	51527	GSK3B interacting protein(GSKIP)
M_137	55038	cell division cycle associated 4(CDCA4)

M_137	3705	inositol-tetrakisphosphate 1-kinase(ITPK1)
M_137	7187	TNF receptor associated factor 3(TRAF3)
M_137	256281	nudix hydrolase 14(NUDT14)
M_137	801	calmodulin 1(CALM1)
M_137	84520	GON7, KEOPS complex subunit homolog(GON7)
M_137	3831	kinesin light chain 1(KLC1)
M_137	207	AKT serine/threonine kinase 1(AKT1)
M_137	7517	X-ray repair cross complementing 3(XRCC3)
M_137	79038	zinc finger FYVE-type containing 21(ZFYVE21)
M_137	115708	tRNA methyltransferase 61A(TRMT61A)
M_137	79446	WD repeat domain 25(WDR25)
M_137	23368	protein phosphatase 1 regulatory subunit
M_137	317762	coiled-coil domain containing 85C(CCDC85C)
M_137	51550	cyclin dependent kinase 2 interacting protein(CINP)
M_137	100128927	zinc finger and BTB domain containing 42(ZBTB42)
M_137	84334	apoptogenic 1, mitochondrial(APOPT1)
M_137	7443	vaccinia related kinase 1(VRK1)
M_137	90141	EF-hand calcium binding domain 11(EFCAB11)
M_137	1397	cysteine rich protein 2(CRIP2)
M_137	3320	heat shock protein 90 alpha family class A member 1(HSP90AA1)
M_137	84193	SET domain containing 3(SETD3)
M_137	122622	adenylosuccinate synthase like 1(ADSSL1)
M_137	23241	phosphofurin acidic cluster sorting protein 2(PACS2)
M_137	9556	chromosome 14 open reading frame 2(C14orf2)
M_137	55778	zinc finger protein 839(ZNF839)
M_137	91833	WD repeat domain 20(WDR20)
M_137	2972	BRF1, RNA polymerase III transcription initiation factor 90 kDa subunit(BRF1)
M_137	26175	transmembrane protein 251(TMEM251)
M_137	3714	jagged 2(JAG2)
M_137	1396	cysteine rich protein 1(CRIP1)

M_137	1983	eukaryotic translation initiation factor 5(EIF5)
M_137	5891	MOK protein kinase(MOK)
M_137	150381	PRR34 antisense RNA 1(PRR34-AS1)
M_137	123096	solute carrier family 25 member 29(SLC25A29)
M_138	55201	microtubule associated protein 1S(MAP1S)
M_138	171023	additional sex combs like 1, transcriptional
M_138	23509	protein O-fucosyltransferase 1(POFUT1)
M_138	163589	tudor domain containing 5(TDRD5)
M_138	79575	abhydrolase domain containing 8(ABHD8)
M_138	100130418	cat eye syndrome chromosome region, candidate 7 (non-protein coding)(CECR7)
M_138	219995	membrane spanning 4-domains A15(MS4A15)
M_138	57110	HRAS like suppressor(HRASLS)
M_138	79709	collagen beta(1-O)galactosyltransferase
M_138	2645	glucokinase(GCK)
M_138	126549	ankyrin repeat and LEM domain containing
M_138	5208	6-phosphofructo-2-kinase/fructose-2,6-biphosphatase 2(PFKFB2)
M_138	8999	cyclin dependent kinase like 2(CDKL2)
M_138	345456	profilin 3(PFN3)
M_138	2984	guanylate cyclase 2C(GUCY2C)
M_138	26017	family with sequence similarity 32 member
M_138	58498	myosin light chain 7(MYL7)
M_138	151174	uncharacterized LOC151174(LOC151174)
M_138	84789	uncharacterized protein MGC2889(MGC2889)
M_138	440590	zyg-11 family member A, cell cycle
M_138	166824	Ras association domain family member 6(RASSF6)
M_138	344905	ATPase 13A5(ATP13A5)
M_138	283761	long intergenic non-protein coding RNA
M_138	79041	transmembrane protein 38A(TMEM38A)
M_138	9777	transmembrane 9 superfamily member 4(TM9SF4)
M_138	4218	RAB8A, member RAS oncogene family(RAB8A)

M_138	151963	Mab-21 domain containing 2(MB21D2)
M_138	90427	Bcl2 modifying factor(BMF)
M_138	9371	kinesin family member 3B(KIF3B)
M_138	9261	mitogen-activated protein kinase-activated protein kinase 2(MAPKAPK2)
M_138	93323	HAUS augmin like complex subunit 8(HAUS8)
M_138	100271715	Rho guanine nucleotide exchange factor
M_138	6954	t-complex 11(TCP11)
M_138	8934	RAB29, member RAS oncogene family(RAB29)
M_138	79712	glycosyltransferase like domain containing 1(GTDC1)
M_138	283212	kelch like family member 35(KLHL35)
M_138	5326	PLAG1 like zinc finger 2(PLAGL2)
M_138	79629	occludin/ELL domain containing 1(OCEL1)
M_138	1339	cytochrome c oxidase subunit 6A2(COX6A2)
M_138	140688	nucleolar protein 4 like(NOL4L)
M_138	3787	potassium voltage-gated channel modifier subfamily S member 1(KCNS1)
M_138	254559	MIR9-3 host gene(MIR9-3HG)
M_138	64344	hypoxia inducible factor 3 alpha subunit(HIF3A)
M_138	2063	nuclear receptor subfamily 2 group F member
M_138	26166	regulator of G-protein signaling 22(RGS22)
M_138	83889	WD repeat domain 87(WDR87)
M_138	5287	phosphatidylinositol-4-phosphate 3-kinase catalytic subunit type 2 beta(PIK3C2B)
M_138	7171	tropomyosin 4(TPM4)
M_138	4879	natriuretic peptide B(NPPB)
M_138	23613	zinc finger MYND-type containing 8(ZMYND8)
M_138	11063	SRY-box 30(SOX30)
M_138	147407	solute carrier family 25 member 52(SLC25A52)
M_138	10934	mortality factor 4 (pseudogene)(MORF4)
M_139	10808	heat shock protein family H (Hsp110) member
M_139	1024	cyclin dependent kinase 8(CDK8)

M_139	79915	ATPase family, AAA domain containing 5(ATAD5)
M_139	84650	emopamil binding protein like(EBPL)
M_139	79758	dehydrogenase/reductase 12(DHRS12)
M_139	10301	deleted in lymphocytic leukemia 1(DLEU1)
M_139	55269	paraspeckle component 1(PSPC1)
M_139	55270	nudix hydrolase 15(NUDT15)
M_139	7347	ubiquitin C-terminal hydrolase L3(UCHL3)
M_139	284992	coiled-coil domain containing 150(CCDC150)
M_139	11340	exosome component 8(EXOSC8)
M_139	170622	COMM domain containing 6(COMMD6)
M_139	5983	replication factor C subunit 3(RFC3)
M_139	7750	zinc finger MYM-type containing 2(ZMYM2)
M_139	219402	mitochondrial translational initiation factor 3(MTIF3)
M_139	55835	centromere protein J(CENPJ)
M_139	10166	solute carrier family 25 member 15(SLC25A15)
M_139	57213	SPRY domain containing 7(SPRYD7)
M_139	2971	general transcription factor IIIA(GTF3A)
M_139	3146	high mobility group box 1(HMGB1)
M_139	9107	myotubularin related protein 6(MTMR6)
M_139	2098	esterase D(ESD)
M_139	26586	cytoskeleton associated protein 2(CKAP2)
M_139	79866	bora, aurora kinase A activator(BORA)
M_139	221143	eukaryotic translation elongation factor 1 alpha lysine methyltransferase 1(EEF1AKMT1)
M_139	4285	mitochondrial intermediate peptidase(MIPEP)
M_139	221154	mitochondrial calcium uptake 2(MICU2)
M_139	5412	ubiquitin like 3(UBL3)
M_139	51131	PHD finger protein 11(PHF11)
M_139	10284	Sin3A associated protein 18(SAP18)
M_139	51082	RNA polymerase I subunit D(POLR1D)
M_139	81624	diaphanous related formin 3(DIAPH3)
M_139	130355	chromosome 2 open reading frame 76(C2orf76)

M_139	23047	PDS5 cohesin associated factor B(PDS5B)
M_139	78988	mitochondrial ribosomal protein L57(MRPL57)
M_139	8100	intraflagellar transport 88(IFT88)
M_139	79621	ribonuclease H2 subunit B(RNASEH2B)
M_139	29880	ALG5, dolichyl-phosphate beta-
M_139	675	BRCA2, DNA repair associated(BRCA2)
M_139	51371	proteasome maturation protein(POMP)
M_139	10910	SGT1 homolog, MIS12 kinetochore complex assembly cochaperone(SUGT1)
M_139	255967	PAN3 poly(A) specific ribonuclease subunit(PAN3)
M_139	55345	zinc finger GRF-type containing 1(ZGRF1)
M_139	7178	tumor protein, translationally-controlled 1(TPT1)
M_139	348738	chromosome 2 open reading frame 48(C2orf48)
M_139	8847	deleted in lymphocytic leukemia 2 (non-protein coding)(DLEU2)
M_140	7532	tyrosine 3-monooxygenase/tryptophan 5-monooxygenase activation protein gamma(YWHAG)
M_140	221908	protein phosphatase 1 regulatory subunit
M_140	222234	family with sequence similarity 185 member
M_140	83787	armadillo repeat containing 10(ARMC10)
M_140	6717	sorcin(SRI)
M_140	11257	TP53 target 1 (non-protein coding)(TP53TG1)
M_140	222194	round spermatid basic protein 1 like(RSBN1L)
M_140	389524	GTF2I repeat domain containing 2B(GTF2IRD2B)
M_140	79027	zinc finger protein 655(ZNF655)
M_140	136332	leucine rich repeats and guanylate kinase domain containing(LRGUK)
M_140	84060	RNA binding motif protein 48(RBM48)
M_140	57798	GATA zinc finger domain containing 1(GATAD1)
M_140	10282	Bet1 golgi vesicular membrane trafficking
M_140	55972	solute carrier family 25 member 40(SLC25A40)
M_140	84849	#N/A

M_140	5782	protein tyrosine phosphatase, non-receptor type
M_140	161582	dyslexia susceptibility 1 candidate 1(DYX1C1)
M_140	26958	coatomer protein complex subunit gamma 2(COPG2)
M_140	4899	nuclear respiratory factor 1(NRF1)
M_140	79161	transmembrane protein 243(TMEM243)
M_140	64921	CAS1 domain containing 1(CASD1)
M_140	8896	BUD31 homolog(BUD31)
M_140	51530	zinc finger C3HC-type containing 1(ZC3HC1)
M_140	5701	proteasome 26S subunit, ATPase 2(PSMC2)
M_140	84928	transmembrane protein 209(TMEM209)
M_140	389541	late endosomal/lysosomal adaptor, MAPK and MTOR activator 4(LAMTOR4)
M_140	29923	hypoxia inducible lipid droplet associated(HILPDA)
M_140	889	KRIT1, ankyrin repeat containing(KRIT1)
M_140	1738	dihydrolipoamide dehydrogenase(DLD)
M_140	222236	N-acyl phosphatidylethanolamine phospholipase D(NAPEPLD)
M_140	222235	F-box and leucine rich repeat protein 13(FBXL13)
M_140	54677	carnitine O-octanoyltransferase(CROT)
M_140	1174	adaptor related protein complex 1 sigma 1
M_140	5001	origin recognition complex subunit 5(ORC5)
M_140	2783	G protein subunit beta 2(GNB2)
M_140	11062	dihydrouridine synthase 4 like(DUS4L)
M_140	91147	transmembrane protein 67(TMEM67)
M_140	5439	RNA polymerase II subunit J(POLR2J)
M_140	10552	actin related protein 2/3 complex subunit
M_140	95681	centrosomal protein 41(CEP41)
M_140	85865	GTP binding protein 10(GTPBP10)
M_140	85025	transmembrane protein 60(TMEM60)
M_140	10165	solute carrier family 25 member 13(SLC25A13)
M_140	60561	RAD50 interactor 1(RINT1)
M_140	57157	putative homeodomain transcription factor 2(PHTF2)

M_140	79846	cilia and flagella associated protein 69(CFAP69)
M_140	7979	split hand/foot malformation (ectrodactyly) type
M_140	55610	VPS50, EARP/GARPII complex subunit(VPS50)
M_140	7978	mitochondrial transcription termination factor
M_140	10980	COP9 signalosome subunit 6(COPS6)
M_140	6856	synaptophysin like 1(SYPL1)
M_140	5189	peroxisomal biogenesis factor 1(PEX1)
M_140	10467	zinc finger HIT-type containing 1(ZNHIT1)
M_140	83943	inner mitochondrial membrane peptidase subunit
M_140	9512	peptidase, mitochondrial processing beta
M_140	221785	zinc finger and SCAN domain containing
M_140	54467	ankyrin repeat and IBR domain containing
M_140	221786	family with sequence similarity 200 member
M_140	25798	brain protein I3(BRI3)
M_140	64598	motile sperm domain containing 3(MOSPD3)
M_140	9069	claudin 12(CLDN12)
M_141	7290	histone cell cycle regulator(HIRA)
M_141	5594	mitogen-activated protein kinase 1(MAPK1)
M_141	51586	mediator complex subunit 15(MED15)
M_141	10043	target of myb1 membrane trafficking protein(TOM1)
M_141	421	armadillo repeat gene deleted in velocardiofacial syndrome(ARVCF)
M_141	728233	phosphatidylinositol 4-kinase alpha pseudogene
M_141	79680	chromosome 22 open reading frame 29(C22orf29)
M_141	23384	sperm antigen with calponin homology and coiled-coil domains 1 like(SPECC1L)
M_141	83606	guanylyl cyclase domain containing 1(GUCD1)
M_141	150274	HscB mitochondrial iron-sulfur cluster
M_141	23786	BCL2 like 13(BCL2L13)
M_141	23759	peptidylprolyl isomerase like 2(PPIL2)
M_141	85379	KIAA1671(KIAA1671)
M_141	10634	growth arrest specific 2 like 1(GAS2L1)

M_141	84222	transmembrane protein 191A
M_141	8459	tyrosylprotein sulfotransferase 2(TPST2)
M_141	9647	protein phosphatase, Mg2+/Mn2+ dependent
M_141	6634	small nuclear ribonucleoprotein D3
M_141	29801	zinc finger DHHC-type containing 8(ZDHHC8)
M_141	1312	catechol-O-methyltransferase(COMT)
M_141	613	BCR, RhoGEF and GTPase activating protein(BCR)
M_141	54487	DGCR8, microprocessor complex subunit(DGCR8)
M_141	23523	calcineurin binding protein 1(CABIN1)
M_141	1399	CRK like proto-oncogene, adaptor protein(CRKL)
M_141	8214	DiGeorge syndrome critical region gene 6(DGCR6)
M_141	637	BH3 interacting domain death agonist(BID)
M_141	84861	kelch like family member 22(KLHL22)
M_141	128989	transport and golgi organization 2
M_141	25786	DiGeorge syndrome critical region gene 11 (non-protein coding)(DGCR11)
M_141	6598	SWI/SNF related, matrix associated, actin dependent regulator of chromatin, subfamily b, member
M_141	23765	interleukin 17 receptor A(IL17RA)
M_141	5297	phosphatidylinositol 4-kinase alpha(PI4KA)
M_141	100037417	D-dopachrome tautomerase-like(DDTL)
M_141	8940	topoisomerase (DNA) III beta(TOP3B)
M_141	8216	leucine zipper like transcription regulator 1(LZTR1)
M_141	23119	HIC ZBTB transcriptional repressor 2(HIC2)
M_141	66035	solute carrier family 2 member 11(SLC2A11)
M_141	7625	zinc finger protein 74(ZNF74)
M_141	55670	peroxisomal biogenesis factor 26(PEX26)
M_141	9993	DiGeorge syndrome critical region gene 2(DGCR2)
M_141	7621	zinc finger protein 70(ZNF70)
M_141	162	adaptor related protein complex 1 beta 1
M_141	529	ATPase H+ transporting V1 subunit E1(ATP6V1E1)
M_141	9262	serine/threonine kinase 17b(STK17B)

M_141	57553	microtubule associated monooxygenase, calponin and LIM domain containing 3(MICAL3)
M_141	10633	RAS like family 10 member A(RASL10A)
M_141	65078	reticulon 4 receptor(RTN4R)
M_141	1417	crystallin beta B3(CRYBB3)
M_142	1608	diacylglycerol kinase gamma(DGKG)
M_142	142680	solute carrier family 34 member 3(SLC34A3)
M_142	58512	DLG associated protein 3(DLGAP3)
M_142	7102	tetraspanin 7(TSPAN7)
M_142	26070	uncharacterized LOC26070(DKFZP434K028)
M_142	649446	DLGAP1 antisense RNA 1(DLGAP1-AS1)
M_142	22841	RAB11 family interacting protein 2(RAB11FIP2)
M_142	150244	zinc finger DHHC-type containing 8 pseudogene 1(ZDHHC8P1)
M_142	89777	serpin family B member 12(SERPINB12)
M_142	347730	leucine rich repeat transmembrane neuronal
M_142	55966	adherens junctions associated protein 1(AJAP1)
M_142	114036	long intergenic non-protein coding RNA
M_142	745	myelin regulatory factor(MYRF)
M_142	729582	disrupted in renal carcinoma 3(DIRC3)
M_142	57482	KIAA1211(KIAA1211)
M_142	2903	glutamate ionotropic receptor NMDA type subunit 2A(GRIN2A)
M_142	10814	complexin 2(CPLX2)
M_142	286256	lipocalin 12(LCN12)
M_142	7050	TGFB induced factor homeobox 1(TGIF1)
M_142	10811	NADPH oxidase activator 1(NOXA1)
M_142	3084	neuregulin 1(NRG1)
M_142	57758	signal peptide, CUB domain and EGF like domain containing 2(SCUBE2)
M_142	26011	teneurin transmembrane protein 4(TENM4)
M_142	4887	neuropeptide Y receptor Y2(NPY2R)

M_142	1427	crystallin gamma S(CRYGS)
M_142	377841	ectonucleoside triphosphate diphosphohydrolase
M_142	196047	EMX2 opposite strand/antisense RNA(EMX2OS)
M_142	92691	transmembrane protein 169(TMEM169)
M_142	335	apolipoprotein A1(APOA1)
M_142	22882	zinc fingers and homeoboxes 2(ZHX2)
M_142	6526	solute carrier family 5 member 3(SLC5A3)
M_142	9229	DLG associated protein 1(DLGAP1)
M_142	63027	solute carrier family 22 member 23(SLC22A23)
M_142	3975	LIM homeobox 1(LHX1)
M_142	84632	actin filament associated protein 1 like 2(AFAP1L2)
M_142	387640	SKI/DACH domain containing 1(SKIDA1)
M_142	91584	plexin A4(PLXNA4)
M_142	7855	frizzled class receptor 5(FZD5)
M_142	55825	peroxisomal trans-2-enoyl-CoA reductase(PECR)
M_142	54437	semaphorin 5B(SEMA5B)
M_142	57571	carnosine synthase 1(CARNS1)
M_142	399726	cancer susceptibility candidate 10(CASC10)
M_142	1496	catenin alpha 2(CTNNA2)
M_142	4684	neural cell adhesion molecule 1(NCAM1)
M_142	5121	Purkinje cell protein 4(PCP4)
M_142	2018	empty spiracles homeobox 2(EMX2)
M_142	10954	protein disulfide isomerase family A member
M_142	401	paired like homeobox 2a(PHOX2A)
M_142	57453	DS cell adhesion molecule like 1(DSCAML1)
M_142	5009	ornithine carbamoyltransferase(OTC)
M_142	727800	ring finger protein 208(RNF208)
M_142	284948	SH2 domain containing 6(SH2D6)
M_142	57348	tweety family member 1(TTYH1)
M_142	7042	transforming growth factor beta 2(TGFB2)
M_142	7074	T-cell lymphoma invasion and metastasis 1(TIAM1)

M_142	23493	hes related family bHLH transcription factor with YRPW motif 2(HEY2)
M_142	100133545	MRPL23 antisense RNA 1(MRPL23-AS1)
M_142	147525	long intergenic non-protein coding RNA
M_142	54932	exonuclease 3'-5' domain containing 3(EXD3)
M_142	100286793	#N/A
M_142	388685	long intergenic non-protein coding RNA
M_142	7541	zinc finger and BTB domain containing 14(ZBTB14)
M_142	2675	GDNF family receptor alpha 2(GFRA2)
M_142	80228	ORAI calcium release-activated calcium modulator
M_142	440738	microtubule associated protein 1 light chain 3 gamma(MAP1LC3C)
M_142	51205	acid phosphatase 6, lysophosphatidic(ACP6)
M_142	84676	tripartite motif containing 63(TRIM63)
M_142	55137	fidgetin, microtubule severing factor(FIGN)
M_142	399888	family with sequence similarity 180 member
M_142	2261	fibroblast growth factor receptor 3(FGFR3)
M_142	342527	smoothelin like 2(SMTNL2)
M_142	11251	prostaglandin D2 receptor 2(PTGDR2)
M_142	339290	long intergenic non-protein coding RNA
M_142	728855	long intergenic non-protein coding RNA
M_142	3483	insulin like growth factor binding protein acid labile subunit(IGFALS)
M_142	9068	angiopoietin like 1(ANGPTL1)
M_142	123	perilipin 2(PLIN2)
M_142	10603	SH2B adaptor protein 2(SH2B2)
M_142	388692	uncharacterized LOC388692(LOC388692)
M_142	162514	transient receptor potential cation channel subfamily V member 3(TRPV3)
M_142	728875	#N/A
M_143	131616	transmembrane protein 42(TMEM42)
M_143	4201	male-enhanced antigen 1(MEA1)

M_143	29964	prickle planar cell polarity protein 4(PRICKLE4)
M_143	4258	microsomal glutathione S-transferase 2(MGST2)
M_143	6015	ring finger protein 1(RING1)
M_143	8673	vesicle associated membrane protein 8(VAMP8)
M_143	25844	Yip1 domain family member 3(YIPF3)
M_143	5252	PHD finger protein 1(PHF1)
M_143	7866	interferon related developmental regulator 2(IFRD2)
M_143	51069	mitochondrial ribosomal protein L2(MRPL2)
M_143	100188893	translocase of outer mitochondrial membrane
M_143	24142	N-acetyltransferase 6(NAT6)
M_143	154467	coiled-coil domain containing 167(CCDC167)
M_143	9136	ribosomal RNA processing 9, small subunit (SSU) processome component, homolog (yeast)(RRP9)
M_143	84996	URB1 antisense RNA 1 (head to head)(URB1-AS1)
M_143	51227	phosphatidylinositol glycan anchor biosynthesis class
M_143	221491	chromosome 6 open reading frame 1(C6orf1)
M_143	84836	abhydrolase domain containing 14B(ABHD14B)
M_143	103910	myosin light chain 12B(MYL12B)
M_143	11165	nudix hydrolase 3(NUDT3)
M_143	10627	myosin light chain 12A(MYL12A)
M_143	2739	glyoxalase I(GLO1)
M_143	7923	hydroxysteroid 17-beta dehydrogenase 8(HSD17B8)
M_143	28973	mitochondrial ribosomal protein S18B(MRPS18B)
M_143	55152	DALR anticodon binding domain containing
M_143	10817	fibroblast growth factor receptor substrate 3(FRS3)
M_143	578	BCL2 antagonist/killer 1(BAK1)
M_143	327	acylaminoacyl-peptide hydrolase(APEH)
M_143	88745	ribosomal RNA processing 36(RRP36)
M_143	51533	PHD finger protein 7(PHF7)
M_143	54681	prolyl 4-hydroxylase, transmembrane(P4HTM)
M_143	116138	kelch domain containing 3(KLHDC3)
M_143	347734	solute carrier family 35 member B2(SLC35B2)

M_143	10039	poly(ADP-ribose) polymerase family member
M_143	25864	abhydrolase domain containing 14A(ABHD14A)
M_143	55168	mitochondrial ribosomal protein S18A(MRPS18A)
M_143	221443	O-acyl-ADP-ribose deacylase 1(OARD1)
M_143	4795	NFKB inhibitor like 1(NFKBIL1)
M_143	10641	NPR2-like, GATOR1 complex subunit(NPRL2)
M_143	10695	canopy FGF signaling regulator 3(CNPY3)
M_143	51645	peptidylprolyl isomerase like 1(PPIL1)
M_143	8705	beta-1,3-galactosyltransferase 4(B3GALT4)
M_143	2876	glutathione peroxidase 1(GPX1)
M_143	6992	protein phosphatase 1 regulatory inhibitor subunit 11(PPP1R11)
M_143	10211	flotillin 1(FLOT1)
M_143	221545	chromosome 6 open reading frame 136(C6orf136)
M_143	95	aminoacylase 1(ACY1)
M_143	64928	mitochondrial ribosomal protein L14(MRPL14)
M_143	64925	coiled-coil domain containing 71(CCDC71)
M_143	441150	chromosome 6 open reading frame 226(C6orf226)
M_143	84418	cysteine rich transmembrane module containing
M_143	8637	eukaryotic translation initiation factor 4E binding protein 3(EIF4EBP3)
M_143	6903	tubulin folding cofactor C(TBCC)
M_143	9587	MAD2L1 binding protein(MAD2L1BP)
M_143	7086	transketolase(TKT)
M_143	10591	2'-deoxynucleoside 5'-phosphate N-hydrolase
M_143	9533	RNA polymerase I subunit C(POLR1C)
M_143	55173	mitochondrial ribosomal protein S10(MRPS10)
M_143	1192	chloride intracellular channel 1(CLIC1)
M_143	646450	ariadne homolog 2 opposite strand(ARIH2OS)
M_143	6882	TATA-box binding protein associated factor
M_143	51368	testis expressed 264(TEX264)
M_143	11186	Ras association domain family member 1(RASSF1)

M_143	7920	abhydrolase domain containing 16A(ABHD16A)
M_143	780	discoidin domain receptor tyrosine kinase 1(DDR1)
M_143	84315	MON1 homolog A, secretory trafficking
M_143	2771	G protein subunit alpha i2(GNAI2)
M_143	23564	dimethylarginine dimethylaminohydrolase 2(DDAH2)
M_143	7869	semaphorin 3B(SEMA3B)
M_143	7867	mitogen-activated protein kinase-activated protein kinase 3(MAPKAPK3)
M_143	387	ras homolog family member A(RHOA)
M_143	788	solute carrier family 25 member 20(SLC25A20)
M_143	1849	dual specificity phosphatase 7(DUSP7)
M_144	3855	keratin 7(KRT7)
M_144	79958	DENN domain containing 1C(DENND1C)
M_144	5639	proline rich and Gla domain 2(PRRG2)
M_144	222	aldehyde dehydrogenase 3 family member
M_144	27134	tight junction protein 3(TJP3)
M_144	171022	ABHD11 antisense RNA 1 (tail to tail)(ABHD11-
M_144	10045	SH2 domain containing 3A(SH2D3A)
M_144	149466	chromosome 1 open reading frame 210(C1orf210)
M_144	4072	epithelial cell adhesion molecule(EPCAM)
M_144	1366	claudin 7(CLDN7)
M_144	54845	epithelial splicing regulatory protein 1(ESRP1)
M_144	79977	grainyhead like transcription factor 2(GRHL2)
M_144	54836	B-box and SPRY domain containing(BSPRY)
M_144	161291	transmembrane protein 30B(TMEM30B)
M_144	92359	crumbs 3, cell polarity complex component(CRB3)
M_144	338440	anoctamin 9(ANO9)
M_144	2041	EPH receptor A1(EPHA1)
M_144	64787	EPS8 like 2(EPS8L2)
M_144	11187	plakophilin 3(PKP3)
M_144	58495	ovo like zinc finger 2(OVOL2)
M_144	147798	transmembrane channel like 4(TMC4)

M_144	10053	adaptor related protein complex 1 mu 2
M_144	146439	BICD family like cargo adaptor 2(BICDL2)
M_144	3898	ladinin 1(LAD1)
M_144	5652	protease, serine 8(PRSS8)
M_144	140893	RBBP8 N-terminal like(RBBP8NL)
M_144	4070	tumor-associated calcium signal transducer
M_144	112724	retinol dehydrogenase 13(RDH13)
M_144	150696	prominin 2(PROM2)
M_144	54869	EPS8 like 1(EPS8L1)
M_144	162387	major facilitator superfamily domain containing 6 like(MFSD6L)
M_144	286676	immunoglobulin like domain containing receptor
M_144	23779	Rho GTPase activating protein 8(ARHGAP8)
M_144	1365	claudin 3(CLDN3)
M_144	83451	abhydrolase domain containing 11(ABHD11)
M_144	6768	suppression of tumorigenicity 14(ST14)
M_144	1364	claudin 4(CLDN4)
M_144	126695	keratinocyte differentiation factor 1(KDF1)
M_144	81607	nectin cell adhesion molecule 4(NECTIN4)
M_144	2065	erb-b2 receptor tyrosine kinase 3(ERBB3)
M_144	553158	PRR5-ARHGAP8 readthrough(PRR5-ARHGAP8)
M_144	6692	serine peptidase inhibitor, Kunitz type 1(SPINT1)
M_144	6337	sodium channel epithelial 1 alpha subunit(SCNN1A)
M_144	128218	transmembrane protein 125(TMEM125)
M_144	91862	MARVEL domain containing 3(MARVELD3)
M_144	3664	interferon regulatory factor 6(IRF6)
M_144	64218	semaphorin 4A(SEMA4A)
M_144	55040	epsin 3(EPN3)
M_144	50848	F11 receptor(F11R)
M_144	928	CD9 molecule(CD9)
M_144	6385	syndecan 4(SDC4)
M_144	114569	mal, T-cell differentiation protein 2

M_144	57402	S100 calcium binding protein A14(S100A14)
M_144	51206	glycoprotein VI platelet(GP6)
M_144	2591	polypeptide N-acetylgalactosaminyltransferase
M_144	5349	FXYD domain containing ion transport regulator
M_144	199713	NLR family pyrin domain containing 7(NLRP7)
M_144	55630	solute carrier family 39 member 4(SLC39A4)
M_144	80162	protein-glucosylgalactosylhydroxylysine
M_144	55080	TAP binding protein like(TAPBPL)
M_144	55655	NLR family pyrin domain containing 2(NLRP2)
M_144	171389	NLR family pyrin domain containing 6(NLRP6)
M_144	387733	interferon induced transmembrane protein 5(IFITM5)
M_145	3992	fatty acid desaturase 1(FADS1)
M_145	388341	leucine rich repeat containing 75A(LRRC75A)
M_145	23208	synaptotagmin 11(SYT11)
M_145	4356	membrane palmitoylated protein 3(MPP3)
M_145	1850	dual specificity phosphatase 8(DUSP8)
M_145	94030	leucine rich repeat containing 4B(LRRC4B)
M_145	284418	family with sequence similarity 71 member
M_145	2737	GLI family zinc finger 3(GLI3)
M_145	2201	fibrillin 2(FBN2)
M_145	55785	FYVE, RhoGEF and PH domain containing 6(FGD6)
M_145	196403	deltex E3 ubiquitin ligase 3(DTX3)
M_145	80117	ADP ribosylation factor like GTPase 14(ARL14)
M_145	81544	glycerophosphodiester phosphodiesterase domain containing 5(GDPD5)
M_145	81551	stathmin 4(STMN4)
M_145	3589	interleukin 11(IL11)
M_145	10763	nestin(NES)
M_145	7481	Wnt family member 11(WNT11)
M_145	338651	KRTAP5-1/KRTAP5-2 antisense RNA 1(KRTAP5-
M_145	387264	keratin associated protein 5-1(KRTAP5-1)
M_145	4355	membrane palmitoylated protein 2(MPP2)

M_145	90139	tetraspanin 18(TSPAN18)
M_145	140706	CCM2 like scaffolding protein(CCM2L)
M_145	8941	cyclin dependent kinase 5 regulatory subunit
M_145	65009	NDRG family member 4(NDRG4)
M_145	65012	solute carrier family 26 member 10(SLC26A10)
M_145	10650	PRELI domain containing 3A(PRELID3A)
M_145	7786	mitogen-activated protein kinase kinase kinase
M_145	125965	cytochrome c oxidase subunit 6B2(COX6B2)
M_145	9746	calsyntenin 3(CLSTN3)
M_145	6574	solute carrier family 20 member 1(SLC20A1)
M_145	80128	tripartite motif containing 46(TRIM46)
M_145	1299	collagen type IX alpha 3 chain(COL9A3)
M_145	9543	immunoglobulin superfamily DCC subclass member 3(IGDCC3)
M_145	7216	trophinin(TRO)
M_145	92369	splA/ryanodine receptor domain and SOCS box containing 4(SPSB4)
M_145	28514	delta like canonical Notch ligand 1(DLL1)
M_145	54587	matrix remodeling associated 8(MXRA8)
M_145	116835	heat shock protein family A (Hsp70) member
M_145	3679	integrin subunit alpha 7(ITGA7)
M_145	9241	noggin(NOG)
M_145	576	adhesion G protein-coupled receptor B2(ADGRB2)
M_145	6483	ST3 beta-galactoside alpha-2,3-sialyltransferase
M_145	84063	kin of IRRE like 2 (Drosophila)(KIRREL2)
M_145	22979	EFR3 homolog B(EFR3B)
M_145	8128	ST8 alpha-N-acetyl-neuraminide alpha-2,8-sialyltransferase 2(ST8SIA2)
M_145	84446	BR serine/threonine kinase 1(BRSK1)
M_145	2768	G protein subunit alpha 12(GNA12)
M_145	114088	tripartite motif containing 9(TRIM9)
M_145	6585	slit guidance ligand 1(SLIT1)

M_145	8577	transmembrane protein with EGF like and two follistatin like domains 1(TMEFF1)
M_145	7280	tubulin beta 2A class IIa(TUBB2A)
M_145	1903	sphingosine-1-phosphate receptor 3(S1PR3)
M_145	27063	ankyrin repeat domain 1(ANKRD1)
M_145	147744	transmembrane protein 190(TMEM190)
M_145	160622	general receptor for phosphoinositides 1 associated scaffold protein(GRASP)
M_145	7846	tubulin alpha 1a(TUBA1A)
M_145	9265	cytohesin 3(CYTH3)
M_145	4920	receptor tyrosine kinase like orphan receptor 2(ROR2)
M_145	222950	neuronal tyrosine phosphorylated phosphoinositide-3-kinase adaptor 1(NYAP1)
M_145	9586	cAMP responsive element binding protein 5(CREB5)
M_145	3797	kinesin family member 3C(KIF3C)
M_145	57568	signal induced proliferation associated 1 like
M_145	6334	sodium voltage-gated channel alpha subunit
M_145	3786	potassium voltage-gated channel subfamily Q member 3(KCNQ3)
M_145	10962	myeloid/lymphoid or mixed-lineage leukemia; translocated to, 11(MLLT11)
M_145	55076	transmembrane protein 45A(TMEM45A)
M_145	57198	ATPase phospholipid transporting 8B2(ATP8B2)
M_145	25999	CAP-Gly domain containing linker protein 3(CLIP3)
M_145	90187	elastin microfibril interfacer 3(EMILIN3)
M_145	53826	FXYD domain containing ion transport regulator
M_145	53822	FXYD domain containing ion transport regulator
M_145	1742	discs large MAGUK scaffold protein 4(DLG4)
M_145	84695	lysyl oxidase like 3(LOXL3)
M_145	147645	V-set and immunoglobulin domain containing 10 like(VSIG10L)
M_145	23090	zinc finger protein 423(ZNF423)

M_145	56603	cytochrome P450 family 26 subfamily B member 1(CYP26B1)
M_145	126129	carnitine palmitoyltransferase 1C(CPT1C)
M_145	4157	melanocortin 1 receptor(MC1R)
M_145	107	adenylate cyclase 1(ADCY1)
M_145	162083	chromosome 16 open reading frame 82(C16orf82)
M_145	57556	semaphorin 6A(SEMA6A)
M_145	6899	T-box 1(TBX1)
M_145	2318	filamin C(FLNC)
M_145	4237	microfibrillar associated protein 2(MFAP2)
M_145	8506	contactin associated protein 1(CNTNAP1)
M_145	80332	ADAM metallopeptidase domain 33(ADAM33)
M_145	401237	cancer susceptibility candidate 15 (non-protein coding)(CASC15)
M_145	9254	calcium voltage-gated channel auxiliary subunit alpha2delta 2(CACNA2D2)
M_145	3955	LFNG O-fucosylpeptide 3-beta-N-acetylglucosaminyltransferase(LFNG)
M_145	286223	chromosome 9 open reading frame 47(C9orf47)
M_145	256297	pancreas specific transcription factor, 1a(PTF1A)
M_145	644353	zinc finger CCHC-type containing 18(ZCCHC18)
M_145	23148	NAC alpha domain containing(NACAD)
M_145	150465	tubulin tyrosine ligase(TTL)
M_145	6624	fascin actin-bundling protein 1(FSCN1)
M_145	3872	keratin 17(KRT17)
M_145	1949	ephrin B3(EFNB3)
M_145	196500	PILR alpha associated neural protein(PIANP)
M_145	347273	muscle related coiled-coil protein(MURC)
M_145	83439	transcription factor 7 like 1(TCF7L1)
M_145	4330	MN1 proto-oncogene, transcriptional regulator(MN1)
M_145	10536	prolyl 3-hydroxylase 3(P3H3)
M_145	401720	fidgetin like 2(FIGNL2)

M_145	2583	beta-1,4-N-acetyl-galactosaminyltransferase
M_145	219539	yippee like 4(YPEL4)
M_145	2307	forkhead box S1(FOXS1)
M_145	10381	tubulin beta 3 class III(TUBB3)
M_145	9415	fatty acid desaturase 2(FADS2)
M_145	9315	neuronal regeneration related protein(NREP)
M_145	115557	Rho guanine nucleotide exchange factor
M_145	8851	cyclin dependent kinase 5 regulatory subunit
M_145	54905	cytochrome P450 family 2 subfamily W member
M_145	9900	synaptic vesicle glycoprotein 2A(SV2A)
M_145	79805	vasohibin 2(VASH2)
M_145	10609	prolyl 3-hydroxylase family member 4 (non-
M_145	6425	secreted frizzled related protein 5(SFRP5)
M_145	4131	microtubule associated protein 1B(MAP1B)
M_145	4038	LDL receptor related protein 4(LRP4)
M_145	56265	carboxypeptidase X, M14 family member 1(CPXM1)
M_145	85366	myosin light chain kinase 2(MYLK2)
M_145	81872	keratin associated protein 2-1(KRTAP2-1)
M_145	333	amyloid beta precursor like protein 1(APLP1)
M_145	119	adducin 2(ADD2)
M_145	8822	fibroblast growth factor 17(FGF17)
M_145	56896	dihydropyrimidinase like 5(DPYSL5)
M_145	4868	NPHS1, nephrin(NPHS1)
M_145	404037	hyaluronan and proteoglycan link protein 4(HAPLN4)
M_145	728392	uncharacterized LOC728392(LOC728392)
M_145	1840	deltex E3 ubiquitin ligase 1(DTX1)
M_145	2535	frizzled class receptor 2(FZD2)
M_145	79998	ankyrin repeat domain 53(ANKRD53)
M_145	347733	tubulin beta 2B class IIb(TUBB2B)
M_145	388419	BTB domain containing 17(BTBD17)
M_145	149345	shisa family member 4(SHISA4)
M_145	29942	purine rich element binding protein G(PURG)

M_145	401612	solute carrier family 25 member 53(SLC25A53)
M_145	60681	FK506 binding protein 10(FKBP10)
M_145	162494	rhomboid like 3(RHBDL3)
M_145	89796	neuron navigator 1(NAV1)
M_145	84630	tau tubulin kinase 1(TTBK1)
M_145	84206	mex-3 RNA binding family member B(MEX3B)
M_145	80727	tweety family member 3(TTYH3)
M_145	402665	IgLON family member 5(IGLON5)
M_145	9080	claudin 9(CLDN9)
M_145	9468	phosphate cytidylyltransferase 1, choline,
M_145	7059	thrombospondin 3(THBS3)
M_145	9074	claudin 6(CLDN6)
M_145	246	arachidonate 15-lipoxygenase(ALOX15)
M_145	2199	fibulin 2(FBLN2)
M_145	440021	keratin associated protein 5-2(KRTAP5-2)
M_145	439915	keratin associated protein 5-5(KRTAP5-5)
M_145	5837	phosphorylase, glycogen, muscle(PYGM)
M_145	64175	prolyl 3-hydroxylase 1(P3H1)
M_145	2487	frizzled-related protein(FRZB)
M_145	7455	zonadhesin (gene/pseudogene)(ZAN)
M_145	4825	NK6 homeobox 1(NKX6-1)
M_145	201191	sterile alpha motif domain containing 14(SAMD14)
M_145	8224	synapsin III(SYN3)
M_145	8425	latent transforming growth factor beta binding protein 4(LTBP4)
M_145	6608	smoothened, frizzled class receptor(SMO)
M_145	54328	G protein-coupled receptor 173(GPR173)
M_145	27122	dickkopf WNT signaling pathway inhibitor 3(DKK3)
M_145	253738	early B-cell factor 3(EBF3)
M_145	51617	HMP19 protein(HMP19)
M_145	6382	syndecan 1(SDC1)
M_145	55033	FK506 binding protein 14(FKBP14)

M_145	5745	parathyroid hormone 1 receptor(PTH1R)
M_145	2784	G protein subunit beta 3(GNB3)
M_145	27344	proprotein convertase subtilisin/kexin type 1 inhibitor(PCSK1N)
M_145	23046	kinesin family member 21B(KIF21B)
M_145	83758	retinol binding protein 5(RBP5)
M_145	246213	solute carrier family 17 member 8(SLC17A8)
M_145	57670	KIAA1549(KIAA1549)
M_145	389432	sterile alpha motif domain containing 5(SAMD5)
M_145	29	active BCR-related(ABR)
M_145	22981	ninein like(NINL)
M_145	163175	leucine rich repeat LGI family member 4(LGI4)
M_145	283383	adhesion G protein-coupled receptor D1(ADGRD1)
M_145	4634	myosin light chain 3(MYL3)
M_145	114804	ring finger protein 157(RNF157)
M_145	58504	Rho GTPase activating protein 22(ARHGAP22)
M_145	374654	kinesin family member 7(KIF7)
M_145	158399	zinc finger protein 483(ZNF483)
M_145	4185	ADAM metallopeptidase domain 11(ADAM11)
M_145	57452	polypeptide N-acetylgalactosaminyltransferase
M_145	286238	uncharacterized LOC286238(LOC286238)
M_145	2719	glypican 3(GPC3)
M_145	339483	myotubularin related protein 9-like,
M_145	57597	BAH domain and coiled-coil containing 1(BAHCC1)
M_145	55203	leucine rich repeat LGI family member 2(LGI2)
M_145	54972	transmembrane protein 132A(TMEM132A)
M_145	146325	proline rich 35(PRR35)
M_145	64919	B-cell CLL/lymphoma 11B(BCL11B)
M_146	55765	chromosome 1 open reading frame 106(C1orf106)
M_146	23508	tetratricopeptide repeat domain 9(TTC9)
M_146	374918	IGF like family member 1(IGFL1)
M_146	3852	keratin 5(KRT5)

M_146	3851	keratin 4(KRT4)
M_146	135656	diffuse panbronchiolitis critical region 1(DPCR1)
M_146	768239	prosaposin-like 1 (gene/pseudogene)(PSAPL1)
M_146	66002	cytochrome P450 family 4 subfamily F member
M_146	66004	Ly6/neurotoxin 1(LYNX1)
M_146	121391	keratin 74(KRT74)
M_146	2036	erythrocyte membrane protein band 4.1 like
M_146	394263	mucin 21, cell surface associated(MUC21)
M_146	10904	bladder cancer associated protein(BLCAP)
M_146	5318	plakophilin 2(PKP2)
M_146	5317	plakophilin 1(PKP1)
M_146	55287	transmembrane protein 40(TMEM40)
M_146	3958	galectin 3(LGALS3)
M_146	343990	KIAA1211 like(KIAA1211L)
M_146	51523	CXXC finger protein 5(CXXC5)
M_146	154796	angiomotin(AMOT)
M_146	2520	gastrin(GAST)
M_146	28954	RRAD and GEM like GTPase 1(REM1)
M_146	23286	WW and C2 domain containing 1(WWC1)
M_146	3860	keratin 13(KRT13)
M_146	11283	cytochrome P450 family 4 subfamily F member
M_146	5097	protocadherin 1(PCDH1)
M_146	3866	keratin 15(KRT15)
M_146	7477	Wnt family member 7B(WNT7B)
M_146	51450	paired related homeobox 2(PRRX2)
M_146	130814	PQ loop repeat containing 3(PQLC3)
M_146	9022	chloride intracellular channel 3(CLIC3)
M_146	4051	cytochrome P450 family 4 subfamily F member
M_146	79098	chromosome 1 open reading frame 116(C1orf116)
M_146	56910	StAR related lipid transfer domain containing
M_146	54101	receptor interacting serine/threonine kinase 4(RIPK4)
M_146	57834	cytochrome P450 family 4 subfamily F member

M_146	26504	cyclin and CBS domain divalent metal cation transport mediator 4(CNNM4)
M_146	6278	S100 calcium binding protein A7(S100A7)
M_146	3880	keratin 19(KRT19)
M_146	128434	V-set and transmembrane domain containing 2
M_146	10149	adhesion G protein-coupled receptor G2(ADGRG2)
M_146	144100	pleckstrin homology domain containing
M_146	64072	cadherin related 23(CDH23)
M_146	9536	prostaglandin E synthase(PTGES)
M_146	8537	breast carcinoma amplified sequence 1(BCAS1)
M_146	57152	secreted LY6/PLAUR domain containing 1(SLURP1)
M_146	338324	S100 calcium binding protein A7A(S100A7A)
M_146	3857	keratin 9(KRT9)
M_146	94122	synaptotagmin like 5(SYTL5)
M_146	652995	urothelial cancer associated 1 (non-protein
M_146	137797	LY6/PLAUR domain containing 2(LYPD2)
M_146	525	ATPase H+ transporting V1 subunit B1(ATP6V1B1)
M_146	8581	lymphocyte antigen 6 complex, locus D(LY6D)
M_146	115572	family with sequence similarity 46 member
M_146	23220	deltex E3 ubiquitin ligase 4(DTX4)
M_146	84530	serine/arginine repetitive matrix 4(SRRM4)
M_146	4008	LIM domain 7(LMO7)
M_146	85455	dispatched RND transporter family member 2(DISP2)
M_146	1591	cytochrome P450 family 24 subfamily A member 1(CYP24A1)
M_146	6286	S100 calcium binding protein P(S100P)
M_146	114	adenylate cyclase 8(ADCY8)
M_146	688	Kruppel like factor 5(KLF5)
M_146	285768	long intergenic non-protein coding RNA
M_146	94234	forkhead box Q1(FOXQ1)
M_146	414152	chromosome 10 open reading frame 105(C10orf105)
M_146	55654	transmembrane protein 127(TMEM127)

M_146	29841	grainyhead like transcription factor 1(GRHL1)
M_146	40	acid sensing ion channel subunit 2(ASIC2)
M_146	5919	retinoic acid receptor responder 2(RARRES2)
M_146	51442	vestigial like family member 1(VGLL1)
M_146	26353	heat shock protein family B (small) member
M_146	389376	surfactant associated 2(SFTA2)
M_146	81493	syncoilin, intermediate filament protein(SYNC)
M_146	245937	defensin beta 124(DEFB124)
M_146	4992	olfactory receptor family 1 subfamily F member
M_146	54854	family with sequence similarity 83 member
M_146	94026	POM121 transmembrane nucleoporin like
M_146	26184	olfactory receptor family 1 subfamily F member 2 pseudogene(OR1F2P)
M_146	100130386	long intergenic non-protein coding RNA
M_146	5145	phosphodiesterase 6A(PDE6A)
M_146	11156	protein tyrosine phosphatase type IVA, member
M_146	642475	maestro heat like repeat family member 6(MROH6)
M_146	5126	proprotein convertase subtilisin/kexin type 2(PCSK2)
M_146	6280	S100 calcium binding protein A9(S100A9)
M_146	6279	S100 calcium binding protein A8(S100A8)
M_146	23581	caspase 14(CASP14)
M_146	79444	baculoviral IAP repeat containing 7(BIRC7)
M_146	171169	sperm acrosome associated 4(SPACA4)
M_146	140875	long intergenic non-protein coding RNA
M_146	6511	solute carrier family 1 member 6(SLC1A6)
M_146	6283	S100 calcium binding protein A12(S100A12)
M_147	79949	pleckstrin homology domain containing
M_147	4000	lamin A/C(LMNA)
M_147	1356	ceruloplasmin(CP)
M_147	440836	outer dense fiber of sperm tails 3B(ODF3B)
M_147	3359	5-hydroxytryptamine receptor 3A(HTR3A)
M_147	10406	WAP four-disulfide core domain 2(WFDC2)

M_147	6948	transcobalamin 2(TCN2)
M_147	706	translocator protein(TSPO)
M_147	6237	related RAS viral (r-ras) oncogene homolog(RRAS)
M_147	10568	solute carrier family 34 member 2(SLC34A2)
M_147	1476	cystatin B(CSTB)
M_147	79056	proline rich and Gla domain 4(PRRG4)
M_147	55647	RAB20, member RAS oncogene family(RAB20)
M_147	6281	S100 calcium binding protein A10(S100A10)
M_147	3454	interferon alpha and beta receptor subunit 1(IFNAR1)
M_147	5266	peptidase inhibitor 3(PI3)
M_147	6590	secretory leukocyte peptidase inhibitor(SLPI)
M_147	3588	interleukin 10 receptor subunit beta(IL10RB)
M_147	26298	ETS homologous factor(EHF)
M_147	6282	S100 calcium binding protein A11(S100A11)
M_147	8878	sequestosome 1(SQSTM1)
M_147	1999	E74 like ETS transcription factor 3(ELF3)
M_147	2495	ferritin heavy chain 1(FTH1)
M_147	26499	pleckstrin 2(PLEK2)
M_147	140576	S100 calcium binding protein A16(S100A16)
M_147	6277	S100 calcium binding protein A6(S100A6)
M_147	1535	cytochrome b-245 alpha chain(CYBA)
M_147	123036	tandem C2 domains, nuclear(TC2N)
M_147	638	BCL2 interacting killer(BIK)
M_147	6284	S100 calcium binding protein A13(S100A13)
M_147	51084	crystallin lambda 1(CRYL1)
M_147	6271	S100 calcium binding protein A1(S100A1)
M_147	83938	chromosome 10 open reading frame 11(C10orf11)
M_147	51635	dehydrogenase/reductase 7(DHRS7)
M_147	10577	NPC intracellular cholesterol transporter 2(NPC2)
M_147	64866	CUB domain containing protein 1(CDCP1)
M_147	84419	chromosome 15 open reading frame 48(C15orf48)
M_147	2217	Fc fragment of IgG receptor and transporter(FCGRT)

M_147	121838	long intergenic non-protein coding RNA
M_147	4582	mucin 1, cell surface associated(MUC1)
M_147	64078	solute carrier family 28 member 3(SLC28A3)
M_147	3455	interferon alpha and beta receptor subunit 2(IFNAR2)
M_147	3460	interferon gamma receptor 2 (interferon gamma transducer 1)(IFNGR2)
M_147	60598	potassium two pore domain channel subfamily K member 15(KCNK15)
M_147	64170	caspase recruitment domain family member
M_147	4615	myeloid differentiation primary response 88(MYD88)
M_147	5872	RAB13, member RAS oncogene family(RAB13)
M_147	8850	lysine acetyltransferase 2B(KAT2B)
M_147	1992	serpin family B member 1(SERPINB1)
M_147	9695	ER degradation enhancing alpha-mannosidase like protein 1(EDEM1)
M_147	10311	DSCR3 arrestin fold containing(DSCR3)
M_147	6001	regulator of G-protein signaling 10(RGS10)
M_147	1718	24-dehydrocholesterol reductase(DHCR24)
M_147	1051	CCAAT/enhancer binding protein beta(CEBPB)
M_147	1116	chitinase 3 like 1(CHI3L1)
M_147	4608	myosin binding protein H(MYBPH)
M_147	54933	rhomboid like 2(RHBDL2)
M_147	145200	long intergenic non-protein coding RNA
M_147	3732	CD82 molecule(CD82)
M_147	79679	V-set domain containing T cell activation inhibitor
M_147	7127	TNF alpha induced protein 2(TNFAIP2)
M_147	91828	exocyst complex component 3 like 4(EXOC3L4)
M_147	1942	ephrin A1(EFNA1)
M_148	1845	dual specificity phosphatase 3(DUSP3)
M_148	2064	erb-b2 receptor tyrosine kinase 2(ERBB2)
M_148	122773	kelch domain containing 1(KLHDC1)
M_148	51096	UTP18, small subunit processome component(UTP18)

M_148	51629	solute carrier family 25 member 39(SLC25A39)
M_148	6388	stromal cell derived factor 2(SDF2)
M_148	6945	MLX, MAX dimerization protein(MLX)
M_148	26073	DNA polymerase delta interacting protein
M_148	128229	TSSK6 activating cochaperone(TSACC)
M_148	92579	glucose-6-phosphatase catalytic subunit 3(G6PC3)
M_148	201229	LYR motif containing 9(LYRM9)
M_148	493753	cytochrome c oxidase assembly factor 5(COA5)
M_148	4669	N-acetyl-alpha-glucosaminidase(NAGLU)
M_148	147184	transmembrane protein 99(TMEM99)
M_148	28958	cytochrome c oxidase assembly factor 3(COA3)
M_148	124801	LSM12 homolog(LSM12)
M_148	10040	target of myb1 like 1 membrane trafficking
M_148	5718	proteasome 26S subunit, non-ATPase 12(PSMD12)
M_148	10241	calcium binding and coiled-coil domain
M_148	115024	5'-nucleotidase, cytosolic IIIB(NT5C3B)
M_148	144321	GLI pathogenesis-related 1 like 2(GLIPR1L2)
M_148	2896	granulin precursor(GRN)
M_148	90410	intraflagellar transport 20(IFT20)
M_148	348235	spindle and kinetochore associated complex subunit
M_148	10209	eukaryotic translation initiation factor 1(EIF1)
M_148	94103	ORMDL sphingolipid biosynthesis regulator
M_148	116236	abhydrolase domain containing 15(ABHD15)
M_148	5476	cathepsin A(CTSA)
M_148	5691	proteasome subunit beta 3(PSMB3)
M_148	9094	unc-119 lipid binding chaperone(UNC119)
M_148	441549	cerebral dopamine neurotrophic factor(CDNF)
M_148	129531	microtubule interacting and trafficking domain containing 1(MITD1)
M_148	5709	proteasome 26S subunit, non-ATPase 3(PSMD3)
M_148	79922	mitochondrial rRNA methyltransferase 1(MRM1)
M_148	84299	migration and invasion enhancer 1(MIEN1)

M_148	84311	mitochondrial ribosomal protein L45(MRPL45)
M_148	27249	methylmalonic aciduria and homocystinuria, cblD type(MMADHC)
M_148	7283	tubulin gamma 1(TUBG1)
M_148	84313	vacuolar protein sorting 25 homolog(VPS25)
M_148	1353	COX11, cytochrome c oxidase copper
M_148	3858	keratin 10(KRT10)
M_148	84316	N(alpha)-acetyltransferase 38, NatC auxiliary subunit(NAA38)
M_148	11072	dual specificity phosphatase 14(DUSP14)
M_148	9326	zinc finger HIT-type containing 3(ZNHIT3)
M_148	147007	transmembrane protein 199(TMEM199)
M_148	25926	nucleolar protein 11(NOL11)
M_148	5878	RAB5C, member RAS oncogene family(RAB5C)
M_148	10948	StAR related lipid transfer domain containing
M_148	25972	unc-50 inner nuclear membrane RNA binding
M_148	29916	sorting nexin 11(SNX11)
M_148	284106	CDGSH iron sulfur domain 3(CISD3)
M_148	5641	legumain(LGMN)
M_148	90313	tumor protein p53 inducible protein 13(TP53I13)
M_148	125113	keratin 222(KRT222)
M_148	55163	pyridoxamine 5'-phosphate oxidase(PNPO)
M_148	124995	mitochondrial ribosomal protein L10(MRPL10)
M_148	339175	methyltransferase like 2A(METTL2A)
M_148	10190	thioredoxin domain containing 9(TXNDC9)
M_148	79154	dehydrogenase/reductase 11(DHRS11)
M_148	5193	peroxisomal biogenesis factor 12(PEX12)
M_148	93210	post-GPI attachment to proteins 3(PGAP3)
M_148	401115	chromosome 4 open reading frame 48(C4orf48)
M_148	254863	transmembrane protein 256(TMEM256)
M_148	55352	coordinator of PRMT5 and differentiation
M_148	623	bradykinin receptor B1(BDKRB1)

M_148	624	bradykinin receptor B2(BDKRB2)
M_148	5409	phenylethanolamine N-methyltransferase(PNMT)
M_148	7067	thyroid hormone receptor, alpha(THRA)
M_148	79990	pleckstrin homology, MyTH4 and FERM domain containing H3(PLEKHH3)
M_148	8557	titin-cap(TCAP)
M_148	51402	#N/A
M_148	2886	growth factor receptor bound protein 7(GRB7)
M_149	116511	MAS1 proto-oncogene like, G protein-coupled receptor(MAS1L)
M_149	204474	protein disulfide isomerase like, testis
M_149	283111	olfactory receptor family 51 subfamily V member
M_149	149934	nuclear receptor corepressor 1 pseudogene
M_149	5449	POU class 1 homeobox 1(POU1F1)
M_149	219958	olfactory receptor family 1 subfamily S member
M_149	26245	olfactory receptor family 2 subfamily M member
M_149	128861	BPI fold containing family A member 3(BPIFA3)
M_149	503582	arginine-fifty homeobox(ARGFX)
M_149	492307	chromosome 8 open reading frame 22(C8orf22)
M_149	254950	keratin associated protein 15-1(KRTAP15-1)
M_149	344191	even-skipped homeobox 2(EVX2)
M_149	255025	long intergenic non-protein coding RNA
M_149	154907	chromosome 7 open reading frame 66(C7orf66)
M_149	341799	olfactory receptor family 6 subfamily S member
M_149	1406	cone-rod homeobox(CRX)
M_149	4184	sperm mitochondria associated cysteine rich
M_149	3982	lens intrinsic membrane protein 2(LIM2)
M_149	728096	cancer/testis antigen family 47, member A1(CT47A1)
M_149	728062	cancer/testis antigen family 47, member A6(CT47A6)
M_149	284274	small integral membrane protein 21(SMIM21)
M_149	245932	defensin beta 119(DEFB119)
M_149	51802	acid sensing ion channel subunit family member

M_149	729447	G antigen 2A(GAGE2A)
M_149	81833	sperm acrosome associated 1(SPACA1)
M_149	283197	long intergenic non-protein coding RNA
M_149	4490	metallothionein 1B(MT1B)
M_149	81797	olfactory receptor family 12 subfamily D member
M_149	359806	proliferating cell nuclear antigen pseudogene
M_149	10246	solute carrier family 17 member 2(SLC17A2)
M_149	91074	ankyrin repeat domain 30A(ANKRD30A)
M_149	100131816	ubiquitin conjugating enzyme E2 D N-terminal like (pseudogene)(UBE2DNL)
M_149	153745	family with sequence similarity 71 member
M_149	391004	PRAME family member 17(PRAMEF17)
M_149	100129239	chromosome X open reading frame 51A(CXorf51A)
M_149	100129271	chromosome 1 open reading frame 68(C1orf68)
M_149	131149	otolin 1(OTOL1)
M_149	390648	olfactory receptor family 4 subfamily F member
M_149	81696	olfactory receptor family 5 subfamily V member
M_149	3630	insulin(INS)
M_149	79317	olfactory receptor family 4 subfamily K member
M_149	442361	olfactory receptor family 2 subfamily A member
M_149	79295	olfactory receptor family 5 subfamily H member 6 (gene/pseudogene)(OR5H6)
M_149	284123	family with sequence similarity E5(FAM27E5)
M_149	340168	developmental pluripotency associated 5(DPPA5)
M_149	100170228	small ILF3/NF90-associated RNA G2(SNAR-G2)
M_149	128367	olfactory receptor family 10 subfamily X member 1 (gene/pseudogene)(OR10X1)
M_149	390431	olfactory receptor family 4 subfamily K member
M_149	200959	gamma-aminobutyric acid type A receptor rho3 subunit (gene/pseudogene)(GABRR3)
M_149	126370	olfactory receptor family 1 subfamily I member
M_149	337977	keratin associated protein 21-1(KRTAP21-1)

M_149	654348	#N/A
M_149	84643	kinesin family member 2B(KIF2B)
M_149	26686	olfactory receptor family 4 subfamily E member
M_149	90655	TGFB induced factor homeobox 2 like, Y-
M_149	391109	olfactory receptor family 10 subfamily K member
M_149	391107	olfactory receptor family 10 subfamily K member
M_149	56163	ring finger protein 17(RNF17)
M_149	140850	defensin beta 127(DEFB127)
M_149	677845	small nucleolar RNA, H/ACA box 79(SNORA79)
M_149	645037	G antigen 2B(GAGE2B)
M_149	442184	olfactory receptor family 2 subfamily B member
M_149	442186	olfactory receptor family 2 subfamily J member
M_149	83954	#N/A
M_149	120146	tripartite motif containing 64(TRIM64)
M_149	5506	protein phosphatase 1 regulatory subunit
M_149	139562	OTU deubiquitinase 6A(OTUD6A)
M_149	139538	VENT homeobox pseudogene 1(VENTXP1)
M_149	130560	spermatogenesis associated 3(SPATA3)
M_149	729396	G antigen 12J(GAGE12J)
M_149	219469	olfactory receptor family 8 subfamily H member
M_149	219477	olfactory receptor family 8 subfamily J member
M_149	337970	keratin associated protein 19-3(KRTAP19-3)
M_149	337968	keratin associated protein 6-3(KRTAP6-3)
M_149	390264	olfactory receptor family 10 subfamily G member
M_149	9084	variable charge, Y-linked(VCY)
M_149	391253	serine peptidase inhibitor, Kunitz type 4(SPINT4)
M_149	9506	PAGE family member 4(PAGE4)
M_149	2158	coagulation factor IX(F9)
M_149	391194	olfactory receptor family 2 subfamily M member
M_149	282770	olfactory receptor family 10 subfamily AG member 1(OR10AG1)
M_149	645104	clarin 2(CLRN2)

M_149	645051	G antigen 13(GAGE13)
M_149	441669	olfactory receptor family 4 subfamily Q member
M_149	140801	ribosomal protein L10 like(RPL10L)
M_149	441670	olfactory receptor family 4 subfamily M member
M_149	84616	keratin associated protein 4-4(KRTAP4-4)
M_149	139793	PAGE family member 3(PAGE3)
M_149	10895	pro-platelet basic protein pseudogene 2(PPBPP2)
M_149	81050	olfactory receptor family 5 subfamily AC member 2(OR5AC2)
M_149	81127	olfactory receptor family 4 subfamily K member
M_149	81168	olfactory receptor family 8 subfamily J member
M_149	63974	neuronal differentiation 6(NEUROD6)
M_149	100124540	small nucleolar RNA, H/ACA box 11C(SNORA11C)
M_149	392138	olfactory receptor family 2 subfamily A member
M_149	8756	ADAM metallopeptidase domain 7(ADAM7)
M_149	56934	carbonic anhydrase 10(CA10)
M_149	388910	long intergenic non-protein coding RNA
M_149	222029	uncharacterized protein
M_149	3886	keratin 35(KRT35)
M_149	386685	keratin associated protein 10-12(KRTAP10-12)
M_149	132612	adenosine deaminase domain containing 1(ADAD1)
M_149	168741	period circadian clock 3 pseudogene(PER4)
M_149	152138	pyrin domain containing 2(PYDC2)
M_149	3448	interferon alpha 14(IFNA14)
M_149	3451	interferon alpha 17(IFNA17)
M_149	3446	interferon alpha 10(IFNA10)
M_149	122258	sperm acrosome associated 7(SPACA7)
M_149	403315	family with sequence similarity 92, member
M_149	392399	lipocalin 9(LCN9)
M_149	403282	olfactory receptor family 6 subfamily C member
M_149	2994	glycophorin B (MNS blood group)(GYPB)
M_149	26492	olfactory receptor family 8 subfamily G member

M_149	503834	divergent-paired related homeobox(DPRX)
M_149	401067	IQ motif containing F3(IQCF3)
M_149	390157	olfactory receptor family 8 subfamily K member
M_149	692149	small Cajal body-specific RNA 14(SCARNA14)
M_149	140464	polled intersex syndrome regulated transcript 1 (non-protein coding RNA)(PISRT1)
M_149	387486	long intergenic non-protein coding RNA
M_149	285370	long intergenic non-protein coding RNA
M_149	653282	cancer/testis antigen family 47, member A7(CT47A7)
M_149	127062	olfactory receptor family 2 subfamily M member
M_149	319100	trace amine associated receptor 6(TAAR6)
M_149	23538	olfactory receptor family 52 subfamily A member
M_149	441601	septin 7 pseudogene(LOC441601)
M_149	100170219	small ILF3/NF90-associated RNA C4(SNAR-C4)
M_149	219447	olfactory receptor family 5 subfamily AS member
M_149	283914	long intergenic non-protein coding RNA
M_149	389763	SPATA31 subfamily D member 1(SPATA31D1)
M_149	763	carbonic anhydrase 5A(CA5A)
M_149	123970	chromosome 16 open reading frame 78(C16orf78)
M_149	643905	proline rich 21(PRR21)
M_149	340260	UNC homeobox(UNCX)
M_149	3744	potassium voltage-gated channel subfamily A member 10(KCNA10)
M_149	100129128	KH domain containing 1 like(KHDC1L)
M_149	653606	PRAME family member 22(PRAMEF22)
M_149	166655	tripartite motif containing 60(TRIM60)
M_149	644524	NK2 homeobox 4(NKX2-4)
M_149	317703	vomeronasal 1 receptor 4(VN1R4)
M_149	791115	Prader-Willi region non-protein coding RNA
M_149	202865	chromosome 7 open reading frame 33(C7orf33)
M_149	4142	MAS1 proto-oncogene, G protein-coupled
M_149	391003	PRAME family member 18(PRAMEF18)

M_149	58158	neuronal differentiation 4(NEUROD4)
M_149	26716	olfactory receptor family 2 subfamily H member
M_149	3262	human papillomavirus (type 18) E5 central sequence-like 1(HPVC1)
M_149	56287	gastrokine 1(GKN1)
M_149	1485	cancer/testis antigen 1B(CTAG1B)
M_149	932	membrane spanning 4-domains A3(MS4A3)
M_149	125115	keratin 40(KRT40)
M_149	83942	testis specific serine kinase 1B(TSSK1B)
M_149	390445	olfactory receptor family 5 subfamily AU member 1(OR5AU1)
M_149	4760	neuronal differentiation 1(NEUROD1)
M_149	4762	neurogenin 1(NEUROG1)
M_149	284383	olfactory receptor family 2 subfamily Z member
M_149	1669	defensin alpha 4(DEFA4)
M_149	2165	coagulation factor XIII B chain(F13B)
M_149	285622	neuroblastoma breakpoint family member 22, pseudogene(NBPF22P)
M_149	1641	doublecortin(DCX)
M_149	219493	olfactory receptor family 5 subfamily AR member 1 (gene/pseudogene)(OR5AR1)
M_149	440799	purinergic receptor P2X 6 pseudogene(P2RX6P)
M_149	619569	small nucleolar RNA, H/ACA box 41(SNORA41)
M_149	30848	cancer/testis antigen 2(CTAG2)
M_149	126204	NLR family pyrin domain containing 13(NLRP13)
M_149	84103	chromosome 4 open reading frame 17(C4orf17)
M_149	200504	gastrokine 2(GKN2)
M_149	265	amelogenin, X-linked(AMELX)
M_149	89778	serpin family B member 11
M_149	100101938	ankyrin repeat domain 26 pseudogene
M_149	9607	CART prepropeptide(CARTPT)
M_149	51412	actin like 6B(ACTL6B)

M_149	390003	guanylate cyclase 2G, pseudogene(GUCY2GP)
M_149	284546	chromosome 1 open reading frame 185(C1orf185)
M_149	4808	nescient helix-loop-helix 2(NHLH2)
M_149	145497	leucine rich repeat containing 74A(LRRC74A)
M_149	136991	ankyrin repeat, SAM and basic leucine zipper domain containing 1(ASZ1)
M_149	201140	dehydrogenase/reductase 7C(DHRS7C)
M_149	1995	ELAV like RNA binding protein 3(ELAVL3)
M_149	154790	C-type lectin domain family 2 member L(CLEC2L)
M_149	373509	ubiquitin specific peptidase 50(USP50)
M_149	286183	Na+/K+ transporting ATPase interacting 3(NKAIN3)
M_149	403274	olfactory receptor family 5 subfamily H member
M_149	255725	olfactory receptor family 52 subfamily B member
M_149	1814	dopamine receptor D3(DRD3)
M_149	146861	solute carrier family 35 member G3(SLC35G3)
M_149	137814	NK2 homeobox 6(NKX2-6)
M_149	653544	double homeobox 4 like 6(DUX4L6)
M_149	344018	folliculogenesis specific bHLH transcription
M_149	8929	paired like homeobox 2b(PHOX2B)
M_149	27302	bone morphogenetic protein 10(BMP10)
M_149	344838	progestin and adipoQ receptor family member
M_149	284379	solute carrier family 7 member 3
M_149	148545	neuroblastoma breakpoint family member 4(NBPF4)
M_149	131	alcohol dehydrogenase 7 (class IV), mu or sigma polypeptide(ADH7)
M_149	259240	WAP four-disulfide core domain 9(WFDC9)
M_149	653149	neuroblastoma breakpoint family member 6(NBPF6)
M_149	728410	double homeobox 4 like 2(DUX4L2)
M_149	643763	uncharacterized LOC643763(UG0898H09)
M_149	340198	interferon induced transmembrane protein 4 pseudogene(IFITM4P)
M_149	2831	neuropeptides B/W receptor 1(NPBWR1)

M_149	10850	C-C motif chemokine ligand 27(CCL27)
M_149	2986	guanylate cyclase 2F, retinal(GUCY2F)
M_149	283303	MRGPRG antisense RNA 1(MRGPRG-AS1)
M_149	619568	small nucleolar RNA, H/ACA box 4(SNORA4)
M_149	139804	RNA binding motif protein, X-linked like
M_149	3889	keratin 83(KRT83)
M_150	158046	nucleoredoxin-like 2(NXNL2)
M_150	158248	tetratricopeptide repeat domain 16(TTC16)
M_150	124093	coiled-coil domain containing 78(CCDC78)
M_150	1580	cytochrome P450 family 4 subfamily B member
M_150	81693	amnion associated transmembrane protein(AMN)
M_150	7490	Wilms tumor 1(WT1)
M_150	55805	LRP2 binding protein(LRP2BP)
M_150	51352	WT1 antisense RNA(WT1-AS)
M_150	50944	SH3 and multiple ankyrin repeat domains
M_150	130540	amyotrophic lateral sclerosis 2 chromosome region candidate 12(ALS2CR12)
M_150	150248	chromosome 22 open reading frame 15(C22orf15)
M_150	6523	solute carrier family 5 member 1(SLC5A1)
M_150	6850	spleen associated tyrosine kinase(SYK)
M_150	6320	C-type lectin domain family 11 member A(CLEC11A)
M_150	8030	coiled-coil domain containing 6(CCDC6)
M_150	146330	F-box and leucine rich repeat protein 16(FBXL16)
M_150	196385	dynein axonemal heavy chain 10(DNAH10)
M_150	343521	Tctex1 domain containing 4(TCTEX1D4)
M_150	25833	POU class 2 homeobox 3(POU2F3)
M_150	9351	SLC9A3 regulator 2(SLC9A3R2)
M_150	2099	estrogen receptor 1(ESR1)
M_150	5742	prostaglandin-endoperoxide synthase 1(PTGS1)
M_150	1262	cyclic nucleotide gated channel alpha 4(CNGA4)
M_150	10232	mesothelin(MSLN)

M_150	51704	G protein-coupled receptor class C group 5 member B(GPRC5B)
M_150	92196	death associated protein like 1(DAPL1)
M_150	149483	coiled-coil domain containing 17(CCDC17)
M_150	401827	mesothelin-like(MSLNL)
M_150	127579	DC-STAMP domain containing 2(DCST2)
M_150	266675	bestrophin 4(BEST4)
M_150	79841	ATP/GTP binding protein like 2(AGBL2)
M_150	9028	rhomboid like 1(RHBDL1)
M_150	339541	chromosome 1 open reading frame 228(C1orf228)
M_150	441054	chromosome 4 open reading frame 47(C4orf47)
M_150	79006	meteorin, glial cell differentiation regulator(METRN)
M_150	84264	hydroxyacylglutathione hydrolase-like(HAGHL)
M_150	283229	calcium release activated channel regulator
M_150	3570	interleukin 6 receptor(IL6R)
M_150	149095	DC-STAMP domain containing 1(DCST1)
M_150	26636	#N/A
M_150	100126784	uncharacterized LOC100126784(LOC100126784)
M_150	6339	sodium channel epithelial 1 delta subunit(SCNN1D)
M_150	201501	zinc finger and BTB domain containing 7C(ZBTB7C)
M_150	150921	transcription factor 23(TCF23)
M_150	89797	neuron navigator 2(NAV2)
M_150	27065	neuron specific gene family member 1(NSG1)
M_150	5444	paraoxonase 1(PON1)
M_150	7368	UDP glycosyltransferase 8(UGT8)
M_150	2348	folate receptor 1(FOLR1)
M_150	2352	folate receptor 3(FOLR3)
M_150	79906	MORN repeat containing 1(MORN1)
M_150	22844	FERM and PDZ domain containing 1(FRMPD1)
M_150	11001	solute carrier family 27 member 2(SLC27A2)
M_150	10279	protease, serine 16(PRSS16)
M_150	8928	forkhead box H1(FOXH1)

M_150	100129534	small nuclear ribonucleoprotein polypeptide N pseudogene(LOC100129534)
M_151	192666	keratin 24(KRT24)
M_151	23671	transmembrane protein with EGF like and two follistatin like domains 2(TMEFF2)
M_151	117154	dachshund family transcription factor 2(DACH2)
M_151	5268	serpin family B member 5(SERPINB5)
M_151	54886	phospholipid phosphatase related 1(PLPPR1)
M_151	100128554	uncharacterized LOC100128554(LOC100128554)
M_151	56479	potassium voltage-gated channel subfamily Q member 5(KCNQ5)
M_151	340578	DDB1 and CUL4 associated factor 12 like
M_151	27328	protocadherin 11 X-linked(PCDH11X)
M_151	6335	sodium voltage-gated channel alpha subunit
M_151	22943	dickkopf WNT signaling pathway inhibitor 1(DKK1)
M_151	9378	neurexin 1(NRXN1)
M_151	3760	potassium voltage-gated channel subfamily J member 3(KCNJ3)
M_151	10718	neuregulin 3(NRG3)
M_151	114798	SLIT and NTRK like family member 1(SLITRK1)
M_151	253559	cell adhesion molecule 2(CADM2)
M_151	577	adhesion G protein-coupled receptor B3(ADGRB3)
M_151	64881	protocadherin 20(PCDH20)
M_151	22854	netrin G1(NTNG1)
M_151	341640	FRAS1 related extracellular matrix protein 2(FREM2)
M_151	147381	cerebellin 2 precursor(CBLN2)
M_151	27012	potassium voltage-gated channel modifier subfamily V member 1(KCNV1)
M_151	139170	DDB1 and CUL4 associated factor 12 like
M_151	342574	keratin 27(KRT27)
M_151	1620	BMP/retinoic acid inducible neural specific
M_151	2894	glutamate ionotropic receptor delta type subunit

M_151	348980	hyperpolarization activated cyclic nucleotide gated potassium channel 1(HCN1)
M_151	83259	protocadherin 11 Y-linked(PCDH11Y)
M_151	4916	neurotrophic receptor tyrosine kinase 3(NTRK3)
M_151	6091	roundabout guidance receptor 1(ROBO1)
M_151	2554	gamma-aminobutyric acid type A receptor alpha1 subunit(GABRA1)
M_151	2045	EPH receptor A7(EPHA7)
M_151	10891	PPARG coactivator 1 alpha(PPARGC1A)
M_151	4889	neuropeptide Y receptor Y5(NPY5R)
M_151	4886	neuropeptide Y receptor Y1(NPY1R)
M_151	1005	cadherin 7(CDH7)
M_151	643432	tumor suppressor TSG1(TSG1)
M_151	9073	claudin 8(CLDN8)
M_151	22986	sortilin related VPS10 domain containing receptor
M_151	387590	transmembrane phosphatase with tensin homology pseudogene 1(TPTEP1)
M_151	2555	gamma-aminobutyric acid type A receptor alpha2 subunit(GABRA2)
M_151	219578	zinc finger protein 804B(ZNF804B)
M_151	359822	KCNIP4 intronic transcript 1(KCNIP4-IT1)
M_151	285525	Yip1 domain family member 7(YIPF7)
M_151	10777	cAMP regulated phosphoprotein 21(ARPP21)
M_151	6860	synaptotagmin 4(SYT4)
M_151	84631	SLIT and NTRK like family member 2(SLITRK2)
M_151	9142	transmembrane protein 257(TMEM257)
M_151	2890	glutamate ionotropic receptor AMPA type subunit
M_152	282890	zinc finger protein 311(ZNF311)
M_152	399697	cortexin 2(CTXN2)
M_152	131831	glutamate rich 6(ERICH6)
M_152	282996	RNA binding motif protein 20(RBM20)
M_152	644890	meiosis/spermiogenesis associated 1(MEIG1)

M_152	100133985	uncharacterized LOC100133985(LOC100133985)
M_152	282997	PDCD4 antisense RNA 1(PDCD4-AS1)
M_152	220930	ZEB1 antisense RNA 1(ZEB1-AS1)
M_152	6647	superoxide dismutase 1, soluble(SOD1)
M_152	79857	uncharacterized LOC79857(FLJ13224)
M_152	283416	long intergenic non-protein coding RNA
M_152	143282	fibroblast growth factor binding protein 3(FGFBP3)
M_152	220992	zinc finger protein 485(ZNF485)
M_152	64968	mitochondrial ribosomal protein S6(MRPS6)
M_152	200765	tigger transposable element derived 1(TIGD1)
M_152	8624	proteasome assembly chaperone 1(PSMG1)
M_152	128854	testis specific protein, Y-linked 26,
M_152	89894	transmembrane protein 116(TMEM116)
M_152	1824	desmocollin 2(DSC2)
M_152	255758	Tctex1 domain containing 2(TCTEX1D2)
M_152	54753	zinc finger protein 853(ZNF853)
M_152	55231	coiled-coil domain containing 87(CCDC87)
M_152	651302	zinc finger protein 192 pseudogene 1(ZNF192P1)
M_152	144486	CEP83 antisense RNA 1 (head to head)(CEP83-AS1)
M_152	440356	CDIPT antisense RNA 1 (head to head)(CDIPT-AS1)
M_152	54943	DnaJ heat shock protein family (Hsp40) member C28(DNAJC28)
M_152	100009676	ZBTB11 antisense RNA 1(ZBTB11-AS1)
M_152	23682	RAB38, member RAS oncogene family(RAB38)
M_152	5723	phosphoserine phosphatase(PSPH)
M_152	55089	solute carrier family 38 member 4(SLC38A4)
M_152	644538	small integral membrane protein 10(SMIM10)
M_152	54065	small integral membrane protein 11A(SMIM11A)
M_152	10694	chaperonin containing TCP1 subunit 8(CCT8)
M_152	3185	heterogeneous nuclear ribonucleoprotein F(HNRNPF)
M_152	7570	zinc finger protein 22(ZNF22)
M_152	339674	long intergenic non-protein coding RNA

M_152	728819	C1GALT1-specific chaperone 1 like(C1GALT1C1L)
M_152	286016	triosephosphate isomerase 1 pseudogene 2(TPI1P2)
M_152	2631	glioblastoma amplified sequence(GBAS)
M_152	222696	zinc finger and SCAN domain containing
M_152	222699	transducer of ERBB2, 2 pseudogene 1(TOB2P1)
M_152	220979	chromosome 10 open reading frame 25(C10orf25)
M_152	54148	mitochondrial ribosomal protein L39(MRPL39)
M_152	9946	crystallin zeta like 1(CRYZL1)
M_152	203562	transmembrane protein 31(TMEM31)
M_152	1823	desmocollin 1(DSC1)
M_152	27250	programmed cell death 4 (neoplastic transformation inhibitor)(PDCD4)
M_152	9992	potassium voltage-gated channel subfamily E regulatory subunit 2(KCNE2)
M_152	114821	zinc finger BED-type containing 9(ZBED9)
M_152	1828	desmoglein 1(DSG1)
M_152	8187	zinc finger protein 239(ZNF239)
M_152	11077	heat shock transcription factor 2 binding
M_152	908	chaperonin containing TCP1 subunit 6A(CCT6A)
M_152	144571	A2M antisense RNA 1 (head to head)(A2M-AS1)
M_152	100125556	family with sequence similarity 86, member A pseudogene(FAM86JP)
M_152	144715	RAD9 checkpoint clamp component B(RAD9B)
M_152	5608	mitogen-activated protein kinase kinase 6(MAP2K6)
M_152	63979	fidgetin like 1(FIGNL1)
M_152	340267	collagen type XXVIII alpha 1 chain(COL28A1)
M_152	1128	cholinergic receptor muscarinic 1(CHRM1)
M_152	83464	aph-1 homolog B, gamma-secretase subunit(APH1B)
M_152	440072	long intergenic non-protein coding RNA
M_152	2050	EPH receptor B4(EPHB4)
M_152	539	ATP synthase, H+ transporting, mitochondrial F1 complex, O subunit(ATP5O)

M_152	55065	solute carrier family 52 member 1(SLC52A1)
M_152	757	transmembrane protein 50B(TMEM50B)
M_152	30813	visual system homeobox 1(VSX1)
M_152	7479	Wnt family member 8B(WNT8B)
M_152	2170	fatty acid binding protein 3(FABP3)
M_152	23026	myosin XVI(MYO16)
M_152	4601	MAX interactor 1, dimerization protein(MXI1)
M_152	6934	transcription factor 7 like 2(TCF7L2)
M_152	219938	spermatogenesis associated 19(SPATA19)
M_152	143188	uncharacterized LOC143188(LOC143188)
M_152	7582	zinc finger protein 33B(ZNF33B)
M_152	255928	synaptotagmin 14(SYT14)
M_152	522	ATP synthase, H+ transporting, mitochondrial Fo complex subunit F6(ATP5J)
M_152	145783	uncharacterized LOC145783(LOC145783)
M_152	94239	H2A histone family member V(H2AFV)
M_152	200844	chromosome 3 open reading frame 67(C3orf67)
M_152	84532	acyl-CoA synthetase short-chain family member
M_153	284346	zinc finger protein 575(ZNF575)
M_153	23474	ETHE1, persulfide dioxygenase(ETHE1)
M_153	122970	acyl-CoA thioesterase 4(ACOT4)
M_153	284338	proline rich 19(PRR19)
M_153	29950	SERTA domain containing 1(SERTAD1)
M_153	27076	LY6/PLAUR domain containing 3(LYPD3)
M_153	4294	mitogen-activated protein kinase kinase kinase
M_153	55101	ATP5S like(ATP5SL)
M_153	53916	RAB4B, member RAS oncogene family(RAB4B)
M_153	26996	G protein-coupled receptor 160(GPR160)
M_153	56548	carbohydrate sulfotransferase 7(CHST7)
M_153	11100	heterogeneous nuclear ribonucleoprotein U like 1(HNRNPUL1)
M_153	92799	SH3KBP1 binding protein 1(SHKBP1)

M_153	112398	egl-9 family hypoxia inducible factor 2(EGLN2)
M_153	284325	chromosome 19 open reading frame 54(C19orf54)
M_153	84518	cornifelin(CNFN)
M_153	80271	inositol-trisphosphate 3-kinase C(ITPKC)
M_153	6626	small nuclear ribonucleoprotein polypeptide
M_153	126526	chromosome 19 open reading frame 47(C19orf47)
M_153	23646	phospholipase D family member 3(PLD3)
M_153	2077	ETS2 repressor factor(ERF)
M_153	6697	sepiapterin reductase (7,8-dihydrobiopterin:NADP+ oxidoreductase)(SPR)
M_153	27120	dickkopf like acrosomal protein 1(DKKL1)
M_153	208	AKT serine/threonine kinase 2(AKT2)
M_153	645	biliverdin reductase B(BLVRB)
M_153	199731	cell adhesion molecule 4(CADM4)
M_153	162989	death effector domain containing 2(DEDD2)
M_153	113452	transmembrane protein 54(TMEM54)
M_153	29946	SERTA domain containing 3(SERTAD3)
M_153	79934	coenzyme Q8B(COQ8B)
M_153	23152	capicua transcriptional repressor(CIC)
M_153	90324	coiled-coil domain containing 97(CCDC97)
M_153	5050	platelet activating factor acetylhydrolase 1b catalytic subunit 3(PAFAH1B3)
M_153	51177	pleckstrin homology domain containing
M_153	283152	coiled-coil domain containing 153(CCDC153)
M_153	8463	TEA domain transcription factor 2(TEAD2)
M_153	9253	NUMB like, endocytic adaptor protein(NUMBL)
M_153	4082	myristoylated alanine rich protein kinase C substrate(MARCKS)
M_153	79177	zinc finger protein 576(ZNF576)
M_153	1954	multiple EGF like domains 8(MEGF8)
M_153	147872	coiled-coil domain containing 155(CCDC155)
M_153	54913	ribonuclease P/MRP subunit p25(RPP25)

M_153	80776	B9 protein domain 2(B9D2)
M_153	10865	AT-rich interaction domain 5A(ARID5A)
M_153	9363	RAB33A, member RAS oncogene family(RAB33A)
M_153	399512	solute carrier family 25 member 35(SLC25A35)
M_153	3764	potassium voltage-gated channel subfamily J member 8(KCNJ8)
M_153	83694	ribosomal protein S6 kinase like 1(RPS6KL1)
M_153	10060	ATP binding cassette subfamily C member 9(ABCC9)
M_153	64215	DnaJ heat shock protein family (Hsp40) member C1(DNAJC1)
M_153	57716	periaxin(PRX)
M_153	147746	homeodomain interacting protein kinase 4(HIPK4)
M_153	113828	family with sequence similarity 83 member
M_153	25891	peptidase domain containing associated with muscle regeneration 1(PAMR1)
M_153	5251	phosphate regulating endopeptidase homolog, X-
M_153	5305	phosphatidylinositol-5-phosphate 4-kinase type 2 alpha(PIP4K2A)
M_153	2915	glutamate metabotropic receptor 5(GRM5)
M_154	347918	EP400 N-terminal like(EP400NL)
M_154	5380	PMS1 homolog 2, mismatch repair system component pseudogene 2(PMS2P2)
M_154	442578	stromal antigen 3-like 3 (pseudogene)(STAG3L3)
M_154	5387	PMS1 homolog 2, mismatch repair system component pseudogene 3(PMS2P3)
M_154	253018	HLA complex group 27 (non-protein coding)(HCG27)
M_154	375287	RNA binding motif protein 43(RBM43)
M_154	84680	1-aminocyclopropane-1-carboxylate synthase homolog (inactive)(ACCS)
M_154	27031	nephrocystin 3(NPHP3)
M_154	54441	stromal antigen 3-like 1 (pseudogene)(STAG3L1)
M_154	4144	methionine adenosyltransferase 2A(MAT2A)

M_154	338799	long intergenic non-protein coding RNA
M_154	80862	zinc ribbon domain containing 1 antisense, pseudogene(ZNRD1ASP)
M_154	55268	enoyl-CoA hydratase domain containing 2(ECHDC2)
M_154	100129405	misato family member 2, pseudogene(MSTO2P)
M_154	132241	ribosomal protein L32 pseudogene 3(RPL32P3)
M_154	260294	NOP2/Sun RNA methyltransferase family member 5 pseudogene 2(NSUN5P2)
M_154	245	arachidonate 12-lipoxygenase pseudogene
M_154	22889	KIAA0907(KIAA0907)
M_154	401251	suppressor APC domain containing 1(SAPCD1)
M_154	730092	RRN3 homolog, RNA polymerase I transcription factor pseudogene 1(RRN3P1)
M_154	64770	coiled-coil domain containing 14(CCDC14)
M_154	85028	small nucleolar RNA host gene 12(SNHG12)
M_154	4439	mutS homolog 5(MSH5)
M_154	148898	ZNF436 antisense RNA 1(ZNF436-AS1)
M_154	23642	small nucleolar RNA host gene 1(SNHG1)
M_154	11223	macrophage stimulating 1 like(MST1L)
M_154	389791	PTGES2 antisense RNA 1 (head to head)(PTGES2-
M_154	751867	#N/A
M_154	8876	vanin 1(VNN1)
M_154	751071	methyltransferase like 12(METTL12)
M_154	378805	long intergenic non-protein coding RNA, p53 induced transcript(LINC-PINT)
M_154	51703	acyl-CoA synthetase long-chain family member
M_154	55876	gasdermin B(GSDMB)
M_154	724102	small nucleolar RNA host gene 4(SNHG4)
M_154	440944	THUMPD3 antisense RNA 1(THUMPD3-AS1)
M_154	155400	NOP2/Sun RNA methyltransferase family member 5 pseudogene 1(NSUN5P1)
M_154	100287569	long intergenic non-protein coding RNA

M_154	57035	arginine and serine rich protein 1(RSRP1)
M_154	8420	small nucleolar RNA host gene 3(SNHG3)
M_154	10158	PDZK1 interacting protein 1(PDZK1IP1)
M_154	442582	stromal antigen 3-like 2 (pseudogene)(STAG3L2)
M_154	641977	septin 7 pseudogene 2(SEPT7P2)
M_154	7919	DExD-box helicase 39B(DDX39B)
M_154	54103	gamma-secretase activating protein(GSAP)
M_154	729799	SEC14 like 1 pseudogene 1(SEC14L1P1)
M_154	441108	chromosome 5 open reading frame 56(C5orf56)
M_154	414777	HLA complex group 18 (non-protein coding)(HCG18)
M_154	80864	EGF like domain multiple 8(EGFL8)
M_154	7629	zinc finger protein 76(ZNF76)
M_154	9988	cyclin D binding myb like transcription factor
M_154	11209	macrophage stimulating 1 pseudogene 2(MST1P2)
M_154	285989	zinc finger protein 789(ZNF789)
M_154	2166	fatty acid amide hydrolase(FAAH)
M_154	59271	eva-1 homolog C(EVA1C)
M_154	3139	major histocompatibility complex, class I, L (pseudogene)(HLA-L)
M_154	84129	acyl-CoA dehydrogenase family member
M_154	10628	thioredoxin interacting protein(TXNIP)
M_154	80737	von Willebrand factor A domain containing 7(VWA7)
M_154	2330	flavin containing monooxygenase 5(FMO5)
M_154	1797	decapping exoribonuclease(DXO)
M_154	3934	lipocalin 2(LCN2)
M_154	116844	leucine rich alpha-2-glycoprotein 1(LRG1)
M_154	1195	CDC like kinase 1(CLK1)
M_154	91947	arrestin domain containing 4(ARRDC4)
M_155	431705	astacin like metalloendopeptidase(ASTL)
M_155	205147	APC membrane recruitment protein 3(AMER3)
M_155	283869	neuropeptide W(NPW)
M_155	415056	CNTFR antisense RNA 1(CNTFR-AS1)

M_155	115861	nucleoredoxin-like 1(NXNL1)
M_155	84968	paraneoplastic Ma antigen family member
M_155	2670	glial fibrillary acidic protein(GFAP)
M_155	4624	myosin heavy chain 6(MYH6)
M_155	151	adrenoceptor alpha 2B(ADRA2B)
M_155	284402	secretoglobin family 2B member 2(SCGB2B2)
M_155	27151	C3 and PZP like, alpha-2-macroglobulin domain containing 8(CPAMD8)
M_155	283248	REST corepressor 2(RCOR2)
M_155	4232	mesoderm specific transcript(MEST)
M_155	23769	fibronectin leucine rich transmembrane protein
M_155	2901	glutamate ionotropic receptor kainate type subunit
M_155	1358	carboxypeptidase A2(CPA2)
M_155	1357	carboxypeptidase A1(CPA1)
M_155	8869	ST3 beta-galactoside alpha-2,3-sialyltransferase
M_155	770	carbonic anhydrase 11(CA11)
M_155	148641	solute carrier family 35 member F3(SLC35F3)
M_155	80125	coiled-coil domain containing 33(CCDC33)
M_155	3590	interleukin 11 receptor subunit alpha(IL11RA)
M_155	79865	triggering receptor expressed on myeloid cells like 2(TREML2)
M_155	5348	FXYD domain containing ion transport regulator
M_155	342865	V-set and transmembrane domain containing
M_155	29944	paraneoplastic Ma antigen 3(PNMA3)
M_155	6662	SRY-box 9(SOX9)
M_155	547	kinesin family member 1A(KIF1A)
M_155	183	angiotensinogen(AGT)
M_155	89792	galactose-3-O-sulfotransferase 3(GAL3ST3)
M_155	284358	MEF2 activating motif and SAP domain containing transcriptional regulator(MAMSTR)
M_155	4625	myosin heavy chain 7(MYH7)
M_155	64220	stimulated by retinoic acid 6(STRA6)

M_155	9788	MTSS1, I-BAR domain containing(MTSS1)
M_155	1271	ciliary neurotrophic factor receptor(CNTFR)
M_155	84417	chromosome 2 open reading frame 40(C2orf40)
M_155	84101	ubiquitin specific peptidase 44(USP44)
M_155	57165	gap junction protein gamma 2(GJC2)
M_155	2523	fucosyltransferase 1 (H blood group)(FUT1)
M_155	1144	cholinergic receptor nicotinic delta subunit(CHRND)
M_155	126393	heat shock protein family B (small) member
M_155	643719	secretoglobin family 1B member 2,
M_155	57593	early B-cell factor 4(EBF4)
M_155	157381	long intergenic non-protein coding RNA
M_155	284434	NACHT and WD repeat domain containing 1(NWD1)
M_155	114900	C1q and tumor necrosis factor related protein
M_155	283078	mohawk homeobox(MKX)
M_155	93979	carboxypeptidase A5(CPA5)
M_156	440712	chromosome 1 open reading frame 186(C1orf186)
M_156	23550	pleckstrin and Sec7 domain containing 4(PSD4)
M_156	57630	SH3 domain containing ring finger 1(SH3RF1)
M_156	29984	ras homolog family member D(RHOD)
M_156	285375	long intergenic non-protein coding RNA
M_156	57611	immunoglobulin superfamily containing leucine rich repeat 2(ISLR2)
M_156	4739	neural precursor cell expressed, developmentally down-regulated 9(NEDD9)
M_156	8492	protease, serine 12(PRSS12)
M_156	56999	ADAM metallopeptidase with thrombospondin type 1 motif 9(ADAMTS9)
M_156	23138	NEDD4 binding protein 3(N4BP3)
M_156	196475	rhabdomyosarcoma 2 associated transcript (non-protein coding)(RMST)
M_156	9762	leucine zipper tumor suppressor family member
M_156	57565	kelch like family member 14(KLHL14)

M_156	5152	phosphodiesterase 9A(PDE9A)
M_156	84929	fibrinogen C domain containing 1(FIBCD1)
M_156	8437	RAS protein activator like 1(RASAL1)
M_156	9569	GTF2I repeat domain containing 1(GTF2IRD1)
M_156	6659	SRY-box 4(SOX4)
M_156	57214	cell migration inducing hyaluronan binding
M_156	169966	family with sequence similarity 46 member
M_156	2346	folate hydrolase 1(FOLH1)
M_156	79940	long intergenic non-protein coding RNA
M_156	92737	delta/notch like EGF repeat containing(DNER)
M_156	283731	uncharacterized LOC283731(LOC283731)
M_156	7476	Wnt family member 7A(WNT7A)
M_156	4188	MyoD family inhibitor(MDFI)
M_156	26064	retinoic acid induced 14(RAI14)
M_156	658	bone morphogenetic protein receptor type
M_156	23321	tripartite motif containing 2(TRIM2)
M_156	9615	guanine deaminase(GDA)
M_156	162466	phosphoethanolamine/phosphocholine phosphatase(PHOSPHO1)
M_156	84570	collagen type XXV alpha 1 chain(COL25A1)
M_156	285671	ring finger protein 180(RNF180)
M_156	139735	ZFP92 zinc finger protein(ZFP92)
M_156	219595	folate hydrolase 1B(FOLH1B)
M_156	10123	ADP ribosylation factor like GTPase 4C(ARL4C)
M_156	7134	troponin C1, slow skeletal and cardiac type(TNNC1)
M_156	7849	paired box 8(PAX8)
M_156	10446	leucine rich repeat neuronal 2(LRRN2)
M_156	255426	RasGEF domain family member 1C(RASGEF1C)
M_156	377007	kelch like family member 30(KLHL30)
M_156	8817	fibroblast growth factor 18(FGF18)
M_156	286343	leucine rich adaptor protein 1 like(LURAP1L)
M_156	7379	uroplakin 2(UPK2)

M_156	4222	mesenchyme homeobox 1(MEOX1)
M_156	284836	long intergenic non-protein coding RNA
M_156	84978	FERM domain containing 5(FRMD5)
M_156	57113	transient receptor potential cation channel subfamily C member 7(TRPC7)
M_156	653645	TBC1D3P1-DHX40P1 readthrough, transcribed pseudogene(TBC1D3P1-DHX40P1)
M_156	553	arginine vasopressin receptor 1B(AVPR1B)
M_156	93145	olfactomedin 2(OLFM2)
M_156	80725	SRC kinase signaling inhibitor 1(SRCIN1)
M_156	387695	chromosome 10 open reading frame 99(C10orf99)
M_156	4897	neuronal cell adhesion molecule(NRCAM)
M_156	131566	discoidin, CUB and LCCL domain containing
M_156	153579	butyrophilin like 9(BTNL9)
M_156	285659	olfactory receptor family 2 subfamily V member
M_157	3993	LLGL2, scribble cell polarity complex
M_157	146223	CKLF like MARVEL transmembrane domain containing 4(CMTM4)
M_157	147650	sperm acrosome associated 6(SPACA6)
M_157	9620	cadherin EGF LAG seven-pass G-type receptor
M_157	94025	mucin 16, cell surface associated(MUC16)
M_157	24139	echinoderm microtubule associated protein like
M_157	55150	chromosome 19 open reading frame 73(C19orf73)
M_157	6725	src-related kinase lacking C-terminal regulatory tyrosine and N-terminal myristylation sites(SRMS)
M_157	259173	ALS2 C-terminal like(ALS2CL)
M_157	9600	phosphatidylinositol transfer protein membrane associated 1(PITPNM1)
M_157	2125	envoplakin(EVPL)
M_157	55336	F-box and leucine rich repeat protein 8(FBXL8)
M_157	10848	protein phosphatase 1 regulatory subunit 13
M_157	3931	lecithin-cholesterol acyltransferase(LCAT)

M_157	5064	paralemmin(PALM)
M_157	8996	nucleolar protein 3(NOL3)
M_157	7408	vasodilator-stimulated phosphoprotein(VASP)
M_157	6712	spectrin beta, non-erythrocytic 2(SPTBN2)
M_157	115110	uncharacterized LOC115110(LOC115110)
M_157	1013	cadherin 15(CDH15)
M_157	6560	solute carrier family 12 member 4(SLC12A4)
M_157	55357	TBC1 domain family member 2(TBC1D2)
M_157	55561	CDC42 binding protein kinase gamma(CDC42BPG)
M_157	5753	protein tyrosine kinase 6(PTK6)
M_157	29109	formin homology 2 domain containing 1(FHOD1)
M_157	387254	solute carrier family 7 member 5 pseudogene
M_157	653319	KIAA0895 like(KIAA0895L)
M_157	27111	syndecan binding protein 2(SDCBP2)
M_157	80004	epithelial splicing regulatory protein 2(ESRP2)
M_157	8717	TNFRSF1A associated via death domain(TRADD)
M_157	3299	heat shock transcription factor 4(HSF4)
M_157	156	G protein-coupled receptor kinase 2(GRK2)
M_157	4486	macrophage stimulating 1 receptor(MST1R)
M_157	79567	family with sequence similarity 65 member
M_157	339105	protease, serine 53(PRSS53)
M_157	84634	KISS1 receptor(KISS1R)
M_157	2875	glutamic--pyruvic transaminase(GPT)
M_157	94059	leukocyte receptor cluster member 9(LENG9)
M_157	338692	ankyrin repeat domain 13D(ANKRD13D)
M_157	6645	syntrophin beta 2(SNTB2)
M_157	79784	myosin heavy chain 14(MYH14)
M_157	729440	coiled-coil domain containing 61(CCDC61)
M_157	202915	transmembrane protein 184A(TMEM184A)
M_157	8541	PTPRF interacting protein alpha 3(PPFIA3)
M_157	126353	mitotic spindle positioning(MISP)
M_157	3691	integrin subunit beta 4(ITGB4)

M_157	8824	carboxylesterase 2(CES2)
M_157	54795	transient receptor potential cation channel subfamily M member 4(TRPM4)
M_157	11035	receptor interacting serine/threonine kinase 3(RIPK3)
M_157	400931	MIRLET7B host gene(MIRLET7BHG)
M_157	999	cadherin 1(CDH1)
M_157	5143	phosphodiesterase 4C(PDE4C)
M_157	79767	engulfment and cell motility 3(ELMO3)
M_157	151295	solute carrier family 23 member 3(SLC23A3)
M_157	57530	cingulin(CGN)
M_157	8764	TNF receptor superfamily member 14(TNFRSF14)
M_157	4041	LDL receptor related protein 5(LRP5)
M_157	84752	UDP-GlcNAc:betaGal beta-1,3-N-acetylglucosaminyltransferase 9(B3GNT9)
M_157	9564	BCAR1, Cas family scaffolding protein(BCAR1)
M_157	1374	carnitine palmitoyltransferase 1A(CPT1A)
M_157	2524	fucosyltransferase 2(FUT2)
M_157	27106	arrestin domain containing 2(ARRDC2)
M_157	5883	RAD9 checkpoint clamp component A(RAD9A)
M_157	54961	slingshot protein phosphatase 3(SSH3)
M_157	647024	chromosome 6 open reading frame 132(C6orf132)
M_157	200634	keratinocyte associated protein 3(KRTCAP3)
M_157	6553	solute carrier family 9 member A5(SLC9A5)
M_157	92154	MTSS1L, I-BAR domain containing(MTSS1L)
M_157	5657	proteinase 3(PRTN3)
M_157	100294720	neuroblastoma highly expressed 1(NHEG1)
M_157	5029	purinergic receptor P2Y2(P2RY2)
M_157	100130987	uncharacterized LOC100130987(LOC100130987)
M_158	5376	peripheral myelin protein 22(PMP22)
M_158	2252	fibroblast growth factor 7(FGF7)
M_158	27286	sushi repeat containing protein, X-linked 2(SRPX2)
M_158	27129	heat shock protein family B (small) member

M_158	91663	myeloid associated differentiation marker(MYADM)
M_158	389558	family with sequence similarity 180 member
M_158	389136	vestigial like family member 3(VGLL3)
M_158	2669	GTP binding protein overexpressed in skeletal
M_158	284119	polymerase I and transcript release factor(PTRF)
M_158	23022	palladin, cytoskeletal associated protein(PALLD)
M_158	51226	coatomer protein complex subunit zeta 2(COPZ2)
M_158	51339	dishevelled binding antagonist of beta catenin
M_158	7168	tropomyosin 1 (alpha)(TPM1)
M_158	7169	tropomyosin 2 (beta)(TPM2)
M_158	9843	hephaestin(HEPH)
M_158	127435	podocan(PODN)
M_158	1009	cadherin 11(CDH11)
M_158	80114	BicC family RNA binding protein 1(BICC1)
M_158	3487	insulin like growth factor binding protein 4(IGFBP4)
M_158	56917	Meis homeobox 3(MEIS3)
M_158	800	caldesmon 1(CALD1)
M_158	27295	PDZ and LIM domain 3(PDLIM3)
M_158	2134	exostosin like glycosyltransferase 1(EXTL1)
M_158	79827	CXADR like membrane protein(CLMP)
M_158	57158	junctophilin 2(JPH2)
M_158	84561	solute carrier family 12 member 8(SLC12A8)
M_158	7041	transforming growth factor beta 1 induced transcript 1(TGFB1I1)
M_158	219654	zinc finger CCHC-type containing 24(ZCCHC24)
M_158	114801	transmembrane protein 200A(TMEM200A)
M_158	7077	TIMP metallopeptidase inhibitor 2(TIMP2)
M_158	5999	regulator of G-protein signaling 4(RGS4)
M_158	7078	TIMP metallopeptidase inhibitor 3(TIMP3)
M_158	337876	chondroitin sulfate synthase 3(CHSY3)
M_158	84168	anthrax toxin receptor 1(ANTXR1)
M_158	5740	prostaglandin I2 synthase(PTGIS)

M_158	1307	collagen type XVI alpha 1 chain(COL16A1)
M_158	148113	cartilage intermediate layer protein 2(CILP2)
M_158	151887	coiled-coil domain containing 80(CCDC80)
M_158	25802	leiomodin 1(LMOD1)
M_158	6424	secreted frizzled related protein 4(SFRP4)
M_158	253827	methionine sulfoxide reductase B3(MSRB3)
M_158	4323	matrix metallopeptidase 14(MMP14)
M_158	2825	G protein-coupled receptor 1(GPR1)
M_158	84624	fibronectin type III domain containing 1(FNDC1)
M_158	6876	transgelin(TAGLN)
M_158	72	actin, gamma 2, smooth muscle, enteric(ACTG2)
M_158	59	actin, alpha 2, smooth muscle, aorta(ACTA2)
M_158	5156	platelet derived growth factor receptor
M_158	1264	calponin 1(CNN1)
M_158	79987	sushi, von Willebrand factor type A, EGF and pentraxin domain containing 1(SVEP1)
M_158	147906	dishevelled binding antagonist of beta catenin
M_158	10395	DLC1 Rho GTPase activating protein(DLC1)
M_158	10398	myosin light chain 9(MYL9)
M_158	11259	filamin A interacting protein 1 like(FILIP1L)
M_158	83468	glycosyltransferase 8 domain containing 2(GLT8D2)
M_158	5054	serpin family E member 1(SERPINE1)
M_158	8840	WNT1 inducible signaling pathway protein 1(WISP1)
M_158	284297	scavenger receptor cysteine rich family member with 5 domains(SSC5D)
M_158	114897	C1q and tumor necrosis factor related protein
M_158	5924	Ras protein specific guanine nucleotide releasing factor 2(RASGRF2)
M_158	10335	murine retrovirus integration site 1 homolog(MRVI1)
M_158	57493	heart development protein with EGF like domains
M_158	5118	procollagen C-endopeptidase enhancer(PCOLCE)
M_158	4681	neuroblastoma 1, DAN family BMP

M_158	633	biglycan(BGN)
M_158	2115	ETS variant 1(ETV1)
M_158	7057	thrombospondin 1(THBS1)
M_158	2246	fibroblast growth factor 1(FGF1)
M_158	340075	arylsulfatase family member I(ARSI)
M_158	220	aldehyde dehydrogenase 1 family member
M_158	9945	glutamine-fructose-6-phosphate transaminase
M_158	10630	podoplanin(PDPN)
M_158	116535	MAS related GPR family member F(MRGPRF)
M_158	8630	hydroxysteroid 17-beta dehydrogenase 6(HSD17B6)
M_158	56978	PR/SET domain 8(PRDM8)
M_158	5350	phospholamban(PLN)
M_158	387914	shisa family member 2(SHISA2)
M_158	3671	immunoglobulin superfamily containing leucine rich repeat(ISLR)
M_158	90993	cAMP responsive element binding protein 3 like
M_158	639	PR/SET domain 1(PRDM1)
M_158	1311	cartilage oligomeric matrix protein(COMP)
M_158	57333	reticulocalbin 3(RCN3)
M_159	5596	mitogen-activated protein kinase 4(MAPK4)
M_159	1021	cyclin dependent kinase 6(CDK6)
M_159	204010	ribosomal protein SA pseudogene 52(RPSAP52)
M_159	23552	cyclin dependent kinase 20(CDK20)
M_159	26030	pleckstrin homology and RhoGEF domain containing G3(PLEKHG3)
M_159	8091	high mobility group AT-hook 2(HMGA2)
M_159	283659	protogenin(PRTG)
M_159	112885	PHD finger protein 21B(PHF21B)
M_159	10278	embryonal Fyn-associated substrate(EFS)
M_159	81031	solute carrier family 2 member 10(SLC2A10)
M_159	2735	GLI family zinc finger 1(GLI1)
M_159	2736	GLI family zinc finger 2(GLI2)

M_159	55335	nipsnap homolog 3B(NIPSNAP3B)
M_159	9486	carbohydrate sulfotransferase 10(CHST10)
M_159	79608	RIC3 acetylcholine receptor chaperone(RIC3)
M_159	23677	SH3 domain binding protein 4(SH3BP4)
M_159	57653	LOC100499484-C9orf174 readthrough(LOC100499484-C9ORF174)
M_159	26108	pygopus family PHD finger 1(PYGO1)
M_159	79781	IQ motif containing with AAA domain 1(IQCA1)
M_159	646600	IGF2BP2 antisense RNA 1(IGF2BP2-AS1)
M_159	5727	patched 1(PTCH1)
M_159	1400	collapsin response mediator protein 1(CRMP1)
M_159	7275	tubby bipartite transcription factor(TUB)
M_159	84962	ajuba LIM protein(AJUBA)
M_159	3667	insulin receptor substrate 1(IRS1)
M_159	55084	sine oculis binding protein homolog(SOBP)
M_159	51310	solute carrier family 22 member 17(SLC22A17)
M_159	144165	prickle planar cell polarity protein 1(PRICKLE1)
M_159	169841	zinc finger protein 169(ZNF169)
M_159	285175	unc-80 homolog, NALCN activator(UNC80)
M_159	54993	zinc finger and SCAN domain containing 2(ZSCAN2)
M_159	9915	aryl hydrocarbon receptor nuclear translocator
M_159	6710	spectrin beta, erythrocytic(SPTB)
M_159	10644	insulin like growth factor 2 mRNA binding protein
M_159	25805	BMP and activin membrane bound inhibitor(BAMBI)
M_159	286367	#N/A
M_159	3480	insulin like growth factor 1 receptor(IGF1R)
M_159	595	cyclin D1(CCND1)
M_159	3746	potassium voltage-gated channel subfamily C member 1(KCNC1)
M_159	83856	fibronectin type III and SPRY domain containing 1 like(FSD1L)

M_159	30820	potassium voltage-gated channel interacting protein 1(KCNIP1)
M_159	596	BCL2, apoptosis regulator(BCL2)
M_159	758	metallophosphoesterase domain containing
M_159	55214	prolyl 3-hydroxylase 2(P3H2)
M_159	6331	sodium voltage-gated channel alpha subunit
M_160	1238	atypical chemokine receptor 2(ACKR2)
M_160	7434	vasoactive intestinal peptide receptor 2(VIPR2)
M_160	192683	secretory carrier membrane protein 5(SCAMP5)
M_160	116179	transglutaminase 7(TGM7)
M_160	7780	solute carrier family 30 member 2(SLC30A2)
M_160	1582	cytochrome P450 family 8 subfamily B member
M_160	259217	heat shock protein family A (Hsp70) member 12A(HSPA12A)
M_160	84850	GLIS3 antisense RNA 1(GLIS3-AS1)
M_160	79836	LON peptidase N-terminal domain and ring finger 3(LONRF3)
M_160	284749	long intergenic non-protein coding RNA
M_160	220213	OTU deubiquitinase 1(OTUD1)
M_160	1258	cyclic nucleotide gated channel beta 1(CNGB1)
M_160	53345	transmembrane 6 superfamily member 2(TM6SF2)
M_160	8324	frizzled class receptor 7(FZD7)
M_160	53828	FXYD domain containing ion transport regulator
M_160	729993	shisa family member 9(SHISA9)
M_160	83543	allograft inflammatory factor 1 like(AIF1L)
M_160	4101	MAGE family member A2(MAGEA2)
M_160	6853	synapsin I(SYN1)
M_160	9362	copine 6(CPNE6)
M_160	55753	oxoglutarate dehydrogenase-like(OGDHL)
M_160	8838	WNT1 inducible signaling pathway protein 3(WISP3)
M_160	154822	long intergenic non-protein coding RNA

M_160	27092	calcium voltage-gated channel auxiliary subunit gamma 4(CACNG4)
M_160	22859	adhesion G protein-coupled receptor L1(ADGRL1)
M_160	84883	apoptosis inducing factor, mitochondria associated
M_160	63876	PBX/knotted 1 homeobox 2(PKNOX2)
M_160	256815	chromosome 10 open reading frame 67(C10orf67)
M_160	374739	testis, prostate and placenta expressed(TEPP)
M_160	23432	G protein-coupled receptor 161(GPR161)
M_160	27239	G protein-coupled receptor 162(GPR162)
M_160	57167	spalt like transcription factor 4(SALL4)
M_160	10079	ATPase phospholipid transporting 9A
M_160	221687	ring finger protein 182(RNF182)
M_160	9148	neuralized E3 ubiquitin protein ligase 1(NEURL1)
M_160	27160	inhibitor of growth family, X-linked
M_160	10382	tubulin beta 4A class IVa(TUBB4A)
M_160	10529	nebulette(NEBL)
M_160	80303	EF-hand domain family member D1(EFHD1)
M_160	135892	tripartite motif containing 50(TRIM50)
M_160	84909	chromosome 9 open reading frame 3(C9orf3)
M_160	64106	neuropeptide FF receptor 1(NPFFR1)
M_160	257000	tissue differentiation-inducing non-protein coding RNA(TINCR)
M_160	64359	nucleoredoxin(NXN)
M_160	79981	FERM domain containing 1(FRMD1)
M_160	83643	coiled-coil domain containing 3(CCDC3)
M_160	7173	thyroid peroxidase(TPO)
M_160	786	calcium voltage-gated channel auxiliary subunit gamma 1(CACNG1)
M_160	2159	coagulation factor X(F10)
M_160	6751	somatostatin receptor 1(SSTR1)
M_160	9283	G protein-coupled receptor 37 like 1(GPR37L1)
M_160	92293	transmembrane protein 132C(TMEM132C)

M_160	163071	zinc finger protein 114(ZNF114)
M_160	57491	aryl-hydrocarbon receptor repressor(AHRR)
M_160	54466	spindlin family member 2A(SPIN2A)
M_160	84691	family with sequence similarity 71 member
M_160	23251	KIAA1024(KIAA1024)
M_160	57538	alpha kinase 3(ALPK3)
M_160	2925	gastrin releasing peptide receptor(GRPR)
M_160	2568	gamma-aminobutyric acid type A receptor pi
M_160	112937	galactosidase beta 1 like 3(GLB1L3)
M_160	80036	transient receptor potential cation channel subfamily M member 3(TRPM3)
M_160	26038	chromodomain helicase DNA binding protein
M_160	59272	angiotensin I converting enzyme 2(ACE2)
M_160	55698	Rap associating with DIL domain(RADIL)
M_160	1415	crystallin beta B2(CRYBB2)
M_160	56301	solute carrier family 7 member 10(SLC7A10)
M_160	150135	long intergenic non-protein coding RNA
M_160	285848	patatin like phospholipase domain containing
M_160	285593	uncharacterized LOC285593(LOC285593)
M_160	10602	CDC42 effector protein 3(CDC42EP3)
M_160	54979	HRAS like suppressor 2(HRASLS2)
M_160	130271	pleckstrin homology, MyTH4 and FERM domain containing H2(PLEKHH2)
M_161	284185	long intergenic non-protein coding RNA
M_161	284186	transmembrane protein 105(TMEM105)
M_161	6344	secretin receptor(SCTR)
M_161	340024	solute carrier family 6 member 19(SLC6A19)
M_161	653	bone morphogenetic protein 5(BMP5)
M_161	189	alanine-glyoxylate aminotransferase(AGXT)
M_161	54363	hydroxyacid oxidase 1(HAO1)
M_161	258	ameloblastin(AMBN)
M_161	221241	long intergenic non-protein coding RNA

M_161	341276	olfactory receptor family 10 subfamily A member
M_161	51213	leucine zipper protein 4(LUZP4)
M_161	284382	actin like 9(ACTL9)
M_161	402117	von Willebrand factor C domain containing protein 2-like(VWC2L)
M_161	4632	myosin light chain 1(MYL1)
M_161	50805	iroquois homeobox 4(IRX4)
M_161	100132565	golgin A8 family member F(GOLGA8F)
M_161	389396	glycine-N-acyltransferase like 3(GLYATL3)
M_161	64211	LIM homeobox 5(LHX5)
M_161	3950	leukocyte cell derived chemotaxin 2(LECT2)
M_161	147183	keratin 25(KRT25)
M_161	732275	long intergenic non-protein coding RNA
M_161	1392	corticotropin releasing hormone(CRH)
M_161	140807	keratin 72(KRT72)
M_161	285194	tumor suppressor candidate 7 (non-protein
M_161	170961	ankyrin repeat domain 24(ANKRD24)
M_161	347169	olfactory receptor family 1 subfamily B member 1 (gene/pseudogene)(OR1B1)
M_161	120776	olfactory receptor family 2 subfamily D member
M_161	338755	olfactory receptor family 2 subfamily AG member 2(OR2AG2)
M_161	3195	T-cell leukemia homeobox 1(TLX1)
M_161	157506	retinol dehydrogenase 10 (all-trans)(RDH10)
M_161	390058	olfactory receptor family 51 subfamily B member
M_161	100132386	keratin associated protein 4-9(KRTAP4-9)
M_161	10842	protein phosphatase 1 regulatory subunit
M_161	122046	testis expressed 26(TEX26)
M_161	125931	carcinoembryonic antigen related cell adhesion molecule 20(CEACAM20)
M_161	338567	potassium two pore domain channel subfamily K member 18(KCNK18)

M_161	84066	testis expressed 35(TEX35)
M_161	6755	somatostatin receptor 5(SSTR5)
M_161	81282	olfactory receptor family 51 subfamily G member
M_161	100038246	TLX1 neighbor(TLX1NB)
M_161	126123	IZUMO family member 2(IZUMO2)
M_161	57369	gap junction protein delta 2(GJD2)
M_161	1137	cholinergic receptor nicotinic alpha 4
M_161	390992	hes family bHLH transcription factor 3(HES3)
M_161	57642	collagen type XX alpha 1 chain(COL20A1)
M_161	337882	keratin associated protein 19-1(KRTAP19-1)
M_161	348932	solute carrier family 6 member 18(SLC6A18)
M_161	144124	olfactory receptor family 10 subfamily A member
M_161	285735	long intergenic non-protein coding RNA
M_161	91464	intestine specific homeobox(ISX)
M_161	339291	leucine rich repeat containing 30(LRRC30)
M_161	283297	olfactory receptor family 10 subfamily A member
M_161	10660	ladybird homeobox 1(LBX1)
M_161	389158	phospholipid scramblase family member 5(PLSCR5)
M_161	541465	cancer/testis antigen family 45 member A6(CT45A6)
M_161	63946	DMRT like family C2(DMRTC2)
M_161	646486	fatty acid binding protein 12(FABP12)
M_161	347745	Prader Willi/Angelman region RNA 4(PWAR4)
M_161	146336	SSTR5 antisense RNA 1(SSTR5-AS1)
M_161	150681	olfactory receptor family 6 subfamily B member
M_161	171482	family with sequence similarity 9 member A(FAM9A)
M_161	219870	olfactory receptor family 10 subfamily G member
M_161	8403	SRY-box 14(SOX14)
M_161	6550	solute carrier family 9 member A3(SLC9A3)
M_161	100033802	small nucleolar RNA, C/D box 115-26(SNORD115-
M_161	80820	endonuclease/exonuclease/phosphatase family domain containing 1(EEPD1)
M_161	150572	SET and MYND domain containing 1(SMYD1)

M_161	26223	F-box and leucine rich repeat protein 21 (gene/pseudogene)(FBXL21)
M_161	55079	FEZ family zinc finger 2(FEZF2)
M_161	1412	crystallin beta A2(CRYBA2)
M_161	5288	phosphatidylinositol-4-phosphate 3-kinase catalytic subunit type 2 gamma(PIK3C2G)
M_161	266697	POM121 transmembrane nucleoporin like 4, pseudogene(POM121L4P)
M_161	29797	POM121 transmembrane nucleoporin like 8, pseudogene(POM121L8P)
M_161	222546	regulatory factor X6(RFX6)
M_161	63947	DMRT like family C1(DMRTC1)
M_161	85413	solute carrier family 22 member 16(SLC22A16)
M_161	93661	capping actin protein of muscle Z-line alpha subunit 3(CAPZA3)
M_161	3044	hemoglobin subunit beta pseudogene 1(HBBP1)
M_161	51297	BPI fold containing family A member 1(BPIFA1)
M_161	3849	keratin 2(KRT2)
M_161	55711	fatty acyl-CoA reductase 2(FAR2)
M_161	4951	oncomodulin 2(OCM2)
M_161	8590	olfactory receptor family 6 subfamily A member
M_161	60506	nyctalopin(NYX)
M_161	727851	RANBP2-like and GRIP domain containing
M_161	6506	solute carrier family 1 member 2(SLC1A2)
M_161	728656	DMRT like family C1B(DMRTC1B)
M_161	222545	G protein-coupled receptor class C group 6 member A(GPRC6A)
M_161	6534	solute carrier family 6 member 7(SLC6A7)
M_161	1369	carboxypeptidase N subunit 1(CPN1)
M_161	341116	membrane spanning 4-domains A10(MS4A10)
M_161	6547	solute carrier family 8 member A3(SLC8A3)
M_161	389715	transmembrane protein C9orf144B

M_161	735	complement C9(C9)
M_161	3595	interleukin 12 receptor subunit beta 2(IL12RB2)
M_161	83795	potassium two pore domain channel subfamily K member 16(KCNK16)
M_161	4956	outer dense fiber of sperm tails 1(ODF1)
M_161	5535	protein phosphatase 3 regulatory subunit B,
M_161	94235	G protein subunit gamma 8(GNG8)
M_161	25774	glutathione S-transferase theta pseudogene
M_161	642968	family with sequence similarity 163 member
M_161	462	serpin family C member 1(SERPINC1)
M_161	256369	long intergenic non-protein coding RNA
M_161	89822	potassium two pore domain channel subfamily K member 17(KCNK17)
M_161	259308	family with sequence similarity 205 member
M_161	146713	RNA binding protein, fox-1 homolog 3(RBFOX3)
M_161	170392	oncoprotein induced transcript 3(OIT3)
M_161	51179	hydroxyacid oxidase 2(HAO2)
M_161	266629	SEC14 like lipid binding 3(SEC14L3)
M_161	9376	solute carrier family 22 member 8(SLC22A8)
M_161	646799	zygote arrest 1-like(ZAR1L)
M_162	203859	anoctamin 5(ANO5)
M_162	3673	integrin subunit alpha 2(ITGA2)
M_162	266812	nucleosome assembly protein 1 like 5(NAP1L5)
M_162	5602	mitogen-activated protein kinase 10(MAPK10)
M_162	157753	transmembrane protein 74(TMEM74)
M_162	22837	cordon-bleu WH2 repeat protein like 1(COBLL1)
M_162	57604	KIAA1456(KIAA1456)
M_162	9185	RALBP1 associated Eps domain containing 2(REPS2)
M_162	5783	protein tyrosine phosphatase, non-receptor type
M_162	143425	synaptotagmin 9(SYT9)
M_162	169200	transmembrane protein 64(TMEM64)
M_162	388714	flavin containing monooxygenase 6

M_162	2888	growth factor receptor bound protein 14(GRB14)
M_162	338376	interferon epsilon(IFNE)
M_162	79642	arylsulfatase family member J(ARSJ)
M_162	100130264	uncharacterized LOC100130264(LOC100130264)
M_162	55784	multiple C2 and transmembrane domain containing
M_162	63951	DMRT like family A1(DMRTA1)
M_162	444	aspartate beta-hydroxylase(ASPH)
M_162	80111	chromosome 3 open reading frame 36(C3orf36)
M_162	378925	ring finger protein 148(RNF148)
M_162	151162	#N/A
M_162	91404	SEC14 and spectrin domain containing 1(SESTD1)
M_162	667	dystonin(DST)
M_162	112574	sorting nexin 18(SNX18)
M_162	54622	ADP ribosylation factor like GTPase 15(ARL15)
M_162	7091	transducin like enhancer of split 4(TLE4)
M_162	6386	syndecan binding protein(SDCBP)
M_162	5099	protocadherin 7(PCDH7)
M_162	5274	serpin family I member 1(SERPINI1)
M_162	51375	sorting nexin 7(SNX7)
M_162	83641	family with sequence similarity 107 member
M_162	93664	calcium dependent secretion activator 2(CADPS2)
M_162	168433	ring finger protein 133(RNF133)
M_162	57419	solute carrier family 24 member 3(SLC24A3)
M_162	4249	mannosyl (alpha-1,6-)-glycoprotein beta-1,6-N-acetyl-glucosaminyltransferase(MGAT5)
M_162	79628	SH3 domain and tetratricopeptide repeats 2(SH3TC2)
M_162	3790	potassium voltage-gated channel modifier subfamily S member 3(KCNS3)
M_162	2911	glutamate metabotropic receptor 1(GRM1)
M_162	54885	TBC1 domain family member 8B(TBC1D8B)
M_162	6578	solute carrier organic anion transporter family member 2A1(SLCO2A1)

M_162	554202	MIR31 host gene(MIR31HG)
M_162	132989	chromosome 4 open reading frame 36(C4orf36)
M_162	81578	collagen type XXI alpha 1 chain(COL21A1)
M_162	11228	Ras association domain family member 8(RASSF8)
M_162	956	ectonucleoside triphosphate diphosphohydrolase
M_162	114134	solute carrier family 2 member 13(SLC2A13)
M_162	7162	trophoblast glycoprotein(TPBG)
M_162	57144	p21 (RAC1) activated kinase 5(PAK5)
M_162	93556	EGF like and EMI domain containing 1, pseudogene(EGFEM1P)
M_162	493869	glutathione peroxidase 8 (putative)(GPX8)
M_162	2195	FAT atypical cadherin 1(FAT1)
M_162	29018	FOXN3 antisense RNA 2(FOXN3-AS2)
M_162	53918	pelota homolog (Drosophila)(PELO)
M_162	5649	reelin(RELN)
M_162	4885	neuronal pentraxin 2(NPTX2)
M_162	26047	contactin associated protein-like 2(CNTNAP2)
M_162	54947	lysophosphatidylcholine acyltransferase 2(LPCAT2)
M_162	53616	ADAM metallopeptidase domain 22(ADAM22)
M_162	4338	molybdenum cofactor synthesis 2(MOCS2)
M_162	100192420	uncharacterized LOC100192420(FLJ41941)
M_162	375567	von Willebrand factor C domain containing 2(VWC2)
M_163	284217	laminin subunit alpha 1(LAMA1)
M_163	130162	clathrin heavy chain linker domain containing
M_163	23211	zinc finger CCCH-type containing 4(ZC3H4)
M_163	23271	calmodulin regulated spectrin associated protein family member 2(CAMSAP2)
M_163	150864	family with sequence similarity 117 member
M_163	59349	kelch like family member 12(KLHL12)
M_163	5451	POU class 2 homeobox 1(POU2F1)
M_163	9139	CBFA2/RUNX1 translocation partner 2(CBFA2T2)
M_163	9968	mediator complex subunit 12(MED12)

M_163	10274	stromal antigen 1(STAG1)
M_163	23164	myosin phosphatase Rho interacting protein(MPRIP)
M_163	6239	ras responsive element binding protein 1(RREB1)
M_163	5929	RB binding protein 5, histone lysine methyltransferase complex subunit(RBBP5)
M_163	8462	Kruppel like factor 11(KLF11)
M_163	8085	lysine methyltransferase 2D(KMT2D)
M_163	23252	OTU deubiquitinase 3(OTUD3)
M_163	65108	MARCKS like 1(MARCKSL1)
M_163	23233	exocyst complex component 6B(EXOC6B)
M_163	57337	SUMO1/sentrin specific peptidase 7(SENP7)
M_163	64710	nuclear casein kinase and cyclin dependent kinase substrate 1(NUCKS1)
M_163	7072	TIA1 cytotoxic granule associated RNA binding
M_163	9886	Rho related BTB domain containing 1(RHOBTB1)
M_163	9887	SMG7, nonsense mediated mRNA decay
M_163	10765	lysine demethylase 5B(KDM5B)
M_163	54497	HEAT repeat containing 5B(HEATR5B)
M_163	165829	G protein-coupled receptor 156(GPR156)
M_163	200424	tet methylcytosine dioxygenase 3(TET3)
M_163	790	carbamoyl-phosphate synthetase 2, aspartate transcarbamylase, and dihydroorotase(CAD)
M_163	65243	ZFP69 zinc finger protein B(ZFP69B)
M_163	4035	LDL receptor related protein 1(LRP1)
M_163	3189	heterogeneous nuclear ribonucleoprotein
M_163	90957	DExH-box helicase 57(DHX57)
M_163	51594	neuroblastoma amplified sequence(NBAS)
M_163	23435	TAR DNA binding protein(TARDBP)
M_163	6711	spectrin beta, non-erythrocytic 1(SPTBN1)
M_163	10137	RNA binding motif protein 12(RBM12)
M_163	57448	baculoviral IAP repeat containing 6(BIRC6)
M_163	100132215	uncharacterized LOC100132215(LOC100132215)

M_163	55728	NEDD4 binding protein 2(N4BP2)
M_163	80304	WD repeat and coiled coil containing(WDCP)
M_163	57216	VANGL planar cell polarity protein 2(VANGL2)
M_163	9901	SLIT-ROBO Rho GTPase activating protein
M_163	3720	jumonji and AT-rich interaction domain containing
M_163	9793	cytoskeleton associated protein 5(CKAP5)
M_163	4194	MDM4, p53 regulator(MDM4)
M_163	23301	EH domain binding protein 1(EHBP1)
M_163	23054	nuclear receptor coactivator 6(NCOA6)
M_163	140710	suppressor of glucose, autophagy associated
M_163	1660	DExH-box helicase 9(DHX9)
M_163	25778	dual serine/threonine and tyrosine protein
M_163	79811	SAFB like transcription modulator(SLTM)
M_163	79677	structural maintenance of chromosomes 6(SMC6)
M_163	54454	ATPase family, AAA domain containing
M_163	92312	mex-3 RNA binding family member A(MEX3A)
M_163	27332	zinc finger protein 638(ZNF638)
M_163	348654	GEN1, Holliday junction 5' flap endonuclease(GEN1)
M_163	6654	SOS Ras/Rac guanine nucleotide exchange factor
M_163	6651	SON DNA binding protein(SON)
M_163	23528	zinc finger protein 281(ZNF281)
M_163	1106	chromodomain helicase DNA binding protein
M_163	84919	protein phosphatase 1 regulatory subunit
M_163	9736	ubiquitin specific peptidase 34(USP34)
M_163	5335	phospholipase C gamma 1(PLCG1)
M_163	84181	chromodomain helicase DNA binding protein
M_163	8648	nuclear receptor coactivator 1(NCOA1)
M_163	1108	chromodomain helicase DNA binding protein
M_163	221914	glypican 2(GPC2)
M_163	57539	WD repeat domain 35(WDR35)
M_163	1788	DNA methyltransferase 3 alpha(DNMT3A)
M_163	2005	ELK4, ETS transcription factor(ELK4)

M_163	3915	laminin subunit gamma 1(LAMC1)
M_163	463	zinc finger homeobox 3(ZFHX3)
M_163	50618	intersectin 2(ITSN2)
M_163	11190	centrosomal protein 250(CEP250)
M_163	254428	solute carrier family 41 member 1(SLC41A1)
M_163	254065	bromodomain and WD repeat domain containing
M_163	80312	tet methylcytosine dioxygenase 1(TET1)
M_163	27107	zinc finger and BTB domain containing 11(ZBTB11)
M_163	3339	heparan sulfate proteoglycan 2(HSPG2)
M_163	10236	heterogeneous nuclear ribonucleoprotein
M_163	23369	pumilio RNA binding family member 2(PUM2)
M_163	55252	additional sex combs like 2, transcriptional
M_163	79598	centrosomal protein 97(CEP97)
M_163	9877	zinc finger CCCH-type containing 11A(ZC3H11A)
M_163	7840	ALMS1, centrosome and basal body associated protein(ALMS1)
M_163	23020	small nuclear ribonucleoprotein U5 subunit
M_163	55749	cell division cycle and apoptosis regulator 1(CCAR1)
M_163	221527	zinc finger and BTB domain containing 12(ZBTB12)
M_163	2803	golgin A4(GOLGA4)
M_163	9392	transforming growth factor beta receptor associated protein 1(TGFBRAP1)
M_163	8527	diacylglycerol kinase delta(DGKD)
M_163	9203	zinc finger MYM-type containing 3(ZMYM3)
M_163	57498	kinase D-interacting substrate 220kDa(KIDINS220)
M_163	3054	host cell factor C1(HCFC1)
M_163	4854	notch 3(NOTCH3)
M_163	55705	importin 9(IPO9)
M_163	493	ATPase plasma membrane Ca2+ transporting
M_163	51277	DnaJ heat shock protein family (Hsp40) member C27(DNAJC27)
M_163	7175	translocated promoter region, nuclear basket

M_163	25885	RNA polymerase I subunit A(POLR1A)
M_163	26160	intraflagellar transport 172(IFT172)
M_163	9475	Rho associated coiled-coil containing protein kinase 2(ROCK2)
M_163	5784	protein tyrosine phosphatase, non-receptor type
M_163	56987	BBX, HMG-box containing(BBX)
M_163	8844	kinase suppressor of ras 1(KSR1)
M_163	23522	lysine acetyltransferase 6B(KAT6B)
M_163	10657	KH RNA binding domain containing, signal transduction associated 1(KHDRBS1)
M_163	6421	splicing factor proline and glutamine rich(SFPQ)
M_163	63925	zinc finger protein 335(ZNF335)
M_163	84733	chromobox 2(CBX2)
M_163	401944	low density lipoprotein receptor class A domain containing 2(LDLRAD2)
M_163	55432	YOD1 deubiquitinase(YOD1)
M_163	9584	RNA binding motif protein 39(RBM39)
M_163	89932	papilin, proteoglycan like sulfated
M_163	57644	myosin heavy chain 7B(MYH7B)
M_163	9911	transmembrane and coiled-coil domain family
M_163	388730	transmembrane protein 81(TMEM81)
M_163	650623	BEN domain containing 3 pseudogene 3(BEND3P3)
M_163	57178	zinc finger MIZ-type containing 1(ZMIZ1)
M_163	9902	mannose receptor C type 2(MRC2)
M_163	6426	serine and arginine rich splicing factor 1(SRSF1)
M_163	84614	zinc finger and BTB domain containing 37(ZBTB37)
M_163	221037	jumonji domain containing 1C(JMJD1C)
M_164	284339	transmembrane protein 145(TMEM145)
M_164	23303	kinesin family member 13B(KIF13B)
M_164	79012	CaM kinase like vesicle associated(CAMKV)
M_164	343702	XK related 7(XKR7)
M_164	158135	tubulin tyrosine ligase like 11(TTLL11)

M_164	649	bone morphogenetic protein 1(BMP1)
M_164	55715	docking protein 4(DOK4)
M_164	92922	coiled-coil domain containing 102A(CCDC102A)
M_164	54332	ganglioside induced differentiation associated protein 1(GDAP1)
M_164	55806	hair growth associated(HR)
M_164	10174	sorbin and SH3 domain containing 3(SORBS3)
M_164	57805	cell cycle and apoptosis regulator 2(CCAR2)
M_164	169270	zinc finger protein 596(ZNF596)
M_164	157695	testis development related protein(TDRP)
M_164	80723	solute carrier family 35 member G2(SLC35G2)
M_164	2039	dematin actin binding protein(DMTN)
M_164	10900	RUN domain containing 3A(RUNDC3A)
M_164	10361	nucleophosmin/nucleoplasmin 2(NPM2)
M_164	64760	family with sequence similarity 160 member
M_164	388588	small integral membrane protein 1 (Vel blood
M_164	29106	secretogranin III(SCG3)
M_164	23221	Rho related BTB domain containing 2(RHOBTB2)
M_164	541565	chromosome 8 open reading frame 58(C8orf58)
M_164	123099	delta 4-desaturase, sphingolipid 2(DEGS2)
M_164	64753	coiled-coil domain containing 136(CCDC136)
M_164	130612	transmembrane protein 198(TMEM198)
M_164	50861	stathmin 3(STMN3)
M_164	80094	BIN3 intronic transcript 1(BIN3-IT1)
M_164	64236	PDZ and LIM domain 2(PDLIM2)
M_164	30008	EGF containing fibulin like extracellular matrix protein 2(EFEMP2)
M_164	28984	regulator of cell cycle(RGCC)
M_164	56654	neural proliferation, differentiation and control
M_164	8510	matrix metallopeptidase 23B(MMP23B)
M_164	6855	synaptophysin(SYP)
M_164	81786	tripartite motif containing 7(TRIM7)

M_164	126374	Wilms tumor 1 interacting protein(WTIP)
M_164	80757	transmembrane protein 121(TMEM121)
M_164	90668	capping protein regulator and myosin 1 linker
M_164	4054	latent transforming growth factor beta binding protein 3(LTBP3)
M_164	1141	cholinergic receptor nicotinic beta 2
M_164	954	ectonucleoside triphosphate diphosphohydrolase
M_164	6900	contactin 2(CNTN2)
M_164	1674	desmin(DES)
M_164	78997	ganglioside induced differentiation associated protein 1 like 1(GDAP1L1)
M_164	9796	phytanoyl-CoA 2-hydroxylase interacting
M_164	1394	corticotropin releasing hormone receptor 1(CRHR1)
M_164	339834	coiled-coil domain containing 36(CCDC36)
M_164	55545	msh homeobox 2 pseudogene 1(MSX2P1)
M_164	645974	poly(A) binding protein cytoplasmic 1 like
M_164	140766	ADAM metallopeptidase with thrombospondin type 1 motif 14(ADAMTS14)
M_164	594855	complexin 3(CPLX3)
M_164	140876	family with sequence similarity 65 member
M_164	79748	lectin, mannose binding 1 like(LMAN1L)
M_164	340529	poly(A) binding protein cytoplasmic 1 like
M_165	657	bone morphogenetic protein receptor type
M_165	84896	ATPase family, AAA domain containing 1(ATAD1)
M_165	115948	coiled-coil domain containing 151(CCDC151)
M_165	65975	serine/threonine kinase 33(STK33)
M_165	118980	sideroflexin 2(SFXN2)
M_165	119032	BLOC-1 related complex subunit 7(BORCS7)
M_165	728190	NUTM2A antisense RNA 1(NUTM2A-AS1)
M_165	51076	cutC copper transporter(CUTC)
M_165	27343	DNA polymerase lambda(POLL)
M_165	81894	solute carrier family 25 member 28(SLC25A28)

M_165	339778	chromosome 2 open reading frame 70(C2orf70)
M_165	54839	leucine rich repeat containing 49(LRRC49)
M_165	143279	HECT domain E3 ubiquitin protein ligase
M_165	91409	coiled-coil domain containing 74B(CCDC74B)
M_165	79991	STN1, CST complex subunit(STN1)
M_165	10724	meningioma expressed antigen 5
M_165	9044	B-TFIID TATA-box binding protein associated factor 1(BTAF1)
M_165	60370	arginine vasopressin induced 1(AVPI1)
M_165	1975	eukaryotic translation initiation factor 4B(EIF4B)
M_165	11191	phosphatase and tensin homolog pseudogene
M_165	5728	phosphatase and tensin homolog(PTEN)
M_165	53354	pantothenate kinase 1(PANK1)
M_165	118812	MORN repeat containing 4(MORN4)
M_165	118924	FRA10A associated CGG repeat 1(FRA10AC1)
M_165	10023	frequently rearranged in advanced T-cell lymphomas 1(FRAT1)
M_165	90557	coiled-coil domain containing 74A(CCDC74A)
M_165	100144748	killin, p53-regulated DNA replication
M_165	57821	coiled-coil domain containing 181(CCDC181)
M_165	142913	cofilin 1 pseudogene 1(CFL1P1)
M_165	2805	glutamic-oxaloacetic transaminase 1(GOT1)
M_165	728118	NUT family member 2A(NUTM2A)
M_165	728130	NUT family member 2D(NUTM2D)
M_165	56652	twinkle mtDNA helicase(TWNK)
M_165	56906	THAP domain containing 10(THAP10)
M_165	55328	renalase, FAD dependent amine oxidase(RNLS)
M_165	9562	multiple inositol-polyphosphate phosphatase
M_165	3257	HPS1, biogenesis of lysosomal organelles complex 3 subunit 1(HPS1)
M_165	25875	LETM1 domain containing 1(LETMD1)
M_165	6468	F-box and WD repeat domain containing 4(FBXW4)

M_165	23401	frequently rearranged in advanced T-cell lymphomas 2(FRAT2)
M_165	1938	eukaryotic translation elongation factor 2(EEF2)
M_166	84894	leucine rich repeat and Ig domain containing
M_166	389705	chromosome 4 open reading frame 27 pseudogene(LOC389705)
M_166	9834	family with sequence similarity 30, member
M_166	931	membrane spanning 4-domains A1(MS4A1)
M_166	973	CD79a molecule(CD79A)
M_166	974	CD79b molecule(CD79B)
M_166	5450	POU class 2 associating factor 1(POU2AF1)
M_166	51318	mitochondrial ribosomal protein L35(MRPL35)
M_166	168620	basic helix-loop-helix family member a15(BHLHA15)
M_166	3738	potassium voltage-gated channel subfamily A member 3(KCNA3)
M_166	10989	inner membrane mitochondrial protein(IMMT)
M_166	1269	cannabinoid receptor 2(CNR2)
M_166	23336	synemin(SYNM)
M_166	11040	Pim-2 proto-oncogene, serine/threonine kinase(PIM2)
M_166	3782	potassium calcium-activated channel subfamily N member 3(KCNN3)
M_166	54900	lymphocyte transmembrane adaptor 1(LAX1)
M_166	51237	marginal zone B and B1 cell specific protein(MZB1)
M_166	26112	coiled-coil domain containing 69(CCDC69)
M_166	57699	copine 5(CPNE5)
M_166	608	TNF receptor superfamily member 17(TNFRSF17)
M_166	55037	pentatricopeptide repeat domain 3(PTCD3)
M_166	23495	TNF receptor superfamily member 13B(TNFRSF13B)
M_166	640	BLK proto-oncogene, Src family tyrosine
M_166	10251	sprouty RTK signaling antagonist 3(SPRY3)
M_166	158326	FRAS1 related extracellular matrix 1(FREM1)
M_166	51303	FK506 binding protein 11(FKBP11)

M_166	54855	family with sequence similarity 46 member
M_166	270	adenosine monophosphate deaminase 1(AMPD1)
M_166	5023	purinergic receptor P2X 1(P2RX1)
M_166	96610	BMS1, ribosome biogenesis factor pseudogene
M_166	79368	Fc receptor like 2(FCRL2)
M_166	266727	MAM domain containing glycosylphosphatidylinositol anchor 1(MDGA1)
M_166	5079	paired box 5(PAX5)
M_166	2914	glutamate metabotropic receptor 4(GRM4)
M_166	6509	solute carrier family 1 member 4(SLC1A4)
M_166	9350	cerberus 1, DAN family BMP antagonist(CER1)
M_166	8755	ADAM metallopeptidase domain 6
M_166	83416	Fc receptor like 5(FCRL5)
M_166	55217	trimethyllysine hydroxylase, epsilon(TMLHE)
M_166	64795	required for meiotic nuclear division 5 homolog
M_166	3512	joining chain of multimeric IgA and IgM(JCHAIN)
M_166	91319	derlin 3(DERL3)
M_166	2837	urotensin 2 receptor(UTS2R)
M_166	6363	C-C motif chemokine ligand 19(CCL19)
M_166	339403	relaxin/insulin like family peptide receptor 4(RXFP4)
M_166	285074	anaphase promoting complex subunit 1 pseudogene(LOC285074)
M_166	83881	Mix paired-like homeobox(MIXL1)
M_166	26266	solute carrier family 13 member 4(SLC13A4)
M_167	6428	serine and arginine rich splicing factor 3(SRSF3)
M_167	817	calcium/calmodulin dependent protein kinase II delta(CAMK2D)
M_167	51626	dynein cytoplasmic 2 light intermediate chain
M_167	10090	uronyl 2-sulfotransferase(UST)
M_167	113612	cytochrome P450 family 2 subfamily U member
M_167	805	calmodulin 2(CALM2)
M_167	6432	serine and arginine rich splicing factor 7(SRSF7)

M_167	51057	WD repeat containing planar cell polarity
M_167	6675	UDP-N-acetylglucosamine pyrophosphorylase
M_167	79807	glutathione S-transferase C-terminal domain containing(GSTCD)
M_167	25936	NSL1, MIS12 kinetochore complex
M_167	166379	Bardet-Biedl syndrome 12(BBS12)
M_167	148304	chromosome 1 open reading frame 74(C1orf74)
M_167	6635	small nuclear ribonucleoprotein polypeptide
M_167	5071	parkin RBR E3 ubiquitin protein ligase(PARK2)
M_167	9167	cytochrome c oxidase subunit 7A2 like(COX7A2L)
M_167	401152	chromosome 4 open reading frame 3(C4orf3)
M_167	112942	cilia and flagella associated protein 36(CFAP36)
M_167	200728	transmembrane protein 17(TMEM17)
M_167	64792	intraflagellar transport 22(IFT22)
M_167	252839	transmembrane protein 9(TMEM9)
M_167	494143	ChaC cation transport regulator homolog 2(CHAC2)
M_167	25896	integrator complex subunit 7(INTS7)
M_167	9061	3'-phosphoadenosine 5'-phosphosulfate synthase
M_167	1314	coatomer protein complex subunit alpha(COPA)
M_167	619208	family with sequence similarity 229 member
M_167	4126	mannosidase beta(MANBA)
M_167	92703	transmembrane protein 183A(TMEM183A)
M_167	56898	3-hydroxybutyrate dehydrogenase, type 2(BDH2)
M_167	4190	malate dehydrogenase 1(MDH1)
M_167	5281	phosphatidylinositol glycan anchor biosynthesis class
M_167	135138	PARK2 coregulated(PACRG)
M_167	154091	solute carrier family 2 member 12(SLC2A12)
M_167	22920	kinesin associated protein 3(KIFAP3)
M_167	285796	PACRG antisense RNA 1(PACRG-AS1)
M_167	56902	partner of NOB1 homolog(PNO1)
M_167	10380	3'(2'), 5'-bisphosphate nucleotidase 1(BPNT1)
M_167	10575	chaperonin containing TCP1 subunit 4(CCT4)

M_167	22926	activating transcription factor 6(ATF6)
M_167	29922	NME/NM23 family member 7(NME7)
M_167	9419	CXXC repeat containing interactor of PDZ3
M_167	729967	MORN repeat containing 2(MORN2)
M_167	128387	TatD DNase domain containing 3(TATDN3)
M_167	133022	translocation associated membrane protein 1-like 1(TRAM1L1)
M_167	51430	SUN domain containing ossification factor(SUCO)
M_167	55248	transmembrane protein 206(TMEM206)
M_167	391356	peptidyl-tRNA hydrolase domain containing
M_167	93627	TBC1 domain containing kinase(TBCK)
M_167	9019	myelin protein zero like 1(MPZL1)
M_167	642819	zinc finger protein 487(ZNF487)
M_167	10560	solute carrier family 19 member 2(SLC19A2)
M_167	79819	WD repeat domain 78(WDR78)
M_167	1939	eukaryotic translation initiation factor 2D(EIF2D)
M_167	23385	nicastrin(NCSTN)
M_167	2272	fragile histidine triad(FHIT)
M_167	6427	serine and arginine rich splicing factor 2(SRSF2)
M_167	155368	Williams Beuren syndrome chromosome region 27(WBSCR27)
M_168	51690	LSM7 homolog, U6 small nuclear RNA and mRNA degradation associated(LSM7)
M_168	58515	selenoprotein K(SELENOK)
M_168	51650	mitochondrial ribosomal protein S33(MRPS33)
M_168	84833	up-regulated during skeletal muscle growth 5 homolog (mouse)(USMG5)
M_168	79714	coiled-coil domain containing 51(CCDC51)
M_168	81892	SRA stem-loop interacting RNA binding
M_168	7384	ubiquinol-cytochrome c reductase core protein
M_168	7873	mesencephalic astrocyte derived neurotrophic
M_168	381	ADP ribosylation factor 5(ARF5)

M_168	57001	succinate dehydrogenase complex assembly factor 3(SDHAF3)
M_168	154791	formation of mitochondrial complex V assembly factor 1 homolog(FMC1)
M_168	51024	fission, mitochondrial 1(FIS1)
M_168	55967	NADH:ubiquinone oxidoreductase subunit
M_168	8692	hyaluronoglucosaminidase 2(HYAL2)
M_168	116541	mitochondrial ribosomal protein L54(MRPL54)
M_168	373156	glutathione S-transferase kappa 1(GSTK1)
M_168	28972	signal peptidase complex subunit 1(SPCS1)
M_168	4694	NADH:ubiquinone oxidoreductase subunit
M_168	10975	ubiquinol-cytochrome c reductase, complex III subunit XI(UQCR11)
M_168	11070	transmembrane protein 115(TMEM115)
M_168	29925	GDP-mannose pyrophosphorylase B(GMPPB)
M_168	55009	chromosome 19 open reading frame 24(C19orf24)
M_168	8802	succinate-CoA ligase alpha subunit(SUCLG1)
M_168	518	ATP synthase, H+ transporting, mitochondrial Fo complex subunit C3 (subunit 9)(ATP5G3)
M_168	11277	three prime repair exonuclease 1(TREX1)
M_168	6988	T-cell leukemia translocation altered(TCTA)
M_168	10248	POP7 homolog, ribonuclease P/MRP subunit(POP7)
M_168	440957	small integral membrane protein 4(SMIM4)
M_168	388962	bolA family member 3(BOLA3)
M_168	11334	tumor suppressor candidate 2(TUSC2)
M_168	51079	NADH:ubiquinone oxidoreductase subunit
M_168	6742	single stranded DNA binding protein 1(SSBP1)
M_168	4946	ornithine decarboxylase antizyme 1(OAZ1)
M_168	84279	protease associated domain containing 1(PRADC1)
M_168	29999	fascin actin-bundling protein 3(FSCN3)
M_168	9551	ATP synthase, H+ transporting, mitochondrial Fo complex subunit F2(ATP5J2)

M_168	4708	NADH:ubiquinone oxidoreductase subunit
M_168	1347	cytochrome c oxidase subunit 7A2(COX7A2)
M_168	4707	NADH:ubiquinone oxidoreductase subunit
M_168	402682	UFM1 specific peptidase 1 (inactive)(UFSP1)
M_168	1349	cytochrome c oxidase subunit 7B(COX7B)
M_168	1329	cytochrome c oxidase subunit 5B(COX5B)
M_168	1337	cytochrome c oxidase subunit 6A1(COX6A1)
M_168	26517	translocase of inner mitochondrial membrane
M_168	11068	cytochrome b561 family member D2(CYB561D2)
M_168	9296	ATPase H+ transporting V1 subunit F(ATP6V1F)
M_168	2879	glutathione peroxidase 4(GPX4)
M_168	374291	NADH:ubiquinone oxidoreductase core subunit
M_168	25915	NADH:ubiquinone oxidoreductase complex assembly factor 3(NDUFAF3)
M_168	513	ATP synthase, H+ transporting, mitochondrial F1 complex, delta subunit(ATP5D)
M_168	11344	twinfilin actin binding protein 2(TWF2)
M_168	132160	protein phosphatase, Mg2+/Mn2+ dependent
M_168	51246	shisa family member 5(SHISA5)
M_168	211	5'-aminolevulinate synthase 1(ALAS1)
M_168	65010	solute carrier family 26 member 6(SLC26A6)
M_169	89853	multivesicular body subunit 12B(MVB12B)
M_169	53820	ripply transcriptional repressor 3(RIPPLY3)
M_169	51166	aminoadipate aminotransferase(AADAT)
M_169	200373	cilia and flagella associated protein 221(CFAP221)
M_169	3352	5-hydroxytryptamine receptor 1D(HTR1D)
M_169	10683	delta like canonical Notch ligand 3(DLL3)
M_169	135295	serine and arginine rich splicing factor 12(SRSF12)
M_169	120103	solute carrier family 36 member 4(SLC36A4)
M_169	767811	H2B histone family member X, pseudogene(H2BFXP)
M_169	220359	tigger transposable element derived 3(TIGD3)
M_169	256227	STEAP family member 1B(STEAP1B)

M_169	6564	solute carrier family 15 member 1(SLC15A1)
M_169	66000	transmembrane protein 108(TMEM108)
M_169	7113	transmembrane protease, serine 2(TMPRSS2)
M_169	80258	EF-hand domain containing 2(EFHC2)
M_169	2882	glutathione peroxidase 7(GPX7)
M_169	51458	Rh family C glycoprotein(RHCG)
M_169	4135	microtubule associated protein 6(MAP6)
M_169	150356	chondroadherin like(CHADL)
M_169	130026	islet cell autoantigen 1 like(ICA1L)
M_169	5017	ovo like transcriptional repressor 1(OVOL1)
M_169	23349	PHD finger protein 24(PHF24)
M_169	348303	selenoprotein V(SELENOV)
M_169	170850	potassium voltage-gated channel modifier subfamily G member 3(KCNG3)
M_169	286527	thymosin beta 15B(TMSB15B)
M_169	81831	neuropilin and tolloid like 2(NETO2)
M_169	8193	double PHD fingers 1(DPF1)
M_169	25806	ventral anterior homeobox 2(VAX2)
M_169	254263	cornichon family AMPA receptor auxiliary protein
M_169	404550	chromosome 16 open reading frame 74(C16orf74)
M_169	84553	failed axon connections homolog(FAXC)
M_169	84944	maelstrom spermatogenic transposon silencer(MAEL)
M_169	23109	dendrin(DDN)
M_169	430	achaete-scute family bHLH transcription factor
M_169	22843	protein phosphatase, Mg2+/Mn2+ dependent
M_169	144097	chromosome 11 open reading frame 84(C11orf84)
M_169	201780	solute carrier family 10 member 4(SLC10A4)
M_169	1690	cochlin(COCH)
M_169	79661	nei like DNA glycosylase 1(NEIL1)
M_169	64506	cytoplasmic polyadenylation element binding protein
M_169	84179	major facilitator superfamily domain containing

M_169	79730	NOP2/Sun RNA methyltransferase family member 7(NSUN7)
M_169	127731	von Willebrand factor A domain containing
M_169	9331	beta-1,4-galactosyltransferase 6(B4GALT6)
M_169	57412	arsenite methyltransferase(AS3MT)
M_169	5413	septin 5(SEPT5)
M_169	254050	leucine rich repeat containing 43(LRRC43)
M_169	79600	tectonic family member 1(TCTN1)
M_169	349152	DPY19L2 pseudogene 2(DPY19L2P2)
M_169	23366	KIAA0895(KIAA0895)
M_169	7345	ubiquitin C-terminal hydrolase L1(UCHL1)
M_169	84002	UDP-GlcNAc:betaGal beta-1,3-N-acetylglucosaminyltransferase 5(B3GNT5)
M_169	79603	ceramide synthase 4(CERS4)
M_169	285800	proline rich 18(PRR18)
M_169	201161	centromere protein V(CENPV)
M_169	11013	thymosin beta 15a(TMSB15A)
M_169	57501	KIAA1257(KIAA1257)
M_169	6785	ELOVL fatty acid elongase 4(ELOVL4)
M_169	554236	DPY19L2 pseudogene 1(DPY19L2P1)
M_169	55506	H2A histone family member Y2(H2AFY2)
M_169	159989	deuterosome assembly protein 1(DEUP1)
M_169	26049	family with sequence similarity 169 member
M_169	347252	insulin like growth factor binding protein like
M_169	631	beaded filament structural protein 1(BFSP1)
M_169	2245	FYVE, RhoGEF and PH domain containing 1(FGD1)
M_169	387597	immunoglobulin like domain containing receptor
M_169	56952	phosphoribosyl transferase domain containing
M_169	137392	family with sequence similarity 92 member
M_169	56154	testis expressed 15(TEX15)
M_169	23433	ras homolog family member Q(RHOQ)
M_169	10963	stress induced phosphoprotein 1(STIP1)

M_169	6714	SRC proto-oncogene, non-receptor tyrosine
M_169	55194	eva-1 homolog B(EVA1B)
M_169	4353	myeloperoxidase(MPO)
M_169	387804	V-set and transmembrane domain containing
M_169	200162	sperm associated antigen 17(SPAG17)
M_169	55502	hes family bHLH transcription factor 6(HES6)
M_169	203111	glutamate rich 5(ERICH5)
M_169	90423	ATPase H+ transporting V1 subunit E2(ATP6V1E2)
M_169	26281	fibroblast growth factor 20(FGF20)
M_169	65055	receptor accessory protein 1(REEP1)
M_169	54810	GIPC PDZ domain containing family member
M_170	873	carbonyl reductase 1(CBR1)
M_170	139341	FUN14 domain containing 1(FUNDC1)
M_170	23478	SEC11 homolog A, signal peptidase complex
M_170	874	carbonyl reductase 3(CBR3)
M_170	5634	phosphoribosyl pyrophosphate synthetase 2(PRPS2)
M_170	1728	NAD(P)H quinone dehydrogenase 1(NQO1)
M_170	79135	apolipoprotein O(APOO)
M_170	23423	transmembrane p24 trafficking protein 3(TMED3)
M_170	10549	peroxiredoxin 4(PRDX4)
M_170	54469	zinc finger AN1-type containing 6(ZFAND6)
M_170	55151	transmembrane protein 38B(TMEM38B)
M_170	7117	thymosin beta 4, X-linked pseudogene 8(TMSB4XP8)
M_170	5160	pyruvate dehydrogenase (lipoamide) alpha 1(PDHA1)
M_170	9377	cytochrome c oxidase subunit 5A(COX5A)
M_170	6322	sex comb on midleg-like 1 (Drosophila)(SCML1)
M_170	10519	calcium and integrin binding 1(CIB1)
M_170	6627	small nuclear ribonucleoprotein polypeptide
M_170	10588	5,10-methenyltetrahydrofolate synthetase (5-formyltetrahydrofolate cyclo-ligase)(MTHFS)
M_170	6611	spermine synthase(SMS)
M_170	6303	spermidine/spermine N1-acetyltransferase 1(SAT1)

M_170	91703	aminoacylase 3(ACY3)
M_170	26589	mitochondrial ribosomal protein L46(MRPL46)
M_170	4277	MHC class I polypeptide-related sequence B(MICB)
M_170	54939	COMM domain containing 4(COMMD4)
M_170	3419	isocitrate dehydrogenase 3 (NAD(+)) alpha(IDH3A)
M_170	58526	MID1 interacting protein 1(MID1IP1)
M_170	5685	proteasome subunit alpha 4(PSMA4)
M_170	347853	T-box 10(TBX10)
M_170	123207	chromosome 15 open reading frame 40(C15orf40)
M_170	83640	family with sequence similarity 103 member
M_170	64963	mitochondrial ribosomal protein S11(MRPS11)
M_170	10553	HIV-1 Tat interactive protein 2(HTATIP2)
M_170	2810	stratifin(SFN)
M_170	8544	pirin(PIR)
M_170	55829	selenoprotein S(SELENOS)
M_170	3052	holocytochrome c synthase(HCCS)
M_170	2108	electron transfer flavoprotein alpha subunit(ETFA)
M_170	80213	TM2 domain containing 3(TM2D3)
M_170	374659	HD domain containing 3(HDDC3)
M_170	404281	YY2 transcription factor(YY2)
M_170	9367	RAB9A, member RAS oncogene family(RAB9A)
M_170	60490	phosphopantothenoylcysteine decarboxylase(PPCDC)
M_170	55272	IMP3, U3 small nucleolar ribonucleoprotein(IMP3)
M_170	80349	WD repeat domain 61(WDR61)
M_170	9399	stomatin like 1(STOML1)
M_170	26263	F-box protein 22(FBXO22)
M_170	10933	mortality factor 4 like 1(MORF4L1)
M_170	55466	DnaJ heat shock protein family (Hsp40) member A4(DNAJA4)
M_170	145773	family with sequence similarity 81 member
M_170	4828	neuromedin B(NMB)
M_170	123688	hydroxylysine kinase(HYKK)

M_170	123355	leucine rich repeat containing 28(LRRC28)
M_170	692224	FBXO22 antisense RNA 1(FBXO22-AS1)
M_170	481	ATPase Na+/K+ transporting subunit beta 1(ATP1B1)
M_170	92912	ubiquitin conjugating enzyme E2 Q2(UBE2Q2)
M_170	84993	ubiquitin like 7(UBL7)
M_170	3418	isocitrate dehydrogenase (NADP(+)) 2,
M_170	27020	neuroplastin(NPTN)
M_170	10073	snurportin 1(SNUPN)
M_170	79986	zinc finger protein 702, pseudogene(ZNF702P)
M_171	7433	vasoactive intestinal peptide receptor 1(VIPR1)
M_171	7107	G protein-coupled receptor 137B(GPR137B)
M_171	89866	SEC16 homolog B, endoplasmic reticulum export factor(SEC16B)
M_171	55108	BSD domain containing 1(BSDC1)
M_171	80024	solute carrier family 8 member B1(SLC8B1)
M_171	79830	zinc finger MYM-type containing 1(ZMYM1)
M_171	8560	delta 4-desaturase, sphingolipid 1(DEGS1)
M_171	57605	phosphatidylinositol transfer protein membrane associated 2(PITPNM2)
M_171	7579	zinc finger and SCAN domain containing
M_171	51347	TAO kinase 3(TAOK3)
M_171	694	BTG anti-proliferation factor 1(BTG1)
M_171	88455	ankyrin repeat domain 13A(ANKRD13A)
M_171	9698	pumilio RNA binding family member 1(PUM1)
M_171	402415	XK related, X-linked(XKRX)
M_171	23254	kazrin, periplakin interacting protein(KAZN)
M_171	128178	EDAR associated death domain(EDARADD)
M_171	56605	endoplasmic reticulum oxidoreductase 1 beta(ERO1B)
M_171	196463	phospholipase B domain containing 2(PLBD2)
M_171	56994	choline phosphotransferase 1(CHPT1)
M_171	57648	KIAA1522(KIAA1522)
M_171	7188	TNF receptor associated factor 5(TRAF5)

M_171	1912	polyhomeotic homolog 2(PHC2)
M_171	54897	castor zinc finger 1(CASZ1)
M_171	53373	two pore segment channel 1(TPCN1)
M_171	200081	taxilin alpha(TXLNA)
M_171	54621	V-set and immunoglobulin domain containing
M_171	3964	galectin 8(LGALS8)
M_171	64853	axin interactor, dorsalization associated(AIDA)
M_171	23457	ATP binding cassette subfamily B member 9(ABCB9)
M_171	127544	ring finger protein 19B(RNF19B)
M_171	55612	fermitin family member 1(FERMT1)
M_171	92558	BICD family like cargo adaptor 1(BICDL1)
M_171	6538	solute carrier family 6 member 11(SLC6A11)
M_171	9202	zinc finger MYM-type containing 4(ZMYM4)
M_171	653121	zinc finger and BTB domain containing 8A(ZBTB8A)
M_171	9204	zinc finger MYM-type containing 6(ZMYM6)
M_171	4604	myosin binding protein C, slow type(MYBPC1)
M_171	23633	karyopherin subunit alpha 6(KPNA6)
M_171	26507	cyclin and CBS domain divalent metal cation transport mediator 1(CNNM1)
M_171	149076	zinc finger protein 362(ZNF362)
M_171	79158	N-acetylglucosamine-1-phosphate transferase alpha and beta subunits(GNPTAB)
M_171	55223	tripartite motif containing 62(TRIM62)
M_171	307	annexin A4(ANXA4)
M_171	1910	endothelin receptor type B(EDNRB)
M_171	9104	regucalcin(RGN)
M_171	5950	retinol binding protein 4(RBP4)
M_171	10141	long intergenic non-protein coding RNA
M_171	7125	troponin C2, fast skeletal type(TNNC2)
M_171	120224	transmembrane protein 45B(TMEM45B)
M_171	401288	long intergenic non-protein coding RNA
M_171	80069	long intergenic non-protein coding RNA

M_171	779	calcium voltage-gated channel subunit alpha1
M_172	387856	coiled-coil domain containing 184(CCDC184)
M_172	146771	testicular cell adhesion molecule 1,
M_172	284071	meiosis specific with coiled-coil domain(MEIOC)
M_172	3196	T-cell leukemia homeobox 2(TLX2)
M_172	25830	sulfotransferase family 4A member 1(SULT4A1)
M_172	2826	C-C motif chemokine receptor 10(CCR10)
M_172	4744	neurofilament heavy polypeptide(NEFH)
M_172	79187	fibronectin type III and SPRY domain containing
M_172	81626	SHC binding and spindle associated 1 like(SHCBP1L)
M_172	27127	structural maintenance of chromosomes 1B(SMC1B)
M_172	5913	receptor associated protein of the synapse(RAPSN)
M_172	5988	ret finger protein like 1(RFPL1)
M_172	122402	tudor domain containing 9(TDRD9)
M_172	9032	transmembrane 4 L six family member 5(TM4SF5)
M_172	3237	homeobox D11(HOXD11)
M_172	8277	transketolase like 1(TKTL1)
M_172	3743	potassium voltage-gated channel subfamily A member 7(KCNA7)
M_172	645784	ankyrin repeat domain 36B pseudogene
M_172	55286	chromosome 4 open reading frame 19(C4orf19)
M_172	59342	serine carboxypeptidase 1(SCPEP1)
M_172	22996	tetratricopeptide repeat domain 39A(TTC39A)
M_172	29893	PSMC3 interacting protein(PSMC3IP)
M_172	25894	pleckstrin homology and RhoGEF domain containing G4(PLEKHG4)
M_172	9355	LIM homeobox 2(LHX2)
M_172	27175	tubulin gamma 2(TUBG2)
M_172	23217	zinc finger RNA binding protein 2(ZFR2)
M_172	124817	cyclin N-terminal domain containing 1(CNTD1)
M_172	7288	tubby like protein 2(TULP2)
M_172	23539	solute carrier family 16 member 8(SLC16A8)

M_172	10388	synaptonemal complex protein 2(SYCP2)
M_172	3238	homeobox D12(HOXD12)
M_172	60484	hyaluronan and proteoglycan link protein 2(HAPLN2)
M_172	56675	nuclear receptor interacting protein 3(NRIP3)
M_172	10734	stromal antigen 3(STAG3)
M_172	3239	homeobox D13(HOXD13)
M_172	10740	RFPL1 antisense RNA 1(RFPL1S)
M_172	84215	zinc finger protein 541(ZNF541)
M_172	9502	X antigen family member 2(XAGE2)
M_172	2905	glutamate ionotropic receptor NMDA type subunit 2C(GRIN2C)
M_172	669	bisphosphoglycerate mutase(BPGM)
M_172	80329	UL16 binding protein 1(ULBP1)
M_172	79734	potassium channel tetramerization domain containing 17(KCTD17)
M_172	80174	DBF4 zinc finger B(DBF4B)
M_172	83592	aldo-keto reductase family 1 member E2(AKR1E2)
M_172	646024	retinoic acid early transcript 1K
M_172	57168	aspartate beta-hydroxylase domain containing
M_172	50512	podocalyxin like 2(PODXL2)
M_172	79465	UL16 binding protein 3(ULBP3)
M_172	374877	chromosome 19 open reading frame 45(C19orf45)
M_172	146212	potassium channel tetramerization domain containing 19(KCTD19)
M_172	3236	homeobox D10(HOXD10)
M_172	55282	leucine rich repeat containing 36(LRRC36)
M_172	165545	DEAQ-box RNA dependent ATPase 1(DQX1)
M_172	56923	neuromedin U receptor 2(NMUR2)
M_172	3294	hydroxysteroid 17-beta dehydrogenase 2(HSD17B2)
M_172	59350	relaxin/insulin like family peptide receptor 1(RXFP1)
M_173	55891	lens epithelial protein(LENEP)
M_173	55764	intraflagellar transport 122(IFT122)

M_173	388886	leucine rich repeat containing 75B(LRRC75B)
M_173	149954	BPI fold containing family B member 4(BPIFB4)
M_173	54532	ubiquitin specific peptidase 53(USP53)
M_173	245973	ATPase H+ transporting V1 subunit C2(ATP6V1C2)
M_173	54857	glycerophosphodiester phosphodiesterase domain containing 2(GDPD2)
M_173	65268	WNK lysine deficient protein kinase 2(WNK2)
M_173	4114	MAGE family member B3(MAGEB3)
M_173	4115	MAGE family member B4(MAGEB4)
M_173	9132	potassium voltage-gated channel subfamily Q member 4(KCNQ4)
M_173	51560	RAB6B, member RAS oncogene family(RAB6B)
M_173	284729	espin pseudogene(ESPNP)
M_173	139728	pregnancy up-regulated nonubiquitous CaM
M_173	260429	protease, serine 33(PRSS33)
M_173	122060	SLAIN motif family member 1(SLAIN1)
M_173	81579	phospholipase A2 group XIIA(PLA2G12A)
M_173	8444	dual specificity tyrosine phosphorylation regulated kinase 3(DYRK3)
M_173	9573	growth differentiation factor 3(GDF3)
M_173	92235	dual specificity phosphatase 27 (putative)(DUSP27)
M_173	140683	BPI fold containing family A member 2(BPIFA2)
M_173	441476	sperm-tail PG-rich repeat containing 3(STPG3)
M_173	2016	empty spiracles homeobox 1(EMX1)
M_173	12	serpin family A member 3(SERPINA3)
M_173	4829	neuromedin B receptor(NMBR)
M_173	23209	megalencephalic leukoencephalopathy with subcortical cysts 1(MLC1)
M_173	57408	leucine rich repeats and transmembrane domains
M_173	80341	BPI fold containing family B member 2(BPIFB2)
M_173	790952	embryonic stem cell related (non-protein
M_173	4211	Meis homeobox 1(MEIS1)

M_173	139189	diacylglycerol kinase kappa(DGKK)
M_173	29116	myosin regulatory light chain interacting
M_173	2678	gamma-glutamyltransferase 1(GGT1)
M_173	91227	gamma-glutamyltransferase light chain 2(GGTLC2)
M_173	10991	solute carrier family 38 member 3(SLC38A3)
M_173	23418	crumbs 1, cell polarity complex component(CRB1)
M_173	55799	calcium voltage-gated channel auxiliary subunit alpha2delta 3(CACNA2D3)
M_173	816	calcium/calmodulin dependent protein kinase II beta(CAMK2B)
M_173	285154	CYP1B1 antisense RNA 1(CYP1B1-AS1)
M_173	94097	sideroflexin 5(SFXN5)
M_173	59353	transmembrane protein 35A(TMEM35A)
M_173	9829	DnaJ heat shock protein family (Hsp40) member C6(DNAJC6)
M_173	10267	receptor activity modifying protein 1(RAMP1)
M_173	127833	synaptotagmin 2(SYT2)
M_173	2766	guanosine monophosphate reductase(GMPR)
M_173	5104	serpin family A member 5(SERPINA5)
M_173	3400	inhibitor of DNA binding 4, HLH protein(ID4)
M_173	3241	hippocalcin like 1(HPCAL1)
M_173	10912	growth arrest and DNA damage inducible gamma(GADD45G)
M_173	6535	solute carrier family 6 member 8(SLC6A8)
M_173	9290	G protein-coupled receptor 55(GPR55)
M_173	346689	killer cell lectin like receptor G2(KLRG2)
M_173	359710	BPI fold containing family B member 3(BPIFB3)
M_173	51207	dual specificity phosphatase 13(DUSP13)
M_173	1644	dopa decarboxylase(DDC)
M_173	63827	brevican(BCAN)
M_173	360226	protease, serine 41(PRSS41)
M_173	376412	ring finger protein 126 pseudogene 1(RNF126P1)

M_173	1579	cytochrome P450 family 4 subfamily A member 11(CYP4A11)
M_173	10942	protease, serine 21(PRSS21)
M_173	491	ATPase plasma membrane Ca2+ transporting
M_173	168507	polycystin 1 like 1, transient receptor potential channel interacting(PKD1L1)
M_173	2912	glutamate metabotropic receptor 2(GRM2)
M_173	100131213	ZNF503 antisense RNA 2(ZNF503-AS2)
M_173	2679	gamma-glutamyltransferase 3 pseudogene(GGT3P)
M_173	387778	speedy/RINGO cell cycle regulator family member C(SPDYC)
M_173	128653	chromosome 20 open reading frame 141(C20orf141)
M_173	54465	Ewing tumor associated antigen 1(ETAA1)
M_173	644165	breakpoint cluster region pseudogene 3(BCRP3)
M_173	4025	lactoperoxidase(LPO)
M_173	84858	zinc finger protein 503(ZNF503)
M_173	642843	cleavage and polyadenylation specific factor 4
M_173	124220	zymogen granule protein 16B(ZG16B)
M_173	50649	Rho guanine nucleotide exchange factor
M_173	7020	transcription factor AP-2 alpha(TFAP2A)
M_173	646074	POM121 transmembrane nucleoporin like 10, pseudogene(POM121L10P)
M_173	9211	leucine rich glioma inactivated 1(LGI1)
M_173	7076	TIMP metallopeptidase inhibitor 1(TIMP1)
M_173	375133	phosphatidylinositol 4-kinase alpha pseudogene
M_173	3158	3-hydroxy-3-methylglutaryl-CoA synthase
M_173	51298	theg spermatid protein(THEG)
M_173	256394	serpin family A member 11(SERPINA11)
M_173	645367	gamma-glutamyltransferase 8 pseudogene(GGT8P)
M_173	9058	solute carrier family 13 member 2(SLC13A2)
M_173	344167	forkhead box I3(FOXI3)
M_173	200523	testis expressed 37(TEX37)

M_173	820	cathelicidin antimicrobial peptide(CAMP)
M_173	327657	serpin family A member 9(SERPINA9)
M_173	145264	serpin family A member 12(SERPINA12)
M_173	80099	chromosome 7 open reading frame 69(C7orf69)
M_173	10451	vav guanine nucleotide exchange factor 3(VAV3)
M_174	84951	tensin 4(TNS4)
M_174	643008	small integral membrane protein 5(SMIM5)
M_174	57619	shroom family member 3(SHROOM3)
M_174	6539	solute carrier family 6 member 12(SLC6A12)
M_174	2709	gap junction protein beta 5(GJB5)
M_174	80045	G protein-coupled receptor 157(GPR157)
M_174	128488	WAP four-disulfide core domain 12(WFDC12)
M_174	56649	transmembrane protease, serine 4(TMPRSS4)
M_174	83959	solute carrier family 4 member 11(SLC4A11)
M_174	4118	mal, T-cell differentiation protein(MAL)
M_174	64063	protease, serine 22(PRSS22)
M_174	200879	lipase H(LIPH)
M_174	100130933	small integral membrane protein 6(SMIM6)
M_174	2707	gap junction protein beta 3(GJB3)
M_174	64073	chromosome 19 open reading frame 33(C19orf33)
M_174	6549	solute carrier family 9 member A2(SLC9A2)
M_174	5909	RAP1 GTPase activating protein(RAP1GAP)
M_174	4902	neurturin(NRTN)
M_174	149708	WAP four-disulfide core domain 5(WFDC5)
M_174	345079	sosondowah ankyrin repeat domain family member B(SOWAHB)
M_174	80761	uroplakin 3B(UPK3B)
M_174	2752	glutamate-ammonia ligase(GLUL)
M_174	7039	transforming growth factor alpha(TGFA)
M_174	389015	solute carrier family 9 member A4(SLC9A4)
M_174	25861	whirlin(WHRN)
M_174	29943	peptidyl arginine deiminase 1(PADI1)

M_174	828	calcyphosine(CAPS)
M_174	360	aquaporin 3 (Gill blood group)(AQP3)
M_174	7850	interleukin 1 receptor type 2(IL1R2)
M_174	8399	phospholipase A2 group X(PLA2G10)
M_174	10103	tetraspanin 1(TSPAN1)
M_174	127534	gap junction protein beta 4(GJB4)
M_174	135932	transmembrane protein 139(TMEM139)
M_174	51330	TNF receptor superfamily member 12A(TNFRSF12A)
M_174	203197	transmembrane protein 268(TMEM268)
M_174	4585	mucin 4, cell surface associated(MUC4)
M_174	200958	mucin 20, cell surface associated(MUC20)
M_174	9052	G protein-coupled receptor class C group 5 member A(GPRC5A)
M_174	114780	polycystin 1 like 2 (gene/pseudogene)(PKD1L2)
M_174	114771	peptidoglycan recognition protein 3(PGLYRP3)
M_174	57115	peptidoglycan recognition protein 4(PGLYRP4)
M_174	136306	SVOP like(SVOPL)
M_174	57111	RAB25, member RAS oncogene family(RAB25)
M_174	10331	UDP-GlcNAc:betaGal beta-1,3-N-acetylglucosaminyltransferase 3(B3GNT3)
M_174	9175	mitogen-activated protein kinase kinase kinase
M_174	8612	phospholipid phosphatase 2(PLPP2)
M_174	3549	indian hedgehog(IHH)
M_174	1604	CD55 molecule (Cromer blood group)(CD55)
M_174	51700	cytochrome b5 reductase 2(CYB5R2)
M_174	122953	Jun dimerization protein 2(JDP2)
M_174	64077	phospholysine phosphohistidine inorganic pyrophosphate phosphatase(LHPP)
M_174	7483	Wnt family member 9A(WNT9A)
M_174	1962	enoyl-CoA hydratase and 3-hydroxyacyl CoA dehydrogenase(EHHADH)
M_174	10507	semaphorin 4D(SEMA4D)

M_174	230	aldolase, fructose-bisphosphate C(ALDOC)
M_174	200197	TMEM51 antisense RNA 1(TMEM51-AS1)
M_174	283422	long intergenic non-protein coding RNA
M_174	342184	formin 1(FMN1)
M_174	747	diacylglycerol lipase alpha(DAGLA)
M_174	57217	tetratricopeptide repeat domain 7A(TTC7A)
M_174	1673	defensin beta 4A(DEFB4A)
M_174	84766	calcium release activated channel regulator
M_174	3737	potassium voltage-gated channel subfamily A member 2(KCNA2)
M_174	4815	ninjurin 2(NINJ2)
M_175	85439	stonin 2(STON2)
M_175	5797	protein tyrosine phosphatase, receptor type
M_175	440461	Rho GTPase activating protein 27
M_175	5581	protein kinase C epsilon(PRKCE)
M_175	138883	olfactory receptor family 1 subfamily N member
M_175	140733	MACRO domain containing 2(MACROD2)
M_175	55195	chromosome 14 open reading frame 105(C14orf105)
M_175	26740	olfactory receptor family 1 subfamily J member
M_175	26735	olfactory receptor family 1 subfamily L member
M_175	387718	testis expressed 36(TEX36)
M_175	285704	repulsive guidance molecule family member
M_175	158131	olfactory receptor family 1 subfamily Q member
M_175	22875	ectonucleotide pyrophosphatase/phosphodiesterase 4 (putative)(ENPP4)
M_175	5066	peptidylglycine alpha-amidating
M_175	59084	ectonucleotide pyrophosphatase/phosphodiesterase 5 (putative)(ENPP5)
M_175	55636	chromodomain helicase DNA binding protein
M_175	26219	olfactory receptor family 1 subfamily J member
M_175	9514	galactose-3-O-sulfotransferase 1(GAL3ST1)
M_175	57733	glucosylceramidase beta 3 (gene/pseudogene)(GBA3)

M_175	55553	SRY-box 6(SOX6)
M_175	57507	zinc finger protein 608(ZNF608)
M_175	7366	UDP glucuronosyltransferase family 2 member B15(UGT2B15)
M_175	287	ankyrin 2(ANK2)
M_175	116328	chromosome 8 open reading frame 34(C8orf34)
M_175	7364	UDP glucuronosyltransferase family 2 member
M_175	54578	UDP glucuronosyltransferase family 1 member
M_175	4653	myocilin(MYOC)
M_175	6928	HNF1 homeobox B(HNF1B)
M_175	664701	zinc finger protein 826, pseudogene(ZNF826P)
M_175	79823	calmodulin-lysine N-methyltransferase(CAMKMT)
M_175	85569	galanin like peptide(GALP)
M_175	404552	secretoglobin family 1D member 4(SCGB1D4)
M_175	80243	phosphatidylinositol-3,4,5-trisphosphate dependent Rac exchange factor 2(PREX2)
M_175	64714	protein disulfide isomerase family A member
M_175	8635	ribonuclease T2(RNASET2)
M_175	57715	semaphorin 4G(SEMA4G)
M_175	2066	erb-b2 receptor tyrosine kinase 4(ERBB4)
M_175	401145	coiled-coil serine rich protein 1(CCSER1)
M_175	255738	proprotein convertase subtilisin/kexin type 9(PCSK9)
M_175	3026	hyaluronan binding protein 2(HABP2)
M_175	9369	neurexin 3(NRXN3)
M_175	55227	leucine rich repeat containing 1(LRRC1)
M_175	4594	methylmalonyl-CoA mutase(MUT)
M_175	26268	F-box protein 9(FBXO9)
M_175	26737	olfactory receptor family 1 subfamily L member
M_175	347168	olfactory receptor family 1 subfamily J member
M_175	4342	v-mos Moloney murine sarcoma viral oncogene homolog(MOS)
M_175	22858	intestinal cell kinase(ICK)

M_175	57161	pellino E3 ubiquitin protein ligase family member
M_175	145226	retinol dehydrogenase 12 (all-trans/9-cis/11-
M_175	7068	thyroid hormone receptor beta(THRB)
M_175	23607	CD2 associated protein(CD2AP)
M_175	2729	glutamate-cysteine ligase catalytic subunit(GCLC)
M_175	2731	glycine decarboxylase(GLDC)
M_175	25924	myosin VIIA and Rab interacting protein(MYRIP)
M_175	1795	dedicator of cytokinesis 3(DOCK3)
M_176	117166	WAP, follistatin/kazal, immunoglobulin, kunitz and netrin domain containing 1(WFIKKN1)
M_176	400242	DICER1 antisense RNA 1(DICER1-AS1)
M_176	51168	myosin XVA(MYO15A)
M_176	23512	SUZ12 polycomb repressive complex 2
M_176	283870	BRICHOS domain containing 5(BRICD5)
M_176	374887	YjeF N-terminal domain containing 3(YJEFN3)
M_176	27346	transmembrane protein 97(TMEM97)
M_176	124923	uncharacterized serine/threonine-protein kinase SgK494(SGK494)
M_176	147011	protein interacting with cyclin A1(PROCA1)
M_176	147015	dehydrogenase/reductase 13(DHRS13)
M_176	100286844	BCDIN3D antisense RNA 1(BCDIN3D-AS1)
M_176	55813	UTP6, small subunit processome component(UTP6)
M_176	55716	limb development membrane protein 1 like(LMBR1L)
M_176	81854	ZNF205 antisense RNA 1(ZNF205-AS1)
M_176	54475	notchless homolog 1(NLE1)
M_176	25766	pre-mRNA processing factor 40 homolog
M_176	2319	flotillin 2(FLOT2)
M_176	79736	transcription elongation factor, mitochondrial(TEFM)
M_176	114792	kelch like family member 32(KLHL32)
M_176	54058	chromosome 21 open reading frame 58(C21orf58)
M_176	5717	proteasome 26S subunit, non-ATPase 11(PSMD11)
M_176	55288	ras homolog family member T1(RHOT1)

M_176	645811	coiled-coil domain containing 154(CCDC154)
M_176	83871	RAB34, member RAS oncogene family(RAB34)
M_176	9311	acid sensing ion channel subunit 3(ASIC3)
M_176	5892	RAD51 paralog D(RAD51D)
M_176	200172	schlafen like 1(SLFNL1)
M_176	116238	TLC domain containing 1(TLCD1)
M_176	7756	zinc finger protein 207(ZNF207)
M_176	23098	sterile alpha and TIR motif containing 1(SARM1)
M_176	113235	solute carrier family 46 member 1(SLC46A1)
M_176	283596	small nucleolar RNA host gene 10(SNHG10)
M_176	3980	DNA ligase 3(LIG3)
M_176	55592	golgin A2 pseudogene 5(GOLGA2P5)
M_176	144132	dynein heavy chain domain 1(DNHD1)
M_176	91603	zinc finger protein 830(ZNF830)
M_176	284086	NIMA related kinase 8(NEK8)
M_176	100128788	SRRM2 antisense RNA 1(SRRM2-AS1)
M_176	81629	testis specific serine kinase 3(TSSK3)
M_176	9618	TNF receptor associated factor 4(TRAF4)
M_176	3700	inter-alpha-trypsin inhibitor heavy chain family member 4(ITIH4)
M_176	26284	Era like 12S mitochondrial rRNA chaperone
M_176	285962	WEE2 antisense RNA 1(WEE2-AS1)
M_176	84070	family with sequence similarity 186 member
M_176	3083	HGF activator(HGFAC)
M_176	64149	chromosome 17 open reading frame 75(C17orf75)
M_176	100131814	long intergenic non-protein coding RNA
M_176	117584	ring finger and FYVE like domain containing E3 ubiquitin protein ligase(RFFL)
M_176	494551	WEE1 homolog 2(WEE2)
M_176	100126793	ghrelin opposite strand/antisense RNA(GHRLOS)
M_176	57701	NCK associated protein 5 like(NCKAP5L)
M_176	642	bleomycin hydrolase(BLMH)

M_176	147172	leucine rich repeat containing 37B pseudogene 1(LRRC37BP1)
M_176	54752	fibronectin type III domain containing 8(FNDC8)
M_176	84657	long intergenic non-protein coding RNA
M_176	284232	ankyrin repeat domain 20 family member A9, pseudogene(ANKRD20A9P)
M_176	114659	leucine rich repeat containing 37B(LRRC37B)
M_176	494513	deafness, autosomal recessive 59(DFNB59)
M_176	79800	calcium responsive transcription factor(CARF)
M_176	440423	SUZ12 polycomb repressive complex 2 subunit pseudogene 1(SUZ12P1)
M_176	283011	tigger transposable element derived 2
M_176	51379	cytokine receptor like factor 3(CRLF3)
M_176	90784	uncharacterized LOC90784(LOC90784)
M_176	728448	peptidylprolyl isomerase E like pseudogene(PPIEL)
M_176	79957	progestin and adipoQ receptor family member
M_176	97	acylphosphatase 1(ACYP1)
M_176	9527	golgi SNAP receptor complex member 1(GOSR1)
M_176	112	adenylate cyclase 6(ADCY6)
M_176	401127	WD repeat domain 5 pseudogene(LOC401127)
M_176	4763	neurofibromin 1(NF1)
M_177	163115	zinc finger protein 781(ZNF781)
M_177	4256	matrix Gla protein(MGP)
M_177	53637	sphingosine-1-phosphate receptor 5(S1PR5)
M_177	375616	kielin/chordin-like protein(KCP)
M_177	79927	family with sequence similarity 110 member
M_177	164045	HFM1, ATP dependent DNA helicase
M_177	252995	fibronectin type III domain containing 5(FNDC5)
M_177	5228	placental growth factor(PGF)
M_177	84701	cytochrome c oxidase subunit 4I2(COX4I2)
M_177	29118	DEAD-box helicase 25(DDX25)
M_177	50489	CD207 molecule(CD207)

M_177	55906	zinc finger C4H2-type containing(ZC4H2)
M_177	84814	phospholipid phosphatase 7 (inactive)(PLPP7)
M_177	2791	G protein subunit gamma 11(GNG11)
M_177	347	apolipoprotein D(APOD)
M_177	9934	purinergic receptor P2Y14(P2RY14)
M_177	8536	calcium/calmodulin dependent protein kinase
M_177	340554	zinc finger CCCH-type containing 12B(ZC3H12B)
M_177	2563	gamma-aminobutyric acid type A receptor delta subunit(GABRD)
M_177	8651	suppressor of cytokine signaling 1(SOCS1)
M_177	219348	placenta specific 9(PLAC9)
M_177	100113407	transmembrane protein 170B(TMEM170B)
M_177	8785	matrilin 4(MATN4)
M_177	91977	myozenin 3(MYOZ3)
M_177	389119	family with sequence similarity 212 member
M_177	80774	LIM domain containing 2(LIMD2)
M_177	83483	plasmalemma vesicle associated protein(PLVAP)
M_177	79140	coiled-coil domain containing 28B(CCDC28B)
M_177	3490	insulin like growth factor binding protein 7(IGFBP7)
M_177	3563	interleukin 3 receptor subunit alpha(IL3RA)
M_177	54898	ELOVL fatty acid elongase 2(ELOVL2)
M_177	100190938	RAMP2 antisense RNA 1(RAMP2-AS1)
M_177	55859	brain expressed X-linked 1(BEX1)
M_177	7512	X-prolyl aminopeptidase 2(XPNPEP2)
M_177	256933	neuropeptide B(NPB)
M_177	6285	S100 calcium binding protein B(S100B)
M_177	51309	armadillo repeat containing, X-linked 1(ARMCX1)
M_177	57007	atypical chemokine receptor 3(ACKR3)
M_177	146206	capping protein regulator and myosin 1 linker
M_177	10266	receptor activity modifying protein 2(RAMP2)
M_177	128853	dual specificity phosphatase 15(DUSP15)

M_177	11317	recombination signal binding protein for immunoglobulin kappa J region like(RBPJL)
M_177	55323	La ribonucleoprotein domain family member
M_177	1071	cholesteryl ester transfer protein(CETP)
M_177	23462	hes related family bHLH transcription factor with YRPW motif 1(HEY1)
M_177	10736	SIX homeobox 2(SIX2)
M_177	1346	cytochrome c oxidase subunit 7A1(COX7A1)
M_177	84734	family with sequence similarity 167 member
M_177	3384	intercellular adhesion molecule 2(ICAM2)
M_177	10518	calcium and integrin binding family member 2(CIB2)
M_177	81557	MAGE family member D4B(MAGED4B)
M_177	9452	integral membrane protein 2A(ITM2A)
M_177	646892	SH2 domain containing 7(SH2D7)
M_177	1593	cytochrome P450 family 27 subfamily A member 1(CYP27A1)
M_177	64093	SPARC related modular calcium binding 1(SMOC1)
M_177	115650	TNF receptor superfamily member 13C(TNFRSF13C)
M_177	1482	NK2 homeobox 5(NKX2-5)
M_177	83697	solute carrier family 4 member 9(SLC4A9)
M_177	64843	ISL LIM homeobox 2(ISL2)
M_177	231	aldo-keto reductase family 1 member B(AKR1B1)
M_177	1297	collagen type IX alpha 1 chain(COL9A1)
M_177	909	CD1a molecule(CD1A)
M_177	911	CD1c molecule(CD1C)
M_177	910	CD1b molecule(CD1B)
M_177	56033	BARX homeobox 1(BARX1)
M_177	4804	nerve growth factor receptor(NGFR)
M_177	3670	ISL LIM homeobox 1(ISL1)
M_177	4137	microtubule associated protein tau(MAPT)
M_177	1187	chloride voltage-gated channel Ka(CLCNKA)
M_177	79814	agmatinase(AGMAT)

M_177	165530	C-type lectin domain family 4 member F(CLEC4F)
M_177	10882	complement C1q like 1(C1QL1)
M_177	913	CD1e molecule(CD1E)
M_177	100130148	MAPT intronic transcript 1(MAPT-IT1)
M_177	136259	Kruppel like factor 14(KLF14)
M_177	1310	collagen type XIX alpha 1 chain(COL19A1)
M_177	2205	Fc fragment of IgE receptor Ia(FCER1A)
M_177	159296	NK2 homeobox 3(NKX2-3)
M_177	130951	meiosis 1 associated protein(M1AP)
M_177	3120	major histocompatibility complex, class II, DQ beta 2(HLA-DQB2)
M_177	3118	major histocompatibility complex, class II, DQ alpha 2(HLA-DQA2)
M_177	3128	major histocompatibility complex, class II, DR beta 6 (pseudogene)(HLA-DRB6)
M_178	170370	family with sequence similarity 170 member
M_178	100033450	small nucleolar RNA, C/D box 115-13(SNORD115-
M_178	164714	tubulin tyrosine ligase like 8(TTLL8)
M_178	653240	keratin associated protein 4-11(KRTAP4-11)
M_178	386746	MAS related GPR family member G(MRGPRG)
M_178	341392	acyl-CoA synthetase medium-chain family member 4(ACSM4)
M_178	26749	G antigen 2E(GAGE2E)
M_178	317716	BPI fold containing family A member 4, pseudogene(BPIFA4P)
M_178	6561	solute carrier family 13 member 1(SLC13A1)
M_178	85280	keratin associated protein 9-4(KRTAP9-4)
M_178	161514	TBC1 domain family member 21(TBC1D21)
M_178	119678	olfactory receptor family 52 subfamily E member
M_178	119682	olfactory receptor family 51 subfamily L member
M_178	119679	olfactory receptor family 52 subfamily J member
M_178	100132476	keratin associated protein 4-7(KRTAP4-7)

M_178	100132464	family with sequence similarity 99 member B (non-protein coding)(FAM99B)
M_178	730262	peptidylprolyl isomerase A like 4E(PPIAL4E)
M_178	353333	keratin associated protein 10-10(KRTAP10-10)
M_178	148231	long intergenic non-protein coding RNA
M_178	353288	keratin 26(KRT26)
M_178	5675	pregnancy specific beta-1-glycoprotein 6(PSG6)
M_178	5676	pregnancy specific beta-1-glycoprotein 7 (gene/pseudogene)(PSG7)
M_178	341416	olfactory receptor family 6 subfamily C member
M_178	1617	deleted in azoospermia 1(DAZ1)
M_178	116085	solute carrier family 22 member 12(SLC22A12)
M_178	245915	defensin beta 112(DEFB112)
M_178	353140	late cornified envelope 2C(LCE2C)
M_178	10086	HERV-H LTR-associating 1(HHLA1)
M_178	5024	purinergic receptor P2X 3(P2RX3)
M_178	3562	interleukin 3(IL3)
M_178	133558	maestro heat like repeat family member
M_178	8688	keratin 37(KRT37)
M_178	692063	small nucleolar RNA, H/ACA box 32(SNORA32)
M_178	390053	olfactory receptor family 52 subfamily A member 4 pseudogene(OR52A4P)
M_178	390054	olfactory receptor family 52 subfamily A member
M_178	119772	olfactory receptor family 52 subfamily M member
M_178	390036	olfactory receptor family 52 subfamily K member
M_178	119764	olfactory receptor family 4 subfamily X member 2 (gene/pseudogene)(OR4X2)
M_178	2559	gamma-aminobutyric acid type A receptor alpha6 subunit(GABRA6)
M_178	7783	zona pellucida glycoprotein 2(ZP2)
M_178	390061	olfactory receptor family 51 subfamily Q member 1 (gene/pseudogene)(OR51Q1)

M_178	126206	NLR family pyrin domain containing 5(NLRP5)
M_178	26692	olfactory receptor family 2 subfamily W member
M_178	117285	defensin beta 118(DEFB118)
M_178	399814	chromosome 10 open reading frame 120(C10orf120)
M_178	5670	pregnancy specific beta-1-glycoprotein 2(PSG2)
M_178	54959	odontogenic, ameloblast asssociated(ODAM)
M_178	1585	cytochrome P450 family 11 subfamily B member 2(CYP11B2)
M_178	134829	clavesin 2(CLVS2)
M_178	5671	pregnancy specific beta-1-glycoprotein 3(PSG3)
M_178	100151684	RNA, U6atac small nuclear (U12-dependent splicing)(RNU6ATAC)
M_178	112401	baculoviral IAP repeat containing 8(BIRC8)
M_178	613227	HIG1 hypoxia inducible domain family member
M_178	85294	keratin associated protein 2-4(KRTAP2-4)
M_178	57151	lysozyme like 6(LYZL6)
M_178	127247	ankyrin repeat and SOCS box containing 17(ASB17)
M_178	618	brain cytoplasmic RNA 1(BCYRN1)
M_178	134083	olfactory receptor family 2 subfamily Y member
M_178	117195	MAS related GPR family member X3(MRGPRX3)
M_178	643803	keratin associated protein 24-1(KRTAP24-1)
M_178	386680	keratin associated protein 10-5(KRTAP10-5)
M_178	255926	ADAM metallopeptidase domain 5
M_178	254312	long intergenic non-protein coding RNA
M_178	646480	fatty acid binding protein 9(FABP9)
M_178	140881	defensin beta 129(DEFB129)
M_178	644672	claudin 25(CLDN25)
M_178	118425	prostate cancer associated transcript 4 (non-protein coding)(PCAT4)
M_178	339669	testis expressed 33(TEX33)
M_178	219869	olfactory receptor family 10 subfamily G member
M_178	100170227	small ILF3/NF90-associated RNA D(SNAR-D)

M_178	728299	keratin associated protein 19-8(KRTAP19-8)
M_178	390437	olfactory receptor family 4 subfamily N member
M_178	401137	proline rich 27(PRR27)
M_178	729330	otoconin 90(OC90)
M_178	654412	family with sequence similarity 138 member
M_178	643486	bromodomain testis associated pseudogene
M_178	151300	long intergenic non-protein coding RNA
M_178	346673	stimulated by retinoic acid 8(STRA8)
M_178	267012	D-amino acid oxidase activator(DAOA)
M_178	343641	transglutaminase 6(TGM6)
M_178	50511	synaptonemal complex protein 3(SYCP3)
M_178	100287704	uncharacterized LOC100287704(LOC100287704)
M_178	692234	small nucleolar RNA, C/D box 103A(SNORD103A)
M_178	6555	solute carrier family 10 member 2(SLC10A2)
M_178	219954	olfactory receptor family 9 subfamily I member
M_178	387742	family with sequence similarity 99 member A (non-protein coding)(FAM99A)
M_178	219482	olfactory receptor family 5 subfamily M member
M_178	83741	transcription factor AP-2 delta(TFAP2D)
M_178	219479	olfactory receptor family 5 subfamily R member 1 (gene/pseudogene)(OR5R1)
M_178	402644	peptidylprolyl isomerase A (cyclophilin A) pseudogene(LOC402644)
M_178	100124412	family with sequence similarity 138 member
M_178	390321	olfactory receptor family 6 subfamily C member
M_178	375940	deleted in malignant brain tumors 1 pseudogene
M_178	376132	leucine rich repeat containing 10(LRRC10)
M_178	391196	olfactory receptor family 2 subfamily M member
M_178	1448	casein kappa(CSN3)
M_178	344561	G protein-coupled receptor 148(GPR148)
M_178	642636	RAD21 cohesin complex component like
M_178	343406	olfactory receptor family 10 subfamily R member

M_178	440533	pregnancy specific beta-1-glycoprotein 8(PSG8)
M_178	441639	olfactory receptor family 9 subfamily K member
M_178	120775	olfactory receptor family 2 subfamily D member
M_178	100144596	long intergenic non-protein coding RNA
M_178	348249	CCL15-CCL14 readthrough (NMD candidate)(CCL15-CCL14)
M_178	139741	actin related protein T1(ACTRT1)
M_178	84569	lysozyme like 1(LYZL1)
M_178	10214	SSX family member 3(SSX3)
M_178	54657	UDP glucuronosyltransferase family 1 member
M_178	414061	DnaJ heat shock protein family (Hsp40) member B3(DNAJB3)
M_178	337878	keratin associated protein 7-1
M_178	5078	paired box 4(PAX4)
M_178	83755	keratin associated protein 4-12(KRTAP4-12)
M_178	26532	olfactory receptor family 10 subfamily H member
M_178	337879	keratin associated protein 8-1(KRTAP8-1)
M_178	144742	long intergenic non-protein coding RNA
M_178	144776	long intergenic non-protein coding RNA
M_178	115653	killer cell immunoglobulin like receptor, three Ig domains and long cytoplasmic tail 3(KIR3DL3)
M_178	168975	cyclic nucleotide binding domain containing
M_178	149018	late cornified envelope like proline rich 1(LELP1)
M_178	653643	golgin A6 family member D(GOLGA6D)
M_178	222008	V-set and transmembrane domain containing
M_178	653492	pregnancy specific beta-1-glycoprotein 10, pseudogene(PSG10P)
M_178	388960	chromosome 2 open reading frame 78(C2orf78)
M_178	641702	family with sequence similarity 138 member
M_178	344758	G protein-coupled receptor 149(GPR149)
M_178	441525	SPANX family member N4(SPANXN4)
M_178	124538	olfactory receptor family 4 subfamily D member

M_178	118670	family with sequence similarity 24 member
M_178	340654	lipase family member M(LIPM)
M_178	284532	olfactory receptor family 14 subfamily A member 16(OR14A16)
M_178	83897	keratin associated protein 3-2(KRTAP3-2)
M_178	283777	family with sequence similarity 169 member
M_178	219409	GS homeobox 1(GSX1)
M_178	643394	serine peptidase inhibitor, Kazal type 9(SPINK9)
M_178	403284	olfactory receptor family 6 subfamily C member
M_178	9152	solute carrier family 6 member 5(SLC6A5)
M_178	390197	olfactory receptor family 4 subfamily D member
M_178	390195	olfactory receptor family 5 subfamily AN member 1(OR5AN1)
M_178	26248	olfactory receptor family 2 subfamily K member
M_178	26189	olfactory receptor family 1 subfamily A member
M_178	140456	ankyrin repeat and SOCS box containing 11(ASB11)
M_178	390155	olfactory receptor family 5 subfamily T member
M_178	1068	centrin 1(CETN1)
M_178	100008586	G antigen 12F(GAGE12F)
M_178	389075	regulated endocrine specific protein 18(RESP18)
M_178	283092	olfactory receptor family 4 subfamily C member
M_178	1811	solute carrier family 26 member 3(SLC26A3)
M_178	386676	keratin associated protein 10-9(KRTAP10-9)
M_178	692108	small nucleolar RNA, C/D box 67(SNORD67)
M_178	254879	olfactory receptor family 2 subfamily T member
M_178	25763	huntingtin interacting protein M(HYPM)
M_178	390151	olfactory receptor family 8 subfamily H member
M_178	127074	olfactory receptor family 2 subfamily T member
M_178	64902	alanine--glyoxylate aminotransferase 2(AGXT2)
M_178	27296	TP53 target 5(TP53TG5)
M_178	135946	olfactory receptor family 6 subfamily B member
M_178	2694	gastric intrinsic factor(GIF)

M_178	728113	annexin A8-like 1(ANXA8L1)
M_178	403278	olfactory receptor family 5 subfamily K member
M_178	219464	olfactory receptor family 5 subfamily T member
M_178	85438	calcium binding protein, spermatid associated
M_178	283982	long intergenic non-protein coding RNA
M_178	219429	olfactory receptor family 4 subfamily C member
M_178	390439	olfactory receptor family 11 subfamily G member
M_178	6694	secreted phosphoprotein 2(SPP2)
M_178	246705	long intergenic non-protein coding RNA
M_178	149992	ankyrin repeat domain 30B pseudogene
M_178	338599	dual specificity phosphatase and pro isomerase domain containing 1(DUPD1)
M_178	2557	gamma-aminobutyric acid type A receptor alpha4 subunit(GABRA4)
M_178	3747	potassium voltage-gated channel subfamily C member 2(KCNC2)
M_178	2251	fibroblast growth factor 6(FGF6)
M_178	283601	long intergenic non-protein coding RNA
M_178	27164	spalt like transcription factor 3(SALL3)
M_178	114026	zinc finger imprinted 3(ZIM3)
M_178	4618	myogenic factor 6(MYF6)
M_178	431704	regulator of G-protein signaling 21(RGS21)
M_178	768329	#N/A
M_178	284424	MIR7-3 host gene(MIR7-3HG)
M_178	340784	H6 family homeobox 3(HMX3)
M_178	5678	pregnancy specific beta-1-glycoprotein 9(PSG9)
M_178	375323	lipoma HMGIC fusion partner-like 4(LHFPL4)
M_178	283847	telomere repeat binding bouquet formation protein
M_178	23574	p53-responsive gene 1(PRG1)
M_178	253017	trans-2,3-enoyl-CoA reductase-like(TECRL)
M_178	119180	lysozyme like 2(LYZL2)
M_178	345611	immunity related GTPase M(IRGM)

M_178	26138	long intergenic non-protein coding RNA
M_178	128408	basic helix-loop-helix family member e23(BHLHE23)
M_178	285641	solute carrier family 36 member 3(SLC36A3)
M_178	129446	xin actin binding repeat containing 2(XIRP2)
M_178	23040	myelin transcription factor 1 like(MYT1L)
M_178	57663	ubiquitin specific peptidase 29(USP29)
M_178	4544	melatonin receptor 1B(MTNR1B)
M_178	642929	general transcription factor II, i
M_178	50836	taste 2 receptor member 8(TAS2R8)
M_178	2572	glutamate decarboxylase 2(GAD2)
M_178	26664	olfactory receptor family 7 subfamily C member
M_178	644669	ankyrin repeat domain 30B pseudogene(LOC644669)
M_178	145946	spermatogenesis associated 8(SPATA8)
M_178	677803	small nucleolar RNA, H/ACA box 15(SNORA15)
M_178	390260	olfactory receptor family 6 subfamily X member
M_178	5669	pregnancy specific beta-1-glycoprotein 1(PSG1)
M_178	5672	pregnancy specific beta-1-glycoprotein 4(PSG4)
M_178	353332	keratin associated protein 12-1(KRTAP12-1)
M_178	146822	CMT1A duplicated region transcript 15(CDRT15)
M_178	158506	zinc finger protein 645(ZNF645)
M_178	3167	H6 family homeobox 2(HMX2)
M_178	360203	glycosyltransferase 6 domain containing 1(GLT6D1)
M_178	259239	WAP four-disulfide core domain 11(WFDC11)
M_178	84639	interleukin 1 family member 10 (theta)(IL1F10)
M_178	57126	CD177 molecule(CD177)
M_178	160065	prostate and testis expressed 1(PATE1)
M_178	347516	diacylglycerol O-acyltransferase 2 like 6(DGAT2L6)
M_178	4617	myogenic factor 5(MYF5)
M_178	123745	phospholipase A2 group IVE(PLA2G4E)
M_179	171425	citrate lyase beta like(CLYBL)
M_179	340061	transmembrane protein 173(TMEM173)
M_179	9674	KIAA0040(KIAA0040)

M_179	5580	protein kinase C delta(PRKCD)
M_179	133522	PPARG coactivator 1 beta(PPARGC1B)
M_179	1384	carnitine O-acetyltransferase(CRAT)
M_179	23037	PDZ domain containing 2(PDZD2)
M_179	10418	spondin 1(SPON1)
M_179	162073	inositol 1,4,5-trisphosphate receptor interacting protein like 2(ITPRIPL2)
M_179	6238	ribosome binding protein 1(RRBP1)
M_179	220441	ring finger protein 152(RNF152)
M_179	2801	golgin A2(GOLGA2)
M_179	23250	ATPase phospholipid transporting 11A(ATP11A)
M_179	388743	calpain 8(CAPN8)
M_179	2196	FAT atypical cadherin 2(FAT2)
M_179	7111	tropomodulin 1(TMOD1)
M_179	23232	TBC1 domain family member 12(TBC1D12)
M_179	1191	clusterin(CLU)
M_179	9649	Ral GEF with PH domain and SH3 binding motif 1(RALGPS1)
M_179	23338	jade family PHD finger 2(JADE2)
M_179	10783	NIMA related kinase 6(NEK6)
M_179	2155	coagulation factor VII(F7)
M_179	148229	ATPase phospholipid transporting 8B3(ATP8B3)
M_179	10618	trans-golgi network protein 2(TGOLN2)
M_179	57706	DENN domain containing 1A(DENND1A)
M_179	5365	plexin B3(PLXNB3)
M_179	23150	FERM domain containing 4B(FRMD4B)
M_179	6709	spectrin alpha, non-erythrocytic 1(SPTAN1)
M_179	23099	zinc finger and BTB domain containing 43(ZBTB43)
M_179	114991	zinc finger protein 618(ZNF618)
M_179	23092	Rho GTPase activating protein 26(ARHGAP26)
M_179	55061	sushi domain containing 4(SUSD4)
M_179	23048	formin binding protein 1(FNBP1)

M_179	9610	Ras and Rab interactor 1(RIN1)
M_179	8301	phosphatidylinositol binding clathrin assembly protein(PICALM)
M_179	9920	kelch repeat and BTB domain containing
M_179	100129034	uncharacterized LOC100129034(LOC100129034)
M_179	23348	dedicator of cytokinesis 9(DOCK9)
M_179	153090	DAB2 interacting protein(DAB2IP)
M_179	338699	ankyrin repeat domain 42(ANKRD42)
M_179	1203	ceroid-lipofuscinosis, neuronal 5(CLN5)
M_179	6812	syntaxin binding protein 1(STXBP1)
M_179	2308	forkhead box O1(FOXO1)
M_179	6482	ST3 beta-galactoside alpha-2,3-sialyltransferase
M_179	4241	melanotransferrin(MELTF)
M_179	11138	TBC1 domain family member 8(TBC1D8)
M_179	26509	myoferlin(MYOF)
M_179	4056	leukotriene C4 synthase(LTC4S)
M_179	9289	adhesion G protein-coupled receptor G1(ADGRG1)
M_179	3269	histamine receptor H1(HRH1)
M_179	2621	growth arrest specific 6(GAS6)
M_179	57595	PDZ domain containing 4(PDZD4)
M_179	23224	spectrin repeat containing nuclear envelope protein
M_179	9780	piezo type mechanosensitive ion channel component 1(PIEZO1)
M_179	134549	shroom family member 1(SHROOM1)
M_179	8082	sarcospan(SSPN)
M_179	7100	toll like receptor 5(TLR5)
M_179	115207	potassium channel tetramerization domain containing 12(KCTD12)
M_179	10610	ST6 N-acetylgalactosaminide alpha-2,6-sialyltransferase 2(ST6GALNAC2)
M_179	11343	monoglyceride lipase(MGLL)
M_179	23413	neuronal calcium sensor 1(NCS1)

M_179	8690	JRK-like(JRKL)
M_179	84441	mastermind like transcriptional coactivator
M_179	333926	protein phosphatase, Mg2+/Mn2+ dependent
M_179	403341	zinc finger and BTB domain containing 34(ZBTB34)
M_179	4881	natriuretic peptide receptor 1(NPR1)
M_179	10347	ATP binding cassette subfamily A member 7(ABCA7)
M_179	164127	coiled-coil domain containing 185(CCDC185)
M_179	2800	golgin A1(GOLGA1)
M_179	22849	cytoplasmic polyadenylation element binding protein
M_179	26190	F-box and WD repeat domain containing 2(FBXW2)
M_179	23263	MCF.2 cell line derived transforming sequence
M_179	7764	zinc finger protein 217(ZNF217)
M_179	8660	insulin receptor substrate 2(IRS2)
M_179	55930	myosin VC(MYO5C)
M_179	643365	long intergenic non-protein coding RNA
M_179	79365	basic helix-loop-helix family member e41(BHLHE41)
M_179	55755	CDK5 regulatory subunit associated protein
M_179	80235	phosphatidylinositol glycan anchor biosynthesis class
M_179	3690	integrin subunit beta 3(ITGB3)
M_179	83394	PITPNM family member 3(PITPNM3)
M_179	51585	PCF11 cleavage and polyadenylation factor
M_179	3751	potassium voltage-gated channel subfamily D member 2(KCND2)
M_179	55304	serine palmitoyltransferase long chain base subunit 3(SPTLC3)
M_179	57186	Ral GTPase activating protein catalytic alpha subunit 2(RALGAPA2)
M_179	26130	GTPase activating protein and VPS9 domains
M_179	23637	RAB GTPase activating protein 1(RABGAP1)
M_179	284654	R-spondin 1(RSPO1)
M_179	51435	scavenger receptor class A member 3(SCARA3)
M_179	58487	CREB/ATF bZIP transcription factor(CREBZF)

M_179	646982	long intergenic non-protein coding RNA
M_179	439921	matrix remodeling associated 7(MXRA7)
M_179	23052	endonuclease domain containing 1(ENDOD1)
M_179	221	aldehyde dehydrogenase 3 family member
M_179	64788	lipase maturation factor 1(LMF1)
M_179	6869	tachykinin receptor 1(TACR1)
M_179	81622	unc-93 homolog B1 (C. elegans)(UNC93B1)
M_179	3656	interleukin 1 receptor associated kinase 2(IRAK2)
M_179	57484	ring finger protein 150(RNF150)
M_179	8313	axin 2(AXIN2)
M_179	10312	T-cell immune regulator 1, ATPase H+ transporting V0 subunit a3(TCIRG1)
M_179	246330	pellino E3 ubiquitin protein ligase family member
M_179	864	runt related transcription factor 3(RUNX3)
M_179	6653	sortilin related receptor 1(SORL1)
M_179	339366	ADAMTS like 5(ADAMTSL5)
M_179	2627	GATA binding protein 6(GATA6)
M_179	259230	sphingomyelin synthase 1(SGMS1)
M_179	9455	homer scaffolding protein 2(HOMER2)
M_179	81571	MIR600 host gene(MIR600HG)
M_179	29113	chromosome 6 open reading frame 15(C6orf15)
M_179	10272	follistatin like 3(FSTL3)
M_179	1265	calponin 2(CNN2)
M_179	11064	centriolin(CNTRL)
M_179	8784	TNF receptor superfamily member 18(TNFRSF18)
M_180	140838	N-acetylneuraminic acid phosphatase(NANP)
M_180	29097	cornichon family AMPA receptor auxiliary protein
M_180	27131	sorting nexin 5(SNX5)
M_180	1716	deoxyguanosine kinase(DGUOK)
M_180	51022	glutaredoxin 2(GLRX2)
M_180	27247	NFU1 iron-sulfur cluster scaffold(NFU1)
M_180	54935	dual specificity phosphatase 23(DUSP23)

M_180	79133	NADH:ubiquinone oxidoreductase complex assembly factor 5(NDUFAF5)
M_180	100128731	oligosaccharyltransferase complex subunit 4, non-catalytic(OST4)
M_180	83444	INO80 complex subunit B(INO80B)
M_180	27429	HtrA serine peptidase 2(HTRA2)
M_180	92675	D-tyrosyl-tRNA deacylase 1(DTD1)
M_180	128308	mitochondrial ribosomal protein L55(MRPL55)
M_180	84759	polycomb group ring finger 1(PCGF1)
M_180	2647	biogenesis of lysosomal organelles complex 1 subunit 1(BLOC1S1)
M_180	388753	cytochrome c oxidase assembly factor 6(COA6)
M_180	388789	long intergenic non-protein coding RNA
M_180	56655	DNA polymerase epsilon 4, accessory
M_180	6629	small nuclear ribonucleoprotein polypeptide
M_180	2987	guanylate kinase 1(GUK1)
M_180	51239	ankyrin repeat domain 39(ANKRD39)
M_180	51002	TP53RK binding protein(TPRKB)
M_180	23559	WW domain binding protein 1(WBP1)
M_180	29107	nuclear transport factor 2 like export factor 1(NXT1)
M_180	1622	diazepam binding inhibitor, acyl-CoA binding
M_180	57136	adipocyte plasma membrane associated
M_180	150684	copper metabolism domain containing 1(COMMD1)
M_180	550	ancient ubiquitous protein 1(AUP1)
M_180	8655	dynein light chain LC8-type 1(DYNLL1)
M_180	26090	abhydrolase domain containing 12(ABHD12)
M_180	6637	small nuclear ribonucleoprotein polypeptide
M_180	51126	N(alpha)-acetyltransferase 20, NatB catalytic subunit(NAA20)
M_180	10574	chaperonin containing TCP1 subunit 7(CCT7)
M_180	9553	mitochondrial ribosomal protein L33(MRPL33)
M_180	9168	thymosin beta 10(TMSB10)

M_180	11034	destrin, actin depolymerizing factor(DSTN)
M_180	1471	cystatin C(CST3)
M_180	116540	mitochondrial ribosomal protein L53(MRPL53)
M_180	51255	ring finger protein 181(RNF181)
M_180	955	ectonucleoside triphosphate diphosphohydrolase 6 (putative)(ENTPD6)
M_180	5834	phosphorylase, glycogen; brain(PYGB)
M_180	129303	transmembrane protein 150A(TMEM150A)
M_180	8446	dual specificity phosphatase 11(DUSP11)
M_180	10617	STAM binding protein(STAMBP)
M_180	8407	transgelin 2(TAGLN2)
M_180	84908	family with sequence similarity 136 member
M_180	84173	ELMO domain containing 3(ELMOD3)
M_181	116159	cysteine and tyrosine rich 1(CYYR1)
M_181	309	annexin A6(ANXA6)
M_181	477	ATPase Na+/K+ transporting subunit alpha
M_181	6304	SATB homeobox 1(SATB1)
M_181	2662	growth differentiation factor 10(GDF10)
M_181	343450	potassium sodium-activated channel subfamily T member 2(KCNT2)
M_181	654	bone morphogenetic protein 6(BMP6)
M_181	57608	KIAA1462(KIAA1462)
M_181	11095	ADAM metallopeptidase with thrombospondin type 1 motif 8(ADAMTS8)
M_181	351	amyloid beta precursor protein(APP)
M_181	24141	lysosomal associated membrane protein family member 5(LAMP5)
M_181	5332	phospholipase C beta 4(PLCB4)
M_181	3739	potassium voltage-gated channel subfamily A member 4(KCNA4)
M_181	8777	multiple PDZ domain crumbs cell polarity complex component(MPDZ)

M_181	2239	glypican 4(GPC4)
M_181	3776	potassium two pore domain channel subfamily K member 2(KCNK2)
M_181	374462	protein tyrosine phosphatase, receptor type Q(PTPRQ)
M_181	9508	ADAM metallopeptidase with thrombospondin type 1 motif 3(ADAMTS3)
M_181	25890	ABI family member 3 binding protein(ABI3BP)
M_181	776	calcium voltage-gated channel subunit alpha1
M_181	9510	ADAM metallopeptidase with thrombospondin type 1 motif 1(ADAMTS1)
M_181	4133	microtubule associated protein 2(MAP2)
M_181	894	cyclin D2(CCND2)
M_181	30818	potassium voltage-gated channel interacting protein 3(KCNIP3)
M_181	3488	insulin like growth factor binding protein 5(IGFBP5)
M_181	10893	matrix metallopeptidase 24(MMP24)
M_181	10157	aminoadipate-semialdehyde synthase(AASS)
M_181	130733	transmembrane protein 178A(TMEM178A)
M_181	6262	ryanodine receptor 2(RYR2)
M_181	2273	four and a half LIM domains 1(FHL1)
M_181	220965	family with sequence similarity 13 member
M_181	57097	poly(ADP-ribose) polymerase family member
M_181	1363	carboxypeptidase E(CPE)
M_181	6622	synuclein alpha(SNCA)
M_181	84456	l(3)mbt-like 3 (Drosophila)(L3MBTL3)
M_181	23710	GABA type A receptor associated protein like 1(GABARAPL1)
M_181	55026	transmembrane protein 255A(TMEM255A)
M_181	1000	cadherin 2(CDH2)
M_181	80144	Fraser extracellular matrix complex subunit 1(FRAS1)
M_181	10050	solute carrier family 17 member 4(SLC17A4)
M_181	1027	cyclin dependent kinase inhibitor 1B(CDKN1B)

M_181	131096	potassium voltage-gated channel subfamily H member 8(KCNH8)
M_181	57101	anoctamin 2(ANO2)
M_181	79684	Myb/SANT DNA binding domain containing
M_181	80034	cysteine and serine rich nuclear protein 3(CSRNP3)
M_181	245812	canopy FGF signaling regulator 4(CNPY4)
M_181	79971	wntless Wnt ligand secretion mediator(WLS)
M_181	50937	cell adhesion associated, oncogene regulated(CDON)
M_181	85004	RAS like estrogen regulated growth inhibitor(RERG)
M_181	54715	RNA binding protein, fox-1 homolog 1(RBFOX1)
M_181	7139	troponin T2, cardiac type(TNNT2)
M_181	3755	potassium voltage-gated channel modifier subfamily G member 1(KCNG1)
M_181	56895	1-acylglycerol-3-phosphate O-acyltransferase
M_181	5334	phospholipase C like 1(PLCL1)
M_181	399959	mir-100-let-7a-2 cluster host gene(MIR100HG)
M_181	79611	acyl-CoA synthetase short-chain family member
M_181	55366	leucine rich repeat containing G protein-coupled receptor 4(LGR4)
M_181	6586	slit guidance ligand 3(SLIT3)
M_181	57211	adhesion G protein-coupled receptor G6(ADGRG6)
M_181	1288	collagen type IV alpha 6 chain(COL4A6)
M_181	1287	collagen type IV alpha 5 chain(COL4A5)
M_181	79992	AGPAT4 intronic transcript 1(AGPAT4-IT1)
M_181	53353	LDL receptor related protein 1B(LRP1B)
M_181	1602	dachshund family transcription factor 1(DACH1)
M_181	214	activated leukocyte cell adhesion molecule(ALCAM)
M_181	4753	neural EGFL like 2(NELL2)
M_181	4908	neurotrophin 3(NTF3)
M_181	65998	chromosome 11 open reading frame 95(C11orf95)
M_181	10290	SPEG complex locus(SPEG)
M_181	4919	receptor tyrosine kinase like orphan receptor 1(ROR1)

M_181	81491	G protein-coupled receptor 63(GPR63)
M_181	136227	collagen type XXVI alpha 1 chain(COL26A1)
M_181	51059	family with sequence similarity 135 member
M_181	340273	ATP binding cassette subfamily B member 5(ABCB5)
M_181	84189	SLIT and NTRK like family member 6(SLITRK6)
M_181	4852	neuropeptide Y(NPY)
M_181	6783	sulfotransferase family 1E member 1(SULT1E1)
M_181	55504	TNF receptor superfamily member 19(TNFRSF19)
M_181	64168	N-terminal EF-hand calcium binding protein
M_181	50859	SPARC/osteonectin, cwcv and kazal like domains proteoglycan 3(SPOCK3)
M_181	9348	N-deacetylase and N-sulfotransferase 3(NDST3)
M_181	100128164	four and a half LIM domains 1
M_181	60495	heparanase 2 (inactive)(HPSE2)
M_181	57578	unc-79 homolog (C. elegans)(UNC79)
M_181	2550	gamma-aminobutyric acid type B receptor subunit 1(GABBR1)
M_181	7373	collagen type XIV alpha 1 chain(COL14A1)
M_181	91851	chordin like 1(CHRDL1)
M_181	2247	fibroblast growth factor 2(FGF2)
M_181	2257	fibroblast growth factor 12(FGF12)
M_181	5521	protein phosphatase 2 regulatory subunit
M_181	5801	protein tyrosine phosphatase, receptor type R(PTPRR)
M_181	2983	guanylate cyclase 1 soluble subunit beta(GUCY1B3)
M_181	23767	fibronectin leucine rich transmembrane protein
M_181	374860	ankyrin repeat domain 30B(ANKRD30B)
M_181	341947	cytochrome c oxidase subunit 8C(COX8C)
M_181	286319	tumor suppressor candidate 1(TUSC1)
M_181	2770	G protein subunit alpha i1(GNAI1)
M_181	4921	discoidin domain receptor tyrosine kinase 2(DDR2)
M_181	5067	contactin 3(CNTN3)
M_181	283576	zinc finger DHHC-type containing 22(ZDHHC22)

M_181	7932	olfactory receptor family 2 subfamily H member
M_181	26052	dynamin 3(DNM3)
M_181	1047	calmegin(CLGN)
M_181	79071	ELOVL fatty acid elongase 6(ELOVL6)
M_181	57134	mannosidase alpha class 1C member 1(MAN1C1)
M_181	3977	leukemia inhibitory factor receptor alpha(LIFR)
M_181	6909	T-box 2(TBX2)
M_181	3741	potassium voltage-gated channel subfamily A member 5(KCNA5)
M_181	57631	leucine rich repeats and calponin homology domain containing 2(LRCH2)
M_181	124149	ankyrin repeat domain 26 pseudogene
M_181	8538	BARX homeobox 2(BARX2)
M_181	651	bone morphogenetic protein 3(BMP3)
M_181	51454	GULP, engulfment adaptor PTB domain containing
M_181	388135	chromosome 15 open reading frame 59(C15orf59)
M_181	51807	tubulin alpha 8(TUBA8)
M_181	3641	insulin like 4(INSL4)
M_181	9750	family with sequence similarity 65 member
M_181	25925	zinc finger protein 521(ZNF521)
M_181	2138	EYA transcriptional coactivator and phosphatase
M_181	6769	SH3 and cysteine rich domain(STAC)
M_181	220963	solute carrier family 16 member 9(SLC16A9)
M_181	388407	chromosome 17 open reading frame 82(C17orf82)
M_181	56967	chromosome 14 open reading frame 132(C14orf132)
M_181	285456	RPL34 antisense RNA 1 (head to head)(RPL34-AS1)
M_182	124540	musashi RNA binding protein 2(MSI2)
M_182	284161	glycerophosphodiester phosphodiesterase domain containing 1(GDPD1)
M_182	200035	nudix hydrolase 17(NUDT17)
M_182	140735	dynein light chain LC8-type 2(DYNLL2)
M_182	10238	DDB1 and CUL4 associated factor 7(DCAF7)

M_182	51136	ring finger protein, transmembrane 1(RNFT1)
M_182	92335	STE20-related kinase adaptor alpha(STRADA)
M_182	9931	helicase with zinc finger(HELZ)
M_182	8493	protein phosphatase, Mg2+/Mn2+ dependent
M_182	63897	HEAT repeat containing 6(HEATR6)
M_182	51321	archaelysin family metallopeptidase 2(AMZ2)
M_182	8161	coilin(COIL)
M_182	201283	archaelysin family metallopeptidase 2 pseudogene
M_182	9969	mediator complex subunit 13(MED13)
M_182	404093	CUE domain containing 1(CUEDC1)
M_182	1213	clathrin heavy chain(CLTC)
M_182	4591	tripartite motif containing 37(TRIM37)
M_182	79665	DEAH-box helicase 40(DHX40)
M_182	26115	tetratricopeptide repeat, ankyrin repeat and coiled-coil containing 2(TANC2)
M_182	5573	protein kinase cAMP-dependent type I regulatory subunit alpha(PRKAR1A)
M_182	117246	FtsJ homolog 3(FTSJ3)
M_182	10513	amyloid beta precursor protein binding protein
M_182	651250	leucine rich repeat containing 37 member A16, pseudogene(LRRC37A16P)
M_182	90799	centrosomal protein 95(CEP95)
M_182	57508	integrator complex subunit 2(INTS2)
M_182	91369	ankyrin repeat domain 40(ANKRD40)
M_182	252983	syntaxin binding protein 4(STXBP4)
M_182	4215	mitogen-activated protein kinase kinase kinase
M_182	285315	chromosome 3 open reading frame 33(C3orf33)
M_182	84669	ubiquitin specific peptidase 32(USP32)
M_182	5705	proteasome 26S subunit, ATPase 5(PSMC5)
M_182	6198	ribosomal protein S6 kinase B1(RPS6KB1)
M_182	1655	DEAD-box helicase 5(DDX5)
M_182	9110	myotubularin related protein 4(MTMR4)

M_182	81671	vacuole membrane protein 1(VMP1)
M_182	51174	tubulin delta 1(TUBD1)
M_182	55379	leucine rich repeat containing 59(LRRC59)
M_182	64750	SMAD specific E3 ubiquitin protein ligase
M_182	11325	DEAD-box helicase 42(DDX42)
M_182	54828	BCAS3, microtubule associated cell migration
M_182	10672	G protein subunit alpha 13(GNA13)
M_182	201134	centrosomal protein 112(CEP112)
M_182	55028	chromosome 17 open reading frame 80(C17orf80)
M_182	2941	glutathione S-transferase alpha 4(GSTA4)
M_182	7716	vascular endothelial zinc finger 1(VEZF1)
M_182	11011	tousled like kinase 2(TLK2)
M_182	54903	Meckel syndrome, type 1(MKS1)
M_182	11232	DNA polymerase gamma 2, accessory
M_182	2186	bromodomain PHD finger transcription factor(BPTF)
M_182	55852	testis expressed 2(TEX2)
M_182	155382	VPS37D, ESCRT-I subunit(VPS37D)
M_182	5889	RAD51 paralog C(RAD51C)
M_182	51155	hematological and neurological expressed 1(HN1)
M_182	55181	SMG8, nonsense mediated mRNA decay
M_182	284018	chromosome 17 open reading frame 58(C17orf58)
M_182	10401	protein inhibitor of activated STAT 3(PIAS3)
M_182	2081	endoplasmic reticulum to nucleus signaling 1(ERN1)
M_182	8515	integrin subunit alpha 10(ITGA10)
M_182	64764	cAMP responsive element binding protein 3 like
M_182	135886	Williams-Beuren syndrome chromosome region 28(WBSCR28)
M_182	10140	transducer of ERBB2, 1(TOB1)
M_182	148741	ankyrin repeat domain 35(ANKRD35)
M_183	312	annexin A13(ANXA13)
M_183	199964	transmembrane protein 61(TMEM61)
M_183	116372	LY6/PLAUR domain containing 1(LYPD1)

M_183	23057	nicotinamide nucleotide adenylyltransferase
M_183	51171	hydroxysteroid 17-beta dehydrogenase
M_183	401562	lipocalin like 1(LCNL1)
M_183	9388	lipase G, endothelial type(LIPG)
M_183	221301	family with sequence similarity 26 member
M_183	8495	PPFIA binding protein 2(PPFIBP2)
M_183	23612	pleckstrin homology like domain family A member 3(PHLDA3)
M_183	284525	solute carrier family 9 member C2
M_183	2863	G protein-coupled receptor 39(GPR39)
M_183	27198	hydroxycarboxylic acid receptor 1(HCAR1)
M_183	196996	GRAM domain containing 2(GRAMD2)
M_183	247	arachidonate 15-lipoxygenase, type B(ALOX15B)
M_183	50964	sclerostin(SOST)
M_183	54769	DIRAS family GTPase 2(DIRAS2)
M_183	10391	coronin 2B(CORO2B)
M_183	93273	LEM domain containing 1(LEMD1)
M_183	80975	transmembrane protease, serine 5(TMPRSS5)
M_183	114907	F-box protein 32(FBXO32)
M_183	79054	transient receptor potential cation channel subfamily M member 8(TRPM8)
M_183	7809	barttin CLCNK type accessory beta subunit(BSND)
M_183	6330	sodium voltage-gated channel beta subunit 4(SCN4B)
M_183	5357	plastin 1(PLS1)
M_183	253314	eukaryotic translation initiation factor 4E family member 1B(EIF4E1B)
M_183	80019	ubiquitin domain containing 1(UBTD1)
M_183	26287	ankyrin repeat domain 2(ANKRD2)
M_183	91373	UDP-N-acetylglucosamine pyrophosphorylase 1 like 1(UAP1L1)
M_183	157848	NK6 homeobox 3(NKX6-3)

M_183	84141	eva-1 homolog A, regulator of programmed cell death(EVA1A)
M_183	1813	dopamine receptor D2(DRD2)
M_183	128822	cystatin 9(CST9)
M_183	128821	cystatin 9-like(CST9L)
M_183	4651	myosin X(MYO10)
M_183	8309	acyl-CoA oxidase 2(ACOX2)
M_183	143666	uncharacterized LOC143666(LOC143666)
M_183	6641	syntrophin beta 1(SNTB1)
M_183	158376	long intergenic non-protein coding RNA
M_183	57156	transmembrane protein 63C(TMEM63C)
M_183	283209	phosphoglucomutase 2 like 1(PGM2L1)
M_183	83988	neurocalcin delta(NCALD)
M_183	3000	guanylate cyclase 2D, retinal(GUCY2D)
M_183	5730	prostaglandin D2 synthase(PTGDS)
M_183	5327	plasminogen activator, tissue type(PLAT)
M_183	100128285	dynamin 1 pseudogene 35(DNM1P35)
M_183	6327	sodium voltage-gated channel beta subunit 2(SCN2B)
M_183	196993	cancer/testis antigen 62(CT62)
M_183	1004	cadherin 6(CDH6)
M_183	51252	family with sequence similarity 178 member
M_183	2184	fumarylacetoacetate hydrolase(FAH)
M_183	646962	histidine rich carboxyl terminus 1(HRCT1)
M_183	729668	golgin A2 pseudogene 6(GOLGA2P6)
M_183	10863	ADAM metallopeptidase domain 28(ADAM28)
M_183	284111	solute carrier family 13 member 5(SLC13A5)
M_183	54088	long intergenic non-protein coding RNA
M_183	26471	nuclear protein 1, transcriptional regulator(NUPR1)
M_183	162417	N-acetylglutamate synthase(NAGS)
M_183	11170	family with sequence similarity 107 member
M_183	255488	ring finger protein 144B(RNF144B)
M_183	9215	LARGE xylosyl- and glucuronyltransferase

M_183	56656	olfactory receptor family 2 subfamily S member 2 (gene/pseudogene)(OR2S2)
M_183	8910	sarcoglycan epsilon(SGCE)
M_183	79937	contactin associated protein-like 3(CNTNAP3)
M_183	221692	phosphatase and actin regulator 1(PHACTR1)
M_183	100128327	trafficking protein particle complex 3
M_183	5507	protein phosphatase 1 regulatory subunit
M_183	10826	fatty acid hydroxylase domain containing 2(FAXDC2)
M_183	196415	chromosome 12 open reading frame 77(C12orf77)
M_183	50700	retinol dehydrogenase 8 (all-trans)(RDH8)
M_183	5972	renin(REN)
M_183	162517	F-box protein 39(FBXO39)
M_183	55809	transcriptional regulating factor 1(TRERF1)
M_183	142685	ankyrin repeat and SOCS box containing 15(ASB15)
M_183	137872	alcohol dehydrogenase, iron containing 1(ADHFE1)
M_183	158228	family with sequence similarity 201 member
M_183	84336	transmembrane protein 101(TMEM101)
M_183	353324	spermatogenesis associated 12(SPATA12)
M_183	124912	sperm acrosome associated 3(SPACA3)
M_183	29774	POM121 transmembrane nucleoporin like 9, pseudogene(POM121L9P)
M_183	140880	cystatin 11(CST11)
M_183	57471	ermin(ERMN)
M_183	7472	Wnt family member 2(WNT2)
M_183	23554	tetraspanin 12(TSPAN12)
M_183	10815	complexin 1(CPLX1)
M_183	1796	docking protein 1(DOK1)
M_183	90523	muscular LMNA interacting protein(MLIP)
M_183	8529	cytochrome P450 family 4 subfamily F member
M_183	8572	PDZ and LIM domain 4(PDLIM4)
M_183	50604	interleukin 20(IL20)
M_183	23089	paternally expressed 10(PEG10)

M_183	25812	POM121 transmembrane nucleoporin like 1, pseudogene(POM121L1P)
M_183	125875	claudin domain containing 2(CLDND2)
M_184	3821	killer cell lectin like receptor C1(KLRC1)
M_184	4261	class II major histocompatibility complex transactivator(CIITA)
M_184	129607	cytidine/uridine monophosphate kinase 2(CMPK2)
M_184	256586	LysM domain containing 2(LYSMD2)
M_184	972	CD74 molecule(CD74)
M_184	27074	lysosomal associated membrane protein 3(LAMP3)
M_184	8542	apolipoprotein L1(APOL1)
M_184	11274	ubiquitin specific peptidase 18(USP18)
M_184	23586	DExD/H-box helicase 58(DDX58)
M_184	6772	signal transducer and activator of transcription
M_184	51191	HECT and RLD domain containing E3 ubiquitin protein ligase 5(HERC5)
M_184	221472	FYVE, RhoGEF and PH domain containing 2(FGD2)
M_184	6398	secreted and transmembrane 1(SECTM1)
M_184	91543	radical S-adenosyl methionine domain containing
M_184	3119	major histocompatibility complex, class II, DQ beta 1(HLA-DQB1)
M_184	3117	major histocompatibility complex, class II, DQ alpha 1(HLA-DQA1)
M_184	3115	major histocompatibility complex, class II, DP beta 1(HLA-DPB1)
M_184	3112	major histocompatibility complex, class II, DO beta(HLA-DOB)
M_184	8519	interferon induced transmembrane protein 1(IFITM1)
M_184	5265	serpin family A member 1(SERPINA1)
M_184	3122	major histocompatibility complex, class II, DR alpha(HLA-DRA)

M_184	3123	major histocompatibility complex, class II, DR beta 1(HLA-DRB1)
M_184	55008	HECT and RLD domain containing E3 ubiquitin protein ligase family member 6(HERC6)
M_184	2537	interferon alpha inducible protein 6(IFI6)
M_184	55013	mitochondrial calcium uniporter dominant negative beta subunit(MCUB)
M_184	55601	DExD/H-box helicase 60(DDX60)
M_184	10410	interferon induced transmembrane protein 3(IFITM3)
M_184	5699	proteasome subunit beta 10(PSMB10)
M_184	3805	killer cell immunoglobulin like receptor, two Ig domains and long cytoplasmic tail 4(KIR2DL4)
M_184	3600	interleukin 15(IL15)
M_184	6846	X-C motif chemokine ligand 2(XCL2)
M_184	4067	LYN proto-oncogene, Src family tyrosine
M_184	23780	apolipoprotein L2(APOL2)
M_184	91351	DEAD-box helicase 60-like(DDX60L)
M_184	3113	major histocompatibility complex, class II, DP alpha 1(HLA-DPA1)
M_184	684	bone marrow stromal cell antigen 2(BST2)
M_184	4940	2'-5'-oligoadenylate synthetase 3(OAS3)
M_184	4939	2'-5'-oligoadenylate synthetase 2(OAS2)
M_184	4938	2'-5'-oligoadenylate synthetase 1(OAS1)
M_184	3429	interferon alpha inducible protein 27(IFI27)
M_184	10578	granulysin(GNLY)
M_184	3107	major histocompatibility complex, class I, C(HLA-C)
M_184	5610	eukaryotic translation initiation factor 2 alpha kinase 2(EIF2AK2)
M_184	3109	major histocompatibility complex, class II, DM beta(HLA-DMB)
M_184	567	beta-2-microglobulin(B2M)

M_184	3111	major histocompatibility complex, class II, DO alpha(HLA-DOA)
M_184	9636	ISG15 ubiquitin-like modifier(ISG15)
M_184	10581	interferon induced transmembrane protein 2(IFITM2)
M_184	5920	retinoic acid receptor responder 3(RARRES3)
M_184	8638	2'-5'-oligoadenylate synthetase like(OASL)
M_184	4599	MX dynamin like GTPase 1(MX1)
M_184	3108	major histocompatibility complex, class II, DM alpha(HLA-DMA)
M_184	3127	major histocompatibility complex, class II, DR beta 5(HLA-DRB5)
M_184	3601	interleukin 15 receptor subunit alpha(IL15RA)
M_184	10379	interferon regulatory factor 9(IRF9)
M_184	58472	sulfide quinone reductase-like (yeast)(SQRDL)
M_184	200315	apolipoprotein B mRNA editing enzyme catalytic subunit 3A(APOBEC3A)
M_184	4061	lymphocyte antigen 6 complex, locus E(LY6E)
M_184	100133669	uncharacterized LOC100133669(LOC100133669)
M_184	282618	interferon lambda 1(IFNL1)
M_184	3456	interferon beta 1(IFNB1)
M_185	400258	chromosome 14 open reading frame 180(C14orf180)
M_185	390892	olfactory receptor family 7 subfamily A member
M_185	114131	urocortin 3(UCN3)
M_185	810	calmodulin like 3(CALML3)
M_185	140832	WAP four-disulfide core domain 10A(WFDC10A)
M_185	80831	apolipoprotein L5(APOL5)
M_185	340811	aldo-keto reductase family 1 member C8, pseudogene(AKR1C8P)
M_185	63924	cell death inducing DFFA like effector c(CIDEC)
M_185	3991	lipase E, hormone sensitive type(LIPE)
M_185	127	alcohol dehydrogenase 4 (class II), pi
M_185	2819	glycerol-3-phosphate dehydrogenase 1(GPD1)

M_185	119548	pancreatic lipase related protein 3(PNLIPRP3)
M_185	2038	erythrocyte membrane protein band 4.2(EPB42)
M_185	26531	olfactory receptor family 11 subfamily A member
M_185	285189	plasminogen-like A (pseudogene)(PLGLA)
M_185	1109	aldo-keto reductase family 1 member C4(AKR1C4)
M_185	729359	perilipin 4(PLIN4)
M_185	117245	HRAS like suppressor family member 5(HRASLS5)
M_185	81309	olfactory receptor family 4 subfamily C member
M_185	1421	crystallin gamma D(CRYGD)
M_185	493901	ribonuclease A family member 12
M_185	64102	tenomodulin(TNMD)
M_185	1538	cylicin 1(CYLC1)
M_185	283768	golgin A8 family member G(GOLGA8G)
M_185	1646	aldo-keto reductase family 1 member C2(AKR1C2)
M_185	1645	aldo-keto reductase family 1 member C1(AKR1C1)
M_185	440956	IQ motif containing F6(IQCF6)
M_185	386677	keratin associated protein 10-1(KRTAP10-1)
M_185	10249	glycine-N-acyltransferase(GLYAT)
M_185	284827	keratin associated protein 13-4(KRTAP13-4)
M_185	389932	aldo-keto reductase family 1 member C6, pseudogene(AKR1C6P)
M_185	119694	olfactory receptor family 51 subfamily F member
M_185	364	aquaporin 7(AQP7)
M_185	390168	olfactory receptor family 5 subfamily M member
M_185	64232	membrane spanning 4-domains A5(MS4A5)
M_185	338675	olfactory receptor family 5 subfamily AP member
M_185	132141	IQ motif containing F1(IQCF1)
M_185	56246	melanocortin 2 receptor accessory protein(MRAP)
M_185	92552	ataxin 3 like(ATXN3L)
M_185	729786	golgin A8 family member C,
M_185	340547	V-set and immunoglobulin domain containing
M_185	390535	golgin A8 family member E,

M_185	388762	olfactory receptor family 2 subfamily M member 1 pseudogene(OR2M1P)
M_185	57495	NACHT and WD repeat domain containing 2(NWD2)
M_185	219982	olfactory receptor family 5 subfamily A member
M_185	729	complement C6(C6)
M_185	100132979	golgin A8 family member D,
M_185	8644	aldo-keto reductase family 1 member C3(AKR1C3)
M_185	79501	olfactory receptor family 4 subfamily F member
M_185	401994	olfactory receptor family 14 subfamily I member
M_185	56156	testis expressed 13B(TEX13B)
M_185	26682	olfactory receptor family 4 subfamily F member
M_185	145845	uncharacterized LOC145845(LOC145845)
M_185	441432	aquaporin 7 pseudogene 3(AQP7P3)
M_185	375719	aquaporin 7 pseudogene 1(AQP7P1)
M_185	440184	long intergenic non-protein coding RNA
M_185	100133920	methylenetetrahydrofolate dehydrogenase (NADP+ dependent) 1-like pseudogene(LOC100133920)
M_185	56547	matrix metallopeptidase 26(MMP26)
M_185	9075	claudin 2(CLDN2)
M_185	644168	dorsal root ganglia homeobox(DRGX)
M_185	4023	lipoprotein lipase(LPL)
M_185	440503	perilipin 5(PLIN5)
M_185	64109	cytokine receptor-like factor 2(CRLF2)
M_185	2515	ADAM metallopeptidase domain 2(ADAM2)
M_185	7079	TIMP metallopeptidase inhibitor 4(TIMP4)
M_185	4128	monoamine oxidase A(MAOA)
M_185	92129	ripply transcriptional repressor 1(RIPPLY1)
M_185	4212	Meis homeobox 2(MEIS2)
M_185	120237	developing brain homeobox 1(DBX1)
M_185	8622	phosphodiesterase 8B(PDE8B)
M_185	644139	phosphoinositide interacting regulator of transient receptor potential channels(PIRT)

M_185	56261	glycerophosphocholine phosphodiesterase
M_186	4254	KIT ligand(KITLG)
M_186	5764	pleiotrophin(PTN)
M_186	5796	protein tyrosine phosphatase, receptor type
M_186	158158	RAS and EF-hand domain containing(RASEF)
M_186	476	ATPase Na+/K+ transporting subunit alpha
M_186	116039	odd-skipped related transciption factor 2(OSR2)
M_186	284611	family with sequence similarity 102 member
M_186	5013	orthodenticle homeobox 1(OTX1)
M_186	4646	myosin VI(MYO6)
M_186	79191	iroquois homeobox 3(IRX3)
M_186	285045	long intergenic non-protein coding RNA
M_186	6323	sodium voltage-gated channel alpha subunit
M_186	81029	Wnt family member 5B(WNT5B)
M_186	5083	paired box 9(PAX9)
M_186	3777	potassium two pore domain channel subfamily K member 3(KCNK3)
M_186	4257	microsomal glutathione S-transferase 1(MGST1)
M_186	2296	forkhead box C1(FOXC1)
M_186	2059	epidermal growth factor receptor pathway substrate
M_186	100271832	uncharacterized LOC100271832(LOC100271832)
M_186	440097	developing brain homeobox 2(DBX2)
M_186	4052	latent transforming growth factor beta binding protein 1(LTBP1)
M_186	126969	solute carrier family 44 member 3(SLC44A3)
M_186	63973	neurogenin 2(NEUROG2)
M_186	283332	uncharacterized LOC283332(LOC283332)
M_186	5806	pentraxin 3(PTX3)
M_186	645323	long intergenic non-protein coding RNA
M_186	64065	PERP, TP53 apoptosis effector(PERP)
M_186	134957	syntaxin binding protein 5(STXBP5)
M_186	6374	C-X-C motif chemokine ligand 5(CXCL5)

M_186	85480	thymic stromal lymphopoietin(TSLP)
M_186	10265	iroquois homeobox 5(IRX5)
M_186	23017	Fas apoptotic inhibitory molecule 2(FAIM2)
M_186	1825	desmocollin 3(DSC3)
M_186	10370	Cbp/p300 interacting transactivator with Glu/Asp rich carboxy-terminal domain 2(CITED2)
M_186	158521	fragile X mental retardation 1 neighbor(FMR1NB)
M_186	116843	solute carrier family 18 member B1(SLC18B1)
M_186	9053	microtubule associated protein 7(MAP7)
M_186	5307	paired like homeodomain 1(PITX1)
M_186	5197	platelet factor 4 variant 1(PF4V1)
M_186	5080	paired box 6(PAX6)
M_186	3169	forkhead box A1(FOXA1)
M_186	57224	NHS like 1(NHSL1)
M_186	3110	motor neuron and pancreas homeobox 1(MNX1)
M_186	2020	engrailed homeobox 2(EN2)
M_186	2444	fyn related Src family tyrosine kinase(FRK)
M_186	5270	serpin family E member 2(SERPINE2)
M_186	3953	leptin receptor(LEPR)
M_186	54997	tescalcin(TESC)
M_186	130106	calcium and integrin binding family member 4(CIB4)
M_186	1440	colony stimulating factor 3(CSF3)
M_186	2334	AF4/FMR2 family member 2(AFF2)
M_186	85315	progestin and adipoQ receptor family member
M_186	627	brain derived neurotrophic factor(BDNF)
M_186	9783	regulating synaptic membrane exocytosis 3(RIMS3)
M_186	65059	Ras association (RalGDS/AF-6) and pleckstrin homology domains 1(RAPH1)
M_186	79190	iroquois homeobox 6(IRX6)
M_186	27345	potassium calcium-activated channel subfamily M regulatory beta subunit 4(KCNMB4)
M_186	29842	transcription factor CP2 like 1(TFCP2L1)

M_186	83886	protease, serine 27(PRSS27)
M_186	400746	non-compact myelin associated protein(NCMAP)
M_186	554226	ankyrin repeat domain 30B like(ANKRD30BL)
M_186	284613	cytochrome b561 family member D1(CYB561D1)
M_186	5019	3-oxoacid CoA-transferase 1(OXCT1)
M_186	109	adenylate cyclase 3(ADCY3)
M_187	478	ATPase Na+/K+ transporting subunit alpha
M_187	885	cholecystokinin(CCK)
M_187	1804	dipeptidyl peptidase like 6(DPP6)
M_187	255167	long intergenic non-protein coding RNA
M_187	4502	metallothionein 2A(MT2A)
M_187	56660	potassium two pore domain channel subfamily K member 12(KCNK12)
M_187	840	caspase 7(CASP7)
M_187	134147	carboxymethylenebutenolidase homolog(CMBL)
M_187	26468	LIM homeobox 6(LHX6)
M_187	5174	PDZ domain containing 1(PDZK1)
M_187	4843	nitric oxide synthase 2(NOS2)
M_187	114800	coiled-coil domain containing 85A(CCDC85A)
M_187	7274	alpha tocopherol transfer protein(TTPA)
M_187	2899	glutamate ionotropic receptor kainate type subunit
M_187	5621	prion protein(PRNP)
M_187	644314	metallothionein 1I, pseudogene(MT1IP)
M_187	54463	family with sequence similarity 134 member
M_187	11240	peptidyl arginine deiminase 2(PADI2)
M_187	23101	MCF.2 cell line derived transforming sequence-like 2(MCF2L2)
M_187	9228	DLG associated protein 2(DLGAP2)
M_187	8433	undifferentiated embryonic cell transcription factor
M_187	4942	ornithine aminotransferase(OAT)
M_187	6531	solute carrier family 6 member 3(SLC6A3)
M_187	127707	kelch domain containing 7A(KLHDC7A)

M_187	56000	nuclear RNA export factor 3(NXF3)
M_187	8435	sterol O-acyltransferase 2(SOAT2)
M_187	64129	tubulointerstitial nephritis antigen like 1(TINAGL1)
M_187	23043	TRAF2 and NCK interacting kinase(TNIK)
M_187	4500	metallothionein 1L (gene/pseudogene)(MT1L)
M_187	4501	metallothionein 1X(MT1X)
M_187	203427	solute carrier family 25 member 43(SLC25A43)
M_187	127943	Fc receptor like B(FCRLB)
M_187	28	ABO blood group (transferase A, alpha 1-3-N-acetylgalactosaminyltransferase; transferase B, alpha 1-3-galactosyltransferase)(ABO)
M_187	4489	metallothionein 1A(MT1A)
M_187	4493	metallothionein 1E(MT1E)
M_187	4494	metallothionein 1F(MT1F)
M_187	4495	metallothionein 1G(MT1G)
M_187	4496	metallothionein 1H(MT1H)
M_187	4499	metallothionein 1M(MT1M)
M_187	57863	cell adhesion molecule 3(CADM3)
M_187	1002	cadherin 4(CDH4)
M_187	374378	polypeptide N-acetylgalactosaminyltransferase
M_187	441376	alanine and arginine rich domain containing
M_187	4504	metallothionein 3(MT3)
M_187	8739	harakiri, BCL2 interacting protein(HRK)
M_187	9671	WSC domain containing 2(WSCD2)
M_187	85442	kinase non-catalytic C-lobe domain containing
M_187	7104	transmembrane 4 L six family member 4(TM4SF4)
M_187	326343	metallothionein 1D, pseudogene(MT1DP)
M_187	9531	BCL2 associated athanogene 3(BAG3)
M_187	64064	3-oxoacid CoA-transferase 2(OXCT2)
M_187	2700	gap junction protein alpha 3(GJA3)
M_187	6095	RAR related orphan receptor A(RORA)
M_187	5028	purinergic receptor P2Y1(P2RY1)

M_187	64377	carbohydrate sulfotransferase 8(CHST8)
M_187	51378	angiopoietin 4(ANGPT4)
M_187	80320	Sp6 transcription factor(SP6)
M_187	145258	goosecoid homeobox(GSC)
M_187	26010	spermatogenesis associated serine rich 2
M_187	2182	acyl-CoA synthetase long-chain family member
M_187	4010	LIM homeobox transcription factor 1 beta(LMX1B)
M_187	23544	seizure related 6 homolog like(SEZ6L)
M_187	84435	adhesion G protein-coupled receptor A1(ADGRA1)
M_187	4861	neuronal PAS domain protein 1(NPAS1)
M_187	3049	hemoglobin subunit theta 1(HBQ1)
M_187	6510	solute carrier family 1 member 5(SLC1A5)
M_187	2299	forkhead box I1(FOXI1)
M_187	3752	potassium voltage-gated channel subfamily D member 3(KCND3)
M_187	10867	tetraspanin 9(TSPAN9)
M_187	3166	H6 family homeobox 1(HMX1)
M_187	3745	potassium voltage-gated channel subfamily B member 1(KCNB1)
M_187	55351	serine/threonine kinase 32B(STK32B)
M_187	5337	phospholipase D1(PLD1)
M_187	4824	NK3 homeobox 1(NKX3-1)
M_187	80168	monoacylglycerol O-acyltransferase 2(MOGAT2)
M_187	116448	oligodendrocyte transcription factor 1(OLIG1)
M_187	145978	long intergenic non-protein coding RNA
M_187	79623	polypeptide N-acetylgalactosaminyltransferase
M_187	388121	TNF alpha induced protein 8 like 3(TNFAIP8L3)
M_187	79966	stearoyl-CoA desaturase 5(SCD5)
M_187	83698	calneuron 1(CALN1)
M_187	94015	tweety family member 2(TTYH2)
M_187	153	adrenoceptor beta 1(ADRB1)

M_187	2564	gamma-aminobutyric acid type A receptor epsilon subunit(GABRE)
M_187	92211	cadherin related family member 1(CDHR1)
M_187	5141	phosphodiesterase 4A(PDE4A)
M_187	80765	StAR related lipid transfer domain containing
M_187	10215	oligodendrocyte lineage transcription factor 2(OLIG2)
M_187	23228	phospholipase C like 2(PLCL2)
M_187	84970	chromosome 1 open reading frame 94(C1orf94)
M_187	1812	dopamine receptor D1(DRD1)
M_187	732253	testis development related 1 (non-protein
M_187	340745	leucine rich repeat, Ig-like and transmembrane domains 2(LRIT2)
M_187	114784	CUB and Sushi multiple domains 2(CSMD2)
M_187	9365	klotho(KL)
M_187	3775	potassium two pore domain channel subfamily K member 1(KCNK1)
M_188	116154	phosphatase and actin regulator 3(PHACTR3)
M_188	3848	keratin 1(KRT1)
M_188	146760	reticulon 4 receptor like 1(RTN4RL1)
M_188	55200	pleckstrin homology and RhoGEF domain containing G6(PLEKHG6)
M_188	6926	T-box 3(TBX3)
M_188	340120	ankyrin repeat domain 34B(ANKRD34B)
M_188	2852	G protein-coupled estrogen receptor 1(GPER1)
M_188	285126	DnaJ heat shock protein family (Hsp40) member C5 gamma(DNAJC5G)
M_188	92304	secretoglobin family 3A member 1(SCGB3A1)
M_188	90019	synaptotagmin 8(SYT8)
M_188	79442	leucine rich repeat containing 2(LRRC2)
M_188	128272	Rho guanine nucleotide exchange factor
M_188	51761	ATPase phospholipid transporting 8A2(ATP8A2)
M_188	93164	5-hydroxytryptamine receptor 7 pseudogene

M_188	9066	synaptotagmin 7(SYT7)
M_188	254910	late cornified envelope 5A(LCE5A)
M_188	375791	cysteine rich tail 1(CYSRT1)
M_188	57586	synaptotagmin 13(SYT13)
M_188	887	cholecystokinin B receptor(CCKBR)
M_188	283358	beta-1,4-N-acetyl-galactosaminyltransferase
M_188	342897	non-specific cytotoxic cell receptor protein 1 homolog (zebrafish)(NCCRP1)
M_188	124976	sphingolipid transporter 2(SPNS2)
M_188	1947	ephrin B1(EFNB1)
M_188	49860	cornulin(CRNN)
M_188	79152	fatty acid 2-hydroxylase(FA2H)
M_188	27237	Rho guanine nucleotide exchange factor
M_188	57822	grainyhead like transcription factor 3(GRHL3)
M_188	79919	chromosome 2 open reading frame 54(C2orf54)
M_188	2304	forkhead box E1(FOXE1)
M_188	388697	hornerin(HRNR)
M_188	7136	troponin I2, fast skeletal type(TNNI2)
M_188	7781	solute carrier family 30 member 3(SLC30A3)
M_188	9537	tumor protein p53 inducible protein 11(TP53I11)
M_188	2312	filaggrin(FLG)
M_188	6616	synaptosome associated protein 25(SNAP25)
M_188	8911	calcium voltage-gated channel subunit alpha1
M_188	7062	trichohyalin(TCHH)
M_188	55057	absent in melanoma 1-like(AIM1L)
M_188	3713	involucrin(IVL)
M_188	7054	tyrosine hydroxylase(TH)
M_188	388698	filaggrin family member 2(FLG2)
M_188	23467	neuronal pentraxin receptor(NPTXR)
M_188	2562	gamma-aminobutyric acid type A receptor beta3 subunit(GABRB3)
M_188	5818	nectin cell adhesion molecule 1(NECTIN1)

M_188	55964	septin 3(SEPT3)
M_188	10675	chondroitin sulfate proteoglycan 5(CSPG5)
M_188	3625	inhibin beta B subunit(INHBB)
M_188	1816	dopamine receptor D5(DRD5)
M_188	108	adenylate cyclase 2(ADCY2)
M_188	202559	KH RNA binding domain containing, signal transduction associated 2(KHDRBS2)
M_189	401508	family with sequence similarity 74 member
M_189	403244	olfactory receptor family 2 subfamily T member
M_189	138240	chromosome 9 open reading frame 57(C9orf57)
M_189	140856	SCP2 sterol binding domain containing 1(SCP2D1)
M_189	613209	defensin beta 135(DEFB135)
M_189	286094	long intergenic non-protein coding RNA
M_189	5161	pyruvate dehydrogenase (lipoamide) alpha 2(PDHA2)
M_189	136371	ankyrin repeat and SOCS box containing 10(ASB10)
M_189	5458	POU class 4 homeobox 2(POU4F2)
M_189	26707	olfactory receptor family 2 subfamily J member
M_189	136541	protease, serine 58(PRSS58)
M_189	54658	UDP glucuronosyltransferase family 1 member
M_189	162540	signal peptide peptidase like 2C(SPPL2C)
M_189	285692	uncharacterized LOC285692(LOC285692)
M_189	353238	peptidyl arginine deiminase 6(PADI6)
M_189	100124535	small nucleolar RNA, H/ACA box 36C(SNORA36C)
M_189	4593	muscle associated receptor tyrosine kinase(MUSK)
M_189	219956	olfactory receptor family 9 subfamily Q member
M_189	346525	olfactory receptor family 2 subfamily A member
M_189	149830	prion protein (testis specific)(PRNT)
M_189	117196	MAS related GPR family member X4(MRGPRX4)
M_189	254272	TBC1 domain family member 28(TBC1D28)
M_189	134288	transmembrane protein 174(TMEM174)
M_189	138799	olfactory receptor family 13 subfamily C member

M_189	5568	protein kinase cAMP-activated catalytic subunit gamma(PRKACG)
M_189	2692	growth hormone releasing hormone receptor(GHRHR)
M_189	219990	oocyte secreted protein 2(OOSP2)
M_189	79544	olfactory receptor family 4 subfamily K member
M_189	340441	POTE ankyrin domain family member A(POTEA)
M_189	286135	family with sequence similarity 183 member C, pseudogene(FAM183CP)
M_189	692053	small nucleolar RNA, C/D box 9(SNORD9)
M_189	128368	olfactory receptor family 10 subfamily Z member
M_189	219983	olfactory receptor family 4 subfamily D member
M_189	10369	calcium voltage-gated channel auxiliary subunit gamma 2(CACNG2)
M_189	338588	long intergenic non-protein coding RNA
M_189	5969	regenerating family member 1 gamma,
M_189	219959	olfactory receptor family 1 subfamily S member 1 (gene/pseudogene)(OR1S1)
M_189	119765	olfactory receptor family 4 subfamily B member
M_189	403253	olfactory receptor family 4 subfamily A member
M_189	22829	neuroligin 4, Y-linked(NLGN4Y)
M_189	403273	olfactory receptor family 5 subfamily H member
M_189	50835	taste 2 receptor member 9(TAS2R9)
M_189	50834	taste 2 receptor member 1(TAS2R1)
M_189	64111	neuropeptide VF precursor(NPVF)
M_189	50837	taste 2 receptor member 7(TAS2R7)
M_189	390154	olfactory receptor family 5 subfamily T member
M_189	646424	serine peptidase inhibitor, Kazal type 8
M_189	390113	olfactory receptor family 4 subfamily X member 1 (gene/pseudogene)(OR4X1)
M_189	126205	NLR family pyrin domain containing 8(NLRP8)
M_189	245938	defensin beta 125(DEFB125)
M_189	93432	maltase-glucoamylase 2 (putative)(MGAM2)

M_189	84666	resistin like beta(RETNLB)
M_189	6514	solute carrier family 2 member 2(SLC2A2)
M_189	388007	serpin family A member 13,
M_189	135948	olfactory receptor family 2 subfamily F member
M_189	388649	chromosome 1 open reading frame 146(C1orf146)
M_189	441581	FSHD region gene 2 family member B(FRG2B)
M_189	100124692	#N/A
M_189	448831	FSHD region gene 2(FRG2)
M_189	93426	synaptonemal complex central element protein
M_189	143471	proteasome subunit alpha 8(PSMA8)
M_189	644145	exocyst complex component 1
M_189	122042	relaxin/insulin like family peptide receptor 2(RXFP2)
M_189	100128977	MAPT antisense RNA 1(MAPT-AS1)
M_189	1584	cytochrome P450 family 11 subfamily B member 1(CYP11B1)
M_189	284369	SIGLEC family like 1(SIGLECL1)
M_189	51156	serpin family A member 10(SERPINA10)
M_189	5995	retinal G protein coupled receptor(RGR)
M_189	390226	guanylate cyclase 2E, pseudogene(GUCY2EP)
M_189	392391	olfactory receptor family 5 subfamily C member
M_189	57718	protein phosphatase 4 regulatory subunit 4(PPP4R4)
M_189	360030	NANOG neighbor homeobox(NANOGNB)
M_189	653399	glutathione S-transferase theta pseudogene
M_189	8972	maltase-glucoamylase(MGAM)
M_189	343930	mesogenin 1(MSGN1)
M_189	26103	leucine rich repeat, Ig-like and transmembrane domains 1(LRIT1)
M_189	391322	D-dopachrome tautomerase-like(LOC391322)
M_189	359787	developmental pluripotency associated 3(DPPA3)
M_189	130075	olfactory receptor family 9 subfamily A member
M_189	246744	saitohin(STH)

M_189	64816	cytochrome P450 family 3 subfamily A member 43(CYP3A43)
M_189	2952	glutathione S-transferase theta 1(GSTT1)
M_190	115294	protein-L-isoaspartate (D-aspartate) O-methyltransferase domain containing 1(PCMTD1)
M_190	79026	AHNAK nucleoprotein(AHNAK)
M_190	4209	myocyte enhancer factor 2D(MEF2D)
M_190	79901	cytochrome b reductase 1(CYBRD1)
M_190	161176	spectrin repeat containing nuclear envelope family member 3(SYNE3)
M_190	220001	von Willebrand factor C and EGF domains(VWCE)
M_190	2117	ETS variant 3(ETV3)
M_190	80821	DDHD domain containing 1(DDHD1)
M_190	339761	cytochrome P450 family 27 subfamily C member 1(CYP27C1)
M_190	79686	long intergenic non-protein coding RNA
M_190	56606	solute carrier family 2 member 9(SLC2A9)
M_190	2316	filamin A(FLNA)
M_190	7145	tensin 1(TNS1)
M_190	23002	dishevelled associated activator of morphogenesis
M_190	5549	proline and arginine rich end leucine rich repeat protein(PRELP)
M_190	395	Rho GTPase activating protein 6(ARHGAP6)
M_190	51232	cysteine rich transmembrane BMP regulator
M_190	375790	agrin(AGRN)
M_190	25818	kallikrein related peptidase 5(KLK5)
M_190	2355	FOS like 2, AP-1 transcription factor subunit(FOSL2)
M_190	220108	family with sequence similarity 124 member
M_190	205428	chromosome 3 open reading frame 58(C3orf58)
M_190	124739	ubiquitin specific peptidase 43(USP43)
M_190	115265	DNA damage inducible transcript 4 like(DDIT4L)
M_190	3983	actin binding LIM protein 1(ABLIM1)

M_190	10129	FRY microtubule binding protein(FRY)
M_190	6464	SHC adaptor protein 1(SHC1)
M_190	59352	leucine rich repeat containing G protein-coupled receptor 6(LGR6)
M_190	64759	tensin 3(TNS3)
M_190	11346	synaptopodin(SYNPO)
M_190	2331	fibromodulin(FMOD)
M_190	9423	netrin 1(NTN1)
M_190	2274	four and a half LIM domains 2(FHL2)
M_190	57088	phospholipid scramblase 4(PLSCR4)
M_190	54873	palmdelphin(PALMD)
M_190	26137	zinc finger and BTB domain containing 20(ZBTB20)
M_190	57458	transmembrane and coiled-coil domain family
M_190	117583	par-3 family cell polarity regulator beta(PARD3B)
M_190	4606	myosin binding protein C, fast type(MYBPC2)
M_190	494470	ring finger protein 165(RNF165)
M_190	59277	netrin 4(NTN4)
M_190	22925	phospholipase A2 receptor 1(PLA2R1)
M_190	4784	nuclear factor I X(NFIX)
M_190	57664	pleckstrin homology domain containing
M_190	22821	RAS p21 protein activator 3(RASA3)
M_191	4299	AF4/FMR2 family member 1(AFF1)
M_191	5530	protein phosphatase 3 catalytic subunit
M_191	84898	plexin domain containing 2(PLXDC2)
M_191	10611	PDZ and LIM domain 5(PDLIM5)
M_191	7325	ubiquitin conjugating enzyme E2 E2(UBE2E2)
M_191	9871	SEC24 homolog D, COPII coat complex
M_191	81849	ST6 N-acetylgalactosaminide alpha-2,6-sialyltransferase 5(ST6GALNAC5)
M_191	166815	tigger transposable element derived 2(TIGD2)
M_191	710	serpin family G member 1(SERPING1)
M_191	10746	mitogen-activated protein kinase kinase kinase

M_191	10144	family with sequence similarity 13 member
M_191	79705	leucine rich repeat kinase 1(LRRK1)
M_191	27185	disrupted in schizophrenia 1(DISC1)
M_191	152559	progestin and adipoQ receptor family member
M_191	2977	guanylate cyclase 1 soluble subunit alpha
M_191	51302	cytochrome P450 family 39 subfamily A member 1(CYP39A1)
M_191	79658	Rho GTPase activating protein 10(ARHGAP10)
M_191	404201	WDFY3 antisense RNA 2(WDFY3-AS2)
M_191	306	annexin A3(ANXA3)
M_191	2934	gelsolin(GSN)
M_191	92689	family with sequence similarity 114 member
M_191	1040	CDP-diacylglycerol synthase 1(CDS1)
M_191	132660	lin-54 DREAM MuvB core complex
M_191	4306	nuclear receptor subfamily 3 group C member
M_191	124975	gamma-glutamyltransferase 6(GGT6)
M_191	5218	cyclin dependent kinase 14(CDK14)
M_191	8916	HECT and RLD domain containing E3 ubiquitin protein ligase 3(HERC3)
M_191	54790	tet methylcytosine dioxygenase 2(TET2)
M_191	3426	complement factor I(CFI)
M_191	5311	polycystin 2, transient receptor potential cation
M_191	685	betacellulin(BTC)
M_191	153020	RasGEF domain family member 1B(RASGEF1B)
M_191	7424	vascular endothelial growth factor C(VEGFC)
M_191	135228	CD109 molecule(CD109)
M_191	143098	membrane palmitoylated protein 7(MPP7)
M_191	57563	kelch like family member 8(KLHL8)
M_191	55041	pleckstrin homology domain containing
M_191	23001	WD repeat and FYVE domain containing 3(WDFY3)
M_191	26503	solute carrier family 17 member 5(SLC17A5)
M_191	22872	SEC31 homolog A, COPII coat complex

M_191	374	amphiregulin(AREG)
M_191	2069	epiregulin(EREG)
M_191	79953	synapse differentiation inducing 1(SYNDIG1)
M_191	25840	methyltransferase like 7A(METTL7A)
M_191	83857	transmembrane and tetratricopeptide repeat containing 1(TMTC1)
M_191	140836	barrier to autointegration factor 2(BANF2)
M_191	23072	HECT, C2 and WW domain containing E3 ubiquitin protein ligase 1(HECW1)
M_191	341350	ovochymase 1(OVCH1)
M_192	4352	MPL proto-oncogene, thrombopoietin receptor(MPL)
M_192	340156	myosin light chain kinase family member 4(MYLK4)
M_192	1663	DEAD/H-box helicase 11(DDX11)
M_192	84874	zinc finger protein 514(ZNF514)
M_192	253769	WD repeat domain 27(WDR27)
M_192	375248	ankyrin repeat domain 36(ANKRD36)
M_192	728404	#N/A
M_192	677821	small nucleolar RNA, H/ACA box 71E(SNORA71E)
M_192	100129482	zinc finger protein 37B, pseudogene(ZNF37BP)
M_192	10120	ARP1 actin-related protein 1 homolog B, centractin beta(ACTR1B)
M_192	94104	PAX3 and PAX7 binding protein 1(PAXBP1)
M_192	285033	STARD7 antisense RNA 1(STARD7-AS1)
M_192	10748	killer cell lectin like receptor A1,
M_192	64969	mitochondrial ribosomal protein S5(MRPS5)
M_192	9866	tripartite motif containing 66(TRIM66)
M_192	26118	WD repeat and SOCS box containing 1(WSB1)
M_192	26152	zinc finger protein 337(ZNF337)
M_192	57730	ankyrin repeat domain 36B(ANKRD36B)
M_192	407975	miR-17-92a-1 cluster host gene(MIR17HG)
M_192	51455	REV1, DNA directed polymerase(REV1)
M_192	642826	BMS1, ribosome biogenesis factor pseudogene

M_192	79031	phosducin like 3(PDCL3)
M_192	285359	phosducin-like 3 pseudogene 4(PDCL3P4)
M_192	283314	C1RL antisense RNA 1(C1RL-AS1)
M_192	642846	DEAD/H (Asp-Glu-Ala-Asp/His) box polypeptide 11-like(LOC642846)
M_192	55096	endogenous Bornavirus-like nucleoprotein 2(EBLN2)
M_192	51011	fumarylacetoacetate hydrolase domain containing 2A(FAHD2A)
M_192	440081	DEAD/H-box helicase 12, pseudogene(DDX12P)
M_192	200539	ankyrin repeat domain 23(ANKRD23)
M_192	63908	NSF attachment protein beta(NAPB)
M_192	65981	caprin family member 2(CAPRIN2)
M_192	6991	t-complex-associated-testis-expressed 3(TCTE3)
M_192	51332	spectrin beta, non-erythrocytic 5(SPTBN5)
M_192	51601	lipoyltransferase 1(LIPT1)
M_192	55683	KAT8 regulatory NSL complex subunit 3(KANSL3)
M_192	9391	cytosolic iron-sulfur assembly component 1(CIAO1)
M_192	7549	zinc finger protein 2(ZNF2)
M_192	399761	BMS1, ribosome biogenesis factor pseudogene
M_192	51263	mitochondrial ribosomal protein L30(MRPL30)
M_192	150590	chromosome 2 open reading frame 15(C2orf15)
M_192	26505	cyclin and CBS domain divalent metal cation transport mediator 3(CNNM3)
M_192	54910	semaphorin 4C(SEMA4C)
M_192	81562	lectin, mannose binding 2 like(LMAN2L)
M_192	80705	testis specific 10(TSGA10)
M_192	50840	taste 2 receptor member 14(TAS2R14)
M_192	64900	lipin 3(LPIN3)
M_192	5555	proline rich protein HaeIII subfamily 2(PRH2)
M_192	7587	zinc finger protein 37A(ZNF37A)
M_192	89848	FCH and double SH3 domains 1(FCHSD1)
M_192	340595	zinc finger CCHC-type containing 16(ZCCHC16)

M_192	729096	BMS1, ribosome biogenesis factor pseudogene
M_192	221060	chromosome 10 open reading frame 111(C10orf111)
M_193	79366	high mobility group nucleosome binding domain
M_193	7106	tetraspanin 4(TSPAN4)
M_193	3815	KIT proto-oncogene receptor tyrosine kinase(KIT)
M_193	339453	transmembrane protein 240(TMEM240)
M_193	84552	par-6 family cell polarity regulator gamma(PARD6G)
M_193	4081	mab-21 like 1(MAB21L1)
M_193	26022	transmembrane protein 98(TMEM98)
M_193	84871	ATP/GTP binding protein like 4(AGBL4)
M_193	29119	catenin alpha 3(CTNNA3)
M_193	23380	SLIT-ROBO Rho GTPase activating protein
M_193	63035	BCL6 corepressor-like 1(BCORL1)
M_193	79070	KDEL motif containing 1(KDELC1)
M_193	84056	katanin catalytic subunit A1 like 1(KATNAL1)
M_193	158866	zinc finger DHHC-type containing 15(ZDHHC15)
M_193	158880	ubiquitin specific peptidase 51(USP51)
M_193	139599	MAGE family member E2(MAGEE2)
M_193	114928	G protein-coupled receptor associated sorting protein 2(GPRASP2)
M_193	286097	mitochondrial calcium uptake family member
M_193	5179	proenkephalin(PENK)
M_193	79057	proline rich and Gla domain 3(PRRG3)
M_193	79656	BEN domain containing 5(BEND5)
M_193	6451	SH3 domain binding glutamate rich protein
M_193	3742	potassium voltage-gated channel subfamily A member 6(KCNA6)
M_193	10325	Ras related GTP binding B(RRAGB)
M_193	5063	p21 (RAC1) activated kinase 3(PAK3)
M_193	4675	nucleosome assembly protein 1 like 3(NAP1L3)
M_193	4674	nucleosome assembly protein 1 like 2(NAP1L2)
M_193	645996	nucleosome assembly protein 1 like 6(NAP1L6)

M_193	91775	neurexophilin and PC-esterase domain family member 3(NXPE3)
M_193	6594	SWI/SNF related, matrix associated, actin dependent regulator of chromatin, subfamily a, member
M_193	4857	NOVA alternative splicing regulator 1(NOVA1)
M_193	26999	cytoplasmic FMR1 interacting protein 2(CYFIP2)
M_193	23708	G1 to S phase transition 2(GSPT2)
M_193	140886	poly(A) binding protein cytoplasmic 5(PABPC5)
M_193	157773	chromosome 8 open reading frame 48(C8orf48)
M_193	1740	discs large MAGUK scaffold protein 2(DLG2)
M_193	22873	DAZ interacting zinc finger protein 1(DZIP1)
M_193	827	calpain 6(CAPN6)
M_193	9227	lecithin retinol acyltransferase (phosphatidylcholine--retinol O-acyltransferase)(LRAT)
M_193	26960	neurobeachin(NBEA)
M_193	51201	zinc finger DHHC-type containing 2(ZDHHC2)
M_193	26290	polypeptide N-acetylgalactosaminyltransferase
M_193	322	amyloid beta precursor protein binding family B member 1(APBB1)
M_193	340542	brain expressed X-linked 5(BEX5)
M_193	340533	KIAA2022(KIAA2022)
M_193	55879	gamma-aminobutyric acid type A receptor theta subunit(GABRQ)
M_193	91608	RAS like family 10 member B(RASL10B)
M_193	7991	tumor suppressor candidate 3(TUSC3)
M_193	28986	MAGE family member H1(MAGEH1)
M_193	26278	sacsin molecular chaperone(SACS)
M_193	7552	zinc finger protein 711(ZNF711)
M_193	9542	neuregulin 2(NRG2)
M_193	5255	phosphorylase kinase regulatory subunit alpha
M_193	761	carbonic anhydrase 3(CA3)
M_193	64860	armadillo repeat containing, X-linked 5(ARMCX5)

M_193	377677	carbonic anhydrase 13(CA13)
M_193	22924	microtubule associated protein RP/EB family member 3(MAPRE3)
M_193	51209	RAB9B, member RAS oncogene family(RAB9B)
M_193	57692	MAGE family member E1(MAGEE1)
M_193	10916	MAGE family member D2(MAGED2)
M_193	7220	transient receptor potential cation channel subfamily C member 1(TRPC1)
M_193	80823	basic helix-loop-helix domain containing, class B,
M_193	79864	chromosome 11 open reading frame 63(C11orf63)
M_193	308	annexin A5(ANXA5)
M_193	79822	Rho GTPase activating protein 28(ARHGAP28)
M_193	116442	RAB39B, member RAS oncogene family(RAB39B)
M_193	83478	Rho GTPase activating protein 24(ARHGAP24)
M_193	781	calcium voltage-gated channel auxiliary subunit alpha2delta 1(CACNA2D1)
M_193	9500	MAGE family member D1(MAGED1)
M_193	2898	glutamate ionotropic receptor kainate type subunit
M_193	283455	kinase suppressor of ras 2(KSR2)
M_193	29800	zinc finger DHHC-type containing 1(ZDHHC1)
M_193	257068	phosphatidylinositol specific phospholipase C X domain containing 2(PLCXD2)
M_193	78991	prenylcysteine oxidase 1 like(PCYOX1L)
M_193	56899	ankyrin repeat and sterile alpha motif domain containing 1B(ANKS1B)
M_193	9201	doublecortin like kinase 1(DCLK1)
M_193	112464	protein kinase C delta binding protein(PRKCDBP)
M_193	6456	SH3 domain containing GRB2 like 2, endophilin
M_193	139411	patched domain containing 1(PTCHD1)
M_193	23194	F-box and leucine rich repeat protein 7(FBXL7)
M_193	29114	transgelin 3(TAGLN3)
M_193	5217	profilin 2(PFN2)

M_193	84969	TOX high mobility group box family member
M_193	54510	protocadherin 18(PCDH18)
M_193	140290	t-complex 10-like(TCP10L)
M_193	120114	FAT atypical cadherin 3(FAT3)
M_193	22866	connector enhancer of kinase suppressor of Ras
M_193	8522	growth arrest specific 7(GAS7)
M_193	23255	microtubule crosslinking factor 1(MTCL1)
M_193	871	serpin family H member 1(SERPINH1)
M_193	83873	G protein-coupled receptor 61(GPR61)
M_193	22982	disco interacting protein 2 homolog C(DIP2C)
M_193	5137	phosphodiesterase 1C(PDE1C)
M_193	9899	synaptic vesicle glycoprotein 2B(SV2B)
M_194	23302	WSC domain containing 1(WSCD1)
M_194	10220	growth differentiation factor 11(GDF11)
M_194	8074	fibroblast growth factor 23(FGF23)
M_194	146664	mannosyl (alpha-1,6-)-glycoprotein beta-1,6-N-acetyl-glucosaminyltransferase, isozyme B(MGAT5B)
M_194	171019	ADAM metallopeptidase with thrombospondin type 1 motif 19(ADAMTS19)
M_194	284069	family with sequence similarity 171 member
M_194	25789	transmembrane protein 59 like(TMEM59L)
M_194	3674	integrin subunit alpha 2b(ITGA2B)
M_194	54360	cytokine like 1(CYTL1)
M_194	6297	spalt like transcription factor 2(SALL2)
M_194	6299	spalt like transcription factor 1(SALL1)
M_194	81618	integral membrane protein 2C(ITM2C)
M_194	9244	cytokine receptor like factor 1(CRLF1)
M_194	168002	dishevelled binding antagonist of beta catenin
M_194	10562	olfactomedin 4(OLFM4)
M_194	8997	kalirin, RhoGEF kinase(KALRN)
M_194	4440	musashi RNA binding protein 1(MSI1)
M_194	9271	piwi like RNA-mediated gene silencing 1(PIWIL1)

M_194	10500	semaphorin 6C(SEMA6C)
M_194	25841	ankyrin repeat and BTB domain containing 2(ABTB2)
M_194	388228	SH3 domain binding kinase 1(SBK1)
M_194	149461	claudin 19(CLDN19)
M_194	116113	forkhead box P4(FOXP4)
M_194	375057	stum, mechanosensory transduction mediator
M_194	5738	prostaglandin F2 receptor inhibitor(PTGFRN)
M_194	11061	leukocyte cell derived chemotaxin 1(LECT1)
M_194	114299	paralemmin 2(PALM2)
M_194	111	adenylate cyclase 5(ADCY5)
M_194	4917	netrin 3(NTN3)
M_194	222663	signal peptide, CUB domain and EGF like domain containing 3(SCUBE3)
M_194	5100	protocadherin 8(PCDH8)
M_194	845	calsequestrin 2(CASQ2)
M_194	23316	cut like homeobox 2(CUX2)
M_194	8936	WAS protein family member 1(WASF1)
M_194	5076	paired box 2(PAX2)
M_194	3321	immunoglobulin superfamily member 3(IGSF3)
M_194	341405	ankyrin repeat domain 33(ANKRD33)
M_194	1001	cadherin 3(CDH3)
M_194	54756	interleukin 17 receptor D(IL17RD)
M_194	2254	fibroblast growth factor 9(FGF9)
M_194	11211	frizzled class receptor 10(FZD10)
M_194	2642	glucagon receptor(GCGR)
M_194	4062	lymphocyte antigen 6 complex, locus H(LY6H)
M_194	8200	growth differentiation factor 5(GDF5)
M_194	9398	CD101 molecule(CD101)
M_195	222171	proline rich 15(PRR15)
M_195	165215	family with sequence similarity 171 member
M_195	642587	MIR205 host gene(MIR205HG)
M_195	171177	ras homolog family member V(RHOV)

M_195	440073	IQ motif and Sec7 domain 3(IQSEC3)
M_195	347527	arylsulfatase family member H(ARSH)
M_195	79755	zinc finger protein 750(ZNF750)
M_195	2835	G protein-coupled receptor 12(GPR12)
M_195	30811	hormonally up-regulated Neu-associated
M_195	129804	fibulin 7(FBLN7)
M_195	119395	calcium homeostasis modulator 3(CALHM3)
M_195	1381	cellular retinoic acid binding protein 1(CRABP1)
M_195	26525	interleukin 36 receptor antagonist(IL36RN)
M_195	1672	defensin beta 1(DEFB1)
M_195	7022	transcription factor AP-2 gamma(TFAP2C)
M_195	8000	prostate stem cell antigen(PSCA)
M_195	1382	cellular retinoic acid binding protein 2(CRABP2)
M_195	64423	inverted formin, FH2 and WH2 domain
M_195	55313	calcineurin like phosphoesterase domain containing 1(CPPED1)
M_195	54923	Lck interacting transmembrane adaptor 1(LIME1)
M_195	376267	RAB15, member RAS oncogene family(RAB15)
M_195	3868	keratin 16(KRT16)
M_195	50617	ATPase H+ transporting V0 subunit a4(ATP6V0A4)
M_195	2013	epithelial membrane protein 2(EMP2)
M_195	117283	inositol hexakisphosphate kinase 3(IP6K3)
M_195	54492	neuralized E3 ubiquitin protein ligase 1B(NEURL1B)
M_195	1759	dynamin 1(DNM1)
M_195	3861	keratin 14(KRT14)
M_195	1953	multiple EGF like domains 6(MEGF6)
M_195	5603	mitogen-activated protein kinase 13(MAPK13)
M_195	777	calcium voltage-gated channel subunit alpha1
M_195	79153	glycerophosphodiester phosphodiesterase domain containing 3(GDPD3)
M_195	84000	transmembrane protease, serine 13(TMPRSS13)
M_195	23589	calcium regulated heat stable protein 1(CARHSP1)

M_195	51063	calcium homeostasis modulator 2(CALHM2)
M_195	84830	androgen dependent TFPI regulating protein(ADTRP)
M_195	51806	calmodulin like 5(CALML5)
M_195	6439	surfactant protein B(SFTPB)
M_195	6604	SWI/SNF related, matrix associated, actin dependent regulator of chromatin, subfamily d, member
M_195	4151	myoglobin(MB)
M_195	123803	N-terminal asparagine amidase(NTAN1)
M_195	2049	EPH receptor B3(EPHB3)
M_195	9048	artemin(ARTN)
M_195	8110	double PHD fingers 3(DPF3)
M_195	100130449	uncharacterized LOC100130449(PP14571)
M_195	8503	phosphoinositide-3-kinase regulatory subunit
M_195	5979	ret proto-oncogene(RET)
M_195	4682	nucleotide binding protein 1(NUBP1)
M_195	9516	lipopolysaccharide induced TNF factor(LITAF)
M_195	155006	transmembrane protein 213(TMEM213)
M_195	26156	ribosomal L1 domain containing 1(RSL1D1)
M_195	1305	collagen type XIII alpha 1 chain(COL13A1)
M_195	25984	keratin 23(KRT23)
M_195	7356	secretoglobin family 1A member 1(SCGB1A1)
M_195	245936	defensin beta 123(DEFB123)
M_195	5914	retinoic acid receptor alpha(RARA)
M_195	8322	frizzled class receptor 4(FZD4)
M_195	285335	solute carrier family 9 member C1(SLC9C1)
M_195	5304	prolactin induced protein(PIP)
M_195	27289	Rho family GTPase 1(RND1)
M_195	11098	protease, serine 23(PRSS23)
M_195	24147	four jointed box 1(FJX1)
M_195	10873	malic enzyme 3(ME3)
M_195	338596	ST8 alpha-N-acetyl-neuraminide alpha-2,8-sialyltransferase 6(ST8SIA6)

M_195	239	arachidonate 12-lipoxygenase, 12S type(ALOX12)
M_195	93082	neuralized E3 ubiquitin protein ligase 3(NEURL3)
M_195	9381	otoferlin(OTOF)
M_195	389384	chromosome 6 open reading frame 222(C6orf222)
M_195	84283	transmembrane protein 79(TMEM79)
M_195	6530	solute carrier family 6 member 2(SLC6A2)
M_195	2324	fms related tyrosine kinase 4(FLT4)
M_195	255349	transmembrane protein 211(TMEM211)
M_195	51561	interleukin 23 subunit alpha(IL23A)
M_195	6405	semaphorin 3F(SEMA3F)
M_195	149428	BCL2 interacting protein like(BNIPL)
M_195	27201	G protein-coupled receptor 78(GPR78)
M_195	1594	cytochrome P450 family 27 subfamily B member 1(CYP27B1)
M_195	2779	G protein subunit alpha transducin 1(GNAT1)
M_196	6295	S-antigen visual arrestin(SAG)
M_196	388333	speedy/RINGO cell cycle regulator family member E4(SPDYE4)
M_196	29124	galectin 13(LGALS13)
M_196	441911	olfactory receptor family 10 subfamily J member
M_196	650293	seven transmembrane helix receptor(LOC650293)
M_196	168391	polypeptide N-acetylgalactosaminyltransferase-like 5(GALNTL5)
M_196	338917	visual system homeobox 2(VSX2)
M_196	6656	SRY-box 1(SOX1)
M_196	100133121	family with sequence similarity 27 member
M_196	54089	long intergenic non-protein coding RNA
M_196	677795	small nucleolar RNA, H/ACA box 5B(SNORA5B)
M_196	283238	solute carrier family 22 member 24(SLC22A24)
M_196	200159	chromosome 1 open reading frame 100(C1orf100)
M_196	7142	transition protein 2(TNP2)
M_196	5620	protamine 2(PRM2)

M_196	3452	interferon alpha 21(IFNA21)
M_196	93408	myosin light chain 10(MYL10)
M_196	253582	transmembrane protein 244(TMEM244)
M_196	137362	glutamic-oxaloacetic transaminase 1-like 1(GOT1L1)
M_196	414767	long intergenic non-protein coding RNA
M_196	353299	regulator of G-protein signaling like 1(RGSL1)
M_196	26211	olfactory receptor family 2 subfamily F member 1 (gene/pseudogene)(OR2F1)
M_196	5680	pregnancy specific beta-1-glycoprotein 11(PSG11)
M_196	417	ADP-ribosyltransferase 1(ART1)
M_196	58531	protamine 3(PRM3)
M_196	84694	gap junction protein alpha 10(GJA10)
M_196	445372	TRIM6-TRIM34 readthrough(TRIM6-TRIM34)
M_196	594857	neuropeptide S(NPS)
M_196	145645	telomere repeat binding bouquet formation protein
M_196	389207	glutaredoxin and cysteine rich domain containing
M_196	9366	RAB9B, member RAS oncogene family pseudogene 1(RAB9BP1)
M_196	284433	olfactory receptor family 10 subfamily H member
M_196	353132	late cornified envelope 1B(LCE1B)
M_196	127066	olfactory receptor family 14 subfamily C member 36(OR14C36)
M_196	325	amyloid P component, serum(APCS)
M_196	4159	melanocortin 3 receptor(MC3R)
M_196	81099	olfactory receptor family 4 subfamily F member
M_196	139760	G protein-coupled receptor 119(GPR119)
M_196	339521	cyclin-related protein FAM58B(FAM58BP)
M_196	339568	uncharacterized LOC339568(LOC339568)
M_196	149047	uncharacterized MGC27382(MGC27382)
M_196	729467	uncharacterized LOC729467(HSD52)
M_196	26539	olfactory receptor family 10 subfamily H member
M_196	81061	olfactory receptor family 11 subfamily H member

M_196	26534	olfactory receptor family 10 subfamily G member
M_196	26538	olfactory receptor family 10 subfamily H member
M_196	390201	olfactory receptor family 10 subfamily V member
M_196	60467	blepharophimosis, epicanthus inversus and ptosis, candidate 1 (non-protein coding)(BPESC1)
M_196	81870	keratin associated protein 9-9(KRTAP9-9)
M_196	386674	keratin associated protein 10-6(KRTAP10-6)
M_196	387332	TATA-box binding protein like 2(TBPL2)
M_196	254240	BPI fold containing family C(BPIFC)
M_196	646813	DEAH-box helicase 9 pseudogene(LOC646813)
M_196	386607	ZFP91-CNTF readthrough (NMD candidate)(ZFP91-
M_196	286365	olfactory receptor family 13 subfamily D member
M_196	219557	chromosome 7 open reading frame 62(C7orf62)
M_196	64600	phospholipase A2 group IIF(PLA2G2F)
M_196	5454	POU class 3 homeobox 2(POU3F2)
M_196	728495	family with sequence similarity 74 member
M_196	284359	izumo sperm-egg fusion 1(IZUMO1)
M_196	117194	MAS related GPR family member X2(MRGPRX2)
M_196	283902	hepatocellular carcinoma associated transcript 5 (non-protein coding)(HCCAT5)
M_197	23331	tetratricopeptide repeat domain 28(TTC28)
M_197	1036	cysteine dioxygenase type 1(CDO1)
M_197	23051	zinc fingers and homeoboxes 3(ZHX3)
M_197	23102	TBC1 domain family member 2B(TBC1D2B)
M_197	130132	raftlin family member 2(RFTN2)
M_197	10098	tetraspanin 5(TSPAN5)
M_197	4162	melanoma cell adhesion molecule(MCAM)
M_197	23187	pleckstrin homology like domain family B member 1(PHLDB1)
M_197	79834	pseudopodium enriched atypical kinase 1(PEAK1)
M_197	166336	prickle planar cell polarity protein 2(PRICKLE2)

M_197	55616	ArfGAP with SH3 domain, ankyrin repeat and PH domain 3(ASAP3)
M_197	55243	kin of IRRE like (Drosophila)(KIRREL)
M_197	6453	intersectin 1(ITSN1)
M_197	9697	translocation associated membrane protein 2(TRAM2)
M_197	22856	chondroitin sulfate synthase 1(CHSY1)
M_197	63971	kinesin family member 13A(KIF13A)
M_197	3096	human immunodeficiency virus type I enhancer binding protein 1(HIVEP1)
M_197	6310	ataxin 1(ATXN1)
M_197	139221	MUM1 like 1(MUM1L1)
M_197	23515	MORC family CW-type zinc finger 3(MORC3)
M_197	3340	N-deacetylase and N-sulfotransferase 1(NDST1)
M_197	23362	pleckstrin and Sec7 domain containing 3(PSD3)
M_197	11057	abhydrolase domain containing 2(ABHD2)
M_197	323	amyloid beta precursor protein binding family B member 2(APBB2)
M_197	8867	synaptojanin 1(SYNJ1)
M_197	3908	laminin subunit alpha 2(LAMA2)
M_197	4430	myosin IB(MYO1B)
M_197	4628	myosin heavy chain 10(MYH10)
M_197	23466	chromobox 6(CBX6)
M_197	23543	RNA binding protein, fox-1 homolog 2(RBFOX2)
M_197	64838	fibronectin type III domain containing 4(FNDC4)
M_197	90874	zinc finger protein 697(ZNF697)
M_197	11214	A-kinase anchoring protein 13(AKAP13)
M_197	79625	neuron derived neurotrophic factor(NDNF)
M_197	57502	neuroligin 4, X-linked(NLGN4X)
M_197	7798	leucine zipper protein 1(LUZP1)
M_197	80818	zinc finger protein 436(ZNF436)
M_197	9448	mitogen-activated protein kinase kinase kinase kinase 4(MAP4K4)

M_197	145407	chromosome 14 open reading frame 37(C14orf37)
M_197	2917	glutamate metabotropic receptor 7(GRM7)
M_197	80206	formin homology 2 domain containing 3(FHOD3)
M_197	441027	transmembrane protein 150C(TMEM150C)
M_197	93166	PR/SET domain 6(PRDM6)
M_197	83999	kringle containing transmembrane protein
M_197	9644	SH3 and PX domains 2A(SH3PXD2A)
M_197	23345	spectrin repeat containing nuclear envelope protein
M_197	6383	syndecan 2(SDC2)
M_197	9077	DIRAS family GTPase 3(DIRAS3)
M_197	83742	MARVEL domain containing 1(MARVELD1)
M_197	84133	zinc and ring finger 3(ZNRF3)
M_197	22846	vasohibin 1(VASH1)
M_197	7018	transferrin(TF)
M_197	57451	teneurin transmembrane protein 2(TENM2)
M_197	3489	insulin like growth factor binding protein 6(IGFBP6)
M_197	10404	carboxypeptidase Q(CPQ)
M_197	94121	synaptotagmin like 4(SYTL4)
M_197	25770	chromosome 22 open reading frame 31(C22orf31)
M_197	64780	microtubule associated monooxygenase, calponin and LIM domain containing 1(MICAL1)
M_197	2892	glutamate ionotropic receptor AMPA type subunit
M_197	123920	CKLF like MARVEL transmembrane domain containing 3(CMTM3)
M_198	138065	ring finger protein 183(RNF183)
M_198	388815	mir-99a-let-7c cluster host gene(MIR99AHG)
M_198	401546	chromosome 9 open reading frame 152(C9orf152)
M_198	7779	solute carrier family 30 member 1(SLC30A1)
M_198	279	amylase, alpha 2A (pancreatic)(AMY2A)
M_198	415117	syntaxin 19(STX19)
M_198	153769	SH3 domain containing ring finger 2(SH3RF2)
M_198	6778	signal transducer and activator of transcription

M_198	54762	GRAM domain containing 1C(GRAMD1C)
M_198	6565	solute carrier family 15 member 2(SLC15A2)
M_198	9910	RAB GTPase activating protein 1 like(RABGAP1L)
M_198	25854	family with sequence similarity 149 member
M_198	2651	glucosaminyl (N-acetyl) transferase 2, I-branching enzyme (I blood group)(GCNT2)
M_198	8470	sorbin and SH3 domain containing 2(SORBS2)
M_198	121506	endoplasmic reticulum protein 27(ERP27)
M_198	4693	NDP, norrin cystine knot growth factor(NDP)
M_198	140469	myosin IIIB(MYO3B)
M_198	6496	SIX homeobox 3(SIX3)
M_198	4588	mucin 6, oligomeric mucus/gel-forming(MUC6)
M_198	143662	mucin 15, cell surface associated(MUC15)
M_198	55314	transmembrane protein 144(TMEM144)
M_198	64699	transmembrane protease, serine 3(TMPRSS3)
M_198	22998	LIM and calponin homology domains 1(LIMCH1)
M_198	25928	sclerostin domain containing 1(SOSTDC1)
M_198	441869	ankyrin repeat domain 65(ANKRD65)
M_198	10758	TRAF3 interacting protein 2(TRAF3IP2)
M_198	85414	solute carrier family 45 member 3(SLC45A3)
M_198	4129	monoamine oxidase B(MAOB)
M_198	442425	forkhead box B2(FOXB2)
M_198	79315	olfactory receptor family 7 subfamily E member 91 pseudogene(OR7E91P)
M_198	63982	anoctamin 3(ANO3)
M_198	276	amylase, alpha 1A (salivary)(AMY1A)
M_198	347475	coiled-coil domain containing 160(CCDC160)
M_198	57162	pellino E3 ubiquitin protein ligase 1(PELI1)
M_198	132014	interleukin 17 receptor E(IL17RE)
M_198	54848	Rho guanine nucleotide exchange factor
M_198	51090	plasmolipin(PLLP)
M_198	55827	DDB1 and CUL4 associated factor 6(DCAF6)

M_198	729920	isoprenoid synthase domain containing(ISPD)
M_198	79170	proline rich 15 like(PRR15L)
M_198	81788	NUAK family kinase 2(NUAK2)
M_198	9966	tumor necrosis factor superfamily member
M_198	254827	N-acetylated alpha-linked acidic dipeptidase like 2(NAALADL2)
M_198	23007	phospholipase C eta 1(PLCH1)
M_198	144568	alpha-2-macroglobulin like 1(A2ML1)
M_198	391059	ferric chelate reductase 1(FRRS1)
M_198	643965	transmembrane protein 88B(TMEM88B)
M_198	94137	retinitis pigmentosa 1 like 1(RP1L1)
M_198	100188953	long intergenic non-protein coding RNA
M_198	50853	villin like(VILL)
M_198	8991	selenium binding protein 1(SELENBP1)
M_198	285440	cytochrome P450 family 4 subfamily V member
M_198	3818	kallikrein B1(KLKB1)
M_198	389206	BEN domain containing 4(BEND4)
M_199	149620	chitinase, acidic pseudogene 2(CHIAP2)
M_199	134860	trace amine associated receptor 9 (gene/pseudogene)(TAAR9)
M_199	390748	poly(A) binding protein nuclear 1 like,
M_199	338949	transmembrane protein 202(TMEM202)
M_199	8385	#N/A
M_199	54659	UDP glucuronosyltransferase family 1 member
M_199	100133171	#N/A
M_199	83446	coiled-coil domain containing 70(CCDC70)
M_199	196477	coiled-coil glutamate rich protein 1(CCER1)
M_199	259249	MAS related GPR family member X1(MRGPRX1)
M_199	128859	BPI fold containing family B member 6(BPIFB6)
M_199	7021	transcription factor AP-2 beta(TFAP2B)
M_199	254973	olfactory receptor family 1 subfamily L member
M_199	81328	olfactory receptor family 4 subfamily A member

M_199	353323	keratin associated protein 12-2(KRTAP12-2)
M_199	81341	olfactory receptor family 10 subfamily W member 1(OR10W1)
M_199	388503	complement component 3 precursor
M_199	146852	outer dense fiber of sperm tails 4(ODF4)
M_199	441608	olfactory receptor family 5 subfamily B member
M_199	10047	cystatin 8(CST8)
M_199	642597	A-kinase anchor inhibitor 1(AKAIN1)
M_199	3891	keratin 85(KRT85)
M_199	147719	LY6/PLAUR domain containing 4(LYPD4)
M_199	23626	SPO11, initiator of meiotic double stranded
M_199	728036	cancer/testis antigen family 47, member
M_199	154089	long intergenic non-protein coding RNA
M_199	6440	surfactant protein C(SFTPC)
M_199	23314	SATB homeobox 2(SATB2)
M_199	259287	taste 2 receptor member 41(TAS2R41)
M_199	57818	glucose-6-phosphatase catalytic subunit 2(G6PC2)
M_199	6975	tectorin beta(TECTB)
M_199	1442	chorionic somatomammotropin hormone 1(CSH1)
M_199	1443	chorionic somatomammotropin hormone 2(CSH2)
M_199	100129935	lectin, galactoside binding soluble 14 pseudogene(LOC100129935)
M_199	2689	growth hormone 2(GH2)
M_199	150538	SATB2 antisense RNA 1(SATB2-AS1)
M_199	26696	olfactory receptor family 2 subfamily T member
M_199	389320	testis expressed 43(TEX43)
M_199	391112	olfactory receptor family 6 subfamily Y member
M_199	1990	chymotrypsin like elastase family member 1(CELA1)
M_199	389549	FEZ family zinc finger 1(FEZF1)
M_199	150297	chromosome 22 open reading frame 42(C22orf42)
M_199	653427	forkhead box D4-like 5(FOXD4L5)
M_199	3543	immunoglobulin lambda like polypeptide 1(IGLL1)

M_199	134526	acyl-CoA thioesterase 12(ACOT12)
M_199	346562	G protein subunit alpha transducin 3(GNAT3)
M_199	57094	carboxypeptidase A6(CPA6)
M_199	5623	persephin(PSPN)
M_199	10881	actin like 7A(ACTL7A)
M_199	115795	FANCD2 opposite strand(FANCD2OS)
M_200	844	calsequestrin 1(CASQ1)
M_200	56001	nuclear RNA export factor 2(NXF2)
M_200	6658	SRY-box 3(SOX3)
M_200	167838	taxilin beta(TXLNB)
M_200	286411	long intergenic non-protein coding RNA
M_200	22983	microtubule associated serine/threonine kinase
M_200	199699	DAN domain BMP antagonist family member
M_200	94120	synaptotagmin like 3(SYTL3)
M_200	7350	uncoupling protein 1(UCP1)
M_200	8153	Rho family GTPase 2(RND2)
M_200	8464	SPT3 homolog, SAGA and STAGA complex component(SUPT3H)
M_200	23614	pancreatic polypeptide 2, pseudogene(PPY2P)
M_200	729338	centrin 4, pseudogene(CETN4P)
M_200	127665	zinc finger protein 648(ZNF648)
M_200	389421	lin-28 homolog B(LIN28B)
M_200	84839	retina and anterior neural fold homeobox 2(RAX2)
M_200	145241	ADAM metallopeptidase domain 21 pseudogene 1(ADAM21P1)
M_200	151254	amyotrophic lateral sclerosis 2 chromosome region candidate 11(ALS2CR11)
M_200	58473	pleckstrin homology domain containing
M_200	148418	sterile alpha motif domain containing 13(SAMD13)
M_200	8747	ADAM metallopeptidase domain 21(ADAM21)
M_200	131405	tripartite motif containing 71(TRIM71)
M_200	6847	synaptonemal complex protein 1(SYCP1)

M_200	646643	SH3 domain binding kinase family member 2(SBK2)
M_200	128954	GRB2 associated binding protein family member
M_200	164656	transmembrane protease, serine 6(TMPRSS6)
M_200	6447	secretogranin V(SCG5)
M_200	56341	protein arginine methyltransferase 8(PRMT8)
M_200	51308	receptor accessory protein 2(REEP2)
M_200	728343	nuclear RNA export factor 2B(NXF2B)
M_200	285368	proline rich transmembrane protein 3(PRRT3)
M_200	100132354	long intergenic non-protein coding RNA
M_200	50651	solute carrier family 45 member 1(SLC45A1)
M_200	2281	FK506 binding protein 1B(FKBP1B)
M_200	91807	myosin light chain kinase 3(MYLK3)
M_200	2571	glutamate decarboxylase 1(GAD1)
M_200	23632	carbonic anhydrase 14(CA14)
M_200	11189	CUGBP, Elav-like family member 3(CELF3)
M_200	1298	collagen type IX alpha 2 chain(COL9A2)
M_200	29075	long intergenic non-protein coding RNA
M_200	375704	energy homeostasis associated(ENHO)
M_200	5648	mannan binding lectin serine peptidase 1(MASP1)
M_200	6866	tachykinin 3(TAC3)
M_200	387700	solute carrier family 16 member 12(SLC16A12)
M_200	60676	pappalysin 2(PAPPA2)
M_201	311	annexin A11(ANXA11)
M_201	23555	tetraspanin 15(TSPAN15)
M_201	1352	COX10, heme A:farnesyltransferase cytochrome c oxidase assembly factor(COX10)
M_201	598	BCL2 like 1(BCL2L1)
M_201	645027	envoplakin like(EVPLL)
M_201	84293	family with sequence similarity 213 member
M_201	224	aldehyde dehydrogenase 3 family member
M_201	27077	B9 domain containing 1(B9D1)
M_201	147166	tripartite motif containing 16-like(TRIM16L)

M_201	54509	ras homolog family member F, filopodia
M_201	340706	von Willebrand factor A domain containing 2(VWA2)
M_201	379	ADP ribosylation factor like GTPase 4D(ARL4D)
M_201	81619	tetraspanin 14(TSPAN14)
M_201	100192426	uncharacterized LOC100192426(LOC100192426)
M_201	26051	protein phosphatase 1 regulatory subunit
M_201	7566	zinc finger protein 18(ZNF18)
M_201	125170	mitochondrial elongation factor 2(MIEF2)
M_201	2878	glutathione peroxidase 3(GPX3)
M_201	8416	annexin A9(ANXA9)
M_201	55118	cartilage acidic protein 1(CRTAC1)
M_201	55315	solute carrier family 29 member 3(SLC29A3)
M_201	401647	golgin A7 family member B(GOLGA7B)
M_201	642658	scleraxis bHLH transcription factor(SCX)
M_201	131583	family with sequence similarity 43 member
M_201	5368	prepronociceptin(PNOC)
M_201	55090	mediator complex subunit 9(MED9)
M_201	55793	family with sequence similarity 63 member
M_201	10400	phosphatidylethanolamine N-
M_201	6416	mitogen-activated protein kinase kinase 4(MAP2K4)
M_201	6441	surfactant protein D(SFTPD)
M_201	100128292	DLG5 antisense RNA 1(DLG5-AS1)
M_201	60528	elaC ribonuclease Z 2(ELAC2)
M_201	6341	SCO1, cytochrome c oxidase assembly protein(SCO1)
M_201	6470	serine hydroxymethyltransferase 1(SHMT1)
M_201	57060	poly(rC) binding protein 4(PCBP4)
M_201	4059	basal cell adhesion molecule (Lutheran blood
M_201	81502	histocompatibility minor 13(HM13)
M_201	79674	ventricular zone expressed PH domain containing
M_201	57326	PBX homeobox interacting protein 1(PBXIP1)
M_201	54902	tetratricopeptide repeat domain 19(TTC19)
M_201	201158	trans-golgi network vesicle protein 23 homolog

M_201	201164	phospholipase D family member 6(PLD6)
M_201	5150	phosphodiesterase 7A(PDE7A)
M_201	51030	trans-golgi network vesicle protein 23 homolog
M_201	9119	keratin 75(KRT75)
M_201	84612	par-6 family cell polarity regulator beta(PARD6B)
M_202	146779	EF-hand calcium binding domain 3(EFCAB3)
M_202	348021	long intergenic non-protein coding RNA
M_202	677773	small Cajal body-specific RNA 23(SCARNA23)
M_202	348158	acyl-CoA synthetase medium-chain family member 2B(ACSM2B)
M_202	143678	chromosome 11 open reading frame 94(C11orf94)
M_202	692091	small nucleolar RNA, C/D box 23(SNORD23)
M_202	127385	olfactory receptor family 10 subfamily J member
M_202	677818	small nucleolar RNA, H/ACA box 36B(SNORA36B)
M_202	2834	prolactin releasing hormone receptor(PRLHR)
M_202	100169951	small ILF3/NF90-associated RNA A3(SNAR-A3)
M_202	30814	phospholipase A2 group IIE(PLA2G2E)
M_202	2928	goosecoid homeobox 2(GSC2)
M_202	219473	olfactory receptor family 8 subfamily K member 3 (gene/pseudogene)(OR8K3)
M_202	100126780	small ILF3/NF90-associated RNA G1(SNAR-G1)
M_202	100126781	small ILF3/NF90-associated RNA F(SNAR-F)
M_202	100126799	small ILF3/NF90-associated RNA A2(SNAR-A2)
M_202	51052	prolactin releasing hormone(PRLH)
M_202	339976	tripartite motif family like 1(TRIML1)
M_202	727832	golgin A6 family-like 6(GOLGA6L6)
M_202	494141	solute carrier family 25 member 51
M_202	677835	small nucleolar RNA, H/ACA box 56(SNORA56)
M_202	347741	otopetrin 3(OTOP3)
M_202	221122	uncharacterized LOC221122(LOC221122)
M_202	283767	golgin A6 family-like 1(GOLGA6L1)
M_202	401634	golgin A2 pseudogene 3, Y-linked(GOLGA2P3Y)

M_202	391013	phospholipase A2 group IIC(PLA2G2C)
M_202	100130988	chromosome 7 open reading frame 72(C7orf72)
M_202	245912	defensin beta 109 pseudogene 1(DEFB109P1)
M_202	677778	small Cajal body-specific RNA 15(SCARNA15)
M_202	728773	poly(A) binding protein cytoplasmic 1 pseudogene 2(PABPC1P2)
M_202	100131439	CD300 molecule like family member d(CD300LD)
M_202	684959	small nucleolar RNA, H/ACA box 25(SNORA25)
M_202	266695	PHD finger protein 2 pseudogene 1(PHF2P1)
M_202	287015	tripartite motif containing 42(TRIM42)
M_202	6862	T brachyury transcription factor(T)
M_202	122651	ribonuclease A family member 11
M_202	677770	small Cajal body-specific RNA 22(SCARNA22)
M_202	26775	small nucleolar RNA, H/ACA box 72(SNORA72)
M_202	376844	syndecan 4 pseudogene(SDC4P)
M_202	5619	protamine 1(PRM1)
M_202	497049	microtubule affinity regulating kinase 1
M_202	677843	small nucleolar RNA, H/ACA box 77(SNORA77)
M_202	54075	CHODL antisense RNA 1(CHODL-AS1)
M_202	123876	acyl-CoA synthetase medium-chain family member 2A(ACSM2A)
M_202	118430	mucin like 1(MUCL1)
M_202	6360	C-C motif chemokine ligand 16(CCL16)
M_202	339184	coiled-coil domain containing 144 family, N-terminal like(CCDC144NL)
M_202	100124539	small nucleolar RNA, H/ACA box 11B(SNORA11B)
M_202	339	apolipoprotein B mRNA editing enzyme catalytic subunit 1(APOBEC1)
M_202	5502	protein phosphatase 1 regulatory inhibitor subunit 1A(PPP1R1A)
M_202	442459	X-ray repair cross complementing 6 pseudogene
M_202	8789	fructose-bisphosphatase 2(FBP2)

M_202	654321	small nucleolar RNA, H/ACA box 75(SNORA75)
M_202	677826	small nucleolar RNA, H/ACA box 3B(SNORA3B)
M_202	2693	growth hormone secretagogue receptor(GHSR)
M_202	120935	coiled-coil domain containing 38(CCDC38)
M_202	26783	small nucleolar RNA, H/ACA box 65(SNORA65)
M_202	440077	zinc finger protein 705A(ZNF705A)
M_202	23553	hyaluronoglucosaminidase 4(HYAL4)
M_202	126536	long intergenic non-protein coding RNA
M_202	100124533	small Cajal body-specific RNA 27(SCARNA27)
M_202	8811	galanin receptor 2(GALR2)
M_202	728957	zinc finger protein 705D(ZNF705D)
M_202	1215	chymase 1(CMA1)
M_203	54507	ADAMTS like 4(ADAMTSL4)
M_203	1889	endothelin converting enzyme 1(ECE1)
M_203	9922	IQ motif and Sec7 domain 1(IQSEC1)
M_203	84973	small nucleolar RNA host gene 7(SNHG7)
M_203	138311	family with sequence similarity 69 member
M_203	5361	plexin A1(PLXNA1)
M_203	126917	intermediate filament family orphan 2(IFFO2)
M_203	26086	G-protein signaling modulator 1(GPSM1)
M_203	84253	GTPase activating Rap/RanGAP domain like
M_203	219699	unc-5 netrin receptor B(UNC5B)
M_203	79586	chondroitin polymerizing factor(CHPF)
M_203	2817	glypican 1(GPC1)
M_203	149478	BTB domain containing 19(BTBD19)
M_203	89958	suppressor APC domain containing 2(SAPCD2)
M_203	9755	TBK1 binding protein 1(TBKBP1)
M_203	339451	kelch like family member 17(KLHL17)
M_203	114879	oxysterol binding protein like 5(OSBPL5)
M_203	114794	extracellular leucine rich repeat and fibronectin type III domain containing 2(ELFN2)
M_203	26102	uncharacterized LOC26102(DKFZP434A062)

M_203	26153	kinesin family member 26A(KIF26A)
M_203	2004	ELK3, ETS transcription factor(ELK3)
M_203	26012	NMDA receptor synaptonuclear signaling and neuronal migration factor(NSMF)
M_203	378832	COL18A1 antisense RNA 1(COL18A1-AS1)
M_203	441478	NOTCH-regulated ankyrin repeat protein(NRARP)
M_203	84662	GLIS family zinc finger 2(GLIS2)
M_203	116983	ArfGAP with coiled-coil, ankyrin repeat and PH domains 3(ACAP3)
M_203	55160	Rho guanine nucleotide exchange factor 10 like(ARHGEF10L)
M_203	114990	vasorin(VASN)
M_203	169714	quiescin sulfhydryl oxidase 2(QSOX2)
M_203	85352	KIAA1644(KIAA1644)
M_203	23371	tensin 2(TNS2)
M_203	338707	beta-1,4-N-acetyl-galactosaminyltransferase
M_203	20	ATP binding cassette subfamily A member 2(ABCA2)
M_203	26575	regulator of G-protein signaling 17(RGS17)
M_203	81855	sideroflexin 3(SFXN3)
M_203	26508	hes related family bHLH transcription factor with YRPW motif-like(HEYL)
M_203	387509	G protein-coupled receptor 153(GPR153)
M_203	51363	carbohydrate sulfotransferase 15(CHST15)
M_203	80781	collagen type XVIII alpha 1 chain(COL18A1)
M_203	25992	sushi, nidogen and EGF like domains 1(SNED1)
M_203	54863	torsin family 4 member A(TOR4A)
M_203	4853	notch 2(NOTCH2)
M_203	23129	plexin D1(PLXND1)
M_203	56975	FAM20C, golgi associated secretory pathway kinase(FAM20C)
M_203	6663	SRY-box 10(SOX10)
M_204	170261	zinc finger CCHC-type containing 12(ZCCHC12)

M_204	5354	proteolipid protein 1(PLP1)
M_204	57643	zinc finger SWIM-type containing 5(ZSWIM5)
M_204	5662	pleckstrin and Sec7 domain containing(PSD)
M_204	348093	RNA binding protein with multiple splicing
M_204	387097	#N/A
M_204	116173	CKLF like MARVEL transmembrane domain containing 5(CMTM5)
M_204	54549	sidekick cell adhesion molecule 2(SDK2)
M_204	124222	progestin and adipoQ receptor family member
M_204	655	bone morphogenetic protein 7(BMP7)
M_204	4359	myelin protein zero(MPZ)
M_204	9717	SEC14 like lipid binding 5(SEC14L5)
M_204	7473	Wnt family member 3(WNT3)
M_204	94032	calcium/calmodulin dependent protein kinase II inhibitor 2(CAMK2N2)
M_204	6753	somatostatin receptor 3(SSTR3)
M_204	284716	ribosomal modification protein rimK like family member A(RIMKLA)
M_204	26082	uncharacterized LOC26082(DKFZP434L187)
M_204	7140	troponin T3, fast skeletal type(TNNT3)
M_204	3208	hippocalcin(HPCA)
M_204	30062	retina and anterior neural fold homeobox(RAX)
M_204	143379	chromosome 10 open reading frame 82(C10orf82)
M_204	2900	glutamate ionotropic receptor kainate type subunit
M_204	23630	potassium voltage-gated channel subfamily E regulatory subunit 5(KCNE5)
M_204	117247	solute carrier family 16 member 10(SLC16A10)
M_204	257407	chromosome 2 open reading frame 72(C2orf72)
M_204	441394	SGT1 homolog, MIS12 kinetochore complex assembly cochaperone pseudogene 1(SUGT1P1)
M_204	5865	RAB3B, member RAS oncogene family(RAB3B)
M_204	80759	KH homology domain containing 1(KHDC1)

M_204	3706	inositol-trisphosphate 3-kinase A(ITPKA)
M_204	9118	internexin neuronal intermediate filament protein
M_204	57540	dispatched RND transporter family member 3(DISP3)
M_204	9427	endothelin converting enzyme like 1(ECEL1)
M_204	145501	isthmin 2(ISM2)
M_204	138715	AT-rich interaction domain 3C(ARID3C)
M_204	84889	solute carrier family 7 member 3(SLC7A3)
M_204	2676	GDNF family receptor alpha 3(GFRA3)
M_204	27443	CECR2, histone acetyl-lysine reader(CECR2)
M_204	58529	myozenin 1(MYOZ1)
M_204	79933	synaptopodin 2 like(SYNPO2L)
M_204	79412	kringle containing transmembrane protein
M_204	728116	zinc finger and BTB domain containing 8B(ZBTB8B)
M_204	81027	tubulin beta 1 class VI(TUBB1)
M_204	79570	Na+/K+ transporting ATPase interacting 1(NKAIN1)
M_204	90113	von Willebrand factor A domain containing
M_204	256435	ST6 N-acetylgalactosaminide alpha-2,6-sialyltransferase 3(ST6GALNAC3)
M_204	54413	neuroligin 3(NLGN3)
M_204	8326	frizzled class receptor 9(FZD9)
M_204	65989	delta like non-canonical Notch ligand 2(DLK2)
M_204	6620	synuclein beta(SNCB)
M_204	340543	transcription elongation factor A like 5(TCEAL5)
M_204	4882	natriuretic peptide receptor 2(NPR2)
M_204	10642	insulin like growth factor 2 mRNA binding protein
M_204	56936	coiled-coil domain containing 177(CCDC177)
M_204	4058	leukocyte receptor tyrosine kinase(LTK)
M_204	148709	actin pseudogene(LOC148709)
M_204	482	ATPase Na+/K+ transporting subunit beta 2(ATP1B2)
M_204	51440	hippocalcin like 4(HPCAL4)
M_204	9597	SMAD5 antisense RNA 1(SMAD5-AS1)
M_204	9970	nuclear receptor subfamily 1 group I member

M_204	7425	VGF nerve growth factor inducible(VGF)
M_204	551	arginine vasopressin(AVP)
M_204	84134	translocase of outer mitochondrial membrane 40 like(TOMM40L)
M_204	574029	dual specificity phosphatase 5 pseudogene
M_205	134864	trace amine associated receptor 1(TAAR1)
M_205	6568	solute carrier family 17 member 1(SLC17A1)
M_205	347487	chromosome X open reading frame 66(CXorf66)
M_205	85291	keratin associated protein 4-2(KRTAP4-2)
M_205	339788	long intergenic non-protein coding RNA
M_205	152118	chromosome 3 open reading frame 79(C3orf79)
M_205	56157	testis expressed 13A(TEX13A)
M_205	11172	insulin like 6(INSL6)
M_205	8284	lysine demethylase 5D(KDM5D)
M_205	10394	proteoglycan 3, pro eosinophil major basic protein
M_205	10786	solute carrier family 17 member 3(SLC17A3)
M_205	10816	serine peptidase inhibitor, Kunitz type 3(SPINT3)
M_205	643832	#N/A
M_205	3578	interleukin 9(IL9)
M_205	5741	parathyroid hormone(PTH)
M_205	344454	aldehyde oxidase 2 pseudogene(AOX2P)
M_205	7278	tubulin alpha 3c(TUBA3C)
M_205	54490	UDP glucuronosyltransferase family 2 member B28(UGT2B28)
M_205	3565	interleukin 4(IL4)
M_205	256892	olfactory receptor family 51 subfamily F member 1 (gene/pseudogene)(OR51F1)
M_205	160762	coiled-coil domain containing 63(CCDC63)
M_205	51032	chymotrypsin like elastase family member
M_205	26782	small nucleolar RNA, H/ACA box 66(SNORA66)
M_205	353134	late cornified envelope 1D(LCE1D)
M_205	26778	small nucleolar RNA, H/ACA box 70(SNORA70)

M_205	245934	defensin beta 121(DEFB121)
M_205	63036	chymotrypsin like elastase family member
M_205	353142	late cornified envelope 3A(LCE3A)
M_205	390259	brain specific homeobox(BSX)
M_205	389762	SPATA31 subfamily D member 3(SPATA31D3)
M_205	119774	olfactory receptor family 52 subfamily K member
M_205	8369	histone cluster 1 H4 family member g(HIST1H4G)
M_205	393046	olfactory receptor family 2 subfamily A member
M_205	2712	glycerol kinase 2(GK2)
M_205	50616	interleukin 22(IL22)
M_205	83551	trace amine associated receptor 8(TAAR8)
M_205	26077	uncharacterized LOC26077(DKFZP434H168)
M_205	374355	coiled-coil domain containing 172(CCDC172)
M_205	391365	sulfotransferase family 6B member 1(SULT6B1)
M_205	51480	variable charge, X-linked 2(VCX2)
M_205	3080	complement factor H related 2(CFHR2)
M_205	256144	olfactory receptor family 4 subfamily C member
M_205	344658	sterile alpha motif domain containing 7(SAMD7)
M_205	387071	family with sequence similarity 136 member B, pseudogene(FAM136BP)
M_205	100124541	small nucleolar RNA, H/ACA box 11D(SNORA11D)
M_205	84072	HORMA domain containing 1(HORMAD1)
M_205	1504	chymotrypsinogen B1(CTRB1)
M_205	51151	solute carrier family 45 member 2(SLC45A2)
M_205	56918	chromosome 2 open reading frame 83(C2orf83)
M_205	440387	chymotrypsinogen B2(CTRB2)
M_205	1576	cytochrome P450 family 3 subfamily A member
M_205	677812	small nucleolar RNA, H/ACA box 29(SNORA29)
M_205	56676	achaete-scute family bHLH transcription factor
M_205	5539	pancreatic polypeptide(PPY)
M_205	83597	receptor transporter protein 3(RTP3)
M_205	400668	protease, serine 57(PRSS57)

M_205	152816	chromosome 4 open reading frame 26(C4orf26)
M_205	400581	GRB2 related adaptor protein like(GRAPL)
M_206	5582	protein kinase C gamma(PRKCG)
M_206	400224	pleckstrin homology and coiled-coil domain containing D1(PLEKHD1)
M_206	138307	lipocalin 8(LCN8)
M_206	274	bridging integrator 1(BIN1)
M_206	339855	kyphoscoliosis peptidase(KY)
M_206	51195	Rap guanine nucleotide exchange factor like
M_206	23359	family with sequence similarity 189 member
M_206	339768	espin-like(ESPNL)
M_206	11122	protein tyrosine phosphatase, receptor type T(PTPRT)
M_206	167691	LCA5, lebercilin(LCA5)
M_206	248	alkaline phosphatase, intestinal(ALPI)
M_206	1183	chloride voltage-gated channel 4(CLCN4)
M_206	11045	uroplakin 1A(UPK1A)
M_206	285888	canopy FGF signaling regulator 1(CNPY1)
M_206	3780	potassium calcium-activated channel subfamily N member 1(KCNN1)
M_206	646023	ADORA2A antisense RNA 1(ADORA2A-AS1)
M_206	283748	phospholipase A2 group IVD(PLA2G4D)
M_206	59284	calcium voltage-gated channel auxiliary subunit gamma 7(CACNG7)
M_206	59283	calcium voltage-gated channel auxiliary subunit gamma 8(CACNG8)
M_206	440138	ALG11, alpha-1,2-mannosyltransferase(ALG11)
M_206	796	calcitonin related polypeptide alpha(CALCA)
M_206	389692	MAF bZIP transcription factor A(MAFA)
M_206	51085	MLX interacting protein like(MLXIPL)
M_206	23426	glutamate receptor interacting protein 1(GRIP1)
M_206	797	calcitonin related polypeptide beta(CALCB)
M_206	284422	small integral membrane protein 24(SMIM24)

M_206	56475	reprimo, TP53 dependent G2 arrest mediator candidate(RPRM)
M_206	321	amyloid beta precursor protein binding family A member 2(APBA2)
M_206	83699	SH3 domain binding glutamate rich protein like
M_206	9912	Rho GTPase activating protein 44(ARHGAP44)
M_206	2046	EPH receptor A8(EPHA8)
M_206	3768	potassium voltage-gated channel subfamily J member 12(KCNJ12)
M_206	114815	sortilin related VPS10 domain containing receptor
M_206	283521	long intergenic non-protein coding RNA
M_206	540	ATPase copper transporting beta(ATP7B)
M_206	2904	glutamate ionotropic receptor NMDA type subunit 2B(GRIN2B)
M_206	90362	family with sequence similarity 110 member
M_206	495	ATPase H+/K+ transporting alpha subunit(ATP4A)
M_206	64405	cadherin 22(CDH22)
M_206	161725	OTU deubiquitinase 7A(OTUD7A)
M_206	124359	chromodomain Y-like 2(CDYL2)
M_206	3361	5-hydroxytryptamine receptor 5A(HTR5A)
M_206	400954	echinoderm microtubule associated protein like
M_206	8513	lipase F, gastric type(LIPF)
M_206	23732	ferric chelate reductase 1 like(FRRS1L)
M_206	342931	ret finger protein like 4A(RFPL4A)
M_206	142910	lipase family member J(LIPJ)
M_207	285205	long intergenic non-protein coding RNA
M_207	27293	sphingomyelin phosphodiesterase acid like
M_207	7430	ezrin(EZR)
M_207	51429	sorting nexin 9(SNX9)
M_207	4521	nudix hydrolase 1(NUDT1)
M_207	84792	family with sequence similarity 220 member
M_207	56477	C-C motif chemokine ligand 28(CCL28)

M_207	51622	CCZ1 homolog, vacuolar protein trafficking and biogenesis associated(CCZ1)
M_207	54468	meiosis regulator for oocyte development(MIOS)
M_207	11123	RCAN family member 3(RCAN3)
M_207	441191	ring finger protein 216 pseudogene 1(RNF216P1)
M_207	3382	islet cell autoantigen 1(ICA1)
M_207	961	CD47 molecule(CD47)
M_207	5624	protein C, inactivator of coagulation factors Va and VIIIa(PROC)
M_207	8460	tyrosylprotein sulfotransferase 1(TPST1)
M_207	7108	transmembrane 7 superfamily member 2(TM7SF2)
M_207	7965	aminoacyl tRNA synthetase complex interacting multifunctional protein 2(AIMP2)
M_207	259	alpha-1-microglobulin/bikunin precursor(AMBP)
M_207	10804	gap junction protein beta 6(GJB6)
M_207	64417	transmembrane protein 267(TMEM267)
M_207	389458	RBAK downstream neighbor (non-protein
M_207	5879	ras-related C3 botulinum toxin substrate 1 (rho family, small GTP binding protein Rac1)(RAC1)
M_207	221960	CCZ1 homolog B, vacuolar protein trafficking and biogenesis associated(CCZ1B)
M_207	3485	insulin like growth factor binding protein 2(IGFBP2)
M_207	147700	kinesin light chain 3(KLC3)
M_207	79034	chromosome 7 open reading frame 26(C7orf26)
M_207	27102	eukaryotic translation initiation factor 2 alpha kinase 1(EIF2AK1)
M_207	113220	kinesin family member 12(KIF12)
M_207	439927	long intergenic non-protein coding RNA
M_207	26100	WD repeat domain, phosphoinositide interacting
M_207	55146	zinc finger DHHC-type containing 4(ZDHHC4)
M_207	4654	myogenic differentiation 1(MYOD1)
M_207	392862	Grid2 interacting protein(GRID2IP)

M_207	84310	chromosome 7 open reading frame 50(C7orf50)
M_207	151195	cyclin Y like 1(CCNYL1)
M_207	401303	zinc finger protein 815, pseudogene(ZNF815P)
M_207	11014	KDEL endoplasmic reticulum protein retention receptor 2(KDELR2)
M_207	4071	transmembrane 4 L six family member 1(TM4SF1)
M_207	3773	potassium voltage-gated channel subfamily J member 16(KCNJ16)
M_207	56913	core 1 synthase, glycoprotein-N-acetylgalactosamine 3-beta-galactosyltransferase 1(C1GALT1)
M_207	84262	proteasome assembly chaperone 3(PSMG3)
M_207	23548	tetratricopeptide repeat domain 33(TTC33)
M_207	90637	zinc finger AN1-type containing 2A(ZFAND2A)
M_207	151658	long intergenic non-protein coding RNA
M_207	9473	thymocyte selection associated family member
M_207	55081	intraflagellar transport 57(IFT57)
M_207	677779	long intergenic non-protein coding RNA
M_207	112703	family with sequence similarity 71 member
M_207	284361	ER membrane protein complex subunit 10(EMC10)
M_207	100133612	long intergenic non-protein coding RNA

Table S4 The Gene Ontology (GO) enrichment results of the OV marker genes

**GO ID**	***P value***
GO:0043066	1.00E-30
GO:0045944	1.00E-30
GO:0045766	1.00E-30
GO:0070374	1.00E-30
GO:0000122	1.00E-30
GO:0008285	1.00E-30
GO:0051897	1.00E-30
GO:0008284	1.00E-30
GO:0010469	1.00E-30

GO:0001666	1.00E-30
GO:0030335	3.80E-30
GO:0007568	2.10E-27
GO:0048661	1.70E-25
GO:0042493	1.80E-23
GO:0071260	1.90E-22
GO:0019221	2.70E-22
GO:0071456	4.20E-22
GO:0043065	5.80E-22
GO:0048146	7.80E-22
GO:0032355	9.10E-21
GO:0001938	3.00E-20
GO:0008630	1.20E-19
GO:0014068	1.90E-19
GO:0007050	2.80E-19
GO:0045669	5.30E-19
GO:0090090	9.10E-19
GO:0001525	1.90E-18
GO:0032496	4.60E-18
GO:0006954	2.90E-17
GO:0046854	5.20E-17
GO:0018108	8.30E-17
GO:0030308	1.60E-16
GO:0007229	3.20E-16
GO:0007566	3.40E-16
GO:2001237	3.80E-16
GO:0048010	5.90E-16
GO:0043524	7.90E-16
GO:0000187	9.60E-16
GO:0050731	2.20E-15
GO:0010718	2.40E-15
GO:0007179	5.90E-15

GO:0042127	8.40E-15
GO:0035019	9.60E-15
GO:0090263	1.60E-14
GO:0010628	2.00E-14
GO:0008283	2.20E-14
GO:0071222	2.50E-14
GO:0051781	2.60E-14
GO:0050679	2.60E-14
GO:0016032	2.70E-14
GO:0008584	3.10E-14
GO:0033138	3.70E-14
GO:1902895	4.60E-14
GO:0070301	5.20E-14
GO:0009968	5.70E-14
GO:0009612	5.90E-14
GO:0071560	7.40E-14
GO:0030198	8.70E-14
GO:0043406	1.70E-13
GO:0043154	1.70E-13
GO:0006915	1.80E-13
GO:0043552	2.80E-13
GO:0051092	3.50E-13
GO:0045471	4.00E-13
GO:0071356	4.20E-13
GO:0045893	5.70E-13
GO:0031100	6.10E-13
GO:0001558	7.50E-13
GO:0007267	8.20E-13
GO:0002576	8.30E-13
GO:0043536	8.40E-13
GO:0046777	1.50E-12
GO:0042531	1.70E-12

GO:0051091	1.80E-12
GO:0043123	2.10E-12
GO:0006977	2.10E-12
GO:0006955	2.80E-12
GO:0006919	3.10E-12
GO:0046697	3.70E-12
GO:0016477	4.30E-12
GO:0071480	4.70E-12
GO:0016525	4.80E-12
GO:0030324	5.20E-12
GO:0001934	6.00E-12
GO:1902042	1.20E-11
GO:0070373	1.20E-11
GO:0050680	1.20E-11
GO:0043627	1.30E-11
GO:0001701	1.60E-11
GO:1901224	1.70E-11
GO:0033674	1.70E-11
GO:0002223	1.90E-11
GO:0007565	2.10E-11
GO:1900740	2.20E-11
GO:0022617	2.30E-11
GO:0018105	2.40E-11
GO:0010332	2.40E-11
GO:2000811	4.40E-11
GO:0051496	4.40E-11
GO:0010629	5.80E-11
GO:2001240	6.10E-11
GO:0003148	6.10E-11
GO:0043312	6.20E-11
GO:0035987	6.40E-11
GO:0071549	6.70E-11

GO:0030336	7.30E-11
GO:0097192	1.10E-10
GO:0060045	1.30E-10
GO:0071347	1.40E-10
GO:0030513	1.50E-10
GO:0090200	1.60E-10
GO:0070098	1.70E-10
GO:0051591	1.70E-10
GO:0014911	1.70E-10
GO:0071407	1.90E-10
GO:0045599	2.00E-10
GO:0007623	2.10E-10
GO:0071333	2.60E-10
GO:0042060	2.80E-10
GO:0031663	3.50E-10
GO:0043388	3.60E-10
GO:0031295	3.90E-10
GO:0030574	4.80E-10
GO:0045429	4.90E-10
GO:0048012	6.10E-10
GO:1901216	6.50E-10
GO:0060337	6.90E-10
GO:0007507	7.30E-10
GO:0010165	7.60E-10
GO:0000083	7.80E-10
GO:0097150	8.00E-10
GO:0050728	8.30E-10
GO:0035116	8.60E-10
GO:0002053	8.70E-10
GO:0030307	9.70E-10
GO:2000353	1.20E-09
GO:0036092	1.60E-09

GO:0010595	1.80E-09
GO:0001569	1.80E-09
GO:0038061	1.80E-09
GO:0034644	2.10E-09
GO:0038083	2.30E-09
GO:0043525	2.40E-09
GO:0043542	3.00E-09
GO:0034446	3.70E-09
GO:0034614	4.00E-09
GO:0050821	4.20E-09
GO:0051726	4.50E-09
GO:0060412	4.60E-09
GO:0006293	4.70E-09
GO:0060333	5.30E-09
GO:0097421	5.70E-09
GO:0060395	6.40E-09
GO:0051384	7.10E-09
GO:0006367	7.30E-09
GO:0038128	7.50E-09
GO:2000773	7.70E-09
GO:2000379	7.80E-09
GO:0030154	9.10E-09
GO:2000352	9.30E-09
GO:0033627	9.70E-09
GO:0001974	1.40E-08
GO:1900034	1.50E-08
GO:0007595	1.60E-08
GO:0016579	1.70E-08
GO:0001541	1.70E-08
GO:0042542	1.90E-08
GO:0043280	2.10E-08
GO:0006303	2.30E-08

GO:0071230	2.30E-08
GO:0048538	2.40E-08
GO:0048013	2.40E-08
GO:0010862	2.50E-08
GO:0043433	2.70E-08
GO:0001502	2.70E-08
GO:0009887	2.80E-08
GO:0045765	2.80E-08
GO:0009615	2.90E-08
GO:0007160	3.00E-08
GO:0001657	3.00E-08
GO:0007584	3.10E-08
GO:0006296	3.20E-08
GO:0050727	3.50E-08
GO:0000082	3.50E-08
GO:0010863	3.60E-08
GO:0045648	4.60E-08
GO:0010575	4.60E-08
GO:0030501	4.60E-08
GO:0035729	4.90E-08
GO:0045071	5.40E-08
GO:0071276	5.50E-08
GO:0042104	5.60E-08
GO:0002062	5.70E-08
GO:0014823	5.70E-08
GO:0032147	6.20E-08
GO:0030325	6.30E-08
GO:0090050	6.30E-08
GO:0097191	6.40E-08
GO:0048701	6.40E-08
GO:0009611	6.70E-08
GO:0010951	7.90E-08

GO:0071392	8.00E-08
GO:0042307	8.10E-08
GO:0042771	8.60E-08
GO:0071363	8.80E-08
GO:0036297	9.20E-08
GO:0030199	9.40E-08
GO:0045668	9.40E-08
GO:0019430	9.80E-08
GO:0090399	9.90E-08
GO:0071300	1.10E-07
GO:0032967	1.10E-07
GO:0032148	1.20E-07
GO:0043434	1.20E-07
GO:0001954	1.30E-07
GO:1902230	1.40E-07
GO:0070498	1.40E-07
GO:0071364	1.50E-07
GO:0006978	1.50E-07
GO:0006294	1.60E-07
GO:0046718	1.70E-07
GO:0019371	1.80E-07
GO:0044344	1.90E-07
GO:0009791	2.00E-07
GO:0006953	2.20E-07
GO:0030521	2.20E-07
GO:0070911	2.20E-07
GO:0042733	2.40E-07
GO:0045727	2.50E-07
GO:0032872	2.50E-07
GO:0010544	3.00E-07
GO:0003183	3.00E-07
GO:0031658	3.00E-07

GO:0048011	3.30E-07
GO:0036120	3.40E-07
GO:0033280	3.40E-07
GO:0043537	3.50E-07
GO:0030509	3.50E-07
GO:0071380	3.60E-07
GO:0051592	4.20E-07
GO:0001570	4.30E-07
GO:0002040	4.60E-07
GO:0008625	4.80E-07
GO:0043392	4.80E-07
GO:0090023	5.00E-07
GO:0097194	5.10E-07
GO:0032088	5.20E-07
GO:0043407	5.20E-07
GO:0051894	5.50E-07
GO:0014912	6.50E-07
GO:0060391	6.60E-07
GO:2001241	6.60E-07
GO:0071320	6.80E-07
GO:1900745	7.00E-07
GO:0001937	7.20E-07
GO:0050900	7.30E-07
GO:0001658	8.40E-07
GO:0031648	8.80E-07
GO:0042476	8.90E-07
GO:0043491	9.00E-07
GO:0032092	9.10E-07
GO:0000302	9.60E-07
GO:0048662	1.30E-06
GO:2001238	1.30E-06
GO:0033209	1.30E-06

GO:2000378	1.30E-06
GO:0033574	1.30E-06
GO:0042752	1.40E-06
GO:1900026	1.40E-06
GO:0034605	1.40E-06
GO:0009749	1.40E-06
GO:0000086	1.70E-06
GO:0046686	1.70E-06
GO:0007420	1.70E-06
GO:0046326	1.80E-06
GO:0048546	1.80E-06
GO:1903672	1.80E-06
GO:0045662	1.80E-06
GO:0031069	1.80E-06
GO:0060444	1.80E-06
GO:0000079	1.90E-06
GO:1902176	1.90E-06
GO:0035162	1.90E-06
GO:0045651	1.90E-06
GO:0060397	1.90E-06
GO:0009650	1.90E-06
GO:0032922	1.90E-06
GO:0007173	1.90E-06
GO:0045725	2.00E-06
GO:0071732	2.00E-06
GO:0003222	2.00E-06
GO:0008360	2.00E-06
GO:0010888	2.20E-06
GO:0032570	2.20E-06
GO:0001542	2.20E-06
GO:0060484	2.20E-06
GO:1903800	2.20E-06

GO:0075713	2.20E-06
GO:0001649	2.20E-06
GO:0048008	2.40E-06
GO:0045892	2.50E-06
GO:0010634	2.60E-06
GO:0032354	2.60E-06
GO:1903140	2.60E-06
GO:0006290	2.60E-06
GO:0060440	2.60E-06
GO:0046881	2.60E-06
GO:0035234	2.60E-06
GO:0042593	2.60E-06
GO:0045747	2.60E-06
GO:0045597	2.70E-06
GO:0043200	2.70E-06
GO:0032760	2.90E-06
GO:0032729	2.90E-06
GO:0003151	3.00E-06
GO:0032481	3.10E-06
GO:0010971	3.10E-06
GO:0070527	3.10E-06
GO:0030512	3.20E-06
GO:0051216	3.20E-06
GO:0031954	3.40E-06
GO:0045672	3.40E-06
GO:0043410	3.70E-06
GO:0061036	4.10E-06
GO:0008210	4.20E-06
GO:0071481	4.20E-06
GO:0033690	4.20E-06
GO:0000186	4.30E-06
GO:0001822	4.40E-06

GO:0043401	4.40E-06
GO:0007596	4.40E-06
GO:0032735	4.50E-06
GO:0010812	4.50E-06
GO:0044319	4.70E-06
GO:0002063	4.70E-06
GO:0060749	4.80E-06
GO:0060065	4.80E-06
GO:0043518	4.80E-06
GO:0050729	5.10E-06
GO:0045746	5.20E-06
GO:0007283	5.60E-06
GO:0032869	5.70E-06
GO:0006283	5.70E-06
GO:0009636	5.70E-06
GO:0030168	5.90E-06
GO:0009952	5.90E-06
GO:0001889	6.00E-06
GO:0014065	6.90E-06
GO:0051898	7.00E-06
GO:0071479	7.60E-06
GO:0007131	7.60E-06
GO:0043507	8.00E-06
GO:0035994	8.70E-06
GO:0007204	8.80E-06
GO:0010918	8.80E-06
GO:0010944	8.80E-06
GO:1990314	8.80E-06
GO:0032436	9.10E-06
GO:0007155	9.20E-06
GO:0045732	9.40E-06
GO:0030900	1.00E-05

GO:0048704	1.00E-05
GO:0051770	1.10E-05
GO:0030949	1.10E-05
GO:0071548	1.10E-05
GO:0048333	1.10E-05
GO:0045931	1.10E-05
GO:2000573	1.10E-05
GO:0002548	1.10E-05
GO:2000279	1.10E-05
GO:0060766	1.10E-05
GO:0045840	1.10E-05
GO:0070317	1.20E-05
GO:0001843	1.20E-05
GO:0045740	1.20E-05
GO:0030855	1.30E-05
GO:0048536	1.30E-05
GO:0006298	1.30E-05
GO:0032870	1.30E-05
GO:0002479	1.30E-05
GO:0030511	1.30E-05
GO:0043687	1.40E-05
GO:0060326	1.50E-05
GO:0002262	1.50E-05
GO:0030510	1.50E-05
GO:0001756	1.60E-05
GO:0032819	1.60E-05
GO:0038111	1.60E-05
GO:0009408	1.70E-05
GO:0034097	1.70E-05
GO:0045787	1.70E-05
GO:0045786	1.70E-05
GO:0030282	1.80E-05

GO:0030879	1.80E-05
GO:1901215	1.90E-05
GO:0071398	1.90E-05
GO:0045736	1.90E-05
GO:0071277	1.90E-05
GO:0030216	2.00E-05
GO:0030514	2.00E-05
GO:0040008	2.00E-05
GO:0010803	2.10E-05
GO:0032930	2.20E-05
GO:0040037	2.20E-05
GO:0061419	2.20E-05
GO:0042699	2.20E-05
GO:0070345	2.20E-05
GO:0071681	2.20E-05
GO:0003289	2.20E-05
GO:0043570	2.20E-05
GO:0042102	2.20E-05
GO:0030593	2.40E-05
GO:0032526	2.50E-05
GO:0030728	2.60E-05
GO:0071498	2.60E-05
GO:0071236	2.60E-05
GO:0071425	2.60E-05
GO:0097067	2.60E-05
GO:0060068	2.60E-05
GO:0033148	2.60E-05
GO:0003149	2.60E-05
GO:0071285	2.60E-05
GO:0043568	2.60E-05
GO:0061029	2.60E-05
GO:0007281	2.70E-05

GO:0032720	2.70E-05
GO:0070371	2.80E-05
GO:0090307	2.80E-05
GO:0050918	2.90E-05
GO:0010976	2.90E-05
GO:0060716	2.90E-05
GO:0033189	2.90E-05
GO:0046827	2.90E-05
GO:0048469	3.00E-05
GO:0030183	3.00E-05
GO:0006260	3.10E-05
GO:0048873	3.30E-05
GO:0030878	3.30E-05
GO:0060021	3.30E-05
GO:0007165	3.50E-05
GO:0030111	3.50E-05
GO:0007049	3.60E-05
GO:0060548	3.60E-05
GO:0032091	3.80E-05
GO:0035722	3.80E-05
GO:0042475	3.90E-05
GO:0001953	4.10E-05
GO:0090201	4.20E-05
GO:0048714	4.20E-05
GO:0032868	4.20E-05
GO:0065003	4.30E-05
GO:0050678	4.60E-05
GO:0009416	4.60E-05
GO:0050772	4.70E-05
GO:1901990	4.80E-05
GO:0050715	4.80E-05
GO:0007613	5.10E-05

GO:0032715	5.20E-05
GO:0008209	5.40E-05
GO:0055093	5.50E-05
GO:0031103	5.50E-05
GO:0007275	5.50E-05
GO:1902043	5.50E-05
GO:0090280	5.50E-05
GO:0044849	5.50E-05
GO:0050930	5.50E-05
GO:0019372	5.50E-05
GO:0045667	5.70E-05
GO:0043547	5.80E-05
GO:0035635	5.80E-05
GO:0035456	5.80E-05
GO:0001501	5.90E-05
GO:0060020	5.90E-05
GO:0061626	5.90E-05
GO:0071493	5.90E-05
GO:0035655	5.90E-05
GO:1901796	6.00E-05
GO:0001890	6.00E-05
GO:0050927	6.40E-05
GO:1901522	6.40E-05
GO:0010212	6.50E-05
GO:0097284	6.50E-05
GO:0060174	6.50E-05
GO:0003198	6.50E-05
GO:0031532	6.80E-05
GO:0007088	6.80E-05
GO:0007221	7.50E-05
GO:0001958	8.10E-05
GO:0035902	8.20E-05

GO:0008015	8.20E-05
GO:0000724	8.30E-05
GO:0031398	8.50E-05
GO:0010942	8.60E-05
GO:0009314	8.70E-05
GO:0050714	8.70E-05
GO:0007265	9.40E-05
GO:0051259	9.40E-05
GO:0048754	0.0001
GO:0031641	0.00011
GO:0043032	0.00011

Table S5 The gene-gene co-expression relations of 20 OV modules

Module ID	Gene 1	Gene 2
M_11	87	9766
M_11	330	6648
M_11	330	259307
M_11	330	7128
M_11	330	4792
M_11	330	6364
M_11	330	4790
M_11	1490	1958
M_11	1490	2353
M_11	1490	3491
M_11	1490	1843
M_11	1490	2354
M_11	1490	4616
M_11	1490	10365
M_11	1490	1959
M_11	1490	1960
M_11	1490	3164
M_11	1490	7538

M_11	1490	1316
M_11	1958	2353
M_11	1958	2920
M_11	1958	3491
M_11	1958	64332
M_11	1958	1843
M_11	1958	1847
M_11	1958	2354
M_11	1958	3726
M_11	1958	4616
M_11	1958	9592
M_11	1958	467
M_11	1958	1959
M_11	1958	1960
M_11	1958	3164
M_11	1958	3569
M_11	1958	1839
M_11	1958	7538
M_11	1958	1316
M_11	1958	3725
M_11	1958	9021
M_11	1958	7832
M_11	1958	64651
M_11	1958	23645
M_11	2055	8658
M_11	2055	9639
M_11	2055	90459
M_11	2055	79648
M_11	2055	644128
M_11	2055	9258
M_11	2055	55326
M_11	2055	79660

M_11	2055	157285
M_11	2055	8793
M_11	2152	54206
M_11	2152	5583
M_11	2152	150094
M_11	2353	2920
M_11	2353	3491
M_11	2353	64332
M_11	2353	1843
M_11	2353	1847
M_11	2353	2354
M_11	2353	3726
M_11	2353	4616
M_11	2353	9592
M_11	2353	467
M_11	2353	1959
M_11	2353	1960
M_11	2353	3164
M_11	2353	3569
M_11	2353	1839
M_11	2353	7538
M_11	2353	1316
M_11	2353	1052
M_11	2353	3725
M_11	2353	8013
M_11	2353	9021
M_11	2353	7832
M_11	2353	388
M_11	2353	9314
M_11	2353	64651
M_11	2353	23645
M_11	2920	2921

M_11	2920	64332
M_11	2920	80149
M_11	2920	1843
M_11	2920	1847
M_11	2920	2354
M_11	2920	2919
M_11	2920	3576
M_11	2920	3726
M_11	2920	3569
M_11	2920	6372
M_11	2920	1839
M_11	2920	4792
M_11	2920	6364
M_11	2920	7538
M_11	2920	8870
M_11	2920	1052
M_11	2920	9021
M_11	2920	23645
M_11	2920	1326
M_11	2921	8061
M_11	2921	64332
M_11	2921	80149
M_11	2921	2919
M_11	2921	3576
M_11	2921	7124
M_11	2921	6372
M_11	2921	4792
M_11	2921	6364
M_11	2921	8870
M_11	3040	3043
M_11	3040	3039
M_11	3040	5196

M_11	3040	5473
M_11	3040	3042
M_11	3040	212
M_11	3040	388
M_11	3043	2354
M_11	3043	3039
M_11	3043	5196
M_11	3043	5473
M_11	3043	212
M_11	3397	3399
M_11	3397	3398
M_11	3397	51655
M_11	3397	29970
M_11	3397	145173
M_11	3399	387763
M_11	3399	6615
M_11	3491	1843
M_11	3491	2354
M_11	3491	4616
M_11	3491	10365
M_11	3491	387763
M_11	3491	1959
M_11	3491	1960
M_11	3491	3164
M_11	3491	7538
M_11	3491	1316
M_11	3491	6004
M_11	3976	8061
M_11	3976	8553
M_11	3976	9766
M_11	3976	23764
M_11	3976	64332

M_11	3976	80149
M_11	3976	1263
M_11	3976	1847
M_11	3976	2919
M_11	3976	1839
M_11	3976	9021
M_11	4207	79036
M_11	4207	100271849
M_11	4207	54929
M_11	4207	57794
M_11	4207	284439
M_11	5296	5976
M_11	5296	8178
M_11	5296	23031
M_11	5296	23383
M_11	5296	23770
M_11	5296	55295
M_11	5296	9170
M_11	5296	54815
M_11	5296	54929
M_11	5296	57130
M_11	5296	93436
M_11	5296	23373
M_11	5296	57794
M_11	5296	83983
M_11	5296	284439
M_11	5296	3727
M_11	5976	8178
M_11	5976	23031
M_11	5976	23383
M_11	5976	55295
M_11	5976	10147

M_11	5976	54815
M_11	5976	57130
M_11	5976	23373
M_11	5976	57794
M_11	6648	4050
M_11	6648	7128
M_11	7071	8061
M_11	7071	8553
M_11	7071	54206
M_11	7071	80149
M_11	7071	4616
M_11	7071	1839
M_11	7071	9314
M_11	8061	8553
M_11	8061	23764
M_11	8061	25819
M_11	8061	80149
M_11	8061	1263
M_11	8061	1847
M_11	8061	2919
M_11	8061	3576
M_11	8061	8870
M_11	8061	9021
M_11	8178	23031
M_11	8178	23383
M_11	8178	23770
M_11	8178	55295
M_11	8178	79036
M_11	8178	54815
M_11	8178	54929
M_11	8178	57130
M_11	8178	170463

M_11	8178	23373
M_11	8178	55049
M_11	8178	57794
M_11	8178	51291
M_11	8178	3727
M_11	8178	54858
M_11	8553	23764
M_11	8553	80149
M_11	8553	602
M_11	8553	1263
M_11	8553	1847
M_11	8553	3726
M_11	8553	4616
M_11	8553	1839
M_11	8553	7538
M_11	8553	9021
M_11	8553	64651
M_11	8658	9639
M_11	8658	90459
M_11	8658	79648
M_11	8658	644128
M_11	8658	9258
M_11	8658	55326
M_11	8658	79660
M_11	8658	157285
M_11	9639	90459
M_11	9639	79648
M_11	9639	644128
M_11	9639	9258
M_11	9639	55326
M_11	9639	79660
M_11	9639	157285

M_11	9766	5583
M_11	9766	166929
M_11	11316	23770
M_11	11316	54555
M_11	11316	79036
M_11	11316	84769
M_11	11316	115098
M_11	11316	7311
M_11	11316	54929
M_11	11316	64981
M_11	11316	93436
M_11	11316	25804
M_11	11316	55049
M_11	11316	57794
M_11	11316	126382
M_11	11316	25796
M_11	11316	729991
M_11	11316	8625
M_11	23031	23383
M_11	23031	55295
M_11	23031	57130
M_11	23031	170463
M_11	23031	23373
M_11	23031	284439
M_11	23031	51291
M_11	23031	3727
M_11	23031	54858
M_11	23031	5864
M_11	23383	55295
M_11	23383	10147
M_11	23383	54815
M_11	23383	57130

M_11	23383	23373
M_11	23383	57794
M_11	23383	83983
M_11	23383	284439
M_11	23383	80726
M_11	23764	64332
M_11	23764	80149
M_11	23764	1263
M_11	23764	1847
M_11	23764	3726
M_11	23764	4616
M_11	23764	1316
M_11	23764	9021
M_11	23764	166929
M_11	23764	1647
M_11	23764	23645
M_11	23764	11067
M_11	23764	80176
M_11	23770	54555
M_11	23770	55295
M_11	23770	79036
M_11	23770	84769
M_11	23770	115098
M_11	23770	7311
M_11	23770	9170
M_11	23770	51477
M_11	23770	54815
M_11	23770	54929
M_11	23770	57130
M_11	23770	93343
M_11	23770	93436
M_11	23770	170463

M_11	23770	23373
M_11	23770	25804
M_11	23770	55049
M_11	23770	57794
M_11	23770	729991
M_11	23770	8625
M_11	23770	284439
M_11	23770	3727
M_11	29086	54555
M_11	29086	79016
M_11	29086	115098
M_11	29086	55850
M_11	29086	64981
M_11	29086	79086
M_11	29086	93343
M_11	29086	25804
M_11	29086	25796
M_11	29086	8625
M_11	54206	150094
M_11	54555	55295
M_11	54555	79036
M_11	54555	84769
M_11	54555	115098
M_11	54555	7311
M_11	54555	9170
M_11	54555	54929
M_11	54555	57130
M_11	54555	64981
M_11	54555	79086
M_11	54555	93436
M_11	54555	25804
M_11	54555	55049

M_11	54555	57794
M_11	54555	126382
M_11	54555	25796
M_11	54555	729991
M_11	54555	8625
M_11	55295	79036
M_11	55295	9170
M_11	55295	54815
M_11	55295	54929
M_11	55295	57130
M_11	55295	93436
M_11	55295	170463
M_11	55295	23373
M_11	55295	55049
M_11	55295	57794
M_11	55295	83983
M_11	55295	284440
M_11	55295	284439
M_11	55295	3727
M_11	55295	54858
M_11	64332	80149
M_11	64332	1847
M_11	64332	2354
M_11	64332	2919
M_11	64332	3576
M_11	64332	467
M_11	64332	1960
M_11	64332	7128
M_11	64332	7538
M_11	64332	1052
M_11	64332	9021
M_11	64332	1326

M_11	79016	115098
M_11	79016	55850
M_11	79016	64981
M_11	79016	79086
M_11	79016	93343
M_11	79016	25796
M_11	79036	84769
M_11	79036	115098
M_11	79036	100271849
M_11	79036	7311
M_11	79036	54815
M_11	79036	54929
M_11	79036	55850
M_11	79036	93343
M_11	79036	93436
M_11	79036	170463
M_11	79036	23373
M_11	79036	25804
M_11	79036	55049
M_11	79036	57794
M_11	79036	126382
M_11	79036	25796
M_11	79036	729991
M_11	79036	8625
M_11	79036	284439
M_11	79036	3727
M_11	80149	602
M_11	80149	1263
M_11	80149	2919
M_11	80149	3576
M_11	80149	7124
M_11	80149	4792

M_11	80149	6364
M_11	80149	1052
M_11	84769	115098
M_11	84769	54929
M_11	84769	64981
M_11	84769	93436
M_11	84769	25804
M_11	84769	55049
M_11	84769	126382
M_11	84769	25796
M_11	84769	729991
M_11	84769	8625
M_11	84769	5864
M_11	90459	79648
M_11	90459	644128
M_11	90459	9258
M_11	90459	55326
M_11	90459	79660
M_11	90459	157285
M_11	115098	7311
M_11	115098	54929
M_11	115098	55850
M_11	115098	64981
M_11	115098	79086
M_11	115098	93343
M_11	115098	93436
M_11	115098	25804
M_11	115098	55049
M_11	115098	57794
M_11	115098	126382
M_11	115098	25796
M_11	115098	729991

M_11	115098	8625
M_11	115098	3727
M_11	100271849	54929
M_11	100271849	57794
M_11	100271849	284439
M_11	602	5971
M_11	602	7124
M_11	1263	1847
M_11	1263	3726
M_11	1263	4616
M_11	1263	1839
M_11	1263	8870
M_11	1263	9021
M_11	1263	166929
M_11	1263	1647
M_11	1263	80176
M_11	1843	1847
M_11	1843	2354
M_11	1843	3726
M_11	1843	4616
M_11	1843	10365
M_11	1843	387763
M_11	1843	467
M_11	1843	1959
M_11	1843	1960
M_11	1843	3164
M_11	1843	3569
M_11	1843	1839
M_11	1843	7538
M_11	1843	8870
M_11	1843	1316
M_11	1843	3725

M_11	1843	8013
M_11	1843	9021
M_11	1843	388
M_11	1843	9314
M_11	1843	64651
M_11	1843	23645
M_11	1843	5997
M_11	1847	2354
M_11	1847	3726
M_11	1847	4616
M_11	1847	467
M_11	1847	1960
M_11	1847	3164
M_11	1847	3569
M_11	1847	1839
M_11	1847	7538
M_11	1847	8870
M_11	1847	1316
M_11	1847	8013
M_11	1847	9021
M_11	1847	9314
M_11	1847	23645
M_11	2354	3726
M_11	2354	4616
M_11	2354	9592
M_11	2354	387763
M_11	2354	467
M_11	2354	1959
M_11	2354	1960
M_11	2354	3164
M_11	2354	3569
M_11	2354	1839

M_11	2354	7538
M_11	2354	1316
M_11	2354	3725
M_11	2354	8013
M_11	2354	9021
M_11	2354	388
M_11	2354	9314
M_11	2354	64651
M_11	2354	23645
M_11	2919	3576
M_11	2919	7124
M_11	2919	6372
M_11	2919	4792
M_11	2919	6364
M_11	3039	5196
M_11	3039	5473
M_11	3039	212
M_11	3576	7124
M_11	3576	6372
M_11	3576	4792
M_11	3576	6364
M_11	3576	8870
M_11	3576	199675
M_11	3726	4616
M_11	3726	9592
M_11	3726	467
M_11	3726	1959
M_11	3726	1960
M_11	3726	3164
M_11	3726	1839
M_11	3726	7538
M_11	3726	8870

M_11	3726	1052
M_11	3726	3725
M_11	3726	9021
M_11	3726	7832
M_11	3726	64651
M_11	3726	23645
M_11	4321	245972
M_11	4321	4317
M_11	4321	1118
M_11	4616	1959
M_11	4616	7538
M_11	4616	1316
M_11	4616	9021
M_11	5971	7124
M_11	5971	259307
M_11	5971	7128
M_11	5971	4794
M_11	7124	259307
M_11	7124	4050
M_11	7124	7128
M_11	7124	4792
M_11	7124	6364
M_11	7124	8870
M_11	7124	4794
M_11	7124	9235
M_11	7311	55850
M_11	7311	25804
M_11	7311	55049
M_11	7311	8625
M_11	9170	51477
M_11	9170	54815
M_11	9170	54929

M_11	9170	57130
M_11	9170	80714
M_11	9170	93436
M_11	9170	170463
M_11	9170	57794
M_11	9170	83983
M_11	9170	284439
M_11	9170	51291
M_11	9592	467
M_11	9592	3164
M_11	9592	3725
M_11	9592	1844
M_11	9592	7832
M_11	9592	3727
M_11	9592	64651
M_11	10147	57130
M_11	10147	23373
M_11	10147	83983
M_11	10147	284439
M_11	10365	387763
M_11	10365	1959
M_11	10365	1960
M_11	51477	80714
M_11	51477	170463
M_11	54815	54929
M_11	54815	57130
M_11	54815	93436
M_11	54815	23373
M_11	54815	57794
M_11	54815	83983
M_11	54815	51291
M_11	54815	3727

M_11	54929	57130
M_11	54929	93436
M_11	54929	170463
M_11	54929	25804
M_11	54929	55049
M_11	54929	57794
M_11	54929	126382
M_11	54929	25796
M_11	54929	729991
M_11	54929	8625
M_11	54929	284439
M_11	54929	5864
M_11	55850	64981
M_11	55850	79086
M_11	55850	93343
M_11	55850	25796
M_11	55850	8625
M_11	57130	93436
M_11	57130	23373
M_11	57130	57794
M_11	57130	83983
M_11	57130	284439
M_11	57130	51291
M_11	64981	79086
M_11	64981	93343
M_11	64981	25804
M_11	64981	25796
M_11	64981	8625
M_11	79086	93343
M_11	79086	25804
M_11	79086	25796
M_11	79086	8625

M_11	79648	644128
M_11	79648	9258
M_11	79648	55326
M_11	79648	79660
M_11	80714	93436
M_11	80714	83983
M_11	80714	284439
M_11	93343	25796
M_11	93343	8625
M_11	93436	170463
M_11	93436	23373
M_11	93436	25804
M_11	93436	55049
M_11	93436	57794
M_11	93436	83983
M_11	93436	126382
M_11	93436	729991
M_11	93436	8625
M_11	93436	284439
M_11	116211	245972
M_11	116211	4317
M_11	116211	89886
M_11	170463	23373
M_11	170463	55049
M_11	170463	284439
M_11	170463	3727
M_11	170463	5864
M_11	245972	4317
M_11	245972	1118
M_11	245972	150350
M_11	259307	4050
M_11	259307	3695

M_11	259307	4794
M_11	387763	1959
M_11	387763	1960
M_11	387763	3164
M_11	387763	3569
M_11	387763	7538
M_11	387763	8013
M_11	644128	9258
M_11	644128	55326
M_11	644128	79660
M_11	644128	157285
M_11	467	1959
M_11	467	1960
M_11	467	3164
M_11	467	7538
M_11	467	1316
M_11	467	3725
M_11	467	8013
M_11	467	1844
M_11	467	7832
M_11	467	388
M_11	467	9314
M_11	467	64651
M_11	467	23645
M_11	467	1961
M_11	1026	9314
M_11	1959	1960
M_11	1959	3164
M_11	1959	3569
M_11	1959	7538
M_11	1959	1316
M_11	1959	8013

M_11	1959	9021
M_11	1960	3164
M_11	1960	7538
M_11	1960	1316
M_11	1960	3725
M_11	1960	8013
M_11	1960	9021
M_11	1960	1844
M_11	1960	388
M_11	1960	9314
M_11	1960	64651
M_11	1960	23645
M_11	1960	1961
M_11	3164	3569
M_11	3164	1839
M_11	3164	7538
M_11	3164	1316
M_11	3164	3725
M_11	3164	8013
M_11	3164	9021
M_11	3164	1844
M_11	3164	7832
M_11	3164	388
M_11	3164	9314
M_11	3164	64651
M_11	3164	23645
M_11	3164	1961
M_11	3164	150094
M_11	3164	100302650
M_11	3569	1839
M_11	3569	7538
M_11	3569	8870

M_11	3569	1316
M_11	3569	8013
M_11	3569	9021
M_11	3569	4783
M_11	3569	5997
M_11	4050	10537
M_11	4050	4318
M_11	4050	3604
M_11	4317	89886
M_11	4317	199675
M_11	5196	5473
M_11	7128	4792
M_11	7128	4790
M_11	9258	55326
M_11	9258	79660
M_11	9258	157285
M_11	23373	57794
M_11	23373	284439
M_11	23373	54858
M_11	23373	80726
M_11	25804	55049
M_11	25804	126382
M_11	25804	25796
M_11	25804	729991
M_11	25804	8625
M_11	55049	126382
M_11	55049	729991
M_11	55049	8625
M_11	55326	79660
M_11	57794	83983
M_11	57794	729991
M_11	57794	8625

M_11	57794	284439
M_11	83983	284440
M_11	83983	284439
M_11	126382	729991
M_11	126382	8625
M_11	1839	7538
M_11	1839	1316
M_11	1839	1052
M_11	1839	8013
M_11	1839	9021
M_11	3398	84099
M_11	4792	6364
M_11	4792	1052
M_11	6364	3552
M_11	7538	1316
M_11	7538	1052
M_11	7538	3725
M_11	7538	8013
M_11	7538	9021
M_11	7538	64651
M_11	7538	23645
M_11	8870	9021
M_11	25796	8625
M_11	79660	157285
M_11	79660	150094
M_11	79660	83595
M_11	157285	83595
M_11	729991	8625
M_11	1316	1052
M_11	1316	3725
M_11	1316	8013
M_11	1316	9021

M_11	1316	64651
M_11	1316	23645
M_11	1316	5997
M_11	1316	6446
M_11	284440	284439
M_11	1052	9021
M_11	1052	11067
M_11	3042	51327
M_11	3725	1844
M_11	3725	7832
M_11	3725	388
M_11	3725	3727
M_11	3725	64651
M_11	3725	23645
M_11	3725	150094
M_11	3725	677
M_11	3725	149647
M_11	8013	9021
M_11	8013	9314
M_11	8013	64651
M_11	8013	23645
M_11	8013	5997
M_11	8013	3036
M_11	9021	23645
M_11	56729	199675
M_11	1844	7832
M_11	1844	388
M_11	1844	64651
M_11	1844	1961
M_11	1844	2865
M_11	6521	283796
M_11	6521	212

M_11	7832	388
M_11	7832	100302650
M_11	7832	677
M_11	7832	149647
M_11	283796	344387
M_11	212	2815
M_11	388	51655
M_11	388	9314
M_11	388	64651
M_11	388	150094
M_11	51291	50619
M_11	4783	9314
M_11	4794	9235
M_11	9314	64651
M_11	9314	23645
M_11	166929	4790
M_11	64651	23645
M_11	64651	150094
M_11	1647	7277
M_11	23645	100302650
M_11	29970	145173
M_11	5997	6446
M_11	80726	4838
M_11	4790	27071
M_11	8793	145173
M_11	344387	91948
M_25	133	54541
M_25	133	144195
M_25	133	154761
M_25	133	285966
M_25	133	51129
M_25	133	112399

M_25	133	80328
M_25	133	6781
M_25	1750	10481
M_25	1750	27022
M_25	1750	63950
M_25	1750	285987
M_25	1750	1749
M_25	1750	3229
M_25	1750	10655
M_25	2306	3206
M_25	2306	6338
M_25	2306	27022
M_25	2306	63950
M_25	2306	84793
M_25	2306	171586
M_25	2306	3209
M_25	2306	3229
M_25	2306	5165
M_25	2306	80328
M_25	3199	3200
M_25	3199	3205
M_25	3199	3206
M_25	3199	3207
M_25	3199	221883
M_25	3199	3201
M_25	3199	3204
M_25	3199	3198
M_25	3199	3202
M_25	3199	3203
M_25	3199	8935
M_25	3200	3205
M_25	3200	3206

M_25	3200	221883
M_25	3200	3201
M_25	3200	3204
M_25	3200	3198
M_25	3200	3202
M_25	3200	3203
M_25	3200	8935
M_25	3205	3206
M_25	3205	3207
M_25	3205	221883
M_25	3205	3201
M_25	3205	3204
M_25	3205	3226
M_25	3205	3198
M_25	3205	3202
M_25	3205	3203
M_25	3205	3209
M_25	3206	3207
M_25	3206	10481
M_25	3206	84793
M_25	3206	221883
M_25	3206	3201
M_25	3206	3204
M_25	3206	3226
M_25	3206	58524
M_25	3206	3198
M_25	3206	3202
M_25	3206	3203
M_25	3206	51299
M_25	3206	3209
M_25	3206	80328
M_25	3206	2297

M_25	3207	221883
M_25	3207	3202
M_25	3207	3209
M_25	3224	3225
M_25	3224	3223
M_25	3224	3226
M_25	3225	3227
M_25	3225	100124700
M_25	3225	3223
M_25	3225	3226
M_25	3225	4488
M_25	3225	3229
M_25	3227	10481
M_25	3227	360205
M_25	3227	100124700
M_25	3227	3223
M_25	3227	3226
M_25	3227	1045
M_25	3227	3651
M_25	3227	3209
M_25	3227	3229
M_25	6338	6340
M_25	6338	84793
M_25	6340	64711
M_25	6340	112399
M_25	6340	9568
M_25	6340	260434
M_25	6515	144195
M_25	6515	3772
M_25	10481	63950
M_25	10481	285987
M_25	10481	360205

M_25	10481	1761
M_25	10481	3226
M_25	10481	1045
M_25	10481	3651
M_25	10481	30012
M_25	10481	3229
M_25	27022	63950
M_25	27022	84793
M_25	27022	285987
M_25	27022	360205
M_25	27022	57057
M_25	27022	59336
M_25	54541	51129
M_25	54541	687
M_25	63950	84793
M_25	63950	285987
M_25	63950	57057
M_25	83850	92370
M_25	83850	1761
M_25	84793	171586
M_25	84793	5165
M_25	84793	80328
M_25	144195	5292
M_25	154761	285966
M_25	221883	3204
M_25	221883	3198
M_25	221883	3202
M_25	221883	3203
M_25	221883	3209
M_25	285987	1749
M_25	285987	3204
M_25	285987	1045

M_25	285987	57057
M_25	285987	3229
M_25	285987	10716
M_25	360205	1761
M_25	360205	1045
M_25	360205	30012
M_25	360205	3229
M_25	100124700	3223
M_25	100124700	3226
M_25	100124700	3651
M_25	100124700	3229
M_25	1749	1780
M_25	1761	58524
M_25	2300	2303
M_25	2300	4488
M_25	2300	51278
M_25	3201	3204
M_25	3201	3198
M_25	3201	3202
M_25	3201	3203
M_25	3201	8935
M_25	3204	3198
M_25	3204	3202
M_25	3204	3203
M_25	3223	3226
M_25	3223	3229
M_25	3223	55867
M_25	3226	3203
M_25	3226	3209
M_25	3226	3229
M_25	4488	388662
M_25	4488	147495

M_25	58524	6913
M_25	58524	10655
M_25	171586	5165
M_25	171586	80328
M_25	388662	3202
M_25	388662	114787
M_25	388662	2774
M_25	1045	3651
M_25	1045	3229
M_25	3198	3202
M_25	3198	3203
M_25	3198	8935
M_25	3198	80328
M_25	3202	3203
M_25	3202	8935
M_25	3203	3209
M_25	3203	8935
M_25	51299	57057
M_25	51299	2297
M_25	57057	5457
M_25	57057	10716
M_25	57057	343472
M_25	64711	254528
M_25	64711	260434
M_25	112399	29091
M_25	479	9568
M_25	9568	84959
M_25	59336	5731
M_25	10716	343472
M_25	343472	221833
M_26	332	890
M_26	332	891

M_26	332	991
M_26	332	1058
M_26	332	7083
M_26	332	11065
M_26	332	54478
M_26	332	6790
M_26	332	8475
M_26	332	9133
M_26	332	55789
M_26	332	1033
M_26	332	983
M_26	332	5347
M_26	332	57405
M_26	332	113130
M_26	332	3838
M_26	332	6241
M_26	332	220134
M_26	641	90381
M_26	641	9055
M_26	641	55215
M_26	641	10721
M_26	641	990
M_26	641	51514
M_26	699	1058
M_26	699	3832
M_26	699	9928
M_26	699	10112
M_26	699	11004
M_26	699	24137
M_26	699	29127
M_26	699	56992
M_26	699	150468

M_26	699	151648
M_26	699	259266
M_26	699	7153
M_26	699	9493
M_26	699	22974
M_26	699	23397
M_26	699	54821
M_26	699	81610
M_26	699	6491
M_26	699	9787
M_26	699	10721
M_26	699	55635
M_26	699	57650
M_26	699	83540
M_26	699	146909
M_26	699	5347
M_26	699	9833
M_26	699	55355
M_26	699	10403
M_26	699	7272
M_26	699	1062
M_26	701	3832
M_26	701	5888
M_26	701	9928
M_26	701	11339
M_26	701	56992
M_26	701	57082
M_26	701	150468
M_26	701	259266
M_26	701	653820
M_26	701	728833
M_26	701	1063

M_26	701	9493
M_26	701	9824
M_26	701	22974
M_26	701	23397
M_26	701	51203
M_26	701	55388
M_26	701	10721
M_26	701	90417
M_26	890	891
M_26	890	1058
M_26	890	9212
M_26	890	11065
M_26	890	55247
M_26	890	64151
M_26	890	150468
M_26	890	4085
M_26	890	6790
M_26	890	9133
M_26	890	55388
M_26	890	84057
M_26	890	9787
M_26	890	55635
M_26	890	83540
M_26	890	5347
M_26	890	10733
M_26	890	55355
M_26	890	3833
M_26	891	991
M_26	891	995
M_26	891	11065
M_26	891	64105
M_26	891	9133

M_26	891	9232
M_26	891	55789
M_26	891	983
M_26	891	4751
M_26	891	3838
M_26	991	995
M_26	991	1058
M_26	991	9212
M_26	991	10024
M_26	991	11004
M_26	991	11065
M_26	991	83461
M_26	991	4605
M_26	991	4998
M_26	991	6790
M_26	991	8438
M_26	991	9133
M_26	991	81610
M_26	991	1033
M_26	991	6491
M_26	991	9787
M_26	991	55143
M_26	991	55635
M_26	991	983
M_26	991	5347
M_26	991	9833
M_26	991	113130
M_26	991	10403
M_26	991	3838
M_26	995	1058
M_26	995	9212
M_26	995	10112

M_26	995	11065
M_26	995	8475
M_26	995	9133
M_26	995	9232
M_26	995	51203
M_26	995	9787
M_26	995	983
M_26	995	9833
M_26	995	57405
M_26	995	55355
M_26	995	113130
M_26	995	10403
M_26	995	7272
M_26	1058	9212
M_26	1058	10024
M_26	1058	11004
M_26	1058	11065
M_26	1058	24137
M_26	1058	29127
M_26	1058	64151
M_26	1058	83461
M_26	1058	653820
M_26	1058	4605
M_26	1058	6790
M_26	1058	7153
M_26	1058	9133
M_26	1058	22974
M_26	1058	23397
M_26	1058	81610
M_26	1058	9787
M_26	1058	55635
M_26	1058	983

M_26	1058	5347
M_26	1058	9833
M_26	1058	55355
M_26	1058	113130
M_26	1058	10403
M_26	1058	6241
M_26	1652	4282
M_26	1652	80764
M_26	1652	85359
M_26	1652	7353
M_26	1652	64976
M_26	1652	5902
M_26	1652	23753
M_26	1652	150223
M_26	1652	400916
M_26	2305	9212
M_26	2305	10024
M_26	2305	83461
M_26	2305	4605
M_26	2305	9055
M_26	2305	9493
M_26	2305	10635
M_26	2305	22974
M_26	2305	23397
M_26	2305	81610
M_26	2305	9700
M_26	2305	146909
M_26	2305	4288
M_26	2305	55355
M_26	2305	113130
M_26	2305	83903
M_26	2873	51529

M_26	2873	79058
M_26	2873	201254
M_26	2873	6182
M_26	2873	10189
M_26	2873	339229
M_26	2873	284184
M_26	2873	348262
M_26	2873	339231
M_26	3832	9928
M_26	3832	55165
M_26	3832	56992
M_26	3832	57082
M_26	3832	150468
M_26	3832	259266
M_26	3832	1063
M_26	3832	22974
M_26	3832	9585
M_26	3832	4288
M_26	3832	1763
M_26	3832	1062
M_26	4282	80764
M_26	4282	85359
M_26	4282	7353
M_26	4282	64976
M_26	4282	5902
M_26	4282	150223
M_26	4282	400916
M_26	5888	11339
M_26	5888	57082
M_26	5888	9133
M_26	5888	9824
M_26	5888	51203

M_26	5888	84057
M_26	5888	55143
M_26	5888	90417
M_26	5888	113130
M_26	5888	113115
M_26	7083	6182
M_26	7083	8475
M_26	7083	9088
M_26	7083	9232
M_26	7083	10189
M_26	7083	1033
M_26	7083	79019
M_26	7083	3838
M_26	7332	9342
M_26	7332	80764
M_26	7332	85359
M_26	7332	7353
M_26	7332	64976
M_26	7332	5902
M_26	7332	8220
M_26	7332	23753
M_26	7332	128977
M_26	7332	150223
M_26	7332	10587
M_26	7332	27037
M_26	7332	6576
M_26	8914	23306
M_26	8914	55732
M_26	8914	9700
M_26	8914	83903
M_26	8914	11113
M_26	8914	79172

M_26	9212	10024
M_26	9212	11004
M_26	9212	11065
M_26	9212	54478
M_26	9212	83461
M_26	9212	4605
M_26	9212	6790
M_26	9212	7153
M_26	9212	9133
M_26	9212	9156
M_26	9212	9493
M_26	9212	22974
M_26	9212	23397
M_26	9212	55388
M_26	9212	81610
M_26	9212	83540
M_26	9212	5347
M_26	9212	10403
M_26	9212	3833
M_26	9342	80764
M_26	9342	85359
M_26	9342	7353
M_26	9342	64976
M_26	9342	5902
M_26	9342	8220
M_26	9342	128977
M_26	9342	150223
M_26	9342	10587
M_26	9342	27037
M_26	9342	6576
M_26	9928	24137
M_26	9928	56992

M_26	9928	57082
M_26	9928	90381
M_26	9928	150468
M_26	9928	259266
M_26	9928	653820
M_26	9928	728833
M_26	9928	1063
M_26	9928	7153
M_26	9928	9156
M_26	9928	22974
M_26	9928	729533
M_26	9928	9700
M_26	9928	10721
M_26	9928	146909
M_26	9928	4751
M_26	9928	55355
M_26	9928	128239
M_26	9928	11113
M_26	9928	1062
M_26	9928	51514
M_26	10024	11004
M_26	10024	11065
M_26	10024	29127
M_26	10024	83461
M_26	10024	4605
M_26	10024	8438
M_26	10024	9133
M_26	10024	9493
M_26	10024	22974
M_26	10024	23397
M_26	10024	51203
M_26	10024	81610

M_26	10024	9700
M_26	10024	146909
M_26	10024	5347
M_26	10024	55355
M_26	10024	80119
M_26	10112	24137
M_26	10112	29127
M_26	10112	150468
M_26	10112	259266
M_26	10112	9055
M_26	10112	9232
M_26	10112	9493
M_26	10112	22974
M_26	10112	51203
M_26	10112	81610
M_26	10112	9787
M_26	10112	55355
M_26	10112	10403
M_26	10112	3833
M_26	10112	51512
M_26	10460	64151
M_26	10460	653820
M_26	10460	728833
M_26	10460	5347
M_26	11004	11065
M_26	11004	24137
M_26	11004	29127
M_26	11004	64151
M_26	11004	150468
M_26	11004	151648
M_26	11004	4605
M_26	11004	4998

M_26	11004	8438
M_26	11004	9133
M_26	11004	9493
M_26	11004	22974
M_26	11004	23397
M_26	11004	55388
M_26	11004	81610
M_26	11004	6491
M_26	11004	9787
M_26	11004	55143
M_26	11004	55635
M_26	11004	146909
M_26	11004	5347
M_26	11004	9833
M_26	11004	113130
M_26	11004	10403
M_26	11004	3833
M_26	11065	54478
M_26	11065	83461
M_26	11065	653820
M_26	11065	4605
M_26	11065	6790
M_26	11065	8475
M_26	11065	9133
M_26	11065	9232
M_26	11065	22974
M_26	11065	55388
M_26	11065	81610
M_26	11065	84057
M_26	11065	83540
M_26	11065	983
M_26	11065	5347

M_26	11065	55355
M_26	11065	29089
M_26	11339	9133
M_26	11339	9824
M_26	11339	51203
M_26	11339	9768
M_26	11339	90417
M_26	24137	29127
M_26	24137	56992
M_26	24137	64151
M_26	24137	150468
M_26	24137	259266
M_26	24137	285643
M_26	24137	2491
M_26	24137	7153
M_26	24137	8438
M_26	24137	9493
M_26	24137	22974
M_26	24137	23397
M_26	24137	51203
M_26	24137	54821
M_26	24137	81610
M_26	24137	9787
M_26	24137	55635
M_26	24137	146909
M_26	24137	5347
M_26	24137	9833
M_26	24137	113130
M_26	24137	10403
M_26	24137	3833
M_26	24137	128239
M_26	24137	51512

M_26	29127	55247
M_26	29127	64151
M_26	29127	150468
M_26	29127	151648
M_26	29127	7153
M_26	29127	9133
M_26	29127	9493
M_26	29127	22974
M_26	29127	23397
M_26	29127	51203
M_26	29127	54821
M_26	29127	81610
M_26	29127	9700
M_26	29127	9787
M_26	29127	55635
M_26	29127	83540
M_26	29127	146909
M_26	29127	983
M_26	29127	5347
M_26	29127	4751
M_26	29127	55355
M_26	29127	113130
M_26	29127	10403
M_26	29127	7272
M_26	51529	201254
M_26	51529	6182
M_26	51529	10189
M_26	51529	339229
M_26	51529	284184
M_26	51529	348262
M_26	54478	83461
M_26	54478	8475

M_26	54478	83903
M_26	55165	2491
M_26	55165	9493
M_26	55165	983
M_26	55165	51512
M_26	55247	64151
M_26	55247	54821
M_26	55247	81610
M_26	55247	84057
M_26	55247	55635
M_26	55247	5347
M_26	55247	55355
M_26	55872	157570
M_26	55872	157313
M_26	55872	1033
M_26	55872	1164
M_26	56992	57082
M_26	56992	64151
M_26	56992	150468
M_26	56992	151648
M_26	56992	259266
M_26	56992	1063
M_26	56992	7153
M_26	56992	8438
M_26	56992	9156
M_26	56992	9493
M_26	56992	22974
M_26	56992	23397
M_26	56992	54821
M_26	56992	9787
M_26	56992	10721
M_26	56992	83540

M_26	56992	146909
M_26	56992	9833
M_26	56992	55355
M_26	56992	10403
M_26	56992	3833
M_26	56992	993
M_26	56992	128239
M_26	57082	90381
M_26	57082	259266
M_26	57082	285643
M_26	57082	1063
M_26	57082	2491
M_26	57082	9824
M_26	57082	51203
M_26	57082	9700
M_26	57082	10721
M_26	57082	89839
M_26	57082	11113
M_26	57082	1062
M_26	57082	10615
M_26	57082	63967
M_26	64105	9232
M_26	64105	55789
M_26	64105	9768
M_26	64105	29089
M_26	64151	150468
M_26	64151	151648
M_26	64151	259266
M_26	64151	653820
M_26	64151	7153
M_26	64151	9156
M_26	64151	23397

M_26	64151	51203
M_26	64151	55388
M_26	64151	55635
M_26	64151	83540
M_26	64151	5347
M_26	64151	9833
M_26	64151	10403
M_26	64151	3833
M_26	64151	7272
M_26	64151	1062
M_26	79058	201254
M_26	79058	6182
M_26	79058	10189
M_26	79058	339229
M_26	79058	284184
M_26	79058	348262
M_26	79058	57332
M_26	79058	339231
M_26	80764	85359
M_26	80764	7353
M_26	80764	64976
M_26	80764	5902
M_26	80764	8220
M_26	80764	23753
M_26	80764	128977
M_26	80764	150223
M_26	80764	27037
M_26	80764	6576
M_26	80764	27440
M_26	83461	4605
M_26	83461	9232
M_26	83461	10635

M_26	85359	7353
M_26	85359	64976
M_26	85359	5902
M_26	85359	8220
M_26	85359	23753
M_26	85359	128977
M_26	85359	150223
M_26	85359	27037
M_26	85359	6576
M_26	85359	27440
M_26	90381	1063
M_26	90381	8438
M_26	90381	9055
M_26	90381	9493
M_26	90381	55215
M_26	90381	9700
M_26	90381	10721
M_26	90381	146909
M_26	90381	4288
M_26	90381	80119
M_26	90381	993
M_26	90381	128239
M_26	90381	11113
M_26	90381	63967
M_26	150468	151648
M_26	150468	259266
M_26	150468	7153
M_26	150468	22974
M_26	150468	23397
M_26	150468	51203
M_26	150468	9787
M_26	150468	55635

M_26	150468	57650
M_26	150468	83540
M_26	150468	146909
M_26	150468	983
M_26	150468	5347
M_26	150468	9833
M_26	150468	55355
M_26	150468	10403
M_26	150468	3833
M_26	150468	1062
M_26	151648	259266
M_26	151648	653820
M_26	151648	6790
M_26	151648	9493
M_26	151648	54821
M_26	151648	6491
M_26	151648	9787
M_26	151648	10721
M_26	151648	55635
M_26	151648	57650
M_26	151648	83540
M_26	151648	983
M_26	151648	9833
M_26	151648	1894
M_26	151648	7272
M_26	151648	221150
M_26	151648	81930
M_26	157570	157313
M_26	201254	6182
M_26	201254	10189
M_26	201254	339229
M_26	201254	284184

M_26	201254	348262
M_26	201254	339231
M_26	259266	653820
M_26	259266	1063
M_26	259266	7153
M_26	259266	9055
M_26	259266	9156
M_26	259266	9493
M_26	259266	22974
M_26	259266	51203
M_26	259266	729533
M_26	259266	10721
M_26	259266	146909
M_26	259266	4288
M_26	259266	4751
M_26	259266	55355
M_26	259266	128239
M_26	259266	1062
M_26	285643	2491
M_26	285643	9055
M_26	285643	54821
M_26	285643	4288
M_26	285643	128239
M_26	653820	728833
M_26	653820	6790
M_26	653820	7153
M_26	653820	51203
M_26	653820	729533
M_26	653820	83540
M_26	653820	146909
M_26	653820	983
M_26	653820	5347

M_26	653820	4751
M_26	728833	7153
M_26	728833	729533
M_26	728833	57650
M_26	1063	7153
M_26	1063	22974
M_26	1063	729533
M_26	1063	9700
M_26	1063	146909
M_26	1063	4288
M_26	1063	4751
M_26	1063	55355
M_26	1063	128239
M_26	1063	11113
M_26	1063	1062
M_26	1063	51514
M_26	1063	10615
M_26	2491	4998
M_26	2491	8438
M_26	2491	54821
M_26	2491	6491
M_26	2491	10721
M_26	2491	55143
M_26	2491	55635
M_26	2491	9833
M_26	2491	54443
M_26	4085	84057
M_26	4085	1163
M_26	4085	10733
M_26	4085	1164
M_26	4085	29089
M_26	4605	6790

M_26	4605	9156
M_26	4605	22974
M_26	4605	23397
M_26	4605	55388
M_26	4605	81610
M_26	4605	8318
M_26	4605	146909
M_26	4605	990
M_26	4605	5347
M_26	4605	113130
M_26	4605	3838
M_26	4605	1869
M_26	4605	220134
M_26	4998	8438
M_26	4998	55215
M_26	4998	55388
M_26	4998	6491
M_26	4998	55143
M_26	4998	55635
M_26	4998	990
M_26	4998	6241
M_26	4998	63967
M_26	6182	10189
M_26	6182	339229
M_26	6182	284184
M_26	6182	348262
M_26	6182	339231
M_26	6790	9133
M_26	6790	54821
M_26	6790	81610
M_26	6790	55635
M_26	6790	83540

M_26	6790	5347
M_26	6790	57405
M_26	6790	113130
M_26	6790	3838
M_26	6790	6241
M_26	7153	22974
M_26	7153	23397
M_26	7153	55388
M_26	7153	81610
M_26	7153	9787
M_26	7153	55635
M_26	7153	57650
M_26	7153	83540
M_26	7153	146909
M_26	7153	983
M_26	7153	990
M_26	7153	4751
M_26	7153	55355
M_26	7153	3833
M_26	7153	7272
M_26	7153	10615
M_26	7153	51512
M_26	7298	494514
M_26	7298	10403
M_26	7298	79019
M_26	7353	64976
M_26	7353	5902
M_26	7353	8220
M_26	7353	23753
M_26	7353	128977
M_26	7353	150223
M_26	7353	27037

M_26	7353	6576
M_26	7353	27440
M_26	8438	9156
M_26	8438	9493
M_26	8438	54821
M_26	8438	55388
M_26	8438	6491
M_26	8438	10721
M_26	8438	55143
M_26	8438	9833
M_26	8438	113130
M_26	8438	10403
M_26	8438	3833
M_26	8438	63967
M_26	8475	9232
M_26	8475	10189
M_26	8475	1033
M_26	8475	1164
M_26	8475	79019
M_26	8475	3838
M_26	8475	29089
M_26	8475	6241
M_26	9055	9133
M_26	9055	9493
M_26	9055	51203
M_26	9055	55215
M_26	9055	3833
M_26	9055	51512
M_26	9088	78995
M_26	9088	899
M_26	9088	8318
M_26	9088	5347

M_26	9088	1869
M_26	9133	9493
M_26	9133	51203
M_26	9133	9768
M_26	9133	55143
M_26	9133	983
M_26	9133	113130
M_26	9133	3838
M_26	9156	54821
M_26	9156	55388
M_26	9156	8318
M_26	9156	990
M_26	9156	113130
M_26	9156	3833
M_26	9156	1894
M_26	9156	51514
M_26	9232	1033
M_26	9232	1164
M_26	9232	79019
M_26	9232	29089
M_26	9232	3161
M_26	9493	22974
M_26	9493	51203
M_26	9493	81610
M_26	9493	9787
M_26	9493	146909
M_26	9493	9833
M_26	9493	55355
M_26	9493	113130
M_26	9493	10403
M_26	9493	80119
M_26	9493	3833

M_26	9824	51203
M_26	9824	90417
M_26	9824	89839
M_26	10189	339229
M_26	10189	284184
M_26	10189	1164
M_26	10189	348262
M_26	10189	339231
M_26	10635	55789
M_26	10635	9837
M_26	22974	23397
M_26	22974	55388
M_26	22974	81610
M_26	22974	9787
M_26	22974	146909
M_26	22974	5347
M_26	22974	9833
M_26	22974	55355
M_26	22974	113130
M_26	22974	3833
M_26	23397	55388
M_26	23397	81610
M_26	23397	9787
M_26	23397	55635
M_26	23397	57650
M_26	23397	83540
M_26	23397	146909
M_26	23397	5347
M_26	23397	9833
M_26	23397	55355
M_26	23397	113130
M_26	23397	10403

M_26	23397	3833
M_26	23397	7272
M_26	23397	10615
M_26	51203	90417
M_26	51203	983
M_26	51203	3833
M_26	54821	6491
M_26	54821	9787
M_26	54821	10721
M_26	54821	55143
M_26	54821	55635
M_26	54821	9833
M_26	54821	113130
M_26	54821	1894
M_26	54821	7272
M_26	54821	221150
M_26	55215	990
M_26	55215	993
M_26	55215	51514
M_26	55388	990
M_26	55388	9833
M_26	55388	3833
M_26	55388	7272
M_26	55732	91687
M_26	55732	9837
M_26	55732	1763
M_26	55789	29089
M_26	55789	55771
M_26	64976	5902
M_26	64976	8220
M_26	64976	23753
M_26	64976	128977

M_26	64976	150223
M_26	64976	400916
M_26	64976	27037
M_26	64976	6576
M_26	78995	146956
M_26	78995	146909
M_26	78995	990
M_26	78995	1869
M_26	78995	79172
M_26	81610	9787
M_26	81610	55635
M_26	81610	146909
M_26	81610	983
M_26	81610	5347
M_26	81610	9833
M_26	81610	55355
M_26	81610	113130
M_26	81610	3833
M_26	81610	51512
M_26	81610	79733
M_26	84057	10733
M_26	84057	29089
M_26	91687	83540
M_26	91687	9837
M_26	91687	29089
M_26	91687	55010
M_26	91687	151246
M_26	339229	284184
M_26	339229	348262
M_26	339229	339231
M_26	494514	79019
M_26	729533	4751

M_26	729533	51514
M_26	899	79172
M_26	899	10615
M_26	899	284403
M_26	1033	9787
M_26	1033	983
M_26	1033	57405
M_26	1033	1164
M_26	5902	8220
M_26	5902	8318
M_26	5902	23753
M_26	5902	128977
M_26	5902	150223
M_26	5902	10587
M_26	5902	27037
M_26	5902	6576
M_26	5902	79019
M_26	5902	27440
M_26	6491	10721
M_26	6491	55143
M_26	6491	55635
M_26	6491	9833
M_26	6491	1894
M_26	6491	220134
M_26	6491	29899
M_26	6491	54443
M_26	8220	128977
M_26	8220	150223
M_26	8220	10587
M_26	8220	27037
M_26	8220	6576
M_26	8220	27440

M_26	8318	990
M_26	8318	113130
M_26	8318	79019
M_26	8318	1869
M_26	8318	6241
M_26	9700	10721
M_26	9700	146909
M_26	9700	4288
M_26	9700	128239
M_26	9700	11113
M_26	9700	10615
M_26	9700	284403
M_26	9768	1164
M_26	9768	79019
M_26	9768	29089
M_26	9787	55635
M_26	9787	83540
M_26	9787	983
M_26	9787	5347
M_26	9787	9833
M_26	9787	4751
M_26	9787	55355
M_26	9787	10403
M_26	9787	3833
M_26	9787	7272
M_26	10721	57650
M_26	10721	146909
M_26	10721	1894
M_26	10721	128239
M_26	10721	1062
M_26	23753	150223
M_26	23753	400916

M_26	23753	6576
M_26	55143	9833
M_26	55143	113130
M_26	55143	10403
M_26	55143	63967
M_26	55635	83540
M_26	55635	983
M_26	55635	5347
M_26	55635	9833
M_26	55635	4751
M_26	55635	10403
M_26	55635	1894
M_26	55635	7272
M_26	55635	29899
M_26	57650	83540
M_26	57650	1894
M_26	57650	7272
M_26	57650	1062
M_26	57650	151246
M_26	57650	81930
M_26	83540	983
M_26	83540	57405
M_26	83540	4751
M_26	83540	55355
M_26	83540	10403
M_26	83540	29089
M_26	83540	1894
M_26	83540	7272
M_26	83540	221150
M_26	83540	81930
M_26	128977	150223
M_26	128977	27037

M_26	128977	6576
M_26	128977	27440
M_26	146909	5347
M_26	146909	55355
M_26	146909	113130
M_26	146909	3833
M_26	146909	128239
M_26	146909	10615
M_26	146909	51512
M_26	146909	284403
M_26	150223	10587
M_26	150223	27037
M_26	150223	6576
M_26	150223	27440
M_26	150223	54584
M_26	284184	348262
M_26	284184	339231
M_26	983	9833
M_26	983	57405
M_26	983	4751
M_26	983	1763
M_26	990	83903
M_26	990	1763
M_26	990	6241
M_26	990	7272
M_26	990	51514
M_26	1163	1164
M_26	1163	29089
M_26	5347	9833
M_26	5347	55355
M_26	5347	113130
M_26	5347	3833

M_26	5347	3838
M_26	9833	113130
M_26	9833	10403
M_26	9833	3833
M_26	9833	7272
M_26	9833	220134
M_26	22995	89839
M_26	57405	221150
M_26	57405	220134
M_26	79980	9837
M_26	79980	1869
M_26	3619	4288
M_26	4288	128239
M_26	4288	11113
M_26	4288	63967
M_26	4288	79733
M_26	4751	29089
M_26	4751	51514
M_26	9837	55010
M_26	10587	27037
M_26	10587	6576
M_26	10733	1062
M_26	27037	6576
M_26	55355	113130
M_26	55355	10403
M_26	55355	3833
M_26	113130	3833
M_26	113130	221150
M_26	113130	220134
M_26	1164	79019
M_26	1164	29089
M_26	10403	3833

M_26	10403	7272
M_26	10403	220134
M_26	83903	284403
M_26	348262	339231
M_26	1763	51514
M_26	1763	219790
M_26	3833	128239
M_26	3833	51512
M_26	3833	220134
M_26	3838	6241
M_26	1894	7272
M_26	1894	221150
M_26	1894	220134
M_26	1894	81930
M_26	7272	113115
M_26	7272	81930
M_26	27440	100130717
M_26	128239	1062
M_26	128239	10615
M_26	128239	51512
M_26	128239	79733
M_26	221150	81930
M_26	8535	57332
M_26	11113	10615
M_26	1062	10615
M_26	1062	151246
M_26	1062	29899
M_26	1062	54443
M_26	51514	83990
M_26	79172	83990
M_26	339231	125061
M_26	51512	54443

M_26	81930	79733
M_26	85001	125061
M_29	715	716
M_29	715	3383
M_29	716	3383
M_29	716	23213
M_29	718	6288
M_29	718	6289
M_29	718	8740
M_29	718	9180
M_29	718	4837
M_29	6288	6289
M_29	6288	6291
M_29	6288	4837
M_29	6288	55350
M_29	6289	6291
M_29	6289	4837
M_29	6289	55350
M_29	8740	9180
M_29	9953	9955
M_29	9953	84815
M_29	9953	139818
M_29	9955	84815
M_29	9955	3730
M_29	10397	56169
M_29	10397	7942
M_29	10397	7227
M_29	55062	84981
M_29	55062	5526
M_29	55062	22808
M_29	55062	1827
M_29	56169	57194

M_29	5328	414236
M_29	5328	2706
M_29	5328	147920
M_29	5328	5329
M_29	5328	23213
M_29	5732	8828
M_29	5732	57194
M_29	6291	55350
M_29	6291	9641
M_29	8828	861
M_29	8828	7421
M_29	8828	8507
M_29	8828	84171
M_29	414236	2706
M_29	414236	4837
M_29	414236	2180
M_29	861	9180
M_29	861	7421
M_29	861	2180
M_29	861	860
M_29	3566	9180
M_29	9180	4478
M_29	9180	4837
M_29	9180	7421
M_29	9180	5271
M_29	9180	355
M_29	2706	147920
M_29	2706	389336
M_29	4478	57194
M_29	5526	22808
M_29	54757	4837
M_29	90102	139818

M_29	90102	23670
M_29	139818	388650
M_29	139818	3730
M_29	139818	29940
M_29	147920	23213
M_29	147920	90853
M_29	147920	389336
M_29	4837	79883
M_29	4837	4907
M_29	4837	9023
M_29	7421	5873
M_29	23670	51474
M_29	23670	2180
M_29	2180	9764
M_29	2180	1827
M_29	7942	22898
M_29	7942	8714
M_29	9764	22885
M_29	9764	55824
M_29	55350	9641
M_29	5873	4734
M_29	355	57559
M_29	4734	57188
M_29	30846	57616
M_29	30846	22885
M_29	3274	29940
M_29	22885	8829
M_34	125	2167
M_34	125	2532
M_34	125	5346
M_34	125	6403
M_34	125	286753

M_34	125	9370
M_34	125	1149
M_34	125	6358
M_34	125	8639
M_34	125	11185
M_34	125	6366
M_34	285	11082
M_34	285	8862
M_34	285	2321
M_34	285	54567
M_34	1003	2828
M_34	1003	3672
M_34	1003	79742
M_34	1003	221395
M_34	1003	54538
M_34	1003	80177
M_34	1003	161198
M_34	1003	4811
M_34	1003	22918
M_34	1003	51294
M_34	1003	64123
M_34	1003	90952
M_34	1003	3791
M_34	1003	7450
M_34	1003	4855
M_34	1003	10044
M_34	1003	79652
M_34	1003	2022
M_34	1003	22899
M_34	1003	5787
M_34	1003	1306
M_34	1003	10501

M_34	1003	57381
M_34	1282	1284
M_34	1282	2828
M_34	1282	3672
M_34	1282	9507
M_34	1282	50509
M_34	1282	57125
M_34	1282	221395
M_34	1282	54538
M_34	1282	4811
M_34	1282	57124
M_34	1282	3910
M_34	1282	4017
M_34	1282	1306
M_34	1282	11178
M_34	1282	176
M_34	1284	1306
M_34	1359	2206
M_34	1359	2532
M_34	1359	7177
M_34	1359	64499
M_34	1359	1511
M_34	2167	286753
M_34	2167	948
M_34	2167	9370
M_34	2167	6358
M_34	2167	8639
M_34	2167	6366
M_34	2167	114757
M_34	2206	7177
M_34	2206	64499
M_34	2206	1511

M_34	2532	6403
M_34	2532	6358
M_34	2532	6401
M_34	2532	7123
M_34	2532	10268
M_34	2532	11185
M_34	2828	3672
M_34	2828	9507
M_34	2828	50509
M_34	2828	57125
M_34	2828	79742
M_34	2828	221395
M_34	2828	54538
M_34	2828	80177
M_34	2828	51294
M_34	2828	64123
M_34	2828	90952
M_34	2828	2321
M_34	2828	3791
M_34	2828	57124
M_34	2828	90332
M_34	2828	3910
M_34	2828	11178
M_34	3672	221395
M_34	3672	4811
M_34	3672	22918
M_34	3672	64123
M_34	3672	90952
M_34	3672	3791
M_34	3672	254228
M_34	4360	30835
M_34	4360	10990

M_34	4360	2350
M_34	4360	2162
M_34	4360	1601
M_34	5346	286753
M_34	5346	9370
M_34	5346	1149
M_34	5346	3952
M_34	5346	3929
M_34	6403	6358
M_34	6403	6401
M_34	6403	10268
M_34	6403	11185
M_34	7177	64499
M_34	7177	23430
M_34	9507	57124
M_34	9507	3910
M_34	9507	3678
M_34	9507	11178
M_34	9839	25900
M_34	9839	118429
M_34	9839	11010
M_34	9839	22918
M_34	9839	57514
M_34	9839	54331
M_34	9839	1601
M_34	9839	7045
M_34	9839	10184
M_34	11082	8862
M_34	11082	54567
M_34	11117	25960
M_34	11117	51313
M_34	11117	118429

M_34	11117	3090
M_34	11117	25927
M_34	11117	5159
M_34	11117	10186
M_34	11117	6935
M_34	11117	283298
M_34	25900	3603
M_34	25960	118429
M_34	25960	3090
M_34	25960	25927
M_34	25960	4811
M_34	25960	5159
M_34	25960	10186
M_34	25960	6935
M_34	25960	2022
M_34	25960	283298
M_34	30835	10990
M_34	30835	2350
M_34	30835	2162
M_34	30835	940
M_34	50509	57125
M_34	50509	5159
M_34	50509	4017
M_34	51313	118429
M_34	51313	3090
M_34	51313	25927
M_34	51313	1842
M_34	51313	10186
M_34	51313	6935
M_34	57125	4811
M_34	57125	5159
M_34	57125	57124

M_34	57125	4017
M_34	57125	23452
M_34	57125	11178
M_34	64499	23430
M_34	79742	221395
M_34	79742	54538
M_34	79742	80177
M_34	79742	161198
M_34	79742	4811
M_34	79742	22918
M_34	79742	51294
M_34	79742	64123
M_34	79742	90952
M_34	79742	3791
M_34	79742	6358
M_34	79742	7450
M_34	79742	375033
M_34	79742	641700
M_34	79742	10044
M_34	79742	51705
M_34	79742	79652
M_34	79742	116441
M_34	79742	2022
M_34	79742	8490
M_34	79742	22899
M_34	79742	10501
M_34	79742	9079
M_34	79742	255877
M_34	79742	187
M_34	118429	3090
M_34	118429	25927
M_34	118429	1842

M_34	118429	10186
M_34	118429	6935
M_34	126668	126669
M_34	126669	7450
M_34	126669	375033
M_34	221395	54538
M_34	221395	80177
M_34	221395	4811
M_34	221395	22918
M_34	221395	51294
M_34	221395	64123
M_34	221395	90952
M_34	221395	2321
M_34	221395	3791
M_34	221395	22795
M_34	221395	552
M_34	221395	1901
M_34	286753	9370
M_34	286753	1149
M_34	948	8639
M_34	948	51705
M_34	948	64641
M_34	3090	25927
M_34	3090	1842
M_34	3090	4811
M_34	3090	5159
M_34	3090	10186
M_34	3090	6935
M_34	3090	283298
M_34	6362	6357
M_34	6915	126326
M_34	6915	10044

M_34	6915	392617
M_34	6915	255877
M_34	9370	1149
M_34	10990	2350
M_34	10990	6357
M_34	11010	54331
M_34	11010	23643
M_34	11010	7045
M_34	11010	11031
M_34	25927	1842
M_34	25927	10186
M_34	25927	6935
M_34	25927	283298
M_34	25927	54331
M_34	54538	80177
M_34	54538	126326
M_34	54538	161198
M_34	54538	4811
M_34	54538	51294
M_34	54538	64123
M_34	54538	90952
M_34	54538	3791
M_34	54538	7450
M_34	54538	22795
M_34	54538	57124
M_34	54538	4855
M_34	54538	10044
M_34	54538	79652
M_34	54538	22899
M_34	54538	90332
M_34	54538	1306
M_34	54538	10501

M_34	54538	255877
M_34	54538	187
M_34	80177	161198
M_34	80177	51294
M_34	80177	64123
M_34	80177	90952
M_34	80177	3791
M_34	80177	641700
M_34	80177	7123
M_34	80177	51705
M_34	80177	79652
M_34	80177	116441
M_34	80177	8490
M_34	80177	22899
M_34	80177	5787
M_34	80177	8404
M_34	80177	10203
M_34	80177	51267
M_34	80177	9079
M_34	80177	1901
M_34	126326	90952
M_34	126326	375033
M_34	126326	4855
M_34	126326	10044
M_34	126326	22899
M_34	126326	90332
M_34	126326	10501
M_34	161198	64123
M_34	161198	90952
M_34	161198	6358
M_34	161198	7122
M_34	161198	641700

M_34	161198	10044
M_34	161198	51705
M_34	161198	79652
M_34	161198	116441
M_34	161198	2022
M_34	161198	8490
M_34	161198	10268
M_34	161198	22899
M_34	161198	5787
M_34	161198	8404
M_34	161198	10203
M_34	161198	51267
M_34	161198	1901
M_34	161198	10750
M_34	1842	10186
M_34	1842	6935
M_34	1842	283298
M_34	2350	2162
M_34	2350	1601
M_34	4811	51294
M_34	4811	64123
M_34	4811	5159
M_34	4811	10186
M_34	4811	22795
M_34	4811	375033
M_34	4811	79652
M_34	4811	3910
M_34	4811	23452
M_34	4811	1306
M_34	4811	11178
M_34	8862	57574
M_34	22918	51294

M_34	22918	57514
M_34	22918	2162
M_34	22918	9754
M_34	22918	2022
M_34	22918	1601
M_34	22918	7056
M_34	22918	10019
M_34	22936	132720
M_34	22936	4121
M_34	22936	29969
M_34	22936	10924
M_34	51294	64123
M_34	51294	90952
M_34	51294	3791
M_34	51294	7450
M_34	51294	9754
M_34	51294	22795
M_34	51294	4855
M_34	51294	10044
M_34	51294	2022
M_34	51294	22899
M_34	51294	1306
M_34	51294	10501
M_34	51294	255877
M_34	51751	143503
M_34	51751	116441
M_34	51751	8490
M_34	51751	221303
M_34	57514	9754
M_34	57514	54331
M_34	57514	1601
M_34	57514	10154

M_34	64123	90952
M_34	64123	10186
M_34	64123	57124
M_34	64123	641700
M_34	64123	51705
M_34	64123	79652
M_34	64123	116441
M_34	64123	51267
M_34	64123	10501
M_34	64123	9079
M_34	64123	5414
M_34	90952	57124
M_34	90952	641700
M_34	90952	4855
M_34	90952	10044
M_34	90952	79652
M_34	90952	116441
M_34	90952	22899
M_34	90952	51267
M_34	90952	90332
M_34	90952	10501
M_34	90952	5414
M_34	90952	255877
M_34	132720	5468
M_34	132720	59345
M_34	132720	5414
M_34	132720	10924
M_34	132720	5175
M_34	132720	2040
M_34	143503	81285
M_34	2162	54331
M_34	2162	1601

M_34	2321	3791
M_34	2321	4855
M_34	2321	54567
M_34	2321	5787
M_34	2321	90332
M_34	3791	4855
M_34	3791	8490
M_34	3791	22899
M_34	3791	90332
M_34	5159	10186
M_34	5159	57124
M_34	5159	6935
M_34	5159	23452
M_34	5159	11178
M_34	5468	81537
M_34	5468	115123
M_34	5468	9936
M_34	6358	7122
M_34	6358	641700
M_34	6358	7123
M_34	6358	51705
M_34	6358	8490
M_34	6358	11185
M_34	6358	114757
M_34	7122	641700
M_34	7122	7123
M_34	7122	10268
M_34	7122	256949
M_34	7122	114757
M_34	7450	375033
M_34	7450	4855
M_34	7450	54567

M_34	7450	2022
M_34	7450	22899
M_34	7450	5787
M_34	7450	167465
M_34	7450	5138
M_34	9754	94
M_34	9754	3142
M_34	9754	10044
M_34	9754	8728
M_34	9754	170954
M_34	9754	54331
M_34	9754	1601
M_34	10186	6935
M_34	10186	79652
M_34	10186	283298
M_34	10186	23452
M_34	22795	3910
M_34	22795	1306
M_34	57124	11178
M_34	57124	10501
M_34	81537	283537
M_34	375033	4855
M_34	375033	22899
M_34	375033	2869
M_34	375033	27115
M_34	375033	114757
M_34	375033	57381
M_34	641700	7123
M_34	641700	51705
M_34	641700	79652
M_34	641700	116441
M_34	641700	10268

M_34	641700	10203
M_34	641700	51267
M_34	641700	10750
M_34	641700	7035
M_34	94	3142
M_34	94	10044
M_34	94	51267
M_34	94	10501
M_34	3142	10044
M_34	3142	10501
M_34	3142	83552
M_34	4855	10044
M_34	4855	54567
M_34	4855	22899
M_34	4855	5787
M_34	4855	90332
M_34	4855	7075
M_34	4855	167465
M_34	4855	1464
M_34	4855	255877
M_34	6935	283298
M_34	6935	23452
M_34	7123	51705
M_34	7123	116441
M_34	7123	10268
M_34	7123	8611
M_34	8639	5414
M_34	8639	187
M_34	8639	4223
M_34	10044	79652
M_34	10044	2022
M_34	10044	22899

M_34	10044	90332
M_34	10044	2869
M_34	10044	8291
M_34	10044	5414
M_34	10044	10750
M_34	10044	8578
M_34	51705	116441
M_34	51705	8490
M_34	51705	10203
M_34	51705	9079
M_34	51705	8611
M_34	51705	1901
M_34	51705	6886
M_34	54567	7075
M_34	54567	1464
M_34	79652	22899
M_34	79652	51267
M_34	79652	90332
M_34	79652	10501
M_34	79652	9079
M_34	79652	5414
M_34	116441	8490
M_34	2022	22899
M_34	4121	29969
M_34	4121	59345
M_34	8490	114757
M_34	8490	1901
M_34	8490	57381
M_34	8490	221303
M_34	8728	170954
M_34	8728	3678
M_34	8728	2113

M_34	9194	29969
M_34	10268	358
M_34	10268	7035
M_34	11185	8404
M_34	11185	2202
M_34	22899	167465
M_34	29969	6489
M_34	5787	8404
M_34	5787	10203
M_34	5787	167465
M_34	5787	7010
M_34	5787	57381
M_34	8404	10203
M_34	8404	1901
M_34	10203	1901
M_34	10203	7035
M_34	54331	1601
M_34	54331	4094
M_34	54331	7045
M_34	54331	4208
M_34	90332	10501
M_34	90332	392617
M_34	2869	8291
M_34	3910	11178
M_34	4017	23452
M_34	4017	3678
M_34	4017	11178
M_34	23452	3678
M_34	1601	4094
M_34	1601	7045
M_34	3678	7045
M_34	3678	11031

M_34	7075	255877
M_34	1306	187
M_34	7056	9060
M_34	9936	84329
M_34	81035	122786
M_34	81035	7045
M_34	81035	11031
M_34	8291	255877
M_34	9627	27115
M_34	167465	8516
M_34	176	552
M_34	552	63895
M_34	7045	11031
M_34	7010	6886
M_34	8611	1901
M_34	10750	4005
M_34	57381	2078
M_34	358	187
M_34	2078	947
M_47	1124	9865
M_47	1124	54504
M_47	1124	150
M_47	7025	23768
M_47	7025	83872
M_47	7025	441094
M_47	7025	22836
M_47	8642	8913
M_47	8642	11173
M_47	8642	775
M_47	8642	10052
M_47	8642	11167
M_47	8642	27147

M_47	8642	57722
M_47	8642	3912
M_47	8642	81794
M_47	8642	91179
M_47	8642	441631
M_47	8642	54551
M_47	8642	285590
M_47	8913	11173
M_47	8913	23768
M_47	8913	83872
M_47	8913	57722
M_47	8913	344558
M_47	8913	54551
M_47	9464	79804
M_47	9464	56849
M_47	9865	222865
M_47	9865	54551
M_47	9865	7837
M_47	11173	775
M_47	11173	22836
M_47	11173	10052
M_47	11173	11167
M_47	11173	27147
M_47	11173	57722
M_47	11173	3912
M_47	11173	81794
M_47	11173	91179
M_47	11173	441631
M_47	11173	54551
M_47	11173	8482
M_47	23768	55384
M_47	23768	83872

M_47	23768	220164
M_47	23768	51661
M_47	23768	196740
M_47	23768	203447
M_47	23768	22836
M_47	23768	8532
M_47	23768	57722
M_47	23768	79633
M_47	23768	54551
M_47	23768	89795
M_47	55384	728264
M_47	55384	57722
M_47	55384	388015
M_47	55384	79104
M_47	83872	22836
M_47	83872	6925
M_47	83872	8532
M_47	83872	57722
M_47	83872	79633
M_47	83872	114905
M_47	83872	344558
M_47	83872	54551
M_47	83872	7837
M_47	85407	124842
M_47	85407	155051
M_47	85407	222865
M_47	85407	349136
M_47	124842	400591
M_47	124842	55691
M_47	155051	349136
M_47	220164	196740
M_47	220164	203447

M_47	220164	79633
M_47	220164	862
M_47	222865	349136
M_47	728264	57722
M_47	4239	51661
M_47	4239	2006
M_47	4239	6925
M_47	4239	338773
M_47	4239	1038
M_47	51661	22836
M_47	51661	6925
M_47	51661	10234
M_47	51661	54551
M_47	51661	10082
M_47	196740	203447
M_47	196740	1272
M_47	196740	57722
M_47	196740	55022
M_47	196740	114905
M_47	196740	8646
M_47	196740	55959
M_47	203447	1272
M_47	203447	221091
M_47	203447	8532
M_47	203447	55022
M_47	203447	114905
M_47	203447	55959
M_47	775	81794
M_47	775	441631
M_47	775	144347
M_47	775	55083
M_47	775	9891

M_47	1272	221091
M_47	1272	8532
M_47	1272	55022
M_47	1272	114905
M_47	22836	6925
M_47	22836	11167
M_47	22836	27147
M_47	22836	57722
M_47	22836	54551
M_47	221091	114905
M_47	221091	8646
M_47	221091	89795
M_47	5654	8532
M_47	5654	114905
M_47	6925	3912
M_47	8532	344558
M_47	10052	11167
M_47	10052	27147
M_47	10052	285382
M_47	11167	27147
M_47	11167	3912
M_47	11167	91179
M_47	11167	441631
M_47	11167	9638
M_47	11167	8406
M_47	27147	57722
M_47	27147	441631
M_47	27147	285382
M_47	3912	144347
M_47	55022	8646
M_47	55022	25817
M_47	79633	89795

M_47	79633	27303
M_47	114905	83700
M_47	8646	55959
M_47	81794	91179
M_47	81794	285590
M_47	344558	441631
M_47	344558	57537
M_47	388015	79104
M_47	441631	117581
M_47	441631	94031
M_47	25817	57537
M_47	25817	64094
M_47	57537	55959
M_47	57537	56937
M_47	285382	83700
M_47	56944	338773
M_47	56944	558
M_47	558	5452
M_47	9638	83700
M_47	9638	114898
M_47	9638	56849
M_47	54551	7837
M_47	54551	10082
M_47	83700	55959
M_47	7043	144347
M_47	144347	6695
M_47	8406	94031
M_47	285590	7837
M_47	64094	64388
M_91	2850	8693
M_91	2850	57535
M_91	2850	282809

M_91	2850	317649
M_91	2850	60675
M_91	4246	10647
M_91	4246	25803
M_91	4246	93035
M_91	4246	80150
M_91	4246	84281
M_91	4246	4250
M_91	4246	10648
M_91	4246	64798
M_91	4246	10164
M_91	5016	25803
M_91	5016	55638
M_91	5016	57535
M_91	5016	79084
M_91	5016	93035
M_91	5016	51268
M_91	5016	80150
M_91	5016	64798
M_91	5016	9368
M_91	5016	647309
M_91	8534	374387
M_91	8534	27199
M_91	8534	79899
M_91	8693	25803
M_91	8693	55638
M_91	8693	57535
M_91	8693	93035
M_91	8693	282809
M_91	8693	317649
M_91	8693	80150
M_91	8693	84281

M_91	8693	64798
M_91	9220	399687
M_91	9220	51268
M_91	9220	124925
M_91	10647	10753
M_91	10647	80150
M_91	10647	84281
M_91	10647	4250
M_91	10647	10648
M_91	10647	64798
M_91	25803	55638
M_91	25803	57535
M_91	25803	93035
M_91	25803	282809
M_91	25803	29993
M_91	25803	80150
M_91	25803	84281
M_91	25803	64798
M_91	25803	9368
M_91	25803	79899
M_91	55638	57535
M_91	55638	93035
M_91	55638	282809
M_91	55638	80150
M_91	55638	64798
M_91	55638	23604
M_91	57535	79084
M_91	57535	93035
M_91	57535	282809
M_91	57535	80150
M_91	57535	84281
M_91	57535	64798

M_91	57535	9368
M_91	57535	866
M_91	79084	29993
M_91	93035	282809
M_91	93035	317649
M_91	93035	3680
M_91	93035	80150
M_91	93035	81501
M_91	93035	84281
M_91	93035	4250
M_91	93035	64798
M_91	93035	647309
M_91	93035	23604
M_91	96626	100271835
M_91	96626	3987
M_91	199974	260293
M_91	199974	163720
M_91	199974	27199
M_91	199974	284541
M_91	260293	163720
M_91	260293	284541
M_91	260293	79899
M_91	282809	317649
M_91	282809	84281
M_91	282809	64798
M_91	317649	3680
M_91	317649	60675
M_91	317649	152273
M_91	317649	9071
M_91	374387	79899
M_91	399687	51268
M_91	399687	124925

M_91	100271835	3987
M_91	100271835	23657
M_91	3680	60675
M_91	3680	152273
M_91	3680	221184
M_91	3680	196883
M_91	3680	4147
M_91	9699	81501
M_91	9699	22824
M_91	9699	9848
M_91	9699	4147
M_91	10753	92291
M_91	29993	23066
M_91	51268	124925
M_91	51268	284415
M_91	60675	5105
M_91	80150	84281
M_91	80150	4250
M_91	80150	10648
M_91	80150	64798
M_91	81501	79899
M_91	81501	284415
M_91	84281	64798
M_91	152273	221184
M_91	152273	196883
M_91	152273	6533
M_91	163720	284541
M_91	4250	10648
M_91	4250	64798
M_91	4250	10164
M_91	23657	27199
M_91	64798	8814

M_91	221184	6376
M_91	221184	9071
M_91	5105	23305
M_91	9368	163732
M_91	9368	9154
M_91	79899	51776
M_91	8975	22824
M_91	8975	51555
M_91	647309	8814
M_91	9848	51301
M_91	9848	7499
M_91	51301	53832
M_91	196883	283849
M_91	23604	9154
M_91	3708	6533
M_91	9071	30061
M_91	3655	51776
M_91	3655	55605
M_102	1277	1289
M_102	1277	1291
M_102	1277	1292
M_102	1277	7058
M_102	1277	1281
M_102	1277	1290
M_102	1277	1893
M_102	1277	2200
M_102	1277	6678
M_102	1277	1278
M_102	1277	1293
M_102	1277	1295
M_102	1277	4060
M_102	1277	22801

M_102	1277	1462
M_102	1277	2335
M_102	1277	6591
M_102	1277	8038
M_102	1277	81792
M_102	1277	23704
M_102	1277	84627
M_102	1277	165
M_102	1277	1634
M_102	1277	9509
M_102	1277	4313
M_102	1277	83716
M_102	1277	11227
M_102	1289	1291
M_102	1289	1292
M_102	1289	7058
M_102	1289	1281
M_102	1289	1290
M_102	1289	2200
M_102	1289	6678
M_102	1289	1278
M_102	1289	1293
M_102	1289	1295
M_102	1289	4060
M_102	1289	22801
M_102	1289	1462
M_102	1289	2335
M_102	1289	6591
M_102	1289	8038
M_102	1289	81792
M_102	1289	23704
M_102	1289	84627

M_102	1289	165
M_102	1289	9509
M_102	1289	4313
M_102	1289	83716
M_102	1289	11227
M_102	1291	1292
M_102	1291	1278
M_102	1291	1293
M_102	1291	6591
M_102	1291	84627
M_102	1291	114904
M_102	1292	1281
M_102	1292	1893
M_102	1292	6678
M_102	1292	1278
M_102	1292	1293
M_102	1292	6591
M_102	1292	84627
M_102	1292	165
M_102	1292	1634
M_102	1292	9509
M_102	1292	4313
M_102	3624	7058
M_102	3624	1281
M_102	3624	1290
M_102	3624	1513
M_102	3624	1893
M_102	3624	2191
M_102	3624	4320
M_102	3624	5396
M_102	3624	1295
M_102	3624	1300

M_102	3624	4060
M_102	3624	22801
M_102	3624	1462
M_102	3624	131578
M_102	3624	2335
M_102	3624	8038
M_102	3624	10631
M_102	3624	1301
M_102	3624	23704
M_102	3624	165
M_102	3624	4313
M_102	3624	83716
M_102	3624	6423
M_102	3624	4015
M_102	3624	115701
M_102	7058	1281
M_102	7058	1290
M_102	7058	1513
M_102	7058	1893
M_102	7058	2191
M_102	7058	2200
M_102	7058	6678
M_102	7058	9242
M_102	7058	1278
M_102	7058	1295
M_102	7058	4060
M_102	7058	22801
M_102	7058	1462
M_102	7058	131578
M_102	7058	2335
M_102	7058	8038
M_102	7058	10631

M_102	7058	1301
M_102	7058	23704
M_102	7058	165
M_102	7058	9509
M_102	7058	4313
M_102	7058	83716
M_102	7058	4015
M_102	7058	115701
M_102	1281	1290
M_102	1281	1893
M_102	1281	2191
M_102	1281	2200
M_102	1281	5396
M_102	1281	6678
M_102	1281	1278
M_102	1281	1293
M_102	1281	1295
M_102	1281	4060
M_102	1281	22801
M_102	1281	1462
M_102	1281	2335
M_102	1281	6591
M_102	1281	8038
M_102	1281	81792
M_102	1281	1301
M_102	1281	23704
M_102	1281	165
M_102	1281	1634
M_102	1281	9509
M_102	1281	4313
M_102	1281	83716
M_102	1281	11227

M_102	1290	1513
M_102	1290	1893
M_102	1290	2191
M_102	1290	2200
M_102	1290	5396
M_102	1290	6678
M_102	1290	1278
M_102	1290	1293
M_102	1290	4060
M_102	1290	22801
M_102	1290	1462
M_102	1290	2335
M_102	1290	6591
M_102	1290	8038
M_102	1290	10631
M_102	1290	25903
M_102	1290	81792
M_102	1290	1301
M_102	1290	23704
M_102	1290	165
M_102	1290	9509
M_102	1290	4313
M_102	1290	83716
M_102	1513	1893
M_102	1513	2191
M_102	1513	4320
M_102	1513	5176
M_102	1513	5396
M_102	1513	4060
M_102	1513	22801
M_102	1513	1462
M_102	1513	131578

M_102	1513	2335
M_102	1513	10631
M_102	1513	25903
M_102	1513	54829
M_102	1513	1301
M_102	1513	50863
M_102	1513	1634
M_102	1513	4313
M_102	1513	6423
M_102	1893	2191
M_102	1893	4320
M_102	1893	5396
M_102	1893	6678
M_102	1893	9242
M_102	1893	4060
M_102	1893	22801
M_102	1893	1462
M_102	1893	131578
M_102	1893	2335
M_102	1893	8038
M_102	1893	10631
M_102	1893	25903
M_102	1893	81792
M_102	1893	9509
M_102	1893	4313
M_102	2191	5396
M_102	2191	6356
M_102	2191	7130
M_102	2191	9242
M_102	2191	1295
M_102	2191	1300
M_102	2191	4060

M_102	2191	22801
M_102	2191	1462
M_102	2191	131578
M_102	2191	2335
M_102	2191	8038
M_102	2191	10631
M_102	2191	25903
M_102	2191	1301
M_102	2191	50863
M_102	2191	9509
M_102	2191	203430
M_102	2191	6423
M_102	2191	4015
M_102	2191	115701
M_102	2200	6678
M_102	2200	1278
M_102	2200	1293
M_102	2200	1295
M_102	2200	4060
M_102	2200	22801
M_102	2200	1462
M_102	2200	6591
M_102	2200	8038
M_102	2200	81792
M_102	2200	23704
M_102	2200	165
M_102	2200	1634
M_102	2200	9509
M_102	2200	4313
M_102	2200	83716
M_102	4320	4060
M_102	4320	131578

M_102	4320	8038
M_102	4320	10631
M_102	4320	81792
M_102	4320	1301
M_102	4320	4313
M_102	4320	4322
M_102	4320	81832
M_102	4320	1734
M_102	4320	134111
M_102	4320	25907
M_102	4320	84466
M_102	4320	114904
M_102	5176	5396
M_102	5176	9242
M_102	5176	25903
M_102	5176	54829
M_102	5176	1634
M_102	5176	7412
M_102	5396	4060
M_102	5396	22801
M_102	5396	1462
M_102	5396	131578
M_102	5396	6591
M_102	5396	8038
M_102	5396	10631
M_102	5396	25903
M_102	5396	54829
M_102	5396	23704
M_102	5396	50863
M_102	5396	1634
M_102	5396	1909
M_102	5396	83716

M_102	6356	7130
M_102	6356	1300
M_102	6356	6999
M_102	6356	50863
M_102	6356	203430
M_102	6356	27319
M_102	6678	1278
M_102	6678	1293
M_102	6678	4060
M_102	6678	22801
M_102	6678	1462
M_102	6678	6591
M_102	6678	8038
M_102	6678	25903
M_102	6678	81792
M_102	6678	1301
M_102	6678	23704
M_102	6678	165
M_102	6678	1634
M_102	6678	9509
M_102	6678	4313
M_102	6678	83716
M_102	6678	114904
M_102	7130	6999
M_102	7130	4322
M_102	7130	5739
M_102	7130	8877
M_102	7130	27319
M_102	7130	26585
M_102	9242	10631
M_102	9242	7412
M_102	9242	5739

M_102	9242	4015
M_102	1278	1293
M_102	1278	1295
M_102	1278	22801
M_102	1278	1462
M_102	1278	6591
M_102	1278	8038
M_102	1278	81792
M_102	1278	23704
M_102	1278	84627
M_102	1278	165
M_102	1278	1634
M_102	1278	4313
M_102	1278	11227
M_102	1293	22801
M_102	1293	1462
M_102	1293	6591
M_102	1293	8038
M_102	1293	81792
M_102	1293	23704
M_102	1293	84627
M_102	1293	165
M_102	1293	1634
M_102	1293	9509
M_102	1293	4313
M_102	1295	1300
M_102	1295	4060
M_102	1295	22801
M_102	1295	1462
M_102	1295	2335
M_102	1295	165
M_102	1295	1634

M_102	1295	4313
M_102	1300	22801
M_102	1300	131578
M_102	1300	9358
M_102	1300	50863
M_102	1300	203430
M_102	1300	4958
M_102	1300	1833
M_102	4060	22801
M_102	4060	1462
M_102	4060	131578
M_102	4060	6591
M_102	4060	8038
M_102	4060	10631
M_102	4060	54829
M_102	4060	81792
M_102	4060	1301
M_102	4060	23704
M_102	4060	1634
M_102	4060	9509
M_102	4060	4313
M_102	4060	83716
M_102	4060	6423
M_102	22801	1462
M_102	22801	131578
M_102	22801	2335
M_102	22801	23704
M_102	22801	165
M_102	22801	1634
M_102	22801	9509
M_102	22801	4313
M_102	1462	2335

M_102	1462	6591
M_102	1462	8038
M_102	1462	25903
M_102	1462	81792
M_102	1462	23704
M_102	1462	165
M_102	1462	1634
M_102	1462	4313
M_102	1462	83716
M_102	1462	11227
M_102	6999	4322
M_102	6999	81832
M_102	6999	27319
M_102	6999	51554
M_102	6999	131873
M_102	6999	2982
M_102	131578	2335
M_102	131578	8038
M_102	131578	10631
M_102	131578	1301
M_102	131578	9358
M_102	131578	50863
M_102	131578	4313
M_102	131578	203430
M_102	131578	6423
M_102	131578	11227
M_102	2335	8038
M_102	2335	25903
M_102	2335	81792
M_102	2335	1301
M_102	2335	165
M_102	2335	9509

M_102	2335	4313
M_102	2335	4015
M_102	2335	115701
M_102	6591	8038
M_102	6591	81792
M_102	6591	23704
M_102	6591	1634
M_102	6591	9509
M_102	6591	4313
M_102	8038	10631
M_102	8038	25903
M_102	8038	81792
M_102	8038	1301
M_102	8038	23704
M_102	8038	9509
M_102	8038	4313
M_102	8038	83716
M_102	8038	4015
M_102	10631	25903
M_102	10631	54829
M_102	10631	81792
M_102	10631	1301
M_102	10631	50863
M_102	10631	9509
M_102	10631	6423
M_102	10631	4015
M_102	10631	115701
M_102	10631	25907
M_102	10631	26585
M_102	25903	54829
M_102	25903	81792
M_102	25903	9509

M_102	54829	1301
M_102	54829	1909
M_102	54829	83716
M_102	54829	6423
M_102	81792	1301
M_102	81792	23704
M_102	81792	165
M_102	81792	9509
M_102	81792	4313
M_102	81792	83716
M_102	81792	11227
M_102	579	85409
M_102	579	285548
M_102	1301	4313
M_102	1301	83716
M_102	1301	6423
M_102	1301	115701
M_102	1301	26585
M_102	9358	50863
M_102	9358	203430
M_102	9358	1833
M_102	9547	85409
M_102	9547	6423
M_102	9547	140862
M_102	9547	2674
M_102	23704	165
M_102	23704	9509
M_102	23704	4313
M_102	23704	83716
M_102	23704	4015
M_102	50863	203430
M_102	50863	6423

M_102	50863	1833
M_102	84627	165
M_102	85409	4016
M_102	165	1634
M_102	165	9509
M_102	165	4313
M_102	1634	4313
M_102	1634	4958
M_102	7412	5739
M_102	9509	83716
M_102	4313	83716
M_102	4322	81832
M_102	4322	1734
M_102	4322	51554
M_102	4322	131873
M_102	4322	26585
M_102	4322	84466
M_102	81832	1734
M_102	81832	51554
M_102	81832	131873
M_102	81832	256076
M_102	1734	10699
M_102	1734	134111
M_102	1734	51554
M_102	1734	26585
M_102	1909	5592
M_102	5739	8877
M_102	5739	8111
M_102	8877	25907
M_102	203430	1833
M_102	134111	25907
M_102	83716	4015

M_102	51554	2982
M_102	6423	115701
M_102	6423	26585
M_102	6423	115908
M_102	131873	2982
M_102	131873	113157
M_102	131873	256076
M_102	4016	10417
M_102	11227	114904
M_116	244	3853
M_116	244	7051
M_116	244	653145
M_116	244	3854
M_116	244	728643
M_116	244	646
M_116	302	304
M_116	302	1410
M_116	302	3853
M_116	302	79413
M_116	302	2012
M_116	302	3675
M_116	302	301
M_116	302	7052
M_116	304	3853
M_116	304	79413
M_116	304	286887
M_116	304	2012
M_116	304	3675
M_116	304	147
M_116	304	113146
M_116	304	301
M_116	304	7052

M_116	304	1803
M_116	841	843
M_116	841	4065
M_116	841	2820
M_116	843	3554
M_116	843	8837
M_116	843	366
M_116	857	858
M_116	857	10468
M_116	857	10769
M_116	857	79148
M_116	858	4233
M_116	858	10769
M_116	858	79689
M_116	1410	3316
M_116	1410	7051
M_116	1410	113026
M_116	1410	10468
M_116	1730	8808
M_116	1730	29935
M_116	3316	4240
M_116	3316	7051
M_116	3316	10468
M_116	3316	79148
M_116	3316	143903
M_116	3554	8808
M_116	3554	8809
M_116	3696	346389
M_116	3696	388115
M_116	3696	7498
M_116	3762	219833
M_116	3762	389337

M_116	3853	7051
M_116	3853	23650
M_116	3853	286887
M_116	3853	653145
M_116	3853	3854
M_116	3853	144501
M_116	3853	113146
M_116	3909	3918
M_116	3909	4240
M_116	3909	9076
M_116	3909	10686
M_116	3909	79413
M_116	3909	346389
M_116	3909	2012
M_116	3909	3675
M_116	3909	3914
M_116	3909	266977
M_116	3918	9076
M_116	3918	10686
M_116	3918	79413
M_116	3918	1969
M_116	3918	2012
M_116	3918	3675
M_116	3918	3914
M_116	3918	144501
M_116	3918	5493
M_116	3918	301
M_116	3918	266977
M_116	4065	8808
M_116	4065	2820
M_116	4065	8528
M_116	4233	9076

M_116	4233	26136
M_116	4240	9076
M_116	4240	10686
M_116	4240	145864
M_116	4240	147
M_116	4240	7348
M_116	4240	1803
M_116	7051	9806
M_116	7051	64090
M_116	7051	79413
M_116	7051	80740
M_116	7051	113026
M_116	7051	3675
M_116	7051	144501
M_116	7051	388115
M_116	7051	113146
M_116	7051	366
M_116	7051	824
M_116	8808	2820
M_116	8808	151009
M_116	9076	10686
M_116	9076	54438
M_116	9076	1969
M_116	9076	3556
M_116	9076	390
M_116	9076	53836
M_116	9076	366
M_116	9076	7052
M_116	9806	79413
M_116	9806	113026
M_116	9806	286204
M_116	9806	3675

M_116	9806	57636
M_116	9806	388115
M_116	9806	51421
M_116	9806	79148
M_116	9806	53405
M_116	10686	79413
M_116	10686	3556
M_116	10686	9213
M_116	23650	113026
M_116	23650	286887
M_116	23650	144501
M_116	23650	113146
M_116	23650	56241
M_116	23650	10076
M_116	26136	8796
M_116	29935	26548
M_116	29935	145474
M_116	54438	85411
M_116	57118	58985
M_116	57118	163702
M_116	57118	283070
M_116	57118	10468
M_116	57149	123879
M_116	57149	51315
M_116	58985	163702
M_116	58985	283070
M_116	58985	127294
M_116	64090	129807
M_116	64090	388115
M_116	64090	824
M_116	79413	80740
M_116	79413	113026

M_116	79413	3675
M_116	79413	144501
M_116	79413	113146
M_116	80739	80740
M_116	113026	113146
M_116	123879	366
M_116	163702	283070
M_116	219833	388115
M_116	221393	222611
M_116	221393	3675
M_116	221393	3694
M_116	221393	301
M_116	221393	7498
M_116	221393	53836
M_116	221393	7851
M_116	221393	8796
M_116	221393	7348
M_116	222611	7348
M_116	286204	57636
M_116	286204	79148
M_116	286204	169611
M_116	286204	140685
M_116	286204	53405
M_116	286887	3854
M_116	346389	388115
M_116	346389	5493
M_116	346389	266977
M_116	653145	3854
M_116	653145	728643
M_116	653145	646
M_116	653145	79689
M_116	653145	345651

M_116	1969	3675
M_116	1969	57636
M_116	1969	144501
M_116	1969	10769
M_116	1969	390
M_116	1969	5493
M_116	1969	51421
M_116	1969	10626
M_116	1969	7052
M_116	2012	147
M_116	2012	390
M_116	2012	5493
M_116	2012	301
M_116	2012	8796
M_116	2012	7052
M_116	2820	151009
M_116	2820	4929
M_116	3556	9213
M_116	3675	57636
M_116	3675	144501
M_116	3675	113146
M_116	3675	5493
M_116	3675	7052
M_116	3675	84687
M_116	3694	301
M_116	3914	5493
M_116	3914	7498
M_116	3914	266977
M_116	57636	113146
M_116	57636	51421
M_116	57636	169611
M_116	57636	140685

M_116	57636	53405
M_116	144501	388115
M_116	144501	5493
M_116	144501	10626
M_116	144501	266977
M_116	144501	345651
M_116	388115	113146
M_116	388115	5493
M_116	147	7348
M_116	10468	366
M_116	10468	143903
M_116	10468	875
M_116	10769	390
M_116	10769	7052
M_116	10769	345651
M_116	113146	5493
M_116	390	51421
M_116	390	53836
M_116	390	1906
M_116	5493	10076
M_116	51421	10626
M_116	51421	26524
M_116	51421	1906
M_116	56241	53947
M_116	56241	10076
M_116	79148	824
M_116	7498	100126791
M_116	53836	8796
M_116	53836	10626
M_116	53836	266977
M_116	5918	56925
M_116	5918	79689

M_116	8796	266977
M_116	8796	7348
M_116	10626	374286
M_116	79689	7980
M_116	79689	2034
M_116	79689	10095
M_116	140685	29775
M_116	7052	345651
M_116	2034	57520
M_116	11181	374286
M_117	1029	4507
M_117	1029	51198
M_117	1029	79886
M_117	1029	100048912
M_117	1029	1030
M_117	2298	200350
M_117	2298	653404
M_117	2298	55871
M_117	3105	3106
M_117	3105	3134
M_117	3105	3136
M_117	3105	3627
M_117	3105	5696
M_117	3105	5698
M_117	3105	6373
M_117	3105	6890
M_117	3105	3133
M_117	3105	84166
M_117	3105	352961
M_117	3105	6891
M_117	3105	10866
M_117	3105	116071

M_117	3106	3134
M_117	3106	3136
M_117	3106	3627
M_117	3106	5696
M_117	3106	5698
M_117	3106	6373
M_117	3106	80833
M_117	3106	6890
M_117	3106	3133
M_117	3106	51513
M_117	3106	6891
M_117	3106	10866
M_117	3106	197259
M_117	3134	3136
M_117	3134	3627
M_117	3134	5696
M_117	3134	5698
M_117	3134	6373
M_117	3134	6890
M_117	3134	115361
M_117	3134	400759
M_117	3134	3133
M_117	3134	51513
M_117	3134	352961
M_117	3134	6891
M_117	3134	10866
M_117	3134	197259
M_117	3136	5696
M_117	3136	5698
M_117	3136	6890
M_117	3136	3133
M_117	3136	84166

M_117	3136	352961
M_117	3136	6891
M_117	3136	10866
M_117	3136	116071
M_117	3627	5698
M_117	3627	6373
M_117	3627	3580
M_117	3627	3902
M_117	3627	6890
M_117	3627	10346
M_117	3627	115361
M_117	3627	400759
M_117	3627	952
M_117	3627	51513
M_117	3627	81030
M_117	3627	115362
M_117	3627	2633
M_117	3627	6355
M_117	4507	9373
M_117	4507	9933
M_117	4507	10171
M_117	4507	11168
M_117	4507	26953
M_117	4507	54801
M_117	4507	55958
M_117	4507	79886
M_117	4507	115426
M_117	4507	403313
M_117	4507	6619
M_117	4507	23081
M_117	4507	50808
M_117	4507	54875

M_117	4507	55667
M_117	4507	80173
M_117	4507	203228
M_117	4507	203238
M_117	4507	340481
M_117	4507	64922
M_117	4507	10670
M_117	4507	54914
M_117	5696	5698
M_117	5696	3659
M_117	5696	6890
M_117	5696	400759
M_117	5696	3133
M_117	5696	51513
M_117	5696	6891
M_117	5696	10866
M_117	5696	3620
M_117	5698	6373
M_117	5698	80833
M_117	5698	3659
M_117	5698	6890
M_117	5698	115361
M_117	5698	400759
M_117	5698	3133
M_117	5698	51513
M_117	5698	81030
M_117	5698	6891
M_117	5698	10866
M_117	5698	3620
M_117	6348	6351
M_117	6348	3553
M_117	6348	6347

M_117	6373	80830
M_117	6373	3580
M_117	6373	6890
M_117	6373	10346
M_117	6373	115361
M_117	6373	400759
M_117	6373	952
M_117	6373	51513
M_117	6373	81030
M_117	6373	115362
M_117	6373	2633
M_117	6373	3620
M_117	6373	6355
M_117	8743	57552
M_117	9373	9933
M_117	9373	10171
M_117	9373	11168
M_117	9373	26953
M_117	9373	54801
M_117	9373	55958
M_117	9373	79886
M_117	9373	115426
M_117	9373	158358
M_117	9373	6619
M_117	9373	23081
M_117	9373	55667
M_117	9373	80173
M_117	9373	203228
M_117	9373	203238
M_117	9373	340481
M_117	9373	64922
M_117	9373	10670

M_117	9373	79956
M_117	9373	54914
M_117	9933	10171
M_117	9933	11168
M_117	9933	26953
M_117	9933	54801
M_117	9933	55664
M_117	9933	55848
M_117	9933	55958
M_117	9933	57589
M_117	9933	79886
M_117	9933	115426
M_117	9933	158358
M_117	9933	403313
M_117	9933	6619
M_117	9933	23081
M_117	9933	50808
M_117	9933	55667
M_117	9933	5991
M_117	9933	203238
M_117	9933	340481
M_117	9933	79956
M_117	9933	23189
M_117	10171	11168
M_117	10171	26953
M_117	10171	54801
M_117	10171	55664
M_117	10171	55848
M_117	10171	55958
M_117	10171	79886
M_117	10171	90871
M_117	10171	115426

M_117	10171	403313
M_117	10171	6619
M_117	10171	23081
M_117	10171	50808
M_117	10171	55667
M_117	10171	5991
M_117	10171	340481
M_117	10171	10670
M_117	10171	79956
M_117	10171	55064
M_117	11168	26953
M_117	11168	54801
M_117	11168	55958
M_117	11168	79886
M_117	11168	115426
M_117	11168	158358
M_117	11168	6619
M_117	11168	50808
M_117	11168	54875
M_117	11168	55667
M_117	11168	80173
M_117	11168	5991
M_117	11168	203228
M_117	11168	203238
M_117	11168	340481
M_117	11168	10670
M_117	11168	4781
M_117	11168	54914
M_117	26953	54801
M_117	26953	55664
M_117	26953	55958
M_117	26953	57589

M_117	26953	115426
M_117	26953	158358
M_117	26953	403313
M_117	26953	6619
M_117	26953	23081
M_117	26953	50808
M_117	26953	54875
M_117	26953	55667
M_117	26953	5991
M_117	26953	340481
M_117	26953	10670
M_117	26953	4781
M_117	26953	79956
M_117	26953	55871
M_117	51198	100048912
M_117	51198	1030
M_117	51198	4781
M_117	54801	55958
M_117	54801	57589
M_117	54801	79886
M_117	54801	115426
M_117	54801	158358
M_117	54801	6619
M_117	54801	54875
M_117	54801	55667
M_117	54801	80173
M_117	54801	5991
M_117	54801	203238
M_117	54801	340481
M_117	54801	10670
M_117	54801	4781
M_117	54801	54914

M_117	55664	55848
M_117	55664	55958
M_117	55664	57589
M_117	55664	79886
M_117	55664	115426
M_117	55664	158358
M_117	55664	403313
M_117	55664	6619
M_117	55664	23081
M_117	55664	50808
M_117	55664	340481
M_117	55664	10670
M_117	55664	79956
M_117	55664	23189
M_117	55664	55064
M_117	55664	55871
M_117	55848	90871
M_117	55848	50808
M_117	55848	10670
M_117	55958	57589
M_117	55958	79886
M_117	55958	115426
M_117	55958	158358
M_117	55958	403313
M_117	55958	6619
M_117	55958	23081
M_117	55958	50808
M_117	55958	54875
M_117	55958	55667
M_117	55958	80173
M_117	55958	203228
M_117	55958	203238

M_117	55958	340481
M_117	55958	10670
M_117	55958	54914
M_117	57552	1030
M_117	57589	115426
M_117	57589	158358
M_117	57589	403313
M_117	57589	3717
M_117	57589	6619
M_117	57589	23081
M_117	57589	50808
M_117	57589	54875
M_117	57589	55667
M_117	57589	340481
M_117	57589	79956
M_117	57589	54914
M_117	57589	23189
M_117	79886	115426
M_117	79886	6619
M_117	79886	50808
M_117	79886	55667
M_117	79886	80173
M_117	79886	203228
M_117	79886	203238
M_117	79886	340481
M_117	79886	64922
M_117	79886	10670
M_117	80830	80833
M_117	80830	3659
M_117	80830	6890
M_117	80830	10346
M_117	80830	115361

M_117	80830	400759
M_117	80830	51513
M_117	80830	81030
M_117	80830	2633
M_117	80830	6891
M_117	80830	2634
M_117	80830	3620
M_117	80833	3659
M_117	80833	115361
M_117	80833	3133
M_117	80833	6891
M_117	80833	2634
M_117	90871	50808
M_117	90871	10670
M_117	115426	158358
M_117	115426	403313
M_117	115426	6619
M_117	115426	23081
M_117	115426	50808
M_117	115426	54875
M_117	115426	55667
M_117	115426	80173
M_117	115426	5991
M_117	115426	203238
M_117	115426	340481
M_117	115426	10670
M_117	115426	79956
M_117	115426	55064
M_117	158358	403313
M_117	158358	6619
M_117	158358	23081
M_117	158358	50808

M_117	158358	55667
M_117	158358	80173
M_117	158358	5991
M_117	158358	203238
M_117	158358	340481
M_117	158358	4781
M_117	158358	79956
M_117	158358	169522
M_117	200350	100036519
M_117	200350	653404
M_117	403313	23081
M_117	403313	50808
M_117	403313	340481
M_117	403313	79956
M_117	403313	23189
M_117	403313	55064
M_117	403313	55871
M_117	629	6892
M_117	629	717
M_117	1880	5996
M_117	1880	728
M_117	3580	3902
M_117	3580	10346
M_117	3580	115361
M_117	3580	400759
M_117	3580	952
M_117	3580	2633
M_117	3580	11262
M_117	3659	6890
M_117	3659	115361
M_117	3659	51513
M_117	3659	2633

M_117	3659	6891
M_117	3659	3620
M_117	3717	29126
M_117	3717	55667
M_117	3902	400759
M_117	3902	952
M_117	3902	11262
M_117	3902	6355
M_117	5996	5008
M_117	6619	23081
M_117	6619	50808
M_117	6619	54875
M_117	6619	55667
M_117	6619	80173
M_117	6619	5991
M_117	6619	203228
M_117	6619	203238
M_117	6619	340481
M_117	6619	10670
M_117	6619	4781
M_117	6619	79956
M_117	6619	54914
M_117	6890	115361
M_117	6890	400759
M_117	6890	952
M_117	6890	3133
M_117	6890	51513
M_117	6890	81030
M_117	6890	84166
M_117	6890	2633
M_117	6890	6891
M_117	6890	10866

M_117	6890	3620
M_117	6890	116071
M_117	6892	6891
M_117	10346	29126
M_117	10346	115361
M_117	10346	400759
M_117	10346	51513
M_117	10346	81030
M_117	10346	2633
M_117	10346	197259
M_117	10346	94240
M_117	23081	50808
M_117	23081	55667
M_117	23081	80173
M_117	23081	5991
M_117	23081	340481
M_117	23081	4781
M_117	23081	79956
M_117	23081	23189
M_117	23081	55064
M_117	29126	115361
M_117	29126	400759
M_117	29126	952
M_117	29126	51513
M_117	29126	84166
M_117	29126	94240
M_117	50808	55667
M_117	50808	80173
M_117	50808	5991
M_117	50808	203228
M_117	50808	340481
M_117	50808	10670

M_117	50808	79956
M_117	50808	55064
M_117	54875	203238
M_117	54875	340481
M_117	54875	4781
M_117	54875	54914
M_117	55667	80173
M_117	55667	203238
M_117	55667	340481
M_117	55667	340485
M_117	55667	10670
M_117	55667	4781
M_117	55667	79956
M_117	55667	54914
M_117	55667	23189
M_117	80173	5991
M_117	80173	203228
M_117	80173	203238
M_117	80173	340481
M_117	80173	64922
M_117	115361	400759
M_117	115361	952
M_117	115361	3133
M_117	115361	51513
M_117	115361	115362
M_117	115361	2633
M_117	115361	6891
M_117	115361	2634
M_117	115361	3620
M_117	400759	952
M_117	400759	51513
M_117	400759	81030

M_117	400759	2633
M_117	400759	6891
M_117	400759	3620
M_117	400759	116071
M_117	100036519	653404
M_117	100048912	1030
M_117	100048912	1031
M_117	952	11262
M_117	952	6355
M_117	1030	2683
M_117	3133	6891
M_117	3133	10866
M_117	5991	203228
M_117	5991	203238
M_117	51513	81030
M_117	51513	2633
M_117	51513	6891
M_117	81030	2633
M_117	81030	94240
M_117	84166	352961
M_117	84166	10866
M_117	84166	116071
M_117	203228	203238
M_117	203228	64922
M_117	203238	340481
M_117	203238	64922
M_117	203238	79956
M_117	203238	54914
M_117	340481	10670
M_117	340481	158219
M_117	340481	79956
M_117	352961	6891

M_117	352961	10866
M_117	2633	6891
M_117	2633	94240
M_117	3553	5008
M_117	5008	6347
M_117	5008	728
M_117	6891	10866
M_117	57462	340485
M_117	2683	23189
M_117	2683	84102
M_117	6696	54210
M_117	6696	597
M_117	6696	23601
M_117	54210	6556
M_117	54210	8685
M_117	10670	401494
M_117	158219	401494
M_117	158219	84102
M_117	2358	353514
M_117	3620	116071
M_117	353514	597
M_117	597	6347
M_117	79956	54914
M_117	79956	23189
M_117	6347	6355
M_144	928	6337
M_144	928	55080
M_144	1364	1365
M_144	1364	3855
M_144	1364	6337
M_144	1364	10045
M_144	1364	27134

M_144	1364	92359
M_144	1364	146439
M_144	1364	6692
M_144	1364	81607
M_144	1364	83451
M_144	1364	126695
M_144	1364	4072
M_144	1364	5652
M_144	1364	2591
M_144	1364	286676
M_144	1365	92359
M_144	1365	128218
M_144	1365	1366
M_144	1365	83451
M_144	1365	171022
M_144	1365	126695
M_144	1365	4072
M_144	1365	5652
M_144	3855	4070
M_144	3855	6337
M_144	3855	10045
M_144	3855	11187
M_144	3855	64787
M_144	3855	146439
M_144	3855	1366
M_144	3855	6692
M_144	3855	57402
M_144	3855	126695
M_144	3855	150696
M_144	4070	128218
M_144	4070	150696
M_144	4070	6385

M_144	4070	6768
M_144	6337	11187
M_144	6337	23779
M_144	6337	54869
M_144	6337	146439
M_144	6337	147798
M_144	6337	149466
M_144	6337	1366
M_144	6337	6692
M_144	6337	54845
M_144	6337	58495
M_144	6337	126695
M_144	6337	10053
M_144	6337	4072
M_144	6337	5652
M_144	6337	2591
M_144	6337	286676
M_144	6337	161291
M_144	6337	162387
M_144	10045	11187
M_144	10045	27134
M_144	10045	54869
M_144	10045	64787
M_144	10045	92359
M_144	10045	146439
M_144	10045	147798
M_144	10045	10053
M_144	10045	79958
M_144	11187	27134
M_144	11187	54869
M_144	11187	64787
M_144	11187	92359

M_144	11187	146439
M_144	11187	147798
M_144	11187	149466
M_144	11187	1366
M_144	11187	54845
M_144	11187	58495
M_144	11187	3898
M_144	11187	57402
M_144	11187	126695
M_144	11187	338440
M_144	11187	10053
M_144	11187	286676
M_144	23779	54869
M_144	23779	79977
M_144	23779	140893
M_144	23779	146439
M_144	23779	149466
M_144	23779	553158
M_144	23779	1366
M_144	23779	54845
M_144	23779	58495
M_144	23779	91862
M_144	23779	3898
M_144	23779	126695
M_144	23779	10053
M_144	23779	5652
M_144	23779	2591
M_144	23779	161291
M_144	23779	162387
M_144	27134	54869
M_144	27134	79977
M_144	27134	92359

M_144	27134	140893
M_144	27134	146439
M_144	27134	149466
M_144	27134	1366
M_144	27134	6692
M_144	27134	54845
M_144	27134	58495
M_144	27134	81607
M_144	27134	222
M_144	27134	3898
M_144	27134	57402
M_144	27134	126695
M_144	27134	10053
M_144	27134	54836
M_144	27134	2065
M_144	27134	5652
M_144	27134	6768
M_144	27134	286676
M_144	54869	64787
M_144	54869	92359
M_144	54869	146439
M_144	54869	147798
M_144	54869	553158
M_144	54869	1366
M_144	54869	6692
M_144	54869	112724
M_144	54869	5639
M_144	54869	126695
M_144	64787	146439
M_144	64787	338440
M_144	79977	140893
M_144	79977	146439

M_144	79977	149466
M_144	79977	553158
M_144	79977	1366
M_144	79977	3664
M_144	79977	6692
M_144	79977	54845
M_144	79977	58495
M_144	79977	81607
M_144	79977	91862
M_144	79977	222
M_144	79977	3898
M_144	79977	126695
M_144	79977	150696
M_144	79977	54836
M_144	79977	114569
M_144	79977	2065
M_144	79977	4072
M_144	79977	5652
M_144	79977	6768
M_144	79977	286676
M_144	79977	2041
M_144	79977	161291
M_144	80162	387733
M_144	80162	171389
M_144	80162	338440
M_144	92359	146439
M_144	92359	149466
M_144	92359	1366
M_144	92359	54845
M_144	92359	58495
M_144	92359	91862
M_144	92359	3898

M_144	92359	57402
M_144	92359	126695
M_144	92359	10053
M_144	92359	54836
M_144	92359	4072
M_144	92359	5652
M_144	92359	162387
M_144	128218	149466
M_144	128218	1366
M_144	128218	126695
M_144	128218	5652
M_144	128218	162387
M_144	140893	146439
M_144	140893	149466
M_144	140893	553158
M_144	140893	6692
M_144	140893	54845
M_144	140893	58495
M_144	140893	81607
M_144	140893	222
M_144	140893	3898
M_144	140893	150696
M_144	140893	54836
M_144	140893	2065
M_144	140893	4072
M_144	140893	5652
M_144	140893	55040
M_144	140893	2041
M_144	140893	161291
M_144	146439	147798
M_144	146439	149466
M_144	146439	1366

M_144	146439	6692
M_144	146439	54845
M_144	146439	58495
M_144	146439	81607
M_144	146439	3898
M_144	146439	57402
M_144	146439	126695
M_144	146439	10053
M_144	146439	54836
M_144	146439	5652
M_144	146439	286676
M_144	146439	161291
M_144	147798	5639
M_144	147798	286676
M_144	147798	161291
M_144	149466	553158
M_144	149466	1366
M_144	149466	6692
M_144	149466	54845
M_144	149466	58495
M_144	149466	91862
M_144	149466	3898
M_144	149466	57402
M_144	149466	126695
M_144	149466	10053
M_144	149466	54836
M_144	149466	4072
M_144	149466	5652
M_144	149466	2591
M_144	149466	286676
M_144	149466	161291
M_144	149466	162387

M_144	387733	171389
M_144	553158	1366
M_144	553158	6692
M_144	553158	54845
M_144	553158	58495
M_144	553158	3898
M_144	553158	126695
M_144	553158	54836
M_144	553158	4072
M_144	553158	5652
M_144	553158	286676
M_144	553158	2041
M_144	553158	161291
M_144	553158	162387
M_144	1366	6692
M_144	1366	54845
M_144	1366	58495
M_144	1366	83451
M_144	1366	91862
M_144	1366	3898
M_144	1366	57402
M_144	1366	126695
M_144	1366	10053
M_144	1366	54836
M_144	1366	4072
M_144	1366	5652
M_144	1366	2591
M_144	1366	286676
M_144	1366	162387
M_144	3664	91862
M_144	3664	3898
M_144	3664	2065

M_144	3664	4072
M_144	3664	2591
M_144	3664	161291
M_144	3664	162387
M_144	3664	50848
M_144	6692	54845
M_144	6692	81607
M_144	6692	3898
M_144	6692	126695
M_144	6692	54836
M_144	6692	2065
M_144	6692	4072
M_144	6692	5652
M_144	6692	286676
M_144	6692	161291
M_144	51206	112724
M_144	51206	199713
M_144	54845	58495
M_144	54845	81607
M_144	54845	91862
M_144	54845	3898
M_144	54845	57402
M_144	54845	126695
M_144	54845	10053
M_144	54845	54836
M_144	54845	114569
M_144	54845	2065
M_144	54845	4072
M_144	54845	5652
M_144	54845	6768
M_144	54845	286676
M_144	54845	161291

M_144	55655	199713
M_144	58495	81607
M_144	58495	91862
M_144	58495	222
M_144	58495	3898
M_144	58495	57402
M_144	58495	126695
M_144	58495	10053
M_144	58495	54836
M_144	58495	114569
M_144	58495	2065
M_144	58495	4072
M_144	58495	5652
M_144	58495	2591
M_144	58495	5349
M_144	58495	286676
M_144	58495	161291
M_144	58495	162387
M_144	81607	222
M_144	81607	3898
M_144	81607	57402
M_144	81607	150696
M_144	81607	54836
M_144	81607	114569
M_144	81607	2065
M_144	81607	5652
M_144	81607	50848
M_144	81607	64218
M_144	83451	171022
M_144	91862	3898
M_144	91862	126695
M_144	91862	10053

M_144	91862	4072
M_144	91862	5652
M_144	91862	2591
M_144	91862	161291
M_144	91862	162387
M_144	222	3898
M_144	222	57402
M_144	222	150696
M_144	222	114569
M_144	222	2065
M_144	222	5652
M_144	222	55040
M_144	3898	57402
M_144	3898	126695
M_144	3898	54836
M_144	3898	2065
M_144	3898	4072
M_144	3898	5349
M_144	3898	6768
M_144	3898	2041
M_144	3898	161291
M_144	5639	54836
M_144	57402	150696
M_144	57402	114569
M_144	57402	5349
M_144	126695	10053
M_144	126695	54836
M_144	126695	114569
M_144	126695	4072
M_144	126695	5652
M_144	126695	2591
M_144	126695	6768

M_144	126695	286676
M_144	126695	161291
M_144	126695	162387
M_144	150696	2065
M_144	10053	54836
M_144	10053	4072
M_144	10053	5349
M_144	10053	286676
M_144	10053	2041
M_144	10053	162387
M_144	54836	114569
M_144	54836	2065
M_144	54836	4072
M_144	54836	5652
M_144	54836	6768
M_144	54836	286676
M_144	54836	161291
M_144	114569	55630
M_144	2065	4072
M_144	2065	5652
M_144	2065	2591
M_144	2065	5349
M_144	2065	286676
M_144	4072	5652
M_144	4072	2591
M_144	4072	6768
M_144	4072	286676
M_144	4072	2041
M_144	4072	161291
M_144	4072	162387
M_144	5652	2591
M_144	5652	6768

M_144	2591	5349
M_144	2591	286676
M_144	2591	161291
M_144	2591	162387
M_144	6768	161291
M_144	286676	161291
M_144	286676	162387
M_144	2041	162387
M_147	1116	1356
M_147	1116	4608
M_147	1116	8878
M_147	1116	60598
M_147	1356	6271
M_147	1356	6590
M_147	1356	10568
M_147	1356	10406
M_147	1356	3359
M_147	1356	4582
M_147	1356	26298
M_147	1356	121838
M_147	1356	123036
M_147	1356	1718
M_147	3454	3588
M_147	3454	3455
M_147	3454	3460
M_147	3454	8850
M_147	3454	10311
M_147	3588	3455
M_147	3588	3460
M_147	3588	10311
M_147	5266	6590
M_147	5266	1051

M_147	5266	83938
M_147	6271	6284
M_147	6271	6590
M_147	6271	10568
M_147	6271	121838
M_147	6271	83938
M_147	6271	5872
M_147	6277	6284
M_147	6277	140576
M_147	6277	4000
M_147	6277	6282
M_147	6277	1535
M_147	6277	706
M_147	6277	2495
M_147	6277	145200
M_147	6284	140576
M_147	6284	1535
M_147	6590	10568
M_147	6590	6282
M_147	6590	10406
M_147	6590	3359
M_147	6590	121838
M_147	6590	60598
M_147	6590	83938
M_147	7127	10568
M_147	7127	91828
M_147	7127	79949
M_147	7127	4582
M_147	10568	64078
M_147	10568	79949
M_147	10568	1999
M_147	10568	3359

M_147	10568	121838
M_147	10568	1718
M_147	140576	4000
M_147	140576	6282
M_147	140576	706
M_147	140576	2495
M_147	3455	3460
M_147	3455	10311
M_147	6281	6282
M_147	6282	1476
M_147	6282	10577
M_147	6282	55647
M_147	10406	121838
M_147	10406	60598
M_147	10406	6001
M_147	64078	79949
M_147	64078	64866
M_147	64078	26298
M_147	64078	123036
M_147	64078	54933
M_147	64078	64170
M_147	79949	1999
M_147	79949	3359
M_147	79949	26298
M_147	79949	121838
M_147	79949	123036
M_147	79949	54933
M_147	1535	706
M_147	1535	1992
M_147	1999	3359
M_147	1999	4582
M_147	1999	64866

M_147	1999	79056
M_147	1999	79679
M_147	1999	123036
M_147	1999	638
M_147	1999	54933
M_147	3359	4582
M_147	3359	79679
M_147	3359	8878
M_147	3460	1476
M_147	4582	64866
M_147	4582	26298
M_147	4582	79679
M_147	4582	123036
M_147	8850	9695
M_147	51084	51635
M_147	51635	2217
M_147	51635	10577
M_147	64866	79056
M_147	64866	123036
M_147	64866	4615
M_147	706	638
M_147	706	2495
M_147	706	8878
M_147	706	6237
M_147	706	440836
M_147	1476	10577
M_147	1476	84419
M_147	26298	79056
M_147	26298	123036
M_147	26298	1718
M_147	26298	3732
M_147	79056	123036

M_147	79056	54933
M_147	79056	3732
M_147	79679	123036
M_147	79679	3732
M_147	121838	60598
M_147	2217	6948
M_147	2495	8878
M_147	10577	6237
M_147	54933	1718
M_147	54933	3732
M_147	1051	1942
M_147	26499	84419
M_158	800	284119
M_158	800	253827
M_158	800	25802
M_158	800	6876
M_158	800	84168
M_158	800	7078
M_158	800	23022
M_158	2669	5054
M_158	8840	10335
M_158	8840	27286
M_158	8840	11259
M_158	8840	79827
M_158	8840	114801
M_158	8840	389136
M_158	8840	1009
M_158	8840	2825
M_158	8840	9843
M_158	8840	56917
M_158	10335	27286
M_158	10335	114897

M_158	10335	59
M_158	10335	11259
M_158	10335	5924
M_158	10335	114801
M_158	10335	84624
M_158	10335	6424
M_158	10335	284297
M_158	10335	1009
M_158	10335	9843
M_158	10335	7057
M_158	10335	56917
M_158	284119	253827
M_158	284119	25802
M_158	284119	7078
M_158	284119	23022
M_158	284119	57493
M_158	284119	57158
M_158	27286	90993
M_158	27286	114897
M_158	27286	5156
M_158	27286	11259
M_158	27286	5924
M_158	27286	114801
M_158	27286	84624
M_158	27286	6424
M_158	27286	2825
M_158	27286	9843
M_158	90993	114897
M_158	90993	9843
M_158	90993	57333
M_158	114897	5156
M_158	114897	84624

M_158	114897	2825
M_158	114897	9843
M_158	59	633
M_158	59	253827
M_158	59	72
M_158	59	2252
M_158	59	27295
M_158	59	6876
M_158	59	7041
M_158	59	5054
M_158	59	7057
M_158	59	56917
M_158	633	253827
M_158	633	114801
M_158	633	1307
M_158	633	83468
M_158	5156	79827
M_158	5156	114801
M_158	5156	3487
M_158	5156	84624
M_158	5156	6424
M_158	5156	1009
M_158	5156	2825
M_158	11259	114801
M_158	11259	389136
M_158	11259	1009
M_158	11259	2825
M_158	11259	9843
M_158	11259	7057
M_158	51339	253827
M_158	51339	25802
M_158	51339	147906

M_158	51339	84168
M_158	51339	83468
M_158	51339	80114
M_158	5924	84624
M_158	5924	6424
M_158	5924	9843
M_158	10395	57493
M_158	253827	25802
M_158	253827	84168
M_158	253827	7041
M_158	253827	337876
M_158	253827	80114
M_158	72	1264
M_158	72	6876
M_158	1264	25802
M_158	1264	6876
M_158	1264	2134
M_158	2252	27295
M_158	2252	6424
M_158	2252	127435
M_158	2252	151887
M_158	2252	5054
M_158	2252	5376
M_158	2252	7057
M_158	2252	5350
M_158	25802	6876
M_158	25802	284297
M_158	27295	6876
M_158	27295	5054
M_158	27295	7057
M_158	79827	114801
M_158	79827	1009

M_158	79827	2825
M_158	79827	83468
M_158	79827	387914
M_158	114801	1009
M_158	114801	56978
M_158	114801	2115
M_158	114801	80114
M_158	3487	147906
M_158	3487	116535
M_158	3487	219654
M_158	10398	6876
M_158	10398	7169
M_158	10398	91663
M_158	84624	6424
M_158	84624	284297
M_158	84624	2825
M_158	84624	9843
M_158	147906	219654
M_158	147906	5740
M_158	6424	2825
M_158	84168	1307
M_158	84168	83468
M_158	84168	7077
M_158	84168	4323
M_158	127435	284297
M_158	127435	1307
M_158	127435	151887
M_158	284297	1307
M_158	284297	151887
M_158	284297	389558
M_158	284297	5054
M_158	389136	337876

M_158	389136	7057
M_158	1009	83468
M_158	1009	2115
M_158	2825	9843
M_158	116535	3671
M_158	219654	1307
M_158	219654	151887
M_158	219654	5740
M_158	1307	83468
M_158	1307	4323
M_158	7041	5376
M_158	7041	51226
M_158	7041	5118
M_158	7041	4681
M_158	56978	639
M_158	151887	79987
M_158	7078	2246
M_158	2134	148113
M_158	148113	8630
M_158	337876	56917
M_158	389558	9945
M_158	5054	7057
M_158	5054	5999
M_158	5376	51226
M_158	83468	2115
M_158	83468	5118
M_158	83468	4323
M_158	7077	4323
M_158	9945	340075
M_158	51226	340075
M_158	51226	5118
M_158	57493	84561

M_158	340075	5118
M_158	340075	10630
M_158	27129	57158
M_158	57158	7168
M_158	5350	1311
M_158	84561	220
M_184	2537	4939
M_184	2537	9636
M_184	2537	91543
M_184	2537	129607
M_184	2537	4938
M_184	2537	8638
M_184	2537	4599
M_184	2537	3429
M_184	2537	6772
M_184	2537	27074
M_184	2537	55008
M_184	2537	51191
M_184	2537	23586
M_184	2537	10379
M_184	2537	5610
M_184	2537	11274
M_184	3113	3115
M_184	3113	3117
M_184	3113	3108
M_184	3113	3122
M_184	3113	972
M_184	3113	3123
M_184	3113	3111
M_184	3115	3117
M_184	3115	3108
M_184	3115	3122

M_184	3115	972
M_184	3115	3123
M_184	3115	3111
M_184	3117	3119
M_184	3117	3108
M_184	3117	3122
M_184	3117	972
M_184	3117	3123
M_184	3117	3127
M_184	3119	3108
M_184	3119	3123
M_184	3119	3127
M_184	3119	3111
M_184	4061	8519
M_184	4061	10410
M_184	4061	100133669
M_184	4939	4940
M_184	4939	9636
M_184	4939	55601
M_184	4939	91351
M_184	4939	91543
M_184	4939	129607
M_184	4939	4938
M_184	4939	8638
M_184	4939	4599
M_184	4939	6772
M_184	4939	27074
M_184	4939	55008
M_184	4939	51191
M_184	4939	23586
M_184	4940	9636
M_184	4940	55601

M_184	4940	91351
M_184	4940	91543
M_184	4940	129607
M_184	4940	4938
M_184	4940	8638
M_184	4940	4599
M_184	4940	6772
M_184	4940	27074
M_184	4940	55008
M_184	4940	51191
M_184	4940	23586
M_184	8519	10410
M_184	8519	10581
M_184	8519	3429
M_184	8519	684
M_184	8542	23780
M_184	8542	5920
M_184	8542	6398
M_184	9636	55601
M_184	9636	91351
M_184	9636	91543
M_184	9636	129607
M_184	9636	4938
M_184	9636	8638
M_184	9636	4599
M_184	9636	3429
M_184	9636	6772
M_184	9636	27074
M_184	9636	55008
M_184	9636	51191
M_184	9636	23586
M_184	9636	11274

M_184	10410	10581
M_184	55601	91351
M_184	55601	91543
M_184	55601	129607
M_184	55601	4938
M_184	55601	8638
M_184	55601	4599
M_184	55601	27074
M_184	55601	55008
M_184	55601	51191
M_184	55601	23586
M_184	91351	91543
M_184	91351	129607
M_184	91351	4938
M_184	91351	8638
M_184	91351	4599
M_184	91351	55008
M_184	91351	51191
M_184	91351	23586
M_184	91543	129607
M_184	91543	4938
M_184	91543	8638
M_184	91543	4599
M_184	91543	6772
M_184	91543	27074
M_184	91543	55008
M_184	91543	51191
M_184	91543	23586
M_184	129607	4938
M_184	129607	8638
M_184	129607	4599
M_184	129607	6772

M_184	129607	27074
M_184	129607	55008
M_184	129607	51191
M_184	129607	23586
M_184	3108	3122
M_184	3108	972
M_184	3108	3123
M_184	3108	3127
M_184	3108	5699
M_184	3108	3111
M_184	3108	4261
M_184	3108	3109
M_184	3108	58472
M_184	3122	972
M_184	3122	3123
M_184	3122	567
M_184	3122	3111
M_184	3122	4261
M_184	3122	3109
M_184	3600	55013
M_184	3600	5699
M_184	3600	5920
M_184	3600	10578
M_184	3600	3601
M_184	3600	3821
M_184	4938	8638
M_184	4938	4599
M_184	4938	3429
M_184	4938	55008
M_184	4938	51191
M_184	4938	23586
M_184	8638	4599

M_184	8638	3429
M_184	8638	27074
M_184	8638	55008
M_184	8638	51191
M_184	8638	23586
M_184	972	3123
M_184	972	5699
M_184	972	5920
M_184	972	567
M_184	972	3111
M_184	972	4261
M_184	972	58472
M_184	3107	5699
M_184	3107	567
M_184	3123	3127
M_184	3123	5699
M_184	3123	3111
M_184	3123	4261
M_184	3123	3109
M_184	3127	3111
M_184	4599	3429
M_184	4599	27074
M_184	4599	55008
M_184	4599	51191
M_184	4599	23586
M_184	5699	5920
M_184	5699	567
M_184	5699	3601
M_184	5699	58472
M_184	5699	256586
M_184	5920	567
M_184	5920	3429

M_184	5920	3601
M_184	5920	58472
M_184	567	3601
M_184	567	58472
M_184	3429	684
M_184	3429	10379
M_184	10578	3601
M_184	10578	3805
M_184	10578	6846
M_184	4067	5265
M_184	5265	58472
M_184	3111	4261
M_184	3111	3109
M_184	3111	3112
M_184	4261	221472
M_184	3601	58472
M_184	6772	27074
M_184	6772	51191
M_184	6772	23586
M_184	27074	55008
M_184	27074	51191
M_184	27074	23586
M_184	55008	51191
M_184	55008	23586
M_184	51191	23586
M_184	51191	5610
M_184	3805	3821
M_184	11274	200315
M_184	3456	282618
M_184	200315	282618
M_194	4440	388228
M_194	4440	6297

M_194	4440	222663
M_194	9244	25789
M_194	9244	2642
M_194	9271	11211
M_194	11211	114299
M_194	11211	168002
M_194	25789	81618
M_194	25789	54360
M_194	116113	10500
M_194	116113	341405
M_194	388228	6297
M_194	388228	10220
M_194	388228	284069
M_194	388228	10500
M_194	388228	222663
M_194	111	5076
M_194	111	146664
M_194	111	81618
M_194	111	341405
M_194	111	54756
M_194	3674	284069
M_194	5076	146664
M_194	5076	81618
M_194	6297	10220
M_194	6297	284069
M_194	8997	10220
M_194	10220	284069
M_194	10220	81618
M_194	10220	149461
M_194	10220	341405
M_194	10220	8936
M_194	114299	25841

M_194	146664	81618
M_194	284069	81618
M_194	284069	149461
M_194	284069	10500
M_194	284069	341405
M_194	284069	4917
M_194	81618	54360
M_194	81618	6299
M_194	149461	341405
M_194	3321	5738
M_194	3321	1001
M_194	3321	375057
M_194	3321	222663
M_194	5100	10562
M_194	5100	23316
M_194	5100	11061
M_194	5738	9398
M_194	23302	8074
M_194	23302	4062
M_194	25841	23316
M_194	25841	54756
M_194	54360	4062
M_194	54360	171019
M_194	1001	8200
M_194	8074	2642
M_194	8074	23316
M_194	10500	222663
M_194	341405	4917
M_194	6299	23316
M_194	6299	222663
M_194	6299	168002
M_194	845	2254

M_194	2254	168002

Table S6 The GO enrichment results of the 208 modules

**Module ID**	**GO ID**	**P value**
M_0	GO:0070125	0.002
M_0	GO:0070126	0.0022
M_0	GO:0006383	0.0028
M_0	GO:0043001	0.0049
M_0	GO:0016559	0.0063
M_0	GO:0046628	0.0099
M_0	GO:0006747	0.0102
M_0	GO:0042946	0.0102
M_0	GO:0071802	0.0102
M_0	GO:1903709	0.0102
M_0	GO:1902824	0.0102
M_0	GO:0070159	0.0102
M_0	GO:0070145	0.0102
M_0	GO:0042543	0.0102
M_0	GO:1990390	0.0102
M_0	GO:1903699	0.0102
M_0	GO:0001770	0.0102
M_0	GO:0006238	0.0102
M_0	GO:2000640	0.0102
M_0	GO:1901622	0.0102
M_0	GO:0051141	0.0102
M_0	GO:1900148	0.0102
M_0	GO:1900131	0.0102
M_0	GO:0006506	0.0111
M_0	GO:1903378	0.0202
M_0	GO:2000170	0.0202
M_0	GO:1903762	0.0202
M_0	GO:0002940	0.0202

M_0	GO:0072615	0.0202
M_0	GO:0015847	0.0202
M_0	GO:0033384	0.0202
M_0	GO:0043377	0.0202
M_0	GO:0034402	0.0202
M_0	GO:0048026	0.0208
M_0	GO:0035019	0.0221
M_0	GO:0097352	0.0242
M_0	GO:0006892	0.0255
M_0	GO:0000432	0.0302
M_0	GO:0006850	0.0302
M_0	GO:0044211	0.0302
M_0	GO:0097676	0.0302
M_0	GO:1902268	0.0302
M_0	GO:0019287	0.0302
M_0	GO:0044375	0.0302
M_0	GO:0010750	0.0302
M_0	GO:0014908	0.0302
M_0	GO:0061458	0.0306
M_0	GO:0032483	0.04
M_0	GO:0090309	0.04
M_0	GO:0033133	0.04
M_0	GO:0045337	0.04
M_0	GO:0021769	0.04
M_0	GO:1902261	0.04
M_0	GO:0033594	0.04
M_0	GO:0032571	0.04
M_0	GO:1901097	0.04
M_0	GO:0006121	0.04
M_0	GO:0061484	0.04
M_0	GO:0019086	0.04
M_0	GO:0009566	0.0444

M_0	GO:0032212	0.0466
M_0	GO:0048013	0.048
M_0	GO:0071806	0.0494
M_0	GO:0043461	0.0498
M_0	GO:0033211	0.0498
M_0	GO:0071225	0.0498
M_0	GO:0008611	0.0498
M_0	GO:0044206	0.0498
M_0	GO:0006682	0.0498
M_0	GO:0090219	0.0498
M_0	GO:1902219	0.0498
M_0	GO:1990592	0.0498
M_0	GO:0072553	0.0498
M_0	GO:0070221	0.0498
M_0	GO:0006798	0.0498
M_0	GO:0009116	0.0498
M_1	GO:0061511	0.0019
M_1	GO:0007601	0.004
M_1	GO:0050910	0.0049
M_1	GO:0008543	0.0066
M_1	GO:0034213	0.0097
M_1	GO:0036305	0.0097
M_1	GO:0030046	0.0097
M_1	GO:0016189	0.0097
M_1	GO:1902161	0.0097
M_1	GO:0098629	0.0097
M_1	GO:1990697	0.0097
M_1	GO:0031549	0.0097
M_1	GO:0086024	0.0097
M_1	GO:0033609	0.0097
M_1	GO:0072432	0.0097
M_1	GO:0035508	0.0097

M_1	GO:1902476	0.0102
M_1	GO:0036092	0.0126
M_1	GO:0007597	0.0129
M_1	GO:0051897	0.0156
M_1	GO:0043401	0.0171
M_1	GO:0036150	0.0174
M_1	GO:0009187	0.0193
M_1	GO:0097055	0.0193
M_1	GO:0042986	0.0193
M_1	GO:0035377	0.0193
M_1	GO:1904106	0.0193
M_1	GO:0002358	0.0193
M_1	GO:1902943	0.0193
M_1	GO:1902269	0.0193
M_1	GO:1904021	0.0193
M_1	GO:0071579	0.0193
M_1	GO:2000854	0.0193
M_1	GO:2000815	0.0193
M_1	GO:1901898	0.0193
M_1	GO:0015847	0.0193
M_1	GO:1903575	0.0193
M_1	GO:0060314	0.0225
M_1	GO:0046854	0.023
M_1	GO:0043687	0.0255
M_1	GO:0034769	0.0288
M_1	GO:0002331	0.0288
M_1	GO:2000286	0.0288
M_1	GO:0090204	0.0288
M_1	GO:0060168	0.0288
M_1	GO:0071691	0.0288
M_1	GO:0070175	0.0288
M_1	GO:0097195	0.0288

M_1	GO:0048022	0.0288
M_1	GO:0046878	0.0288
M_1	GO:0033387	0.0288
M_1	GO:2000611	0.0288
M_1	GO:0009436	0.0288
M_1	GO:0048265	0.03
M_1	GO:0048147	0.032
M_1	GO:0060048	0.0341
M_1	GO:0050953	0.0371
M_1	GO:0043490	0.0383
M_1	GO:0070858	0.0383
M_1	GO:0035616	0.0383
M_1	GO:0048386	0.0383
M_1	GO:2001013	0.0383
M_1	GO:1901300	0.0383
M_1	GO:0002326	0.0383
M_1	GO:1901911	0.0383
M_1	GO:0035995	0.0383
M_1	GO:1901907	0.0383
M_1	GO:1901909	0.0383
M_1	GO:0019470	0.0383
M_1	GO:0042866	0.0383
M_1	GO:0071543	0.0383
M_1	GO:0071421	0.0383
M_1	GO:0007616	0.0452
M_1	GO:0045494	0.0452
M_1	GO:0002029	0.0474
M_1	GO:0043456	0.0476
M_1	GO:2000370	0.0476
M_1	GO:0060591	0.0476
M_1	GO:0006844	0.0476
M_1	GO:0016199	0.0476

M_1	GO:1903936	0.0476
M_1	GO:0036414	0.0476
M_1	GO:0032224	0.0476
M_1	GO:0070309	0.0476
M_1	GO:1900454	0.0476
M_1	GO:0051454	0.0476
M_1	GO:0010891	0.0476
M_2	GO:0035459	0.000061
M_2	GO:0043968	0.00034
M_2	GO:0006888	0.00052
M_2	GO:0009306	0.00113
M_2	GO:0090314	0.00223
M_2	GO:0048387	0.00262
M_2	GO:0007220	0.00326
M_2	GO:0006355	0.00507
M_2	GO:0061077	0.00529
M_2	GO:0007050	0.00611
M_2	GO:1900034	0.00666
M_2	GO:0051571	0.00738
M_2	GO:0051496	0.008
M_2	GO:2000299	0.00873
M_2	GO:0002502	0.00873
M_2	GO:0034352	0.00873
M_2	GO:0043602	0.00873
M_2	GO:0000416	0.00873
M_2	GO:0001809	0.00873
M_2	GO:0036333	0.00873
M_2	GO:0060383	0.00873
M_2	GO:0070413	0.00873
M_2	GO:0046210	0.00873
M_2	GO:2000388	0.00873
M_2	GO:0051179	0.00877

M_2	GO:0043525	0.00897
M_2	GO:0006406	0.01487
M_2	GO:0034314	0.01682
M_2	GO:0045892	0.01725
M_2	GO:0032608	0.01732
M_2	GO:1904049	0.01739
M_2	GO:0014886	0.01739
M_2	GO:0051083	0.01739
M_2	GO:0090346	0.01739
M_2	GO:1903526	0.01739
M_2	GO:0003420	0.01739
M_2	GO:0097010	0.01739
M_2	GO:0033025	0.01739
M_2	GO:0071403	0.01739
M_2	GO:0046338	0.01739
M_2	GO:2000588	0.01739
M_2	GO:0035574	0.01739
M_2	GO:2000620	0.01739
M_2	GO:0035063	0.01739
M_2	GO:0070476	0.01739
M_2	GO:2000368	0.01739
M_2	GO:1903362	0.01742
M_2	GO:0006357	0.01877
M_2	GO:0030511	0.02147
M_2	GO:0000245	0.02447
M_2	GO:1903895	0.02597
M_2	GO:0006933	0.02597
M_2	GO:0051088	0.02597
M_2	GO:0035803	0.02597
M_2	GO:0034247	0.02597
M_2	GO:0035022	0.02597
M_2	GO:0033387	0.02597

M_2	GO:1901895	0.02597
M_2	GO:0090031	0.02597
M_2	GO:0006446	0.02889
M_2	GO:0018107	0.03054
M_2	GO:0000082	0.03143
M_2	GO:0071466	0.03447
M_2	GO:0035984	0.03448
M_2	GO:1901529	0.03448
M_2	GO:0014834	0.03448
M_2	GO:0060235	0.03448
M_2	GO:0030578	0.03448
M_2	GO:0032202	0.03448
M_2	GO:0032741	0.03448
M_2	GO:0010513	0.03448
M_2	GO:0033353	0.03448
M_2	GO:0035625	0.03448
M_2	GO:0002408	0.03448
M_2	GO:0043137	0.03448
M_2	GO:0045356	0.03448
M_2	GO:2000360	0.03448
M_2	GO:0030866	0.03502
M_2	GO:0009615	0.03737
M_2	GO:0010595	0.03863
M_2	GO:0032467	0.03933
M_2	GO:0033572	0.03933
M_2	GO:0043967	0.04048
M_2	GO:0061001	0.04135
M_2	GO:0018393	0.04187
M_2	GO:1902749	0.04275
M_2	GO:0070200	0.04292
M_2	GO:0006398	0.04292
M_2	GO:0019509	0.04292

M_2	GO:0031914	0.04292
M_2	GO:0045542	0.04292
M_2	GO:0038033	0.04292
M_2	GO:0001554	0.04292
M_2	GO:1901164	0.04292
M_2	GO:1900454	0.04292
M_2	GO:0071557	0.04292
M_2	GO:2000370	0.04292
M_2	GO:0070317	0.0434
M_2	GO:0032880	0.04846
M_2	GO:0006475	0.04972
M_3	GO:0031848	0.000012
M_3	GO:0006995	0.000099
M_3	GO:0009838	0.00076
M_3	GO:0036258	0.00128
M_3	GO:0031571	0.00138
M_3	GO:0010510	0.0014
M_3	GO:0070198	0.00176
M_3	GO:0010501	0.0044
M_3	GO:0007595	0.00468
M_3	GO:0015031	0.00498
M_3	GO:0000920	0.0065
M_3	GO:0015938	0.00714
M_3	GO:0048239	0.00714
M_3	GO:0090410	0.00719
M_3	GO:0000916	0.00719
M_3	GO:0032208	0.00719
M_3	GO:0060381	0.00719
M_3	GO:0060377	0.00719
M_3	GO:0045403	0.00719
M_3	GO:1903770	0.00719
M_3	GO:0097310	0.00719

M_3	GO:1903824	0.00719
M_3	GO:0006430	0.00719
M_3	GO:0046745	0.00719
M_3	GO:2001294	0.00719
M_3	GO:0046092	0.00719
M_3	GO:1903394	0.00719
M_3	GO:0042352	0.00719
M_3	GO:0032211	0.00896
M_3	GO:0016226	0.00896
M_3	GO:0044458	0.01176
M_3	GO:0032205	0.01413
M_3	GO:0046356	0.01433
M_3	GO:0046338	0.01433
M_3	GO:0044209	0.01433
M_3	GO:0032214	0.01433
M_3	GO:0040030	0.01433
M_3	GO:0006624	0.01433
M_3	GO:0003195	0.01433
M_3	GO:0048213	0.01433
M_3	GO:0097309	0.01433
M_3	GO:1904046	0.01433
M_3	GO:0002276	0.01433
M_3	GO:1903576	0.01433
M_3	GO:0019085	0.01433
M_3	GO:0043323	0.01433
M_3	GO:0019550	0.01433
M_3	GO:0019551	0.01433
M_3	GO:0000422	0.01568
M_3	GO:0001816	0.01645
M_3	GO:1900106	0.02142
M_3	GO:0006858	0.02142
M_3	GO:0031117	0.02142

M_3	GO:0015966	0.02142
M_3	GO:0006532	0.02142
M_3	GO:0006533	0.02142
M_3	GO:0044205	0.02142
M_3	GO:0019464	0.02142
M_3	GO:0003192	0.02142
M_3	GO:0090204	0.02142
M_3	GO:1903774	0.02142
M_3	GO:1902775	0.02142
M_3	GO:0019287	0.02142
M_3	GO:0045226	0.02142
M_3	GO:0006419	0.02142
M_3	GO:0006168	0.02142
M_3	GO:0043686	0.02142
M_3	GO:0006891	0.02309
M_3	GO:0043928	0.02343
M_3	GO:0006370	0.02478
M_3	GO:0000045	0.0256
M_3	GO:0097352	0.02616
M_3	GO:0007080	0.02757
M_3	GO:0071459	0.02826
M_3	GO:0019076	0.02829
M_3	GO:0007089	0.02846
M_3	GO:0032202	0.02846
M_3	GO:1903251	0.02846
M_3	GO:0070602	0.02846
M_3	GO:0071733	0.02846
M_3	GO:0060319	0.02846
M_3	GO:0019470	0.02846
M_3	GO:0070370	0.02846
M_3	GO:0044878	0.02846
M_3	GO:0046104	0.02846

M_3	GO:0019918	0.02846
M_3	GO:0002943	0.02846
M_3	GO:0002143	0.02846
M_3	GO:1903566	0.02846
M_3	GO:1902035	0.02846
M_3	GO:0031627	0.02846
M_3	GO:0010724	0.02846
M_3	GO:0019086	0.02846
M_3	GO:0043378	0.02846
M_3	GO:0000301	0.02846
M_3	GO:0031146	0.03351
M_3	GO:0008033	0.03384
M_3	GO:0051028	0.03444
M_3	GO:0006085	0.03493
M_3	GO:1904430	0.03545
M_3	GO:0006489	0.03545
M_3	GO:0060296	0.03545
M_3	GO:0070200	0.03545
M_3	GO:0090611	0.03545
M_3	GO:1904354	0.03545
M_3	GO:0045540	0.03664
M_3	GO:0043985	0.04239
M_3	GO:0097052	0.04239
M_3	GO:2000270	0.04239
M_3	GO:0002741	0.04239
M_3	GO:0006207	0.04239
M_3	GO:0042177	0.04303
M_3	GO:0032466	0.04928
M_3	GO:0051013	0.04928
M_3	GO:0071051	0.04928
M_3	GO:0031087	0.04928
M_3	GO:0006477	0.04928

M_3	GO:0072319	0.04928
M_4	GO:0043297	0.0037
M_4	GO:0006355	0.0042
M_4	GO:1903955	0.0045
M_4	GO:0038180	0.0068
M_4	GO:0070986	0.0079
M_4	GO:0035735	0.0085
M_4	GO:0008049	0.0105
M_4	GO:0001174	0.0105
M_4	GO:0021547	0.0105
M_4	GO:0021501	0.0105
M_4	GO:1903569	0.0105
M_4	GO:0035229	0.0105
M_4	GO:0018272	0.0105
M_4	GO:1901373	0.0105
M_4	GO:0015787	0.0105
M_4	GO:0071266	0.0105
M_4	GO:0015728	0.0105
M_4	GO:1902073	0.0105
M_4	GO:0070183	0.0105
M_4	GO:0044524	0.0105
M_4	GO:1990114	0.0105
M_4	GO:0051780	0.0105
M_4	GO:0009313	0.0105
M_4	GO:0005980	0.0135
M_4	GO:1902043	0.0135
M_4	GO:0006895	0.0184
M_4	GO:0015031	0.0193
M_4	GO:0007224	0.02
M_4	GO:1904046	0.0209
M_4	GO:1903778	0.0209
M_4	GO:0032385	0.0209

M_4	GO:0015910	0.0209
M_4	GO:1990569	0.0209
M_4	GO:0006434	0.0209
M_4	GO:0035279	0.0209
M_4	GO:0097069	0.0209
M_4	GO:2000744	0.0209
M_4	GO:1902766	0.0209
M_4	GO:0090285	0.0209
M_4	GO:0044752	0.0209
M_4	GO:0019343	0.0209
M_4	GO:0060400	0.0209
M_4	GO:0010842	0.0221
M_4	GO:0006397	0.0237
M_4	GO:0008380	0.0272
M_4	GO:0042327	0.03
M_4	GO:2000301	0.0312
M_4	GO:0019254	0.0312
M_4	GO:0019276	0.0312
M_4	GO:0031081	0.0312
M_4	GO:0070814	0.0312
M_4	GO:0042109	0.0312
M_4	GO:0045200	0.0312
M_4	GO:0006433	0.0312
M_4	GO:1904381	0.0312
M_4	GO:0009236	0.0312
M_4	GO:0019065	0.0312
M_4	GO:0032765	0.0312
M_4	GO:0035879	0.0312
M_4	GO:0070966	0.0312
M_4	GO:0097327	0.0312
M_4	GO:1903445	0.0312
M_4	GO:0006065	0.0312

M_4	GO:0019079	0.0321
M_4	GO:0006884	0.0324
M_4	GO:0008643	0.0341
M_4	GO:0000398	0.0366
M_4	GO:2001234	0.037
M_4	GO:0042795	0.0374
M_4	GO:0006915	0.04
M_4	GO:0006534	0.0411
M_4	GO:0031591	0.0414
M_4	GO:0048254	0.0414
M_4	GO:0060235	0.0414
M_4	GO:0016259	0.0414
M_4	GO:0061086	0.0414
M_4	GO:0051697	0.0414
M_4	GO:0031627	0.0414
M_4	GO:0006490	0.0414
M_4	GO:1903527	0.0414
M_4	GO:0060800	0.0414
M_4	GO:0097056	0.0414
M_4	GO:1900535	0.0414
M_4	GO:0033615	0.0414
M_4	GO:0072513	0.0414
M_4	GO:0035522	0.0414
M_4	GO:0002906	0.0414
M_4	GO:0033353	0.0414
M_4	GO:0019346	0.0414
M_4	GO:2001274	0.0414
M_4	GO:0046726	0.0414
M_4	GO:0031860	0.0414
M_4	GO:0034080	0.0418
M_4	GO:0052548	0.0418
M_4	GO:0042407	0.0443

M_4	GO:0042981	0.0453
M_4	GO:0097009	0.0468
M_4	GO:0001569	0.0494
M_4	GO:0030488	0.0494
M_5	GO:0006357	1E-30
M_5	GO:0006355	0.0000011
M_5	GO:0000184	0.000041
M_5	GO:0006614	0.000066
M_5	GO:0090309	0.0007
M_5	GO:0006413	0.0016
M_5	GO:0009744	0.0024
M_5	GO:0000492	0.0032
M_5	GO:0071169	0.0032
M_5	GO:2001241	0.0073
M_5	GO:0045892	0.0078
M_5	GO:0032802	0.0085
M_5	GO:0071166	0.0108
M_5	GO:0090340	0.0109
M_5	GO:1990117	0.0109
M_5	GO:0090301	0.0109
M_5	GO:2000314	0.0109
M_5	GO:1903461	0.0109
M_5	GO:0051232	0.0109
M_5	GO:0032976	0.0109
M_5	GO:1902466	0.0109
M_5	GO:0098504	0.0109
M_5	GO:0098506	0.0109
M_5	GO:0090108	0.0109
M_5	GO:1901194	0.0109
M_5	GO:0006669	0.0109
M_5	GO:0000718	0.0109
M_5	GO:1903723	0.0109

M_5	GO:0070262	0.0144
M_5	GO:0000122	0.0194
M_5	GO:0051170	0.0198
M_5	GO:1900112	0.0215
M_5	GO:0000028	0.0217
M_5	GO:0045724	0.0217
M_5	GO:1990173	0.0217
M_5	GO:2000324	0.0217
M_5	GO:1903438	0.0217
M_5	GO:0017186	0.0217
M_5	GO:0006550	0.0217
M_5	GO:0090187	0.0217
M_5	GO:0045358	0.0217
M_5	GO:0002352	0.0217
M_5	GO:0002358	0.0217
M_5	GO:0014043	0.0217
M_5	GO:0009098	0.0217
M_5	GO:0009099	0.0217
M_5	GO:1904550	0.0217
M_5	GO:1903006	0.0217
M_5	GO:2001178	0.0217
M_5	GO:0060265	0.0217
M_5	GO:1903538	0.0217
M_5	GO:0045004	0.0217
M_5	GO:1900110	0.0217
M_5	GO:0006977	0.0232
M_5	GO:0006297	0.0257
M_5	GO:0000387	0.0298
M_5	GO:0060058	0.0324
M_5	GO:0010836	0.0324
M_5	GO:1904431	0.0324
M_5	GO:0046601	0.0324

M_5	GO:0006808	0.0324
M_5	GO:1902445	0.0324
M_5	GO:0035494	0.0324
M_5	GO:1902263	0.0324
M_5	GO:0035166	0.0324
M_5	GO:0046985	0.0324
M_5	GO:0071899	0.0324
M_5	GO:0000720	0.0324
M_5	GO:0045903	0.0324
M_5	GO:0002904	0.0324
M_5	GO:0060266	0.0324
M_5	GO:1901837	0.0324
M_5	GO:0048597	0.0324
M_5	GO:1902512	0.0324
M_5	GO:1901536	0.0324
M_5	GO:1902661	0.0324
M_5	GO:2000619	0.0324
M_5	GO:0000379	0.0324
M_5	GO:1900126	0.0324
M_5	GO:1900114	0.0324
M_5	GO:0034644	0.035
M_5	GO:0048208	0.0354
M_5	GO:0006281	0.036
M_5	GO:0050690	0.0396
M_5	GO:0048147	0.0396
M_5	GO:0060011	0.0429
M_5	GO:0046666	0.0429
M_5	GO:0071733	0.0429
M_5	GO:0006642	0.0429
M_5	GO:0000710	0.0429
M_5	GO:0048254	0.0429
M_5	GO:0002143	0.0429

M_5	GO:0048550	0.0429
M_5	GO:0001777	0.0429
M_5	GO:0006295	0.0429
M_5	GO:0097296	0.0429
M_5	GO:2000653	0.0429
M_5	GO:2000623	0.0429
M_5	GO:0089700	0.0429
M_5	GO:1900113	0.0429
M_5	GO:0009991	0.0437
M_5	GO:0006283	0.0455
M_5	GO:0045944	0.0482
M_6	GO:0006974	0.00018
M_6	GO:0007094	0.00051
M_6	GO:2000643	0.0007
M_6	GO:0090394	0.0009
M_6	GO:0045176	0.00136
M_6	GO:0065003	0.00192
M_6	GO:0000902	0.0044
M_6	GO:0036120	0.00457
M_6	GO:0032401	0.00503
M_6	GO:0008057	0.00508
M_6	GO:2001040	0.00508
M_6	GO:1902443	0.00508
M_6	GO:0097552	0.00508
M_6	GO:0072749	0.00508
M_6	GO:0044648	0.00508
M_6	GO:0034354	0.00508
M_6	GO:0070267	0.00508
M_6	GO:0034474	0.00508
M_6	GO:1902036	0.0071
M_6	GO:0043161	0.00762
M_6	GO:0000729	0.00766

M_6	GO:0006511	0.00773
M_6	GO:0071480	0.00885
M_6	GO:0070889	0.01013
M_6	GO:0010609	0.01013
M_6	GO:1902908	0.01013
M_6	GO:2000791	0.01013
M_6	GO:1903056	0.01013
M_6	GO:2000584	0.01013
M_6	GO:1902524	0.01013
M_6	GO:0019878	0.01013
M_6	GO:0072434	0.01013
M_6	GO:0007283	0.01112
M_6	GO:0000209	0.01167
M_6	GO:0090263	0.01184
M_6	GO:0042594	0.01316
M_6	GO:0016579	0.01347
M_6	GO:0032487	0.01516
M_6	GO:1902164	0.01516
M_6	GO:0002331	0.01516
M_6	GO:0090292	0.01516
M_6	GO:0021817	0.01516
M_6	GO:1902523	0.01516
M_6	GO:2000645	0.01516
M_6	GO:0019805	0.01516
M_6	GO:1904378	0.01516
M_6	GO:1901674	0.01516
M_6	GO:0010212	0.01994
M_6	GO:0006726	0.02017
M_6	GO:0000451	0.02017
M_6	GO:0071926	0.02017
M_6	GO:0046340	0.02017
M_6	GO:0002357	0.02017

M_6	GO:0031860	0.02017
M_6	GO:0071500	0.02017
M_6	GO:1902527	0.02017
M_6	GO:0050717	0.02017
M_6	GO:1903626	0.02017
M_6	GO:0000724	0.02033
M_6	GO:1903507	0.02071
M_6	GO:0048538	0.02103
M_6	GO:0007129	0.02285
M_6	GO:0007599	0.02476
M_6	GO:0033227	0.02515
M_6	GO:0038170	0.02515
M_6	GO:0015939	0.02515
M_6	GO:0090235	0.02515
M_6	GO:0071816	0.02515
M_6	GO:0006432	0.02515
M_6	GO:1900454	0.02515
M_6	GO:0075525	0.02515
M_6	GO:1904354	0.02515
M_6	GO:0007131	0.02974
M_6	GO:0045141	0.02978
M_6	GO:0071440	0.0301
M_6	GO:1902966	0.0301
M_6	GO:0042138	0.0301
M_6	GO:0061087	0.0301
M_6	GO:0060304	0.0301
M_6	GO:0021869	0.0301
M_6	GO:0032876	0.0301
M_6	GO:0031642	0.0301
M_6	GO:0010760	0.0301
M_6	GO:0006977	0.03292
M_6	GO:0021799	0.03469

M_6	GO:0097065	0.03503
M_6	GO:0021707	0.03503
M_6	GO:0060449	0.03503
M_6	GO:0070424	0.03503
M_6	GO:0090650	0.03503
M_6	GO:0072307	0.03503
M_6	GO:0051321	0.03579
M_6	GO:0009306	0.03608
M_6	GO:0035019	0.03622
M_6	GO:0033129	0.03993
M_6	GO:2000480	0.03993
M_6	GO:1902459	0.03993
M_6	GO:0035331	0.03993
M_6	GO:0036289	0.03993
M_6	GO:0000019	0.03993
M_6	GO:0070197	0.03993
M_6	GO:0006975	0.03993
M_6	GO:0050847	0.03993
M_6	GO:0006303	0.04199
M_6	GO:0006284	0.04199
M_6	GO:0070050	0.04481
M_6	GO:0090286	0.04481
M_6	GO:0060242	0.04481
M_6	GO:0002091	0.04481
M_6	GO:0034498	0.04481
M_6	GO:0042355	0.04481
M_6	GO:0034199	0.04481
M_6	GO:0008283	0.04588
M_6	GO:0071985	0.04967
M_6	GO:0033148	0.04967
M_6	GO:0034214	0.04967
M_6	GO:1903978	0.04967

M_6	GO:0006569	0.04967
M_6	GO:0018026	0.04967
M_6	GO:0035721	0.04967
M_6	GO:0045792	0.04967
M_6	GO:0030432	0.04967
M_6	GO:0075522	0.04967
M_7	GO:0021860	0.000031
M_7	GO:0046716	0.00058
M_7	GO:0010041	0.00068
M_7	GO:1903071	0.00141
M_7	GO:0000398	0.00239
M_7	GO:0015693	0.00431
M_7	GO:1903830	0.00767
M_7	GO:0046390	0.00822
M_7	GO:0046101	0.00828
M_7	GO:0032048	0.00828
M_7	GO:1904045	0.00833
M_7	GO:0046038	0.00833
M_7	GO:2000719	0.00833
M_7	GO:0000495	0.00833
M_7	GO:1900095	0.00833
M_7	GO:0007624	0.00833
M_7	GO:1901254	0.00833
M_7	GO:1900483	0.00833
M_7	GO:0046856	0.01062
M_7	GO:0040008	0.01063
M_7	GO:0006612	0.01186
M_7	GO:0021895	0.01556
M_7	GO:0000715	0.01556
M_7	GO:0006357	0.01608
M_7	GO:1904021	0.0166
M_7	GO:0007284	0.0166

M_7	GO:1900210	0.0166
M_7	GO:0051542	0.0166
M_7	GO:0009405	0.0166
M_7	GO:0015910	0.0166
M_7	GO:0060816	0.0166
M_7	GO:1903547	0.0166
M_7	GO:0006178	0.0166
M_7	GO:0032263	0.0166
M_7	GO:0010734	0.0166
M_7	GO:0034418	0.0166
M_7	GO:0060003	0.0166
M_7	GO:0017198	0.0166
M_7	GO:1902530	0.0166
M_7	GO:2000616	0.0166
M_7	GO:2000642	0.0166
M_7	GO:0034760	0.0166
M_7	GO:0010607	0.0166
M_7	GO:0044830	0.0166
M_7	GO:0018002	0.0166
M_7	GO:0015679	0.0166
M_7	GO:0070423	0.01943
M_7	GO:0070911	0.01967
M_7	GO:0006294	0.02417
M_7	GO:0055070	0.02465
M_7	GO:1900114	0.0248
M_7	GO:2000301	0.0248
M_7	GO:2000409	0.0248
M_7	GO:0016267	0.0248
M_7	GO:0042414	0.0248
M_7	GO:0061684	0.0248
M_7	GO:0032918	0.0248
M_7	GO:0070537	0.0248

M_7	GO:0006168	0.0248
M_7	GO:0070682	0.0248
M_7	GO:0071423	0.0248
M_7	GO:2000820	0.0248
M_7	GO:1904154	0.0248
M_7	GO:0006740	0.0248
M_7	GO:0015729	0.0248
M_7	GO:0015709	0.0248
M_7	GO:0034247	0.0248
M_7	GO:0070901	0.0248
M_7	GO:0009051	0.0248
M_7	GO:0002188	0.0248
M_7	GO:1900453	0.0248
M_7	GO:0090063	0.0248
M_7	GO:0070979	0.02576
M_7	GO:0042407	0.02906
M_7	GO:0008033	0.03241
M_7	GO:2001206	0.03292
M_7	GO:0035965	0.03292
M_7	GO:0035984	0.03292
M_7	GO:0019230	0.03292
M_7	GO:1903874	0.03292
M_7	GO:0051697	0.03292
M_7	GO:1903595	0.03292
M_7	GO:0098908	0.03292
M_7	GO:0071922	0.03292
M_7	GO:0007525	0.03292
M_7	GO:0048633	0.03292
M_7	GO:0071284	0.03292
M_7	GO:0071279	0.03292
M_7	GO:0031120	0.03292
M_7	GO:0019322	0.03292

M_7	GO:1990481	0.03292
M_7	GO:1990180	0.03292
M_7	GO:0043103	0.03292
M_7	GO:0046208	0.03292
M_7	GO:0050904	0.03292
M_7	GO:1900186	0.03292
M_7	GO:0045737	0.03612
M_7	GO:0007098	0.03784
M_7	GO:0061052	0.04098
M_7	GO:0031547	0.04098
M_7	GO:0019732	0.04098
M_7	GO:0021637	0.04098
M_7	GO:0032264	0.04098
M_7	GO:0006102	0.04098
M_7	GO:1904428	0.04098
M_7	GO:0071422	0.04098
M_7	GO:0034197	0.04098
M_7	GO:0002329	0.04098
M_7	GO:1903377	0.04098
M_7	GO:0015853	0.04098
M_7	GO:0071816	0.04098
M_7	GO:2000391	0.04098
M_7	GO:0051028	0.04459
M_7	GO:0030100	0.04553
M_7	GO:0006475	0.04806
M_7	GO:1903862	0.04898
M_7	GO:0090315	0.04898
M_7	GO:0010793	0.04898
M_7	GO:0032876	0.04898
M_7	GO:0006824	0.04898
M_7	GO:0070973	0.04898
M_7	GO:0043308	0.04898

M_7	GO:1902065	0.04898
M_7	GO:0060304	0.04898
M_7	GO:0006015	0.04898
M_7	GO:0045964	0.04898
M_8	GO:0006427	0.000094
M_8	GO:0006228	0.0001
M_8	GO:0006183	0.00014
M_8	GO:0033128	0.00028
M_8	GO:0006241	0.0003
M_8	GO:0042273	0.00147
M_8	GO:0015949	0.00273
M_8	GO:0070979	0.00301
M_8	GO:1902603	0.00486
M_8	GO:0007080	0.00507
M_8	GO:0034501	0.00789
M_8	GO:0009437	0.00789
M_8	GO:0006457	0.00804
M_8	GO:0045618	0.00905
M_8	GO:1990758	0.0097
M_8	GO:0070715	0.0097
M_8	GO:0002882	0.0097
M_8	GO:0060731	0.0097
M_8	GO:0060735	0.0097
M_8	GO:0018106	0.0097
M_8	GO:0072752	0.0097
M_8	GO:1902213	0.0097
M_8	GO:0007576	0.0097
M_8	GO:0010797	0.0097
M_8	GO:0052106	0.0097
M_8	GO:0085032	0.0097
M_8	GO:0000296	0.0097
M_8	GO:0010070	0.0097

M_8	GO:1902629	0.0097
M_8	GO:0006049	0.0097
M_8	GO:1900170	0.0097
M_8	GO:0006890	0.00981
M_8	GO:0002218	0.01173
M_8	GO:0000398	0.01176
M_8	GO:0048268	0.01436
M_8	GO:0051403	0.01557
M_8	GO:1902750	0.01903
M_8	GO:0031577	0.01923
M_8	GO:0021539	0.01931
M_8	GO:0002588	0.01931
M_8	GO:0006425	0.01931
M_8	GO:0006429	0.01931
M_8	GO:2001045	0.01931
M_8	GO:1901485	0.01931
M_8	GO:0060928	0.01931
M_8	GO:0016320	0.01931
M_8	GO:0006043	0.01931
M_8	GO:2000327	0.01931
M_8	GO:0072334	0.01931
M_8	GO:0070373	0.02331
M_8	GO:0006355	0.02507
M_8	GO:0006611	0.02593
M_8	GO:0048294	0.02883
M_8	GO:0006420	0.02883
M_8	GO:0006438	0.02883
M_8	GO:0006407	0.02883
M_8	GO:0070537	0.02883
M_8	GO:0010845	0.02883
M_8	GO:0070966	0.02883
M_8	GO:0010643	0.02883

M_8	GO:0006556	0.02883
M_8	GO:1902037	0.02883
M_8	GO:0002184	0.02883
M_8	GO:0006046	0.02883
M_8	GO:0015680	0.02883
M_8	GO:0002277	0.02883
M_8	GO:0045917	0.02883
M_8	GO:1901837	0.02883
M_8	GO:0042981	0.03231
M_8	GO:0006886	0.03285
M_8	GO:0015991	0.03409
M_8	GO:0000289	0.03409
M_8	GO:0006412	0.03525
M_8	GO:0071459	0.03784
M_8	GO:0048105	0.03826
M_8	GO:0007258	0.03826
M_8	GO:0061086	0.03826
M_8	GO:0051572	0.03826
M_8	GO:0006344	0.03826
M_8	GO:0002051	0.03826
M_8	GO:0071962	0.03826
M_8	GO:1904424	0.03826
M_8	GO:0060699	0.03826
M_8	GO:0000414	0.03826
M_8	GO:0001880	0.03826
M_8	GO:0043328	0.03826
M_8	GO:0031120	0.03826
M_8	GO:2001181	0.03826
M_8	GO:0007406	0.03826
M_8	GO:1901857	0.03826
M_8	GO:0031860	0.03826
M_8	GO:0006888	0.03978

M_8	GO:0035195	0.0404
M_8	GO:0043928	0.04061
M_8	GO:0006417	0.04249
M_8	GO:0006986	0.04257
M_8	GO:0071681	0.04759
M_8	GO:1903772	0.04759
M_8	GO:0006398	0.04759
M_8	GO:1904354	0.04759
M_8	GO:0031914	0.04759
M_8	GO:0034184	0.04759
M_8	GO:0010606	0.04759
M_8	GO:0000387	0.04996
M_8	GO:0090503	0.04996
M_9	GO:0051607	4E-10
M_9	GO:0045869	0.00000066
M_9	GO:0070383	0.00000066
M_9	GO:0060337	0.00000082
M_9	GO:0016554	0.0000026
M_9	GO:0080111	0.000023
M_9	GO:0010529	0.000024
M_9	GO:0002230	0.000051
M_9	GO:0009972	0.000073
M_9	GO:0000454	0.00022
M_9	GO:2000670	0.00022
M_9	GO:0060333	0.00024
M_9	GO:0045893	0.00025
M_9	GO:0071360	0.00027
M_9	GO:0045071	0.00047
M_9	GO:2001034	0.00074
M_9	GO:0072734	0.00074
M_9	GO:0009597	0.00074
M_9	GO:0006309	0.00076

M_9	GO:0001817	0.00123
M_9	GO:0045824	0.00124
M_9	GO:0032480	0.00141
M_9	GO:0072643	0.00151
M_9	GO:0016032	0.00176
M_9	GO:0035456	0.002
M_9	GO:1903265	0.00202
M_9	GO:0046598	0.00259
M_9	GO:0061635	0.00259
M_9	GO:0034058	0.00322
M_9	GO:0035457	0.00322
M_9	GO:0046007	0.00391
M_9	GO:0043123	0.00391
M_9	GO:0010506	0.00447
M_9	GO:1904469	0.00466
M_9	GO:0035563	0.00466
M_9	GO:0034315	0.00829
M_9	GO:0042033	0.00862
M_9	GO:0042095	0.00863
M_9	GO:0070241	0.00868
M_9	GO:0046032	0.00868
M_9	GO:0034346	0.00868
M_9	GO:1903434	0.00868
M_9	GO:1901224	0.00868
M_9	GO:0097194	0.00988
M_9	GO:0006974	0.01032
M_9	GO:0034389	0.01161
M_9	GO:0006471	0.01268
M_9	GO:0051865	0.01395
M_9	GO:0034344	0.01717
M_9	GO:0046709	0.01728
M_9	GO:0051877	0.01728

M_9	GO:0048203	0.01728
M_9	GO:2000156	0.01728
M_9	GO:0006313	0.01728
M_9	GO:0060702	0.01728
M_9	GO:0002519	0.01728
M_9	GO:0070094	0.01728
M_9	GO:0042231	0.01728
M_9	GO:0071344	0.01728
M_9	GO:1902216	0.01728
M_9	GO:0000493	0.01728
M_9	GO:0019060	0.01728
M_9	GO:1901301	0.01728
M_9	GO:0010868	0.01728
M_9	GO:2000556	0.01728
M_9	GO:0032834	0.01728
M_9	GO:1902524	0.01728
M_9	GO:1903378	0.01728
M_9	GO:1903929	0.01728
M_9	GO:2000639	0.01728
M_9	GO:1902044	0.01728
M_9	GO:2001190	0.01728
M_9	GO:0046294	0.01728
M_9	GO:0097401	0.01728
M_9	GO:0045355	0.01728
M_9	GO:0045358	0.01728
M_9	GO:0042531	0.0174
M_9	GO:0002456	0.01813
M_9	GO:2000778	0.01821
M_9	GO:0050718	0.02121
M_9	GO:0032481	0.02203
M_9	GO:0045087	0.02397
M_9	GO:0039530	0.02565

M_9	GO:0021747	0.02581
M_9	GO:0018312	0.02581
M_9	GO:1901666	0.02581
M_9	GO:0030200	0.02581
M_9	GO:0051097	0.02581
M_9	GO:1903093	0.02581
M_9	GO:0070681	0.02581
M_9	GO:0009236	0.02581
M_9	GO:0071492	0.02581
M_9	GO:0034473	0.02581
M_9	GO:0034476	0.02581
M_9	GO:0048022	0.02581
M_9	GO:0023021	0.02581
M_9	GO:0048388	0.02581
M_9	GO:2001184	0.02581
M_9	GO:0090086	0.02581
M_9	GO:0070979	0.02776
M_9	GO:0034080	0.0295
M_9	GO:1903955	0.03129
M_9	GO:0051092	0.03299
M_9	GO:0043928	0.03313
M_9	GO:0050798	0.03366
M_9	GO:0071346	0.03396
M_9	GO:0019076	0.0341
M_9	GO:1902806	0.03411
M_9	GO:2000346	0.03426
M_9	GO:0048133	0.03426
M_9	GO:0001920	0.03426
M_9	GO:0002730	0.03426
M_9	GO:1901537	0.03426
M_9	GO:1900245	0.03426
M_9	GO:0039534	0.03426

M_9	GO:0050717	0.03426
M_9	GO:0042253	0.03426
M_9	GO:0002051	0.03426
M_9	GO:0033088	0.03426
M_9	GO:0006231	0.03426
M_9	GO:2000562	0.03426
M_9	GO:2000563	0.03426
M_9	GO:0006710	0.03426
M_9	GO:0035549	0.03426
M_9	GO:0031120	0.03426
M_9	GO:0060368	0.03426
M_9	GO:0051142	0.03426
M_9	GO:0019918	0.03426
M_9	GO:2001181	0.03426
M_9	GO:0006659	0.03426
M_9	GO:0045356	0.03426
M_9	GO:2001200	0.03426
M_9	GO:0045070	0.03691
M_9	GO:0006513	0.03852
M_9	GO:0006919	0.03887
M_9	GO:0006915	0.03889
M_9	GO:0034446	0.04058
M_9	GO:0051897	0.04177
M_9	GO:0031118	0.0421
M_9	GO:2000481	0.04264
M_9	GO:0031547	0.04264
M_9	GO:1902412	0.04264
M_9	GO:0045794	0.04264
M_9	GO:0019509	0.04264
M_9	GO:0071494	0.04264
M_9	GO:0006269	0.04264
M_9	GO:0032237	0.04264

M_9	GO:0006226	0.04264
M_9	GO:0050689	0.04264
M_9	GO:2000660	0.04264
M_9	GO:2000646	0.04264
M_9	GO:0015853	0.04264
M_9	GO:2001205	0.04264
M_9	GO:0019985	0.0492
M_9	GO:0032091	0.04973
M_9	GO:0060760	0.04976
M_10	GO:0045945	0.0013
M_10	GO:1902902	0.0015
M_10	GO:0071285	0.0018
M_10	GO:0043687	0.002
M_10	GO:0043101	0.0027
M_10	GO:0010950	0.0034
M_10	GO:0032968	0.0035
M_10	GO:0032211	0.0043
M_10	GO:0034724	0.0049
M_10	GO:1904247	0.0049
M_10	GO:1990744	0.0049
M_10	GO:0000173	0.0049
M_10	GO:0006148	0.0049
M_10	GO:0006738	0.0049
M_10	GO:0009956	0.0049
M_10	GO:0061136	0.0056
M_10	GO:0010499	0.0061
M_10	GO:0045736	0.0095
M_10	GO:0015951	0.0098
M_10	GO:0019543	0.0098
M_10	GO:0046327	0.0098
M_10	GO:0045872	0.0098
M_10	GO:0042357	0.0098

M_10	GO:0006617	0.0098
M_10	GO:0060849	0.0098
M_10	GO:0030389	0.0098
M_10	GO:0034418	0.0098
M_10	GO:0098508	0.0098
M_10	GO:0006286	0.0098
M_10	GO:0046778	0.0098
M_10	GO:0016579	0.0117
M_10	GO:0000387	0.0142
M_10	GO:0008630	0.0142
M_10	GO:1903679	0.0147
M_10	GO:2000297	0.0147
M_10	GO:0018312	0.0147
M_10	GO:0090361	0.0147
M_10	GO:0010836	0.0147
M_10	GO:1903507	0.0188
M_10	GO:1904637	0.0195
M_10	GO:0060011	0.0195
M_10	GO:0002949	0.0195
M_10	GO:1901842	0.0195
M_10	GO:0032202	0.0195
M_10	GO:0006116	0.0195
M_10	GO:0033615	0.0195
M_10	GO:0019918	0.0195
M_10	GO:0006772	0.0195
M_10	GO:1901796	0.0236
M_10	GO:0030263	0.0243
M_10	GO:0090646	0.0243
M_10	GO:0097039	0.0243
M_10	GO:0043985	0.0291
M_10	GO:0021861	0.0339
M_10	GO:0006382	0.0339

M_10	GO:0018344	0.0339
M_10	GO:0032324	0.0339
M_10	GO:0023035	0.0386
M_10	GO:0006610	0.0386
M_10	GO:0006616	0.0386
M_10	GO:0061051	0.0386
M_10	GO:0051026	0.0386
M_10	GO:0032981	0.0395
M_10	GO:0048208	0.0417
M_10	GO:0006278	0.0425
M_10	GO:0046548	0.0433
M_10	GO:0045657	0.0433
M_10	GO:0035562	0.0433
M_10	GO:0043044	0.0452
M_10	GO:0061157	0.0475
M_10	GO:0070970	0.048
M_10	GO:0006335	0.048
M_10	GO:0001682	0.048
M_10	GO:0042118	0.048
M_10	GO:0090502	0.05
M_11	GO:0045944	1.2E-12
M_11	GO:0071222	2.3E-10
M_11	GO:0000122	6.9E-09
M_11	GO:0090023	0.000000011
M_11	GO:0006955	0.000000048
M_11	GO:0051591	0.0000002
M_11	GO:0051726	0.00000036
M_11	GO:0042127	0.00000038
M_11	GO:0010469	0.00000064
M_11	GO:0006954	0.0000007
M_11	GO:0001706	0.0000008
M_11	GO:0007623	0.0000008

M_11	GO:0070098	0.00000082
M_11	GO:0044344	0.0000043
M_11	GO:0070498	0.0000049
M_11	GO:0061158	0.0000066
M_11	GO:0032870	0.0000078
M_11	GO:0043392	0.0000087
M_11	GO:0071850	0.0000089
M_11	GO:0060426	0.000015
M_11	GO:0035994	0.000019
M_11	GO:0051412	0.000037
M_11	GO:0035914	0.000039
M_11	GO:0061469	0.000042
M_11	GO:0042542	0.000044
M_11	GO:0043153	0.000045
M_11	GO:0045668	0.000046
M_11	GO:0071356	0.000072
M_11	GO:0043066	0.000076
M_11	GO:1904582	0.000085
M_11	GO:0000294	0.000085
M_11	GO:0008285	0.00011
M_11	GO:0048661	0.00013
M_11	GO:0060707	0.00016
M_11	GO:0070301	0.00018
M_11	GO:0034097	0.00019
M_11	GO:0045600	0.0002
M_11	GO:0032496	0.0002
M_11	GO:0045651	0.00021
M_11	GO:0007249	0.00024
M_11	GO:0071409	0.00025
M_11	GO:0015671	0.00026
M_11	GO:0070373	0.00029
M_11	GO:0038061	0.00031

M_11	GO:0045444	0.00032
M_11	GO:0019221	0.00036
M_11	GO:0045429	0.00037
M_11	GO:0051090	0.00037
M_11	GO:0043280	0.0004
M_11	GO:0010744	0.0004
M_11	GO:1901653	0.00043
M_11	GO:0010941	0.00047
M_11	GO:0071347	0.00048
M_11	GO:0071364	0.0005
M_11	GO:0045415	0.0005
M_11	GO:0060708	0.0005
M_11	GO:0010888	0.00057
M_11	GO:0000185	0.00057
M_11	GO:0009611	0.00059
M_11	GO:0009612	0.0007
M_11	GO:0071277	0.00078
M_11	GO:0048382	0.00083
M_11	GO:0071376	0.00083
M_11	GO:0045766	0.00086
M_11	GO:1900745	0.00091
M_11	GO:0009314	0.00099
M_11	GO:0045616	0.00118
M_11	GO:0038066	0.0012
M_11	GO:0042536	0.00124
M_11	GO:0060136	0.00124
M_11	GO:1902172	0.00124
M_11	GO:0060710	0.00124
M_11	GO:0045672	0.00136
M_11	GO:0043065	0.00146
M_11	GO:0032922	0.00167
M_11	GO:0061614	0.0017

M_11	GO:0000188	0.00173
M_11	GO:0071499	0.00173
M_11	GO:0032364	0.00173
M_11	GO:0006977	0.00178
M_11	GO:0000060	0.0019
M_11	GO:0032270	0.00191
M_11	GO:0043433	0.00221
M_11	GO:1901222	0.00226
M_11	GO:0045080	0.00229
M_11	GO:0051549	0.00229
M_11	GO:0032966	0.00229
M_11	GO:0060452	0.00229
M_11	GO:0071316	0.00229
M_11	GO:0045647	0.00229
M_11	GO:0032715	0.00258
M_11	GO:0042119	0.00273
M_11	GO:0061014	0.00283
M_11	GO:0042744	0.00288
M_11	GO:0032691	0.0029
M_11	GO:2000304	0.00293
M_11	GO:0045657	0.00293
M_11	GO:0033209	0.00343
M_11	GO:0009629	0.00364
M_11	GO:0007179	0.00373
M_11	GO:2001240	0.00376
M_11	GO:0060674	0.00433
M_11	GO:0036499	0.00438
M_11	GO:0002042	0.00439
M_11	GO:0031953	0.00442
M_11	GO:0060213	0.00442
M_11	GO:0032570	0.00479
M_11	GO:0032088	0.00491

M_11	GO:0071276	0.00516
M_11	GO:0001836	0.00524
M_11	GO:0045779	0.00528
M_11	GO:0048711	0.00528
M_11	GO:0001938	0.00591
M_11	GO:0030574	0.00597
M_11	GO:2000379	0.00601
M_11	GO:0050691	0.00614
M_11	GO:0051028	0.0064
M_11	GO:0051403	0.0067
M_11	GO:0044849	0.00719
M_11	GO:0015701	0.00731
M_11	GO:0002446	0.00769
M_11	GO:0034614	0.0078
M_11	GO:0048566	0.00816
M_11	GO:0035987	0.00881
M_11	GO:0031086	0.00919
M_11	GO:0007622	0.00921
M_11	GO:1901676	0.00925
M_11	GO:0072750	0.00925
M_11	GO:0045083	0.00925
M_11	GO:0051545	0.00925
M_11	GO:1904246	0.00925
M_11	GO:0061670	0.00925
M_11	GO:1902631	0.00925
M_11	GO:0021660	0.00925
M_11	GO:0021666	0.00925
M_11	GO:0034148	0.00925
M_11	GO:0090010	0.00925
M_11	GO:0001966	0.00925
M_11	GO:0038097	0.00925
M_11	GO:0010085	0.00925

M_11	GO:0061048	0.00925
M_11	GO:1903917	0.00925
M_11	GO:0036164	0.00925
M_11	GO:0097403	0.00925
M_11	GO:0070429	0.00925
M_11	GO:2000872	0.00925
M_11	GO:0048327	0.00925
M_11	GO:0002041	0.00925
M_11	GO:0001315	0.00925
M_11	GO:0003069	0.00925
M_11	GO:0072303	0.00925
M_11	GO:0009444	0.00925
M_11	GO:0050726	0.00925
M_11	GO:0048790	0.00925
M_11	GO:0060802	0.00925
M_11	GO:0060806	0.00925
M_11	GO:1901835	0.00925
M_11	GO:2000810	0.00926
M_11	GO:0035313	0.00937
M_11	GO:0032495	0.00937
M_11	GO:0035970	0.00937
M_11	GO:0070102	0.01055
M_11	GO:0042981	0.01056
M_11	GO:0032868	0.01149
M_11	GO:2001275	0.0118
M_11	GO:0043011	0.0118
M_11	GO:0030168	0.012
M_11	GO:0042832	0.01311
M_11	GO:2000178	0.01311
M_11	GO:0043434	0.01356
M_11	GO:0043312	0.01431
M_11	GO:0036120	0.01448

M_11	GO:0033962	0.01448
M_11	GO:0060716	0.01448
M_11	GO:0046697	0.01448
M_11	GO:0006974	0.01459
M_11	GO:0010803	0.01504
M_11	GO:0046330	0.01569
M_11	GO:0060395	0.01577
M_11	GO:0045669	0.01577
M_11	GO:0043491	0.01674
M_11	GO:0051291	0.01689
M_11	GO:0010884	0.01739
M_11	GO:0051384	0.01778
M_11	GO:2000278	0.01815
M_11	GO:0070427	0.0183
M_11	GO:0010957	0.0183
M_11	GO:0000272	0.01832
M_11	GO:0010868	0.01841
M_11	GO:0002268	0.01841
M_11	GO:0045082	0.01841
M_11	GO:0035026	0.01841
M_11	GO:0001831	0.01841
M_11	GO:0071506	0.01841
M_11	GO:0072573	0.01841
M_11	GO:0002876	0.01841
M_11	GO:0002879	0.01841
M_11	GO:2000342	0.01841
M_11	GO:2000349	0.01841
M_11	GO:2000334	0.01841
M_11	GO:0002543	0.01841
M_11	GO:0035349	0.01841
M_11	GO:0014740	0.01841
M_11	GO:0033505	0.01841

M_11	GO:2000777	0.01841
M_11	GO:1901383	0.01841
M_11	GO:0060460	0.01841
M_11	GO:0019075	0.01841
M_11	GO:0042942	0.01841
M_11	GO:0061394	0.01841
M_11	GO:1900224	0.01841
M_11	GO:0080121	0.01841
M_11	GO:1902310	0.01841
M_11	GO:2000627	0.01841
M_11	GO:0090291	0.01841
M_11	GO:0050725	0.01841
M_11	GO:0002384	0.01841
M_11	GO:0097533	0.01841
M_11	GO:0045994	0.01841
M_11	GO:0003366	0.01841
M_11	GO:0003342	0.01841
M_11	GO:0072593	0.01866
M_11	GO:0045765	0.01871
M_11	GO:0098586	0.01876
M_11	GO:1902895	0.02053
M_11	GO:0050901	0.02053
M_11	GO:0042531	0.02057
M_11	GO:0009615	0.02229
M_11	GO:0038095	0.02288
M_11	GO:0045662	0.02388
M_11	GO:0045786	0.02417
M_11	GO:0010575	0.02564
M_11	GO:0045665	0.02721
M_11	GO:0071385	0.02722
M_11	GO:2000630	0.02732
M_11	GO:0070424	0.02733

M_11	GO:0033687	0.02735
M_11	GO:0048730	0.02736
M_11	GO:2000144	0.02745
M_11	GO:0035767	0.02745
M_11	GO:0071480	0.02745
M_11	GO:1901671	0.02749
M_11	GO:0045079	0.02749
M_11	GO:0046985	0.02749
M_11	GO:0010693	0.02749
M_11	GO:0071677	0.02749
M_11	GO:0010621	0.02749
M_11	GO:0032765	0.02749
M_11	GO:0050975	0.02749
M_11	GO:0002541	0.02749
M_11	GO:0031630	0.02749
M_11	GO:0034140	0.02749
M_11	GO:0010956	0.02749
M_11	GO:0071931	0.02749
M_11	GO:2000182	0.02749
M_11	GO:0035166	0.02749
M_11	GO:0001922	0.02749
M_11	GO:0061031	0.02749
M_11	GO:0045226	0.02749
M_11	GO:0035284	0.02749
M_11	GO:1903984	0.02749
M_11	GO:0009051	0.02749
M_11	GO:2001180	0.02749
M_11	GO:2001183	0.02749
M_11	GO:0070433	0.02749
M_11	GO:0007113	0.02749
M_11	GO:0051365	0.02749
M_11	GO:0030185	0.02749

M_11	GO:0098759	0.02749
M_11	GO:1900625	0.02749
M_11	GO:0007500	0.02749
M_11	GO:0034059	0.02749
M_11	GO:0060559	0.02749
M_11	GO:1902714	0.02749
M_11	GO:0043547	0.02766
M_11	GO:0031668	0.02797
M_11	GO:0030509	0.02854
M_11	GO:0050679	0.02873
M_11	GO:0001656	0.02883
M_11	GO:0014065	0.02894
M_11	GO:0007568	0.02917
M_11	GO:0010837	0.0293
M_11	GO:0048662	0.0293
M_11	GO:1902230	0.0293
M_11	GO:0090503	0.03088
M_11	GO:0031663	0.03094
M_11	GO:0002576	0.0311
M_11	GO:0045736	0.03121
M_11	GO:0006367	0.03184
M_11	GO:0007517	0.0319
M_11	GO:0032722	0.03212
M_11	GO:0022617	0.03229
M_11	GO:0007265	0.03243
M_11	GO:0048008	0.03286
M_11	GO:0010467	0.03336
M_11	GO:0043549	0.0344
M_11	GO:0010942	0.03479
M_11	GO:0030513	0.03516
M_11	GO:0061418	0.03516
M_11	GO:0070536	0.03516

M_11	GO:0009991	0.03543
M_11	GO:0020027	0.03606
M_11	GO:0060761	0.03615
M_11	GO:1903025	0.03627
M_11	GO:0001660	0.03628
M_11	GO:0032204	0.03641
M_11	GO:0060623	0.03648
M_11	GO:0045054	0.03648
M_11	GO:0045085	0.03648
M_11	GO:0003166	0.03648
M_11	GO:0006021	0.03648
M_11	GO:0034136	0.03648
M_11	GO:0002677	0.03648
M_11	GO:0060005	0.03648
M_11	GO:1904637	0.03648
M_11	GO:0061043	0.03648
M_11	GO:0061030	0.03648
M_11	GO:1990441	0.03648
M_11	GO:0070318	0.03648
M_11	GO:1990168	0.03648
M_11	GO:0002925	0.03648
M_11	GO:1900127	0.03648
M_11	GO:0002085	0.03648
M_11	GO:0007296	0.03648
M_11	GO:0032058	0.03648
M_11	GO:0045578	0.03648
M_11	GO:0070294	0.03648
M_11	GO:0071421	0.03648
M_11	GO:0070212	0.03648
M_11	GO:0070213	0.03648
M_11	GO:2000676	0.03648
M_11	GO:1903347	0.03648

M_11	GO:0007598	0.03648
M_11	GO:0072108	0.03648
M_11	GO:0060574	0.03648
M_11	GO:0048861	0.03648
M_11	GO:0045918	0.03648
M_11	GO:0032755	0.03683
M_11	GO:1901216	0.03698
M_11	GO:0097009	0.03721
M_11	GO:0043524	0.03758
M_11	GO:0035556	0.03874
M_11	GO:0045597	0.03905
M_11	GO:0051973	0.0393
M_11	GO:0030218	0.03989
M_11	GO:0051092	0.0402
M_11	GO:0007274	0.04143
M_11	GO:0001707	0.04279
M_11	GO:0007565	0.04341
M_11	GO:0032526	0.04343
M_11	GO:0050852	0.04385
M_11	GO:0032680	0.04429
M_11	GO:1900153	0.04487
M_11	GO:0010574	0.04491
M_11	GO:0042541	0.04513
M_11	GO:0060664	0.04539
M_11	GO:1903208	0.04539
M_11	GO:0045084	0.04539
M_11	GO:1903772	0.04539
M_11	GO:0071504	0.04539
M_11	GO:1903898	0.04539
M_11	GO:0010641	0.04539
M_11	GO:0001661	0.04539
M_11	GO:2000391	0.04539

M_11	GO:0072679	0.04539
M_11	GO:0045347	0.04539
M_11	GO:0055118	0.04539
M_11	GO:0090074	0.04539
M_11	GO:0044828	0.04539
M_11	GO:1903936	0.04539
M_11	GO:2001268	0.04539
M_11	GO:1901164	0.04539
M_11	GO:0045586	0.04539
M_11	GO:0035523	0.04539
M_11	GO:2000660	0.04539
M_11	GO:0035509	0.04539
M_11	GO:0045653	0.04539
M_11	GO:0070842	0.04539
M_11	GO:0000738	0.04539
M_11	GO:0060591	0.04539
M_11	GO:0060509	0.04539
M_11	GO:0048286	0.04583
M_11	GO:0007507	0.04635
M_11	GO:0001558	0.04745
M_11	GO:0007566	0.04761
M_11	GO:0043068	0.04894
M_11	GO:0071479	0.04942
M_12	GO:0051415	0.00056
M_12	GO:0051694	0.00077
M_12	GO:0090557	0.00132
M_12	GO:0006600	0.00164
M_12	GO:0001711	0.00427
M_12	GO:0034975	0.00427
M_12	GO:0031122	0.00498
M_12	GO:0032968	0.0054
M_12	GO:0036148	0.0054

M_12	GO:1901350	0.00616
M_12	GO:0006062	0.00616
M_12	GO:0090389	0.00616
M_12	GO:0051160	0.00616
M_12	GO:0039520	0.00616
M_12	GO:0035037	0.00616
M_12	GO:0043105	0.00616
M_12	GO:0051089	0.00616
M_12	GO:0036150	0.00733
M_12	GO:0032402	0.00875
M_12	GO:0002479	0.01111
M_12	GO:0036152	0.01111
M_12	GO:0036151	0.01195
M_12	GO:0071264	0.01229
M_12	GO:1904527	0.01229
M_12	GO:0051685	0.01229
M_12	GO:0000455	0.01229
M_12	GO:0046081	0.01229
M_12	GO:0060733	0.01229
M_12	GO:0021935	0.01229
M_12	GO:0046370	0.01229
M_12	GO:0016340	0.01229
M_12	GO:0071477	0.01229
M_12	GO:0006807	0.01561
M_12	GO:0071108	0.01656
M_12	GO:0000454	0.01838
M_12	GO:0035166	0.01838
M_12	GO:0071596	0.01838
M_12	GO:1902626	0.01838
M_12	GO:0000023	0.01838
M_12	GO:0051088	0.01838
M_12	GO:2001106	0.01838

M_12	GO:0046103	0.01838
M_12	GO:0006154	0.01838
M_12	GO:1903422	0.01838
M_12	GO:0006654	0.02291
M_12	GO:0002821	0.02434
M_12	GO:0033499	0.02443
M_12	GO:0031591	0.02443
M_12	GO:0031585	0.02443
M_12	GO:0032057	0.02443
M_12	GO:1902817	0.02443
M_12	GO:0044878	0.02443
M_12	GO:0042766	0.02443
M_12	GO:0043103	0.02443
M_12	GO:0031120	0.02443
M_12	GO:0032417	0.02443
M_12	GO:0051388	0.03045
M_12	GO:0019640	0.03045
M_12	GO:0006226	0.03045
M_12	GO:0006798	0.03045
M_12	GO:0044828	0.03045
M_12	GO:0010961	0.03045
M_12	GO:0031398	0.03574
M_12	GO:1902774	0.03643
M_12	GO:0032792	0.03643
M_12	GO:0036492	0.03643
M_12	GO:0042796	0.03643
M_12	GO:0030579	0.03643
M_12	GO:0006552	0.03643
M_12	GO:1900425	0.03643
M_12	GO:0009838	0.03643
M_12	GO:0050942	0.03643
M_12	GO:0010571	0.03643

M_12	GO:0035333	0.04237
M_12	GO:0072711	0.04237
M_12	GO:0032466	0.04237
M_12	GO:2000601	0.04237
M_12	GO:2000483	0.04237
M_12	GO:0002553	0.04237
M_12	GO:0035331	0.04828
M_12	GO:0070417	0.04828
M_12	GO:0030050	0.04828
M_12	GO:0050847	0.04828
M_12	GO:0042117	0.04828
M_12	GO:0045647	0.04828
M_12	GO:0017183	0.04828
M_12	GO:0051225	0.04846
M_13	GO:0090110	0.0013
M_13	GO:0006044	0.0025
M_13	GO:0061077	0.0039
M_13	GO:2000560	0.0039
M_13	GO:2000438	0.0039
M_13	GO:0006193	0.0039
M_13	GO:0032889	0.0039
M_13	GO:0060466	0.0039
M_13	GO:0071660	0.0039
M_13	GO:0048232	0.0076
M_13	GO:0006655	0.0078
M_13	GO:0070904	0.0079
M_13	GO:0051877	0.0079
M_13	GO:0021987	0.0089
M_13	GO:0042769	0.0098
M_13	GO:0032218	0.0118
M_13	GO:0043504	0.0118
M_13	GO:1900063	0.0118

M_13	GO:1903721	0.0118
M_13	GO:0006272	0.0118
M_13	GO:1902990	0.0118
M_13	GO:1902953	0.0118
M_13	GO:0000086	0.0129
M_13	GO:0046726	0.0157
M_13	GO:0080154	0.0157
M_13	GO:0038108	0.0157
M_13	GO:0035549	0.0157
M_13	GO:0046208	0.0157
M_13	GO:0048499	0.0157
M_13	GO:0006458	0.0172
M_13	GO:0007030	0.0192
M_13	GO:0060296	0.0195
M_13	GO:0015939	0.0195
M_13	GO:0097068	0.0195
M_13	GO:0032077	0.0195
M_13	GO:0097039	0.0195
M_13	GO:0070309	0.0195
M_13	GO:1900028	0.0195
M_13	GO:0050915	0.0195
M_13	GO:0006102	0.0195
M_13	GO:1990592	0.0195
M_13	GO:0000738	0.0195
M_13	GO:0003400	0.0234
M_13	GO:1902065	0.0234
M_13	GO:0071651	0.0234
M_13	GO:0043161	0.0256
M_13	GO:0010633	0.0271
M_13	GO:0032049	0.0273
M_13	GO:0007144	0.0273
M_13	GO:0046604	0.0273

M_13	GO:0006771	0.0273
M_13	GO:1902741	0.0273
M_13	GO:0044829	0.0273
M_13	GO:0043123	0.028
M_13	GO:0060027	0.0311
M_13	GO:1990000	0.0311
M_13	GO:0014824	0.0311
M_13	GO:1902808	0.0345
M_13	GO:0022417	0.0349
M_13	GO:0021766	0.0379
M_13	GO:0015851	0.0387
M_13	GO:0033623	0.0387
M_13	GO:0035542	0.0387
M_13	GO:0045732	0.0401
M_13	GO:0046929	0.0425
M_13	GO:2000344	0.0425
M_13	GO:0072520	0.0425
M_13	GO:0080009	0.0425
M_13	GO:0060340	0.0425
M_13	GO:0050910	0.0425
M_13	GO:0060546	0.0425
M_13	GO:0006264	0.0425
M_13	GO:0019852	0.0425
M_13	GO:0006707	0.0463
M_13	GO:2001256	0.0463
M_13	GO:0070885	0.0463
M_13	GO:1904251	0.0463
M_13	GO:0033160	0.0463
M_13	GO:1904469	0.0463
M_13	GO:0015937	0.05
M_13	GO:0006596	0.05
M_13	GO:0007183	0.05

M_13	GO:0035723	0.05
M_13	GO:0016024	0.05
M_14	GO:0002250	2.5E-20
M_14	GO:0006955	2E-18
M_14	GO:0031295	6.9E-15
M_14	GO:0050852	3.1E-10
M_14	GO:0045059	0.00000013
M_14	GO:0006968	0.00000013
M_14	GO:0032609	0.00000043
M_14	GO:0019835	0.00000068
M_14	GO:0072540	0.0000012
M_14	GO:0030101	0.0000021
M_14	GO:0038083	0.0000025
M_14	GO:0042127	0.0000026
M_14	GO:0032729	0.0000028
M_14	GO:0045954	0.000005
M_14	GO:0045087	0.000006
M_14	GO:0045060	0.0000078
M_14	GO:0070098	0.00001
M_14	GO:0010820	0.000011
M_14	GO:0042110	0.000013
M_14	GO:0050862	0.000018
M_14	GO:0035589	0.000029
M_14	GO:0002669	0.00005
M_14	GO:0046641	0.000052
M_14	GO:0007169	0.00006
M_14	GO:0033634	0.000099
M_14	GO:0007204	0.00014
M_14	GO:0071222	0.0002
M_14	GO:0050690	0.00023
M_14	GO:0008626	0.00025
M_14	GO:0050850	0.0003

M_14	GO:0038114	0.00046
M_14	GO:0050776	0.00066
M_14	GO:0006954	0.00066
M_14	GO:0010818	0.00068
M_14	GO:0046007	0.00089
M_14	GO:0038110	0.00107
M_14	GO:0045086	0.00107
M_14	GO:0046854	0.00113
M_14	GO:0042102	0.00116
M_14	GO:0002407	0.00123
M_14	GO:0019722	0.00123
M_14	GO:0032740	0.00126
M_14	GO:0070234	0.00126
M_14	GO:0035723	0.00126
M_14	GO:0042267	0.00173
M_14	GO:0046649	0.00178
M_14	GO:0032695	0.00193
M_14	GO:0032496	0.0021
M_14	GO:0030593	0.00215
M_14	GO:0032693	0.00218
M_14	GO:0002827	0.00218
M_14	GO:0030217	0.00225
M_14	GO:0090026	0.00244
M_14	GO:0032753	0.00294
M_14	GO:0070233	0.00302
M_14	GO:0051897	0.00338
M_14	GO:0043383	0.00394
M_14	GO:0019724	0.00399
M_14	GO:0002517	0.004
M_14	GO:0009615	0.00408
M_14	GO:0002644	0.00411
M_14	GO:0002302	0.00411

M_14	GO:0032078	0.00411
M_14	GO:0045368	0.00411
M_14	GO:0043310	0.00411
M_14	GO:0038115	0.00411
M_14	GO:0038116	0.00411
M_14	GO:0030887	0.00411
M_14	GO:2000522	0.00411
M_14	GO:2000526	0.00411
M_14	GO:2000545	0.00411
M_14	GO:0002728	0.00411
M_14	GO:0035705	0.00411
M_14	GO:0090265	0.00411
M_14	GO:0002851	0.00411
M_14	GO:2000464	0.00411
M_14	GO:0097022	0.00411
M_14	GO:0002518	0.00411
M_14	GO:0002362	0.00411
M_14	GO:0032689	0.00533
M_14	GO:0035025	0.00547
M_14	GO:0001782	0.00588
M_14	GO:0007202	0.00588
M_14	GO:0043368	0.00708
M_14	GO:0090023	0.00717
M_14	GO:0045622	0.00788
M_14	GO:0002361	0.00809
M_14	GO:0070105	0.0082
M_14	GO:0045404	0.0082
M_14	GO:0060809	0.0082
M_14	GO:0033082	0.0082
M_14	GO:2000525	0.0082
M_14	GO:2000502	0.0082
M_14	GO:2000329	0.0082

M_14	GO:2000349	0.0082
M_14	GO:0045082	0.0082
M_14	GO:2000451	0.0082
M_14	GO:2000473	0.0082
M_14	GO:2000439	0.0082
M_14	GO:0051209	0.0089
M_14	GO:0051603	0.00929
M_14	GO:0032715	0.01011
M_14	GO:0007155	0.01012
M_14	GO:0051482	0.01064
M_14	GO:0032649	0.0108
M_14	GO:0072676	0.01098
M_14	GO:0002292	0.01113
M_14	GO:0043029	0.01119
M_14	GO:0002685	0.0112
M_14	GO:0051260	0.01171
M_14	GO:0006915	0.01203
M_14	GO:0001865	0.01211
M_14	GO:0048302	0.01212
M_14	GO:0045590	0.01228
M_14	GO:0002636	0.01228
M_14	GO:0043366	0.01228
M_14	GO:0048388	0.01228
M_14	GO:0032831	0.01228
M_14	GO:0002277	0.01228
M_14	GO:0033092	0.01228
M_14	GO:0010956	0.01228
M_14	GO:2000547	0.01228
M_14	GO:0048294	0.01228
M_14	GO:0031296	0.01228
M_14	GO:1902164	0.01228
M_14	GO:0045065	0.01228

M_14	GO:0050851	0.01338
M_14	GO:0043388	0.01349
M_14	GO:0042130	0.01456
M_14	GO:0034260	0.01471
M_14	GO:0032720	0.01598
M_14	GO:0038094	0.01601
M_14	GO:0002725	0.01612
M_14	GO:0045576	0.01616
M_14	GO:0050863	0.01626
M_14	GO:0002677	0.01634
M_14	GO:0001920	0.01634
M_14	GO:0002408	0.01634
M_14	GO:1903238	0.01634
M_14	GO:2000552	0.01634
M_14	GO:0046666	0.01634
M_14	GO:0030644	0.01634
M_14	GO:2000330	0.01634
M_14	GO:0045085	0.01634
M_14	GO:0045077	0.01634
M_14	GO:0097029	0.01634
M_14	GO:0002357	0.01634
M_14	GO:0046330	0.01642
M_14	GO:0050853	0.01814
M_14	GO:0070228	0.01959
M_14	GO:0002676	0.02011
M_14	GO:0038113	0.02038
M_14	GO:0097350	0.02038
M_14	GO:0070100	0.02038
M_14	GO:0032826	0.02038
M_14	GO:0051136	0.02038
M_14	GO:0035744	0.02038
M_14	GO:0002885	0.02038

M_14	GO:0031064	0.02038
M_14	GO:0002513	0.02038
M_14	GO:0033625	0.02038
M_14	GO:0097190	0.02268
M_14	GO:0072678	0.02279
M_14	GO:0032623	0.0228
M_14	GO:0050900	0.02335
M_14	GO:0032633	0.02344
M_14	GO:0002467	0.02409
M_14	GO:2000110	0.02441
M_14	GO:0032914	0.02441
M_14	GO:0032792	0.02441
M_14	GO:0031584	0.02441
M_14	GO:0097527	0.02441
M_14	GO:0043382	0.02441
M_14	GO:0032713	0.02441
M_14	GO:2000503	0.02441
M_14	GO:0042129	0.02472
M_14	GO:2000320	0.02803
M_14	GO:0002606	0.02842
M_14	GO:1902715	0.02842
M_14	GO:0032714	0.02842
M_14	GO:0060753	0.02842
M_14	GO:0021796	0.02842
M_14	GO:0035771	0.02842
M_14	GO:2000669	0.02842
M_14	GO:0010536	0.02842
M_14	GO:0002223	0.0285
M_14	GO:0007266	0.03152
M_14	GO:0006925	0.03199
M_14	GO:0007172	0.03242
M_14	GO:0035754	0.03242

M_14	GO:2000510	0.03242
M_14	GO:0035655	0.03242
M_14	GO:0007186	0.03375
M_14	GO:0032635	0.03563
M_14	GO:0038155	0.03639
M_14	GO:0001766	0.03639
M_14	GO:0001714	0.03639
M_14	GO:0048304	0.03639
M_14	GO:0072610	0.03639
M_14	GO:0032088	0.0372
M_14	GO:0006508	0.03818
M_14	GO:0050869	0.0393
M_14	GO:0045580	0.03974
M_14	GO:0001771	0.03981
M_14	GO:0002829	0.03982
M_14	GO:0035457	0.04036
M_14	GO:0001768	0.04036
M_14	GO:0030097	0.04048
M_14	GO:0002456	0.04209
M_14	GO:0007163	0.04329
M_14	GO:0050870	0.04353
M_14	GO:0051354	0.04373
M_14	GO:0032700	0.0443
M_14	GO:0042535	0.0443
M_14	GO:0016032	0.04525
M_14	GO:0002544	0.04695
M_14	GO:0071346	0.04703
M_14	GO:0006959	0.04736
M_14	GO:0003376	0.04823
M_14	GO:0010759	0.04823
M_14	GO:0051712	0.04823
M_14	GO:0043922	0.04823

M_14	GO:0006919	0.04879
M_14	GO:0050868	0.04886
M_15	GO:0051865	0.00035
M_15	GO:2000323	0.00049
M_15	GO:0098780	0.00286
M_15	GO:0000423	0.00474
M_15	GO:1904240	0.00577
M_15	GO:0001971	0.00577
M_15	GO:0032581	0.00577
M_15	GO:0010164	0.00577
M_15	GO:0051391	0.00577
M_15	GO:2001136	0.00577
M_15	GO:0033633	0.00577
M_15	GO:0034398	0.00577
M_15	GO:0036085	0.00577
M_15	GO:0097231	0.00577
M_15	GO:0006293	0.00643
M_15	GO:0070911	0.00977
M_15	GO:0070585	0.01125
M_15	GO:2000397	0.0115
M_15	GO:0009785	0.0115
M_15	GO:0051012	0.0115
M_15	GO:0006423	0.0115
M_15	GO:2001162	0.0115
M_15	GO:0006294	0.01207
M_15	GO:0010847	0.0172
M_15	GO:0006858	0.0172
M_15	GO:1902309	0.0172
M_15	GO:0036378	0.0172
M_15	GO:0000973	0.0172
M_15	GO:0034727	0.0172
M_15	GO:2000118	0.0172

M_15	GO:1903774	0.0172
M_15	GO:0002101	0.0172
M_15	GO:1903070	0.0172
M_15	GO:0016557	0.0172
M_15	GO:0035552	0.0172
M_15	GO:0035553	0.0172
M_15	GO:0006296	0.01922
M_15	GO:0015031	0.0204
M_15	GO:0032483	0.02286
M_15	GO:0010025	0.02286
M_15	GO:0090403	0.02286
M_15	GO:1903061	0.02286
M_15	GO:2001168	0.02286
M_15	GO:2000653	0.02286
M_15	GO:0019509	0.0285
M_15	GO:0008611	0.0285
M_15	GO:0046015	0.0285
M_15	GO:0045084	0.0285
M_15	GO:0008333	0.03007
M_15	GO:0098706	0.0341
M_15	GO:2001016	0.0341
M_15	GO:0061732	0.0341
M_15	GO:2000270	0.0341
M_15	GO:0048205	0.0341
M_15	GO:0033591	0.0341
M_15	GO:0010193	0.0341
M_15	GO:0015014	0.0341
M_15	GO:1903551	0.0341
M_15	GO:0060754	0.0341
M_15	GO:0045046	0.0341
M_15	GO:0043161	0.03614
M_15	GO:0035336	0.03933

M_15	GO:0045410	0.03967
M_15	GO:0032049	0.03967
M_15	GO:0001826	0.03967
M_15	GO:1903361	0.03967
M_15	GO:0032922	0.0401
M_15	GO:0006368	0.04215
M_15	GO:0007040	0.04415
M_15	GO:0002367	0.04496
M_15	GO:0017183	0.04521
M_15	GO:0006627	0.04521
M_15	GO:0019919	0.04521
M_15	GO:0060137	0.04521
M_15	GO:2000074	0.04521
M_15	GO:1900364	0.04521
M_15	GO:0022615	0.04521
M_15	GO:0071670	0.04521
M_15	GO:0036066	0.04521
M_15	GO:0031666	0.04521
M_16	GO:0006334	1E-30
M_16	GO:0006325	1.3E-21
M_16	GO:0006342	1.4E-18
M_16	GO:0002227	0.000000045
M_16	GO:0098532	0.00000023
M_16	GO:0016584	0.00000056
M_16	GO:0019731	0.0000013
M_16	GO:0000183	0.000003
M_16	GO:0080182	0.000003
M_16	GO:0016567	0.000018
M_16	GO:0050830	0.000022
M_16	GO:0060964	0.00015
M_16	GO:0006335	0.00016
M_16	GO:0038111	0.00061

M_16	GO:0051290	0.00134
M_16	GO:0045652	0.00338
M_16	GO:0032200	0.00569
M_16	GO:0031640	0.00584
M_16	GO:0045653	0.00967
M_16	GO:0050829	0.01128
M_16	GO:0071169	0.01542
M_16	GO:0051574	0.01924
M_16	GO:0070914	0.02305
M_16	GO:0006337	0.03626
M_16	GO:0010804	0.04
M_17	GO:0016926	0.0011
M_17	GO:0060124	0.0014
M_17	GO:0001935	0.0029
M_17	GO:0006446	0.0033
M_17	GO:0048194	0.0034
M_17	GO:0070830	0.004
M_17	GO:1903078	0.0043
M_17	GO:2000369	0.0056
M_17	GO:1902680	0.0062
M_17	GO:1900260	0.0063
M_17	GO:0036351	0.0063
M_17	GO:0036352	0.0063
M_17	GO:2000436	0.0063
M_17	GO:0060386	0.0063
M_17	GO:0018009	0.0063
M_17	GO:1990613	0.0063
M_17	GO:0015876	0.0063
M_17	GO:1904643	0.0063
M_17	GO:0036498	0.0067
M_17	GO:0048745	0.0069
M_17	GO:0071333	0.0072

M_17	GO:0046039	0.0107
M_17	GO:0005977	0.0122
M_17	GO:0000002	0.0124
M_17	GO:0044029	0.0125
M_17	GO:1990519	0.0125
M_17	GO:0006408	0.0125
M_17	GO:0051389	0.0125
M_17	GO:0050852	0.0126
M_17	GO:0042787	0.014
M_17	GO:0050690	0.0142
M_17	GO:0097009	0.0182
M_17	GO:0048010	0.0185
M_17	GO:0071409	0.0187
M_17	GO:2000312	0.0187
M_17	GO:0060689	0.0187
M_17	GO:0010636	0.0187
M_17	GO:1903760	0.0187
M_17	GO:1903764	0.0187
M_17	GO:0002191	0.0187
M_17	GO:0051684	0.0187
M_17	GO:2001271	0.0187
M_17	GO:0060964	0.0189
M_17	GO:0006370	0.0192
M_17	GO:1990830	0.0196
M_17	GO:0090168	0.0249
M_17	GO:0015917	0.0249
M_17	GO:0002318	0.0249
M_17	GO:0014042	0.0249
M_17	GO:1903286	0.0249
M_17	GO:0046952	0.0249
M_17	GO:0046167	0.0249
M_17	GO:0033031	0.0249

M_17	GO:0051660	0.0249
M_17	GO:0097494	0.0249
M_17	GO:0043553	0.0249
M_17	GO:0038028	0.0249
M_17	GO:1901097	0.0249
M_17	GO:1903588	0.0249
M_17	GO:0043622	0.0249
M_17	GO:2000653	0.0249
M_17	GO:0031146	0.0261
M_17	GO:0033138	0.0264
M_17	GO:0006613	0.0309
M_17	GO:0010032	0.031
M_17	GO:0021960	0.031
M_17	GO:0070535	0.031
M_17	GO:0048170	0.031
M_17	GO:1903753	0.031
M_17	GO:0006177	0.031
M_17	GO:0010501	0.0338
M_17	GO:0045197	0.0352
M_17	GO:0045747	0.0352
M_17	GO:0046580	0.0365
M_17	GO:0060022	0.0371
M_17	GO:1900264	0.0371
M_17	GO:0021957	0.0371
M_17	GO:0006552	0.0371
M_17	GO:0060613	0.0371
M_17	GO:0036481	0.0371
M_17	GO:2000270	0.0371
M_17	GO:0051661	0.0371
M_17	GO:0038031	0.0371
M_17	GO:0021631	0.0371
M_17	GO:0046833	0.0371

M_17	GO:0036297	0.0379
M_17	GO:0038095	0.0382
M_17	GO:0036092	0.0394
M_17	GO:0043488	0.0399
M_17	GO:1901215	0.04
M_17	GO:0016925	0.0408
M_17	GO:0040016	0.0431
M_17	GO:0021796	0.0431
M_17	GO:0006657	0.0431
M_17	GO:1902231	0.0431
M_17	GO:1902741	0.0431
M_17	GO:0030953	0.0431
M_17	GO:0072307	0.0431
M_17	GO:0006768	0.0431
M_17	GO:0019563	0.0431
M_17	GO:0031295	0.0437
M_17	GO:0032515	0.0452
M_17	GO:0021800	0.0492
M_17	GO:0051683	0.0492
M_17	GO:0034351	0.0492
M_17	GO:0019896	0.0492
M_17	GO:0006368	0.0492
M_18	GO:0097150	0.0035
M_18	GO:0046272	0.004
M_18	GO:0090367	0.004
M_18	GO:0071707	0.004
M_18	GO:0070202	0.004
M_18	GO:2000830	0.004
M_18	GO:0002926	0.004
M_18	GO:0098530	0.004
M_18	GO:1901919	0.004
M_18	GO:0036261	0.004

M_18	GO:2001145	0.004
M_18	GO:0090044	0.004
M_18	GO:1903465	0.004
M_18	GO:0061709	0.008
M_18	GO:0035607	0.008
M_18	GO:0006286	0.008
M_18	GO:0048378	0.008
M_18	GO:0014740	0.008
M_18	GO:0014806	0.008
M_18	GO:1902310	0.008
M_18	GO:0045976	0.008
M_18	GO:0097089	0.008
M_18	GO:0061723	0.008
M_18	GO:0046839	0.0096
M_18	GO:0006288	0.0119
M_18	GO:0002071	0.0119
M_18	GO:0097176	0.0119
M_18	GO:0051088	0.0119
M_18	GO:0045213	0.0119
M_18	GO:0090272	0.0119
M_18	GO:0034241	0.0119
M_18	GO:0021847	0.0119
M_18	GO:0034727	0.0119
M_18	GO:0000375	0.012
M_18	GO:0050999	0.0122
M_18	GO:0006631	0.0133
M_18	GO:0070164	0.0159
M_18	GO:0006021	0.0159
M_18	GO:0042997	0.0159
M_18	GO:0018343	0.0159
M_18	GO:0051106	0.0159
M_18	GO:0072757	0.0159

M_18	GO:2000546	0.0159
M_18	GO:0060665	0.0159
M_18	GO:1903347	0.0159
M_18	GO:2001206	0.0159
M_18	GO:0070640	0.0159
M_18	GO:0043536	0.019
M_18	GO:0030242	0.0198
M_18	GO:0015853	0.0198
M_18	GO:0006287	0.0198
M_18	GO:0045541	0.0198
M_18	GO:0040009	0.0198
M_18	GO:0000727	0.0198
M_18	GO:0090080	0.0198
M_18	GO:0035509	0.0198
M_18	GO:0001764	0.0207
M_18	GO:0032508	0.0218
M_18	GO:1904528	0.0237
M_18	GO:1903215	0.0237
M_18	GO:0033278	0.0237
M_18	GO:0010966	0.0237
M_18	GO:0003100	0.0237
M_18	GO:0001302	0.0237
M_18	GO:0003057	0.0237
M_18	GO:0022027	0.0237
M_18	GO:0043966	0.024
M_18	GO:0044806	0.0276
M_18	GO:1902570	0.0276
M_18	GO:0018344	0.0276
M_18	GO:0072711	0.0276
M_18	GO:0046604	0.0276
M_18	GO:0045454	0.0294
M_18	GO:0006625	0.0303

M_18	GO:0042117	0.0315
M_18	GO:0032511	0.0315
M_18	GO:0001561	0.0315
M_18	GO:0044387	0.0315
M_18	GO:0060484	0.0315
M_18	GO:0090045	0.0315
M_18	GO:0051549	0.0315
M_18	GO:0042795	0.0319
M_18	GO:0042473	0.0354
M_18	GO:0006290	0.0354
M_18	GO:0032933	0.0354
M_18	GO:0061549	0.0354
M_18	GO:0034454	0.0354
M_18	GO:0002084	0.0354
M_18	GO:0001542	0.0354
M_18	GO:0044341	0.0354
M_18	GO:0071679	0.0354
M_18	GO:2000304	0.0354
M_18	GO:0042355	0.0354
M_18	GO:0000290	0.0393
M_18	GO:0000012	0.0393
M_18	GO:0006355	0.0394
M_18	GO:0030433	0.0416
M_18	GO:0007435	0.0425
M_18	GO:0090181	0.0426
M_18	GO:0001833	0.0431
M_18	GO:0006527	0.0431
M_18	GO:0010763	0.0431
M_18	GO:0071340	0.0431
M_18	GO:0031284	0.0431
M_18	GO:0045793	0.0431
M_18	GO:0070262	0.0463

M_18	GO:0006368	0.0464
M_18	GO:1903140	0.0469
M_18	GO:0071044	0.0469
M_18	GO:0002192	0.0469
M_18	GO:2001256	0.0469
M_18	GO:0035563	0.0469
M_18	GO:0033631	0.0469
M_18	GO:0000045	0.0483
M_19	GO:2000002	0.0002
M_19	GO:0042149	0.00083
M_19	GO:0036500	0.00088
M_19	GO:1904262	0.00203
M_19	GO:0046856	0.00319
M_19	GO:0036499	0.00399
M_19	GO:0009235	0.00399
M_19	GO:0035481	0.00451
M_19	GO:0072428	0.00451
M_19	GO:0060975	0.00451
M_19	GO:0015854	0.00451
M_19	GO:0006713	0.00451
M_19	GO:1903926	0.00451
M_19	GO:0070981	0.00451
M_19	GO:0071529	0.00451
M_19	GO:0036153	0.00451
M_19	GO:0032361	0.00451
M_19	GO:0060691	0.00451
M_19	GO:1903577	0.00451
M_19	GO:0030421	0.00451
M_19	GO:0003259	0.00451
M_19	GO:0003210	0.00451
M_19	GO:0048075	0.00451
M_19	GO:0003236	0.00451

M_19	GO:1900100	0.00451
M_19	GO:0042664	0.00451
M_19	GO:0070368	0.00451
M_19	GO:1990442	0.00451
M_19	GO:0035364	0.00451
M_19	GO:0035344	0.00451
M_19	GO:0010506	0.00525
M_19	GO:0051726	0.00571
M_19	GO:0022008	0.00651
M_19	GO:1904562	0.009
M_19	GO:0015862	0.009
M_19	GO:0035470	0.009
M_19	GO:2000016	0.009
M_19	GO:0038018	0.009
M_19	GO:0044324	0.009
M_19	GO:0031247	0.009
M_19	GO:0090082	0.009
M_19	GO:0007518	0.009
M_19	GO:0035335	0.01015
M_19	GO:0045893	0.01043
M_19	GO:0001707	0.01182
M_19	GO:0006990	0.01347
M_19	GO:0060921	0.01347
M_19	GO:0060913	0.01347
M_19	GO:1904640	0.01347
M_19	GO:1990418	0.01347
M_19	GO:0000390	0.01347
M_19	GO:1903489	0.01347
M_19	GO:0021762	0.01469
M_19	GO:0072655	0.01737
M_19	GO:0043553	0.01792
M_19	GO:0007258	0.01792

M_19	GO:0031427	0.01792
M_19	GO:0030644	0.01792
M_19	GO:0003241	0.01792
M_19	GO:0045716	0.01792
M_19	GO:0042662	0.01792
M_19	GO:0007586	0.02139
M_19	GO:0015853	0.02235
M_19	GO:1903232	0.02235
M_19	GO:0071332	0.02235
M_19	GO:0090080	0.02235
M_19	GO:0008078	0.02235
M_19	GO:1903416	0.02235
M_19	GO:0006661	0.02589
M_19	GO:0032792	0.02676
M_19	GO:0045348	0.02676
M_19	GO:1903026	0.02676
M_19	GO:0006572	0.02676
M_19	GO:0060304	0.02676
M_19	GO:0001302	0.02676
M_19	GO:0000389	0.02676
M_19	GO:2001033	0.02676
M_19	GO:0031647	0.0268
M_19	GO:0031848	0.03115
M_19	GO:2000210	0.03115
M_19	GO:0014010	0.03115
M_19	GO:0007000	0.03115
M_19	GO:1903599	0.03115
M_19	GO:2000347	0.03115
M_19	GO:0035356	0.03115
M_19	GO:0009880	0.03381
M_19	GO:0060215	0.03552
M_19	GO:0001955	0.03552

M_19	GO:0006975	0.03552
M_19	GO:0060947	0.03552
M_19	GO:0046784	0.03552
M_19	GO:0032815	0.03552
M_19	GO:0010898	0.03552
M_19	GO:0006511	0.03926
M_19	GO:0009308	0.03977
M_19	GO:0007614	0.03987
M_19	GO:0034551	0.03987
M_19	GO:0048671	0.03987
M_19	GO:0061303	0.03987
M_19	GO:0010988	0.03987
M_19	GO:0003139	0.03987
M_19	GO:0031017	0.03987
M_19	GO:0042632	0.04189
M_19	GO:0043069	0.04366
M_19	GO:0000463	0.0442
M_19	GO:0006388	0.0442
M_19	GO:0006307	0.0442
M_19	GO:0006600	0.0442
M_19	GO:0003211	0.0442
M_19	GO:0001756	0.04509
M_19	GO:0035330	0.04851
M_19	GO:0006983	0.04851
M_19	GO:0006751	0.04851
M_19	GO:0030836	0.04851
M_19	GO:0007288	0.04851
M_19	GO:0051024	0.04851
M_19	GO:0042532	0.04851
M_19	GO:1990440	0.04851
M_20	GO:0007156	1E-30
M_20	GO:0007267	1E-30

M_20	GO:0007399	1E-30
M_20	GO:0007416	4.9E-23
M_20	GO:0016339	6.4E-23
M_20	GO:0007268	6.4E-12
M_20	GO:0060989	0.003
M_20	GO:0050808	0.019
M_20	GO:0009988	0.02
M_21	GO:0051225	0.00019
M_21	GO:0016446	0.00048
M_21	GO:0000038	0.00184
M_21	GO:1901805	0.00205
M_21	GO:1904457	0.00205
M_21	GO:1902380	0.00205
M_21	GO:0070434	0.00205
M_21	GO:0006298	0.00208
M_21	GO:0007030	0.00318
M_21	GO:0070165	0.00411
M_21	GO:0032764	0.00411
M_21	GO:1903464	0.00411
M_21	GO:0001676	0.00601
M_21	GO:0048294	0.00615
M_21	GO:0023021	0.00615
M_21	GO:0006680	0.00615
M_21	GO:0090063	0.00615
M_21	GO:0002181	0.00815
M_21	GO:1903061	0.0082
M_21	GO:0033031	0.0082
M_21	GO:0070370	0.0082
M_21	GO:0045629	0.0082
M_21	GO:0006625	0.00859
M_21	GO:0001895	0.00908
M_21	GO:1902219	0.01023

M_21	GO:2000427	0.0143
M_21	GO:2000767	0.0143
M_21	GO:0032436	0.01531
M_21	GO:0006614	0.01531
M_21	GO:1903265	0.01632
M_21	GO:0043380	0.01632
M_21	GO:1901029	0.01632
M_21	GO:0002634	0.01835
M_21	GO:0043589	0.01835
M_21	GO:0006637	0.01859
M_21	GO:0090084	0.02037
M_21	GO:0046512	0.02037
M_21	GO:0000463	0.02037
M_21	GO:0071356	0.022
M_21	GO:0010466	0.02207
M_21	GO:0060546	0.02238
M_21	GO:0071474	0.02238
M_21	GO:0032463	0.02238
M_21	GO:0002903	0.02439
M_21	GO:0097201	0.02439
M_21	GO:0006705	0.02439
M_21	GO:0045591	0.02439
M_21	GO:0000184	0.02558
M_21	GO:0048642	0.0264
M_21	GO:2000773	0.03239
M_21	GO:0006704	0.03637
M_21	GO:0031065	0.03637
M_21	GO:1901673	0.03637
M_21	GO:0070935	0.03637
M_21	GO:0035024	0.03835
M_21	GO:1902236	0.03835
M_21	GO:0010829	0.03835

M_21	GO:0051131	0.04033
M_21	GO:0006402	0.04046
M_21	GO:0050995	0.0423
M_21	GO:0033561	0.04427
M_21	GO:0042738	0.0482
M_21	GO:0030308	0.04856
M_21	GO:0055114	0.04939
M_22	GO:0034472	0.0000011
M_22	GO:0048496	0.00062
M_22	GO:0007207	0.00093
M_22	GO:0007214	0.00101
M_22	GO:0007197	0.0013
M_22	GO:0051965	0.00171
M_22	GO:0007156	0.00205
M_22	GO:0045494	0.00271
M_22	GO:0050877	0.00352
M_22	GO:1902476	0.00521
M_22	GO:0051932	0.00536
M_22	GO:0016339	0.00664
M_22	GO:0097120	0.00707
M_22	GO:0030818	0.00795
M_22	GO:0098746	0.00799
M_22	GO:0001808	0.00799
M_22	GO:0002787	0.00799
M_22	GO:0097115	0.00799
M_22	GO:0048789	0.00799
M_22	GO:1901846	0.00799
M_22	GO:0030832	0.00802
M_22	GO:0007157	0.01017
M_22	GO:0060078	0.01203
M_22	GO:0007268	0.01222
M_22	GO:0006940	0.01306

M_22	GO:0098609	0.0154
M_22	GO:0046486	0.01587
M_22	GO:1901383	0.01592
M_22	GO:0097535	0.01592
M_22	GO:0097536	0.01592
M_22	GO:0007402	0.01592
M_22	GO:0007208	0.01592
M_22	GO:0006542	0.01592
M_22	GO:1902232	0.01592
M_22	GO:0000902	0.01635
M_22	GO:0043252	0.01818
M_22	GO:0051491	0.02236
M_22	GO:0007352	0.02378
M_22	GO:0002331	0.02378
M_22	GO:0042427	0.02378
M_22	GO:0043397	0.02378
M_22	GO:0003331	0.02378
M_22	GO:0021965	0.02378
M_22	GO:0071691	0.02378
M_22	GO:2000302	0.02378
M_22	GO:0033563	0.02378
M_22	GO:0010845	0.02378
M_22	GO:0048240	0.02383
M_22	GO:0007601	0.02495
M_22	GO:0007605	0.02972
M_22	GO:0019228	0.03011
M_22	GO:0050953	0.03083
M_22	GO:0060011	0.03159
M_22	GO:0016080	0.03159
M_22	GO:0045636	0.03159
M_22	GO:0035965	0.03159
M_22	GO:0044336	0.03159

M_22	GO:0097113	0.03159
M_22	GO:0097114	0.03159
M_22	GO:0010025	0.03159
M_22	GO:0006663	0.03159
M_22	GO:0019740	0.03159
M_22	GO:0006311	0.03159
M_22	GO:2000346	0.03159
M_22	GO:0010841	0.03159
M_22	GO:0003056	0.03159
M_22	GO:0007525	0.03159
M_22	GO:0010513	0.03159
M_22	GO:0044278	0.03159
M_22	GO:0006643	0.03173
M_22	GO:0007271	0.03177
M_22	GO:0044331	0.03519
M_22	GO:0007187	0.03749
M_22	GO:0043029	0.03875
M_22	GO:0035315	0.03875
M_22	GO:0008616	0.03933
M_22	GO:1901164	0.03933
M_22	GO:0010961	0.03933
M_22	GO:2000809	0.03933
M_22	GO:0031133	0.03933
M_22	GO:0006682	0.03933
M_22	GO:0051884	0.03933
M_22	GO:0072553	0.03933
M_22	GO:0051966	0.04018
M_22	GO:0006182	0.04434
M_22	GO:0061512	0.04626
M_22	GO:2000171	0.04673
M_22	GO:0042713	0.04701
M_22	GO:0090158	0.04701

M_22	GO:0010757	0.04701
M_22	GO:1902474	0.04701
M_22	GO:0060316	0.04701
M_22	GO:0035524	0.04701
M_22	GO:0018242	0.04701
M_22	GO:1900244	0.04701
M_22	GO:0061002	0.04701
M_22	GO:0051271	0.04706
M_22	GO:0007420	0.04929
M_23	GO:0010918	0.0004
M_23	GO:0010457	0.00099
M_23	GO:0090297	0.00337
M_23	GO:1903007	0.00337
M_23	GO:0010808	0.00337
M_23	GO:0072389	0.00337
M_23	GO:1990046	0.00337
M_23	GO:0019886	0.0044
M_23	GO:0006258	0.00672
M_23	GO:1900210	0.00672
M_23	GO:0051228	0.00672
M_23	GO:0032877	0.00672
M_23	GO:0071049	0.00672
M_23	GO:1904498	0.01007
M_23	GO:0071930	0.01007
M_23	GO:0006990	0.01007
M_23	GO:0006680	0.01007
M_23	GO:0090063	0.01007
M_23	GO:0070936	0.01327
M_23	GO:0006457	0.0134
M_23	GO:0071034	0.0134
M_23	GO:0010040	0.0134
M_23	GO:0006409	0.0134

M_23	GO:0032510	0.0134
M_23	GO:0033499	0.0134
M_23	GO:0031914	0.01673
M_23	GO:0045347	0.01673
M_23	GO:0070842	0.01673
M_23	GO:0060718	0.01673
M_23	GO:0036498	0.01797
M_23	GO:0000915	0.02004
M_23	GO:1903862	0.02004
M_23	GO:0006513	0.02084
M_23	GO:0016567	0.02325
M_23	GO:0000467	0.02334
M_23	GO:0071051	0.02334
M_23	GO:0071038	0.02334
M_23	GO:0032466	0.02334
M_23	GO:0097151	0.02334
M_23	GO:0034475	0.02663
M_23	GO:0071035	0.02663
M_23	GO:1903715	0.02663
M_23	GO:2001171	0.02991
M_23	GO:0046548	0.02991
M_23	GO:0016081	0.02991
M_23	GO:0002091	0.02991
M_23	GO:0034454	0.02991
M_23	GO:1903748	0.02991
M_23	GO:1900426	0.02991
M_23	GO:0001188	0.02991
M_23	GO:0046487	0.02991
M_23	GO:0046549	0.03318
M_23	GO:0007016	0.03318
M_23	GO:0016139	0.03318
M_23	GO:0038003	0.03318

M_23	GO:0071985	0.03318
M_23	GO:0000012	0.03318
M_23	GO:0034427	0.03318
M_23	GO:0035845	0.03318
M_23	GO:0034214	0.03318
M_23	GO:0042147	0.0333
M_23	GO:0006888	0.0338
M_23	GO:0043161	0.03637
M_23	GO:0031953	0.03644
M_23	GO:0007017	0.03805
M_23	GO:0016188	0.03969
M_23	GO:0034982	0.03969
M_23	GO:0006068	0.03969
M_23	GO:0006069	0.03969
M_23	GO:0060707	0.03969
M_23	GO:0071712	0.04292
M_23	GO:0032802	0.04292
M_23	GO:0045006	0.04292
M_23	GO:0006915	0.04333
M_23	GO:0061640	0.04556
M_23	GO:0043508	0.04615
M_23	GO:0070365	0.04936
M_23	GO:0048172	0.04936
M_24	GO:0006564	0.00079
M_24	GO:0097284	0.00243
M_24	GO:0030705	0.00381
M_24	GO:0043981	0.00382
M_24	GO:0043982	0.00382
M_24	GO:0045727	0.00448
M_24	GO:0043984	0.00612
M_24	GO:0070129	0.00612
M_24	GO:0031460	0.00622

M_24	GO:1904404	0.00622
M_24	GO:0097045	0.00622
M_24	GO:0042128	0.00622
M_24	GO:0070627	0.00622
M_24	GO:0051086	0.00622
M_24	GO:1903259	0.00622
M_24	GO:0071528	0.00622
M_24	GO:0043538	0.00622
M_24	GO:0015692	0.00622
M_24	GO:0015689	0.00622
M_24	GO:0071301	0.00622
M_24	GO:2000632	0.00622
M_24	GO:0093001	0.00622
M_24	GO:0051131	0.00677
M_24	GO:2000114	0.00746
M_24	GO:0000278	0.00752
M_24	GO:0071157	0.00891
M_24	GO:0010586	0.00968
M_24	GO:0043312	0.01046
M_24	GO:0002543	0.01241
M_24	GO:0046619	0.01241
M_24	GO:1902304	0.01241
M_24	GO:0051025	0.01241
M_24	GO:1903775	0.01241
M_24	GO:0044565	0.01241
M_24	GO:0043000	0.01241
M_24	GO:0090065	0.01241
M_24	GO:0019264	0.01241
M_24	GO:0006431	0.01241
M_24	GO:0060702	0.01241
M_24	GO:0000354	0.01241
M_24	GO:0006396	0.01317

M_24	GO:0071353	0.01586
M_24	GO:0010039	0.01685
M_24	GO:0016032	0.01811
M_24	GO:1990120	0.01855
M_24	GO:2000301	0.01855
M_24	GO:0016480	0.01855
M_24	GO:0000973	0.01855
M_24	GO:0090156	0.01855
M_24	GO:0006543	0.01855
M_24	GO:1990785	0.01855
M_24	GO:0072717	0.01855
M_24	GO:0036265	0.01855
M_24	GO:0006933	0.01855
M_24	GO:1902630	0.01855
M_24	GO:0051973	0.0189
M_24	GO:0014047	0.02106
M_24	GO:1901998	0.02331
M_24	GO:0030049	0.02447
M_24	GO:0035621	0.02466
M_24	GO:0035616	0.02466
M_24	GO:0007089	0.02466
M_24	GO:0036369	0.02466
M_24	GO:0070574	0.02466
M_24	GO:1900060	0.02466
M_24	GO:0061589	0.02466
M_24	GO:1903874	0.02466
M_24	GO:0030423	0.02466
M_24	GO:0045716	0.02466
M_24	GO:1904628	0.02466
M_24	GO:1904637	0.02466
M_24	GO:0035087	0.02466
M_24	GO:0010724	0.02466

M_24	GO:1990441	0.02466
M_24	GO:0071421	0.02466
M_24	GO:0032480	0.02935
M_24	GO:0006406	0.02987
M_24	GO:0050689	0.03073
M_24	GO:0006537	0.03073
M_24	GO:0010606	0.03073
M_24	GO:1903298	0.03073
M_24	GO:0051599	0.03073
M_24	GO:0061590	0.03073
M_24	GO:0061591	0.03073
M_24	GO:0045794	0.03073
M_24	GO:1901843	0.03073
M_24	GO:0070221	0.03073
M_24	GO:1901525	0.03073
M_24	GO:0006269	0.03073
M_24	GO:0070200	0.03073
M_24	GO:0071494	0.03073
M_24	GO:0009448	0.03073
M_24	GO:0034198	0.03192
M_24	GO:0046931	0.03645
M_24	GO:1904417	0.03676
M_24	GO:0006868	0.03676
M_24	GO:2001046	0.03676
M_24	GO:0060527	0.03676
M_24	GO:0006824	0.03676
M_24	GO:0006566	0.03676
M_24	GO:0045359	0.03676
M_24	GO:1902949	0.03676
M_24	GO:0035434	0.03676
M_24	GO:1902065	0.03676
M_24	GO:0007185	0.03676

M_24	GO:0098903	0.03676
M_24	GO:0010265	0.03676
M_24	GO:0046833	0.03676
M_24	GO:0016925	0.04014
M_24	GO:1903799	0.04237
M_24	GO:0045472	0.04238
M_24	GO:0019530	0.04276
M_24	GO:0010523	0.04276
M_24	GO:0035280	0.04276
M_24	GO:0031658	0.04276
M_24	GO:0090650	0.04276
M_24	GO:0097211	0.04276
M_24	GO:0006977	0.04752
M_24	GO:1903798	0.04786
M_24	GO:0006886	0.04827
M_24	GO:0006776	0.04871
M_24	GO:0046600	0.04871
M_24	GO:0016926	0.04871
M_24	GO:0048227	0.04871
M_24	GO:1990000	0.04871
M_24	GO:1903715	0.04871
M_24	GO:0061577	0.04871
M_24	GO:0002138	0.04871
M_24	GO:0070197	0.04871
M_24	GO:2000643	0.04871
M_24	GO:0045008	0.04871
M_25	GO:0009952	1.2E-19
M_25	GO:0045944	5.6E-09
M_25	GO:0048704	7.6E-09
M_25	GO:0009954	0.0000002
M_25	GO:0006357	0.00000021
M_25	GO:0030326	0.00000027

M_25	GO:0000122	0.0000096
M_25	GO:0048665	0.000037
M_25	GO:0001501	0.0002
M_25	GO:0009953	0.0003
M_25	GO:0045617	0.00037
M_25	GO:0014807	0.00064
M_25	GO:0048703	0.00097
M_25	GO:0060272	0.00116
M_25	GO:0001656	0.00117
M_25	GO:0001759	0.00295
M_25	GO:0002076	0.00295
M_25	GO:0060065	0.00327
M_25	GO:0001764	0.00356
M_25	GO:1904659	0.0036
M_25	GO:1903403	0.00428
M_25	GO:0051355	0.00428
M_25	GO:0007634	0.00428
M_25	GO:1900036	0.00428
M_25	GO:0007495	0.00428
M_25	GO:2000287	0.00428
M_25	GO:0036306	0.00428
M_25	GO:0072267	0.00428
M_25	GO:0003421	0.00428
M_25	GO:0071387	0.00428
M_25	GO:0060678	0.00428
M_25	GO:2001208	0.00428
M_25	GO:0060045	0.00432
M_25	GO:0048706	0.00458
M_25	GO:0030878	0.0047
M_25	GO:0003148	0.00509
M_25	GO:0035115	0.00776
M_25	GO:0030513	0.00825

M_25	GO:0021658	0.00854
M_25	GO:0035470	0.00854
M_25	GO:0060764	0.00854
M_25	GO:0048343	0.00854
M_25	GO:0060876	0.00854
M_25	GO:0035922	0.00854
M_25	GO:0060577	0.00854
M_25	GO:0003193	0.00854
M_25	GO:0021599	0.00854
M_25	GO:0021568	0.00854
M_25	GO:1902257	0.00854
M_25	GO:2001055	0.00854
M_25	GO:0055078	0.00856
M_25	GO:0001569	0.00929
M_25	GO:0071392	0.00982
M_25	GO:0050905	0.01073
M_25	GO:0010360	0.01279
M_25	GO:0015820	0.01279
M_25	GO:0060743	0.01279
M_25	GO:0097376	0.01279
M_25	GO:0010159	0.01279
M_25	GO:0035284	0.01279
M_25	GO:0090427	0.01279
M_25	GO:0021986	0.01279
M_25	GO:0072076	0.01279
M_25	GO:0072011	0.01279
M_25	GO:0010909	0.01279
M_25	GO:0051795	0.01279
M_25	GO:0061146	0.01279
M_25	GO:0035880	0.01279
M_25	GO:0060435	0.01279
M_25	GO:0021524	0.01279

M_25	GO:0060166	0.01279
M_25	GO:0001974	0.01331
M_25	GO:0007588	0.01363
M_25	GO:0001709	0.01394
M_25	GO:0010107	0.01394
M_25	GO:0030154	0.01561
M_25	GO:0021615	0.01702
M_25	GO:0048370	0.01702
M_25	GO:0060574	0.01702
M_25	GO:0021764	0.01702
M_25	GO:0060535	0.01702
M_25	GO:0060364	0.01702
M_25	GO:0070837	0.01702
M_25	GO:0045906	0.01702
M_25	GO:0060480	0.01702
M_25	GO:0051594	0.01702
M_25	GO:1990410	0.01702
M_25	GO:0003272	0.02096
M_25	GO:0046879	0.02102
M_25	GO:0060903	0.02123
M_25	GO:0072144	0.02123
M_25	GO:0010991	0.02123
M_25	GO:0097411	0.02123
M_25	GO:2000582	0.02123
M_25	GO:0051793	0.02123
M_25	GO:0001661	0.02123
M_25	GO:0051005	0.02123
M_25	GO:0048880	0.02123
M_25	GO:0001666	0.02275
M_25	GO:0001525	0.02329
M_25	GO:0009653	0.02354
M_25	GO:0035725	0.02396

M_25	GO:0032792	0.02542
M_25	GO:0043615	0.02542
M_25	GO:0002176	0.02542
M_25	GO:0097647	0.02542
M_25	GO:0060527	0.02542
M_25	GO:0003175	0.02542
M_25	GO:2000675	0.02542
M_25	GO:0035019	0.02644
M_25	GO:0048844	0.02644
M_25	GO:0001942	0.02668
M_25	GO:0042733	0.02744
M_25	GO:0035878	0.02921
M_25	GO:0060575	0.02922
M_25	GO:0003416	0.02925
M_25	GO:0060017	0.02959
M_25	GO:0045656	0.02959
M_25	GO:0006701	0.02959
M_25	GO:0007144	0.02959
M_25	GO:0061312	0.02959
M_25	GO:0021902	0.02959
M_25	GO:0021796	0.02959
M_25	GO:0051792	0.02959
M_25	GO:0003180	0.02959
M_25	GO:1900107	0.02959
M_25	GO:0048702	0.02959
M_25	GO:0050891	0.02985
M_25	GO:0050909	0.03252
M_25	GO:0042472	0.03361
M_25	GO:2000020	0.03375
M_25	GO:0045647	0.03375
M_25	GO:0018401	0.03375
M_25	GO:0060638	0.03375

M_25	GO:0003223	0.03375
M_25	GO:0060484	0.03375
M_25	GO:0097084	0.03375
M_25	GO:0010510	0.03375
M_25	GO:0009887	0.03498
M_25	GO:0030182	0.03638
M_25	GO:0042473	0.03788
M_25	GO:0043587	0.03788
M_25	GO:0015824	0.03788
M_25	GO:0051601	0.03788
M_25	GO:0015816	0.03788
M_25	GO:0008354	0.03788
M_25	GO:0003344	0.03788
M_25	GO:0070561	0.03788
M_25	GO:0031017	0.03788
M_25	GO:0048589	0.03899
M_25	GO:0050896	0.03996
M_25	GO:0060021	0.04007
M_25	GO:0060038	0.04099
M_25	GO:0060351	0.04153
M_25	GO:0032275	0.04201
M_25	GO:0042118	0.04201
M_25	GO:0007191	0.04201
M_25	GO:0038063	0.04201
M_25	GO:0032328	0.04201
M_25	GO:0060346	0.04201
M_25	GO:2001214	0.04201
M_25	GO:0035542	0.04201
M_25	GO:0001503	0.04515
M_25	GO:0045835	0.04611
M_25	GO:0090331	0.04611
M_25	GO:0043116	0.04611

M_25	GO:0003198	0.04611
M_25	GO:0010870	0.04611
M_25	GO:0019852	0.04611
M_25	GO:0021521	0.04611
M_25	GO:0009611	0.04809
M_25	GO:0010092	0.04894
M_25	GO:0030166	0.04967
M_25	GO:0035050	0.04972
M_25	GO:0006171	0.04975
M_26	GO:0051301	1E-27
M_26	GO:0000281	5.3E-15
M_26	GO:0034080	1.4E-13
M_26	GO:0008283	7.3E-13
M_26	GO:0007052	2.9E-12
M_26	GO:0000079	5.6E-10
M_26	GO:0007080	1.1E-09
M_26	GO:0051988	1.3E-09
M_26	GO:0007059	1.6E-09
M_26	GO:0051256	0.000000016
M_26	GO:0032467	0.000000022
M_26	GO:0007088	0.000000023
M_26	GO:0090307	0.000000028
M_26	GO:0034501	0.000000048
M_26	GO:0000070	0.0000001
M_26	GO:0006890	0.0000001
M_26	GO:0000915	0.00000011
M_26	GO:0007018	0.00000019
M_26	GO:0051382	0.00000026
M_26	GO:0019886	0.00000032
M_26	GO:0007094	0.00000034
M_26	GO:0010389	0.00000045
M_26	GO:0031145	0.00000057

M_26	GO:0006977	0.00000084
M_26	GO:0000086	0.0000015
M_26	GO:0033314	0.0000023
M_26	GO:0000083	0.0000029
M_26	GO:0007077	0.0000034
M_26	GO:0010971	0.0000045
M_26	GO:0051315	0.0000048
M_26	GO:0032508	0.0000051
M_26	GO:0036297	0.0000059
M_26	GO:0051754	0.0000078
M_26	GO:0007049	0.000011
M_26	GO:0007076	0.000012
M_26	GO:0006260	0.000018
M_26	GO:0000712	0.00002
M_26	GO:0000076	0.000025
M_26	GO:0044806	0.000027
M_26	GO:0045132	0.000034
M_26	GO:0030071	0.000035
M_26	GO:0051983	0.000042
M_26	GO:0045842	0.000043
M_26	GO:0008608	0.000052
M_26	GO:0007095	0.000056
M_26	GO:0071897	0.000068
M_26	GO:0031297	0.0001
M_26	GO:0048146	0.00011
M_26	GO:0006302	0.00011
M_26	GO:0000278	0.00015
M_26	GO:0070979	0.00016
M_26	GO:0051310	0.00018
M_26	GO:0006270	0.00018
M_26	GO:1904668	0.00021
M_26	GO:1904498	0.00026

M_26	GO:0007079	0.00026
M_26	GO:0010705	0.00026
M_26	GO:0032465	0.00026
M_26	GO:0045143	0.00033
M_26	GO:0045737	0.00034
M_26	GO:0090656	0.00041
M_26	GO:0007051	0.00042
M_26	GO:0060236	0.00048
M_26	GO:0040001	0.00049
M_26	GO:0016321	0.00051
M_26	GO:0072757	0.00051
M_26	GO:0007064	0.00057
M_26	GO:0071539	0.00058
M_26	GO:0044770	0.00063
M_26	GO:0000082	0.00068
M_26	GO:0051383	0.0008
M_26	GO:0098535	0.00084
M_26	GO:0031536	0.00084
M_26	GO:0000132	0.00122
M_26	GO:0046602	0.00125
M_26	GO:0051661	0.00126
M_26	GO:0010760	0.00126
M_26	GO:0032466	0.00175
M_26	GO:0072711	0.00175
M_26	GO:0090267	0.00175
M_26	GO:0045184	0.00185
M_26	GO:0051445	0.00228
M_26	GO:0006281	0.0024
M_26	GO:0016572	0.00283
M_26	GO:0048478	0.00297
M_26	GO:0000733	0.00297
M_26	GO:0008284	0.00301

M_26	GO:0007098	0.00304
M_26	GO:0021987	0.00338
M_26	GO:0071479	0.00351
M_26	GO:0072520	0.00448
M_26	GO:1904385	0.00448
M_26	GO:0070317	0.00525
M_26	GO:2000042	0.00534
M_26	GO:0060707	0.00534
M_26	GO:0045787	0.00594
M_26	GO:0071712	0.00627
M_26	GO:2000781	0.00627
M_26	GO:0055015	0.00627
M_26	GO:0072425	0.00627
M_26	GO:0007019	0.00651
M_26	GO:0051321	0.00823
M_26	GO:0044843	0.00834
M_26	GO:0007062	0.00864
M_26	GO:0045003	0.00925
M_26	GO:0046680	0.0093
M_26	GO:0071932	0.0093
M_26	GO:0006843	0.0093
M_26	GO:1902480	0.0093
M_26	GO:1903126	0.0093
M_26	GO:1902983	0.0093
M_26	GO:1902977	0.0093
M_26	GO:0061078	0.0093
M_26	GO:0007344	0.0093
M_26	GO:2000751	0.0093
M_26	GO:2000775	0.0093
M_26	GO:1903096	0.0093
M_26	GO:0021685	0.0093
M_26	GO:0045769	0.0093

M_26	GO:0051456	0.0093
M_26	GO:0033316	0.0093
M_26	GO:0045870	0.0093
M_26	GO:0033301	0.0093
M_26	GO:0019088	0.0093
M_26	GO:0071168	0.00937
M_26	GO:0016446	0.00948
M_26	GO:0000920	0.01068
M_26	GO:1904355	0.01068
M_26	GO:0007099	0.01168
M_26	GO:0051056	0.01255
M_26	GO:0044772	0.01334
M_26	GO:0007346	0.01338
M_26	GO:0010569	0.01433
M_26	GO:1900182	0.01581
M_26	GO:0019985	0.01594
M_26	GO:1902850	0.01667
M_26	GO:0090344	0.01759
M_26	GO:0031577	0.01765
M_26	GO:0000022	0.01797
M_26	GO:0051307	0.01809
M_26	GO:0031106	0.01842
M_26	GO:0007057	0.01852
M_26	GO:1903438	0.01852
M_26	GO:1904172	0.01852
M_26	GO:0031938	0.01852
M_26	GO:0071283	0.01852
M_26	GO:0097681	0.01852
M_26	GO:1901291	0.01852
M_26	GO:0000732	0.01852
M_26	GO:0032877	0.01852
M_26	GO:0070194	0.01852

M_26	GO:0021693	0.01852
M_26	GO:0033624	0.01852
M_26	GO:0007284	0.01852
M_26	GO:1900195	0.01852
M_26	GO:1990426	0.01852
M_26	GO:0000320	0.01852
M_26	GO:0060045	0.01915
M_26	GO:0006468	0.02091
M_26	GO:0007283	0.02159
M_26	GO:0006284	0.02178
M_26	GO:0000819	0.02194
M_26	GO:0045740	0.02347
M_26	GO:0007091	0.02537
M_26	GO:0044774	0.02545
M_26	GO:2000816	0.02636
M_26	GO:0046605	0.02673
M_26	GO:0000706	0.02732
M_26	GO:0090306	0.02749
M_26	GO:0009263	0.02751
M_26	GO:0032487	0.02766
M_26	GO:0071930	0.02766
M_26	GO:0046601	0.02766
M_26	GO:0043988	0.02766
M_26	GO:0071139	0.02766
M_26	GO:1904117	0.02766
M_26	GO:1902990	0.02766
M_26	GO:2000984	0.02766
M_26	GO:0031291	0.02766
M_26	GO:0014038	0.02766
M_26	GO:0043504	0.02766
M_26	GO:0006267	0.02766
M_26	GO:0071373	0.02766

M_26	GO:0019860	0.02766
M_26	GO:0071314	0.02766
M_26	GO:0045875	0.02766
M_26	GO:0034421	0.02766
M_26	GO:0048589	0.02788
M_26	GO:0007131	0.02805
M_26	GO:0000018	0.02996
M_26	GO:1902749	0.03255
M_26	GO:1901796	0.03476
M_26	GO:0045840	0.03522
M_26	GO:0071459	0.0354
M_26	GO:0010833	0.036
M_26	GO:0034644	0.03623
M_26	GO:0009157	0.0365
M_26	GO:0051106	0.0367
M_26	GO:0071962	0.0367
M_26	GO:0071922	0.0367
M_26	GO:0007089	0.0367
M_26	GO:0060574	0.0367
M_26	GO:0043137	0.0367
M_26	GO:0060623	0.0367
M_26	GO:0016240	0.0367
M_26	GO:1990086	0.0367
M_26	GO:0045950	0.0367
M_26	GO:0044878	0.0367
M_26	GO:0046104	0.0367
M_26	GO:0002906	0.0367
M_26	GO:0090521	0.0367
M_26	GO:0030951	0.0367
M_26	GO:0006231	0.0367
M_26	GO:0006235	0.0367
M_26	GO:0072429	0.0367

M_26	GO:0006289	0.03962
M_26	GO:0032212	0.03974
M_26	GO:0097421	0.03974
M_26	GO:0051973	0.03974
M_26	GO:0071392	0.0419
M_26	GO:0010629	0.04271
M_26	GO:0070301	0.0436
M_26	GO:0051653	0.04411
M_26	GO:0007100	0.04488
M_26	GO:0035404	0.04514
M_26	GO:0016579	0.04519
M_26	GO:0010032	0.04567
M_26	GO:0072719	0.04567
M_26	GO:0090235	0.04567
M_26	GO:0090238	0.04567
M_26	GO:0019441	0.04567
M_26	GO:0000727	0.04567
M_26	GO:0070345	0.04567
M_26	GO:0044314	0.04567
M_26	GO:0060718	0.04567
M_26	GO:0030263	0.04567
M_26	GO:0051987	0.04567
M_26	GO:0035519	0.04567
M_26	GO:0018105	0.04716
M_26	GO:0048699	0.04756
M_26	GO:0071456	0.04958
M_27	GO:0000413	0.00024
M_27	GO:0031053	0.00251
M_27	GO:0035722	0.00522
M_27	GO:0071211	0.00765
M_27	GO:0070130	0.00765
M_27	GO:0090461	0.00765

M_27	GO:0009789	0.00765
M_27	GO:0019061	0.00765
M_27	GO:0036090	0.00765
M_27	GO:0061736	0.00765
M_27	GO:0006297	0.01323
M_27	GO:0032201	0.01323
M_27	GO:0042450	0.01524
M_27	GO:0046778	0.01524
M_27	GO:1904613	0.01524
M_27	GO:0070150	0.01524
M_27	GO:0042144	0.01524
M_27	GO:0090615	0.01524
M_27	GO:1990428	0.01524
M_27	GO:0061034	0.01524
M_27	GO:0001975	0.01553
M_27	GO:0098779	0.02261
M_27	GO:0019391	0.02277
M_27	GO:0046947	0.02277
M_27	GO:0044778	0.02277
M_27	GO:0060939	0.02277
M_27	GO:0007181	0.02277
M_27	GO:0017187	0.02277
M_27	GO:0015966	0.02277
M_27	GO:0016476	0.02277
M_27	GO:0051586	0.02277
M_27	GO:0006366	0.02335
M_27	GO:0045321	0.02988
M_27	GO:0070125	0.02998
M_27	GO:0000301	0.03025
M_27	GO:0060979	0.03025
M_27	GO:0034699	0.03025
M_27	GO:0046836	0.03025

M_27	GO:0070236	0.03025
M_27	GO:0044528	0.03025
M_27	GO:0006574	0.03025
M_27	GO:0010701	0.03025
M_27	GO:0032417	0.03025
M_27	GO:0090071	0.03025
M_27	GO:0035621	0.03025
M_27	GO:0006457	0.03097
M_27	GO:0070126	0.03175
M_27	GO:0043312	0.03196
M_27	GO:0006296	0.03248
M_27	GO:0042769	0.03412
M_27	GO:0007049	0.03714
M_27	GO:0071806	0.03752
M_27	GO:0071225	0.03767
M_27	GO:0040031	0.03767
M_27	GO:0044314	0.03767
M_27	GO:0003150	0.03767
M_27	GO:0060318	0.03767
M_27	GO:0035519	0.03767
M_27	GO:0061734	0.03767
M_27	GO:0006177	0.03767
M_27	GO:0006104	0.04503
M_27	GO:1902774	0.04503
M_27	GO:0042256	0.04503
M_27	GO:1904417	0.04503
M_27	GO:0030579	0.04503
M_27	GO:0010807	0.04503
M_27	GO:0045040	0.04503
M_27	GO:0042373	0.04503
M_27	GO:0022028	0.04503
M_28	GO:0009407	0.000091

M_28	GO:0043200	0.00018
M_28	GO:0071672	0.00018
M_28	GO:1901687	0.00023
M_28	GO:0071316	0.00083
M_28	GO:0034551	0.00107
M_28	GO:0001682	0.00133
M_28	GO:0070125	0.00144
M_28	GO:0070126	0.00157
M_28	GO:0019852	0.00162
M_28	GO:0035722	0.0021
M_28	GO:0006120	0.00224
M_28	GO:0071243	0.00346
M_28	GO:0006749	0.00407
M_28	GO:0032981	0.00537
M_28	GO:0000481	0.00554
M_28	GO:0071247	0.00554
M_28	GO:0014810	0.00554
M_28	GO:1902220	0.00554
M_28	GO:1903801	0.00554
M_28	GO:0035732	0.00554
M_28	GO:0006117	0.00554
M_28	GO:0043153	0.00652
M_28	GO:0045292	0.00955
M_28	GO:0045948	0.01044
M_28	GO:0000291	0.01084
M_28	GO:1902463	0.01104
M_28	GO:0036496	0.01104
M_28	GO:2000157	0.01104
M_28	GO:0090135	0.01104
M_28	GO:0014040	0.01104
M_28	GO:0070476	0.01104
M_28	GO:1903094	0.01104

M_28	GO:0060139	0.01104
M_28	GO:0071460	0.01104
M_28	GO:0045585	0.01104
M_28	GO:0045859	0.0117
M_28	GO:0006890	0.01209
M_28	GO:0032355	0.01376
M_28	GO:0043928	0.01433
M_28	GO:0042278	0.01566
M_28	GO:0006805	0.01625
M_28	GO:0016480	0.01652
M_28	GO:1901315	0.01652
M_28	GO:0060620	0.01652
M_28	GO:0060392	0.01652
M_28	GO:0071586	0.01652
M_28	GO:0060154	0.01652
M_28	GO:0015827	0.01652
M_28	GO:2000078	0.01652
M_28	GO:0018364	0.01652
M_28	GO:0042631	0.01652
M_28	GO:0015680	0.01652
M_28	GO:0019050	0.01652
M_28	GO:2000785	0.01782
M_28	GO:0032570	0.01782
M_28	GO:0000387	0.01782
M_28	GO:0033133	0.02197
M_28	GO:0051771	0.02197
M_28	GO:0051725	0.02197
M_28	GO:0030043	0.02197
M_28	GO:0033590	0.02197
M_28	GO:0021678	0.02197
M_28	GO:0097167	0.02197
M_28	GO:0072429	0.02197

M_28	GO:0033574	0.02263
M_28	GO:0033211	0.02738
M_28	GO:0071224	0.02738
M_28	GO:0000727	0.02738
M_28	GO:0010936	0.02738
M_28	GO:0051344	0.02738
M_28	GO:0034316	0.02738
M_28	GO:0045084	0.02738
M_28	GO:2000630	0.02738
M_28	GO:0051169	0.02743
M_28	GO:0034201	0.03277
M_28	GO:0042138	0.03277
M_28	GO:0060316	0.03277
M_28	GO:0032055	0.03277
M_28	GO:0033591	0.03277
M_28	GO:0071638	0.03277
M_28	GO:0005981	0.03277
M_28	GO:0060754	0.03277
M_28	GO:0097345	0.03783
M_28	GO:2000394	0.03813
M_28	GO:0007084	0.03813
M_28	GO:0007021	0.03813
M_28	GO:0097500	0.03813
M_28	GO:2000210	0.03813
M_28	GO:0035726	0.03813
M_28	GO:0007256	0.03813
M_28	GO:0000395	0.03813
M_28	GO:0001842	0.03813
M_28	GO:0032259	0.042
M_28	GO:0032927	0.04345
M_28	GO:0075713	0.04345
M_28	GO:0060155	0.04345

M_28	GO:1900364	0.04345
M_28	GO:2000035	0.04345
M_28	GO:0006457	0.04872
M_28	GO:0060315	0.04875
M_28	GO:2000234	0.04875
M_28	GO:0010918	0.04875
M_28	GO:0022417	0.04875
M_29	GO:1901741	0.000036
M_29	GO:0050918	0.000059
M_29	GO:0097490	0.000078
M_29	GO:0097491	0.000078
M_29	GO:0022614	0.00013
M_29	GO:0061549	0.00047
M_29	GO:0006953	0.00076
M_29	GO:0048010	0.00132
M_29	GO:0010884	0.0029
M_29	GO:1902946	0.0036
M_29	GO:1902285	0.0036
M_29	GO:1902378	0.00365
M_29	GO:0001697	0.00365
M_29	GO:1903435	0.00365
M_29	GO:0060301	0.00365
M_29	GO:0010477	0.00365
M_29	GO:0021649	0.00365
M_29	GO:0019089	0.00365
M_29	GO:0003148	0.00373
M_29	GO:0042127	0.00419
M_29	GO:0010575	0.00435
M_29	GO:0042730	0.00435
M_29	GO:0051491	0.00501
M_29	GO:0045907	0.00607
M_29	GO:1903375	0.00729

M_29	GO:1902336	0.00729
M_29	GO:0044111	0.00729
M_29	GO:0001698	0.00729
M_29	GO:0038195	0.00729
M_29	GO:0014846	0.00729
M_29	GO:0021828	0.00729
M_29	GO:0048203	0.00729
M_29	GO:0097374	0.00729
M_29	GO:0001970	0.00729
M_29	GO:0006196	0.00729
M_29	GO:0001798	0.00729
M_29	GO:0097102	0.00729
M_29	GO:0046086	0.00729
M_29	GO:0010768	0.00729
M_29	GO:0061441	0.00729
M_29	GO:1902042	0.00764
M_29	GO:1901998	0.00849
M_29	GO:0002063	0.0103
M_29	GO:0007596	0.0106
M_29	GO:0060058	0.01092
M_29	GO:0045626	0.01092
M_29	GO:0036486	0.01092
M_29	GO:0038183	0.01092
M_29	GO:0060978	0.01092
M_29	GO:0010980	0.01092
M_29	GO:2000097	0.01092
M_29	GO:0043316	0.01092
M_29	GO:0010951	0.01212
M_29	GO:0006935	0.01276
M_29	GO:0043371	0.01432
M_29	GO:0045576	0.01435
M_29	GO:0036337	0.01453

M_29	GO:0060686	0.01453
M_29	GO:0038165	0.01453
M_29	GO:1901166	0.01453
M_29	GO:1990834	0.01453
M_29	GO:0061551	0.01453
M_29	GO:0015722	0.01453
M_29	GO:0044691	0.01453
M_29	GO:2001137	0.01453
M_29	GO:1903690	0.01453
M_29	GO:0097278	0.01453
M_29	GO:0002051	0.01453
M_29	GO:0043378	0.01453
M_29	GO:0060326	0.01563
M_29	GO:0001934	0.01655
M_29	GO:0006958	0.01752
M_29	GO:1903307	0.01787
M_29	GO:0048846	0.01789
M_29	GO:2000401	0.0179
M_29	GO:0060666	0.01813
M_29	GO:0033211	0.01813
M_29	GO:1904261	0.01813
M_29	GO:0015939	0.01813
M_29	GO:0060385	0.01813
M_29	GO:0002457	0.01813
M_29	GO:0018057	0.01813
M_29	GO:0097368	0.01813
M_29	GO:0051388	0.01813
M_29	GO:0021637	0.01813
M_29	GO:2001268	0.01813
M_29	GO:0046813	0.01813
M_29	GO:0036498	0.02094
M_29	GO:0030201	0.02108

M_29	GO:0072678	0.02111
M_29	GO:0034201	0.02172
M_29	GO:1902966	0.02172
M_29	GO:0097527	0.02172
M_29	GO:0072683	0.02172
M_29	GO:0015015	0.02172
M_29	GO:0060745	0.02172
M_29	GO:0006955	0.0222
M_29	GO:0008360	0.02284
M_29	GO:0032489	0.0253
M_29	GO:0060558	0.0253
M_29	GO:0045630	0.0253
M_29	GO:2000427	0.0253
M_29	GO:0032287	0.0253
M_29	GO:0021785	0.0253
M_29	GO:0097475	0.0253
M_29	GO:0035771	0.0253
M_29	GO:0048842	0.0253
M_29	GO:0021612	0.0253
M_29	GO:0043320	0.0253
M_29	GO:0071455	0.0253
M_29	GO:0002438	0.02844
M_29	GO:0030854	0.02886
M_29	GO:0002291	0.02886
M_29	GO:0035754	0.02886
M_29	GO:2000643	0.02886
M_29	GO:0050847	0.02886
M_29	GO:0006024	0.03144
M_29	GO:1903428	0.03198
M_29	GO:0070561	0.03241
M_29	GO:0042908	0.03241
M_29	GO:0002315	0.03241

M_29	GO:0060982	0.03241
M_29	GO:0071679	0.03241
M_29	GO:0090557	0.03241
M_29	GO:0035860	0.03241
M_29	GO:0071394	0.03241
M_29	GO:0071260	0.03303
M_29	GO:0001938	0.03303
M_29	GO:0031669	0.03457
M_29	GO:0021675	0.03496
M_29	GO:0071985	0.03595
M_29	GO:0061299	0.03595
M_29	GO:0032836	0.03595
M_29	GO:0044539	0.03595
M_29	GO:0006930	0.03595
M_29	GO:2001269	0.03595
M_29	GO:0030948	0.03595
M_29	GO:0031529	0.03895
M_29	GO:1903670	0.03898
M_29	GO:0010820	0.03947
M_29	GO:0042178	0.03947
M_29	GO:0016264	0.03947
M_29	GO:0071803	0.03947
M_29	GO:0030036	0.03995
M_29	GO:0055074	0.04198
M_29	GO:0070885	0.04298
M_29	GO:0090197	0.04298
M_29	GO:1902287	0.04298
M_29	GO:0070269	0.04298
M_29	GO:2000650	0.04298
M_29	GO:0030208	0.04298
M_29	GO:1990830	0.0437
M_29	GO:0031638	0.04536

M_29	GO:0010839	0.04648
M_29	GO:0048935	0.04648
M_29	GO:0036109	0.04648
M_29	GO:0045198	0.04648
M_29	GO:0001771	0.04648
M_29	GO:0034497	0.04648
M_29	GO:0002087	0.04648
M_29	GO:0043306	0.04648
M_29	GO:0040036	0.04925
M_29	GO:0002693	0.04997
M_29	GO:0042487	0.04997
M_29	GO:0006957	0.04997
M_29	GO:0001867	0.04997
M_30	GO:0002098	0.0037
M_30	GO:0006516	0.0041
M_30	GO:1903204	0.0041
M_30	GO:0048208	0.0054
M_30	GO:0018400	0.0054
M_30	GO:0001552	0.0054
M_30	GO:0051257	0.0054
M_30	GO:0043972	0.0054
M_30	GO:0039521	0.0054
M_30	GO:0001680	0.0054
M_30	GO:0000011	0.0054
M_30	GO:0007403	0.0054
M_30	GO:1904577	0.0054
M_30	GO:1904501	0.0054
M_30	GO:0061324	0.0054
M_30	GO:0051413	0.0054
M_30	GO:0015031	0.0069
M_30	GO:2000368	0.0107
M_30	GO:2000355	0.0107

M_30	GO:1990791	0.0107
M_30	GO:0038158	0.0107
M_30	GO:0002337	0.0107
M_30	GO:0090298	0.0107
M_30	GO:0072053	0.0107
M_30	GO:0072054	0.0107
M_30	GO:1901291	0.0107
M_30	GO:0046168	0.0107
M_30	GO:0045976	0.0107
M_30	GO:0044830	0.0107
M_30	GO:0043060	0.0107
M_30	GO:0044565	0.0107
M_30	GO:0010124	0.0107
M_30	GO:0090038	0.0107
M_30	GO:2000017	0.0107
M_30	GO:1901874	0.0107
M_30	GO:0044334	0.0107
M_30	GO:0045041	0.0107
M_30	GO:0000289	0.0112
M_30	GO:0000737	0.0112
M_30	GO:0006355	0.0128
M_30	GO:0043928	0.0135
M_30	GO:0006298	0.0135
M_30	GO:0007249	0.0143
M_30	GO:0050832	0.0151
M_30	GO:0006513	0.0157
M_30	GO:0060066	0.016
M_30	GO:0003340	0.016
M_30	GO:0034727	0.016
M_30	GO:0097676	0.016
M_30	GO:2000286	0.016
M_30	GO:0060492	0.016

M_30	GO:0044805	0.016
M_30	GO:0031081	0.016
M_30	GO:0010909	0.016
M_30	GO:0030997	0.016
M_30	GO:0043686	0.016
M_30	GO:0036023	0.016
M_30	GO:0060769	0.016
M_30	GO:0034473	0.016
M_30	GO:0034476	0.016
M_30	GO:0000379	0.016
M_30	GO:0045007	0.016
M_30	GO:0061198	0.016
M_30	GO:0006654	0.0177
M_30	GO:0018279	0.0186
M_30	GO:0016321	0.0213
M_30	GO:1990180	0.0213
M_30	GO:0007060	0.0213
M_30	GO:0018171	0.0213
M_30	GO:0051790	0.0213
M_30	GO:0070602	0.0213
M_30	GO:0045950	0.0213
M_30	GO:0022009	0.0213
M_30	GO:1904016	0.0213
M_30	GO:1903553	0.0213
M_30	GO:0071315	0.0213
M_30	GO:0044336	0.0213
M_30	GO:0061428	0.0213
M_30	GO:0048538	0.0233
M_30	GO:2001034	0.0265
M_30	GO:2000467	0.0265
M_30	GO:0051884	0.0265
M_30	GO:0051036	0.0265

M_30	GO:0048298	0.0265
M_30	GO:0061550	0.0265
M_30	GO:0060916	0.0265
M_30	GO:0071681	0.0265
M_30	GO:0075044	0.0265
M_30	GO:0090611	0.0265
M_30	GO:1902617	0.0265
M_30	GO:0010891	0.0265
M_30	GO:2000373	0.0318
M_30	GO:0000915	0.0318
M_30	GO:0071763	0.0318
M_30	GO:0071042	0.0318
M_30	GO:0060440	0.0318
M_30	GO:0032020	0.0318
M_30	GO:0045743	0.0318
M_30	GO:0060789	0.0318
M_30	GO:0043401	0.0364
M_30	GO:0035773	0.0367
M_30	GO:0032635	0.0368
M_30	GO:0000467	0.037
M_30	GO:0051792	0.037
M_30	GO:0031119	0.037
M_30	GO:0090267	0.037
M_30	GO:0060373	0.037
M_30	GO:0071038	0.037
M_30	GO:0032049	0.037
M_30	GO:0030223	0.037
M_30	GO:2000601	0.037
M_30	GO:0071455	0.037
M_30	GO:0006768	0.037
M_30	GO:0006687	0.04
M_30	GO:0006661	0.0403

M_30	GO:0006308	0.0413
M_30	GO:0044774	0.0415
M_30	GO:0035315	0.0418
M_30	GO:0001561	0.0421
M_30	GO:0017183	0.0421
M_30	GO:0090160	0.0421
M_30	GO:0006501	0.0421
M_30	GO:0072033	0.0421
M_30	GO:0007341	0.0421
M_30	GO:0060484	0.0421
M_30	GO:0071035	0.0421
M_30	GO:0097264	0.0421
M_30	GO:0034475	0.0421
M_30	GO:0071493	0.0421
M_30	GO:0045008	0.0421
M_30	GO:0010898	0.0421
M_30	GO:0000278	0.0434
M_30	GO:0005975	0.0468
M_30	GO:0061179	0.0473
M_30	GO:0048304	0.0473
M_30	GO:1904263	0.0473
M_30	GO:0061549	0.0473
M_30	GO:0003266	0.0473
M_30	GO:0071550	0.0473
M_30	GO:0090073	0.0473
M_30	GO:0090043	0.0473
M_30	GO:0097400	0.0473
M_30	GO:0001766	0.0473
M_30	GO:0034498	0.0473
M_30	GO:0048617	0.0473
M_30	GO:0022417	0.0473
M_31	GO:0043312	5.4E-16

M_31	GO:0006954	1E-14
M_31	GO:0050776	1.2E-13
M_31	GO:0007229	5.3E-09
M_31	GO:0045087	0.000000009
M_31	GO:0002755	0.00000016
M_31	GO:0006911	0.00000036
M_31	GO:0006968	0.00000038
M_31	GO:0042102	0.0000028
M_31	GO:0034142	0.0000035
M_31	GO:0006955	0.0000041
M_31	GO:0002250	0.0000049
M_31	GO:0030889	0.0000066
M_31	GO:0043547	0.0000087
M_31	GO:0038096	0.00001
M_31	GO:0071800	0.000027
M_31	GO:0045730	0.000029
M_31	GO:0007155	0.000029
M_31	GO:0045416	0.000041
M_31	GO:0001805	0.000063
M_31	GO:0034125	0.000063
M_31	GO:0001812	0.000063
M_31	GO:0031663	0.000084
M_31	GO:0002430	0.00013
M_31	GO:0002291	0.00018
M_31	GO:2000363	0.00019
M_31	GO:2001183	0.00019
M_31	GO:2001189	0.00019
M_31	GO:0002774	0.00019
M_31	GO:0050900	0.00024
M_31	GO:0001774	0.00025
M_31	GO:0006898	0.00027
M_31	GO:2000249	0.00031

M_31	GO:0032729	0.00035
M_31	GO:0001915	0.00037
M_31	GO:0045356	0.00038
M_31	GO:0002322	0.00038
M_31	GO:0045963	0.00038
M_31	GO:1902564	0.00038
M_31	GO:0043315	0.00038
M_31	GO:0032497	0.00038
M_31	GO:0008360	0.00038
M_31	GO:0030225	0.00043
M_31	GO:0097242	0.00044
M_31	GO:0032753	0.0005
M_31	GO:0071222	0.00052
M_31	GO:0034162	0.0006
M_31	GO:0010572	0.00062
M_31	GO:0071224	0.00062
M_31	GO:0034113	0.00068
M_31	GO:0030316	0.00072
M_31	GO:0002407	0.00079
M_31	GO:0035666	0.00089
M_31	GO:0000147	0.00093
M_31	GO:0045359	0.00093
M_31	GO:0051666	0.00093
M_31	GO:2001199	0.00093
M_31	GO:0050859	0.00093
M_31	GO:0043305	0.00093
M_31	GO:0032755	0.00095
M_31	GO:0030098	0.00109
M_31	GO:0042130	0.00111
M_31	GO:2000406	0.00114
M_31	GO:0050718	0.00114
M_31	GO:0034154	0.0013

M_31	GO:0034128	0.0013
M_31	GO:0045630	0.0013
M_31	GO:0001782	0.00141
M_31	GO:0050852	0.00154
M_31	GO:0030593	0.00164
M_31	GO:0043372	0.00168
M_31	GO:0032715	0.0017
M_31	GO:1903980	0.00172
M_31	GO:0031666	0.00172
M_31	GO:0032733	0.00173
M_31	GO:0050853	0.00195
M_31	GO:0060326	0.002
M_31	GO:1901224	0.00203
M_31	GO:0050766	0.00261
M_31	GO:0019722	0.00265
M_31	GO:0001771	0.0027
M_31	GO:0071223	0.00274
M_31	GO:0045429	0.00318
M_31	GO:0042554	0.00324
M_31	GO:0031529	0.00329
M_31	GO:0032700	0.00333
M_31	GO:0002281	0.00333
M_31	GO:0072672	0.00333
M_31	GO:0042535	0.00333
M_31	GO:0007159	0.00335
M_31	GO:0007204	0.00347
M_31	GO:0045078	0.00397
M_31	GO:0045591	0.00397
M_31	GO:0032720	0.00418
M_31	GO:0032722	0.00419
M_31	GO:0030890	0.00426
M_31	GO:0043085	0.00438

M_31	GO:0032760	0.00461
M_31	GO:0045579	0.00467
M_31	GO:0007264	0.00489
M_31	GO:0050830	0.005
M_31	GO:0043303	0.00507
M_31	GO:0007165	0.0054
M_31	GO:0071361	0.00542
M_31	GO:0048010	0.00565
M_31	GO:0006909	0.00582
M_31	GO:0043032	0.00605
M_31	GO:0007252	0.00622
M_31	GO:0035589	0.00622
M_31	GO:0042110	0.00673
M_31	GO:0043113	0.00704
M_31	GO:0032930	0.00707
M_31	GO:0002758	0.00711
M_31	GO:0051607	0.00763
M_31	GO:0071611	0.00794
M_31	GO:0044546	0.00794
M_31	GO:0032604	0.00794
M_31	GO:0046633	0.00795
M_31	GO:0030502	0.00797
M_31	GO:0034166	0.00799
M_31	GO:0034158	0.00799
M_31	GO:2000350	0.00799
M_31	GO:0035644	0.00799
M_31	GO:0009104	0.00799
M_31	GO:0045660	0.00799
M_31	GO:0002622	0.00799
M_31	GO:0014739	0.00799
M_31	GO:0030845	0.00799
M_31	GO:1990771	0.00799

M_31	GO:0002344	0.00799
M_31	GO:0070392	0.00799
M_31	GO:0032712	0.00799
M_31	GO:0060305	0.00799
M_31	GO:0071846	0.00799
M_31	GO:0033026	0.00799
M_31	GO:0043017	0.00799
M_31	GO:1903082	0.00799
M_31	GO:0010920	0.00799
M_31	GO:0010925	0.00799
M_31	GO:2001193	0.00799
M_31	GO:0016046	0.00799
M_31	GO:0060101	0.00799
M_31	GO:0035772	0.00799
M_31	GO:0071656	0.00799
M_31	GO:0032685	0.00799
M_31	GO:1902569	0.00799
M_31	GO:0002721	0.00799
M_31	GO:0033382	0.00799
M_31	GO:0033373	0.00799
M_31	GO:0061485	0.00799
M_31	GO:0035548	0.00799
M_31	GO:0001811	0.00799
M_31	GO:0001814	0.00799
M_31	GO:0051134	0.00799
M_31	GO:0038083	0.00874
M_31	GO:0071404	0.00892
M_31	GO:0090026	0.00892
M_31	GO:0050860	0.00892
M_31	GO:0033628	0.00967
M_31	GO:0070527	0.01017
M_31	GO:0030449	0.01104

M_31	GO:0050868	0.01119
M_31	GO:0045671	0.01302
M_31	GO:0050710	0.01365
M_31	GO:0030217	0.01427
M_31	GO:0006801	0.01483
M_31	GO:0038095	0.01487
M_31	GO:0032637	0.01541
M_31	GO:0032621	0.01581
M_31	GO:0002588	0.01592
M_31	GO:0002522	0.01592
M_31	GO:0002519	0.01592
M_31	GO:0002625	0.01592
M_31	GO:0070560	0.01592
M_31	GO:2000473	0.01592
M_31	GO:2000426	0.01592
M_31	GO:0002605	0.01592
M_31	GO:2000417	0.01592
M_31	GO:0003366	0.01592
M_31	GO:0071226	0.01592
M_31	GO:0038156	0.01592
M_31	GO:1903905	0.01592
M_31	GO:0002316	0.01592
M_31	GO:0090264	0.01592
M_31	GO:1902216	0.01592
M_31	GO:0032701	0.01592
M_31	GO:0045404	0.01592
M_31	GO:0032707	0.01592
M_31	GO:0044027	0.01592
M_31	GO:0038043	0.01592
M_31	GO:1902567	0.01592
M_31	GO:0045728	0.01592
M_31	GO:0001798	0.01592

M_31	GO:2000521	0.01592
M_31	GO:2001202	0.01592
M_31	GO:0002859	0.01592
M_31	GO:0043318	0.01592
M_31	GO:0071461	0.01592
M_31	GO:0032499	0.01592
M_31	GO:0002479	0.01818
M_31	GO:1904646	0.01818
M_31	GO:0032481	0.01882
M_31	GO:0032689	0.01928
M_31	GO:0007267	0.01941
M_31	GO:0002283	0.01942
M_31	GO:0070098	0.02033
M_31	GO:0032088	0.02144
M_31	GO:0071219	0.02166
M_31	GO:0038093	0.02198
M_31	GO:0014065	0.02199
M_31	GO:0050690	0.02236
M_31	GO:0002820	0.02316
M_31	GO:0036336	0.02331
M_31	GO:0032732	0.02333
M_31	GO:0046488	0.02338
M_31	GO:0009595	0.02347
M_31	GO:0042590	0.02348
M_31	GO:0002220	0.02353
M_31	GO:0002374	0.02363
M_31	GO:0045588	0.02378
M_31	GO:0002652	0.02378
M_31	GO:0030886	0.02378
M_31	GO:1903974	0.02378
M_31	GO:0038123	0.02378
M_31	GO:0002378	0.02378

M_31	GO:0071727	0.02378
M_31	GO:0032765	0.02378
M_31	GO:0070433	0.02378
M_31	GO:0002266	0.02378
M_31	GO:0002277	0.02378
M_31	GO:2001180	0.02378
M_31	GO:0071673	0.02378
M_31	GO:0006933	0.02378
M_31	GO:1902523	0.02378
M_31	GO:0002554	0.02378
M_31	GO:0090314	0.02383
M_31	GO:1901216	0.02415
M_31	GO:0071260	0.02459
M_31	GO:0090023	0.02534
M_31	GO:0070266	0.02534
M_31	GO:0051092	0.02542
M_31	GO:0007200	0.02544
M_31	GO:0050829	0.02886
M_31	GO:0030866	0.02934
M_31	GO:0071346	0.02941
M_31	GO:0002819	0.0296
M_31	GO:0031623	0.02976
M_31	GO:0045576	0.02989
M_31	GO:0050672	0.02992
M_31	GO:0038094	0.03002
M_31	GO:0051056	0.03066
M_31	GO:2001198	0.03115
M_31	GO:0033004	0.03115
M_31	GO:0046629	0.03116
M_31	GO:0035696	0.03159
M_31	GO:0014063	0.03159
M_31	GO:0038124	0.03159

M_31	GO:0002309	0.03159
M_31	GO:0071726	0.03159
M_31	GO:1903223	0.03159
M_31	GO:1903238	0.03159
M_31	GO:0050904	0.03159
M_31	GO:0071672	0.03159
M_31	GO:0071315	0.03159
M_31	GO:0045077	0.03159
M_31	GO:0043378	0.03159
M_31	GO:0010897	0.03159
M_31	GO:0051142	0.03159
M_31	GO:0060333	0.03546
M_31	GO:0043299	0.03651
M_31	GO:0050707	0.0369
M_31	GO:0071276	0.03696
M_31	GO:0051482	0.03696
M_31	GO:0002431	0.03739
M_31	GO:0070374	0.03749
M_31	GO:0007599	0.03752
M_31	GO:0045123	0.0386
M_31	GO:0002578	0.03906
M_31	GO:0055118	0.03933
M_31	GO:0071225	0.03933
M_31	GO:0038145	0.03933
M_31	GO:0045347	0.03933
M_31	GO:1901164	0.03933
M_31	GO:0090241	0.03933
M_31	GO:1902262	0.03933
M_31	GO:1903753	0.03933
M_31	GO:1904044	0.03933
M_31	GO:0090074	0.03933
M_31	GO:0097411	0.03933

M_31	GO:2001186	0.03933
M_31	GO:0033625	0.03933
M_31	GO:0036016	0.03933
M_31	GO:0034316	0.03933
M_31	GO:1900454	0.03933
M_31	GO:2001205	0.03933
M_31	GO:0045084	0.03933
M_31	GO:0035509	0.03933
M_31	GO:0001932	0.03939
M_31	GO:0051707	0.04021
M_31	GO:0009611	0.04234
M_31	GO:0051602	0.04434
M_31	GO:0071621	0.04531
M_31	GO:0061515	0.04585
M_31	GO:0032623	0.04616
M_31	GO:0002292	0.0465
M_31	GO:0001768	0.04669
M_31	GO:1900227	0.04701
M_31	GO:0008626	0.04701
M_31	GO:2000110	0.04701
M_31	GO:2000270	0.04701
M_31	GO:0070358	0.04701
M_31	GO:0002924	0.04701
M_31	GO:0042536	0.04701
M_31	GO:0032687	0.04701
M_31	GO:2000553	0.04701
M_31	GO:0002740	0.04701
M_31	GO:1900425	0.04701
M_31	GO:0060729	0.04701
M_31	GO:0009408	0.04807
M_31	GO:0030838	0.04858
M_31	GO:0045860	0.04936

M_32	GO:0000389	0.00048
M_32	GO:0006281	0.00052
M_32	GO:0016575	0.00056
M_32	GO:0002281	0.00172
M_32	GO:0000122	0.00267
M_32	GO:0000381	0.00511
M_32	GO:0036399	0.00571
M_32	GO:0031940	0.00571
M_32	GO:0016182	0.00571
M_32	GO:0003247	0.00571
M_32	GO:0043519	0.00571
M_32	GO:0071629	0.00571
M_32	GO:0036071	0.00571
M_32	GO:0050754	0.00571
M_32	GO:0071348	0.00571
M_32	GO:0048026	0.00692
M_32	GO:1902036	0.00888
M_32	GO:0008612	0.01138
M_32	GO:0010609	0.01138
M_32	GO:0006601	0.01138
M_32	GO:1990619	0.01138
M_32	GO:2000845	0.01138
M_32	GO:0071642	0.01138
M_32	GO:0050725	0.01138
M_32	GO:1903646	0.01138
M_32	GO:0045872	0.01138
M_32	GO:0006260	0.01213
M_32	GO:0042147	0.01271
M_32	GO:0032200	0.017
M_32	GO:0034729	0.01703
M_32	GO:1902174	0.01703
M_32	GO:0006391	0.01703

M_32	GO:1990502	0.01703
M_32	GO:1902732	0.01703
M_32	GO:1903070	0.01703
M_32	GO:0006267	0.01703
M_32	GO:0060761	0.02226
M_32	GO:0006726	0.02264
M_32	GO:0033152	0.02264
M_32	GO:2000491	0.02264
M_32	GO:0048499	0.02264
M_32	GO:0046368	0.02264
M_32	GO:0090170	0.02264
M_32	GO:0002326	0.02264
M_32	GO:0090261	0.02264
M_32	GO:0032510	0.02264
M_32	GO:1902817	0.02264
M_32	GO:0051648	0.02782
M_32	GO:0033132	0.02822
M_32	GO:0016191	0.02822
M_32	GO:0035926	0.02822
M_32	GO:0033578	0.02822
M_32	GO:0072675	0.02822
M_32	GO:0010216	0.02822
M_32	GO:0043525	0.03188
M_32	GO:1904294	0.03345
M_32	GO:2000373	0.03377
M_32	GO:1900244	0.03377
M_32	GO:0045634	0.03377
M_32	GO:0045359	0.03377
M_32	GO:0046498	0.03377
M_32	GO:0045348	0.03377
M_32	GO:0060136	0.03377
M_32	GO:0070944	0.03377

M_32	GO:0071651	0.03377
M_32	GO:0030174	0.03377
M_32	GO:0060770	0.03377
M_32	GO:0090090	0.03477
M_32	GO:0070936	0.03556
M_32	GO:0032270	0.0374
M_32	GO:0045080	0.0389
M_32	GO:0006348	0.0389
M_32	GO:0034128	0.03929
M_32	GO:2000343	0.03929
M_32	GO:2000210	0.03929
M_32	GO:1902231	0.03929
M_32	GO:1903071	0.03929
M_32	GO:0046777	0.04153
M_32	GO:0006508	0.04269
M_32	GO:0036503	0.04411
M_32	GO:0046600	0.04477
M_32	GO:0042117	0.04477
M_32	GO:0036289	0.04477
M_32	GO:1903800	0.04477
M_32	GO:0071493	0.04477
M_32	GO:0043276	0.0498
M_32	GO:0034333	0.04994
M_33	GO:0071630	0.00015
M_33	GO:0032402	0.00054
M_33	GO:0019323	0.00072
M_33	GO:0009052	0.00072
M_33	GO:0032981	0.00106
M_33	GO:0006303	0.00106
M_33	GO:0085020	0.00133
M_33	GO:0006986	0.00172
M_33	GO:0000338	0.00212

M_33	GO:0008637	0.00355
M_33	GO:0034497	0.00363
M_33	GO:0006120	0.00437
M_33	GO:0000082	0.00492
M_33	GO:0010592	0.00695
M_33	GO:0030041	0.00697
M_33	GO:0016441	0.007
M_33	GO:1904456	0.00702
M_33	GO:1990074	0.00702
M_33	GO:0072671	0.00702
M_33	GO:1902045	0.00702
M_33	GO:2001247	0.00702
M_33	GO:0002842	0.00702
M_33	GO:0061445	0.00702
M_33	GO:0070932	0.00855
M_33	GO:0043687	0.01089
M_33	GO:0000715	0.01123
M_33	GO:0042026	0.0132
M_33	GO:0051306	0.01392
M_33	GO:0019516	0.01399
M_33	GO:2000327	0.01399
M_33	GO:0032979	0.01399
M_33	GO:0002368	0.01399
M_33	GO:2000158	0.01399
M_33	GO:1904211	0.01399
M_33	GO:0002415	0.01399
M_33	GO:0036292	0.01399
M_33	GO:0019243	0.01399
M_33	GO:2000017	0.01399
M_33	GO:2000663	0.01399
M_33	GO:0006227	0.01399
M_33	GO:0006233	0.01399

M_33	GO:0045041	0.01399
M_33	GO:0035526	0.01399
M_33	GO:0051946	0.01399
M_33	GO:0001831	0.01399
M_33	GO:0051290	0.01641
M_33	GO:0061668	0.02075
M_33	GO:1901315	0.02092
M_33	GO:0042998	0.02092
M_33	GO:0039689	0.02092
M_33	GO:1900063	0.02092
M_33	GO:0010387	0.02092
M_33	GO:0097638	0.02092
M_33	GO:0090204	0.02092
M_33	GO:0044805	0.02092
M_33	GO:1902775	0.02092
M_33	GO:0003274	0.02092
M_33	GO:0019249	0.02092
M_33	GO:2001180	0.02092
M_33	GO:0007228	0.02092
M_33	GO:2000504	0.02092
M_33	GO:2000666	0.02092
M_33	GO:0097252	0.02092
M_33	GO:0000045	0.02406
M_33	GO:1902042	0.02637
M_33	GO:0090168	0.02779
M_33	GO:0030578	0.02779
M_33	GO:0046452	0.02779
M_33	GO:0048254	0.02779
M_33	GO:0061589	0.02779
M_33	GO:0051697	0.02779
M_33	GO:1901896	0.02779
M_33	GO:0097167	0.02779

M_33	GO:1903644	0.02779
M_33	GO:2000676	0.02779
M_33	GO:0006235	0.02779
M_33	GO:0071475	0.02779
M_33	GO:0014706	0.0279
M_33	GO:0017148	0.02803
M_33	GO:0045759	0.03434
M_33	GO:1904430	0.03462
M_33	GO:0046940	0.03462
M_33	GO:0061590	0.03462
M_33	GO:0061591	0.03462
M_33	GO:0070345	0.03462
M_33	GO:0003289	0.03462
M_33	GO:0006432	0.03462
M_33	GO:0001757	0.03462
M_33	GO:0044314	0.03462
M_33	GO:0048669	0.03462
M_33	GO:0032481	0.03463
M_33	GO:0048515	0.03463
M_33	GO:0046939	0.04127
M_33	GO:0010587	0.0414
M_33	GO:0036481	0.0414
M_33	GO:0070358	0.0414
M_33	GO:0016576	0.0414
M_33	GO:0006189	0.0414
M_33	GO:0045719	0.0414
M_33	GO:0046826	0.0414
M_33	GO:0031642	0.0414
M_33	GO:1901621	0.0414
M_33	GO:1903352	0.0414
M_33	GO:0034983	0.04813
M_33	GO:0034141	0.04813

M_33	GO:0003360	0.04813
M_33	GO:0040016	0.04813
M_33	GO:1902260	0.04813
M_33	GO:0033504	0.04813
M_33	GO:1903003	0.04813
M_33	GO:0061312	0.04813
M_33	GO:0019064	0.04813
M_33	GO:0071476	0.04813
M_33	GO:0006511	0.04835
M_34	GO:0030198	3.5E-10
M_34	GO:0001525	0.000000019
M_34	GO:0045766	0.00000015
M_34	GO:0016525	0.00000039
M_34	GO:0002040	0.000002
M_34	GO:0014068	0.0000024
M_34	GO:0070374	0.000011
M_34	GO:0007165	0.000013
M_34	GO:0007160	0.000013
M_34	GO:0043537	0.000015
M_34	GO:0045602	0.000027
M_34	GO:0035912	0.000044
M_34	GO:0001955	0.000044
M_34	GO:0001666	0.000068
M_34	GO:0035759	0.000087
M_34	GO:0070543	0.000087
M_34	GO:0043552	0.00013
M_34	GO:0002576	0.0002
M_34	GO:0044130	0.00022
M_34	GO:0048014	0.00026
M_34	GO:0072011	0.00026
M_34	GO:0002548	0.00026
M_34	GO:0001569	0.00028

M_34	GO:0090051	0.00034
M_34	GO:0050901	0.00041
M_34	GO:0031623	0.00041
M_34	GO:0072012	0.00049
M_34	GO:1990410	0.00052
M_34	GO:2000491	0.00052
M_34	GO:0045906	0.00052
M_34	GO:0051897	0.00053
M_34	GO:0071560	0.00069
M_34	GO:0006954	0.00073
M_34	GO:0071711	0.00082
M_34	GO:0061299	0.00085
M_34	GO:1903142	0.00086
M_34	GO:0032720	0.00097
M_34	GO:0070098	0.00098
M_34	GO:0071346	0.00121
M_34	GO:0035634	0.00127
M_34	GO:0008217	0.00133
M_34	GO:0071222	0.00145
M_34	GO:0050873	0.00157
M_34	GO:0061298	0.00171
M_34	GO:0061614	0.00176
M_34	GO:0007157	0.00186
M_34	GO:0048247	0.00199
M_34	GO:0050728	0.00203
M_34	GO:0045026	0.00233
M_34	GO:0032966	0.00235
M_34	GO:0030593	0.00293
M_34	GO:0030195	0.00298
M_34	GO:0010739	0.003
M_34	GO:0060100	0.003
M_34	GO:0010811	0.00314

M_34	GO:0018108	0.0032
M_34	GO:0014823	0.00322
M_34	GO:0001974	0.00322
M_34	GO:0030334	0.00327
M_34	GO:0050729	0.00331
M_34	GO:0007155	0.00338
M_34	GO:0006952	0.00362
M_34	GO:0010863	0.00365
M_34	GO:0060055	0.00369
M_34	GO:0071223	0.00373
M_34	GO:2001214	0.00373
M_34	GO:0038063	0.00373
M_34	GO:0043951	0.00373
M_34	GO:0071356	0.00439
M_34	GO:0030194	0.00443
M_34	GO:0050927	0.00449
M_34	GO:0031953	0.00453
M_34	GO:0002003	0.00453
M_34	GO:0017015	0.0052
M_34	GO:2000279	0.00535
M_34	GO:0050872	0.00541
M_34	GO:0001935	0.00598
M_34	GO:0007159	0.006
M_34	GO:0051145	0.00615
M_34	GO:0071260	0.00617
M_34	GO:0035162	0.00628
M_34	GO:0010745	0.00635
M_34	GO:0008284	0.0065
M_34	GO:0071347	0.00711
M_34	GO:0006112	0.00733
M_34	GO:0042953	0.00736
M_34	GO:0016477	0.00779

M_34	GO:0030595	0.00793
M_34	GO:0030949	0.00844
M_34	GO:0050866	0.00923
M_34	GO:0045713	0.00931
M_34	GO:0071298	0.00936
M_34	GO:0070994	0.00936
M_34	GO:0070946	0.00936
M_34	GO:1904325	0.00936
M_34	GO:2000486	0.00936
M_34	GO:2000478	0.00936
M_34	GO:1900035	0.00936
M_34	GO:0060981	0.00936
M_34	GO:0038193	0.00936
M_34	GO:0003185	0.00936
M_34	GO:0003138	0.00936
M_34	GO:2000230	0.00936
M_34	GO:1901585	0.00936
M_34	GO:0036323	0.00936
M_34	GO:0085018	0.00936
M_34	GO:2000590	0.00936
M_34	GO:0071329	0.00936
M_34	GO:2000529	0.00936
M_34	GO:1904651	0.00936
M_34	GO:2000548	0.00936
M_34	GO:2000534	0.00936
M_34	GO:1902204	0.00936
M_34	GO:1902748	0.00936
M_34	GO:0050928	0.00936
M_34	GO:0060217	0.00936
M_34	GO:0046546	0.00938
M_34	GO:0035313	0.00959
M_34	GO:0032516	0.00959

M_34	GO:0003222	0.00959
M_34	GO:0043547	0.00987
M_34	GO:0010862	0.01024
M_34	GO:0016339	0.01024
M_34	GO:0032355	0.01039
M_34	GO:2000353	0.0108
M_34	GO:0019934	0.0108
M_34	GO:0071380	0.0108
M_34	GO:0000122	0.01116
M_34	GO:0006198	0.01208
M_34	GO:0043627	0.0126
M_34	GO:0071320	0.01273
M_34	GO:0048661	0.0134
M_34	GO:0032332	0.01342
M_34	GO:0030220	0.01342
M_34	GO:0032760	0.01459
M_34	GO:0048745	0.01482
M_34	GO:0035584	0.01482
M_34	GO:0060216	0.01482
M_34	GO:0001816	0.01574
M_34	GO:0090050	0.01628
M_34	GO:0070555	0.01702
M_34	GO:0001954	0.01743
M_34	GO:0050900	0.01744
M_34	GO:0045765	0.01779
M_34	GO:0003094	0.0178
M_34	GO:0035690	0.01786
M_34	GO:0001936	0.01818
M_34	GO:0030029	0.01826
M_34	GO:0022617	0.01842
M_34	GO:0032615	0.01858
M_34	GO:1904022	0.01864

M_34	GO:1904027	0.01864
M_34	GO:0015920	0.01864
M_34	GO:0071288	0.01864
M_34	GO:0071231	0.01864
M_34	GO:0035026	0.01864
M_34	GO:0060587	0.01864
M_34	GO:0038041	0.01864
M_34	GO:0030382	0.01864
M_34	GO:0061373	0.01864
M_34	GO:0060694	0.01864
M_34	GO:0006668	0.01864
M_34	GO:0045799	0.01864
M_34	GO:1901475	0.01864
M_34	GO:0002232	0.01864
M_34	GO:2000334	0.01864
M_34	GO:1990408	0.01864
M_34	GO:0035377	0.01864
M_34	GO:0035378	0.01864
M_34	GO:2000490	0.01864
M_34	GO:0072254	0.01864
M_34	GO:0050758	0.01864
M_34	GO:0030221	0.01864
M_34	GO:0007521	0.01864
M_34	GO:0097323	0.01864
M_34	GO:0097324	0.01864
M_34	GO:0033159	0.01864
M_34	GO:0072554	0.01864
M_34	GO:1903056	0.01864
M_34	GO:0060375	0.01864
M_34	GO:0003245	0.01864
M_34	GO:0051466	0.01864
M_34	GO:1901558	0.01864

M_34	GO:0001300	0.01864
M_34	GO:1902283	0.01864
M_34	GO:1900168	0.01864
M_34	GO:0060183	0.01864
M_34	GO:2000584	0.01864
M_34	GO:0071306	0.01864
M_34	GO:1900135	0.01864
M_34	GO:1904613	0.01864
M_34	GO:0060265	0.01864
M_34	GO:1990051	0.01864
M_34	GO:0007186	0.02011
M_34	GO:0045777	0.02079
M_34	GO:0051894	0.021
M_34	GO:0050731	0.02152
M_34	GO:0032869	0.02189
M_34	GO:0030217	0.02241
M_34	GO:1902036	0.02269
M_34	GO:0032967	0.02269
M_34	GO:0098609	0.02349
M_34	GO:0019216	0.02388
M_34	GO:0001958	0.02443
M_34	GO:0030511	0.02443
M_34	GO:0007179	0.02577
M_34	GO:0030335	0.02582
M_34	GO:0035025	0.02623
M_34	GO:0045648	0.02623
M_34	GO:0001938	0.02652
M_34	GO:0010942	0.02672
M_34	GO:0071300	0.02691
M_34	GO:0006898	0.02742
M_34	GO:0045923	0.02753
M_34	GO:0030155	0.02755

M_34	GO:0010871	0.02766
M_34	GO:0072102	0.02766
M_34	GO:0032878	0.02769
M_34	GO:0038091	0.02782
M_34	GO:0046968	0.02782
M_34	GO:1904381	0.02782
M_34	GO:0045715	0.02782
M_34	GO:0042631	0.02782
M_34	GO:0000103	0.02782
M_34	GO:0030185	0.02782
M_34	GO:0071657	0.02782
M_34	GO:0072262	0.02782
M_34	GO:0003383	0.02782
M_34	GO:0072277	0.02782
M_34	GO:0060025	0.02782
M_34	GO:0035441	0.02782
M_34	GO:0033326	0.02782
M_34	GO:2000111	0.02782
M_34	GO:2000297	0.02782
M_34	GO:2000721	0.02782
M_34	GO:0035793	0.02782
M_34	GO:0046878	0.02782
M_34	GO:2000504	0.02782
M_34	GO:0055096	0.02782
M_34	GO:0090210	0.02782
M_34	GO:0007202	0.02808
M_34	GO:2000352	0.02808
M_34	GO:0007507	0.02938
M_34	GO:0010629	0.0297
M_34	GO:0008015	0.03017
M_34	GO:0009968	0.03043
M_34	GO:0032733	0.03192

M_34	GO:0060021	0.0322
M_34	GO:0010469	0.03222
M_34	GO:0007389	0.03274
M_34	GO:0090023	0.03392
M_34	GO:0042308	0.03557
M_34	GO:0060976	0.03562
M_34	GO:0043549	0.03581
M_34	GO:0030513	0.03596
M_34	GO:0045907	0.03596
M_34	GO:0030947	0.03631
M_34	GO:0001945	0.03674
M_34	GO:0033686	0.03693
M_34	GO:0071726	0.03693
M_34	GO:0032497	0.03693
M_34	GO:1903237	0.03693
M_34	GO:0035984	0.03693
M_34	GO:0002125	0.03693
M_34	GO:0022009	0.03693
M_34	GO:1904447	0.03693
M_34	GO:0072278	0.03693
M_34	GO:1903588	0.03693
M_34	GO:0060018	0.03693
M_34	GO:1900041	0.03693
M_34	GO:0007598	0.03693
M_34	GO:0003199	0.03693
M_34	GO:0045629	0.03693
M_34	GO:0032849	0.03693
M_34	GO:0045636	0.03693
M_34	GO:0038124	0.03693
M_34	GO:0007621	0.03693
M_34	GO:0038108	0.03693
M_34	GO:2001013	0.03693

M_34	GO:0003273	0.03693
M_34	GO:0060355	0.03693
M_34	GO:0030578	0.03693
M_34	GO:0031394	0.03693
M_34	GO:0048023	0.03693
M_34	GO:0050904	0.03693
M_34	GO:0070093	0.03693
M_34	GO:0043312	0.03782
M_34	GO:0030224	0.03805
M_34	GO:0034383	0.03805
M_34	GO:0098655	0.03905
M_34	GO:0050829	0.04297
M_34	GO:0010595	0.04338
M_34	GO:0032526	0.04343
M_34	GO:0045746	0.04458
M_34	GO:0002755	0.04458
M_34	GO:0045123	0.04469
M_34	GO:0006909	0.04488
M_34	GO:0061384	0.04519
M_34	GO:0072378	0.04545
M_34	GO:0033034	0.04568
M_34	GO:0061037	0.04572
M_34	GO:0042092	0.04575
M_34	GO:0046545	0.04591
M_34	GO:1900747	0.04594
M_34	GO:0001757	0.04594
M_34	GO:2000987	0.04594
M_34	GO:0038033	0.04594
M_34	GO:0061304	0.04594
M_34	GO:2000370	0.04594
M_34	GO:0072144	0.04594
M_34	GO:2000467	0.04594

M_34	GO:2000481	0.04594
M_34	GO:0010891	0.04594
M_34	GO:0051970	0.04594
M_34	GO:0001992	0.04594
M_34	GO:0010572	0.04594
M_34	GO:0051461	0.04594
M_34	GO:0021540	0.04594
M_34	GO:0060437	0.04594
M_34	GO:0006114	0.04594
M_34	GO:0034197	0.04594
M_34	GO:0018057	0.04594
M_34	GO:0007189	0.04628
M_34	GO:0046777	0.04761
M_34	GO:0006816	0.04823
M_34	GO:0060612	0.04915
M_34	GO:0051482	0.04915
M_34	GO:0007369	0.04945
M_35	GO:0019373	0.00000011
M_35	GO:0042738	0.00000019
M_35	GO:0009804	0.000065
M_35	GO:0014050	0.000096
M_35	GO:0030916	0.00013
M_35	GO:0048853	0.00041
M_35	GO:0006805	0.00144
M_35	GO:0090096	0.00217
M_35	GO:0018979	0.00217
M_35	GO:0060070	0.00429
M_35	GO:0010607	0.00433
M_35	GO:0044324	0.00433
M_35	GO:0044335	0.00433
M_35	GO:0018931	0.00433
M_35	GO:0060128	0.00433

M_35	GO:0090134	0.00433
M_35	GO:0046226	0.00433
M_35	GO:0033563	0.00649
M_35	GO:0014054	0.00649
M_35	GO:0060129	0.00649
M_35	GO:0034628	0.00649
M_35	GO:0001658	0.00749
M_35	GO:0061317	0.00865
M_35	GO:0035965	0.00865
M_35	GO:0014063	0.00865
M_35	GO:0001692	0.00865
M_35	GO:0014034	0.00865
M_35	GO:0051793	0.0108
M_35	GO:0060157	0.0108
M_35	GO:0061624	0.0108
M_35	GO:0008078	0.0108
M_35	GO:0015840	0.01295
M_35	GO:0042985	0.01295
M_35	GO:0001839	0.01509
M_35	GO:0007197	0.01509
M_35	GO:0050957	0.01509
M_35	GO:0021798	0.01509
M_35	GO:0051792	0.01509
M_35	GO:0009744	0.01509
M_35	GO:0045656	0.01509
M_35	GO:2000096	0.01722
M_35	GO:0009750	0.01722
M_35	GO:2000252	0.01722
M_35	GO:0030917	0.01936
M_35	GO:0000290	0.02149
M_35	GO:0003198	0.02361
M_35	GO:0042487	0.02995

M_35	GO:0015671	0.02995
M_35	GO:0043584	0.03206
M_35	GO:0070371	0.03592
M_35	GO:0044346	0.03626
M_35	GO:0014061	0.03626
M_35	GO:0010592	0.03835
M_35	GO:0006833	0.04044
M_35	GO:0001759	0.04044
M_35	GO:0023019	0.04252
M_35	GO:0070848	0.04301
M_35	GO:0032986	0.0446
M_35	GO:1904659	0.0446
M_35	GO:0060445	0.0446
M_35	GO:0051412	0.0446
M_35	GO:0008045	0.04875
M_35	GO:0021544	0.04875
M_35	GO:0030539	0.04875
M_36	GO:0006489	0.00037
M_36	GO:0071763	0.00055
M_36	GO:0042791	0.00055
M_36	GO:0042797	0.00055
M_36	GO:0051085	0.00065
M_36	GO:0009142	0.00074
M_36	GO:0006348	0.00129
M_36	GO:0071712	0.00276
M_36	GO:0051568	0.00352
M_36	GO:0035269	0.00368
M_36	GO:0032515	0.00438
M_36	GO:0036475	0.00606
M_36	GO:0002333	0.00611
M_36	GO:0035971	0.00611
M_36	GO:0046901	0.00611

M_36	GO:0018012	0.00611
M_36	GO:0018013	0.00611
M_36	GO:0018016	0.00611
M_36	GO:0043048	0.00611
M_36	GO:1903839	0.00611
M_36	GO:1904531	0.00611
M_36	GO:2000806	0.00611
M_36	GO:0035572	0.00611
M_36	GO:0035573	0.00611
M_36	GO:0097503	0.00719
M_36	GO:0051894	0.00934
M_36	GO:0001579	0.01218
M_36	GO:1900275	0.01218
M_36	GO:0090135	0.01218
M_36	GO:1990051	0.01218
M_36	GO:0044029	0.01218
M_36	GO:0071049	0.01218
M_36	GO:0036245	0.01218
M_36	GO:0036511	0.01218
M_36	GO:0036512	0.01218
M_36	GO:0036509	0.01218
M_36	GO:0031175	0.01218
M_36	GO:0015949	0.01348
M_36	GO:0070126	0.01773
M_36	GO:0060830	0.01821
M_36	GO:0006858	0.01821
M_36	GO:1904100	0.01821
M_36	GO:0044211	0.01821
M_36	GO:2000984	0.01821
M_36	GO:1900042	0.01821
M_36	GO:1903721	0.01821
M_36	GO:0001922	0.01821

M_36	GO:0006407	0.01821
M_36	GO:1902512	0.01821
M_36	GO:0036510	0.01821
M_36	GO:0045746	0.02034
M_36	GO:0018279	0.02364
M_36	GO:0070086	0.02421
M_36	GO:1901300	0.02421
M_36	GO:0030035	0.02421
M_36	GO:0038189	0.02421
M_36	GO:0002322	0.02421
M_36	GO:0071034	0.02421
M_36	GO:2001168	0.02421
M_36	GO:0090521	0.02421
M_36	GO:1904382	0.02421
M_36	GO:0050790	0.02615
M_36	GO:0031113	0.02836
M_36	GO:0034227	0.03017
M_36	GO:0044206	0.03017
M_36	GO:0002329	0.03017
M_36	GO:0019348	0.03017
M_36	GO:0071494	0.03017
M_36	GO:0051496	0.03343
M_36	GO:0048524	0.03592
M_36	GO:0007029	0.03608
M_36	GO:1904417	0.0361
M_36	GO:1900244	0.0361
M_36	GO:0042796	0.0361
M_36	GO:0051661	0.0361
M_36	GO:0060992	0.0361
M_36	GO:0070973	0.0361
M_36	GO:1904528	0.0361
M_36	GO:0043488	0.03721

M_36	GO:0006664	0.04145
M_36	GO:0032489	0.04199
M_36	GO:0000467	0.04199
M_36	GO:0035948	0.04199
M_36	GO:0042760	0.04199
M_36	GO:0071051	0.04199
M_36	GO:0071038	0.04199
M_36	GO:1902715	0.04199
M_36	GO:0090650	0.04199
M_36	GO:0070301	0.04679
M_36	GO:0060020	0.04784
M_36	GO:0033129	0.04784
M_36	GO:0017183	0.04784
M_36	GO:1903265	0.04784
M_36	GO:0032790	0.04784
M_36	GO:0006627	0.04784
M_36	GO:0051584	0.04784
M_36	GO:0071035	0.04784
M_36	GO:0048743	0.04784
M_36	GO:2000096	0.04784
M_36	GO:0006449	0.04784
M_36	GO:1900364	0.04784
M_36	GO:0006975	0.04784
M_36	GO:0034351	0.04784
M_36	GO:0072321	0.04784
M_36	GO:0034475	0.04784
M_36	GO:0051865	0.04896
M_37	GO:0048025	0.0017
M_37	GO:1903506	0.003
M_37	GO:0061433	0.0031
M_37	GO:1904050	0.0031
M_37	GO:0070446	0.0031

M_37	GO:0060803	0.0031
M_37	GO:1902396	0.0062
M_37	GO:1990258	0.0062
M_37	GO:1900195	0.0062
M_37	GO:0000494	0.0062
M_37	GO:0006434	0.0062
M_37	GO:2000777	0.0062
M_37	GO:0051935	0.0092
M_37	GO:0060025	0.0092
M_37	GO:1903422	0.0092
M_37	GO:0007181	0.0092
M_37	GO:0060672	0.0092
M_37	GO:0001541	0.0116
M_37	GO:1904628	0.0123
M_37	GO:0061428	0.0123
M_37	GO:0097056	0.0123
M_37	GO:0034553	0.0123
M_37	GO:0048254	0.0123
M_37	GO:0032417	0.0123
M_37	GO:0048549	0.0123
M_37	GO:0009994	0.0151
M_37	GO:0001824	0.0152
M_37	GO:0051987	0.0153
M_37	GO:2000382	0.0153
M_37	GO:1900226	0.0153
M_37	GO:0070245	0.0153
M_37	GO:1900425	0.0184
M_37	GO:0010807	0.0184
M_37	GO:0034983	0.0214
M_37	GO:0060075	0.0214
M_37	GO:1902416	0.0214
M_37	GO:0035333	0.0214

M_37	GO:0071219	0.0238
M_37	GO:0033085	0.0244
M_37	GO:2000321	0.0244
M_37	GO:0051026	0.0244
M_37	GO:0071313	0.0244
M_37	GO:1900364	0.0244
M_37	GO:0030050	0.0244
M_37	GO:0043687	0.0254
M_37	GO:0032786	0.0269
M_37	GO:0070933	0.0274
M_37	GO:0016584	0.0274
M_37	GO:0006348	0.0274
M_37	GO:0048096	0.0274
M_37	GO:0016081	0.0274
M_37	GO:0060996	0.03
M_37	GO:0030154	0.0328
M_37	GO:0045899	0.0334
M_37	GO:0090042	0.0334
M_37	GO:0051056	0.0356
M_37	GO:0071773	0.036
M_37	GO:1900119	0.0364
M_37	GO:0033523	0.0364
M_37	GO:0051775	0.0364
M_37	GO:0007183	0.0394
M_37	GO:0045836	0.0394
M_37	GO:0032785	0.0394
M_37	GO:1900025	0.0394
M_37	GO:0071872	0.0394
M_37	GO:0010801	0.0394
M_37	GO:0043248	0.0423
M_37	GO:0080182	0.0453
M_37	GO:0035729	0.0453

M_37	GO:0000183	0.0453
M_37	GO:0007292	0.0467
M_37	GO:1901800	0.0476
M_37	GO:0031629	0.0482
M_37	GO:0001711	0.0482
M_37	GO:0048012	0.0482
M_37	GO:0045603	0.0482
M_38	GO:0003341	8.7E-23
M_38	GO:0036159	2.6E-14
M_38	GO:0060285	2.6E-09
M_38	GO:0044458	0.000000009
M_38	GO:0060271	0.000000073
M_38	GO:0030317	0.00000011
M_38	GO:0003356	0.00000031
M_38	GO:0070286	0.00000036
M_38	GO:0036158	0.00000054
M_38	GO:0060287	0.0000037
M_38	GO:0035082	0.0000058
M_38	GO:0007368	0.000015
M_38	GO:0035469	0.0001
M_38	GO:0071910	0.00015
M_38	GO:0071907	0.00021
M_38	GO:0003352	0.00032
M_38	GO:0044782	0.00059
M_38	GO:2000574	0.00065
M_38	GO:0003351	0.00069
M_38	GO:0001947	0.00089
M_38	GO:0060294	0.00166
M_38	GO:0060972	0.00203
M_38	GO:0001539	0.0028
M_38	GO:0021591	0.00287
M_38	GO:1901248	0.0032

M_38	GO:0031275	0.0032
M_38	GO:0002897	0.0032
M_38	GO:0072016	0.0032
M_38	GO:0002635	0.00638
M_38	GO:1902856	0.00956
M_38	GO:1903348	0.00956
M_38	GO:1904158	0.00956
M_38	GO:0042073	0.01157
M_38	GO:0007018	0.01244
M_38	GO:0048515	0.01516
M_38	GO:0045409	0.01588
M_38	GO:0035502	0.01903
M_38	GO:0002924	0.01903
M_38	GO:0007283	0.02439
M_38	GO:0006108	0.02529
M_38	GO:0033085	0.02529
M_38	GO:0000492	0.02529
M_38	GO:0030030	0.02545
M_38	GO:0007288	0.03461
M_38	GO:2000291	0.03461
M_38	GO:0006107	0.0377
M_38	GO:0007052	0.04909
M_38	GO:0031573	0.04996
M_39	GO:0006357	0.00024
M_39	GO:0045104	0.00309
M_39	GO:0072707	0.00337
M_39	GO:0039563	0.00337
M_39	GO:0039564	0.00337
M_39	GO:0039502	0.00337
M_39	GO:0006378	0.00502
M_39	GO:2000397	0.00672
M_39	GO:0072738	0.00672

M_39	GO:0034342	0.00672
M_39	GO:0051548	0.00672
M_39	GO:0000448	0.00672
M_39	GO:0042351	0.00672
M_39	GO:0035915	0.00672
M_39	GO:0016480	0.01007
M_39	GO:1902512	0.01007
M_39	GO:0032218	0.01007
M_39	GO:0021747	0.01007
M_39	GO:1900365	0.01007
M_39	GO:0010847	0.01007
M_39	GO:0015770	0.01007
M_39	GO:0045071	0.01327
M_39	GO:0043328	0.0134
M_39	GO:0090261	0.0134
M_39	GO:0016080	0.0134
M_39	GO:0071896	0.0134
M_39	GO:0070212	0.0134
M_39	GO:0070213	0.0134
M_39	GO:0034340	0.01653
M_39	GO:0097039	0.01673
M_39	GO:0055129	0.01673
M_39	GO:1903936	0.01673
M_39	GO:1904491	0.01673
M_39	GO:0051964	0.02004
M_39	GO:2001033	0.02004
M_39	GO:0032020	0.02004
M_39	GO:2000348	0.02004
M_39	GO:0003400	0.02004
M_39	GO:0051666	0.02004
M_39	GO:1900045	0.02004
M_39	GO:0043615	0.02004

M_39	GO:0000147	0.02004
M_39	GO:0016255	0.02004
M_39	GO:0010793	0.02004
M_39	GO:1904528	0.02004
M_39	GO:0060136	0.02004
M_39	GO:0051902	0.02334
M_39	GO:0071051	0.02334
M_39	GO:0019673	0.02334
M_39	GO:0045654	0.02334
M_39	GO:0045110	0.02334
M_39	GO:0006771	0.02334
M_39	GO:0032088	0.02579
M_39	GO:0061408	0.02663
M_39	GO:0007172	0.02663
M_39	GO:0043497	0.02663
M_39	GO:0032927	0.02663
M_39	GO:0034475	0.02663
M_39	GO:0038007	0.02663
M_39	GO:0036155	0.02663
M_39	GO:0030262	0.02945
M_39	GO:0006621	0.02991
M_39	GO:0006265	0.02991
M_39	GO:0060287	0.03318
M_39	GO:0034379	0.03318
M_39	GO:0090084	0.03318
M_39	GO:0034427	0.03318
M_39	GO:0035865	0.03318
M_39	GO:0030182	0.03488
M_39	GO:0060088	0.03644
M_39	GO:1904385	0.03644
M_39	GO:0002903	0.03969
M_39	GO:0071044	0.03969

M_39	GO:0031125	0.03969
M_39	GO:0070269	0.03969
M_39	GO:0031581	0.03969
M_39	GO:0000460	0.04226
M_39	GO:0045006	0.04292
M_39	GO:0098789	0.04292
M_39	GO:0071028	0.04292
M_39	GO:0010839	0.04292
M_39	GO:0046710	0.04615
M_39	GO:0090494	0.04615
M_39	GO:0001921	0.04615
M_39	GO:0055089	0.04615
M_39	GO:0006122	0.04615
M_39	GO:2000060	0.04615
M_39	GO:0034356	0.04936
M_39	GO:0007097	0.04936
M_40	GO:0070898	0.00048
M_40	GO:0050765	0.00786
M_40	GO:0007049	0.00803
M_40	GO:0061565	0.00902
M_40	GO:0061566	0.00902
M_40	GO:0061567	0.00902
M_40	GO:0061568	0.00902
M_40	GO:0061569	0.00902
M_40	GO:0061570	0.00902
M_40	GO:0061571	0.00902
M_40	GO:0061508	0.00902
M_40	GO:0032913	0.00902
M_40	GO:0070899	0.00902
M_40	GO:0039023	0.00902
M_40	GO:0010139	0.00902
M_40	GO:0039008	0.00902

M_40	GO:0030862	0.00902
M_40	GO:0006186	0.00902
M_40	GO:0006174	0.00902
M_40	GO:0043004	0.00902
M_40	GO:0010795	0.00902
M_40	GO:0075521	0.00902
M_40	GO:0006756	0.00902
M_40	GO:0015782	0.00902
M_40	GO:0001544	0.00902
M_40	GO:0010509	0.00902
M_40	GO:0032597	0.00902
M_40	GO:0006557	0.00902
M_40	GO:1902685	0.00902
M_40	GO:0032600	0.00902
M_40	GO:0002100	0.00902
M_40	GO:0070121	0.00902
M_40	GO:0044053	0.00902
M_40	GO:0009617	0.01526
M_40	GO:0016239	0.01619
M_40	GO:0032543	0.01619
M_40	GO:1901407	0.01785
M_40	GO:0019255	0.01796
M_40	GO:0009405	0.01796
M_40	GO:0046657	0.01796
M_40	GO:0009257	0.01796
M_40	GO:0070676	0.01796
M_40	GO:0019060	0.01796
M_40	GO:0001827	0.01796
M_40	GO:0001828	0.01796
M_40	GO:0038016	0.01796
M_40	GO:2000642	0.01796
M_40	GO:0006597	0.01796

M_40	GO:0019408	0.01796
M_40	GO:0098507	0.01796
M_40	GO:0003032	0.01796
M_40	GO:0019100	0.01796
M_40	GO:0006479	0.02126
M_40	GO:0016032	0.02228
M_40	GO:0001916	0.0228
M_40	GO:0070911	0.0228
M_40	GO:0061099	0.02448
M_40	GO:0045648	0.02448
M_40	GO:0006635	0.02538
M_40	GO:2000757	0.02667
M_40	GO:0010360	0.02681
M_40	GO:0035928	0.02681
M_40	GO:1990785	0.02681
M_40	GO:0006362	0.02681
M_40	GO:0072139	0.02681
M_40	GO:0006452	0.02681
M_40	GO:0039689	0.02681
M_40	GO:0006420	0.02681
M_40	GO:0071386	0.02681
M_40	GO:2000768	0.02681
M_40	GO:0070681	0.02681
M_40	GO:0016557	0.02681
M_40	GO:0035711	0.02681
M_40	GO:0006267	0.02681
M_40	GO:0090234	0.02681
M_40	GO:0061198	0.02681
M_40	GO:1904222	0.02681
M_40	GO:1904219	0.02681
M_40	GO:0006850	0.02681
M_40	GO:2000619	0.02681

M_40	GO:0031204	0.02681
M_40	GO:0045905	0.02681
M_40	GO:0032268	0.02703
M_40	GO:0006334	0.02899
M_40	GO:0006363	0.03168
M_40	GO:0061024	0.03525
M_40	GO:0070423	0.03555
M_40	GO:0071500	0.03559
M_40	GO:0006344	0.03559
M_40	GO:0015917	0.03559
M_40	GO:0097026	0.03559
M_40	GO:1901300	0.03559
M_40	GO:2000825	0.03559
M_40	GO:0006235	0.03559
M_40	GO:0035844	0.03559
M_40	GO:0008295	0.03559
M_40	GO:0036337	0.03559
M_40	GO:0065001	0.03559
M_40	GO:1902035	0.03559
M_40	GO:0006696	0.03559
M_40	GO:0006041	0.03559
M_40	GO:0034553	0.03559
M_40	GO:0045901	0.03559
M_40	GO:0000027	0.03756
M_40	GO:0006370	0.03756
M_40	GO:0032212	0.03756
M_40	GO:0051973	0.03756
M_40	GO:0030488	0.03756
M_40	GO:0002755	0.04169
M_40	GO:0006361	0.04169
M_40	GO:0006270	0.04382
M_40	GO:1903232	0.04429

M_40	GO:0032383	0.04429
M_40	GO:0008611	0.04429
M_40	GO:0060718	0.04429
M_40	GO:1990592	0.04429
M_40	GO:0048840	0.04429
M_40	GO:0035744	0.04429
M_40	GO:0006226	0.04429
M_40	GO:0030091	0.04429
M_40	GO:0075044	0.04429
M_40	GO:0035694	0.04429
M_40	GO:0070934	0.04429
M_40	GO:0045347	0.04429
M_40	GO:0006807	0.04642
M_40	GO:0033146	0.04819
M_40	GO:0008654	0.04825
M_41	GO:0006930	0.00082
M_41	GO:0038003	0.00082
M_41	GO:0090307	0.00101
M_41	GO:0051016	0.00303
M_41	GO:0045454	0.00308
M_41	GO:0043491	0.00373
M_41	GO:0018230	0.00406
M_41	GO:0035602	0.00434
M_41	GO:0035603	0.00434
M_41	GO:0035604	0.00434
M_41	GO:0061573	0.00434
M_41	GO:1901307	0.00434
M_41	GO:0098749	0.00434
M_41	GO:0031161	0.00434
M_41	GO:0061163	0.00434
M_41	GO:1990456	0.00434
M_41	GO:0042104	0.00482

M_41	GO:0046856	0.00522
M_41	GO:0070536	0.00847
M_41	GO:0035607	0.00866
M_41	GO:0046203	0.00866
M_41	GO:0060915	0.00866
M_41	GO:0006780	0.00866
M_41	GO:0001732	0.00866
M_41	GO:0002282	0.00866
M_41	GO:0060595	0.00866
M_41	GO:0060529	0.00866
M_41	GO:0016598	0.00866
M_41	GO:0060667	0.00866
M_41	GO:0060327	0.00866
M_41	GO:0060615	0.00866
M_41	GO:1904059	0.00866
M_41	GO:0060454	0.00866
M_41	GO:0071418	0.00866
M_41	GO:1900026	0.00899
M_41	GO:0040018	0.00899
M_41	GO:0051301	0.00926
M_41	GO:0008608	0.00952
M_41	GO:2000255	0.01296
M_41	GO:0009447	0.01296
M_41	GO:0002188	0.01296
M_41	GO:0044571	0.01296
M_41	GO:1904139	0.01296
M_41	GO:0060523	0.01296
M_41	GO:0090176	0.01296
M_41	GO:0021847	0.01296
M_41	GO:0070541	0.01296
M_41	GO:0007264	0.01595
M_41	GO:0060669	0.01704

M_41	GO:1990168	0.01724
M_41	GO:0046208	0.01724
M_41	GO:0048386	0.01724
M_41	GO:0070236	0.01724
M_41	GO:0038028	0.01724
M_41	GO:0018171	0.01724
M_41	GO:0097056	0.01724
M_41	GO:1990481	0.01724
M_41	GO:0044791	0.02151
M_41	GO:0060916	0.02151
M_41	GO:0075525	0.02151
M_41	GO:0009446	0.02151
M_41	GO:0097350	0.02151
M_41	GO:0060501	0.02151
M_41	GO:0060664	0.02151
M_41	GO:1904058	0.02151
M_41	GO:0010815	0.02151
M_41	GO:2000467	0.02151
M_41	GO:0035523	0.02151
M_41	GO:0007062	0.02378
M_41	GO:0050882	0.02575
M_41	GO:0071947	0.02575
M_41	GO:0051415	0.02575
M_41	GO:0032876	0.02575
M_41	GO:0060527	0.02575
M_41	GO:0006631	0.02753
M_41	GO:0007010	0.02776
M_41	GO:0051896	0.02912
M_41	GO:0033674	0.02987
M_41	GO:0031848	0.02998
M_41	GO:0060687	0.02998
M_41	GO:0060463	0.02998

M_41	GO:0060449	0.02998
M_41	GO:0016477	0.03341
M_41	GO:0030916	0.03419
M_41	GO:0006782	0.03419
M_41	GO:0032808	0.03419
M_41	GO:0038007	0.03419
M_41	GO:0010700	0.03419
M_41	GO:0061101	0.03419
M_41	GO:0021860	0.03419
M_41	GO:0060484	0.03419
M_41	GO:0060180	0.03419
M_41	GO:0006072	0.03838
M_41	GO:0055091	0.03838
M_41	GO:0097428	0.03838
M_41	GO:2000807	0.03838
M_41	GO:0001514	0.03838
M_41	GO:2001135	0.03838
M_41	GO:0048755	0.03838
M_41	GO:0031017	0.03838
M_41	GO:1900272	0.03838
M_41	GO:0008543	0.04119
M_41	GO:0075522	0.04256
M_41	GO:0060068	0.04256
M_41	GO:0070970	0.04256
M_41	GO:0001553	0.04256
M_41	GO:0048280	0.04256
M_41	GO:0003149	0.04256
M_41	GO:0006307	0.04256
M_41	GO:0019371	0.04671
M_41	GO:0090161	0.04671
M_41	GO:0000055	0.04671
M_41	GO:0042532	0.04671

M_41	GO:0051593	0.04671
M_41	GO:0060174	0.04671
M_41	GO:0033327	0.04671
M_41	GO:0010498	0.04739
M_42	GO:0006357	0.00094
M_42	GO:0000724	0.00161
M_42	GO:1903351	0.00326
M_42	GO:0006253	0.00616
M_42	GO:0009245	0.00616
M_42	GO:0035752	0.00616
M_42	GO:2000657	0.00616
M_42	GO:0071454	0.00616
M_42	GO:0034775	0.00616
M_42	GO:0047496	0.00733
M_42	GO:1904357	0.01111
M_42	GO:0016260	0.01229
M_42	GO:0043311	0.01229
M_42	GO:0001575	0.01229
M_42	GO:1901255	0.01229
M_42	GO:0006481	0.01229
M_42	GO:0006424	0.01229
M_42	GO:0070329	0.01229
M_42	GO:0045819	0.01229
M_42	GO:0032435	0.01537
M_42	GO:0002184	0.01838
M_42	GO:0070291	0.01838
M_42	GO:1904431	0.01838
M_42	GO:0017187	0.01838
M_42	GO:0044571	0.01838
M_42	GO:1903895	0.01838
M_42	GO:1990637	0.01838
M_42	GO:0032212	0.01858

M_42	GO:0051973	0.01858
M_42	GO:0006281	0.01951
M_42	GO:0032204	0.02341
M_42	GO:0030641	0.02432
M_42	GO:0035616	0.02443
M_42	GO:0006295	0.02443
M_42	GO:1901537	0.02443
M_42	GO:0032053	0.02443
M_42	GO:0090170	0.02443
M_42	GO:2001168	0.02443
M_42	GO:1901097	0.02443
M_42	GO:0048861	0.02443
M_42	GO:0000086	0.02904
M_42	GO:1901525	0.03045
M_42	GO:0042262	0.03045
M_42	GO:0010216	0.03045
M_42	GO:1904353	0.03613
M_42	GO:1904417	0.03643
M_42	GO:0042791	0.03643
M_42	GO:0042797	0.03643
M_42	GO:0010792	0.03643
M_42	GO:0002741	0.03643
M_42	GO:1990668	0.03643
M_42	GO:0060440	0.03643
M_42	GO:0042373	0.03643
M_42	GO:0051568	0.03807
M_42	GO:0000184	0.03888
M_42	GO:0045652	0.03947
M_42	GO:0007000	0.04237
M_42	GO:1902416	0.04237
M_42	GO:1901098	0.04237
M_42	GO:0006678	0.04237

M_42	GO:0031952	0.0475
M_42	GO:0042420	0.04828
M_42	GO:0015889	0.04828
M_42	GO:0006975	0.04828
M_42	GO:0015809	0.04828
M_42	GO:0060020	0.04828
M_42	GO:0070127	0.04828
M_42	GO:0033129	0.04828
M_42	GO:0061308	0.04828
M_42	GO:1900364	0.04828
M_42	GO:0009249	0.04828
M_42	GO:0035754	0.04828
M_42	GO:0030388	0.04828
M_42	GO:0007042	0.04828
M_42	GO:0006681	0.04828
M_42	GO:0006627	0.04828
M_42	GO:0071169	0.04828
M_42	GO:1990416	0.04828
M_43	GO:1902958	0.0001
M_43	GO:1903384	0.0001
M_43	GO:0032148	0.00028
M_43	GO:0010592	0.00073
M_43	GO:0051881	0.00096
M_43	GO:1903298	0.00098
M_43	GO:0032091	0.0027
M_43	GO:2001171	0.00345
M_43	GO:0071233	0.00345
M_43	GO:0090073	0.00345
M_43	GO:1903955	0.00399
M_43	GO:1990253	0.00428
M_43	GO:0007596	0.00462
M_43	GO:0006986	0.0047

M_43	GO:0071340	0.0052
M_43	GO:0035999	0.0062
M_43	GO:0055074	0.00628
M_43	GO:0006369	0.0065
M_43	GO:0010952	0.00724
M_43	GO:0015937	0.00728
M_43	GO:0060081	0.00728
M_43	GO:1900025	0.00728
M_43	GO:0090141	0.00967
M_43	GO:1900034	0.00976
M_43	GO:1903197	0.01005
M_43	GO:1903168	0.01005
M_43	GO:1903189	0.01005
M_43	GO:1902511	0.01005
M_43	GO:1903178	0.01005
M_43	GO:1903122	0.01005
M_43	GO:0009234	0.01005
M_43	GO:1903200	0.01005
M_43	GO:0018323	0.01005
M_43	GO:0032194	0.01005
M_43	GO:1902010	0.01005
M_43	GO:1903852	0.01005
M_43	GO:1904193	0.01005
M_43	GO:1904197	0.01005
M_43	GO:1904151	0.01005
M_43	GO:1904140	0.01005
M_43	GO:0046295	0.01005
M_43	GO:1904213	0.01005
M_43	GO:1904206	0.01005
M_43	GO:1902389	0.01005
M_43	GO:0070782	0.01005
M_43	GO:2000277	0.01005

M_43	GO:0031930	0.01005
M_43	GO:0043610	0.01005
M_43	GO:1903073	0.01005
M_43	GO:0021528	0.01005
M_43	GO:0042371	0.01005
M_43	GO:0033274	0.01005
M_43	GO:0044721	0.01005
M_43	GO:0035853	0.01005
M_43	GO:1904544	0.01005
M_43	GO:0036471	0.01005
M_43	GO:0097298	0.01005
M_43	GO:0016243	0.01005
M_43	GO:0070625	0.01005
M_43	GO:0036250	0.01005
M_43	GO:1904056	0.01005
M_43	GO:0032516	0.01098
M_43	GO:0070886	0.01098
M_43	GO:0036297	0.01309
M_43	GO:2000251	0.01382
M_43	GO:0032868	0.01492
M_43	GO:0051497	0.01693
M_43	GO:0048025	0.01693
M_43	GO:0042276	0.01859
M_43	GO:0035794	0.01971
M_43	GO:1902110	0.01982
M_43	GO:1902884	0.0199
M_43	GO:0071451	0.01991
M_43	GO:0035307	0.01995
M_43	GO:0080144	0.01999
M_43	GO:0016561	0.01999
M_43	GO:0086092	0.01999
M_43	GO:1903181	0.01999

M_43	GO:0072356	0.01999
M_43	GO:1903281	0.01999
M_43	GO:0061386	0.01999
M_43	GO:0019521	0.01999
M_43	GO:0006051	0.01999
M_43	GO:0050787	0.01999
M_43	GO:1903538	0.01999
M_43	GO:1903691	0.01999
M_43	GO:0098508	0.01999
M_43	GO:2000157	0.01999
M_43	GO:0002528	0.01999
M_43	GO:0071048	0.01999
M_43	GO:1903094	0.01999
M_43	GO:2001045	0.01999
M_43	GO:0070829	0.01999
M_43	GO:0006481	0.01999
M_43	GO:0006437	0.01999
M_43	GO:1990519	0.01999
M_43	GO:0006172	0.01999
M_43	GO:1902227	0.01999
M_43	GO:0032076	0.01999
M_43	GO:0036114	0.01999
M_43	GO:0036116	0.01999
M_43	GO:0034445	0.01999
M_43	GO:1901094	0.01999
M_43	GO:0014736	0.01999
M_43	GO:0006401	0.02195
M_43	GO:0050821	0.02282
M_43	GO:0072593	0.02352
M_43	GO:2000463	0.02588
M_43	GO:0002479	0.02785
M_43	GO:0032869	0.02893

M_43	GO:1901031	0.02956
M_43	GO:0098779	0.02968
M_43	GO:1901984	0.0297
M_43	GO:0019249	0.02984
M_43	GO:0046947	0.02984
M_43	GO:0010133	0.02984
M_43	GO:0016094	0.02984
M_43	GO:0070495	0.02984
M_43	GO:0022007	0.02984
M_43	GO:0061198	0.02984
M_43	GO:0071650	0.02984
M_43	GO:0006014	0.02984
M_43	GO:0034628	0.02984
M_43	GO:1902903	0.02984
M_43	GO:1903679	0.02984
M_43	GO:0009051	0.02984
M_43	GO:0051490	0.02984
M_43	GO:0071386	0.02984
M_43	GO:1901671	0.02984
M_43	GO:0090118	0.02984
M_43	GO:0090258	0.02984
M_43	GO:0031333	0.03031
M_43	GO:0048662	0.03412
M_43	GO:1903827	0.0359
M_43	GO:0022900	0.03612
M_43	GO:0090090	0.0374
M_43	GO:0043392	0.03786
M_43	GO:0034080	0.03857
M_43	GO:0030307	0.03904
M_43	GO:1903426	0.03929
M_43	GO:0010833	0.03935
M_43	GO:0052548	0.03957

M_43	GO:0009438	0.03959
M_43	GO:1990034	0.03959
M_43	GO:0046098	0.03959
M_43	GO:0016560	0.03959
M_43	GO:1903401	0.03959
M_43	GO:2001181	0.03959
M_43	GO:1904000	0.03959
M_43	GO:1902527	0.03959
M_43	GO:1900535	0.03959
M_43	GO:2000491	0.03959
M_43	GO:0014042	0.03959
M_43	GO:1903527	0.03959
M_43	GO:0071034	0.03959
M_43	GO:0008295	0.03959
M_43	GO:0038189	0.03959
M_43	GO:2000825	0.03959
M_43	GO:0097118	0.03959
M_43	GO:0019322	0.03959
M_43	GO:0019470	0.03959
M_43	GO:0018094	0.03959
M_43	GO:1990086	0.03959
M_43	GO:0006741	0.03959
M_43	GO:0010569	0.04088
M_43	GO:0034644	0.04385
M_43	GO:0000027	0.04564
M_43	GO:0071456	0.04584
M_43	GO:0006302	0.04748
M_43	GO:0007616	0.04809
M_43	GO:2000048	0.04924
M_43	GO:0070940	0.04924
M_43	GO:2001268	0.04924
M_43	GO:0060633	0.04924

M_43	GO:0098735	0.04924
M_43	GO:0030473	0.04924
M_43	GO:1903826	0.04924
M_43	GO:1900028	0.04924
M_43	GO:0036091	0.04924
M_43	GO:0030263	0.04924
M_43	GO:0051987	0.04924
M_43	GO:0000738	0.04924
M_43	GO:0061734	0.04924
M_43	GO:0033184	0.04924
M_43	GO:0050689	0.04924
M_43	GO:0072656	0.04924
M_43	GO:0006432	0.04924
M_43	GO:2000809	0.04924
M_43	GO:1904504	0.04924
M_43	GO:1900226	0.04924
M_43	GO:1904058	0.04924
M_44	GO:0060294	0.00048
M_44	GO:0070462	0.00171
M_44	GO:0060404	0.00171
M_44	GO:0038043	0.00342
M_44	GO:0030317	0.00673
M_44	GO:1903251	0.00683
M_44	GO:0035694	0.00853
M_44	GO:1902260	0.01193
M_44	GO:0036302	0.01193
M_44	GO:0035881	0.01362
M_44	GO:0060050	0.01531
M_44	GO:0032782	0.01531
M_44	GO:1904668	0.02204
M_44	GO:0070863	0.02204
M_44	GO:0007017	0.02311

M_44	GO:0060579	0.02539
M_44	GO:0023014	0.03092
M_44	GO:0007156	0.03192
M_44	GO:0035695	0.03206
M_44	GO:0060271	0.03374
M_44	GO:0032674	0.03537
M_44	GO:0036158	0.03537
M_44	GO:1901687	0.03703
M_44	GO:0010842	0.03703
M_44	GO:0044458	0.03868
M_44	GO:0001578	0.04091
M_44	GO:0035082	0.04546
M_44	GO:0007018	0.04779
M_45	GO:0098740	0.0000086
M_45	GO:0060065	0.000027
M_45	GO:0060157	0.000086
M_45	GO:0008584	0.00026
M_45	GO:0008595	0.0003
M_45	GO:0005513	0.00047
M_45	GO:0007368	0.00052
M_45	GO:0006957	0.00077
M_45	GO:0071333	0.00082
M_45	GO:0035729	0.00089
M_45	GO:0030509	0.00109
M_45	GO:0038092	0.00114
M_45	GO:0044344	0.00282
M_45	GO:0097210	0.00291
M_45	GO:1900239	0.00297
M_45	GO:0034255	0.00297
M_45	GO:0038185	0.00297
M_45	GO:0000256	0.00297
M_45	GO:0007387	0.00297

M_45	GO:0007388	0.00297
M_45	GO:0021831	0.00297
M_45	GO:0019101	0.00297
M_45	GO:0061350	0.00297
M_45	GO:0061354	0.00297
M_45	GO:2001250	0.00297
M_45	GO:0061347	0.00297
M_45	GO:0061348	0.00297
M_45	GO:0061349	0.00297
M_45	GO:0001080	0.00297
M_45	GO:0010469	0.00488
M_45	GO:0048286	0.00539
M_45	GO:0045944	0.0058
M_45	GO:0060067	0.00593
M_45	GO:0034971	0.00593
M_45	GO:0060014	0.00593
M_45	GO:0060809	0.00593
M_45	GO:0060599	0.00593
M_45	GO:0032902	0.00593
M_45	GO:0017143	0.00593
M_45	GO:0051885	0.00593
M_45	GO:0006601	0.00593
M_45	GO:0097325	0.00593
M_45	GO:0003290	0.00593
M_45	GO:2000724	0.00593
M_45	GO:0072615	0.00593
M_45	GO:0030103	0.00593
M_45	GO:0060775	0.00593
M_45	GO:1902613	0.00593
M_45	GO:0071560	0.00745
M_45	GO:0001560	0.00888
M_45	GO:0070859	0.00888

M_45	GO:0001555	0.00888
M_45	GO:0018963	0.00888
M_45	GO:1900222	0.00888
M_45	GO:0072277	0.00888
M_45	GO:0014707	0.00888
M_45	GO:0007538	0.00888
M_45	GO:0003408	0.00888
M_45	GO:0045163	0.00888
M_45	GO:0060435	0.00888
M_45	GO:2000721	0.00888
M_45	GO:1902714	0.00888
M_45	GO:0048850	0.00888
M_45	GO:0048022	0.00888
M_45	GO:0035054	0.00888
M_45	GO:0071373	0.00888
M_45	GO:0030154	0.01031
M_45	GO:0000122	0.01083
M_45	GO:0070858	0.01182
M_45	GO:0060686	0.01182
M_45	GO:0014826	0.01182
M_45	GO:0021759	0.01182
M_45	GO:0018894	0.01182
M_45	GO:0018879	0.01182
M_45	GO:0003257	0.01182
M_45	GO:0033686	0.01182
M_45	GO:2000587	0.01182
M_45	GO:0070253	0.01182
M_45	GO:0060750	0.01182
M_45	GO:0001880	0.01182
M_45	GO:0043401	0.01201
M_45	GO:0001947	0.01327
M_45	GO:0003231	0.01428

M_45	GO:0072162	0.01475
M_45	GO:0033132	0.01475
M_45	GO:0097070	0.01475
M_45	GO:1904179	0.01475
M_45	GO:2000195	0.01475
M_45	GO:0051891	0.01475
M_45	GO:1900020	0.01475
M_45	GO:0003289	0.01475
M_45	GO:0010961	0.01475
M_45	GO:0070245	0.01475
M_45	GO:0060762	0.01475
M_45	GO:0060744	0.01475
M_45	GO:0002074	0.01475
M_45	GO:0071346	0.01514
M_45	GO:0021891	0.01734
M_45	GO:0060029	0.01768
M_45	GO:2001015	0.01768
M_45	GO:1902474	0.01768
M_45	GO:0060613	0.01768
M_45	GO:0003402	0.01768
M_45	GO:0007354	0.01768
M_45	GO:0021853	0.01768
M_45	GO:0060426	0.01768
M_45	GO:0003100	0.01768
M_45	GO:0060992	0.01768
M_45	GO:0060907	0.01768
M_45	GO:0009635	0.01768
M_45	GO:0038031	0.01768
M_45	GO:0071638	0.01768
M_45	GO:0002741	0.01768
M_45	GO:0046881	0.01768
M_45	GO:1900425	0.01768

M_45	GO:0051964	0.01768
M_45	GO:0071222	0.01778
M_45	GO:0006367	0.01881
M_45	GO:0071774	0.01957
M_45	GO:0030510	0.0199
M_45	GO:0060028	0.0206
M_45	GO:0060012	0.0206
M_45	GO:1904468	0.0206
M_45	GO:0048341	0.0206
M_45	GO:0035356	0.0206
M_45	GO:0097623	0.0206
M_45	GO:0007442	0.0206
M_45	GO:0060290	0.0206
M_45	GO:2000049	0.0206
M_45	GO:0061370	0.0206
M_45	GO:0097211	0.0206
M_45	GO:0032755	0.02302
M_45	GO:0072201	0.02351
M_45	GO:0060638	0.02351
M_45	GO:0009750	0.02351
M_45	GO:0042747	0.02351
M_45	GO:0021800	0.02351
M_45	GO:0090037	0.02351
M_45	GO:2000020	0.02351
M_45	GO:0019896	0.02351
M_45	GO:0045080	0.02351
M_45	GO:0001763	0.02517
M_45	GO:0048048	0.02594
M_45	GO:0007193	0.02637
M_45	GO:0019695	0.0264
M_45	GO:0060539	0.0264
M_45	GO:0010988	0.0264

M_45	GO:0048570	0.0264
M_45	GO:1900426	0.0264
M_45	GO:0048617	0.0264
M_45	GO:0008090	0.0264
M_45	GO:0060068	0.0293
M_45	GO:0001553	0.0293
M_45	GO:0035457	0.0293
M_45	GO:0010459	0.0293
M_45	GO:1902004	0.0293
M_45	GO:0030072	0.03075
M_45	GO:0010595	0.03113
M_45	GO:0010820	0.03218
M_45	GO:0060340	0.03218
M_45	GO:0090009	0.03218
M_45	GO:0071372	0.03218
M_45	GO:0006874	0.0323
M_45	GO:1904469	0.03505
M_45	GO:0014889	0.03505
M_45	GO:0032099	0.03505
M_45	GO:0007494	0.03505
M_45	GO:2000052	0.03505
M_45	GO:0060766	0.03505
M_45	GO:0008284	0.03568
M_45	GO:0006703	0.03792
M_45	GO:0090179	0.03792
M_45	GO:0046415	0.03792
M_45	GO:0071872	0.03792
M_45	GO:0045198	0.03792
M_45	GO:0002087	0.03792
M_45	GO:0045836	0.03792
M_45	GO:0007218	0.03885
M_45	GO:0051091	0.03937

M_45	GO:0008207	0.04006
M_45	GO:0060079	0.04021
M_45	GO:0090280	0.04078
M_45	GO:0055089	0.04078
M_45	GO:0060158	0.04078
M_45	GO:0071361	0.04078
M_45	GO:0001867	0.04078
M_45	GO:0030449	0.04089
M_45	GO:0009607	0.04092
M_45	GO:0000187	0.04097
M_45	GO:0046628	0.04363
M_45	GO:2000484	0.04363
M_45	GO:0016082	0.04363
M_45	GO:0070268	0.04366
M_45	GO:0007267	0.04485
M_45	GO:0072148	0.04647
M_45	GO:0044241	0.04647
M_45	GO:0040037	0.04647
M_45	GO:0007635	0.04647
M_45	GO:0035988	0.04647
M_45	GO:0048070	0.04647
M_45	GO:0086013	0.04647
M_45	GO:0051457	0.04647
M_45	GO:0060322	0.04848
M_45	GO:0010832	0.0493
M_45	GO:0036342	0.0493
M_45	GO:0071236	0.0493
M_45	GO:0032367	0.0493
M_45	GO:1900116	0.0493
M_45	GO:0009792	0.04995
M_46	GO:0031424	4.9E-10
M_46	GO:0044278	0.00000027

M_46	GO:0006805	0.0000054
M_46	GO:0019373	0.000084
M_46	GO:0018149	0.0001
M_46	GO:0042738	0.00013
M_46	GO:0016098	0.00025
M_46	GO:0002933	0.00025
M_46	GO:0045617	0.00035
M_46	GO:0050832	0.0004
M_46	GO:0051673	0.00059
M_46	GO:0010838	0.00059
M_46	GO:0097267	0.00059
M_46	GO:0031284	0.00089
M_46	GO:0070268	0.00117
M_46	GO:0070989	0.00169
M_46	GO:0002227	0.00272
M_46	GO:0045609	0.00411
M_46	GO:0019428	0.00411
M_46	GO:0061170	0.00411
M_46	GO:0033626	0.00411
M_46	GO:0019628	0.00411
M_46	GO:0016269	0.00411
M_46	GO:0042667	0.00411
M_46	GO:0050829	0.00483
M_46	GO:0050830	0.00534
M_46	GO:0042737	0.00787
M_46	GO:1904106	0.0082
M_46	GO:0043434	0.00863
M_46	GO:0007586	0.00869
M_46	GO:0019730	0.01118
M_46	GO:0006882	0.01119
M_46	GO:0002314	0.01228
M_46	GO:0009822	0.01228

M_46	GO:0042668	0.01228
M_46	GO:0019731	0.0129
M_46	GO:0006116	0.01634
M_46	GO:0031640	0.01686
M_46	GO:0006953	0.0173
M_46	GO:0090303	0.0173
M_46	GO:0098700	0.02038
M_46	GO:0061624	0.02038
M_46	GO:0071557	0.02038
M_46	GO:0032747	0.02038
M_46	GO:0044245	0.02441
M_46	GO:0035948	0.02842
M_46	GO:0035672	0.02842
M_46	GO:0030388	0.03242
M_46	GO:0071578	0.03242
M_46	GO:0032000	0.03639
M_46	GO:0097400	0.03639
M_46	GO:0002315	0.03639
M_46	GO:0008202	0.03873
M_46	GO:0030311	0.04036
M_46	GO:0006570	0.04036
M_46	GO:0051715	0.04036
M_46	GO:0070072	0.04036
M_46	GO:0072537	0.04036
M_46	GO:1900004	0.0443
M_46	GO:0016554	0.04823
M_47	GO:0072137	0.000041
M_47	GO:0007157	0.000055
M_47	GO:0030512	0.00012
M_47	GO:0086045	0.00013
M_47	GO:0043931	0.00027
M_47	GO:0045668	0.00054

M_47	GO:0030514	0.00063
M_47	GO:0030198	0.00089
M_47	GO:0001558	0.00235
M_47	GO:0070584	0.00247
M_47	GO:0070483	0.00371
M_47	GO:2000763	0.00371
M_47	GO:2000764	0.00371
M_47	GO:0071881	0.00371
M_47	GO:0071882	0.00371
M_47	GO:0021919	0.00371
M_47	GO:0090138	0.00371
M_47	GO:0010045	0.00371
M_47	GO:1901231	0.00371
M_47	GO:1901233	0.00371
M_47	GO:0090249	0.00371
M_47	GO:0002380	0.00371
M_47	GO:0042147	0.00388
M_47	GO:0030010	0.00555
M_47	GO:0060721	0.00741
M_47	GO:1903929	0.00741
M_47	GO:0086053	0.00741
M_47	GO:0038098	0.00741
M_47	GO:0014846	0.00741
M_47	GO:0086021	0.00741
M_47	GO:0042704	0.00741
M_47	GO:0061182	0.00741
M_47	GO:0060378	0.00741
M_47	GO:0021812	0.00741
M_47	GO:0035585	0.00741
M_47	GO:0061032	0.00741
M_47	GO:0030501	0.00745
M_47	GO:0007155	0.00779

M_47	GO:0086091	0.00787
M_47	GO:0035329	0.0083
M_47	GO:0042476	0.00831
M_47	GO:0050919	0.00874
M_47	GO:0061572	0.01084
M_47	GO:0021885	0.01096
M_47	GO:0098912	0.01109
M_47	GO:0072092	0.01109
M_47	GO:0003192	0.01109
M_47	GO:2001170	0.01109
M_47	GO:0048565	0.01248
M_47	GO:0048705	0.01252
M_47	GO:0051496	0.01316
M_47	GO:0010712	0.01456
M_47	GO:0002318	0.01476
M_47	GO:0060244	0.01476
M_47	GO:0048549	0.01476
M_47	GO:0061325	0.01476
M_47	GO:0061343	0.01476
M_47	GO:0002051	0.01476
M_47	GO:0097187	0.01476
M_47	GO:0060364	0.01476
M_47	GO:0043932	0.01476
M_47	GO:0060300	0.01476
M_47	GO:1900158	0.01476
M_47	GO:0003253	0.01476
M_47	GO:0003273	0.01476
M_47	GO:0003219	0.01476
M_47	GO:2001274	0.01476
M_47	GO:0007525	0.01476
M_47	GO:0070346	0.01476
M_47	GO:0002357	0.01476

M_47	GO:0035625	0.01476
M_47	GO:0048515	0.01829
M_47	GO:0060718	0.01842
M_47	GO:0097350	0.01842
M_47	GO:0032811	0.01842
M_47	GO:0010991	0.01842
M_47	GO:1904179	0.01842
M_47	GO:0086046	0.01842
M_47	GO:0010936	0.01842
M_47	GO:0060348	0.01882
M_47	GO:0001658	0.0209
M_47	GO:0002718	0.02146
M_47	GO:0060907	0.02206
M_47	GO:0003278	0.02206
M_47	GO:0007156	0.02305
M_47	GO:0016055	0.02312
M_47	GO:0072307	0.02569
M_47	GO:0003357	0.02569
M_47	GO:0007256	0.02569
M_47	GO:2000669	0.02569
M_47	GO:0048702	0.02569
M_47	GO:0033504	0.02569
M_47	GO:0035507	0.02569
M_47	GO:0030509	0.02631
M_47	GO:0010463	0.02803
M_47	GO:0048539	0.02931
M_47	GO:2000096	0.02931
M_47	GO:0061577	0.02931
M_47	GO:0032825	0.02931
M_47	GO:1903012	0.02931
M_47	GO:0060371	0.02931
M_47	GO:0010700	0.02931

M_47	GO:0071493	0.02931
M_47	GO:0070509	0.02933
M_47	GO:0022617	0.03162
M_47	GO:0008543	0.03236
M_47	GO:0010635	0.03291
M_47	GO:0060982	0.03291
M_47	GO:0048263	0.03291
M_47	GO:0061179	0.03291
M_47	GO:0035860	0.03291
M_47	GO:2001171	0.03291
M_47	GO:0045955	0.03291
M_47	GO:0003266	0.03291
M_47	GO:0086002	0.03547
M_47	GO:0001780	0.03596
M_47	GO:0035457	0.0365
M_47	GO:0060068	0.0365
M_47	GO:0032836	0.0365
M_47	GO:0048251	0.0365
M_47	GO:0007411	0.03745
M_47	GO:0034446	0.03973
M_47	GO:0071773	0.04004
M_47	GO:0051654	0.04008
M_47	GO:0098911	0.04008
M_47	GO:0016264	0.04008
M_47	GO:0010172	0.04008
M_47	GO:0060394	0.04008
M_47	GO:0002523	0.04008
M_47	GO:0033690	0.04008
M_47	GO:1902902	0.04364
M_47	GO:0060391	0.04364
M_47	GO:2001241	0.04364
M_47	GO:0009650	0.04719

M_47	GO:0001967	0.04719
M_47	GO:0043652	0.04719
M_47	GO:0001845	0.04719
M_47	GO:0070208	0.04719
M_47	GO:2000345	0.04719
M_47	GO:0048935	0.04719
M_48	GO:0043069	0.0015
M_48	GO:0001580	0.0018
M_48	GO:0070475	0.0018
M_48	GO:0006488	0.0055
M_48	GO:0000725	0.0058
M_48	GO:1900276	0.0059
M_48	GO:1900737	0.0059
M_48	GO:1902153	0.0059
M_48	GO:1901980	0.0059
M_48	GO:0015761	0.0059
M_48	GO:1904024	0.0059
M_48	GO:1901726	0.0059
M_48	GO:0006463	0.0059
M_48	GO:2000055	0.0059
M_48	GO:0070184	0.0059
M_48	GO:0045209	0.0059
M_48	GO:0038002	0.0059
M_48	GO:1901006	0.0059
M_48	GO:0051402	0.0087
M_48	GO:0061621	0.0094
M_48	GO:0046326	0.01
M_48	GO:1900275	0.0117
M_48	GO:0035606	0.0117
M_48	GO:0033277	0.0117
M_48	GO:0090149	0.0117
M_48	GO:0034721	0.0117

M_48	GO:0046166	0.0117
M_48	GO:0021897	0.0117
M_48	GO:0060434	0.0117
M_48	GO:0035261	0.0117
M_48	GO:1904579	0.0117
M_48	GO:1901094	0.0117
M_48	GO:0044335	0.0117
M_48	GO:0061458	0.0173
M_48	GO:2000302	0.0175
M_48	GO:0036466	0.0175
M_48	GO:0090118	0.0175
M_48	GO:0002314	0.0175
M_48	GO:1900063	0.0175
M_48	GO:0023016	0.0175
M_48	GO:0052501	0.0175
M_48	GO:0046121	0.0175
M_48	GO:2000819	0.0175
M_48	GO:0045204	0.0175
M_48	GO:0035022	0.0175
M_48	GO:0031146	0.0231
M_48	GO:0021914	0.0233
M_48	GO:0060535	0.0233
M_48	GO:0036343	0.0233
M_48	GO:0071279	0.0233
M_48	GO:0090244	0.0233
M_48	GO:0070318	0.0233
M_48	GO:0038028	0.0233
M_48	GO:0016560	0.0233
M_48	GO:0021678	0.0233
M_48	GO:0033600	0.0233
M_48	GO:0080154	0.0233
M_48	GO:0007296	0.0233

M_48	GO:0072385	0.0233
M_48	GO:0016052	0.0285
M_48	GO:0043456	0.0291
M_48	GO:1903301	0.0291
M_48	GO:0010032	0.0291
M_48	GO:0033299	0.0291
M_48	GO:0006003	0.0291
M_48	GO:0033211	0.0291
M_48	GO:0090188	0.0291
M_48	GO:0090245	0.0291
M_48	GO:0021540	0.0291
M_48	GO:0031536	0.0291
M_48	GO:0034392	0.0291
M_48	GO:0060762	0.0291
M_48	GO:1901525	0.0291
M_48	GO:0044340	0.0291
M_48	GO:0060744	0.0291
M_48	GO:1900244	0.0348
M_48	GO:0010637	0.0348
M_48	GO:0035900	0.0348
M_48	GO:0002924	0.0348
M_48	GO:0010792	0.0348
M_48	GO:0050859	0.0348
M_48	GO:0045046	0.0348
M_48	GO:1901621	0.0348
M_48	GO:0043983	0.0404
M_48	GO:0097500	0.0404
M_48	GO:0031087	0.0404
M_48	GO:0048702	0.0404
M_48	GO:1903546	0.0404
M_48	GO:0090650	0.0404
M_48	GO:0019563	0.0404

M_48	GO:2001244	0.0429
M_48	GO:0071901	0.0445
M_48	GO:0060059	0.0461
M_48	GO:0090435	0.0461
M_48	GO:0090160	0.0461
M_48	GO:0030388	0.0461
M_48	GO:0031340	0.0461
M_49	GO:0050911	0.000000013
M_49	GO:0007186	0.000041
M_49	GO:1902838	0.0027
M_49	GO:0086072	0.0027
M_49	GO:0000117	0.0054
M_49	GO:0009631	0.0054
M_49	GO:0006583	0.0054
M_49	GO:1990116	0.0054
M_49	GO:0021847	0.008
M_49	GO:0098904	0.008
M_49	GO:0070537	0.008
M_49	GO:0006511	0.0093
M_49	GO:0051865	0.0106
M_49	GO:0035522	0.0107
M_49	GO:0045901	0.0107
M_49	GO:0032259	0.0108
M_49	GO:0008611	0.0133
M_49	GO:0071376	0.0133
M_49	GO:0045653	0.0133
M_49	GO:0072344	0.016
M_49	GO:2000381	0.0213
M_49	GO:0070417	0.0213
M_49	GO:0008354	0.0239
M_49	GO:1904293	0.0343
M_49	GO:0070633	0.0343

M_49	GO:1903351	0.0369
M_49	GO:2000060	0.0369
M_49	GO:0046629	0.0369
M_49	GO:0048739	0.0447
M_49	GO:0006978	0.0498
M_51	GO:1901911	0.00013
M_51	GO:1901907	0.00013
M_51	GO:1901909	0.00013
M_51	GO:0071543	0.00013
M_51	GO:0071504	0.00021
M_51	GO:0019933	0.00025
M_51	GO:0018406	0.00044
M_51	GO:0001657	0.00046
M_51	GO:1902476	0.00071
M_51	GO:0071374	0.00074
M_51	GO:0043087	0.00089
M_51	GO:0060346	0.00093
M_51	GO:0032836	0.00093
M_51	GO:0001886	0.00135
M_51	GO:0006629	0.00177
M_51	GO:0071425	0.00343
M_51	GO:0060078	0.00404
M_51	GO:0002253	0.00462
M_51	GO:0021972	0.00462
M_51	GO:0042413	0.00462
M_51	GO:0090246	0.00462
M_51	GO:0022004	0.00462
M_51	GO:0050929	0.00462
M_51	GO:0001988	0.00462
M_51	GO:0060156	0.00462
M_51	GO:0021588	0.00462
M_51	GO:2000080	0.00462

M_51	GO:0010232	0.00462
M_51	GO:0044345	0.00462
M_51	GO:0001764	0.00469
M_51	GO:0007214	0.00591
M_51	GO:0001525	0.00825
M_51	GO:0030308	0.0083
M_51	GO:0090263	0.00917
M_51	GO:0009258	0.00923
M_51	GO:0010652	0.00923
M_51	GO:0014835	0.00923
M_51	GO:0051885	0.00923
M_51	GO:0090260	0.00923
M_51	GO:0070378	0.00923
M_51	GO:0042700	0.00923
M_51	GO:0021836	0.00923
M_51	GO:0003294	0.00923
M_51	GO:0097338	0.00923
M_51	GO:0021551	0.00923
M_51	GO:0060931	0.00923
M_51	GO:0090024	0.00923
M_51	GO:2000054	0.00923
M_51	GO:2000041	0.00923
M_51	GO:0071676	0.00923
M_51	GO:1904352	0.00923
M_51	GO:0048632	0.00923
M_51	GO:0002931	0.01138
M_51	GO:0007271	0.01138
M_51	GO:0046676	0.01201
M_51	GO:0061184	0.01381
M_51	GO:0001545	0.01381
M_51	GO:0010593	0.01381
M_51	GO:0003365	0.01381

M_51	GO:0046947	0.01381
M_51	GO:0015742	0.01381
M_51	GO:0019254	0.01381
M_51	GO:0060112	0.01381
M_51	GO:0015820	0.01381
M_51	GO:0061364	0.01381
M_51	GO:0071373	0.01381
M_51	GO:0060061	0.01381
M_51	GO:0045578	0.01837
M_51	GO:0014034	0.01837
M_51	GO:0090244	0.01837
M_51	GO:0003104	0.01837
M_51	GO:0090027	0.01837
M_51	GO:0071672	0.01837
M_51	GO:0050893	0.01837
M_51	GO:0044336	0.01837
M_51	GO:0051932	0.01916
M_51	GO:0045599	0.01916
M_51	GO:0017157	0.02059
M_51	GO:0030514	0.02076
M_51	GO:0070374	0.02194
M_51	GO:0042490	0.02241
M_51	GO:0032962	0.02291
M_51	GO:2000987	0.02291
M_51	GO:0021797	0.02291
M_51	GO:0031547	0.02291
M_51	GO:0070100	0.02291
M_51	GO:0042699	0.02291
M_51	GO:0051414	0.02291
M_51	GO:0034197	0.02291
M_51	GO:0042391	0.02623
M_51	GO:0051058	0.02717

M_51	GO:0060527	0.02743
M_51	GO:1903997	0.02743
M_51	GO:0033278	0.02743
M_51	GO:0006552	0.02743
M_51	GO:0030579	0.02743
M_51	GO:0003406	0.02743
M_51	GO:2000270	0.02743
M_51	GO:0015015	0.02743
M_51	GO:1901621	0.02743
M_51	GO:0061196	0.02743
M_51	GO:0045669	0.0286
M_51	GO:0050877	0.03021
M_51	GO:0003407	0.03144
M_51	GO:0060012	0.03192
M_51	GO:0035385	0.03192
M_51	GO:0060687	0.03192
M_51	GO:0060373	0.03192
M_51	GO:2000271	0.03192
M_51	GO:0007416	0.03589
M_51	GO:0016055	0.03639
M_51	GO:0017185	0.0364
M_51	GO:0046322	0.0364
M_51	GO:0060371	0.0364
M_51	GO:0070445	0.0364
M_51	GO:0035095	0.0364
M_51	GO:0030154	0.03666
M_51	GO:0071300	0.04058
M_51	GO:1902430	0.04086
M_51	GO:0007614	0.04086
M_51	GO:0046951	0.04086
M_51	GO:0015824	0.04086
M_51	GO:0033689	0.04086

M_51	GO:2001135	0.04086
M_51	GO:0048664	0.04086
M_51	GO:0046548	0.04086
M_51	GO:0034199	0.04086
M_51	GO:0001843	0.04292
M_51	GO:0061299	0.0453
M_51	GO:0043951	0.0453
M_51	GO:0006554	0.0453
M_51	GO:0042758	0.0453
M_51	GO:0018095	0.0453
M_51	GO:0038063	0.0453
M_51	GO:0033629	0.0453
M_51	GO:1902004	0.0453
M_51	GO:0034378	0.0453
M_51	GO:0034379	0.0453
M_51	GO:0006228	0.0453
M_51	GO:0040017	0.04539
M_51	GO:0008306	0.04716
M_51	GO:0035264	0.04868
M_51	GO:0043116	0.04971
M_51	GO:0042135	0.04971
M_51	GO:0040015	0.04971
M_51	GO:0016264	0.04971
M_51	GO:1900004	0.04971
M_51	GO:0021554	0.04971
M_51	GO:0015867	0.04971
M_51	GO:0006183	0.04971
M_51	GO:0045899	0.04971
M_52	GO:0046329	0.00016
M_52	GO:0006452	0.00021
M_52	GO:0045905	0.00021
M_52	GO:0051081	0.0004

M_52	GO:0045901	0.00041
M_52	GO:0008090	0.00239
M_52	GO:0045953	0.00361
M_52	GO:1902902	0.00431
M_52	GO:2000574	0.00431
M_52	GO:0051028	0.00443
M_52	GO:0051898	0.00479
M_52	GO:0021819	0.00507
M_52	GO:1900029	0.00588
M_52	GO:0050965	0.00675
M_52	GO:0090630	0.00707
M_52	GO:0007004	0.00751
M_52	GO:0032695	0.00767
M_52	GO:0042249	0.00828
M_52	GO:0051299	0.00829
M_52	GO:1901594	0.00833
M_52	GO:0032920	0.00833
M_52	GO:0032919	0.00833
M_52	GO:0032203	0.00833
M_52	GO:0090226	0.00833
M_52	GO:0031448	0.00833
M_52	GO:0070377	0.00833
M_52	GO:2001153	0.00833
M_52	GO:0050960	0.00833
M_52	GO:0046204	0.00833
M_52	GO:0098529	0.00833
M_52	GO:0072362	0.00833
M_52	GO:0071538	0.00833
M_52	GO:0000045	0.00838
M_52	GO:0006406	0.01024
M_52	GO:0035024	0.01075
M_52	GO:0032922	0.01084

M_52	GO:0051497	0.01188
M_52	GO:0047496	0.01306
M_52	GO:0000132	0.01556
M_52	GO:0035305	0.01647
M_52	GO:0007291	0.0166
M_52	GO:0032470	0.0166
M_52	GO:0001172	0.0166
M_52	GO:0030821	0.0166
M_52	GO:0002032	0.0166
M_52	GO:0098905	0.0166
M_52	GO:0016598	0.0166
M_52	GO:0090212	0.0166
M_52	GO:0036309	0.0166
M_52	GO:0060454	0.0166
M_52	GO:0003064	0.0166
M_52	GO:0072368	0.0166
M_52	GO:0090076	0.0166
M_52	GO:0002790	0.01672
M_52	GO:2001234	0.01678
M_52	GO:0003009	0.01805
M_52	GO:0032967	0.01825
M_52	GO:2000311	0.01825
M_52	GO:0006611	0.01948
M_52	GO:0007628	0.01967
M_52	GO:0060079	0.02042
M_52	GO:0016032	0.02334
M_52	GO:0048017	0.02467
M_52	GO:0032527	0.02468
M_52	GO:0035963	0.0248
M_52	GO:2000466	0.0248
M_52	GO:1902309	0.0248
M_52	GO:0032918	0.0248

M_52	GO:0009447	0.0248
M_52	GO:0051659	0.0248
M_52	GO:0034499	0.0248
M_52	GO:0010730	0.0248
M_52	GO:0090176	0.0248
M_52	GO:0060083	0.0248
M_52	GO:0035553	0.0248
M_52	GO:0015742	0.0248
M_52	GO:2001170	0.0248
M_52	GO:0050968	0.0248
M_52	GO:0097116	0.0248
M_52	GO:0022007	0.0248
M_52	GO:0021766	0.02739
M_52	GO:1900026	0.03077
M_52	GO:0030178	0.03078
M_52	GO:0043487	0.03248
M_52	GO:0006370	0.03252
M_52	GO:0032008	0.03252
M_52	GO:0060761	0.0328
M_52	GO:1901097	0.03292
M_52	GO:0061086	0.03292
M_52	GO:0039534	0.03292
M_52	GO:0070827	0.03292
M_52	GO:0031627	0.03292
M_52	GO:0051660	0.03292
M_52	GO:0043622	0.03292
M_52	GO:0048549	0.03292
M_52	GO:0000451	0.03292
M_52	GO:0001878	0.03292
M_52	GO:0046452	0.03292
M_52	GO:0015811	0.03292
M_52	GO:0097167	0.03292

M_52	GO:0015980	0.03296
M_52	GO:0070301	0.03311
M_52	GO:0007099	0.0343
M_52	GO:0014047	0.03612
M_52	GO:0035329	0.03798
M_52	GO:0034122	0.03798
M_52	GO:0033157	0.04018
M_52	GO:0071502	0.04098
M_52	GO:0048298	0.04098
M_52	GO:1902410	0.04098
M_52	GO:1900748	0.04098
M_52	GO:0021540	0.04098
M_52	GO:0044340	0.04098
M_52	GO:1904354	0.04098
M_52	GO:1900825	0.04098
M_52	GO:0034392	0.04098
M_52	GO:0002034	0.04098
M_52	GO:1904491	0.04098
M_52	GO:1990049	0.04098
M_52	GO:2000809	0.04098
M_52	GO:0051344	0.04098
M_52	GO:0072553	0.04098
M_52	GO:0075044	0.04098
M_52	GO:0021942	0.04098
M_52	GO:0042699	0.04098
M_52	GO:1901723	0.04098
M_52	GO:0071557	0.04098
M_52	GO:0045773	0.04181
M_52	GO:0043087	0.04462
M_52	GO:0045785	0.04566
M_52	GO:0038066	0.04841
M_52	GO:0007268	0.04855

M_52	GO:0035973	0.04898
M_52	GO:1900227	0.04898
M_52	GO:0016255	0.04898
M_52	GO:1902474	0.04898
M_52	GO:0051661	0.04898
M_52	GO:0090315	0.04898
M_52	GO:0042256	0.04898
M_52	GO:0045719	0.04898
M_52	GO:0006189	0.04898
M_52	GO:0050859	0.04898
M_52	GO:0010793	0.04898
M_52	GO:0010757	0.04898
M_52	GO:0048680	0.04898
M_52	GO:1900045	0.04898
M_52	GO:1901165	0.04898
M_52	GO:0046469	0.04898
M_52	GO:1902514	0.04898
M_52	GO:0043308	0.04898
M_52	GO:0060992	0.04898
M_52	GO:0009052	0.04898
M_52	GO:0010637	0.04898
M_52	GO:2000323	0.04898
M_53	GO:0060548	0.00036
M_53	GO:0007183	0.00094
M_53	GO:0006346	0.00248
M_53	GO:0045332	0.00297
M_53	GO:1903724	0.00354
M_53	GO:0006666	0.00354
M_53	GO:0003251	0.00354
M_53	GO:0061040	0.00354
M_53	GO:0030513	0.00571
M_53	GO:0072134	0.00707

M_53	GO:0034414	0.00707
M_53	GO:1904209	0.00707
M_53	GO:0003220	0.00707
M_53	GO:2001288	0.00707
M_53	GO:1900224	0.00707
M_53	GO:0010498	0.00829
M_53	GO:0042149	0.00925
M_53	GO:0010718	0.01014
M_53	GO:0007352	0.01058
M_53	GO:0035633	0.01058
M_53	GO:0001923	0.01058
M_53	GO:1904222	0.01058
M_53	GO:1904219	0.01058
M_53	GO:1903542	0.01058
M_53	GO:0070541	0.01058
M_53	GO:0006421	0.01058
M_53	GO:0030104	0.01391
M_53	GO:0032053	0.01408
M_53	GO:0072133	0.01408
M_53	GO:0003415	0.01408
M_53	GO:0032510	0.01408
M_53	GO:0018171	0.01408
M_53	GO:1902109	0.01408
M_53	GO:0015917	0.01408
M_53	GO:0070370	0.01408
M_53	GO:0033686	0.01408
M_53	GO:0007030	0.01445
M_53	GO:0060395	0.01736
M_53	GO:0002667	0.01757
M_53	GO:0021650	0.01757
M_53	GO:1902083	0.01757
M_53	GO:0030242	0.01757

M_53	GO:0060023	0.01757
M_53	GO:0090611	0.01757
M_53	GO:0070940	0.01757
M_53	GO:0061052	0.01757
M_53	GO:0035019	0.01853
M_53	GO:0060956	0.02105
M_53	GO:1903215	0.02105
M_53	GO:0046881	0.02105
M_53	GO:0007182	0.02105
M_53	GO:0032534	0.02105
M_53	GO:0048859	0.02105
M_53	GO:0045454	0.02351
M_53	GO:1902031	0.02452
M_53	GO:0003360	0.02452
M_53	GO:0036302	0.02452
M_53	GO:0003190	0.02452
M_53	GO:2000617	0.02452
M_53	GO:0048733	0.02452
M_53	GO:0051597	0.02452
M_53	GO:0006108	0.02797
M_53	GO:0032909	0.02797
M_53	GO:0010917	0.02797
M_53	GO:2000321	0.02797
M_53	GO:1901029	0.02797
M_53	GO:0015825	0.03141
M_53	GO:0048617	0.03141
M_53	GO:0034115	0.03141
M_53	GO:0017085	0.03141
M_53	GO:0042118	0.03484
M_53	GO:0048340	0.03484
M_53	GO:0032525	0.03484
M_53	GO:0046501	0.03484

M_53	GO:0031053	0.03484
M_53	GO:0003198	0.03826
M_53	GO:0019852	0.03826
M_53	GO:0072520	0.03826
M_53	GO:0060766	0.04166
M_53	GO:0060391	0.04166
M_53	GO:2000650	0.04166
M_53	GO:0043201	0.04166
M_53	GO:0035563	0.04166
M_53	GO:1902237	0.04166
M_53	GO:0010614	0.04436
M_53	GO:0006813	0.04489
M_53	GO:2000009	0.04506
M_53	GO:0034497	0.04506
M_53	GO:0045598	0.04719
M_53	GO:0007099	0.04773
M_53	GO:2000269	0.04844
M_53	GO:0043517	0.04844
M_53	GO:0010457	0.04844
M_53	GO:0042711	0.04844
M_54	GO:0006438	0.000053
M_54	GO:0006367	0.00056
M_54	GO:1902894	0.00113
M_54	GO:0001829	0.00178
M_54	GO:1904568	0.00422
M_54	GO:0070478	0.00422
M_54	GO:1901389	0.00422
M_54	GO:0070143	0.00422
M_54	GO:1900370	0.00422
M_54	GO:1990646	0.00422
M_54	GO:0006511	0.0048
M_54	GO:0008366	0.00837

M_54	GO:0071929	0.00843
M_54	GO:0015862	0.00843
M_54	GO:1904379	0.00843
M_54	GO:0048611	0.00843
M_54	GO:0097309	0.00843
M_54	GO:0035281	0.00843
M_54	GO:1901187	0.00843
M_54	GO:0090402	0.00843
M_54	GO:0014835	0.00843
M_54	GO:1903892	0.00843
M_54	GO:0060532	0.00843
M_54	GO:0072738	0.00843
M_54	GO:0061145	0.00843
M_54	GO:0006370	0.00905
M_54	GO:0007616	0.00957
M_54	GO:1904378	0.01262
M_54	GO:0035986	0.01262
M_54	GO:0071596	0.01262
M_54	GO:2001184	0.01262
M_54	GO:0032485	0.01262
M_54	GO:1903660	0.01262
M_54	GO:1900222	0.01262
M_54	GO:0001890	0.01263
M_54	GO:0000462	0.01298
M_54	GO:0018393	0.01647
M_54	GO:0035092	0.01657
M_54	GO:0035616	0.01679
M_54	GO:0046016	0.01679
M_54	GO:0010735	0.01679
M_54	GO:0003257	0.01679
M_54	GO:0036297	0.01821
M_54	GO:0051169	0.02027

M_54	GO:0045598	0.02091
M_54	GO:1903936	0.02094
M_54	GO:0071816	0.02094
M_54	GO:0043415	0.02094
M_54	GO:0071494	0.02094
M_54	GO:0070535	0.02094
M_54	GO:0043401	0.0234
M_54	GO:0009299	0.02477
M_54	GO:2000288	0.02508
M_54	GO:0055059	0.02508
M_54	GO:0002741	0.02508
M_54	GO:1901727	0.02508
M_54	GO:0022028	0.02508
M_54	GO:1900227	0.02508
M_54	GO:1902949	0.02508
M_54	GO:0032435	0.02912
M_54	GO:0010668	0.0292
M_54	GO:0097500	0.0292
M_54	GO:1903546	0.0292
M_54	GO:0010887	0.0292
M_54	GO:0006303	0.02998
M_54	GO:0014068	0.03085
M_54	GO:0032966	0.0333
M_54	GO:0060947	0.0333
M_54	GO:0070197	0.0333
M_54	GO:0006776	0.0333
M_54	GO:0035912	0.0333
M_54	GO:0035093	0.0333
M_54	GO:0090400	0.0333
M_54	GO:0060534	0.0333
M_54	GO:0060484	0.0333
M_54	GO:0075713	0.0333

M_54	GO:0035914	0.03352
M_54	GO:0006290	0.03739
M_54	GO:0043589	0.03739
M_54	GO:0006268	0.03739
M_54	GO:0071233	0.03739
M_54	GO:0090336	0.03739
M_54	GO:0042748	0.03739
M_54	GO:0060586	0.03739
M_54	GO:0031936	0.03739
M_54	GO:0001570	0.04007
M_54	GO:0042428	0.04146
M_54	GO:0071223	0.04146
M_54	GO:0000463	0.04146
M_54	GO:0098734	0.04146
M_54	GO:2000774	0.04146
M_54	GO:0019062	0.04146
M_54	GO:0016322	0.04146
M_54	GO:1900242	0.04146
M_54	GO:0042692	0.04462
M_54	GO:0030431	0.04497
M_54	GO:0001833	0.04551
M_54	GO:0034244	0.04551
M_54	GO:0045793	0.04551
M_54	GO:0090009	0.04551
M_54	GO:1990440	0.04551
M_54	GO:0033160	0.04954
M_54	GO:1901642	0.04954
M_54	GO:0014842	0.04954
M_54	GO:0045161	0.04954
M_54	GO:0010759	0.04954
M_54	GO:0045059	0.04954
M_54	GO:0033523	0.04954

M_55	GO:0007175	0.0031
M_55	GO:0034063	0.0036
M_55	GO:0045821	0.0052
M_55	GO:0042789	0.0058
M_55	GO:0033168	0.0061
M_55	GO:0043990	0.0061
M_55	GO:2000176	0.0061
M_55	GO:0006683	0.0061
M_55	GO:0021870	0.0061
M_55	GO:0002939	0.0061
M_55	GO:0035750	0.0061
M_55	GO:2001165	0.0061
M_55	GO:0071644	0.0061
M_55	GO:0072382	0.0061
M_55	GO:1903621	0.0061
M_55	GO:0042795	0.0088
M_55	GO:0050769	0.0098
M_55	GO:0006351	0.0102
M_55	GO:0034644	0.0118
M_55	GO:0019547	0.0121
M_55	GO:0009257	0.0121
M_55	GO:0070564	0.0121
M_55	GO:0032290	0.0121
M_55	GO:0000105	0.0121
M_55	GO:0014040	0.0121
M_55	GO:0042245	0.0121
M_55	GO:0051563	0.0121
M_55	GO:0021849	0.0121
M_55	GO:0034696	0.0121
M_55	GO:0016062	0.0121
M_55	GO:0071641	0.0121
M_55	GO:0048560	0.0121

M_55	GO:1904379	0.0121
M_55	GO:0002038	0.0121
M_55	GO:0046511	0.0121
M_55	GO:0006378	0.0157
M_55	GO:0071108	0.016
M_55	GO:0070536	0.016
M_55	GO:0000045	0.0163
M_55	GO:0043988	0.018
M_55	GO:1904100	0.018
M_55	GO:1990737	0.018
M_55	GO:0001922	0.018
M_55	GO:0002101	0.018
M_55	GO:0033512	0.018
M_55	GO:0002265	0.018
M_55	GO:0021502	0.018
M_55	GO:0071650	0.018
M_55	GO:0070901	0.018
M_55	GO:0070101	0.018
M_55	GO:0030961	0.018
M_55	GO:2000666	0.018
M_55	GO:0006210	0.018
M_55	GO:0035552	0.018
M_55	GO:0000350	0.018
M_55	GO:0045737	0.02
M_55	GO:0006369	0.0221
M_55	GO:0006355	0.0235
M_55	GO:0016246	0.0238
M_55	GO:0060574	0.024
M_55	GO:0006574	0.024
M_55	GO:0019346	0.024
M_55	GO:0006696	0.024
M_55	GO:0016240	0.024

M_55	GO:0061030	0.024
M_55	GO:0032057	0.024
M_55	GO:0006499	0.024
M_55	GO:0016080	0.024
M_55	GO:0031048	0.024
M_55	GO:0030423	0.024
M_55	GO:2000562	0.024
M_55	GO:0035087	0.024
M_55	GO:0050893	0.024
M_55	GO:0018343	0.024
M_55	GO:2000676	0.024
M_55	GO:0000301	0.024
M_55	GO:0000724	0.0241
M_55	GO:0035735	0.0244
M_55	GO:0048538	0.0291
M_55	GO:0034205	0.0296
M_55	GO:1903377	0.0299
M_55	GO:1902412	0.0299
M_55	GO:0010626	0.0299
M_55	GO:0090235	0.0299
M_55	GO:0051541	0.0299
M_55	GO:0006398	0.0299
M_55	GO:0044828	0.0299
M_55	GO:0010991	0.0299
M_55	GO:0097411	0.0299
M_55	GO:1904504	0.0299
M_55	GO:0061419	0.0299
M_55	GO:0008333	0.0329
M_55	GO:0006446	0.0337
M_55	GO:0000480	0.0358
M_55	GO:0017196	0.0358
M_55	GO:0043987	0.0358

M_55	GO:0006572	0.0358
M_55	GO:0006686	0.0358
M_55	GO:0042796	0.0358
M_55	GO:0048205	0.0358
M_55	GO:1990535	0.0358
M_55	GO:0002248	0.0358
M_55	GO:0006104	0.0358
M_55	GO:1900425	0.0358
M_55	GO:0008104	0.0381
M_55	GO:0001755	0.0395
M_55	GO:0007131	0.0409
M_55	GO:0060075	0.0416
M_55	GO:1904468	0.0416
M_55	GO:0035333	0.0416
M_55	GO:0032287	0.0416
M_55	GO:0035280	0.0416
M_55	GO:0048702	0.0416
M_55	GO:1903599	0.0416
M_55	GO:0007144	0.0416
M_55	GO:1903546	0.0416
M_55	GO:0007256	0.0416
M_55	GO:0046886	0.0416
M_55	GO:0090647	0.0416
M_55	GO:0032364	0.0416
M_55	GO:0046543	0.0416
M_55	GO:0031122	0.0452
M_55	GO:0033129	0.0474
M_55	GO:0010510	0.0474
M_55	GO:0017185	0.0474
M_55	GO:0032909	0.0474
M_55	GO:0060693	0.0474
M_55	GO:0042148	0.0474

M_55	GO:0034720	0.0474
M_55	GO:0000707	0.0474
M_55	GO:1903715	0.0474
M_55	GO:0032825	0.0474
M_55	GO:0015871	0.0474
M_55	GO:1900364	0.0474
M_55	GO:0070244	0.0474
M_55	GO:0019896	0.0474
M_55	GO:0001947	0.0496
M_56	GO:0000082	0.000000026
M_56	GO:0006270	0.000000084
M_56	GO:0006268	0.0000053
M_56	GO:0035176	0.000062
M_56	GO:0032201	0.00011
M_56	GO:0051383	0.00016
M_56	GO:0036444	0.00071
M_56	GO:0051561	0.00071
M_56	GO:0051301	0.00096
M_56	GO:0090110	0.00143
M_56	GO:2000773	0.00187
M_56	GO:0006622	0.00265
M_56	GO:0031145	0.00287
M_56	GO:0006346	0.00324
M_56	GO:0072431	0.00401
M_56	GO:0032298	0.00405
M_56	GO:0009992	0.00405
M_56	GO:0035945	0.00405
M_56	GO:0035946	0.00405
M_56	GO:1902426	0.00405
M_56	GO:2000655	0.00405
M_56	GO:0006343	0.00405
M_56	GO:0010849	0.00405

M_56	GO:0071441	0.00405
M_56	GO:2001229	0.00405
M_56	GO:0006169	0.00405
M_56	GO:0006175	0.00405
M_56	GO:0044209	0.00809
M_56	GO:2000276	0.00809
M_56	GO:0031393	0.00809
M_56	GO:0097681	0.00809
M_56	GO:1902595	0.00809
M_56	GO:0000962	0.00809
M_56	GO:0071344	0.00809
M_56	GO:2001178	0.00809
M_56	GO:0042595	0.00809
M_56	GO:1990619	0.00809
M_56	GO:1902975	0.00809
M_56	GO:0007613	0.01113
M_56	GO:2001243	0.01119
M_56	GO:0031648	0.01144
M_56	GO:2000111	0.01211
M_56	GO:0031508	0.01211
M_56	GO:0000958	0.01211
M_56	GO:0000720	0.01211
M_56	GO:0051097	0.01211
M_56	GO:0060392	0.01211
M_56	GO:0006391	0.01211
M_56	GO:1902445	0.01211
M_56	GO:2000619	0.01211
M_56	GO:0071163	0.01211
M_56	GO:0010501	0.01494
M_56	GO:0032508	0.01494
M_56	GO:0002326	0.01611
M_56	GO:0033152	0.01611

M_56	GO:0043137	0.01611
M_56	GO:0070827	0.01611
M_56	GO:0006344	0.01611
M_56	GO:0006642	0.01611
M_56	GO:1900113	0.01611
M_56	GO:0006914	0.01933
M_56	GO:0030174	0.01982
M_56	GO:0006269	0.0201
M_56	GO:0033153	0.0201
M_56	GO:1904179	0.0201
M_56	GO:0010216	0.0201
M_56	GO:0000727	0.0201
M_56	GO:0072708	0.0201
M_56	GO:0010032	0.0201
M_56	GO:2000481	0.0201
M_56	GO:0001915	0.02407
M_56	GO:0045348	0.02407
M_56	GO:0090324	0.02407
M_56	GO:0000965	0.02407
M_56	GO:0035234	0.02407
M_56	GO:0003400	0.02407
M_56	GO:0006686	0.02407
M_56	GO:2000348	0.02407
M_56	GO:0006729	0.02803
M_56	GO:0007084	0.02803
M_56	GO:0034983	0.02803
M_56	GO:1901897	0.02803
M_56	GO:0035356	0.02803
M_56	GO:0006625	0.03106
M_56	GO:0030512	0.03191
M_56	GO:0030210	0.03197
M_56	GO:0032071	0.03197

M_56	GO:0051152	0.03197
M_56	GO:0090400	0.03197
M_56	GO:0031937	0.03197
M_56	GO:2000480	0.03197
M_56	GO:0006271	0.03542
M_56	GO:0033210	0.0359
M_56	GO:0001542	0.0359
M_56	GO:0045876	0.0359
M_56	GO:2000105	0.03926
M_56	GO:0002638	0.03981
M_56	GO:2000774	0.03981
M_56	GO:0001553	0.03981
M_56	GO:0000012	0.03981
M_56	GO:0070857	0.03981
M_56	GO:0006307	0.03981
M_56	GO:0051574	0.03981
M_56	GO:1902916	0.03981
M_56	GO:0002328	0.0437
M_56	GO:0006559	0.0437
M_56	GO:0060394	0.0437
M_56	GO:0031987	0.0437
M_56	GO:0033327	0.0437
M_56	GO:0070301	0.04562
M_56	GO:0001678	0.04725
M_56	GO:0050872	0.04758
M_56	GO:0060766	0.04758
M_56	GO:0070914	0.04758
M_56	GO:0035563	0.04758
M_56	GO:1902237	0.04758
M_56	GO:0051259	0.04892
M_56	GO:0070125	0.04958
M_57	GO:0045724	0.0034

M_57	GO:0042247	0.0042
M_57	GO:0015904	0.0042
M_57	GO:0030209	0.0042
M_57	GO:1903615	0.0042
M_57	GO:0042407	0.0078
M_57	GO:2000818	0.0083
M_57	GO:0048632	0.0083
M_57	GO:0071955	0.0083
M_57	GO:1900248	0.0083
M_57	GO:0003290	0.0083
M_57	GO:1903758	0.0083
M_57	GO:0051066	0.0083
M_57	GO:0048822	0.0083
M_57	GO:0000472	0.0124
M_57	GO:0090234	0.0124
M_57	GO:0060819	0.0124
M_57	GO:1900222	0.0124
M_57	GO:2000182	0.0124
M_57	GO:0072318	0.0124
M_57	GO:1902723	0.0124
M_57	GO:1902725	0.0124
M_57	GO:0031587	0.0124
M_57	GO:0006357	0.0138
M_57	GO:0030043	0.0166
M_57	GO:0042904	0.0166
M_57	GO:0010916	0.0166
M_57	GO:0006398	0.0207
M_57	GO:0048298	0.0207
M_57	GO:0016191	0.0207
M_57	GO:1903142	0.0207
M_57	GO:0090241	0.0207
M_57	GO:0010044	0.0207

M_57	GO:0003289	0.0207
M_57	GO:1904491	0.0207
M_57	GO:1904504	0.0207
M_57	GO:0050428	0.0247
M_57	GO:0000480	0.0247
M_57	GO:1900223	0.0247
M_57	GO:2000064	0.0247
M_57	GO:0044770	0.0251
M_57	GO:0008277	0.0287
M_57	GO:0050861	0.0288
M_57	GO:0042297	0.0288
M_57	GO:0006729	0.0288
M_57	GO:1903599	0.0288
M_57	GO:0090160	0.0329
M_57	GO:0023035	0.0329
M_57	GO:1903012	0.0329
M_57	GO:0014732	0.0329
M_57	GO:0006013	0.0329
M_57	GO:0085020	0.0329
M_57	GO:0071578	0.0329
M_57	GO:0090385	0.0329
M_57	GO:0006355	0.0346
M_57	GO:0000447	0.0369
M_57	GO:0036353	0.0369
M_57	GO:0051151	0.0369
M_57	GO:0060539	0.0369
M_57	GO:0002091	0.0369
M_57	GO:0034770	0.0409
M_57	GO:0010452	0.0409
M_57	GO:0009629	0.0409
M_57	GO:0003149	0.0409
M_57	GO:0034214	0.0409

M_57	GO:0006559	0.0449
M_57	GO:0030836	0.0449
M_57	GO:0005984	0.0489
M_57	GO:0019532	0.0489
M_57	GO:0002903	0.0489
M_57	GO:0035751	0.0489
M_57	GO:0070493	0.0489
M_57	GO:0050872	0.0489
M_57	GO:1990403	0.0489
M_57	GO:0035437	0.0489
M_58	GO:0009440	0.000028
M_58	GO:0000098	0.000041
M_58	GO:0045540	0.000043
M_58	GO:0075525	0.00028
M_58	GO:0045040	0.00042
M_58	GO:0010867	0.00182
M_58	GO:1902894	0.00182
M_58	GO:0032922	0.00321
M_58	GO:0071404	0.00412
M_58	GO:0042789	0.00459
M_58	GO:0030220	0.00459
M_58	GO:0071963	0.00537
M_58	GO:0043969	0.00537
M_58	GO:0072255	0.00537
M_58	GO:0036316	0.00537
M_58	GO:1901303	0.00537
M_58	GO:0035352	0.00537
M_58	GO:1903919	0.00537
M_58	GO:0016132	0.00537
M_58	GO:0070903	0.00537
M_58	GO:0061395	0.00537
M_58	GO:0072363	0.00537

M_58	GO:0097117	0.00537
M_58	GO:0033488	0.00537
M_58	GO:0006695	0.00605
M_58	GO:0042632	0.00683
M_58	GO:0060271	0.00739
M_58	GO:0010512	0.0107
M_58	GO:0035350	0.0107
M_58	GO:1903966	0.0107
M_58	GO:1903979	0.0107
M_58	GO:1903923	0.0107
M_58	GO:0035349	0.0107
M_58	GO:0070676	0.0107
M_58	GO:0032796	0.0107
M_58	GO:0031022	0.0107
M_58	GO:0071506	0.0107
M_58	GO:0080121	0.0107
M_58	GO:0072368	0.0107
M_58	GO:0072369	0.0107
M_58	GO:0001732	0.0107
M_58	GO:1903146	0.01124
M_58	GO:0045444	0.01169
M_58	GO:0030324	0.01225
M_58	GO:0032148	0.01273
M_58	GO:0090630	0.01293
M_58	GO:0006687	0.01484
M_58	GO:0070814	0.01601
M_58	GO:1904117	0.01601
M_58	GO:0090118	0.01601
M_58	GO:0044208	0.01601
M_58	GO:1990737	0.01601
M_58	GO:0090292	0.01601
M_58	GO:0051835	0.01601

M_58	GO:0097680	0.01601
M_58	GO:0021773	0.01601
M_58	GO:0035928	0.01601
M_58	GO:0002191	0.01601
M_58	GO:0021817	0.01601
M_58	GO:0035793	0.01601
M_58	GO:0006990	0.01601
M_58	GO:0010796	0.01601
M_58	GO:0010899	0.01601
M_58	GO:0023052	0.01888
M_58	GO:0090370	0.02129
M_58	GO:0032483	0.02129
M_58	GO:0019518	0.02129
M_58	GO:0070836	0.02129
M_58	GO:0009298	0.02129
M_58	GO:0019346	0.02129
M_58	GO:0061084	0.02129
M_58	GO:0018076	0.02129
M_58	GO:0003104	0.02129
M_58	GO:0032057	0.02129
M_58	GO:0002143	0.02129
M_58	GO:0010986	0.02129
M_58	GO:0097113	0.02129
M_58	GO:0097114	0.02129
M_58	GO:0033490	0.02129
M_58	GO:0010735	0.02129
M_58	GO:1900127	0.02129
M_58	GO:0033489	0.02129
M_58	GO:0071475	0.02129
M_58	GO:0006886	0.02267
M_58	GO:0016052	0.02648
M_58	GO:0045542	0.02654

M_58	GO:0051295	0.02654
M_58	GO:0072126	0.02654
M_58	GO:0006844	0.02654
M_58	GO:2000969	0.02654
M_58	GO:0048170	0.02654
M_58	GO:0045794	0.02654
M_58	GO:0036091	0.02654
M_58	GO:2000582	0.02654
M_58	GO:0006102	0.02654
M_58	GO:0010891	0.02654
M_58	GO:0045815	0.02953
M_58	GO:0045652	0.03063
M_58	GO:0010742	0.03149
M_58	GO:0010871	0.03177
M_58	GO:1900223	0.03177
M_58	GO:0046958	0.03177
M_58	GO:0035900	0.03177
M_58	GO:0048205	0.03177
M_58	GO:0038001	0.03177
M_58	GO:0006189	0.03177
M_58	GO:0046826	0.03177
M_58	GO:0003062	0.03177
M_58	GO:0051415	0.03177
M_58	GO:0035176	0.03288
M_58	GO:0071398	0.03288
M_58	GO:0006635	0.03457
M_58	GO:0010886	0.03697
M_58	GO:0010887	0.03697
M_58	GO:1902416	0.03697
M_58	GO:0042297	0.03697
M_58	GO:0046477	0.03697
M_58	GO:0006657	0.03697

M_58	GO:0038203	0.03697
M_58	GO:0097107	0.03697
M_58	GO:0090650	0.03697
M_58	GO:0071499	0.03697
M_58	GO:0007623	0.03709
M_58	GO:0051321	0.04126
M_58	GO:0000492	0.04214
M_58	GO:0035655	0.04214
M_58	GO:0001561	0.04214
M_58	GO:0006013	0.04214
M_58	GO:0048227	0.04214
M_58	GO:0075713	0.04214
M_58	GO:0007172	0.04214
M_58	GO:0072366	0.04214
M_58	GO:0035095	0.04214
M_58	GO:0001666	0.04364
M_58	GO:0032481	0.04503
M_58	GO:0009749	0.04603
M_58	GO:0007507	0.04614
M_58	GO:0002223	0.04633
M_58	GO:0048678	0.04633
M_58	GO:0061179	0.04728
M_58	GO:0070508	0.04728
M_58	GO:0060363	0.04728
M_58	GO:0090286	0.04728
M_58	GO:0032000	0.04728
M_58	GO:0015866	0.04728
M_58	GO:0090043	0.04728
M_58	GO:0034382	0.04728
M_58	GO:0014068	0.04763
M_58	GO:0090181	0.04979
M_59	GO:0050992	0.000032

M_59	GO:0009240	0.00032
M_59	GO:2000020	0.00088
M_59	GO:1903045	0.00571
M_59	GO:2000570	0.00571
M_59	GO:0033342	0.00571
M_59	GO:0003148	0.00888
M_59	GO:0048843	0.00888
M_59	GO:0043547	0.00928
M_59	GO:1903375	0.01138
M_59	GO:0016260	0.01138
M_59	GO:0019303	0.01138
M_59	GO:0051083	0.01138
M_59	GO:0071894	0.01138
M_59	GO:0070329	0.01138
M_59	GO:0021828	0.01138
M_59	GO:0071048	0.01138
M_59	GO:0018008	0.01138
M_59	GO:0007147	0.01138
M_59	GO:1902595	0.01138
M_59	GO:0035526	0.01138
M_59	GO:0051056	0.01539
M_59	GO:0019222	0.01545
M_59	GO:0030488	0.01607
M_59	GO:0030501	0.01698
M_59	GO:0035617	0.01703
M_59	GO:0036486	0.01703
M_59	GO:1903292	0.01703
M_59	GO:0006362	0.01703
M_59	GO:0051684	0.01703
M_59	GO:0051612	0.01703
M_59	GO:0003221	0.01703
M_59	GO:0070447	0.01703

M_59	GO:2000813	0.01703
M_59	GO:0010710	0.01703
M_59	GO:0000350	0.01703
M_59	GO:0070317	0.01984
M_59	GO:0050919	0.01984
M_59	GO:0055007	0.02061
M_59	GO:0042059	0.02084
M_59	GO:1904447	0.02264
M_59	GO:0021943	0.02264
M_59	GO:0061551	0.02264
M_59	GO:0001920	0.02264
M_59	GO:0097490	0.02264
M_59	GO:0097491	0.02264
M_59	GO:0007161	0.02264
M_59	GO:0060282	0.02264
M_59	GO:0007258	0.02264
M_59	GO:0033499	0.02264
M_59	GO:0072429	0.02264
M_59	GO:0061024	0.02355
M_59	GO:0009056	0.02694
M_59	GO:0008154	0.02798
M_59	GO:0035694	0.02822
M_59	GO:0030091	0.02822
M_59	GO:0060666	0.02822
M_59	GO:2000195	0.02822
M_59	GO:0061734	0.02822
M_59	GO:0006682	0.02822
M_59	GO:0060385	0.02822
M_59	GO:1990049	0.02822
M_59	GO:0046940	0.02822
M_59	GO:2000821	0.02822
M_59	GO:0048880	0.02822

M_59	GO:0021637	0.02822
M_59	GO:0030242	0.02822
M_59	GO:0098735	0.02822
M_59	GO:0045599	0.02837
M_59	GO:0010467	0.02919
M_59	GO:0071560	0.02952
M_59	GO:0043124	0.02952
M_59	GO:0061098	0.03309
M_59	GO:0051058	0.03353
M_59	GO:0009191	0.03377
M_59	GO:0061156	0.03377
M_59	GO:1904417	0.03377
M_59	GO:1903551	0.03377
M_59	GO:0071321	0.03377
M_59	GO:1903078	0.03556
M_59	GO:0071320	0.03556
M_59	GO:0002040	0.03887
M_59	GO:0016101	0.03909
M_59	GO:0007506	0.03929
M_59	GO:0021785	0.03929
M_59	GO:0036123	0.03929
M_59	GO:1990048	0.03929
M_59	GO:1902231	0.03929
M_59	GO:0044557	0.03929
M_59	GO:0031848	0.03929
M_59	GO:0021612	0.03929
M_59	GO:0072383	0.03929
M_59	GO:0007256	0.03929
M_59	GO:0006616	0.04477
M_59	GO:2000252	0.04477
M_59	GO:0048227	0.04477
M_59	GO:0032825	0.04477

M_59	GO:0051683	0.04477
M_59	GO:0003223	0.04477
M_59	GO:0006450	0.04477
M_59	GO:2000096	0.04477
M_59	GO:0090045	0.04477
M_59	GO:0048541	0.04477
M_59	GO:0071313	0.04477
M_59	GO:0031340	0.04477
M_59	GO:0000082	0.04928
M_60	GO:0031119	0.00011
M_60	GO:0006833	0.00037
M_60	GO:0007588	0.00048
M_60	GO:0032943	0.00226
M_60	GO:1903597	0.00228
M_60	GO:0015706	0.00228
M_60	GO:0035665	0.00228
M_60	GO:0032738	0.00228
M_60	GO:0018279	0.00357
M_60	GO:0034220	0.00445
M_60	GO:2000340	0.00456
M_60	GO:0071288	0.00456
M_60	GO:2000170	0.00456
M_60	GO:0036245	0.00456
M_60	GO:1990173	0.00456
M_60	GO:0043686	0.00683
M_60	GO:0002019	0.00683
M_60	GO:0042631	0.00683
M_60	GO:1990785	0.00683
M_60	GO:1990502	0.00683
M_60	GO:0006574	0.0091
M_60	GO:1990743	0.0091
M_60	GO:1990481	0.0091

M_60	GO:0015670	0.0091
M_60	GO:1900086	0.01137
M_60	GO:0034137	0.01137
M_60	GO:0071221	0.01362
M_60	GO:0015793	0.01362
M_60	GO:0003097	0.01362
M_60	GO:1902033	0.01362
M_60	GO:0060047	0.01568
M_60	GO:2000343	0.01588
M_60	GO:0060373	0.01588
M_60	GO:0000160	0.01588
M_60	GO:0045410	0.01588
M_60	GO:0034141	0.01588
M_60	GO:0097151	0.01588
M_60	GO:0060371	0.01812
M_60	GO:0072205	0.01812
M_60	GO:0071838	0.01812
M_60	GO:0072161	0.01812
M_60	GO:0051151	0.02037
M_60	GO:0090073	0.02037
M_60	GO:0042447	0.02037
M_60	GO:0086015	0.02037
M_60	GO:0071223	0.0226
M_60	GO:0034145	0.0226
M_60	GO:0000290	0.0226
M_60	GO:0046541	0.0226
M_60	GO:0030259	0.0226
M_60	GO:0002003	0.02484
M_60	GO:0070268	0.02698
M_60	GO:0003084	0.02929
M_60	GO:0030157	0.02929
M_60	GO:0044130	0.02929

M_60	GO:0042476	0.03302
M_60	GO:0070935	0.04033
M_60	GO:0007250	0.04033
M_60	GO:0086014	0.04033
M_60	GO:0023014	0.04137
M_60	GO:1900273	0.04252
M_60	GO:0009312	0.04252
M_60	GO:0014910	0.04367
M_60	GO:0014911	0.0469
M_60	GO:0086012	0.04907
M_61	GO:2000234	0.000012
M_61	GO:0006283	0.000041
M_61	GO:0006294	0.00046
M_61	GO:0006363	0.00057
M_61	GO:0006370	0.00075
M_61	GO:0006610	0.00075
M_61	GO:0006361	0.00088
M_61	GO:0061179	0.00096
M_61	GO:0006622	0.0044
M_61	GO:0015936	0.00487
M_61	GO:2000208	0.00525
M_61	GO:0031651	0.00525
M_61	GO:0071878	0.00525
M_61	GO:0070963	0.00525
M_61	GO:0070060	0.00525
M_61	GO:0090198	0.00525
M_61	GO:0045175	0.00525
M_61	GO:0000735	0.00525
M_61	GO:1990603	0.00525
M_61	GO:0061011	0.00525
M_61	GO:0001570	0.0072
M_61	GO:0006359	0.00879

M_61	GO:0050821	0.01005
M_61	GO:0019677	0.01048
M_61	GO:0043311	0.01048
M_61	GO:0090327	0.01048
M_61	GO:1903087	0.01048
M_61	GO:0007206	0.01048
M_61	GO:1900135	0.01048
M_61	GO:1903646	0.01048
M_61	GO:0061017	0.01048
M_61	GO:0006368	0.01048
M_61	GO:0070126	0.01185
M_61	GO:0000460	0.0145
M_61	GO:0010165	0.0145
M_61	GO:1990166	0.01567
M_61	GO:0010621	0.01567
M_61	GO:0070433	0.01567
M_61	GO:0006742	0.01567
M_61	GO:0051138	0.01567
M_61	GO:0034140	0.01567
M_61	GO:0061010	0.01567
M_61	GO:0006296	0.01613
M_61	GO:0061028	0.01782
M_61	GO:0000413	0.01869
M_61	GO:1902809	0.02084
M_61	GO:0031427	0.02084
M_61	GO:0000710	0.02084
M_61	GO:0007023	0.02084
M_61	GO:0070898	0.02084
M_61	GO:0003104	0.02084
M_61	GO:0046452	0.02084
M_61	GO:0097029	0.02084
M_61	GO:0031048	0.02084

M_61	GO:0035621	0.02084
M_61	GO:0043570	0.02599
M_61	GO:0035926	0.02599
M_61	GO:0034184	0.02599
M_61	GO:0034137	0.02599
M_61	GO:0060431	0.02599
M_61	GO:0051351	0.03083
M_61	GO:0071103	0.03085
M_61	GO:0006824	0.0311
M_61	GO:0043111	0.0311
M_61	GO:0006552	0.0311
M_61	GO:0051096	0.0311
M_61	GO:0002741	0.0311
M_61	GO:0071638	0.0311
M_61	GO:0060613	0.0311
M_61	GO:0048007	0.0311
M_61	GO:0022027	0.0311
M_61	GO:0070358	0.0311
M_61	GO:0019367	0.03619
M_61	GO:0006768	0.03619
M_61	GO:0006729	0.03619
M_61	GO:0034625	0.03619
M_61	GO:0034626	0.03619
M_61	GO:0007021	0.03619
M_61	GO:0034141	0.03619
M_61	GO:0010764	0.03619
M_61	GO:0072593	0.0377
M_61	GO:0009411	0.03802
M_61	GO:0072608	0.04126
M_61	GO:0000492	0.04126
M_61	GO:0032790	0.04126
M_61	GO:0046654	0.04126

M_61	GO:0075713	0.04126
M_61	GO:0032981	0.04457
M_61	GO:0060100	0.04629
M_61	GO:1901409	0.04629
M_61	GO:0010944	0.04629
M_61	GO:0042119	0.04708
M_61	GO:0006367	0.04717
M_62	GO:0006357	1.8E-18
M_62	GO:0044839	0.00005
M_62	GO:0070106	0.0027
M_62	GO:0033127	0.0039
M_62	GO:0007076	0.0059
M_62	GO:1903408	0.0072
M_62	GO:0071245	0.0072
M_62	GO:0010424	0.0072
M_62	GO:0015700	0.0072
M_62	GO:0048698	0.0072
M_62	GO:0031065	0.0074
M_62	GO:0034970	0.0144
M_62	GO:0034971	0.0144
M_62	GO:0001579	0.0144
M_62	GO:0070563	0.0144
M_62	GO:0008612	0.0144
M_62	GO:0003420	0.0144
M_62	GO:0035407	0.0144
M_62	GO:0002940	0.0144
M_62	GO:0046203	0.0144
M_62	GO:1903526	0.0144
M_62	GO:0000083	0.0174
M_62	GO:0010020	0.0216
M_62	GO:0018125	0.0216
M_62	GO:0016185	0.0216

M_62	GO:0090116	0.0216
M_62	GO:1902856	0.0216
M_62	GO:1902661	0.0216
M_62	GO:0006656	0.0238
M_62	GO:1902807	0.0284
M_62	GO:0090309	0.0287
M_62	GO:0043137	0.0287
M_62	GO:0006659	0.0287
M_62	GO:0071072	0.0287
M_62	GO:0051697	0.0287
M_62	GO:0050821	0.0292
M_62	GO:0009994	0.0355
M_62	GO:2000370	0.0357
M_62	GO:0019509	0.0357
M_62	GO:0033211	0.0357
M_62	GO:1903142	0.0357
M_62	GO:0071816	0.0357
M_62	GO:0006432	0.0357
M_62	GO:0010216	0.0357
M_62	GO:0075525	0.0357
M_62	GO:0000086	0.0399
M_62	GO:0008063	0.0427
M_62	GO:0010571	0.0427
M_62	GO:0009838	0.0427
M_62	GO:0010793	0.0427
M_62	GO:1901621	0.0427
M_62	GO:0035385	0.0497
M_62	GO:0048102	0.0497
M_62	GO:0023041	0.0497
M_62	GO:0032049	0.0497
M_62	GO:0031658	0.0497
M_62	GO:0044351	0.0497

M_63	GO:0051598	0.000048
M_63	GO:0007076	0.00093
M_63	GO:0045589	0.00248
M_63	GO:2001173	0.00285
M_63	GO:2001246	0.00285
M_63	GO:1904042	0.00285
M_63	GO:0046318	0.00285
M_63	GO:0070550	0.00285
M_63	GO:1903109	0.00285
M_63	GO:0046690	0.00285
M_63	GO:0009645	0.00285
M_63	GO:0009061	0.00285
M_63	GO:0006099	0.00375
M_63	GO:0032008	0.00422
M_63	GO:1901998	0.00526
M_63	GO:0071050	0.0057
M_63	GO:0090164	0.0057
M_63	GO:0035261	0.0057
M_63	GO:0071036	0.0057
M_63	GO:0071037	0.0057
M_63	GO:0010142	0.0057
M_63	GO:1903285	0.0057
M_63	GO:0009631	0.0057
M_63	GO:0043630	0.0057
M_63	GO:0033566	0.0057
M_63	GO:0036324	0.0057
M_63	GO:0030505	0.0057
M_63	GO:1903775	0.0057
M_63	GO:0034087	0.0057
M_63	GO:0032007	0.00795
M_63	GO:0061458	0.00822
M_63	GO:1990108	0.00854

M_63	GO:0006435	0.00854
M_63	GO:0043418	0.00854
M_63	GO:0061010	0.00854
M_63	GO:0006740	0.00854
M_63	GO:0010332	0.01114
M_63	GO:0044528	0.01137
M_63	GO:2001274	0.01137
M_63	GO:1902527	0.01137
M_63	GO:0003431	0.01137
M_63	GO:0071733	0.01137
M_63	GO:0033353	0.01137
M_63	GO:0006121	0.01137
M_63	GO:1990414	0.01137
M_63	GO:0006302	0.01234
M_63	GO:0033313	0.01363
M_63	GO:1901563	0.01419
M_63	GO:0045542	0.01419
M_63	GO:1904491	0.01419
M_63	GO:0045053	0.01419
M_63	GO:1904428	0.01419
M_63	GO:0032354	0.01668
M_63	GO:0042796	0.017
M_63	GO:0071042	0.017
M_63	GO:2000348	0.017
M_63	GO:0060992	0.017
M_63	GO:0007185	0.017
M_63	GO:0071921	0.017
M_63	GO:0061038	0.017
M_63	GO:0010825	0.017
M_63	GO:0032088	0.01892
M_63	GO:0071051	0.01981
M_63	GO:0071038	0.01981

M_63	GO:0007144	0.01981
M_63	GO:0060352	0.01981
M_63	GO:0032364	0.01981
M_63	GO:0001938	0.02088
M_63	GO:0072358	0.02204
M_63	GO:0071169	0.02261
M_63	GO:0042747	0.02261
M_63	GO:0071035	0.02261
M_63	GO:0030422	0.02261
M_63	GO:0006919	0.02505
M_63	GO:2000628	0.0254
M_63	GO:0051601	0.0254
M_63	GO:0071394	0.0254
M_63	GO:0010918	0.0254
M_63	GO:0070125	0.02615
M_63	GO:0070126	0.02726
M_63	GO:0030948	0.02818
M_63	GO:0031053	0.02818
M_63	GO:0043129	0.02818
M_63	GO:0042428	0.02818
M_63	GO:0034088	0.02818
M_63	GO:0045945	0.03096
M_63	GO:0070431	0.03096
M_63	GO:0048703	0.03096
M_63	GO:0070131	0.03096
M_63	GO:0071372	0.03096
M_63	GO:0016075	0.03308
M_63	GO:0035404	0.03373
M_63	GO:0030950	0.03373
M_63	GO:0071044	0.03373
M_63	GO:0014029	0.03373
M_63	GO:0006105	0.03373

M_63	GO:0033197	0.03373
M_63	GO:0055064	0.03649
M_63	GO:0071872	0.03649
M_63	GO:0071397	0.03649
M_63	GO:0009086	0.03649
M_63	GO:0071481	0.03649
M_63	GO:0048194	0.03924
M_63	GO:2001224	0.03924
M_63	GO:0031054	0.03924
M_63	GO:0055089	0.03924
M_63	GO:0071361	0.03924
M_63	GO:1904380	0.03924
M_63	GO:0021794	0.03924
M_63	GO:0032688	0.03924
M_63	GO:0035435	0.03924
M_63	GO:0006915	0.03997
M_63	GO:0006702	0.04198
M_63	GO:0031000	0.04198
M_63	GO:0033540	0.04198
M_63	GO:0019827	0.04252
M_63	GO:0050727	0.04447
M_63	GO:0048820	0.04472
M_63	GO:0009792	0.04707
M_63	GO:0042448	0.04745
M_63	GO:0071380	0.04745
M_64	GO:0009249	0.00052
M_64	GO:0032259	0.00216
M_64	GO:1990845	0.0022
M_64	GO:0051497	0.00344
M_64	GO:0030037	0.0044
M_64	GO:1902846	0.0044
M_64	GO:0055048	0.0044

M_64	GO:1903765	0.0044
M_64	GO:1902365	0.0044
M_64	GO:0006580	0.0044
M_64	GO:0071602	0.0044
M_64	GO:2000454	0.0044
M_64	GO:0031116	0.00536
M_64	GO:0034067	0.00579
M_64	GO:0006890	0.00643
M_64	GO:0000303	0.00669
M_64	GO:0021997	0.00877
M_64	GO:2000376	0.00877
M_64	GO:0006891	0.0115
M_64	GO:0051684	0.01313
M_64	GO:0044778	0.01313
M_64	GO:0048388	0.01313
M_64	GO:0046882	0.01313
M_64	GO:0097680	0.01313
M_64	GO:0018125	0.01313
M_64	GO:0071400	0.01313
M_64	GO:0006421	0.01313
M_64	GO:0061073	0.01313
M_64	GO:0031424	0.01703
M_64	GO:0045578	0.01747
M_64	GO:0060244	0.01747
M_64	GO:0070236	0.01747
M_64	GO:0006490	0.01747
M_64	GO:0045077	0.01747
M_64	GO:0072385	0.01747
M_64	GO:0009409	0.01962
M_64	GO:0032868	0.02136
M_64	GO:0010866	0.02152
M_64	GO:0060117	0.02152

M_64	GO:0021960	0.02179
M_64	GO:0072014	0.02179
M_64	GO:1990511	0.02179
M_64	GO:0090235	0.02179
M_64	GO:0061052	0.02179
M_64	GO:0021540	0.02179
M_64	GO:0097527	0.02609
M_64	GO:0046881	0.02609
M_64	GO:0060613	0.02609
M_64	GO:0006689	0.02609
M_64	GO:0034773	0.02609
M_64	GO:0030953	0.03037
M_64	GO:0061511	0.03037
M_64	GO:0045650	0.03037
M_64	GO:0036462	0.03037
M_64	GO:0032532	0.03037
M_64	GO:1901098	0.03037
M_64	GO:0006657	0.03037
M_64	GO:0050957	0.03037
M_64	GO:0097049	0.03037
M_64	GO:1902260	0.03037
M_64	GO:0035356	0.03037
M_64	GO:0050896	0.03294
M_64	GO:1990542	0.03428
M_64	GO:0048563	0.03463
M_64	GO:0006853	0.03463
M_64	GO:0036155	0.03463
M_64	GO:0046322	0.03463
M_64	GO:0042424	0.03888
M_64	GO:0019695	0.03888
M_64	GO:0034498	0.03888
M_64	GO:0071550	0.03888

M_64	GO:0016584	0.03888
M_64	GO:0061179	0.03888
M_64	GO:0042541	0.03888
M_64	GO:0032482	0.04305
M_64	GO:0021670	0.0431
M_64	GO:0071985	0.0431
M_64	GO:1901387	0.0431
M_64	GO:0006930	0.0431
M_64	GO:0033148	0.0431
M_64	GO:0046512	0.0431
M_64	GO:0030241	0.04731
M_64	GO:0060088	0.04731
M_64	GO:0042135	0.04731
M_64	GO:0060546	0.04731
M_64	GO:0010763	0.04731
M_64	GO:0060340	0.04731
M_64	GO:0033314	0.04731
M_65	GO:1901673	0.0046
M_65	GO:1990280	0.0057
M_65	GO:1902889	0.0057
M_65	GO:0031509	0.0057
M_65	GO:1990755	0.0057
M_65	GO:0007225	0.0057
M_65	GO:1902340	0.0057
M_65	GO:0097036	0.0057
M_65	GO:0033386	0.0057
M_65	GO:0006695	0.0086
M_65	GO:0000453	0.0113
M_65	GO:0030821	0.0113
M_65	GO:0016031	0.0113
M_65	GO:2000815	0.0113
M_65	GO:0070096	0.0113

M_65	GO:0021935	0.0113
M_65	GO:0019285	0.0113
M_65	GO:0033030	0.0113
M_65	GO:1902425	0.0113
M_65	GO:1903518	0.0113
M_65	GO:0006424	0.0113
M_65	GO:0006428	0.0113
M_65	GO:0097037	0.0113
M_65	GO:0033384	0.0113
M_65	GO:0030488	0.0158
M_65	GO:0008654	0.0158
M_65	GO:0032200	0.0167
M_65	GO:0071731	0.0167
M_65	GO:0002378	0.0169
M_65	GO:0006106	0.0169
M_65	GO:0044208	0.0169
M_65	GO:0001927	0.0169
M_65	GO:0043504	0.0169
M_65	GO:0031508	0.0169
M_65	GO:0042998	0.0169
M_65	GO:0018312	0.0169
M_65	GO:0034164	0.0169
M_65	GO:0097176	0.0169
M_65	GO:0006391	0.0169
M_65	GO:2001170	0.0169
M_65	GO:0031081	0.0169
M_65	GO:0097051	0.0169
M_65	GO:0006433	0.0169
M_65	GO:0042254	0.0173
M_65	GO:0045337	0.0224
M_65	GO:1903061	0.0224
M_65	GO:0070212	0.0224

M_65	GO:0007023	0.0224
M_65	GO:0045900	0.0224
M_65	GO:1903373	0.0224
M_65	GO:0000462	0.0225
M_65	GO:0050684	0.0265
M_65	GO:0015031	0.0274
M_65	GO:0006999	0.0277
M_65	GO:0060296	0.0279
M_65	GO:0055129	0.0279
M_65	GO:0051344	0.0279
M_65	GO:0097368	0.0279
M_65	GO:0008611	0.0279
M_65	GO:0070934	0.0279
M_65	GO:0032383	0.0279
M_65	GO:0090230	0.0279
M_65	GO:1904044	0.0279
M_65	GO:0060008	0.0331
M_65	GO:1904357	0.0331
M_65	GO:0042426	0.0334
M_65	GO:0010990	0.0334
M_65	GO:0032687	0.0334
M_65	GO:2000664	0.0334
M_65	GO:2000667	0.0334
M_65	GO:2000373	0.0334
M_65	GO:0034773	0.0334
M_65	GO:0018242	0.0334
M_65	GO:0006839	0.0368
M_65	GO:0035333	0.0389
M_65	GO:0030913	0.0389
M_65	GO:0018344	0.0389
M_65	GO:0007256	0.0389
M_65	GO:0000972	0.0389

M_65	GO:0035726	0.0389
M_65	GO:0021785	0.0389
M_65	GO:0032364	0.0389
M_65	GO:0018243	0.0389
M_65	GO:0051865	0.0426
M_65	GO:0006108	0.0443
M_65	GO:1902857	0.0443
M_65	GO:0018401	0.0443
M_65	GO:0006616	0.0443
M_65	GO:1903376	0.0443
M_65	GO:0006879	0.0466
M_65	GO:0090557	0.0497
M_65	GO:0051601	0.0497
M_65	GO:2000234	0.0497
M_65	GO:0006072	0.0497
M_65	GO:0048671	0.0497
M_65	GO:0090336	0.0497
M_65	GO:0046598	0.0497
M_65	GO:0046548	0.0497
M_65	GO:0097400	0.0497
M_65	GO:0060539	0.0497
M_65	GO:0051901	0.0497
M_66	GO:0090168	0.00025
M_66	GO:0000724	0.00054
M_66	GO:0060213	0.00226
M_66	GO:0031648	0.00228
M_66	GO:0090656	0.00483
M_66	GO:0007131	0.00508
M_66	GO:0006312	0.00536
M_66	GO:0032508	0.00623
M_66	GO:0090502	0.00625
M_66	GO:0042140	0.00656

M_66	GO:0042275	0.00656
M_66	GO:0090284	0.00656
M_66	GO:2000568	0.00656
M_66	GO:0090625	0.00656
M_66	GO:0045947	0.00679
M_66	GO:0017148	0.00913
M_66	GO:0007141	0.00988
M_66	GO:0001556	0.01073
M_66	GO:0000729	0.01253
M_66	GO:0016320	0.01309
M_66	GO:0045659	0.01309
M_66	GO:0045645	0.01309
M_66	GO:0035350	0.01309
M_66	GO:0032214	0.01309
M_66	GO:1902269	0.01309
M_66	GO:0035279	0.01309
M_66	GO:1904017	0.01309
M_66	GO:0097213	0.01309
M_66	GO:0031663	0.01626
M_66	GO:0032436	0.01748
M_66	GO:0032092	0.01844
M_66	GO:0016071	0.01931
M_66	GO:0008594	0.01957
M_66	GO:1901315	0.01957
M_66	GO:0070427	0.01957
M_66	GO:0035880	0.01957
M_66	GO:0033387	0.01957
M_66	GO:0034421	0.01957
M_66	GO:0007223	0.02207
M_66	GO:0007080	0.02327
M_66	GO:0045876	0.02579
M_66	GO:0034088	0.02579

M_66	GO:0019518	0.026
M_66	GO:0060800	0.026
M_66	GO:0007089	0.026
M_66	GO:0032911	0.026
M_66	GO:0006021	0.026
M_66	GO:0040038	0.026
M_66	GO:0006659	0.026
M_66	GO:0048105	0.026
M_66	GO:0033091	0.026
M_66	GO:0042766	0.026
M_66	GO:0044878	0.026
M_66	GO:0031860	0.026
M_66	GO:0031591	0.026
M_66	GO:0033615	0.026
M_66	GO:1903527	0.026
M_66	GO:0031627	0.026
M_66	GO:0035087	0.026
M_66	GO:2000623	0.026
M_66	GO:0006613	0.03229
M_66	GO:0034184	0.0324
M_66	GO:0071224	0.0324
M_66	GO:0071225	0.0324
M_66	GO:1990511	0.0324
M_66	GO:1902083	0.0324
M_66	GO:0090611	0.0324
M_66	GO:1904354	0.0324
M_66	GO:0000480	0.03875
M_66	GO:1900264	0.03875
M_66	GO:0090306	0.03875
M_66	GO:1904417	0.03875
M_66	GO:0045627	0.03875
M_66	GO:0033278	0.03875

M_66	GO:0006572	0.03875
M_66	GO:1901165	0.03875
M_66	GO:0009838	0.03875
M_66	GO:0061668	0.03875
M_66	GO:0015014	0.03875
M_66	GO:0030174	0.03875
M_66	GO:0006413	0.04208
M_66	GO:0015031	0.04257
M_66	GO:0000707	0.04468
M_66	GO:0032466	0.04506
M_66	GO:0018406	0.04506
M_66	GO:1902455	0.04506
M_66	GO:1902416	0.04506
M_66	GO:0035280	0.04506
M_66	GO:1902570	0.04506
M_66	GO:0016236	0.0455
M_66	GO:0060964	0.04738
M_67	GO:0043518	0.00066
M_67	GO:0021618	0.00274
M_67	GO:0098838	0.00274
M_67	GO:0009443	0.00274
M_67	GO:0071110	0.00274
M_67	GO:0051958	0.00547
M_67	GO:0070781	0.00547
M_67	GO:0006364	0.0075
M_67	GO:1901666	0.0082
M_67	GO:0048597	0.0082
M_67	GO:0001712	0.0082
M_67	GO:0000720	0.0082
M_67	GO:0021965	0.0082
M_67	GO:0016925	0.0086
M_67	GO:0070936	0.00893

M_67	GO:0046084	0.01092
M_67	GO:0019556	0.01092
M_67	GO:0019557	0.01092
M_67	GO:0042816	0.01092
M_67	GO:0006311	0.01092
M_67	GO:0032776	0.01092
M_67	GO:0071816	0.01363
M_67	GO:0006189	0.01633
M_67	GO:0015760	0.01633
M_67	GO:0097049	0.01903
M_67	GO:0006768	0.01903
M_67	GO:0006382	0.01903
M_67	GO:0003360	0.01903
M_67	GO:0030388	0.02171
M_67	GO:0015884	0.02171
M_67	GO:0044387	0.02171
M_67	GO:0019919	0.02171
M_67	GO:0036289	0.02171
M_67	GO:0046654	0.02171
M_67	GO:0051026	0.02171
M_67	GO:0036066	0.02171
M_67	GO:0006002	0.0244
M_67	GO:0034498	0.0244
M_67	GO:1904153	0.0244
M_67	GO:0021610	0.0244
M_67	GO:0018026	0.02707
M_67	GO:0006325	0.02761
M_67	GO:0090009	0.02974
M_67	GO:0035999	0.0324
M_67	GO:0031115	0.0324
M_67	GO:0006397	0.03352
M_67	GO:0016024	0.03505

M_67	GO:0034969	0.03505
M_67	GO:0040034	0.03505
M_67	GO:0010225	0.0377
M_67	GO:0035435	0.0377
M_67	GO:0003352	0.0377
M_67	GO:0050884	0.04034
M_67	GO:0006004	0.04297
M_67	GO:0006544	0.04297
M_67	GO:0035458	0.04297
M_67	GO:0043923	0.04559
M_67	GO:0010224	0.04821
M_67	GO:0044030	0.04821
M_67	GO:0006349	0.04821
M_68	GO:0050911	4.8E-11
M_68	GO:0007186	0.0000036
M_68	GO:0031424	0.000027
M_68	GO:0050830	0.0026
M_68	GO:0002779	0.0032
M_68	GO:0048240	0.0041
M_68	GO:0001519	0.0064
M_68	GO:0060163	0.0064
M_68	GO:0030574	0.0072
M_68	GO:0072249	0.0096
M_68	GO:0045087	0.0098
M_68	GO:0002357	0.0127
M_68	GO:0032827	0.0127
M_68	GO:1904628	0.0127
M_68	GO:0070173	0.0159
M_68	GO:0006844	0.0159
M_68	GO:0009056	0.0172
M_68	GO:0042742	0.0174
M_68	GO:0051694	0.0222

M_68	GO:0009253	0.0222
M_68	GO:0021798	0.0222
M_68	GO:0051792	0.0222
M_68	GO:0022617	0.024
M_68	GO:0035627	0.0284
M_68	GO:0097119	0.0284
M_68	GO:0052696	0.0284
M_68	GO:0007338	0.0288
M_68	GO:0050829	0.029
M_68	GO:0043129	0.0315
M_68	GO:0033234	0.0346
M_68	GO:0052697	0.0346
M_68	GO:0033700	0.0377
M_68	GO:0021889	0.0377
M_68	GO:0021978	0.0408
M_68	GO:0016045	0.0408
M_68	GO:0002087	0.0408
M_68	GO:0048853	0.0438
M_68	GO:0021527	0.0438
M_68	GO:0044849	0.0438
M_68	GO:0010875	0.0469
M_68	GO:2000010	0.05
M_69	GO:0071318	0.00011
M_69	GO:0045540	0.00025
M_69	GO:0035511	0.00053
M_69	GO:0045008	0.00146
M_69	GO:0010501	0.0047
M_69	GO:0033539	0.00531
M_69	GO:0007049	0.00578
M_69	GO:0055119	0.00681
M_69	GO:2000850	0.00731
M_69	GO:0021993	0.00736

M_69	GO:0018117	0.00736
M_69	GO:0046359	0.00736
M_69	GO:0097510	0.00736
M_69	GO:0060409	0.00736
M_69	GO:0070902	0.00736
M_69	GO:1903586	0.00736
M_69	GO:1903515	0.00736
M_69	GO:0044602	0.00736
M_69	GO:0072365	0.00736
M_69	GO:0051463	0.00736
M_69	GO:1900402	0.00736
M_69	GO:0016236	0.00758
M_69	GO:0030220	0.00848
M_69	GO:0048025	0.00937
M_69	GO:0060716	0.00937
M_69	GO:0000381	0.01029
M_69	GO:0043153	0.01129
M_69	GO:0006297	0.0123
M_69	GO:0032470	0.01467
M_69	GO:0007057	0.01467
M_69	GO:0033140	0.01467
M_69	GO:0033277	0.01467
M_69	GO:1901485	0.01467
M_69	GO:0015920	0.01467
M_69	GO:0009785	0.01467
M_69	GO:0031247	0.01467
M_69	GO:1903233	0.01467
M_69	GO:0061015	0.01467
M_69	GO:0044861	0.01467
M_69	GO:0006393	0.01467
M_69	GO:0061582	0.01467
M_69	GO:0035281	0.01467

M_69	GO:0021592	0.01467
M_69	GO:0060125	0.01467
M_69	GO:2000588	0.01467
M_69	GO:2001295	0.01467
M_69	GO:1902544	0.01467
M_69	GO:0043335	0.01467
M_69	GO:0045004	0.01467
M_69	GO:0045292	0.01651
M_69	GO:0051301	0.01692
M_69	GO:0002026	0.01795
M_69	GO:0070979	0.02046
M_69	GO:0032268	0.02091
M_69	GO:0000462	0.02147
M_69	GO:2000374	0.02193
M_69	GO:0000472	0.02193
M_69	GO:1903445	0.02193
M_69	GO:0033292	0.02193
M_69	GO:0009236	0.02193
M_69	GO:0070681	0.02193
M_69	GO:0070682	0.02193
M_69	GO:1990036	0.02193
M_69	GO:1903774	0.02193
M_69	GO:0002188	0.02193
M_69	GO:0019287	0.02193
M_69	GO:2001271	0.02193
M_69	GO:0006272	0.02193
M_69	GO:0044313	0.02193
M_69	GO:0010899	0.02193
M_69	GO:0006470	0.02273
M_69	GO:0032092	0.02487
M_69	GO:0042593	0.02564
M_69	GO:0046676	0.02879

M_69	GO:0034770	0.02892
M_69	GO:0031999	0.02913
M_69	GO:0021555	0.02913
M_69	GO:0006409	0.02913
M_69	GO:0021678	0.02913
M_69	GO:0046836	0.02913
M_69	GO:1990481	0.02913
M_69	GO:0032497	0.02913
M_69	GO:0006296	0.03029
M_69	GO:0010626	0.03628
M_69	GO:0075044	0.03628
M_69	GO:0034316	0.03628
M_69	GO:1901525	0.03628
M_69	GO:0006287	0.03628
M_69	GO:0051168	0.04286
M_69	GO:0034260	0.04336
M_69	GO:0000480	0.04338
M_69	GO:1900264	0.04338
M_69	GO:2000323	0.04338
M_69	GO:0017196	0.04338
M_69	GO:0043985	0.04338
M_69	GO:0034201	0.04338
M_69	GO:0034773	0.04338
M_69	GO:0070358	0.04338
M_69	GO:0048205	0.04338
M_69	GO:0072344	0.04338
M_70	GO:1990809	0.00012
M_70	GO:0000710	0.00012
M_70	GO:0043570	0.0002
M_70	GO:0043111	0.0003
M_70	GO:0051096	0.0003
M_70	GO:0006290	0.00071

M_70	GO:0036297	0.0014
M_70	GO:0008340	0.00152
M_70	GO:0016446	0.00231
M_70	GO:0045943	0.00437
M_70	GO:0048694	0.00451
M_70	GO:0060734	0.00451
M_70	GO:0045128	0.00451
M_70	GO:0072703	0.00451
M_70	GO:0097222	0.00451
M_70	GO:0035246	0.00889
M_70	GO:0070124	0.009
M_70	GO:0000961	0.009
M_70	GO:0000962	0.009
M_70	GO:0000964	0.009
M_70	GO:0019255	0.009
M_70	GO:1903860	0.009
M_70	GO:0030328	0.009
M_70	GO:2000627	0.009
M_70	GO:0060152	0.009
M_70	GO:0030488	0.01026
M_70	GO:0033120	0.01085
M_70	GO:0048009	0.01145
M_70	GO:0000375	0.01321
M_70	GO:0006065	0.01347
M_70	GO:0017187	0.01347
M_70	GO:1990737	0.01347
M_70	GO:0000958	0.01347
M_70	GO:0035928	0.01347
M_70	GO:0030327	0.01347
M_70	GO:0016344	0.01347
M_70	GO:1901895	0.01347
M_70	GO:0033326	0.01347

M_70	GO:0010824	0.01469
M_70	GO:0015031	0.01653
M_70	GO:0032057	0.01792
M_70	GO:0071787	0.01792
M_70	GO:0045629	0.01792
M_70	GO:0035984	0.01792
M_70	GO:0019918	0.01792
M_70	GO:0016560	0.01792
M_70	GO:0006311	0.01792
M_70	GO:0006499	0.01792
M_70	GO:0089700	0.01792
M_70	GO:2000172	0.01792
M_70	GO:0009411	0.02084
M_70	GO:0000045	0.02176
M_70	GO:0048298	0.02235
M_70	GO:0006915	0.02257
M_70	GO:0043687	0.02404
M_70	GO:0032922	0.02556
M_70	GO:0006896	0.02643
M_70	GO:0071042	0.02676
M_70	GO:0006011	0.02676
M_70	GO:0045627	0.02676
M_70	GO:0031642	0.02676
M_70	GO:0032876	0.02676
M_70	GO:0033206	0.02676
M_70	GO:0071638	0.02676
M_70	GO:0008630	0.02946
M_70	GO:2000271	0.03115
M_70	GO:0086036	0.03115
M_70	GO:0000056	0.03115
M_70	GO:0048102	0.03115
M_70	GO:0031119	0.03115

M_70	GO:2000659	0.03115
M_70	GO:0070417	0.03552
M_70	GO:0032790	0.03552
M_70	GO:0060052	0.03552
M_70	GO:0035093	0.03552
M_70	GO:0001561	0.03552
M_70	GO:0090160	0.03552
M_70	GO:1901029	0.03552
M_70	GO:0032471	0.03552
M_70	GO:0071447	0.03552
M_70	GO:0047497	0.03552
M_70	GO:0048208	0.03576
M_70	GO:0006470	0.03781
M_70	GO:0033601	0.03987
M_70	GO:2000234	0.03987
M_70	GO:0048304	0.03987
M_70	GO:0006002	0.03987
M_70	GO:0034498	0.03987
M_70	GO:1904153	0.03987
M_70	GO:0045830	0.04368
M_70	GO:0042176	0.04405
M_70	GO:0071985	0.0442
M_70	GO:1901387	0.0442
M_70	GO:0051292	0.0442
M_70	GO:0030311	0.0442
M_70	GO:0071277	0.04838
M_70	GO:0034644	0.04838
M_70	GO:0006983	0.04851
M_70	GO:0006048	0.04851
M_70	GO:0000055	0.04851
M_71	GO:0051415	0.000092
M_71	GO:0090307	0.00055

M_71	GO:0006293	0.00126
M_71	GO:0006294	0.0024
M_71	GO:0051417	0.00251
M_71	GO:0036494	0.00251
M_71	GO:0051102	0.00251
M_71	GO:0006296	0.00388
M_71	GO:0031393	0.00502
M_71	GO:0033566	0.00502
M_71	GO:0097359	0.00502
M_71	GO:0043323	0.00502
M_71	GO:0006423	0.00502
M_71	GO:0000973	0.00752
M_71	GO:2000819	0.00752
M_71	GO:0031134	0.00752
M_71	GO:0034247	0.00752
M_71	GO:0097680	0.00752
M_71	GO:0035019	0.00963
M_71	GO:0033152	0.01001
M_71	GO:1904424	0.01001
M_71	GO:0071034	0.01001
M_71	GO:0051725	0.01001
M_71	GO:1903912	0.01001
M_71	GO:0006295	0.01001
M_71	GO:0033153	0.0125
M_71	GO:0071033	0.0125
M_71	GO:0070345	0.0125
M_71	GO:0031338	0.01336
M_71	GO:0010941	0.01394
M_71	GO:0006283	0.01447
M_71	GO:0008626	0.01498
M_71	GO:1902513	0.01498
M_71	GO:0046833	0.01498

M_71	GO:2000394	0.01745
M_71	GO:0006768	0.01745
M_71	GO:0090267	0.01745
M_71	GO:0048102	0.01745
M_71	GO:0090160	0.01992
M_71	GO:0032815	0.01992
M_71	GO:0030853	0.01992
M_71	GO:0075713	0.01992
M_71	GO:0006610	0.01992
M_71	GO:0070417	0.01992
M_71	GO:0090630	0.02192
M_71	GO:0002084	0.02238
M_71	GO:0060124	0.02238
M_71	GO:1904153	0.02238
M_71	GO:0019236	0.02238
M_71	GO:0000012	0.02484
M_71	GO:0010763	0.02729
M_71	GO:0042532	0.02729
M_71	GO:0002328	0.02729
M_71	GO:0010923	0.02759
M_71	GO:0006913	0.02834
M_71	GO:0051103	0.02974
M_71	GO:0071712	0.03217
M_71	GO:0009650	0.03217
M_71	GO:0042976	0.03217
M_71	GO:0045579	0.03217
M_71	GO:0071285	0.03217
M_71	GO:0051315	0.03217
M_71	GO:1904380	0.03461
M_71	GO:0034501	0.03461
M_71	GO:0032486	0.03461
M_71	GO:0010225	0.03461

M_71	GO:0043488	0.03651
M_71	GO:0034356	0.03703
M_71	GO:0035372	0.03703
M_71	GO:0045116	0.03703
M_71	GO:0030033	0.03945
M_71	GO:0034975	0.03945
M_71	GO:2000001	0.03945
M_71	GO:0016973	0.03945
M_71	GO:0032695	0.03945
M_71	GO:0035023	0.04339
M_71	GO:0000185	0.04428
M_71	GO:0071539	0.04428
M_71	GO:0007049	0.04682
M_71	GO:0031145	0.04812
M_71	GO:0016075	0.04908
M_72	GO:1904261	0.00018
M_72	GO:0023014	0.00285
M_72	GO:0006337	0.00303
M_72	GO:1903568	0.00434
M_72	GO:0072274	0.00434
M_72	GO:0061519	0.00434
M_72	GO:1990245	0.00434
M_72	GO:0035212	0.00434
M_72	GO:1900062	0.00434
M_72	GO:0021682	0.00434
M_72	GO:0034203	0.00434
M_72	GO:1903387	0.00434
M_72	GO:0032958	0.00608
M_72	GO:0031098	0.00655
M_72	GO:0071108	0.00847
M_72	GO:1903955	0.00847
M_72	GO:0060060	0.00866

M_72	GO:0097198	0.00866
M_72	GO:1903575	0.00866
M_72	GO:0016340	0.00866
M_72	GO:0006429	0.00866
M_72	GO:1900220	0.00866
M_72	GO:0051012	0.00866
M_72	GO:0010470	0.0118
M_72	GO:0014044	0.01281
M_72	GO:0061357	0.01296
M_72	GO:1902856	0.01296
M_72	GO:0048677	0.01296
M_72	GO:0072249	0.01296
M_72	GO:0019464	0.01296
M_72	GO:0016476	0.01296
M_72	GO:1902775	0.01296
M_72	GO:0035441	0.01296
M_72	GO:0097676	0.01296
M_72	GO:0048705	0.01649
M_72	GO:0048864	0.01697
M_72	GO:0035987	0.01699
M_72	GO:0031114	0.01704
M_72	GO:1903690	0.01724
M_72	GO:0045716	0.01724
M_72	GO:0038162	0.01724
M_72	GO:0071847	0.01724
M_72	GO:0035522	0.01724
M_72	GO:0034340	0.02133
M_72	GO:1902525	0.02151
M_72	GO:2001205	0.02151
M_72	GO:0045541	0.02151
M_72	GO:0051301	0.02449
M_72	GO:0010793	0.02575

M_72	GO:0034453	0.0271
M_72	GO:0007010	0.0287
M_72	GO:0000381	0.02882
M_72	GO:2000479	0.0296
M_72	GO:1903608	0.02998
M_72	GO:0090091	0.02998
M_72	GO:0031087	0.02998
M_72	GO:0072383	0.02998
M_72	GO:1903003	0.02998
M_72	GO:1901098	0.02998
M_72	GO:0071476	0.02998
M_72	GO:0032435	0.03059
M_72	GO:1901799	0.03342
M_72	GO:2000480	0.03419
M_72	GO:2000643	0.03419
M_72	GO:0032927	0.03419
M_72	GO:0060836	0.03419
M_72	GO:0035331	0.03419
M_72	GO:0043044	0.03614
M_72	GO:0048617	0.03838
M_72	GO:0046548	0.03838
M_72	GO:0032933	0.03838
M_72	GO:0034199	0.03838
M_72	GO:0006265	0.03838
M_72	GO:0033689	0.03838
M_72	GO:0033601	0.03838
M_72	GO:0071679	0.03838
M_72	GO:0033235	0.04256
M_72	GO:0018026	0.04256
M_72	GO:0007016	0.04256
M_72	GO:0000290	0.04256
M_72	GO:0014894	0.04256

M_72	GO:0010717	0.04513
M_72	GO:0072520	0.04671
M_72	GO:0050772	0.04723
M_72	GO:0007409	0.04856
M_73	GO:0050911	2.8E-15
M_73	GO:0045892	8.4E-11
M_73	GO:0007186	3.7E-10
M_73	GO:0045596	0.00000012
M_73	GO:0008284	0.000001
M_73	GO:0043066	0.0000096
M_73	GO:0006346	0.0018
M_73	GO:2000212	0.003
M_73	GO:0045423	0.0089
M_73	GO:0042109	0.0089
M_73	GO:0007198	0.0118
M_73	GO:0006798	0.0148
M_73	GO:0006164	0.0176
M_73	GO:0006015	0.0177
M_73	GO:0014062	0.0206
M_73	GO:0051673	0.0264
M_73	GO:0097400	0.0264
M_73	GO:0019236	0.0264
M_73	GO:0007195	0.0293
M_73	GO:1901750	0.0293
M_73	GO:0042428	0.0293
M_73	GO:0090084	0.0293
M_73	GO:0006751	0.0322
M_73	GO:0045414	0.0379
M_73	GO:0060158	0.0408
M_73	GO:0042754	0.0408
M_73	GO:0045408	0.0436
M_73	GO:0051967	0.0436

M_73	GO:0048149	0.0436
M_73	GO:1900017	0.0465
M_73	GO:0051481	0.0465
M_73	GO:0048311	0.0493
M_74	GO:0006189	0.00034
M_74	GO:0009113	0.00199
M_74	GO:0005980	0.00295
M_74	GO:0070987	0.00408
M_74	GO:1901186	0.00475
M_74	GO:1904586	0.00479
M_74	GO:2000798	0.00479
M_74	GO:2000706	0.00479
M_74	GO:0009107	0.00479
M_74	GO:1901189	0.00479
M_74	GO:1990828	0.00479
M_74	GO:1901297	0.00479
M_74	GO:0043041	0.00479
M_74	GO:0061024	0.00644
M_74	GO:0010800	0.00792
M_74	GO:0032979	0.00957
M_74	GO:1901355	0.00957
M_74	GO:0051455	0.00957
M_74	GO:0006043	0.00957
M_74	GO:0019408	0.00957
M_74	GO:0000105	0.00957
M_74	GO:0060844	0.00957
M_74	GO:0048211	0.00957
M_74	GO:0036228	0.00957
M_74	GO:0032472	0.00957
M_74	GO:0072554	0.00957
M_74	GO:0061723	0.00957
M_74	GO:0010543	0.00965

M_74	GO:1904383	0.01432
M_74	GO:0006065	0.01432
M_74	GO:0016095	0.01432
M_74	GO:0006046	0.01432
M_74	GO:0009957	0.01432
M_74	GO:0006543	0.01432
M_74	GO:0097101	0.01432
M_74	GO:0061092	0.01432
M_74	GO:0000398	0.017
M_74	GO:0060379	0.01883
M_74	GO:0006378	0.01886
M_74	GO:0016259	0.01904
M_74	GO:0019482	0.01904
M_74	GO:2000065	0.01904
M_74	GO:0090370	0.01904
M_74	GO:1903899	0.01904
M_74	GO:0021764	0.01904
M_74	GO:0097167	0.01904
M_74	GO:0032348	0.01904
M_74	GO:1900127	0.01904
M_74	GO:0060486	0.01904
M_74	GO:0097056	0.01904
M_74	GO:1900245	0.01904
M_74	GO:0045747	0.02136
M_74	GO:0009056	0.02205
M_74	GO:0006999	0.02348
M_74	GO:2000138	0.02375
M_74	GO:0032237	0.02375
M_74	GO:0010606	0.02375
M_74	GO:0009256	0.02375
M_74	GO:0061419	0.02375
M_74	GO:1903371	0.02375

M_74	GO:0006489	0.02375
M_74	GO:0038083	0.02673
M_74	GO:0006164	0.02811
M_74	GO:0072602	0.02843
M_74	GO:0006011	0.02843
M_74	GO:0010994	0.02843
M_74	GO:0019262	0.02843
M_74	GO:0009912	0.02843
M_74	GO:0003160	0.02843
M_74	GO:0060335	0.02843
M_74	GO:2000323	0.02843
M_74	GO:0006893	0.0296
M_74	GO:0042246	0.03058
M_74	GO:0035690	0.03258
M_74	GO:0060013	0.03309
M_74	GO:0036302	0.03309
M_74	GO:0007253	0.03309
M_74	GO:1903896	0.03309
M_74	GO:0003256	0.03309
M_74	GO:0048733	0.03309
M_74	GO:1900246	0.03309
M_74	GO:0001955	0.03773
M_74	GO:0060059	0.03773
M_74	GO:1902857	0.03773
M_74	GO:2000074	0.03773
M_74	GO:0035912	0.03773
M_74	GO:0021940	0.03773
M_74	GO:0009249	0.03773
M_74	GO:0044332	0.03773
M_74	GO:0006616	0.03773
M_74	GO:0097084	0.03773
M_74	GO:0045842	0.03773

M_74	GO:0051601	0.04234
M_74	GO:0051151	0.04234
M_74	GO:0070933	0.04234
M_74	GO:0001514	0.04234
M_74	GO:0003139	0.04234
M_74	GO:0045955	0.04234
M_74	GO:0006511	0.04325
M_74	GO:0045787	0.04526
M_74	GO:0061157	0.04643
M_74	GO:0019388	0.04694
M_74	GO:0061314	0.04694
M_74	GO:0008343	0.04694
M_74	GO:0051292	0.04694
M_74	GO:0048280	0.04694
M_74	GO:0006283	0.04795
M_75	GO:0006895	0.0045
M_75	GO:1901340	0.005
M_75	GO:0006055	0.005
M_75	GO:0098705	0.005
M_75	GO:1903883	0.005
M_75	GO:1903886	0.005
M_75	GO:1902605	0.005
M_75	GO:0007014	0.005
M_75	GO:0070869	0.005
M_75	GO:0018964	0.005
M_75	GO:0032897	0.0059
M_75	GO:0000715	0.0059
M_75	GO:0021591	0.0069
M_75	GO:0070911	0.0075
M_75	GO:0006294	0.0093
M_75	GO:0003016	0.0099
M_75	GO:0052746	0.01

M_75	GO:0035498	0.01
M_75	GO:0090387	0.01
M_75	GO:0008355	0.01
M_75	GO:0046511	0.01
M_75	GO:1901255	0.01
M_75	GO:0097111	0.01
M_75	GO:0061188	0.01
M_75	GO:0006679	0.01
M_75	GO:0033577	0.01
M_75	GO:0097352	0.0133
M_75	GO:0010266	0.015
M_75	GO:0034140	0.015
M_75	GO:0070541	0.015
M_75	GO:0021586	0.015
M_75	GO:0070682	0.015
M_75	GO:0071284	0.0199
M_75	GO:1901537	0.0199
M_75	GO:0042126	0.0199
M_75	GO:0016240	0.0199
M_75	GO:0060005	0.0199
M_75	GO:0042766	0.0199
M_75	GO:0061470	0.0199
M_75	GO:1990834	0.0199
M_75	GO:2001168	0.0199
M_75	GO:1903539	0.0199
M_75	GO:0006490	0.0199
M_75	GO:0061084	0.0199
M_75	GO:0072385	0.0199
M_75	GO:1903169	0.0247
M_75	GO:0035610	0.0249
M_75	GO:0035609	0.0249
M_75	GO:1904504	0.0249

M_75	GO:0051410	0.0249
M_75	GO:0045329	0.0249
M_75	GO:0008616	0.0249
M_75	GO:0010961	0.0249
M_75	GO:0071557	0.0249
M_75	GO:0034184	0.0249
M_75	GO:0072719	0.0249
M_75	GO:0031133	0.0249
M_75	GO:0010044	0.0249
M_75	GO:0001661	0.0249
M_75	GO:0045053	0.0249
M_75	GO:0060179	0.0249
M_75	GO:0035176	0.0291
M_75	GO:0042426	0.0298
M_75	GO:0009635	0.0298
M_75	GO:0098902	0.0298
M_75	GO:0045634	0.0298
M_75	GO:0042256	0.0298
M_75	GO:0035973	0.0298
M_75	GO:0071651	0.0298
M_75	GO:0006686	0.0298
M_75	GO:0010212	0.0322
M_75	GO:0007059	0.0335
M_75	GO:2001056	0.0337
M_75	GO:1902570	0.0346
M_75	GO:1903071	0.0346
M_75	GO:0038203	0.0346
M_75	GO:0048102	0.0346
M_75	GO:0051597	0.0346
M_75	GO:1903608	0.0346
M_75	GO:0035264	0.0366
M_75	GO:0015871	0.0395

M_75	GO:1903265	0.0395
M_75	GO:0006782	0.0395
M_75	GO:1900364	0.0395
M_75	GO:0006548	0.0395
M_75	GO:0051045	0.0395
M_75	GO:0051549	0.0395
M_75	GO:0030307	0.0432
M_75	GO:0048570	0.0443
M_75	GO:0090557	0.0443
M_75	GO:0021702	0.0443
M_75	GO:2000320	0.0443
M_75	GO:2000628	0.0443
M_75	GO:0032025	0.0491
M_75	GO:0046512	0.0491
M_75	GO:0032525	0.0491
M_75	GO:0000730	0.0491
M_75	GO:0016322	0.0491
M_75	GO:0031053	0.0491
M_75	GO:1901838	0.0491
M_76	GO:0070373	6.1E-09
M_76	GO:0010801	0.00000017
M_76	GO:0000188	0.0000035
M_76	GO:0001657	0.0000089
M_76	GO:0040037	0.000034
M_76	GO:0051387	0.000097
M_76	GO:0046580	0.00015
M_76	GO:0060373	0.00034
M_76	GO:0060449	0.00034
M_76	GO:0030574	0.00057
M_76	GO:0035335	0.0007
M_76	GO:0043407	0.00082
M_76	GO:0060394	0.00087

M_76	GO:0060252	0.00104
M_76	GO:0034616	0.00143
M_76	GO:0043517	0.00143
M_76	GO:0030512	0.00276
M_76	GO:0022617	0.0035
M_76	GO:0000132	0.00388
M_76	GO:0003174	0.004
M_76	GO:1990029	0.00405
M_76	GO:0000032	0.00405
M_76	GO:0002264	0.00405
M_76	GO:0061611	0.00405
M_76	GO:0086054	0.00405
M_76	GO:0086055	0.00405
M_76	GO:0098906	0.00405
M_76	GO:0086044	0.00405
M_76	GO:0045081	0.00405
M_76	GO:0032461	0.00422
M_76	GO:1902895	0.00422
M_76	GO:0061621	0.00457
M_76	GO:1990830	0.00601
M_76	GO:0032436	0.00619
M_76	GO:0022405	0.00802
M_76	GO:0001582	0.00809
M_76	GO:0032916	0.00809
M_76	GO:0071105	0.00809
M_76	GO:0010652	0.00809
M_76	GO:0009098	0.00809
M_76	GO:0009099	0.00809
M_76	GO:0035922	0.00809
M_76	GO:0003193	0.00809
M_76	GO:0003294	0.00809
M_76	GO:0060940	0.00809

M_76	GO:0086053	0.00809
M_76	GO:0098905	0.00809
M_76	GO:0086021	0.00809
M_76	GO:0097112	0.00809
M_76	GO:0060412	0.00984
M_76	GO:0042059	0.01089
M_76	GO:0090311	0.01199
M_76	GO:0032474	0.01211
M_76	GO:0021912	0.01211
M_76	GO:0003340	0.01211
M_76	GO:0007352	0.01211
M_76	GO:0098910	0.01211
M_76	GO:0098904	0.01211
M_76	GO:0045668	0.01314
M_76	GO:0034260	0.01433
M_76	GO:0045599	0.01494
M_76	GO:0070670	0.01592
M_76	GO:0071918	0.01611
M_76	GO:0021913	0.01611
M_76	GO:0009298	0.01611
M_76	GO:0042997	0.01611
M_76	GO:0030578	0.01611
M_76	GO:0060368	0.01611
M_76	GO:0048133	0.01611
M_76	GO:0060979	0.01611
M_76	GO:0042663	0.01611
M_76	GO:0035021	0.01611
M_76	GO:0097296	0.01611
M_76	GO:0019049	0.01611
M_76	GO:1900133	0.01611
M_76	GO:0030514	0.0162
M_76	GO:0048146	0.01817

M_76	GO:0060662	0.0201
M_76	GO:1900825	0.0201
M_76	GO:1900020	0.0201
M_76	GO:0003105	0.0201
M_76	GO:0060437	0.0201
M_76	GO:0010991	0.0201
M_76	GO:0010989	0.0201
M_76	GO:0060762	0.0201
M_76	GO:0032922	0.02094
M_76	GO:0060395	0.02239
M_76	GO:0048844	0.02388
M_76	GO:0090398	0.02388
M_76	GO:0060842	0.02407
M_76	GO:0071104	0.02407
M_76	GO:0010694	0.02407
M_76	GO:0045345	0.02407
M_76	GO:1903243	0.02407
M_76	GO:0050917	0.02407
M_76	GO:0003284	0.02407
M_76	GO:0007182	0.02407
M_76	GO:0006013	0.02764
M_76	GO:0007183	0.02765
M_76	GO:0060012	0.02803
M_76	GO:0060013	0.02803
M_76	GO:0031087	0.02803
M_76	GO:0060290	0.02803
M_76	GO:0036089	0.02803
M_76	GO:0044351	0.02803
M_76	GO:0010727	0.02803
M_76	GO:0015914	0.02858
M_76	GO:0007275	0.02894
M_76	GO:0071345	0.02925

M_76	GO:1901224	0.03023
M_76	GO:0043405	0.03193
M_76	GO:0032909	0.03197
M_76	GO:0039536	0.03197
M_76	GO:0060371	0.03197
M_76	GO:1902857	0.03197
M_76	GO:0015871	0.03197
M_76	GO:0002725	0.03197
M_76	GO:0051409	0.03197
M_76	GO:0030210	0.03197
M_76	GO:0000281	0.03276
M_76	GO:0008543	0.03519
M_76	GO:0016525	0.03543
M_76	GO:0001706	0.03549
M_76	GO:0034333	0.03555
M_76	GO:2000320	0.0359
M_76	GO:0010944	0.0359
M_76	GO:0033689	0.0359
M_76	GO:0086015	0.0359
M_76	GO:0010739	0.0359
M_76	GO:0048617	0.0359
M_76	GO:0043406	0.03679
M_76	GO:0003184	0.03926
M_76	GO:0048340	0.03981
M_76	GO:0003183	0.03981
M_76	GO:1901203	0.03981
M_76	GO:0031053	0.03981
M_76	GO:0009629	0.03981
M_76	GO:2001214	0.03981
M_76	GO:0045666	0.04274
M_76	GO:0014870	0.0437
M_76	GO:0016264	0.0437

M_76	GO:0003198	0.0437
M_76	GO:0010454	0.0437
M_76	GO:0072520	0.0437
M_76	GO:0050713	0.0437
M_76	GO:0071340	0.0437
M_76	GO:0060736	0.0437
M_76	GO:0046007	0.0437
M_76	GO:0009887	0.04664
M_76	GO:0035050	0.04709
M_76	GO:1902806	0.04731
M_76	GO:0042340	0.04758
M_76	GO:0071107	0.04758
M_76	GO:0051775	0.04758
M_76	GO:0003161	0.04758
M_76	GO:0010455	0.04758
M_76	GO:0045779	0.04758
M_76	GO:2001204	0.04758
M_76	GO:0045078	0.04758
M_77	GO:0010792	0.00019
M_77	GO:0048013	0.00299
M_77	GO:1902206	0.0036
M_77	GO:1900753	0.0036
M_77	GO:0032775	0.0036
M_77	GO:0070104	0.0036
M_77	GO:0090648	0.0036
M_77	GO:1990786	0.0036
M_77	GO:0060821	0.0036
M_77	GO:0001835	0.0036
M_77	GO:0072755	0.0036
M_77	GO:0048297	0.0036
M_77	GO:1990570	0.0036
M_77	GO:1902215	0.0036

M_77	GO:1902233	0.0036
M_77	GO:0061025	0.0066
M_77	GO:0003091	0.00702
M_77	GO:0071264	0.00718
M_77	GO:0060733	0.00718
M_77	GO:0097338	0.00718
M_77	GO:0003165	0.00718
M_77	GO:0036269	0.00718
M_77	GO:0090222	0.00718
M_77	GO:1902227	0.00718
M_77	GO:0007080	0.00741
M_77	GO:0032258	0.01075
M_77	GO:0003383	0.01075
M_77	GO:1903070	0.01075
M_77	GO:0034421	0.01075
M_77	GO:0060548	0.01204
M_77	GO:0006888	0.01329
M_77	GO:0006021	0.01431
M_77	GO:1903899	0.01431
M_77	GO:2000562	0.01431
M_77	GO:0039534	0.01431
M_77	GO:2000587	0.01431
M_77	GO:1903347	0.01431
M_77	GO:1902202	0.01431
M_77	GO:0010389	0.0168
M_77	GO:0030242	0.01785
M_77	GO:0044791	0.01785
M_77	GO:0044828	0.01785
M_77	GO:2000002	0.01785
M_77	GO:0022614	0.01785
M_77	GO:0070940	0.01785
M_77	GO:0002074	0.01785

M_77	GO:0071494	0.01785
M_77	GO:0035509	0.01785
M_77	GO:0086073	0.02139
M_77	GO:0018242	0.02139
M_77	GO:1900223	0.02139
M_77	GO:0015918	0.02462
M_77	GO:1902866	0.02491
M_77	GO:0060013	0.02491
M_77	GO:0045650	0.02491
M_77	GO:0044829	0.02491
M_77	GO:0060336	0.02491
M_77	GO:0018243	0.02491
M_77	GO:0046784	0.02841
M_77	GO:0071578	0.02841
M_77	GO:0039536	0.02841
M_77	GO:0051045	0.02841
M_77	GO:2000480	0.02841
M_77	GO:0090394	0.03191
M_77	GO:0034199	0.03191
M_77	GO:1904263	0.03191
M_77	GO:1902430	0.03191
M_77	GO:0060339	0.03191
M_77	GO:0060159	0.03191
M_77	GO:1990253	0.03539
M_77	GO:0036444	0.03539
M_77	GO:0002934	0.03539
M_77	GO:1900242	0.03539
M_77	GO:0071346	0.0368
M_77	GO:0008206	0.03833
M_77	GO:0030241	0.03886
M_77	GO:0098911	0.03886
M_77	GO:0032060	0.03886

M_77	GO:0051451	0.03886
M_77	GO:0090331	0.03886
M_77	GO:0031953	0.03886
M_77	GO:0032463	0.03886
M_77	GO:0071468	0.03886
M_77	GO:0042532	0.03886
M_77	GO:1903140	0.04232
M_77	GO:0034982	0.04232
M_77	GO:0097201	0.04232
M_77	GO:1902237	0.04232
M_77	GO:1990830	0.04248
M_77	GO:0032436	0.04332
M_77	GO:0051315	0.04577
M_77	GO:0034497	0.04577
M_77	GO:0030240	0.0492
M_77	GO:0046855	0.0492
M_77	GO:0016540	0.0492
M_77	GO:0043248	0.0492
M_77	GO:0045722	0.0492
M_78	GO:0043252	0.000081
M_78	GO:0015721	0.00014
M_78	GO:1902225	0.00325
M_78	GO:1901253	0.0065
M_78	GO:0018002	0.0065
M_78	GO:0017198	0.0065
M_78	GO:0035378	0.0065
M_78	GO:0007283	0.00682
M_78	GO:2000866	0.00973
M_78	GO:0007500	0.00973
M_78	GO:0033602	0.00973
M_78	GO:0010501	0.00982
M_78	GO:0018343	0.01295

M_78	GO:0051919	0.01295
M_78	GO:0007525	0.01295
M_78	GO:0031999	0.01295
M_78	GO:1903237	0.01295
M_78	GO:0002677	0.01295
M_78	GO:0006710	0.01295
M_78	GO:1990511	0.01616
M_78	GO:0045759	0.01937
M_78	GO:0010760	0.01937
M_78	GO:1903862	0.01937
M_78	GO:0097647	0.01937
M_78	GO:0006880	0.01937
M_78	GO:0061370	0.02256
M_78	GO:0009253	0.02256
M_78	GO:0038171	0.02256
M_78	GO:0000160	0.02256
M_78	GO:0060405	0.02256
M_78	GO:0045162	0.02256
M_78	GO:1900246	0.02256
M_78	GO:0031622	0.02256
M_78	GO:0002866	0.02256
M_78	GO:0051001	0.02256
M_78	GO:0030317	0.02298
M_78	GO:0030154	0.02352
M_78	GO:0038007	0.02574
M_78	GO:0043045	0.02574
M_78	GO:0071420	0.02574
M_78	GO:0010739	0.02891
M_78	GO:0070327	0.02891
M_78	GO:0060586	0.02891
M_78	GO:0021859	0.02891
M_78	GO:0007601	0.02947

M_78	GO:1902476	0.03262
M_78	GO:0032463	0.03522
M_78	GO:1900004	0.03522
M_78	GO:0060736	0.03522
M_78	GO:0033004	0.03522
M_78	GO:0045779	0.03836
M_78	GO:0006069	0.03836
M_78	GO:0018298	0.04149
M_78	GO:0006703	0.04149
M_78	GO:0032725	0.04149
M_78	GO:0019233	0.04202
M_78	GO:0006873	0.04461
M_78	GO:0016998	0.04462
M_78	GO:0007218	0.04588
M_78	GO:1902187	0.04773
M_78	GO:2000479	0.04773
M_79	GO:0050911	7.8E-19
M_79	GO:0007186	5.9E-13
M_79	GO:0051552	3.9E-10
M_79	GO:0052696	3.2E-09
M_79	GO:0052697	8.4E-09
M_79	GO:0042573	0.000018
M_79	GO:0009804	0.00015
M_79	GO:0005998	0.00234
M_79	GO:0060470	0.00234
M_79	GO:0001582	0.00468
M_79	GO:1903860	0.00468
M_79	GO:0042732	0.007
M_79	GO:0050717	0.00933
M_79	GO:0060011	0.00933
M_79	GO:0042699	0.01165
M_79	GO:0019640	0.01165

M_79	GO:0048733	0.01627
M_79	GO:0006701	0.01627
M_79	GO:1900426	0.02087
M_79	GO:2000318	0.02316
M_79	GO:0046325	0.03228
M_79	GO:2000484	0.03455
M_79	GO:0045780	0.03907
M_79	GO:0002827	0.03907
M_79	GO:0030828	0.04356
M_79	GO:0046716	0.0458
M_80	GO:0048490	0.00092
M_80	GO:0032438	0.00193
M_80	GO:0002626	0.00251
M_80	GO:0051638	0.00251
M_80	GO:0036047	0.00251
M_80	GO:0036049	0.00251
M_80	GO:0010106	0.00251
M_80	GO:0045016	0.00251
M_80	GO:1904283	0.00251
M_80	GO:0070926	0.00251
M_80	GO:0061699	0.00251
M_80	GO:0008089	0.00291
M_80	GO:0008033	0.00458
M_80	GO:0006282	0.00479
M_80	GO:0060816	0.00502
M_80	GO:0042351	0.00502
M_80	GO:0035600	0.00502
M_80	GO:0060545	0.00502
M_80	GO:1904434	0.00502
M_80	GO:1904437	0.00502
M_80	GO:0075732	0.00502
M_80	GO:1902559	0.00502

M_80	GO:1990145	0.00502
M_80	GO:0046832	0.00502
M_80	GO:1903507	0.00575
M_80	GO:2000813	0.00752
M_80	GO:1901026	0.00752
M_80	GO:1900390	0.00752
M_80	GO:0071163	0.00752
M_80	GO:0001927	0.00752
M_80	GO:1990641	0.00752
M_80	GO:0071896	0.01001
M_80	GO:2001186	0.0125
M_80	GO:0006432	0.0125
M_80	GO:0070345	0.0125
M_80	GO:0030174	0.01498
M_80	GO:0033567	0.01498
M_80	GO:0086073	0.01498
M_80	GO:0050942	0.01498
M_80	GO:0050428	0.01498
M_80	GO:0097527	0.01498
M_80	GO:2000535	0.01745
M_80	GO:0019673	0.01745
M_80	GO:0044351	0.01745
M_80	GO:0034720	0.01992
M_80	GO:0060155	0.01992
M_80	GO:0098711	0.01992
M_80	GO:0036289	0.01992
M_80	GO:0002725	0.01992
M_80	GO:1990000	0.01992
M_80	GO:0003223	0.01992
M_80	GO:0060333	0.02147
M_80	GO:0002710	0.02189
M_80	GO:0060586	0.02238

M_80	GO:0060159	0.02238
M_80	GO:0071550	0.02238
M_80	GO:2000234	0.02238
M_80	GO:0000733	0.02238
M_80	GO:0046598	0.02238
M_80	GO:0002934	0.02484
M_80	GO:0051561	0.02484
M_80	GO:0035646	0.02484
M_80	GO:0036444	0.02484
M_80	GO:0051639	0.02484
M_80	GO:0051292	0.02484
M_80	GO:0071281	0.02484
M_80	GO:1902018	0.02484
M_80	GO:2000774	0.02484
M_80	GO:0006139	0.02491
M_80	GO:1900121	0.02729
M_80	GO:0098911	0.02729
M_80	GO:0009225	0.02911
M_80	GO:0035563	0.02974
M_80	GO:0010566	0.03217
M_80	GO:0042271	0.03217
M_80	GO:0006089	0.03217
M_80	GO:0045651	0.03217
M_80	GO:0046415	0.03217
M_80	GO:0043248	0.03461
M_80	GO:2000059	0.03461
M_80	GO:0043517	0.03461
M_80	GO:0043506	0.03635
M_80	GO:2001032	0.03703
M_80	GO:0033540	0.03703
M_80	GO:0001956	0.03703
M_80	GO:1902187	0.03703

M_80	GO:0000731	0.03945
M_80	GO:0044030	0.04428
M_80	GO:0050860	0.04428
M_80	GO:0006349	0.04428
M_80	GO:0046597	0.04428
M_80	GO:0034138	0.04668
M_80	GO:0051092	0.04761
M_80	GO:0006783	0.04908
M_80	GO:0010667	0.04908
M_80	GO:0043046	0.04908
M_81	GO:0051282	0.0032
M_81	GO:0071615	0.0033
M_81	GO:0009403	0.0033
M_81	GO:0070407	0.0033
M_81	GO:0003058	0.0033
M_81	GO:0046105	0.0033
M_81	GO:0002127	0.0033
M_81	GO:0033572	0.0061
M_81	GO:0008615	0.0065
M_81	GO:0000349	0.0065
M_81	GO:0097274	0.0065
M_81	GO:0098840	0.0065
M_81	GO:0006649	0.0065
M_81	GO:0030890	0.0079
M_81	GO:0007586	0.0089
M_81	GO:0000375	0.0094
M_81	GO:0045218	0.0097
M_81	GO:0044375	0.0097
M_81	GO:0031296	0.0097
M_81	GO:0019087	0.0097
M_81	GO:0003099	0.0097
M_81	GO:0060585	0.0097

M_81	GO:0002943	0.013
M_81	GO:0030951	0.013
M_81	GO:0048295	0.013
M_81	GO:0046452	0.013
M_81	GO:0018894	0.013
M_81	GO:0035814	0.013
M_81	GO:0014826	0.013
M_81	GO:0045054	0.013
M_81	GO:0021943	0.013
M_81	GO:0030163	0.0132
M_81	GO:0006914	0.0145
M_81	GO:0010044	0.0162
M_81	GO:0010989	0.0162
M_81	GO:1904306	0.0162
M_81	GO:0043305	0.0194
M_81	GO:0016098	0.0194
M_81	GO:0003100	0.0194
M_81	GO:1990535	0.0194
M_81	GO:0042795	0.0218
M_81	GO:0009820	0.0226
M_81	GO:0006564	0.0226
M_81	GO:0046604	0.0226
M_81	GO:0097500	0.0226
M_81	GO:0009744	0.0226
M_81	GO:0060267	0.0257
M_81	GO:0015889	0.0257
M_81	GO:0046654	0.0257
M_81	GO:0014824	0.0257
M_81	GO:0071277	0.0267
M_81	GO:2001135	0.0289
M_81	GO:0021830	0.0289
M_81	GO:0015866	0.0289

M_81	GO:0097267	0.0289
M_81	GO:0031017	0.0289
M_81	GO:2000628	0.0289
M_81	GO:0044267	0.032
M_81	GO:0044539	0.0321
M_81	GO:0010459	0.0321
M_81	GO:0002638	0.0321
M_81	GO:1902018	0.0321
M_81	GO:0036444	0.0321
M_81	GO:0071467	0.0346
M_81	GO:0015867	0.0352
M_81	GO:0030656	0.0352
M_81	GO:0071468	0.0352
M_81	GO:0016559	0.0384
M_81	GO:0032099	0.0384
M_81	GO:0060965	0.0384
M_81	GO:0048711	0.0384
M_81	GO:0045019	0.0384
M_81	GO:0051056	0.0394
M_81	GO:0050665	0.0415
M_81	GO:0070986	0.0415
M_81	GO:0018298	0.0415
M_81	GO:2000194	0.0415
M_81	GO:0003084	0.0415
M_81	GO:0045198	0.0415
M_81	GO:0045055	0.0444
M_81	GO:1990126	0.0446
M_81	GO:0030238	0.0446
M_81	GO:0061158	0.0446
M_81	GO:0070989	0.0477
M_81	GO:0090136	0.0477
M_81	GO:1903358	0.0477

M_82	GO:1902871	0.000012
M_82	GO:0021893	0.000012
M_82	GO:0021882	0.000012
M_82	GO:0061621	0.000092
M_82	GO:0043456	0.00012
M_82	GO:0046533	0.00018
M_82	GO:0018401	0.00034
M_82	GO:0040015	0.00066
M_82	GO:0048715	0.00079
M_82	GO:0010827	0.00177
M_82	GO:0022900	0.00214
M_82	GO:0021544	0.00297
M_82	GO:0006094	0.00317
M_82	GO:0035795	0.00348
M_82	GO:0019310	0.00354
M_82	GO:0003327	0.00354
M_82	GO:0060580	0.00354
M_82	GO:0042843	0.00354
M_82	GO:1903572	0.00354
M_82	GO:0046293	0.00354
M_82	GO:0046835	0.00379
M_82	GO:0009954	0.00536
M_82	GO:0044210	0.00707
M_82	GO:0038190	0.00707
M_82	GO:0097533	0.00707
M_82	GO:0003326	0.00707
M_82	GO:0003329	0.00707
M_82	GO:1902336	0.00707
M_82	GO:0003169	0.00707
M_82	GO:0060400	0.00707
M_82	GO:0045746	0.00719
M_82	GO:0060948	0.01058

M_82	GO:0038091	0.01058
M_82	GO:0071586	0.01058
M_82	GO:0030327	0.01058
M_82	GO:0008292	0.01058
M_82	GO:0005989	0.01058
M_82	GO:0021530	0.01058
M_82	GO:0007595	0.01203
M_82	GO:2000378	0.01252
M_82	GO:0007269	0.01265
M_82	GO:0071279	0.01408
M_82	GO:0048549	0.01408
M_82	GO:0007290	0.01408
M_82	GO:0002949	0.01408
M_82	GO:0060319	0.01408
M_82	GO:0070837	0.01408
M_82	GO:1904016	0.01408
M_82	GO:1902109	0.01408
M_82	GO:0006998	0.01736
M_82	GO:1900086	0.01757
M_82	GO:0006003	0.01757
M_82	GO:0097411	0.01757
M_82	GO:0061419	0.01757
M_82	GO:0038033	0.01757
M_82	GO:0002023	0.01757
M_82	GO:2000467	0.01757
M_82	GO:0035694	0.01757
M_82	GO:0072656	0.01757
M_82	GO:1990144	0.02105
M_82	GO:0010637	0.02105
M_82	GO:0060754	0.02105
M_82	GO:0006015	0.02105
M_82	GO:0008626	0.02105

M_82	GO:1901727	0.02105
M_82	GO:0000389	0.02105
M_82	GO:1901374	0.02452
M_82	GO:0035280	0.02452
M_82	GO:0097475	0.02452
M_82	GO:0010940	0.02452
M_82	GO:0048102	0.02452
M_82	GO:0036123	0.02452
M_82	GO:1903599	0.02452
M_82	GO:0043983	0.02452
M_82	GO:0031077	0.02452
M_82	GO:0048842	0.02452
M_82	GO:0008154	0.02784
M_82	GO:0045607	0.02797
M_82	GO:1903715	0.02797
M_82	GO:0070900	0.02797
M_82	GO:0010917	0.02797
M_82	GO:0007042	0.02797
M_82	GO:0006450	0.02797
M_82	GO:0044154	0.02797
M_82	GO:0090037	0.02797
M_82	GO:0010510	0.02797
M_82	GO:0051156	0.03044
M_82	GO:0071260	0.03116
M_82	GO:0021766	0.03116
M_82	GO:0060982	0.03141
M_82	GO:0042908	0.03141
M_82	GO:0002084	0.03141
M_82	GO:0071679	0.03141
M_82	GO:0035860	0.03141
M_82	GO:0090286	0.03141
M_82	GO:1903589	0.03141

M_82	GO:0048755	0.03141
M_82	GO:0051573	0.03141
M_82	GO:0006096	0.03274
M_82	GO:0006468	0.03462
M_82	GO:1902093	0.03484
M_82	GO:0010666	0.03484
M_82	GO:0043653	0.03484
M_82	GO:0043129	0.03484
M_82	GO:0051561	0.03484
M_82	GO:0042593	0.03668
M_82	GO:0042475	0.03804
M_82	GO:0032429	0.03826
M_82	GO:0071474	0.03826
M_82	GO:0019852	0.03826
M_82	GO:0021554	0.03826
M_82	GO:0010469	0.04063
M_82	GO:1990830	0.04127
M_82	GO:0045475	0.04166
M_82	GO:0035278	0.04166
M_82	GO:0051764	0.04166
M_82	GO:2001256	0.04166
M_82	GO:0008637	0.04461
M_82	GO:0033169	0.04844
M_82	GO:0031054	0.04844
M_82	GO:0045717	0.04844
M_82	GO:0050930	0.04844
M_83	GO:0050911	1.1E-26
M_83	GO:0007186	1.8E-14
M_83	GO:0015671	0.0008
M_83	GO:0006957	0.0008
M_83	GO:0007321	0.003
M_83	GO:0060695	0.003

M_83	GO:0042744	0.0039
M_83	GO:0006583	0.006
M_83	GO:0060621	0.006
M_83	GO:0019835	0.0068
M_83	GO:0030718	0.009
M_83	GO:0010903	0.009
M_83	GO:0060164	0.012
M_83	GO:0006726	0.012
M_83	GO:0046340	0.012
M_83	GO:0021764	0.012
M_83	GO:0000711	0.012
M_83	GO:0021960	0.015
M_83	GO:0002740	0.018
M_83	GO:0071947	0.018
M_83	GO:0007506	0.021
M_83	GO:0030300	0.021
M_83	GO:0006413	0.0217
M_83	GO:0034371	0.024
M_83	GO:0007601	0.0244
M_83	GO:0042989	0.0269
M_83	GO:0070327	0.0269
M_83	GO:0045416	0.0269
M_83	GO:0002118	0.0269
M_83	GO:0010873	0.0269
M_83	GO:0034378	0.0299
M_83	GO:0048712	0.0299
M_83	GO:0031424	0.0303
M_83	GO:0045835	0.0328
M_83	GO:0018158	0.0357
M_83	GO:0034384	0.0357
M_83	GO:0018206	0.0357
M_83	GO:0033700	0.0357

M_83	GO:0021819	0.0386
M_83	GO:0034374	0.0386
M_83	GO:0006662	0.0386
M_83	GO:0051291	0.0409
M_83	GO:0001675	0.0415
M_83	GO:0030449	0.0423
M_83	GO:0060192	0.0444
M_83	GO:0034380	0.0444
M_83	GO:1900153	0.0473
M_83	GO:0021542	0.0473
M_84	GO:0006537	0.00007
M_84	GO:0006538	0.00025
M_84	GO:0016540	0.00063
M_84	GO:0048704	0.00135
M_84	GO:0045745	0.00172
M_84	GO:0009952	0.00238
M_84	GO:0061374	0.00268
M_84	GO:0000053	0.00268
M_84	GO:0070684	0.00268
M_84	GO:0061381	0.00268
M_84	GO:0070268	0.00336
M_84	GO:0061379	0.00536
M_84	GO:0071418	0.00536
M_84	GO:0002803	0.00803
M_84	GO:1900106	0.00803
M_84	GO:0071400	0.00803
M_84	GO:0007412	0.00803
M_84	GO:0021615	0.01069
M_84	GO:0061030	0.01069
M_84	GO:0010046	0.01069
M_84	GO:0021855	0.01069
M_84	GO:0071934	0.01069

M_84	GO:0045329	0.01334
M_84	GO:0001992	0.01334
M_84	GO:0072719	0.01334
M_84	GO:0009056	0.01354
M_84	GO:2001023	0.01599
M_84	GO:0006526	0.01599
M_84	GO:0031642	0.01599
M_84	GO:0010966	0.01599
M_84	GO:0008344	0.01726
M_84	GO:0010764	0.01863
M_84	GO:0042723	0.01863
M_84	GO:0033504	0.01863
M_84	GO:0071499	0.01863
M_84	GO:0010898	0.02127
M_84	GO:0071493	0.02127
M_84	GO:0060539	0.02389
M_84	GO:0070997	0.02641
M_84	GO:0030948	0.02651
M_84	GO:0000050	0.02913
M_84	GO:0006531	0.02913
M_84	GO:0051386	0.02913
M_84	GO:0019532	0.03173
M_84	GO:0007494	0.03173
M_84	GO:0060272	0.03173
M_84	GO:0048681	0.03173
M_84	GO:1903818	0.03173
M_84	GO:0006805	0.034
M_84	GO:0000052	0.03433
M_84	GO:0006703	0.03433
M_84	GO:0048935	0.03433
M_84	GO:0030213	0.03433
M_84	GO:0032310	0.03433

M_84	GO:0021794	0.03692
M_84	GO:1902358	0.03692
M_84	GO:0030207	0.03692
M_84	GO:0008595	0.03951
M_84	GO:0090051	0.03951
M_84	GO:0050808	0.04568
M_84	GO:0030214	0.04723
M_85	GO:0019082	0.0021
M_85	GO:0090472	0.0021
M_85	GO:0071364	0.003
M_85	GO:0032804	0.0041
M_85	GO:0032902	0.0041
M_85	GO:0070889	0.0041
M_85	GO:2000393	0.0041
M_85	GO:0033058	0.0062
M_85	GO:0044375	0.0062
M_85	GO:0016557	0.0062
M_85	GO:1904395	0.0062
M_85	GO:0055005	0.0062
M_85	GO:0006435	0.0062
M_85	GO:0032911	0.0082
M_85	GO:1902817	0.0082
M_85	GO:1900086	0.0102
M_85	GO:0006287	0.0102
M_85	GO:0070375	0.0102
M_85	GO:0071277	0.0112
M_85	GO:0042256	0.0123
M_85	GO:0051126	0.0163
M_85	GO:0017185	0.0163
M_85	GO:0006013	0.0163
M_85	GO:0002084	0.0183
M_85	GO:0045714	0.0183

M_85	GO:0000002	0.0198
M_85	GO:0043951	0.0204
M_85	GO:0010666	0.0204
M_85	GO:0006264	0.0224
M_85	GO:0072015	0.0224
M_85	GO:0090527	0.0224
M_85	GO:0016559	0.0244
M_85	GO:0007173	0.0248
M_85	GO:0006995	0.0264
M_85	GO:1990776	0.0284
M_85	GO:0051044	0.0304
M_85	GO:0015732	0.0324
M_85	GO:0032793	0.0324
M_85	GO:0006465	0.0324
M_85	GO:0051127	0.0344
M_85	GO:0048311	0.0344
M_85	GO:0006198	0.0364
M_85	GO:0048490	0.0364
M_85	GO:1903206	0.0364
M_85	GO:2000251	0.0364
M_85	GO:0048041	0.0374
M_85	GO:0043687	0.0381
M_85	GO:1902170	0.0383
M_85	GO:0006491	0.0383
M_85	GO:0036120	0.0403
M_85	GO:0051004	0.0403
M_85	GO:0043043	0.0421
M_85	GO:0097320	0.0423
M_85	GO:0050995	0.0423
M_85	GO:0055093	0.0423
M_85	GO:1900745	0.0423
M_85	GO:0045663	0.0423

M_85	GO:0032418	0.0432
M_85	GO:2000145	0.0477
M_85	GO:0010499	0.0482
M_85	GO:0051894	0.0482
M_86	GO:2000646	0.00033
M_86	GO:0006777	0.00049
M_86	GO:0016255	0.00049
M_86	GO:0070131	0.00175
M_86	GO:0019510	0.00577
M_86	GO:0032447	0.00577
M_86	GO:0018192	0.00577
M_86	GO:0071171	0.00577
M_86	GO:0071268	0.00577
M_86	GO:1902979	0.00577
M_86	GO:0046111	0.00577
M_86	GO:0015786	0.00577
M_86	GO:0060407	0.00577
M_86	GO:0060169	0.00577
M_86	GO:0045225	0.00577
M_86	GO:0006157	0.00577
M_86	GO:0006781	0.00577
M_86	GO:0042795	0.0077
M_86	GO:0016032	0.00909
M_86	GO:0042745	0.0114
M_86	GO:0034970	0.0115
M_86	GO:0035600	0.0115
M_86	GO:1990264	0.0115
M_86	GO:0018283	0.0115
M_86	GO:0075732	0.0115
M_86	GO:1903033	0.0115
M_86	GO:0070256	0.0115
M_86	GO:0036511	0.0115

M_86	GO:0036512	0.0115
M_86	GO:0036509	0.0115
M_86	GO:0042323	0.0115
M_86	GO:0046627	0.01459
M_86	GO:0000398	0.01516
M_86	GO:0070423	0.01547
M_86	GO:0045292	0.01638
M_86	GO:0071930	0.0172
M_86	GO:0002669	0.0172
M_86	GO:0002636	0.0172
M_86	GO:1904154	0.0172
M_86	GO:2000405	0.0172
M_86	GO:0031134	0.0172
M_86	GO:0030576	0.0172
M_86	GO:0002314	0.0172
M_86	GO:0046103	0.0172
M_86	GO:0045903	0.0172
M_86	GO:0015729	0.0172
M_86	GO:0015709	0.0172
M_86	GO:0044571	0.0172
M_86	GO:0035720	0.0172
M_86	GO:1902037	0.0172
M_86	GO:0006154	0.0172
M_86	GO:0046061	0.0172
M_86	GO:0036510	0.0172
M_86	GO:1902626	0.0172
M_86	GO:0071423	0.0172
M_86	GO:0023052	0.0178
M_86	GO:0030968	0.01904
M_86	GO:0006891	0.01922
M_86	GO:0035308	0.02277
M_86	GO:0071922	0.02286

M_86	GO:0043103	0.02286
M_86	GO:0051572	0.02286
M_86	GO:1902202	0.02286
M_86	GO:0002143	0.02286
M_86	GO:0002906	0.02286
M_86	GO:1902035	0.02286
M_86	GO:0033353	0.02286
M_86	GO:0006231	0.02286
M_86	GO:1904382	0.02286
M_86	GO:0043328	0.02286
M_86	GO:0070085	0.02698
M_86	GO:0070525	0.02823
M_86	GO:0060501	0.0285
M_86	GO:0019348	0.0285
M_86	GO:0090230	0.0285
M_86	GO:1990592	0.0285
M_86	GO:0009597	0.0285
M_86	GO:1903898	0.0285
M_86	GO:0070940	0.0285
M_86	GO:0006226	0.0285
M_86	GO:0035519	0.0285
M_86	GO:0071422	0.0285
M_86	GO:0070534	0.03007
M_86	GO:0030154	0.03129
M_86	GO:0046642	0.0341
M_86	GO:0017196	0.0341
M_86	GO:0043985	0.0341
M_86	GO:0042985	0.0341
M_86	GO:0042256	0.0341
M_86	GO:0002439	0.0341
M_86	GO:2000675	0.0341
M_86	GO:0045652	0.03494

M_86	GO:0007059	0.03524
M_86	GO:1901796	0.03566
M_86	GO:0032868	0.03567
M_86	GO:0045617	0.03967
M_86	GO:0000972	0.03967
M_86	GO:0035356	0.03967
M_86	GO:0006564	0.03967
M_86	GO:0040016	0.03967
M_86	GO:0035948	0.03967
M_86	GO:1903896	0.03967
M_86	GO:0043353	0.03967
M_86	GO:0010890	0.03967
M_86	GO:0006355	0.04401
M_86	GO:0051865	0.04415
M_86	GO:0000492	0.04521
M_86	GO:2000480	0.04521
M_86	GO:0016926	0.04521
M_86	GO:0032261	0.04521
M_86	GO:1903265	0.04521
M_86	GO:0019919	0.04521
M_86	GO:0006449	0.04521
M_86	GO:0070900	0.04521
M_86	GO:0006987	0.04521
M_86	GO:0033632	0.04521
M_86	GO:0036066	0.04521
M_86	GO:0070244	0.04521
M_86	GO:0048541	0.04521
M_86	GO:0045842	0.04521
M_86	GO:0001947	0.04553
M_87	GO:0045747	0.0017
M_87	GO:0042761	0.0019
M_87	GO:0035338	0.0036

M_87	GO:0032981	0.0041
M_87	GO:1902583	0.005
M_87	GO:0019353	0.005
M_87	GO:1901639	0.005
M_87	GO:0035863	0.005
M_87	GO:0032468	0.005
M_87	GO:1903676	0.005
M_87	GO:0032482	0.0064
M_87	GO:0000045	0.0099
M_87	GO:2000233	0.01
M_87	GO:0046709	0.01
M_87	GO:2000077	0.01
M_87	GO:0007174	0.01
M_87	GO:0000964	0.01
M_87	GO:2000616	0.01
M_87	GO:0032472	0.01
M_87	GO:0034087	0.01
M_87	GO:1902954	0.01
M_87	GO:0001113	0.01
M_87	GO:0071109	0.01
M_87	GO:0034080	0.0106
M_87	GO:0006270	0.0148
M_87	GO:0006891	0.0148
M_87	GO:1901984	0.0148
M_87	GO:0030961	0.015
M_87	GO:0019391	0.015
M_87	GO:0044205	0.015
M_87	GO:1904339	0.015
M_87	GO:0039019	0.015
M_87	GO:1903288	0.015
M_87	GO:0016077	0.015
M_87	GO:0036493	0.015

M_87	GO:1903542	0.015
M_87	GO:2000466	0.015
M_87	GO:1903679	0.015
M_87	GO:0000350	0.015
M_87	GO:0006643	0.0195
M_87	GO:1990180	0.0199
M_87	GO:0071962	0.0199
M_87	GO:0060018	0.0199
M_87	GO:0090383	0.0199
M_87	GO:1903278	0.0199
M_87	GO:1903912	0.0199
M_87	GO:0060800	0.0199
M_87	GO:1903595	0.0199
M_87	GO:1990441	0.0199
M_87	GO:0043967	0.0202
M_87	GO:1900034	0.0206
M_87	GO:0030497	0.0246
M_87	GO:0071806	0.0247
M_87	GO:0030242	0.0249
M_87	GO:0006269	0.0249
M_87	GO:0060023	0.0249
M_87	GO:0048496	0.0249
M_87	GO:1903898	0.0249
M_87	GO:0098535	0.0249
M_87	GO:0006528	0.0249
M_87	GO:1990592	0.0249
M_87	GO:0060633	0.0249
M_87	GO:0036016	0.0249
M_87	GO:0007029	0.0252
M_87	GO:0016925	0.0271
M_87	GO:0006207	0.0298
M_87	GO:0060770	0.0298

M_87	GO:0033278	0.0298
M_87	GO:0045719	0.0298
M_87	GO:0018242	0.0298
M_87	GO:0061724	0.0298
M_87	GO:0000389	0.0298
M_87	GO:0051310	0.0302
M_87	GO:0006888	0.0323
M_87	GO:0006968	0.0344
M_87	GO:0019367	0.0346
M_87	GO:0006768	0.0346
M_87	GO:0048318	0.0346
M_87	GO:0034625	0.0346
M_87	GO:0034626	0.0346
M_87	GO:0042760	0.0346
M_87	GO:0040016	0.0346
M_87	GO:0006382	0.0346
M_87	GO:0010890	0.0346
M_87	GO:0051597	0.0346
M_87	GO:1901030	0.0391
M_87	GO:0000492	0.0395
M_87	GO:0051683	0.0395
M_87	GO:0090385	0.0395
M_87	GO:0007172	0.0395
M_87	GO:0022615	0.0395
M_87	GO:0090481	0.0395
M_87	GO:0006616	0.0395
M_87	GO:1901029	0.0395
M_87	GO:0034720	0.0395
M_87	GO:0045008	0.0395
M_87	GO:0001514	0.0443
M_87	GO:0017085	0.0443
M_87	GO:0071679	0.0443

M_87	GO:0050821	0.0467
M_87	GO:0042770	0.0489
M_87	GO:0006930	0.0491
M_87	GO:0043653	0.0491
M_87	GO:0048280	0.0491
M_87	GO:0070544	0.0491
M_88	GO:0030838	0.0016
M_88	GO:0061623	0.0023
M_88	GO:0070078	0.0023
M_88	GO:0070079	0.0023
M_88	GO:0038192	0.0023
M_88	GO:0002432	0.0023
M_88	GO:0019402	0.0023
M_88	GO:0018395	0.0023
M_88	GO:0031001	0.0023
M_88	GO:0030302	0.0023
M_88	GO:0030974	0.0046
M_88	GO:1990264	0.0046
M_88	GO:0060266	0.0068
M_88	GO:0000379	0.0068
M_88	GO:0071934	0.0091
M_88	GO:0072757	0.0091
M_88	GO:0071442	0.0091
M_88	GO:1990414	0.0091
M_88	GO:0048499	0.0091
M_88	GO:0033499	0.0091
M_88	GO:1903753	0.0114
M_88	GO:0090335	0.0133
M_88	GO:0006164	0.0135
M_88	GO:0006015	0.0136
M_88	GO:0042723	0.0159
M_88	GO:2000535	0.0159

M_88	GO:0043320	0.0159
M_88	GO:0043654	0.0159
M_88	GO:0035635	0.0177
M_88	GO:0050847	0.0181
M_88	GO:0044387	0.0181
M_88	GO:0090336	0.0204
M_88	GO:0006887	0.0206
M_88	GO:0033148	0.0226
M_88	GO:0006983	0.0248
M_88	GO:0034244	0.0248
M_88	GO:0055074	0.027
M_88	GO:0048715	0.0271
M_88	GO:0060670	0.0271
M_88	GO:2000042	0.0271
M_88	GO:0045591	0.0271
M_88	GO:0051764	0.0271
M_88	GO:0009223	0.0293
M_88	GO:0035723	0.0293
M_88	GO:0036109	0.0293
M_88	GO:0031274	0.0293
M_88	GO:0045055	0.0309
M_88	GO:0007210	0.0315
M_88	GO:0043508	0.0315
M_88	GO:0002467	0.0337
M_88	GO:0033540	0.0337
M_88	GO:0033539	0.0337
M_88	GO:0008286	0.034
M_88	GO:0035970	0.0359
M_88	GO:0038096	0.036
M_88	GO:0061003	0.0381
M_88	GO:0043950	0.0381
M_88	GO:2000251	0.0403

M_88	GO:0006337	0.0425
M_88	GO:0032211	0.0447
M_88	GO:0002029	0.0447
M_88	GO:0046135	0.0491
M_89	GO:0006607	0.000000088
M_89	GO:0046604	0.00036
M_89	GO:2000271	0.00036
M_89	GO:0006368	0.00048
M_89	GO:2000786	0.00094
M_89	GO:0031023	0.00109
M_89	GO:1902237	0.00113
M_89	GO:0042795	0.00323
M_89	GO:0007051	0.00348
M_89	GO:0010525	0.00422
M_89	GO:0017126	0.00422
M_89	GO:0007499	0.00422
M_89	GO:0001928	0.00422
M_89	GO:0001111	0.00422
M_89	GO:0060178	0.00422
M_89	GO:0006422	0.00422
M_89	GO:0061646	0.00422
M_89	GO:2001253	0.00422
M_89	GO:0033320	0.00422
M_89	GO:0009303	0.00709
M_89	GO:0006363	0.00756
M_89	GO:0000082	0.00805
M_89	GO:0071955	0.00843
M_89	GO:0060529	0.00843
M_89	GO:0031990	0.00843
M_89	GO:2000124	0.00843
M_89	GO:1903233	0.00843
M_89	GO:0009826	0.00843

M_89	GO:0000717	0.00843
M_89	GO:0036292	0.00843
M_89	GO:0097359	0.00843
M_89	GO:0001113	0.00843
M_89	GO:1904562	0.00843
M_89	GO:0060139	0.00843
M_89	GO:1904674	0.00843
M_89	GO:0019085	0.00843
M_89	GO:0034402	0.00843
M_89	GO:0006370	0.00905
M_89	GO:0006361	0.01011
M_89	GO:0043029	0.01179
M_89	GO:0016344	0.01262
M_89	GO:0048319	0.01262
M_89	GO:0097051	0.01262
M_89	GO:0003383	0.01262
M_89	GO:0036493	0.01262
M_89	GO:0007509	0.01262
M_89	GO:1903292	0.01262
M_89	GO:0060399	0.01262
M_89	GO:1902263	0.01262
M_89	GO:0010482	0.01262
M_89	GO:0060197	0.01262
M_89	GO:0060154	0.01262
M_89	GO:0048807	0.01262
M_89	GO:0001712	0.01262
M_89	GO:0034499	0.01262
M_89	GO:0001844	0.01659
M_89	GO:1903373	0.01679
M_89	GO:1903912	0.01679
M_89	GO:1903061	0.01679
M_89	GO:0006931	0.01679

M_89	GO:1903690	0.01679
M_89	GO:0019086	0.01679
M_89	GO:1990441	0.01679
M_89	GO:0036092	0.01892
M_89	GO:0035019	0.02059
M_89	GO:0070242	0.02068
M_89	GO:0051653	0.02071
M_89	GO:1904428	0.02094
M_89	GO:2001034	0.02094
M_89	GO:0090188	0.02094
M_89	GO:1904261	0.02094
M_89	GO:0032237	0.02094
M_89	GO:1903898	0.02094
M_89	GO:0060157	0.02094
M_89	GO:0070245	0.02094
M_89	GO:0070201	0.0212
M_89	GO:0033206	0.02508
M_89	GO:0006685	0.02508
M_89	GO:0001302	0.02508
M_89	GO:0042796	0.02508
M_89	GO:0070358	0.02508
M_89	GO:0010793	0.02508
M_89	GO:0071385	0.02579
M_89	GO:0034453	0.02579
M_89	GO:0070816	0.02883
M_89	GO:0045617	0.0292
M_89	GO:0050957	0.0292
M_89	GO:1903896	0.0292
M_89	GO:0090091	0.0292
M_89	GO:0030953	0.0292
M_89	GO:0001839	0.0292
M_89	GO:0006695	0.03262

M_89	GO:0031571	0.03297
M_89	GO:2000381	0.0333
M_89	GO:0033085	0.0333
M_89	GO:0000019	0.0333
M_89	GO:0007172	0.0333
M_89	GO:0048563	0.0333
M_89	GO:0043583	0.0368
M_89	GO:0032464	0.03739
M_89	GO:0010838	0.03739
M_89	GO:0032933	0.03739
M_89	GO:0097011	0.03739
M_89	GO:0048478	0.03739
M_89	GO:1901409	0.03739
M_89	GO:0043247	0.03739
M_89	GO:0060363	0.03739
M_89	GO:0000733	0.03739
M_89	GO:0006348	0.03739
M_89	GO:0043589	0.03739
M_89	GO:0006265	0.03739
M_89	GO:0048617	0.03739
M_89	GO:0006283	0.03815
M_89	GO:0009395	0.04097
M_89	GO:0016072	0.04138
M_89	GO:0060513	0.04146
M_89	GO:0051574	0.04146
M_89	GO:1903025	0.04146
M_89	GO:0032525	0.04146
M_89	GO:0006310	0.04258
M_89	GO:0006281	0.04365
M_89	GO:0048013	0.04502
M_89	GO:0090161	0.04551
M_89	GO:0045945	0.04551

M_89	GO:0035404	0.04954
M_89	GO:0045475	0.04954
M_89	GO:0090239	0.04954
M_89	GO:2000042	0.04954
M_89	GO:2000651	0.04954
M_89	GO:0006511	0.04961
M_90	GO:0070268	5E-14
M_90	GO:0018149	1.3E-09
M_90	GO:0034116	0.000047
M_90	GO:0031639	0.00028
M_90	GO:0090188	0.00031
M_90	GO:0060662	0.00031
M_90	GO:0051258	0.00042
M_90	GO:0042730	0.00045
M_90	GO:0043152	0.00046
M_90	GO:2000352	0.0005
M_90	GO:0050801	0.0006
M_90	GO:0045907	0.00075
M_90	GO:0002576	0.00076
M_90	GO:1900026	0.00082
M_90	GO:1902042	0.00106
M_90	GO:0042908	0.00109
M_90	GO:0051592	0.00475
M_90	GO:0010951	0.00479
M_90	GO:0007010	0.00513
M_90	GO:0071693	0.00559
M_90	GO:0009949	0.00559
M_90	GO:0086030	0.00559
M_90	GO:1990708	0.00559
M_90	GO:0060782	0.00559
M_90	GO:0060783	0.00559
M_90	GO:2000227	0.00559

M_90	GO:0043063	0.00559
M_90	GO:0035238	0.00559
M_90	GO:0071412	0.00559
M_90	GO:0060683	0.00559
M_90	GO:0035874	0.00559
M_90	GO:0061189	0.00559
M_90	GO:2000062	0.00559
M_90	GO:2000063	0.00559
M_90	GO:0061106	0.00559
M_90	GO:0035623	0.00559
M_90	GO:1990401	0.00559
M_90	GO:0097185	0.00559
M_90	GO:0006037	0.00559
M_90	GO:0045977	0.00559
M_90	GO:1901810	0.00559
M_90	GO:2000357	0.00559
M_90	GO:2000358	0.00559
M_90	GO:0072377	0.00559
M_90	GO:2000971	0.00559
M_90	GO:0097485	0.00562
M_90	GO:0006855	0.00789
M_90	GO:0035902	0.00855
M_90	GO:0042060	0.00888
M_90	GO:0072378	0.00904
M_90	GO:0070374	0.00945
M_90	GO:0032414	0.01102
M_90	GO:0010766	0.01105
M_90	GO:0002729	0.01116
M_90	GO:0038161	0.01116
M_90	GO:0031635	0.01116
M_90	GO:0042137	0.01116
M_90	GO:2000729	0.01116

M_90	GO:0060516	0.01116
M_90	GO:2000707	0.01116
M_90	GO:0016539	0.01116
M_90	GO:0046331	0.01116
M_90	GO:0061153	0.01116
M_90	GO:0010512	0.01116
M_90	GO:0036345	0.01116
M_90	GO:1903937	0.01116
M_90	GO:0019376	0.01116
M_90	GO:0060327	0.01116
M_90	GO:0060458	0.01116
M_90	GO:1901899	0.01116
M_90	GO:2000978	0.01116
M_90	GO:0090277	0.01173
M_90	GO:0042593	0.012
M_90	GO:0045921	0.01282
M_90	GO:0016121	0.01669
M_90	GO:0014858	0.01669
M_90	GO:0007228	0.01669
M_90	GO:0035425	0.01669
M_90	GO:0072156	0.01669
M_90	GO:0060769	0.01669
M_90	GO:1990822	0.01669
M_90	GO:0072136	0.01669
M_90	GO:0021558	0.01669
M_90	GO:0014054	0.01669
M_90	GO:0045200	0.01669
M_90	GO:0042270	0.01669
M_90	GO:1904339	0.01669
M_90	GO:0006154	0.01669
M_90	GO:1903027	0.01669
M_90	GO:0060523	0.01669

M_90	GO:0035793	0.01669
M_90	GO:0033092	0.01669
M_90	GO:0070649	0.01669
M_90	GO:0010751	0.01669
M_90	GO:0033685	0.01669
M_90	GO:0000432	0.01669
M_90	GO:1902896	0.01669
M_90	GO:0023016	0.01669
M_90	GO:0045608	0.01669
M_90	GO:0042668	0.01669
M_90	GO:0002426	0.01669
M_90	GO:0046103	0.01669
M_90	GO:0051280	0.01669
M_90	GO:0060459	0.01669
M_90	GO:0060447	0.01669
M_90	GO:0045959	0.01669
M_90	GO:2000974	0.01669
M_90	GO:0060112	0.01669
M_90	GO:0051930	0.01817
M_90	GO:0070527	0.01972
M_90	GO:0070741	0.02201
M_90	GO:0001951	0.02219
M_90	GO:1904000	0.02219
M_90	GO:0035910	0.02219
M_90	GO:1901529	0.02219
M_90	GO:0070779	0.02219
M_90	GO:1903899	0.02219
M_90	GO:0021555	0.02219
M_90	GO:0021557	0.02219
M_90	GO:1900738	0.02219
M_90	GO:1990834	0.02219
M_90	GO:0090370	0.02219

M_90	GO:0033686	0.02219
M_90	GO:0040038	0.02219
M_90	GO:0021938	0.02219
M_90	GO:0015811	0.02219
M_90	GO:0015842	0.02219
M_90	GO:0036438	0.02219
M_90	GO:0060480	0.02219
M_90	GO:0043103	0.02219
M_90	GO:0007418	0.02219
M_90	GO:0070294	0.02219
M_90	GO:0060164	0.02219
M_90	GO:0006805	0.02271
M_90	GO:0060548	0.0248
M_90	GO:0006955	0.02499
M_90	GO:0048538	0.02516
M_90	GO:0060675	0.02748
M_90	GO:0060840	0.0275
M_90	GO:0071504	0.02766
M_90	GO:0046877	0.02766
M_90	GO:0061009	0.02766
M_90	GO:0060664	0.02766
M_90	GO:0090238	0.02766
M_90	GO:0098712	0.02766
M_90	GO:0098700	0.02766
M_90	GO:0060916	0.02766
M_90	GO:0051295	0.02766
M_90	GO:0033132	0.02766
M_90	GO:1900175	0.02766
M_90	GO:0007596	0.02798
M_90	GO:0007160	0.02893
M_90	GO:0050714	0.03142
M_90	GO:0045010	0.03253

M_90	GO:0050951	0.0328
M_90	GO:0003143	0.03288
M_90	GO:0060789	0.0331
M_90	GO:0048859	0.0331
M_90	GO:0060685	0.0331
M_90	GO:0061309	0.0331
M_90	GO:0033603	0.0331
M_90	GO:0044245	0.0331
M_90	GO:0038001	0.0331
M_90	GO:0072049	0.0331
M_90	GO:0006572	0.0331
M_90	GO:1903078	0.03427
M_90	GO:0002224	0.03577
M_90	GO:0010544	0.03815
M_90	GO:0043268	0.0382
M_90	GO:0021861	0.03851
M_90	GO:1903896	0.03851
M_90	GO:0072141	0.03851
M_90	GO:0032466	0.03851
M_90	GO:0036089	0.03851
M_90	GO:0071476	0.03851
M_90	GO:0032762	0.03851
M_90	GO:2000049	0.03851
M_90	GO:0032532	0.03851
M_90	GO:0060463	0.03851
M_90	GO:0097151	0.03851
M_90	GO:0006032	0.03851
M_90	GO:0007442	0.03851
M_90	GO:2000394	0.03851
M_90	GO:0060395	0.04051
M_90	GO:0007612	0.04136
M_90	GO:0002320	0.04348

M_90	GO:0061626	0.04389
M_90	GO:0009414	0.04389
M_90	GO:0072282	0.04389
M_90	GO:0097084	0.04389
M_90	GO:0071420	0.04389
M_90	GO:0060020	0.04389
M_90	GO:0090281	0.04389
M_90	GO:2000543	0.04389
M_90	GO:2000096	0.04389
M_90	GO:0072050	0.04389
M_90	GO:0010510	0.04389
M_90	GO:0060452	0.04389
M_90	GO:0060484	0.04389
M_90	GO:0016266	0.0458
M_90	GO:0060993	0.04799
M_90	GO:0051014	0.04924
M_90	GO:0048671	0.04924
M_90	GO:0048617	0.04924
M_90	GO:0003266	0.04924
M_90	GO:0001542	0.04924
M_90	GO:0070327	0.04924
M_90	GO:0055091	0.04924
M_90	GO:0015886	0.04924
M_90	GO:0006591	0.04924
M_90	GO:0098703	0.04924
M_90	GO:0007196	0.04924
M_90	GO:0060124	0.04924
M_90	GO:0008217	0.04958
M_91	GO:0016266	0.0015
M_91	GO:0060074	0.0022
M_91	GO:0006044	0.0022
M_91	GO:0010506	0.0031

M_91	GO:0015734	0.0037
M_91	GO:0097291	0.0037
M_91	GO:0031104	0.0037
M_91	GO:2000424	0.0037
M_91	GO:1903028	0.0037
M_91	GO:0033345	0.0037
M_91	GO:0033514	0.0037
M_91	GO:0060345	0.0037
M_91	GO:1903988	0.0037
M_91	GO:0051041	0.0037
M_91	GO:0071377	0.0038
M_91	GO:0018146	0.0052
M_91	GO:0035774	0.0055
M_91	GO:0019543	0.0074
M_91	GO:0090164	0.0074
M_91	GO:0034395	0.0074
M_91	GO:0061402	0.0074
M_91	GO:0046327	0.0074
M_91	GO:0034635	0.0074
M_91	GO:0050902	0.0074
M_91	GO:0002121	0.0074
M_91	GO:0032416	0.0074
M_91	GO:0071356	0.0085
M_91	GO:1904640	0.0111
M_91	GO:0060025	0.0111
M_91	GO:0044313	0.0111
M_91	GO:0051365	0.0111
M_91	GO:1904378	0.0111
M_91	GO:0034241	0.0111
M_91	GO:0016560	0.0148
M_91	GO:0015855	0.0148
M_91	GO:2000360	0.0148

M_91	GO:0061669	0.0148
M_91	GO:0060528	0.0148
M_91	GO:1900738	0.0148
M_91	GO:0060480	0.0148
M_91	GO:0003333	0.0171
M_91	GO:0046847	0.0184
M_91	GO:2001244	0.0184
M_91	GO:0003096	0.0184
M_91	GO:0072675	0.0184
M_91	GO:0071332	0.0184
M_91	GO:0035523	0.0184
M_91	GO:0002933	0.0221
M_91	GO:0060770	0.0221
M_91	GO:0032914	0.0221
M_91	GO:0010265	0.0221
M_91	GO:0050882	0.0221
M_91	GO:0030593	0.023
M_91	GO:0097151	0.0257
M_91	GO:1990048	0.0257
M_91	GO:0010668	0.0257
M_91	GO:0006477	0.0257
M_91	GO:0006032	0.0257
M_91	GO:0038203	0.0257
M_91	GO:1900006	0.0264
M_91	GO:0051683	0.0293
M_91	GO:0019896	0.0293
M_91	GO:0042045	0.0293
M_91	GO:0032782	0.0329
M_91	GO:0051601	0.0329
M_91	GO:0060586	0.0329
M_91	GO:0045657	0.0329
M_91	GO:0030643	0.0329

M_91	GO:0045792	0.0365
M_91	GO:0007191	0.0365
M_91	GO:0035878	0.0365
M_91	GO:0060088	0.0401
M_91	GO:0036006	0.0401
M_91	GO:0010454	0.0401
M_91	GO:0006559	0.0401
M_91	GO:0002523	0.0401
M_91	GO:0060576	0.0401
M_91	GO:2000786	0.0401
M_91	GO:0030072	0.0415
M_91	GO:0010455	0.0436
M_91	GO:0014067	0.0436
M_91	GO:0035246	0.0436
M_91	GO:0006107	0.0436
M_91	GO:1901642	0.0436
M_91	GO:0031581	0.0436
M_91	GO:1904294	0.0472
M_91	GO:0010642	0.0472
M_91	GO:0045198	0.0472
M_92	GO:0045859	0.000055
M_92	GO:0061737	0.000096
M_92	GO:0009607	0.00092
M_92	GO:1904604	0.00314
M_92	GO:1904155	0.00314
M_92	GO:1904156	0.00314
M_92	GO:1903954	0.00314
M_92	GO:1904597	0.00314
M_92	GO:0006954	0.00473
M_92	GO:1901207	0.00627
M_92	GO:1903762	0.00627
M_92	GO:0042412	0.00627

M_92	GO:0034314	0.00666
M_92	GO:1900453	0.00939
M_92	GO:0051562	0.00939
M_92	GO:0060474	0.00939
M_92	GO:0039689	0.00939
M_92	GO:0035544	0.00939
M_92	GO:1990637	0.00939
M_92	GO:0097340	0.01228
M_92	GO:0018094	0.0125
M_92	GO:2001200	0.0125
M_92	GO:1902261	0.0125
M_92	GO:0006968	0.0143
M_92	GO:0033087	0.0156
M_92	GO:0010255	0.0156
M_92	GO:0072679	0.0156
M_92	GO:2000354	0.01869
M_92	GO:0061087	0.01869
M_92	GO:0050771	0.01985
M_92	GO:0090647	0.02177
M_92	GO:0048290	0.02177
M_92	GO:0035356	0.02177
M_92	GO:0006853	0.02485
M_92	GO:2000510	0.02485
M_92	GO:1903980	0.02485
M_92	GO:0010917	0.02485
M_92	GO:0032411	0.02753
M_92	GO:0048096	0.02791
M_92	GO:1900272	0.02791
M_92	GO:0042147	0.02926
M_92	GO:0070997	0.03038
M_92	GO:0071639	0.03096
M_92	GO:0071501	0.03096

M_92	GO:2001223	0.03401
M_92	GO:0002523	0.03401
M_92	GO:0050713	0.03401
M_92	GO:1990001	0.03704
M_92	GO:0031665	0.04007
M_92	GO:0007171	0.04007
M_92	GO:0007218	0.04302
M_92	GO:0030238	0.04308
M_92	GO:0090110	0.04308
M_92	GO:0009437	0.04308
M_92	GO:0050930	0.04308
M_92	GO:0044849	0.04308
M_92	GO:0030299	0.04308
M_92	GO:0043981	0.04609
M_92	GO:0043982	0.04609
M_92	GO:0035020	0.04609
M_92	GO:0051546	0.04609
M_92	GO:0080182	0.04609
M_92	GO:0050884	0.04609
M_92	GO:0031629	0.04909
M_92	GO:0035864	0.04909
M_93	GO:0015015	0.00012
M_93	GO:0034120	0.00291
M_93	GO:0070473	0.00291
M_93	GO:0014813	0.00291
M_93	GO:0051930	0.00447
M_93	GO:0033209	0.00563
M_93	GO:0000494	0.00581
M_93	GO:1903547	0.00581
M_93	GO:1904057	0.00581
M_93	GO:0045585	0.00581
M_93	GO:0070378	0.00581

M_93	GO:0060721	0.00581
M_93	GO:1990258	0.00581
M_93	GO:1902731	0.00871
M_93	GO:0060083	0.00871
M_93	GO:0060392	0.00871
M_93	GO:0090027	0.01159
M_93	GO:0035625	0.01159
M_93	GO:0034465	0.01159
M_93	GO:1990523	0.01159
M_93	GO:0061484	0.01159
M_93	GO:0071603	0.01159
M_93	GO:1904220	0.01159
M_93	GO:0002317	0.01159
M_93	GO:0045794	0.01447
M_93	GO:0046618	0.01447
M_93	GO:0060478	0.01447
M_93	GO:0032811	0.01447
M_93	GO:0001525	0.01536
M_93	GO:0045743	0.01734
M_93	GO:0045964	0.01734
M_93	GO:2000288	0.01734
M_93	GO:0035900	0.01734
M_93	GO:0043410	0.01963
M_93	GO:0006477	0.0202
M_93	GO:0010700	0.02306
M_93	GO:1903589	0.0259
M_93	GO:0042355	0.0259
M_93	GO:0034454	0.0259
M_93	GO:0046598	0.0259
M_93	GO:0071985	0.02874
M_93	GO:0032525	0.02874
M_93	GO:1904322	0.03157

M_93	GO:0031987	0.03157
M_93	GO:0071372	0.03157
M_93	GO:0032060	0.03157
M_93	GO:0048672	0.03157
M_93	GO:0042416	0.03439
M_93	GO:0001886	0.03439
M_93	GO:0030497	0.03439
M_93	GO:0015693	0.03439
M_93	GO:0007171	0.0372
M_93	GO:0042574	0.0372
M_93	GO:0042761	0.0372
M_93	GO:1900025	0.0372
M_93	GO:0030007	0.04001
M_93	GO:0021527	0.04001
M_93	GO:0032012	0.04559
M_93	GO:0044342	0.04559
M_93	GO:1903830	0.04559
M_93	GO:0035067	0.04837
M_93	GO:0036342	0.04837
M_93	GO:0010832	0.04837
M_93	GO:0007342	0.04837
M_93	GO:0010469	0.04962
M_94	GO:0006629	0.0012
M_94	GO:0048172	0.0016
M_94	GO:0045055	0.002
M_94	GO:0045932	0.0039
M_94	GO:0021686	0.0039
M_94	GO:0032244	0.0039
M_94	GO:0001980	0.0039
M_94	GO:0032900	0.0039
M_94	GO:2000081	0.0039
M_94	GO:0048337	0.0039

M_94	GO:0010189	0.0039
M_94	GO:0040040	0.0039
M_94	GO:0021933	0.0039
M_94	GO:0061193	0.0039
M_94	GO:0003136	0.0039
M_94	GO:0045221	0.0039
M_94	GO:0021874	0.0039
M_94	GO:0033396	0.0039
M_94	GO:0008052	0.0039
M_94	GO:0060384	0.0043
M_94	GO:0009386	0.0079
M_94	GO:0070194	0.0079
M_94	GO:0048697	0.0079
M_94	GO:0042137	0.0079
M_94	GO:0007402	0.0079
M_94	GO:0048343	0.0079
M_94	GO:0006788	0.0079
M_94	GO:0070256	0.0079
M_94	GO:0021941	0.0079
M_94	GO:0097401	0.0079
M_94	GO:0002731	0.0079
M_94	GO:0019074	0.0079
M_94	GO:1901856	0.0079
M_94	GO:0042323	0.0079
M_94	GO:0007616	0.0084
M_94	GO:0050890	0.0085
M_94	GO:0060079	0.0087
M_94	GO:0051930	0.0093
M_94	GO:0030049	0.0103
M_94	GO:0060291	0.0106
M_94	GO:0010387	0.0118
M_94	GO:0046013	0.0118

M_94	GO:1904339	0.0118
M_94	GO:0045626	0.0118
M_94	GO:0000103	0.0118
M_94	GO:0031444	0.0118
M_94	GO:0097176	0.0118
M_94	GO:1900453	0.0118
M_94	GO:0061184	0.0118
M_94	GO:0045214	0.0142
M_94	GO:0008344	0.0156
M_94	GO:0061317	0.0157
M_94	GO:1903751	0.0157
M_94	GO:0009298	0.0157
M_94	GO:0030644	0.0157
M_94	GO:0035814	0.0157
M_94	GO:1901896	0.0157
M_94	GO:0015705	0.0195
M_94	GO:0032229	0.0195
M_94	GO:0055118	0.0195
M_94	GO:0044791	0.0195
M_94	GO:0038113	0.0195
M_94	GO:0098700	0.0195
M_94	GO:0033299	0.0195
M_94	GO:0090245	0.0195
M_94	GO:0030097	0.0205
M_94	GO:0060087	0.0234
M_94	GO:0033278	0.0234
M_94	GO:0042167	0.0273
M_94	GO:0006768	0.0273
M_94	GO:0000160	0.0273
M_94	GO:0014050	0.0273
M_94	GO:2000049	0.0273
M_94	GO:0014883	0.0273

M_94	GO:0035771	0.0273
M_94	GO:1900451	0.0273
M_94	GO:2000347	0.0273
M_94	GO:0035914	0.0295
M_94	GO:0036465	0.0303
M_94	GO:0038114	0.0311
M_94	GO:0006013	0.0311
M_94	GO:0070244	0.0311
M_94	GO:0040011	0.0335
M_94	GO:0032411	0.0346
M_94	GO:0006971	0.0349
M_94	GO:0006924	0.0349
M_94	GO:0045657	0.0349
M_94	GO:0016081	0.0349
M_94	GO:0051938	0.0349
M_94	GO:0044341	0.0349
M_94	GO:0021702	0.0349
M_94	GO:0031915	0.0349
M_94	GO:1900272	0.0349
M_94	GO:0035627	0.0349
M_94	GO:0016188	0.0382
M_94	GO:0043069	0.0384
M_94	GO:0043951	0.0387
M_94	GO:2001269	0.0387
M_94	GO:0090084	0.0387
M_94	GO:1900242	0.0387
M_94	GO:0010469	0.0404
M_94	GO:0007420	0.0421
M_94	GO:0006264	0.0425
M_94	GO:0071340	0.0425
M_94	GO:0003093	0.0425
M_94	GO:0007193	0.0443

M_94	GO:1902476	0.0462
M_94	GO:0002674	0.0463
M_94	GO:0006068	0.0463
M_94	GO:0006069	0.0463
M_94	GO:0038180	0.0463
M_94	GO:0038110	0.0463
M_94	GO:0003376	0.0463
M_94	GO:0043922	0.0463
M_94	GO:0045741	0.0463
M_94	GO:0006874	0.0491
M_94	GO:0001973	0.05
M_94	GO:0045651	0.05
M_94	GO:0035723	0.05
M_94	GO:0002087	0.05
M_94	GO:0060397	0.05
M_94	GO:0071688	0.05
M_94	GO:0031274	0.05
M_94	GO:0003084	0.05
M_95	GO:0035095	0.000009
M_95	GO:0048791	0.000032
M_95	GO:0060084	0.000091
M_95	GO:0042593	0.00011
M_95	GO:0060079	0.00059
M_95	GO:1903861	0.00488
M_95	GO:1903444	0.00554
M_95	GO:1902747	0.00554
M_95	GO:0032901	0.00554
M_95	GO:1902891	0.00554
M_95	GO:0003353	0.00554
M_95	GO:0018350	0.00554
M_95	GO:0097497	0.00554
M_95	GO:1903759	0.00554

M_95	GO:0010021	0.00554
M_95	GO:1901053	0.00554
M_95	GO:0021822	0.00554
M_95	GO:0002509	0.00554
M_95	GO:0044258	0.00554
M_95	GO:0034220	0.00601
M_95	GO:0007026	0.00712
M_95	GO:0007269	0.00935
M_95	GO:0007271	0.01025
M_95	GO:0009386	0.01104
M_95	GO:0002458	0.01104
M_95	GO:0097536	0.01104
M_95	GO:1901475	0.01104
M_95	GO:0002930	0.01104
M_95	GO:1903892	0.01104
M_95	GO:1902303	0.01104
M_95	GO:1902608	0.01104
M_95	GO:0006530	0.01104
M_95	GO:0071642	0.01104
M_95	GO:2001111	0.01104
M_95	GO:2000340	0.01104
M_95	GO:0071477	0.01104
M_95	GO:0019858	0.01104
M_95	GO:0003064	0.01104
M_95	GO:0016488	0.01104
M_95	GO:0030103	0.01104
M_95	GO:0042309	0.01104
M_95	GO:0007274	0.01299
M_95	GO:0008089	0.01351
M_95	GO:0003073	0.01449
M_95	GO:0031587	0.01652
M_95	GO:0014056	0.01652

M_95	GO:0007231	0.01652
M_95	GO:1990822	0.01652
M_95	GO:0006589	0.01652
M_95	GO:0031117	0.01652
M_95	GO:0051795	0.01652
M_95	GO:0010730	0.01652
M_95	GO:2000410	0.01652
M_95	GO:0061073	0.01652
M_95	GO:0086091	0.01692
M_95	GO:0017158	0.01843
M_95	GO:0048167	0.0192
M_95	GO:0007218	0.02054
M_95	GO:0007626	0.02091
M_95	GO:0043622	0.02197
M_95	GO:1990034	0.02197
M_95	GO:2000852	0.02197
M_95	GO:0018120	0.02197
M_95	GO:1900186	0.02197
M_95	GO:0050877	0.02679
M_95	GO:0050650	0.02714
M_95	GO:2000969	0.02738
M_95	GO:0051464	0.02738
M_95	GO:0008628	0.02738
M_95	GO:0086046	0.02738
M_95	GO:0002443	0.0276
M_95	GO:0006629	0.03212
M_95	GO:0032602	0.03257
M_95	GO:0042426	0.03277
M_95	GO:0006777	0.03277
M_95	GO:0071895	0.03277
M_95	GO:1902513	0.03277
M_95	GO:0021979	0.03277

M_95	GO:0051971	0.03277
M_95	GO:0038001	0.03277
M_95	GO:0071651	0.03277
M_95	GO:0071321	0.03277
M_95	GO:2000675	0.03277
M_95	GO:0048168	0.03363
M_95	GO:0032868	0.03372
M_95	GO:0031638	0.03624
M_95	GO:0016079	0.03666
M_95	GO:0042421	0.03813
M_95	GO:0000160	0.03813
M_95	GO:0023041	0.03813
M_95	GO:0097623	0.03813
M_95	GO:0097151	0.03813
M_95	GO:0003190	0.03813
M_95	GO:0002778	0.03813
M_95	GO:0051013	0.03813
M_95	GO:0033564	0.03813
M_95	GO:0006940	0.03851
M_95	GO:0007612	0.04059
M_95	GO:0007268	0.04281
M_95	GO:0045927	0.04316
M_95	GO:0042420	0.04345
M_95	GO:0098915	0.04345
M_95	GO:1902857	0.04345
M_95	GO:0044597	0.04345
M_95	GO:0044598	0.04345
M_95	GO:0042048	0.04345
M_95	GO:0038007	0.04345
M_95	GO:1901029	0.04345
M_95	GO:0030032	0.04364
M_95	GO:0032782	0.04875

M_95	GO:0090336	0.04875
M_95	GO:0061303	0.04875
M_95	GO:0022417	0.04875
M_95	GO:0002024	0.04875
M_95	GO:1990573	0.04875
M_95	GO:1902430	0.04875
M_95	GO:0031943	0.04875
M_95	GO:0008090	0.04875
M_95	GO:0061072	0.04875
M_95	GO:0070327	0.04875
M_96	GO:0006107	0.000028
M_96	GO:0060397	0.000036
M_96	GO:0035723	0.000036
M_96	GO:0038111	0.00012
M_96	GO:0046449	0.00016
M_96	GO:0006549	0.00026
M_96	GO:0000255	0.00026
M_96	GO:0045648	0.00035
M_96	GO:0031338	0.00046
M_96	GO:0019530	0.00054
M_96	GO:0043983	0.00054
M_96	GO:0046902	0.00071
M_96	GO:0006101	0.00083
M_96	GO:0006573	0.00092
M_96	GO:0071364	0.00113
M_96	GO:0006600	0.00114
M_96	GO:0090630	0.00124
M_96	GO:0015937	0.00136
M_96	GO:0038110	0.00167
M_96	GO:0006105	0.00167
M_96	GO:0007595	0.00181
M_96	GO:0043981	0.00262

M_96	GO:0043982	0.00262
M_96	GO:0043984	0.00422
M_96	GO:0042789	0.00422
M_96	GO:0006103	0.00422
M_96	GO:1901798	0.00467
M_96	GO:0030162	0.00478
M_96	GO:1900182	0.00495
M_96	GO:0035066	0.00504
M_96	GO:1903452	0.00514
M_96	GO:0071275	0.00514
M_96	GO:0046079	0.00514
M_96	GO:0046544	0.00514
M_96	GO:0006886	0.00574
M_96	GO:0007095	0.00616
M_96	GO:0016578	0.00616
M_96	GO:0031098	0.00872
M_96	GO:0046543	0.01014
M_96	GO:0071929	0.01025
M_96	GO:0033140	0.01025
M_96	GO:1902425	0.01025
M_96	GO:0072720	0.01025
M_96	GO:0072739	0.01025
M_96	GO:0048211	0.01025
M_96	GO:1902728	0.01025
M_96	GO:0018003	0.01025
M_96	GO:0018008	0.01025
M_96	GO:0048822	0.01025
M_96	GO:2001295	0.01025
M_96	GO:0006338	0.01231
M_96	GO:0040014	0.01367
M_96	GO:0050435	0.01391
M_96	GO:0031325	0.01394

M_96	GO:0007080	0.01468
M_96	GO:0006779	0.01518
M_96	GO:0045588	0.01533
M_96	GO:0007079	0.01533
M_96	GO:0032929	0.01533
M_96	GO:0032258	0.01533
M_96	GO:0035494	0.01533
M_96	GO:0031291	0.01533
M_96	GO:0006419	0.01533
M_96	GO:0000390	0.01533
M_96	GO:0010730	0.01533
M_96	GO:0006891	0.01547
M_96	GO:0031146	0.01794
M_96	GO:0007257	0.01794
M_96	GO:0045540	0.01967
M_96	GO:0010040	0.02039
M_96	GO:0072716	0.02039
M_96	GO:0070318	0.02039
M_96	GO:0002159	0.02039
M_96	GO:0061502	0.02039
M_96	GO:0071603	0.02039
M_96	GO:0060750	0.02039
M_96	GO:2000676	0.02039
M_96	GO:0006357	0.02089
M_96	GO:0006085	0.02517
M_96	GO:0072708	0.02543
M_96	GO:0090074	0.02543
M_96	GO:0071681	0.02543
M_96	GO:0060744	0.02543
M_96	GO:0042220	0.02625
M_96	GO:0000186	0.02828
M_96	GO:0043966	0.02874

M_96	GO:0015936	0.02946
M_96	GO:0006885	0.03025
M_96	GO:0070562	0.03043
M_96	GO:0042985	0.03043
M_96	GO:0000147	0.03043
M_96	GO:0003406	0.03043
M_96	GO:0060335	0.03043
M_96	GO:0002154	0.03043
M_96	GO:0051666	0.03043
M_96	GO:0071569	0.03043
M_96	GO:0086073	0.03043
M_96	GO:0060745	0.03043
M_96	GO:2000737	0.0325
M_96	GO:0006368	0.0341
M_96	GO:0043405	0.03483
M_96	GO:2000347	0.03542
M_96	GO:0040016	0.03542
M_96	GO:0051013	0.03542
M_96	GO:0090240	0.03542
M_96	GO:0035948	0.03542
M_96	GO:0032819	0.03542
M_96	GO:0035726	0.03542
M_96	GO:0030953	0.03542
M_96	GO:0043687	0.03912
M_96	GO:0044154	0.04037
M_96	GO:0006853	0.04037
M_96	GO:0046600	0.04037
M_96	GO:0045647	0.04037
M_96	GO:0010510	0.04037
M_96	GO:0038114	0.04037
M_96	GO:0072710	0.04037
M_96	GO:0006610	0.04037

M_96	GO:0032825	0.04037
M_96	GO:0006450	0.04037
M_96	GO:0048541	0.04037
M_96	GO:0044387	0.04037
M_96	GO:0030210	0.04037
M_96	GO:0030262	0.04487
M_96	GO:0032784	0.04491
M_96	GO:0048208	0.04528
M_96	GO:0048312	0.04531
M_96	GO:0033210	0.04531
M_96	GO:0038155	0.04531
M_96	GO:0001188	0.04531
M_96	GO:2001171	0.04531
M_96	GO:0033601	0.04531
M_96	GO:0030521	0.04778
M_96	GO:0031670	0.04975
M_97	GO:0006261	0.00026
M_97	GO:0070317	0.00085
M_97	GO:0043687	0.00205
M_97	GO:0032780	0.00273
M_97	GO:0045454	0.00436
M_97	GO:0006888	0.00474
M_97	GO:0048635	0.00486
M_97	GO:1901962	0.00491
M_97	GO:0021541	0.00491
M_97	GO:0010260	0.00491
M_97	GO:0002037	0.00491
M_97	GO:0035459	0.00664
M_97	GO:0007049	0.00755
M_97	GO:1990116	0.00979
M_97	GO:2000845	0.00979
M_97	GO:0070829	0.00979

M_97	GO:0000493	0.00979
M_97	GO:0090222	0.00979
M_97	GO:2000233	0.00979
M_97	GO:0006099	0.01074
M_97	GO:0045204	0.01466
M_97	GO:0007113	0.01466
M_97	GO:0006045	0.01466
M_97	GO:0000958	0.01466
M_97	GO:0030043	0.01949
M_97	GO:1904628	0.01949
M_97	GO:0044528	0.01949
M_97	GO:0038026	0.01949
M_97	GO:1990809	0.01949
M_97	GO:0006041	0.01949
M_97	GO:1903373	0.01949
M_97	GO:0034316	0.02431
M_97	GO:0097368	0.02431
M_97	GO:0006003	0.02431
M_97	GO:1900825	0.02431
M_97	GO:0000727	0.02431
M_97	GO:0043065	0.02555
M_97	GO:0051168	0.02874
M_97	GO:0034773	0.0291
M_97	GO:0070973	0.0291
M_97	GO:0002759	0.0291
M_97	GO:0010571	0.0291
M_97	GO:0072344	0.0291
M_97	GO:0006104	0.0291
M_97	GO:0061732	0.0291
M_97	GO:0090324	0.0291
M_97	GO:0031584	0.0291
M_97	GO:0008380	0.02946

M_97	GO:0000395	0.03387
M_97	GO:2000049	0.03387
M_97	GO:0018023	0.03823
M_97	GO:0045039	0.03861
M_97	GO:0072321	0.03861
M_97	GO:0010917	0.03861
M_97	GO:0046784	0.03861
M_97	GO:0048678	0.03946
M_97	GO:0022417	0.04333
M_97	GO:0034454	0.04333
M_97	GO:0070050	0.04333
M_97	GO:0097428	0.04333
M_97	GO:0060996	0.04715
M_97	GO:0006388	0.04803
M_97	GO:0032275	0.04803
M_97	GO:0007016	0.04803
M_97	GO:0001682	0.04803
M_97	GO:0035457	0.04803
M_98	GO:0050911	6.4E-20
M_98	GO:0007186	4.1E-11
M_98	GO:0033141	0.000003
M_98	GO:0002323	0.000016
M_98	GO:0031424	0.000022
M_98	GO:0030049	0.000047
M_98	GO:0006579	0.000078
M_98	GO:1900005	0.000078
M_98	GO:0043330	0.000083
M_98	GO:0050817	0.00015
M_98	GO:1901318	0.00016
M_98	GO:0042426	0.00039
M_98	GO:0071267	0.00054
M_98	GO:0060337	0.00105

M_98	GO:0008343	0.00114
M_98	GO:0042100	0.00124
M_98	GO:0002286	0.00163
M_98	GO:0015747	0.00167
M_98	GO:0051481	0.00299
M_98	GO:0030183	0.0034
M_98	GO:0006959	0.00368
M_98	GO:0021852	0.00514
M_98	GO:1901995	0.00514
M_98	GO:0009590	0.00514
M_98	GO:0097254	0.00514
M_98	GO:0048137	0.00514
M_98	GO:0070409	0.00514
M_98	GO:0033477	0.00514
M_98	GO:1903718	0.00514
M_98	GO:0043252	0.00783
M_98	GO:0050666	0.01025
M_98	GO:1904231	0.01025
M_98	GO:1904234	0.01025
M_98	GO:2000663	0.01025
M_98	GO:0051692	0.01025
M_98	GO:0051695	0.01025
M_98	GO:0086097	0.01025
M_98	GO:0031989	0.01025
M_98	GO:0040030	0.01025
M_98	GO:0070075	0.01025
M_98	GO:0048240	0.01034
M_98	GO:0010469	0.01518
M_98	GO:0071400	0.01533
M_98	GO:0015742	0.01533
M_98	GO:0005989	0.01533
M_98	GO:2000666	0.01533

M_98	GO:0030573	0.01533
M_98	GO:0019731	0.01967
M_98	GO:0030035	0.02039
M_98	GO:2001137	0.02039
M_98	GO:0031427	0.02039
M_98	GO:2000360	0.02039
M_98	GO:2000552	0.02039
M_98	GO:0007406	0.02039
M_98	GO:0048023	0.02039
M_98	GO:0016199	0.02543
M_98	GO:0060478	0.02543
M_98	GO:0048840	0.02543
M_98	GO:0002023	0.02543
M_98	GO:0006880	0.03043
M_98	GO:0071286	0.03043
M_98	GO:0006526	0.03043
M_98	GO:0006689	0.03043
M_98	GO:0006207	0.03043
M_98	GO:0033504	0.03542
M_98	GO:2000210	0.03542
M_98	GO:0031640	0.03696
M_98	GO:0043266	0.04012
M_98	GO:0060054	0.04037
M_98	GO:0001778	0.04037
M_98	GO:0090281	0.04037
M_98	GO:0071313	0.04037
M_98	GO:0051607	0.0409
M_98	GO:0014823	0.04406
M_98	GO:0060046	0.04484
M_98	GO:0048664	0.04531
M_98	GO:0035860	0.04531
M_98	GO:0009128	0.04531

M_99	GO:1904647	0.0022
M_99	GO:0090350	0.0022
M_99	GO:0007098	0.0023
M_99	GO:0045494	0.0026
M_99	GO:0000448	0.0043
M_99	GO:0021691	0.0043
M_99	GO:0003408	0.0065
M_99	GO:1903348	0.0065
M_99	GO:0043585	0.0065
M_99	GO:0033076	0.0065
M_99	GO:0009822	0.0065
M_99	GO:0019391	0.0065
M_99	GO:1903566	0.0086
M_99	GO:0042766	0.0086
M_99	GO:0006344	0.0086
M_99	GO:0032053	0.0086
M_99	GO:0030317	0.0106
M_99	GO:0031064	0.0108
M_99	GO:0010626	0.0108
M_99	GO:1990049	0.0108
M_99	GO:0021942	0.0108
M_99	GO:0045053	0.0108
M_99	GO:0016098	0.0129
M_99	GO:0008626	0.0129
M_99	GO:1990048	0.0151
M_99	GO:0001843	0.0153
M_99	GO:0047497	0.0172
M_99	GO:0009804	0.0172
M_99	GO:1901409	0.0194
M_99	GO:0048096	0.0194
M_99	GO:0048755	0.0194
M_99	GO:0035845	0.0215

M_99	GO:0032926	0.0215
M_99	GO:0007288	0.0236
M_99	GO:0003205	0.0253
M_99	GO:0006915	0.0253
M_99	GO:0008340	0.0278
M_99	GO:0001867	0.03
M_99	GO:0010457	0.03
M_99	GO:0042921	0.0321
M_99	GO:0070989	0.0321
M_99	GO:0021930	0.0321
M_99	GO:0000122	0.0323
M_99	GO:0036159	0.0342
M_99	GO:0007076	0.0342
M_99	GO:0003222	0.0342
M_99	GO:0050684	0.037
M_99	GO:0006098	0.0384
M_99	GO:0060041	0.0384
M_99	GO:0006337	0.0404
M_99	GO:0060294	0.0404
M_99	GO:0002089	0.0425
M_99	GO:0045724	0.0446
M_99	GO:0003351	0.0487
M_100	GO:0006672	0.0000052
M_100	GO:0036065	0.00014
M_100	GO:0043312	0.00018
M_100	GO:0002682	0.00036
M_100	GO:0042355	0.00092
M_100	GO:0010758	0.00112
M_100	GO:1904469	0.00167
M_100	GO:0050900	0.00239
M_100	GO:0034656	0.00262
M_100	GO:2000811	0.00338

M_100	GO:0007250	0.00379
M_100	GO:0009312	0.00422
M_100	GO:0032475	0.00514
M_100	GO:0035675	0.00514
M_100	GO:0000917	0.00514
M_100	GO:1903965	0.00514
M_100	GO:0003336	0.00514
M_100	GO:0046712	0.00514
M_100	GO:0046067	0.00514
M_100	GO:0046057	0.00514
M_100	GO:0006256	0.00514
M_100	GO:0097503	0.00514
M_100	GO:0039702	0.00514
M_100	GO:0045332	0.00616
M_100	GO:0050718	0.00783
M_100	GO:0045638	0.0089
M_100	GO:0060086	0.01025
M_100	GO:0006097	0.01025
M_100	GO:0050653	0.01025
M_100	GO:0038158	0.01025
M_100	GO:0014839	0.01025
M_100	GO:1901143	0.01025
M_100	GO:0009826	0.01025
M_100	GO:0002415	0.01025
M_100	GO:0048250	0.01025
M_100	GO:0038016	0.01025
M_100	GO:0016561	0.01025
M_100	GO:0030974	0.01025
M_100	GO:2000538	0.01025
M_100	GO:0002859	0.01025
M_100	GO:0010752	0.01025
M_100	GO:0043318	0.01025

M_100	GO:0036258	0.01034
M_100	GO:0043278	0.01243
M_100	GO:0051603	0.01347
M_100	GO:0006486	0.01397
M_100	GO:0007080	0.01468
M_100	GO:0033572	0.01468
M_100	GO:1902042	0.01468
M_100	GO:0006740	0.01533
M_100	GO:0070859	0.01533
M_100	GO:0043402	0.01533
M_100	GO:0045959	0.01533
M_100	GO:1903027	0.01533
M_100	GO:0019276	0.01533
M_100	GO:0008292	0.01533
M_100	GO:0051138	0.01533
M_100	GO:1903385	0.01533
M_100	GO:0007173	0.01688
M_100	GO:0018279	0.0171
M_100	GO:2000346	0.02039
M_100	GO:0046398	0.02039
M_100	GO:0007621	0.02039
M_100	GO:0006696	0.02039
M_100	GO:0048133	0.02039
M_100	GO:0061526	0.02039
M_100	GO:1903874	0.02039
M_100	GO:0051793	0.02543
M_100	GO:0030091	0.02543
M_100	GO:0045338	0.02543
M_100	GO:0006102	0.02543
M_100	GO:0030263	0.02543
M_100	GO:0033209	0.02604
M_100	GO:0006953	0.02625

M_100	GO:0043401	0.02975
M_100	GO:0098706	0.03043
M_100	GO:0010871	0.03043
M_100	GO:0032960	0.03043
M_100	GO:0006824	0.03043
M_100	GO:0043987	0.03043
M_100	GO:0060696	0.03043
M_100	GO:0060304	0.03043
M_100	GO:0022027	0.03043
M_100	GO:0015014	0.03043
M_100	GO:0032922	0.0325
M_100	GO:0007157	0.0336
M_100	GO:0007084	0.03542
M_100	GO:0036462	0.03542
M_100	GO:0035726	0.03542
M_100	GO:1902570	0.03542
M_100	GO:0045598	0.03757
M_100	GO:0070059	0.03811
M_100	GO:0051592	0.0388
M_100	GO:0019218	0.03972
M_100	GO:0031468	0.03995
M_100	GO:0034144	0.04037
M_100	GO:0030853	0.04037
M_100	GO:0071578	0.04037
M_100	GO:0015889	0.04037
M_100	GO:0015871	0.04037
M_100	GO:0007172	0.04037
M_100	GO:0061469	0.04037
M_100	GO:0015677	0.04037
M_100	GO:0030210	0.04037
M_100	GO:0045008	0.04037
M_100	GO:0017158	0.04284

M_100	GO:0098703	0.04531
M_100	GO:0002315	0.04531
M_100	GO:0060312	0.04531
M_100	GO:0060339	0.04531
M_100	GO:0035947	0.04531
M_100	GO:0043589	0.04531
M_100	GO:0097400	0.04531
M_100	GO:0034454	0.04531
M_100	GO:0051974	0.04531
M_101	GO:0030182	0.000099
M_101	GO:0070474	0.00028
M_101	GO:0061303	0.00036
M_101	GO:0007158	0.00117
M_101	GO:0001502	0.00184
M_101	GO:0043586	0.00203
M_101	GO:0048013	0.00214
M_101	GO:0051394	0.0032
M_101	GO:0060299	0.0032
M_101	GO:1900826	0.0032
M_101	GO:0014053	0.0032
M_101	GO:1904450	0.0032
M_101	GO:0038009	0.0032
M_101	GO:0017015	0.00524
M_101	GO:2001240	0.00527
M_101	GO:0007620	0.00628
M_101	GO:0009450	0.00638
M_101	GO:0061153	0.00638
M_101	GO:2001288	0.00638
M_101	GO:0071560	0.00804
M_101	GO:0010718	0.00833
M_101	GO:0046968	0.00956
M_101	GO:0033602	0.00956

M_101	GO:1902722	0.00956
M_101	GO:0009449	0.00956
M_101	GO:0071899	0.00956
M_101	GO:0033292	0.00956
M_101	GO:0014873	0.00956
M_101	GO:0009957	0.00956
M_101	GO:0097116	0.00956
M_101	GO:0060492	0.00956
M_101	GO:0034059	0.00956
M_101	GO:1902951	0.00956
M_101	GO:0070649	0.00956
M_101	GO:0043392	0.01157
M_101	GO:0035176	0.01246
M_101	GO:0070164	0.01273
M_101	GO:0035995	0.01273
M_101	GO:0040038	0.01273
M_101	GO:0021943	0.01273
M_101	GO:0097118	0.01273
M_101	GO:0010701	0.01273
M_101	GO:0070836	0.01273
M_101	GO:0071603	0.01273
M_101	GO:0007275	0.01326
M_101	GO:0001992	0.01588
M_101	GO:0060762	0.01588
M_101	GO:0033153	0.01588
M_101	GO:0071866	0.01588
M_101	GO:0051295	0.01588
M_101	GO:0046618	0.01588
M_101	GO:0060406	0.01588
M_101	GO:0061052	0.01588
M_101	GO:1902262	0.01588
M_101	GO:0016055	0.01712

M_101	GO:0048747	0.01835
M_101	GO:0051666	0.01903
M_101	GO:0032713	0.01903
M_101	GO:0070172	0.01903
M_101	GO:1903997	0.01903
M_101	GO:0060710	0.01903
M_101	GO:1903215	0.01903
M_101	GO:0071895	0.01903
M_101	GO:0000147	0.01903
M_101	GO:0042713	0.01903
M_101	GO:0032696	0.01903
M_101	GO:0060684	0.01903
M_101	GO:0045964	0.01903
M_101	GO:0060455	0.01903
M_101	GO:0061002	0.01903
M_101	GO:0030512	0.02053
M_101	GO:0071300	0.02053
M_101	GO:0042475	0.02209
M_101	GO:0030223	0.02217
M_101	GO:0032714	0.02217
M_101	GO:0048341	0.02217
M_101	GO:0042297	0.02217
M_101	GO:0071864	0.02217
M_101	GO:0097151	0.02217
M_101	GO:0060373	0.02217
M_101	GO:0051001	0.02217
M_101	GO:0021861	0.02217
M_101	GO:1903546	0.02217
M_101	GO:0097065	0.02217
M_101	GO:0048733	0.02217
M_101	GO:0036089	0.02217
M_101	GO:0060021	0.0234

M_101	GO:0022407	0.02449
M_101	GO:0014033	0.02495
M_101	GO:0001778	0.02529
M_101	GO:0030854	0.02529
M_101	GO:0045607	0.02529
M_101	GO:1903800	0.02529
M_101	GO:0060638	0.02529
M_101	GO:0021873	0.02529
M_101	GO:0060137	0.02529
M_101	GO:0007601	0.02816
M_101	GO:0033601	0.02841
M_101	GO:0016081	0.02841
M_101	GO:0070050	0.02841
M_101	GO:0097119	0.02841
M_101	GO:0048069	0.02841
M_101	GO:0061072	0.02841
M_101	GO:0060068	0.03152
M_101	GO:0019062	0.03152
M_101	GO:0035845	0.03152
M_101	GO:0045792	0.03152
M_101	GO:0048712	0.03152
M_101	GO:0051451	0.03461
M_101	GO:0042135	0.03461
M_101	GO:0060033	0.03461
M_101	GO:0031652	0.03461
M_101	GO:0090331	0.03461
M_101	GO:0042756	0.03461
M_101	GO:0071468	0.03461
M_101	GO:0050966	0.03461
M_101	GO:0060174	0.03461
M_101	GO:0071773	0.03737
M_101	GO:0060272	0.0377

M_101	GO:0048266	0.0377
M_101	GO:2001256	0.0377
M_101	GO:0032230	0.04078
M_101	GO:0001967	0.04078
M_101	GO:2000009	0.04078
M_101	GO:0042271	0.04078
M_101	GO:0014819	0.04078
M_101	GO:0042487	0.04385
M_101	GO:2000060	0.04385
M_101	GO:0044849	0.04385
M_101	GO:0042711	0.04385
M_101	GO:0006805	0.04674
M_101	GO:0071599	0.04691
M_101	GO:0060347	0.04691
M_101	GO:0007512	0.04691
M_101	GO:0070886	0.04996
M_101	GO:1901017	0.04996
M_101	GO:0021542	0.04996
M_102	GO:0030199	9.8E-19
M_102	GO:0030198	1.3E-18
M_102	GO:0007155	1.5E-14
M_102	GO:0070208	1.8E-09
M_102	GO:0035987	0.000000017
M_102	GO:0001501	0.000000021
M_102	GO:0071230	0.00000018
M_102	GO:0030574	0.00000053
M_102	GO:0032331	0.0000016
M_102	GO:0001957	0.0000016
M_102	GO:0043588	0.000022
M_102	GO:0007160	0.000027
M_102	GO:1903225	0.000057
M_102	GO:0003429	0.000064

M_102	GO:0045766	0.000068
M_102	GO:0016525	0.000071
M_102	GO:0033627	0.00011
M_102	GO:0022617	0.00013
M_102	GO:0030335	0.00014
M_102	GO:0071356	0.00014
M_102	GO:0018057	0.00019
M_102	GO:0090090	0.00022
M_102	GO:0060087	0.00028
M_102	GO:0035989	0.00028
M_102	GO:0060325	0.00034
M_102	GO:0032496	0.0004
M_102	GO:0007507	0.0005
M_102	GO:0060346	0.00051
M_102	GO:0051603	0.00052
M_102	GO:0042060	0.00057
M_102	GO:0001649	0.00063
M_102	GO:0043589	0.00067
M_102	GO:0001503	0.00081
M_102	GO:0048251	0.00084
M_102	GO:0042340	0.00122
M_102	GO:0030208	0.00122
M_102	GO:0001568	0.0013
M_102	GO:0046426	0.00146
M_102	GO:0030207	0.00168
M_102	GO:0002063	0.00288
M_102	GO:0035904	0.00304
M_102	GO:0001525	0.00314
M_102	GO:0007517	0.0034
M_102	GO:0042474	0.00344
M_102	GO:0002544	0.00379
M_102	GO:0097435	0.00416

M_102	GO:0060021	0.00423
M_102	GO:1902548	0.00434
M_102	GO:0090177	0.00434
M_102	GO:0061185	0.0044
M_102	GO:1902362	0.0044
M_102	GO:0018277	0.0044
M_102	GO:1904237	0.0044
M_102	GO:0052565	0.0044
M_102	GO:0035921	0.0044
M_102	GO:1902203	0.0044
M_102	GO:1900387	0.0044
M_102	GO:2001113	0.0044
M_102	GO:1902618	0.0044
M_102	GO:0046521	0.0044
M_102	GO:0009314	0.00441
M_102	GO:0030206	0.00536
M_102	GO:0001958	0.00579
M_102	GO:0019221	0.00586
M_102	GO:0030168	0.00587
M_102	GO:0048592	0.006
M_102	GO:0008217	0.00607
M_102	GO:0030324	0.00615
M_102	GO:0018146	0.00717
M_102	GO:1902230	0.00717
M_102	GO:0009612	0.00734
M_102	GO:0048706	0.00736
M_102	GO:0030282	0.00762
M_102	GO:0070563	0.00877
M_102	GO:0052047	0.00877
M_102	GO:0090291	0.00877
M_102	GO:1904209	0.00877
M_102	GO:0086100	0.00877

M_102	GO:0019442	0.00877
M_102	GO:0097325	0.00877
M_102	GO:0070171	0.00877
M_102	GO:2000041	0.00877
M_102	GO:0060279	0.00877
M_102	GO:0003050	0.00877
M_102	GO:2001202	0.00877
M_102	GO:0071306	0.00877
M_102	GO:0071307	0.00877
M_102	GO:1900155	0.00877
M_102	GO:0007229	0.00895
M_102	GO:0001569	0.00977
M_102	GO:0034097	0.01193
M_102	GO:0050776	0.01224
M_102	GO:0038065	0.01297
M_102	GO:2000312	0.01313
M_102	GO:0014707	0.01313
M_102	GO:0021773	0.01313
M_102	GO:0048210	0.01313
M_102	GO:0060414	0.01313
M_102	GO:0046882	0.01313
M_102	GO:0060763	0.01313
M_102	GO:0010710	0.01313
M_102	GO:0035583	0.01313
M_102	GO:0051216	0.01322
M_102	GO:0071364	0.01335
M_102	GO:0048839	0.01379
M_102	GO:0032964	0.014
M_102	GO:0071560	0.01539
M_102	GO:0030514	0.01563
M_102	GO:0007267	0.01567
M_102	GO:0006469	0.01599

M_102	GO:0045578	0.01747
M_102	GO:0043932	0.01747
M_102	GO:0090191	0.01747
M_102	GO:0071279	0.01747
M_102	GO:0035962	0.01747
M_102	GO:0090244	0.01747
M_102	GO:0042701	0.01747
M_102	GO:1990523	0.01747
M_102	GO:0003257	0.01747
M_102	GO:0003273	0.01747
M_102	GO:0090027	0.01747
M_102	GO:0044691	0.01747
M_102	GO:0007161	0.01747
M_102	GO:0060244	0.01747
M_102	GO:0030167	0.01747
M_102	GO:0042662	0.01747
M_102	GO:2001206	0.01747
M_102	GO:1901509	0.01747
M_102	GO:0045077	0.01747
M_102	GO:0010716	0.01747
M_102	GO:0035582	0.01747
M_102	GO:1900158	0.01747
M_102	GO:0016477	0.01842
M_102	GO:0002576	0.01882
M_102	GO:0010596	0.01888
M_102	GO:0060351	0.02071
M_102	GO:0048146	0.02116
M_102	GO:1900747	0.02179
M_102	GO:1900086	0.02179
M_102	GO:0061056	0.02179
M_102	GO:0019441	0.02179
M_102	GO:0010957	0.02179

M_102	GO:0022614	0.02179
M_102	GO:0046813	0.02179
M_102	GO:2001205	0.02179
M_102	GO:0018149	0.0222
M_102	GO:0045669	0.02604
M_102	GO:0032914	0.02609
M_102	GO:0014719	0.02609
M_102	GO:0043152	0.02609
M_102	GO:0060676	0.02609
M_102	GO:0045112	0.02609
M_102	GO:0033591	0.02609
M_102	GO:0060907	0.02609
M_102	GO:0048050	0.02609
M_102	GO:0046881	0.02609
M_102	GO:2000503	0.02609
M_102	GO:0060770	0.02609
M_102	GO:0019800	0.02609
M_102	GO:0007584	0.02871
M_102	GO:0060028	0.03037
M_102	GO:0045650	0.03037
M_102	GO:0045656	0.03037
M_102	GO:0014816	0.03037
M_102	GO:0043654	0.03037
M_102	GO:0030326	0.0308
M_102	GO:0003417	0.03335
M_102	GO:0051147	0.03405
M_102	GO:0014824	0.03463
M_102	GO:0001955	0.03463
M_102	GO:2000035	0.03463
M_102	GO:1901224	0.03508
M_102	GO:0050900	0.03584
M_102	GO:0006954	0.03595

M_102	GO:0007179	0.03661
M_102	GO:0001822	0.03695
M_102	GO:0071300	0.03701
M_102	GO:0007568	0.03763
M_102	GO:0042472	0.03817
M_102	GO:0009308	0.03876
M_102	GO:0001514	0.03888
M_102	GO:0060539	0.03888
M_102	GO:0007614	0.03888
M_102	GO:0048263	0.03888
M_102	GO:0033689	0.03888
M_102	GO:0042541	0.03888
M_102	GO:0035860	0.03888
M_102	GO:1990314	0.03888
M_102	GO:0048664	0.03888
M_102	GO:0051901	0.03888
M_102	GO:0030308	0.04011
M_102	GO:0060429	0.04042
M_102	GO:0001666	0.04053
M_102	GO:0006029	0.04163
M_102	GO:0010717	0.04217
M_102	GO:0060326	0.04293
M_102	GO:0014032	0.04305
M_102	GO:0038063	0.0431
M_102	GO:0009629	0.0431
M_102	GO:0033629	0.0431
M_102	GO:0046512	0.0431
M_102	GO:0045667	0.04576
M_102	GO:0044267	0.04588
M_102	GO:0010466	0.04614
M_102	GO:0001938	0.0462
M_102	GO:0090331	0.04731

M_102	GO:0060576	0.04731
M_102	GO:0048484	0.04731
M_102	GO:0060394	0.04731
M_102	GO:0003198	0.04731
M_102	GO:0050910	0.04731
M_102	GO:0033690	0.04731
M_102	GO:0051451	0.04731
M_102	GO:2001223	0.04731
M_102	GO:0071372	0.04731
M_102	GO:0070098	0.04943
M_102	GO:0071773	0.04998
M_103	GO:0060528	0.00021
M_103	GO:0038202	0.00072
M_103	GO:0045955	0.00125
M_103	GO:0006228	0.00155
M_103	GO:0006183	0.00189
M_103	GO:0006241	0.00309
M_103	GO:0000712	0.00511
M_103	GO:0007221	0.00511
M_103	GO:0042981	0.00549
M_103	GO:0060843	0.00599
M_103	GO:0032458	0.00599
M_103	GO:0072287	0.00599
M_103	GO:0072721	0.00599
M_103	GO:1903755	0.00599
M_103	GO:0021881	0.00599
M_103	GO:0006386	0.00599
M_103	GO:0003270	0.00599
M_103	GO:1903849	0.00599
M_103	GO:1904515	0.00599
M_103	GO:1900477	0.00599
M_103	GO:0016226	0.0063

M_103	GO:0007050	0.00649
M_103	GO:0061136	0.00816
M_103	GO:0032008	0.00959
M_103	GO:0016567	0.01012
M_103	GO:0016032	0.01095
M_103	GO:0021915	0.01107
M_103	GO:0061418	0.01113
M_103	GO:0043547	0.01127
M_103	GO:0060823	0.01195
M_103	GO:0000455	0.01195
M_103	GO:0048320	0.01195
M_103	GO:0072237	0.01195
M_103	GO:0016256	0.01195
M_103	GO:0036146	0.01195
M_103	GO:0045994	0.01195
M_103	GO:0003169	0.01195
M_103	GO:0045967	0.01195
M_103	GO:0003213	0.01195
M_103	GO:0019243	0.01195
M_103	GO:0090035	0.01195
M_103	GO:0010729	0.01195
M_103	GO:0015949	0.013
M_103	GO:0051056	0.01477
M_103	GO:0018105	0.01529
M_103	GO:0032148	0.0157
M_103	GO:0051443	0.01665
M_103	GO:0072017	0.01771
M_103	GO:1904431	0.01787
M_103	GO:0006046	0.01787
M_103	GO:0045608	0.01787
M_103	GO:0030047	0.01787
M_103	GO:2000974	0.01787

M_103	GO:1902263	0.01787
M_103	GO:0003192	0.01787
M_103	GO:0003162	0.01787
M_103	GO:0042779	0.01787
M_103	GO:0060948	0.01787
M_103	GO:2001184	0.01787
M_103	GO:0001743	0.01787
M_103	GO:0071492	0.01787
M_103	GO:0045007	0.01787
M_103	GO:0002283	0.0182
M_103	GO:2001027	0.02376
M_103	GO:0072218	0.02376
M_103	GO:0009794	0.02376
M_103	GO:0003431	0.02376
M_103	GO:0018076	0.02376
M_103	GO:0003273	0.02376
M_103	GO:0060979	0.02376
M_103	GO:0003219	0.02376
M_103	GO:0003241	0.02376
M_103	GO:0007161	0.02376
M_103	GO:0070164	0.02376
M_103	GO:0070948	0.02376
M_103	GO:0030910	0.02376
M_103	GO:0072429	0.02376
M_103	GO:0035735	0.02394
M_103	GO:0007257	0.02394
M_103	GO:0006367	0.02747
M_103	GO:1900034	0.02859
M_103	GO:0043535	0.02919
M_103	GO:0072044	0.02935
M_103	GO:0072144	0.02961
M_103	GO:0030091	0.02961

M_103	GO:0021797	0.02961
M_103	GO:0007386	0.02961
M_103	GO:0015705	0.02961
M_103	GO:0006114	0.02961
M_103	GO:0034316	0.02961
M_103	GO:1901525	0.02961
M_103	GO:0061419	0.02961
M_103	GO:0034198	0.0298
M_103	GO:0032007	0.03229
M_103	GO:0045747	0.03229
M_103	GO:0060842	0.03543
M_103	GO:0009912	0.03543
M_103	GO:1900264	0.03543
M_103	GO:2001033	0.03543
M_103	GO:0017196	0.03543
M_103	GO:0030579	0.03543
M_103	GO:0003160	0.03543
M_103	GO:0000706	0.03543
M_103	GO:0060956	0.03543
M_103	GO:0019262	0.03543
M_103	GO:0072602	0.03543
M_103	GO:0072344	0.03543
M_103	GO:0010792	0.03543
M_103	GO:0071321	0.03543
M_103	GO:0046533	0.03543
M_103	GO:0060687	0.04121
M_103	GO:0031119	0.04121
M_103	GO:1902231	0.04121
M_103	GO:0003180	0.04121
M_103	GO:0003256	0.04121
M_103	GO:0031659	0.04121
M_103	GO:0032364	0.04121

M_103	GO:0007368	0.04256
M_103	GO:0071902	0.04321
M_103	GO:0007507	0.04321
M_103	GO:0016241	0.04625
M_103	GO:0007042	0.04696
M_103	GO:0072205	0.04696
M_103	GO:0047497	0.04696
M_103	GO:0043045	0.04696
M_103	GO:0046784	0.04696
M_103	GO:0006975	0.04696
M_103	GO:1990416	0.04696
M_103	GO:0045008	0.04696
M_103	GO:0001889	0.04842
M_104	GO:0060285	0.000021
M_104	GO:0036158	0.00003
M_104	GO:0071907	0.00017
M_104	GO:0060287	0.00037
M_104	GO:0036159	0.00097
M_104	GO:0032092	0.00194
M_104	GO:0031439	0.00291
M_104	GO:0015821	0.00291
M_104	GO:0034477	0.00291
M_104	GO:0090326	0.00291
M_104	GO:2000975	0.00291
M_104	GO:0090503	0.00518
M_104	GO:0042245	0.00581
M_104	GO:0006867	0.00581
M_104	GO:1902823	0.00581
M_104	GO:0071050	0.00581
M_104	GO:0071036	0.00581
M_104	GO:2000331	0.00581
M_104	GO:0071037	0.00581

M_104	GO:1903181	0.00581
M_104	GO:0043630	0.00581
M_104	GO:0031648	0.00604
M_104	GO:0061357	0.00871
M_104	GO:1990120	0.00871
M_104	GO:1990108	0.00871
M_104	GO:0070350	0.00871
M_104	GO:0035552	0.00871
M_104	GO:0035553	0.00871
M_104	GO:1901026	0.00871
M_104	GO:0089709	0.00871
M_104	GO:1900365	0.00871
M_104	GO:0006977	0.01157
M_104	GO:0070778	0.01159
M_104	GO:0015803	0.01159
M_104	GO:0042853	0.01159
M_104	GO:0038108	0.01159
M_104	GO:0060161	0.01159
M_104	GO:0090521	0.01159
M_104	GO:0007040	0.01238
M_104	GO:0001947	0.01279
M_104	GO:0060135	0.01279
M_104	GO:0060296	0.01447
M_104	GO:0003356	0.01447
M_104	GO:0010606	0.01447
M_104	GO:0090238	0.01447
M_104	GO:0035469	0.01447
M_104	GO:0010629	0.01478
M_104	GO:0006868	0.01734
M_104	GO:0071042	0.01734
M_104	GO:0071910	0.01734
M_104	GO:0050882	0.01734

M_104	GO:0007049	0.01791
M_104	GO:0030433	0.01799
M_104	GO:0032088	0.01964
M_104	GO:0071051	0.0202
M_104	GO:0015808	0.0202
M_104	GO:0032049	0.0202
M_104	GO:0071038	0.0202
M_104	GO:1903071	0.0202
M_104	GO:0014832	0.0202
M_104	GO:0071035	0.02306
M_104	GO:0006449	0.02306
M_104	GO:0090037	0.02306
M_104	GO:1901339	0.02306
M_104	GO:0014824	0.02306
M_104	GO:0015825	0.0259
M_104	GO:2000304	0.0259
M_104	GO:0048387	0.0259
M_104	GO:0018026	0.02874
M_104	GO:0006307	0.02874
M_104	GO:0035721	0.02874
M_104	GO:0043653	0.02874
M_104	GO:0051292	0.02874
M_104	GO:0006228	0.02874
M_104	GO:1902950	0.02874
M_104	GO:0072015	0.03157
M_104	GO:0060213	0.03157
M_104	GO:1903441	0.03157
M_104	GO:0007288	0.03157
M_104	GO:0006183	0.03157
M_104	GO:0040015	0.03157
M_104	GO:0032463	0.03157
M_104	GO:0030968	0.03316

M_104	GO:0071044	0.03439
M_104	GO:0007077	0.03439
M_104	GO:0060391	0.03439
M_104	GO:0010867	0.03439
M_104	GO:2001242	0.03606
M_104	GO:0098789	0.0372
M_104	GO:0050862	0.0372
M_104	GO:0021987	0.03947
M_104	GO:1990126	0.04001
M_104	GO:0071361	0.04001
M_104	GO:1904380	0.04001
M_104	GO:0006241	0.04001
M_104	GO:0033147	0.04001
M_104	GO:0016055	0.04127
M_104	GO:0051967	0.0428
M_104	GO:0015865	0.0428
M_104	GO:0019511	0.0428
M_104	GO:0090141	0.0428
M_104	GO:0031998	0.0428
M_104	GO:0034063	0.0428
M_104	GO:0090335	0.0428
M_104	GO:0051457	0.04559
M_104	GO:0044065	0.04559
M_104	GO:0016973	0.04559
M_104	GO:0045056	0.04559
M_104	GO:1903364	0.04778
M_104	GO:0021756	0.04837
M_105	GO:0006704	0.0000019
M_105	GO:0006705	0.000032
M_105	GO:0072162	0.00011
M_105	GO:0006702	0.00031
M_105	GO:0048845	0.00041

M_105	GO:0048251	0.00051
M_105	GO:0008207	0.00096
M_105	GO:0006869	0.00132
M_105	GO:0010811	0.00144
M_105	GO:0036149	0.00152
M_105	GO:0036148	0.0017
M_105	GO:0051412	0.00232
M_105	GO:0036150	0.00232
M_105	GO:0032967	0.00329
M_105	GO:2000979	0.00342
M_105	GO:0072168	0.00342
M_105	GO:0072169	0.00342
M_105	GO:2000066	0.00342
M_105	GO:0072184	0.00342
M_105	GO:0002194	0.00342
M_105	GO:0090321	0.00342
M_105	GO:2000019	0.00342
M_105	GO:0097380	0.00342
M_105	GO:0061369	0.00342
M_105	GO:0072259	0.00342
M_105	GO:0090425	0.00342
M_105	GO:0061114	0.00342
M_105	GO:0043049	0.00342
M_105	GO:0086010	0.00345
M_105	GO:0036152	0.00356
M_105	GO:0036151	0.00383
M_105	GO:0050482	0.00412
M_105	GO:0048662	0.00441
M_105	GO:0007190	0.00441
M_105	GO:0019228	0.00603
M_105	GO:0030501	0.00638
M_105	GO:1990172	0.00684

M_105	GO:0030237	0.00684
M_105	GO:2000225	0.00684
M_105	GO:0048389	0.00684
M_105	GO:0072183	0.00684
M_105	GO:0061518	0.00684
M_105	GO:1901492	0.00684
M_105	GO:0009115	0.00684
M_105	GO:0035262	0.00684
M_105	GO:0060849	0.00684
M_105	GO:0002282	0.00684
M_105	GO:0072208	0.00684
M_105	GO:0046619	0.00684
M_105	GO:0051025	0.00684
M_105	GO:0032349	0.00684
M_105	GO:0090094	0.00684
M_105	GO:0006654	0.00749
M_105	GO:0008585	0.0078
M_105	GO:0006898	0.00973
M_105	GO:0034651	0.01007
M_105	GO:0032342	0.01007
M_105	GO:0098912	0.01024
M_105	GO:0072190	0.01024
M_105	GO:0038172	0.01024
M_105	GO:0023016	0.01024
M_105	GO:0060748	0.01024
M_105	GO:0055005	0.01024
M_105	GO:0007231	0.01024
M_105	GO:0072034	0.01024
M_105	GO:0001545	0.01024
M_105	GO:0002025	0.01024
M_105	GO:0061184	0.01024
M_105	GO:0060414	0.01024

M_105	GO:0036023	0.01024
M_105	GO:0060129	0.01024
M_105	GO:0060298	0.01363
M_105	GO:0030910	0.01363
M_105	GO:0060214	0.01363
M_105	GO:0061589	0.01363
M_105	GO:0072180	0.01363
M_105	GO:0072268	0.01363
M_105	GO:0042816	0.01363
M_105	GO:0070858	0.01363
M_105	GO:0006663	0.01363
M_105	GO:0006651	0.01363
M_105	GO:0032431	0.01363
M_105	GO:0061205	0.01363
M_105	GO:0006936	0.01677
M_105	GO:0070309	0.01701
M_105	GO:1900748	0.01701
M_105	GO:0061590	0.01701
M_105	GO:0061591	0.01701
M_105	GO:0061302	0.01701
M_105	GO:0086045	0.01701
M_105	GO:0042699	0.01701
M_105	GO:1901843	0.01701
M_105	GO:0035694	0.01701
M_105	GO:0060126	0.01701
M_105	GO:0021766	0.01856
M_105	GO:0030324	0.01928
M_105	GO:0072174	0.02038
M_105	GO:0072143	0.02038
M_105	GO:0071503	0.02038
M_105	GO:0055009	0.02373
M_105	GO:0038030	0.02373

M_105	GO:2000394	0.02373
M_105	GO:0021707	0.02373
M_105	GO:0002826	0.02373
M_105	GO:0070509	0.02529
M_105	GO:0070307	0.02664
M_105	GO:0060059	0.02708
M_105	GO:0060020	0.02708
M_105	GO:0034638	0.02708
M_105	GO:0001865	0.02708
M_105	GO:0072239	0.02708
M_105	GO:0033080	0.02708
M_105	GO:0010917	0.02708
M_105	GO:2000543	0.02708
M_105	GO:0048793	0.02708
M_105	GO:0001938	0.02933
M_105	GO:0042908	0.03041
M_105	GO:0002024	0.03041
M_105	GO:1901978	0.03041
M_105	GO:0051006	0.03041
M_105	GO:0032233	0.03327
M_105	GO:0035865	0.03373
M_105	GO:0050679	0.03539
M_105	GO:0001946	0.03645
M_105	GO:0016042	0.03672
M_105	GO:0030241	0.03705
M_105	GO:0040015	0.03705
M_105	GO:0071372	0.03705
M_105	GO:0051024	0.03705
M_105	GO:2000344	0.03705
M_105	GO:0010518	0.03978
M_105	GO:0090197	0.04035
M_105	GO:2000650	0.04035

M_105	GO:0045741	0.04035
M_105	GO:0042340	0.04035
M_105	GO:0010595	0.04044
M_105	GO:0008406	0.04124
M_105	GO:0045836	0.04363
M_105	GO:0032754	0.04363
M_105	GO:0034374	0.04363
M_105	GO:0006703	0.04363
M_105	GO:0055085	0.04423
M_105	GO:0070374	0.04425
M_105	GO:0030240	0.04691
M_105	GO:0035725	0.04992
M_106	GO:0006357	1.6E-17
M_106	GO:0006355	0.00075
M_106	GO:0001193	0.00405
M_106	GO:1904274	0.00405
M_106	GO:1903645	0.00405
M_106	GO:1903638	0.00405
M_106	GO:0046588	0.00405
M_106	GO:0014057	0.00809
M_106	GO:0021966	0.00809
M_106	GO:0000432	0.01211
M_106	GO:0035544	0.01211
M_106	GO:0033685	0.01211
M_106	GO:0086047	0.01211
M_106	GO:2000649	0.01433
M_106	GO:0019086	0.01611
M_106	GO:0015811	0.01611
M_106	GO:0035106	0.01611
M_106	GO:0046684	0.01611
M_106	GO:0043353	0.02803
M_106	GO:0018406	0.02803

M_106	GO:0014827	0.02803
M_106	GO:0019227	0.02803
M_106	GO:0070459	0.02803
M_106	GO:0060371	0.03197
M_106	GO:0007217	0.03197
M_106	GO:0070474	0.03197
M_106	GO:0001682	0.03981
M_106	GO:0035646	0.03981
M_106	GO:1902093	0.03981
M_106	GO:0043117	0.03981
M_106	GO:0060340	0.0437
M_106	GO:0060856	0.0437
M_106	GO:0051024	0.0437
M_106	GO:0045078	0.04758
M_107	GO:0050911	4E-11
M_107	GO:1903507	0.000000046
M_107	GO:0007186	0.0000015
M_107	GO:0007195	0.00019
M_107	GO:0060158	0.00039
M_107	GO:0051967	0.00045
M_107	GO:0048149	0.00045
M_107	GO:0051481	0.00051
M_107	GO:1901386	0.00073
M_107	GO:0048148	0.00081
M_107	GO:0071880	0.00089
M_107	GO:0007194	0.00147
M_107	GO:0014059	0.00158
M_107	GO:0061100	0.00211
M_107	GO:0061103	0.00211
M_107	GO:1904588	0.00211
M_107	GO:0003359	0.00211
M_107	GO:0060165	0.00211

M_107	GO:0007400	0.00211
M_107	GO:0021750	0.00211
M_107	GO:0001963	0.0022
M_107	GO:0051482	0.00291
M_107	GO:0061102	0.00422
M_107	GO:0061104	0.00422
M_107	GO:0060163	0.00422
M_107	GO:0038194	0.00422
M_107	GO:0071259	0.00422
M_107	GO:0050883	0.00422
M_107	GO:0006355	0.00485
M_107	GO:0003358	0.00632
M_107	GO:0060166	0.00632
M_107	GO:0021530	0.00632
M_107	GO:0006968	0.00666
M_107	GO:0021902	0.01469
M_107	GO:0043266	0.01514
M_107	GO:0061549	0.01885
M_107	GO:0034214	0.02093
M_107	GO:0021892	0.02299
M_107	GO:0006559	0.02299
M_107	GO:0050713	0.02299
M_107	GO:0051593	0.02299
M_107	GO:0034374	0.02712
M_107	GO:0021527	0.02918
M_107	GO:0060579	0.03123
M_107	GO:0000921	0.03123
M_107	GO:0097340	0.03328
M_107	GO:0042136	0.03532
M_107	GO:0042423	0.04345
M_107	GO:0010226	0.04749
M_108	GO:0001580	0.00043

M_108	GO:0022400	0.00307
M_108	GO:0016038	0.0036
M_108	GO:0015723	0.0036
M_108	GO:0072227	0.0036
M_108	GO:0018885	0.0036
M_108	GO:1901086	0.0036
M_108	GO:0018910	0.0036
M_108	GO:0015694	0.0036
M_108	GO:2000355	0.00718
M_108	GO:0016122	0.00718
M_108	GO:0072233	0.00718
M_108	GO:0003335	0.00718
M_108	GO:0014718	0.00718
M_108	GO:0001507	0.00718
M_108	GO:0016121	0.01075
M_108	GO:0072240	0.01075
M_108	GO:2000297	0.01075
M_108	GO:0030886	0.01075
M_108	GO:0070315	0.01075
M_108	GO:0001546	0.01075
M_108	GO:0051092	0.01372
M_108	GO:0030644	0.01431
M_108	GO:0015722	0.01431
M_108	GO:0072218	0.01431
M_108	GO:0031427	0.01431
M_108	GO:0001880	0.01431
M_108	GO:0002074	0.01785
M_108	GO:0008582	0.01785
M_108	GO:2001015	0.02139
M_108	GO:0016098	0.02139
M_108	GO:0010994	0.02139
M_108	GO:0021869	0.02139

M_108	GO:0010193	0.02139
M_108	GO:0002741	0.02139
M_108	GO:0006685	0.02139
M_108	GO:0060336	0.02491
M_108	GO:0007506	0.02491
M_108	GO:0009253	0.02491
M_108	GO:0097264	0.02841
M_108	GO:0015889	0.02841
M_108	GO:0030050	0.02841
M_108	GO:0009308	0.03122
M_108	GO:0090129	0.03191
M_108	GO:2000304	0.03191
M_108	GO:0031915	0.03191
M_108	GO:0071472	0.03191
M_108	GO:0070327	0.03191
M_108	GO:0043031	0.03191
M_108	GO:0071277	0.03209
M_108	GO:0007601	0.03802
M_108	GO:0060340	0.03886
M_108	GO:2000344	0.03886
M_108	GO:0007168	0.03886
M_108	GO:0071340	0.03886
M_108	GO:0060088	0.03886
M_108	GO:0050910	0.03886
M_108	GO:0033234	0.03886
M_108	GO:0031284	0.03886
M_108	GO:0034384	0.04232
M_108	GO:1990403	0.04232
M_108	GO:0006068	0.04232
M_108	GO:0071257	0.04232
M_108	GO:0090630	0.04248
M_108	GO:0042574	0.04577

M_108	GO:0018298	0.04577
M_108	GO:0007229	0.04764
M_108	GO:0042953	0.0492
M_108	GO:0016998	0.0492
M_109	GO:0006357	0.0000004
M_109	GO:0045796	0.0000053
M_109	GO:0010949	0.0000053
M_109	GO:0034462	0.0023
M_109	GO:0006507	0.0023
M_109	GO:0071401	0.0023
M_109	GO:0033344	0.0041
M_109	GO:0002017	0.0047
M_109	GO:2000870	0.0047
M_109	GO:0031283	0.0047
M_109	GO:0010983	0.0047
M_109	GO:0009450	0.0047
M_109	GO:0097241	0.0047
M_109	GO:0000472	0.007
M_109	GO:0001555	0.007
M_109	GO:0006540	0.007
M_109	GO:0006083	0.007
M_109	GO:0060395	0.0079
M_109	GO:0007040	0.0081
M_109	GO:1900041	0.0093
M_109	GO:0010897	0.0093
M_109	GO:0009786	0.0093
M_109	GO:0042126	0.0093
M_109	GO:0007565	0.0095
M_109	GO:0007588	0.0107
M_109	GO:0010507	0.011
M_109	GO:1990049	0.0116
M_109	GO:0051410	0.0116

M_109	GO:0046618	0.0116
M_109	GO:0042632	0.0123
M_109	GO:2000354	0.014
M_109	GO:0000480	0.014
M_109	GO:0010694	0.014
M_109	GO:0006678	0.0163
M_109	GO:0097211	0.0163
M_109	GO:0008154	0.0183
M_109	GO:0090281	0.0186
M_109	GO:0006501	0.0186
M_109	GO:0006681	0.0186
M_109	GO:1902459	0.0186
M_109	GO:0010595	0.02
M_109	GO:0000447	0.0209
M_109	GO:0001714	0.0209
M_109	GO:0042908	0.0209
M_109	GO:1901750	0.0232
M_109	GO:0007168	0.0255
M_109	GO:0046007	0.0255
M_109	GO:0006751	0.0255
M_109	GO:0045919	0.0255
M_109	GO:0042135	0.0255
M_109	GO:0010867	0.0277
M_109	GO:0060965	0.0277
M_109	GO:0030819	0.0293
M_109	GO:0002430	0.03
M_109	GO:0071397	0.03
M_109	GO:0070633	0.03
M_109	GO:0046459	0.03
M_109	GO:1902187	0.0345
M_109	GO:0051450	0.0345
M_109	GO:0051044	0.0345

M_109	GO:0045603	0.0368
M_109	GO:2000773	0.0368
M_109	GO:0006750	0.0368
M_109	GO:0051127	0.0391
M_109	GO:0072643	0.0391
M_109	GO:0019934	0.0391
M_109	GO:0009636	0.0402
M_109	GO:2001275	0.0413
M_109	GO:0006704	0.0413
M_109	GO:0072112	0.0413
M_109	GO:0006833	0.0436
M_109	GO:0042789	0.0436
M_109	GO:0050852	0.0441
M_109	GO:2000757	0.0458
M_109	GO:0006811	0.0466
M_109	GO:0007413	0.048
M_110	GO:0007026	0.0034
M_110	GO:0045234	0.0038
M_110	GO:1904120	0.0038
M_110	GO:0060243	0.0038
M_110	GO:0070845	0.0038
M_110	GO:0070846	0.0038
M_110	GO:0035998	0.0038
M_110	GO:1902981	0.0038
M_110	GO:0071108	0.0064
M_110	GO:0070536	0.0064
M_110	GO:0033138	0.0067
M_110	GO:0061188	0.0075
M_110	GO:0090035	0.0075
M_110	GO:0072334	0.0075
M_110	GO:0014916	0.0075
M_110	GO:0035574	0.0075

M_110	GO:1902975	0.0075
M_110	GO:0071630	0.0113
M_110	GO:0006272	0.0113
M_110	GO:1902882	0.0146
M_110	GO:1901300	0.015
M_110	GO:0033490	0.015
M_110	GO:0033489	0.015
M_110	GO:0071500	0.015
M_110	GO:0006511	0.0183
M_110	GO:0070201	0.0186
M_110	GO:0070842	0.0187
M_110	GO:1901858	0.0187
M_110	GO:1902083	0.0187
M_110	GO:0061734	0.0187
M_110	GO:0071557	0.0187
M_110	GO:0014809	0.0187
M_110	GO:0043620	0.0218
M_110	GO:0032914	0.0224
M_110	GO:0032876	0.0224
M_110	GO:0043983	0.0261
M_110	GO:0034983	0.0261
M_110	GO:0007253	0.0261
M_110	GO:0072423	0.0261
M_110	GO:0010727	0.0261
M_110	GO:0032532	0.0261
M_110	GO:0036123	0.0261
M_110	GO:0006729	0.0261
M_110	GO:0034720	0.0298
M_110	GO:2000465	0.0298
M_110	GO:0046654	0.0298
M_110	GO:0048069	0.0334
M_110	GO:0090073	0.0334

M_110	GO:0034454	0.0334
M_110	GO:0071394	0.0334
M_110	GO:0000380	0.0366
M_110	GO:0070544	0.0371
M_110	GO:2000121	0.0371
M_110	GO:0036500	0.0371
M_110	GO:0033234	0.0407
M_110	GO:0090042	0.0407
M_110	GO:0006273	0.0407
M_110	GO:1901096	0.0407
M_110	GO:1990440	0.0407
M_110	GO:0010870	0.0407
M_110	GO:0002003	0.0407
M_110	GO:0000183	0.0436
M_110	GO:0021903	0.0443
M_110	GO:2000651	0.0443
M_110	GO:0042416	0.0443
M_110	GO:2000042	0.0443
M_110	GO:0031647	0.0466
M_110	GO:0034497	0.0479
M_110	GO:0010839	0.0479
M_111	GO:0030728	0.0000028
M_111	GO:0048387	0.000005
M_111	GO:0042335	0.000047
M_111	GO:0034653	0.000094
M_111	GO:0051122	0.00016
M_111	GO:2000609	0.00016
M_111	GO:0071316	0.00043
M_111	GO:0006590	0.0008
M_111	GO:0009755	0.00082
M_111	GO:0042743	0.00096
M_111	GO:0050665	0.00119

M_111	GO:0019372	0.00139
M_111	GO:0043651	0.00206
M_111	GO:0016486	0.00278
M_111	GO:0061436	0.00285
M_111	GO:0043046	0.00285
M_111	GO:1903319	0.00394
M_111	GO:0009087	0.004
M_111	GO:0048855	0.004
M_111	GO:0003029	0.004
M_111	GO:0042593	0.00465
M_111	GO:0006730	0.00481
M_111	GO:0035025	0.00518
M_111	GO:0033198	0.00637
M_111	GO:0098655	0.00674
M_111	GO:0042744	0.00679
M_111	GO:1901899	0.00798
M_111	GO:0002542	0.00798
M_111	GO:0009258	0.00798
M_111	GO:0071469	0.00798
M_111	GO:0036114	0.00798
M_111	GO:0036116	0.00798
M_111	GO:0014718	0.00798
M_111	GO:0048627	0.00798
M_111	GO:0007267	0.00814
M_111	GO:0007274	0.0086
M_111	GO:0051482	0.01008
M_111	GO:0007056	0.01194
M_111	GO:0044026	0.01194
M_111	GO:0061368	0.01194
M_111	GO:0006556	0.01194
M_111	GO:0021553	0.01194
M_111	GO:0030538	0.01194

M_111	GO:0048619	0.01194
M_111	GO:0042446	0.01304
M_111	GO:0034765	0.01467
M_111	GO:0086010	0.01577
M_111	GO:0051919	0.01589
M_111	GO:2000346	0.01589
M_111	GO:0019556	0.01589
M_111	GO:0019557	0.01589
M_111	GO:0070318	0.01589
M_111	GO:1900535	0.01589
M_111	GO:0044336	0.01589
M_111	GO:0000186	0.01769
M_111	GO:0019369	0.01902
M_111	GO:0060385	0.01982
M_111	GO:0051599	0.01982
M_111	GO:0033153	0.01982
M_111	GO:0046513	0.02039
M_111	GO:0007605	0.02068
M_111	GO:0050727	0.02355
M_111	GO:2001046	0.02374
M_111	GO:0010756	0.02374
M_111	GO:0019800	0.02374
M_111	GO:0070257	0.02374
M_111	GO:2000143	0.02374
M_111	GO:2000288	0.02374
M_111	GO:0015015	0.02374
M_111	GO:0071805	0.0238
M_111	GO:0033674	0.02555
M_111	GO:1901844	0.02764
M_111	GO:0006701	0.02764
M_111	GO:0051792	0.02764
M_111	GO:0014832	0.02764

M_111	GO:0050909	0.02864
M_111	GO:0031340	0.03153
M_111	GO:0060155	0.03153
M_111	GO:0033216	0.03153
M_111	GO:0001887	0.03153
M_111	GO:0030154	0.03248
M_111	GO:0006936	0.03352
M_111	GO:0048266	0.03491
M_111	GO:0042989	0.0354
M_111	GO:0070857	0.03926
M_111	GO:0071281	0.03926
M_111	GO:0030432	0.03926
M_111	GO:0055114	0.0421
M_111	GO:0002003	0.0431
M_111	GO:0050966	0.0431
M_111	GO:0002862	0.0431
M_111	GO:0010524	0.04634
M_111	GO:0001678	0.04659
M_111	GO:0038180	0.04692
M_111	GO:0055062	0.04692
M_111	GO:0070493	0.04692
M_112	GO:0018916	9.4E-11
M_112	GO:0050911	6.5E-10
M_112	GO:0042178	0.00000003
M_112	GO:0070458	0.000000031
M_112	GO:1901687	0.00000066
M_112	GO:0007186	0.0000067
M_112	GO:0006749	0.000035
M_112	GO:0051930	0.00022
M_112	GO:0031214	0.00072
M_112	GO:0031640	0.00089
M_112	GO:1903494	0.0032

M_112	GO:1903496	0.0032
M_112	GO:1903488	0.0032
M_112	GO:0050832	0.00558
M_112	GO:0031424	0.00568
M_112	GO:0019730	0.00636
M_112	GO:0002305	0.00638
M_112	GO:0008065	0.00956
M_112	GO:0002803	0.00956
M_112	GO:0070175	0.00956
M_112	GO:0098700	0.01588
M_112	GO:0014809	0.01588
M_112	GO:0042742	0.01688
M_112	GO:0015760	0.01903
M_112	GO:0060316	0.01903
M_112	GO:0086043	0.02217
M_112	GO:1902715	0.02217
M_112	GO:0071313	0.02529
M_112	GO:0060371	0.02529
M_112	GO:0015816	0.02841
M_112	GO:0060315	0.02841
M_112	GO:0086016	0.03152
M_112	GO:0046541	0.03152
M_112	GO:0003376	0.0377
M_112	GO:0046415	0.04078
M_112	GO:0015812	0.04078
M_112	GO:0016998	0.04385
M_112	GO:0046838	0.04691
M_112	GO:0042268	0.04996
M_112	GO:0010881	0.04996
M_113	GO:0071673	0.000028
M_113	GO:0045669	0.000028
M_113	GO:0007413	0.000035

M_113	GO:0001822	0.000041
M_113	GO:0072126	0.000093
M_113	GO:0007494	0.0006
M_113	GO:0048566	0.00092
M_113	GO:0051965	0.00101
M_113	GO:0007158	0.00109
M_113	GO:0036120	0.00171
M_113	GO:0001938	0.00179
M_113	GO:0014032	0.00219
M_113	GO:1904566	0.00308
M_113	GO:1990256	0.00308
M_113	GO:0051758	0.00308
M_113	GO:0090242	0.00308
M_113	GO:0021813	0.00308
M_113	GO:0050783	0.00308
M_113	GO:0010976	0.00402
M_113	GO:0035332	0.00616
M_113	GO:0090362	0.00616
M_113	GO:0061386	0.00616
M_113	GO:0030505	0.00616
M_113	GO:2001111	0.00616
M_113	GO:0021589	0.00616
M_113	GO:2000439	0.00616
M_113	GO:2001076	0.00616
M_113	GO:0014003	0.00642
M_113	GO:0060548	0.00881
M_113	GO:2000721	0.00922
M_113	GO:0014016	0.00922
M_113	GO:0070295	0.00922
M_113	GO:0097477	0.00922
M_113	GO:1900625	0.00922
M_113	GO:0097195	0.00922

M_113	GO:0045226	0.00922
M_113	GO:1903385	0.00922
M_113	GO:0070649	0.00922
M_113	GO:0046426	0.0108
M_113	GO:0030323	0.01193
M_113	GO:0060282	0.01227
M_113	GO:0042904	0.01227
M_113	GO:0040038	0.01227
M_113	GO:0035799	0.01227
M_113	GO:0046449	0.01227
M_113	GO:1900127	0.01227
M_113	GO:0089700	0.01227
M_113	GO:0007157	0.01291
M_113	GO:0007356	0.01532
M_113	GO:0055118	0.01532
M_113	GO:0090500	0.01532
M_113	GO:0060023	0.01532
M_113	GO:0000255	0.01532
M_113	GO:0051295	0.01532
M_113	GO:0021942	0.01532
M_113	GO:0038033	0.01532
M_113	GO:0006549	0.01532
M_113	GO:0043123	0.016
M_113	GO:0010694	0.01835
M_113	GO:0032914	0.01835
M_113	GO:0046069	0.01835
M_113	GO:0060022	0.01835
M_113	GO:0007185	0.01835
M_113	GO:0002001	0.01835
M_113	GO:1901727	0.01835
M_113	GO:0045719	0.01835
M_113	GO:0035108	0.02076

M_113	GO:0043406	0.02122
M_113	GO:0045666	0.02132
M_113	GO:0010668	0.02138
M_113	GO:0006771	0.02138
M_113	GO:0003357	0.02138
M_113	GO:0036302	0.02138
M_113	GO:0048842	0.02138
M_113	GO:0019530	0.02138
M_113	GO:0007275	0.0229
M_113	GO:0006776	0.0244
M_113	GO:0097264	0.0244
M_113	GO:0071420	0.0244
M_113	GO:0071447	0.0244
M_113	GO:2000480	0.0244
M_113	GO:0051549	0.0244
M_113	GO:0072332	0.02587
M_113	GO:2001028	0.02691
M_113	GO:0009855	0.02709
M_113	GO:0019695	0.02741
M_113	GO:0061303	0.02741
M_113	GO:0006573	0.02741
M_113	GO:0060539	0.02741
M_113	GO:0030643	0.02741
M_113	GO:0010838	0.02741
M_113	GO:0033689	0.02741
M_113	GO:0007193	0.02828
M_113	GO:0051639	0.03041
M_113	GO:0097105	0.03041
M_113	GO:0043951	0.03041
M_113	GO:0006600	0.03041
M_113	GO:0003211	0.03041
M_113	GO:0048712	0.03041

M_113	GO:0007162	0.03087
M_113	GO:0003279	0.03295
M_113	GO:0032060	0.0334
M_113	GO:0030836	0.0334
M_113	GO:0031953	0.0334
M_113	GO:0045793	0.0334
M_113	GO:0021554	0.0334
M_113	GO:0051593	0.0334
M_113	GO:0060174	0.0334
M_113	GO:0031076	0.03577
M_113	GO:0006105	0.03638
M_113	GO:0006107	0.03638
M_113	GO:0030208	0.03638
M_113	GO:0051764	0.03638
M_113	GO:0033631	0.03638
M_113	GO:0050773	0.03746
M_113	GO:0030182	0.03787
M_113	GO:0008406	0.03907
M_113	GO:0035641	0.03935
M_113	GO:0060253	0.03935
M_113	GO:0042976	0.03935
M_113	GO:0009143	0.03935
M_113	GO:0060397	0.03935
M_113	GO:0042574	0.03935
M_113	GO:0061029	0.03935
M_113	GO:2001044	0.03935
M_113	GO:0060134	0.03935
M_113	GO:0050672	0.04168
M_113	GO:0051645	0.04231
M_113	GO:0044849	0.04231
M_113	GO:0046325	0.04231
M_113	GO:1901201	0.04231

M_113	GO:0035810	0.04231
M_113	GO:0022038	0.04231
M_113	GO:0030730	0.04527
M_113	GO:0072075	0.04527
M_113	GO:0071356	0.04724
M_113	GO:0071453	0.04776
M_113	GO:0032793	0.04821
M_113	GO:2000773	0.04821
M_113	GO:0035988	0.04821
M_113	GO:0006465	0.04821
M_113	GO:0040007	0.04905
M_114	GO:0035280	0.00015
M_114	GO:0035278	0.00048
M_114	GO:0031054	0.00066
M_114	GO:0006122	0.00066
M_114	GO:0010586	0.00195
M_114	GO:0060092	0.00274
M_114	GO:0042840	0.00274
M_114	GO:0061537	0.00274
M_114	GO:0018146	0.00285
M_114	GO:0007223	0.00413
M_114	GO:0019853	0.00547
M_114	GO:0010501	0.00705
M_114	GO:0010862	0.00765
M_114	GO:0045007	0.0082
M_114	GO:0045652	0.0086
M_114	GO:0060395	0.01066
M_114	GO:1901668	0.01092
M_114	GO:0016266	0.01214
M_114	GO:0021540	0.01363
M_114	GO:0070934	0.01363
M_114	GO:0019640	0.01363

M_114	GO:0000738	0.01363
M_114	GO:0009060	0.01577
M_114	GO:0007185	0.01633
M_114	GO:0000467	0.01903
M_114	GO:1903608	0.01903
M_114	GO:0034625	0.01903
M_114	GO:0034626	0.01903
M_114	GO:0019367	0.01903
M_114	GO:0017183	0.02171
M_114	GO:0006782	0.02171
M_114	GO:0002024	0.0244
M_114	GO:0070544	0.02707
M_114	GO:0046185	0.02707
M_114	GO:1900121	0.02974
M_114	GO:0019430	0.03435
M_114	GO:0042761	0.03505
M_114	GO:0036109	0.03505
M_114	GO:0015937	0.03505
M_114	GO:0033169	0.0377
M_114	GO:0046855	0.0377
M_114	GO:0045944	0.03832
M_114	GO:1904262	0.04034
M_114	GO:0043968	0.04297
M_114	GO:0043651	0.04559
M_114	GO:0042753	0.04821
M_114	GO:0035338	0.04821
M_114	GO:0048041	0.04994
M_115	GO:0006357	2.1E-26
M_115	GO:0070895	0.0000056
M_115	GO:0006355	0.00012
M_115	GO:0006749	0.00036
M_115	GO:1901687	0.00126

M_115	GO:0044791	0.01193
M_115	GO:2000660	0.01193
M_115	GO:0043508	0.03306
M_115	GO:0006702	0.03538
M_115	GO:0042448	0.04
M_115	GO:0043651	0.04
M_115	GO:0006704	0.04231
M_115	GO:2000678	0.0469
M_115	GO:0010804	0.04918
M_116	GO:0008544	0.0000031
M_116	GO:0031581	0.000044
M_116	GO:0070268	0.000057
M_116	GO:1900138	0.00011
M_116	GO:0019065	0.00011
M_116	GO:0030198	0.00016
M_116	GO:0070836	0.00021
M_116	GO:0001525	0.00032
M_116	GO:0046718	0.00056
M_116	GO:0001937	0.00091
M_116	GO:0031340	0.00097
M_116	GO:0030216	0.0014
M_116	GO:0001666	0.0017
M_116	GO:0060546	0.00189
M_116	GO:0032355	0.00212
M_116	GO:0071548	0.00222
M_116	GO:0010952	0.00256
M_116	GO:0007229	0.00275
M_116	GO:0043123	0.00377
M_116	GO:0032793	0.00404
M_116	GO:0033627	0.00551
M_116	GO:0035024	0.00569
M_116	GO:0010951	0.00573

M_116	GO:0014866	0.00594
M_116	GO:0042313	0.00599
M_116	GO:0031583	0.00599
M_116	GO:0052362	0.00599
M_116	GO:0039650	0.00599
M_116	GO:0055111	0.00599
M_116	GO:1903545	0.00599
M_116	GO:1903609	0.00599
M_116	GO:1900085	0.00599
M_116	GO:0044147	0.00599
M_116	GO:0001899	0.00599
M_116	GO:0001576	0.00599
M_116	GO:2000661	0.00599
M_116	GO:1903944	0.00599
M_116	GO:0006535	0.00599
M_116	GO:0043179	0.00599
M_116	GO:2001213	0.00599
M_116	GO:0061436	0.0063
M_116	GO:0090050	0.00694
M_116	GO:0016338	0.00829
M_116	GO:0007155	0.00959
M_116	GO:0030334	0.01124
M_116	GO:1903845	0.01177
M_116	GO:0051866	0.01195
M_116	GO:0034696	0.01195
M_116	GO:0086098	0.01195
M_116	GO:2000474	0.01195
M_116	GO:0014839	0.01195
M_116	GO:0060715	0.01195
M_116	GO:0018153	0.01195
M_116	GO:0009115	0.01195
M_116	GO:0006127	0.01195

M_116	GO:0034342	0.01195
M_116	GO:0005993	0.01195
M_116	GO:2000536	0.01195
M_116	GO:0032804	0.01195
M_116	GO:0019343	0.01195
M_116	GO:0019478	0.01195
M_116	GO:0071385	0.01197
M_116	GO:0010259	0.013
M_116	GO:0097194	0.01404
M_116	GO:0090023	0.01478
M_116	GO:0045987	0.0157
M_116	GO:0045907	0.0157
M_116	GO:0060544	0.01755
M_116	GO:0070673	0.01771
M_116	GO:0030185	0.01787
M_116	GO:0008065	0.01787
M_116	GO:0038172	0.01787
M_116	GO:0070814	0.01787
M_116	GO:0003365	0.01787
M_116	GO:1903598	0.01787
M_116	GO:0014028	0.01787
M_116	GO:2000286	0.01787
M_116	GO:0060585	0.01787
M_116	GO:0021986	0.01787
M_116	GO:0001560	0.01787
M_116	GO:1903348	0.01787
M_116	GO:0070495	0.01787
M_116	GO:0043418	0.01787
M_116	GO:0043163	0.01787
M_116	GO:0033484	0.01787
M_116	GO:1901740	0.01787
M_116	GO:0019448	0.01787

M_116	GO:0070101	0.01787
M_116	GO:0051603	0.01902
M_116	GO:0086091	0.01963
M_116	GO:1902042	0.01963
M_116	GO:0035329	0.02068
M_116	GO:0051482	0.02174
M_116	GO:0030104	0.0234
M_116	GO:1902807	0.02344
M_116	GO:0032652	0.02363
M_116	GO:0001994	0.02376
M_116	GO:0060298	0.02376
M_116	GO:0014826	0.02376
M_116	GO:0071315	0.02376
M_116	GO:0071918	0.02376
M_116	GO:0048550	0.02376
M_116	GO:0010716	0.02376
M_116	GO:0033031	0.02376
M_116	GO:0031394	0.02376
M_116	GO:0015722	0.02376
M_116	GO:0071284	0.02376
M_116	GO:0045629	0.02376
M_116	GO:0036343	0.02376
M_116	GO:0015855	0.02376
M_116	GO:0044860	0.02376
M_116	GO:0006565	0.02376
M_116	GO:0060355	0.02376
M_116	GO:0019346	0.02376
M_116	GO:0048386	0.02376
M_116	GO:0051771	0.02376
M_116	GO:0097296	0.02376
M_116	GO:0060161	0.02376
M_116	GO:2001200	0.02376

M_116	GO:0045321	0.02387
M_116	GO:0051898	0.02507
M_116	GO:0001974	0.02507
M_116	GO:0007588	0.02583
M_116	GO:0033574	0.02622
M_116	GO:0043277	0.02739
M_116	GO:0043086	0.02754
M_116	GO:0007589	0.02773
M_116	GO:0071560	0.02826
M_116	GO:0043535	0.02891
M_116	GO:0001765	0.02906
M_116	GO:2000535	0.02934
M_116	GO:0072126	0.02961
M_116	GO:1900747	0.02961
M_116	GO:0042262	0.02961
M_116	GO:0034392	0.02961
M_116	GO:0098914	0.02961
M_116	GO:0071376	0.02961
M_116	GO:0060662	0.02961
M_116	GO:0048625	0.02961
M_116	GO:0038044	0.02961
M_116	GO:0044791	0.02961
M_116	GO:0097350	0.02961
M_116	GO:0070309	0.02961
M_116	GO:0071866	0.02961
M_116	GO:2000391	0.02961
M_116	GO:0035987	0.03104
M_116	GO:0007613	0.03117
M_116	GO:0007595	0.03229
M_116	GO:0060070	0.03258
M_116	GO:0070498	0.03301
M_116	GO:0048731	0.0335

M_116	GO:0045104	0.03356
M_116	GO:0071621	0.03498
M_116	GO:0010193	0.03543
M_116	GO:0019800	0.03543
M_116	GO:0052405	0.03543
M_116	GO:0071389	0.03543
M_116	GO:0098903	0.03543
M_116	GO:0010760	0.03543
M_116	GO:0003100	0.03543
M_116	GO:0015793	0.03543
M_116	GO:0045627	0.03543
M_116	GO:0006863	0.03543
M_116	GO:1901299	0.03543
M_116	GO:0072602	0.03543
M_116	GO:0098706	0.03543
M_116	GO:2001199	0.03543
M_116	GO:0006689	0.03543
M_116	GO:0003057	0.03543
M_116	GO:0007185	0.03543
M_116	GO:0050766	0.03617
M_116	GO:0018149	0.03624
M_116	GO:0006909	0.03736
M_116	GO:1903078	0.03885
M_116	GO:0048661	0.04022
M_116	GO:0031295	0.04022
M_116	GO:0045063	0.04085
M_116	GO:2000347	0.04121
M_116	GO:2000483	0.04121
M_116	GO:0051001	0.04121
M_116	GO:0019563	0.04121
M_116	GO:1903071	0.04121
M_116	GO:0071455	0.04121

M_116	GO:0007021	0.04121
M_116	GO:0034141	0.04121
M_116	GO:0045650	0.04121
M_116	GO:0045656	0.04121
M_116	GO:1903361	0.04121
M_116	GO:0045602	0.04121
M_116	GO:0036462	0.04121
M_116	GO:0070459	0.04121
M_116	GO:1901844	0.04121
M_116	GO:0072358	0.04227
M_116	GO:0035635	0.04611
M_116	GO:0060137	0.04696
M_116	GO:0060267	0.04696
M_116	GO:0014824	0.04696
M_116	GO:0061051	0.04696
M_116	GO:0070831	0.04696
M_116	GO:0032471	0.04696
M_116	GO:0030853	0.04696
M_116	GO:0098915	0.04696
M_116	GO:0033632	0.04696
M_116	GO:0035655	0.04696
M_116	GO:0042045	0.04696
M_116	GO:0015677	0.04696
M_116	GO:0014732	0.04696
M_116	GO:0006955	0.04941
M_117	GO:0002479	7.3E-13
M_117	GO:0060333	5.2E-11
M_117	GO:0002476	7.9E-11
M_117	GO:0002480	4.9E-10
M_117	GO:0060337	8.8E-10
M_117	GO:0001916	0.00000024
M_117	GO:0006954	0.00000085

M_117	GO:0030593	0.0000011
M_117	GO:0071347	0.0000025
M_117	GO:1990668	0.000003
M_117	GO:0071346	0.0000031
M_117	GO:0070374	0.000004
M_117	GO:0048245	0.0000043
M_117	GO:0070098	0.0000048
M_117	GO:0031658	0.0000052
M_117	GO:0019885	0.0000052
M_117	GO:0010818	0.000017
M_117	GO:0046967	0.000028
M_117	GO:0071356	0.000033
M_117	GO:0002548	0.000046
M_117	GO:0006955	0.000057
M_117	GO:2000866	0.000085
M_117	GO:0043615	0.00042
M_117	GO:2000503	0.00042
M_117	GO:2000427	0.00059
M_117	GO:0010469	0.00059
M_117	GO:0007050	0.00078
M_117	GO:0016032	0.00097
M_117	GO:0033280	0.00102
M_117	GO:0006952	0.00127
M_117	GO:0051607	0.00145
M_117	GO:0060340	0.00152
M_117	GO:0007204	0.00165
M_117	GO:0048246	0.00178
M_117	GO:0043922	0.00182
M_117	GO:1900025	0.00214
M_117	GO:0090280	0.00249
M_117	GO:0006959	0.00258
M_117	GO:0051770	0.00286

M_117	GO:0031663	0.00304
M_117	GO:0043950	0.00368
M_117	GO:0032693	0.00368
M_117	GO:0090026	0.00412
M_117	GO:0042832	0.00459
M_117	GO:0048002	0.00489
M_117	GO:0071222	0.00502
M_117	GO:0033194	0.00508
M_117	GO:2000564	0.00531
M_117	GO:0032760	0.00536
M_117	GO:0002845	0.00537
M_117	GO:0035409	0.00537
M_117	GO:2000173	0.00537
M_117	GO:2000795	0.00537
M_117	GO:0050823	0.00537
M_117	GO:0048685	0.00537
M_117	GO:0034276	0.00537
M_117	GO:0015707	0.00537
M_117	GO:0006876	0.00537
M_117	GO:0035505	0.00537
M_117	GO:0072616	0.00537
M_117	GO:0035684	0.00537
M_117	GO:0002489	0.00537
M_117	GO:0036145	0.00537
M_117	GO:0032496	0.00568
M_117	GO:0043491	0.00601
M_117	GO:0050729	0.00683
M_117	GO:0010499	0.00728
M_117	GO:0007267	0.00762
M_117	GO:0010575	0.00916
M_117	GO:0051281	0.00983
M_117	GO:0002717	0.01061

M_117	GO:0021740	0.0107
M_117	GO:2000458	0.0107
M_117	GO:0038178	0.0107
M_117	GO:0070839	0.0107
M_117	GO:0046041	0.0107
M_117	GO:2000791	0.0107
M_117	GO:1902728	0.0107
M_117	GO:0031959	0.0107
M_117	GO:0032759	0.0107
M_117	GO:0072560	0.0107
M_117	GO:0090285	0.0107
M_117	GO:2000566	0.0107
M_117	GO:2000502	0.0107
M_117	GO:0036269	0.0107
M_117	GO:2000393	0.0107
M_117	GO:0007193	0.01076
M_117	GO:0090023	0.01197
M_117	GO:0046627	0.01273
M_117	GO:0050850	0.01273
M_117	GO:0001953	0.0135
M_117	GO:0043123	0.01381
M_117	GO:0042157	0.01578
M_117	GO:0002534	0.01587
M_117	GO:0035986	0.01601
M_117	GO:2000111	0.01601
M_117	GO:0042270	0.01601
M_117	GO:0060559	0.01601
M_117	GO:0033092	0.01601
M_117	GO:0060058	0.01601
M_117	GO:0060689	0.01601
M_117	GO:0045590	0.01601
M_117	GO:0046968	0.01601

M_117	GO:0005989	0.01601
M_117	GO:0061141	0.01601
M_117	GO:2000078	0.01601
M_117	GO:0060399	0.01601
M_117	GO:0032632	0.01601
M_117	GO:1901740	0.01601
M_117	GO:0045429	0.01679
M_117	GO:0030449	0.02037
M_117	GO:0030890	0.02039
M_117	GO:0032623	0.02068
M_117	GO:0032611	0.02069
M_117	GO:0070664	0.02093
M_117	GO:1900113	0.02129
M_117	GO:0038165	0.02129
M_117	GO:1901509	0.02129
M_117	GO:0014805	0.02129
M_117	GO:0070574	0.02129
M_117	GO:0002678	0.02129
M_117	GO:0071421	0.02129
M_117	GO:1900041	0.02129
M_117	GO:0061470	0.02129
M_117	GO:0034124	0.02129
M_117	GO:0006710	0.02129
M_117	GO:0045085	0.02129
M_117	GO:0002309	0.02129
M_117	GO:0060355	0.02129
M_117	GO:2001181	0.02129
M_117	GO:0070164	0.02129
M_117	GO:0060486	0.02129
M_117	GO:0097296	0.02129
M_117	GO:0050999	0.02134
M_117	GO:0050766	0.02144

M_117	GO:0043547	0.02228
M_117	GO:0009408	0.02305
M_117	GO:0008285	0.02342
M_117	GO:0002250	0.02427
M_117	GO:0035722	0.0253
M_117	GO:0051169	0.02629
M_117	GO:0010216	0.02654
M_117	GO:0006166	0.02654
M_117	GO:0060510	0.02654
M_117	GO:0019509	0.02654
M_117	GO:0060509	0.02654
M_117	GO:0060662	0.02654
M_117	GO:1900028	0.02654
M_117	GO:0021960	0.02654
M_117	GO:0045084	0.02654
M_117	GO:0070487	0.02654
M_117	GO:2001186	0.02654
M_117	GO:0019441	0.02654
M_117	GO:0061737	0.02654
M_117	GO:0043525	0.02845
M_117	GO:0007566	0.02845
M_117	GO:0007155	0.03123
M_117	GO:0050702	0.0315
M_117	GO:1900227	0.03177
M_117	GO:0010193	0.03177
M_117	GO:0032430	0.03177
M_117	GO:0033591	0.03177
M_117	GO:0035234	0.03177
M_117	GO:0010756	0.03177
M_117	GO:0000480	0.03177
M_117	GO:1902774	0.03177
M_117	GO:1900045	0.03177

M_117	GO:0043308	0.03177
M_117	GO:0035634	0.03177
M_117	GO:1902023	0.03177
M_117	GO:0002439	0.03177
M_117	GO:0060335	0.03177
M_117	GO:0045345	0.03177
M_117	GO:0002819	0.03414
M_117	GO:0010332	0.03637
M_117	GO:0043524	0.03678
M_117	GO:0045410	0.03697
M_117	GO:0031622	0.03697
M_117	GO:0048733	0.03697
M_117	GO:0014050	0.03697
M_117	GO:0000395	0.03697
M_117	GO:0034393	0.03697
M_117	GO:0002666	0.03697
M_117	GO:0002606	0.03697
M_117	GO:0006968	0.03877
M_117	GO:0050918	0.03877
M_117	GO:0030816	0.04
M_117	GO:0035690	0.04
M_117	GO:0009653	0.04002
M_117	GO:0014824	0.04214
M_117	GO:0075713	0.04214
M_117	GO:0034351	0.04214
M_117	GO:0060054	0.04214
M_117	GO:0045080	0.04214
M_117	GO:1903423	0.04214
M_117	GO:0007341	0.04214
M_117	GO:0006610	0.04214
M_117	GO:0033129	0.04214
M_117	GO:0032729	0.04375

M_117	GO:0042531	0.04503
M_117	GO:0043312	0.04513
M_117	GO:0015833	0.04548
M_117	GO:0032731	0.04685
M_117	GO:0010992	0.04728
M_117	GO:0048143	0.04728
M_117	GO:0071679	0.04728
M_117	GO:0038155	0.04728
M_117	GO:0045136	0.04728
M_117	GO:0061635	0.04728
M_117	GO:0090308	0.04728
M_117	GO:0071394	0.04728
M_117	GO:0060586	0.04728
M_117	GO:0000447	0.04728
M_117	GO:0010835	0.04728
M_117	GO:1904263	0.04728
M_117	GO:0060339	0.04728
M_118	GO:0030336	0.00013
M_118	GO:0098706	0.00013
M_118	GO:0015677	0.00024
M_118	GO:0072619	0.00297
M_118	GO:0015828	0.00297
M_118	GO:1903712	0.00297
M_118	GO:0045186	0.00297
M_118	GO:1902491	0.00297
M_118	GO:0048792	0.00297
M_118	GO:0014047	0.0051
M_118	GO:0035332	0.00593
M_118	GO:0002316	0.00593
M_118	GO:2000296	0.00593
M_118	GO:0036345	0.00593
M_118	GO:0097498	0.00593

M_118	GO:0070904	0.00593
M_118	GO:0043006	0.00593
M_118	GO:0042059	0.00597
M_118	GO:0045786	0.00771
M_118	GO:1902722	0.00888
M_118	GO:0008594	0.00888
M_118	GO:1902498	0.00888
M_118	GO:0010750	0.00888
M_118	GO:0060414	0.00888
M_118	GO:1903078	0.01042
M_118	GO:0048661	0.01081
M_118	GO:0007178	0.01112
M_118	GO:1901509	0.01182
M_118	GO:0060040	0.01182
M_118	GO:0006726	0.01182
M_118	GO:0071896	0.01182
M_118	GO:0061470	0.01182
M_118	GO:0070779	0.01182
M_118	GO:0070837	0.01182
M_118	GO:0033590	0.01182
M_118	GO:0033594	0.01182
M_118	GO:0010942	0.01314
M_118	GO:1900020	0.01475
M_118	GO:0032237	0.01475
M_118	GO:0098712	0.01475
M_118	GO:0035926	0.01475
M_118	GO:0010991	0.01475
M_118	GO:1901256	0.01475
M_118	GO:2000359	0.01475
M_118	GO:0072656	0.01475
M_118	GO:0042494	0.01475
M_118	GO:0002223	0.01549

M_118	GO:0035556	0.01574
M_118	GO:1901224	0.01689
M_118	GO:0048644	0.01742
M_118	GO:0071286	0.01768
M_118	GO:0070141	0.01768
M_118	GO:1904177	0.01768
M_118	GO:0043152	0.01768
M_118	GO:2000667	0.01768
M_118	GO:0060613	0.01768
M_118	GO:0006468	0.02006
M_118	GO:0031659	0.0206
M_118	GO:2001182	0.0206
M_118	GO:0071476	0.0206
M_118	GO:0006898	0.0222
M_118	GO:0034333	0.02226
M_118	GO:0035331	0.02351
M_118	GO:0032959	0.02351
M_118	GO:0042117	0.02351
M_118	GO:0014051	0.02351
M_118	GO:0031666	0.02351
M_118	GO:0072205	0.02351
M_118	GO:0034144	0.02351
M_118	GO:1903800	0.02351
M_118	GO:0071447	0.02351
M_118	GO:0055072	0.02467
M_118	GO:0071287	0.0264
M_118	GO:0060242	0.0264
M_118	GO:0014820	0.0264
M_118	GO:0048143	0.0264
M_118	GO:0046485	0.0264
M_118	GO:0043069	0.0291
M_118	GO:1900044	0.0293

M_118	GO:0015851	0.0293
M_118	GO:0042118	0.0293
M_118	GO:0046549	0.0293
M_118	GO:0010595	0.03113
M_118	GO:0042743	0.03165
M_118	GO:0032060	0.03218
M_118	GO:0010960	0.03218
M_118	GO:0043116	0.03218
M_118	GO:0045176	0.03218
M_118	GO:0060394	0.03218
M_118	GO:0019852	0.03218
M_118	GO:0007494	0.03505
M_118	GO:0060766	0.03505
M_118	GO:0002903	0.03505
M_118	GO:0051764	0.03505
M_118	GO:0010642	0.03792
M_118	GO:0032725	0.03792
M_118	GO:0061029	0.03792
M_118	GO:0006957	0.04078
M_118	GO:0090136	0.04363
M_118	GO:0090051	0.04363
M_118	GO:0030324	0.04444
M_118	GO:0007286	0.04644
M_118	GO:0032930	0.04647
M_118	GO:1900424	0.04647
M_118	GO:2000310	0.04647
M_118	GO:0045725	0.04647
M_118	GO:0045055	0.04794
M_118	GO:0017148	0.04795
M_118	GO:0042438	0.0493
M_118	GO:0036149	0.0493
M_118	GO:0045780	0.0493

M_119	GO:0060819	0.000025
M_119	GO:0036353	0.0003
M_119	GO:0034656	0.00085
M_119	GO:1903420	0.00291
M_119	GO:0002561	0.00291
M_119	GO:0015790	0.00291
M_119	GO:0036138	0.00291
M_119	GO:0043973	0.00291
M_119	GO:0006592	0.00291
M_119	GO:0010034	0.00291
M_119	GO:0042265	0.00291
M_119	GO:0038061	0.00403
M_119	GO:0015783	0.00581
M_119	GO:0006784	0.00581
M_119	GO:0002268	0.00581
M_119	GO:1990569	0.00581
M_119	GO:1901143	0.00581
M_119	GO:0042264	0.00581
M_119	GO:0097111	0.00581
M_119	GO:0033979	0.00581
M_119	GO:0000462	0.00634
M_119	GO:0051092	0.00771
M_119	GO:0021775	0.00871
M_119	GO:0021776	0.00871
M_119	GO:0045875	0.00871
M_119	GO:1990166	0.00871
M_119	GO:0046985	0.00871
M_119	GO:0098586	0.00895
M_119	GO:0045893	0.00913
M_119	GO:0070498	0.01118
M_119	GO:0051893	0.01118
M_119	GO:0031122	0.01157

M_119	GO:1903566	0.01159
M_119	GO:0071787	0.01159
M_119	GO:0071922	0.01159
M_119	GO:0070213	0.01159
M_119	GO:0060623	0.01159
M_119	GO:1990034	0.01159
M_119	GO:0061428	0.01159
M_119	GO:0002263	0.01183
M_119	GO:0010815	0.01447
M_119	GO:0034184	0.01447
M_119	GO:0045541	0.01447
M_119	GO:0055129	0.01447
M_119	GO:0016032	0.01495
M_119	GO:0043433	0.01537
M_119	GO:0090166	0.01734
M_119	GO:0048205	0.01734
M_119	GO:1901621	0.01734
M_119	GO:0070973	0.01734
M_119	GO:0035524	0.01734
M_119	GO:0006970	0.01817
M_119	GO:0000972	0.0202
M_119	GO:0007000	0.0202
M_119	GO:1902741	0.0202
M_119	GO:0000160	0.0202
M_119	GO:0015808	0.0202
M_119	GO:0007346	0.02262
M_119	GO:0045647	0.02306
M_119	GO:0010992	0.0259
M_119	GO:0021702	0.0259
M_119	GO:0042447	0.0259
M_119	GO:0015816	0.0259
M_119	GO:0032933	0.0259

M_119	GO:0097428	0.0259
M_119	GO:0035562	0.0259
M_119	GO:0000122	0.02809
M_119	GO:0046085	0.02874
M_119	GO:2001214	0.02874
M_119	GO:0019240	0.02874
M_119	GO:0010960	0.03157
M_119	GO:0007064	0.03375
M_119	GO:0014029	0.03439
M_119	GO:1900181	0.03656
M_119	GO:0008340	0.0372
M_119	GO:2000781	0.0372
M_119	GO:0070198	0.04001
M_119	GO:0046040	0.04001
M_119	GO:0045717	0.04001
M_119	GO:2000059	0.04001
M_119	GO:0000209	0.04032
M_119	GO:0002467	0.0428
M_119	GO:0090141	0.0428
M_119	GO:0007252	0.0428
M_119	GO:1902187	0.0428
M_119	GO:0006123	0.04559
M_119	GO:0032012	0.04559
M_119	GO:1903830	0.04559
M_119	GO:0060828	0.04705
M_119	GO:1904355	0.04837
M_120	GO:0006614	1E-30
M_120	GO:0000184	1E-30
M_120	GO:0006413	1E-30
M_120	GO:0002181	1E-30
M_120	GO:0006364	1.1E-12
M_120	GO:0000027	1.2E-11

M_120	GO:0000028	1.2E-09
M_120	GO:2000059	0.0000011
M_120	GO:0000461	0.000034
M_120	GO:2000435	0.000034
M_120	GO:1904667	0.000064
M_120	GO:0006414	0.00011
M_120	GO:0042273	0.00018
M_120	GO:0042274	0.00058
M_120	GO:0034101	0.00069
M_120	GO:0000462	0.00082
M_120	GO:0000447	0.0012
M_120	GO:0006412	0.00245
M_120	GO:0042254	0.00468
M_120	GO:0034463	0.00588
M_120	GO:1902546	0.00588
M_120	GO:0061481	0.00588
M_120	GO:0043128	0.00588
M_120	GO:1902167	0.00588
M_120	GO:2001272	0.00588
M_120	GO:0033119	0.00868
M_120	GO:1901796	0.00946
M_120	GO:0071480	0.01171
M_120	GO:1990145	0.01172
M_120	GO:1904352	0.01172
M_120	GO:0030822	0.01172
M_120	GO:0005999	0.01172
M_120	GO:0006425	0.01172
M_120	GO:2000156	0.01172
M_120	GO:0050821	0.01473
M_120	GO:0042407	0.01514
M_120	GO:1902255	0.01737
M_120	GO:0051343	0.01754

M_120	GO:0042998	0.01754
M_120	GO:0097327	0.01754
M_120	GO:1902626	0.01754
M_120	GO:0072717	0.01754
M_120	GO:1902445	0.01754
M_120	GO:1902164	0.01754
M_120	GO:0045079	0.01754
M_120	GO:0006407	0.01754
M_120	GO:0042769	0.02097
M_120	GO:1901798	0.02288
M_120	GO:0002309	0.02331
M_120	GO:0032079	0.02331
M_120	GO:0043490	0.02331
M_120	GO:0006659	0.02331
M_120	GO:0006417	0.02464
M_120	GO:0008542	0.02876
M_120	GO:0098914	0.02906
M_120	GO:0046813	0.02906
M_120	GO:1903208	0.02906
M_120	GO:0010891	0.02906
M_120	GO:2000630	0.02906
M_120	GO:1904044	0.02906
M_120	GO:0006177	0.02906
M_120	GO:0035722	0.02996
M_120	GO:1902231	0.03444
M_120	GO:0022605	0.03477
M_120	GO:0035900	0.03477
M_120	GO:0009052	0.03477
M_120	GO:0060372	0.03477
M_120	GO:0072344	0.03477
M_120	GO:0046651	0.03946
M_120	GO:0048318	0.04044

M_120	GO:0006032	0.04044
M_120	GO:0097623	0.04044
M_120	GO:0000056	0.04044
M_120	GO:0034983	0.04044
M_120	GO:0007093	0.04056
M_120	GO:0070498	0.04155
M_120	GO:0006977	0.04293
M_120	GO:0045471	0.04371
M_120	GO:0098915	0.04609
M_120	GO:0036155	0.04609
M_120	GO:2000543	0.04609
M_120	GO:0010898	0.04609
M_120	GO:0006610	0.04609
M_120	GO:0060452	0.04609
M_120	GO:0051092	0.04872
M_121	GO:0040032	0.004
M_121	GO:1904535	0.004
M_121	GO:1904506	0.004
M_121	GO:2000828	0.008
M_121	GO:0019542	0.008
M_121	GO:0019427	0.008
M_121	GO:0019413	0.008
M_121	GO:0048866	0.008
M_121	GO:0000732	0.008
M_121	GO:0044211	0.012
M_121	GO:0033484	0.012
M_121	GO:1902990	0.012
M_121	GO:0060168	0.012
M_121	GO:0060214	0.016
M_121	GO:0036438	0.016
M_121	GO:0097494	0.016
M_121	GO:0006772	0.016

M_121	GO:1904430	0.02
M_121	GO:0090611	0.02
M_121	GO:1902083	0.02
M_121	GO:0044206	0.02
M_121	GO:0005981	0.024
M_121	GO:0060789	0.024
M_121	GO:0060956	0.024
M_121	GO:0010265	0.024
M_121	GO:0001302	0.024
M_121	GO:0002176	0.024
M_121	GO:0045162	0.028
M_121	GO:0045039	0.032
M_121	GO:0017183	0.032
M_121	GO:0060836	0.032
M_121	GO:0007042	0.032
M_121	GO:0006987	0.032
M_121	GO:0007614	0.035
M_121	GO:0031017	0.035
M_121	GO:0010835	0.035
M_121	GO:0016032	0.036
M_121	GO:0015986	0.039
M_121	GO:0051715	0.039
M_121	GO:0010459	0.039
M_121	GO:0007191	0.039
M_121	GO:0007016	0.039
M_121	GO:0038003	0.039
M_121	GO:0046085	0.039
M_121	GO:0001942	0.041
M_121	GO:0042984	0.043
M_121	GO:0090161	0.043
M_121	GO:0006620	0.043
M_121	GO:0042147	0.045

M_121	GO:0006368	0.046
M_121	GO:1902902	0.047
M_121	GO:0006069	0.047
M_121	GO:0000244	0.047
M_122	GO:0070102	0.0011
M_122	GO:0045795	0.0029
M_122	GO:0038154	0.0029
M_122	GO:0010879	0.0029
M_122	GO:0036111	0.0029
M_122	GO:0008089	0.0037
M_122	GO:0043171	0.004
M_122	GO:0090149	0.0057
M_122	GO:0038109	0.0057
M_122	GO:0019285	0.0057
M_122	GO:0006408	0.0057
M_122	GO:0006617	0.0057
M_122	GO:0007260	0.0084
M_122	GO:0002904	0.0085
M_122	GO:0070634	0.0085
M_122	GO:0070859	0.0085
M_122	GO:0072156	0.0085
M_122	GO:0018105	0.0102
M_122	GO:0038165	0.0114
M_122	GO:0038028	0.0114
M_122	GO:0070294	0.0114
M_122	GO:0048861	0.0114
M_122	GO:0036112	0.0114
M_122	GO:0050904	0.0114
M_122	GO:0051771	0.0114
M_122	GO:0070120	0.0142
M_122	GO:0030321	0.0142
M_122	GO:0035426	0.0142

M_122	GO:0006886	0.0147
M_122	GO:0022027	0.017
M_122	GO:0010825	0.017
M_122	GO:0042426	0.017
M_122	GO:2000675	0.017
M_122	GO:0050428	0.017
M_122	GO:0000281	0.017
M_122	GO:2000973	0.0198
M_122	GO:0033007	0.0198
M_122	GO:0018344	0.0198
M_122	GO:0072383	0.0198
M_122	GO:0030953	0.0198
M_122	GO:0044829	0.0198
M_122	GO:0019885	0.0198
M_122	GO:0006501	0.0226
M_122	GO:0006013	0.0226
M_122	GO:0002327	0.0226
M_122	GO:0006616	0.0226
M_122	GO:0006890	0.0251
M_122	GO:0002480	0.0254
M_122	GO:0070508	0.0254
M_122	GO:0045714	0.0254
M_122	GO:0010873	0.0254
M_122	GO:0034498	0.0254
M_122	GO:0034454	0.0254
M_122	GO:0034199	0.0254
M_122	GO:1902476	0.0256
M_122	GO:0070072	0.0282
M_122	GO:0000290	0.0282
M_122	GO:0006554	0.0282
M_122	GO:0070106	0.031
M_122	GO:0070757	0.031

M_122	GO:0036006	0.031
M_122	GO:0019886	0.0319
M_122	GO:0019532	0.0337
M_122	GO:0048711	0.0337
M_122	GO:0071044	0.0337
M_122	GO:0051988	0.0337
M_122	GO:0002474	0.0351
M_122	GO:0036109	0.0365
M_122	GO:0031274	0.0365
M_122	GO:0055064	0.0365
M_122	GO:0090110	0.0392
M_122	GO:1902358	0.0392
M_122	GO:0010457	0.0392
M_122	GO:0043508	0.0392
M_122	GO:0000921	0.042
M_122	GO:0033540	0.042
M_122	GO:0030033	0.0447
M_122	GO:0097340	0.0447
M_122	GO:0008286	0.0464
M_122	GO:0072673	0.0474
M_122	GO:0032367	0.0474
M_123	GO:0006357	1.2E-09
M_123	GO:0060370	0.000038
M_123	GO:0002860	0.00026
M_123	GO:0042271	0.00097
M_123	GO:0034375	0.00167
M_123	GO:2001142	0.0036
M_123	GO:0071879	0.0036
M_123	GO:0010905	0.0036
M_123	GO:0045951	0.0036
M_123	GO:0046814	0.0036
M_123	GO:1904227	0.0036

M_123	GO:0014853	0.0036
M_123	GO:0015695	0.00362
M_123	GO:0032990	0.00368
M_123	GO:0043252	0.00391
M_123	GO:0032438	0.00391
M_123	GO:0006363	0.00553
M_123	GO:0019836	0.00718
M_123	GO:1903518	0.00718
M_123	GO:0044027	0.00718
M_123	GO:2000077	0.00718
M_123	GO:0030382	0.00718
M_123	GO:0033364	0.00718
M_123	GO:0000717	0.00718
M_123	GO:0006361	0.00741
M_123	GO:0015698	0.01074
M_123	GO:0006288	0.01075
M_123	GO:0032289	0.01075
M_123	GO:2000466	0.01075
M_123	GO:0051659	0.01075
M_123	GO:1902723	0.01075
M_123	GO:0010836	0.01075
M_123	GO:0001654	0.01294
M_123	GO:1990414	0.01431
M_123	GO:0032510	0.01431
M_123	GO:0061084	0.01431
M_123	GO:1903779	0.01449
M_123	GO:0007157	0.0173
M_123	GO:2000467	0.01785
M_123	GO:0015705	0.01785
M_123	GO:0036016	0.01785
M_123	GO:0061052	0.01785
M_123	GO:0098914	0.01785

M_123	GO:0045719	0.02139
M_123	GO:0050882	0.02139
M_123	GO:0014722	0.02139
M_123	GO:0043068	0.02142
M_123	GO:0007197	0.02491
M_123	GO:0019064	0.02491
M_123	GO:0061370	0.02491
M_123	GO:0040016	0.02491
M_123	GO:0006283	0.0284
M_123	GO:0060155	0.02841
M_123	GO:0051823	0.02841
M_123	GO:0035646	0.03539
M_123	GO:0018095	0.03539
M_123	GO:1902004	0.03539
M_123	GO:0051654	0.03886
M_123	GO:0015747	0.04232
M_123	GO:0019532	0.04232
M_123	GO:0009650	0.04577
M_123	GO:0010745	0.04577
M_123	GO:0050862	0.04577
M_123	GO:0071285	0.04577
M_123	GO:0086069	0.0492
M_123	GO:0043249	0.0492
M_123	GO:1902358	0.0492
M_123	GO:0046325	0.0492
M_123	GO:0003214	0.0492
M_123	GO:0001675	0.0492
M_124	GO:0050911	2.8E-13
M_124	GO:0007186	3.6E-10
M_124	GO:0031424	0.0000032
M_124	GO:0018149	0.0000097
M_124	GO:0070268	0.00012

M_124	GO:1902173	0.00228
M_124	GO:0072272	0.00456
M_124	GO:0038188	0.00456
M_124	GO:0051835	0.00683
M_124	GO:0072086	0.0091
M_124	GO:0001887	0.01812
M_124	GO:0090286	0.02037
M_124	GO:0010269	0.02484
M_124	GO:0015671	0.03151
M_124	GO:0061436	0.04471
M_124	GO:0007606	0.04487
M_124	GO:0007398	0.0469
M_125	GO:0098789	0.00088
M_125	GO:0044128	0.00342
M_125	GO:0018293	0.00342
M_125	GO:0016180	0.00503
M_125	GO:0000349	0.00684
M_125	GO:0046668	0.00684
M_125	GO:0006369	0.00749
M_125	GO:0006378	0.00995
M_125	GO:0002215	0.01024
M_125	GO:0043988	0.01024
M_125	GO:1990120	0.01024
M_125	GO:0061187	0.01024
M_125	GO:0070534	0.01129
M_125	GO:1902807	0.01348
M_125	GO:0071787	0.01363
M_125	GO:0035087	0.01363
M_125	GO:2001137	0.01363
M_125	GO:0034553	0.01363
M_125	GO:0046726	0.01363
M_125	GO:0039534	0.01363

M_125	GO:0030423	0.01363
M_125	GO:0006121	0.01363
M_125	GO:0061624	0.01701
M_125	GO:0070120	0.01701
M_125	GO:0006303	0.0203
M_125	GO:0016055	0.02036
M_125	GO:0090166	0.02038
M_125	GO:0046533	0.02038
M_125	GO:0050916	0.02038
M_125	GO:0050917	0.02038
M_125	GO:0043987	0.02038
M_125	GO:0048680	0.02038
M_125	GO:0019563	0.02373
M_125	GO:0010886	0.02373
M_125	GO:0006283	0.02595
M_125	GO:0000422	0.02595
M_125	GO:0097264	0.02708
M_125	GO:0033129	0.02708
M_125	GO:0072321	0.02708
M_125	GO:0061469	0.02708
M_125	GO:0045039	0.02708
M_125	GO:0060291	0.02795
M_125	GO:0016081	0.03041
M_125	GO:0034498	0.03041
M_125	GO:0060056	0.03041
M_125	GO:0048143	0.03041
M_125	GO:0051974	0.03041
M_125	GO:0070544	0.03373
M_125	GO:0048280	0.03373
M_125	GO:0006388	0.03373
M_125	GO:0000290	0.03373
M_125	GO:0001833	0.03705

M_125	GO:0006379	0.03906
M_125	GO:0048711	0.04035
M_125	GO:0000244	0.04035
M_125	GO:0070914	0.04035
M_125	GO:0031175	0.04327
M_125	GO:0072422	0.0463
M_125	GO:1904380	0.04691
M_125	GO:0022038	0.04691
M_125	GO:0001867	0.04691
M_125	GO:0040007	0.04786
M_126	GO:1902358	0.00096
M_126	GO:0071539	0.00159
M_126	GO:0070125	0.0031
M_126	GO:0070126	0.0033
M_126	GO:1902356	0.00331
M_126	GO:0061444	0.00331
M_126	GO:0072715	0.00331
M_126	GO:0030393	0.00331
M_126	GO:0007072	0.00331
M_126	GO:0043181	0.00331
M_126	GO:1904570	0.00331
M_126	GO:1904574	0.00331
M_126	GO:0036258	0.00442
M_126	GO:0002086	0.00661
M_126	GO:0055114	0.0081
M_126	GO:0015709	0.0099
M_126	GO:0030200	0.0099
M_126	GO:0005985	0.0099
M_126	GO:0042732	0.0099
M_126	GO:0061073	0.0099
M_126	GO:0000023	0.0099
M_126	GO:0071423	0.0099

M_126	GO:0045747	0.01058
M_126	GO:0050885	0.01147
M_126	GO:0030641	0.01301
M_126	GO:0005997	0.01318
M_126	GO:0002943	0.01318
M_126	GO:0007023	0.01318
M_126	GO:0019640	0.01645
M_126	GO:0055129	0.01645
M_126	GO:0070221	0.01645
M_126	GO:0071422	0.01645
M_126	GO:0061309	0.0197
M_126	GO:0061156	0.0197
M_126	GO:0072344	0.0197
M_126	GO:0021894	0.02295
M_126	GO:0032049	0.02295
M_126	GO:0007021	0.02295
M_126	GO:0007000	0.02295
M_126	GO:0045602	0.02295
M_126	GO:2000766	0.02295
M_126	GO:0016236	0.02554
M_126	GO:0014041	0.02619
M_126	GO:0039536	0.02619
M_126	GO:1990416	0.02619
M_126	GO:0018401	0.02619
M_126	GO:0036066	0.02619
M_126	GO:0070933	0.02941
M_126	GO:0046598	0.02941
M_126	GO:0071233	0.02941
M_126	GO:0038155	0.02941
M_126	GO:0090394	0.02941
M_126	GO:0010324	0.0323
M_126	GO:0061314	0.03263

M_126	GO:0030948	0.03263
M_126	GO:0034378	0.03263
M_126	GO:0034379	0.03263
M_126	GO:0072070	0.03583
M_126	GO:0072015	0.03583
M_126	GO:0060213	0.03583
M_126	GO:0045945	0.03583
M_126	GO:0032436	0.03729
M_126	GO:0072017	0.03902
M_126	GO:0019532	0.03902
M_126	GO:0035278	0.03902
M_126	GO:0031115	0.03902
M_126	GO:0003184	0.03902
M_126	GO:2000052	0.03902
M_126	GO:2000232	0.03902
M_126	GO:0010642	0.04221
M_126	GO:0034497	0.04221
M_126	GO:0043652	0.04221
M_126	GO:0007175	0.04538
M_126	GO:0035435	0.04538
M_126	GO:1903543	0.04854
M_126	GO:0050884	0.04854
M_127	GO:0031468	0.0005
M_127	GO:0046835	0.0015
M_127	GO:0015887	0.0022
M_127	GO:0015878	0.0022
M_127	GO:0035611	0.0022
M_127	GO:0006888	0.003
M_127	GO:0019303	0.0044
M_127	GO:0051228	0.0044
M_127	GO:0035483	0.0067
M_127	GO:0045200	0.0067

M_127	GO:0090234	0.0067
M_127	GO:0070901	0.0067
M_127	GO:0097051	0.0067
M_127	GO:0035965	0.0089
M_127	GO:0006696	0.0089
M_127	GO:0070093	0.0089
M_127	GO:0006635	0.0106
M_127	GO:0031064	0.0111
M_127	GO:0032811	0.0111
M_127	GO:2000987	0.0111
M_127	GO:0051461	0.0111
M_127	GO:0051464	0.0111
M_127	GO:0003400	0.0133
M_127	GO:0042791	0.0133
M_127	GO:0042797	0.0133
M_127	GO:0060455	0.0133
M_127	GO:1903361	0.0155
M_127	GO:0051013	0.0155
M_127	GO:0019321	0.0172
M_127	GO:0019896	0.0177
M_127	GO:0060452	0.0177
M_127	GO:2000252	0.0177
M_127	GO:0034199	0.0199
M_127	GO:0033689	0.0199
M_127	GO:0006348	0.0199
M_127	GO:0070050	0.0199
M_127	GO:0045792	0.022
M_127	GO:0032836	0.022
M_127	GO:0061088	0.022
M_127	GO:0034214	0.022
M_127	GO:0043117	0.022
M_127	GO:0042756	0.0242

M_127	GO:0080009	0.0242
M_127	GO:0032099	0.0264
M_127	GO:0006707	0.0264
M_127	GO:0016024	0.0286
M_127	GO:0030157	0.0286
M_127	GO:0072425	0.0286
M_127	GO:0090148	0.0286
M_127	GO:0090280	0.0307
M_127	GO:1990126	0.0307
M_127	GO:0060547	0.0329
M_127	GO:0035970	0.035
M_127	GO:0071786	0.035
M_127	GO:0006646	0.035
M_127	GO:0051457	0.035
M_127	GO:0036149	0.0372
M_127	GO:0043950	0.0372
M_127	GO:0030859	0.0393
M_127	GO:0006098	0.0393
M_127	GO:0007030	0.0408
M_127	GO:0007130	0.0436
M_127	GO:0072488	0.0457
M_127	GO:0032506	0.0479
M_127	GO:0071577	0.05
M_128	GO:0072221	0.00017
M_128	GO:0060290	0.00035
M_128	GO:0034371	0.00046
M_128	GO:0034378	0.00073
M_128	GO:0033700	0.00107
M_128	GO:0010248	0.00126
M_128	GO:0036376	0.00169
M_128	GO:0034375	0.00218
M_128	GO:0043691	0.00218

M_128	GO:0061365	0.00405
M_128	GO:0015730	0.00411
M_128	GO:0072286	0.00411
M_128	GO:0034972	0.00411
M_128	GO:0060817	0.00411
M_128	GO:0010987	0.00411
M_128	GO:0021983	0.00614
M_128	GO:0051480	0.00676
M_128	GO:0070328	0.0081
M_128	GO:0015913	0.0082
M_128	GO:0072233	0.0082
M_128	GO:0021526	0.0082
M_128	GO:0061104	0.0082
M_128	GO:0001827	0.0082
M_128	GO:0034445	0.0082
M_128	GO:0060621	0.0082
M_128	GO:0046005	0.0082
M_128	GO:0009566	0.00849
M_128	GO:1900271	0.00959
M_128	GO:0019432	0.01011
M_128	GO:1903489	0.01228
M_128	GO:0003358	0.01228
M_128	GO:0030718	0.01228
M_128	GO:0010133	0.01228
M_128	GO:0030573	0.01228
M_128	GO:0070634	0.01228
M_128	GO:0010903	0.01228
M_128	GO:0006982	0.01228
M_128	GO:0033344	0.01232
M_128	GO:0030819	0.01534
M_128	GO:0030641	0.01619
M_128	GO:0072218	0.01634

M_128	GO:0019556	0.01634
M_128	GO:0019557	0.01634
M_128	GO:0071918	0.01634
M_128	GO:0002155	0.01634
M_128	GO:0060486	0.01634
M_128	GO:0046010	0.01634
M_128	GO:0010916	0.01634
M_128	GO:0010897	0.01634
M_128	GO:0021759	0.01634
M_128	GO:0030900	0.0193
M_128	GO:2000821	0.02038
M_128	GO:0038170	0.02038
M_128	GO:0060510	0.02038
M_128	GO:1990511	0.02038
M_128	GO:0010989	0.02038
M_128	GO:0051005	0.02038
M_128	GO:0001523	0.02101
M_128	GO:0007605	0.02226
M_128	GO:0030317	0.02243
M_128	GO:0034372	0.02408
M_128	GO:0035634	0.02441
M_128	GO:0009624	0.02441
M_128	GO:0035502	0.02441
M_128	GO:0030299	0.02804
M_128	GO:0003310	0.02842
M_128	GO:0030300	0.02842
M_128	GO:0035873	0.02842
M_128	GO:0042421	0.02842
M_128	GO:0060430	0.02842
M_128	GO:0021798	0.02842
M_128	GO:0032489	0.02842
M_128	GO:0072205	0.03242

M_128	GO:0035093	0.03242
M_128	GO:1902459	0.03242
M_128	GO:0010898	0.03242
M_128	GO:0035461	0.03242
M_128	GO:0042632	0.03539
M_128	GO:0061549	0.03639
M_128	GO:0007614	0.03639
M_128	GO:0034382	0.03639
M_128	GO:0070327	0.03639
M_128	GO:0046487	0.03639
M_128	GO:0006072	0.03639
M_128	GO:1990573	0.03639
M_128	GO:0051006	0.03639
M_128	GO:0010873	0.03639
M_128	GO:0060124	0.03639
M_128	GO:2001054	0.04036
M_128	GO:0034379	0.04036
M_128	GO:1902093	0.04036
M_128	GO:0043129	0.04036
M_128	GO:0038003	0.04036
M_128	GO:0042753	0.04372
M_128	GO:0021892	0.0443
M_128	GO:0021877	0.0443
M_128	GO:0021521	0.0443
M_128	GO:0045919	0.0443
M_128	GO:0031284	0.0443
M_128	GO:0033327	0.0443
M_128	GO:0019532	0.04823
M_128	GO:0006068	0.04823
M_128	GO:1902476	0.04981
M_129	GO:0048704	1.7E-12
M_129	GO:0009952	1.1E-09

M_129	GO:0045944	0.0000019
M_129	GO:0090190	0.000027
M_129	GO:1902725	0.000029
M_129	GO:0061551	0.000058
M_129	GO:0030910	0.000058
M_129	GO:0061055	0.000058
M_129	GO:0072095	0.000058
M_129	GO:0072107	0.000058
M_129	GO:0061197	0.000096
M_129	GO:0008582	0.000096
M_129	GO:0001580	0.00029
M_129	GO:2000117	0.00041
M_129	GO:0007601	0.00046
M_129	GO:0051451	0.00052
M_129	GO:0006068	0.00063
M_129	GO:0018298	0.00074
M_129	GO:0072075	0.00099
M_129	GO:0051923	0.00113
M_129	GO:0060216	0.00177
M_129	GO:0042474	0.00177
M_129	GO:0050427	0.00235
M_129	GO:0030878	0.00256
M_129	GO:0060037	0.003
M_129	GO:0044328	0.00314
M_129	GO:0044329	0.00314
M_129	GO:2001108	0.00314
M_129	GO:0044330	0.00314
M_129	GO:0021570	0.00314
M_129	GO:1904025	0.00314
M_129	GO:0035116	0.00347
M_129	GO:0000122	0.00411
M_129	GO:0035115	0.00424

M_129	GO:0032467	0.00569
M_129	GO:0021610	0.00616
M_129	GO:0051977	0.00627
M_129	GO:0097156	0.00627
M_129	GO:2001055	0.00627
M_129	GO:2000324	0.00627
M_129	GO:0014728	0.00627
M_129	GO:1990379	0.00627
M_129	GO:2000729	0.00627
M_129	GO:0071482	0.00649
M_129	GO:0048538	0.00841
M_129	GO:0048701	0.00917
M_129	GO:0021546	0.00922
M_129	GO:0097155	0.00939
M_129	GO:0090427	0.00939
M_129	GO:0021569	0.00939
M_129	GO:0035880	0.00939
M_129	GO:0010705	0.00939
M_129	GO:1902723	0.00939
M_129	GO:1902953	0.00939
M_129	GO:0008344	0.01002
M_129	GO:0006584	0.01204
M_129	GO:0002011	0.01204
M_129	GO:0048633	0.0125
M_129	GO:0090191	0.0125
M_129	GO:1901842	0.0125
M_129	GO:1990743	0.0125
M_129	GO:0021615	0.0125
M_129	GO:0072513	0.0125
M_129	GO:1902255	0.0125
M_129	GO:0072193	0.0125
M_129	GO:0009584	0.01337

M_129	GO:0007602	0.01337
M_129	GO:0050821	0.01386
M_129	GO:0045586	0.0156
M_129	GO:0070373	0.01672
M_129	GO:0090158	0.01869
M_129	GO:0021631	0.01869
M_129	GO:0014719	0.01869
M_129	GO:0098528	0.01869
M_129	GO:1902961	0.01869
M_129	GO:0090324	0.01869
M_129	GO:0021957	0.01869
M_129	GO:0042472	0.02028
M_129	GO:0061312	0.02177
M_129	GO:0021612	0.02177
M_129	GO:0014883	0.02177
M_129	GO:0060021	0.02263
M_129	GO:0001523	0.02357
M_129	GO:0050896	0.02397
M_129	GO:0009308	0.02419
M_129	GO:0014732	0.02485
M_129	GO:1903715	0.02485
M_129	GO:0035360	0.02485
M_129	GO:0071316	0.02485
M_129	GO:0006776	0.02485
M_129	GO:0048539	0.02485
M_129	GO:0048013	0.02616
M_129	GO:0014842	0.02741
M_129	GO:0015824	0.02791
M_129	GO:0015816	0.02791
M_129	GO:0046548	0.02791
M_129	GO:1900272	0.02791
M_129	GO:0035860	0.02791

M_129	GO:0008354	0.02791
M_129	GO:0048387	0.02791
M_129	GO:0001574	0.03096
M_129	GO:0014894	0.03096
M_129	GO:1902004	0.03096
M_129	GO:0008343	0.03096
M_129	GO:0007399	0.03245
M_129	GO:0010463	0.03347
M_129	GO:0060856	0.03401
M_129	GO:0003198	0.03401
M_129	GO:0050910	0.03401
M_129	GO:0072178	0.03401
M_129	GO:0048681	0.03704
M_129	GO:0070885	0.03704
M_129	GO:0045820	0.03704
M_129	GO:2001014	0.03865
M_129	GO:0001771	0.04007
M_129	GO:0048935	0.04007
M_129	GO:0006688	0.04234
M_129	GO:0060997	0.0424
M_129	GO:0030238	0.04308
M_129	GO:0043517	0.04308
M_129	GO:0006805	0.04525
M_129	GO:0009812	0.04609
M_129	GO:0007216	0.04609
M_129	GO:0050965	0.04609
M_129	GO:0071599	0.04609
M_129	GO:1903358	0.04609
M_129	GO:0045056	0.04909
M_130	GO:0044281	0.00036
M_130	GO:0051256	0.00044
M_130	GO:0071850	0.00103

M_130	GO:0007096	0.00149
M_130	GO:0006260	0.00243
M_130	GO:0009398	0.0032
M_130	GO:0046491	0.0032
M_130	GO:0046177	0.0032
M_130	GO:0070179	0.0032
M_130	GO:1990384	0.0032
M_130	GO:0010722	0.0032
M_130	GO:0006218	0.0032
M_130	GO:0009231	0.0032
M_130	GO:0035335	0.00391
M_130	GO:0006090	0.00467
M_130	GO:0045210	0.00638
M_130	GO:0005986	0.00638
M_130	GO:0018283	0.00638
M_130	GO:1904231	0.00638
M_130	GO:1904234	0.00638
M_130	GO:0006428	0.00638
M_130	GO:0072369	0.00638
M_130	GO:0002038	0.00638
M_130	GO:0060978	0.00956
M_130	GO:0031204	0.00956
M_130	GO:0035879	0.00956
M_130	GO:0097327	0.00956
M_130	GO:1902817	0.01273
M_130	GO:0045716	0.01273
M_130	GO:0042866	0.01273
M_130	GO:0019626	0.01273
M_130	GO:0071787	0.01273
M_130	GO:2000623	0.01273
M_130	GO:1990809	0.01273
M_130	GO:0006741	0.01273

M_130	GO:0006613	0.01568
M_130	GO:0061624	0.01588
M_130	GO:0044206	0.01588
M_130	GO:0002023	0.01588
M_130	GO:0046826	0.01903
M_130	GO:0006552	0.01903
M_130	GO:0071286	0.01903
M_130	GO:0006777	0.01903
M_130	GO:0060017	0.02217
M_130	GO:2000394	0.02217
M_130	GO:0006564	0.02217
M_130	GO:0033864	0.02217
M_130	GO:1902031	0.02217
M_130	GO:0044387	0.02529
M_130	GO:0090481	0.02529
M_130	GO:0006616	0.02529
M_130	GO:0030388	0.02529
M_130	GO:0006108	0.02529
M_130	GO:0003223	0.02529
M_130	GO:0051289	0.02643
M_130	GO:0048478	0.02841
M_130	GO:0060982	0.02841
M_130	GO:1902473	0.02841
M_130	GO:0001514	0.02841
M_130	GO:0006002	0.02841
M_130	GO:0010988	0.02841
M_130	GO:0008354	0.02841
M_130	GO:0002084	0.02841
M_130	GO:0097428	0.02841
M_130	GO:0006000	0.03097
M_130	GO:0019388	0.03152
M_130	GO:0006335	0.03152

M_130	GO:0042118	0.03152
M_130	GO:1902950	0.03152
M_130	GO:0045820	0.0377
M_130	GO:0060391	0.0377
M_130	GO:0048715	0.0377
M_130	GO:0071888	0.0377
M_130	GO:0006069	0.0377
M_130	GO:2000042	0.0377
M_130	GO:0006662	0.04078
M_130	GO:1900025	0.04078
M_130	GO:0072425	0.04078
M_130	GO:1904668	0.04078
M_130	GO:0016540	0.04385
M_130	GO:0051895	0.04691
M_130	GO:0097067	0.04691
M_130	GO:1902165	0.04691
M_130	GO:0003222	0.04996
M_131	GO:0042795	0.00093
M_131	GO:0032402	0.00179
M_131	GO:0007601	0.00241
M_131	GO:0010971	0.00247
M_131	GO:0071951	0.00274
M_131	GO:1901675	0.00274
M_131	GO:0040018	0.00368
M_131	GO:0051595	0.00547
M_131	GO:2000342	0.00547
M_131	GO:0060324	0.00765
M_131	GO:0010796	0.0082
M_131	GO:1902268	0.0082
M_131	GO:0060743	0.0082
M_131	GO:0090170	0.01092
M_131	GO:0038108	0.01092

M_131	GO:0050893	0.01092
M_131	GO:0070213	0.01092
M_131	GO:0046726	0.01092
M_131	GO:0045415	0.01092
M_131	GO:0060296	0.01363
M_131	GO:0070375	0.01363
M_131	GO:0045053	0.01363
M_131	GO:0044770	0.01543
M_131	GO:0060440	0.01633
M_131	GO:0060613	0.01633
M_131	GO:0043111	0.01633
M_131	GO:1903546	0.01903
M_131	GO:0071499	0.01903
M_131	GO:0030953	0.01903
M_131	GO:0044829	0.01903
M_131	GO:0051300	0.01903
M_131	GO:0045732	0.01945
M_131	GO:0048227	0.02171
M_131	GO:0007042	0.02171
M_131	GO:0051152	0.02171
M_131	GO:0031937	0.02171
M_131	GO:0060020	0.02171
M_131	GO:0006987	0.02171
M_131	GO:0016926	0.02171
M_131	GO:1903800	0.02171
M_131	GO:0046548	0.0244
M_131	GO:0034498	0.0244
M_131	GO:0060502	0.0244
M_131	GO:0034454	0.0244
M_131	GO:0021702	0.0244
M_131	GO:0034115	0.0244
M_131	GO:0048478	0.0244

M_131	GO:0032436	0.02632
M_131	GO:0007016	0.02707
M_131	GO:0006384	0.02707
M_131	GO:0033235	0.02707
M_131	GO:0035845	0.02707
M_131	GO:0021697	0.02914
M_131	GO:0043619	0.02974
M_131	GO:0031987	0.02974
M_131	GO:0033234	0.02974
M_131	GO:2000291	0.02974
M_131	GO:0060442	0.0324
M_131	GO:1902855	0.0324
M_131	GO:0030497	0.0324
M_131	GO:0010952	0.03464
M_131	GO:0009642	0.03505
M_131	GO:0033262	0.03505
M_131	GO:0006596	0.03505
M_131	GO:0045198	0.03505
M_131	GO:0042761	0.03505
M_131	GO:0000187	0.0353
M_131	GO:2001224	0.0377
M_131	GO:1903351	0.0377
M_131	GO:0042754	0.0377
M_131	GO:0090051	0.04034
M_131	GO:0034356	0.04034
M_131	GO:0051457	0.04297
M_131	GO:0031573	0.04297
M_131	GO:2000001	0.04297
M_131	GO:0003085	0.04297
M_131	GO:0032465	0.04342
M_131	GO:0072673	0.04559
M_131	GO:0021756	0.04559

M_131	GO:2000641	0.04559
M_131	GO:0007346	0.04677
M_131	GO:0032872	0.04775
M_131	GO:0000076	0.04821
M_131	GO:0071539	0.04821
M_131	GO:0001573	0.04821
M_131	GO:0045893	0.04918
M_132	GO:0043923	0.0013
M_132	GO:0006337	0.0017
M_132	GO:0006491	0.0017
M_132	GO:0061014	0.0029
M_132	GO:0035128	0.0032
M_132	GO:0100057	0.0032
M_132	GO:1901581	0.0032
M_132	GO:1901582	0.0032
M_132	GO:0016032	0.0033
M_132	GO:0006357	0.0051
M_132	GO:0051123	0.0056
M_132	GO:2000249	0.0056
M_132	GO:0031076	0.0062
M_132	GO:0030702	0.0064
M_132	GO:0030822	0.0064
M_132	GO:0003430	0.0064
M_132	GO:0002635	0.0064
M_132	GO:0010768	0.0064
M_132	GO:1900369	0.0064
M_132	GO:0045944	0.0077
M_132	GO:0051607	0.0086
M_132	GO:1904222	0.0096
M_132	GO:1904219	0.0096
M_132	GO:0060830	0.0096
M_132	GO:0045071	0.012

M_132	GO:2000825	0.0127
M_132	GO:0061484	0.0127
M_132	GO:0061470	0.0127
M_132	GO:0070384	0.0127
M_132	GO:0031048	0.0127
M_132	GO:0061055	0.0127
M_132	GO:0036369	0.0127
M_132	GO:1904382	0.0127
M_132	GO:0034242	0.0127
M_132	GO:0009597	0.0159
M_132	GO:2000051	0.0159
M_132	GO:0006306	0.0163
M_132	GO:0048278	0.0189
M_132	GO:0010571	0.019
M_132	GO:0003406	0.019
M_132	GO:1903026	0.019
M_132	GO:1901165	0.019
M_132	GO:0071300	0.0205
M_132	GO:0042795	0.0211
M_132	GO:0006382	0.0222
M_132	GO:0006564	0.0222
M_132	GO:0035280	0.0222
M_132	GO:0072711	0.0222
M_132	GO:0044387	0.0253
M_132	GO:0090435	0.0253
M_132	GO:0030916	0.0253
M_132	GO:0032927	0.0253
M_132	GO:0048793	0.0253
M_132	GO:0060534	0.0253
M_132	GO:1900364	0.0253
M_132	GO:1904153	0.0284
M_132	GO:0015886	0.0284

M_132	GO:0015825	0.0284
M_132	GO:2000320	0.0284
M_132	GO:0048096	0.0284
M_132	GO:1900112	0.0284
M_132	GO:0006621	0.0284
M_132	GO:2000628	0.0284
M_132	GO:0060339	0.0284
M_132	GO:1901409	0.0284
M_132	GO:0001188	0.0284
M_132	GO:0006338	0.0291
M_132	GO:0035721	0.0315
M_132	GO:0090161	0.0346
M_132	GO:0072520	0.0346
M_132	GO:0046007	0.0346
M_132	GO:1902806	0.0373
M_132	GO:0015747	0.0377
M_132	GO:0045893	0.0399
M_132	GO:0000212	0.0408
M_132	GO:0071318	0.0408
M_132	GO:0043547	0.0409
M_132	GO:0060009	0.0438
M_132	GO:0042953	0.0438
M_132	GO:0061158	0.0438
M_132	GO:0031054	0.0438
M_132	GO:0060831	0.0438
M_132	GO:0070198	0.0438
M_132	GO:2000059	0.0438
M_132	GO:1904380	0.0438
M_132	GO:0032486	0.0438
M_132	GO:0000183	0.0469
M_132	GO:0030889	0.05
M_132	GO:1903830	0.05

M_133	GO:0060748	0.000042
M_133	GO:2000310	0.0016
M_133	GO:0032332	0.0023
M_133	GO:0010842	0.0031
M_133	GO:0010738	0.0037
M_133	GO:1900827	0.0038
M_133	GO:0061358	0.0038
M_133	GO:0097640	0.0038
M_133	GO:0097639	0.0038
M_133	GO:0019102	0.0038
M_133	GO:0060807	0.0038
M_133	GO:0060520	0.0038
M_133	GO:0001543	0.0038
M_133	GO:0003142	0.0038
M_133	GO:0002537	0.0038
M_133	GO:0072660	0.0038
M_133	GO:0010650	0.0075
M_133	GO:0007493	0.0075
M_133	GO:2000043	0.0075
M_133	GO:0002121	0.0075
M_133	GO:0031635	0.0075
M_133	GO:0001828	0.0075
M_133	GO:0060599	0.0075
M_133	GO:0021740	0.0075
M_133	GO:0045720	0.0075
M_133	GO:0048866	0.0075
M_133	GO:0048286	0.0086
M_133	GO:0007200	0.01
M_133	GO:1903445	0.0113
M_133	GO:0060913	0.0113
M_133	GO:0060769	0.0113
M_133	GO:1902811	0.0113

M_133	GO:2000768	0.0113
M_133	GO:0072139	0.0113
M_133	GO:0097638	0.0113
M_133	GO:0010133	0.0113
M_133	GO:1904158	0.0113
M_133	GO:0034436	0.0113
M_133	GO:0045726	0.0113
M_133	GO:0061010	0.0113
M_133	GO:0061031	0.0113
M_133	GO:0033634	0.015
M_133	GO:0048808	0.015
M_133	GO:0060214	0.015
M_133	GO:1901318	0.015
M_133	GO:0019470	0.015
M_133	GO:0042997	0.015
M_133	GO:2000741	0.015
M_133	GO:0003308	0.015
M_133	GO:0038026	0.015
M_133	GO:0071315	0.015
M_133	GO:0006651	0.015
M_133	GO:0042662	0.015
M_133	GO:1902261	0.015
M_133	GO:0032100	0.015
M_133	GO:0003382	0.0164
M_133	GO:0046545	0.0182
M_133	GO:0015705	0.0187
M_133	GO:0006798	0.0187
M_133	GO:0061009	0.0187
M_133	GO:0034112	0.0223
M_133	GO:0071286	0.0224
M_133	GO:0060956	0.0224
M_133	GO:0055059	0.0224

M_133	GO:0032534	0.0224
M_133	GO:0038001	0.0224
M_133	GO:0060316	0.0224
M_133	GO:0072112	0.0257
M_133	GO:0051057	0.0258
M_133	GO:1900038	0.0261
M_133	GO:2000766	0.0261
M_133	GO:0060352	0.0261
M_133	GO:0097107	0.0261
M_133	GO:1902260	0.0261
M_133	GO:0001542	0.0293
M_133	GO:0050847	0.0298
M_133	GO:2000035	0.0298
M_133	GO:0072102	0.0298
M_133	GO:0090281	0.0298
M_133	GO:0061051	0.0298
M_133	GO:0071233	0.0334
M_133	GO:0048617	0.0334
M_133	GO:0034447	0.0334
M_133	GO:1904263	0.0334
M_133	GO:0071394	0.0334
M_133	GO:0043031	0.0334
M_133	GO:1990253	0.0371
M_133	GO:0007191	0.0371
M_133	GO:0033148	0.0371
M_133	GO:0051124	0.0371
M_133	GO:0007016	0.0371
M_133	GO:0006809	0.0402
M_133	GO:0030241	0.0407
M_133	GO:0042135	0.0407
M_133	GO:0060736	0.0407
M_133	GO:0010960	0.0407

M_133	GO:0043116	0.0407
M_133	GO:0072015	0.0407
M_133	GO:0006527	0.0407
M_133	GO:0045945	0.0407
M_133	GO:0072520	0.0407
M_133	GO:0033327	0.0407
M_133	GO:1902476	0.0426
M_133	GO:0010524	0.0437
M_133	GO:0048016	0.0437
M_133	GO:0001508	0.0439
M_133	GO:0001678	0.0439
M_133	GO:0060742	0.0443
M_133	GO:0021903	0.0443
M_133	GO:2000651	0.0443
M_133	GO:0090630	0.0462
M_133	GO:0003073	0.0471
M_133	GO:0010765	0.0472
M_133	GO:0043568	0.0479
M_133	GO:0060732	0.0479
M_133	GO:0032802	0.0479
M_133	GO:0000052	0.0479
M_133	GO:0072001	0.0484
M_134	GO:0016254	0.001
M_134	GO:0035543	0.0032
M_134	GO:0046732	0.0032
M_134	GO:0032455	0.0032
M_134	GO:0034642	0.0032
M_134	GO:0042986	0.0064
M_134	GO:0044210	0.0064
M_134	GO:0014005	0.0096
M_134	GO:0051612	0.0096
M_134	GO:0061205	0.0127

M_134	GO:0002175	0.0127
M_134	GO:0070236	0.0127
M_134	GO:1990414	0.0127
M_134	GO:0070370	0.0127
M_134	GO:0072659	0.0155
M_134	GO:0015853	0.0159
M_134	GO:0098735	0.0159
M_134	GO:0071205	0.0159
M_134	GO:1902514	0.019
M_134	GO:0043111	0.019
M_134	GO:0038203	0.0222
M_134	GO:0010523	0.0222
M_134	GO:0030913	0.0222
M_134	GO:0045162	0.0222
M_134	GO:0007422	0.0246
M_134	GO:0048630	0.0253
M_134	GO:0009893	0.0258
M_134	GO:0090073	0.0284
M_134	GO:0070050	0.0284
M_134	GO:1990314	0.0284
M_134	GO:0015866	0.0284
M_134	GO:0070327	0.0284
M_134	GO:0072177	0.0284
M_134	GO:0034379	0.0315
M_134	GO:0001731	0.0315
M_134	GO:0060287	0.0315
M_134	GO:0016575	0.0316
M_134	GO:0015867	0.0346
M_134	GO:0006527	0.0346
M_134	GO:0031284	0.0346
M_134	GO:0048672	0.0346
M_134	GO:0010508	0.0364

M_134	GO:0038180	0.0377
M_134	GO:2000042	0.0377
M_134	GO:0043097	0.0377
M_134	GO:0018119	0.0408
M_134	GO:0048642	0.0408
M_134	GO:0045814	0.0433
M_134	GO:0034435	0.0438
M_134	GO:0060993	0.0463
M_134	GO:2000696	0.0469
M_134	GO:0035372	0.0469
M_134	GO:1902187	0.0469
M_134	GO:0040011	0.0476
M_134	GO:0010878	0.05
M_134	GO:1902307	0.05
M_134	GO:0043968	0.05
M_135	GO:2000601	0.00034
M_135	GO:0016032	0.00238
M_135	GO:0045454	0.00254
M_135	GO:0016601	0.00286
M_135	GO:0046223	0.00405
M_135	GO:0071788	0.00405
M_135	GO:0071045	0.00405
M_135	GO:0034670	0.00405
M_135	GO:1903788	0.00405
M_135	GO:0003252	0.00405
M_135	GO:0052066	0.00405
M_135	GO:0048010	0.00565
M_135	GO:0032915	0.00809
M_135	GO:0032916	0.00809
M_135	GO:0014916	0.00809
M_135	GO:0006529	0.00809
M_135	GO:2000536	0.00809

M_135	GO:0036228	0.00809
M_135	GO:0071460	0.00809
M_135	GO:0006431	0.00809
M_135	GO:0048812	0.01143
M_135	GO:0035735	0.01144
M_135	GO:0010970	0.01152
M_135	GO:1902037	0.01211
M_135	GO:1902731	0.01211
M_135	GO:0071596	0.01211
M_135	GO:0035022	0.01211
M_135	GO:0090156	0.01211
M_135	GO:0045715	0.01211
M_135	GO:0043277	0.01314
M_135	GO:0008542	0.01433
M_135	GO:1900060	0.01611
M_135	GO:0031591	0.01611
M_135	GO:0006741	0.01611
M_135	GO:0043490	0.01611
M_135	GO:0044528	0.01611
M_135	GO:0002949	0.01611
M_135	GO:0070778	0.01611
M_135	GO:0006574	0.01611
M_135	GO:0045906	0.01611
M_135	GO:1903373	0.01611
M_135	GO:0006409	0.01611
M_135	GO:0015798	0.01611
M_135	GO:0035621	0.01611
M_135	GO:0061419	0.0201
M_135	GO:0038027	0.0201
M_135	GO:0038044	0.0201
M_135	GO:2000582	0.0201
M_135	GO:0060509	0.0201

M_135	GO:0003186	0.0201
M_135	GO:0051036	0.0201
M_135	GO:0036091	0.0201
M_135	GO:1902065	0.02407
M_135	GO:0016255	0.02407
M_135	GO:0042791	0.02407
M_135	GO:0042797	0.02407
M_135	GO:0060136	0.02407
M_135	GO:0050748	0.02407
M_135	GO:0008380	0.02667
M_135	GO:0072577	0.02771
M_135	GO:0043353	0.02803
M_135	GO:1903071	0.02803
M_135	GO:0000972	0.02803
M_135	GO:0021798	0.02803
M_135	GO:0071499	0.02803
M_135	GO:0060430	0.02803
M_135	GO:1903608	0.02803
M_135	GO:0048842	0.02803
M_135	GO:0009414	0.03197
M_135	GO:0061626	0.03197
M_135	GO:0060836	0.03197
M_135	GO:0042048	0.03197
M_135	GO:0001672	0.03197
M_135	GO:0030422	0.03197
M_135	GO:0006468	0.03238
M_135	GO:0045333	0.03484
M_135	GO:0042073	0.03498
M_135	GO:0042473	0.0359
M_135	GO:2000234	0.0359
M_135	GO:0042780	0.0359
M_135	GO:0010944	0.0359

M_135	GO:0006348	0.0359
M_135	GO:1990314	0.0359
M_135	GO:0075522	0.03981
M_135	GO:0033148	0.03981
M_135	GO:0035721	0.03981
M_135	GO:0003183	0.03981
M_135	GO:0001682	0.03981
M_135	GO:0006335	0.03981
M_135	GO:1902916	0.03981
M_135	GO:2000121	0.03981
M_135	GO:1904385	0.0437
M_135	GO:0033690	0.0437
M_135	GO:0045899	0.0437
M_135	GO:0007409	0.04499
M_135	GO:0018206	0.04758
M_136	GO:0030576	0.000032
M_136	GO:0042026	0.0031
M_136	GO:1904589	0.0033
M_136	GO:0060904	0.0033
M_136	GO:0072229	0.0033
M_136	GO:1990832	0.0033
M_136	GO:1902860	0.0033
M_136	GO:2000011	0.0033
M_136	GO:1902904	0.0033
M_136	GO:0018160	0.0033
M_136	GO:1990261	0.0033
M_136	GO:0051085	0.0039
M_136	GO:0071480	0.0039
M_136	GO:0070979	0.0044
M_136	GO:0006099	0.005
M_136	GO:0045292	0.0056
M_136	GO:0035407	0.0066

M_136	GO:1904313	0.0066
M_136	GO:0019074	0.0066
M_136	GO:0001834	0.0066
M_136	GO:0006550	0.0066
M_136	GO:0021589	0.0066
M_136	GO:0046356	0.0066
M_136	GO:1903382	0.0066
M_136	GO:0021691	0.0066
M_136	GO:0070317	0.007
M_136	GO:1900034	0.0093
M_136	GO:0061684	0.0099
M_136	GO:0010767	0.0099
M_136	GO:0071931	0.0099
M_136	GO:0072318	0.0099
M_136	GO:1903895	0.0099
M_136	GO:0002353	0.0099
M_136	GO:0031204	0.0099
M_136	GO:0071320	0.0128
M_136	GO:0033489	0.0132
M_136	GO:0002155	0.0132
M_136	GO:2000672	0.0132
M_136	GO:1903891	0.0132
M_136	GO:0046952	0.0132
M_136	GO:0006121	0.0132
M_136	GO:0033490	0.0132
M_136	GO:0000077	0.0155
M_136	GO:0006085	0.0162
M_136	GO:0035519	0.0164
M_136	GO:0090074	0.0164
M_136	GO:2001034	0.0164
M_136	GO:1903298	0.0164
M_136	GO:0006489	0.0164

M_136	GO:0010032	0.0164
M_136	GO:0044314	0.0164
M_136	GO:0019348	0.0164
M_136	GO:0051321	0.0167
M_136	GO:0000381	0.0174
M_136	GO:0032981	0.019
M_136	GO:0015760	0.0197
M_136	GO:0010994	0.0197
M_136	GO:0061087	0.0197
M_136	GO:0048208	0.0202
M_136	GO:0007032	0.0225
M_136	GO:0006729	0.0229
M_136	GO:0044829	0.0229
M_136	GO:0006782	0.0262
M_136	GO:0006987	0.0262
M_136	GO:0006975	0.0262
M_136	GO:0072321	0.0262
M_136	GO:2000643	0.0262
M_136	GO:0039536	0.0262
M_136	GO:0085020	0.0262
M_136	GO:0000724	0.0294
M_136	GO:0061635	0.0294
M_136	GO:0048096	0.0294
M_136	GO:0046951	0.0294
M_136	GO:0071287	0.0294
M_136	GO:0035542	0.0326
M_136	GO:0042023	0.0326
M_136	GO:0034058	0.0326
M_136	GO:1903897	0.0326
M_136	GO:1904322	0.0358
M_136	GO:1990440	0.0358
M_136	GO:0006983	0.0358

M_136	GO:2000615	0.0358
M_136	GO:0001833	0.0358
M_136	GO:0021554	0.0358
M_136	GO:0031647	0.0367
M_136	GO:0035563	0.039
M_136	GO:0035437	0.039
M_136	GO:0033120	0.0415
M_136	GO:0046602	0.0422
M_136	GO:0015937	0.0422
M_136	GO:0090399	0.0422
M_136	GO:1904668	0.0422
M_136	GO:0072425	0.0422
M_136	GO:0006342	0.0434
M_136	GO:0021510	0.0441
M_136	GO:0033169	0.0454
M_136	GO:0051645	0.0454
M_136	GO:0032688	0.0454
M_136	GO:0035435	0.0454
M_136	GO:0034620	0.0472
M_136	GO:0050687	0.0478
M_136	GO:0045116	0.0485
M_137	GO:1902176	0.001
M_137	GO:0050999	0.0013
M_137	GO:1990134	0.0024
M_137	GO:0060709	0.0024
M_137	GO:0045429	0.0035
M_137	GO:0071140	0.0048
M_137	GO:1902499	0.0048
M_137	GO:0043335	0.0048
M_137	GO:1903087	0.0048
M_137	GO:0072355	0.0048
M_137	GO:1990575	0.0048

M_137	GO:0052746	0.0048
M_137	GO:1900034	0.005
M_137	GO:0035617	0.0072
M_137	GO:0061684	0.0072
M_137	GO:0051343	0.0072
M_137	GO:0060741	0.0072
M_137	GO:0044208	0.0072
M_137	GO:1903721	0.0072
M_137	GO:0089709	0.0072
M_137	GO:1990418	0.0072
M_137	GO:0043161	0.009
M_137	GO:0072655	0.0092
M_137	GO:0090657	0.0096
M_137	GO:0032079	0.0096
M_137	GO:1903400	0.0096
M_137	GO:1903401	0.0096
M_137	GO:0061084	0.0096
M_137	GO:0031999	0.0096
M_137	GO:0010748	0.0096
M_137	GO:0051300	0.0116
M_137	GO:0006844	0.0119
M_137	GO:0072656	0.0119
M_137	GO:0043987	0.0143
M_137	GO:0009912	0.0143
M_137	GO:0090166	0.0143
M_137	GO:0008063	0.0143
M_137	GO:0060316	0.0143
M_137	GO:0070141	0.0143
M_137	GO:1902949	0.0143
M_137	GO:0045040	0.0143
M_137	GO:0051444	0.0163
M_137	GO:0031659	0.0167

M_137	GO:0032287	0.0167
M_137	GO:0034393	0.0167
M_137	GO:1901844	0.0167
M_137	GO:0009304	0.0167
M_137	GO:0090267	0.0167
M_137	GO:0007084	0.0167
M_137	GO:0036289	0.019
M_137	GO:0042492	0.019
M_137	GO:0035331	0.019
M_137	GO:0009249	0.019
M_137	GO:0042148	0.019
M_137	GO:0071493	0.019
M_137	GO:0035655	0.019
M_137	GO:0000707	0.019
M_137	GO:0010033	0.0194
M_137	GO:0097011	0.0214
M_137	GO:0100002	0.0214
M_137	GO:0060315	0.0214
M_137	GO:0010918	0.0214
M_137	GO:0006924	0.0214
M_137	GO:0045792	0.0237
M_137	GO:0018026	0.0237
M_137	GO:0010452	0.0237
M_137	GO:0006384	0.0237
M_137	GO:0001731	0.0237
M_137	GO:0032957	0.0237
M_137	GO:0033138	0.0248
M_137	GO:0030162	0.0255
M_137	GO:0045945	0.0261
M_137	GO:0045899	0.0261
M_137	GO:0006531	0.0261
M_137	GO:0005513	0.0261

M_137	GO:0080009	0.0261
M_137	GO:0010763	0.0261
M_137	GO:0000722	0.0261
M_137	GO:0031396	0.0282
M_137	GO:0071257	0.0284
M_137	GO:0007077	0.0284
M_137	GO:0045475	0.0284
M_137	GO:0016579	0.0285
M_137	GO:0034497	0.0307
M_137	GO:0010801	0.0307
M_137	GO:0070897	0.0323
M_137	GO:0046040	0.0331
M_137	GO:0090083	0.0354
M_137	GO:0008631	0.036
M_137	GO:1901796	0.0361
M_137	GO:0000165	0.0363
M_137	GO:1901800	0.037
M_137	GO:0090656	0.0377
M_137	GO:0045725	0.0377
M_137	GO:2000010	0.0377
M_137	GO:0032516	0.0377
M_137	GO:0010881	0.0377
M_137	GO:1903364	0.0384
M_137	GO:0018027	0.04
M_137	GO:0071236	0.04
M_137	GO:0090201	0.04
M_137	GO:0071380	0.04
M_137	GO:0060644	0.04
M_137	GO:0046907	0.0421
M_137	GO:0031952	0.0436
M_137	GO:0051000	0.0446
M_137	GO:0060716	0.0469

M_137	GO:0051131	0.0469
M_137	GO:0045061	0.0469
M_137	GO:0042026	0.0481
M_138	GO:0007168	0.00029
M_138	GO:0046835	0.00168
M_138	GO:1904028	0.00234
M_138	GO:0006182	0.00434
M_138	GO:0010848	0.00468
M_138	GO:0030719	0.00468
M_138	GO:1902490	0.00468
M_138	GO:1903758	0.00468
M_138	GO:0048210	0.007
M_138	GO:1902897	0.007
M_138	GO:0035359	0.007
M_138	GO:2001244	0.0076
M_138	GO:0051594	0.00933
M_138	GO:0033133	0.00933
M_138	GO:0048386	0.00933
M_138	GO:1902952	0.00933
M_138	GO:0035522	0.00933
M_138	GO:0006003	0.01165
M_138	GO:0032024	0.01199
M_138	GO:0032482	0.01331
M_138	GO:0006007	0.01364
M_138	GO:0060430	0.01627
M_138	GO:0072383	0.01627
M_138	GO:0044351	0.01627
M_138	GO:0070197	0.01857
M_138	GO:0047497	0.01857
M_138	GO:0036066	0.01857
M_138	GO:0048539	0.01857
M_138	GO:0032464	0.02087

M_138	GO:0070072	0.02316
M_138	GO:0034378	0.02316
M_138	GO:0051223	0.02387
M_138	GO:1903441	0.02545
M_138	GO:0042535	0.02545
M_138	GO:0006915	0.02583
M_138	GO:0045721	0.03001
M_138	GO:0050862	0.03001
M_138	GO:0070863	0.03001
M_138	GO:0006089	0.03001
M_138	GO:0001921	0.03228
M_138	GO:0035810	0.03228
M_138	GO:1902259	0.03455
M_138	GO:0050965	0.03455
M_138	GO:0035815	0.03455
M_138	GO:0006000	0.03455
M_138	GO:0045725	0.03681
M_138	GO:2000010	0.03681
M_138	GO:0006123	0.03681
M_138	GO:0006004	0.03681
M_138	GO:0044320	0.03907
M_138	GO:0007100	0.04132
M_138	GO:0045821	0.04132
M_138	GO:0070935	0.04132
M_138	GO:0006110	0.04476
M_138	GO:0043046	0.0458
M_138	GO:0043266	0.04707
M_139	GO:0010778	0.0021
M_139	GO:0002270	0.0021
M_139	GO:0031619	0.0021
M_139	GO:0043947	0.0021
M_139	GO:0032072	0.0021

M_139	GO:0043150	0.0021
M_139	GO:0070124	0.0042
M_139	GO:0046294	0.0042
M_139	GO:2000426	0.0042
M_139	GO:0017055	0.0042
M_139	GO:0045819	0.0042
M_139	GO:1990426	0.0042
M_139	GO:1990575	0.0042
M_139	GO:0034165	0.0042
M_139	GO:0032425	0.0042
M_139	GO:0002840	0.0042
M_139	GO:0000278	0.0055
M_139	GO:0051562	0.0063
M_139	GO:0006203	0.0063
M_139	GO:2000819	0.0063
M_139	GO:0034473	0.0063
M_139	GO:0034476	0.0063
M_139	GO:0035711	0.0063
M_139	GO:0051598	0.0084
M_139	GO:2001200	0.0084
M_139	GO:0051106	0.0084
M_139	GO:0033600	0.0084
M_139	GO:1903751	0.0084
M_139	GO:0000281	0.0096
M_139	GO:0070200	0.0105
M_139	GO:0097350	0.0105
M_139	GO:1903753	0.0105
M_139	GO:0034137	0.0105
M_139	GO:0042262	0.0105
M_139	GO:0010606	0.0105
M_139	GO:1900264	0.0126
M_139	GO:0051135	0.0126

M_139	GO:0045345	0.0126
M_139	GO:0071042	0.0126
M_139	GO:0000467	0.0147
M_139	GO:0071038	0.0147
M_139	GO:0061511	0.0147
M_139	GO:0032790	0.0168
M_139	GO:0034475	0.0168
M_139	GO:1902857	0.0168
M_139	GO:0071035	0.0168
M_139	GO:0006627	0.0168
M_139	GO:0006265	0.0189
M_139	GO:0048304	0.0189
M_139	GO:0048478	0.0189
M_139	GO:1903748	0.0189
M_139	GO:0051561	0.0209
M_139	GO:0036444	0.0209
M_139	GO:0034427	0.0209
M_139	GO:0000290	0.0209
M_139	GO:0034145	0.0209
M_139	GO:0071639	0.0209
M_139	GO:0000050	0.023
M_139	GO:0002281	0.023
M_139	GO:0001833	0.023
M_139	GO:0000722	0.023
M_139	GO:0000731	0.0244
M_139	GO:0051298	0.0245
M_139	GO:0051103	0.0251
M_139	GO:0071028	0.0271
M_139	GO:0043248	0.0292
M_139	GO:0033151	0.0292
M_139	GO:0015879	0.0292
M_139	GO:0010225	0.0292

M_139	GO:0034656	0.0312
M_139	GO:2000001	0.0333
M_139	GO:0002218	0.0348
M_139	GO:0002643	0.0353
M_139	GO:0045063	0.0353
M_139	GO:0046599	0.0353
M_139	GO:0090026	0.0374
M_139	GO:0006978	0.0394
M_139	GO:0010389	0.0399
M_139	GO:0006271	0.0414
M_139	GO:1990774	0.0414
M_139	GO:0043371	0.0414
M_139	GO:0070987	0.0414
M_139	GO:0048025	0.0414
M_139	GO:0032727	0.0435
M_139	GO:0006309	0.0435
M_139	GO:0042276	0.0435
M_139	GO:0007064	0.0455
M_139	GO:1901687	0.0455
M_139	GO:0006401	0.0456
M_139	GO:1902017	0.0463
M_139	GO:0044458	0.0475
M_139	GO:0002407	0.0475
M_139	GO:0007026	0.0475
M_139	GO:0032201	0.0475
M_139	GO:0007141	0.0475
M_139	GO:0006297	0.0475
M_139	GO:0042738	0.0495
M_139	GO:0042104	0.0495
M_139	GO:0001556	0.0495
M_139	GO:2000778	0.0495
M_140	GO:0006627	0.00024

M_140	GO:1902504	0.00297
M_140	GO:0051068	0.00297
M_140	GO:1903999	0.00593
M_140	GO:0061300	0.00593
M_140	GO:0009106	0.00593
M_140	GO:0070292	0.00593
M_140	GO:0006393	0.00593
M_140	GO:0060152	0.00593
M_140	GO:0035425	0.00888
M_140	GO:0070291	0.00888
M_140	GO:2000825	0.01182
M_140	GO:0002943	0.01182
M_140	GO:0043490	0.01182
M_140	GO:2000587	0.01182
M_140	GO:0070778	0.01182
M_140	GO:0015031	0.01209
M_140	GO:0055118	0.01475
M_140	GO:0045454	0.01689
M_140	GO:0061732	0.01768
M_140	GO:0035900	0.01768
M_140	GO:0006880	0.01768
M_140	GO:0006091	0.0196
M_140	GO:0006283	0.01986
M_140	GO:1901844	0.0206
M_140	GO:0030433	0.02411
M_140	GO:0060315	0.0264
M_140	GO:0006890	0.02695
M_140	GO:0018095	0.0293
M_140	GO:0010459	0.0293
M_140	GO:0048280	0.0293
M_140	GO:0000338	0.0293
M_140	GO:0045899	0.03218

M_140	GO:0016558	0.03505
M_140	GO:0051791	0.03505
M_140	GO:0010826	0.03505
M_140	GO:1901077	0.03505
M_140	GO:0022904	0.03595
M_140	GO:1901841	0.03792
M_140	GO:0010389	0.03833
M_140	GO:0006122	0.04078
M_140	GO:0009437	0.04078
M_140	GO:0033540	0.04363
M_140	GO:1901800	0.04583
M_140	GO:0036159	0.04647
M_140	GO:0006465	0.04647
M_140	GO:0060628	0.04647
M_141	GO:2000271	0.00012
M_141	GO:0030036	0.00032
M_141	GO:0007049	0.0011
M_141	GO:0048015	0.00207
M_141	GO:0050668	0.00245
M_141	GO:0016036	0.00245
M_141	GO:0046786	0.00245
M_141	GO:0006336	0.00433
M_141	GO:0030702	0.0049
M_141	GO:0043314	0.0049
M_141	GO:1900110	0.0049
M_141	GO:0006478	0.0049
M_141	GO:0019858	0.0049
M_141	GO:0051171	0.00505
M_141	GO:0048538	0.00521
M_141	GO:0015966	0.00735
M_141	GO:1902661	0.00735
M_141	GO:0060313	0.00735

M_141	GO:0060268	0.00978
M_141	GO:0045963	0.00978
M_141	GO:0090170	0.00978
M_141	GO:1900113	0.00978
M_141	GO:0035814	0.00978
M_141	GO:0032747	0.01221
M_141	GO:0045046	0.01464
M_141	GO:0016576	0.01464
M_141	GO:2000664	0.01464
M_141	GO:2000667	0.01464
M_141	GO:0060440	0.01464
M_141	GO:0097345	0.01671
M_141	GO:0060017	0.01706
M_141	GO:0023041	0.01706
M_141	GO:0090240	0.01706
M_141	GO:2000617	0.01706
M_141	GO:0006919	0.01888
M_141	GO:0044387	0.01947
M_141	GO:0060020	0.01947
M_141	GO:0061308	0.01947
M_141	GO:0002692	0.01947
M_141	GO:0042420	0.01947
M_141	GO:0097400	0.02188
M_141	GO:0097011	0.02188
M_141	GO:0007614	0.02188
M_141	GO:0042473	0.02188
M_141	GO:0006265	0.02188
M_141	GO:0010918	0.02188
M_141	GO:0032464	0.02188
M_141	GO:0097428	0.02188
M_141	GO:0001188	0.02188
M_141	GO:0007155	0.02253

M_141	GO:1901838	0.02428
M_141	GO:0031053	0.02428
M_141	GO:0072584	0.02428
M_141	GO:0072537	0.02428
M_141	GO:0042135	0.02668
M_141	GO:0048681	0.02907
M_141	GO:0016558	0.02907
M_141	GO:0097284	0.02907
M_141	GO:0039692	0.02907
M_141	GO:0033160	0.02907
M_141	GO:0046854	0.03035
M_141	GO:0039694	0.03308
M_141	GO:0065002	0.03315
M_141	GO:0022038	0.03383
M_141	GO:1903351	0.03383
M_141	GO:0097067	0.03621
M_141	GO:0034380	0.03621
M_141	GO:1900017	0.03857
M_141	GO:0035970	0.03857
M_141	GO:0015074	0.03857
M_141	GO:0043923	0.04094
M_141	GO:2000641	0.04094
M_141	GO:1904355	0.04094
M_141	GO:0006337	0.04564
M_141	GO:0051170	0.04741
M_141	GO:0060216	0.04799
M_141	GO:0060716	0.04799
M_141	GO:0016226	0.04799
M_142	GO:0031643	0.000012
M_142	GO:0003222	0.000023
M_142	GO:0035910	0.000074
M_142	GO:2000543	0.00034

M_142	GO:0060038	0.00053
M_142	GO:0003184	0.00079
M_142	GO:0001964	0.00091
M_142	GO:0003214	0.00109
M_142	GO:0003215	0.00203
M_142	GO:0060716	0.00225
M_142	GO:0051048	0.00245
M_142	GO:0048485	0.00248
M_142	GO:0043576	0.00297
M_142	GO:0006593	0.00354
M_142	GO:0035499	0.00354
M_142	GO:1904268	0.00354
M_142	GO:1902178	0.00354
M_142	GO:0021793	0.00354
M_142	GO:0060354	0.00354
M_142	GO:0021703	0.00354
M_142	GO:2001311	0.00354
M_142	GO:0097379	0.00354
M_142	GO:1903701	0.00354
M_142	GO:2000723	0.00354
M_142	GO:0021642	0.00354
M_142	GO:0035852	0.00354
M_142	GO:0035847	0.00354
M_142	GO:0035849	0.00354
M_142	GO:0042493	0.00433
M_142	GO:0050919	0.00536
M_142	GO:0048666	0.00588
M_142	GO:0046486	0.00678
M_142	GO:0060067	0.00707
M_142	GO:0042450	0.00707
M_142	GO:0030860	0.00707
M_142	GO:0036304	0.00707

M_142	GO:0038129	0.00707
M_142	GO:0021784	0.00707
M_142	GO:0003195	0.00707
M_142	GO:0021842	0.00707
M_142	GO:0042704	0.00707
M_142	GO:2000744	0.00707
M_142	GO:0021623	0.00707
M_142	GO:0021644	0.00707
M_142	GO:0060715	0.00707
M_142	GO:0097272	0.00707
M_142	GO:0033306	0.00707
M_142	GO:0070781	0.00707
M_142	GO:0000165	0.0072
M_142	GO:0051930	0.00758
M_142	GO:0023052	0.00879
M_142	GO:0050772	0.009
M_142	GO:0003151	0.00985
M_142	GO:0010718	0.01014
M_142	GO:0003170	0.01026
M_142	GO:0021885	0.01044
M_142	GO:0051795	0.01058
M_142	GO:0010693	0.01058
M_142	GO:0032289	0.01058
M_142	GO:0010621	0.01058
M_142	GO:2000255	0.01058
M_142	GO:1902256	0.01058
M_142	GO:0033058	0.01058
M_142	GO:2000768	0.01058
M_142	GO:0003274	0.01058
M_142	GO:0060912	0.01058
M_142	GO:0060948	0.01058
M_142	GO:0045213	0.01058

M_142	GO:0097477	0.01058
M_142	GO:2000820	0.01058
M_142	GO:0010903	0.01058
M_142	GO:0045726	0.01058
M_142	GO:0060061	0.01058
M_142	GO:0042177	0.01289
M_142	GO:1901382	0.01408
M_142	GO:0072278	0.01408
M_142	GO:0061205	0.01408
M_142	GO:0014878	0.01408
M_142	GO:0032286	0.01408
M_142	GO:0003199	0.01408
M_142	GO:0015798	0.01408
M_142	GO:0050923	0.01408
M_142	GO:0021615	0.01408
M_142	GO:1903539	0.01408
M_142	GO:0046010	0.01408
M_142	GO:0001705	0.01408
M_142	GO:0030182	0.01687
M_142	GO:0021960	0.01757
M_142	GO:0021942	0.01757
M_142	GO:0060633	0.01757
M_142	GO:0051891	0.01757
M_142	GO:0014012	0.01757
M_142	GO:0003150	0.01757
M_142	GO:0003289	0.01757
M_142	GO:0021523	0.01757
M_142	GO:0010936	0.01757
M_142	GO:0021637	0.01757
M_142	GO:0042636	0.01757
M_142	GO:0060718	0.01757
M_142	GO:0060071	0.01973

M_142	GO:0046425	0.02021
M_142	GO:0015909	0.02034
M_142	GO:0060842	0.02105
M_142	GO:0061156	0.02105
M_142	GO:0009133	0.02105
M_142	GO:0021937	0.02105
M_142	GO:0072284	0.02105
M_142	GO:0035973	0.02105
M_142	GO:0060956	0.02105
M_142	GO:0033603	0.02105
M_142	GO:0002740	0.02105
M_142	GO:1904338	0.02105
M_142	GO:0035502	0.02105
M_142	GO:0007416	0.02174
M_142	GO:0014068	0.02222
M_142	GO:0019915	0.02222
M_142	GO:0007409	0.02296
M_142	GO:0098609	0.0233
M_142	GO:0035773	0.0242
M_142	GO:0032489	0.02452
M_142	GO:0003357	0.02452
M_142	GO:0021796	0.02452
M_142	GO:0031077	0.02452
M_142	GO:0030300	0.02452
M_142	GO:0021612	0.02452
M_142	GO:0007422	0.02744
M_142	GO:0048841	0.02756
M_142	GO:0043507	0.02757
M_142	GO:0043497	0.02797
M_142	GO:0060059	0.02797
M_142	GO:0001561	0.02797
M_142	GO:0014732	0.02797

M_142	GO:0045607	0.02797
M_142	GO:0032909	0.02797
M_142	GO:0006548	0.02797
M_142	GO:0051823	0.02797
M_142	GO:2000252	0.02797
M_142	GO:0035912	0.02797
M_142	GO:0048793	0.02797
M_142	GO:0061626	0.02797
M_142	GO:0034371	0.02797
M_142	GO:0097264	0.02797
M_142	GO:0044332	0.02797
M_142	GO:0010898	0.02797
M_142	GO:0007626	0.02937
M_142	GO:0003279	0.03056
M_142	GO:0010873	0.03141
M_142	GO:0072177	0.03141
M_142	GO:0034115	0.03141
M_142	GO:0031915	0.03141
M_142	GO:0070508	0.03141
M_142	GO:0051006	0.03141
M_142	GO:0021702	0.03141
M_142	GO:0030643	0.03141
M_142	GO:0055091	0.03141
M_142	GO:0035860	0.03141
M_142	GO:0002091	0.03141
M_142	GO:0044341	0.03141
M_142	GO:0090394	0.03141
M_142	GO:0009953	0.03174
M_142	GO:0060068	0.03484
M_142	GO:0042428	0.03484
M_142	GO:0043951	0.03484
M_142	GO:0003149	0.03484

M_142	GO:0032525	0.03484
M_142	GO:0019240	0.03484
M_142	GO:0034378	0.03484
M_142	GO:0061314	0.03484
M_142	GO:0007193	0.03646
M_142	GO:0060413	0.03766
M_142	GO:0003181	0.03767
M_142	GO:0045823	0.0377
M_142	GO:0030431	0.0377
M_142	GO:0060379	0.03826
M_142	GO:0000050	0.03826
M_142	GO:0048703	0.03826
M_142	GO:0090009	0.03826
M_142	GO:0050713	0.03826
M_142	GO:2001223	0.03826
M_142	GO:0003300	0.04048
M_142	GO:0046834	0.0406
M_142	GO:0060561	0.04103
M_142	GO:0021871	0.04103
M_142	GO:1904469	0.04166
M_142	GO:0018158	0.04166
M_142	GO:0042416	0.04166
M_142	GO:0006020	0.04166
M_142	GO:0060670	0.04166
M_142	GO:0018206	0.04166
M_142	GO:0033700	0.04166
M_142	GO:1902287	0.04166
M_142	GO:0003161	0.04166
M_142	GO:0007494	0.04166
M_142	GO:0034384	0.04166
M_142	GO:0021517	0.04443
M_142	GO:0021781	0.04506

M_142	GO:0055015	0.04506
M_142	GO:0060134	0.04506
M_142	GO:0006995	0.04506
M_142	GO:0007171	0.04506
M_142	GO:0031589	0.04617
M_142	GO:0072001	0.04762
M_142	GO:0097553	0.04844
M_142	GO:0090280	0.04844
M_142	GO:0021527	0.04844
M_142	GO:0060263	0.04844
M_142	GO:2001212	0.04844
M_142	GO:0009790	0.04861
M_143	GO:0070125	0.000014
M_143	GO:0070126	0.000015
M_143	GO:0006195	0.0017
M_143	GO:1902905	0.0035
M_143	GO:0001998	0.0035
M_143	GO:0071544	0.0035
M_143	GO:1902616	0.0035
M_143	GO:1903673	0.0035
M_143	GO:1903614	0.0035
M_143	GO:0044319	0.0049
M_143	GO:1902766	0.007
M_143	GO:0009609	0.007
M_143	GO:0009159	0.007
M_143	GO:1902559	0.007
M_143	GO:0097498	0.007
M_143	GO:0052651	0.007
M_143	GO:0005999	0.007
M_143	GO:0046166	0.007
M_143	GO:0002352	0.007
M_143	GO:0050919	0.0077

M_143	GO:0006749	0.0091
M_143	GO:0042278	0.0099
M_143	GO:1990166	0.0104
M_143	GO:0048597	0.0104
M_143	GO:0043366	0.0104
M_143	GO:0006420	0.0104
M_143	GO:0051702	0.0137
M_143	GO:0016240	0.0139
M_143	GO:0009438	0.0139
M_143	GO:0051106	0.0139
M_143	GO:0007023	0.0139
M_143	GO:0010046	0.0139
M_143	GO:1901911	0.0139
M_143	GO:0060355	0.0139
M_143	GO:1903595	0.0139
M_143	GO:1901907	0.0139
M_143	GO:1901909	0.0139
M_143	GO:0061086	0.0139
M_143	GO:0010332	0.0163
M_143	GO:0070527	0.0163
M_143	GO:0044854	0.0173
M_143	GO:0033227	0.0173
M_143	GO:0061302	0.0173
M_143	GO:0038027	0.0173
M_143	GO:1902262	0.0173
M_143	GO:0002224	0.0175
M_143	GO:0009410	0.0202
M_143	GO:0090324	0.0207
M_143	GO:0050428	0.0207
M_143	GO:0009052	0.0207
M_143	GO:0003100	0.0207
M_143	GO:0061087	0.0207

M_143	GO:0051881	0.0228
M_143	GO:0032226	0.0241
M_143	GO:2000049	0.0241
M_143	GO:0008635	0.0241
M_143	GO:1903896	0.0241
M_143	GO:0044351	0.0241
M_143	GO:0007021	0.0241
M_143	GO:0034141	0.0241
M_143	GO:0033864	0.0241
M_143	GO:0021861	0.0241
M_143	GO:0035385	0.0241
M_143	GO:0006987	0.0275
M_143	GO:0043497	0.0275
M_143	GO:0006853	0.0275
M_143	GO:0018401	0.0275
M_143	GO:0032471	0.0275
M_143	GO:0043534	0.0306
M_143	GO:1903044	0.0309
M_143	GO:0045955	0.0309
M_143	GO:0051924	0.0341
M_143	GO:0060068	0.0343
M_143	GO:0038063	0.0343
M_143	GO:0001574	0.0343
M_143	GO:0045792	0.0343
M_143	GO:0006415	0.0348
M_143	GO:0006273	0.0377
M_143	GO:0070254	0.0377
M_143	GO:0071803	0.0377
M_143	GO:0010269	0.0377
M_143	GO:0006527	0.0377
M_143	GO:0002862	0.0377
M_143	GO:0048010	0.0385

M_143	GO:0051726	0.039
M_143	GO:0030950	0.041
M_143	GO:0051103	0.041
M_143	GO:0018158	0.041
M_143	GO:0009650	0.0443
M_143	GO:0006995	0.0443
M_143	GO:0001973	0.0443
M_143	GO:0031665	0.0443
M_143	GO:0006703	0.0443
M_143	GO:0045198	0.0443
M_143	GO:0010248	0.0443
M_143	GO:0000052	0.0443
M_143	GO:0034116	0.0443
M_143	GO:0019372	0.0477
M_143	GO:0010225	0.0477
M_143	GO:0035810	0.0477
M_144	GO:0070830	0.00000064
M_144	GO:0060672	0.000031
M_144	GO:0016338	0.000055
M_144	GO:0001843	0.00018
M_144	GO:0070268	0.00048
M_144	GO:1900029	0.00092
M_144	GO:2000647	0.00137
M_144	GO:0061436	0.0019
M_144	GO:0050718	0.00321
M_144	GO:0060887	0.00325
M_144	GO:1902939	0.00325
M_144	GO:0050900	0.00361
M_144	GO:0001657	0.00501
M_144	GO:0002590	0.0065
M_144	GO:0005993	0.0065
M_144	GO:0009644	0.0065

M_144	GO:2001287	0.0065
M_144	GO:0055107	0.0065
M_144	GO:0007162	0.00699
M_144	GO:0043087	0.00779
M_144	GO:0090132	0.00936
M_144	GO:0010482	0.00973
M_144	GO:0051496	0.01024
M_144	GO:0001755	0.01243
M_144	GO:1903553	0.01295
M_144	GO:0002159	0.01295
M_144	GO:0015917	0.01295
M_144	GO:0048550	0.01295
M_144	GO:0060214	0.01295
M_144	GO:0061589	0.01295
M_144	GO:0090191	0.01295
M_144	GO:0042060	0.01418
M_144	GO:2000048	0.01616
M_144	GO:0061590	0.01616
M_144	GO:0061591	0.01616
M_144	GO:1900028	0.01616
M_144	GO:0098609	0.01754
M_144	GO:0051271	0.0183
M_144	GO:0010633	0.01898
M_144	GO:0010792	0.01937
M_144	GO:0018242	0.01937
M_144	GO:0018243	0.02256
M_144	GO:0060463	0.02256
M_144	GO:0001842	0.02256
M_144	GO:0030913	0.02256
M_144	GO:0045617	0.02256
M_144	GO:0045606	0.02531
M_144	GO:1902373	0.02574

M_144	GO:0071578	0.02574
M_144	GO:0009414	0.02574
M_144	GO:0060670	0.02791
M_144	GO:0007165	0.0286
M_144	GO:0001766	0.02891
M_144	GO:0070255	0.02891
M_144	GO:1902414	0.02891
M_144	GO:0090557	0.02891
M_144	GO:0043616	0.03101
M_144	GO:1902476	0.03262
M_144	GO:0060674	0.03465
M_144	GO:1900016	0.03522
M_144	GO:0050713	0.03522
M_144	GO:0003334	0.03522
M_144	GO:0032463	0.03522
M_144	GO:0090331	0.03522
M_144	GO:0002862	0.03522
M_144	GO:1990830	0.03541
M_144	GO:0006068	0.03836
M_144	GO:0070374	0.03892
M_144	GO:0010839	0.04149
M_144	GO:0070863	0.04149
M_144	GO:1900025	0.04149
M_144	GO:0009607	0.04701
M_144	GO:0008543	0.04746
M_144	GO:0060347	0.04773
M_144	GO:1903543	0.04773
M_144	GO:0045618	0.04773
M_144	GO:0072659	0.04877
M_144	GO:2000147	0.04943
M_144	GO:0010837	0.04999
M_145	GO:0061053	0.00000086

M_145	GO:0090090	0.0000042
M_145	GO:0030282	0.000017
M_145	GO:0021722	0.000097
M_145	GO:2001037	0.000097
M_145	GO:0035567	0.0001
M_145	GO:0050885	0.00012
M_145	GO:0009954	0.00023
M_145	GO:0021819	0.00025
M_145	GO:0046710	0.00032
M_145	GO:0042733	0.00042
M_145	GO:0000226	0.00053
M_145	GO:0001764	0.00075
M_145	GO:0007386	0.00095
M_145	GO:0046037	0.0011
M_145	GO:0060070	0.00118
M_145	GO:0090103	0.00164
M_145	GO:0061037	0.00186
M_145	GO:0017185	0.00261
M_145	GO:0090037	0.00261
M_145	GO:0045879	0.00286
M_145	GO:0043587	0.00333
M_145	GO:0048570	0.00333
M_145	GO:0002315	0.00333
M_145	GO:0042475	0.00342
M_145	GO:0045880	0.0038
M_145	GO:0030259	0.00414
M_145	GO:0003149	0.00414
M_145	GO:0001574	0.00414
M_145	GO:0060346	0.00414
M_145	GO:0009887	0.00449
M_145	GO:0009653	0.00473
M_145	GO:0017015	0.00489

M_145	GO:0007223	0.00491
M_145	GO:0003323	0.00498
M_145	GO:0021510	0.00587
M_145	GO:0030516	0.00679
M_145	GO:0070986	0.00704
M_145	GO:0036109	0.00704
M_145	GO:0021549	0.00748
M_145	GO:0048384	0.00808
M_145	GO:0048853	0.00816
M_145	GO:0019511	0.00936
M_145	GO:0055002	0.00967
M_145	GO:0003151	0.00979
M_145	GO:0071678	0.00987
M_145	GO:1900243	0.00987
M_145	GO:0060248	0.00987
M_145	GO:0018166	0.00987
M_145	GO:0072285	0.00987
M_145	GO:0060853	0.00987
M_145	GO:0060857	0.00987
M_145	GO:0060873	0.00987
M_145	GO:0060875	0.00987
M_145	GO:0021688	0.00987
M_145	GO:0030844	0.00987
M_145	GO:0000412	0.00987
M_145	GO:0043390	0.00987
M_145	GO:2000057	0.00987
M_145	GO:0070367	0.00987
M_145	GO:0060366	0.00987
M_145	GO:0060367	0.00987
M_145	GO:0007371	0.00987
M_145	GO:1903141	0.00987
M_145	GO:0022018	0.00987

M_145	GO:0030517	0.01186
M_145	GO:0043651	0.01196
M_145	GO:0045664	0.01437
M_145	GO:0001755	0.01468
M_145	GO:0014002	0.0147
M_145	GO:0090190	0.01485
M_145	GO:0007189	0.0151
M_145	GO:0055008	0.01594
M_145	GO:0042474	0.01639
M_145	GO:0002052	0.01639
M_145	GO:0030198	0.01766
M_145	GO:0098773	0.01941
M_145	GO:0061162	0.01956
M_145	GO:1902367	0.01965
M_145	GO:1901074	0.01965
M_145	GO:0032915	0.01965
M_145	GO:0060775	0.01965
M_145	GO:0021693	0.01965
M_145	GO:0097535	0.01965
M_145	GO:0021644	0.01965
M_145	GO:1901953	0.01965
M_145	GO:0048627	0.01965
M_145	GO:0072560	0.01965
M_145	GO:0046331	0.01965
M_145	GO:0043314	0.01965
M_145	GO:2001303	0.01965
M_145	GO:1902613	0.01965
M_145	GO:0060302	0.01965
M_145	GO:0097102	0.01965
M_145	GO:1901874	0.01965
M_145	GO:2000329	0.01965
M_145	GO:2000313	0.01965

M_145	GO:0001947	0.02054
M_145	GO:0035249	0.02134
M_145	GO:0008045	0.02141
M_145	GO:0030325	0.02141
M_145	GO:0016338	0.02141
M_145	GO:0045595	0.02299
M_145	GO:0021766	0.0231
M_145	GO:0022011	0.02321
M_145	GO:0050771	0.02438
M_145	GO:0031424	0.02462
M_145	GO:0031116	0.02506
M_145	GO:0051965	0.02541
M_145	GO:0000278	0.02655
M_145	GO:0031069	0.02698
M_145	GO:0002820	0.02923
M_145	GO:0021773	0.02934
M_145	GO:0021775	0.02934
M_145	GO:0021776	0.02934
M_145	GO:0035963	0.02934
M_145	GO:0007228	0.02934
M_145	GO:0072138	0.02934
M_145	GO:0043585	0.02934
M_145	GO:1904395	0.02934
M_145	GO:0033563	0.02934
M_145	GO:0060594	0.02934
M_145	GO:0090176	0.02934
M_145	GO:0030538	0.02934
M_145	GO:0090271	0.02934
M_145	GO:0090272	0.02934
M_145	GO:0021912	0.02934
M_145	GO:0035583	0.02934
M_145	GO:0035544	0.02934

M_145	GO:2000078	0.02934
M_145	GO:0045608	0.02934
M_145	GO:0060313	0.02934
M_145	GO:0071874	0.02934
M_145	GO:0060197	0.02934
M_145	GO:0030509	0.02943
M_145	GO:0018107	0.02958
M_145	GO:0031175	0.02998
M_145	GO:0007269	0.03067
M_145	GO:0071300	0.03084
M_145	GO:0002053	0.03098
M_145	GO:0007420	0.0368
M_145	GO:0060325	0.03739
M_145	GO:0060675	0.03817
M_145	GO:0046639	0.03874
M_145	GO:0034653	0.03892
M_145	GO:1990743	0.03892
M_145	GO:0051594	0.03892
M_145	GO:0032971	0.03892
M_145	GO:0032348	0.03892
M_145	GO:0072180	0.03892
M_145	GO:0048769	0.03892
M_145	GO:1903553	0.03892
M_145	GO:0060024	0.03892
M_145	GO:0048633	0.03892
M_145	GO:0030035	0.03892
M_145	GO:0021913	0.03892
M_145	GO:0021938	0.03892
M_145	GO:2000065	0.03892
M_145	GO:0061113	0.03892
M_145	GO:2000672	0.03892
M_145	GO:0060364	0.03892

M_145	GO:0002175	0.03892
M_145	GO:0097113	0.03892
M_145	GO:0051799	0.03892
M_145	GO:1900186	0.03892
M_145	GO:0048747	0.03934
M_145	GO:0030513	0.03962
M_145	GO:0048699	0.04157
M_145	GO:0043507	0.04165
M_145	GO:0040018	0.04191
M_145	GO:0035023	0.04196
M_145	GO:0042060	0.0439
M_145	GO:0048048	0.04425
M_145	GO:0045807	0.04443
M_145	GO:0061077	0.04475
M_145	GO:0060840	0.04779
M_145	GO:0021910	0.04815
M_145	GO:0072177	0.04815
M_145	GO:0044091	0.04822
M_145	GO:0048846	0.04823
M_145	GO:1902412	0.04841
M_145	GO:0014052	0.04841
M_145	GO:0001757	0.04841
M_145	GO:0003150	0.04841
M_145	GO:0060023	0.04841
M_145	GO:1900020	0.04841
M_145	GO:2000821	0.04841
M_145	GO:0090235	0.04841
M_145	GO:0072014	0.04841
M_145	GO:0071205	0.04841
M_145	GO:0051122	0.04841
M_145	GO:0098700	0.04841
M_145	GO:0018057	0.04841

M_145	GO:0033153	0.04841
M_145	GO:0032091	0.04983
M_146	GO:0070268	5.1E-11
M_146	GO:0070488	0.000019
M_146	GO:0034334	0.000056
M_146	GO:0036101	0.00011
M_146	GO:0050832	0.00028
M_146	GO:0045110	0.00038
M_146	GO:0030593	0.00094
M_146	GO:0002523	0.001
M_146	GO:0032119	0.00119
M_146	GO:0007010	0.0016
M_146	GO:0051092	0.00313
M_146	GO:0002544	0.0037
M_146	GO:0007605	0.00373
M_146	GO:0045103	0.00412
M_146	GO:0016332	0.00434
M_146	GO:0003026	0.00434
M_146	GO:0070247	0.00434
M_146	GO:0034230	0.00434
M_146	GO:0034231	0.00434
M_146	GO:0072060	0.00434
M_146	GO:0019730	0.0044
M_146	GO:0043312	0.00487
M_146	GO:0050829	0.00562
M_146	GO:0014002	0.00653
M_146	GO:0072053	0.00866
M_146	GO:0072054	0.00866
M_146	GO:0035606	0.00866
M_146	GO:2000521	0.00866
M_146	GO:0038195	0.00866
M_146	GO:0042361	0.00866

M_146	GO:0042377	0.00866
M_146	GO:0003091	0.01007
M_146	GO:0042742	0.0103
M_146	GO:0002548	0.01154
M_146	GO:2001189	0.01296
M_146	GO:0045218	0.01296
M_146	GO:0003365	0.01296
M_146	GO:0042369	0.01296
M_146	GO:0042376	0.01296
M_146	GO:0003095	0.01296
M_146	GO:0071677	0.01296
M_146	GO:0072061	0.01296
M_146	GO:0007257	0.01302
M_146	GO:0050729	0.01533
M_146	GO:0070778	0.01724
M_146	GO:1901842	0.01724
M_146	GO:0042360	0.01724
M_146	GO:0022009	0.01724
M_146	GO:0060535	0.01724
M_146	GO:0002159	0.01724
M_146	GO:0030070	0.01724
M_146	GO:0072236	0.02151
M_146	GO:0098712	0.02151
M_146	GO:0050915	0.02151
M_146	GO:0019732	0.02151
M_146	GO:0045113	0.02151
M_146	GO:0048496	0.02151
M_146	GO:0006914	0.02259
M_146	GO:0050896	0.02311
M_146	GO:2001244	0.02459
M_146	GO:0002934	0.02542
M_146	GO:0035634	0.02575

M_146	GO:0060729	0.02575
M_146	GO:0060710	0.02575
M_146	GO:0086073	0.02575
M_146	GO:0060482	0.02575
M_146	GO:0032534	0.02575
M_146	GO:0045600	0.02625
M_146	GO:0050957	0.02998
M_146	GO:0032364	0.02998
M_146	GO:0072205	0.03419
M_146	GO:1902373	0.03419
M_146	GO:0035331	0.03419
M_146	GO:0007219	0.03496
M_146	GO:0071300	0.03614
M_146	GO:0043587	0.03838
M_146	GO:0070561	0.03838
M_146	GO:0043123	0.03882
M_146	GO:0043117	0.04256
M_146	GO:0032602	0.04513
M_146	GO:0018119	0.04611
M_146	GO:0006693	0.04617
M_146	GO:0043542	0.04662
M_146	GO:0019371	0.04671
M_146	GO:0098911	0.04671
M_146	GO:0060736	0.04671
M_146	GO:0070431	0.04671
M_146	GO:0060576	0.04671
M_146	GO:0010960	0.04671
M_146	GO:0032494	0.04671
M_146	GO:0010820	0.04671
M_147	GO:0010951	0.00007
M_147	GO:0043312	0.00016
M_147	GO:0051601	0.00037

M_147	GO:0045408	0.00106
M_147	GO:0032755	0.00217
M_147	GO:0009615	0.0023
M_147	GO:0044795	0.00325
M_147	GO:0002416	0.00325
M_147	GO:0014895	0.00325
M_147	GO:0003106	0.00325
M_147	GO:1902463	0.0065
M_147	GO:0019747	0.0065
M_147	GO:1900042	0.00973
M_147	GO:0015864	0.00973
M_147	GO:1901329	0.00973
M_147	GO:0036510	0.00973
M_147	GO:0014028	0.00973
M_147	GO:1904154	0.00973
M_147	GO:0010266	0.00973
M_147	GO:0045747	0.01024
M_147	GO:0042987	0.01275
M_147	GO:0030951	0.01295
M_147	GO:0033490	0.01295
M_147	GO:0033489	0.01295
M_147	GO:0015855	0.01295
M_147	GO:1904382	0.01295
M_147	GO:0090383	0.01295
M_147	GO:0046836	0.01295
M_147	GO:0003199	0.01295
M_147	GO:0018076	0.01295
M_147	GO:0070498	0.01383
M_147	GO:0042130	0.0148
M_147	GO:0006954	0.01514
M_147	GO:0060337	0.01582
M_147	GO:0019640	0.01616

M_147	GO:0032747	0.01616
M_147	GO:0097368	0.01616
M_147	GO:0014012	0.01616
M_147	GO:0034137	0.01616
M_147	GO:1904044	0.01616
M_147	GO:0072656	0.01616
M_147	GO:0036498	0.01683
M_147	GO:0002223	0.01843
M_147	GO:0043123	0.01847
M_147	GO:0050703	0.01937
M_147	GO:0072602	0.01937
M_147	GO:1903243	0.01937
M_147	GO:0070257	0.01937
M_147	GO:0006880	0.01937
M_147	GO:0035973	0.01937
M_147	GO:0006824	0.01937
M_147	GO:1902961	0.01937
M_147	GO:0061002	0.01937
M_147	GO:0032760	0.01953
M_147	GO:0071347	0.02219
M_147	GO:0015860	0.02256
M_147	GO:1903147	0.02256
M_147	GO:0006032	0.02256
M_147	GO:0035948	0.02256
M_147	GO:0010940	0.02256
M_147	GO:0007084	0.02256
M_147	GO:0003180	0.02256
M_147	GO:0033864	0.02256
M_147	GO:0090240	0.02256
M_147	GO:0071476	0.02256
M_147	GO:0032482	0.0248
M_147	GO:0015889	0.02574

M_147	GO:0090385	0.02574
M_147	GO:0070244	0.02574
M_147	GO:0072201	0.02574
M_147	GO:0046600	0.02574
M_147	GO:0071260	0.02667
M_147	GO:0007165	0.02708
M_147	GO:0060242	0.02891
M_147	GO:0060056	0.02891
M_147	GO:0061635	0.02891
M_147	GO:0010944	0.02891
M_147	GO:0044341	0.02891
M_147	GO:0051901	0.02891
M_147	GO:1900426	0.02891
M_147	GO:0030643	0.02891
M_147	GO:0032464	0.02891
M_147	GO:0010835	0.02891
M_147	GO:0035455	0.03154
M_147	GO:0050830	0.03193
M_147	GO:0035457	0.03207
M_147	GO:1902004	0.03207
M_147	GO:1901387	0.03207
M_147	GO:0001765	0.03207
M_147	GO:0003183	0.03207
M_147	GO:0033629	0.03207
M_147	GO:1904385	0.03522
M_147	GO:0090343	0.03522
M_147	GO:0032494	0.03522
M_147	GO:1990440	0.03522
M_147	GO:0060252	0.03836
M_147	GO:0048266	0.03836
M_147	GO:0045019	0.03836
M_147	GO:0060253	0.04149

M_147	GO:0032740	0.04149
M_147	GO:0055015	0.04149
M_147	GO:0050665	0.04149
M_147	GO:0071688	0.04149
M_147	GO:0043970	0.04149
M_147	GO:1902166	0.04149
M_147	GO:0044130	0.04149
M_147	GO:0098780	0.04462
M_147	GO:0007210	0.04462
M_147	GO:0060547	0.04773
M_147	GO:0097286	0.04773
M_147	GO:0051607	0.04877
M_148	GO:1901185	0.000074
M_148	GO:0033617	0.0021
M_148	GO:0036372	0.0036
M_148	GO:1902239	0.0036
M_148	GO:0000188	0.0039
M_148	GO:0038128	0.0055
M_148	GO:0051603	0.0066
M_148	GO:0000453	0.0072
M_148	GO:0042823	0.0072
M_148	GO:2001287	0.0072
M_148	GO:0042418	0.0072
M_148	GO:0017055	0.0072
M_148	GO:0008050	0.0072
M_148	GO:0045925	0.0072
M_148	GO:0008615	0.0072
M_148	GO:0006624	0.0072
M_148	GO:0043312	0.0076
M_148	GO:0090156	0.0108
M_148	GO:1900186	0.0143
M_148	GO:0033088	0.0143

M_148	GO:0002155	0.0143
M_148	GO:0048769	0.0143
M_148	GO:0043328	0.0143
M_148	GO:0035995	0.0143
M_148	GO:1900060	0.0143
M_148	GO:0045931	0.0154
M_148	GO:0008535	0.0173
M_148	GO:0033132	0.0179
M_148	GO:0060509	0.0179
M_148	GO:0008616	0.0179
M_148	GO:0015760	0.0214
M_148	GO:0043985	0.0214
M_148	GO:0006701	0.0249
M_148	GO:1901098	0.0249
M_148	GO:0000281	0.0263
M_148	GO:0097264	0.0284
M_148	GO:0048227	0.0284
M_148	GO:0007042	0.0284
M_148	GO:0030916	0.0284
M_148	GO:0046548	0.0319
M_148	GO:0071277	0.0321
M_148	GO:0006886	0.0352
M_148	GO:0070072	0.0354
M_148	GO:1901838	0.0354
M_148	GO:0098792	0.0354
M_148	GO:0035845	0.0354
M_148	GO:0070131	0.0389
M_148	GO:0030241	0.0389
M_148	GO:0045945	0.0389
M_148	GO:0040015	0.0389
M_148	GO:0010269	0.0389
M_148	GO:0070125	0.04

M_148	GO:0070126	0.0416
M_148	GO:0045943	0.0417
M_148	GO:0045475	0.0423
M_148	GO:0016558	0.0423
M_148	GO:0060119	0.0451
M_148	GO:0071712	0.0458
M_148	GO:0006703	0.0458
M_148	GO:0000212	0.0458
M_148	GO:0016311	0.0476
M_148	GO:0030490	0.0486
M_148	GO:0007175	0.0492
M_148	GO:0042953	0.0492
M_148	GO:0030240	0.0492
M_148	GO:0036295	0.0492
M_148	GO:2001028	0.0492
M_148	GO:0035335	0.0494
M_149	GO:0050911	1E-30
M_149	GO:0007186	1.8E-26
M_149	GO:0031424	0.00024
M_149	GO:0033141	0.00049
M_149	GO:0002323	0.00166
M_149	GO:0035881	0.00167
M_149	GO:0050829	0.00419
M_149	GO:0055015	0.00454
M_149	GO:0016998	0.00527
M_149	GO:0043330	0.00533
M_149	GO:0021542	0.00688
M_149	GO:0061452	0.00788
M_149	GO:0046534	0.00788
M_149	GO:0021723	0.00788
M_149	GO:0048894	0.00788
M_149	GO:0045818	0.00788

M_149	GO:0007275	0.01458
M_149	GO:0030183	0.01499
M_149	GO:0050883	0.01569
M_149	GO:0010430	0.01569
M_149	GO:0060730	0.01569
M_149	GO:0003326	0.01569
M_149	GO:0003329	0.01569
M_149	GO:0032812	0.01569
M_149	GO:0032416	0.01569
M_149	GO:0060133	0.01569
M_149	GO:0007286	0.02111
M_149	GO:1990167	0.02345
M_149	GO:0060266	0.02345
M_149	GO:0035483	0.02345
M_149	GO:0003358	0.02345
M_149	GO:0055014	0.02345
M_149	GO:0098583	0.02345
M_149	GO:0021934	0.02345
M_149	GO:0010750	0.02345
M_149	GO:0033861	0.02345
M_149	GO:0071626	0.02345
M_149	GO:0060160	0.02345
M_149	GO:0019222	0.02876
M_149	GO:0050830	0.03047
M_149	GO:0048634	0.03113
M_149	GO:1990168	0.03114
M_149	GO:0060298	0.03114
M_149	GO:0070253	0.03114
M_149	GO:1901166	0.03114
M_149	GO:0070093	0.03114
M_149	GO:0060161	0.03114
M_149	GO:1902952	0.03114

M_149	GO:0060337	0.03138
M_149	GO:0042100	0.03517
M_149	GO:0007356	0.03877
M_149	GO:0021650	0.03877
M_149	GO:2000138	0.03877
M_149	GO:0015739	0.03877
M_149	GO:0032962	0.03877
M_149	GO:0031536	0.03877
M_149	GO:0090325	0.03877
M_149	GO:0019732	0.03877
M_149	GO:1901078	0.03877
M_149	GO:0035523	0.03877
M_149	GO:1900226	0.03877
M_149	GO:0051169	0.03936
M_149	GO:0002286	0.04233
M_149	GO:0070172	0.04635
M_149	GO:0051971	0.04635
M_149	GO:1990535	0.04635
M_149	GO:2000675	0.04635
M_149	GO:0022898	0.04658
M_150	GO:0018917	0.0024
M_150	GO:0003147	0.0024
M_150	GO:0072302	0.0024
M_150	GO:0050894	0.0024
M_150	GO:0061713	0.0024
M_150	GO:0045401	0.0024
M_150	GO:2000824	0.0048
M_150	GO:2001076	0.0048
M_150	GO:0097089	0.0048
M_150	GO:0071226	0.0048
M_150	GO:0071231	0.0048
M_150	GO:0002384	0.0048

M_150	GO:0061032	0.0048
M_150	GO:0045425	0.0048
M_150	GO:0035802	0.0048
M_150	GO:0072166	0.0048
M_150	GO:0035054	0.0072
M_150	GO:0021564	0.0072
M_150	GO:0043366	0.0072
M_150	GO:0002554	0.0072
M_150	GO:0045588	0.0072
M_150	GO:0060523	0.0072
M_150	GO:0001947	0.0088
M_150	GO:0015884	0.0093
M_150	GO:1904447	0.0096
M_150	GO:0046959	0.0096
M_150	GO:0060750	0.0096
M_150	GO:0001951	0.0096
M_150	GO:0018879	0.0096
M_150	GO:0003253	0.0096
M_150	GO:0002175	0.0096
M_150	GO:0035610	0.0119
M_150	GO:0007356	0.0119
M_150	GO:0021563	0.0119
M_150	GO:0006682	0.0119
M_150	GO:1900086	0.0119
M_150	GO:1902617	0.0119
M_150	GO:0098814	0.0119
M_150	GO:2000195	0.0119
M_150	GO:0098535	0.0119
M_150	GO:0070120	0.0119
M_150	GO:0003025	0.0119
M_150	GO:0072284	0.0143
M_150	GO:0060745	0.0143

M_150	GO:0060907	0.0143
M_150	GO:0090237	0.0143
M_150	GO:0060527	0.0143
M_150	GO:0051135	0.0143
M_150	GO:0001547	0.0143
M_150	GO:0060013	0.0167
M_150	GO:0042760	0.0167
M_150	GO:0010536	0.0167
M_150	GO:0097107	0.0167
M_150	GO:0030913	0.0167
M_150	GO:0043313	0.0167
M_150	GO:1900164	0.0167
M_150	GO:0060687	0.0167
M_150	GO:0048318	0.0167
M_150	GO:0015889	0.019
M_150	GO:0061626	0.019
M_150	GO:0060923	0.019
M_150	GO:0001561	0.019
M_150	GO:1903799	0.019
M_150	GO:2000020	0.019
M_150	GO:0042048	0.019
M_150	GO:0003139	0.0214
M_150	GO:0060339	0.0214
M_150	GO:0060539	0.0214
M_150	GO:0050896	0.0221
M_150	GO:0060068	0.0237
M_150	GO:0044539	0.0237
M_150	GO:1903025	0.0237
M_150	GO:0007016	0.0237
M_150	GO:0038063	0.0237
M_150	GO:0051124	0.0237
M_150	GO:0032836	0.0237

M_150	GO:0019371	0.0261
M_150	GO:0021554	0.0261
M_150	GO:0045899	0.0261
M_150	GO:0002281	0.0261
M_150	GO:0043922	0.0284
M_150	GO:2001256	0.0284
M_150	GO:0034384	0.0284
M_150	GO:0038110	0.0284
M_150	GO:0043306	0.0307
M_150	GO:0045579	0.0307
M_150	GO:0019372	0.0331
M_150	GO:0001921	0.0331
M_150	GO:0033147	0.0331
M_150	GO:0072075	0.0354
M_150	GO:0010875	0.0354
M_150	GO:0065003	0.0357
M_150	GO:0003222	0.0377
M_150	GO:0051090	0.0389
M_150	GO:0007342	0.04
M_150	GO:0045780	0.04
M_150	GO:0002827	0.04
M_150	GO:0032816	0.04
M_150	GO:0070102	0.04
M_150	GO:0033630	0.04
M_150	GO:0090330	0.04
M_150	GO:0071404	0.0423
M_150	GO:0060749	0.0423
M_150	GO:0046641	0.0423
M_150	GO:0071371	0.0423
M_150	GO:0072112	0.0423
M_150	GO:0071625	0.0423
M_150	GO:0060074	0.0446

M_150	GO:0048268	0.0446
M_150	GO:0003215	0.0446
M_150	GO:0060065	0.0469
M_150	GO:0019370	0.0469
M_150	GO:0060231	0.0469
M_150	GO:0046697	0.0469
M_150	GO:0032928	0.0469
M_150	GO:0046655	0.0492
M_150	GO:0009235	0.0492
M_150	GO:1904659	0.0492
M_151	GO:0051965	8.8E-09
M_151	GO:0007156	0.0000024
M_151	GO:0034765	0.000062
M_151	GO:0035176	0.00028
M_151	GO:0051932	0.00048
M_151	GO:0071625	0.00084
M_151	GO:0060078	0.001
M_151	GO:0035235	0.00137
M_151	GO:0007214	0.00163
M_151	GO:0007218	0.00203
M_151	GO:0048691	0.0024
M_151	GO:1904635	0.0024
M_151	GO:1904639	0.0024
M_151	GO:0048174	0.0024
M_151	GO:0071250	0.0024
M_151	GO:0045813	0.0024
M_151	GO:0019056	0.0024
M_151	GO:2000184	0.0024
M_151	GO:1901296	0.0024
M_151	GO:0050772	0.00415
M_151	GO:0021842	0.00479
M_151	GO:0021836	0.00479

M_151	GO:0045720	0.00479
M_151	GO:1901860	0.00479
M_151	GO:0090082	0.00479
M_151	GO:1901558	0.00479
M_151	GO:0050925	0.00479
M_151	GO:0007507	0.0054
M_151	GO:0071805	0.0058
M_151	GO:0048661	0.00716
M_151	GO:1904640	0.00718
M_151	GO:0097116	0.00718
M_151	GO:0090258	0.00718
M_151	GO:0060596	0.00718
M_151	GO:0060112	0.00718
M_151	GO:0002009	0.00785
M_151	GO:0050877	0.00792
M_151	GO:0034220	0.00953
M_151	GO:1904637	0.00956
M_151	GO:0097118	0.00956
M_151	GO:1901857	0.00956
M_151	GO:0090244	0.00956
M_151	GO:0071873	0.00956
M_151	GO:0042662	0.00956
M_151	GO:0042663	0.00956
M_151	GO:0014878	0.00956
M_151	GO:0033600	0.00956
M_151	GO:0060079	0.01172
M_151	GO:0007613	0.0118
M_151	GO:0071300	0.01189
M_151	GO:2000821	0.01193
M_151	GO:0002865	0.01193
M_151	GO:0032229	0.01193
M_151	GO:0098914	0.01193

M_151	GO:0016199	0.01193
M_151	GO:1900748	0.01193
M_151	GO:0070100	0.01193
M_151	GO:0071332	0.01193
M_151	GO:0003151	0.01222
M_151	GO:0007409	0.01349
M_151	GO:0051964	0.0143
M_151	GO:0051552	0.0143
M_151	GO:1904338	0.0143
M_151	GO:1902949	0.0143
M_151	GO:0031339	0.0143
M_151	GO:0007267	0.01531
M_151	GO:0048013	0.01575
M_151	GO:0090647	0.01666
M_151	GO:0014050	0.01666
M_151	GO:0035385	0.01666
M_151	GO:0042297	0.01666
M_151	GO:0070374	0.0176
M_151	GO:1902476	0.01846
M_151	GO:0098915	0.01902
M_151	GO:0014732	0.01902
M_151	GO:2000096	0.01902
M_151	GO:0071313	0.01902
M_151	GO:0071420	0.01902
M_151	GO:2001171	0.02138
M_151	GO:0048671	0.02138
M_151	GO:0097119	0.02138
M_151	GO:0045955	0.02138
M_151	GO:0007614	0.02138
M_151	GO:0090129	0.02138
M_151	GO:0048755	0.02138
M_151	GO:1990573	0.02138

M_151	GO:0016322	0.02372
M_151	GO:0014049	0.02372
M_151	GO:0046549	0.02372
M_151	GO:0035865	0.02372
M_151	GO:0048712	0.02372
M_151	GO:0019233	0.02395
M_151	GO:0046929	0.02606
M_151	GO:2001223	0.02606
M_151	GO:0060394	0.02606
M_151	GO:0071372	0.02606
M_151	GO:0045176	0.02606
M_151	GO:0072178	0.02606
M_151	GO:0030900	0.02727
M_151	GO:0048266	0.0284
M_151	GO:0045820	0.0284
M_151	GO:0043201	0.0284
M_151	GO:0070268	0.02954
M_151	GO:0050768	0.03039
M_151	GO:1900452	0.03073
M_151	GO:0048642	0.03073
M_151	GO:0007171	0.03073
M_151	GO:0060134	0.03073
M_151	GO:0042711	0.03306
M_151	GO:0045722	0.03306
M_151	GO:0046321	0.03306
M_151	GO:0006012	0.03306
M_151	GO:0003214	0.03306
M_151	GO:0033604	0.03306
M_151	GO:0097067	0.03538
M_151	GO:0060292	0.03684
M_151	GO:2000310	0.03769
M_151	GO:0007158	0.03769

M_151	GO:1990845	0.03769
M_151	GO:0071871	0.03769
M_151	GO:0014912	0.03769
M_151	GO:0034332	0.03825
M_151	GO:1900116	0.04
M_151	GO:2000251	0.04231
M_151	GO:0030534	0.04321
M_151	GO:0031290	0.0446
M_151	GO:1903861	0.0446
M_151	GO:0051497	0.0469
M_151	GO:0008219	0.04767
M_151	GO:0051412	0.04918
M_152	GO:0035722	0.00043
M_152	GO:0042776	0.00132
M_152	GO:0080129	0.0032
M_152	GO:0097332	0.0032
M_152	GO:0042407	0.00468
M_152	GO:0032212	0.00527
M_152	GO:0070268	0.00551
M_152	GO:0086091	0.0059
M_152	GO:0044334	0.00638
M_152	GO:0019542	0.00638
M_152	GO:0019427	0.00638
M_152	GO:0019413	0.00638
M_152	GO:0060940	0.00638
M_152	GO:0048213	0.00638
M_152	GO:1902159	0.00638
M_152	GO:1901979	0.00956
M_152	GO:2001245	0.00956
M_152	GO:0032218	0.00956
M_152	GO:0072709	0.00956
M_152	GO:0006433	0.00956

M_152	GO:0016267	0.00956
M_152	GO:0010909	0.00956
M_152	GO:2000984	0.00956
M_152	GO:0006458	0.01157
M_152	GO:0060040	0.01273
M_152	GO:0000301	0.01273
M_152	GO:0034465	0.01273
M_152	GO:0003056	0.01273
M_152	GO:0090383	0.01273
M_152	GO:0050821	0.01455
M_152	GO:0007156	0.01554
M_152	GO:1901387	0.01561
M_152	GO:0048625	0.01588
M_152	GO:1901843	0.01588
M_152	GO:0043570	0.01588
M_152	GO:1903232	0.01588
M_152	GO:0045541	0.01588
M_152	GO:0060087	0.01903
M_152	GO:0045743	0.01903
M_152	GO:2000675	0.01903
M_152	GO:1902177	0.01903
M_152	GO:0086073	0.01903
M_152	GO:0007207	0.01903
M_152	GO:0071479	0.01943
M_152	GO:0060047	0.02126
M_152	GO:0071073	0.02217
M_152	GO:0006564	0.02217
M_152	GO:0032287	0.02217
M_152	GO:0007197	0.02217
M_152	GO:1902260	0.02217
M_152	GO:0035333	0.02217
M_152	GO:0006771	0.02217

M_152	GO:0060052	0.02529
M_152	GO:0098915	0.02529
M_152	GO:0032511	0.02529
M_152	GO:0060155	0.02529
M_152	GO:0006975	0.02529
M_152	GO:0061077	0.02705
M_152	GO:0008090	0.02841
M_152	GO:0048263	0.02841
M_152	GO:0055091	0.02841
M_152	GO:0006357	0.02954
M_152	GO:0035646	0.03152
M_152	GO:0044539	0.03152
M_152	GO:0046541	0.03152
M_152	GO:0035865	0.03152
M_152	GO:0043117	0.03152
M_152	GO:0032308	0.03152
M_152	GO:0070125	0.03224
M_152	GO:0070126	0.0336
M_152	GO:0060088	0.03461
M_152	GO:0098911	0.03461
M_152	GO:1900016	0.03461
M_152	GO:0006069	0.0377
M_152	GO:0007569	0.03923
M_152	GO:0050665	0.04078
M_152	GO:0071318	0.04078
M_152	GO:0043508	0.04385
M_152	GO:0071435	0.04691
M_152	GO:0051770	0.04691
M_152	GO:0021930	0.04691
M_152	GO:0046931	0.04996
M_152	GO:0032930	0.04996
M_152	GO:0015074	0.04996

M_152	GO:0031573	0.04996
M_153	GO:0055113	0.0029
M_153	GO:0097473	0.0029
M_153	GO:0071486	0.0029
M_153	GO:0032788	0.0029
M_153	GO:0032789	0.0029
M_153	GO:0008366	0.0056
M_153	GO:0007206	0.0057
M_153	GO:0021849	0.0057
M_153	GO:2000225	0.0057
M_153	GO:1902938	0.0085
M_153	GO:1990418	0.0085
M_153	GO:0097155	0.0085
M_153	GO:1904383	0.0085
M_153	GO:0090172	0.0085
M_153	GO:0005976	0.0112
M_153	GO:0010748	0.0114
M_153	GO:0060971	0.0114
M_153	GO:0070221	0.0142
M_153	GO:0070813	0.0142
M_153	GO:0003143	0.0148
M_153	GO:0006104	0.017
M_153	GO:0032482	0.0194
M_153	GO:0071907	0.0198
M_153	GO:0090647	0.0198
M_153	GO:0007256	0.0198
M_153	GO:0048842	0.0198
M_153	GO:0032287	0.0198
M_153	GO:0006729	0.0198
M_153	GO:0042167	0.0198
M_153	GO:0018401	0.0226
M_153	GO:0098915	0.0226

M_153	GO:0007341	0.0226
M_153	GO:0031340	0.0226
M_153	GO:0007196	0.0254
M_153	GO:0032000	0.0254
M_153	GO:0071374	0.0254
M_153	GO:1990573	0.0254
M_153	GO:0010918	0.0254
M_153	GO:0021670	0.0282
M_153	GO:0001682	0.0282
M_153	GO:0046323	0.0305
M_153	GO:0016042	0.0309
M_153	GO:2000786	0.031
M_153	GO:0051451	0.031
M_153	GO:0051764	0.0337
M_153	GO:1900363	0.0337
M_153	GO:0021692	0.0365
M_153	GO:0046459	0.0365
M_153	GO:0090220	0.042
M_153	GO:0048368	0.042
M_153	GO:0042074	0.042
M_153	GO:0035855	0.042
M_153	GO:0045725	0.0447
M_153	GO:0006744	0.0447
M_153	GO:0034397	0.0447
M_153	GO:0072673	0.0474
M_153	GO:0060644	0.0474
M_154	GO:0006298	0.000035
M_154	GO:0070475	0.0002
M_154	GO:0016446	0.00043
M_154	GO:2000167	0.00194
M_154	GO:0071908	0.00194
M_154	GO:0071909	0.00194

M_154	GO:0071228	0.00388
M_154	GO:0015891	0.00581
M_154	GO:0006556	0.00581
M_154	GO:0048496	0.00967
M_154	GO:0035469	0.00967
M_154	GO:0015939	0.00967
M_154	GO:2000002	0.00967
M_154	GO:0071910	0.01159
M_154	GO:0061051	0.01542
M_154	GO:0051026	0.01542
M_154	GO:0046784	0.01542
M_154	GO:0060027	0.01542
M_154	GO:0033601	0.01734
M_154	GO:0018026	0.01924
M_154	GO:0034214	0.01924
M_154	GO:1902004	0.01924
M_154	GO:0060287	0.01924
M_154	GO:0070269	0.02305
M_154	GO:0033089	0.02305
M_154	GO:0072189	0.02495
M_154	GO:0071028	0.02495
M_154	GO:0033539	0.02873
M_154	GO:0018027	0.0325
M_154	GO:0071539	0.03438
M_154	GO:0000712	0.03438
M_154	GO:0035338	0.03438
M_154	GO:1902176	0.03813
M_154	GO:0003283	0.03813
M_154	GO:0002544	0.04
M_154	GO:0010758	0.04186
M_154	GO:0006730	0.04929
M_154	GO:0050779	0.04929

M_154	GO:0030511	0.04929
M_155	GO:0007512	0.00052
M_155	GO:0090190	0.00085
M_155	GO:0014898	0.00114
M_155	GO:0002027	0.0012
M_155	GO:0002026	0.00185
M_155	GO:0070471	0.00223
M_155	GO:0007522	0.00228
M_155	GO:0031449	0.00228
M_155	GO:0010625	0.00228
M_155	GO:0060784	0.00228
M_155	GO:0061143	0.00228
M_155	GO:0071939	0.00228
M_155	GO:0060517	0.00228
M_155	GO:0035622	0.00228
M_155	GO:0045777	0.00322
M_155	GO:0030049	0.00357
M_155	GO:0007199	0.00456
M_155	GO:0014036	0.00456
M_155	GO:0061145	0.00456
M_155	GO:0098840	0.00456
M_155	GO:0019100	0.00456
M_155	GO:0014728	0.00456
M_155	GO:0034633	0.00456
M_155	GO:0060532	0.00456
M_155	GO:0060468	0.00456
M_155	GO:0070105	0.00456
M_155	GO:0010734	0.00456
M_155	GO:0055010	0.00537
M_155	GO:0060538	0.00656
M_155	GO:0072034	0.00683
M_155	GO:0060221	0.00683

M_155	GO:0031630	0.00683
M_155	GO:0003331	0.00683
M_155	GO:0043585	0.00683
M_155	GO:0070447	0.00683
M_155	GO:0003051	0.00683
M_155	GO:0014873	0.00683
M_155	GO:0072190	0.00683
M_155	GO:0072197	0.00683
M_155	GO:0002019	0.00683
M_155	GO:1903598	0.00683
M_155	GO:1901201	0.00888
M_155	GO:0030035	0.0091
M_155	GO:0072289	0.0091
M_155	GO:0060018	0.0091
M_155	GO:2001013	0.0091
M_155	GO:0035106	0.0091
M_155	GO:2000360	0.0091
M_155	GO:0051387	0.0091
M_155	GO:0070384	0.0091
M_155	GO:0003415	0.0091
M_155	GO:1903278	0.0091
M_155	GO:0007039	0.0091
M_155	GO:0061205	0.0091
M_155	GO:0003056	0.0091
M_155	GO:0072193	0.0091
M_155	GO:0002018	0.0091
M_155	GO:2000741	0.0091
M_155	GO:0035625	0.0091
M_155	GO:0014068	0.00966
M_155	GO:0015888	0.01137
M_155	GO:1990049	0.01137
M_155	GO:0097070	0.01137

M_155	GO:2000138	0.01137
M_155	GO:0032811	0.01137
M_155	GO:0070120	0.01137
M_155	GO:0071504	0.01137
M_155	GO:0002034	0.01137
M_155	GO:0009311	0.01335
M_155	GO:0060729	0.01362
M_155	GO:0061156	0.01362
M_155	GO:0046533	0.01362
M_155	GO:0022027	0.01362
M_155	GO:0061046	0.01362
M_155	GO:0061038	0.01362
M_155	GO:0060426	0.01362
M_155	GO:0071260	0.01368
M_155	GO:0007631	0.0153
M_155	GO:0010536	0.01588
M_155	GO:0003360	0.01588
M_155	GO:0097065	0.01588
M_155	GO:1990048	0.01588
M_155	GO:0033864	0.01588
M_155	GO:0086036	0.01588
M_155	GO:0055009	0.01588
M_155	GO:0014883	0.01588
M_155	GO:2000794	0.01588
M_155	GO:0042297	0.01588
M_155	GO:0060020	0.01812
M_155	GO:0048630	0.01812
M_155	GO:0030916	0.01812
M_155	GO:0070474	0.01812
M_155	GO:0060534	0.01812
M_155	GO:2000020	0.01812
M_155	GO:0014824	0.01812

M_155	GO:0010700	0.01812
M_155	GO:0072102	0.01812
M_155	GO:0060322	0.01914
M_155	GO:0014061	0.01987
M_155	GO:0043583	0.02009
M_155	GO:0070314	0.02037
M_155	GO:0042355	0.02037
M_155	GO:0006621	0.02037
M_155	GO:0060539	0.02037
M_155	GO:0010873	0.02037
M_155	GO:0050905	0.02105
M_155	GO:1901203	0.0226
M_155	GO:2001054	0.0226
M_155	GO:0010666	0.0226
M_155	GO:0001574	0.0226
M_155	GO:0060079	0.02479
M_155	GO:0030309	0.02484
M_155	GO:1904385	0.02484
M_155	GO:0060174	0.02484
M_155	GO:0007548	0.0261
M_155	GO:0071773	0.02663
M_155	GO:1902894	0.02707
M_155	GO:0060900	0.02707
M_155	GO:0003184	0.02707
M_155	GO:1904469	0.02707
M_155	GO:0050680	0.02833
M_155	GO:0034374	0.02929
M_155	GO:0001967	0.02929
M_155	GO:0061029	0.02929
M_155	GO:0060009	0.03151
M_155	GO:0051580	0.03151
M_155	GO:0090266	0.03151

M_155	GO:0003188	0.03151
M_155	GO:0006937	0.03235
M_155	GO:0060048	0.03254
M_155	GO:0048745	0.0329
M_155	GO:0035815	0.03372
M_155	GO:0072160	0.03372
M_155	GO:0048856	0.03436
M_155	GO:0051969	0.03593
M_155	GO:0032930	0.03593
M_155	GO:0010744	0.03593
M_155	GO:0007155	0.0372
M_155	GO:0007565	0.03795
M_155	GO:0048739	0.03813
M_155	GO:0030502	0.03813
M_155	GO:0071498	0.03813
M_155	GO:0006883	0.04033
M_155	GO:2001275	0.04033
M_155	GO:0030540	0.04033
M_155	GO:0006508	0.04047
M_155	GO:0007165	0.0422
M_155	GO:0030903	0.04252
M_155	GO:0034104	0.04252
M_155	GO:0032332	0.04252
M_155	GO:0031145	0.04374
M_155	GO:0010667	0.04471
M_155	GO:0001502	0.04471
M_155	GO:0032331	0.04471
M_155	GO:0036065	0.04471
M_155	GO:0045742	0.04578
M_155	GO:0061049	0.0469
M_155	GO:0071880	0.0469
M_155	GO:0097503	0.0469

M_155	GO:0060487	0.04907
M_156	GO:0035609	0.000076
M_156	GO:0031290	0.0013
M_156	GO:0032332	0.0013
M_156	GO:0001502	0.0014
M_156	GO:0001958	0.0024
M_156	GO:2000612	0.0028
M_156	GO:0006147	0.0028
M_156	GO:0045167	0.0028
M_156	GO:2000761	0.0028
M_156	GO:0035659	0.0028
M_156	GO:0061001	0.0048
M_156	GO:0039003	0.0056
M_156	GO:2000594	0.0056
M_156	GO:0038194	0.0056
M_156	GO:0032972	0.0056
M_156	GO:1900215	0.0056
M_156	GO:1900218	0.0056
M_156	GO:0072305	0.0056
M_156	GO:0001550	0.0056
M_156	GO:0014886	0.0056
M_156	GO:0002086	0.0056
M_156	GO:0002063	0.0061
M_156	GO:0060066	0.0084
M_156	GO:2000611	0.0084
M_156	GO:1902731	0.0084
M_156	GO:0035019	0.0086
M_156	GO:0007417	0.0107
M_156	GO:1902807	0.011
M_156	GO:0072289	0.0111
M_156	GO:0072278	0.0111
M_156	GO:2000546	0.0111

M_156	GO:0038162	0.0111
M_156	GO:0035910	0.0111
M_156	GO:0022009	0.0111
M_156	GO:0045636	0.0111
M_156	GO:0014834	0.0111
M_156	GO:0072108	0.0111
M_156	GO:0007416	0.0138
M_156	GO:0001757	0.0139
M_156	GO:0001992	0.0139
M_156	GO:0031133	0.0139
M_156	GO:1900020	0.0139
M_156	GO:0061056	0.0139
M_156	GO:0060385	0.0139
M_156	GO:0003289	0.0139
M_156	GO:0003229	0.0164
M_156	GO:0072284	0.0167
M_156	GO:0001957	0.0167
M_156	GO:0061038	0.0167
M_156	GO:0014719	0.0167
M_156	GO:0046069	0.0167
M_156	GO:0035108	0.0188
M_156	GO:0003357	0.0194
M_156	GO:0072307	0.0194
M_156	GO:0019227	0.0194
M_156	GO:0014883	0.0194
M_156	GO:0045162	0.0194
M_156	GO:0021707	0.0194
M_156	GO:0030334	0.0194
M_156	GO:0009953	0.0203
M_156	GO:0060054	0.0222
M_156	GO:0051152	0.0222
M_156	GO:0060993	0.0245

M_156	GO:0035630	0.0276
M_156	GO:0006828	0.0276
M_156	GO:0003183	0.0276
M_156	GO:0007220	0.0276
M_156	GO:0042428	0.0276
M_156	GO:0003211	0.0276
M_156	GO:0090263	0.0279
M_156	GO:0002328	0.0303
M_156	GO:0060174	0.0303
M_156	GO:0090527	0.0303
M_156	GO:0008652	0.0307
M_156	GO:0030950	0.0331
M_156	GO:0060707	0.0331
M_156	GO:0030213	0.0358
M_156	GO:0070307	0.0358
M_156	GO:0042415	0.0358
M_156	GO:0060997	0.037
M_156	GO:0043249	0.0385
M_156	GO:2000146	0.0409
M_156	GO:0030949	0.0412
M_156	GO:0071599	0.0412
M_156	GO:0003179	0.043
M_156	GO:0032012	0.0438
M_156	GO:0006646	0.0438
M_156	GO:1902043	0.0465
M_156	GO:0019934	0.0465
M_156	GO:0006195	0.0483
M_156	GO:0030214	0.0492
M_156	GO:0042994	0.0492
M_156	GO:0006516	0.0492
M_156	GO:0071371	0.0492
M_156	GO:0009950	0.0492

M_157	GO:0098719	0.0011
M_157	GO:0048012	0.0013
M_157	GO:1904199	0.0034
M_157	GO:0014876	0.0034
M_157	GO:0090251	0.0034
M_157	GO:0003108	0.0034
M_157	GO:0060048	0.0045
M_157	GO:0060764	0.0067
M_157	GO:1903949	0.0067
M_157	GO:0014736	0.0067
M_157	GO:0060545	0.0067
M_157	GO:0060490	0.0067
M_157	GO:0060488	0.0067
M_157	GO:0060489	0.0067
M_157	GO:2000452	0.0067
M_157	GO:0044331	0.0069
M_157	GO:0045668	0.0092
M_157	GO:0086047	0.0101
M_157	GO:0086048	0.0101
M_157	GO:0090107	0.0101
M_157	GO:0060160	0.0101
M_157	GO:0016339	0.0114
M_157	GO:0051660	0.0134
M_157	GO:0048105	0.0134
M_157	GO:0042853	0.0134
M_157	GO:1902109	0.0134
M_157	GO:0097029	0.0134
M_157	GO:0038083	0.0138
M_157	GO:0045600	0.0163
M_157	GO:0035426	0.0167
M_157	GO:1903298	0.0167
M_157	GO:0038145	0.0167

M_157	GO:0070235	0.0167
M_157	GO:0061304	0.0167
M_157	GO:1904179	0.0167
M_157	GO:0010968	0.0167
M_157	GO:0086045	0.0167
M_157	GO:0071681	0.0167
M_157	GO:1902262	0.0167
M_157	GO:0009056	0.0168
M_157	GO:0007156	0.0179
M_157	GO:0008064	0.0196
M_157	GO:0070257	0.02
M_157	GO:0000902	0.0204
M_157	GO:0045988	0.0233
M_157	GO:1902231	0.0233
M_157	GO:0042632	0.0245
M_157	GO:0016477	0.0255
M_157	GO:0097581	0.0263
M_157	GO:0051683	0.0266
M_157	GO:0048539	0.0266
M_157	GO:1990000	0.0266
M_157	GO:0006853	0.0266
M_157	GO:0007217	0.0266
M_157	GO:0061408	0.0266
M_157	GO:0044332	0.0266
M_157	GO:0042981	0.027
M_157	GO:0051289	0.0291
M_157	GO:0032000	0.0299
M_157	GO:0071550	0.0299
M_157	GO:0042355	0.0299
M_157	GO:0030311	0.0332
M_157	GO:0035878	0.0332
M_157	GO:0045217	0.0332

M_157	GO:0098911	0.0364
M_157	GO:0090527	0.0364
M_157	GO:0060033	0.0364
M_157	GO:0034372	0.0364
M_157	GO:0045176	0.0364
M_157	GO:0033690	0.0364
M_157	GO:0072672	0.0364
M_157	GO:0090630	0.0377
M_157	GO:0071257	0.0397
M_157	GO:0031581	0.0397
M_157	GO:1990001	0.0397
M_157	GO:0031115	0.0397
M_157	GO:0051798	0.0397
M_157	GO:0023051	0.0418
M_157	GO:0007229	0.0423
M_157	GO:0033605	0.0429
M_157	GO:0071285	0.0429
M_157	GO:0055064	0.0429
M_157	GO:0090179	0.0429
M_157	GO:0071318	0.0429
M_157	GO:0021692	0.0429
M_157	GO:0006873	0.0454
M_157	GO:0033169	0.0461
M_157	GO:0009437	0.0461
M_157	GO:0034435	0.0461
M_157	GO:0038061	0.0488
M_157	GO:0035635	0.0494
M_157	GO:0042074	0.0494
M_157	GO:0048172	0.0494
M_157	GO:0007097	0.0494
M_157	GO:0035729	0.0494
M_157	GO:0060547	0.0494

M_157	GO:0035589	0.0494
M_157	GO:0050765	0.0494
M_157	GO:0051291	0.0496
M_158	GO:0051895	0.000032
M_158	GO:0040037	0.000039
M_158	GO:0051897	0.000041
M_158	GO:0030198	0.000047
M_158	GO:0030324	0.000051
M_158	GO:0090131	0.00017
M_158	GO:0090090	0.00019
M_158	GO:0010757	0.00026
M_158	GO:0051045	0.00048
M_158	GO:0051918	0.00077
M_158	GO:1900119	0.00113
M_158	GO:0010952	0.00132
M_158	GO:0045579	0.00133
M_158	GO:0007155	0.00177
M_158	GO:0006936	0.00179
M_158	GO:0006937	0.00196
M_158	GO:0002576	0.00211
M_158	GO:0008285	0.00306
M_158	GO:1901331	0.00422
M_158	GO:0035491	0.00422
M_158	GO:2000119	0.00422
M_158	GO:0021768	0.00422
M_158	GO:0061044	0.00422
M_158	GO:1901202	0.00422
M_158	GO:1990654	0.00422
M_158	GO:0003065	0.00422
M_158	GO:0003017	0.00422
M_158	GO:0010754	0.00422
M_158	GO:1904328	0.00422

M_158	GO:0030206	0.00496
M_158	GO:0010628	0.00502
M_158	GO:0030511	0.00536
M_158	GO:0001501	0.00545
M_158	GO:0030335	0.00756
M_158	GO:0002605	0.00843
M_158	GO:0001300	0.00843
M_158	GO:0061033	0.00843
M_158	GO:0033082	0.00843
M_158	GO:0038098	0.00843
M_158	GO:1901731	0.00843
M_158	GO:2000860	0.00843
M_158	GO:2000098	0.00843
M_158	GO:0090038	0.00843
M_158	GO:1902081	0.00843
M_158	GO:0086092	0.00843
M_158	GO:1901877	0.00843
M_158	GO:0006986	0.00871
M_158	GO:0070527	0.01121
M_158	GO:0046854	0.01158
M_158	GO:0030049	0.01179
M_158	GO:0002581	0.01262
M_158	GO:0060838	0.01262
M_158	GO:0032487	0.01262
M_158	GO:0072277	0.01262
M_158	GO:1903984	0.01262
M_158	GO:1902174	0.01262
M_158	GO:0090271	0.01262
M_158	GO:0060166	0.01262
M_158	GO:0038091	0.01262
M_158	GO:0035790	0.01262
M_158	GO:1901895	0.01262

M_158	GO:0010751	0.01262
M_158	GO:0048619	0.01262
M_158	GO:0030336	0.01271
M_158	GO:0045766	0.01334
M_158	GO:0033574	0.01359
M_158	GO:0010718	0.01421
M_158	GO:0043410	0.01581
M_158	GO:0048701	0.01616
M_158	GO:0001945	0.01658
M_158	GO:0006710	0.01679
M_158	GO:2001027	0.01679
M_158	GO:0060665	0.01679
M_158	GO:1990834	0.01679
M_158	GO:0070384	0.01679
M_158	GO:0090027	0.01679
M_158	GO:1903588	0.01679
M_158	GO:1901842	0.01679
M_158	GO:0086023	0.01679
M_158	GO:0097187	0.01679
M_158	GO:1901509	0.01679
M_158	GO:0035582	0.01679
M_158	GO:0010748	0.01679
M_158	GO:0010951	0.01875
M_158	GO:0036092	0.01892
M_158	GO:0045652	0.01964
M_158	GO:0046426	0.01964
M_158	GO:0034097	0.02094
M_158	GO:0045542	0.02094
M_158	GO:0042494	0.02094
M_158	GO:0072144	0.02094
M_158	GO:0030091	0.02094
M_158	GO:0060501	0.02094

M_158	GO:0060681	0.02094
M_158	GO:2000051	0.02094
M_158	GO:0051136	0.02094
M_158	GO:0009725	0.02188
M_158	GO:0071230	0.02419
M_158	GO:0021957	0.02508
M_158	GO:0032914	0.02508
M_158	GO:0030579	0.02508
M_158	GO:0060316	0.02508
M_158	GO:2000739	0.02508
M_158	GO:0043615	0.02508
M_158	GO:0070278	0.02508
M_158	GO:0019800	0.02508
M_158	GO:0022617	0.02599
M_158	GO:0016055	0.02829
M_158	GO:0010544	0.02883
M_158	GO:0060013	0.0292
M_158	GO:2000483	0.0292
M_158	GO:0051694	0.0292
M_158	GO:0090091	0.0292
M_158	GO:1901897	0.0292
M_158	GO:0086036	0.0292
M_158	GO:2000544	0.0292
M_158	GO:0031532	0.02935
M_158	GO:0022414	0.02936
M_158	GO:0003281	0.02998
M_158	GO:0043462	0.03254
M_158	GO:1903671	0.03289
M_158	GO:0008360	0.0332
M_158	GO:0035360	0.0333
M_158	GO:0051549	0.0333
M_158	GO:0002138	0.0333

M_158	GO:0002072	0.0333
M_158	GO:0001763	0.03628
M_158	GO:0034115	0.03739
M_158	GO:0010838	0.03739
M_158	GO:0006002	0.03739
M_158	GO:0048263	0.03739
M_158	GO:0048845	0.03739
M_158	GO:0008543	0.03911
M_158	GO:0001553	0.04146
M_158	GO:0010459	0.04146
M_158	GO:0033629	0.04146
M_158	GO:0001893	0.04496
M_158	GO:2000392	0.04551
M_158	GO:0090331	0.04551
M_158	GO:0060576	0.04551
M_158	GO:0006048	0.04551
M_158	GO:0003334	0.04551
M_158	GO:0019371	0.04551
M_158	GO:0032060	0.04551
M_158	GO:0042535	0.04551
M_158	GO:0033327	0.04551
M_158	GO:0010763	0.04551
M_158	GO:1990440	0.04551
M_158	GO:0048738	0.04743
M_158	GO:0050829	0.04809
M_158	GO:0030239	0.04839
M_158	GO:0048266	0.04954
M_158	GO:0055062	0.04954
M_158	GO:0010759	0.04954
M_158	GO:0060707	0.04954
M_158	GO:0045019	0.04954
M_158	GO:0001886	0.04954

M_158	GO:0030208	0.04954
M_159	GO:0060032	0.0000048
M_159	GO:0021938	0.000029
M_159	GO:0097421	0.000058
M_159	GO:0060831	0.00043
M_159	GO:2000773	0.00057
M_159	GO:0030850	0.00085
M_159	GO:0021696	0.00202
M_159	GO:0000165	0.00211
M_159	GO:0009954	0.00216
M_159	GO:1904530	0.00223
M_159	GO:0043375	0.00223
M_159	GO:0010157	0.00223
M_159	GO:0032848	0.00223
M_159	GO:0035978	0.00223
M_159	GO:0046671	0.00223
M_159	GO:2000691	0.00223
M_159	GO:0003131	0.00223
M_159	GO:2000685	0.00223
M_159	GO:0031052	0.00223
M_159	GO:0021508	0.00223
M_159	GO:0010039	0.0023
M_159	GO:0060603	0.00244
M_159	GO:0032355	0.00275
M_159	GO:0048009	0.0029
M_159	GO:0021549	0.0034
M_159	GO:0048546	0.00374
M_159	GO:0045668	0.00412
M_159	GO:0021983	0.00412
M_159	GO:0051389	0.00445
M_159	GO:0021997	0.00445
M_159	GO:0090402	0.00445

M_159	GO:0000320	0.00445
M_159	GO:0007418	0.00649
M_159	GO:0015891	0.00666
M_159	GO:0098912	0.00666
M_159	GO:0035986	0.00666
M_159	GO:0021965	0.00666
M_159	GO:0009957	0.00666
M_159	GO:0006808	0.00666
M_159	GO:0086047	0.00666
M_159	GO:0086048	0.00666
M_159	GO:0021775	0.00666
M_159	GO:0021776	0.00666
M_159	GO:0021747	0.00666
M_159	GO:2001038	0.00666
M_159	GO:0001658	0.00788
M_159	GO:0002326	0.00888
M_159	GO:0045636	0.00888
M_159	GO:0014042	0.00888
M_159	GO:0021759	0.00888
M_159	GO:0010468	0.00902
M_159	GO:0001822	0.00925
M_159	GO:0008284	0.01029
M_159	GO:1904031	0.01082
M_159	GO:0086045	0.01108
M_159	GO:0086046	0.01108
M_159	GO:0048041	0.01179
M_159	GO:0022898	0.01311
M_159	GO:0042593	0.01328
M_159	GO:0043697	0.01328
M_159	GO:0070141	0.01328
M_159	GO:0060372	0.01328
M_159	GO:2001033	0.01328

M_159	GO:0045740	0.01401
M_159	GO:0042493	0.01429
M_159	GO:0009615	0.01468
M_159	GO:0007411	0.01491
M_159	GO:0007442	0.01548
M_159	GO:0097500	0.01548
M_159	GO:0045656	0.01548
M_159	GO:0031658	0.01548
M_159	GO:0003360	0.01548
M_159	GO:0071864	0.01548
M_159	GO:0051902	0.01548
M_159	GO:0060373	0.01548
M_159	GO:1903546	0.01548
M_159	GO:0010523	0.01548
M_159	GO:0042475	0.01604
M_159	GO:0001843	0.01604
M_159	GO:0009791	0.0171
M_159	GO:0035331	0.01767
M_159	GO:0043497	0.01767
M_159	GO:0060371	0.01767
M_159	GO:0048743	0.01767
M_159	GO:0043583	0.01907
M_159	GO:1902808	0.01939
M_159	GO:0033601	0.01986
M_159	GO:0032000	0.01986
M_159	GO:0071679	0.01986
M_159	GO:0045869	0.01986
M_159	GO:0033689	0.01986
M_159	GO:0021670	0.02205
M_159	GO:2000774	0.02205
M_159	GO:0014894	0.02205
M_159	GO:0072203	0.02205

M_159	GO:0060513	0.02205
M_159	GO:0046323	0.0237
M_159	GO:0090527	0.02422
M_159	GO:0040015	0.02422
M_159	GO:0045760	0.02422
M_159	GO:0033327	0.02422
M_159	GO:1901379	0.02581
M_159	GO:0007275	0.02611
M_159	GO:1900037	0.0264
M_159	GO:0033197	0.0264
M_159	GO:0033089	0.0264
M_159	GO:1903818	0.0264
M_159	GO:0043922	0.0264
M_159	GO:0050680	0.02703
M_159	GO:0009642	0.02857
M_159	GO:0071397	0.02857
M_159	GO:2000345	0.02857
M_159	GO:0001701	0.03087
M_159	GO:0090090	0.03151
M_159	GO:0014065	0.03198
M_159	GO:0033033	0.03289
M_159	GO:0046628	0.03289
M_159	GO:0010875	0.03289
M_159	GO:0019511	0.03289
M_159	GO:0008589	0.03338
M_159	GO:0043066	0.03401
M_159	GO:0051262	0.03434
M_159	GO:0035988	0.03504
M_159	GO:0035864	0.03504
M_159	GO:2000679	0.03504
M_159	GO:0045725	0.03504
M_159	GO:0021542	0.03504

M_159	GO:0035313	0.03504
M_159	GO:0000122	0.03565
M_159	GO:2000811	0.03719
M_159	GO:0060644	0.03719
M_159	GO:0060307	0.03719
M_159	GO:0033673	0.0379
M_159	GO:0032026	0.03934
M_159	GO:0060749	0.03934
M_159	GO:0010224	0.03934
M_159	GO:0006582	0.03934
M_159	GO:2001275	0.03934
M_159	GO:0097062	0.03934
M_159	GO:0021522	0.04047
M_159	GO:0002320	0.04148
M_159	GO:0038111	0.04148
M_159	GO:0003323	0.04148
M_159	GO:0002076	0.04148
M_159	GO:2000637	0.04148
M_159	GO:0097242	0.04148
M_159	GO:0007605	0.04181
M_159	GO:0032331	0.04362
M_159	GO:0090102	0.04466
M_159	GO:1904659	0.04575
M_159	GO:0014911	0.04575
M_159	GO:0051412	0.04575
M_159	GO:0043496	0.04575
M_159	GO:0007289	0.04575
M_159	GO:0010165	0.04671
M_159	GO:0044321	0.04788
M_159	GO:0032469	0.04788
M_159	GO:0060045	0.05
M_159	GO:0045606	0.05

M_159	GO:0007095	0.05
M_159	GO:0071157	0.05
M_160	GO:0090129	0.00041
M_160	GO:0002003	0.00062
M_160	GO:2000311	0.00329
M_160	GO:0042941	0.00342
M_160	GO:0042666	0.00342
M_160	GO:0061090	0.00342
M_160	GO:0007218	0.00559
M_160	GO:0042942	0.00684
M_160	GO:1903598	0.01024
M_160	GO:0015827	0.01024
M_160	GO:0003051	0.01024
M_160	GO:0071691	0.01024
M_160	GO:0036343	0.01363
M_160	GO:0014834	0.01363
M_160	GO:0007598	0.01363
M_160	GO:0038170	0.01701
M_160	GO:0046813	0.01701
M_160	GO:0048170	0.01701
M_160	GO:0038031	0.02038
M_160	GO:1901621	0.02038
M_160	GO:0098532	0.02373
M_160	GO:0035093	0.02708
M_160	GO:0060452	0.02708
M_160	GO:0060054	0.02708
M_160	GO:0021940	0.02708
M_160	GO:0015825	0.03041
M_160	GO:0048712	0.03373
M_160	GO:0035845	0.03373
M_160	GO:0034446	0.03434
M_160	GO:0007288	0.03705

M_160	GO:0033234	0.03705
M_160	GO:0001833	0.03705
M_160	GO:0097091	0.03705
M_160	GO:0070588	0.03829
M_160	GO:0045741	0.04035
M_160	GO:0031115	0.04035
M_160	GO:2000726	0.04035
M_160	GO:0030819	0.043
M_160	GO:0031274	0.04363
M_161	GO:0050911	0.0000047
M_161	GO:0030322	0.000053
M_161	GO:0007186	0.00012
M_161	GO:0048664	0.00076
M_161	GO:0035725	0.00084
M_161	GO:0007548	0.00136
M_161	GO:0043584	0.00218
M_161	GO:0006625	0.00398
M_161	GO:0003311	0.00468
M_161	GO:0002395	0.00468
M_161	GO:0090104	0.00468
M_161	GO:1903118	0.00468
M_161	GO:0032980	0.00468
M_161	GO:2000266	0.00468
M_161	GO:0046296	0.00468
M_161	GO:0048561	0.00468
M_161	GO:0021920	0.00468
M_161	GO:0071444	0.00468
M_161	GO:0045109	0.00514
M_161	GO:0003333	0.00534
M_161	GO:0035774	0.00865
M_161	GO:0019836	0.00934
M_161	GO:2000854	0.00934

M_161	GO:1900111	0.00934
M_161	GO:0042322	0.00934
M_161	GO:1900229	0.00934
M_161	GO:0019265	0.00934
M_161	GO:0050828	0.00934
M_161	GO:0071676	0.00934
M_161	GO:0046724	0.00934
M_161	GO:0021558	0.01398
M_161	GO:0019448	0.01398
M_161	GO:0021502	0.01398
M_161	GO:1900114	0.01398
M_161	GO:0009436	0.01398
M_161	GO:0033685	0.01398
M_161	GO:0071314	0.01398
M_161	GO:0070268	0.01563
M_161	GO:0007601	0.01592
M_161	GO:0021575	0.01841
M_161	GO:0070779	0.01859
M_161	GO:1990034	0.01859
M_161	GO:0021555	0.01859
M_161	GO:0021557	0.01859
M_161	GO:0042866	0.01859
M_161	GO:0042853	0.01859
M_161	GO:0007290	0.01859
M_161	GO:0010025	0.01859
M_161	GO:2000065	0.01859
M_161	GO:0070093	0.01859
M_161	GO:0032348	0.01859
M_161	GO:0060164	0.01859
M_161	GO:0010841	0.01859
M_161	GO:0030070	0.01859
M_161	GO:0003309	0.02293

M_161	GO:0008628	0.02319
M_161	GO:0098712	0.02319
M_161	GO:0051461	0.02319
M_161	GO:0051464	0.02319
M_161	GO:0038170	0.02319
M_161	GO:0032811	0.02319
M_161	GO:0060431	0.02319
M_161	GO:0021797	0.02319
M_161	GO:0010815	0.02319
M_161	GO:2000987	0.02319
M_161	GO:0009649	0.02746
M_161	GO:0021937	0.02776
M_161	GO:0021853	0.02776
M_161	GO:0043569	0.02776
M_161	GO:0060710	0.02776
M_161	GO:0043697	0.02776
M_161	GO:0035524	0.02776
M_161	GO:0015837	0.03168
M_161	GO:0016101	0.03208
M_161	GO:0021902	0.03231
M_161	GO:0097065	0.03231
M_161	GO:0003310	0.03231
M_161	GO:0014062	0.03231
M_161	GO:0060449	0.03231
M_161	GO:0051384	0.03293
M_161	GO:0021537	0.03523
M_161	GO:0006704	0.03642
M_161	GO:0035095	0.03684
M_161	GO:0001561	0.03684
M_161	GO:0002138	0.03684
M_161	GO:0010700	0.03684
M_161	GO:0003344	0.04135

M_161	GO:0098703	0.04135
M_161	GO:0021702	0.04135
M_161	GO:0060456	0.04584
M_161	GO:0060291	0.04936
M_162	GO:0070651	0.0029
M_162	GO:1990504	0.0029
M_162	GO:1902078	0.0029
M_162	GO:0033343	0.0029
M_162	GO:0070481	0.0029
M_162	GO:0007155	0.0042
M_162	GO:0071109	0.0058
M_162	GO:0042264	0.0058
M_162	GO:0034334	0.0087
M_162	GO:0070966	0.0087
M_162	GO:0045163	0.0087
M_162	GO:0044339	0.0087
M_162	GO:1903553	0.0116
M_162	GO:0015798	0.0116
M_162	GO:0038026	0.0116
M_162	GO:0007258	0.0116
M_162	GO:0006663	0.0116
M_162	GO:0097114	0.0116
M_162	GO:2000969	0.0145
M_162	GO:0060179	0.0145
M_162	GO:0071205	0.0145
M_162	GO:0033591	0.0173
M_162	GO:0006777	0.0173
M_162	GO:0010694	0.0173
M_162	GO:0051971	0.0173
M_162	GO:0008306	0.0201
M_162	GO:0032287	0.0202
M_162	GO:0009134	0.0202

M_162	GO:0036089	0.0202
M_162	GO:0042297	0.0202
M_162	GO:0038042	0.0231
M_162	GO:0032790	0.0231
M_162	GO:0021800	0.0231
M_162	GO:0090129	0.0259
M_162	GO:0007196	0.0259
M_162	GO:0002091	0.0259
M_162	GO:0008090	0.0259
M_162	GO:0006971	0.0259
M_162	GO:0060100	0.0259
M_162	GO:0043589	0.0259
M_162	GO:0097119	0.0259
M_162	GO:0051639	0.0287
M_162	GO:0006930	0.0287
M_162	GO:0050966	0.0316
M_162	GO:0007229	0.0325
M_162	GO:0031581	0.0344
M_162	GO:0071107	0.0344
M_162	GO:0071257	0.0344
M_162	GO:0061024	0.0361
M_162	GO:0038065	0.0372
M_162	GO:0009143	0.0372
M_162	GO:0021819	0.0372
M_162	GO:0021794	0.04
M_162	GO:0007626	0.0423
M_162	GO:0016082	0.0428
M_162	GO:1903543	0.0428
M_162	GO:0034656	0.0428
M_162	GO:0070365	0.0428
M_162	GO:2000310	0.0456
M_162	GO:0032793	0.0456

M_162	GO:0015732	0.0456
M_162	GO:0097120	0.0456
M_162	GO:0022011	0.0475
M_162	GO:0006929	0.0475
M_162	GO:0061003	0.0484
M_162	GO:0002098	0.0484
M_162	GO:0045780	0.0484
M_162	GO:0021756	0.0484
M_162	GO:0071025	0.0484
M_162	GO:0006897	0.0497
M_163	GO:0019046	0.000043
M_163	GO:1990167	0.00013
M_163	GO:0006211	0.00013
M_163	GO:1901673	0.00021
M_163	GO:0045944	0.00022
M_163	GO:0006357	0.00024
M_163	GO:0007296	0.00025
M_163	GO:0046833	0.00063
M_163	GO:0090267	0.00088
M_163	GO:1903608	0.00088
M_163	GO:0000122	0.00106
M_163	GO:0071108	0.00119
M_163	GO:0006897	0.00126
M_163	GO:0048024	0.0014
M_163	GO:0033601	0.00149
M_163	GO:0000380	0.00184
M_163	GO:0035871	0.00186
M_163	GO:0006325	0.00203
M_163	GO:0045019	0.0027
M_163	GO:1990830	0.00304
M_163	GO:0070989	0.00424
M_163	GO:0080182	0.00424

M_163	GO:0001701	0.00431
M_163	GO:0051568	0.00468
M_163	GO:0036342	0.00545
M_163	GO:0032508	0.00623
M_163	GO:0031453	0.00656
M_163	GO:0000435	0.00656
M_163	GO:0039695	0.00656
M_163	GO:0071765	0.00656
M_163	GO:1900082	0.00656
M_163	GO:1903249	0.00656
M_163	GO:0071701	0.00656
M_163	GO:0000388	0.00656
M_163	GO:0098736	0.00656
M_163	GO:0006355	0.0067
M_163	GO:0000724	0.00676
M_163	GO:2000637	0.00679
M_163	GO:0008380	0.00709
M_163	GO:0070932	0.00751
M_163	GO:0007266	0.01026
M_163	GO:0071140	0.01309
M_163	GO:0031990	0.01309
M_163	GO:0034721	0.01309
M_163	GO:0044727	0.01309
M_163	GO:0060382	0.01309
M_163	GO:0060490	0.01309
M_163	GO:0060488	0.01309
M_163	GO:0060489	0.01309
M_163	GO:1904059	0.01309
M_163	GO:1904017	0.01309
M_163	GO:1903691	0.01309
M_163	GO:0061346	0.01309
M_163	GO:0046832	0.01309

M_163	GO:0000354	0.01309
M_163	GO:0080111	0.01347
M_163	GO:0007205	0.01445
M_163	GO:0048844	0.01626
M_163	GO:0001523	0.01844
M_163	GO:0043487	0.01902
M_163	GO:0051222	0.01932
M_163	GO:0090310	0.01957
M_163	GO:0001555	0.01957
M_163	GO:2001038	0.01957
M_163	GO:0090116	0.01957
M_163	GO:0044205	0.01957
M_163	GO:1900042	0.01957
M_163	GO:0022007	0.01957
M_163	GO:0002265	0.01957
M_163	GO:0006404	0.01957
M_163	GO:0030997	0.01957
M_163	GO:0060763	0.01957
M_163	GO:0010751	0.01957
M_163	GO:0044313	0.01957
M_163	GO:0030178	0.0197
M_163	GO:0043001	0.0209
M_163	GO:0097421	0.0209
M_163	GO:0045746	0.02327
M_163	GO:0070849	0.02561
M_163	GO:0019932	0.02573
M_163	GO:0050434	0.0258
M_163	GO:1990168	0.026
M_163	GO:1903347	0.026
M_163	GO:1903912	0.026
M_163	GO:0048105	0.026
M_163	GO:0032741	0.026

M_163	GO:0002155	0.026
M_163	GO:2000864	0.026
M_163	GO:0030423	0.026
M_163	GO:0030951	0.026
M_163	GO:2000587	0.026
M_163	GO:2000623	0.026
M_163	GO:0035549	0.026
M_163	GO:0071559	0.0261
M_163	GO:0007173	0.02682
M_163	GO:0035735	0.02833
M_163	GO:0071364	0.02833
M_163	GO:0043045	0.03212
M_163	GO:0072104	0.0324
M_163	GO:2000481	0.0324
M_163	GO:0090245	0.0324
M_163	GO:0051599	0.0324
M_163	GO:0003150	0.0324
M_163	GO:0000738	0.0324
M_163	GO:1903898	0.0324
M_163	GO:0010961	0.0324
M_163	GO:0035787	0.0324
M_163	GO:1902083	0.0324
M_163	GO:0070934	0.0324
M_163	GO:0036091	0.0324
M_163	GO:0051414	0.0324
M_163	GO:0035523	0.0324
M_163	GO:1901660	0.0324
M_163	GO:0035509	0.0324
M_163	GO:0050684	0.03333
M_163	GO:0031936	0.03843
M_163	GO:0010825	0.03875
M_163	GO:2000373	0.03875

M_163	GO:1900223	0.03875
M_163	GO:0071286	0.03875
M_163	GO:1902961	0.03875
M_163	GO:0006526	0.03875
M_163	GO:1902966	0.03875
M_163	GO:1902177	0.03875
M_163	GO:1903243	0.03875
M_163	GO:0003402	0.03875
M_163	GO:0061038	0.03875
M_163	GO:0060992	0.03875
M_163	GO:0007182	0.03875
M_163	GO:0045743	0.03875
M_163	GO:0035822	0.03875
M_163	GO:0006207	0.03875
M_163	GO:0045652	0.04421
M_163	GO:0048146	0.04421
M_163	GO:2000394	0.04506
M_163	GO:0060028	0.04506
M_163	GO:1900246	0.04506
M_163	GO:2000973	0.04506
M_163	GO:0051001	0.04506
M_163	GO:0044829	0.04506
M_163	GO:2000767	0.04506
M_163	GO:0044806	0.04506
M_163	GO:1902741	0.04506
M_163	GO:0048702	0.04506
M_163	GO:0000395	0.04506
M_163	GO:0001826	0.04506
M_163	GO:1903078	0.04579
M_163	GO:0010942	0.04971
M_164	GO:0008053	0.0014
M_164	GO:0001100	0.0023

M_164	GO:0021771	0.0023
M_164	GO:0010578	0.0023
M_164	GO:2000003	0.0023
M_164	GO:1902462	0.0023
M_164	GO:0071206	0.0023
M_164	GO:0000266	0.0027
M_164	GO:1901731	0.0047
M_164	GO:0036363	0.0047
M_164	GO:2000474	0.0047
M_164	GO:0048823	0.0047
M_164	GO:0060629	0.0047
M_164	GO:0070560	0.0047
M_164	GO:0035585	0.0047
M_164	GO:0051496	0.0054
M_164	GO:0003331	0.007
M_164	GO:0090272	0.007
M_164	GO:0060084	0.007
M_164	GO:0060168	0.007
M_164	GO:0045163	0.007
M_164	GO:0006667	0.007
M_164	GO:0006749	0.0081
M_164	GO:0000003	0.009
M_164	GO:0009181	0.0093
M_164	GO:0035021	0.0093
M_164	GO:2000852	0.0093
M_164	GO:0048499	0.0093
M_164	GO:2000741	0.0093
M_164	GO:0042320	0.0116
M_164	GO:0071376	0.0116
M_164	GO:2000048	0.0116
M_164	GO:0031133	0.0116
M_164	GO:0001661	0.0116

M_164	GO:2001046	0.014
M_164	GO:0045759	0.014
M_164	GO:0033603	0.014
M_164	GO:0021853	0.014
M_164	GO:0042138	0.014
M_164	GO:0090315	0.014
M_164	GO:0021631	0.014
M_164	GO:0051458	0.0163
M_164	GO:0032226	0.0163
M_164	GO:0051013	0.0163
M_164	GO:0031659	0.0163
M_164	GO:0035095	0.0186
M_164	GO:0021562	0.0186
M_164	GO:0035331	0.0186
M_164	GO:0045188	0.0186
M_164	GO:0090263	0.02
M_164	GO:0019236	0.0209
M_164	GO:0048251	0.0232
M_164	GO:0018095	0.0232
M_164	GO:1901203	0.0232
M_164	GO:0072537	0.0232
M_164	GO:0043653	0.0232
M_164	GO:0006626	0.0238
M_164	GO:0010763	0.0255
M_164	GO:0031284	0.0255
M_164	GO:0090527	0.0255
M_164	GO:0016188	0.0277
M_164	GO:0010801	0.03
M_164	GO:0045836	0.03
M_164	GO:0043652	0.03
M_164	GO:1900025	0.03
M_164	GO:0048168	0.0316

M_164	GO:0031589	0.0321
M_164	GO:0033169	0.0323
M_164	GO:0001675	0.0323
M_164	GO:0034380	0.0345
M_164	GO:0071850	0.0345
M_164	GO:0034656	0.0345
M_164	GO:0051895	0.0345
M_164	GO:0048172	0.0345
M_164	GO:0007567	0.0368
M_164	GO:0007096	0.0381
M_164	GO:2000353	0.0391
M_164	GO:0022010	0.0391
M_164	GO:0097090	0.0391
M_164	GO:1901386	0.0436
M_164	GO:2000637	0.0436
M_164	GO:0001666	0.0444
M_164	GO:0007338	0.0447
M_164	GO:0071305	0.0458
M_164	GO:0042053	0.0458
M_164	GO:0001502	0.0458
M_164	GO:0035584	0.0458
M_164	GO:0007413	0.048
M_165	GO:0036158	0.00058
M_165	GO:0002053	0.00104
M_165	GO:0002931	0.00163
M_165	GO:2000808	0.00171
M_165	GO:1904414	0.00171
M_165	GO:0048352	0.00171
M_165	GO:0021998	0.00171
M_165	GO:0048250	0.00342
M_165	GO:0051548	0.00342
M_165	GO:0019550	0.00342

M_165	GO:0019551	0.00342
M_165	GO:0048378	0.00342
M_165	GO:0097010	0.00342
M_165	GO:0006532	0.00513
M_165	GO:0006533	0.00513
M_165	GO:1903984	0.00513
M_165	GO:0042733	0.00535
M_165	GO:0090071	0.00683
M_165	GO:0032286	0.00683
M_165	GO:1903690	0.00683
M_165	GO:0060024	0.00683
M_165	GO:0001880	0.00683
M_165	GO:0006287	0.00853
M_165	GO:0048382	0.00853
M_165	GO:0060179	0.00853
M_165	GO:1903232	0.00853
M_165	GO:0003186	0.00853
M_165	GO:0006114	0.00853
M_165	GO:0055072	0.00865
M_165	GO:0061002	0.01023
M_165	GO:0006797	0.01023
M_165	GO:0031642	0.01023
M_165	GO:0051902	0.01193
M_165	GO:0061312	0.01193
M_165	GO:0060290	0.01193
M_165	GO:0031658	0.01193
M_165	GO:2000767	0.01193
M_165	GO:0097107	0.01193
M_165	GO:0061626	0.01362
M_165	GO:1990416	0.01362
M_165	GO:0032966	0.01362
M_165	GO:0035912	0.01362

M_165	GO:0003223	0.01362
M_165	GO:0006268	0.01531
M_165	GO:1990314	0.01531
M_165	GO:0090394	0.01531
M_165	GO:0035562	0.01531
M_165	GO:0007613	0.01621
M_165	GO:0045792	0.017
M_165	GO:0001731	0.017
M_165	GO:0010459	0.017
M_165	GO:0003183	0.017
M_165	GO:0010666	0.017
M_165	GO:0034214	0.017
M_165	GO:0097105	0.017
M_165	GO:0006264	0.01868
M_165	GO:0051593	0.01868
M_165	GO:0060736	0.01868
M_165	GO:0032355	0.01957
M_165	GO:0014067	0.02036
M_165	GO:0060044	0.02036
M_165	GO:0003161	0.02036
M_165	GO:0045475	0.02036
M_165	GO:0071257	0.02036
M_165	GO:0048681	0.02036
M_165	GO:0060391	0.02036
M_165	GO:0006107	0.02036
M_165	GO:0009743	0.02083
M_165	GO:0060134	0.02204
M_165	GO:0015937	0.02204
M_165	GO:1904668	0.02204
M_165	GO:0042711	0.02372
M_165	GO:0071361	0.02372
M_165	GO:0006390	0.02372

M_165	GO:2000060	0.02372
M_165	GO:0048853	0.02372
M_165	GO:0046855	0.02372
M_165	GO:0055089	0.02372
M_165	GO:0050765	0.02539
M_165	GO:0034356	0.02539
M_165	GO:0051895	0.02539
M_165	GO:0051967	0.02539
M_165	GO:0051481	0.02706
M_165	GO:0071871	0.02706
M_165	GO:1901017	0.02706
M_165	GO:0021542	0.02706
M_165	GO:0003272	0.02706
M_165	GO:0003222	0.02706
M_165	GO:0014912	0.02706
M_165	GO:0016446	0.02706
M_165	GO:0055070	0.02873
M_165	GO:0044320	0.02873
M_165	GO:0002902	0.03039
M_165	GO:0006516	0.03039
M_165	GO:0009950	0.03039
M_165	GO:1902074	0.03039
M_165	GO:0060074	0.03206
M_165	GO:0006044	0.03206
M_165	GO:0003215	0.03206
M_165	GO:0032211	0.03372
M_165	GO:0006825	0.03537
M_165	GO:0007398	0.03537
M_165	GO:0090344	0.03703
M_165	GO:0048678	0.0375
M_165	GO:0060045	0.03868
M_165	GO:0002092	0.04032

M_165	GO:0060914	0.04032
M_165	GO:1902895	0.04032
M_165	GO:0010719	0.04197
M_165	GO:2000463	0.04197
M_165	GO:0003148	0.04197
M_165	GO:0046856	0.04197
M_165	GO:0035902	0.04197
M_165	GO:0030511	0.04361
M_165	GO:0033032	0.04525
M_165	GO:0060292	0.04853
M_165	GO:0032869	0.04868
M_166	GO:0002250	0.000012
M_166	GO:0042074	0.00055
M_166	GO:0050855	0.0012
M_166	GO:0035249	0.00126
M_166	GO:0042100	0.00217
M_166	GO:0002642	0.00234
M_166	GO:2000549	0.00234
M_166	GO:0015826	0.00234
M_166	GO:0034589	0.00234
M_166	GO:1900176	0.00468
M_166	GO:0046005	0.00468
M_166	GO:0031443	0.00468
M_166	GO:0002554	0.007
M_166	GO:0035811	0.007
M_166	GO:0002408	0.00933
M_166	GO:2001274	0.00933
M_166	GO:0010841	0.00933
M_166	GO:0015811	0.00933
M_166	GO:0003419	0.00933
M_166	GO:0003056	0.00933
M_166	GO:0035814	0.00933

M_166	GO:0097029	0.00933
M_166	GO:0035582	0.00933
M_166	GO:0032264	0.01165
M_166	GO:2000382	0.01165
M_166	GO:0032229	0.01165
M_166	GO:0045329	0.01165
M_166	GO:0003105	0.01165
M_166	GO:0045759	0.01396
M_166	GO:0015746	0.01396
M_166	GO:0006868	0.01396
M_166	GO:0045627	0.01396
M_166	GO:0035524	0.01396
M_166	GO:0038171	0.01627
M_166	GO:0051001	0.01627
M_166	GO:0002606	0.01627
M_166	GO:0015808	0.01627
M_166	GO:2000669	0.01627
M_166	GO:0051260	0.01787
M_166	GO:0070125	0.01802
M_166	GO:0006959	0.01847
M_166	GO:0060267	0.01857
M_166	GO:0070126	0.0188
M_166	GO:0048263	0.02087
M_166	GO:0072610	0.02087
M_166	GO:0051573	0.02087
M_166	GO:0007196	0.02087
M_166	GO:0015825	0.02087
M_166	GO:0048312	0.02087
M_166	GO:1904153	0.02087
M_166	GO:0001768	0.02316
M_166	GO:2000253	0.02316
M_166	GO:0021670	0.02316

M_166	GO:0003094	0.02485
M_166	GO:0007218	0.02507
M_166	GO:0033004	0.02545
M_166	GO:0050853	0.02622
M_166	GO:1901533	0.02773
M_166	GO:0051255	0.02773
M_166	GO:0001771	0.03001
M_166	GO:0007320	0.03001
M_166	GO:0007200	0.03109
M_166	GO:0030183	0.03207
M_166	GO:1902358	0.03228
M_166	GO:0021527	0.03228
M_166	GO:0030968	0.03256
M_166	GO:0033209	0.03405
M_166	GO:0071435	0.03455
M_166	GO:0030595	0.03632
M_166	GO:0030889	0.03681
M_166	GO:0040037	0.03681
M_166	GO:0010832	0.03907
M_166	GO:0097094	0.04132
M_166	GO:0071731	0.04356
M_166	GO:0033622	0.04356
M_166	GO:0010560	0.04356
M_166	GO:1903861	0.04356
M_166	GO:0023019	0.0458
M_166	GO:0007154	0.04725
M_166	GO:0006851	0.04804
M_167	GO:0045292	0.0001
M_167	GO:0035735	0.00017
M_167	GO:0000381	0.00058
M_167	GO:0034620	0.0007
M_167	GO:0031124	0.00258

M_167	GO:0046355	0.00268
M_167	GO:0046587	0.00268
M_167	GO:0019290	0.00268
M_167	GO:1903893	0.00268
M_167	GO:0006406	0.00303
M_167	GO:0045184	0.00444
M_167	GO:1902530	0.00536
M_167	GO:1904049	0.00536
M_167	GO:1902283	0.00536
M_167	GO:1903382	0.00536
M_167	GO:1902897	0.00803
M_167	GO:1903542	0.00803
M_167	GO:0016476	0.00803
M_167	GO:0010636	0.00803
M_167	GO:0000103	0.00803
M_167	GO:0006458	0.00826
M_167	GO:0097250	0.01069
M_167	GO:0051582	0.01069
M_167	GO:0071934	0.01069
M_167	GO:0090521	0.01069
M_167	GO:0044314	0.01334
M_167	GO:0070842	0.01334
M_167	GO:0061734	0.01334
M_167	GO:0035519	0.01334
M_167	GO:0033132	0.01334
M_167	GO:0044828	0.01334
M_167	GO:1903377	0.01334
M_167	GO:0006886	0.01585
M_167	GO:0050428	0.01599
M_167	GO:1902514	0.01599
M_167	GO:0010637	0.01599
M_167	GO:0010994	0.01599

M_167	GO:0042723	0.01863
M_167	GO:1901897	0.01863
M_167	GO:1901844	0.01863
M_167	GO:0006477	0.01863
M_167	GO:0072383	0.01863
M_167	GO:0035333	0.01863
M_167	GO:1903599	0.01863
M_167	GO:0061077	0.01949
M_167	GO:1901725	0.02127
M_167	GO:0015884	0.02127
M_167	GO:0032790	0.02127
M_167	GO:1903265	0.02127
M_167	GO:0006108	0.02127
M_167	GO:0085020	0.02127
M_167	GO:0006890	0.02232
M_167	GO:0042073	0.02244
M_167	GO:0046951	0.02389
M_167	GO:0097267	0.02389
M_167	GO:0071287	0.02389
M_167	GO:0070050	0.02389
M_167	GO:1990830	0.0248
M_167	GO:0010666	0.02651
M_167	GO:0001731	0.02651
M_167	GO:0035721	0.02651
M_167	GO:0075522	0.02651
M_167	GO:0006228	0.02651
M_167	GO:0006183	0.02913
M_167	GO:0006048	0.02913
M_167	GO:0006751	0.02913
M_167	GO:1990440	0.02913
M_167	GO:0030208	0.03173
M_167	GO:2000650	0.03173

M_167	GO:0006107	0.03173
M_167	GO:0033197	0.03173
M_167	GO:0051583	0.03433
M_167	GO:0042415	0.03433
M_167	GO:0002093	0.03433
M_167	GO:0030157	0.03433
M_167	GO:0006241	0.03692
M_167	GO:1903351	0.03692
M_167	GO:0009313	0.03951
M_167	GO:0090141	0.03951
M_167	GO:0016254	0.03951
M_167	GO:0035372	0.03951
M_167	GO:0006163	0.03972
M_167	GO:0034975	0.04209
M_167	GO:0006750	0.04209
M_167	GO:0010881	0.04209
M_167	GO:0050427	0.04375
M_167	GO:0090201	0.04466
M_167	GO:0007224	0.0469
M_167	GO:0032185	0.04723
M_167	GO:0006516	0.04723
M_167	GO:1902254	0.04979
M_167	GO:1903861	0.04979
M_168	GO:0032981	5.5E-10
M_168	GO:0006120	0.0000096
M_168	GO:0042776	0.000014
M_168	GO:0070584	0.000024
M_168	GO:0042407	0.0001
M_168	GO:0009060	0.00016
M_168	GO:0045039	0.00022
M_168	GO:0006105	0.00052
M_168	GO:0022900	0.00072

M_168	GO:0006123	0.00093
M_168	GO:1902600	0.00111
M_168	GO:2000778	0.00212
M_168	GO:0015797	0.00285
M_168	GO:0015724	0.00285
M_168	GO:0052567	0.00285
M_168	GO:0006979	0.00292
M_168	GO:0015991	0.0033
M_168	GO:2001150	0.0057
M_168	GO:0000961	0.0057
M_168	GO:0045728	0.0057
M_168	GO:1904579	0.0057
M_168	GO:0046724	0.0057
M_168	GO:0032618	0.0057
M_168	GO:1902268	0.00854
M_168	GO:0070295	0.00854
M_168	GO:0019087	0.00854
M_168	GO:0097250	0.01137
M_168	GO:0009298	0.01137
M_168	GO:0033615	0.01137
M_168	GO:0015990	0.01137
M_168	GO:0034553	0.01137
M_168	GO:0070945	0.01137
M_168	GO:0072675	0.01419
M_168	GO:0051454	0.01419
M_168	GO:0071332	0.01419
M_168	GO:0030321	0.01419
M_168	GO:1902617	0.01419
M_168	GO:0007585	0.01481
M_168	GO:0071300	0.01658
M_168	GO:0051096	0.017
M_168	GO:0006104	0.017

M_168	GO:0006119	0.01868
M_168	GO:0042775	0.01956
M_168	GO:0010764	0.01981
M_168	GO:0019064	0.01981
M_168	GO:0032532	0.01981
M_168	GO:0006782	0.02261
M_168	GO:0071493	0.02261
M_168	GO:0042045	0.02261
M_168	GO:0072321	0.02261
M_168	GO:0042117	0.02261
M_168	GO:0032471	0.02261
M_168	GO:0061469	0.02261
M_168	GO:0006890	0.02505
M_168	GO:0042989	0.0254
M_168	GO:0060586	0.0254
M_168	GO:0070125	0.02615
M_168	GO:0015986	0.02654
M_168	GO:0070126	0.02726
M_168	GO:0051561	0.02818
M_168	GO:0071639	0.02818
M_168	GO:0071609	0.02818
M_168	GO:0001682	0.02818
M_168	GO:0043653	0.02818
M_168	GO:0009142	0.02862
M_168	GO:0006264	0.03096
M_168	GO:0032700	0.03096
M_168	GO:0090197	0.03373
M_168	GO:0016559	0.03373
M_168	GO:0006596	0.03649
M_168	GO:0032469	0.03848
M_168	GO:1902358	0.03924
M_168	GO:0035810	0.03924

M_168	GO:0006122	0.03924
M_168	GO:0019372	0.03924
M_168	GO:0090141	0.04198
M_168	GO:2000484	0.04198
M_168	GO:0007286	0.04321
M_168	GO:0006465	0.04472
M_168	GO:0035458	0.04472
M_168	GO:0043280	0.04883
M_169	GO:0018406	0.00031
M_169	GO:0046847	0.00147
M_169	GO:1990268	0.00388
M_169	GO:1904271	0.00388
M_169	GO:1902684	0.00388
M_169	GO:0018872	0.00388
M_169	GO:0001572	0.00388
M_169	GO:1903743	0.00388
M_169	GO:0002149	0.00388
M_169	GO:1904556	0.00388
M_169	GO:1904340	0.00388
M_169	GO:0021956	0.00388
M_169	GO:0051729	0.00775
M_169	GO:2000386	0.00775
M_169	GO:0032263	0.00775
M_169	GO:1904027	0.00775
M_169	GO:0071228	0.00775
M_169	GO:0086098	0.00775
M_169	GO:0006178	0.00775
M_169	GO:0019075	0.00775
M_169	GO:0031508	0.0116
M_169	GO:1901994	0.0116
M_169	GO:1904158	0.0116
M_169	GO:0007412	0.0116

M_169	GO:2000312	0.0116
M_169	GO:1901837	0.0116
M_169	GO:0006168	0.0116
M_169	GO:0070634	0.0116
M_169	GO:0033512	0.0116
M_169	GO:0071393	0.0116
M_169	GO:0001545	0.0116
M_169	GO:0007129	0.01376
M_169	GO:0007140	0.01434
M_169	GO:0060708	0.01544
M_169	GO:0001878	0.01544
M_169	GO:1903251	0.01544
M_169	GO:0016080	0.01544
M_169	GO:0007386	0.01926
M_169	GO:2001286	0.01926
M_169	GO:0032074	0.01926
M_169	GO:0032264	0.01926
M_169	GO:0021523	0.01926
M_169	GO:0051388	0.01926
M_169	GO:0010626	0.01926
M_169	GO:0010641	0.01926
M_169	GO:0098535	0.01926
M_169	GO:0033625	0.01926
M_169	GO:1904491	0.01926
M_169	GO:0007286	0.01985
M_169	GO:0032259	0.0221
M_169	GO:0048524	0.02283
M_169	GO:0097052	0.02307
M_169	GO:0098706	0.02307
M_169	GO:0046100	0.02307
M_169	GO:0015837	0.02659
M_169	GO:0008360	0.02673

M_169	GO:0019367	0.02686
M_169	GO:0051902	0.02686
M_169	GO:0000395	0.02686
M_169	GO:2000394	0.02686
M_169	GO:0015808	0.02686
M_169	GO:0034625	0.02686
M_169	GO:0034626	0.02686
M_169	GO:0014827	0.02686
M_169	GO:2000766	0.02686
M_169	GO:0035672	0.02686
M_169	GO:0045008	0.03064
M_169	GO:0071169	0.03064
M_169	GO:0097106	0.03064
M_169	GO:0007172	0.03064
M_169	GO:0015677	0.03064
M_169	GO:0050847	0.03064
M_169	GO:1990416	0.03064
M_169	GO:0019896	0.03064
M_169	GO:0060271	0.03099
M_169	GO:0008286	0.0328
M_169	GO:0001756	0.03435
M_169	GO:0051974	0.0344
M_169	GO:0042989	0.0344
M_169	GO:0008090	0.0344
M_169	GO:0046598	0.0344
M_169	GO:0002679	0.0344
M_169	GO:0006569	0.03815
M_169	GO:0030311	0.03815
M_169	GO:0000122	0.04126
M_169	GO:0043149	0.04146
M_169	GO:0010269	0.04189
M_169	GO:0050966	0.04189

M_169	GO:0071803	0.04189
M_169	GO:0032463	0.04189
M_169	GO:0000244	0.04561
M_169	GO:0021517	0.0487
M_169	GO:0007549	0.04932
M_169	GO:0042761	0.04932
M_169	GO:0034374	0.04932
M_169	GO:0070307	0.04932
M_169	GO:0044130	0.04932
M_170	GO:0006099	0.00012
M_170	GO:0055114	0.00085
M_170	GO:0007026	0.00218
M_170	GO:0036031	0.00303
M_170	GO:0018063	0.00303
M_170	GO:0080164	0.00303
M_170	GO:2000256	0.00303
M_170	GO:1904465	0.00303
M_170	GO:0006805	0.00427
M_170	GO:0006597	0.00604
M_170	GO:0046657	0.00604
M_170	GO:1903281	0.00604
M_170	GO:0061015	0.00604
M_170	GO:0006097	0.00604
M_170	GO:0044861	0.00604
M_170	GO:2001162	0.00604
M_170	GO:0010482	0.00905
M_170	GO:0045903	0.00905
M_170	GO:0007113	0.00905
M_170	GO:1902683	0.00905
M_170	GO:0009447	0.00905
M_170	GO:0038163	0.00905
M_170	GO:0042376	0.00905

M_170	GO:0032918	0.00905
M_170	GO:1903288	0.00905
M_170	GO:2000255	0.00905
M_170	GO:1903278	0.01205
M_170	GO:1903976	0.01205
M_170	GO:0046208	0.01205
M_170	GO:0006741	0.01205
M_170	GO:0051771	0.01205
M_170	GO:0050689	0.01504
M_170	GO:0048170	0.01504
M_170	GO:0002865	0.01504
M_170	GO:0006102	0.01504
M_170	GO:0045653	0.01504
M_170	GO:0045454	0.01751
M_170	GO:0015942	0.01802
M_170	GO:0045719	0.01802
M_170	GO:0045743	0.01802
M_170	GO:0052405	0.01802
M_170	GO:0042373	0.01802
M_170	GO:2000110	0.01802
M_170	GO:0006015	0.01802
M_170	GO:0061732	0.01802
M_170	GO:0006167	0.02099
M_170	GO:0006853	0.02395
M_170	GO:0022417	0.02691
M_170	GO:1990573	0.02691
M_170	GO:0070125	0.02913
M_170	GO:0006554	0.02985
M_170	GO:0090084	0.02985
M_170	GO:0070126	0.03036
M_170	GO:0006596	0.03218
M_170	GO:0019371	0.03279

M_170	GO:0003334	0.03279
M_170	GO:0048742	0.03279
M_170	GO:0006983	0.03279
M_170	GO:0006913	0.03446
M_170	GO:0006979	0.03552
M_170	GO:0009396	0.03572
M_170	GO:0035999	0.03572
M_170	GO:0051775	0.03572
M_170	GO:0015937	0.03864
M_170	GO:0060253	0.03864
M_170	GO:0010248	0.03864
M_170	GO:0010839	0.03864
M_170	GO:0042271	0.03864
M_170	GO:0030007	0.04155
M_170	GO:0046325	0.04155
M_170	GO:0046629	0.04155
M_170	GO:0019471	0.04445
M_170	GO:0051571	0.04445
M_170	GO:0080182	0.04445
M_170	GO:0007252	0.04445
M_170	GO:0033539	0.04445
M_170	GO:0070886	0.04734
M_170	GO:0045723	0.04734
M_170	GO:0006465	0.04734
M_170	GO:0006123	0.04734
M_170	GO:0086013	0.04734
M_170	GO:0043968	0.04734
M_171	GO:0048264	0.0025
M_171	GO:0035645	0.0025
M_171	GO:1903629	0.0025
M_171	GO:1903611	0.0025
M_171	GO:2001203	0.0025

M_171	GO:1903634	0.0025
M_171	GO:0051886	0.0025
M_171	GO:0030049	0.0043
M_171	GO:0014043	0.005
M_171	GO:1903625	0.005
M_171	GO:0016256	0.005
M_171	GO:0086100	0.005
M_171	GO:0034633	0.005
M_171	GO:0007497	0.005
M_171	GO:0055107	0.005
M_171	GO:0019853	0.005
M_171	GO:1902679	0.0051
M_171	GO:0086010	0.0065
M_171	GO:1901671	0.0075
M_171	GO:0048807	0.0075
M_171	GO:1901896	0.01
M_171	GO:1901318	0.01
M_171	GO:0006663	0.01
M_171	GO:0014826	0.01
M_171	GO:0030070	0.01
M_171	GO:0060406	0.0125
M_171	GO:0033299	0.0125
M_171	GO:0060157	0.0125
M_171	GO:0051344	0.0125
M_171	GO:0070973	0.015
M_171	GO:0007275	0.0152
M_171	GO:0006357	0.0166
M_171	GO:0035672	0.0175
M_171	GO:0086036	0.0175
M_171	GO:1900246	0.0175
M_171	GO:2000483	0.0175
M_171	GO:2000271	0.0175

M_171	GO:0006657	0.0175
M_171	GO:0042593	0.0184
M_171	GO:0022604	0.0189
M_171	GO:0071313	0.0199
M_171	GO:0060059	0.0199
M_171	GO:0042045	0.0199
M_171	GO:0031620	0.0199
M_171	GO:0022417	0.0224
M_171	GO:1903011	0.0248
M_171	GO:0060068	0.0248
M_171	GO:0098792	0.0248
M_171	GO:0051024	0.0273
M_171	GO:0030241	0.0273
M_171	GO:0048484	0.0273
M_171	GO:2001256	0.0297
M_171	GO:0010867	0.0297
M_171	GO:0060044	0.0297
M_171	GO:0045019	0.0297
M_171	GO:0016559	0.0297
M_171	GO:0015812	0.0322
M_171	GO:0070863	0.0322
M_171	GO:0030240	0.0346
M_171	GO:0035810	0.0346
M_171	GO:0043508	0.0346
M_171	GO:0006469	0.036
M_171	GO:1903052	0.0364
M_171	GO:0060347	0.037
M_171	GO:0019471	0.037
M_171	GO:0051546	0.037
M_171	GO:0035815	0.037
M_171	GO:0045723	0.0395
M_171	GO:0034975	0.0395

M_171	GO:0045603	0.0395
M_171	GO:0006646	0.0395
M_171	GO:0007283	0.0414
M_171	GO:0034765	0.0416
M_171	GO:0019934	0.0419
M_171	GO:0048739	0.0419
M_171	GO:0033630	0.0419
M_171	GO:2000647	0.0419
M_171	GO:0030277	0.0443
M_171	GO:0046329	0.0457
M_171	GO:0006811	0.0466
M_171	GO:2000637	0.0467
M_171	GO:0051092	0.0476
M_171	GO:0071711	0.0491
M_171	GO:0060065	0.0491
M_172	GO:0007130	0.000016
M_172	GO:0042271	0.00047
M_172	GO:0007141	0.00151
M_172	GO:1903540	0.00251
M_172	GO:1900075	0.00251
M_172	GO:0009566	0.00319
M_172	GO:0043006	0.00502
M_172	GO:0051729	0.00502
M_172	GO:0048936	0.00502
M_172	GO:0001913	0.0062
M_172	GO:1903539	0.00735
M_172	GO:0008065	0.00752
M_172	GO:0048619	0.00752
M_172	GO:0033693	0.00752
M_172	GO:0033058	0.00752
M_172	GO:0007131	0.00783
M_172	GO:0051260	0.00844

M_172	GO:0031122	0.00871
M_172	GO:0006772	0.01001
M_172	GO:0048808	0.01001
M_172	GO:0042733	0.01126
M_172	GO:0043456	0.0125
M_172	GO:0002023	0.0125
M_172	GO:0007283	0.01289
M_172	GO:0006007	0.01466
M_172	GO:0010571	0.01498
M_172	GO:1902513	0.01498
M_172	GO:0060527	0.01498
M_172	GO:0035873	0.01745
M_172	GO:1903546	0.01745
M_172	GO:0097500	0.01745
M_172	GO:0007144	0.01745
M_172	GO:0060687	0.01745
M_172	GO:0030101	0.01762
M_172	GO:1901626	0.02238
M_172	GO:0001501	0.02421
M_172	GO:0009953	0.02516
M_172	GO:0048935	0.02668
M_172	GO:0048484	0.02729
M_172	GO:0021520	0.03217
M_172	GO:0000212	0.03217
M_172	GO:0006703	0.03217
M_172	GO:0021978	0.03217
M_172	GO:0097553	0.03461
M_172	GO:0045199	0.03461
M_172	GO:0050774	0.03703
M_172	GO:0034587	0.04428
M_172	GO:0030539	0.04565
M_172	GO:0007625	0.04668

M_172	GO:0007143	0.04801
M_172	GO:0060602	0.04908
M_172	GO:2000678	0.04908
M_172	GO:0010529	0.04908
M_172	GO:0043046	0.04908
M_173	GO:1901750	0.0000078
M_173	GO:0006751	0.000011
M_173	GO:0010951	0.00051
M_173	GO:0006750	0.00193
M_173	GO:0007605	0.00307
M_173	GO:0021895	0.00399
M_173	GO:0061682	0.00411
M_173	GO:0018969	0.00411
M_173	GO:2000487	0.00411
M_173	GO:0044140	0.00411
M_173	GO:0003409	0.00411
M_173	GO:0019216	0.00467
M_173	GO:0035115	0.00717
M_173	GO:0061107	0.00809
M_173	GO:0015951	0.0082
M_173	GO:0006867	0.0082
M_173	GO:0052314	0.0082
M_173	GO:0015881	0.0082
M_173	GO:0061402	0.0082
M_173	GO:0010142	0.0082
M_173	GO:0021623	0.0082
M_173	GO:0003404	0.0082
M_173	GO:0003127	0.0082
M_173	GO:0031989	0.0082
M_173	GO:1990408	0.0082
M_173	GO:0006508	0.01007
M_173	GO:0019730	0.01149

M_173	GO:0035617	0.01228
M_173	GO:0033076	0.01228
M_173	GO:0019344	0.01228
M_173	GO:0051365	0.01228
M_173	GO:0060741	0.01228
M_173	GO:0070315	0.01228
M_173	GO:0035720	0.01228
M_173	GO:0060830	0.01228
M_173	GO:0072318	0.01228
M_173	GO:0003095	0.01228
M_173	GO:0042427	0.01228
M_173	GO:0046219	0.01228
M_173	GO:0089709	0.01228
M_173	GO:0015842	0.01634
M_173	GO:0060971	0.01634
M_173	GO:0046684	0.01634
M_173	GO:0007227	0.01634
M_173	GO:0021914	0.01634
M_173	GO:0036101	0.01634
M_173	GO:0006814	0.01738
M_173	GO:0016191	0.02038
M_173	GO:0032803	0.02038
M_173	GO:1901164	0.02038
M_173	GO:0010989	0.02038
M_173	GO:0045669	0.02298
M_173	GO:0097647	0.02441
M_173	GO:0015746	0.02441
M_173	GO:0070172	0.02441
M_173	GO:0048859	0.02441
M_173	GO:0002248	0.02441
M_173	GO:0030032	0.02529
M_173	GO:0015808	0.02842

M_173	GO:0038171	0.02842
M_173	GO:0021796	0.02842
M_173	GO:0051823	0.03242
M_173	GO:0070445	0.03242
M_173	GO:0051045	0.03242
M_173	GO:0045665	0.03327
M_173	GO:0046951	0.03639
M_173	GO:0060050	0.03639
M_173	GO:0097267	0.03639
M_173	GO:0090129	0.03639
M_173	GO:0007196	0.03639
M_173	GO:0010944	0.03639
M_173	GO:0047484	0.04036
M_173	GO:0071281	0.04036
M_173	GO:0035721	0.04036
M_173	GO:0006600	0.04036
M_173	GO:0072584	0.04036
M_173	GO:0048712	0.04036
M_173	GO:0032525	0.04036
M_173	GO:0021766	0.04093
M_173	GO:0051873	0.0443
M_173	GO:0010172	0.0443
M_173	GO:0090009	0.0443
M_173	GO:0090527	0.0443
M_173	GO:0001501	0.04821
M_173	GO:0060019	0.04823
M_173	GO:0060044	0.04823
M_173	GO:0030208	0.04823
M_173	GO:0021559	0.04823
M_173	GO:2000651	0.04823
M_173	GO:0048715	0.04823
M_173	GO:0042416	0.04823

M_173	GO:0045605	0.04823
M_174	GO:0051714	0.000032
M_174	GO:0007154	0.000059
M_174	GO:0032827	0.000064
M_174	GO:0009253	0.00022
M_174	GO:0045176	0.00058
M_174	GO:0016045	0.00082
M_174	GO:0016266	0.00108
M_174	GO:0098719	0.0011
M_174	GO:0072498	0.0011
M_174	GO:0051453	0.00228
M_174	GO:0019730	0.00253
M_174	GO:0050830	0.0029
M_174	GO:0060220	0.00331
M_174	GO:1903697	0.00331
M_174	GO:0030704	0.00331
M_174	GO:0048074	0.00331
M_174	GO:0050712	0.00331
M_174	GO:0035445	0.00331
M_174	GO:0032689	0.00471
M_174	GO:0045880	0.00501
M_174	GO:0051977	0.00661
M_174	GO:0016539	0.00661
M_174	GO:0006542	0.00661
M_174	GO:0019676	0.00661
M_174	GO:0021633	0.00661
M_174	GO:1900220	0.00661
M_174	GO:0006654	0.00702
M_174	GO:0015701	0.00892
M_174	GO:0010693	0.0099
M_174	GO:0070295	0.0099
M_174	GO:0072092	0.0099

M_174	GO:1902600	0.01014
M_174	GO:0003382	0.01285
M_174	GO:0001755	0.01285
M_174	GO:0033088	0.01318
M_174	GO:0006931	0.01318
M_174	GO:2000563	0.01318
M_174	GO:0046340	0.01318
M_174	GO:0060708	0.01318
M_174	GO:0071926	0.01318
M_174	GO:0090370	0.01318
M_174	GO:0019369	0.01332
M_174	GO:0032237	0.01645
M_174	GO:2000660	0.01645
M_174	GO:0090238	0.01645
M_174	GO:0036414	0.01645
M_174	GO:0050679	0.0179
M_174	GO:0002223	0.01904
M_174	GO:0015840	0.0197
M_174	GO:0003406	0.0197
M_174	GO:0015793	0.0197
M_174	GO:0071300	0.02193
M_174	GO:0042044	0.02258
M_174	GO:0070486	0.02274
M_174	GO:0090650	0.02295
M_174	GO:0045916	0.02295
M_174	GO:0008104	0.02344
M_174	GO:0035136	0.02578
M_174	GO:0030388	0.02619
M_174	GO:0045188	0.02619
M_174	GO:0042048	0.02619
M_174	GO:0071805	0.02786
M_174	GO:0007411	0.02787

M_174	GO:0061303	0.02941
M_174	GO:0001766	0.02941
M_174	GO:0006538	0.02941
M_174	GO:0061072	0.02941
M_174	GO:0030311	0.03263
M_174	GO:0032308	0.03263
M_174	GO:0060088	0.03583
M_174	GO:0048672	0.03583
M_174	GO:1900016	0.03583
M_174	GO:0033089	0.03902
M_174	GO:0060019	0.03902
M_174	GO:0045741	0.03902
M_174	GO:0060707	0.03902
M_174	GO:0043482	0.03902
M_174	GO:0015812	0.04221
M_174	GO:0007175	0.04538
M_174	GO:0002064	0.04804
M_174	GO:0090136	0.04854
M_174	GO:0007216	0.04854
M_174	GO:0033540	0.04854
M_174	GO:0006000	0.04854
M_175	GO:0052695	0.00015
M_175	GO:0050911	0.0002
M_175	GO:2000650	0.00052
M_175	GO:1903442	0.00285
M_175	GO:0038133	0.00285
M_175	GO:0036371	0.00285
M_175	GO:1903017	0.00285
M_175	GO:0051296	0.00285
M_175	GO:0036255	0.00285
M_175	GO:0061296	0.00285
M_175	GO:0003226	0.00285

M_175	GO:0035565	0.00285
M_175	GO:0061026	0.00285
M_175	GO:2001031	0.00285
M_175	GO:0018032	0.00285
M_175	GO:0051692	0.0057
M_175	GO:0044752	0.0057
M_175	GO:0098907	0.0057
M_175	GO:0039020	0.0057
M_175	GO:0072181	0.0057
M_175	GO:0036309	0.0057
M_175	GO:0032805	0.0057
M_175	GO:0001519	0.0057
M_175	GO:0042595	0.0057
M_175	GO:2000490	0.0057
M_175	GO:0097069	0.0057
M_175	GO:1902103	0.0057
M_175	GO:0021551	0.0057
M_175	GO:0008050	0.0057
M_175	GO:0061017	0.0057
M_175	GO:0035669	0.0057
M_175	GO:0007595	0.00795
M_175	GO:0098910	0.00854
M_175	GO:0019464	0.00854
M_175	GO:0033292	0.00854
M_175	GO:0014734	0.00854
M_175	GO:0035720	0.00854
M_175	GO:0021553	0.00854
M_175	GO:1900212	0.00854
M_175	GO:0042278	0.00854
M_175	GO:0001755	0.00966
M_175	GO:0007621	0.01137
M_175	GO:0032911	0.01137

M_175	GO:0001920	0.01137
M_175	GO:2000741	0.01137
M_175	GO:0072095	0.01137
M_175	GO:0051725	0.01137
M_175	GO:0042663	0.01137
M_175	GO:0007186	0.01198
M_175	GO:0010989	0.01419
M_175	GO:0086046	0.01419
M_175	GO:0021778	0.01419
M_175	GO:0060509	0.01419
M_175	GO:0006682	0.01419
M_175	GO:0014068	0.01481
M_175	GO:0045454	0.01568
M_175	GO:0009410	0.01643
M_175	GO:0051058	0.01671
M_175	GO:1903997	0.017
M_175	GO:0002154	0.017
M_175	GO:0070257	0.017
M_175	GO:0038031	0.017
M_175	GO:0008063	0.017
M_175	GO:0032024	0.0175
M_175	GO:0030913	0.01981
M_175	GO:0036302	0.01981
M_175	GO:0001826	0.01981
M_175	GO:0045162	0.01981
M_175	GO:0046477	0.01981
M_175	GO:0048752	0.01981
M_175	GO:0051597	0.01981
M_175	GO:0010880	0.02218
M_175	GO:0030050	0.02261
M_175	GO:0051409	0.02261
M_175	GO:0042048	0.02261

M_175	GO:0052696	0.0254
M_175	GO:0051901	0.0254
M_175	GO:0006534	0.0254
M_175	GO:0006621	0.0254
M_175	GO:0009791	0.02726
M_175	GO:0016139	0.02818
M_175	GO:0006930	0.02818
M_175	GO:0043653	0.02818
M_175	GO:0035721	0.02818
M_175	GO:0051900	0.03036
M_175	GO:0052697	0.03096
M_175	GO:0002281	0.03096
M_175	GO:0010763	0.03096
M_175	GO:0071372	0.03096
M_175	GO:0019852	0.03096
M_175	GO:2001223	0.03096
M_175	GO:0031397	0.0337
M_175	GO:2000726	0.03373
M_175	GO:0021889	0.03373
M_175	GO:0043410	0.03511
M_175	GO:0035641	0.03649
M_175	GO:0032230	0.03649
M_175	GO:0000212	0.03649
M_175	GO:0060123	0.03649
M_175	GO:0050667	0.03924
M_175	GO:0071361	0.03924
M_175	GO:0016540	0.03924
M_175	GO:0043584	0.04198
M_175	GO:0009404	0.04198
M_175	GO:0035729	0.04198
M_175	GO:0007512	0.04198
M_175	GO:0070365	0.04198

M_175	GO:0019511	0.04198
M_175	GO:0006750	0.04472
M_175	GO:2000010	0.04472
M_175	GO:0007158	0.04472
M_175	GO:0034975	0.04472
M_175	GO:0010881	0.04472
M_175	GO:0003222	0.04472
M_175	GO:0071380	0.04745
M_175	GO:0060644	0.04745
M_175	GO:0060307	0.04745
M_175	GO:0046130	0.04745
M_175	GO:0021772	0.04922
M_175	GO:0048259	0.04937
M_176	GO:0001578	0.0019
M_176	GO:0061534	0.0025
M_176	GO:0042930	0.0025
M_176	GO:0061400	0.0025
M_176	GO:0098829	0.0025
M_176	GO:0034640	0.0025
M_176	GO:0051958	0.005
M_176	GO:0021897	0.005
M_176	GO:0035038	0.005
M_176	GO:0090298	0.005
M_176	GO:1903905	0.005
M_176	GO:1904117	0.0075
M_176	GO:2001271	0.0075
M_176	GO:1900194	0.0075
M_176	GO:0043418	0.0075
M_176	GO:0050968	0.0075
M_176	GO:0035811	0.0075
M_176	GO:0001546	0.0075
M_176	GO:0006288	0.0075

M_176	GO:1904447	0.01
M_176	GO:0061535	0.01
M_176	GO:0061484	0.01
M_176	GO:0035021	0.01
M_176	GO:0007406	0.01
M_176	GO:0021764	0.01
M_176	GO:0044860	0.01
M_176	GO:2001268	0.0125
M_176	GO:0050915	0.0125
M_176	GO:0006283	0.0145
M_176	GO:1902513	0.015
M_176	GO:0060729	0.015
M_176	GO:0048209	0.0175
M_176	GO:0001826	0.0175
M_176	GO:0034128	0.0175
M_176	GO:0047497	0.0199
M_176	GO:0098597	0.0199
M_176	GO:0042148	0.0199
M_176	GO:0090385	0.0199
M_176	GO:0000707	0.0199
M_176	GO:0048478	0.0224
M_176	GO:0015886	0.0224
M_176	GO:0090073	0.0224
M_176	GO:0060631	0.0224
M_176	GO:0001765	0.0248
M_176	GO:0035865	0.0248
M_176	GO:0048712	0.0248
M_176	GO:0046929	0.0273
M_176	GO:0033314	0.0273
M_176	GO:0050966	0.0273
M_176	GO:0006273	0.0273
M_176	GO:0035330	0.0273

M_176	GO:1904322	0.0273
M_176	GO:1902992	0.0273
M_176	GO:0006171	0.0292
M_176	GO:2001241	0.0297
M_176	GO:0048715	0.0297
M_176	GO:0006390	0.0346
M_176	GO:0048853	0.0346
M_176	GO:0043248	0.0346
M_176	GO:0050965	0.037
M_176	GO:0001832	0.0395
M_176	GO:0048820	0.0395
M_176	GO:0036159	0.0395
M_176	GO:0010951	0.0414
M_176	GO:1900116	0.0419
M_176	GO:1902043	0.0419
M_176	GO:0071380	0.0419
M_176	GO:0006468	0.0423
M_176	GO:0007250	0.0443
M_176	GO:0070734	0.0467
M_176	GO:0048745	0.0491
M_177	GO:0048006	1.2E-09
M_177	GO:0048007	3.7E-09
M_177	GO:0001916	0.0000035
M_177	GO:0021524	0.000048
M_177	GO:0002317	0.000097
M_177	GO:0048536	0.00035
M_177	GO:0021520	0.00123
M_177	GO:0060347	0.00164
M_177	GO:0060413	0.00187
M_177	GO:0010832	0.00212
M_177	GO:0031290	0.00265
M_177	GO:0060929	0.00405

M_177	GO:0051308	0.00405
M_177	GO:0006061	0.00405
M_177	GO:0048621	0.00405
M_177	GO:0060588	0.00405
M_177	GO:0003168	0.00405
M_177	GO:0097238	0.00405
M_177	GO:0033388	0.00405
M_177	GO:0003148	0.00457
M_177	GO:0060037	0.00494
M_177	GO:0003203	0.00572
M_177	GO:0060928	0.00809
M_177	GO:2000098	0.00809
M_177	GO:0043377	0.00809
M_177	GO:0038156	0.00809
M_177	GO:0036304	0.00809
M_177	GO:0003342	0.00809
M_177	GO:0097168	0.00809
M_177	GO:0046370	0.00809
M_177	GO:0048936	0.00809
M_177	GO:0097055	0.00809
M_177	GO:0018931	0.00809
M_177	GO:0002040	0.00866
M_177	GO:0060412	0.00984
M_177	GO:0030198	0.01066
M_177	GO:0002636	0.01211
M_177	GO:1902732	0.01211
M_177	GO:0060913	0.01211
M_177	GO:0072137	0.01211
M_177	GO:0003350	0.01211
M_177	GO:0055014	0.01211
M_177	GO:0055005	0.01211
M_177	GO:2000820	0.01211

M_177	GO:2000813	0.01211
M_177	GO:0072061	0.01211
M_177	GO:0071657	0.01211
M_177	GO:0031296	0.01211
M_177	GO:0090258	0.01211
M_177	GO:0003285	0.01211
M_177	GO:0003221	0.01211
M_177	GO:0051835	0.01211
M_177	GO:2000405	0.01211
M_177	GO:1902988	0.01211
M_177	GO:0032870	0.01221
M_177	GO:0033209	0.01395
M_177	GO:0090090	0.01425
M_177	GO:0010501	0.01494
M_177	GO:2001212	0.0159
M_177	GO:0060971	0.01611
M_177	GO:0003166	0.01611
M_177	GO:0010735	0.01611
M_177	GO:1903588	0.01611
M_177	GO:0097250	0.01611
M_177	GO:0008295	0.01611
M_177	GO:0071475	0.01611
M_177	GO:1990410	0.01611
M_177	GO:0030154	0.01668
M_177	GO:0051414	0.0201
M_177	GO:0014012	0.0201
M_177	GO:1904428	0.0201
M_177	GO:0034197	0.0201
M_177	GO:0051891	0.0201
M_177	GO:0090074	0.0201
M_177	GO:0036016	0.0201
M_177	GO:0048880	0.0201

M_177	GO:1901985	0.02378
M_177	GO:0071385	0.02388
M_177	GO:0006475	0.02394
M_177	GO:0060754	0.02407
M_177	GO:0032730	0.02407
M_177	GO:0042985	0.02407
M_177	GO:0097647	0.02407
M_177	GO:0060842	0.02407
M_177	GO:0072038	0.02407
M_177	GO:1902474	0.02407
M_177	GO:0071638	0.02407
M_177	GO:0003278	0.02407
M_177	GO:2001015	0.02407
M_177	GO:0001570	0.02413
M_177	GO:0045766	0.02633
M_177	GO:0060333	0.02792
M_177	GO:0019367	0.02803
M_177	GO:0034625	0.02803
M_177	GO:0034626	0.02803
M_177	GO:0003190	0.02803
M_177	GO:1902416	0.02803
M_177	GO:0090091	0.02803
M_177	GO:0051597	0.02803
M_177	GO:0045944	0.0316
M_177	GO:0048541	0.03197
M_177	GO:0009414	0.03197
M_177	GO:0072161	0.03197
M_177	GO:0044597	0.03197
M_177	GO:0044598	0.03197
M_177	GO:1990000	0.03197
M_177	GO:0035912	0.03197
M_177	GO:0010917	0.03197

M_177	GO:0070831	0.03197
M_177	GO:0019896	0.03197
M_177	GO:0097084	0.03197
M_177	GO:1990416	0.03197
M_177	GO:0072386	0.03197
M_177	GO:0045665	0.03241
M_177	GO:0001525	0.03303
M_177	GO:0022900	0.03484
M_177	GO:0034333	0.03555
M_177	GO:0048312	0.0359
M_177	GO:0055091	0.0359
M_177	GO:0090336	0.0359
M_177	GO:1903748	0.0359
M_177	GO:0048143	0.0359
M_177	GO:0003139	0.0359
M_177	GO:0003266	0.0359
M_177	GO:0051639	0.03981
M_177	GO:0061314	0.03981
M_177	GO:0016322	0.03981
M_177	GO:0003211	0.03981
M_177	GO:2001214	0.03981
M_177	GO:0045666	0.04055
M_177	GO:0003208	0.04264
M_177	GO:0048935	0.0431
M_177	GO:1900016	0.0437
M_177	GO:0034372	0.0437
M_177	GO:0043116	0.0437
M_177	GO:0060379	0.0437
M_177	GO:0042532	0.0437
M_177	GO:0031623	0.04562
M_177	GO:0030239	0.04707
M_177	GO:0045591	0.04758

M_177	GO:0003376	0.04758
M_177	GO:0003184	0.04758
M_177	GO:0045078	0.04758
M_177	GO:0021559	0.04758
M_177	GO:0006919	0.04759
M_177	GO:1902476	0.04858
M_177	GO:0007565	0.04953
M_178	GO:0050911	1E-30
M_178	GO:0007186	1.5E-21
M_178	GO:0031424	1.9E-10
M_178	GO:0007565	0.000013
M_178	GO:0016998	0.00022
M_178	GO:0007187	0.00036
M_178	GO:0050829	0.00078
M_178	GO:0048743	0.00202
M_178	GO:0070197	0.00202
M_178	GO:0060415	0.00389
M_178	GO:1902358	0.00636
M_178	GO:0060280	0.00868
M_178	GO:0019481	0.00868
M_178	GO:1900758	0.00868
M_178	GO:1903804	0.00868
M_178	GO:0042693	0.00934
M_178	GO:1901741	0.01045
M_178	GO:0001502	0.01283
M_178	GO:0060078	0.01408
M_178	GO:0045663	0.0141
M_178	GO:0018149	0.0146
M_178	GO:0070268	0.01643
M_178	GO:0061379	0.01728
M_178	GO:0070352	0.01728
M_178	GO:0038060	0.01728

M_178	GO:0019265	0.01728
M_178	GO:0060468	0.01728
M_178	GO:0050828	0.01728
M_178	GO:0002017	0.01728
M_178	GO:0006789	0.01728
M_178	GO:0007214	0.01969
M_178	GO:0006540	0.02581
M_178	GO:0009436	0.02581
M_178	GO:0001546	0.02581
M_178	GO:0061368	0.02581
M_178	GO:0018094	0.03426
M_178	GO:0048133	0.03426
M_178	GO:0043315	0.03426
M_178	GO:0080154	0.03426
M_178	GO:0021759	0.03426
M_178	GO:0007283	0.0375
M_178	GO:0050830	0.03887
M_178	GO:1902476	0.04001
M_178	GO:0075044	0.04264
M_178	GO:1904058	0.04264
M_178	GO:0051454	0.04264
M_179	GO:1902430	0.000016
M_179	GO:1902963	0.000034
M_179	GO:0045332	0.00031
M_179	GO:0038027	0.00033
M_179	GO:0043547	0.00041
M_179	GO:1900223	0.0005
M_179	GO:0002576	0.00091
M_179	GO:0006888	0.00101
M_179	GO:1902004	0.00147
M_179	GO:0070527	0.00147
M_179	GO:0090630	0.00196

M_179	GO:0016188	0.00213
M_179	GO:0021819	0.00251
M_179	GO:0035815	0.00335
M_179	GO:0031338	0.00457
M_179	GO:1900142	0.00582
M_179	GO:1902997	0.00582
M_179	GO:1902998	0.00582
M_179	GO:1902955	0.00582
M_179	GO:1902948	0.00582
M_179	GO:1901076	0.00582
M_179	GO:2000753	0.00582
M_179	GO:2000755	0.00582
M_179	GO:1904426	0.00582
M_179	GO:1904400	0.00582
M_179	GO:1902771	0.00582
M_179	GO:1902847	0.00582
M_179	GO:2000533	0.00582
M_179	GO:0010037	0.00582
M_179	GO:1900163	0.00582
M_179	GO:0043114	0.00644
M_179	GO:0034162	0.00656
M_179	GO:0010804	0.00656
M_179	GO:0001503	0.00764
M_179	GO:0043277	0.00771
M_179	GO:0061024	0.00903
M_179	GO:0008286	0.00904
M_179	GO:0043407	0.00921
M_179	GO:0007613	0.01049
M_179	GO:0071260	0.01063
M_179	GO:0048015	0.01144
M_179	GO:0032571	0.0115
M_179	GO:0021814	0.0115

M_179	GO:0097241	0.01161
M_179	GO:1902995	0.01161
M_179	GO:1902960	0.01161
M_179	GO:0061518	0.01161
M_179	GO:1900248	0.01161
M_179	GO:0032912	0.01161
M_179	GO:2000124	0.01161
M_179	GO:0031022	0.01161
M_179	GO:0033595	0.01161
M_179	GO:0071307	0.01161
M_179	GO:0034146	0.01161
M_179	GO:0036324	0.01161
M_179	GO:0007493	0.01161
M_179	GO:0033159	0.01161
M_179	GO:0008306	0.01295
M_179	GO:0008015	0.0136
M_179	GO:0051604	0.01411
M_179	GO:0045907	0.01486
M_179	GO:0002687	0.01622
M_179	GO:1904612	0.01737
M_179	GO:0019254	0.01737
M_179	GO:0046878	0.01737
M_179	GO:1902953	0.01737
M_179	GO:0021817	0.01737
M_179	GO:0032485	0.01737
M_179	GO:0032423	0.01737
M_179	GO:0031630	0.01737
M_179	GO:0045715	0.01737
M_179	GO:0060083	0.01737
M_179	GO:0023021	0.01737
M_179	GO:0010593	0.01737
M_179	GO:0051490	0.01737

M_179	GO:0061181	0.01737
M_179	GO:1900365	0.01737
M_179	GO:0007412	0.01737
M_179	GO:0003051	0.01737
M_179	GO:1903296	0.01737
M_179	GO:0007616	0.01763
M_179	GO:0032091	0.01802
M_179	GO:0002755	0.01859
M_179	GO:0018279	0.02163
M_179	GO:0006897	0.02181
M_179	GO:0043312	0.0225
M_179	GO:1900221	0.02265
M_179	GO:0030947	0.02267
M_179	GO:0003413	0.0229
M_179	GO:1990743	0.02309
M_179	GO:0032911	0.02309
M_179	GO:0032483	0.02309
M_179	GO:0043553	0.02309
M_179	GO:0035106	0.02309
M_179	GO:0007598	0.02309
M_179	GO:0032286	0.02309
M_179	GO:0070602	0.02309
M_179	GO:0010748	0.02309
M_179	GO:0033634	0.02309
M_179	GO:0003104	0.02309
M_179	GO:0007039	0.02309
M_179	GO:0038026	0.02309
M_179	GO:0043378	0.02309
M_179	GO:0097494	0.02309
M_179	GO:0032079	0.02309
M_179	GO:0060486	0.02309
M_179	GO:2001137	0.02309

M_179	GO:0014910	0.02375
M_179	GO:0051223	0.02814
M_179	GO:0034260	0.02825
M_179	GO:0051891	0.02878
M_179	GO:0046813	0.02878
M_179	GO:0032962	0.02878
M_179	GO:1900747	0.02878
M_179	GO:0043570	0.02878
M_179	GO:0033578	0.02878
M_179	GO:0097068	0.02878
M_179	GO:0060510	0.02878
M_179	GO:0032224	0.02878
M_179	GO:0032229	0.02878
M_179	GO:0033625	0.02878
M_179	GO:0031914	0.02878
M_179	GO:0045053	0.02878
M_179	GO:0010641	0.02878
M_179	GO:1902617	0.02878
M_179	GO:2000370	0.02878
M_179	GO:0006954	0.02999
M_179	GO:0070534	0.03062
M_179	GO:0070085	0.03331
M_179	GO:0032460	0.03412
M_179	GO:1903243	0.03443
M_179	GO:0001957	0.03443
M_179	GO:0042985	0.03443
M_179	GO:1902961	0.03443
M_179	GO:1902966	0.03443
M_179	GO:0008063	0.03443
M_179	GO:1902949	0.03443
M_179	GO:0010193	0.03443
M_179	GO:2000270	0.03443

M_179	GO:0050748	0.03443
M_179	GO:0090306	0.03443
M_179	GO:0010756	0.03443
M_179	GO:0090166	0.03443
M_179	GO:0042713	0.03443
M_179	GO:0010807	0.03443
M_179	GO:0010694	0.03443
M_179	GO:0006686	0.03443
M_179	GO:0034504	0.03451
M_179	GO:0090090	0.03655
M_179	GO:0032355	0.03729
M_179	GO:0034351	0.03967
M_179	GO:0097286	0.03969
M_179	GO:0072577	0.03977
M_179	GO:1903896	0.04006
M_179	GO:0051694	0.04006
M_179	GO:0003310	0.04006
M_179	GO:0019064	0.04006
M_179	GO:0071455	0.04006
M_179	GO:0034154	0.04006
M_179	GO:0043983	0.04006
M_179	GO:2000669	0.04006
M_179	GO:0060430	0.04006
M_179	GO:0090091	0.04006
M_179	GO:1900165	0.04006
M_179	GO:0021796	0.04006
M_179	GO:0090647	0.04006
M_179	GO:0070498	0.04082
M_179	GO:0040008	0.04193
M_179	GO:0014909	0.04487
M_179	GO:1903363	0.04542
M_179	GO:0007217	0.04565

M_179	GO:0006987	0.04565
M_179	GO:0046600	0.04565
M_179	GO:0035754	0.04565
M_179	GO:0001778	0.04565
M_179	GO:0071420	0.04565
M_179	GO:0071447	0.04565
M_179	GO:0007042	0.04565
M_179	GO:0034144	0.04565
M_179	GO:2000510	0.04565
M_179	GO:0032825	0.04565
M_179	GO:0043497	0.04565
M_179	GO:0098711	0.04565
M_179	GO:0070474	0.04565
M_179	GO:0070417	0.04565
M_179	GO:0060947	0.04565
M_179	GO:0036155	0.04565
M_179	GO:0016266	0.0492
M_180	GO:0070125	0.0014
M_180	GO:0070126	0.0015
M_180	GO:0007628	0.0019
M_180	GO:0046380	0.0025
M_180	GO:0006185	0.0025
M_180	GO:0046711	0.0025
M_180	GO:0060311	0.0025
M_180	GO:0042774	0.0025
M_180	GO:0010711	0.0025
M_180	GO:0046054	0.0025
M_180	GO:0006660	0.0025
M_180	GO:0008617	0.0025
M_180	GO:0019742	0.0025
M_180	GO:0043067	0.0026
M_180	GO:0007174	0.005

M_180	GO:0052651	0.005
M_180	GO:0046122	0.005
M_180	GO:0098507	0.005
M_180	GO:0060313	0.0075
M_180	GO:0034436	0.0075
M_180	GO:0030961	0.0075
M_180	GO:0006045	0.0075
M_180	GO:0010716	0.01
M_180	GO:0030043	0.01
M_180	GO:0002949	0.01
M_180	GO:0046070	0.01
M_180	GO:0046060	0.0125
M_180	GO:2000582	0.0125
M_180	GO:0042262	0.0125
M_180	GO:0009635	0.015
M_180	GO:0017196	0.015
M_180	GO:0019673	0.0175
M_180	GO:0018394	0.0196
M_180	GO:0042747	0.0199
M_180	GO:0060155	0.0199
M_180	GO:0048227	0.0199
M_180	GO:0002084	0.0224
M_180	GO:0097428	0.0224
M_180	GO:0036353	0.0224
M_180	GO:0042989	0.0224
M_180	GO:0051014	0.0224
M_180	GO:0006915	0.0245
M_180	GO:2001269	0.0248
M_180	GO:0035335	0.0256
M_180	GO:0009157	0.0273
M_180	GO:0030836	0.0273
M_180	GO:0014067	0.0297

M_180	GO:0051775	0.0297
M_180	GO:2001241	0.0297
M_180	GO:0009743	0.0318
M_180	GO:2000009	0.0322
M_180	GO:1902306	0.0322
M_180	GO:0060009	0.0346
M_180	GO:0016540	0.0346
M_180	GO:0035815	0.037
M_180	GO:0034656	0.037
M_180	GO:0043101	0.0395
M_180	GO:0035458	0.0395
M_180	GO:0005980	0.0419
M_180	GO:0055070	0.0419
M_180	GO:0032026	0.0443
M_180	GO:0006907	0.0443
M_180	GO:0048490	0.0443
M_180	GO:0051170	0.0485
[truncated: 729,516 more chars]
